# SyntenyTracker: a tool for defining homologous synteny blocks using radiation hybrid maps and whole-genome sequence

**DOI:** 10.1186/1756-0500-2-148

**Published:** 2009-07-23

**Authors:** Ravikiran Donthu, Harris A Lewin, Denis M Larkin

**Affiliations:** 1 Department of Animal Sciences, University of Illinois at Urbana-Champaign, Urbana, Illinois, 61801, USA; 2 Institute for Genomic Biology, University of Illinois at Urbana-Champaign, Urbana, Illinois, 61801, USA

## Abstract

**Background:**

The recent availability of genomic sequences and BAC libraries for a large number of mammals provides an excellent opportunity for identifying comparatively-anchored markers that are useful for creating high-resolution radiation-hybrid (RH) and BAC-based comparative maps. To use these maps for multispecies genome comparison and evolutionary inference, robust bioinformatic tools are required for the identification of chromosomal regions shared between genomes and to localize the positions of evolutionary breakpoints that are the signatures of chromosomal rearrangements. Here we report an automated tool for the identification of homologous synteny blocks (HSBs) between genomes that tolerates errors common in RH comparative maps and can be used for automated whole-genome analysis of chromosome rearrangements that occur during evolution.

**Findings:**

We developed an algorithm and software tool (SyntenyTracker) that can be used for automated definition of HSBs using pair-wise RH or gene-based comparative maps as input. To verify correct implementation of the underlying algorithm, SyntenyTracker was used to identify HSBs in the cattle and human genomes. Results demonstrated 96% agreement with HSBs defined manually using the same set of rules. A comparison of SyntenyTracker with the AutoGRAPH synteny tool was performed using identical datasets containing 14,380 genes with 1:1 orthology in human and mouse. Discrepancies between the results using the two tools and advantages of SyntenyTracker are reported.

**Conclusion:**

SyntenyTracker was shown to be an efficient and accurate automated tool for defining HSBs using datasets that may contain minor errors resulting from limitations in map construction methodologies. The utility of SyntenyTracker will become more important for comparative genomics as the number of mapped and sequenced genomes increases.

## Background

Understanding of the comparative organization and evolution of mammalian genomes has dramatically improved with the availability of complete genome sequences and detailed physical maps of chromosomes for a growing number of species [1]. Recently, the National Human Genome Research Institute sponsored genome sequencing of 24 mammalian species representing 15 orders, but a majority of these genomes will be sequenced to only 2× coverage [2]. Despite the limited coverage, these genomic sequences are an excellent resource for constructing high-resolution radiation-hybrid (RH) comparative maps following a procedures described earlier for BAC-end sequences [3,4]. High-resolution RH comparative maps can provide as high level of granularity of comparative information as 7× genome sequence assemblies but at the fraction of the cost [5]. For example, RH maps have been used to discover specific features within evolutionary chromosomal breakpoint regions and homologous synteny blocks (HSBs) [1].

Automated tools for HSB identification [6-9] use different approaches to tolerate errors arising during the construction of RH maps. Earlier we proposed a set of rules to compensate for errors in comparative maps built with RH mapping data [1]. Here we report the development of an algorithm and a program that automatically defines HSBs on RH and gene-based comparative maps using this rule set.

## Methods

**Figure 1:**
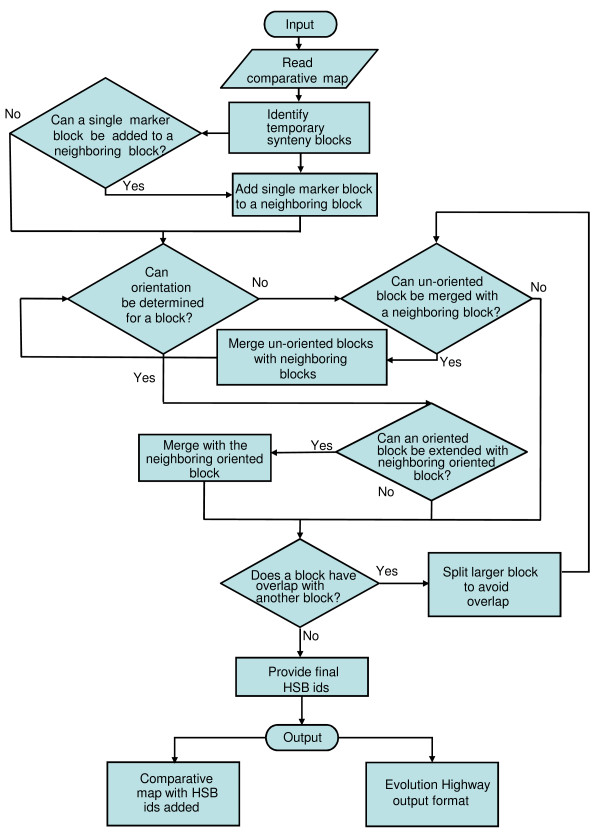
**Schematic representation of the algorithm for identification of HSBs with SyntenyTracker**.

As an input, SyntenyTracker uses a tab-delimited file containing information that includes chromosome assignment of orthologous markers in two genomes, position of each marker in the chromosomes of both genomes, and marker identifiers. The markers in the input table are sorted on the basis of their chromosome assignments and positions in one of the two genomes. This genome is termed as the "reference genome." The second genome is called the "target genome" (see additional file 1: Table S1 for an example of the input file format). Coordinates are provided in base pairs or map units, thus making SyntenyTracker suitable for building HSBs from any comparative map. Description of the SyntenyTracker algorithm is presented in Figure 1. For the pseudocode implementation of the algorithm see additional file 1. SyntenyTracker provides output as two text files. The first file contains the original input with HSB identifiers added to each line. The second file contains information on the chromosome assignment, start and end chromosome coordinates and relative orientation of each HSB in the genomes compared.

The SyntenyTracker tool is freely available online . The user can select from two modes that include "Radiation Hybrid" mode and "Orthologous Gene" mode. The major difference between these modes is that in the "Radiation Hybrid" mode definition of the HSB orientation takes into consideration possible "flips" of adjacent markers on a comparative map.

### Testing

**Figure 2:**
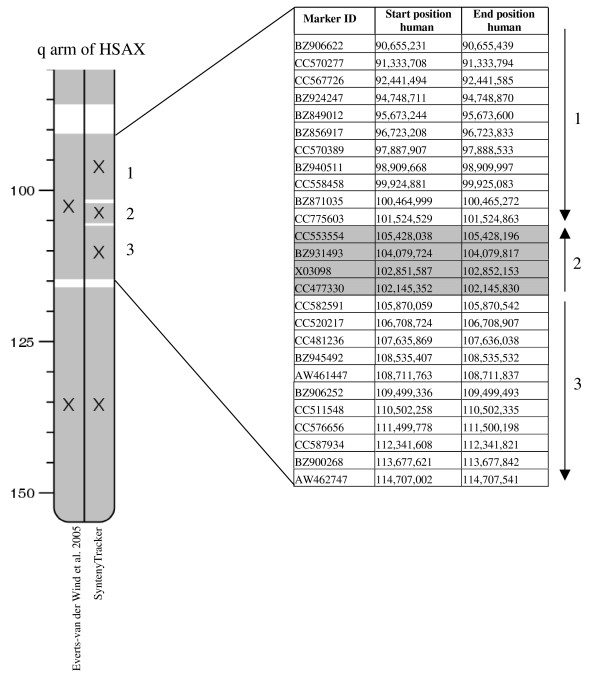
**An inversion of CC553554, BZ931493, and X03098 markers (3.2 human-Mb) on BTAX was identified by SyntenyTracker but not by manual analysis **[4].

We tested SyntenyTracker with several datasets, including a cattle-human RH comparative map comprised of 3,204 markers [4], and a dataset containing 14,380 orthologous gene pairs with one-to-one relationships between the human and mouse genomes (Ensembl release 42). For the cattle-human comparative map, among the 196 HSBs defined by SyntenyTracker, 189 HSBs completely match HSBs manually defined by Everts-van der Wind and coworkers [4] using the same set of rules that we implemented in SyntenyTracker (see additional file 2 for SyntenyTracker output compared to manually defined HSBs). On BTA16, SyntenyTracker combined two HSBs defined by Everts-van der Wind and coworkers [4]. In this case, singleton markers interrupting the HSBs were ignored by SyntenyTracker according to predefined settings [1]. In two cases, on BTA25 and BTA26, blocks of "out-of-place" markers (for definition of "out-of-place" marker see additional file 1) defined as HSBs by Everts-van der Wind et al. [4] were ignored by SyntenyTracker. In Everts-van der Wind et al. [4] two HSBs were defined in region 0–832 map units on BTA3 because of two closely linked "out-of-place" markers that mapped to BTA16. SyntenyTracker combined these two HSBs ignoring the "out-of-place" markers according to the rule that does not allow "out-of-place" markers to break other HSBs. Similarly, two HSBs on BTA5 and another two on BTA15 were merged. On BTAX, SyntenyTracker detected a missing inversion defined by three consecutive markers (CC553554, BZ931493, X03098) and identified three HSBs whereas Everts-van der Wind and coworkers found one (Figure 2). Thus, SyntenyTracker is useful for identifying errors made by manual assignment using predefined rules [1,4].

To verify the quality of HSB definition by SyntenyTracker we selected another tool that was designed to work with radiation hybrid comparative maps for a detailed comparison. Among many synteny-defining tools we found that only AutoGRAPH [6] was designed to work with RH comparative maps. Other popular tools, e.g. GRIMM-Synteny [7] were made to work with sequenced genomes and were not suitable for the comparison.

To define "conserved segments ordered" (CSO), the equivalent of HSBs AutoGRAPH first assigns a numerical integer to the markers in both reference and tested genomes and calculates adjacency penalties between consecutive markers on tested genomes. AutoGRAPH breaks a CSO if the adjacency penalty exceeds the penalty chosen by the user. The main difference with SyntenyTracker definition of HSBs is that SyntenyTracker checks the size of inversions and compares them to the threshold selected by the user. The number of markers in an inversion cannot be less than 3. (See additional file 1 for rules for defining HSBs). A change in the order of markers caused by single marker is ignored by SytenyTracker unlike AutoGRAPH. In addition SyntenyTracker checks if there are any markers in other reference chromosomes that could interrupt the order of the markers in an HSB.

Using the same comparative map dataset we performed comparison of HSB definitions made by SyntenyTracker with analogous CSO defined by AutoGRAPH [6]. Using AutoGRAPH with default parameters, we were not able to define HSBs for the whole-genome dataset due to limitations in the web application. To run the comparison with AutoGRAPH we had to break our dataset into smaller datasets, each corresponding to an individual reference genome chromosome. Therefore, to define HSBs on the cattle-human RH comparative map, AutoGRAPH was run 30 times for each of the 30 reference chromosomes. This resulted in definition of 180 HSBs (see additional file 2: Table S1 for HSBs defined manually, with SyntenyTracker and AutoGRAPH; additional file 2: Table S2 for complete summary of the differences in SyntenyTracker and AutoGRAPH HSB definitions). In 10 cases, AutoGRAPH combined two HSBs defined by SyntenyTracker because of an interrupting HSB that was located on another reference chromosome. On BTA2 and BTAX, inversions defined by more than three consecutive markers were unaccounted for by AutoGRAPH. In four cases AutoGRAPH did not account for markers mapped to the same positions on the RH map, resulting in deletion of four HSBs. In one case (on BTA26) AutoGRAPH defined two "out-of-place" markers as an HSB. In two additional cases HSBs were broken because of the presence of singleton markers.

We then compared AutoGRAPH and SyntenyTracker definitions of HSBs on the same set of orthologous genes in the human and mouse genomes. The set of 14,380 othologous markers was analyzed in one run with SyntenyTracker and in 23 runs by AutoGRAPH. SyntenyTracker defined 313 HSBs, whereas AutoGRAPH defined 358 (see additional file 3: Table S1 for the list of HSBs defined by SyntenyTracker and AutoGRAPH on the set of human-mouse orthologous markers, and additional file 3: Table S2 for the summary of discrepancies in SyntenyTracker and AutoGRAPH HSB definition). The majority of discrepant cases can be grouped into 3 categories. The first category includes cases when AutoGRAPH broke HSBs defined by SyntenyTracker because of a single marker from other regions of the same or other orthologous chromosomes positioned within these regions. The second category includes cases when SyntenyTracker ignores small inversions because of default parameters that require 3 consecutive markers ≥300 Kb apart to define orientation of the block. The first two categories of discrepancies in HSB definitions between SyntenyTracker and AutoGRAPH can be explained by small differences in the rules used by these two tools and can be avoided by adjusting HSB definition parameters in either of the tools. The last category includes cases when AutoGRAPH joined HSBs defined by SyntenyTracker. For such cases an interrupting HSB was located on another reference chromosome, and therefore genuine chromosomal rearrangements were likely missed using AutoGRAPH. This can only be fixed by changing the algorithm of the tool to work with the whole-genome set rather than with an individual chromosome (see additional file 3: Table S2 for the list of discrepancies).

We investigated all 15 cases of singleton gene markers that caused AutoGRAPH to break the HSBs. The same markers were ignored by SyntenyTracker. For these 15 genes we examined consistency of orthologous relationships in different builds of the human and mouse genomes. For 10 of 15 genes we found inconsistency in the definition of human-mouse orthology pairs in different genome builds or annotation sources, indicating a problem in defining 1 to 1 orthology between these human genes and their mouse counterparts (see additional file 3: Table S3 for the list of discrepancies and results of orthology analysis).

To verify that the SyntenyTracker algorithm is robust and that the results obtained from its use are not affected by modifications to the input file that do not change the comparative map, we have done the following tests: a) the order of target genome chromosomes in the original human-mouse orthologous gene input file was changed; b) the order of markers within both reference and target chromosomes was inverted. HSBs defined using such modified input files were compared to the HSBs defined using the original input file. All HSBs from the modified input completely matched original HSBs.

To examine how SyntenyTracker accounts for the uncertainties in the order of markers on the RH comparative map when several markers are mapped to exactly the same position on the RH map, we changed the order of such markers on the human-cattle comparative map [4], defined HSBs, and compared them to the HSBs defined from the original map. No differences in HSB definition were detected.

## Conclusion

SyntenyTracker is able to define HSBs on whole-genome comparative data following the set of rules defined by Murphy and coworkers [1]. We demonstrated that SyntenyTracker identifies HSBs with high accuracy and is useful for the identification of errors in HSB definition made during manual annotation. Compared to the AutoGRAPH synteny block definition tool, SyntenyTracker demonstrated higher quality of HSB definition in those cases when proper definition of an HSB was dependent on simultaneous analysis of several reference and target chromosomes. Also, SyntenyTracker does not define breakpoints supported by only one (or two very closely located) markers because these markers may not represent truly orthologous anchors between genomes. SyntenyTracker is thus a powerful tool for multispecies comparative genome analysis and will have increased utility as more mammalian genomes are mapped and sequenced.

## Availability and requirements

**Project name**: SyntenyTracker

**Project home page**: 

**Operating system(s)**: Platform independent (web-based application)

**Programming language**: Perl, CGI-Perl

**Other requirements**: A web browser

**License**: None for usage

**Any restrictions to use by non-academics**: None

## Competing interests

The authors declare that they have no competing interests.

## Authors' contributions

RD designed, implemented and tested the algorithm, and drafted the manuscript. DML and HAL supervised the work. All authors read and approved the final manuscript.

## Supplementary Material

Additional file 1 **Explanation of HSB definition rules and SyntenyTracker algorithm**. This file describes the rules for HSB definition implemented in SyntenyTracker, as well as the algorithm SyntenyTracker follows and contains supplementary tables and figures illustrating how the algorithm works. Click here for file

Additional file 2 **Definition of HSBs on the cattle-human radiation hybrid comparative map**. Comparison of HSBs defined manually, with SyntenyTracker and AutoGRAPH on the cattle-human radiation hybrid map dataset [4]. Click here for file

Additional file 3 **Definition of HSBs on a set of human and mouse orthologs**. Comparison of HSB definitions between SyntenyTracker and AutoGRAPH on a set of human and mouse orthologs. Click here for file
