# Supplementary material for: SyntenyTracker: a tool for defining homologous synteny blocks using radiation hybrid maps and whole-genome sequence
Source: BMC Res Notes. 2009 Jul 23;2:148. doi: 10.1186/1756-0500-2-148 (PMC2726151; doi:10.1186/1756-0500-2-148)
Supplement: Additional file 1 — Explanation of HSB definition rules and SyntenyTracker algorithm. This file describes the rules for HSB definition implemented in SyntenyTracker, as well as the algorithm SyntenyTracker follows and contains supplementary tables and figures illustrating how the algorithm works. [file 1756-0500-2-148-S1.pdf]

Supplementary information:

Input file

As input, SyntenyTracker uses a tab-delimited file containing information about chromosome assignment of orthologous markers in two genomes, position of each marker in the chromosomes of both genomes, and marker identifiers. The markers in the input table are sorted based on their chromosome assignments and positions in one of the two genomes. This genome is termed as the "reference genome". The second genome is called the "target genome" (Table 1). For each marker an input row contains "marker id", "reference species chromosome", "target species chromosome" and chromosome starting and ending sequence coordinates in the reference and target species chromosome.

Definitions and rules for HSB identification

To facilitate the identification of HSBs and minimize the level of errors resulting from erroneous marker placements in RH maps and errors in the identification of orthologous markers in the reference and target genomes, a set of definitions and rules described previously [1] was followed.

Definitions:

1. Homologous synteny block (HSB): An HSB is a set of consecutive orthologous markers $A = (a_1,\ldots, a_n)$ with $n \geq 2$ in homologous regions of the reference R(A) and target R\`(A) chromosomes respectively. No other HSB B exists on the comparative map which contains a marker b with coordinates c(b) and c\`(b) for which R(A) = R(B), R\`(A) = R\`(B), $c(b) \geq c(a_1)$ and $c(b) \leq c(a_n)$, $c`(b) \geq c`(a_1)$, and $c`(b) \leq c`(a_n)$ except for the cases described in rules 1 and 2.

2. An internal rearrangement (inversion): An internal rearrangement (inversion) is defined by a minimum of three consecutive markers m_1, m_2, m_3 with the coordinates in the reference chromosome $c(m_1)$, $c(m_2)$, $c(m_3)$ and target chromosome $c`(m_1)$, $c`(m_2)$, $c`(m_3)$ for which $c`(m_1) < c`(m_2) < c`(m_3)$ or $c`(m_1) > c`(m_2) > c`(m_3)$, $|c`(m_1) - c`(m_2)| \geq Th$ (user-defined threshold distance) and $|c`(m_2) - c`(m_3)| \geq Th$.
3. A comparative singleton: A comparative singleton is a single marker x with coordinates in the reference $c(x)$ and target $c`(x)$ genome orthologous chromosomes, and not a part of any HSB.

Rules:

1. A comparative singleton (x) can be moved to its expected position $c`_{ex}(x)$ within an HSB $B=(m_1,...,m_n)$ if the chromosome $R(x) = R(B)$, $R`(x) = R`(B)$, $c`(m_j) < c`_{exp}(x) < c`(m_{j+1})$ or $c`(m_j) > c`_{exp}(x) > c`(m_{j-1})$ and $|c`_{exp}(x) – c(x)| \leq Th$.
2. Any two consecutive singleton markers m_i and m_{i+1} for which chromosomes $R(m_i) = R(m_{i+1})$ and $R`(m_i) = R`(m_{i+1})$ with the distance $|c`(m_i) - c`(m_{i+1})| \leq Th$ are together treated as a comparative singleton marker, and are allowed to move to their expected positions within an HSB B, if $|c`_{exp}(m_i) – c`(m_i)| \leq Th$ or $| c`_{exp}(m_{i+1})- c`(m_{i+1})| \leq Th$ and $R(m_i) = R(B)$ and $R`(m_i) = R`(B)$.

Implementation

Step 1: Within the target genome chromosomes, the markers that are located more than a user-defined threshold distance from the neighboring markers are flagged. Also, all markers with the chromosome assignments different from the markers immediately upstream in either the target or reference genomes are flagged. The algorithm assigns a temporary block number to all sets of

consecutive markers starting with a flagged marker and ending with a marker that immediately precedes the next flagged marker.

Step 2: All the blocks defined in Step 1 are scanned using a sliding window of 5 blocks. Whenever the third block of a window consists of a single marker or two markers whose positions are separated by less than a user-defined threshold distance (1 Mbp in our analysis) in the target genome chromosome, a search is performed to locate the expected position of the marker(s) within the other 4 blocks. In Table 2, the position between the marker positions 121.4 and 123.5 is the expected position of the marker at position 122.2. The expected position is less than the user-defined threshold distance (2 Mbp in our analysis) from the current position of the marker in the target chromosome. Also, there is no other block from a different chromosomal region of the same or different reference chromosome that falls in between the expected and current position of the marker. Therefore, the single marker is allowed to move to the expected position. A single marker or two markers with their positions separated by a user-defined threshold distance in the target genome that fail to move to their expected positions are classified as “out-of-place” marker(s).

Step 3: The orientation of the temporary blocks in the target genome chromosomes relative to the reference genome chromosomes is defined by finding all consecutive markers within each block defined in step 2 that are separated by a user-defined distance in the target genome chromosome divided by three and comparing their relative order within the target genome chromosome. If the markers are found in ascending order in the target genome chromosome the block is assigned a positive orientation; if they are in descending order, the orientation of the block is defined as

negative. In some cases the orientation of the block cannot be defined. In Table 3, marker positions 32.0, 34.5, 36.0, and 38.0 were selected. Increasing order of 2 consecutive markers defines a positive orientation, and decreasing order of 2 consecutive markers defines a negative orientation. Marker positions 32.0 and 38.0, 34.5 and 36.0 provide an evidence for positive orientation of the block, resulting in positive orientation for the whole block. In Table 4a, there are an equal number of evidences for positive (between marker positions 71.0 and 81.0) and negative (between marker positions 76.0 and 75.0) orientation. In such cases, the remaining unselected markers of the block are also selected and are examined for the evidence of orientation as previously described (Table 4b). In Table 5a, all the markers of the block are selected, and still there is an equal amount of evidences for positive (between marker positions 89.2 and 92.3) and negative (between marker positions 90.2 and 88.0) orientation. In such cases, all consecutive pairs of markers are checked for the orientation (Table 5b). In the "Radiation Hybrid" mode additional checks are performed for the "flips" of two consecutive markers. Marker positions, 89.2 and 90.2, 90.2 and 91.2, 91.2 and 88.0, 88.0 and 92.3 provide three evidences for positive orientation, and single evidence for negative orientation, resulting in positive orientation for the block. When there are an equal number of evidences for both positive and negative orientations, the algorithm checks if the block contains at least 3 consecutive markers satisfying definition no. 2 and defines the orientation of the whole block based on positions of the first and last marker in target chromosome.

Step 4: Blocks with undefined orientation will be merged with the closest end of the upstream or downstream blocks if the closest end of the upstream or downstream block is not farther than the user-defined block length, if both blocks are found on the same reference and target genome

chromosomes, and no other blocks above the user-defined threshold size for “block length” are located between them on the target chromosome. In Table 6, the orientation of block 67 is undefined, and is closer to block 66, so these two blocks are merged.

Step 5: The blocks formed in step 4 could be extended in another scan with a sliding window of two blocks. Blocks with defined orientation will be merged with adjacent block on the target genome chromosome, if both blocks have the same orientation in the target genome chromosome, the order of markers in these blocks taken together is contiguous, and no other blocks above the user-defined block length are located in between them on the target chromosome. In Table 7, a window of two blocks is shown. The orientation of both blocks is positive, and both blocks have markers from BTA15 and HSA11, and no blocks from a different chromosome region in the target genome fall in between. Marker positions 37.0 and 42.0 provide an evidence for positive orientation, so these two blocks are merged.

Step 6: Finally, an automated checking is performed to identify any blocks above the block length threshold that overlap with larger blocks. When such blocks are found, the larger blocks will be broken into a set of smaller blocks to avoid HSB overlaps. In Table 8, the marker positions 25.0, 26.0, 26.5, and 27.3 fall in between the marker positions 23.0 and 28.0 of block 49. To avoid the overlap, block 49 is broken into two blocks. In further scans, the blocks thus formed could be extended as described previously by merging with neighboring blocks. At the end of all the steps, the blocks are considered as HSBs.

Pseudocode of algorithm implementation

The input file contains information about the orthologous markers ($m_1,\ldots, m_n$) in both the reference and target genomes. The table contains the information about the reference and target genome chromosome numbers R_i and $R`_i$ respectively, and positions of the markers in both genome chromosomes, $c(m_i)$ for the reference and $c`(m_i)$ for the target chromosome. The markers on the comparative map are sorted based on their order in the reference chromosomes $c(m_{i-1}) < c(m_i)<c(m_{i+1})$. The program scans the coordinates of markers, $(c`(m_1),\ldots, c`(m_n))$ and if $|c`(m_{i-1}) - c`(m_i)| \geq Th$ (user-defined threshold distance), marker m_i is flagged. Also, for all cases for which $R(m_{i-1}) \neq R(m_i)$ or $R`(m_{i-1}) \neq R`(m_i)$ marker m_i is flagged. A temporary block number B_i is assigned to a subset of markers ($m_{j,}$ . . ., m_q), where m_j is flagged marker and m_{q+1} is next flagged marker. After that the program scans all temporary blocks using a sliding window of 5 blocks ($B_{i-2}, B_{i-1}, B_i, B_{i+1}, B_{i+2}$). If block B_i is a singleton marker with position $c`(B_i)$, the program checks for the expected position of the marker in the other 4 blocks. The singleton can be moved to its expected position $c`_{exp}(B_i)$ within another block $B_k = (m_j,...,m_q)$ if the chromosome $R(B_i) = R(B_k)$, $R`(B_i) = R`(B_k)$, and $c`(m_m)<c`_{exp}(B_i)<c`(m_{m+1})$ or $c`(m_m) > c`_{exp}(B_i) > c`(m_{m-1})$ and $|c`_{exp}(B_i) – c(B_i)| \leq Th$ (user-defined threshold distance). To define an orientation for a block, $B_i = (m_j, . . ., m_q)$, there should be three consecutive markers, $m_k$, $m_m$, $m_o$, for which $|c`(m_k)-c`(m_m)| > Th$ and $|c`(m_m)-c`(m_o)| > Th$. The orientation of the block is defined as positive, if $c`(m_k) < c`(m_m) < c`(m_o)$, and negative if $c`(m_k) > c`(m_m) > c`(m_o)$. A block $B_k = (m_j,...,m_q)$ for which the orientation could not be determined is merged with the closest end of block $B_{k-1} = (m_c,...,m_{j-1})$, if $|c`(m_j) – c`(m_{j-1})| \leq Th$ or $| c`(m_j) – c`(m_c)| \leq Th$ or $|c`(m_q) – c`(m_{j-1})| \leq Th$ or $| c`(m_q) – c`(m_c)| \leq Th$, $R(B_{k-1}) = R(B_k)$ and $R`(B_{k-1}) = R`(B_k)$ or with block $B_{k+1}=(m_{q+1},...,m_t)$ if $|c`(m_q) – c`(m_{q+1})| \leq Th$ or $| c`(m_q) – c`(m_t)| \leq Th$ or $|c`(m_j) – c`(m_{q+1})| \leq Th$ or $| c`(m_j) – c`(m_t)| \leq Th$, and $R(B_{k+1}) =$

$R(B_k)$ and $R`(B_{k+1}) = R`(B_k)$. Thus a new block, $B = (m_c,\ldots,m_{j-1}, m_j,\ldots,m_q)$ or $B = (m_j,\ldots,m_q, m_{q+1},\ldots,m_t)$ is formed. The algorithm checks if there are any two adjacent blocks $B_k = (m_a,\ldots,m_g)$ and $B_{k+1} = (m_o,\ldots,m_v)$ with the same orientation and $R(B_k) = R(B_{k+1})$, $R`(B_k)= R`(B_{k+1})$ for which $c`(m_a) < c`(m_g) < c`(m_o) < c`(m_v)$, and merges them into a new block, $B=(m_a,\ldots,m_g, m_o,\ldots,m_v)$, if a block $D=(m_{g+1},\ldots,m_{o-1})$ does not exist. The program breaks any overlapping blocks $B=(m_j,\ldots,m_q)$ and $C=(m_l,\ldots,m_o)$ if $|c`(m_l)–c`(m_o)| > Th$ into smaller blocks $B^* = (m_j,\ldots,m_{l-1})$ and $B^{**} = (m_{o+1},\ldots,m_q)$. Finally, the program classifies remaining single marker blocks. If B_i is a singleton block and there is no HSB $A = (m_j,\ldots,m_q)$ on the whole map for which $R(B_i) = R(A)$, $R`(B_i) = R`(A)$ and $c`(B_i) \geq c`(m_j)$ and $c`(B_i) \leq c`(m_q)$, B_i is classified as the singleton marker. If there is an HSB $A= (m_j,\ldots,m_q)$ on the comparative map for which $R(B_i) = R(A)$, $R`(B_i) = R`(A)$ and $c`(B_i) \geq c`(m_j)$ and $c`(B_i) \leq c`(m_q)$, then B_i is classified as the “out-of-place” marker.

REFERENCES

1. Murphy WJ, Larkin DM, Everts-van der Wind AE, Bourque G, Tesler G, Auvil L, Beever JE, Chowdhary BP, Galibert F, Gatzke L, Hitte C, Meyers SN, Milan D, Ostrander EA, Pape G, Parker HG, Raudsepp T, Rogatcheva MB, Schook LB, Skow LC, Welge M, Womack JE, O'Brien SJ, Pevzner PA, Lewin HA: **Dynamics of mammalian chromosome evolution inferred from multispecies comparative maps.** *Science* 2005, **309:**613-617.

Table 1. The structure of SyntenyTracker input file.

HSA (Target)	Human start (Mb)	Human end (Mb)	Marker ID	BTA (Reference)	Cattle start (cR)	Cattle end (cR)
12	104.0	104.8	1	5	23	23
12	105.0	105.7	2	5	28	28
12	106.0	106.9	3	5	33	33
12	107.0	107.3	4	5	38	38
22	31.0	31.3	5	5	39	39
22	31.7	31.9	6	5	41	41
22	32.0	32.3	7	5	43	43
22	33.0	33.5	8	5	46	46
22	38.4	39.0	9	5	49	49
22	39.8	40.5	10	5	52	52
22	41.4	41.9	11	5	56	56

Table 2. Implementation of the rule no. 2.

Marker ID	BTA	HSA	Human coord (Mb)	Block
1	15	11	119.0	107
2	15	11	121.0	107
3	15	11	121.4	107
4	15	11	123.5	107
5	15	2	181.8	108
6	15	2	180.4	108
7	15	11	122.2	107
8	15	2	179.9	108
9	15	2	177.1	108

Table 3. Example of a block with positive orientation.

Marker ID	BTA	HSA	Human coord (Mb)	Block	Orientation
1	15	11	32.0	109	+
2	15	11	32.2	109	+
3	15	11	34.5	109	+
4	15	11	34.9	109	+
5	15	11	36.0	109	+
6	15	11	38.0	109	+

Markers highlighted gray are separated by the distance ≥ 1Mbp

Table 4a. An alternative approach to the orientation identification.

Marker ID	BTA	HSA	Human coord (Mb)	Block	Orientation
1	15	11	71.0	56	+
2	15	11	71.2	56	+
3	15	11	76.0	56	+
4	15	11	75.0	56	+
5	15	11	81.0	56	+
6	15	11	81.8	56	+

Markers highlighted gray are separated by the distance ≥ 1Mbp

Table 4b. An alternative approach to the orientation identification.

Marker ID	BTA	HSA	Human coord (Mb)	Block	Orientation
1	15	11	71.0	56	+
2	15	11	71.2	56	+
3	15	11	76.0	56	+
4	15	11	75.0	56	+
5	15	11	81.0	56	+
6	15	11	81.8	56	+

Table 5a. A block whose orientation was defined.

Marker ID	BTA	HSA	Human coord (Mb)	Block	Orientation
1	15	11	89.2	105	+
2	15	11	90.2	+105	+
3	15	11 -	91.2	105	+
4	15	11	88.0	105	+
5	15	11	92.3	105	+

Markers highlighted gray are separated by the distance ≥ 1Mbp

Table 5b. A block whose orientation was defined.

Marker ID	BTA	HSA	Human coord (Mb)	Block	Orientation
1	15	11	89.2	105	+
2	15	11	90.2	105	+
3	15	11	91.2	105	+
4	15	11	88.0	105	+
5	15	11	92.3	105	+

Table 6. A block with undefined orientation is merged with a neighboring block.

Marker ID	BTA	HSA	Human coord (Mb)	Block		Block
4	15	11	23.5	66		66
5	15	11	24.2	66		66
6	15	11	25.0	66		66
7	15	11	28.0	66		66
8	15	11	32.1	67	→	66
9	15	11	32.0	67		66
10	15	11	29.0	67		66
11	15	11	133.0	68		67
12	15	11	135.0	68		67
13	15	11	137.0	68		67
14	15	11	137.2	68		67

Table 7. Two consecutive blocks merged.

Marker ID	BTA	HSA	Human coord (Mb)	Orientation	Block
1	15	11	32.0	+	10
2	15	11	33.0	+	10
3	15	11	36.0	+	10
4	15	11	37.0	+	10
5	15	11	42.0	+	11
6	15	11	43.0	+	11
7	15	11	46.0	+	11
8	15	11	48.0	+	11

→

Block
10
10
10
10
10
10
10
10

Table 8. An example where a block is broken into two blocks, because of a sub-block.

Marker ID	BTA	HSA	Human coord (Mb)	Block	Orientation
1	15	11	21.0	49	+
2	15	11	22.0	49	+
3	15	11	23.0	49	+
4	15	11	28.0	49	+
5	15	11	29.0	49	+
6	15	11	31.0	49	+

Marker ID	BTA	HSA	Human coord (Mb)	Block	Orien
1	15	11	25.0	12	+
2	15	11	26.0	12	+
3	15	11	26.5	12	+
5	15	11	27.3	12	+

Marker ID	BTA	HSA	Human coord (Mb)	Block	Orien
1	15	11	21.0	49	+
2	15	11	22.0	49	+
3	15	11	23.0	49	+

Marker ID	BTA	HSA	Human coord (Mb)	Block	Orien
4	15	11	28.0	50	+
5	15	11	29.0	50	+
6	15	11	31.0	50	+
