# Supplementary material for: SyntenyTracker: a tool for defining homologous synteny blocks using radiation hybrid maps and whole-genome sequence
Source: BMC Res Notes. 2009 Jul 23;2:148. doi: 10.1186/1756-0500-2-148 (PMC2726151; doi:10.1186/1756-0500-2-148)
Supplement: Additional file 2 — Definition of HSBs on the cattle-human radiation hybrid comparative map. Comparison of HSBs defined manually, with SyntenyTracker and AutoGRAPH on the cattle-human radiation hybrid map dataset [4]. [file 1756-0500-2-148-S2.pdf]

Table 1. Comparison of HSBs defined manually, with SyntenyTracker and AutoGRAPH (Derrien et al., 2007) on the cattle-human radiation hybrid map dataset (Everts-van der Wind et al., 2005).

Marker_order	HSA	HSA_start	HSA_end	Marker_name1	Marker_name2	Marker_name3	BTA	BTA_start	BTA_end	HSB_Everts_van_der_Wind_et_al_2005	HSB_SyntenyTracker	HSB_AutoGRAPH	Comment	Comment	Comment
1	21	34874409	34874774	CC475154	CC475154	CC475154	1	0	0	1	1	1			
2	21	33818882	33819247	BZ898554	BZ898554	BZ898554	1	12	12	1	1	1			
4	21	32563218	32563413	BZ928253	BZ928253	BZ928253	1	40	40	1	1	1			
5	21	31329689	31330250	BZ889881	BZ889881	BZ889881	1	43	43	1	1	1			
6	21	30257610	30257736	CC528091	CC528091	CC528091	1	46	46	1	1	1			
7	21	29553152	29553688	CC531314	CC531314	CC531314	1	53	53	1	1	1			
8	21	28639072	28639466	CC773677	CC773677	CC773677	1	56	56	1	1	1			
9	21	27195477	27195617	BZ858731	BZ858731	BZ858731	1	56	56	1	1	1			
10	21	25953160	25953442	BZ902891	BZ902891	BZ902891	1	76	76	1	1	1			
11	21	24727388	24727556	BZ932218	BZ932218	BZ932218	1	76	76	1	1	1			
12	21	23442541	23443006	BZ870005	BZ870005	BZ870005	1	79	79	1	1	1			
13	21	22208999	22209056	CC546846	CC546846	CC546846	1	79	79	1	1	1			
14	21	21152220	21152771	CC479207	CC479207	CC479207	1	82	82	1	1	1			
15	21	20554234	20554534	BZ870492	BZ870492	BZ870492	1	82	82	1	1	1			
16	21	19613515	19613665	CC533925	CC533925	CC533925	1	106	106	1	1	1			
17	21	18636937	18637293	CC580618	CC580618	CC580618	1	110	110	1	1	1			
18	21	17795453	17795618	BZ926663	BZ926663	BZ926663	1	116	116	1	1	1			
20	21	16879052	16879243	BZ864060	BZ864060	BZ864060	1	127	127	1	1	1			
21	21	15860717	15861098	BZ857194	BZ857194	BZ857194	1	133	133	1	1	1			
22	21	14736544	14736678	CC485525	CC485525	CC485525	1	140	140	1	1	1			
23	21	14437358	14437438	BZ942230	BZ942230	BZ942230	1	144	144	1	1	1			
24	3	76402003	76402248	BZ939604	BZ939604	BZ939604	1	158	158	2	2	2			
25	3	77749353	77749574	AW653888	AW653888	AW653888	1	172	172	2	2	2			
26	3	78446058	78446781	BZ923868	BZ923868	BZ923868	1	172	172	2	2	2			
27	3	79500395	79500804	BZ916136	BZ916136	BZ916136	1	179	179	2	2	2			
28	3	80376296	80376901	BZ905406	BZ905406	BZ905406	1	190	190	2	2	2			
29	3	81646312	81646665	BZ956569	BZ956569	BZ956569	1	210	210	2	2	2			
30	3	82669360	82669758	BZ934143	BZ934143	BZ934143	1	230	230	2	2	2			
31	3	83789264	83789561	BZ919346	BZ919346	BZ919346	1	252	252	2	2	2			
32	3	83986114	83986427	CC472704	CC472704	CC472704	1	252	252	2	2	2			
33	3	85215902	85216070	BZ923016	BZ923016	BZ923016	1	270	270	2	2	2			
34	3	86495827	86496166	BZ935163	BZ935163	BZ935163	1	287	287	2	2	2			
35	3	87391643	87391947	X12657	X12657	X12657	1	295	295	2	2	2			
36	3	88568289	88568877	BZ933336	BZ933336	BZ933336	1	302	302	2	2	2			
37	3	90057915	90058187	BZ950059	BZ950059	BZ950059	1	302	302	2	2	2			
38	3	95075473	95075942	X12891	X12891	X12891	1	306	306	2	2	2			
39	3	95991693	95991736	BZ899514	BZ899514	BZ899514	1	310	310	2	2	2			
40	3	96706758	96707531	CC519730	CC519730	CC519730	1	310	310	2	2	2			
41	3	97780716	97781135	BZ896609	BZ896609	BZ896609	1	314	314	2	2	2			
42	3	98806326	98806583	BZ920963	BZ920963	BZ920963	1	314	314	2	2	2			
43	3	99766469	99767005	BZ945344	BZ945344	BZ945344	1	318	318	2	2	2			
44	3	100844087	100844504	BZ942331	BZ942331	BZ942331	1	326	326	2	2	2			
45	3	101896188	101896347	BZ949524	BZ949524	BZ949524	1	329	329	2	2	2			
46	3	103053609	103053855	BZ899633	BZ899633	BZ899633	1	333	333	2	2	2			
47	3	104088922	104089278	BZ943464	BZ943464	BZ943464	1	333	333	2	2	2			
48	3	105589832	105590157	BZ917443	BZ917443	BZ917443	1	333	333	2	2	2			
50	3	106813926	106814320	BZ910182	BZ910182	BZ910182	1	347	347	2	2	2			
52	3	108067548	108067685	BZ935101	BZ935101	BZ935101	1	360	360	2	2	2			
53	3	109010881	109011129	AW352867	AW352867	AW352867	1	367	367	2	2	2			
54	3	109680322	109680553	BZ903805	BZ903805	BZ903805	1	374	374	2	2	2			
55	3	110537750	110537872	BZ922952	BZ922952	BZ922952	1	374	374	2	2	2			
56	3	111592744	111593108	BZ918043	BZ918043	BZ918043	1	374	374	2	2	2			
57	3	112851236	112851502	AW289280	AW289280	AW289280	1	432	432	2	2	2			
58	3	113840249	113840584	BZ920071	BZ920071	BZ920071	1	435	435	2	2	2			
59	3	115105108	115105604	BZ916765	BZ916765	BZ916765	1	442	442	2	2	2			
60	3	116254635	116254797	BZ952772	BZ952772	BZ952772	1	453	453	2	2	2			
61	3	117341236	117341464	BZ945369	BZ945369	BZ945369	1	458	458	2	2	2			
62	3	118474552	118474682	BZ934678	BZ934678	BZ934678	1	458	458	2	2	2			
63	3	119539138	119539698	BZ910495	BZ910495	BZ910495	1	464	464	2	2	2			
64	3	120585697	120585745	BZ949712	BZ949712	BZ949712	1	466	466	2	2	2			
65	3	120871134	120871447	AW289252	AW289252	AW289252	1	466	466	2	2	2			
66	3	121645368	121645650	BZ902109	BZ902109	BZ902109	1	469	469	2	2	2			
67	3	122608726	122608826	BZ935559	BZ935559	BZ935559	1	475	475	2	2	2			
68	3	123380971	123381064	BZ926006	BZ926006	BZ926006	1	477	477	2	2	2			
69	3	123613967	123614091	BF440429	BF440429	BF440429	1	477	477	2	2	2			
70	3	124303786	124304166	AW289373	AW289373	AW289373	1	477	477	2	2	2			
71	3	125454098	125454189	BZ951965	BZ951965	BZ951965	1	488	488	2	2	2			
72	3	126179249	126179596	AW289285	AW289285	AW289285	1	494	494	2	2	2			
73	3	126740087	126740494	BZ911166	BZ911166	BZ911166	1	502	502	2	2	2			
74	3	198727213	198727572	BZ896764	BZ896764	BZ896764	1	509	509	3	3	3			
75	3	198145897	198146397	AW289257	AW289257	AW289257	1	509	509	3	3	3			
76	3	197447527	197447713	BZ906058	BZ906058	BZ906058	1	512	512	3	3	3			
77	3	196722729	196722862	AW315482	AW315482	AW315482	1	514	514	3	3	3			
78	3	196326813	196326977	BZ943583	BZ943583	BZ943583	1	517	517	3	3	3			
79	3	195176358	195176565	BZ927156	BZ927156	BZ927156	1	517	517	3	3	3			
80	3	194419498	194419596	BZ906031	BZ906031	BZ906031	1	517	517	3	3	3			
81	3	193499608	193499664	BZ925622	BZ925622	BZ925622	1	517	517	3	3	3			
82	3	192463094	192463305	BZ924583	BZ924583	BZ924583	1	519	519	3	3	3			
83	3	191034472	191035077	BZ956529	BZ956529	BZ956529	1	525	525	3	3	3			
84	3	190373036	190373078	BZ920927	BZ920927	BZ920927	1	527	527	3	3	3			
86	3	189400957	189401007	BZ901844	BZ901844	BZ901844	1	572	572	3	3	3			
87	3	188196782	188196841	BZ916894	BZ916894	BZ916894	1	579	579	3	3	3			
88	3	187813628	187813841	X16577	X16577	X16577	1	579	579	3	3	3			
89	3	186423942	186424161	BZ901112	BZ901112	BZ901112	1	581	581	3	3	3			
90	3	185588306	185588576	CC533049	CC533049	CC533049	1	584	584	3	3	3			
91	3	184652195	184652549	BZ844541	BZ844541	BZ844541	1	586	586	3	3	3			
92	3	184089897	184089987	CC524102	CC524102	CC524102	1	589	589	3	3	3			
93	3	183741105	183741487	BZ935969	BZ935969	BZ935969	1	589	589	3	3	3			
94	3	182899029	182899211	CC556340	CC556340	CC556340	1	594	594	3	3	3			
95	3	181606651	181606836	BZ941829	BZ941829	BZ941829	1	602	602	3	3	3			
96	3	180906825	180907209	CC490444	CC490444	CC490444	1	613	613	3	3	3			
97	3	180353246	180353374	BZ944850	BZ944850	BZ944850	1	616	616	3	3	3			
98	3	179374630	179374955	CC494841	CC494841	CC494841	1	621	621	3	3	3			
99	3	178731947	178731983	CC481705	CC481705	CC481705	1	628	628	3	3	3			
100	3	177896534	177896947	BZ926449	BZ926449	BZ926449	1	639	639	3	3	3			
101	3	176865145	176865825	BZ944372	BZ944372	BZ944372	1	647	647	3	3	3			
102	3	175514660	175515091	BZ927934	BZ927934	BZ927934	1	647	647	3	3	3			
103	3	174673453	174673560	BZ941618	BZ941618	BZ941618	1	650	650	3	3	3			
104	3	173364522	173364689	BZ944088	BZ944088	BZ944088	1	653	653	3	3	3			
105	3	172498034	172498699	BZ947793	BZ947793	BZ947793	1	659	659	3	3	3			
107	3	172293818	172294049	BF440275	BF440275	BF440275	1	673	673	3	3	3			
108	3	171194496	171194650	AW289224	AW289224	AW289224	1	678	678	3	3	3			
109	3	170201969	170202390	BZ952238	BZ952238	BZ952238	1	686	686	3	3	3			
110	3	169057906	169058360	BZ908762	BZ908762	BZ908762	1	698	698	3	3	3			
111	3	167697446	167697545	BZ918002	BZ918002	BZ918002	1	703	703	3	3	3			
112	3	166378807	166379219	BZ945644	BZ945644	BZ945644	1	709	709	3	3	3			
113	3	165175528	165175867	BZ923062	BZ923062	BZ923062	1	714	714	3	3	3			
114	3	165755848	165756175	CC495469	CC495469	CC495469	1	720	720	3	3	3			
115	3	164426275	164426649	CC553880	CC553880	CC553880	1	723	723	3	3	3			
117	3	163472169	163472351	BZ940504	BZ940504	BZ940504	1	742	742	3	3	3			
118	3	162408614	162408967	BZ897381	BZ897381	BZ897381	1	754	754	3	3	3			
119	3	161327181	161327333	BZ950903	BZ950903	BZ950903	1	756	756	3	3	3			
120	3	161194169	161194358	U14416	U14416	U14416	1	765	765	3	3	3			
121	3	159262988	159263528	BZ919063	BZ919063	BZ919063	1	771	771	3	3	3			
122	3	158404935	158405071	BZ909621	BZ909621	BZ909621	1	777	777	3	3	3			
123	3	157299688	157299742	BZ948894	BZ948894	BZ948894	1	777	777	3	3	3			
124	3	156411798	156412052	BZ950052	BZ950052	BZ950052	1	780	780	3	3	3			
126	3	155286056	155286357	CC447824	CC447824	CC447824	1	782	782	3	3	3			
127	3	154532839	154533258	BZ913666	BZ913666	BZ913666	1	785	785	3	3	3			
128	3	153436378	153436791	BZ909998	BZ909998	BZ909998	1	785	785	3	3	3			
129	3	152467023	152467223	BZ951826	BZ951826	BZ951826	1	790	790	3	3	3			
130	3	151613741	151614060	BZ936863	BZ936863	BZ936863	1	790	790	3	3	3			
131	3	150763257	150763357	BZ840528	BZ840528	BZ840528	1	793	793	3	3	3			
132	3	150036801	150036937	BZ912504	BZ912504	BZ912504	1	793	793	3	3	3			
134	3	148820405	148821059	BZ904948	BZ904948	BZ904948	1	804	804	3	3	3			
135	3	147904582	147905186	BZ921602	BZ921602	BZ921602	1	804	804	3	3	3			
136	3	146839872	146840059	BZ928175	BZ928175	BZ928175	1	807	807	3	3	3			
137	3	145523427	145524054	CC768595	CC768595	CC768595	1	816	816	3	3	3			
138	3	144664097	144664380	BZ947134	BZ947134	BZ947134	1	816	816	3	3	3			
139	3	144257850	144258386	AW289396	AW289396	AW289396	1	818	818	3	3	3			
140	3	143986242	143986581	AF012901	AF012901	AF012901	1	821	821	3	3	3			
141	3	142649826	142650432	AW289248	AW289248	AW289248	1	821	821	3	3	3			
142	3	141760282	141760439	CC523653	CC523653	CC523653	1	821	821	3	3	3			
143	3	140995689	140995836	BZ921837	BZ921837	BZ921837	1	824	824	3	3	3			
144	3	139823619	139824023	BZ923022	BZ923022	BZ923022	1	827	827	3	3	3			
145	3	138849074	138849801	CC497549	CC497549	CC497549	1	833	833	3	3	3			
146	3	138150405	138150480	AW425735	AW425735	AW425735	1	836	836	3	3	3			
147	3	137039804	137039995	BZ897698	BZ897698	BZ897698	1	836	836	3	3	3			
149	3	135970165	135970223	BZ943944	BZ943944	BZ943944	1	844	844	3	3	3			
150	3	134976971	134977164	U02564	U02564	U02564	1	844	844	3	3	3			
151	3	134220550	134220814	BZ938116	BZ938116	BZ938116	1	847	847	3	3	3			
152	3	132970283	132970390	BZ944579	BZ944579	BZ944579	1	849	849	3	3	3			
153	3	132202791	132203153	U83002	U83002	U83002	1	860	860	3	3	3			
154	21	39572529	39572573	BZ937962	BZ937962	BZ937962	1	870	870	4	4	4			
155	21	40589800	40590120	BZ834955	BZ834955	BZ834955	1	870	870	4	4	4			
157	21	41752322	41752575	U88329	U88329	U88329	1	873	873	4	4	4			
158	21	42642997	42643205	CC472975	CC472975	CC472975	1	876	876	4	4	4			
159	21	43172578	43172684	CC472894	CC472894	CC472894	1	884	884	4	4	4			
160	21	44334066	44334250	BZ899575	BZ899575	BZ899575	1	887	887	4	4	4			
161	21	45147648	45147891	Y12672	Y12672	Y12672	1	889	889	4	4	4			
162	21	46233403	46233503	CC764488	CC764488	CC764488	1	889	889	4	4	4			
163	21	46810046	46810269	BZ885885	BZ885885	BZ885885	1	889	889	4	4	4			
164	8	391329	391449	BZ953345	BZ953345	BZ953345	1	892	892	Singleton	5	Singleton	here	AG lost HSB by deleting markers	1 -> 0
165	8	494544	494590	BZ955172	BZ955172	BZ955172	1	892	892	Singleton	5	deleted	AG here		
166	21	35837795	35838079	CC767848	CC767848	CC767848	1	897	897	6	6	5			
167	21	36366616	36367102	AW461769	AW461769	AW461769	1	899	899	6	6	5			
168	21	36785605	36785940	BZ943680	BZ943680	BZ943680	1	902	902	6	6	5			
169	21	37799436	37799865	BZ946785	BZ946785	BZ946785	1	902	902	6	6	5			
170	3	15828804	15828939	BZ911536	BZ911536	BZ911536	1	905	905	7	7	6			
171	3	16855104	16855297	BZ936646	BZ936646	BZ936646	1	910	910	7	7	6			
172	3	17010123	17010194	BZ834991	BZ834991	BZ834991	1	921	921	7	7	6			
173	3	17940690	17940972	BZ939747	BZ939747	BZ939747	1	975	975	7	7	6			
174	3	18134857	18135153	BZ838106	BZ838106	BZ838106	1	990	990	7	7	6			
175	3	19417046	19417404	BZ945051	BZ945051	BZ945051	1	993	993	7	7	6			
176	15	25902035	25902169	CC534164	CC534164	CC534164	2	0	0	8	8	7			
177	15	26181547	26181983	BZ860228	BZ860228	BZ860228	2	15	15	8	8	7		AG is wrong in merging these two blocks, as other blocks comes in between	2 -> 1
178	15	20557314	20557458	AW289383	AW289383	AW289383	2	33	33	9	9	7	AG here		
179	15	20405155	20405308	CC580299	CC580299	CC580299	2	35	35	9	9	7			
180	2	130832665	130832779	BZ924577	BZ924577	BZ924577	2	40	40	10	10	8			
181	2	131633117	131633255	AW415510	AW415510	AW415510	2	48	48	10	10	8			
182	2	129341065	129341301	BZ928273	BZ928273	BZ928273	2	56	56	10	10	8			
183	2	128354657	128354835	BZ939646	BZ939646	BZ939646	2	61	61	10	10	8			
184	2	191180317	191180490	BZ929978	BZ929978	BZ929978	2	64	64	11	11	9			
185	2	191050269	191050512	AW353074	AW353074	AW353074	2	73	73	11	11	9			
186	2	188157670	188158336	BZ921980	BZ921980	BZ921980	2	81	81	11	11	9			
187	2	189962996	189963252	BZ932803	BZ932803	BZ932803	2	84	84	11	11	9			
188	2	188896485	188896994	BZ946646	BZ946646	BZ946646	2	84	84	11	11	9			
189	2	187524055	187524388	BZ855272	BZ855272	BZ855272	2	92	92	11	11	9			
190	2	186495742	186496056	BZ935738	BZ935738	BZ935738	2	98	98	11	11	9			
192	2	185532263	185532386	BZ897211	BZ897211	BZ897211	2	118	118	11	11	9			
193	2	184720808	184721557	BZ837900	BZ837900	BZ837900	2	124	124	11	11	9			
194	2	183556311	183557036	U24164	U24164	U24164	2	156	156	11	11	9			
195	2	182914317	182914510	BZ921457	BZ921457	BZ921457	2	160	160	11	11	9			
196	2	181673470	181674015	BZ909979	BZ909979	BZ909979	2	182	182	11	11	9			
197	2	180565067	180565265	BZ947568	BZ947568	BZ947568	2	210	210	11	11	9			
198	2	179283165	179283570	BZ949304	BZ949304	BZ949304	2	233	233	11	11	9			
199	2	178242028	178242405	BZ933942	BZ933942	BZ933942	2	236	236	11	11	9			
201	2	176840359	176840668	BZ937928	BZ937928	BZ937928	2	256	256	11	11	9			
202	2	175793477	175794061	BZ916661	BZ916661	BZ916661	2	264	264	11	11	9			
203	2	174795872	174796094	BZ948919	BZ948919	BZ948919	2	271	271	11	11	9			
204	2	173252449	173252562	BZ906944	BZ906944	BZ906944	2	275	275	11	11	9			
205	2	172164447	172164683	BZ899861	BZ899861	BZ899861	2	279	279	11	11	9			
206	2	171109055	171109250	BZ939721	BZ939721	BZ939721	2	279	279	11	11	9			
207	2	169559350	169559553	CC592299	CC592299	CC592299	2	287	287	11	11	9			
208	2	170851481	170851774	CC474483	CC474483	CC474483	2	295	295	11	11	9			
209	2	168756950	168757359	BZ936840	BZ936840	BZ936840	2	312	312	11	11	9			
210	2	167556116	167556497	BZ926088	BZ926088	BZ926088	2	325	325	11	11	9		AG missed an inversion here	3 -> 1
212	2	163041017	163041504	BZ927381	BZ927381	BZ927381	2	330	330	12	12	9	AG here		
213	2	162540704	162540988	CC571689	CC571689	CC571689	2	335	335	12	12	9			
214	2	164148525	164148782	BZ940932	BZ940932	BZ940932	2	345	345	12	12	9			
216	2	165160792	165161214	BZ918736	BZ918736	BZ918736	2	355	355	12	12	9			
217	2	166225011	166225118	BZ911373	BZ911373	BZ911373	2	361	361	12	12	9			
218	2	161429955	161430412	BZ930452	BZ930452	BZ930452	2	376	376	13	13	9			
219	2	160430819	160430983	BZ924652	BZ924652	BZ924652	2	380	380	13	13	9			
220	2	159456695	159456884	BZ931753	BZ931753	BZ931753	2	385	385	13	13	9			
221	2	158447940	158448208	AW326496	AW326496	AW326496	2	394	394	13	13	9			
222	2	158262151	158262617	BZ943404	BZ943404	BZ943404	2	399	399	13	13	9			
223	2	157170440	157171007	BZ944538	BZ944538	BZ944538	2	416	416	13	13	9			
224	2	156167483	156168093	BZ915537	BZ915537	BZ915537	2	425	425	13	13	9			
225	2	154905913	154906149	BZ917935	BZ917935	BZ917935	2	442	442	13	13	9			
226	2	153789556	153789951	BZ940627	BZ940627	BZ940627	2	455	455	13	13	9			
227	2	153442689	153443181	AW289336	AW289336	AW289336	2	455	455	13	13	9			
228	2	151952492	151952892	AA908007	AA908007	AA908007	2	463	463	13	13	9			
229	2	150978669	150978969	BZ832650	BZ832650	BZ832650	2	471	471	13	13	9			
230	2	149989840	149990126	BZ944425	BZ944425	BZ944425	2	476	476	13	13	9			
231	2	149468164	149468499	BZ906909	BZ906909	BZ906909	2	476	476	13	13	9			
232	2	147131789	147132069	BZ864117	BZ864117	BZ864117	2	492	492	13	13	9			
233	2	148142171	148142255	BZ912628	BZ912628	BZ912628	2	507	507	13	13	9			
234	2	145987735	145987940	BZ905033	BZ905033	BZ905033	2	512	512	13	13	9			
235	2	144849170	144849335	BZ917723	BZ917723	BZ917723	2	521	521	13	13	9			
236	2	143746776	143746956	AW656747	AW656747	AW656747	2	521	521	13	13	9			
237	2	142598784	142599068	BZ953683	BZ953683	BZ953683	2	536	536	13	13	9			
238	2	141606558	141606912	BZ934314	BZ934314	BZ934314	2	536	536	13	13	9			
239	2	140523739	140523878	BZ902767	BZ902767	BZ902767	2	536	536	13	13	9			
240	2	139356592	139357187	BZ899212	BZ899212	BZ899212	2	547	547	13	13	9			
241	2	137977913	137978150	BZ914177	BZ914177	BZ914177	2	553	553	13	13	9			
242	2	136586280	136586346	BZ941970	BZ941970	BZ941970	2	584	584	13	13	9			
243	2	135047345	135047711	AW307678	AW307678	AW307678	2	605	605	13	13	9			
244	2	134172655	134173194	BZ911648	BZ911648	BZ911648	2	605	605	13	13	9			
245	2	133113440	133113503	BZ942274	BZ942274	BZ942274	2	631	631	13	13	9			
247	2	114431810	114432396	AW289253	AW289253	AW289253	2	673	673	14	14	10			
248	2	115131083	115131522	BZ919492	BZ919492	BZ919492	2	673	673	14	14	10			
249	2	116163622	116163760	BZ899208	BZ899208	BZ899208	2	678	678	14	14	10			
250	2	117038994	117039115	BZ884291	BZ884291	BZ884291	2	684	684	14	14	10			
251	2	117865790	117865941	BZ945011	BZ945011	BZ945011	2	690	690	14	14	10			
252	2	118798998	118799520	CC505535	CC505535	CC505535	2	696	696	14	14	10			
253	2	119204885	119205004	BZ950876	BZ950876	BZ950876	2	696	696	14	14	10			

254	2	119791265	119791334	CC491613	CC491613	CC491613	2	702	702	14	14	10		
255	2	121019478	121019794	BZ907417	BZ907417	BZ907417	2	702	702	14	14	10		
256	2	120824931	120825306	AW669304	AW669304	AW669304	2	702	702	14	14	10		
257	2	121965463	121965571	BZ925702	BZ925702	BZ925702	2	702	702	14	14	10		
258	2	122956937	122957281	CC573374	CC573374	CC573374	2	715	715	14	14	10		
259	2	122978146	122978540	BZ948549	BZ948549	BZ948549	2	715	715	14	14	10		
260	2	124500405	124500672	BZ885291	BZ885291	BZ885291	2	728	728	14	14	10		
261	2	124539607	124540124	BZ941667	BZ941667	BZ941667	2	734	734	14	14	10		
262	2	126027347	126027814	BZ945414	BZ945414	BZ945414	2	758	758	14	14	10		
263	2	127152788	127153033	CC769033	CC769033	CC769033	2	769	769	14	14	10		
264	2	192242348	192242588	BZ925299	BZ925299	BZ925299	2	805	805	15	15	11		
265	2	193309366	193309620	BZ933243	BZ933243	BZ933243	2	810	810	15	15	11		
266	2	194653148	194653429	CC477506	CC477506	CC477506	2	830	830	15	15	11		
267	2	195258828	195259020	BZ935576	BZ935576	BZ935576	2	830	830	15	15	11		
268	2	196425850	196425994	BZ925572	BZ925572	BZ925572	2	830	830	15	15	11		
269	2	197676901	197677502	AW289239	AW289239	AW289239	2	835	835	15	15	11		
270	2	197732479	197732752	BZ956433	BZ956433	BZ956433	2	840	840	15	15	11		
271	2	198779350	198779695	BZ944867	BZ944867	BZ944867	2	845	845	15	15	11		
272	2	199777187	199777753	BZ922169	BZ922169	BZ922169	2	845	845	15	15	11		
273	2	200818604	200818902	BZ919896	BZ919896	BZ919896	2	855	855	15	15	11		
274	2	201347802	201348094	AW289179	AW289179	AW289179	2	855	855	15	15	11		
275	2	202138789	202138833	BZ948363	BZ948363	BZ948363	2	860	860	15	15	11		
276	2	204416859	204417247	X93304	X93304	X93304	2	866	866	15	15	11		
277	2	203259337	203259704	BZ949214	BZ949214	BZ949214	2	871	871	15	15	11		
279	2	205461001	205461297	BZ947329	BZ947329	BZ947329	2	893	893	15	15	11		
280	2	206498625	206498735	CC474021	CC474021	CC474021	2	893	893	15	15	11		
281	2	207522425	207522588	BZ924812	BZ924812	BZ924812	2	893	893	15	15	11		
282	2	208586072	208586322	BZ909579	BZ909579	BZ909579	2	893	893	15	15	11		
283	2	209149649	209150304	BZ946290	BZ946290	BZ946290	2	897	897	15	15	11		
284	2	210680881	210681306	BZ907123	BZ907123	BZ907123	2	897	897	15	15	11		
285	2	211506537	211506599	BZ946502	BZ946502	BZ946502	2	902	902	15	15	11		
286	2	212272847	212272954	CC771713	CC771713	CC771713	2	916	916	15	15	11		
287	2	213207825	213208361	BZ914526	BZ914526	BZ914526	2	920	920	15	15	11		
288	2	214933193	214933601	BZ925641	BZ925641	BZ925641	2	941	941	15	15	11		
289	2	214030315	214030561	BZ922076	BZ922076	BZ922076	2	944	944	15	15	11		
290	2	216072383	216072830	CC447378	CC447378	CC447378	2	948	948	15	15	11		
291	2	217258231	217258434	BZ922604	BZ922604	BZ922604	2	948	948	15	15	11		
292	2	218321201	218321612	BZ907294	BZ907294	BZ907294	2	948	948	15	15	11		
293	2	218854446	218855404	U19947	U19947	U19947	2	951	951	15	15	11		
294	2	218930633	218930835	AW289209	AW289209	AW289209	2	955	955	15	15	11		
295	2	219872690	219873034	AW447613	AW447613	AW447613	2	963	963	15	15	11		
296	2	220680102	220680659	BZ910262	BZ910262	BZ910262	2	963	963	15	15	11		
297	2	221669310	221669412	BZ947191	BZ947191	BZ947191	2	970	970	15	15	11		
298	2	222638344	222638976	BZ908871	BZ908871	BZ908871	2	970	970	15	15	11		
299	2	223746839	223747066	BZ948393	BZ948393	BZ948393	2	974	974	15	15	11		
300	2	224287600	224288077	AW447344	AW447344	AW447344	2	981	981	15	15	11		
301	2	225106233	225106334	BZ919600	BZ919600	BZ919600	2	981	981	15	15	11		
302	2	226272898	226273277	BZ922439	BZ922439	BZ922439	2	988	988	15	15	11		
303	2	227228861	227228905	BZ914460	BZ914460	BZ914460	2	988	988	15	15	11		
304	2	228176571	228176788	BZ954904	BZ954904	BZ954904	2	999	999	15	15	11		
305	2	229631000	229631569	BZ902874	BZ902874	BZ902874	2	1007	1007	15	15	11		
306	2	230603709	230603836	BZ934427	BZ934427	BZ934427	2	1007	1007	15	15	11		
307	2	231751458	231751560	BZ917471	BZ917471	BZ917471	2	1013	1013	15	15	11		
308	2	232758808	232758912	CC530565	CC530565	CC530565	2	1034	1034	15	15	11		
309	1	32624297	32624772	BZ942912	BZ942912	BZ942912	2	1059	1059	16	16	12		
310	1	32042949	32043595	AW289350	AW289350	AW289350	2	1074	1074	16	16	12		
311	1	31147495	31147727	BZ914861	BZ914861	BZ914861	2	1077	1077	16	16	12		
312	1	30874410	30874972	AW428607	AW428607	AW428607	2	1077	1077	16	16	12		
313	1	29704026	29704460	CC536866	CC536866	CC536866	2	1077	1077	16	16	12		
314	1	29260271	29260475	BZ919725	BZ919725	BZ919725	2	1080	1080	16	16	12		
315	1	28246998	28247210	BZ948644	BZ948644	BZ948644	2	1080	1080	16	16	12		
316	1	27369412	27369609	BZ944283	BZ944283	BZ944283	2	1087	1087	16	16	12		
317	1	26953516	26953698	AW437332	AW437332	AW437332	2	1090	1090	16	16	12		
318	1	26332668	26333027	AW289302	AW289302	AW289302	2	1090	1090	16	16	12		
319	1	25700230	25700354	BZ944434	BZ944434	BZ944434	2	1097	1097	16	16	12		
320	1	24413849	24414157	BZ946016	BZ946016	BZ946016	2	1120	1120	16	16	12		
322	1	23291023	23291485	BZ914597	BZ914597	BZ914597	2	1143	1143	16	16	12		
323	1	22719467	22719752	AW289232	AW289232	AW289232	2	1148	1148	16	16	12		
324	1	22163224	22163415	AW261138	AW261138	AW261138	2	1163	1163	16	16	12		
325	1	21122609	21122899	CC531371	CC531371	CC531371	2	1172	1172	16	16	12		
327	1	20050168	20050248	BZ921605	BZ921605	BZ921605	2	1178	1178	16	16	12		
328	1	19232875	19233295	BZ924433	BZ924433	BZ924433	2	1187	1187	16	16	12		
329	1	18209422	18209574	BZ954082	BZ954082	BZ954082	2	1190	1190	16	16	12		
330	1	17046307	17046417	S68064	S68064	S68064	2	1190	1190	16	16	12		
331	1	164636627	164636876	AW482123	AW482123	AW482123	3	0	0	17	17	13		
332	1	164217678	164217858	BZ923861	BZ923861	BZ923861	3	7	7	17	17	13		
333	1	163189089	163189313	BZ930502	BZ930502	BZ930502	3	7	7	17	17	13		
334	1	162252511	162252930	BZ931498	BZ931498	BZ931498	3	13	13	17	17	13		
335	1	161081899	161082175	BZ924794	BZ924794	BZ924794	3	23	23	17	17	13		
336	1	160028086	160028308	BZ949113	BZ949113	BZ949113	3	34	34	17	17	13		
337	1	159301042	159301285	BE217503	BE217503	BE217503	3	42	42	17	17	13		
338	1	158625193	158625436	BZ917759	BZ917759	BZ917759	3	45	45	17	17	13		
339	1	158372813	158373104	X75671	X75671	X75671	3	45	45	17	17	13		
340	1	156494160	156494249	BE217553	BE217553	BE217553	3	58	58	17	17	13		
342	1	156826820	156826888	BZ903632	BZ903632	BZ903632	3	62	62	17	17	13		
343	1	155601758	155601867	BZ949355	BZ949355	BZ949355	3	66	66	17	17	13		
344	1	154882695	154882763	AW289420	AW289420	AW289420	3	70	70	17	17	13		
345	1	154472857	154473100	BZ952269	BZ952269	BZ952269	3	70	70	17	17	13		
347	1	153435108	153435783	X75887	X75887	X75887	3	96	96	17	17	13		
348	1	153200089	153200349	BZ945746	BZ945746	BZ945746	3	103	103	17	17	13		
349	1	152129896	152129961	BZ948493	BZ948493	BZ948493	3	114	114	17	17	13		
350	1	151972772	151972950	L41543	L41543	L41543	3	118	118	17	17	13		
351	1	151764585	151764798	AW289261	AW289261	AW289261	3	118	118	17	17	13		
352	1	150979148	150979342	CC447538	CC447538	CC447538	3	121	121	17	17	13		
353	1	150329246	150329473	D89056	D89056	D89056	3	121	121	17	17	13		
354	1	150160007	150160144	D49548	D49548	D49548	3	128	128	17	17	13		
355	1	150204725	150204907	D49550	D49550	D49550	3	131	131	17	17	13		
356	1	149117113	149117215	CC550079	CC550079	CC550079	3	140	140	17	17	13		
357	1	148359803	148359948	M64924	M64924	M64924	3	143	143	17	17	13		
358	1	147750854	147751027	AW267074	AW267074	AW267074	3	153	153	17	17	13		
359	1	147360105	147360317	AW289401	AW289401	AW289401	3	157	157	17	17	13		
360	1	146162143	146162408	BZ923674	BZ923674	BZ923674	3	157	157	17	17	13		
362	1	144567531	144567738	BZ843734	BZ843734	BZ843734	3	161	161	17	17	13		
364	1	144173394	144173561	CC490961	CC490961	CC490961	3	168	168	17	17	13		
365	1	142929045	142929309	BZ912979	BZ912979	BZ912979	3	168	168	17	17	13		
366	1	119905867	119905975	CC529149	CC529149	CC529149	3	180	180	18	17	13	ST here	discrepant case in HSB definitions between SyntenyTracker and Evers-van der Wind et al
367	1	120153466	120153587	BZ932149	BZ932149	BZ932149	3	183	183	18	17	13		
368	1	119182689	119183013	BZ913674	BZ913674	BZ913674	3	187	187	18	17	13		
369	1	117766888	117766992	BZ931347	BZ931347	BZ931347	3	199	199	18	17	13		
370	1	116905967	116906220	BZ955729	BZ955729	BZ955729	3	210	210	18	17	13		
371	1	116178859	116179169	BZ923697	BZ923697	BZ923697	3	225	225	18	17	13		
372	1	115540747	115541428	Y09566	Y09566	Y09566	3	235	235	18	17	13		
373	1	115288635	115288961	K01939	K01939	K01939	3	242	242	18	17	13		
374	1	114959132	114959480	AW267011	AW267011	AW267011	3	246	246	18	17	13		
375	1	114648042	114648673	BZ941876	BZ941876	BZ941876	3	246	246	18	17	13		
376	1	113938223	113938733	CC775139	CC775139	CC775139	3	250	250	18	17	13		
377	1	112925764	112926197	AW289236	AW289236	AW289236	3	250	250	18	17	13		
378	1	112116304	112116710	BZ938965	BZ938965	BZ938965	3	250	250	18	17	13		
379	1	110889548	110889773	CC516843	CC516843	CC516843	3	257	257	18	17	13		
380	1	110337797	110338184	BZ921952	BZ921952	BZ921952	3	261	261	18	17	13		
381	1	110177828	110178872	X05010	X05010	X05010	3	261	261	18	17	13		
383	1	108953824	108954090	AW358002	AW358002	AW358002	3	277	277	18	17	13		
384	1	107826176	107826417	AW417792	AW417792	AW417792	3	277	277	18	17	13		
385	1	107649854	107650219	BZ905380	BZ905380	BZ905380	3	277	277	18	17	13		
386	1	107312372	107312629	AW327015	AW327015	AW327015	3	281	281	18	17	13		
387	1	106276768	106277008	BZ942440	BZ942440	BZ942440	3	288	288	18	17	13		
389	1	105439187	105439295	BZ848262	BZ848262	BZ848262	3	321	321	18	17	13		
390	1	104570531	104571023	BZ933635	BZ933635	BZ933635	3	321	321	18	17	13		
391	1	104051578	104051779	BZ850739	BZ850739	BZ850739	3	325	325	18	17	13		
392	1	103827706	103827865	Z48929	Z48929	Z48929	3	325	325	18	17	13		
393	1	103169479	103169630	BZ839540	BZ839540	BZ839540	3	330	330	18	17	13		
394	1	102869312	102869440	BZ932758	BZ932758	BZ932758	3	342	342	18	17	13		
395	1	101416351	101418785	M31210	M31210	M31210	3	354	354	18	17	13		
396	1	101259647	101260026	BZ918735	BZ918735	BZ918735	3	354	354	18	17	13		
398	1	100916394	100916512	BF440476	BF440476	BF440476	3	358	358	18	17	13		
399	1	100036517	100036768	BZ923683	BZ923683	BZ923683	3	362	362	18	17	13		
400	1	98879245	98879352	BZ937405	BZ937405	BZ937405	3	366	366	18	17	13		
401	1	97876925	97877126	U20981	U20981	U20981	3	375	375	18	17	13		
402	1	97713792	97714400	BZ914051	BZ914051	BZ914051	3	375	375	18	17	13		
403	1	96884825	96885014	BZ951122	BZ951122	BZ951122	3	375	375	18	17	13		
404	1	95789711	95790095	BZ918040	BZ918040	BZ918040	3	384	384	18	17	13		
405	1	94710667	94710847	S74147	S74147	S74147	3	384	384	18	17	13		
406	1	93893330	93893533	CC568771	CC568771	CC568771	3	388	388	18	17	13		
408	1	92052508	92052839	BZ928737	BZ928737	BZ928737	3	392	392	18	17	13		
409	1	91702896	91702986	AW289255	AW289255	AW289255	3	397	397	18	17	13		
410	1	91025336	91025981	BZ934118	BZ934118	BZ934118	3	401	401	18	17	13		
411	1	90121381	90122035	CC502559	CC502559	CC502559	3	409	409	18	17	13		
412	1	90064115	90064346	BZ930459	BZ930459	BZ930459	3	409	409	18	17	13		
413	1	89230654	89230801	AW289360	AW289360	AW289360	3	417	417	18	17	13		
414	1	89030546	89031078	U83023	U83023	U83023	3	426	426	18	17	13		
415	1	88472562	88473000	BZ947066	BZ947066	BZ947066	3	426	426	18	17	13		
416	1	87299022	87299304	CC769477	CC769477	CC769477	3	430	430	18	17	13		
417	1	85937357	85937518	BZ910884	BZ910884	BZ910884	3	430	430	18	17	13		
418	1	85007690	85008100	BZ945533	BZ945533	BZ945533	3	444	444	18	17	13		
419	1	83927654	83928207	BZ956256	BZ956256	BZ956256	3	453	453	18	17	13		
420	1	83171133	83171192	BZ955127	BZ955127	BZ955127	3	466	466	18	17	13		
421	1	82151668	82152011	BZ921214	BZ921214	BZ921214	3	475	475	18	17	13		
422	1	81015128	81015659	BZ915772	BZ915772	BZ915772	3	475	475	18	17	13		
423	1	79556910	79557501	BZ920037	BZ920037	BZ920037	3	484	484	18	17	13		
424	1	79751914	79752130	CC587853	CC587853	CC587853	3	495	495	18	17	13		
425	1	78592731	78592922	BZ945213	BZ945213	BZ945213	3	500	500	18	17	13		
426	1	77289560	77289918	BZ947320	BZ947320	BZ947320	3	500	500	18	17	13		
427	1	76378624	76378761	BZ896736	BZ896736	BZ896736	3	510	510	18	17	13		
428	1	75399050	75399514	BZ909807	BZ909807	BZ909807	3	515	515	18	17	13		
429	1	74387646	74388193	BZ917315	BZ917315	BZ917315	3	525	525	18	17	13		
430	1	73243075	73243623	BZ919395	BZ919395	BZ919395	3	534	534	18	17	13		
431	1	71960392	71960949	BZ940334	BZ940334	BZ940334	3	538	538	18	17	13		
432	1	70964803	70965385	BZ913984	BZ913984	BZ913984	3	538	538	18	17	13		
433	1	69660333	69660511	BZ930144	BZ930144	BZ930144	3	543	543	18	17	13		
434	1	68606496	68607635	X66277	X66277	X66277	3	547	547	18	17	13		
435	1	68582407	68582584	BZ918042	BZ918042	BZ918042	3	547	547	18	17	13		
437	1	67610645	67610747	CC496513	CC496513	CC496513	3	565	565	18	17	13		
438	1	66504640	66504857	BZ938895	BZ938895	BZ938895	3	569	569	18	17	13		
439	1	65396573	65396632	CC558277	CC558277	CC558277	3	573	573	18	17	13		
440	1	64270628	64270918	BZ901906	BZ901906	BZ901906	3	573	573	18	17	13		
441	1	63837334	63837937	AW289332	AW289332	AW289332	3	582	582	18	17	13		
443	1	62774747	62774979	CC591083	CC591083	CC591083	3	587	587	18	17	13		
444	1	61425233	61425500	BZ929647	BZ929647	BZ929647	3	596	596	18	17	13		
445	1	60322936	60323321	BZ913376	BZ913376	BZ913376	3	605	605	18	17	13		
447	1	58960434	58960749	AF069514	AF069514	AF069514	3	605	605	18	17	13		
448	1	57815799	57816019	BZ904321	BZ904321	BZ904321	3	614	614	18	17	13		
449	1	56816656	56817077	BZ922909	BZ922909	BZ922909	3	614	614	18	17	13		
450	1	55725054	55725283	BZ901502	BZ901502	BZ901502	3	622	622	18	17	13		
451	1	54663258	54663501	BZ908424	BZ908424	BZ908424	3	638	638	18	17	13		
452	1	52256259	52256320	BZ921967	BZ921967	BZ921967	3	649	649	18	17	13		
453	1	53453269	53453452	BZ912030	BZ912030	BZ912030	3	652	652	18	17	13		
454	1	51222632	51222917	BZ927348	BZ927348	BZ927348	3	659	659	18	17	13		
455	1	50460914	50461213	BZ909445	BZ909445	BZ909445	3	671	671	18	17	13		
456	1	49424954	49425455	CC512043	CC512043	CC512043	3	679	679	18	17	13		
457	1	48983656	48983989	BZ949170	BZ949170	BZ949170	3	683	683	18	17	13		
458	1	48475566	48475786	BZ939653	BZ939653	BZ939653	3	700	700	18	17	13		
459	1	47470190	47470273	BZ954086	BZ954086	BZ954086	3	711	711	18	17	13		
460	1	46473659	46473819	AW461454	AW461454	AW461454	3	727	727	18	17	13		
461	1	44966728	44967208	BZ907051	BZ907051	BZ907051	3	735	735	18	17	13		
462	1	44912764	44912894	BE217560	BE217560	BE217560	3	743	743	18	17	13		
463	1	43921711	43921866	BZ950997	BZ950997	BZ950997	3	756	756	18	17	13		
464	1	43447068	43447354	X71423	X71423	X71423	3	756	756	18	17	13		
465	1	42671223	42671421	BZ945376	BZ945376	BZ945376	3	764	764	18	17	13		
466	1	41475865	41475952	BZ897757	BZ897757	BZ897757	3	777	777	18	17	13		
467	1	40171519	40171825	CC567147	CC567147	CC567147	3	780	780	18	17	13		
468	1	39620406	39620955	AW289374	AW289374	AW289374	3	792	792	18	17	13		
469	1	39140090	39140415	AW289183	AW289183	AW289183	3	795	795	18	17	13		
470	1	38165333	38165599	CC447361	CC447361	CC447361	3	818	818	18	17	13		
471	1	37263132	37263644	CC474577	CC474577	CC474577	3	822	822	18	17	13		
472	1	36271458	36271756	AW659637	AW659637	AW659637	3	825	825	18	17	13		
473	1	36058044	36058316	BZ908779	BZ908779	BZ908779	3	825	825	18	17	13		
474	1	34981728	34981905	BZ951517	BZ951517	BZ951517	3	828	828	18	17	13		
475	1	33935925	33936080	BZ914104	BZ914104	BZ914104	3	832	832	18	17	13		
476	2	233540241	233540729	BZ904797	BZ904797	BZ904797	3	843	843	19	18	14		
477	2	234025606	234025745	J02955	J02955	J02955	3	847	847	19	18	14		
478	2	235186180	235186521	AW311712	AW311712	AW311712	3	851	851	19	18	14		
479	2	236542458	236542650	BZ926992	BZ926992	BZ926992	3	855	855	19	18	14		
480	2	237524309	237524440	BZ934938	BZ934938	BZ934938	3	859	859	19	18	14		
481	2	238839683	238839812	CC570347	CC570347	CC570347	3	859	859	19	18	14		
482	2	239452499	239452768	BZ916735	BZ916735	BZ916735	3	863	863	19	18	14		
483	2	240620522	240620599	BZ901132	BZ901132	BZ901132	3	867	867	19	18	14		
484	2	241842051	241842246	AW336101	AW336101	AW336101	3	878	878	19	18	14		
485	7	55522977	55523155	U83072	U83072	U83072	4	0	0	20	19	15		
486	7	54490671	54490931	BZ857334	BZ857334	BZ857334	4	16	16	20	19	15		
487	7	53600085	53600357	CC530790	CC530790	CC530790	4	16	16	20	19	15		
488	7	52885478	52885659	BZ951931	BZ951931	BZ951931	4	23	23	20	19	15		
489	7	51870765	51870853	CC764626	CC764626	CC764626	4	36	36	20	19	15		
490	7	50869601	50869689	CC520986	CC520986	CC520986	4	36	36	20	19	15		
491	7	49874883	49875041	BZ846980	BZ846980	BZ846980	4	39	39	20	19	15		
492	7	48685810	48686138	CC551178	CC551178	CC551178	4	42	42	20	19	15		
493	7	47850747	47850887	CC488422	CC488422	CC488422	4	42	42	20	19	deleted		
494	7	90404473	90404754	CC585186	CC585186	CC585186	4	42	42	21	20	deleted		
495	7	91674516	91674977	AW462850	AW462850	AW462850	4	45	45	21	20	16		
496	7	92374703	92374928	BZ902073	BZ902073	BZ902073	4	48	48	21	20	16		
497	7	92482223	92482609	BZ887363	BZ887363	BZ887363	4	54	54	21	20	16		
498	7	93314466	93314618	BZ935690	BZ935690	BZ935690	4	65	65	21	20	16		
499	7	94261641	94262251	CC490310	CC490310	CC490310	4	68	68	21	20	16		
500	7	95301087	95301678	CC546458	CC546458	CC546458	4	72	72	21	20	16		
501	7	96265246	96265706	BZ913469	BZ913469	BZ913469	4	76	76	21	20	16		
502	7	97013837	97014071	X00076	X00076	X00076	4	87	87	21	20	16		
503	7	7330272	7330912	BZ878623	BZ878623	BZ878623	4	103	103	22	21	17		
504	7	8416079	8416532	BZ920164	BZ920164	BZ920164	4	119	119	22	21	17		
505	7	9117257	9117698	CC768450	CC768450	CC768450	4	119	119	22	21	17		
506	7	9424351	9424563	CC480424	CC480424	CC480424	4	123	123	22	21	17		

507	7	10218395	10219179	CC514109	CC514109	CC514109	4	131	131	22	21	17			
508	7	11241762	11242077	BZ939900	BZ939900	BZ939900	4	135	135	22	21	17			
509	7	12439449	12439670	D26549	D26549	D26549	4	154	154	22	21	17			
510	7	12448645	12449101	BZ931126	BZ931126	BZ931126	4	154	154	22	21	17			
511	7	13534033	13534276	BZ857090	BZ857090	BZ857090	4	161	161	22	21	17			
512	7	14434076	14434394	CC469592	CC469592	CC469592	4	167	167	22	21	17			
513	7	15556303	15557077	CC499795	CC499795	CC499795	4	174	174	22	21	17			
514	7	16494039	16494288	CC764889	CC764889	CC764889	4	174	174	22	21	17			
515	7	17509584	17509849	CC531188	CC531188	CC531188	4	184	184	22	21	17			
516	7	18507648	18507913	CC502226	CC502226	CC502226	4	190	190	22	21	17			
517	7	19434658	19435114	CC466397	CC466397	CC466397	4	190	190	22	21	17			
518	7	20513516	20513842	BZ910615	BZ910615	BZ910615	4	193	193	22	21	17			
519	7	21578120	21578464	BZ837067	BZ837067	BZ837067	4	196	196	22	21	17			
520	7	22994904	22995053	BZ944844	BZ944844	BZ944844	4	203	203	22	21	17			
521	7	23338773	23339316	AW289294	AW289294	AW289294	4	209	209	22	21	17			
522	7	87110153	87110277	CC514299	CC514299	CC514299	4	209	209	23	22	deleted			
523	7	86840001	86840211	BE217416	BE217416	BE217416	4	209	209	23	22	deleted			
524	7	86635356	86635542	U65745	U65745	U65745	4	209	209	23	22	deleted			
525	7	85933634	85934166	CC498427	CC498427	CC498427	4	216	216	23	22	18			
526	7	83943403	83943676	CC595041	CC595041	CC595041	4	219	219	23	22	18			
527	7	82867884	82868567	CC546970	CC546970	CC546970	4	219	219	23	22	18			
528	7	81891197	81891425	CC767630	CC767630	CC767630	4	219	219	23	22	18			
529	7	80834546	80834987	CC573716	CC573716	CC573716	4	225	225	23	22	18			
530	7	79920604	79920846	X91503	X91503	X91503	4	229	229	23	22	18			
531	7	79768175	79768397	CC541504	CC541504	CC541504	4	232	232	23	22	18			
532	7	79491253	79493343	X03642	X03642	X03642	4	236	236	23	22	18			
533	7	78735235	78735550	CC508041	CC508041	CC508041	4	236	236	23	22	18			
534	7	77837317	77838008	CC471384	CC471384	CC471384	4	236	236	23	22	18			
535	7	76470414	76470558	BZ845464	BZ845464	BZ845464	4	239	239	23	22	18			
536	7	76772409	76772569	BZ868341	BZ868341	BZ868341	4	243	243	23	22	18			
537	7	102645521	102646081	CC508340	CC508340	CC508340	4	254	254	24	23	19			
538	7	103360577	103361207	AW289217	AW289217	AW289217	4	257	257	24	23	19			
539	7	103680256	103680720	CC589195	CC589195	CC589195	4	261	261	24	23	19			
540	7	104248631	104248868	AW483805	AW483805	AW483805	4	275	275	24	23	19			
541	7	104693274	104693814	CC519712	CC519712	CC519712	4	278	278	24	23	19			
542	7	105772693	105772751	CC584898	CC584898	CC584898	4	285	285	24	23	19			
543	7	106395231	106395586	AW357054	AW357054	AW357054	4	285	285	24	23	deleted			
544	7	116756536	116756968	CC517995	CC517995	CC517995	4	292	292	25	24	20			
545	7	116017303	116017495	AW464296	AW464296	AW464296	4	295	295	25	24	20			
546	7	115742701	115742901	BZ914154	BZ914154	BZ914154	4	295	295	25	24	20			
547	7	114742334	114742385	BZ886868	BZ886868	BZ886868	4	295	295	25	24	20			
548	7	113708232	113708692	CC532890	CC532890	CC532890	4	299	299	25	24	20			
549	7	112053158	112053805	AW289361	AW289361	AW289361	4	302	302	25	24	20			
550	7	112686159	112686568	CC534797	CC534797	CC534797	4	306	306	25	24	20			
552	7	111764559	111764922	BZ897910	BZ897910	BZ897910	4	330	330	25	24	20			
553	7	110841835	110841910	CC516047	CC516047	CC516047	4	343	343	25	24	20			
554	7	109846149	109846486	CC498153	CC498153	CC498153	4	347	347	25	24	20			
555	7	108838742	108839124	CC500413	CC500413	CC500413	4	347	347	25	24	20			
556	7	107893487	107893750	CC774120	CC774120	CC774120	4	359	359	25	24	20			
558	7	37128596	37129042	BZ947997	BZ947997	BZ947997	4	377	377	26	25	21			
559	7	36218995	36219258	BZ863307	BZ863307	BZ863307	4	380	380	26	25	21			
560	7	35254974	35255289	BZ908035	BZ908035	BZ908035	4	384	384	26	25	21			
561	7	34591283	34591668	CC510473	CC510473	CC510473	4	384	384	26	25	21			
562	7	33774887	33775059	BZ928674	BZ928674	BZ928674	4	387	387	26	25	21			
563	7	32834825	32834963	CC533615	CC533615	CC533615	4	387	387	26	25	21			
564	7	31842304	31842350	BZ835315	BZ835315	BZ835315	4	387	387	26	25	21			
565	7	30919253	30919423	CC560273	CC560273	CC560273	4	394	394	26	25	21			
566	7	29993693	29994195	CC569534	CC569534	CC569534	4	414	414	26	25	21			
567	7	29056839	29057317	CC517191	CC517191	CC517191	4	418	418	26	25	21			
568	7	28111367	28111627	BZ852153	BZ852153	BZ852153	4	422	422	26	25	21			
569	7	27076759	27076980	BZ950169	BZ950169	BZ950169	4	427	427	26	25	21			
570	7	26186325	26186494	AW346349	AW346349	AW346349	4	431	431	26	25	21			
571	7	25056442	25056572	CC503269	CC503269	CC503269	4	436	436	26	25	21			
572	7	23866994	23867086	BZ941133	BZ941133	BZ941133	4	450	450	26	25	21			
574	7	88122820	88122918	CC552942	CC552942	CC552942	4	454	454	27	26	22			
575	7	89438291	89438697	BZ949775	BZ949775	BZ949775	4	461	461	27	26	22			
576	7	46852676	46853378	BZ871449	BZ871449	BZ871449	4	465	465	28	27	23			
577	7	45733576	45734088	M76478	M76478	M76478	4	465	465	28	27	23			
578	7	45725999	45726174	CC767575	CC767575	CC767575	4	465	465	28	27	23			
579	7	45387369	45388056	M25579	M25579	M25579	4	469	469	28	27	23			
580	7	44914085	44914239	CC523866	CC523866	CC523866	4	469	469	28	27	23			
581	7	43886344	43886522	CC774334	CC774334	CC774334	4	473	473	28	27	23			
582	7	42505678	42506230	BZ918094	BZ918094	BZ918094	4	485	485	28	27	23			
583	7	41938189	41938603	BZ873654	BZ873654	BZ873654	4	497	497	28	27	23			
584	7	40850661	40851081	CC593826	CC593826	CC593826	4	497	497	28	27	23			
585	7	39814634	39815032	CC496257	CC496257	CC496257	4	501	501	28	27	23			
586	7	38925632	38925968	BZ936282	BZ936282	BZ936282	4	501	501	28	27	deleted		AG is wrong in merging these two blocks, as other blocks comes in between	2 -> 1
588	7	117979966	117980046	CC479182	CC479182	CC479182	4	523	523	29	28	23	AG here		
589	7	118874111	118874200	BZ868117	BZ868117	BZ868117	4	538	538	29	28	23			
590	7	119902958	119903540	BZ849606	BZ849606	BZ849606	4	557	557	29	28	23			
591	7	120972367	120972628	BZ876586	BZ876586	BZ876586	4	565	565	29	28	23			
592	7	121274819	121274990	AW430276	AW430276	AW430276	4	569	569	29	28	23			
593	7	122890131	122890296	CC492581	CC492581	CC492581	4	574	574	29	28	23			
594	7	122199330	122199517	CC497750	CC497750	CC497750	4	583	583	29	28	23			
595	7	123802997	123803173	BZ901102	BZ901102	BZ901102	4	599	599	29	28	23			
596	7	124981879	124982282	CC520547	CC520547	CC520547	4	617	617	29	28	23			
597	7	125874002	125874068	BZ951950	BZ951950	BZ951950	4	628	628	29	28	23			
598	7	127122886	127123164	CC547739	CC547739	CC547739	4	643	643	29	28	23			
599	7	128099395	128099849	AW267076	AW267076	AW267076	4	667	667	29	28	23			
600	7	129064523	129064818	AW289176	AW289176	AW289176	4	671	671	29	28	23			
601	7	129184438	129184873	BZ862656	BZ862656	BZ862656	4	675	675	29	28	23			
602	7	129621618	129621879	M61851	M61851	M61851	4	679	679	29	28	23			
603	7	130136922	130137154	CC546178	CC546178	CC546178	4	690	690	29	28	23			
604	7	131071628	131072061	CC560915	CC560915	CC560915	4	690	690	29	28	23			
605	7	132082388	132082557	CC500550	CC500550	CC500550	4	690	690	29	28	23			
606	7	133152426	133152979	BZ945646	BZ945646	BZ945646	4	694	694	29	28	23			
607	7	134056302	134056477	CC582448	CC582448	CC582448	4	712	712	29	28	23			
608	7	135071687	135071955	AW267090	AW267090	AW267090	4	725	725	29	28	23			
609	7	136159835	136160082	CC484525	CC484525	CC484525	4	732	732	29	28	23			
610	7	137021183	137021253	BF440577	BF440577	BF440577	4	736	736	29	28	23			
611	7	137130375	137130612	CC768899	CC768899	CC768899	4	736	736	29	28	23			
612	7	138621614	138621814	CC580649	CC580649	CC580649	4	740	740	29	28	23			
613	7	139480451	139480519	CC475889	CC475889	CC475889	4	763	763	29	28	23			
615	7	140448858	140449056	CC773301	CC773301	CC773301	4	770	770	29	28	23			
616	7	141418871	141419053	BZ905854	BZ905854	BZ905854	4	777	777	29	28	23			
617	7	141944694	141944861	D38507	D38507	D38507	4	777	777	29	28	23			
618	7	142027115	142027383	AJ006347	AJ006347	AJ006347	4	780	780	29	28	23			
619	7	142361308	142361514	CC765767	CC765767	CC765767	4	790	790	29	28	23			
620	7	142648542	142648922	BZ875861	BZ875861	BZ875861	4	790	790	29	28	23			
622	7	143744459	143744680	CC544433	CC544433	CC544433	4	801	801	29	28	23			
623	7	144659213	144659428	CC520232	CC520232	CC520232	4	805	805	29	28	23			
624	7	145668935	145669460	CC468247	CC468247	CC468247	4	813	813	29	28	23			
625	7	146650920	146651328	CC571555	CC571555	CC571555	4	822	822	29	28	23			
626	7	147622843	147622996	CC579867	CC579867	CC579867	4	840	840	29	28	23			
627	7	148592920	148593030	CC575477	CC575477	CC575477	4	840	840	29	28	23			
628	7	149707927	149708117	BZ868730	BZ868730	BZ868730	4	850	850	29	28	23			
629	7	150191806	150191897	X82440	X82440	X82440	4	850	850	29	28	23			
630	7	151270500	151270835	AW487065	AW487065	AW487065	4	861	861	29	28	23			
631	7	152114288	152114381	CC574649	CC574649	CC574649	4	864	864	29	28	23			
632	7	153447811	153447907	CC580623	CC580623	CC580623	4	877	877	29	28	23			
633	7	154313538	154313663	CC562122	CC562122	CC562122	4	884	884	29	28	23			
634	7	155586613	155587122	CC571670	CC571670	CC571670	4	887	887	29	28	23			
635	7	156349754	156349837	CC569644	CC569644	CC569644	4	890	890	29	28	23			
636	7	157134098	157134232	CC556048	CC556048	CC556048	4	900	900	29	28	23			
637	12	69732779	69732992	CC491422	CC491422	CC491422	5	0	0	30	29	24			
638	12	70600956	70601336	CC550888	CC550888	CC550888	5	0	0	30	29	deleted			
639	12	71661479	71661855	CC548760	CC548760	CC548760	5	10	10	30	29	24			
640	12	72696121	72696286	CC495733	CC495733	CC495733	5	20	20	30	29	24			
643	12	73637561	73637698	CC494122	CC494122	CC494122	5	48	48	30	29	24			
644	12	74662243	74662405	CC536551	CC536551	CC536551	5	56	56	30	29	24			
645	12	75711371	75711485	CC571291	CC571291	CC571291	5	59	59	30	29	24			
647	12	76665523	76665932	CC555157	CC555157	CC555157	5	85	85	30	29	24			
648	12	77697599	77697686	CC577462	CC577462	CC577462	5	91	91	30	29	24			
649	12	78741509	78741657	CC536249	CC536249	CC536249	5	108	108	30	29	24			
650	12	79615079	79616351	X52526	X52526	X52526	5	108	108	30	29	24			
652	12	80580744	80580910	CC534803	CC534803	CC534803	5	118	118	30	29	24			
654	12	81721741	81721819	CC472518	CC472518	CC472518	5	135	135	30	29	24			
655	12	82766724	82767007	CC474349	CC474349	CC474349	5	142	142	30	29	24			
656	12	83757871	83758136	AW669970	AW669970	AW669970	5	148	148	30	29	24			
657	12	84774579	84774844	M18621	M18621	M18621	5	155	155	30	29	24			
658	12	85124984	85125113	CC543187	CC543187	CC543187	5	155	155	30	29	24			
659	12	86159480	86159764	BZ854168	BZ854168	BZ854168	5	162	162	30	29	24			
660	12	87215214	87215389	CC501438	CC501438	CC501438	5	162	162	30	29	24			
661	12	88235961	88236055	CC772429	CC772429	CC772429	5	174	174	30	29	24			
662	12	89230374	89230454	BZ951683	BZ951683	BZ951683	5	196	196	30	29	24			
663	12	89951572	89953132	AF036962	AF036962	AF036962	5	201	201	30	29	24			
664	12	90004997	90005167	AW267150	AW267150	AW267150	5	201	201	30	29	24			
665	12	90042247	90042499	AW266912	AW266912	AW266912	5	205	205	30	29	24			
666	12	90076226	90076298	CC482161	CC482161	CC482161	5	205	205	30	29	24			
667	12	91039376	91040693	AF014008	AF014008	AF014008	5	208	208	30	29	24			
668	12	91125590	91125818	BZ841627	BZ841627	BZ841627	5	208	208	30	29	24			
670	12	92091717	92091965	BZ947114	BZ947114	BZ947114	5	220	220	30	29	24			
672	12	93154399	93154522	CC582929	CC582929	CC582929	5	233	233	30	29	24			
673	12	94048994	94049288	BZ860833	BZ860833	BZ860833	5	256	256	30	29	24			
674	12	53249186	53249420	M94867	M94867	M94867	5	280	280	31	30	25			
677	12	53022255	53022340	X75935	X75935	X75935	5	291	291	31	30	25			
679	12	52091806	52092090	AW267153	AW267153	AW267153	5	301	301	31	30	25			
680	12	51149710	51149838	K03534	K03534	K03534	5	301	301	31	30	25			
683	12	51122472	51122886	CC587019	CC587019	CC587019	5	304	304	31	30	25			
684	12	50099430	50099781	CC502158	CC502158	CC502158	5	304	304	31	30	25			
685	12	49035971	49036497	CC507502	CC507502	CC507502	5	308	308	31	30	25			
686	12	48684142	48684483	AW479054	AW479054	AW479054	5	308	308	31	30	25			
687	12	48444490	48444803	AF093741	AF093741	AF093741	5	308	308	31	30	25			
690	12	47956371	47956498	BZ855220	BZ855220	BZ855220	5	323	323	31	30	25			
691	12	47251242	47251352	U63110	U63110	U63110	5	323	323	31	30	25			
692	12	46845319	46845386	CC537786	CC537786	CC537786	5	330	330	31	30	25			
693	12	46724047	46724308	AW356797	AW356797	AW356797	5	333	333	31	30	25			
694	12	46666620	46684368	L10347	L10347	L10347	5	337	337	31	30	25			
695	12	45872237	45872411	CC514396	CC514396	CC514396	5	341	341	31	30	25			
696	12	44878474	44878807	CC770103	CC770103	CC770103	5	344	344	31	30	25			
697	12	44009337	44009583	BZ832800	BZ832800	BZ832800	5	348	348	31	30	25			
698	12	41347761	41347850	CC771589	CC771589	CC771589	5	352	352	31	30	25			
699	12	40342356	40342810	BZ865499	BZ865499	BZ865499	5	365	365	31	30	25			
701	12	39377944	39378196	BZ844794	BZ844794	BZ844794	5	373	373	31	30	25			
702	12	38330060	38330627	BZ879493	BZ879493	BZ879493	5	373	373	31	30	25			
703	12	37354588	37354648	BZ922495	BZ922495	BZ922495	5	373	373	31	30	25			
704	12	69112657	69113033	AW632320	AW632320	AW632320	5	377	377	32	31	26			
705	12	68685470	68685572	BZ910903	BZ910903	BZ910903	5	377	377	32	31	26			
706	12	68028329	68028771	M95097	M95097	M95097	5	381	381	32	31	26			
707	12	66838848	66840308	Z54144	Z54144	Z54144	5	385	385	32	31	26			
708	12	66502236	66502873	BZ841315	BZ841315	BZ841315	5	385	385	32	31	26			
709	12	65596360	65596586	BZ935988	BZ935988	BZ935988	5	389	389	32	31	26			
710	12	64559560	64559795	BZ870422	BZ870422	BZ870422	5	394	394	32	31	26			
712	12	63499190	63499341	CC588856	CC588856	CC588856	5	398	398	32	31	26			
713	12	62591705	62591875	CC491981	CC491981	CC491981	5	398	398	32	31	26			
714	12	61311116	61311378	BZ831160	BZ831160	BZ831160	5	406	406	32	31	26			
715	12	60229439	60229716	BZ919691	BZ919691	BZ919691	5	410	410	32	31	26			
716	12	59124426	59124578	BZ889150	BZ889150	BZ889150	5	410	410	32	31	26			
717	12	58339502	58339818	CC521894	CC521894	CC521894	5	415	415	32	31	26			
718	12	57209808	57210432	CC555460	CC555460	CC555460	5	419	419	32	31	26			
719	12	56160650	56160743	CC516237	CC516237	CC516237	5	426	426	32	31	26			
721	12	55134284	55134713	K02818	K02818	K02818	5	430	430	32	31	26			
722	12	54637166	54637723	M81193	M81193	M81193	5	430	430	32	31	26			
723	12	54172421	54172599	BZ885308	BZ885308	BZ885308	5	437	437	32	31	26			
724	12	54374613	54374747	AW446663	AW446663	AW446663	5	441	441	32	31	26			
725	12	95165688	95166066	AW289389	AW289389	AW289389	5	445	445	33	32	27			
727	12	95996005	95996644	CC574714	CC574714	CC574714	5	449	449	33	32	27			
728	12	97025303	97025478	CC586694	CC586694	CC586694	5	452	452	33	32	27			
729	12	97497309	97497953	X77338	X77338	X77338	5	452	452	33	32	27			
730	12	98075189	98075275	BZ955659	BZ955659	BZ955659	5	457	457	33	32	27			
731	12	99004612	99004871	CC492179	CC492179	CC492179	5	457	457	33	32	27			
732	12	99994768	99995347	CC543865	CC543865	CC543865	5	461	461	33	32	27			
733	12	100633456	100633550	BE217470	BE217470	BE217470	5	461	461	33	32	27			
734	12	101297925	101298813	X15726	X15726	X15726	5	461	461	33	32	27			
735	12	101974760	101975000	CC472700	CC472700	CC472700	5	465	465	33	32	27			
736	12	102668581	102668651	AW484933	AW484933	AW484933	5	469	469	33	32	27			
737	12	103946236	103946321	BZ901784	BZ901784	BZ901784	5	469	469	33	32	27			
738	12	104924999	104925225	CC542945	CC542945	CC542945	5	473	473	33	32	27			
739	12	105844520	105844705	CC532927	CC532927	CC532927	5	487	487	33	32	27			
740	22	31129226	31129442	BZ932472	BZ932472	BZ932472	5	492	492	34	33	28			
741	22	31772691	31772843	CC508178	CC508178	CC508178	5	497	497	34	33	28			
742	22	32560079	32560355	CC540296	CC540296	CC540296	5	501	501	34	33	28			
743	22	33475996	33476331	BZ925941	BZ925941	BZ925941	5	501	501	34	33	28			
744	22	34261880	34262272	CC548186	CC548186	CC548186	5	510	510	34	33	28			
745	22	35328795	35329037	BZ831051	BZ831051	BZ831051	5	510	510	34	33	28			
747	22	36215993	36216107	CC523697	CC523697	CC523697	5	530	530	34	33	28			
748	12	6459823	6460142	AW425851	AW425851	AW425851	5	545	545	out-of-place	out-of-place	Singleton			
749	12	32791352	32791707	BZ950954	BZ950954	BZ950954	5	549	549	35	34	29			
750	12	32441924	32442321	CC510431	CC510431	CC510431	5	549	549	35	34	29			
751	12	31446647	31446820	CC566439	CC566439	CC566439	5	554	554	35	34	29			
752	12	30283324	30283440	BZ937673	BZ937673	BZ937673	5	559	559	35	34	29			
753	12	29283880	29283975	CC581810	CC581810	CC581810	5	559	559	35	34	29			
754	12	28280451	28280569	BZ953500	BZ953500	BZ953500	5	570	570	35	34	29			
755	12	27322024	27322391	CC483912	CC483912	CC483912	5	570	570	35	34	29			
756	12	26418950	26419547	BZ831509	BZ831509	BZ831509	5	570	570	35	34	29			
757	12	25564055	25564298	CC555130	CC555130	CC555130	5	575	575	35	34	29			
758	12	24604192	24604477	BZ861157	BZ861157	BZ861157	5	584	584	35	34	29			
760	12	23570950	23571518	CC537687	CC537687	CC537687	5	594	594	35	34	29			
762	12	22511922	22512258	CC523085	CC523085	CC523085	5	604	604	35	34	29			
763	12	21648653	21649249	CC503835	CC503835	CC503835	5	608	608	35	34	29			
764	12	20651674	20651908	BZ908158	BZ908158	BZ908158	5	608	608	35	34	29			
765	12	19500286	19501408	AF013213	AF013213	AF013213	5	613	613	35	34	29			
766	12	18625199	18625375	BZ857549	BZ857549	BZ857549	5	618	618	35	34	29			
767	12	17678113	17678556	BZ886512	BZ886512	BZ886512	5	623	623	35	34	29			
768	12	16656557	16656945	BZ868933	BZ868933	BZ868933	5	623	623	35	34	29			
770	12	15683442	15684243	CC582262	CC582262	CC582262	5	628	628	35	34	29			
771	12	14665241	14665421	AF081464	AF081464	AF081464	5	628	628	35	34	29			
772	12	13789137	13789489	CC766642	CC766642	CC766642	5	633	633	35	34	29			
773	12	12998528	12998976	CC501542	CC501542	CC501542	5	639	639	35	34	29			

774	12	11931993	11932576	CC492988	CC492988	CC492988	5	639	639	35	34	29			
775	12	11039262	11039469	BZ874300	BZ874300	BZ874300	5	639	639	35	34	29			
776	12	10210576	10210866	D89049	D89049	D89049	5	644	644	35	34	29			
777	12	8985633	8985808	M17025	M17025	M17025	5	644	644	35	34	29			
778	12	8094193	8094587	BZ905626	BZ905626	BZ905626	5	650	650	35	34	29			
779	12	7043360	7043580	CC548413	CC548413	CC548413	5	655	655	36	34	29	ST here	discrepant cases in HSB definitions between SyntenyTracker and Everts-van der Wind et al	
781	12	6217358	6217689	M81720	M81720	M81720	5	655	655	36	34	29			
782	12	5177758	5177905	CC480296	CC480296	CC480296	5	670	670	36	34	29			
784	12	4329189	4329534	BZ927078	BZ927078	BZ927078	5	674	674	36	34	29			
785	12	3167967	3168365	CC770412	CC770412	CC770412	5	678	678	36	34	29			
787	12	2290550	2291004	CC595288	CC595288	CC595288	5	695	695	36	34	29			
788	12	1833366	1833483	CC508553	CC508553	CC508553	5	699	699	36	34	29			
789	12	1262521	1262687	CC763960	CC763960	CC763960	5	699	699	36	34	29			
790	22	16648034	16648179	AW347612	AW347612	AW347612	5	699	699	37	35	deleted			
791	22	16821966	16822120	CC478740	CC478740	CC478740	5	707	707	37	35	30			
792	22	16926149	16926343	CC495717	CC495717	CC495717	5	710	710	37	35	30			
795	22	36494513	36494936	AW463030	AW463030	AW463030	5	714	714	38	36	31			
796	22	36934304	36934528	CC522426	CC522426	CC522426	5	718	718	38	36	31			
797	22	37011503	37011643	AW465847	AW465847	AW465847	5	718	718	38	36	31			
798	22	37998541	37998983	CC501001	CC501001	CC501001	5	722	722	38	36	31			
799	22	39093144	39093591	BZ839074	BZ839074	BZ839074	5	726	726	38	36	31			
800	22	40417049	40417534	BZ865444	BZ865444	BZ865444	5	751	751	38	36	31			
801	22	41164713	41164841	BZ880204	BZ880204	BZ880204	5	772	772	38	36	31			
802	22	41952226	41952395	CC476227	CC476227	CC476227	5	784	784	38	36	31			
804	22	43014871	43015104	CC499495	CC499495	CC499495	5	790	790	38	36	31			
806	22	43846010	43846088	CC493446	CC493446	CC493446	5	802	802	38	36	31			
807	22	44409410	44409598	CC770408	CC770408	CC770408	5	805	805	38	36	31			
808	22	45628435	45628555	CC537174	CC537174	CC537174	5	808	808	38	36	31			
809	22	46445601	46445761	CC563840	CC563840	CC563840	5	811	811	38	36	31			
810	22	47268882	47269111	BZ885527	BZ885527	BZ885527	5	814	814	38	36	31			
811	22	48468255	48468373	BZ860298	BZ860298	BZ860298	5	817	817	38	36	31			
812	22	48987753	48987855	AW484143	AW484143	AW484143	5	826	826	38	36	31			
813	22	49473146	49473658	X68212	X68212	X68212	5	826	826	38	36	31			
814	4	165763228	165763665	BZ839769	BZ839769	BZ839769	6	0	0	39	37	32			
816	4	165361532	165361728	BZ839793	BZ839793	BZ839793	6	4	4	39	37	32			
817	4	164733872	164734360	CC479967	CC479967	CC479967	6	16	16	39	37	32		AG is wrong in merging these two blocks, as other blocks comes in between	2 ->1
818	4	122479772	122480086	CC541186	CC541186	CC541186	6	32	32	40	38	32	AG here		
819	4	121508507	121508971	CC585365	CC585365	CC585365	6	40	40	40	38	32			
820	4	120491317	120491546	BZ920664	BZ920664	BZ920664	6	57	57	40	38	32			
821	4	119557937	119558004	AW461695	AW461695	AW461695	6	66	66	40	38	32			
822	4	119421712	119421956	BZ878015	BZ878015	BZ878015	6	70	70	40	38	32			
823	4	118307671	118308045	BZ908457	BZ908457	BZ908457	6	95	95	40	38	32			
824	4	117168624	117168702	CC529946	CC529946	CC529946	6	100	100	40	38	32			
825	4	117008232	117008844	CC499312	CC499312	CC499312	6	104	104	40	38	32			
826	4	116224292	116225023	CC537180	CC537180	CC537180	6	119	119	40	38	32			
827	4	115181769	115182527	CC519593	CC519593	CC519593	6	123	123	40	38	32			
829	4	114329179	114329548	BZ838237	BZ838237	BZ838237	6	130	130	40	38	32			
830	4	113321626	113321803	BZ941823	BZ941823	BZ941823	6	134	134	40	38	32			
831	4	112360154	112360645	CC771919	CC771919	CC771919	6	137	137	40	38	32			
832	4	111268230	111268673	CC555181	CC555181	CC555181	6	141	141	40	38	32			
833	4	111024993	111025262	BE217562	BE217562	BE217562	6	141	141	40	38	32			
834	4	110130829	110131222	BZ831816	BZ831816	BZ831816	6	144	144	40	38	32			
835	4	109520411	109520786	BZ833294	BZ833294	BZ833294	6	148	148	40	38	32			
836	4	108452819	108453052	BZ881520	BZ881520	BZ881520	6	152	152	40	38	32			
837	4	107470899	107471208	CC513945	CC513945	CC513945	6	156	156	40	38	32			
838	4	106324588	106324918	BZ863645	BZ863645	BZ863645	6	165	165	40	38	32			
839	4	105341878	105342463	BZ919177	BZ919177	BZ919177	6	178	178	40	38	32			
840	4	104417034	104417229	BZ882895	BZ882895	BZ882895	6	178	178	40	38	32			
841	4	104074090	104074459	AW266941	AW266941	AW266941	6	183	183	40	38	32			
842	4	103717232	103717759	BZ900461	BZ900461	BZ900461	6	198	198	40	38	32			
843	4	102425514	102425793	CC527810	CC527810	CC527810	6	202	202	40	38	32			
844	4	101147876	101147980	AW289359	AW289359	AW289359	6	206	206	40	38	32			
846	4	100698750	100699159	BZ885846	BZ885846	BZ885846	6	218	218	40	38	32			
847	4	99523512	99524023	BZ955226	BZ955226	BZ955226	6	230	230	40	38	32			
848	4	98447722	98447829	CC521461	CC521461	CC521461	6	239	239	40	38	32			
849	4	97394393	97394616	BZ864247	BZ864247	BZ864247	6	244	244	40	38	32			
850	4	96498266	96498551	BZ897709	BZ897709	BZ897709	6	264	264	40	38	32			
851	4	95242696	95243081	CC492227	CC492227	CC492227	6	264	264	40	38	32			
852	4	94291258	94291435	CC585366	CC585366	CC585366	6	273	273	40	38	32			
853	4	93267608	93267824	CC521028	CC521028	CC521028	6	278	278	40	38	32			
854	4	92186099	92186310	CC512565	CC512565	CC512565	6	278	278	40	38	32			
855	4	91044456	91044657	BZ899685	BZ899685	BZ899685	6	282	282	40	38	32			
856	4	89668992	89669220	BE664068	BE664068	BE664068	6	295	295	40	38	32			
857	4	18064723	18064790	CC581382	CC581382	CC581382	6	310	310	41	39	33			
858	4	19255066	19255895	CC524804	CC524804	CC524804	6	330	330	41	39	33			
859	4	20274304	20274547	BF652805	BF652805	BF652805	6	343	343	41	39	33			
860	4	21478748	21478883	CC526569	CC526569	CC526569	6	343	343	41	39	33			
861	4	22527736	22528229	CC570378	CC570378	CC570378	6	350	350	41	39	33			
864	4	23494872	23495273	CC580112	CC580112	CC580112	6	385	385	41	39	33			
865	4	24574354	24574544	CC576598	CC576598	CC576598	6	401	401	41	39	33			
866	4	25513021	25513326	BZ889411	BZ889411	BZ889411	6	401	401	41	39	33			
867	4	26762942	26763294	BZ907175	BZ907175	BZ907175	6	410	410	41	39	33			
868	4	28255453	28256028	BZ883331	BZ883331	BZ883331	6	414	414	41	39	33			
869	4	29073780	29074028	BZ949499	BZ949499	BZ949499	6	436	436	41	39	33			
871	4	30443627	30444071	CC552404	CC552404	CC552404	6	473	473	41	39	33			
872	4	31419214	31419429	CC520450	CC520450	CC520450	6	482	482	41	39	33			
873	4	32560075	32560502	CC498713	CC498713	CC498713	6	500	500	41	39	33			
874	4	33391783	33392312	BZ951628	BZ951628	BZ951628	6	509	509	41	39	33			
875	4	34407668	34407771	CC562367	CC562367	CC562367	6	522	522	41	39	33			
876	4	35706299	35706899	CC763699	CC763699	CC763699	6	529	529	41	39	33			
877	4	37112643	37112824	CC502049	CC502049	CC502049	6	542	542	41	39	33			
878	4	37435217	37435310	AW315312	AW315312	AW315312	6	550	550	41	39	33			
879	4	37844694	37844914	AW659593	AW659593	AW659593	6	554	554	41	39	33			
880	4	37956606	37956887	BZ853679	BZ853679	BZ853679	6	554	554	41	39	33			
881	4	38524187	38524601	AW425871	AW425871	AW425871	6	559	559	41	39	33			
882	4	39112405	39112660	AW484032	AW484032	AW484032	6	569	569	41	39	33			
883	4	39334539	39334742	AF095792	AF095792	AF095792	6	577	577	41	39	33			
884	4	40020528	40020740	CC528198	CC528198	CC528198	6	592	592	41	39	33			
885	4	41218673	41218770	BZ870510	BZ870510	BZ870510	6	621	621	41	39	33			
886	4	41802839	41803153	AW359255	AW359255	AW359255	6	643	643	41	39	33			
887	4	42287517	42287636	BZ888222	BZ888222	BZ888222	6	647	647	41	39	33			
888	4	43251962	43251997	BZ920062	BZ920062	BZ920062	6	660	660	41	39	33			
890	4	44354950	44355099	CC466552	CC466552	CC466552	6	660	660	41	39	33			
891	4	45423218	45423785	CC572631	CC572631	CC572631	6	665	665	41	39	33			
892	4	46093082	46093551	X12361	X12361	X12361	6	674	674	41	39	33			
893	4	46387523	46387664	BZ910951	BZ910951	BZ910951	6	674	674	41	39	33			
894	4	47469281	47469541	BZ856197	BZ856197	BZ856197	6	686	686	41	39	33			
895	4	48432449	48432891	CC531368	CC531368	CC531368	6	698	698	41	39	33			
896	4	48895472	48895740	CC541039	CC541039	CC541039	6	706	706	41	39	33			
897	4	52548175	52548449	CC587331	CC587331	CC587331	6	706	706	41	39	33			
898	4	53639704	53640136	BZ952420	BZ952420	BZ952420	6	710	710	41	39	33			
900	4	54804212	54804502	CC520984	CC520984	CC520984	6	726	726	41	39	33			
901	4	54679010	54679224	BE217464	BE217464	BE217464	6	730	730	41	39	33			
902	4	55419912	55420150	AW266986	AW266986	AW266986	6	750	750	41	39	33			
903	4	55722017	55722084	CC529259	CC529259	CC529259	6	750	750	41	39	33			
904	4	56749185	56749231	BZ899215	BZ899215	BZ899215	6	758	758	41	39	33			
905	4	57209633	57210274	AF013091	AF013091	AF013091	6	765	765	41	39	33			
906	4	57355092	57355637	AW289223	AW289223	AW289223	6	765	765	41	39	33			
907	4	57732408	57732662	BZ865321	BZ865321	BZ865321	6	778	778	41	39	33			
908	4	58756824	58756937	CC582433	CC582433	CC582433	6	796	796	41	39	33			
909	4	59702948	59703410	CC474742	CC474742	CC474742	6	800	800	41	39	33			
910	4	60462797	60462943	CC550121	CC550121	CC550121	6	800	800	41	39	33			
911	4	61496530	61497347	CC517016	CC517016	CC517016	6	800	800	41	39	33			
912	4	62544034	62544455	CC470126	CC470126	CC470126	6	809	809	41	39	33			
913	4	63808004	63808313	CC495525	CC495525	CC495525	6	817	817	41	39	33			
914	4	64999579	64999762	BZ843260	BZ843260	BZ843260	6	825	825	41	39	33			
915	4	66045303	66045370	BZ868471	BZ868471	BZ868471	6	839	839	41	39	33			
916	4	67142085	67142518	CC506073	CC506073	CC506073	6	856	856	41	39	33			
917	4	68187321	68187645	BZ932040	BZ932040	BZ932040	6	860	860	41	39	33			
918	4	68448599	68449621	AF034950	AF034950	AF034950	6	863	863	41	39	33			
919	4	68520128	68520334	BZ857285	BZ857285	BZ857285	6	863	863	41	39	33			
920	4	69032013	69032397	BZ898706	BZ898706	BZ898706	6	867	867	41	39	33			
921	4	69245158	69245248	CC584883	CC584883	CC584883	6	875	875	41	39	33			
922	4	70857267	70857423	CC592770	CC592770	CC592770	6	886	886	41	39	33			
923	4	70890634	70890818	X56395	X56395	X56395	6	894	894	41	39	33			
924	4	72015261	72015644	CC520715	CC520715	CC520715	6	902	902	41	39	33			
926	4	72901944	72902750	CC533562	CC533562	CC533562	6	911	911	41	39	33			
927	4	73895926	73896361	BZ896619	BZ896619	BZ896619	6	921	921	41	39	33			
928	4	74759142	74759318	CC486461	CC486461	CC486461	6	926	926	41	39	33			
929	4	75329352	75329436	AW463349	AW463349	AW463349	6	941	941	41	39	33			
930	4	76072337	76072563	CC480323	CC480323	CC480323	6	941	941	41	39	33			
933	4	77166370	77166712	BZ939762	BZ939762	BZ939762	6	994	994	41	39	33			
934	4	77437563	77437739	AW462095	AW462095	AW462095	6	1009	1009	41	39	33			
935	4	77588629	77588804	BE217509	BE217509	BE217509	6	1009	1009	41	39	33			
936	4	78152106	78152216	CC589119	CC589119	CC589119	6	1012	1012	41	39	33			
937	4	79113495	79113559	CC486313	CC486313	CC486313	6	1026	1026	41	39	33			
938	4	80197695	80198045	CC533234	CC533234	CC533234	6	1039	1039	41	39	33			
939	4	80841262	80841744	BZ884130	BZ884130	BZ884130	6	1043	1043	41	39	33			
940	4	82324049	82324539	CC561508	CC561508	CC561508	6	1043	1043	41	39	33			
941	4	82745839	82746216	BF440269	BF440269	BF440269	6	1047	1047	41	39	33			
943	4	83763410	83763495	AW325685	AW325685	AW325685	6	1076	1076	41	39	33			
944	4	84600347	84600827	BZ861871	BZ861871	BZ861871	6	1088	1088	41	39	33			
945	4	85654368	85654576	BZ842188	BZ842188	BZ842188	6	1096	1096	41	39	33			
946	4	86734355	86734634	CC512806	CC512806	CC512806	6	1100	1100	41	39	33			
947	4	87671529	87671654	CC768714	CC768714	CC768714	6	1104	1104	41	39	33			
948	4	88751837	88752148	BZ944321	BZ944321	BZ944321	6	1108	1108	41	39	33			
949	4	88757751	88757912	AW427728	AW427728	AW427728	6	1120	1120	41	39	33			
951	4	6539971	6540328	CC596055	CC596055	CC596055	6	1134	1134	42	40	34			
953	4	5941145	5941228	U96722	U96722	U96722	6	1138	1138	42	40	34			
954	4	5451497	5451700	CC579234	CC579234	CC579234	6	1138	1138	42	40	34			
955	4	4387601	4387825	AW484424	AW484424	AW484424	6	1138	1138	42	40	34			
956	4	3116687	3116762	CC582560	CC582560	CC582560	6	1146	1146	42	40	34			
957	4	2078145	2078455	CC502755	CC502755	CC502755	6	1154	1154	42	40	34			
958	4	941437	941739	CC488268	CC488268	CC488268	6	1154	1154	42	40	34			
959	4	9752608	9752728	AW428597	AW428597	AW428597	6	1182	1182	43	41	35			
960	4	11613766	11614028	CC519487	CC519487	CC519487	6	1185	1185	43	41	35			
961	4	10661259	10661420	CC588098	CC588098	CC588098	6	1185	1185	43	41	35			
962	4	12683463	12683684	CC474429	CC474429	CC474429	6	1185	1185	43	41	35			
963	4	13971029	13971262	BZ886679	BZ886679	BZ886679	6	1195	1195	43	41	35			
964	4	14864442	14864533	CC518331	CC518331	CC518331	6	1198	1198	43	41	35			
965	4	15902778	15903236	CC763342	CC763342	CC763342	6	1198	1198	43	41	35			
966	4	16967258	16967305	BZ923225	BZ923225	BZ923225	6	1216	1216	43	41	35			
967	4	17164830	17164996	AW462484	AW462484	AW462484	6	1219	1219	43	41	35			
968	4	9557597	9558213	BZ832277	BZ832277	BZ832277	6	1231	1231	44	42	36			
969	4	8535669	8535730	AW425166	AW425166	AW425166	6	1239	1239	44	42	36			
970	4	8128836	8128996	CC537302	CC537302	CC537302	6	1251	1251	44	42	36			
972	4	7816989	7817089	BZ886368	BZ886368	BZ886368	6	1254	1254	44	42	36			
973	4	7173876	7174301	AW462174	AW462174	AW462174	6	1254	1254	44	42	36			
974	4	6836050	6836158	AW462096	AW462096	AW462096	6	1263	1263	44	42	36			
975	1	224889520	224889761	AW485469	AW485469	AW485469	7	0	0	45	43	37			
976	1	224836532	224836672	CC549620	CC549620	CC549620	7	9	9	45	43	37			
977	1	224709261	224709541	BZ954984	BZ954984	BZ954984	7	9	9	45	43	deleted			
978	1	224641838	224641981	X67029	X67029	X67029	7	9	9	45	43	deleted			
980	5	178577969	178578296	CC478860	CC478860	CC478860	7	17	17	46	44	38			
981	5	179698097	179698371	BZ870570	BZ870570	BZ870570	7	20	20	46	44	38			
982	19	19515095	19515219	BZ903765	BZ903765	BZ903765	7	32	32	47	45	39			
983	19	19165746	19165971	AW447133	AW447133	AW447133	7	35	35	47	45	39			
984	19	18756684	18756903	X74326	X74326	X74326	7	43	43	47	45	39			
985	19	18486792	18486924	CC544911	CC544911	CC544911	7	46	46	47	45	39			
987	19	17476232	17476339	CC522915	CC522915	CC522915	7	64	64	47	45	39			
988	19	16456989	16457078	CC570190	CC570190	CC570190	7	73	73	47	45	39			
990	19	15419941	15420401	BZ882503	BZ882503	BZ882503	7	83	83	47	45	39			
991	19	15141868	15141998	CC447801	CC447801	CC447801	7	87	87	47	45	39			
992	19	14806913	14807011	CC583776	CC583776	CC583776	7	91	91	47	45	39			
993	19	14065436	14065606	X67154	X67154	X67154	7	104	104	47	45	39			
994	19	13641714	13642188	BZ859240	BZ859240	BZ859240	7	104	104	47	45	39			
996	19	13126074	13126308	AW289324	AW289324	AW289324	7	119	119	47	45	39			
997	19	12666629	12666841	BZ942760	BZ942760	BZ942760	7	133	133	47	45	39			
998	19	11546970	11547087	CC470594	CC470594	CC470594	7	153	153	47	45	39			
999	19	11546482	11546667	BF440357	BF440357	BF440357	7	153	153	47	45	39			
1000	19	10957862	10957946	CC763429	CC763429	CC763429	7	166	166	47	45	39			
1001	19	10780246	10780389	AW289273	AW289273	AW289273	7	171	171	47	45	39			
1002	19	10255161	10255459	L41844	L41844	L41844	7	171	171	47	45	39			
1003	19	10233576	10233652	AW289205	AW289205	AW289205	7	175	175	47	45	39			
1004	19	9839143	9839371	CC476302	CC476302	CC476302	7	180	180	47	45	39			
1005	19	8837760	8837880	CC551316	CC551316	CC551316	7	188	188	47	45	39			
1006	19	7890600	7890652	AW461439	AW461439	AW461439	7	207	207	47	45	39			
1007	19	7700211	7700432	CC567486	CC567486	CC567486	7	215	215	47	45	39			
1008	19	6671521	6671711	BZ888743	BZ888743	BZ888743	7	223	223	47	45	39			
1009	19	5543273	5543394	CC498294	CC498294	CC498294	7	232	232	47	45	39			
1010	19	5104337	5104608	AW289202	AW289202	AW289202	7	232	232	47	45	39			
1011	19	3447532	3447754	BZ874222	BZ874222	BZ874222	7	236	236	47	45	39			
1012	19	2157716	2157817	CC481348	CC481348	CC481348	7	241	241	47	45	39			
1013	19	4506970	4507407	CC531456	CC531456	CC531456	7	246	246	47	45	39			
1014	5	132046071	132046178	M77120	M77120	M77120	7	250	250	48	46	40			
1015	5	131851487	131851759	AW289222	AW289222	AW289222	7	255	255	48	46	40			
1016	5	131573876	131574056	BE217484	BE217484	BE217484	7	259	259	48	46	40			
1017	5	131426653	131426723	L31893	L31893	L31893	7	268	268	48	46	40			
1018	5	130722556	130723233	CC547431	CC547431	CC547431	7	277	277	48	46	40			
1019	5	129580135	129580537	CC516121	CC516121	CC516121	7	282	282	48	46	40			
1020	5	128466880	128467181	CC479287	CC479287	CC479287	7	286	286	48	46	40			
1021	5	127564422	127564570	BZ949745	BZ949745	BZ949745	7	286	286	48	46	40			
1022	5	126464579	126464636	BZ952486	BZ952486	BZ952486	7	295	295	48	46	40			
1023	5	125380532	125380738	CC762630	CC762630	CC762630	7	304	304	48	46	40			
1024	5	124265895	124266463	BZ903721	BZ903721	BZ903721	7	318	318	48	46	40			
1025	5	123173756	123174331	CC545868	CC545868	CC545868	7	318	318	48	46	40			
1027	5	122708712	122708835	AW357995	AW357995	AW357995	7	334	334	48	46	40			
1028	5	122062619	122063227	CC768511	CC768511	CC768511	7	334	334	48	46	40			
1029	5	121053231	121053723	CC541767	CC541767	CC541767	7	334	334	48	46	40			
1030	5	119873742	119874046	BZ932665	BZ932665	BZ932665	7	343	343	48	46	40			
1031	5	119194306	119194582	BZ840228	BZ840228	BZ840228	7	343	343	48	46	40			
1032	5	118134835	118134980	BZ911749	BZ911749	BZ911749	7	351	351	48	46	40			
1033	5	117105358	117105858	BZ937411	BZ937411	BZ937411	7	359	359	48	46	40			
1034	5	116114421	116114525	BZ883977	BZ883977	BZ883977	7	368	368	48	46	40		AG is wrong in merging these two blocks, as other blocks comes in between	2 -> 1
1035	5	177474906	177475077	BZ954134	BZ954134	BZ954134	7	379	379	49	47	40	AG here		
1036	5	176757787	176757987	CC771234	CC771234	CC771234	7	379	379	49	47	40			
1037	1	243913051	243913365	BZ933147	BZ933147	BZ933147	7	397	397	50	48	41			
1038	1	244423201	244423352	CC563229	CC563229	CC563229	7	410	410	50	48	41			
1039	1	245314776	245314911	AW358619	AW358619	AW358619	7	433	433	50	48	41			

1040	19	277505	277542	CC578938	CC578938	CC578938	7	467	467	51	49	42
1041	19	1276404	1276527	BZ901546	BZ901546	BZ901546	7	471	471	51	49	42
1042	19	1529414	1529540	AW632312	AW632312	AW632312	7	471	471	51	49	42
1043	19	1932233	1932309	AW261150	AW261150	AW261150	7	476	476	51	49	42
1044	5	132239612	132239879	AW314850	AW314850	AW314850	7	476	476	52	50	deleted
1045	5	132806004	132806285	BZ937600	BZ937600	BZ937600	7	489	489	52	50	43
1046	5	133335958	133336455	X75068	X75068	X75068	7	497	497	52	50	43
1047	5	133560686	133561527	X72858	X72858	X72858	7	501	501	52	50	43
1048	5	134898143	134898600	CC768198	CC768198	CC768198	7	527	527	52	50	43
1049	5	135884803	135885410	CC551706	CC551706	CC551706	7	540	540	52	50	43
1050	5	136339746	136340000	AW359237	AW359237	AW359237	7	540	540	52	50	43
1052	5	137087653	137088014	CC525807	CC525807	CC525807	7	556	556	52	50	43
1053	5	137115161	137115700	AW289339	AW289339	AW289339	7	556	556	52	50	43
1054	5	138101717	138101872	BZ911786	BZ911786	BZ911786	7	565	565	52	50	43
1055	5	139157019	139157072	BZ881009	BZ881009	BZ881009	7	573	573	52	50	43
1056	5	139991926	139992217	U48356	U48356	U48356	7	573	573	52	50	43
1057	5	140148501	140148577	BZ888174	BZ888174	BZ888174	7	573	573	52	50	43
1058	5	141141286	141141824	BZ861104	BZ861104	BZ861104	7	573	573	52	50	43
1059	5	141360420	141360648	AW428576	AW428576	AW428576	7	577	577	52	50	43
1060	5	142103330	142103750	CC587863	CC587863	CC587863	7	585	585	52	50	43
1061	5	143185013	143185108	CC479340	CC479340	CC479340	7	589	589	52	50	43
1062	5	144230399	144230549	BZ944716	BZ944716	BZ944716	7	603	603	52	50	43
1063	5	145300639	145300876	CC557183	CC557183	CC557183	7	616	616	52	50	43
1064	5	145949260	145949935	AW289211	AW289211	AW289211	7	637	637	52	50	43
1065	5	146182590	146182943	CC477180	CC477180	CC477180	7	644	644	52	50	43
1066	5	147072059	147072550	BZ860778	BZ860778	BZ860778	7	655	655	52	50	43
1067	5	148186682	148187474	CC483684	CC483684	CC483684	7	684	684	52	50	43
1068	5	149171771	149172185	BZ843681	BZ843681	BZ843681	7	715	715	52	50	43
1069	5	149761393	149761490	AW289266	AW289266	AW289266	7	740	740	52	50	43
1071	5	150678581	150678760	AW461573	AW461573	AW461573	7	788	788	52	50	43
1072	5	150388480	150388739	AW266929	AW266929	AW266929	7	788	788	52	50	43
1073	5	150058967	150059143	CC584482	CC584482	CC584482	7	797	797	52	50	43
1074	5	151022197	151022405	AW266930	AW266930	AW266930	7	817	817	52	50	43
1075	5	151083969	151084188	CC590913	CC590913	CC590913	7	832	832	52	50	43
1076	5	151982526	151982860	CC582993	CC582993	CC582993	7	841	841	52	50	43
1077	5	153030382	153030650	BZ870791	BZ870791	BZ870791	7	865	865	52	50	43
1078	5	154130945	154131060	BZ917719	BZ917719	BZ917719	7	869	869	52	50	43
1080	5	154984697	154984967	BZ905998	BZ905998	BZ905998	7	883	883	52	50	43
1081	5	156037855	156038294	BZ951679	BZ951679	BZ951679	7	887	887	52	50	43
1082	5	157154336	157154819	CC531675	CC531675	CC531675	7	887	887	52	50	43
1083	5	158086177	158086342	BZ920099	BZ920099	BZ920099	7	892	892	52	50	43
1084	5	159096910	159097024	CC497805	CC497805	CC497805	7	906	906	52	50	43
1085	5	160128796	160129323	BZ913937	BZ913937	BZ913937	7	906	906	52	50	43
1087	5	161152435	161152922	BZ847699	BZ847699	BZ847699	7	926	926	52	50	43
1088	5	161256693	161257416	X05717	X05717	X05717	7	931	931	52	50	43
1089	5	162213197	162213335	BZ909547	BZ909547	BZ909547	7	946	946	52	50	43
1090	5	163165942	163166029	CC471837	CC471837	CC471837	7	962	962	52	50	43
1091	5	163931585	163931703	BZ846772	BZ846772	BZ846772	7	967	967	52	50	43
1092	5	164022267	164022400	CC475167	CC475167	CC475167	7	972	972	52	50	43
1093	5	165068595	165068897	BZ881131	BZ881131	BZ881131	7	987	987	52	50	43
1094	5	166095605	166095859	CC509309	CC509309	CC509309	7	1005	1005	52	50	43
1095	5	167081560	167081846	BZ915633	BZ915633	BZ915633	7	1005	1005	52	50	deleted
1096	5	80090362	80090620	BZ913783	BZ913783	BZ913783	7	1021	1021	53	51	44
1097	5	80661646	80661969	AW314133	AW314133	AW314133	7	1048	1048	53	51	44
1098	5	81115981	81116347	CC487999	CC487999	CC487999	7	1048	1048	53	51	44
1099	5	82432294	82432682	CC494938	CC494938	CC494938	7	1083	1083	53	51	44
1100	5	82972312	82973398	U02292	U02292	U02292	7	1099	1099	53	51	44
1101	5	83141883	83142326	BZ946767	BZ946767	BZ946767	7	1099	1099	53	51	44
1102	5	83911452	83912227	CC525453	CC525453	CC525453	7	1118	1118	53	51	44
1103	5	85198930	85199156	BZ891101	BZ891101	BZ891101	7	1130	1130	53	51	44
1104	5	86377688	86377977	CC521453	CC521453	CC521453	7	1152	1152	53	51	44
1105	5	87476448	87476763	CC532431	CC532431	CC532431	7	1167	1167	53	51	44
1106	5	88068280	88068564	AW289198	AW289198	AW289198	7	1182	1182	53	51	44
1107	5	88635353	88635415	CC590259	CC590259	CC590259	7	1191	1191	53	51	44
1108	5	89671480	89671670	CC515604	CC515604	CC515604	7	1200	1200	53	51	44
1109	5	90749392	90749529	BZ875785	BZ875785	BZ875785	7	1208	1208	53	51	44
1110	5	91846510	91846597	BZ836902	BZ836902	BZ836902	7	1212	1212	53	51	44
1111	5	92838198	92838471	BZ887450	BZ887450	BZ887450	7	1224	1224	53	51	44
1113	5	93852208	93852418	BZ876868	BZ876868	BZ876868	7	1235	1235	53	51	44
1114	5	94891456	94891763	BZ938128	BZ938128	BZ938128	7	1242	1242	53	51	44
1115	5	95890600	95890738	BZ951943	BZ951943	BZ951943	7	1250	1250	53	51	44
1116	5	96456168	96456372	AW314546	AW314546	AW314546	7	1255	1255	53	51	44
1117	5	96910013	96910119	CC530755	CC530755	CC530755	7	1270	1270	53	51	44
1118	5	98133845	98134167	BZ930206	BZ930206	BZ930206	7	1288	1288	53	51	44
1120	5	99002020	99002424	BZ925399	BZ925399	BZ925399	7	1307	1307	53	51	44
1121	5	100025212	100025496	CC525525	CC525525	CC525525	7	1318	1318	53	51	44
1122	5	101030868	101031077	CC483975	CC483975	CC483975	7	1325	1325	53	51	44
1124	5	101950040	101950518	CC593804	CC593804	CC593804	7	1328	1328	53	51	44
1125	5	102950412	102950928	CC577385	CC577385	CC577385	7	1336	1336	53	51	44
1126	5	104978330	104978717	CC590330	CC590330	CC590330	7	1344	1344	53	51	44
1127	5	104095140	104095638	BZ907418	BZ907418	BZ907418	7	1348	1348	53	51	44
1128	5	106037196	106037388	CC578005	CC578005	CC578005	7	1370	1370	53	51	44
1129	5	106740601	106740916	AW485404	AW485404	AW485404	7	1370	1370	53	51	44
1130	5	107022010	107022094	CC559825	CC559825	CC559825	7	1370	1370	53	51	44
1131	5	108044992	108045328	CC561997	CC561997	CC561997	7	1389	1389	53	51	44
1132	5	108896668	108897020	CC595292	CC595292	CC595292	7	1410	1410	53	51	44
1133	5	109725853	109726214	BZ921795	BZ921795	BZ921795	7	1410	1410	53	51	deleted
1135	4	170061557	170061775	CC522148	CC522148	CC522148	8	0	0	54	52	45
1136	4	170223519	170223805	AW464696	AW464696	AW464696	8	16	16	54	52	45
1137	4	170986403	170986567	CC500643	CC500643	CC500643	8	20	20	54	52	45
1138	4	171632491	171632656	CC566492	CC566492	CC566492	8	24	24	54	52	45
1140	4	173044366	173044859	CC510345	CC510345	CC510345	8	44	44	54	52	45
1141	4	174432156	174432341	BZ926647	BZ926647	BZ926647	8	54	54	54	52	45
1142	4	175526306	175526900	BZ872734	BZ872734	BZ872734	8	57	57	54	52	45
1143	4	176621233	176621314	CC498901	CC498901	CC498901	8	60	60	54	52	45
1144	8	11733867	11734116	AW266916	AW266916	AW266916	8	66	66	55	53	46
1145	8	11653144	11653533	CC515941	CC515941	CC515941	8	70	70	55	53	46
1146	8	10958948	10959168	CC475217	CC475217	CC475217	8	76	76	55	53	46
1147	8	10323066	10323335	U37150	U37150	U37150	8	86	86	55	53	46
1149	8	9919225	9919299	CC549513	CC549513	CC549513	8	89	89	55	53	46
1150	8	28806129	28806394	CC509153	CC509153	CC509153	8	99	99	56	54	47
1151	8	27854241	27854542	BZ932607	BZ932607	BZ932607	8	103	103	56	54	47
1152	8	27518364	27518781	J05391	J05391	J05391	8	103	103	56	54	deleted
1153	9	32562906	32563068	AW327178	AW327178	AW327178	8	103	103	57	55	deleted
1154	9	32189838	32190160	CC582025	CC582025	CC582025	8	106	106	57	55	48
1155	9	31213143	31213479	CC536082	CC536082	CC536082	8	122	122	57	55	48
1156	9	29907216	29907939	CC553770	CC553770	CC553770	8	130	130	57	55	48
1157	9	29068207	29068489	BZ869911	BZ869911	BZ869911	8	144	144	57	55	48
1158	9	28197719	28197884	BZ867418	BZ867418	BZ867418	8	144	144	57	55	48
1160	9	27099287	27099733	X71424	X71424	X71424	8	192	192	57	55	48
1161	9	26907303	26907787	BZ954292	BZ954292	BZ954292	8	192	192	57	55	48
1162	9	26055844	26056295	CC536261	CC536261	CC536261	8	196	196	57	55	48
1163	9	25043165	25043727	BZ846905	BZ846905	BZ846905	8	200	200	57	55	48
1165	9	24066710	24066831	CC542582	CC542582	CC542582	8	208	208	57	55	48
1166	9	23027353	23028023	CC501306	CC501306	CC501306	8	222	222	57	55	48
1167	9	22020884	22020983	BZ901788	BZ901788	BZ901788	8	233	233	57	55	48
1169	9	20917941	20918448	BZ930819	BZ930819	BZ930819	8	262	262	57	55	48
1170	9	19917487	19917842	CC543482	CC543482	CC543482	8	262	262	57	55	48
1171	9	18612293	18612849	CC572298	CC572298	CC572298	8	273	273	57	55	48
1172	9	17604277	17604558	CC545975	CC545975	CC545975	8	273	273	57	55	48
1173	9	16678755	16679105	BZ860064	BZ860064	BZ860064	8	278	278	57	55	48
1174	9	15736406	15736542	BZ936404	BZ936404	BZ936404	8	288	288	57	55	48
1175	9	14814070	14814161	BZ910885	BZ910885	BZ910885	8	288	288	57	55	48
1176	9	13890407	13890800	CC504863	CC504863	CC504863	8	293	293	57	55	48
1177	9	12806940	12807246	CC538887	CC538887	CC538887	8	302	302	57	55	48
1178	9	11798611	11799225	CC566828	CC566828	CC566828	8	306	306	57	55	48
1179	9	10667467	10668082	CC587820	CC587820	CC587820	8	320	320	57	55	48
1180	9	9671951	9672357	CC586969	CC586969	CC586969	8	329	329	57	55	48
1181	9	8577293	8577807	BZ951668	BZ951668	BZ951668	8	333	333	57	55	48
1182	9	7773926	7774200	BZ874683	BZ874683	BZ874683	8	351	351	57	55	48
1183	9	6488658	6488955	BZ869880	BZ869880	BZ869880	8	370	370	57	55	48
1185	9	6114920	6115086	CC585812	CC585812	CC585812	8	387	387	57	55	48
1186	9	5067449	5067676	CC593151	CC593151	CC593151	8	392	392	57	55	48
1187	9	4196283	4197202	CC475247	CC475247	CC475247	8	392	392	57	55	48
1188	9	3164949	3165018	CC475150	CC475150	CC475150	8	423	423	57	55	48
1189	9	2643827	2644347	AF016537	AF016537	AF016537	8	436	436	57	55	48
1190	9	2203373	2203598	CC775749	CC775749	CC775749	8	444	444	57	55	48
1191	9	1197663	1197747	CC547714	CC547714	CC547714	8	444	444	57	55	48
1192	9	454989	455070	AW487143	AW487143	AW487143	8	448	448	57	55	48
1193	9	267990	268118	CC525200	CC525200	CC525200	8	451	451	57	55	48
1195	9	68373995	68374224	AW428593	AW428593	AW428593	8	451	451	58	56	deleted
1196	9	68374737	68374852	AW427130	AW427130	AW427130	8	451	451	58	56	deleted
1197	9	68548002	68548129	BZ945607	BZ945607	BZ945607	8	455	455	58	56	49
1198	9	69522803	69523051	BZ834341	BZ834341	BZ834341	8	455	455	58	56	49
1199	9	70414453	70414699	CC577630	CC577630	CC577630	8	467	467	58	56	49
1200	9	71060296	71060494	BE217448	BE217448	BE217448	8	485	485	58	56	49
1201	9	71394164	71394529	BZ952525	BZ952525	BZ952525	8	492	492	58	56	49
1202	9	72447311	72447464	BZ905319	BZ905319	BZ905319	8	528	528	58	56	49
1203	9	72884707	72884824	BE217478	BE217478	BE217478	8	528	528	58	56	49
1204	9	73359756	73359923	BZ916333	BZ916333	BZ916333	8	531	531	58	56	49
1205	9	74366715	74367365	BZ851633	BZ851633	BZ851633	8	536	536	58	56	49
1206	9	75374508	75374791	BZ847149	BZ847149	BZ847149	8	546	546	58	56	49
1208	9	76458769	76459273	CC469487	CC469487	CC469487	8	570	570	58	56	49
1209	9	77348512	77348725	BZ946507	BZ946507	BZ946507	8	575	575	58	56	49
1210	9	78378501	78378622	BZ942115	BZ942115	BZ942115	8	577	577	58	56	49
1211	9	79402944	79403130	CC565564	CC565564	CC565564	8	583	583	58	56	49
1212	9	80283717	80284050	BZ880083	BZ880083	BZ880083	8	593	593	58	56	49
1213	9	81494562	81494741	BZ859283	BZ859283	BZ859283	8	616	616	58	56	49
1214	9	82562764	82563463	CC472145	CC472145	CC472145	8	618	618	58	56	49
1216	9	35341225	35341506	BZ936059	BZ936059	BZ936059	8	621	621	59	57	50
1217	9	35796052	35796241	X66865	X66865	X66865	8	621	621	59	57	50
1218	9	36462628	36462680	BZ877028	BZ877028	BZ877028	8	624	624	59	57	50
1219	9	37428233	37428450	AW479677	AW479677	AW479677	8	626	626	59	57	50
1220	9	37856108	37856487	AW289238	AW289238	AW289238	8	626	626	59	57	50
1221	9	38446092	38446286	CC468834	CC468834	CC468834	8	668	668	59	57	50
1222	9	97165575	97165753	BZ917613	BZ917613	BZ917613	8	684	684	60	58	51
1223	9	97922592	97922732	CC503570	CC503570	CC503570	8	705	705	60	58	51
1224	9	98952460	98952587	BZ874978	BZ874978	BZ874978	8	705	705	60	58	51
1225	9	100094836	100095251	BZ862965	BZ862965	BZ862965	8	716	716	60	58	51
1226	8	20149292	20149438	AW484959	AW484959	AW484959	8	776	776	61	59	52
1227	8	19857630	19857874	M16966	M16966	M16966	8	782	782	61	59	52
1228	8	20602219	20602559	CC564785	CC564785	CC564785	8	794	794	61	59	52
1229	8	21597153	21597251	BZ950977	BZ950977	BZ950977	8	794	794	61	59	52
1230	8	22125047	22125262	AW358555	AW358555	AW358555	8	805	805	61	59	52
1231	8	22507187	22507323	AW315330	AW315330	AW315330	8	815	815	61	59	52
1232	8	22528971	22529061	CC771284	CC771284	CC771284	8	815	815	61	59	52
1233	8	23720801	23721268	BZ888740	BZ888740	BZ888740	8	815	815	61	59	52
1234	8	24799790	24800425	CC573318	CC573318	CC573318	8	820	820	61	59	52
1235	8	25888046	25888347	CC771004	CC771004	CC771004	8	826	826	61	59	52
1236	8	26963982	26964532	CC509264	CC509264	CC509264	8	837	837	61	59	52
1237	9	33100642	33100760	Z71853	Z71853	Z71853	8	842	842	62	60	53
1238	9	33238287	33238511	BZ847030	BZ847030	BZ847030	8	842	842	62	60	53
1239	9	34390969	34391221	CC541524	CC541524	CC541524	8	857	857	62	60	53
1240	9	34600720	34601017	AW289344	AW289344	AW289344	8	863	863	62	60	53
1241	9	83875596	83875676	BZ844111	BZ844111	BZ844111	8	881	881	63	61	54
1242	9	84868603	84868739	AW353017	AW353017	AW353017	8	892	892	63	61	54
1243	9	85848000	85848255	CC589694	CC589694	CC589694	8	929	929	63	61	54
1245	9	86827883	86828038	BZ914519	BZ914519	BZ914519	8	954	954	63	61	54
1246	9	87618194	87618286	CC588903	CC588903	CC588903	8	963	963	63	61	54
1248	9	94977185	94977352	U73585	U73585	U73585	8	984	984	64	62	55
1249	9	95575706	95575955	CC568112	CC568112	CC568112	8	992	992	64	62	55
1250	9	94134571	94134917	BZ939826	BZ939826	BZ939826	8	1010	1010	64	62	55
1251	9	93089604	93089680	CC486779	CC486779	CC486779	8	1013	1013	64	62	55
1252	9	92553397	92553702	AW289231	AW289231	AW289231	8	1017	1017	64	62	55
1253	9	91565465	91565701	AW486041	AW486041	AW486041	8	1045	1045	64	62	55
1254	9	90889815	90890156	CC494631	CC494631	CC494631	8	1055	1055	64	62	55
1256	9	90739935	90740345	BF440376	BF440376	BF440376	8	1060	1060	64	62	55
1257	9	90616587	90616696	BZ838212	BZ838212	BZ838212	8	1065	1065	64	62	55
1258	9	89235746	89235911	CC506176	CC506176	CC506176	8	1070	1070	64	62	55
1259	9	89225642	89225834	AW437778	AW437778	AW437778	8	1075	1075	64	62	55
1260	9	88322542	88322998	AW312157	AW312157	AW312157	8	1081	1081	64	62	55
1261	9	101033739	101033979	CC582087	CC582087	CC582087	8	1086	1086	65	63	56
1262	9	101814583	101814949	BZ849642	BZ849642	BZ849642	8	1101	1101	65	63	56
1263	9	102906413	102906661	CC582072	CC582072	CC582072	8	1105	1105	65	63	56
1264	9	103881927	103882139	BZ834044	BZ834044	BZ834044	8	1109	1109	65	63	56
1265	9	104839849	104840165	CC503301	CC503301	CC503301	8	1126	1126	65	63	56
1266	9	105871175	105871463	CC479606	CC479606	CC479606	8	1126	1126	65	63	56
1267	9	106961168	106961519	CC466413	CC466413	CC466413	8	1136	1136	65	63	56
1268	9	107959873	107960171	BZ925180	BZ925180	BZ925180	8	1136	1136	65	63	56
1269	9	109024591	109024752	BZ850979	BZ850979	BZ850979	8	1141	1141	65	63	56
1270	9	110097237	110097540	CC560010	CC560010	CC560010	8	1151	1151	65	63	56
1271	9	111216611	111217066	BZ948507	BZ948507	BZ948507	8	1161	1161	65	63	56
1272	9	112408620	112408732	BZ920953	BZ920953	BZ920953	8	1161	1161	65	63	56
1273	9	113743149	113743320	BZ834490	BZ834490	BZ834490	8	1165	1165	65	63	56
1274	9	114174901	114175071	BE217544	BE217544	BE217544	8	1169	1169	65	63	56
1275	9	114178036	114178152	BF440396	BF440396	BF440396	8	1169	1169	65	63	56
1276	9	114865777	114865914	CC540398	CC540398	CC540398	8	1174	1174	65	63	56
1277	9	115814851	115815203	CC521665	CC521665	CC521665	8	1184	1184	65	63	56
1278	9	117843311	117843651	CC522350	CC522350	CC522350	8	1188	1188	65	63	56
1280	9	116887493	116887743	BZ921782	BZ921782	BZ921782	8	1197	1197	65	63	56
1281	9	118068126	118068539	CC513725	CC513725	CC513725	8	1197	1197	65	63	56
1282	9	119121529	119121740	BZ849893	BZ849893	BZ849893	8	1202	1202	65	63	56
1284	9	119940284	119940514	CC466169	CC466169	CC466169	8	1207	1207	65	63	56
1285	2	53844	53998	BZ949458	BZ949458	BZ949458	8	1290	1290	66	64	57
1286	2	1438891	1439079	CC505882	CC505882	CC505882	8	1307	1307	66	64	57
1287	2	1647633	1647909	BZ934029	BZ934029	BZ934029	8	1307	1307	66	64	57
1288	6	64696973	64697201	CC551766	CC551766	CC551766	9	0	0	67	65	58
1289	6	65347477	65347625	BZ956076	BZ956076	BZ956076	9	0	0	67	65	58
1290	6	66724300	66724717	BZ838773	BZ838773	BZ838773	9	3	3	67	65	58
1291	6	68187982	68188607	CC580094	CC580094	CC580094	9	10	10	67	65	58
1292	6	68811511	68811740	CC472191	CC472191	CC472191	9	21	21	67	65	58
1293	6	70151300	70151554	BZ925750	BZ925750	BZ925750	9	37	37	67	65	58
1295	6	70935719	70936185	BZ845716	BZ845716	BZ845716	9	49	49	67	65	58
1297	6	71627017	71627463	AW289346	AW289346	AW289346	9	78	78	67	65	58
1298	6	72099237	72099765	BZ933613	BZ933613	BZ933613	9	87	87	67	65	58
1299	6	73226704	73226877	BZ881087	BZ881087	BZ881087	9	100	100	67	65	58
1300	6	74258568	74258815	CC513210	CC513210	CC513210	9	108	108	67	65	58
1301	6	75163047	75163575	BZ849625	BZ849625	BZ849625	9	112	112	67	65	58
1302	6	75850902	75851233	AW426863	AW426863	AW426863	9	112	112	67	65	58

1303	6	76442347	76442556	BZ881050	BZ881050	BZ881050	9	112	112	67	65	58			
1304	6	77463803	77464553	CC538797	CC538797	CC538797	9	125	125	67	65	58			
1305	6	78456959	78457209	BZ918452	BZ918452	BZ918452	9	125	125	67	65	58			
1307	6	79538150	79538323	BZ933591	BZ933591	BZ933591	9	142	142	67	65	58			
1308	6	80517313	80518012	CC524182	CC524182	CC524182	9	146	146	67	65	58			
1309	6	81296431	81297133	CC539484	CC539484	CC539484	9	150	150	67	65	58			
1310	6	82333309	82333556	BZ856750	BZ856750	BZ856750	9	164	164	67	65	58			
1311	6	83132928	83133356	AW484355	AW484355	AW484355	9	168	168	67	65	58			
1312	6	83322532	83322603	CC499749	CC499749	CC499749	9	168	168	67	65	58			
1313	6	84348739	84349228	CC558654	CC558654	CC558654	9	176	176	67	65	58			
1314	6	127065287	127065418	BZ856163	BZ856163	BZ856163	9	187	187	68	66	59			
1315	6	126031203	126031712	BZ929443	BZ929443	BZ929443	9	190	190	68	66	59			
1316	6	125154173	125154437	BZ830834	BZ830834	BZ830834	9	190	190	68	66	59			
1318	6	124039983	124040256	CC509374	CC509374	CC509374	9	194	194	68	66	59			
1319	6	123037847	123038260	CC562966	CC562966	CC562966	9	194	194	68	66	59			
1320	6	122016280	122016567	CC771351	CC771351	CC771351	9	197	197	68	66	59			
1321	6	121809683	121810841	J05535	J05535	J05535	9	209	209	68	66	59			
1322	6	121304915	121305068	CC557426	CC557426	CC557426	9	221	221	68	66	59			
1323	6	120080318	120080623	CC773683	CC773683	CC773683	9	229	229	68	66	59			
1324	6	118986962	118987361	AW358402	AW358402	AW358402	9	237	237	68	66	59			
1325	6	117988706	117989276	AW289419	AW289419	AW289419	9	237	237	68	66	59			
1326	6	117830891	117831175	BZ840423	BZ840423	BZ840423	9	245	245	68	66	59			
1327	6	116851920	116852385	CC546536	CC546536	CC546536	9	245	245	68	66	59			
1328	6	115779723	115779798	BZ904208	BZ904208	BZ904208	9	257	257	68	66	59			
1329	6	114588255	114588523	CC772710	CC772710	CC772710	9	264	264	68	66	59			
1330	6	113663602	113663687	CC531121	CC531121	CC531121	9	271	271	68	66	59			
1331	6	112586006	112586135	BZ857855	BZ857855	BZ857855	9	283	283	68	66	59			
1332	6	111844321	111844396	CC502828	CC502828	CC502828	9	302	302	68	66	59			
1333	6	110870056	110870435	BZ857193	BZ857193	BZ857193	9	307	307	68	66	59			
1334	6	109111151	109111312	AW289271	AW289271	AW289271	9	316	316	68	66	59			
1335	6	109092191	109092589	AW426785	AW426785	AW426785	9	316	316	68	66	59			
1336	6	108687784	108687956	CC552571	CC552571	CC552571	9	316	316	68	66	59			
1337	6	109734219	109734448	CC529928	CC529928	CC529928	9	320	320	68	66	59			
1338	6	107887129	107887570	CC591721	CC591721	CC591721	9	320	320	68	66	59			
1339	6	106756475	106756737	CC500336	CC500336	CC500336	9	325	325	68	66	59			
1340	6	105832504	105832995	AW462014	AW462014	AW462014	9	325	325	68	66	59			
1341	6	105732672	105733104	BZ917982	BZ917982	BZ917982	9	325	325	68	66	59			
1342	6	104536543	104537215	BZ852995	BZ852995	BZ852995	9	330	330	68	66	59			
1343	6	104194408	104194780	BZ931344	BZ931344	BZ931344	9	348	348	68	66	59			
1344	6	103472807	103473143	BZ923661	BZ923661	BZ923661	9	353	353	68	66	59			
1346	6	102413700	102414014	BZ846068	BZ846068	BZ846068	9	353	353	68	66	59			
1347	6	101418876	101419247	BZ907668	BZ907668	BZ907668	9	363	363	68	66	59			
1348	6	100308293	100308994	BZ887919	BZ887919	BZ887919	9	368	368	68	66	59			
1349	6	99955259	99955451	AW465187	AW465187	AW465187	9	372	372	68	66	59			
1350	6	99382888	99383297	CC591465	CC591465	CC591465	9	382	382	68	66	59			
1351	6	98237804	98238252	BZ878734	BZ878734	BZ878734	9	382	382	68	66	59			
1352	6	97154427	97154632	CC563277	CC563277	CC563277	9	382	382	68	66	59			
1353	6	96262444	96262639	BZ834621	BZ834621	BZ834621	9	394	394	68	66	59			
1354	6	95266623	95266774	BZ863210	BZ863210	BZ863210	9	400	400	68	66	59			
1355	6	94505235	94505483	BZ836309	BZ836309	BZ836309	9	400	400	68	66	59			
1356	6	94177404	94177610	AW658548	AW658548	AW658548	9	407	407	68	66	59			
1357	6	93405055	93405382	CC594948	CC594948	CC594948	9	414	414	68	66	59			
1358	6	92181992	92182158	BZ857891	BZ857891	BZ857891	9	421	421	68	66	59			
1359	6	91236437	91236877	BZ885970	BZ885970	BZ885970	9	429	429	68	66	59			
1360	6	91147772	91147881	CC552158	CC552158	CC552158	9	429	429	68	66	59			
1361	6	90101794	90102060	BZ868653	BZ868653	BZ868653	9	429	429	68	66	59			
1362	6	89729783	89730030	AW485279	AW485279	AW485279	9	429	429	68	66	59			
1363	6	89240448	89240951	CC592225	CC592225	CC592225	9	436	436	68	66	59			
1364	6	88193367	88193520	CC774696	CC774696	CC774696	9	458	458	68	66	59			
1365	6	87103230	87103456	CC528617	CC528617	CC528617	9	463	463	68	66	59			
1366	6	86087252	86087727	CC581285	CC581285	CC581285	9	463	463	68	66	59			
1367	6	85225230	85225580	CC591397	CC591397	CC591397	9	474	474	68	66	59			
1368	6	86410499	86410959	AW461357	AW461357	AW461357	9	480	480	68	66	59			
1369	6	128331367	128331851	CC771581	CC771581	CC771581	9	480	480	69	67	deleted			
1370	6	129351496	129351722	CC508891	CC508891	CC508891	9	493	493	69	67	60			
1371	6	130547349	130547537	CC543650	CC543650	CC543650	9	493	493	69	67	60			
1372	6	131645772	131646085	AW315844	AW315844	AW315844	9	499	499	69	67	60			
1373	6	132311010	132311626	AW267007	AW267007	AW267007	9	505	505	69	67	60			
1374	6	132900482	132900836	CC535603	CC535603	CC535603	9	516	516	69	67	60			
1376	6	134004075	134004342	CC553217	CC553217	CC553217	9	528	528	69	67	60			
1377	6	135102794	135103075	CC765338	CC765338	CC765338	9	535	535	69	67	60			
1378	6	136150443	136150760	CC550473	CC550473	CC550473	9	540	540	69	67	60			
1379	6	137183527	137183784	CC563446	CC563446	CC563446	9	540	540	69	67	60			
1380	6	138242037	138242204	BZ921317	BZ921317	BZ921317	9	546	546	69	67	60			
1381	6	139265073	139265647	CC595376	CC595376	CC595376	9	552	552	69	67	60			
1382	6	140323681	140324097	BZ843235	BZ843235	BZ843235	9	552	552	69	67	60			
1383	6	141615721	141616274	CC511324	CC511324	CC511324	9	552	552	69	67	60			
1384	6	142689649	142689994	BZ927478	BZ927478	BZ927478	9	557	557	69	67	60			
1385	6	143753545	143753772	BZ889129	BZ889129	BZ889129	9	557	557	69	67	60			
1386	6	144853638	144853908	BZ903329	BZ903329	BZ903329	9	564	564	69	67	60			
1387	6	145711939	145712467	CC506551	CC506551	CC506551	9	564	564	69	67	60			
1388	6	146717676	146717849	CC580737	CC580737	CC580737	9	564	564	69	67	60			
1389	6	147907565	147907894	BZ874112	BZ874112	BZ874112	9	583	583	69	67	60			
1390	6	148903412	148903952	CC529991	CC529991	CC529991	9	589	589	69	67	60			
1391	6	149964200	149964646	CC763649	CC763649	CC763649	9	595	595	69	67	60			
1392	6	150939339	150939468	CC511533	CC511533	CC511533	9	595	595	69	67	60			
1393	6	151236887	151236980	CC594335	CC594335	CC594335	9	595	595	69	67	60			
1394	6	152222945	152223759	CC517949	CC517949	CC517949	9	595	595	69	67	60			
1395	6	153170284	153170663	BZ921721	BZ921721	BZ921721	9	601	601	69	67	60			
1396	6	154253169	154253694	CC514658	CC514658	CC514658	9	601	601	69	67	60			
1399	6	154504199	154504721	U89677	U89677	U89677	9	606	606	69	67	60			
1400	6	155218290	155218759	CC554454	CC554454	CC554454	9	611	611	69	67	60			
1401	6	156234396	156234564	CC506601	CC506601	CC506601	9	616	616	69	67	60			
1402	6	157143310	157143466	BZ860162	BZ860162	BZ860162	9	626	626	69	67	60			
1404	6	157683071	157683145	AW289234	AW289234	AW289234	9	647	647	69	67	60			
1405	6	158074257	158074667	BZ872544	BZ872544	BZ872544	9	647	647	69	67	60			
1407	6	159372108	159372742	CC518770	CC518770	CC518770	9	668	668	69	67	60			
1408	6	160070570	160070834	BE217584	BE217584	BE217584	9	668	668	69	67	60			
1409	6	159157571	159157776	AW266923	AW266923	AW266923	9	674	674	69	67	60			
1410	6	160464650	160464915	J03527	J03527	J03527	9	692	692	69	67	60			
1411	6	160844632	160845031	X79402	X79402	X79402	9	692	692	69	67	60			
1412	6	161544782	161544891	CC487082	CC487082	CC487082	9	698	698	69	67	60			
1413	6	162635700	162636033	BZ939712	BZ939712	BZ939712	9	698	698	69	67	60			
1414	6	163706616	163706922	CC502567	CC502567	CC502567	9	708	708	69	67	60			
1415	6	166914957	166915014	CC490268	CC490268	CC490268	9	719	719	69	67	60			
1416	6	165000837	165001400	CC487875	CC487875	CC487875	9	724	724	69	67	60			
1417	6	165287699	165288052	BZ888776	BZ888776	BZ888776	9	729	729	69	67	60			
1418	6	165471500	165471774	BZ852633	BZ852633	BZ852633	9	729	729	69	67	60			
1419	6	168250923	168250992	CC494155	CC494155	CC494155	9	746	746	69	67	60			
1420	6	168948192	168948437	BZ905058	BZ905058	BZ905058	9	752	752	69	67	60			
1421	6	170513611	170513839	CC585295	CC585295	CC585295	9	764	764	69	67	60			
1422	5	111000432	111000638	BZ952666	BZ952666	BZ952666	10	0	0	70	68	61			
1423	5	112065576	112065802	BZ940920	BZ940920	BZ940920	10	9	9	70	68	61			
1424	5	113104542	113104805	BZ852351	BZ852351	BZ852351	10	27	27	70	68	61			
1425	5	114159239	114159666	BZ881580	BZ881580	BZ881580	10	40	40	70	68	61		AG is wrong in merging these two blocks, as other blocks comes in between	2 -> 1
1426	5	174392440	174392648	BZ873338	BZ873338	BZ873338	10	43	43	71	69	61	AG here		
1427	5	175673379	175673488	CC501706	CC501706	CC501706	10	48	48	71	69	61			
1428	5	75026261	75026643	CC763937	CC763937	CC763937	10	57	57	72	70	62			
1429	5	75990118	75990298	BZ907510	BZ907510	BZ907510	10	63	63	72	70	62			
1430	5	76958074	76958459	BZ903104	BZ903104	BZ903104	10	71	71	72	70	62			
1432	5	78300477	78300895	AW660499	AW660499	AW660499	10	95	95	72	70	62			
1433	5	79076693	79076888	CC592472	CC592472	CC592472	10	95	95	72	70	62			
1434	15	63733225	63733563	AF025664	AF025664	AF025664	10	131	131	73	71	63			
1435	15	64594833	64595082	CC501260	CC501260	CC501260	10	138	138	73	71	63			
1436	15	65656995	65657074	BZ890063	BZ890063	BZ890063	10	152	152	73	71	63			
1437	15	66246620	66246988	BF440257	BF440257	BF440257	10	155	155	73	71	63			
1438	15	66517007	66517146	CC571662	CC571662	CC571662	10	155	155	73	71	63			
1440	15	67347595	67349805	AF003927	AF003927	AF003927	10	176	176	73	71	63			
1441	15	67561181	67561280	CC587196	CC587196	CC587196	10	179	179	73	71	63			
1442	15	69822833	69823396	CC595437	CC595437	CC595437	10	207	207	73	71	63			
1443	15	70890653	70891015	CC478523	CC478523	CC478523	10	211	211	73	71	63			
1444	15	71828222	71828517	CC539192	CC539192	CC539192	10	211	211	73	71	deleted			
1445	14	24715598	24715662	CC516641	CC516641	CC516641	10	214	214	74	72	64			
1446	14	23969893	23969991	AW461579	AW461579	AW461579	10	214	214	74	72	64			
1447	14	23852035	23852265	BZ913836	BZ913836	BZ913836	10	214	214	74	72	64			
1448	14	23009719	23010173	CC766403	CC766403	CC766403	10	218	218	74	72	64			
1449	14	22664568	22664641	AW462183	AW462183	AW462183	10	218	218	74	72	64			
1450	14	22485517	22485764	AW430545	AW430545	AW430545	10	218	218	74	72	64			
1451	14	22381458	22381770	AB010921	AB010921	AB010921	10	218	218	74	72	64			
1452	14	22013819	22013873	BZ866362	BZ866362	BZ866362	10	225	225	74	72	64			
1453	14	20889501	20889810	AW487190	AW487190	AW487190	10	237	237	74	72	64			
1454	14	20880944	20881267	BZ845692	BZ845692	BZ845692	10	237	237	74	72	64			
1455	14	20612915	20613179	AW426554	AW426554	AW426554	10	240	240	74	72	64			
1456	14	20237352	20237804	AW655683	AW655683	AW655683	10	240	240	74	72	64			
1457	14	19985897	19986006	AW657300	AW657300	AW657300	10	243	243	74	72	64			
1458	14	19914152	19914566	BZ885761	BZ885761	BZ885761	10	254	254	74	72	64			
1459	15	31116941	31117199	CC561705	CC561705	CC561705	10	273	273	75	73	65			
1460	15	32169541	32169858	CC530576	CC530576	CC530576	10	281	281	75	73	65			
1461	15	33205165	33205599	BZ857088	BZ857088	BZ857088	10	292	292	75	73	65			
1462	15	34170952	34171288	BZ935638	BZ935638	BZ935638	10	303	303	75	73	65			
1463	15	35201038	35201437	CC515022	CC515022	CC515022	10	306	306	75	73	65			
1464	15	36015602	36016240	CC551338	CC551338	CC551338	10	306	306	75	73	65			
1465	15	37118968	37119272	BZ954976	BZ954976	BZ954976	10	306	306	75	73	65			
1467	15	39299276	39299492	CC528058	CC528058	CC528058	10	321	321	75	73	65			
1468	15	38500116	38500642	AW289409	AW289409	AW289409	10	325	325	75	73	65			
1469	15	40624466	40624846	X73745	X73745	X73745	10	332	332	75	73	65			
1470	15	41281215	41281616	BZ863117	BZ863117	BZ863117	10	332	332	75	73	65			
1471	15	40266710	40266881	CC775880	CC775880	CC775880	10	336	336	75	73	65			
1472	14	45668881	45669207	CC568659	CC568659	CC568659	10	340	340	76	74	66			
1473	14	46670014	46670335	CC547713	CC547713	CC547713	10	343	343	76	74	66			
1474	14	47789375	47789627	BZ935902	BZ935902	BZ935902	10	347	347	76	74	66			
1475	14	48812019	48812190	BZ836705	BZ836705	BZ836705	10	355	355	76	74	66			
1476	14	49769432	49769622	CC553437	CC553437	CC553437	10	355	355	76	74	66			
1477	15	62151831	62152222	AW314155	AW314155	AW314155	10	362	362	77	75	67			
1478	15	62235189	62235398	AW266943	AW266943	AW266943	10	369	369	77	75	67			
1479	15	62330598	62330788	BZ944935	BZ944935	BZ944935	10	369	369	77	75	67			
1481	15	62767098	62767227	AW312570	AW312570	AW312570	10	379	379	77	75	67		This singleton prevented merging of two blocks	1 -> 2
1482	14	50724637	50724982	BZ840807	BZ840807	BZ840807	10	389	389	76	74	singleton	AG here		
1483	15	61839465	61839625	AW289301	AW289301	AW289301	10	401	401	77	75	68			
1484	15	61346540	61346945	AW428595	AW428595	AW428595	10	401	401	77	75	68			
1485	15	61339947	61340326	CC772991	CC772991	CC772991	10	401	401	77	75	68			
1486	15	61150743	61150953	AW289408	AW289408	AW289408	10	401	401	77	75	68			
1487	15	60112909	60113084	BZ848925	BZ848925	BZ848925	10	404	404	77	75	68			
1488	15	59200861	59201028	CC480901	CC480901	CC480901	10	404	404	77	75	68			
1489	15	58440431	58440549	M14056	M14056	M14056	10	408	408	77	75	68			
1490	15	58161604	58161872	CC514892	CC514892	CC514892	10	415	415	77	75	68			
1491	15	56931150	56931415	BZ955635	BZ955635	BZ955635	10	415	415	77	75	68			
1492	15	56689787	56690010	Z21961	Z21961	Z21961	10	415	415	77	75	68			
1493	15	55810965	55811273	CC501894	CC501894	CC501894	10	422	422	77	75	68			
1494	15	54780357	54780461	BZ948099	BZ948099	BZ948099	10	430	430	77	75	68			
1495	15	53720474	53720827	CC588344	CC588344	CC588344	10	444	444	77	75	68			
1496	15	52605063	52605203	BZ884734	BZ884734	BZ884734	10	444	444	77	75	68			
1497	15	51790796	51791088	BZ908314	BZ908314	BZ908314	10	447	447	77	75	68			
1498	15	50757473	50757583	CC764923	CC764923	CC764923	10	453	453	77	75	68			
1499	15	49766953	49767059	BZ865852	BZ865852	BZ865852	10	453	453	77	75	68			
1500	15	49565757	49565827	CC570059	CC570059	CC570059	10	456	456	77	75	68			
1501	15	48620431	48620796	CC523061	CC523061	CC523061	10	460	460	77	75	68			
1502	15	48425732	48426008	CC570677	CC570677	CC570677	10	463	463	77	75	68			
1503	15	47394851	47395263	BZ911871	BZ911871	BZ911871	10	469	469	77	75	68			
1504	15	46958961	46959294	AW267025	AW267025	AW267025	10	469	469	77	75	68			
1505	15	46265668	46266095	CC494715	CC494715	CC494715	10	472	472	77	75	68			
1506	15	45289344	45289606	CC553489	CC553489	CC553489	10	472	472	77	75	68			
1507	15	44307068	44307160	CC475410	CC475410	CC475410	10	472	472	77	75	68			
1508	15	43202212	43202515	BZ940928	BZ940928	BZ940928	10	479	479	77	75	68			
1509	14	52393776	52394080	AW266985	AW266985	AW266985	10	494	494	78	76	69			
1510	14	52397592	52397843	AW267158	AW267158	AW267158	10	500	500	78	76	69			
1511	14	52582245	52583432	AF045022	AF045022	AF045022	10	503	503	78	76	69			
1512	14	52639232	52639476	BZ900511	BZ900511	BZ900511	10	503	503	78	76	69			
1513	14	53657793	53658061	BZ839564	BZ839564	BZ839564	10	512	512	78	76	69			
1514	14	54713560	54713991	CC591389	CC591389	CC591389	10	515	515	78	76	69			
1515	14	55718040	55718244	CC579683	CC579683	CC579683	10	525	525	78	76	69			
1516	14	56755222	56755467	CC570810	CC570810	CC570810	10	528	528	78	76	69			
1517	14	56743336	56743654	AW289333	AW289333	AW289333	10	531	531	78	76	69			
1518	14	57806843	57807027	CC490977	CC490977	CC490977	10	537	537	78	76	69			
1519	14	57897218	57897606	AW289426	AW289426	AW289426	10	540	540	78	76	69			
1520	14	58829325	58829963	CC767108	CC767108	CC767108	10	550	550	78	76	69			
1521	14	59654938	59655648	CC552650	CC552650	CC552650	10	550	550	78	76	69			
1522	14	60617180	60617675	CC511458	CC511458	CC511458	10	554	554	78	76	69			
1523	14	61284090	61284729	AW267005	AW267005	AW267005	10	557	557	78	76	69			
1524	14	61661460	61661727	BZ881718	BZ881718	BZ881718	10	557	557	78	76	69			
1525	14	62422964	62423332	BZ911263	BZ911263	BZ911263	10	570	570	78	76	69			
1526	14	62907837	62908451	AW428583	AW428583	AW428583	10	577	577	78	76	69			
1527	14	63437714	63437779	BZ925968	BZ925968	BZ925968	10	577	577	78	76	69			
1528	14	64414303	64414625	CC482722	CC482722	CC482722	10	583	583	78	76	69			
1529	14	65383140	65383452	BZ831073	BZ831073	BZ831073	10	586	586	78	76	69			
1530	14	66626573	66626746	BZ918046	BZ918046	BZ918046	10	592	592	78	76	69			
1531	14	67675582	67676501	CC479519	CC479519	CC479519	10	592	592	78	76	69			
1532	14	68605674	68605720	CC485610	CC485610	CC485610	10	595	595	78	76	69			
1533	14	69709827	69709979	BZ866777	BZ866777	BZ866777	10	601	601	78	76	69			
1534	14	70777563	70778192	BZ906429	BZ906429	BZ906429	10	604	604	78	76	69			
1535	14	72076808	72076907	AW266901	AW266901	AW266901	10	604	604	78	76	69			
1536	14	72657676	72657829	AW289321	AW289321	AW289321	10	621	621	78	76	69			
1537	14	72707441	72707531	CC590639	CC590639	CC590639	10	621	621	78	76	69			
1538	14	73690914	73691159	CC763200	CC763200	CC763200	10	625	625	78	76	69			
1539	14	74036598	74037521	U35363	U35363	U35363	10	628	628	78	76	69			
1540	14	74817238	74817608	AF069515	AF069515	AF069515	10	628	628	78	76	69			
1541	14	75721228	75721547	BZ921204	BZ921204	BZ921204	10	632	632	78	76	69			
1542	14	76717621	76718078	CC543890	CC543890	CC543890	10	632	632	78	76	69			
1543	14	78021005	78021138	BZ897076	BZ897076	BZ897076	10	635	635	78	76	69			
1544	14	79032492	79032848	CC468420	CC468420	CC468420	10	639	639	78	76	69			
1545	14	79397135	79398404	L27869	L27869	L27869	10	642	642	78	76	69			
1546	14	79919340	79919536	BZ874065	BZ874065	BZ874065	10	642	642	78	76	69			
1547	14	81009028	81009449	AF013068	AF013068	AF013068	10	642	642	78	76	69			
1548	14	82167743	82167991	BZ891756	BZ891756	BZ891756	10	646	646	78	76	69			
1549	14	83449600	83450038	BZ951336	BZ951336	BZ951336	10	649	649	78	76	69			
1550	14	84522021	84522199	BZ952973	BZ952973	BZ952973	10	649	649	78	76	69			
1551	14	85161715	85161981	AW652828	AW652828	AW652828	10	656	656	78	76	69			
1552	14	85740861	85741140	CC486025	CC486025	CC486025	10	663	663	78	76	69			
1553	14	86697695	86698099	CC571536	CC571536	CC571536	10	666	666	78	76	69			

1555	14	87763536	87763669	BZ931998	BZ931998	BZ931998	10	681	681	78	76	69
1556	14	88406097	88406210	CC524401	CC524401	CC524401	10	684	684	78	76	69
1557	14	89190844	89190943	BZ891552	BZ891552	BZ891552	10	687	687	78	76	69
1558	14	90183042	90183125	CC514173	CC514173	CC514173	10	694	694	78	76	69
1559	2	95128669	95128744	BZ953184	BZ953184	BZ953184	11	0	0	79	77	70
1560	2	95195801	95196013	AW336882	AW336882	AW336882	11	8	8	79	77	70
1561	2	96724766	96724905	BZ932543	BZ932543	BZ932543	11	8	8	79	77	70
1562	2	97173318	97173474	AW483908	AW483908	AW483908	11	11	11	79	77	70
1563	2	97788385	97788716	CC533310	CC533310	CC533310	11	17	17	79	77	70
1564	2	98325262	98325464	BZ953931	BZ953931	BZ953931	11	23	23	79	77	70
1565	2	98684735	98685022	AW481580	AW481580	AW481580	11	26	26	79	77	70
1566	2	99510847	99511094	BZ896936	BZ896936	BZ896936	11	30	30	79	77	70
1567	2	100314429	100314586	CC491027	CC491027	CC491027	11	39	39	79	77	70
1568	2	101575256	101575631	BZ908848	BZ908848	BZ908848	11	44	44	79	77	70
1569	2	101966394	101966508	AW461551	AW461551	AW461551	11	47	47	79	77	70
1570	2	102711391	102711909	BZ889374	BZ889374	BZ889374	11	50	50	79	77	70
1571	2	104116315	104116455	BZ926855	BZ926855	BZ926855	11	59	59	79	77	70
1572	2	105308720	105308985	BZ952395	BZ952395	BZ952395	11	68	68	79	77	70
1573	2	74667914	74668074	AW632130	AW632130	AW632130	11	79	79	80	78	71
1574	2	74517349	74517571	BZ942883	BZ942883	BZ942883	11	89	89	80	78	71
1576	2	74353357	74353522	AW266953	AW266953	AW266953	11	89	89	80	78	71
1577	2	74085500	74085691	AW482999	AW482999	AW482999	11	92	92	80	78	71
1578	2	73385272	73385526	BZ933507	BZ933507	BZ933507	11	103	103	80	78	71
1579	2	73212796	73213021	AW327157	AW327157	AW327157	11	108	108	80	78	71
1580	2	72629440	72629487	BZ922292	BZ922292	BZ922292	11	111	111	80	78	71
1581	2	71742067	71742916	CC473171	CC473171	CC473171	11	120	120	80	78	71
1582	2	71565792	71566190	AW487346	AW487346	AW487346	11	122	122	80	78	71
1583	2	70588114	70588720	BZ955721	BZ955721	BZ955721	11	128	128	80	78	71
1584	2	31484523	31484752	X98491	X98491	X98491	11	131	131	81	79	72
1585	2	32359528	32359753	AW358065	AW358065	AW358065	11	145	145	81	79	72
1586	2	32993691	32993984	BZ898163	BZ898163	BZ898163	11	151	151	81	79	72
1587	2	33535761	33536071	AW632471	AW632471	AW632471	11	151	151	81	79	72
1588	2	33653592	33653789	BZ943157	BZ943157	BZ943157	11	163	163	81	79	72
1589	2	34664823	34665156	BZ909811	BZ909811	BZ909811	11	178	178	81	79	72
1590	2	35981312	35981522	BZ939849	BZ939849	BZ939849	11	180	180	81	79	72
1591	2	37088906	37089000	BZ928079	BZ928079	BZ928079	11	183	183	81	79	72
1592	2	37764916	37765532	BZ920139	BZ920139	BZ920139	11	188	188	81	79	72
1593	2	38888311	38888498	AW464949	AW464949	AW464949	11	210	210	81	79	72
1594	2	39299879	39300344	BZ950076	BZ950076	BZ950076	11	213	213	81	79	72
1595	2	40594449	40594590	BZ930927	BZ930927	BZ930927	11	222	222	81	79	72
1596	2	41707615	41708080	BZ906947	BZ906947	BZ906947	11	228	228	81	79	72
1598	2	42471055	42471337	AW267084	AW267084	AW267084	11	237	237	81	79	72
1599	2	42981508	42981705	BZ916795	BZ916795	BZ916795	11	237	237	81	79	72
1600	2	43832927	43833333	BZ898519	BZ898519	BZ898519	11	243	243	81	79	72
1601	2	44371105	44372843	AJ005458	AJ005458	AJ005458	11	246	246	81	79	72
1602	2	44884012	44884256	BZ915604	BZ915604	BZ915604	11	252	252	81	79	72
1603	2	46048430	46048776	BZ937491	BZ937491	BZ937491	11	258	258	81	79	72
1604	2	46876331	46876712	BZ909550	BZ909550	BZ909550	11	261	261	81	79	72
1605	2	47584336	47584449	AW266975	AW266975	AW266975	11	271	271	81	79	72
1606	2	48826347	48827639	U20504	U20504	U20504	11	278	278	81	79	72
1607	2	47977787	47978439	BZ930065	BZ930065	BZ930065	11	282	282	81	79	72
1608	2	49188709	49189218	BZ948537	BZ948537	BZ948537	11	285	285	81	79	72
1609	2	50353732	50354005	BZ938075	BZ938075	BZ938075	11	288	288	81	79	72
1610	2	51166286	51167621	L14855	L14855	L14855	11	288	288	81	79	72
1611	2	51881457	51881574	BZ956118	BZ956118	BZ956118	11	291	291	81	79	72
1612	2	53027253	53027830	BZ942662	BZ942662	BZ942662	11	310	310	81	79	72
1613	2	54053032	54053388	BZ948266	BZ948266	BZ948266	11	315	315	81	79	72
1614	2	55390715	55390848	BZ901665	BZ901665	BZ901665	11	337	337	81	79	72
1615	2	55688519	55688795	BF440164	BF440164	BF440164	11	340	340	81	79	72
1616	2	56451158	56451354	BZ948263	BZ948263	BZ948263	11	351	351	81	79	72
1617	2	57882891	57883121	BZ952492	BZ952492	BZ952492	11	357	357	81	79	72
1618	2	58783544	58784038	BZ935016	BZ935016	BZ935016	11	369	369	81	79	72
1620	2	59830560	59831009	BZ900922	BZ900922	BZ900922	11	374	374	81	79	72
1623	2	61170237	61170921	BZ920913	BZ920913	BZ920913	11	383	383	81	79	72
1624	2	75840895	75841321	BZ925709	BZ925709	BZ925709	11	388	388	82	80	73
1625	2	75631577	75631745	AW356401	AW356401	AW356401	11	393	393	82	80	73
1626	2	106152738	106152903	AW312031	AW312031	AW312031	11	418	418	83	81	74
1627	2	106905026	106905287	CC762637	CC762637	CC762637	11	427	427	83	81	74
1628	2	108036486	108036820	CC537463	CC537463	CC537463	11	432	432	83	81	74
1630	2	111129509	111129899	BZ947794	BZ947794	BZ947794	11	451	451	83	81	74
1631	2	111642053	111642222	AW446001	AW446001	AW446001	11	453	453	83	81	74
1632	2	113126320	113126769	BZ915916	BZ915916	BZ915916	11	462	462	83	81	74
1633	2	113672545	113672698	AW428019	AW428019	AW428019	11	479	479	83	81	74
1634	2	89000597	89001004	CC475816	CC475816	CC475816	11	497	497	84	82	75
1635	2	88189506	88189705	BZ924229	BZ924229	BZ924229	11	502	502	84	82	75
1636	2	86916288	86916814	AW289381	AW289381	AW289381	11	507	507	84	82	75
1637	2	86628026	86628518	BZ939600	BZ939600	BZ939600	11	507	507	84	82	75
1638	2	85932006	85932186	CC472289	CC472289	CC472289	11	510	510	84	82	75
1639	2	85787686	85787969	AW289187	AW289187	AW289187	11	513	513	84	82	75
1640	2	85697219	85697387	M81593	M81593	M81593	11	513	513	84	82	75
1641	2	84732672	84732786	BZ924161	BZ924161	BZ924161	11	513	513	84	82	75
1642	2	83570974	83571064	BZ903772	BZ903772	BZ903772	11	518	518	84	82	75
1643	2	82298773	82298861	BZ907565	BZ907565	BZ907565	11	526	526	84	82	75
1644	2	81201982	81202232	BZ951026	BZ951026	BZ951026	11	532	532	84	82	75
1645	2	80286427	80287003	CC514824	CC514824	CC514824	11	549	549	84	82	75
1646	2	79463908	79464132	BZ943098	BZ943098	BZ943098	11	554	554	84	82	75
1647	2	78001061	78001377	BZ943635	BZ943635	BZ943635	11	568	568	84	82	75
1648	2	76897250	76897405	BZ950712	BZ950712	BZ950712	11	570	570	84	82	75
1649	2	61325857	61326076	AW656448	AW656448	AW656448	11	595	595	85	83	76
1650	2	62695635	62696066	BZ955708	BZ955708	BZ955708	11	611	611	85	83	76
1651	2	63743479	63743650	AW479517	AW479517	AW479517	11	621	621	85	83	76
1652	2	63808445	63808566	BZ906320	BZ906320	BZ906320	11	626	626	85	83	76
1653	2	64706245	64706586	BZ921785	BZ921785	BZ921785	11	629	629	85	83	76
1654	2	65880510	65880753	BZ952530	BZ952530	BZ952530	11	637	637	85	83	76
1655	2	66963790	66964146	BZ915155	BZ915155	BZ915155	11	640	640	85	83	76
1656	2	67548812	67549122	AW346055	AW346055	AW346055	11	642	642	85	83	76
1657	2	67984336	67984571	BZ949272	BZ949272	BZ949272	11	656	656	85	83	76
1658	2	69206030	69206525	BZ940166	BZ940166	BZ940166	11	662	662	85	83	76
1659	2	69964172	69965012	M22248	M22248	M22248	11	683	683	85	83	76
1660	2	30546852	30547178	BZ852621	BZ852621	BZ852621	11	697	697	86	84	77
1661	2	29690932	29691353	BZ954944	BZ954944	BZ954944	11	708	708	86	84	77
1662	2	29082246	29082377	AW289382	AW289382	AW289382	11	711	711	86	84	77
1663	2	28935227	28935545	AW266973	AW266973	AW266973	11	714	714	86	84	77
1664	2	28544908	28545168	BZ932217	BZ932217	BZ932217	11	717	717	86	84	77
1665	2	27761988	27762215	BE217471	BE217471	BE217471	11	720	720	86	84	77
1666	2	27501197	27501546	BZ922126	BZ922126	BZ922126	11	720	720	86	84	77
1667	2	26271050	26271712	AW289307	AW289307	AW289307	11	732	732	86	84	77
1668	2	25367522	25367765	AW289242	AW289242	AW289242	11	738	738	86	84	77
1669	2	24488699	24488830	BZ939451	BZ939451	BZ939451	11	745	745	86	84	77
1671	2	23736760	23737025	CC574061	CC574061	CC574061	11	745	745	86	84	77
1672	2	22783464	22783916	BZ951925	BZ951925	BZ951925	11	745	745	86	84	77
1673	2	21870972	21871643	BZ952996	BZ952996	BZ952996	11	759	759	86	84	77
1674	2	20790685	20790929	CC526911	CC526911	CC526911	11	765	765	86	84	77
1675	2	20638401	20638493	BZ940170	BZ940170	BZ940170	11	765	765	86	84	77
1676	2	20371179	20371732	AW289319	AW289319	AW289319	11	775	775	86	84	77
1677	2	20035632	20035688	CC499962	CC499962	CC499962	11	778	778	86	84	77
1678	2	18903046	18903493	BZ926427	BZ926427	BZ926427	11	778	778	86	84	77
1679	2	17490251	17490384	BZ935336	BZ935336	BZ935336	11	788	788	86	84	77
1680	2	16565609	16566177	CC538667	CC538667	CC538667	11	794	794	86	84	77
1681	2	15505745	15506522	CC541176	CC541176	CC541176	11	794	794	86	84	77
1683	2	14485149	14485291	BZ912105	BZ912105	BZ912105	11	797	797	86	84	77
1684	2	13805174	13805709	BZ899107	BZ899107	BZ899107	11	804	804	86	84	77
1685	2	12751071	12751511	BZ842576	BZ842576	BZ842576	11	814	814	86	84	77
1686	2	11502294	11502881	CC533994	CC533994	CC533994	11	814	814	86	84	77
1687	2	11291518	11291771	BZ933204	BZ933204	BZ933204	11	814	814	86	84	77
1688	2	10532239	10532449	M92441	M92441	M92441	11	814	814	86	84	77
1689	2	10333051	10333120	BZ935417	BZ935417	BZ935417	11	821	821	86	84	77
1690	2	10219955	10220144	AW289345	AW289345	AW289345	11	821	821	86	84	77
1691	2	9081425	9081599	BZ945284	BZ945284	BZ945284	11	824	824	86	84	77
1692	2	7599969	7600127	BZ899955	BZ899955	BZ899955	11	832	832	86	84	77
1693	2	6514515	6514588	BZ897195	BZ897195	BZ897195	11	839	839	86	84	77
1694	2	5354185	5354412	BZ910238	BZ910238	BZ910238	11	839	839	86	84	77
1695	2	4237801	4238158	BZ923032	BZ923032	BZ923032	11	839	839	86	84	77
1696	9	121626948	121627361	AW314025	AW314025	AW314025	11	842	842	87	85	78
1697	9	121968765	121968823	BZ870761	BZ870761	BZ870761	11	845	845	87	85	78
1698	9	123077388	123078060	CC560438	CC560438	CC560438	11	852	852	87	85	78
1700	9	124195427	124195481	CC506235	CC506235	CC506235	11	859	859	87	85	78
1701	9	125002608	125002943	CC492656	CC492656	CC492656	11	865	865	87	85	78
1702	9	126061637	126061934	CC524465	CC524465	CC524465	11	865	865	87	85	78
1703	9	127517635	127517776	L26088	L26088	L26088	11	879	879	87	85	78
1704	9	127052186	127052619	BZ864240	BZ864240	BZ864240	11	883	883	87	85	78
1705	9	128206582	128206730	CC580620	CC580620	CC580620	11	883	883	87	85	78
1706	9	128848811	128848925	AW266944	AW266944	AW266944	11	890	890	87	85	78
1707	9	129095010	129095344	CC482120	CC482120	CC482120	11	901	901	87	85	78
1708	9	130263297	130263411	CC544505	CC544505	CC544505	11	909	909	87	85	78
1709	9	131307896	131308131	BZ877689	BZ877689	BZ877689	11	916	916	87	85	78
1710	9	131427967	131428047	AW261140	AW261140	AW261140	11	916	916	87	85	78
1711	9	132409742	132410078	BZ883828	BZ883828	BZ883828	11	919	919	87	85	78
1712	9	132969979	132970180	M28402	M28402	M28402	11	927	927	87	85	78
1713	9	133370055	133370313	CC531293	CC531293	CC531293	11	930	930	87	85	78
1715	9	134348051	134348197	CC572020	CC572020	CC572020	11	946	946	87	85	78
1717	9	135207932	135208143	CC535290	CC535290	CC535290	11	950	950	87	85	78
1718	9	136322196	136322376	CC526826	CC526826	CC526826	11	950	950	87	85	78
1719	9	137374624	137374680	AW464717	AW464717	AW464717	11	950	950	87	85	78
1720	9	137784325	137784503	AW298829	AW298829	AW298829	11	957	957	87	85	78
1721	9	137932983	137933105	CC565991	CC565991	CC565991	11	961	961	87	85	78
1722	13	60409894	60409972	CC550734	CC550734	CC550734	12	0	0	88	86	79
1723	13	59488020	59488346	CC485194	CC485194	CC485194	12	4	4	88	86	79
1724	13	58616838	58617254	BZ833200	BZ833200	BZ833200	12	7	7	88	86	79
1726	13	57729537	57729827	BZ935113	BZ935113	BZ935113	12	16	16	88	86	79
1727	13	57186219	57186426	BZ910386	BZ910386	BZ910386	12	20	20	88	86	79
1728	13	56770222	56770426	BZ915232	BZ915232	BZ915232	12	29	29	88	86	79
1729	13	55801688	55802110	CC523805	CC523805	CC523805	12	39	39	88	86	79
1730	13	55369659	55369949	BZ903177	BZ903177	BZ903177	12	39	39	88	86	79
1731	13	54727559	54727788	BZ898611	BZ898611	BZ898611	12	54	54	88	86	79
1732	13	53809259	53809401	BZ836780	BZ836780	BZ836780	12	79	79	88	86	79
1733	13	52613500	52613825	CC762543	CC762543	CC762543	12	87	87	88	86	79
1734	13	52175674	52175951	M65081	M65081	M65081	12	91	91	88	86	79
1735	13	51642965	51643390	AW631701	AW631701	AW631701	12	95	95	88	86	79
1736	13	40029030	40029534	AW621146	AW621146	AW621146	12	95	95	89	87	80
1737	13	40404197	40404824	AW428606	AW428606	AW428606	12	95	95	89	87	80
1738	13	40415956	40416131	BZ878682	BZ878682	BZ878682	12	103	103	89	87	80
1739	13	41384098	41384368	CC479819	CC479819	CC479819	12	111	111	89	87	80
1740	13	43287220	43287642	BZ900603	BZ900603	BZ900603	12	122	122	89	87	80
1741	13	44275177	44275766	CC566688	CC566688	CC566688	12	122	122	89	87	80
1742	13	42332117	42332405	CC495228	CC495228	CC495228	12	126	126	89	87	80
1743	13	45268963	45269352	CC497831	CC497831	CC497831	12	143	143	89	87	80
1744	13	46495736	46495996	BZ932308	BZ932308	BZ932308	12	143	143	89	87	80
1745	13	47269394	47269482	BZ880993	BZ880993	BZ880993	12	151	151	89	87	80
1746	13	48314810	48314937	BZ881375	BZ881375	BZ881375	12	163	163	89	87	80
1747	13	49403116	49403308	BZ854992	BZ854992	BZ854992	12	166	166	89	87	80
1748	13	50704212	50704639	CC471853	CC471853	CC471853	12	170	170	89	87	80
1749	13	51238672	51238744	AW486050	AW486050	AW486050	12	170	170	89	87	80
1750	13	51624690	51624873	CC508552	CC508552	CC508552	12	173	173	89	87	80
1751	13	39195391	39195610	CC549412	CC549412	CC549412	12	188	188	90	88	81
1752	13	38275833	38276145	BZ882244	BZ882244	BZ882244	12	191	191	90	88	81
1753	13	37218041	37218594	X99792	X99792	X99792	12	194	194	90	88	81
1754	13	36235939	36236170	BZ834381	BZ834381	BZ834381	12	198	198	90	88	81
1755	13	35397338	35397808	BZ878619	BZ878619	BZ878619	12	201	201	90	88	81
1756	13	34378920	34379199	BZ837338	BZ837338	BZ837338	12	204	204	90	88	81
1757	13	33391295	33391641	CC570070	CC570070	CC570070	12	210	210	90	88	81
1758	13	32171904	32172019	CC509562	CC509562	CC509562	12	217	217	90	88	81
1759	13	31077507	31078224	CC550076	CC550076	CC550076	12	234	234	90	88	81
1760	13	29961078	29961237	CC508834	CC508834	CC508834	12	237	237	90	88	81
1761	13	29237380	29237675	AW289323	AW289323	AW289323	12	237	237	90	88	81
1762	13	28002149	28002400	CC594078	CC594078	CC594078	12	237	237	90	88	81
1763	13	27817575	27817723	AW289364	AW289364	AW289364	12	240	240	90	88	81
1764	13	27095143	27095276	BZ887421	BZ887421	BZ887421	12	247	247	90	88	81
1765	13	26148615	26148739	CC571980	CC571980	CC571980	12	247	247	90	88	81
1766	13	25177525	25177783	BZ876335	BZ876335	BZ876335	12	261	261	90	88	81
1768	13	24721355	24721522	AW480838	AW480838	AW480838	12	265	265	90	88	81
1769	13	24174020	24174206	BZ886962	BZ886962	BZ886962	12	281	281	90	88	81
1770	13	23055464	23055773	BZ944119	BZ944119	BZ944119	12	285	285	90	88	81
1771	13	21893301	21893962	BZ939065	BZ939065	BZ939065	12	289	289	90	88	81
1772	13	21208431	21208681	BZ937103	BZ937103	BZ937103	12	293	293	90	88	81
1773	13	68461811	68462157	AW314792	AW314792	AW314792	12	293	293	out-of-place	out-of-place	deleted
1774	13	20259663	20259739	CC568616	CC568616	CC568616	12	301	301	90	88	81
1775	13	19143311	19143451	BZ927508	BZ927508	BZ927508	12	309	309	90	88	81
1776	13	65253773	65253982	BZ831597	BZ831597	BZ831597	12	309	309	91	89	deleted
1777	13	65913868	65914058	CC544164	CC544164	CC544164	12	313	313	91	89	82
1778	13	66846638	66846976	BZ861437	BZ861437	BZ861437	12	317	317	91	89	82
1779	13	67692458	67693026	CC554527	CC554527	CC554527	12	321	321	91	89	82
1780	13	68611552	68612147	BZ918870	BZ918870	BZ918870	12	325	325	91	89	82
1781	13	69320866	69321299	CC525521	CC525521	CC525521	12	325	325	91	89	82
1782	13	70349783	70349838	BZ885444	BZ885444	BZ885444	12	340	340	91	89	82
1783	13	71333526	71334000	CC590903	CC590903	CC590903	12	340	340	91	89	82
1784	13	72234112	72234281	AW430453	AW430453	AW430453	12	340	340	91	89	82
1785	13	72392609	72392715	BZ945146	BZ945146	BZ945146	12	344	344	91	89	82
1786	13	73158654	73159140	AW346306	AW346306	AW346306	12	349	349	91	89	82
1787	13	74256749	74257383	BZ861253	BZ861253	BZ861253	12	367	367	91	89	82
1788	13	75297352	75297626	CC490042	CC490042	CC490042	12	375	375	91	89	82
1789	13	76228205	76228501	BZ846580	BZ846580	BZ846580	12	382	382	91	89	82
1790	13	77056281	77056390	CC539314	CC539314	CC539314	12	386	386	91	89	82
1792	13	78061794	78062320	BZ954236	BZ954236	BZ954236	12	394	394	91	89	82
1793	13	79023117	79023310	CC522438	CC522438	CC522438	12	394	394	91	89	82
1794	13	79930545	79930971	BZ882698	BZ882698	BZ882698	12	402	402	91	89	82
1795	13	80703062	80703564	CC527303	CC527303	CC527303	12	402	402	91	89	82
1796	13	81946721	81947334	CC484153	CC484153	CC484153	12	406	406	91	89	82
1797	13	83272303	83272400	CC543792	CC543792	CC543792	12	411	411	91	89	82
1798	13	85205526	85205812	CC587169	CC587169	CC587169	12	425	425	91	89	82
1799	13	84295729	84296063	CC481411	CC481411	CC481411	12	429	429	91	89	82
1800	13	86724308	86725028	BZ886439	BZ886439	BZ886439	12	434	434	91	89	82
1801	13	87205363	87205875	CC557838	CC557838	CC557838	12	439	439	91	89	82
1802	13	88386937	88387153	CC515061	CC515061	CC515061	12	444	444	91	89	82
1803	13	89020826	89021404	BZ924139	BZ924139	BZ924139	12	444	444	91	89	82
1804	13	90282051	90282301	CC768546	CC768546	CC768546	12	444	444	91	89	82
1805	13	91325337	91325622	BZ932778	BZ932778	BZ932778	12	464	464	91	89	82
1807	13	92197603	92197834	CC516443	CC516443	CC516443	12	472	472	91	89	82
1808	13	93227511	93227793	BZ947903	BZ947903	BZ947903	12	472	472	91	89	82

1809	13	94471425	94471494	BZ922176	BZ922176	BZ922176	12	496	496	91	89	82	
1810	13	95513695	95513941	BZ883697	BZ883697	BZ883697	12	517	517	91	89	82	
1811	13	96686275	96686542	BZ842445	BZ842445	BZ842445	12	533	533	91	89	82	
1812	13	97739091	97739207	BZ841235	BZ841235	BZ841235	12	543	543	91	89	82	
1813	13	98802168	98802418	BZ954737	BZ954737	BZ954737	12	547	547	91	89	82	
1814	13	99865912	99866440	CC486421	CC486421	CC486421	12	557	557	91	89	82	
1815	13	101188809	101188943	CC582006	CC582006	CC582006	12	572	572	91	89	82	
1816	13	102088352	102088805	CC539979	CC539979	CC539979	12	572	572	91	89	82	
1817	13	102280589	102280691	BZ853173	BZ853173	BZ853173	12	577	577	91	89	82	
1818	13	102364684	102365387	CC555774	CC555774	CC555774	12	577	577	91	89	82	
1819	13	103408828	103409439	CC542659	CC542659	CC542659	12	577	577	91	89	82	
1820	13	104183477	104183641	CC540658	CC540658	CC540658	12	581	581	91	89	82	
1822	13	105398078	105398537	CC536029	CC536029	CC536029	12	602	602	91	89	82	
1823	13	106443851	106444458	CC551271	CC551271	CC551271	12	606	606	91	89	82	
1824	13	107586385	107586572	BZ896623	BZ896623	BZ896623	12	617	617	91	89	82	
1826	13	108575396	108575722	BZ909620	BZ909620	BZ909620	12	638	638	91	89	82	
1827	13	109807782	109807936	CC555395	CC555395	CC555395	12	643	643	91	89	82	
1829	13	110655606	110655752	CC511279	CC511279	CC511279	12	652	652	91	89	82	
1830	13	111762345	111762441	BZ885532	BZ885532	BZ885532	12	657	657	91	89	82	
1831	13	112783510	112783609	CC544928	CC544928	CC544928	12	692	692	91	89	82	
1832	13	112851231	112851773	X00673	X00673	X00673	12	702	702	91	89	82	
1833	13	113024836	113024980	L09113	L09113	L09113	12	702	702	91	89	deleted	
1834	20	8183547	8183781	CC470305	CC470305	CC470305	13	0	0	92	90	83	
1835	20	8693770	8694005	J03137	J03137	J03137	13	8	8	92	90	83	
1836	20	9216137	9216605	BZ898957	BZ898957	BZ898957	13	11	11	92	90	83	
1837	20	10282030	10282641	CC587701	CC587701	CC587701	13	17	17	92	90	83	
1838	20	10552949	10553309	AW267000	AW267000	AW267000	13	26	26	92	90	83	
1839	20	10555309	10555434	AW416418	AW416418	AW416418	13	33	33	92	90	83	
1840	20	11239772	11239856	CC534153	CC534153	CC534153	13	42	42	92	90	83	
1841	20	12218282	12218632	CC479427	CC479427	CC479427	13	57	57	92	90	83	
1842	20	13262035	13262454	CC477540	CC477540	CC477540	13	78	78	92	90	83	
1843	20	14303929	14304101	CC568704	CC568704	CC568704	13	88	88	92	90	83	
1844	20	15340233	15340502	BZ846431	BZ846431	BZ846431	13	102	102	92	90	83	
1845	20	16660807	16660981	AW462538	AW462538	AW462538	13	112	112	92	90	83	
1846	10	12968413	12968665	BZ863771	BZ863771	BZ863771	13	130	130	93	91	84	
1847	10	11871358	11871671	CC584524	CC584524	CC584524	13	157	157	93	91	84	
1848	10	10712064	10712468	BZ848795	BZ848795	BZ848795	13	180	180	93	91	84	
1849	10	9591820	9592319	CC532463	CC532463	CC532463	13	190	190	93	91	84	
1851	10	8663584	8663655	CC550213	CC550213	CC550213	13	197	197	93	91	84	
1852	10	7595728	7596017	CC496972	CC496972	CC496972	13	197	197	93	91	84	
1853	10	6670522	6670983	BZ906185	BZ906185	BZ906185	13	197	197	93	91	84	
1854	10	27083626	27083853	CC515837	CC515837	CC515837	13	209	209	singleton	Singleton	singleton	
1855	10	35508602	35508875	CC529324	CC529324	CC529324	13	216	216	94	92	85	
1856	10	34741404	34741981	CC585079	CC585079	CC585079	13	219	219	94	92	85	
1857	10	33818029	33818638	CC560668	CC560668	CC560668	13	223	223	94	92	85	
1858	10	19750350	19751171	BZ891499	BZ891499	BZ891499	13	232	232	95	93	86	
1859	10	20673742	20674003	CC500379	CC500379	CC500379	13	232	232	95	93	86	
1860	10	21692204	21692348	BZ913728	BZ913728	BZ913728	13	232	232	95	93	86	
1861	10	22863908	22864228	AW289368	AW289368	AW289368	13	240	240	95	93	86	
1862	10	23793104	23793260	CC473067	CC473067	CC473067	13	258	258	95	93	86	
1863	10	24782334	24782757	CC484042	CC484042	CC484042	13	258	258	95	93	deleted	
1864	10	25917515	25918065	BZ886452	BZ886452	BZ886452	13	258	258	95	93	deleted	
1865	10	36866727	36866906	BZ900194	BZ900194	BZ900194	13	258	258	singleton	Singleton	deleted	
1866	10	13370361	13370545	AF011925	AF011925	AF011925	13	258	258	96	94	deleted	
1867	10	13916409	13916720	CC769552	CC769552	CC769552	13	258	258	96	94	deleted	
1868	10	14930794	14930948	CC578975	CC578975	CC578975	13	269	269	96	94	87	
1869	10	15916391	15916779	BZ863038	BZ863038	BZ863038	13	274	274	96	94	87	
1870	10	16982568	16982877	BZ921480	BZ921480	BZ921480	13	285	285	96	94	87	
1871	10	17679444	17679553	CC591511	CC591511	CC591511	13	285	285	96	94	87	
1872	10	18722312	18722710	CC492765	CC492765	CC492765	13	285	285	96	94	87	
1873	10	32702848	32703080	BZ909411	BZ909411	BZ909411	13	290	290	97	95	88	
1874	10	31643886	31644070	BZ875181	BZ875181	BZ875181	13	300	300	97	95	88	
1875	10	30655449	30655749	CC586447	CC586447	CC586447	13	300	300	97	95	88	
1876	10	29809472	29809750	AF025996	AF025996	AF025996	13	300	300	97	95	88	
1877	10	28951640	28951945	AW658318	AW658318	AW658318	13	300	300	97	95	88	
1878	10	27819599	27820029	CC547777	CC547777	CC547777	13	310	310	97	95	88	
1879	10	28004008	28004101	AW462460	AW462460	AW462460	13	310	310	97	95	88	
1880	20	17362911	17363081	BZ948834	BZ948834	BZ948834	13	318	318	98	96	89	
1881	20	18401460	18401665	BZ845872	BZ845872	BZ845872	13	327	327	98	96	89	
1882	20	20568461	20568636	CC526619	CC526619	CC526619	13	331	331	98	96	89	
1883	20	19592629	19592825	CC559113	CC559113	CC559113	13	331	331	98	96	89	
1884	20	21508079	21508255	CC466769	CC466769	CC466769	13	331	331	98	96	89	
1885	20	22229861	22229906	BZ919357	BZ919357	BZ919357	13	331	331	98	96	89	
1886	20	23348713	23348846	CC532673	CC532673	CC532673	13	331	331	98	96	89	
1887	20	23563880	23564003	Y10811	Y10811	Y10811	13	339	339	98	96	89	
1888	20	24472164	24472282	CC582994	CC582994	CC582994	13	347	347	98	96	89	
1890	10	5722648	5722843	BZ870107	BZ870107	BZ870107	13	359	359	99	97	90	
1891	10	5489750	5489802	AW461566	AW461566	AW461566	13	359	359	99	97	90	
1892	10	4404614	4405050	CC578894	CC578894	CC578894	13	363	363	99	97	90	
1893	10	2760319	2760472	CC555191	CC555191	CC555191	13	367	367	99	97	90	
1894	10	3884285	3884472	BZ882161	BZ882161	BZ882161	13	371	371	99	97	90	
1895	10	1641312	1641693	CC549364	CC549364	CC549364	13	375	375	99	97	90	
1896	10	1053013	1053153	AW356149	AW356149	AW356149	13	375	375	99	97	90	
1897	10	148819	149446	CC487237	CC487237	CC487237	13	375	375	99	97	90	
1898	20	7041250	7041401	CC769776	CC769776	CC769776	13	383	383	100	98	91	
1899	20	5852336	5852747	U88551	U88551	U88551	13	383	383	100	98	91	
1900	20	4785408	4785877	BZ921973	BZ921973	BZ921973	13	383	383	100	98	91	
1901	20	3995726	3995870	BZ899470	BZ899470	BZ899470	13	391	391	100	98	91	
1902	20	3152016	3152071	AW289304	AW289304	AW289304	13	399	399	100	98	91	
1903	20	2626452	2626899	CC514264	CC514264	CC514264	13	399	399	100	98	91	
1904	20	1742616	1742957	CC497713	CC497713	CC497713	13	402	402	100	98	91	
1905	20	25374515	25374772	BZ908049	BZ908049	BZ908049	13	402	402	singleton	Singleton	deleted	
1906	20	62244532	62244836	CC567737	CC567737	CC567737	13	406	406	101	99	92	
1907	20	61417160	61417324	CC503739	CC503739	CC503739	13	412	412	101	99	92	
1908	20	60446077	60446362	BZ875209	BZ875209	BZ875209	13	416	416	101	99	92	
1909	20	59446611	59447031	CC558412	CC558412	CC558412	13	437	437	101	99	92	
1910	20	58357132	58357569	CC585694	CC585694	CC585694	13	440	440	101	99	92	
1911	20	57430401	57430851	BZ944092	BZ944092	BZ944092	13	440	440	101	99	92	
1913	20	56919132	56919601	X03404	X03404	X03404	13	462	462	101	99	92	
1914	20	56527141	56527602	CC558887	CC558887	CC558887	13	478	478	101	99	92	
1915	20	55387155	55387348	AW289274	AW289274	AW289274	13	486	486	101	99	92	
1916	20	55382977	55383304	CC512700	CC512700	CC512700	13	494	494	101	99	92	
1917	20	54406014	54406721	CC471494	CC471494	CC471494	13	502	502	101	99	92	
1918	20	54376944	54377180	AW267142	AW267142	AW267142	13	509	509	101	99	92	
1919	20	1246932	1247097	CC578347	CC578347	CC578347	13	520	520	102	100	93	
1920	20	983962	984189	CC536365	CC536365	CC536365	13	520	520	102	100	deleted	
1921	20	548817	549334	CC573940	CC573940	CC573940	13	523	523	102	100	93	This marker was jumped to the bottom block by ST and manually
1922	20	29309116	29309264	BZ841113	BZ841113	BZ841113	13	534	534	103	101	singleton	This marker was jumped to the top block by ST and manually
1923	20	367095	367193	AW652840	AW652840	AW652840	13	541	541	102	100	singleton	
1924	20	30246148	30246383	AW326128	AW326128	AW326128	13	552	552	103	101	94	
1925	20	30420372	30420617	CC768906	CC768906	CC768906	13	556	556	103	101	94	
1926	20	31436991	31437229	BZ880334	BZ880334	BZ880334	13	559	559	103	101	94	
1927	20	32414649	32414913	CC476909	CC476909	CC476909	13	559	559	103	101	94	
1928	20	33226175	33226407	L39065	L39065	L39065	13	567	567	103	101	94	
1929	20	33407945	33408074	CC583336	CC583336	CC583336	13	567	567	103	101	94	
1930	20	34000784	34001104	BF440198	BF440198	BF440198	13	578	578	103	101	94	
1931	20	34508599	34508839	CC526165	CC526165	CC526165	13	606	606	103	101	94	
1932	20	35303118	35303436	AW261152	AW261152	AW261152	13	610	610	103	101	94	
1933	20	35778268	35778595	BZ956466	BZ956466	BZ956466	13	620	620	103	101	94	
1934	20	36201212	36201472	X60686	X60686	X60686	13	628	628	103	101	94	
1935	20	36399007	36399290	X52563	X52563	X52563	13	639	639	103	101	94	
1936	20	36738944	36739395	CC489570	CC489570	CC489570	13	647	647	103	101	94	
1937	20	37685727	37686170	CC527492	CC527492	CC527492	13	668	668	103	101	94	
1938	20	38587911	38588110	BZ882151	BZ882151	BZ882151	13	668	668	103	101	94	
1939	20	39538476	39538540	CC563305	CC563305	CC563305	13	676	676	103	101	94	
1940	20	40595010	40595085	CC532822	CC532822	CC532822	13	683	683	103	101	94	
1942	20	41743792	41743958	CC534399	CC534399	CC534399	13	691	691	103	101	94	
1943	20	42803793	42804037	CC571705	CC571705	CC571705	13	691	691	103	101	94	
1944	20	43852101	43852202	CC565772	CC565772	CC565772	13	691	691	103	101	94	
1945	20	44961995	44962774	CC530071	CC530071	CC530071	13	712	712	103	101	94	
1946	20	45919866	45920589	CC576203	CC576203	CC576203	13	721	721	103	101	94	
1947	20	47066931	47067425	BZ931499	BZ931499	BZ931499	13	724	724	103	101	94	
1948	20	47989365	47989669	AW289276	AW289276	AW289276	13	730	730	103	101	94	
1949	20	48180785	48180918	BZ886347	BZ886347	BZ886347	13	740	740	103	101	94	
1951	20	48953788	48954078	CC520680	CC520680	CC520680	13	774	774	103	101	94	
1952	20	50093090	50093295	CC566819	CC566819	CC566819	13	783	783	103	101	94	
1953	20	50688697	50688916	CC482682	CC482682	CC482682	13	828	828	103	101	94	
1954	20	52045975	52046271	BZ901044	BZ901044	BZ901044	13	836	836	103	101	94	
1955	20	52887177	52887760	CC767181	CC767181	CC767181	13	838	838	103	101	94	
1956	20	53101792	53102069	CC492354	CC492354	CC492354	13	841	841	103	101	94	
1957	20	53923899	53923989	CC537861	CC537861	CC537861	13	848	848	103	101	94	
1958	20	54155793	54155927	CC508793	CC508793	CC508793	13	856	856	103	101	94	
1959	8	145705482	145705793	CC505317	CC505317	CC505317	14	0	0	104	102	95	
1960	8	144972020	144972235	AW462639	AW462639	AW462639	14	24	24	104	102	95	
1962	8	143853583	143853840	BZ890526	BZ890526	BZ890526	14	33	33	104	102	95	
1963	8	142896393	142896801	CC587005	CC587005	CC587005	14	33	33	104	102	95	
1964	8	141898160	141898365	CC771913	CC771913	CC771913	14	33	33	104	102	95	
1966	8	140567810	140567982	BZ926698	BZ926698	BZ926698	14	45	45	104	102	95	
1967	8	139276653	139277053	BZ861497	BZ861497	BZ861497	14	54	54	104	102	95	
1968	8	138214144	138214915	CC501752	CC501752	CC501752	14	54	54	104	102	95	
1970	8	137093531	137093807	BZ869505	BZ869505	BZ869505	14	80	80	104	102	95	
1971	8	136012492	136012970	BZ954910	BZ954910	BZ954910	14	86	86	104	102	95	
1973	8	133967874	133968617	X02815	X02815	X02815	14	105	105	104	102	95	
1974	8	134971058	134971134	BZ880216	BZ880216	BZ880216	14	108	108	104	102	95	
1976	8	132706756	132707367	CC582411	CC582411	CC582411	14	129	129	104	102	95	
1977	8	131573900	131574147	CC522480	CC522480	CC522480	14	149	149	104	102	95	
1978	8	130591182	130591523	BZ861624	BZ861624	BZ861624	14	158	158	104	102	95	
1979	8	129439513	129439826	CC539161	CC539161	CC539161	14	164	164	104	102	95	
1981	8	128562005	128562324	BZ938174	BZ938174	BZ938174	14	171	171	104	102	95	
1982	8	127717458	127717728	CC526724	CC526724	CC526724	14	174	174	104	102	95	
1983	8	126644075	126644200	BZ910160	BZ910160	BZ910160	14	177	177	104	102	95	
1984	8	125603553	125603635	BZ872125	BZ872125	BZ872125	14	187	187	104	102	95	
1985	8	124611633	124612035	AW466086	AW466086	AW466086	14	193	193	104	102	95	
1987	8	123831333	123831508	CC565230	CC565230	CC565230	14	196	196	104	102	95	
1988	8	122865969	122866355	CC512444	CC512444	CC512444	14	196	196	104	102	95	
1989	8	122049872	122050055	CC562902	CC562902	CC562902	14	200	200	104	102	95	
1990	8	48702625	48702751	CC579984	CC579984	CC579984	14	208	208	105	103	96	
1991	8	49028647	49028899	CC481105	CC481105	CC481105	14	208	208	105	103	96	
1992	8	49658530	49658865	BZ879532	BZ879532	BZ879532	14	218	218	105	103	96	
1993	8	50878666	50878819	CC509329	CC509329	CC509329	14	244	244	105	103	96	
1994	8	51706664	51706843	BZ955812	BZ955812	BZ955812	14	244	244	105	103	96	
1995	8	52853882	52854043	CC594152	CC594152	CC594152	14	247	247	105	103	96	
1996	8	53698359	53698643	AW289400	AW289400	AW289400	14	249	249	105	103	96	
1997	8	54954366	54954701	U89254	U89254	U89254	14	249	249	105	103	96	
1998	8	55990614	55990820	CC775413	CC775413	CC775413	14	252	252	105	103	96	
1999	8	56831162	56831567	CC763831	CC763831	CC763831	14	252	252	105	103	96	
2000	8	57911036	57911441	BZ922125	BZ922125	BZ922125	14	260	260	105	103	96	
2001	8	58888693	58889045	CC566016	CC566016	CC566016	14	260	260	105	103	96	
2002	8	59918733	59919248	CC511880	CC511880	CC511880	14	263	263	105	103	96	
2003	8	61087143	61087668	BZ953058	BZ953058	BZ953058	14	263	263	105	103	96	
2005	8	62094907	62095317	CC771526	CC771526	CC771526	14	276	276	105	103	96	
2006	8	62575670	62575899	AW261136	AW261136	AW261136	14	276	276	105	103	96	
2007	8	62666930	62667136	BZ898210	BZ898210	BZ898210	14	282	282	105	103	96	
2008	8	63731983	63732044	BZ939149	BZ939149	BZ939149	14	285	285	105	103	96	
2009	8	64768219	64768651	BZ925908	BZ925908	BZ925908	14	287	287	105	103	96	
2010	8	65795788	65795971	BZ956263	BZ956263	BZ956263	14	299	299	105	103	96	
2011	8	66794176	66794304	BZ918446	BZ918446	BZ918446	14	329	329	105	103	96	
2012	8	67737119	67737288	CC587900	CC587900	CC587900	14	333	333	105	103	96	
2013	8	68823620	68824417	CC591419	CC591419	CC591419	14	333	333	105	103	96	
2014	8	69681347	69681452	BZ934329	BZ934329	BZ934329	14	336	336	105	103	96	
2016	8	70799007	70799594	BZ844786	BZ844786	BZ844786	14	339	339	105	103	96	
2017	8	71595091	71595500	CC519284	CC519284	CC519284	14	350	350	105	103	96	
2018	8	72605495	72605668	CC595117	CC595117	CC595117	14	350	350	105	103	96	
2019	8	73684568	73684647	CC582915	CC582915	CC582915	14	353	353	105	103	96	
2020	8	74624394	74624691	AW267147	AW267147	AW267147	14	353	353	105	103	96	
2021	8	75052634	75052865	BZ920596	BZ920596	BZ920596	14	357	357	105	103	96	
2022	8	75920914	75921086	BZ838498	BZ838498	BZ838498	14	361	361	105	103	96	
2023	8	76814441	76814797	BZ939006	BZ939006	BZ939006	14	361	361	105	103	96	
2024	8	77810980	77811762	CC539828	CC539828	CC539828	14	372	372	105	103	96	
2025	8	78857314	78857801	BZ849172	BZ849172	BZ849172	14	379	379	105	103	96	
2026	8	79807845	79808623	J04156	J04156	J04156	14	382	382	105	103	96	
2027	8	79962589	79962802	CC505028	CC505028	CC505028	14	382	382	105	103	96	
2028	8	80962457	80962934	CC512807	CC512807	CC512807	14	382	382	105	103	96	
2029	8	82083893	82084035	CC499034	CC499034	CC499034	14	386	386	105	103	96	
2030	8	82358154	82358328	U55188	U55188	U55188	14	389	389	105	103	96	
2031	8	82557855	82558012	X89244	X89244	X89244	14	400	400	105	103	96	
2032	8	120500470	120500728	AF055076	AF055076	AF055076	14	400	400	106	104	deleted	
2033	8	119874457	119874886	CC765724	CC765724	CC765724	14	403	403	106	104	97	
2034	8	118820557	118820908	BZ932339	BZ932339	BZ932339	14	410	410	106	104	97	
2035	8	118602239	118602473	AW464786	AW464786	AW464786	14	417	417	106	104	97	
2036	8	117727637	117728453	BZ868745	BZ868745	BZ868745	14	424	424	106	104	97	
2037	8	117551884	117552315	BZ915486	BZ915486	BZ915486	14	443	443	106	104	97	
2038	8	117247687	117247944	CC557766	CC557766	CC557766	14	448	448	106	104	97	
2039	8	115674315	115674717	CC592690	CC592690	CC592690	14	471	471	106	104	97	
2040	8	114508699	114508813	CC502613	CC502613	CC502613	14	498	498	106	104	97	
2041	8	113427276	113427850	CC773178	CC773178	CC773178	14	506	506	106	104	97	
2042	8	112550883	112551263	BZ907096	BZ907096	BZ907096	14	515	515	106	104	97	
2043	8	111671315	111671542	BZ933774	BZ933774	BZ933774	14	548	548	106	104	97	
2044	8	110701559	110701681	CC528682	CC528682	CC528682	14	548	548	106	104	97	
2045	8	109882841	109883054	CC555993	CC555993	CC555993	14	551	551	106	104	97	
2046	8	108834693	108835082	CC532988	CC532988	CC532988	14	555	555	106	104	97	
2047	8	107854290	107854597	BZ885423	BZ885423	BZ885423	14	558	558	106	104	97	
2048	8	106885229	106885352	BZ846254	BZ846254	BZ846254	14	561	561	106	104	97	
2049	8	105988844	105989151	BZ952885	BZ952885	BZ952885	14	565	565	106	104	97	
2050	8	104997734	104997948	CC490247	CC490247	CC490247	14	572	572	106	104	97	
2051	8	103950911	103951153	CC558789	CC558789	CC558789	14	584	584	106	104	97	
2052	8	103258156	103258705	BZ869233	BZ869233	BZ869233	14	587	587	106	104	97	
2053	8	102770072	102770811	D10884	D10884	D10884	14	598	598	106	104	97	
2054	8	102285850	102285996	BZ939202	BZ939202	BZ939202	14	602	602	106	104	97	
2055	8	101786331	101786439	U83076	U83076	U83076	14	602	602	106	104	97	
2056	8	100196899	100197403	BZ940696	BZ940696	BZ940696	14	602	602	106	104	97	
2057	8	99147881	99147952	BZ949690	BZ949690	BZ949690	14	602	602	106	104	97	
2058	8	98025126	98025315	BZ831476	BZ831476	BZ831476	14	623	623	106	104	97	
2059	8	97102816	97103456	CC526242	CC526242	CC526242	14	634	634	106	104	97	
2060	8	95869362	95869507	BZ881960	BZ881960	BZ881960	14	638	638	106	104	97	
2061	8	95935149	95935364	CC586302	CC586302	CC586302	14	638	638	106	104	97	
2062	8	94867074	94867196	CC773564	CC773564	CC773564	14	642	642	106	104	97	
2063	8	93790333	93790737	BZ834224	BZ834224	BZ834224	14	651	651	106	104	97	
2064	8	92717801	92717970	BZ881058	BZ881058	BZ881058	14	651	651	106	104	97	

2065	8	91721444	91721638	CC587432	CC587432	CC587432	14	651	651	106	104	97		
2066	8	90688119	90688820	CC559878	CC559878	CC559878	14	656	656	106	104	97		
2067	8	90846660	90846960	CC554922	CC554922	CC554922	14	656	656	106	104	97		
2068	8	89439276	89439511	BZ867957	BZ867957	BZ867957	14	665	665	106	104	97		
2069	8	88077896	88078078	CC558033	CC558033	CC558033	14	680	680	106	104	97		
2070	8	86545628	86545787	AW359112	AW359112	AW359112	14	680	680	106	104	97		
2071	8	87306779	87306901	CC509209	CC509209	CC509209	14	680	680	106	104	97		
2072	8	85368578	85369346	CC510731	CC510731	CC510731	14	685	685	106	104	97		
2073	8	84183504	84183651	BZ883248	BZ883248	BZ883248	14	700	700	106	104	97		
2074	8	82753909	82754022	J05394	J05394	J05394	14	708	708	106	104	97		
2075	11	105350152	105350655	BZ904394	BZ904394	BZ904394	15	0	0	107	105	98		
2076	11	103999903	104000177	CC561722	CC561722	CC561722	15	9	9	107	105	98		
2077	11	103770038	103770456	BZ941449	BZ941449	BZ941449	15	18	18	107	105	98		
2078	11	102957520	102957722	BZ936687	BZ936687	BZ936687	15	24	24	107	105	98		
2079	11	101774025	101774095	AW289413	AW289413	AW289413	15	42	42	107	105	98		
2080	11	100417744	100418228	BZ939858	BZ939858	BZ939858	15	51	51	107	105	98		
2081	11	99486633	99486755	BZ927106	BZ927106	BZ927106	15	64	64	107	105	98		
2082	11	98057943	98058502	BZ955507	BZ955507	BZ955507	15	73	73	107	105	98		
2083	11	97468044	97468208	CC524771	CC524771	CC524771	15	83	83	107	105	98		
2084	11	96432094	96432194	BZ912680	BZ912680	BZ912680	15	90	90	107	105	98		
2086	11	95141849	95142073	AW437796	AW437796	AW437796	15	104	104	107	105	98		
2087	11	95097992	95098333	BZ955534	BZ955534	BZ955534	15	107	107	107	105	98		
2088	11	94205732	94206216	BZ941759	BZ941759	BZ941759	15	114	114	107	105	98		
2089	11	106279226	106279419	CC536484	CC536484	CC536484	15	117	117	108	106	99		
2090	11	107119094	107119579	BZ951933	BZ951933	BZ951933	15	124	124	108	106	99		
2091	11	108398683	108398767	BZ956575	BZ956575	BZ956575	15	127	127	108	106	99		
2092	11	109838207	109838400	D00471	D00471	D00471	15	152	152	108	106	99		
2093	11	111252548	111253240	BZ902157	BZ902157	BZ902157	15	164	164	108	106	99		
2094	11	111470738	111470969	U50987	U50987	U50987	15	168	168	108	106	99		
2095	11	112353401	112353972	BZ927848	BZ927848	BZ927848	15	185	185	108	106	99		
2096	11	112651193	112651983	X16451	X16451	X16451	15	188	188	108	106	99		
2097	11	116208688	116208982	BE217514	BE217514	BE217514	15	188	188	108	106	99		
2098	11	113523776	113524252	BZ899722	BZ899722	BZ899722	15	197	197	108	106	99		
2099	11	114892040	114892310	BZ907632	BZ907632	BZ907632	15	201	201	108	106	99		
2100	11	116208819	116208982	BE217430	BE217430	BE217430	15	206	206	108	106	99		
2101	11	116211677	116211986	AW266863	AW266863	AW266863	15	210	210	108	106	99		
2102	11	116543346	116544030	D49678	D49678	D49678	15	218	218	108	106	99		
2103	11	116584727	116585054	BZ941912	BZ941912	BZ941912	15	227	227	108	106	99		
2104	11	117762225	117762609	BZ900304	BZ900304	BZ900304	15	231	231	108	106	99		
2105	11	118426948	118427043	U83019	U83019	U83019	15	244	244	108	106	99		
2106	11	119036912	119037264	AW289317	AW289317	AW289317	15	252	252	108	106	99		
2109	11	120925850	120926231	BZ929011	BZ929011	BZ929011	15	264	264	108	106	99		
2110	11	121770107	121770523	BZ896953	BZ896953	BZ896953	15	268	268	108	106	99		
2111	11	123005063	123005213	CC545465	CC545465	CC545465	15	272	272	108	106	99		
2112	11	18003675	18003790	BZ949997	BZ949997	BZ949997	15	272	272	109	107	deleted		
2113	11	17966720	17966880	AW417711	AW417711	AW417711	15	277	277	109	107	100		
2114	11	17308248	17308459	AW428590	AW428590	AW428590	15	277	277	109	107	100		
2115	11	16733957	16734158	BZ936721	BZ936721	BZ936721	15	286	286	109	107	100		
2116	11	15992968	15993462	BZ910135	BZ910135	BZ910135	15	294	294	109	107	100		
2117	11	14739651	14739764	BZ916579	BZ916579	BZ916579	15	302	302	109	107	100		
2118	11	13954314	13954630	BZ907353	BZ907353	BZ907353	15	311	311	109	107	100		
2119	11	13366840	13367266	AW464552	AW464552	AW464552	15	311	311	109	107	100		
2120	11	12138967	12139553	BZ955657	BZ955657	BZ955657	15	319	319	109	107	100		
2121	11	11266744	11267044	BZ840058	BZ840058	BZ840058	15	327	327	109	107	100		
2122	11	10275886	10276179	BZ909975	BZ909975	BZ909975	15	331	331	109	107	100		
2123	11	9147758	9148239	BZ898384	BZ898384	BZ898384	15	335	335	109	107	100		
2124	11	8519726	8520139	BZ901233	BZ901233	BZ901233	15	343	343	109	107	100		
2125	11	7124759	7124974	BZ955804	BZ955804	BZ955804	15	355	355	109	107	100		
2126	11	6590732	6590781	BE217530	BE217530	BE217530	15	364	364	109	107	100		
2127	11	5610085	5610256	BZ932604	BZ932604	BZ932604	15	367	367	109	107	100		
2128	11	5204158	5204653	X00376	X00376	X00376	15	371	371	109	107	100		
2129	11	4976836	4977257	BZ935869	BZ935869	BZ935869	15	375	375	109	107	100		
2130	11	4116233	4116373	AW244890	AW244890	AW244890	15	379	379	109	107	100		
2131	11	71320012	71320141	BZ934882	BZ934882	BZ934882	15	390	390	110	108	101		
2132	11	71413119	71413424	AW463658	AW463658	AW463658	15	397	397	110	108	101		
2133	11	71978558	71978735	BZ897822	BZ897822	BZ897822	15	401	401	110	108	101		
2134	11	72227011	72227458	X73790	X73790	X73790	15	401	401	110	108	101		
2135	11	72922009	72922179	BZ942903	BZ942903	BZ942903	15	409	409	110	108	101		
2136	11	73394863	73395081	AF092048	AF092048	AF092048	15	413	413	110	108	101		
2137	11	74838563	74838716	BZ937307	BZ937307	BZ937307	15	431	431	110	108	101		
2138	11	74960470	74960953	AW653706	AW653706	AW653706	15	431	431	110	108	101		
2140	11	75840131	75840718	BZ938691	BZ938691	BZ938691	15	443	443	110	108	101		
2141	11	26287445	26288021	BZ898298	BZ898298	BZ898298	15	455	455	111	109	102		
2142	11	27074942	27075512	CC503764	CC503764	CC503764	15	467	467	111	109	102		
2143	11	27817116	27817742	BZ903879	BZ903879	BZ903879	15	483	483	111	109	102		
2145	11	28170057	28170443	BZ902707	BZ902707	BZ902707	15	494	494	111	109	102		
2146	11	28646950	28647188	BZ900212	BZ900212	BZ900212	15	498	498	111	109	102		
2147	11	29660983	29661361	BZ925132	BZ925132	BZ925132	15	505	505	111	109	102		
2148	11	29988366	29991275	X57033	X57033	X57033	15	505	505	111	109	102		
2149	11	30373583	30374094	BZ935672	BZ935672	BZ935672	15	509	509	111	109	102		
2150	11	31098240	31098454	BZ933009	BZ933009	BZ933009	15	513	513	111	109	102		
2151	11	32083482	32083646	AW266879	AW266879	AW266879	15	516	516	111	109	102		
2152	11	32424262	32424636	BZ913019	BZ913019	BZ913019	15	524	524	111	109	102		
2153	11	33509915	33510028	BZ951441	BZ951441	BZ951441	15	537	537	111	109	102		
2154	11	33812276	33812355	BZ908190	BZ908190	BZ908190	15	546	546	111	109	102		
2155	11	35207250	35207592	S63418	S63418	S63418	15	550	550	111	109	102		
2156	11	35297598	35297998	BZ937718	BZ937718	BZ937718	15	550	550	111	109	102		
2158	11	36208050	36208342	BZ931055	BZ931055	BZ931055	15	554	554	111	109	102		
2159	11	37435019	37435182	BZ930929	BZ930929	BZ930929	15	554	554	111	109	102		
2160	11	38488144	38488533	BZ931300	BZ931300	BZ931300	15	563	563	111	109	102		
2161	11	39568433	39568828	BE217538	BE217538	BE217538	15	563	563	111	109	102		
2162	11	40071417	40071779	CC447440	CC447440	CC447440	15	571	571	111	109	102		
2164	11	41909455	41909896	BZ938943	BZ938943	BZ938943	15	576	576	111	109	102		
2165	11	42297994	42298174	BZ903650	BZ903650	BZ903650	15	576	576	111	109	102		
2166	11	43666615	43666761	BZ930289	BZ930289	BZ930289	15	576	576	111	109	102		
2168	11	44085801	44086376	AF089748	AF089748	AF089748	15	596	596	111	109	102		
2169	11	44860537	44860705	CC547809	CC547809	CC547809	15	607	607	111	109	102		
2171	11	46221363	46221592	BZ918609	BZ918609	BZ918609	15	621	621	111	109	102		
2172	11	46706105	46706292	J00041	J00041	J00041	15	621	621	111	109	102		
2174	11	47303340	47303510	BZ901239	BZ901239	BZ901239	15	631	631	111	109	102		
2175	11	48103276	48103403	BZ903982	BZ903982	BZ903982	15	636	636	111	109	102		
2176	11	48304688	48304777	CC540672	CC540672	CC540672	15	657	657	111	109	102		
2177	11	51314853	51314922	BZ855504	BZ855504	BZ855504	15	657	657	111	109	102		
2178	11	57138541	57138634	AW428604	AW428604	AW428604	15	666	666	112	109	102	ST here	discrepant case in HSB definitions between SyntenyTracker and Everts-van der Wind et al
2179	11	57791000	57791195	CC543770	CC543770	CC543770	15	670	670	112	109	102		
2180	11	58050923	58051060	AW289379	AW289379	AW289379	15	675	675	112	109	102		
2181	11	58233993	58234231	AF022073	AF022073	AF022073	15	683	683	112	109	102		
2182	11	59162056	59162465	AW428581	AW428581	AW428581	15	688	688	112	109	102		
2183	11	59377226	59377369	AW478378	AW478378	AW478378	15	688	688	112	109	102		
2184	11	59396546	59396605	BZ921156	BZ921156	BZ921156	15	709	709	112	109	102		
2185	11	133731402	133731503	BZ863012	BZ863012	BZ863012	15	709	709	singleton	Singleton	deleted		
2186	1	200757464	200757598	BZ938148	BZ938148	BZ938148	16	0	0	113	110	103	ST here	discrepant case in HSB definitions between SyntenyTracker and Everts-van der Wind et al
2187	1	201891800	201891956	BZ939973	BZ939973	BZ939973	16	2	2	113	110	103		
2188	1	202414763	202414985	AW267148	AW267148	AW267148	16	20	20	113	110	103		
2190	1	120517059	120517150	AW267020	AW267020	AW267020	16	24	24	114	out-of-place	singleton	ST here	ST is correct in calling them out-of-place markers
2191	1	120469660	120469887	BZ948329	BZ948329	BZ948329	16	24	24	114	out-of-place	deleted		
2193	1	203661529	203661730	Z31694	Z31694	Z31694	16	44	44	115	110	singleton		
2194	1	203674796	203674918	AW326993	AW326993	AW326993	16	44	44	115	110	deleted		
2195	1	192974209	192974541	BZ901614	BZ901614	BZ901614	16	51	51	116	111	104		
2196	1	191814955	191815429	BZ917733	BZ917733	BZ917733	16	72	72	116	111	104		
2197	1	190992409	190992727	BZ951725	BZ951725	BZ951725	16	83	83	116	111	104		
2198	1	189844231	189844466	BZ921749	BZ921749	BZ921749	16	88	88	116	111	104		
2199	1	189036793	189036935	BZ937741	BZ937741	BZ937741	16	88	88	116	111	104		
2200	1	187738146	187738348	BZ948508	BZ948508	BZ948508	16	103	103	116	111	104		
2201	1	187131274	187131482	CC495501	CC495501	CC495501	16	114	114	116	111	104		
2202	1	186177895	186178195	BZ954987	BZ954987	BZ954987	16	114	114	116	111	104		
2203	1	184859184	184859836	CC562100	CC562100	CC562100	16	125	125	116	111	104		
2204	1	213020281	213020740	BZ955516	BZ955516	BZ955516	16	127	127	117	112	105		
2206	1	214122845	214123435	BZ944687	BZ944687	BZ944687	16	144	144	117	112	105		
2207	1	215517008	215517143	BZ937266	BZ937266	BZ937266	16	158	158	117	112	105		
2208	1	216699661	216699801	BZ950494	BZ950494	BZ950494	16	163	163	117	112	105		
2209	1	217800076	217800214	BZ912078	BZ912078	BZ912078	16	172	172	117	112	105		
2211	1	219295252	219295881	AW289394	AW289394	AW289394	16	221	221	117	112	105		
2212	1	219768370	219768727	X15543	X15543	X15543	16	226	226	117	112	105		
2213	1	219934057	219934560	BZ884839	BZ884839	BZ884839	16	231	231	117	112	105		
2214	1	220992283	220992590	BZ945071	BZ945071	BZ945071	16	236	236	117	112	105		
2215	1	222275601	222275733	BZ939769	BZ939769	BZ939769	16	239	239	117	112	105		
2216	1	223121033	223121374	BZ924566	BZ924566	BZ924566	16	264	264	117	112	105		
2217	1	223126308	223126398	AW447142	AW447142	AW447142	16	267	267	117	112	105		
2218	1	242754927	242755596	BZ945863	BZ945863	BZ945863	16	272	272	118	113	106		
2219	1	241197934	241198373	AW425782	AW425782	AW425782	16	278	278	118	113	106		
2220	1	239804402	239804762	BZ904319	BZ904319	BZ904319	16	280	280	118	113	106		
2221	1	239059370	239059578	CC775774	CC775774	CC775774	16	286	286	118	113	106		
2223	1	237893097	237893528	BZ941771	BZ941771	BZ941771	16	292	292	118	113	106		
2224	1	237264856	237265203	AF011359	AF011359	AF011359	16	294	294	118	113	106		
2225	1	165907340	165907421	CC594788	CC594788	CC594788	16	297	297	119	114	107		
2226	1	166433417	166433807	L12039	L12039	L12039	16	300	300	119	114	107		
2227	1	166555059	166555233	AW326638	AW326638	AW326638	16	305	305	119	114	107		
2228	1	167979603	167979849	BZ945895	BZ945895	BZ945895	16	317	317	119	114	107		
2229	1	168241431	168241555	BE217459	BE217459	BE217459	16	355	355	119	114	107		
2231	1	169098730	169099248	BZ947456	BZ947456	BZ947456	16	359	359	119	114	107		
2232	1	12621576	12621810	CC581894	CC581894	CC581894	16	362	362	120	115	108		
2233	1	12611425	12611730	AF061742	AF061742	AF061742	16	369	369	120	115	108		
2234	1	11968974	11969200	AF054274	AF054274	AF054274	16	376	376	120	115	108		
2235	1	11019450	11019911	BZ950709	BZ950709	BZ950709	16	383	383	120	115	108		
2236	1	9905156	9905255	BZ917077	BZ917077	BZ917077	16	383	383	120	115	108		
2237	1	8763098	8763252	BZ940466	BZ940466	BZ940466	16	383	383	120	115	108		
2238	1	7658602	7659127	BZ907149	BZ907149	BZ907149	16	397	397	120	115	108		
2239	1	6455493	6455729	AW289372	AW289372	AW289372	16	400	400	120	115	108		
2240	1	6192887	6193181	BZ921313	BZ921313	BZ921313	16	403	403	120	115	108		
2241	1	4912751	4912967	CC580574	CC580574	CC580574	16	403	403	120	115	108		
2242	1	3622753	3622943	BZ952478	BZ952478	BZ952478	16	407	407	120	115	108		
2243	1	2472357	2472699	AW462578	AW462578	AW462578	16	410	410	120	115	108		
2244	1	1716124	1716220	BE217548	BE217548	BE217548	16	413	413	120	115	108		
2245	1	1349334	1349656	AW632181	AW632181	AW632181	16	413	413	120	115	108		
2246	1	14340372	14340831	BZ948416	BZ948416	BZ948416	16	419	419	121	116	109		
2247	1	15516308	15516600	BZ906530	BZ906530	BZ906530	16	419	419	121	116	109		
2248	1	15539182	15539341	X97635	X97635	X97635	16	431	431	121	116	109		
2249	1	170186155	170186305	AF080228	AF080228	AF080228	16	446	446	122	117	110		
2250	1	170537682	170537872	BZ922903	BZ922903	BZ922903	16	458	458	122	117	110		
2251	1	171512968	171513103	CC763750	CC763750	CC763750	16	467	467	122	117	110		
2252	1	172548208	172548314	BZ899420	BZ899420	BZ899420	16	473	473	122	117	110		
2253	1	172645647	172646181	AW289177	AW289177	AW289177	16	473	473	122	117	110		
2254	1	173596297	173596418	BZ936710	BZ936710	BZ936710	16	473	473	122	117	110		
2255	1	174387449	174387993	CC568380	CC568380	CC568380	16	478	478	122	117	110		
2256	1	175155158	175155322	BZ913462	BZ913462	BZ913462	16	481	481	122	117	110		
2257	1	176038528	176038743	BZ941051	BZ941051	BZ941051	16	481	481	122	117	110		
2258	1	177100872	177100967	BZ924236	BZ924236	BZ924236	16	487	487	122	117	110		
2259	1	178190027	178190523	BZ934506	BZ934506	BZ934506	16	510	510	122	117	110		
2260	1	178233110	178233193	BZ941186	BZ941186	BZ941186	16	514	514	122	117	110		
2261	1	179300925	179301307	AF011361	AF011361	AF011361	16	546	546	122	117	110		
2262	1	179588057	179588303	U83069	U83069	U83069	16	553	553	122	117	110		
2263	1	179796346	179796846	BZ910068	BZ910068	BZ910068	16	556	556	122	117	110		
2264	1	180260881	180261066	AF079303	AF079303	AF079303	16	556	556	122	117	110		
2265	1	180921060	180921221	BZ919156	BZ919156	BZ919156	16	559	559	122	117	110		
2266	1	182001018	182001239	CC466128	CC466128	CC466128	16	562	562	122	117	110		
2267	1	183009526	183009749	AF056218	AF056218	AF056218	16	581	581	122	117	110		
2268	1	183377191	183378297	AF031699	AF031699	AF031699	16	594	594	122	117	110		
2269	1	183666320	183666688	BZ834504	BZ834504	BZ834504	16	594	594	122	117	110		
2270	1	183679998	183680306	BZ943255	BZ943255	BZ943255	16	594	594	122	117	110		
2271	1	211839130	211839479	BZ943191	BZ943191	BZ943191	16	600	600	123	118	111		
2272	1	210893756	210893930	AW632140	AW632140	AW632140	16	600	600	123	118	111		
2273	1	210708817	210709163	BZ934435	BZ934435	BZ934435	16	603	603	123	118	111		
2274	1	209734887	209735356	BZ934726	BZ934726	BZ934726	16	606	606	123	118	111		
2275	1	209298905	209299031	AW266983	AW266983	AW266983	16	609	609	123	118	111		
2276	1	208822686	208822942	BZ945875	BZ945875	BZ945875	16	612	612	123	118	111		
2277	1	207935272	207935474	AW432047	AW432047	AW432047	16	612	612	123	118	111		
2278	1	207934714	207934913	BZ929761	BZ929761	BZ929761	16	615	615	123	118	111		
2279	1	207244694	207245876	Y13430	Y13430	Y13430	16	634	634	123	118	111		
2280	1	206797304	206797538	BZ947349	BZ947349	BZ947349	16	637	637	123	118	111		
2282	1	205876681	205877266	BZ897730	BZ897730	BZ897730	16	640	640	123	118	111		
2283	1	204668101	204668241	BZ897571	BZ897571	BZ897571	16	649	649	123	118	111		
2284	1	193858532	193859142	AW428582	AW428582	AW428582	16	651	651	124	119	112		
2285	1	195022947	195023164	AW482289	AW482289	AW482289	16	660	660	124	119	112		
2286	1	195695843	195695942	BZ904901	BZ904901	BZ904901	16	666	666	124	119	112		
2287	1	196646524	196646832	BZ908423	BZ908423	BZ908423	16	674	674	124	119	112		
2288	1	197873292	197873349	BZ909367	BZ909367	BZ909367	16	679	679	124	119	112		
2289	1	198166324	198166475	AW485011	AW485011	AW485011	16	682	682	124	119	112		
2290	1	198556560	198556724	AV609435	AV609435	AV609435	16	682	682	124	119	112		
2291	1	199503996	199504323	BZ916395	BZ916395	BZ916395	16	688	688	124	119	112		
2293	4	166481656	166481808	CC530847	CC530847	CC530847	17	11	11	125	120	113		
2294	4	166621698	166621845	AW267122	AW267122	AW267122	17	17	17	125	120	113		
2295	4	167047862	167047955	BZ891644	BZ891644	BZ891644	17	25	25	125	120	113		
2296	4	156492686	156493992	U50144	U50144	U50144	17	31	31	126	121	114		
2297	4	155067091	155067370	AV592376	AV592376	AV592376	17	31	31	126	121	114		
2298	4	153602963	153603153	BE477144	BE477144	BE477144	17	45	45	126	121	114		
2299	4	153815189	153815572	BF440731	BF440731	BF440731	17	51	51	126	121	114		
2301	4	152293790	152293894	BZ854271	BZ854271	BZ854271	17	57	57	126	121	114		
2302	4	151106785	151106882	BZ848461	BZ848461	BZ848461	17	80	80	126	121	114		
2303	4	149912879	149913340	BZ956358	BZ956358	BZ956358	17	88	88	126	121	114		
2304	4	148826091	148826597	CC519101	CC519101	CC519101	17	112	112	126	121	114		
2306	4	148764406	148764858	X57765	X57765	X57765	17	132	132	126	121	114		
2309	4	147894396	147894552	BZ866430	BZ866430	BZ866430	17	152	152	126	121	114		
2310	4	146772097	146772282	BZ839637	BZ839637	BZ839637	17	169	169	126	121	114		
2311	4	145659953	145660279	BZ917545	BZ917545	BZ917545	17	198	198	126	121	114		
2312	4	144637665	144637890	BZ901868	BZ901868	BZ901868	17	207	207	126	121	114		
2313	4	143452361	143452684	BZ925452	BZ925452	BZ925452	17	214	214	126	121	114		
2314	4	142472640	142472762	BZ843304	BZ843304	BZ843304	17	217	217	126	121	114		
2315	4	141831822	141832125	AW289192	AW289192	AW289192	17	217	217	126	121	114		
2316	4	141412120	141412806	CC564369	CC564369	CC564369	17	217	217	126	121	114		
2318	4	140659619	140659830	CC509895	CC509895	CC509895	17	220	220	126	121	114		
2319	4	139358842	139359539	CC526181	CC526181	CC526181	17	220	220	126	121	114		
2320	4	138366779	138367355	BZ869997	BZ869997	BZ869997	17	234	234	126	121	114		
2321	4	137364981	137365436	CC560470	CC560470	CC560470	17	240	240	126	121	114		
2322	4	136323357	136323737	BZ937755	BZ937755	BZ937755	17	254	254	126	121	114		
2323	4	135356788	135357178	BZ875624	BZ875624	BZ875624	17	275	275	126	121	114		
2324	4	134308691	134309036	CC545658	CC545658	CC545658	17	292	292	126	121	114		
2326	4	133290934	133291214	CC594816	CC594816	CC594816	17	307	307	126	121	114		
2327	4	132323299	132323495	CC581354	CC581354	CC581354	17	315	315	126	121	114		

2328	4	131554805	131555230	CC587165	CC587165	CC587165	17	319	319	126	121	114			
2329	4	130626935	130627040	CC471090	CC471090	CC471090	17	319	319	126	121	114			
2330	4	129549153	129549636	BE217446	BE217446	BE217446	17	322	322	126	121	114			
2331	4	128087488	128087735	BZ856434	BZ856434	BZ856434	17	322	322	126	121	114			
2332	4	126865061	126865428	CC577916	CC577916	CC577916	17	326	326	126	121	114			
2333	4	125949470	125950145	BZ943650	BZ943650	BZ943650	17	330	330	126	121	114			
2334	4	124764463	124764624	BZ867931	BZ867931	BZ867931	17	330	330	126	121	114			
2335	4	123730398	123730632	M12791	M12791	M12791	17	330	330	126	121	114			
2336	4	162788815	162789204	CC770472	CC770472	CC770472	17	350	350	127	122	115			
2337	4	161611778	161612345	CC500703	CC500703	CC500703	17	362	362	127	122	115			
2339	4	160519596	160520221	CC499519	CC499519	CC499519	17	377	377	127	122	115			
2340	4	159995769	159995970	L11668	L11668	L11668	17	377	377	127	122	115			
2341	4	159359654	159359843	CC556740	CC556740	CC556740	17	384	384	127	122	115			
2342	4	158496638	158496755	BZ848147	BZ848147	BZ848147	17	397	397	127	122	115			
2343	4	157363142	157363659	BZ951467	BZ951467	BZ951467	17	409	409	127	122	115			
2344	12	131891720	131891844	AW267154	AW267154	AW267154	17	439	439	128	123	116			
2345	12	131220333	131220426	CC529345	CC529345	CC529345	17	446	446	128	123	116			
2346	12	130118154	130118225	BZ836603	BZ836603	BZ836603	17	449	449	128	123	116			
2347	12	129019680	129020040	BZ954528	BZ954528	BZ954528	17	464	464	128	123	116			
2348	12	127992329	127992508	BZ914992	BZ914992	BZ914992	17	493	493	128	123	116			
2349	12	126936765	126936954	CC775508	CC775508	CC775508	17	499	499	128	123	116			
2350	12	125816888	125817142	CC584599	CC584599	CC584599	17	502	502	128	123	116			
2351	12	124899400	124899497	BZ923723	BZ923723	BZ923723	17	505	505	128	123	116			
2353	12	123735712	123735880	CC562433	CC562433	CC562433	17	518	518	128	123	116			
2354	12	122665164	122665289	BZ936180	BZ936180	BZ936180	17	518	518	128	123	116			
2355	12	121539376	121539573	CC517369	CC517369	CC517369	17	527	527	128	123	116			
2356	12	120662222	120662321	CC762885	CC762885	CC762885	17	527	527	128	123	116			
2357	12	119600612	119600962	BZ845959	BZ845959	BZ845959	17	537	537	128	123	116			
2358	12	119225456	119225587	Y00120	Y00120	Y00120	17	540	540	128	123	116			
2359	12	117738092	117738252	BZ929323	BZ929323	BZ929323	17	540	540	128	123	116			
2360	12	116408150	116408366	CC769367	CC769367	CC769367	17	543	543	128	123	116			
2361	12	115437982	115438153	BZ900964	BZ900964	BZ900964	17	546	546	128	123	116			
2362	12	114288073	114288366	BZ890071	BZ890071	BZ890071	17	550	550	128	123	116			
2363	12	113300007	113300339	CC762218	CC762218	CC762218	17	550	550	128	123	116			
2364	12	112173164	112173291	CC499603	CC499603	CC499603	17	553	553	128	123	116			
2365	12	110543631	110544212	AW428602	AW428602	AW428602	17	560	560	128	123	116			
2366	12	109148507	109148881	BZ938971	BZ938971	BZ938971	17	566	566	128	123	116			
2367	12	107990519	107990754	CC771011	CC771011	CC771011	17	569	569	128	123	116			
2368	12	107541351	107541976	AW289349	AW289349	AW289349	17	583	583	128	123	116			
2369	12	107542808	107543271	AW289325	AW289325	AW289325	17	589	589	128	123	116			
2370	12	106880032	106880262	BZ885044	BZ885044	BZ885044	17	589	589	128	123	116			
2371	22	23927568	23927875	AF013259	AF013259	AF013259	17	599	599	129	124	117			
2372	22	24434924	24435091	M73216	M73216	M73216	17	599	599	129	124	117			
2373	22	25787407	25787698	BZ926003	BZ926003	BZ926003	17	609	609	129	124	117			
2374	22	26853133	26853221	CC481950	CC481950	CC481950	17	609	609	129	124	117			
2376	22	27101889	27102271	CC568437	CC568437	CC568437	17	619	619	129	124	117			
2377	22	28053686	28053775	BZ869215	BZ869215	BZ869215	17	619	619	129	124	117			
2378	22	28966284	28968511	U63311	U63311	U63311	17	630	630	129	124	117			
2379	22	29041450	29041594	CC503537	CC503537	CC503537	17	630	630	129	124	117			
2380	22	30003557	30003976	CC583163	CC583163	CC583163	17	641	641	129	124	117			
2381	22	30618004	30618199	BZ861032	BZ861032	BZ861032	17	645	645	129	124	117			
2382	22	30806698	30807186	CC543145	CC543145	CC543145	17	652	652	129	124	117			
2383	22	23292334	23292785	CC476601	CC476601	CC476601	17	690	690	130	125	118			
2384	22	22450211	22450881	AI184253	AI184253	AI184253	17	697	697	130	125	118			
2386	22	19676069	19677872	NM_006767	NM_006767	NM_006767	17	705	705	130	125	118			
2387	22	20380000	20380119	BZ847359	BZ847359	BZ847359	17	709	709	130	125	118			
2388	22	19566514	19568663	NM_004782	NM_004782	NM_004782	17	709	709	130	125	118			
2389	22	19120908	19121115	BZ924710	BZ924710	BZ924710	17	713	713	130	125	118			
2390	22	18324697	18324916	AW462471	AW462471	AW462471	17	734	734	130	125	118			
2391	22	18497912	18498042	CC534458	CC534458	CC534458	17	738	738	130	125	118			
2392	22	17293033	17293236	NM_005974	NM_005974	NM_005974	17	748	748	130	125	118			
2393	16	68852466	68852613	AW428059	AW428059	AW428059	18	0	0	131	126	119			
2394	16	68923088	68923330	AW425927	AW425927	AW425927	18	0	0	131	126	119			
2395	16	68753784	68753877	AA908013	AA908013	AA908013	18	3	3	131	126	119			
2396	16	73477643	73477972	CC770793	CC770793	CC770793	18	8	8	132	127	120			
2398	16	74245632	74245790	CC564655	CC564655	CC564655	18	20	20	132	127	120			
2399	16	75306620	75306985	BZ943242	BZ943242	BZ943242	18	26	26	132	127	120			
2400	16	76429529	76429694	BZ860652	BZ860652	BZ860652	18	32	32	132	127	120			
2401	16	76621841	76622303	U22298	U22298	U22298	18	38	38	132	127	120			
2402	16	78597864	78597980	CC594487	CC594487	CC594487	18	44	44	132	127	120			
2403	16	79605232	79605331	CC585456	CC585456	CC585456	18	63	63	132	127	120			
2404	16	80377101	80377320	BZ845189	BZ845189	BZ845189	18	73	73	132	127	120			
2406	16	81183286	81183489	BZ877608	BZ877608	BZ877608	18	83	83	132	127	120			
2408	16	82177432	82177771	BZ888720	BZ888720	BZ888720	18	101	101	132	127	120			
2409	16	82741233	82741489	CC573057	CC573057	CC573057	18	108	108	132	127	120			
2410	16	84043064	84043493	CC518201	CC518201	CC518201	18	117	117	132	127	120			
2411	16	84842871	84842990	BZ911650	BZ911650	BZ911650	18	117	117	132	127	120			
2412	16	85980486	85980828	BZ939189	BZ939189	BZ939189	18	124	124	132	127	120			
2413	16	87096033	87096209	CC509619	CC509619	CC509619	18	124	124	132	127	120			
2414	16	88289496	88289587	AW487657	AW487657	AW487657	18	127	127	132	127	120			
2415	19	33853867	33854340	CC767636	CC767636	CC767636	18	131	131	singleton	Singleton	singleton			
2416	16	45260303	45260490	BZ875566	BZ875566	BZ875566	18	134	134	133	128	121			
2418	16	46291182	46291465	AW654244	AW654244	AW654244	18	153	153	133	128	121			
2419	16	47130224	47130279	AW357770	AW357770	AW357770	18	169	169	133	128	121			
2420	16	48199046	48199165	CC478158	CC478158	CC478158	18	177	177	133	128	121			
2421	16	49199687	49199894	CC540093	CC540093	CC540093	18	177	177	133	128	121			
2422	16	50301614	50301929	BZ944003	BZ944003	BZ944003	18	181	181	133	128	121			
2423	16	51352305	51352416	BZ946536	BZ946536	BZ946536	18	201	201	133	128	121			
2424	16	52373611	52373968	CC545626	CC545626	CC545626	18	222	222	133	128	121			
2425	16	53438478	53438778	CC526403	CC526403	CC526403	18	222	222	133	128	121			
2427	16	54096751	54097211	AW659176	AW659176	AW659176	18	226	226	133	128	121			
2428	16	54248102	54248384	X79015	X79015	X79015	18	238	238	133	128	121			
2429	16	54953726	54953916	AW478071	AW478071	AW478071	18	250	250	133	128	121			
2430	16	55708859	55709031	CC559807	CC559807	CC559807	18	258	258	133	128	121			
2431	16	56608716	56608866	BZ925203	BZ925203	BZ925203	18	267	267	133	128	121			
2432	16	57616305	57616419	BZ911989	BZ911989	BZ911989	18	276	276	133	128	121			
2433	16	58344238	58344729	CC552672	CC552672	CC552672	18	289	289	133	128	121			
2434	16	59495608	59496232	BZ944519	BZ944519	BZ944519	18	306	306	133	128	121			
2435	16	60491909	60492322	CC535926	CC535926	CC535926	18	323	323	133	128	121			
2436	16	61474937	61475202	CC470368	CC470368	CC470368	18	323	323	133	128	121			
2437	16	62467888	62467986	CC771586	CC771586	CC771586	18	323	323	133	128	121			
2438	16	63484659	63484866	CC549971	CC549971	CC549971	18	359	359	133	128	121			
2439	16	64458768	64458954	CC505957	CC505957	CC505957	18	362	362	133	128	121			
2441	16	65513360	65513555	AW312225	AW312225	AW312225	18	381	381	133	128	121			
2442	16	66462621	66462876	AW461589	AW461589	AW461589	18	387	387	133	128	121			
2443	16	67614329	67614432	BZ881004	BZ881004	BZ881004	18	390	390	133	128	121			
2444	16	68500473	68501002	CC494141	CC494141	CC494141	18	393	393	133	128	121			
2445	16	72492139	72492346	CC504207	CC504207	CC504207	18	396	396	134	129	122			
2446	16	71431224	71431375	CC761882	CC761882	CC761882	18	402	402	134	129	122			
2447	16	70458237	70458360	CC563319	CC563319	CC563319	18	405	405	134	129	122			
2449	16	69492434	69492637	BZ837565	BZ837565	BZ837565	18	414	414	134	129	122			
2450	19	34864702	34864871	BZ918439	BZ918439	BZ918439	18	417	417	135	130	123			
2451	19	35834240	35834814	CC492774	CC492774	CC492774	18	423	423	135	130	123			
2452	19	36826909	36826971	BZ926713	BZ926713	BZ926713	18	429	429	135	130	123			
2453	19	37860731	37860829	BZ852760	BZ852760	BZ852760	18	429	429	135	130	123			
2454	19	38853261	38853481	BZ840340	BZ840340	BZ840340	18	435	435	135	130	123			
2456	19	39649637	39649759	AW461623	AW461623	AW461623	18	439	439	135	130	123			
2457	19	40348982	40349075	AW289186	AW289186	AW289186	18	442	442	135	130	123			
2458	19	41328940	41329057	J05065	J05065	J05065	18	446	446	135	130	123			
2459	19	41901271	41901850	BZ843163	BZ843163	BZ843163	18	450	450	135	130	123			
2460	19	43163132	43163315	BZ855487	BZ855487	BZ855487	18	460	460	135	130	123			
2461	19	44309477	44309706	CC526028	CC526028	CC526028	18	489	489	135	130	123			
2462	19	44589504	44591398	L42319	L42319	L42319	18	489	489	135	130	123			
2463	19	45391630	45391717	CC550540	CC550540	CC550540	18	501	501	135	130	123			
2464	19	45524063	45524168	CC476810	CC476810	CC476810	18	516	516	135	130	123			
2465	19	46589509	46589738	CC587102	CC587102	CC587102	18	516	516	135	130	123			
2466	19	47605694	47605878	BZ872350	BZ872350	BZ872350	18	538	538	135	130	123			
2467	19	48844879	48845109	S70635	S70635	S70635	18	549	549	135	130	123			
2468	19	49896246	49896399	CC472977	CC472977	CC472977	18	552	552	135	130	123			
2470	19	50721236	50721380	AW289225	AW289225	AW289225	18	563	563	135	130	123			
2471	19	50958383	50958632	BZ946535	BZ946535	BZ946535	18	563	563	135	130	123			
2472	19	52059541	52059761	BZ879794	BZ879794	BZ879794	18	574	574	135	130	123			
2474	19	52945977	52946102	BF440193	BF440193	BF440193	18	596	596	135	130	123			
2476	19	54151261	54151424	U92569	U92569	U92569	18	625	625	135	130	123			
2477	19	54685544	54685646	AW481051	AW481051	AW481051	18	640	640	135	130	123			
2478	19	54738199	54738305	AW462197	AW462197	AW462197	18	647	647	135	130	123			
2480	19	55439285	55439475	CC764170	CC764170	CC764170	18	687	687	135	130	123			
2481	19	56549213	56549376	AW652761	AW652761	AW652761	18	727	727	135	130	123			
2482	19	57055961	57056059	BZ911891	BZ911891	BZ911891	18	745	745	135	130	123			
2483	19	58261721	58261749	CC520566	CC520566	CC520566	18	793	793	135	130	123			
2484	19	59353752	59354231	CC591588	CC591588	CC591588	18	875	875	135	130	123			
2485	19	60895065	60895265	BZ926226	BZ926226	BZ926226	18	875	875	135	130	123			
2486	19	62013771	62013929	AW464096	AW464096	AW464096	18	878	878	135	130	123			
2487	19	60445341	60445513	BZ845186	BZ845186	BZ845186	18	880	880	135	130	123			
2489	19	63506623	63506910	CC529034	CC529034	CC529034	18	900	900	135	130	123			
2490	17	47575940	47576445	BZ953513	BZ953513	BZ953513	19	0	0	136	131	124			
2491	17	47751301	47751372	CC567784	CC567784	CC567784	19	8	8	136	131	124			
2492	17	48305521	48305706	BZ831399	BZ831399	BZ831399	19	10	10	136	131	124			
2493	17	49338894	49339190	CC520239	CC520239	CC520239	19	15	15	136	131	124			
2494	17	50222154	50222552	BZ855112	BZ855112	BZ855112	19	31	31	136	131	124			
2495	17	51294405	51294563	BZ953277	BZ953277	BZ953277	19	60	60	136	131	124			
2496	17	52039137	52039419	BZ831713	BZ831713	BZ831713	19	68	68	136	131	124			
2497	17	53192755	53192861	CC768297	CC768297	CC768297	19	73	73	136	131	124			
2498	17	54259315	54259495	BZ930178	BZ930178	BZ930178	19	84	84	136	131	124			
2499	17	55126040	55126321	AW267139	AW267139	AW267139	19	89	89	136	131	124			
2500	17	55079539	55079809	U31757	U31757	U31757	19	95	95	136	131	124			
2501	17	55248020	55248491	CC555392	CC555392	CC555392	19	97	97	136	131	124			
2502	17	57327265	57327568	CC495528	CC495528	CC495528	19	108	108	136	131	124			
2503	17	56388952	56389250	CC555439	CC555439	CC555439	19	131	131	136	131	124			
2504	17	31933890	31934008	BZ852341	BZ852341	BZ852341	19	151	151	137	132	125			
2505	17	32954736	32954927	CC519940	CC519940	CC519940	19	162	162	137	132	125			
2506	17	30497560	30497786	CC554483	CC554483	CC554483	19	181	181	137	132	125			
2507	17	29670186	29670731	S67956	S67956	S67956	19	194	194	137	132	125			
2508	17	28635236	28635550	CC562949	CC562949	CC562949	19	205	205	137	132	125			
2509	17	27838610	27839983	S73375	S73375	S73375	19	218	218	137	132	125			
2510	17	26638002	26638242	BE217405	BE217405	BE217405	19	232	232	137	132	125		AG is wrong in merging these two blocks, as other blocks comes in between	2 ->1
2511	17	22622137	22622360	CC562628	CC562628	CC562628	19	238	238	138	133	125	AG here		
2512	17	23670445	23670547	CC582187	CC582187	CC582187	19	252	252	138	133	125			
2513	17	24316872	24316996	BZ891168	BZ891168	BZ891168	19	273	273	138	133	125			
2514	17	24901220	24901597	AW477536	AW477536	AW477536	19	293	293	138	133	125			
2515	17	25572821	25573107	CC762824	CC762824	CC762824	19	299	299	138	133	125			
2516	17	955873	955915	AW289314	AW289314	AW289314	19	313	313	139	134	126			
2517	17	1845522	1845921	CC774033	CC774033	CC774033	19	330	330	139	134	126			
2518	17	2531760	2534033	D30615	D30615	D30615	19	340	340	139	134	126			
2519	17	3729157	3729246	BE217423	BE217423	BE217423	19	354	354	139	134	126			
2520	17	3774101	3774260	AW289347	AW289347	AW289347	19	364	364	139	134	126			
2521	17	4016218	4016374	BE217550	BE217550	BE217550	19	382	382	139	134	126			
2522	17	5978989	5979503	CC570791	CC570791	CC570791	19	395	395	139	134	126			
2523	17	4745020	4745265	X02597	X02597	X02597	19	410	410	139	134	126			
2524	17	6882626	6882934	AW464274	AW464274	AW464274	19	416	416	139	134	126			
2525	17	7065503	7065842	CC529913	CC529913	CC529913	19	420	420	139	134	126			
2526	17	7985797	7986206	CC763189	CC763189	CC763189	19	426	426	139	134	126			
2527	17	9044561	9044683	CC485723	CC485723	CC485723	19	442	442	139	134	126			
2528	17	9754778	9755157	AW426868	AW426868	AW426868	19	472	472	139	134	126			
2529	17	10095356	10095490	CC542058	CC542058	CC542058	19	490	490	139	134	126			
2530	17	11104952	11105178	CC517808	CC517808	CC517808	19	514	514	139	134	126			
2531	17	11479822	11480000	CC553120	CC553120	CC553120	19	530	530	139	134	126			
2532	17	12491177	12491379	CC581238	CC581238	CC581238	19	538	538	139	134	126			
2533	17	13331770	13331954	CC584838	CC584838	CC584838	19	543	543	139	134	126			
2534	17	14347038	14347171	BZ935958	BZ935958	BZ935958	19	556	556	139	134	126			
2535	17	15750934	15751000	BZ860649	BZ860649	BZ860649	19	558	558	139	134	126			
2536	17	14636693	14637098	CC545891	CC545891	CC545891	19	561	561	139	134	126			
2537	17	16468239	16468664	BZ869086	BZ869086	BZ869086	19	566	566	139	134	126		AG is wrong in merging these two blocks, as other blocks comes in between	2 -> 1
2538	17	19934450	19934817	CC594649	CC594649	CC594649	19	573	573	140	135	126	AG here		
2539	17	18821605	18821887	CC505122	CC505122	CC505122	19	597	597	140	135	126			
2540	17	17602434	17602617	BZ904625	BZ904625	BZ904625	19	616	616	140	135	126			
2541	17	20936081	20936208	BZ896574	BZ896574	BZ896574	19	628	628	singleton	Singleton	126	AG here	AG is wrong in merging this marker with the top block	
2542	17	46467871	46468227	CC517765	CC517765	CC517765	19	636	636	141	136	127			
2543	17	45338776	45339197	CC514638	CC514638	CC514638	19	638	638	141	136	127			
2544	17	44400975	44401212	CC541699	CC541699	CC541699	19	650	650	141	136	127			
2545	17	43542724	43543006	BZ889712	BZ889712	BZ889712	19	658	658	141	136	127			
2546	17	43054307	43054915	AW289329	AW289329	AW289329	19	665	665	141	136	127			
2547	17	33962552	33962646	BZ886550	BZ886550	BZ886550	19	675	675	142	137	128			
2548	17	34981894	34982415	CC582934	CC582934	CC582934	19	679	679	142	137	128			
2549	17	35866162	35866430	CC535989	CC535989	CC535989	19	691	691	142	137	128			
2550	17	36055852	36056019	CC483278	CC483278	CC483278	19	696	696	142	137	128			
2551	17	37324395	37324454	BZ887864	BZ887864	BZ887864	19	711	711	142	137	128			
2552	17	38168256	38168538	AW484551	AW484551	AW484551	19	715	715	142	137	128			
2553	17	38704493	38704670	BZ934550	BZ934550	BZ934550	19	715	715	142	137	128			
2554	17	39256815	39256881	CC490787	CC490787	CC490787	19	753	753	142	137	128			
2555	17	40249056	40249381	CC765011	CC765011	CC765011	19	794	794	142	137	128			
2556	17	41303883	41304134	BZ941836	BZ941836	BZ941836	19	800	800	142	137	128			
2557	17	42195806	42196282	CC565877	CC565877	CC565877	19	807	807	142	137	128			
2558	17	58322198	58322356	BZ834021	BZ834021	BZ834021	19	820	820	143	138	129			
2559	17	59244454	59244626	BZ874992	BZ874992	BZ874992	19	824	824	143	138	129			
2560	17	60041264	60041845	CC585529	CC585529	CC585529	19	829	829	143	138	129		AG is wrong in merging this marker with the top block	
2561	17	62799302	62799585	CC505793	CC505793	CC505793	19	834	834	out-of-place	out-of-place	129	AG here		
2562	17	78421388	78421542	U61233	U61233	U61233	19	843	843	144	139	130			
2563	17	77230593	77230793	X04823	X04823	X04823	19	869	869	144	139	130			
2564	17	76265643	76265794	BZ939473	BZ939473	BZ939473	19	891	891	144	139	130			
2565	17	74709530	74709950	CC471900	CC471900	CC471900	19	916	916	144	139	130			
2566	17	73681693	73695190	M15205	M15205	M15205	19	964	964	144	139	130			
2567	17	73074649	73075046	BZ833454	BZ833454	BZ833454	19	976	976	144	139	130			
2568	17	72241197	72241446	BE217412	BE217412	BE217412	19	987	987	144	139	130			
2569	17	71207421	71207572	CC593430	CC593430	CC593430	19	1004	1004	144	139	130			
2570	17	70304887	70305161	CC523006	CC523006	CC523006	19	1016	1016	144	139	130			
2571	17	69387285	69387413	CC764696	CC764696	CC764696	19	1028	1028	144	139	130			
2572	17	68169147	68169319	CC479797	CC479797	CC479797	19	1060	1060	144	139	130			
2573	17	67774137	67774567	BZ860972	BZ860972	BZ860972	19	1065	1065	144	139	130			
2574	17	67108400	67108626	BZ901552	BZ901552	BZ901552	19	1073	1073	144	139	130			
2575	17	66077440	66077580	CC470018	CC470018	CC470018	19	1089	1089	144	139	130			
2576	17	65282929	65283411	CC519138	CC519138	CC519138	19	1096	1096	144	139	130			
2577	17	64943776	64943886	BZ945658	BZ945658	BZ945658	19	1111	1111	144	139	130			
2578	17	63942281	63942389	CC770324	CC770324	CC770324	19	1129	1129	144	139	130			
2579	17	61983766	61984227	CC493878	CC493878	CC493878	19	1137	1137	144	139	130			
2580	17	61641003	61641232	AW345161	AW345161	AW345161	19	1146	1146	144	139	130			
2581	17	60960543	60960701	BZ869478	BZ869478	BZ869478	19	1156	1156	144	139	130			
2583	5	168139001	168139274	BZ915169	BZ915169	BZ915169	20	10	10	145	140	131			
2584	5	169122223	169122545	BZ923420	BZ923420	BZ923420	20	19	19	145	140	131			

2585	5	170156927	170157178	BZ883716	BZ883716	BZ883716	20	25	25	145	140	131
2586	5	171202118	171202421	CC535673	CC535673	CC535673	20	38	38	145	140	131
2587	5	172291964	172292159	BZ870995	BZ870995	BZ870995	20	50	50	145	140	131
2588	5	172471873	172472135	BZ849143	BZ849143	BZ849143	20	50	50	145	140	131
2589	5	172845862	172846177	CC770008	CC770008	CC770008	20	53	53	145	140	131
2591	5	173292015	173292081	CC532530	CC532530	CC532530	20	70	70	145	140	131
2593	5	74049828	74049909	AW289200	AW289200	AW289200	20	99	99	146	141	132
2594	5	72986406	72986891	BZ927566	BZ927566	BZ927566	20	102	102	146	141	132
2595	5	71881750	71881999	BZ861675	BZ861675	BZ861675	20	106	106	146	141	132
2596	5	70888475	70888526	CC500233	CC500233	CC500233	20	106	106	146	141	132
2597	5	70273939	70274137	AF035323	AF035323	AF035323	20	109	109	146	141	132
2598	5	68750912	68751128	BZ880812	BZ880812	BZ880812	20	116	116	146	141	132
2599	5	67584161	67584715	BZ879837	BZ879837	BZ879837	20	119	119	146	141	132
2600	5	66580399	66580705	CC482374	CC482374	CC482374	20	123	123	146	141	132
2601	5	65542468	65542576	CC476290	CC476290	CC476290	20	138	138	146	141	132
2602	5	65511666	65512472	AW289180	AW289180	AW289180	20	138	138	146	141	132
2603	5	64533041	64533352	CC523832	CC523832	CC523832	20	145	145	146	141	132
2604	5	63497077	63497408	CC566529	CC566529	CC566529	20	157	157	146	141	132
2605	5	62405714	62406342	CC529092	CC529092	CC529092	20	161	161	146	141	132
2606	5	61801692	61802055	CC563347	CC563347	CC563347	20	169	169	146	141	132
2607	5	61722450	61722560	AW289315	AW289315	AW289315	20	177	177	146	141	132
2608	5	60988323	60988491	CC586997	CC586997	CC586997	20	184	184	146	141	132
2610	5	59978938	59979198	CC533683	CC533683	CC533683	20	195	195	146	141	132
2611	5	59053325	59054001	CC501841	CC501841	CC501841	20	199	199	146	141	132
2612	5	58251252	58251971	CC488301	CC488301	CC488301	20	210	210	146	141	132
2613	5	57157627	57157781	CC566568	CC566568	CC566568	20	220	220	146	141	132
2614	5	56191236	56191587	CC472921	CC472921	CC472921	20	220	220	146	141	132
2615	5	55095567	55095657	CC595532	CC595532	CC595532	20	223	223	146	141	132
2616	5	54039475	54039782	CC542419	CC542419	CC542419	20	226	226	146	141	132
2617	5	53217339	53217512	AW244894	AW244894	AW244894	20	230	230	146	141	132
2618	5	52809869	52810128	CC774706	CC774706	CC774706	20	233	233	146	141	132
2619	5	52387564	52387739	L25886	L25886	L25886	20	233	233	146	141	132
2621	5	51877403	51877925	CC526556	CC526556	CC526556	20	244	244	146	141	132
2622	5	50892409	50892537	CC585943	CC585943	CC585943	20	248	248	146	141	132
2623	5	49676201	49676297	CC521543	CC521543	CC521543	20	260	260	146	141	132
2624	5	45865417	45865743	CC488096	CC488096	CC488096	20	275	275	146	141	132
2625	5	45639610	45639836	BZ930455	BZ930455	BZ930455	20	278	278	146	141	132
2626	5	44603652	44603922	CC545088	CC545088	CC545088	20	294	294	146	141	132
2627	5	43691698	43691936	L02543	L02543	L02543	20	309	309	146	141	132
2628	5	42835975	42836450	D25220	D25220	D25220	20	320	320	146	141	132
2629	5	41841384	41841625	BZ939302	BZ939302	BZ939302	20	330	330	146	141	132
2630	5	40803294	40803698	CC572194	CC572194	CC572194	20	330	330	146	141	132
2631	5	39791738	39792359	CC525812	CC525812	CC525812	20	333	333	146	141	132
2632	5	38967663	38967899	BZ934737	BZ934737	BZ934737	20	357	357	146	141	132
2633	5	37868956	37869027	BZ915127	BZ915127	BZ915127	20	357	357	146	141	132
2634	5	37038912	37039253	CC505115	CC505115	CC505115	20	374	374	146	141	132
2635	5	35869562	35869712	BZ886635	BZ886635	BZ886635	20	383	383	146	141	132
2636	5	34858790	34858949	BZ866925	BZ866925	BZ866925	20	387	387	146	141	132
2637	5	33841801	33841935	CC596142	CC596142	CC596142	20	397	397	146	141	132
2638	5	32850639	32850784	BZ879841	BZ879841	BZ879841	20	425	425	146	141	132
2639	5	31773408	31773671	CC510228	CC510228	CC510228	20	447	447	146	141	132
2640	5	30668667	30669180	CC587609	CC587609	CC587609	20	464	464	146	141	132
2641	5	29608752	29609339	BZ931717	BZ931717	BZ931717	20	478	478	146	141	132
2643	5	28399104	28399418	CC488860	CC488860	CC488860	20	492	492	146	141	132
2644	5	27167900	27168200	CC567251	CC567251	CC567251	20	505	505	146	141	132
2645	5	26217446	26217799	CC553299	CC553299	CC553299	20	509	509	146	141	132
2646	5	24867009	24867105	CC524554	CC524554	CC524554	20	533	533	146	141	132
2647	5	24547129	24547383	AF013085	AF013085	AF013085	20	540	540	146	141	132
2648	5	23853707	23853883	BZ914829	BZ914829	BZ914829	20	543	543	146	141	132
2649	5	22535922	22536260	CC493750	CC493750	CC493750	20	564	564	146	141	132
2650	5	21449012	21449156	CC520996	CC520996	CC520996	20	590	590	146	141	132
2651	5	20268165	20268203	BZ846099	BZ846099	BZ846099	20	600	600	146	141	132
2652	5	19264976	19265121	CC501934	CC501934	CC501934	20	607	607	146	141	132
2653	5	18279173	18279496	BZ927231	BZ927231	BZ927231	20	614	614	146	141	132
2654	5	16087163	16087598	BZ846201	BZ846201	BZ846201	20	627	627	146	141	132
2655	5	17235973	17236083	BZ902773	BZ902773	BZ902773	20	637	637	146	141	132
2657	5	13967562	13967913	CC578133	CC578133	CC578133	20	675	675	146	141	132
2658	5	12943668	12943918	CC556510	CC556510	CC556510	20	683	683	146	141	132
2659	5	11814137	11814709	CC560395	CC560395	CC560395	20	699	699	146	141	132
2661	5	10581252	10581624	BZ926035	BZ926035	BZ926035	20	724	724	146	141	132
2662	5	9509549	9509961	BZ954162	BZ954162	BZ954162	20	737	737	146	141	132
2663	5	8606703	8606843	CC481538	CC481538	CC481538	20	752	752	146	141	132
2664	5	7573878	7574070	CC541236	CC541236	CC541236	20	752	752	146	141	132
2665	5	6307578	6307866	CC564722	CC564722	CC564722	20	760	760	146	141	132
2666	5	5500535	5500609	CC479930	CC479930	CC479930	20	767	767	146	141	132
2668	5	4418934	4419177	CC771203	CC771203	CC771203	20	775	775	146	141	132
2669	5	3507587	3507859	CC503913	CC503913	CC503913	20	775	775	146	141	132
2670	5	2243951	2244261	CC573749	CC573749	CC573749	20	783	783	146	141	132
2671	5	528714	528800	BZ902065	BZ902065	BZ902065	20	814	814	146	141	132
2672	15	21625068	21625376	BZ932701	BZ932701	BZ932701	21	0	0	147	142	133
2673	15	22793991	22794601	AW289286	AW289286	AW289286	21	3	3	147	142	133
2674	15	22934693	22934846	CC492289	CC492289	CC492289	21	3	3	147	142	133
2676	15	23202684	23202987	BZ888341	BZ888341	BZ888341	21	3	3	147	142	133
2677	15	99634268	99634444	BZ834030	BZ834030	BZ834030	21	119	119	148	143	134
2678	15	97285245	97285329	U33122	U33122	U33122	21	143	143	148	143	134
2679	15	96315923	96316408	BZ881462	BZ881462	BZ881462	21	151	151	148	143	134
2680	15	95322983	95323317	BZ861796	BZ861796	BZ861796	21	155	155	148	143	134
2682	15	94350703	94350776	BZ935275	BZ935275	BZ935275	21	155	155	148	143	134
2683	15	93232046	93232335	BZ953292	BZ953292	BZ953292	21	163	163	148	143	134
2684	15	92278637	92278879	CC588559	CC588559	CC588559	21	166	166	148	143	134
2685	15	91297594	91297902	CC469826	CC469826	CC469826	21	170	170	148	143	134
2686	15	90131490	90131598	BZ889745	BZ889745	BZ889745	21	187	187	148	143	134
2687	15	85018425	85018917	CC477951	CC477951	CC477951	21	187	187	149	144	deleted
2688	15	84060006	84060166	CC535938	CC535938	CC535938	21	191	191	149	144	135
2689	15	86093204	86093320	CC471417	CC471417	CC471417	21	211	211	149	144	135
2690	15	87243618	87243769	S80643	S80643	S80643	21	216	216	149	144	135
2691	15	88081754	88081999	CC563473	CC563473	CC563473	21	220	220	149	144	135
2692	15	89093812	89094417	CC505228	CC505228	CC505228	21	225	225	149	144	135
2693	15	89225485	89226421	X75956	X75956	X75956	21	229	229	149	144	135
2694	15	83066113	83066176	CC557163	CC557163	CC557163	21	253	253	150	145	136
2695	15	81176930	81177142	CC559273	CC559273	CC559273	21	268	268	150	145	136
2696	15	82167893	82168219	CC590948	CC590948	CC590948	21	273	273	150	145	136
2697	15	79957737	79958158	CC530547	CC530547	CC530547	21	273	273	150	145	136
2698	15	77143814	77144036	CC588410	CC588410	CC588410	21	277	277	150	145	136
2699	15	78898402	78898793	CC526762	CC526762	CC526762	21	281	281	150	145	136
2700	15	78050077	78050429	AW289296	AW289296	AW289296	21	281	281	150	145	136
2701	15	27348345	27348608	AW357228	AW357228	AW357228	21	286	286	151	146	137
2702	15	27074418	27075038	CC593376	CC593376	CC593376	21	290	290	151	146	137
2705	15	28035466	28035937	CC474727	CC474727	CC474727	21	303	303	151	146	137
2706	15	29022246	29022571	BZ872596	BZ872596	BZ872596	21	316	316	151	146	137
2708	15	30205341	30205479	CC561692	CC561692	CC561692	21	346	346	151	146	137
2709	15	29554923	29555276	AW487183	AW487183	AW487183	21	355	355	151	146	137
2710	15	76248475	76249007	U07980	U07980	U07980	21	359	359	152	147	138
2711	15	74919835	74920142	CC582906	CC582906	CC582906	21	374	374	152	147	138
2712	15	75894314	75894671	BZ909371	BZ909371	BZ909371	21	379	379	152	147	138
2713	15	73686458	73686529	CC581008	CC581008	CC581008	21	379	379	152	147	138
2714	15	72646338	72646642	CC482179	CC482179	CC482179	21	384	384	152	147	138
2715	15	72923680	72923773	AW289207	AW289207	AW289207	21	388	388	152	147	138
2719	14	26931718	26932020	BZ911207	BZ911207	BZ911207	21	421	421	153	148	139
2720	14	27997744	27997927	CC504245	CC504245	CC504245	21	434	434	153	148	139
2721	14	29056091	29056256	BZ856723	BZ856723	BZ856723	21	446	446	153	148	139
2722	14	29984869	29985137	CC569928	CC569928	CC569928	21	454	454	153	148	139
2723	14	30419546	30419725	AW446703	AW446703	AW446703	21	465	465	153	148	139
2724	14	31084728	31084798	BZ866194	BZ866194	BZ866194	21	477	477	153	148	139
2725	14	31696295	31696768	AW315952	AW315952	AW315952	21	500	500	153	148	139
2726	14	32135831	32135957	BZ851054	BZ851054	BZ851054	21	500	500	153	148	139
2727	14	33087187	33087298	BZ922331	BZ922331	BZ922331	21	512	512	153	148	139
2728	14	34319030	34319289	BZ841650	BZ841650	BZ841650	21	523	523	153	148	139
2729	14	34941532	34941624	BF440215	BF440215	BF440215	21	527	527	153	148	139
2730	14	35106030	35106285	CC546482	CC546482	CC546482	21	531	531	153	148	139
2731	14	37086330	37086684	CC569497	CC569497	CC569497	21	535	535	153	148	139
2732	14	35837924	35838144	AW356421	AW356421	AW356421	21	543	543	153	148	139
2733	14	36131286	36131401	CC513853	CC513853	CC513853	21	552	552	153	148	139
2734	14	38087409	38087734	CC774729	CC774729	CC774729	21	565	565	153	148	139
2735	14	38571175	38571493	AW354499	AW354499	AW354499	21	565	565	153	148	139
2737	14	39668839	39668987	CC537855	CC537855	CC537855	21	581	581	153	148	139
2738	14	40680884	40681308	BZ871863	BZ871863	BZ871863	21	581	581	153	148	139
2739	14	41443109	41443233	BZ900244	BZ900244	BZ900244	21	585	585	153	148	139
2740	14	42241740	42242311	CC502912	CC502912	CC502912	21	589	589	153	148	139
2741	14	43429333	43429586	CC520031	CC520031	CC520031	21	589	589	153	148	139
2742	14	44582543	44582695	BZ951156	BZ951156	BZ951156	21	589	589	153	148	139
2743	14	44355828	44355857	BZ913867	BZ913867	BZ913867	21	598	598	153	148	139
2744	15	41850595	41850937	D16235	D16235	D16235	21	610	610	154	149	140
2745	15	42033144	42033197	CC563964	CC563964	CC563964	21	610	610	154	149	140
2746	15	42174195	42174459	CC534820	CC534820	CC534820	21	614	614	154	149	140
2747	15	42376114	42376242	CC588995	CC588995	CC588995	21	614	614	154	149	140
2748	14	90994352	90994814	AW289256	AW289256	AW289256	21	623	623	155	150	141
2749	14	91265471	91265560	CC771259	CC771259	CC771259	21	632	632	155	150	141
2750	14	92468477	92469014	U73523	U73523	U73523	21	632	632	155	150	141
2752	14	93123337	93123606	CC586015	CC586015	CC586015	21	632	632	155	150	141
2753	14	94150625	94150801	CC479955	CC479955	CC479955	21	640	640	155	150	141
2754	14	94925698	94926040	CC509062	CC509062	CC509062	21	640	640	155	150	141
2755	14	95992359	95992638	BZ855724	BZ855724	BZ855724	21	640	640	155	150	141
2756	14	97049028	97049354	CC596423	CC596423	CC596423	21	640	640	155	150	141
2760	14	98041492	98042182	CC517873	CC517873	CC517873	21	640	640	155	150	141
2761	14	98999605	98999838	BZ933100	BZ933100	BZ933100	21	644	644	155	150	141
2762	14	99938191	99938360	CC559999	CC559999	CC559999	21	653	653	155	150	141
2763	14	100270621	100271105	AB009278	AB009278	AB009278	21	653	653	155	150	141
2765	14	100756929	100757291	CC590935	CC590935	CC590935	21	653	653	155	150	141
2766	14	101572561	101572745	CC774331	CC774331	CC774331	21	657	657	155	150	141
2767	14	102673421	102673554	BZ918242	BZ918242	BZ918242	21	669	669	155	150	141
2768	14	103640374	103640479	CC542063	CC542063	CC542063	21	673	673	155	150	141
2769	14	104310262	104310476	X61036	X61036	X61036	21	673	673	155	150	141
2770	14	105391488	105391837	BZ900741	BZ900741	BZ900741	21	673	673	155	150	141
2771	3	27733544	27733923	AW445450	AW445450	AW445450	22	0	0	156	151	142
2773	3	28679221	28679634	BZ920613	BZ920613	BZ920613	22	18	18	156	151	142
2774	3	30690382	30690761	BZ956264	BZ956264	BZ956264	22	20	20	156	151	142
2775	3	30777861	30778154	BZ946107	BZ946107	BZ946107	22	25	25	156	151	142
2776	3	29598881	29599060	BZ898745	BZ898745	BZ898745	22	33	33	156	151	142
2777	3	31516158	31516288	BZ927256	BZ927256	BZ927256	22	49	49	156	151	142
2778	3	32175325	32175599	AW652674	AW652674	AW652674	22	62	62	156	151	142
2779	3	32969871	32970219	AW631752	AW631752	AW631752	22	73	73	156	151	142
2780	3	37695571	37695615	BE217575	BE217575	BE217575	22	88	88	156	151	142
2781	3	35538176	35538611	BZ932684	BZ932684	BZ932684	22	105	105	156	151	142
2782	3	34179193	34179408	BZ952051	BZ952051	BZ952051	22	117	117	156	151	142
2783	3	36750115	36750572	BZ906231	BZ906231	BZ906231	22	122	122	156	151	142
2784	3	37267886	37267979	AW358332	AW358332	AW358332	22	128	128	156	151	142
2785	3	37695571	37695615	BZ932541	BZ932541	BZ932541	22	141	141	156	151	142
2786	3	38499632	38499900	U57707	U57707	U57707	22	147	147	156	151	142
2787	3	38807874	38807973	BZ910983	BZ910983	BZ910983	22	157	157	156	151	142
2788	3	40328463	40328844	AW289354	AW289354	AW289354	22	167	167	156	151	142
2790	3	42294039	42294491	CC478087	CC478087	CC478087	22	203	203	156	151	142
2791	3	42569694	42569990	BZ931203	BZ931203	BZ931203	22	208	208	156	151	142
2792	3	43143061	43143680	CC559587	CC559587	CC559587	22	214	214	156	151	142
2793	3	44227969	44228475	BZ852869	BZ852869	BZ852869	22	219	219	156	151	142
2794	3	9283364	9283687	BZ917720	BZ917720	BZ917720	22	239	239	157	152	143
2795	3	8584138	8584438	BZ909150	BZ909150	BZ909150	22	253	253	157	152	143
2796	3	7494772	7495040	BZ906986	BZ906986	BZ906986	22	261	261	157	152	143
2797	3	6250403	6250707	BZ937713	BZ937713	BZ937713	22	266	266	157	152	143
2799	3	5218676	5219360	BZ944420	BZ944420	BZ944420	22	282	282	157	152	143
2800	3	4863357	4863815	AW658776	AW658776	AW658776	22	282	282	157	152	143
2801	3	4412019	4412451	BZ929545	BZ929545	BZ929545	22	288	288	157	152	143
2802	3	3389060	3389469	BZ924764	BZ924764	BZ924764	22	297	297	157	152	143
2803	3	2128835	2129060	BZ917496	BZ917496	BZ917496	22	313	313	157	152	143
2804	3	1219384	1219922	BZ912422	BZ912422	BZ912422	22	313	313	157	152	143
2805	3	75349198	75349489	CC587730	CC587730	CC587730	22	322	322	158	153	144
2806	3	74758420	74758899	CC477997	CC477997	CC477997	22	332	332	158	153	144
2807	3	74309335	74309926	BZ947177	BZ947177	BZ947177	22	344	344	158	153	144
2808	3	73430087	73430457	BZ938739	BZ938739	BZ938739	22	347	347	158	153	144
2809	3	72336166	72336581	BZ938134	BZ938134	BZ938134	22	350	350	158	153	144
2810	3	71553019	71553410	BZ943359	BZ943359	BZ943359	22	368	368	158	153	144
2811	3	70186372	70186509	BZ912312	BZ912312	BZ912312	22	380	380	158	153	144
2812	3	69186574	69187037	AW289243	AW289243	AW289243	22	383	383	158	153	144
2814	3	66884534	66884847	CC548073	CC548073	CC548073	22	400	400	158	153	144
2815	3	68039497	68039736	BZ928999	BZ928999	BZ928999	22	403	403	158	153	144
2816	3	66510803	66510998	U31301	U31301	U31301	22	406	406	158	153	144
2817	3	65326367	65326561	BZ907944	BZ907944	BZ907944	22	431	431	158	153	144
2818	3	63983922	63984215	CC530859	CC530859	CC530859	22	450	450	158	153	144
2819	3	64054588	64054843	AW652688	AW652688	AW652688	22	454	454	158	153	144
2820	3	62883806	62883923	CC479847	CC479847	CC479847	22	475	475	158	153	144
2821	3	62970444	62970624	BZ951069	BZ951069	BZ951069	22	479	479	158	153	144
2822	3	61754961	61755342	BZ864001	BZ864001	BZ864001	22	490	490	158	153	144
2823	3	60851588	60851909	BZ930324	BZ930324	BZ930324	22	497	497	158	153	144
2825	3	59883135	59883650	BZ939442	BZ939442	BZ939442	22	515	515	158	153	144
2826	3	58861067	58861228	BZ904182	BZ904182	BZ904182	22	515	515	158	153	144
2827	3	57971203	57971640	CC542842	CC542842	CC542842	22	525	525	158	153	144
2828	3	57099050	57099343	AW289230	AW289230	AW289230	22	525	525	158	153	144
2829	3	56741246	56741470	CC520072	CC520072	CC520072	22	525	525	158	153	144
2830	3	56702269	56702347	BZ939171	BZ939171	BZ939171	22	525	525	158	153	144
2831	3	55634836	55635287	BZ904337	BZ904337	BZ904337	22	547	547	158	153	144
2832	3	54909935	54910298	CC493752	CC493752	CC493752	22	551	551	158	153	144
2833	3	54062218	54062385	BZ947836	BZ947836	BZ947836	22	551	551	158	153	144
2834	3	53171052	53171236	BZ948417	BZ948417	BZ948417	22	563	563	158	153	144
2835	3	52220518	52221012	CC473696	CC473696	CC473696	22	575	575	158	153	144
2836	3	51362691	51362893	BZ910022	BZ910022	BZ910022	22	586	586	158	153	144
2837	3	51978208	51978391	BZ942808	BZ942808	BZ942808	22	604	604	158	153	144
2838	3	50207201	50207404	X02440	X02440	X02440	22	633	633	158	153	144
2839	3	49543232	49545636	AB009079	AB009079	AB009079	22	643	643	158	153	144
2840	3	48758915	48759075	BZ926243	BZ926243	BZ926243	22	657	657	158	153	144
2841	3	47789003	47789565	BZ905246	BZ905246	BZ905246	22	671	671	158	153	144
2842	3	45991691	45992014	BZ880647	BZ880647	BZ880647	22	680	680	158	153	144
2843	3	46218455	46218522	AW428591	AW428591	AW428591	22	680	680	158	153	144
2844	3	45760269	45760484	AW462454	AW462454	AW462454	22	684	684	158	153	144
2845	3	46472361	46472461	L24753	L24753	L24753	22	692	692	158	153	144
2846	3	45381419	45381502	BZ933775	BZ933775	BZ933775	22	705	705	158	153	144
2847	3	10422464	10422581	BZ936552	BZ936552	BZ936552	22	721	721	159	154	145
2848	3	11748796	11748859	BZ916680	BZ916680	BZ916680	22	725	725	159	154	145
2849	3	14384650	14384790	CC556313	CC556313	CC556313	22	763	763	159	154	145
2850	3	13002008	13002120	BZ913002	BZ913002	BZ913002	22	767	767	159	154	145

2851	3	13402808	13402909	BZ939105	BZ939105	BZ939105	22	770	770	159	154	145			
2852	3	130467088	130467334	X70019	X70019	X70019	22	789	789	160	155	146			
2853	3	128810373	128810564	AW354958	AW354958	AW354958	22	810	810	160	155	146			
2854	3	128200383	128200648	CC485838	CC485838	CC485838	22	819	819	160	155	146			
2855	6	54438089	54438228	CC554778	CC554778	CC554778	23	0	0	161	156	147			
2856	6	55411905	55412338	BZ896558	BZ896558	BZ896558	23	7	7	161	156	147			
2858	6	56437228	56437652	CC553822	CC553822	CC553822	23	22	22	161	156	147			
2859	6	56999925	57000067	AW266887	AW266887	AW266887	23	32	32	161	156	147			
2860	6	57466680	57466889	CC496629	CC496629	CC496629	23	37	37	161	156	147			
2861	6	58439018	58439084	BZ946213	BZ946213	BZ946213	23	39	39	161	156	147			
2862	6	62745778	62746183	BZ930083	BZ930083	BZ930083	23	81	81	161	156	147			
2863	6	33364799	33365561	CC591860	CC591860	CC591860	23	108	108	162	157	148			
2864	6	34177497	34177661	CC520595	CC520595	CC520595	23	108	108	162	157	148			
2865	6	35192318	35192808	BZ952963	BZ952963	BZ952963	23	110	110	162	157	148			
2866	6	36095234	36095402	BZ852523	BZ852523	BZ852523	23	116	116	162	157	148			
2867	6	37097212	37097400	BZ866173	BZ866173	BZ866173	23	130	130	162	157	148			
2869	6	38149103	38149195	BZ925525	BZ925525	BZ925525	23	136	136	162	157	148			
2870	6	38996981	38997235	BZ888689	BZ888689	BZ888689	23	136	136	162	157	148			
2871	6	39964344	39964554	BZ923098	BZ923098	BZ923098	23	157	157	162	157	148			
2872	6	41108493	41109164	CC588514	CC588514	CC588514	23	171	171	162	157	148			
2873	6	42142111	42142208	BZ846044	BZ846044	BZ846044	23	174	174	162	157	148			
2874	6	43236797	43237130	BZ919541	BZ919541	BZ919541	23	189	189	162	157	148			
2876	6	44338180	44338454	CC527624	CC527624	CC527624	23	219	219	162	157	148			
2877	6	45150878	45151526	CC769602	CC769602	CC769602	23	224	224	162	157	148			
2878	6	46237352	46237509	CC492532	CC492532	CC492532	23	226	226	162	157	148			
2879	6	47203219	47203351	BZ948608	BZ948608	BZ948608	23	237	237	162	157	148			
2880	6	48281512	48282220	CC489788	CC489788	CC489788	23	245	245	162	157	148			
2881	6	50687100	50687321	CC526543	CC526543	CC526543	23	263	263	162	157	148			
2882	6	49591285	49591483	BZ922724	BZ922724	BZ922724	23	275	275	162	157	148			
2883	6	51620781	51620877	CC564262	CC564262	CC564262	23	287	287	162	157	148			
2884	6	52248876	52249322	AW289423	AW289423	AW289423	23	293	293	162	157	148			
2885	6	52452396	52452536	AW417159	AW417159	AW417159	23	293	293	162	157	148			
2886	6	53240349	53240606	AW347590	AW347590	AW347590	23	293	293	162	157	148			
2887	6	32659874	32660116	U90922	U90922	U90922	23	295	295	163	158	149			
2888	6	31651047	31654371	AF011926	AF011926	AF011926	23	301	301	163	158	149			
2889	6	32259295	32259509	M91212	M91212	M91212	23	307	307	163	158	149			
2890	6	31062016	31062398	CC770129	CC770129	CC770129	23	325	325	163	158	149			
2891	6	30171413	30171656	CC502313	CC502313	CC502313	23	356	356	163	158	149			
2892	6	29290602	29291027	BZ873607	BZ873607	BZ873607	23	356	356	163	158	149			
2893	6	28128995	28129125	CC774095	CC774095	CC774095	23	356	356	163	158	149			
2894	6	27475655	27475806	BZ868300	BZ868300	BZ868300	23	359	359	163	158	149			
2895	6	26227216	26227430	CC523412	CC523412	CC523412	23	359	359	163	158	149			
2896	6	26020843	26021102	CC521866	CC521866	CC521866	23	359	359	163	158	149			
2897	6	24758972	24759066	CC469680	CC469680	CC469680	23	371	371	163	158	149			
2898	6	23674686	23675097	BZ879663	BZ879663	BZ879663	23	375	375	163	158	149			
2899	6	23353358	23353412	BZ942535	BZ942535	BZ942535	23	375	375	163	158	149			
2900	6	22359712	22359990	CC549051	CC549051	CC549051	23	379	379	163	158	149			
2902	6	21306201	21306372	CC506269	CC506269	CC506269	23	387	387	163	158	149			
2903	6	20208878	20209235	CC475203	CC475203	CC475203	23	408	408	163	158	149			
2904	6	19469047	19469202	CC475636	CC475636	CC475636	23	411	411	163	158	149			
2905	6	18407128	18407395	BZ851620	BZ851620	BZ851620	23	415	415	163	158	149			
2906	6	17320604	17321016	CC579498	CC579498	CC579498	23	423	423	163	158	149			
2907	6	16302126	16302393	CC530122	CC530122	CC530122	23	426	426	163	158	149			
2908	6	15256921	15257018	CC507661	CC507661	CC507661	23	430	430	163	158	149			
2909	6	14374545	14374895	CC775678	CC775678	CC775678	23	430	430	163	158	149			
2910	6	13175666	13175761	BZ842044	BZ842044	BZ842044	23	441	441	163	158	149			
2912	6	12089544	12089757	BZ881180	BZ881180	BZ881180	23	457	457	163	158	149			
2913	6	11103185	11103547	CC548051	CC548051	CC548051	23	465	465	163	158	149			
2914	6	10112667	10112928	CC773631	CC773631	CC773631	23	465	465	163	158	149			
2915	6	9064357	9064943	BZ883137	BZ883137	BZ883137	23	477	477	163	158	149			
2916	6	8114693	8114859	CC554716	CC554716	CC554716	23	477	477	163	158	149			
2917	6	7101837	7101996	CC568662	CC568662	CC568662	23	489	489	163	158	149			
2918	6	6189638	6189930	CC513564	CC513564	CC513564	23	496	496	163	158	149			
2919	6	5336919	5337245	BZ886461	BZ886461	BZ886461	23	500	500	163	158	149			
2920	6	4215719	4216273	CC518276	CC518276	CC518276	23	507	507	163	158	149			
2921	6	3485310	3485543	CC477026	CC477026	CC477026	23	510	510	163	158	149			
2922	6	2899814	2899958	D55670	D55670	D55670	23	524	524	163	158	149			
2923	6	2568201	2568330	CC590141	CC590141	CC590141	23	534	534	163	158	149			
2924	6	1562146	1562324	CC507621	CC507621	CC507621	23	541	541	163	158	149			
2925	6	341176	341407	BZ901656	BZ901656	BZ901656	23	544	544	163	158	149			
2926	18	75083552	75083952	BZ856100	BZ856100	BZ856100	24	0	0	164	159	150			
2927	18	75486268	75486492	CC472793	CC472793	CC472793	24	3	3	164	159	150			
2928	18	74107021	74107092	CC543759	CC543759	CC543759	24	5	5	164	159	150			
2929	18	72625377	72625531	CC499301	CC499301	CC499301	24	20	20	164	159	150			
2930	18	72344926	72345156	BZ943012	BZ943012	BZ943012	24	20	20	164	159	150			
2931	18	71342335	71342388	BZ920065	BZ920065	BZ920065	24	34	34	164	159	150			
2932	18	70273324	70273720	CC517338	CC517338	CC517338	24	39	39	164	159	150			
2933	18	69297120	69297389	CC771709	CC771709	CC771709	24	54	54	164	159	150			
2934	18	68218602	68218868	BZ932869	BZ932869	BZ932869	24	63	63	164	159	150			
2935	18	66983570	66984089	BZ925654	BZ925654	BZ925654	24	74	74	164	159	150			
2936	18	66084873	66085010	BZ942223	BZ942223	BZ942223	24	74	74	164	159	150			
2937	18	65250683	65250912	CC515340	CC515340	CC515340	24	74	74	164	159	150			
2938	18	63996484	63996613	BZ880385	BZ880385	BZ880385	24	88	88	164	159	150			
2939	18	63226284	63226675	CC477190	CC477190	CC477190	24	91	91	164	159	150			
2940	18	62250482	62251156	CC766072	CC766072	CC766072	24	97	97	164	159	150			
2941	18	61355739	61356339	CC490776	CC490776	CC490776	24	109	109	164	159	150			
2942	18	60263587	60263759	BZ854264	BZ854264	BZ854264	24	115	115	164	159	150			
2943	18	39104463	39104800	CC765795	CC765795	CC765795	24	131	131	165	160	151			
2944	18	38314239	38314925	CC489723	CC489723	CC489723	24	131	131	165	160	151			
2945	18	37371973	37372264	BZ951733	BZ951733	BZ951733	24	131	131	165	160	151			
2946	18	36217694	36218086	BZ930578	BZ930578	BZ930578	24	137	137	165	160	151			
2947	18	35214208	35214381	CC581814	CC581814	CC581814	24	146	146	165	160	151			
2948	18	34087067	34087245	CC507633	CC507633	CC507633	24	152	152	165	160	151			
2950	18	33133500	33133792	CC495691	CC495691	CC495691	24	158	158	165	160	151			
2951	18	32628408	32628697	AW461557	AW461557	AW461557	24	161	161	165	160	151			
2952	18	31943317	31943403	BE217451	BE217451	BE217451	24	167	167	165	160	151			
2953	18	31859568	31859819	BZ935847	BZ935847	BZ935847	24	167	167	165	160	151			
2954	18	30940793	30941475	CC497313	CC497313	CC497313	24	171	171	165	160	151			
2956	18	31171188	31171622	AW358730	AW358730	AW358730	24	177	177	165	160	151			
2957	18	30024073	30024232	BZ938692	BZ938692	BZ938692	24	183	183	165	160	151			
2959	18	29118370	29118684	BZ859196	BZ859196	BZ859196	24	190	190	165	160	151			
2960	18	28132735	28133180	CC504454	CC504454	CC504454	24	199	199	165	160	151			
2961	18	27188255	27189141	X57784	X57784	X57784	24	199	199	165	160	151			
2962	18	25881236	25881569	BZ867228	BZ867228	BZ867228	24	214	214	165	160	151			
2963	18	24920300	24920975	CC503074	CC503074	CC503074	24	218	218	165	160	151			
2965	18	23899325	23899794	CC595240	CC595240	CC595240	24	221	221	165	160	151			
2966	18	22958028	22958135	CC487070	CC487070	CC487070	24	221	221	165	160	151			
2967	18	22689975	22690452	AB012950	AB012950	AB012950	24	221	221	165	160	151			
2968	18	21766884	21767284	CC540932	CC540932	CC540932	24	229	229	165	160	151			
2969	18	20821242	20821563	BZ852577	BZ852577	BZ852577	24	233	233	165	160	151			
2970	18	18921021	18921413	CC508103	CC508103	CC508103	24	241	241	165	160	151			
2971	18	19899906	19900169	CC482262	CC482262	CC482262	24	248	248	165	160	151			
2972	18	17868274	17868477	BZ954631	BZ954631	BZ954631	24	276	276	165	160	151			
2973	18	16813634	16814008	CC534666	CC534666	CC534666	24	280	280	165	160	151			
2974	18	201131	201608	AW461839	AW461839	AW461839	24	280	280	166	161	deleted			
2975	18	213322	213499	CC505745	CC505745	CC505745	24	280	280	166	161	deleted			
2976	18	659105	659178	U78863	U78863	U78863	24	283	283	166	161	152			
2977	18	899501	899910	U78859	U78859	U78859	24	283	283	166	161	152			
2978	18	1496657	1497052	CC765629	CC765629	CC765629	24	286	286	166	161	152			
2979	18	2712585	2712734	CC509874	CC509874	CC509874	24	293	293	166	161	152			
2980	18	3544352	3544393	CC522773	CC522773	CC522773	24	305	305	166	161	152			
2981	18	4740524	4740849	BZ882463	BZ882463	BZ882463	24	321	321	166	161	152			
2982	18	5596112	5596501	BZ880043	BZ880043	BZ880043	24	333	333	166	161	152			
2983	18	6597441	6597687	CC554651	CC554651	CC554651	24	340	340	166	161	152			
2984	18	7070279	7070447	AF010231	AF010231	AF010231	24	340	340	166	161	152			
2985	18	7503186	7503589	CC565243	CC565243	CC565243	24	348	348	166	161	152			
2986	18	8059737	8059990	AW462710	AW462710	AW462710	24	348	348	166	161	152			
2987	18	9852480	9852523	AW428566	AW428566	AW428566	24	359	359	166	161	152			
2988	18	8809185	8809263	CC531991	CC531991	CC531991	24	363	363	166	161	152			
2989	18	10730828	10731067	AW465522	AW465522	AW465522	24	367	367	166	161	152			
2990	18	11862265	11862520	CC595724	CC595724	CC595724	24	367	367	166	161	152			
2991	18	12436702	12436834	AW326764	AW326764	AW326764	24	367	367	166	161	152			
2992	18	12941805	12941952	CC762509	CC762509	CC762509	24	370	370	166	161	152			
2993	18	13874034	13875624	X74501	X74501	X74501	24	374	374	166	161	152			
2994	18	41352622	41352870	BZ953865	BZ953865	BZ953865	24	386	386	167	162	153			
2995	18	43430540	43430772	CC519994	CC519994	CC519994	24	404	404	167	162	153			
2996	18	44315547	44315648	BZ837223	BZ837223	BZ837223	24	408	408	167	162	153			
2997	18	42435632	42435976	CC483514	CC483514	CC483514	24	408	408	167	162	153			
2998	18	45299640	45299754	BZ855950	BZ855950	BZ855950	24	408	408	167	162	153			
3000	18	46727918	46728062	AW289322	AW289322	AW289322	24	413	413	167	162	153			
3001	18	47376489	47376804	BZ875899	BZ875899	BZ875899	24	417	417	167	162	153			
3002	18	48457366	48457692	BZ932571	BZ932571	BZ932571	24	426	426	167	162	153			
3003	18	49344353	49344790	CC592006	CC592006	CC592006	24	434	434	167	162	153			
3004	18	50237662	50237833	CC483954	CC483954	CC483954	24	437	437	167	162	153			
3005	18	51314669	51315170	CC556161	CC556161	CC556161	24	437	437	167	162	153			
3006	18	52291694	52291886	CC582756	CC582756	CC582756	24	441	441	167	162	153			
3007	18	53287400	53287727	CC542978	CC542978	CC542978	24	445	445	167	162	153			
3008	18	53389622	53389772	L34173	L34173	L34173	24	449	449	167	162	153			
3010	18	54334916	54335390	BZ908134	BZ908134	BZ908134	24	456	456	167	162	153			
3011	18	55147443	55147898	AW289404	AW289404	AW289404	24	460	460	167	162	153			
3012	18	56623310	56623792	CC525040	CC525040	CC525040	24	464	464	167	162	153			
3013	18	57629905	57630349	CC553155	CC553155	CC553155	24	468	468	167	162	153			
3014	18	58539228	58539496	CC577644	CC577644	CC577644	24	468	468	167	162	153			
3015	18	59384638	59385203	CC541389	CC541389	CC541389	24	468	468	167	162	153			
3017	16	1106653	1106716	CC523209	CC523209	CC523209	25	42	42	168	163	154			
3018	16	2174736	2174920	CC526987	CC526987	CC526987	25	61	61	168	163	154			
3019	16	3048387	3048623	CC772620	CC772620	CC772620	25	75	75	168	163	154			
3020	16	3982257	3982389	BZ841821	BZ841821	BZ841821	25	81	81	168	163	154			
3021	16	5048447	5048641	BZ863322	BZ863322	BZ863322	25	95	95	168	163	154			
3022	16	6235403	6235593	CC517037	CC517037	CC517037	25	105	105	168	163	154			
3023	16	7138427	7138704	CC591791	CC591791	CC591791	25	108	108	168	163	154			
3024	16	7877437	7877748	BZ856380	BZ856380	BZ856380	25	114	114	168	163	154			
3025	16	9096534	9096810	CC489868	CC489868	CC489868	25	133	133	168	163	154			
3026	16	9995820	9996420	CC502027	CC502027	CC502027	25	154	154	168	163	154			
3027	16	10978556	10978760	BZ837182	BZ837182	BZ837182	25	167	167	168	163	154			
3028	16	12149010	12149398	BZ844519	BZ844519	BZ844519	25	184	184	168	163	154			
3030	16	13199809	13200209	BZ924364	BZ924364	BZ924364	25	194	194	168	163	154			
3031	16	15440304	15440398	BZ890471	BZ890471	BZ890471	25	216	216	168	163	154			
3032	16	14512681	14512805	CC500382	CC500382	CC500382	25	220	220	168	163	154			
3033	16	16767357	16767674	BZ872178	BZ872178	BZ872178	25	227	227	168	163	154			
3034	16	17778555	17778988	BZ914207	BZ914207	BZ914207	25	227	227	168	163	154			
3035	16	18963670	18963904	CC466791	CC466791	CC466791	25	238	238	168	163	154			
3036	16	19040211	19040461	BF146286	BF146286	BF146286	25	241	241	168	163	154			
3037	12	7350362	7350523	CC576394	CC576394	CC576394	25	259	259	169	out-of-place	singleton	ST here	ST is correct in calling them out-of-place markers	
3038	12	7344608	7344690	CC568434	CC568434	CC568434	25	259	259	169	out-of-place	deleted		because these two markers fall inside a HSB that was broken by Everts-van der Wind et al	
3039	16	20155365	20155545	BZ950119	BZ950119	BZ950119	25	262	262	170	164	155			
3040	16	21124318	21124728	BZ884302	BZ884302	BZ884302	25	266	266	170	164	155			
3041	16	22091060	22091286	BZ848714	BZ848714	BZ848714	25	278	278	170	164	155			
3042	16	23039770	23040123	CC533984	CC533984	CC533984	25	295	295	170	164	155			
3044	16	24133480	24133990	M13974	M13974	M13974	25	310	310	170	164	155			
3045	16	24838209	24838817	AW428603	AW428603	AW428603	25	322	322	170	164	155			
3046	16	26055263	26055408	BZ915833	BZ915833	BZ915833	25	322	322	170	164	155			
3047	16	27282047	27282435	AW632669	AW632669	AW632669	25	329	329	170	164	155			
3048	16	28172687	28172732	CC537344	CC537344	CC537344	25	344	344	170	164	155			
3049	16	29721586	29721759	AW426804	AW426804	AW426804	25	373	373	170	164	155			
3050	16	28901673	28901756	BZ935165	BZ935165	BZ935165	25	382	382	170	164	155			
3051	16	30273354	30273528	BZ845312	BZ845312	BZ845312	25	407	407	170	164	155			
3052	16	31328921	31329035	CC477234	CC477234	CC477234	25	411	411	170	164	155			
3053	7	55825554	55825776	BZ841965	BZ841965	BZ841965	25	422	422	171	165	156			
3054	7	65857583	65857849	BZ900787	BZ900787	BZ900787	25	433	433	171	165	156		AG is wrong in merging these two blocks, as other blocks comes in between	3 ->1
3055	7	71207007	71207179	CC529945	CC529945	CC529945	25	440	440	172	166	156	AG here		
3056	7	70118402	70118578	BZ939953	BZ939953	BZ939953	25	448	448	172	166	156			
3057	7	68036636	68037061	CC517686	CC517686	CC517686	25	455	455	172	166	156			
3059	7	72065678	72065979	AW289229	AW289229	AW289229	25	504	504	173	167	156			
3060	7	73245471	73245625	CC537081	CC537081	CC537081	25	504	504	173	167	156			
3061	7	73638248	73638423	AW657333	AW657333	AW657333	25	507	507	173	167	156			
3062	7	74913679	74913799	BZ844985	BZ844985	BZ844985	25	522	522	173	167	156			
3063	7	75707437	75707615	U05775	U05775	U05775	25	530	530	173	167	156			
3064	7	75787031	75787165	CC507323	CC507323	CC507323	25	535	535	173	167	156			
3065	7	101693745	101693868	CC521621	CC521621	CC521621	25	566	566	174	168	157			
3066	7	100610571	100610771	CC528735	CC528735	CC528735	25	586	586	174	168	157			
3067	7	100365104	100365378	X16383	X16383	X16383	25	607	607	174	168	157			
3068	7	99965217	99965968	U44762	U44762	U44762	25	624	624	174	168	157			
3069	7	99920966	99921381	M16480	M16480	M16480	25	630	630	174	168	157			
3070	7	99648931	99649356	BZ876324	BZ876324	BZ876324	25	630	630	174	168	157			
3071	7	98681227	98681469	U96448	U96448	U96448	25	641	641	174	168	157			
3072	7	97898939	97899063	AW632816	AW632816	AW632816	25	643	643	174	168	157			
3074	7	5498528	5498700	CC764016	CC764016	CC764016	25	651	651	175	169	158			
3075	7	5040055	5040216	AW485976	AW485976	AW485976	25	659	659	175	169	158			
3076	7	3353880	3354163	CC567335	CC567335	CC567335	25	664	664	175	169	158			
3077	7	2356569	2356871	BZ911555	BZ911555	BZ911555	25	669	669	175	169	158			
3078	7	2176466	2176664	AW336223	AW336223	AW336223	25	669	669	175	169	158			
3079	7	1249563	1249849	BZ879114	BZ879114	BZ879114	25	678	678	175	169	158			
3080	11	55917706	55918228	BZ913644	BZ913644	BZ913644	26	0	0	176	out-of-place	159	ST here	ST is correct in calling them out-of-place markers	because these two markers falls inside a HSB that was broken by Everts-van der Wind et al
3081	11	56440229	56440391	BZ901124	BZ901124	BZ901124	26	12	12	176	out-of-place	159	AG here	Out-of-place markers were defined as HSBs by AG	0 -> 1
3082	10	58833759	58834206	CC762517	CC762517	CC762517	26	17	17	177	170	160			
3083	10	57789774	57789909	AW326775	AW326775	AW326775	26	64	64	177	170	160			
3084	10	56618147	56618213	CC559926	CC559926	CC559926	26	90	90	177	170	160			
3085	10	55519405	55519704	CC584849	CC584849	CC584849	26	98	98	177	170	160			
3086	10	54436128	54436301	CC475522	CC475522	CC475522	26	106	106	177	170	160			
3087	10	53718696	53718958	CC770220	CC770220	CC770220	26	112	112	177	170	160			
3088	10	52750659	52750869	CC545032	CC545032	CC545032	26	137	137	177	170	160			
3089	10	51735087	51735750	CC533772	CC533772	CC533772	26	158	158	177	170	160			
3090	10	89245834	89246233	CC478067	CC478067	CC478067	26	158	158	178	171	deleted			
3091	10	90262408	90262517	BE217490	BE217490	BE217490	26	170	170	178	171	161			
3092	10	91169335	91169540	AW289367	AW289367	AW289367	26	201	201	178	171	161			
3093	10	92182561	92182804	BZ954997	BZ954997	BZ954997	26	201	201	178	171	161			
3095	10	93234860	93235030	BZ859874	BZ859874	BZ859874	26	212	212	178	171	161			
3096	10	95169977	95170077	BZ876467	BZ876467	BZ876467	26	216	216	178	171	161			
3097	10	94236784	94237003	CC527725	CC527725	CC527725	26	216	216	178	171	161			
3099	10	96337650	96338064	CC775747	CC775747	CC775747	26	220	220	178	171	161			
3100	10	97240762	97240978	CC517635	CC517635	CC517635	26	235	235	178	171	161			
3101	10	98054192	98054447	X04122	X04122	X04122	26	242	242	178	171	161			
3102	10	98293783	98293971	BZ867537	BZ867537	BZ867537	26	249	249	178	171	161			
3103	10	99251967	99252122	BZ947075	BZ947075	BZ947075	26	252	252	178	171	161			
3104	10	100310071	100310411	BZ833124	BZ833124	BZ833124	26	255	255	178	171	161			
3105	10	101147230	101147433	X66020	X66020	X66020	26	255	255	178	171	161			
3107	10	102300649	102300900	BZ945772	BZ945772	BZ945772	26	282	282	178	171	161			
3108	10	103305713	103305890	BZ925982	BZ925982	BZ925982	26	282	282	178	171	161			

3109	10	104364931	104365095	CC473615	CC473615	CC473615	26	287	287	178	171	161			
3110	10	104839461	104839661	U73690	U73690	U73690	26	291	291	178	171	161			
3111	10	105435863	105436038	CC593624	CC593624	CC593624	26	299	299	178	171	161			
3112	10	106447315	106447712	CC496195	CC496195	CC496195	26	309	309	178	171	161			
3113	10	107445593	107445822	CC585779	CC585779	CC585779	26	317	317	178	171	161			
3114	10	108453028	108453255	BZ941034	BZ941034	BZ941034	26	337	337	178	171	161			
3115	10	109432965	109433101	CC520635	CC520635	CC520635	26	382	382	178	171	161			
3116	10	110375958	110376224	BZ866535	BZ866535	BZ866535	26	394	394	178	171	161			
3117	10	111455505	111455779	CC466338	CC466338	CC466338	26	398	398	178	171	161			
3119	10	112534067	112534218	CC549607	CC549607	CC549607	26	405	405	178	171	161			
3120	10	113571846	113572006	BZ865995	BZ865995	BZ865995	26	405	405	178	171	161			
3121	10	114201373	114201600	AW289417	AW289417	AW289417	26	415	415	178	171	161			
3122	10	115666415	115666931	AF013092	AF013092	AF013092	26	437	437	178	171	161			
3123	10	116463728	116463972	CC503321	CC503321	CC503321	26	457	457	178	171	161			
3124	10	117644162	117644670	BZ849205	BZ849205	BZ849205	26	464	464	178	171	161			
3125	10	118627668	118627827	CC565193	CC565193	CC565193	26	467	467	178	171	161			
3126	10	119541337	119541811	BZ861918	BZ861918	BZ861918	26	467	467	178	171	161			
3127	10	120344562	120344755	AW327236	AW327236	AW327236	26	470	470	178	171	161			
3128	10	121775890	121776123	BZ869536	BZ869536	BZ869536	26	470	470	178	171	161			
3129	10	122782518	122782825	CC499337	CC499337	CC499337	26	477	477	178	171	161			
3130	10	123842846	123843042	CC475045	CC475045	CC475045	26	489	489	178	171	161			
3131	10	124264024	124264402	AW267015	AW267015	AW267015	26	489	489	178	171	161			
3133	10	125013455	125013629	BZ870511	BZ870511	BZ870511	26	499	499	178	171	161			
3135	10	125759623	125760013	CC498680	CC498680	CC498680	26	505	505	178	171	161			
3136	10	126849981	126850252	CC542542	CC542542	CC542542	26	516	516	178	171	161			
3137	10	128027240	128027321	CC572279	CC572279	CC572279	26	530	530	178	171	161			
3138	10	129041946	129042509	CC541563	CC541563	CC541563	26	534	534	178	171	161			
3139	10	130253524	130253728	BZ885017	BZ885017	BZ885017	26	537	537	178	171	161			
3140	10	131104294	131104564	BZ923326	BZ923326	BZ923326	26	551	551	178	171	161			
3141	10	132264903	132265371	BZ882266	BZ882266	BZ882266	26	565	565	178	171	161			
3142	10	134583548	134583723	CC487757	CC487757	CC487757	26	583	583	178	171	161			
3143	10	135082545	135082865	AW426132	AW426132	AW426132	26	589	589	178	171	161			
3144	8	1802080	1802198	BZ887871	BZ887871	BZ887871	27	0	0	179	172	162			
3145	8	2819371	2819725	BZ940868	BZ940868	BZ940868	27	0	0	179	172	162			
3146	8	3553288	3553591	CC467485	CC467485	CC467485	27	3	3	179	172	162			
3147	8	4557931	4558202	BZ846725	BZ846725	BZ846725	27	18	18	179	172	162			
3148	8	5504815	5505037	CC547920	CC547920	CC547920	27	48	48	179	172	162			
3150	8	6347713	6348192	AF094699	AF094699	AF094699	27	48	48	179	172	162			
3151	8	6469186	6469278	CC772376	CC772376	CC772376	27	71	71	179	172	162			
3153	4	176997854	176997950	BZ854808	BZ854808	BZ854808	27	122	122	180	173	163			
3154	4	177804989	177805637	CC561430	CC561430	CC561430	27	147	147	180	173	163			
3155	4	178782364	178782471	CC765073	CC765073	CC765073	27	162	162	180	173	163			
3156	4	179837004	179837294	CC519877	CC519877	CC519877	27	172	172	180	173	163			
3157	4	180915181	180915606	BZ910025	BZ910025	BZ910025	27	182	182	180	173	163			
3158	4	181916929	181917509	CC563383	CC563383	CC563383	27	187	187	180	173	163			
3160	4	183802849	183803150	BZ873245	BZ873245	BZ873245	27	208	208	180	173	163			
3161	4	184794601	184794747	BZ857437	BZ857437	BZ857437	27	213	213	180	173	163			
3162	4	182884537	182884740	CC768672	CC768672	CC768672	27	229	229	180	173	163			
3163	4	185990782	185991120	AW336113	AW336113	AW336113	27	235	235	180	173	163			
3164	4	186441064	186441557	M24102	M24102	M24102	27	247	247	180	173	163			
3166	4	187902372	187902522	AW356960	AW356960	AW356960	27	272	272	180	173	163			
3167	4	187009768	187009947	BZ888622	BZ888622	BZ888622	27	282	282	180	173	163			
3169	4	188950699	188950849	CC506040	CC506040	CC506040	27	317	317	180	173	163			
3170	4	189889935	189890352	BZ838880	BZ838880	BZ838880	27	336	336	180	173	163			
3171	8	17961119	17961260	AW461677	AW461677	AW461677	27	362	362	181	174	164			
3172	8	17452054	17452387	CC529063	CC529063	CC529063	27	380	380	181	174	164			
3173	8	17131136	17131710	AW289341	AW289341	AW289341	27	385	385	181	174	164			
3174	8	16508369	16508590	BZ902552	BZ902552	BZ902552	27	389	389	181	174	164			
3175	8	16042639	16042925	X54183	X54183	X54183	27	411	411	181	174	164			
3176	8	15575592	15575758	BZ866372	BZ866372	BZ866372	27	415	415	181	174	164			
3177	8	14447792	14448102	CC529930	CC529930	CC529930	27	415	415	181	174	164			
3178	8	13440789	13441002	CC507003	CC507003	CC507003	27	424	424	181	174	164			
3179	8	12924410	12924587	CC590187	CC590187	CC590187	27	428	428	181	174	164		AG is wrong in merging these two blocks, as other blocks comes in between	2 -> 1
3180	8	8219223	8219348	CC488006	CC488006	CC488006	27	433	433	182	175	164	AG here		
3181	8	9279110	9279244	BZ861672	BZ861672	BZ861672	27	437	437	182	175	164			
3182	8	29846977	29847601	BZ870336	BZ870336	BZ870336	27	437	437	183	176	deleted			
3183	8	30970680	30970726	CC569958	CC569958	CC569958	27	437	437	183	176	deleted			
3185	8	32032503	32032628	BZ856071	BZ856071	BZ856071	27	451	451	183	176	165			
3186	8	33038344	33038761	BZ914972	BZ914972	BZ914972	27	456	456	183	176	165			
3187	8	34043557	34043874	CC484383	CC484383	CC484383	27	456	456	183	176	165			
3188	8	35880930	35881421	CC469562	CC469562	CC469562	27	464	464	183	176	165			
3189	8	36899118	36899435	CC522923	CC522923	CC522923	27	464	464	183	176	165			
3190	8	37867937	37868426	CC540934	CC540934	CC540934	27	473	473	183	176	165			
3191	8	40958198	40958359	CC584934	CC584934	CC584934	27	482	482	183	176	165			
3192	8	38999782	39000006	BZ946381	BZ946381	BZ946381	27	486	486	183	176	165			
3193	8	39959273	39959468	CC513312	CC513312	CC513312	27	490	490	183	176	165			
3194	8	41487585	41487656	AW289269	AW289269	AW289269	27	504	504	183	176	165			
3197	8	41908818	41909119	AW358694	AW358694	AW358694	27	520	520	183	176	165			
3198	8	41691667	41691728	BZ843582	BZ843582	BZ843582	27	526	526	183	176	165			
3200	8	42421261	42421465	AW464753	AW464753	AW464753	27	536	536	183	176	165			
3201	8	42692485	42692563	CC563114	CC563114	CC563114	27	536	536	183	176	165			
3202	8	43121222	43121464	CC548187	CC548187	CC548187	27	541	541	183	176	165		Deletion of markers by AG resulted in loosing an HSB	1 -> 0
3203	8	18640866	18640987	BZ942895	BZ942895	BZ942895	27	541	541	184	177	deleted	AG here		
3204	8	19724217	19724324	CC498750	CC498750	CC498750	27	545	545	184	177	singleton			
3205	3	27194781	27195244	BZ919176	BZ919176	BZ919176	27	545	545	singleton	Singleton	deleted			
3208	3	23137787	23138131	BZ928615	BZ928615	BZ928615	27	586	586	185	178	166			
3209	3	21775795	21776118	BZ922514	BZ922514	BZ922514	27	590	590	185	178	166			
3210	3	24884613	24884932	BZ913729	BZ913729	BZ913729	27	609	609	185	178	166			
3211	3	25624587	25624678	CC523630	CC523630	CC523630	27	619	619	185	178	166			
3212	3	25902908	25903003	BZ956184	BZ956184	BZ956184	27	628	628	185	178	166			
3213	1	225891602	225891710	BZ900694	BZ900694	BZ900694	28	0	0	186	179	167			
3214	1	226724129	226724492	AW464595	AW464595	AW464595	28	16	16	186	179	167			
3216	1	228451070	228451407	CC762568	CC762568	CC762568	28	55	55	186	179	167			
3217	1	229802559	229803024	BZ942524	BZ942524	BZ942524	28	71	71	186	179	167			
3218	1	230869797	230869974	BZ886862	BZ886862	BZ886862	28	81	81	186	179	167			
3219	1	231602090	231602216	BZ941881	BZ941881	BZ941881	28	102	102	186	179	167			
3220	1	232151336	232151615	AW289305	AW289305	AW289305	28	109	109	186	179	167			
3221	1	233215250	233215511	BZ953668	BZ953668	BZ953668	28	117	117	186	179	167			
3222	1	234131590	234131670	CC587357	CC587357	CC587357	28	127	127	186	179	167			
3223	1	234948329	234948743	BZ929271	BZ929271	BZ929271	28	127	127	186	179	167			
3224	1	235616444	235616526	BZ935058	BZ935058	BZ935058	28	137	137	186	179	167			
3225	1	236396772	236398600	U08286	U08286	U08286	28	144	144	186	179	167		Deletion of markers by AG resulted in loosing an HSB	1 -> 0
3226	10	38386624	38386970	AW289245	AW289245	AW289245	28	144	144	187	180	deleted	AG here		
3227	10	43011374	43011730	AW289342	AW289342	AW289342	28	150	150	187	180	singleton			
3228	10	59934482	59934724	CC521188	CC521188	CC521188	28	150	150	188	181	deleted			
3229	10	61012192	61012489	CC590941	CC590941	CC590941	28	150	150	188	181	deleted			
3230	10	61981977	61982494	CC475663	CC475663	CC475663	28	155	155	188	181	168			
3231	10	63063496	63063678	BZ942043	BZ942043	BZ942043	28	161	161	188	181	168			
3232	10	64151300	64151773	BZ862650	BZ862650	BZ862650	28	166	166	188	181	168			
3233	10	65302208	65302528	CC557210	CC557210	CC557210	28	175	175	188	181	168			
3234	10	66424965	66425435	CC478245	CC478245	CC478245	28	175	175	188	181	168			
3235	10	68510801	68511068	CC537389	CC537389	CC537389	28	189	189	188	181	168			
3236	10	67439617	67440215	CC588236	CC588236	CC588236	28	194	194	188	181	168			
3237	10	69565753	69566064	CC570976	CC570976	CC570976	28	202	202	188	181	168			
3238	10	70621387	70621559	BZ931028	BZ931028	BZ931028	28	205	205	188	181	168			
3239	10	71681196	71681626	BZ928265	BZ928265	BZ928265	28	207	207	188	181	168			
3240	10	72690123	72690344	BZ868940	BZ868940	BZ868940	28	216	216	188	181	168			
3241	10	73704088	73704337	CC774978	CC774978	CC774978	28	231	231	188	181	168			
3242	10	75466987	75467337	CC504182	CC504182	CC504182	28	246	246	188	181	168			
3243	10	76473551	76473798	BZ862250	BZ862250	BZ862250	28	266	266	188	181	168			
3244	10	77634868	77635261	BZ936556	BZ936556	BZ936556	28	278	278	188	181	168			
3245	10	78679557	78680112	CC512019	CC512019	CC512019	28	278	278	188	181	168			
3246	10	79765421	79765641	CC550317	CC550317	CC550317	28	289	289	188	181	168			
3247	10	80726176	80726279	CC472414	CC472414	CC472414	28	308	308	188	181	168			
3248	10	81916618	81916729	BZ906211	BZ906211	BZ906211	28	320	320	188	181	168			
3249	10	81918712	81918992	Z11742	Z11742	Z11742	28	326	326	188	181	168			
3250	10	82859421	82859466	CC487469	CC487469	CC487469	28	350	350	188	181	168			
3251	10	83955966	83956162	CC517126	CC517126	CC517126	28	364	364	188	181	168			
3252	10	85018381	85018773	BZ858700	BZ858700	BZ858700	28	383	383	188	181	168			
3253	10	85997325	85997484	S67535	S67535	S67535	28	383	383	188	181	168			
3254	10	86227283	86227372	BZ867157	BZ867157	BZ867157	28	383	383	188	181	168			
3255	10	87097132	87097197	BZ876471	BZ876471	BZ876471	28	386	386	188	181	168			
3256	10	88216969	88217156	CC477830	CC477830	CC477830	28	389	389	188	181	168		Deletion of markers by AG resulted in loosing an HSB	1 -> 0
3257	10	43784420	43784640	CC595484	CC595484	CC595484	28	401	401	189	182	singleton	AG here		
3258	10	44749073	44749274	BZ832830	BZ832830	BZ832830	28	401	401	189	182	deleted			
3259	10	45347700	45347835	BZ846736	BZ846736	BZ846736	28	401	401	189	182	deleted			
3260	10	48181976	48182084	CC513525	CC513525	CC513525	28	401	401	189	182	deleted			
3261	10	49620836	49621368	CC578724	CC578724	CC578724	28	401	401	189	182	deleted			
3262	10	50187946	50188222	BZ880756	BZ880756	BZ880756	28	401	401	189	182	deleted			
3263	10	50757717	50757891	U78975	U78975	U78975	28	409	409	189	182	singleton			
3264	11	92784292	92784387	BZ919809	BZ919809	BZ919809	29	0	0	190	183	169			
3265	11	92162691	92163134	BZ898158	BZ898158	BZ898158	29	7	7	190	183	169			
3266	11	91862924	91863186	BZ919428	BZ919428	BZ919428	29	10	10	190	183	169			
3267	11	90945097	90945380	CC485774	CC485774	CC485774	29	15	15	190	183	169			
3268	11	89677469	89677797	CC447793	CC447793	CC447793	29	23	23	190	183	169			
3269	11	89923500	89923744	BZ900973	BZ900973	BZ900973	29	30	30	190	183	169			
3270	11	88222752	88223015	BZ930457	BZ930457	BZ930457	29	46	46	190	183	169			
3271	11	87355506	87355962	BZ915122	BZ915122	BZ915122	29	46	46	190	183	deleted		Presence of singletons made AG to break the HSB defined by ST	1 -> 2
3272	11	49334573	49334952	BZ914329	BZ914329	BZ914329	29	56	56	out-of-place	out-of-place	singleton	AG here		
3273	11	86339305	86339480	BE217506	BE217506	BE217506	29	87	87	190	183	170			
3274	11	85509671	85509974	BZ901563	BZ901563	BZ901563	29	106	106	190	183	170			
3276	11	84673912	84674066	AW315850	AW315850	AW315850	29	113	113	190	183	170			
3277	11	83704638	83705265	BZ951039	BZ951039	BZ951039	29	116	116	190	183	170			
3278	11	83022884	83023514	BZ921801	BZ921801	BZ921801	29	118	118	190	183	170			
3279	11	79012775	79013322	BZ935392	BZ935392	BZ935392	29	141	141	190	183	170			
3280	11	78192403	78192625	BZ949696	BZ949696	BZ949696	29	146	146	190	183	170			
3281	11	76898659	76898898	BZ955148	BZ955148	BZ955148	29	156	156	190	183	170			
3282	11	24659959	24660078	CC529935	CC529935	CC529935	29	179	179	191	184	171			
3283	11	24817593	24817825	BZ933262	BZ933262	BZ933262	29	190	190	191	184	171			
3284	11	23434075	23434135	BZ940564	BZ940564	BZ940564	29	214	214	191	184	171			
3285	11	22753805	22754023	BZ932447	BZ932447	BZ932447	29	225	225	191	184	171			
3286	11	21612469	21612727	BZ946384	BZ946384	BZ946384	29	237	237	191	184	171			
3287	11	20844466	20844811	BZ920356	BZ920356	BZ920356	29	237	237	191	184	171			
3288	11	20383257	20383435	BZ908549	BZ908549	BZ908549	29	245	245	191	184	171			
3289	11	19142310	19142750	BZ905352	BZ905352	BZ905352	29	248	248	191	184	171			
3290	11	18680823	18681132	AW461867	AW461867	AW461867	29	262	262	191	184	171			
3291	11	18256921	18257197	AW267134	AW267134	AW267134	29	278	278	191	184	171			
3292	11	124142322	124142710	BZ956113	BZ956113	BZ956113	29	302	302	192	185	172			
3293	11	125413914	125414347	BZ900418	BZ900418	BZ900418	29	310	310	192	185	172			
3294	11	126341369	126341645	CC534838	CC534838	CC534838	29	316	316	192	185	172			
3295	11	127300854	127301399	BZ933943	BZ933943	BZ933943	29	318	318	192	185	172			
3297	11	127836287	127836866	AW289328	AW289328	AW289328	29	334	334	192	185	172			
3298	11	128662643	128662756	BZ949426	BZ949426	BZ949426	29	334	334	192	185	172			
3299	11	129824020	129824539	BZ932751	BZ932751	BZ932751	29	347	347	192	185	172			
3300	11	130960131	130960637	BZ907953	BZ907953	BZ907953	29	352	352	192	185	172			
3301	11	132318028	132318520	X12672	X12672	X12672	29	354	354	192	185	172			
3302	11	132573971	132574078	BZ928713	BZ928713	BZ928713	29	354	354	192	185	deleted			
3303	11	60902861	60903106	BZ950502	BZ950502	BZ950502	29	391	391	193	186	173			
3305	11	62039954	62040476	AW289287	AW289287	AW289287	29	404	404	193	186	173			
3306	11	62508825	62509191	BZ912781	BZ912781	BZ912781	29	406	406	193	186	173			
3307	11	63433022	63433542	AW668868	AW668868	AW668868	29	452	452	193	186	173			
3308	11	63816866	63817331	BZ944599	BZ944599	BZ944599	29	457	457	193	186	173			
3309	11	64455778	64456025	BZ919434	BZ919434	BZ919434	29	460	460	193	186	173			
3310	11	64801647	64801802	BZ896890	BZ896890	BZ896890	29	463	463	193	186	173			
3311	11	66087671	66087729	AW289265	AW289265	AW289265	29	473	473	193	186	173			
3312	11	66395711	66395881	BZ898137	BZ898137	BZ898137	29	476	476	193	186	173			
3313	11	67562185	67562237	BZ927273	BZ927273	BZ927273	29	479	479	193	186	173			
3315	11	68208644	68208800	S68957	S68957	S68957	29	485	485	193	186	173			
3316	11	69277013	69277199	CC586412	CC586412	CC586412	29	488	488	193	186	173			
3317	11	70222977	70223222	BZ887224	BZ887224	BZ887224	29	488	488	193	186	deleted			
3318	11	2922250	2922318	AW266993	AW266993	AW266993	29	488	488	194	187	deleted			
3319	11	2786121	2786276	BZ910110	BZ910110	BZ910110	29	494	494	194	187	174			
3320	11	2375044	2375196	AW266935	AW266935	AW266935	29	503	503	194	187	174			
3321	11	237275	237450	AW462407	AW462407	AW462407	29	516	516	194	187	174			
3322	X	116022414	116022901	BZ840181	BZ840181	BZ840181	X	0	0	195	188	175			
3323	X	117060425	117060821	BZ879944	BZ879944	BZ879944	X	21	21	195	188	175			
3325	X	118170643	118170909	BZ936929	BZ936929	BZ936929	X	44	44	195	188	175			
3327	X	119465774	119465911	BZ922559	BZ922559	BZ922559	X	69	69	195	188	175			
3328	X	121038430	121038943	CC535539	CC535539	CC535539	X	79	79	195	188	175			
3329	X	121933563	121933760	CC767640	CC767640	CC767640	X	84	84	195	188	175			
3331	X	120048645	120048889	BZ883856	BZ883856	BZ883856	X	100	100	195	188	175			
3332	X	122461262	122461712	AW289303	AW289303	AW289303	X	119	119	195	188	175			
3333	X	123874293	123874587	CC764892	CC764892	CC764892	X	125	125	195	188	175			
3334	X	125160811	125161006	CC447617	CC447617	CC447617	X	137	137	195	188	175			
3336	X	126071856	126072234	BZ884723	BZ884723	BZ884723	X	169	169	195	188	175			
3337	X	127509809	127510330	BZ910053	BZ910053	BZ910053	X	179	179	195	188	175			
3338	X	128271771	128271979	BZ928085	BZ928085	BZ928085	X	184	184	195	188	175			
3339	X	129200883	129201356	CC514904	CC514904	CC514904	X	203	203	195	188	175			
3340	X	130447851	130448317	BZ852712	BZ852712	BZ852712	X	209	209	195	188	175			
3341	X	131543591	131543715	CC574775	CC574775	CC574775	X	215	215	195	188	175			
3342	X	132590158	132590310	CC573770	CC573770	CC573770	X	229	229	195	188	175			
3343	X	133707390	133707546	BZ949239	BZ949239	BZ949239	X	229	229	195	188	175			
3344	X	133891913	133892430	AW464203	AW464203	AW464203	X	229	229	195	188	175			
3345	X	135590611	135590868	CC566281	CC566281	CC566281	X	235	235	195	188	175			
3346	X	136689010	136689172	CC542106	CC542106	CC542106	X	276	276	195	188	175			
3347	X	137663161	137663306	BZ927965	BZ927965	BZ927965	X	297	297	195	188	175			
3348	X	138103919	138104079	CC772270	CC772270	CC772270	X	302	302	195	188	175			
3349	X	139407425	139408021	CC771367	CC771367	CC771367	X	346	346	195	188	175			
3350	X	140466980	140467039	BZ862720	BZ862720	BZ862720	X	354	354	195	188	175			
3351	X	141221326	141222070	CC553143	CC553143	CC553143	X	385	385	195	188	175			
3352	X	142394570	142394820	CC521183	CC521183	CC521183	X	390	390	195	188	175			
3353	X	143536033	143536247	BZ902304	BZ902304	BZ902304	X	402	402	195	188	175			
3354	X	144526258	144526593	CC487196	CC487196	CC487196	X	419	419	195	188	175			
3355	X	145698042	145698344	CC524076	CC524076	CC524076	X	439	439	195	188	175			
3357	X	146799337	146799902	CC490948	CC490948	CC490948	X	481	481	195	188	175			
3358	X	147723320	147723609	CC492357	CC492357	CC492357	X	492	492	195	188	175			
3359	X	149176325	149176608	CC537568	CC537568	CC537568	X	529	529	195	188	175			
3360	X	150518458	150518780	CC528045	CC528045	CC528045	X	551	551	195	188	175			
3362	X	151007153	151007605	X12362	X12362	X12362	X	581	581	195	188	175			
3363	X	151338539	151338683	BZ952226	BZ952226	BZ952226	X	590	590	195	188	175			
3364	X	152399020	152399353	CC539137	CC539137	CC539137	X	649	649	195	188	175			
3365	X	152522663	152522846	BZ865569	BZ865569	BZ865569	X	665	665	195	188	175			
3367	X	153565098	153565234	AW481424	AW481424	AW481424	X	677	677	195	188	175			
3368	X	154734343	154734393	BZ884969	BZ884969	BZ884969	X	704	704	195	188	175			
3369	X	90655231	90655439	BZ906622	BZ906622	BZ906622	X	734	734	196	189	176			
3370	X	91333708	91333794	CC570277	CC570277	CC570277	X	736	736	196	189	176			
3372	X	92441494	92441585	CC567726	CC567726	CC567726	X	756	756	196	189	176			
3373	X	94748711	94748870	BZ924247	BZ924247	BZ924247	X	773	773	196	189	176			
3374	X	95673244	95673600	BZ849012	BZ849012	BZ849012	X	785	785	196	189	176			

3375	X	96723208	96723833	BZ856917	BZ856917	BZ856917	X	797	797	196	189	176			
3376	X	97887907	97888533	CC570389	CC570389	CC570389	X	816	816	196	189	176			
3377	X	98909668	98909997	BZ940511	BZ940511	BZ940511	X	828	828	196	189	176			
3378	X	99924881	99925083	CC558458	CC558458	CC558458	X	852	852	196	189	176			
3380	X	100464999	100465272	BZ871035	BZ871035	BZ871035	X	859	859	196	189	176			
3381	X	101524529	101524863	CC775603	CC775603	CC775603	X	866	866	196	189	176	AG here	Inversion missed by AG	3 ->1
3382	X	105428038	105428196	CC553554	CC553554	CC553554	X	889	889	196	190	176	ST here	Inversion missed by Everts-van der Wind et al	
3383	X	104079724	104079817	BZ931493	BZ931493	BZ931493	X	904	904	196	190	176			
3384	X	102851587	102852153	X03098	X03098	X03098	X	918	918	196	190	176			
3385	X	102145352	102145830	CC477330	CC477330	CC477330	X	935	935	196	190	176			
3386	X	105870059	105870542	CC582591	CC582591	CC582591	X	982	982	196	191	176			
3387	X	106708724	106708907	CC520217	CC520217	CC520217	X	991	991	196	191	176			
3388	X	107635869	107636038	CC481236	CC481236	CC481236	X	1000	1000	196	191	176			
3389	X	108535407	108535532	BZ945492	BZ945492	BZ945492	X	1000	1000	196	191	176			
3390	X	108711763	108711837	AW461447	AW461447	AW461447	X	1005	1005	196	191	176			
3392	X	109499336	109499493	BZ906252	BZ906252	BZ906252	X	1014	1014	196	191	176			
3393	X	110502258	110502335	CC511548	CC511548	CC511548	X	1014	1014	196	191	176			
3394	X	111499778	111500198	CC576656	CC576656	CC576656	X	1019	1019	196	191	176			
3395	X	112341608	112341821	CC587934	CC587934	CC587934	X	1024	1024	196	191	176			
3396	X	113677621	113677842	BZ900268	BZ900268	BZ900268	X	1029	1029	196	191	176			
3397	X	114707002	114707541	AW462747	AW462747	AW462747	X	1038	1038	196	191	176			
3398	X	85794537	85794703	BZ849002	BZ849002	BZ849002	X	1042	1042	197	192	177			
3399	X	84890405	84890801	CC473032	CC473032	CC473032	X	1047	1047	197	192	177			
3400	X	83300016	83300100	BZ909102	BZ909102	BZ909102	X	1056	1056	197	192	177			
3401	X	82563207	82563401	BZ948204	BZ948204	BZ948204	X	1056	1056	197	192	177			
3402	X	81647436	81648173	CC566579	CC566579	CC566579	X	1060	1060	197	192	177			
3403	X	80425267	80426016	CC555240	CC555240	CC555240	X	1070	1070	197	192	177			
3404	X	78424172	78424361	CC562874	CC562874	CC562874	X	1095	1095	197	192	177			
3405	X	77231818	77232078	BZ949504	BZ949504	BZ949504	X	1095	1095	197	192	177			
3406	X	76568554	76569078	AW289378	AW289378	AW289378	X	1099	1099	197	192	177			
3407	X	76005744	76005919	CC517219	CC517219	CC517219	X	1108	1108	197	192	177			
3408	X	74420295	74420375	BZ929507	BZ929507	BZ929507	X	1112	1112	197	192	177			
3410	X	73537463	73537817	BZ955438	BZ955438	BZ955438	X	1127	1127	197	192	177			
3411	X	72828595	72829222	CC574702	CC574702	CC574702	X	1144	1144	197	192	177			
3412	X	71541396	71541719	CC506657	CC506657	CC506657	X	1156	1156	197	192	177			
3413	X	69667776	69667840	BZ847684	BZ847684	BZ847684	X	1174	1174	197	192	177			
3414	X	71049399	71049697	CC567042	CC567042	CC567042	X	1178	1178	197	192	177			
3415	X	68749067	68749227	BZ933208	BZ933208	BZ933208	X	1188	1188	197	192	177			
3416	X	67706418	67706552	CC513396	CC513396	CC513396	X	1193	1193	197	192	177			
3417	X	66714475	66714583	CC566852	CC566852	CC566852	X	1198	1198	197	192	177			
3418	X	65886117	65886215	BZ864547	BZ864547	BZ864547	X	1203	1203	197	192	177			
3419	X	51140005	51140188	BZ855348	BZ855348	BZ855348	X	1213	1213	198	193	178			
3420	X	50381277	50381706	CC560237	CC560237	CC560237	X	1213	1213	198	193	178			
3421	X	49386982	49387133	BZ873598	BZ873598	BZ873598	X	1218	1218	198	193	178			
3422	X	48595444	48595828	CC586477	CC586477	CC586477	X	1218	1218	198	193	178			
3424	X	47259683	47259990	BZ835125	BZ835125	BZ835125	X	1223	1223	198	193	178			
3425	X	46696704	46696992	CC501756	CC501756	CC501756	X	1223	1223	198	193	178			
3426	X	64732669	64733054	CC517135	CC517135	CC517135	X	1266	1266	199	194	179			
3427	X	63197731	63197896	BZ860185	BZ860185	BZ860185	X	1270	1270	199	194	179		AG is wrong in merging these two blocks, as other blocks comes in between	2 -> 1
3428	X	52906744	52906890	BZ869950	BZ869950	BZ869950	X	1300	1300	200	195	179	AG here		
3429	X	53853282	53853504	BZ911301	BZ911301	BZ911301	X	1318	1318	200	195	179			
3430	X	54872800	54872879	BZ831748	BZ831748	BZ831748	X	1336	1336	200	195	179			
3431	X	57820502	57820698	BZ891254	BZ891254	BZ891254	X	1350	1350	200	195	179			
3432	X	45651865	45652076	CC565070	CC565070	CC565070	X	1366	1366	201	196	180			
3433	X	44626494	44626558	BZ928347	BZ928347	BZ928347	X	1366	1366	201	196	180			
3434	X	43511522	43511750	CC595137	CC595137	CC595137	X	1380	1380	201	196	180			
3435	X	43361976	43362313	AW267052	AW267052	AW267052	X	1380	1380	201	196	180			
3436	X	41652009	41652259	BZ878158	BZ878158	BZ878158	X	1389	1389	201	196	180			
3437	X	42671917	42672728	BZ864069	BZ864069	BZ864069	X	1389	1389	201	196	180			
3438	X	40631696	40631798	BZ940484	BZ940484	BZ940484	X	1393	1393	201	196	180			
3439	X	39669326	39669556	BZ937544	BZ937544	BZ937544	X	1398	1398	201	196	180			
3440	X	38575761	38576101	CC764303	CC764303	CC764303	X	1403	1403	201	196	180			
3442	X	37815229	37815457	BZ919820	BZ919820	BZ919820	X	1413	1413	201	196	180			
3443	X	37419345	37419605	AF036097	AF036097	AF036097	X	1413	1413	201	196	180			
3444	X	34749863	34750481	CC531735	CC531735	CC531735	X	1413	1413	201	196	180			
3445	X	36306009	36306195	BZ831005	BZ831005	BZ831005	X	1418	1418	201	196	180			
3446	X	35266328	35266783	CC585642	CC585642	CC585642	X	1418	1418	201	196	180			
3447	X	33759479	33760172	CC560121	CC560121	CC560121	X	1418	1418	201	196	180			
3448	X	32720346	32720754	CC577844	CC577844	CC577844	X	1418	1418	201	196	180			
3449	X	32142782	32142949	CC518632	CC518632	CC518632	X	1418	1418	201	196	180			
3450	X	30960417	30960732	CC765676	CC765676	CC765676	X	1418	1418	201	196	180			
3453	X	30014029	30014141	BZ951619	BZ951619	BZ951619	X	1438	1438	201	196	180			
3454	X	28988512	28988574	CC529463	CC529463	CC529463	X	1443	1443	201	196	180			
3455	X	27721904	27722244	CC498328	CC498328	CC498328	X	0	0	201	196	180			
3456	X	26461406	26461710	CC565587	CC565587	CC565587	X	14	14	201	196	180			
3457	X	24503688	24503849	CC567328	CC567328	CC567328	X	34	34	201	196	180			
3458	X	25612657	25612560	BZ891621	BZ891621	BZ891621	X	34	34	201	196	180			
3459	X	23456827	23457077	CC485137	CC485137	CC485137	X	40	40	201	196	180			
3460	X	22510723	22510869	BZ887399	BZ887399	BZ887399	X	45	45	201	196	180			
3461	X	21653195	21653562	CC773473	CC773473	CC773473	X	45	45	201	196	180			
3462	X	20904201	20904627	BZ898280	BZ898280	BZ898280	X	45	45	201	196	180			
3463	X	20019679	20019907	CC475753	CC475753	CC475753	X	63	63	201	196	180			
3464	X	19932781	19933299	AW465031	AW465031	AW465031	X	63	63	201	196	180			
3465	X	19430055	19430302	CC519438	CC519438	CC519438	X	63	63	201	196	180			
3466	X	18605321	18605689	AW314533	AW314533	AW314533	X	76	76	201	196	180			
3467	X	17622196	17622481	BZ938515	BZ938515	BZ938515	X	76	76	201	196	180			
3468	X	16831858	16832448	CC472579	CC472579	CC472579	X	83	83	201	196	180			
3469	X	15918827	15919392	CC564485	CC564485	CC564485	X	83	83	201	196	180			
3471	X	14776430	14776605	CC541823	CC541823	CC541823	X	90	90	201	196	180			
3472	X	13744621	13744789	BZ853371	BZ853371	BZ853371	X	97	97	201	196	180			
3473	X	11076319	11076751	M86932	M86932	M86932	X	0	0	201	196	180			
3474	X	9492634	9492781	AW314864	AW314864	AW314864	X	13	13	201	196	180			
3475	X	8850801	8851012	CC526415	CC526415	CC526415	X	28	28	201	196	180			
3476	X	8031739	8031782	CC483846	CC483846	CC483846	X	39	39	201	196	180			
3478	X	7090867	7090952	BZ873986	BZ873986	BZ873986	X	45	45	201	196	180			
3479	X	6114593	6114748	BZ946391	BZ946391	BZ946391	X	45	45	201	196	180			
3482	X	3254325	3254426	BZ918292	BZ918292	BZ918292	X	74	74	201	196	180			
3483	X	1615459	1615603	AW656774	AW656774	AW656774	X	94	94	201	196	180			
3484	X	1551960	1552450	M24103	M24103	M24103	X	100	100	201	196	180			

Table 2. Discrepancies in HSB definitions between SyntenyTracker and AutoGRAPH using cattle-human RH comparative map.

Comparison	Number of cases
Reasons for HSBs defined by SyntenyTracker being joined by AutoGRAPH	
Sub-blocks on other reference chromosomes	10
Inversion missed by AutoGRAPH	2
Reasons for HSBs defined by SyntenyTracker being broken by AutoGRAPH into >1 HSB	
Presence of singletons	2
Out-of-place markers were called HSBs by AutoGRAPH	1
Deletion of markers by AutoGRAPH resulted in losing of HSB defined by SyntenyTracker	4
