# Supplementary material for: SyntenyTracker: a tool for defining homologous synteny blocks using radiation hybrid maps and whole-genome sequence
Source: BMC Res Notes. 2009 Jul 23;2:148. doi: 10.1186/1756-0500-2-148 (PMC2726151; doi:10.1186/1756-0500-2-148)
Supplement: Additional file 3 — Definition of HSBs on a set of human and mouse orthologs. Comparison of HSB definitions between SyntenyTracker and AutoGRAPH on a set of human and mouse orthologs. [file 1756-0500-2-148-S3.pdf]

Table 1. Comparison of HSB definitions between SyntenyTracker and AutoGRAPH on a set of human and mouse orthologs

Marker_order	HSA	HSA_start	HSA_end	Marker_name1	Marker_name2	Marker_name3	MMU	MMU_start	MMU_end	HSB_SyntenyTracker	HSB_AutoGRAPH	Comment	Comment
1	X	51166507	51168427	ENSMUSG00000073294	ENSMUSG00000073294	ENSMUSG00000073294	X	5256713	5258895	1	1		
2	X	50670524	50676347	ENSMUSG00000023279	ENSMUSG00000023279	ENSMUSG00000023279	X	5489598	5496190	1	1		
3	X	50351387	50573784	ENSMUSG00000068270	ENSMUSG00000068270	ENSMUSG00000068270	X	5575591	5810043	1	1		
4	X	50128637	50230477	ENSMUSG00000062393	ENSMUSG00000062393	ENSMUSG00000062393	X	6030442	6103762	1	1		
5	X	49856156	50111649	ENSMUSG00000051592	ENSMUSG00000051592	ENSMUSG00000051592	X	6136607	6187895	1	1		
6	X	49842146	49852404	ENSMUSG00000050089	ENSMUSG00000050089	ENSMUSG00000050089	X	6224474	6235563	1	1		
7	X	49573965	49750632	ENSMUSG00000004317	ENSMUSG00000004317	ENSMUSG00000004317	X	6316432	6345645	1	1		
8	X	49013261	49031021	ENSMUSG00000039556	ENSMUSG00000039556	ENSMUSG00000039556	X	6715909	6731079	1	1		
9	X	48994739	49002641	ENSMUSG00000039521	ENSMUSG00000039521	ENSMUSG00000039521	X	6743278	6752193	1	1		
10	X	48978871	48993931	ENSMUSG00000031143	ENSMUSG00000031143	ENSMUSG00000031143	X	6750770	6762351	1	1		
11	X	48948467	48976777	ENSMUSG00000031142	ENSMUSG00000031142	ENSMUSG00000031142	X	6764103	6792152	1	1		
12	X	48931209	48943605	ENSMUSG00000031144	ENSMUSG00000031144	ENSMUSG00000031144	X	6795645	6810210	1	1		
13	X	48918095	48929789	ENSMUSG00000031145	ENSMUSG00000031145	ENSMUSG00000031145	X	6814348	6824916	1	1		
14	X	48907187	48911766	ENSMUSG00000031147	ENSMUSG00000031147	ENSMUSG00000031147	X	6831587	6838048	1	1		
15	X	48819243	48824449	ENSMUSG00000039382	ENSMUSG00000039382	ENSMUSG00000039382	X	6879183	6885146	1	1		
16	X	48815764	48818677	ENSMUSG00000031149	ENSMUSG00000031149	ENSMUSG00000031149	X	6885505	6888023	1	1		
17	X	48803458	48814453	ENSMUSG00000031150	ENSMUSG00000031150	ENSMUSG00000031150	X	6888673	6898283	1	1		
18	X	48774099	48787934	ENSMUSG00000000134	ENSMUSG00000000134	ENSMUSG00000000134	X	6919632	6932160	1	1		
19	X	48715078	48743619	ENSMUSG00000031153	ENSMUSG00000031153	ENSMUSG00000031153	X	6946954	6977522	1	1		
20	X	48703583	48713195	ENSMUSG00000009731	ENSMUSG00000009731	ENSMUSG00000009731	X	6980802	6995239	1	1		
21	X	48664432	48699837	ENSMUSG00000031154	ENSMUSG00000031154	ENSMUSG00000031154	X	6999076	7031813	1	1		
22	X	48655403	48661245	ENSMUSG00000031155	ENSMUSG00000031155	ENSMUSG00000031155	X	7035265	7040391	1	1		
23	X	48645403	48653861	ENSMUSG00000031156	ENSMUSG00000031156	ENSMUSG00000031156	X	7041273	7050981	1	1		
24	X	48635674	48640370	ENSMUSG00000031158	ENSMUSG00000031158	ENSMUSG00000031158	X	7056357	7064611	1	1		
25	X	48574465	48578984	ENSMUSG00000039278	ENSMUSG00000039278	ENSMUSG00000039278	X	7076817	7080783	1	1		
26	X	48572227	48573492	ENSMUSG00000031160	ENSMUSG00000031160	ENSMUSG00000031160	X	7081235	7085566	1	1		
27	X	48545406	48568336	ENSMUSG00000031161	ENSMUSG00000031161	ENSMUSG00000031161	X	7087081	7104696	1	1		
28	X	48529906	48537659	ENSMUSG00000031162	ENSMUSG00000031162	ENSMUSG00000031162	X	7116225	7124835	1	1		
29	X	48509187	48516795	ENSMUSG00000031163	ENSMUSG00000031163	ENSMUSG00000031163	X	7161160	7175451	1	1		
30	X	48439930	48452347	ENSMUSG00000039231	ENSMUSG00000039231	ENSMUSG00000039231	X	7219809	7229428	1	1		
31	X	48427112	48434762	ENSMUSG00000031165	ENSMUSG00000031165	ENSMUSG00000031165	X	7238425	7247411	1	1		
32	X	48340810	48348521	ENSMUSG00000031166	ENSMUSG00000031166	ENSMUSG00000031166	X	7280263	7289397	1	1		
33	X	48282789	48305941	ENSMUSG00000039201	ENSMUSG00000039201	ENSMUSG00000039201	X	7311438	7333140	1	1		
34	X	48264490	48272048	ENSMUSG00000031168	ENSMUSG00000031168	ENSMUSG00000031168	X	7342288	7350471	1	1		
35	X	48219493	48229696	ENSMUSG00000031171	ENSMUSG00000031171	ENSMUSG00000031171	X	7395627	7409365	1	1		
36	X	48201864	48213509	ENSMUSG00000031170	ENSMUSG00000031170	ENSMUSG00000031170	X	7428353	7437133	1	1		
37	X	37315824	37421566	ENSMUSG00000047344	ENSMUSG00000047344	ENSMUSG00000047344	X	8356932	8425044	2	2		
38	X	37429931	37476322	ENSMUSG00000015342	ENSMUSG00000015342	ENSMUSG00000015342	X	8429763	8468132	2	2		
39	X	37524208	37557658	ENSMUSG00000015340	ENSMUSG00000015340	ENSMUSG00000015340	X	8592987	8626250	2	2		
40	X	37580954	37591711	ENSMUSG00000031176	ENSMUSG00000031176	ENSMUSG00000031176	X	8811229	8819942	2	2		
41	X	37735014	37735439	ENSMUSG00000040456	ENSMUSG00000040456	ENSMUSG00000040456	X	9003906	9004459	2	2		
42	X	37750779	37873013	ENSMUSG00000066783	ENSMUSG00000066783	ENSMUSG00000066783	X	9042620	9151502	2	2		
43	X	38028646	38071732	ENSMUSG00000031174	ENSMUSG00000031174	ENSMUSG00000031174	X	9315183	9373666	2	2		
44	X	38096302	38165553	ENSMUSG00000031173	ENSMUSG00000031173	ENSMUSG00000031173	X	9409318	9477983	2	2		
45	X	38545651	38550630	ENSMUSG00000008035	ENSMUSG00000008035	ENSMUSG00000008035	X	9874441	9876649	2	2		
46	X	39796015	39841602	ENSMUSG00000040363	ENSMUSG00000040363	ENSMUSG00000040363	X	11193696	11237013	2	2		
47	X	40325104	40350833	ENSMUSG00000031007	ENSMUSG00000031007	ENSMUSG00000031007	X	11744830	11773924	2	2		
48	X	40373229	40391743	ENSMUSG00000044148	ENSMUSG00000044148	ENSMUSG00000044148	X	11811843	11830517	2	2		
49	X	40392502	40479727	ENSMUSG00000064127	ENSMUSG00000064127	ENSMUSG00000064127	X	11837201	11918658	2	2		
50	X	40867767	40977988	ENSMUSG00000031010	ENSMUSG00000031010	ENSMUSG00000031010	X	12228496	12326457	2	2		
51	X	41191631	41219907	ENSMUSG00000051228	ENSMUSG00000051228	ENSMUSG00000051228	X	12642889	12644298	2	2		
52	X	41263462	41667660	ENSMUSG00000031012	ENSMUSG00000031012	ENSMUSG00000031012	X	12678006	13003515	2	2		
53	X	41433170	41441470	ENSMUSG00000040229	ENSMUSG00000040229	ENSMUSG00000040229	X	12789097	12797320	2	2		
54	X	41468352	41474332	ENSMUSG00000047678	ENSMUSG00000047678	ENSMUSG00000047678	X	12818322	12824392	2	2		
55	X	42521561	42522430	ENSMUSG00000045217	ENSMUSG00000045217	ENSMUSG00000045217	X	14267791	14268339	2	2		
56	X	43400353	43491011	ENSMUSG00000025037	ENSMUSG00000025037	ENSMUSG00000025037	X	15776657	15844677	2	2		
57	X	43510802	43626637	ENSMUSG00000040147	ENSMUSG00000040147	ENSMUSG00000040147	X	15866998	15927979	2	2		
58	X	43692966	43717694	ENSMUSG00000040138	ENSMUSG00000040138	ENSMUSG00000040138	X	16042480	16068723	2	2		
59	X	43892859	44087862	ENSMUSG00000025038	ENSMUSG00000025038	ENSMUSG00000025038	X	16289224	16438625	2	2		
60	X	44267829	44287191	ENSMUSG00000025040	ENSMUSG00000025040	ENSMUSG00000025040	X	16713528	16729256	2	2		
61	X	44588193	44589076	ENSMUSG00000025043	ENSMUSG00000025043	ENSMUSG00000025043	X	17302816	17303655	2	2		
62	X	44617701	44856791	ENSMUSG00000037369	ENSMUSG00000037369	ENSMUSG00000037369	X	17319626	17436317	2	2		
63	X	44892563	44945090	ENSMUSG00000037358	ENSMUSG00000037358	ENSMUSG00000037358	X	17575386	17618233	2	2		
64	X	46318136	46342781	ENSMUSG00000037347	ENSMUSG00000037347	ENSMUSG00000037347	X	19216529	19254480	2	2		
65	X	46349697	46503434	ENSMUSG00000037341	ENSMUSG00000037341	ENSMUSG00000037341	X	19262714	19448723	2	2		
66	X	46581319	46626737	ENSMUSG00000060090	ENSMUSG00000060090	ENSMUSG00000060090	X	19521562	19562216	2	2		
67	X	46656812	46805584	ENSMUSG00000037315	ENSMUSG00000037315	ENSMUSG00000037315	X	19582761	19676896	2	2		
68	X	46822698	46837651	ENSMUSG00000023070	ENSMUSG00000023070	ENSMUSG00000023070	X	19706777	19719046	2	2		
69	X	46935204	46959471	ENSMUSG00000001924	ENSMUSG00000001924	ENSMUSG00000001924	X	19825408	19839472	2	2		
70	X	46962576	46974340	ENSMUSG00000031065	ENSMUSG00000031065	ENSMUSG00000031065	X	19845452	19856836	2	2		
71	X	46977258	46992670	ENSMUSG00000031066	ENSMUSG00000031066	ENSMUSG00000031066	X	19860891	19877498	2	2		
72	X	47305546	47316249	ENSMUSG00000001127	ENSMUSG00000001127	ENSMUSG00000001127	X	20005537	20017478	2	2		
73	X	47316247	47364196	ENSMUSG00000037217	ENSMUSG00000037217	ENSMUSG00000037217	X	20017470	20077855	2	2		
74	X	47326634	47331131	ENSMUSG00000001131	ENSMUSG00000001131	ENSMUSG00000001131	X	20027223	20031691	2	2		
75	X	47368569	47374648	ENSMUSG00000001128	ENSMUSG00000001128	ENSMUSG00000001128	X	20082494	20088465	2	2		
76	X	47720555	47803970	ENSMUSG00000054737	ENSMUSG00000054737	ENSMUSG00000054737	X	20187023	20193734	2	2		
77	X	115216003	115220253	ENSMUSG00000068122	ENSMUSG00000068122	ENSMUSG00000068122	X	20641585	20646123	3	3		
78	X	115481818	115506653	ENSMUSG00000031089	ENSMUSG00000031089	ENSMUSG00000031089	X	20871917	20898920	3	3		
79	X	116915805	116991729	ENSMUSG00000036782	ENSMUSG00000036782	ENSMUSG00000036782	X	22376230	22472166	3	3		
80	X	117364070	117467949	ENSMUSG00000036769	ENSMUSG00000036769	ENSMUSG00000036769	X	22850076	22962949	3	3		
81	X	117513889	117704154	ENSMUSG00000031093	ENSMUSG00000031093	ENSMUSG00000031093	X	32320277	32507674	3	3		
82	X	117745563	117812530	ENSMUSG00000017057	ENSMUSG00000017057	ENSMUSG00000017057	X	32543584	32602707	3	3		
83	X	117992609	118035974	ENSMUSG00000016239	ENSMUSG00000016239	ENSMUSG00000016239	X	32759854	32798301	3	3		
84	X	118254244	118262457	ENSMUSG00000006373	ENSMUSG00000006373	ENSMUSG00000006373	X	33029670	33037528	3	3		
85	X	118606328	118623886	ENSMUSG00000044149	ENSMUSG00000044149	ENSMUSG00000044149	X	33318985	33334344	3	3		
86	X	118635630	118711361	ENSMUSG00000050379	ENSMUSG00000050379	ENSMUSG00000050379	X	33343690	33420902	3	3		
87	X	118776604	118778155	ENSMUSG00000044400	ENSMUSG00000044400	ENSMUSG00000044400	X	33480290	33481863	3	3		
88	X	118852017	118870996	ENSMUSG00000036572	ENSMUSG00000036572	ENSMUSG00000036572	X	33523125	33541676	3	3		
89	X	118943046	118961763	ENSMUSG00000016409	ENSMUSG00000016409	ENSMUSG00000016409	X	33558261	33582191	3	3		
90	X	118913828	118938707	ENSMUSG00000036551	ENSMUSG00000036551	ENSMUSG00000036551	X	33582143	33600287	3	3		
91	X	118889478	118894653	ENSMUSG00000016427	ENSMUSG00000016427	ENSMUSG00000016427	X	33619036	33622588	3	3		
92	X	118888466	118889818	ENSMUSG00000036537	ENSMUSG00000036537	ENSMUSG00000036537	X	33622726	33623908	3	3		
93	X	119268635	119276279	ENSMUSG00000048047	ENSMUSG00000048047	ENSMUSG00000048047	X	34434468	34439167	3	3		
94	X	119276534	119329349	ENSMUSG00000036502	ENSMUSG00000036502	ENSMUSG00000036502	X	34441933	34497024	3	3		
95	X	119379995	119400254	ENSMUSG00000016327	ENSMUSG00000016327	ENSMUSG00000016327	X	34560894	34581340	3	3		
96	X	119446367	119487052	ENSMUSG00000016534	ENSMUSG00000016534	ENSMUSG00000016534	X	34645986	34701081	3	3		
97	X	119544526	119593677	ENSMUSG00000031095	ENSMUSG00000031095	ENSMUSG00000031095	X	34777901	34820823	3	3		
98	X	119622204	119630195	ENSMUSG00000000355	ENSMUSG00000000355	ENSMUSG00000000355	X	34845285	34858149	3	3		
99	X	122145839	122450474	ENSMUSG00000001986	ENSMUSG00000001986	ENSMUSG00000001986	X	37646141	37923227	3	3		
100	X	122922236	123063029	ENSMUSG00000025862	ENSMUSG00000025862	ENSMUSG00000025862	X	38395700	38520801	3	3		
101	X	123307875	123334686	ENSMUSG00000005696	ENSMUSG00000005696	ENSMUSG00000005696	X	38747189	38766724	3	3		
102	X	125781428	125783450	ENSMUSG00000037086	ENSMUSG00000037086	ENSMUSG00000037086	X	41335531	41337415	3	3		
103	X	127012622	127014058	ENSMUSG00000046615	ENSMUSG00000046615	ENSMUSG00000046615	X	42573634	42574974	3	3		
104	X	128408163	128485158	ENSMUSG00000031099	ENSMUSG00000031099	ENSMUSG00000031099	X	44053997	44137146	3	3		
105	X	128501929	128554209	ENSMUSG00000001173	ENSMUSG00000001173	ENSMUSG00000001173	X	44157083	44210495	3	3		
106	X	128700631	128731206	ENSMUSG00000037005	ENSMUSG00000037005	ENSMUSG00000037005	X	44353502	44381608	3	3		
107	X	128741641	128756858	ENSMUSG00000031101	ENSMUSG00000031101	ENSMUSG00000031101	X	44391154	44406190	3	3		
108	X	128766596	128805554	ENSMUSG00000036985	ENSMUSG00000036985	ENSMUSG00000036985	X	44416598	44453505	3	3		
109	X	128944350	129019254	ENSMUSG00000036959	ENSMUSG00000036959	ENSMUSG00000036959	X	44613401	44651332	3	3		
110	X	129026530	129072372	ENSMUSG00000031103	ENSMUSG00000031103	ENSMUSG00000031103	X	44657866	44668983	3	3		
111	X	129091018	129127489	ENSMUSG00000036932	ENSMUSG00000036932	ENSMUSG00000036932	X	44719571	44758053	3	3		
112	X	128964459	129146527	ENSMUSG00000031104	ENSMUSG00000031104	ENSMUSG00000031104	X	44764097	44774859	3	3		
113	X	129164366	129230554	ENSMUSG00000036916	ENSMUSG00000036916	ENSMUSG00000036916	X	44786254	44839038	3	3		
114	X	129301934	129334605	ENSMUSG00000031105	ENSMUSG00000031105	ENSMUSG00000031105	X	44868205	44906921	3	3		
115	X	129346095	129347102	ENSMUSG00000051209	ENSMUSG00000051209	ENSMUSG00000051209	X	44917878	44918885	3	3		
116	X	129585036	129864889	ENSMUSG00000031109	ENSMUSG00000031109	ENSMUSG00000031109	X	45255149	45532869	3	3		
117	X	130019897	130051534	ENSMUSG00000036198	ENSMUSG00000036198	ENSMUSG00000036198	X	45715190	45744868	3	3		
118	X	130235166	130265778	ENSMUSG00000031111	ENSMUSG00000031111	ENSMUSG00000031111	X	46027171	46042366	3	3		
119	X	130984974	131037652	ENSMUSG00000031112	ENSMUSG00000031112	ENSMUSG00000031112	X	47114883	47135951	3	3		
120	X	131038718	131089731	ENSMUSG00000036131	ENSMUSG00000036131	ENSMUSG00000036131	X	47139810	47140661	3	3		
121	X	131164734	131179870	ENSMUSG00000050029	ENSMUSG00000050029	ENSMUSG00000050029	X	47248533	47262645	3	3		
122	X	131339858	131401399	ENSMUSG00000036109	ENSMUSG00000036109	ENSMUSG00000036109	X	47368441	47409221	3	3		
123	X	131589932	131920302	ENSMUSG00000062184	ENSMUSG00000062184	ENSMUSG00000062184	X	47634097	47926007	3	3		
124	X	131986325	131989966	ENSMUSG00000055780	ENSMUSG00000055780	ENSMUSG00000055780	X	47998586	48042380	3	3		
125	X	132261797	132377184	ENSMUSG00000031119	ENSMUSG00000031119	ENSMUSG00000031119	X	48297547	48409879	3	3		
126	X	132497439	132947588	ENSMUSG00000055653	ENSMUSG00000055653	ENSMUSG00000055653	X	48517053	48858548	3	3		
127	X	133206497	133207474	ENSMUSG00000073207	ENSMUSG00000073207	ENSMUSG00000073207	X	49035827	49044095	3	3		
128	X	133334949	133390486	ENSMUSG00000025626	ENSMUSG00000025626	ENSMUSG00000025626	X	49161226	49198205	3	3		
129	X	133421849	133462364	ENSMUSG00000025630	ENSMUSG00000025630	ENSMUSG00000025630	X	49232760	49266287	3	3		
130	X	133527534	133620179	ENSMUSG00000061082	ENSMUSG00000061082	ENSMUSG00000061082	X	49314629	49359032	3	3		
131	X	133731270	133757954	ENSMUSG00000036022	ENSMUSG00000036022	ENSMUSG00000036022	X	49488061	49513658	3	3		
132	X	133849328	133876963	ENSMUSG00000023074	ENSMUSG00000023074	ENSMUSG00000023074	X	49589648	49615119	3	3		
133	X	134118550	134133417	ENSMUSG00000071763	ENSMUSG00000071763	ENSMUSG00000071763	X	49881151	49885946	3	3		
134	X	133952634	133953440	ENSMUSG00000056304	ENSMUSG00000056304	ENSMUSG00000056304	X	49911160	49911366	3	3		
135	X	134306387	134325004	ENSMUSG00000073176	ENSMUSG00000073176	ENSMUSG00000073176	X	52693184	52711299	3	3		
136	X	134482215	134544092	ENSMUSG00000035967	ENSMUSG00000035967	ENSMUSG00000035967	X	52801661	52854623	3	3		
137	X	134871897	134883800	ENSMUSG00000061273	ENSMUSG00000061273	ENSMUSG00000061273	X	52932296	52944703	3	3		
138	X	134895328	134957082	ENSMUSG00000060681	ENSMUSG00000060681	ENSMUSG00000060681	X	52956644	53008677	3	3		
139	X	135079751	135121184	ENSMUSG00000023092	ENSMUSG00000023092	ENSMUSG00000023092	X	53078571	53140126	3	3		
140	X	135397712	135403605	ENSMUSG00000031130	ENSMUSG00000031130	ENSMUSG00000031130	X	53389858	53395542	3	3		
141	X	135406904	135422167	ENSMUSG00000067873	ENSMUSG00000067873	ENSMUSG00000067873	X	53400399	53413967	3	3		
142	X	135441977	135466632	ENSMUSG00000031131	ENSMUSG00000031131	ENSMUSG00000031131	X	53434890	53452230	3	3		
143	X	135558002	135570215	ENSMUSG00000031132	ENSMUSG00000031132	ENSMUSG00000031132	X	53558927	53570826	3	3		
144	X	135575372	135691913	ENSMUSG00000031133	ENSMUSG00000031133	ENSMUSG00000031133	X	53578271	53685513	3	3		
145	X	135939973	135941499	ENSMUSG00000036357	ENSMUSG00000036357	ENSMUSG00000036357	X	53846588	53850485	3	3		
146	X	136476012	136481921	ENSMUSG00000067860	ENSMUSG00000067860	ENSMUSG00000067860	X	54377965	54382688	3	3		
147	X	137392569	137393621	ENSMUSG00000046180	ENSMUSG00000046180	ENSMUSG00000046180	X	55265616	55267088	3	3		
148	X	137541401	137894912	ENSMUSG00000031137	ENSMUSG00000031137	ENSMUSG00000031137	X	55408937	55481220	3	3		
149	X	138440561	138473283	ENSMUSG00000031138	ENSMUSG00000031138	ENSMUSG00000031138	X	56346248	56377542	3	3		
150	X	138491595	138601981	ENSMUSG00000031139	ENSMUSG00000031139	ENSMUSG00000031139	X	56402749	56525873	3	3		
151	X	138636174	138742113	ENSMUSG00000062949	ENSMUSG00000062949	ENSMUSG00000062949	X	56575935	56668337	3	3		
152	X	139412818	139415334	ENSMUSG00000045179	ENSMUSG00000045179	ENSMUSG00000045179	X	57152743	57153870	3	3		
153	X	140097596	140098976	ENSMUSG00000057615	ENSMUSG00000057615	ENSMUSG00000057615	X	57970279	57971623	3	3		
154	X	21868612	21922874	ENSMUSG00000067829	ENSMUSG00000067829	ENSMUSG00000067829	X	59036250	59037350	out-of-place			
155	X	142543610	142551262	ENSMUSG00000046699	ENSMUSG00000046699	ENSMUSG00000046699	X	60530109	60537661	3	4	here	Presence of a singleton made AutoGRAPH to break the block
156	X	144707173	144715050	ENSMUSG00000036790	ENSMUSG00000036790	ENSMUSG00000036790	X	62914570	62917110	3	4	presence of singletons	1 -> 2
157	X	146801262	146840337	ENSMUSG00000000838	ENSMUSG00000000838	ENSMUSG00000000838	X	64939220	64978630	3	4		
158	X	146870541	146915879	ENSMUSG00000062170	ENSMUSG00000062170	ENSMUSG00000062170	X	65022599	65065107	3	4		
159	X	147389831	147889899	ENSMUSG00000031189	ENSMUSG00000031189	ENSMUSG00000031189	X	65620996	66128702	3	4		
160	X	148366427	148394782	ENSMUSG00000035847	ENSMUSG00000035847	ENSMUSG00000035847	X	66603734	66625640	3	4		
161	X	148486015	148521375	ENSMUSG00000073139	ENSMUSG00000073139	ENSMUSG00000073139	X	66720316	66737571	3	4		
162	X	149487727	149592272	ENSMUSG00000031337	ENSMUSG00000031337	ENSMUSG00000031337	X	67471323	67574530	3	4		
163	X	149612505	149684234	ENSMUSG00000015214	ENSMUSG00000015214	ENSMUSG00000015214	X	67625316	67679752	3	4		
164	X	149685468	149817837	ENSMUSG00000035776	ENSMUSG00000035776	ENSMUSG00000035776	X	67680621	67753160	3	4		
165	X	150095783	150100595	ENSMUSG00000056380	ENSMUSG00000056380	ENSMUSG00000056380	X	67924323	67928645	3	4		
166	X	150617435	150625052	ENSMUSG00000033361	ENSMUSG00000033361	ENSMUSG00000033361	X	68219218	68220304	3	4		
167	X	150635163	150642322	ENSMUSG00000053593	ENSMUSG00000053593	ENSMUSG00000053593	X	68225706	68241766	3	4		
168	X	150653874	150664692	ENSMUSG00000005864	ENSMUSG00000005864	ENSMUSG00000005864	X	68244595	68262938	3	4		
169	X	150872252	150893807	ENSMUSG00000031340	ENSMUSG00000031340	ENSMUSG00000031340	X	68510152	68527441	3	4		
170	X	151085362	151370486	ENSMUSG00000031343	ENSMUSG00000031343	ENSMUSG00000031343	X	68686537	68908906	3	4		
171	X	151557293	151572481	ENSMUSG00000031344	ENSMUSG00000031344	ENSMUSG00000031344	X	69078156	69091264	3	4		
172	X	151746173	151749977	ENSMUSG00000031347	ENSMUSG00000031347	ENSMUSG00000031347	X	69166924	69169566	3	4		
173	X	151750167	151788929	ENSMUSG00000031349	ENSMUSG00000031349	ENSMUSG00000031349	X	69171241	69211248	3	4		
174	X	151839560	151892671	ENSMUSG00000031351	ENSMUSG00000031351	ENSMUSG00000031351	X	69252032	69281663	3	4		
175	X	151908024	151911414	ENSMUSG00000050424	ENSMUSG00000050424	ENSMUSG00000050424	X	69288466	69289806	3	4		
176	X	151975963	151978288	ENSMUSG00000046287	ENSMUSG00000046287	ENSMUSG00000046287	X	69317507	69320911	3	4		
177	X	152263320	152267171	ENSMUSG00000031365	ENSMUSG00000031365	ENSMUSG00000031365	X	69595355	69611684	3	4		
178	X	152336975	152340064	ENSMUSG00000031374	ENSMUSG00000031374	ENSMUSG00000031374	X	69663823	69679707	3	4		
179	X	152366530	152389239	ENSMUSG00000031371	ENSMUSG00000031371	ENSMUSG00000031371	X	69690033	69711749	3	4		
180	X	152413591	152428206	ENSMUSG00000031375	ENSMUSG00000031375	ENSMUSG00000031375	X	69736364	69748653	3	4		
181	X	152436328	152499241	ENSMUSG00000031376	ENSMUSG00000031376	ENSMUSG00000031376	X	69755806	69823725	3	4		
182	X	152561182	152569975	ENSMUSG00000031383	ENSMUSG00000031383	ENSMUSG00000031383	X	69892161	69896240	3	4		
183	X	152588384	152592451	ENSMUSG00000002012	ENSMUSG00000002012	ENSMUSG00000002012	X	69908714	69912548	3	4		
184	X	152619148	152643364	ENSMUSG00000002015	ENSMUSG00000002015	ENSMUSG00000002015	X	69938903	69968895	3	4		
185	X	152643517	152663410	ENSMUSG00000031378	ENSMUSG00000031378	ENSMUSG00000031378	X	69969429	69991252	3	4		
186	X	152682905	152697990	ENSMUSG00000031385	ENSMUSG00000031385	ENSMUSG00000031385	X	70011278	70025164	3	4		
187	X	152699712	152704378	ENSMUSG00000002007	ENSMUSG00000002007	ENSMUSG00000002007	X	70027171	70031645	3	4		
188	X	152712165	152717147	ENSMUSG00000002014	ENSMUSG00000002014	ENSMUSG00000002014	X	70039782	70043542	3	4		
189	X	152720817	152749216	ENSMUSG00000002006	ENSMUSG00000002006	ENSMUSG00000002006	X	70046077	70077689	3	4		
190	X	152780163	152804794	ENSMUSG00000031391	ENSMUSG00000031391	ENSMUSG00000031391	X	70106693	70133479	3	4		
191	X	152823383	152825814	ENSMUSG00000031390	ENSMUSG00000031390	ENSMUSG00000031390	X	70144822	70147146	3	4		
192	X	152826024	152844908	ENSMUSG00000031389	ENSMUSG00000031389	ENSMUSG00000031389	X	70147076	70163949	3	4		

193	X	152853917	152863426	ENSMUSG00000031387	ENSMUSG00000031387	ENSMUSG00000031387	X	70174842	70183538	3	4
194	X	152866204	152889829	ENSMUSG00000031386	ENSMUSG00000031386	ENSMUSG00000031386	X	70195515	70219035	3	4
195	X	152929145	152938625	ENSMUSG00000031392	ENSMUSG00000031392	ENSMUSG00000031392	X	70267703	70276633	3	4
196	X	152948879	153016371	ENSMUSG00000031393	ENSMUSG00000031393	ENSMUSG00000031393	X	70288072	70338332	3	4
197	X	153177218	153211894	ENSMUSG00000031397	ENSMUSG00000031397	ENSMUSG00000031397	X	70429979	70461220	3	4
198	X	153230159	153256123	ENSMUSG00000031328	ENSMUSG00000031328	ENSMUSG00000031328	X	70476183	70499156	3	4
199	X	153260751	153263077	ENSMUSG00000001964	ENSMUSG00000001964	ENSMUSG00000001964	X	70507559	70510466	3	4
200	X	153282773	153293621	ENSMUSG00000019088	ENSMUSG00000019088	ENSMUSG00000019088	X	70525938	70535053	3	4
201	X	153293071	153303259	ENSMUSG00000009995	ENSMUSG00000009995	ENSMUSG00000009995	X	70535470	70542860	3	4
202	X	153310208	153318056	ENSMUSG00000019087	ENSMUSG00000019087	ENSMUSG00000019087	X	70549826	70557410	3	4
203	X	153318460	153325008	ENSMUSG00000015291	ENSMUSG00000015291	ENSMUSG00000015291	X	70557732	70564580	3	4
204	X	153325696	153332202	ENSMUSG00000001962	ENSMUSG00000001962	ENSMUSG00000001962	X	70565935	70572228	3	4
205	X	153339815	153355183	ENSMUSG00000031398	ENSMUSG00000031398	ENSMUSG00000031398	X	70581819	70596483	3	4
206	X	153368842	153372189	ENSMUSG00000032806	ENSMUSG00000032806	ENSMUSG00000032806	X	70621941	70626014	3	4
207	X	153387734	153397754	ENSMUSG00000031399	ENSMUSG00000031399	ENSMUSG00000031399	X	70638060	70643837	3	4
208	X	153423653	153446454	ENSMUSG00000004221	ENSMUSG00000004221	ENSMUSG00000004221	X	70677332	70701825	3	4
209	X	153556724	153632526	ENSMUSG00000032750	ENSMUSG00000032750	ENSMUSG00000032750	X	71241270	71337625	3	4
210	X	153644229	153659158	ENSMUSG00000031403	ENSMUSG00000031403	ENSMUSG00000031403	X	71348804	71362284	3	4
211	X	153660153	153686977	ENSMUSG00000031402	ENSMUSG00000031402	ENSMUSG00000031402	X	71362453	71383669	3	4
212	X	153704817	153714140	ENSMUSG00000073094	ENSMUSG00000073094	ENSMUSG00000073094	X	71398777	71416473	3	4
213	X	153717263	153904192	ENSMUSG00000031196	ENSMUSG00000031196	ENSMUSG00000031196	X	71425575	71635036	3	4
214	X	153943098	153952829	ENSMUSG00000031200	ENSMUSG00000031200	ENSMUSG00000031200	X	71663164	71669263	3	4
215	X	153952997	154004505	ENSMUSG00000031201	ENSMUSG00000031201	ENSMUSG00000031201	X	71669439	71706720	3	4
216	X	154097744	154121291	ENSMUSG00000031197	ENSMUSG00000031197	ENSMUSG00000031197	X	71767033	71787655	3	4
217	X	154140720	154147068	ENSMUSG00000031202	ENSMUSG00000031202	ENSMUSG00000031202	X	71824778	71830945	3	4
218	X	114701740	114791241	ENSMUSG00000016382	ENSMUSG00000016382	ENSMUSG00000016382	X	72038383	72127258	Singleton	
219	X	37093469	37201469	ENSMUSG00000047996	ENSMUSG00000047996	ENSMUSG00000047996	X	74705406	74736631	4	5
220	X	36238869	36313099	ENSMUSG00000071735	ENSMUSG00000071735	ENSMUSG00000071735	X	75519297	75571798	4	5
221	X	35847806	35918185	ENSMUSG00000073077	ENSMUSG00000073077	ENSMUSG00000073077	X	75735314	75769941	4	5
222	X	34555104	34585326	ENSMUSG00000025666	ENSMUSG00000025666	ENSMUSG00000025666	X	77323435	77349182	4	5
223	X	31047257	33139632	ENSMUSG00000045103	ENSMUSG00000045103	ENSMUSG00000045103	X	79389349	81450263	4	5
224	X	30755480	30795240	ENSMUSG00000035476	ENSMUSG00000035476	ENSMUSG00000035476	X	81826741	81887033	4	5
225	X	30486862	30505882	ENSMUSG00000025058	ENSMUSG00000025058	ENSMUSG00000025058	X	82074664	82141970	4	5
226	X	30232244	30237636	ENSMUSG00000025056	ENSMUSG00000025056	ENSMUSG00000025056	X	82444494	82448663	4	5
227	X	30158474	30165528	ENSMUSG00000071732	ENSMUSG00000071732	ENSMUSG00000071732	X	82570132	82598375	4	5
228	X	27907570	27909393	ENSMUSG00000035395	ENSMUSG00000035395	ENSMUSG00000035395	X	85656997	85659240	4	5
229	X	26066381	26068773	ENSMUSG00000067649	ENSMUSG00000067649	ENSMUSG00000067649	X	88372370	88373353	4	5
230	X	24931732	24943986	ENSMUSG00000035277	ENSMUSG00000035277	ENSMUSG00000035277	X	89539365	89551080	4	5
231	X	24621957	24925024	ENSMUSG00000006678	ENSMUSG00000006678	ENSMUSG00000006678	X	89557486	89884874	4	5
232	X	24486125	24575274	ENSMUSG00000035246	ENSMUSG00000035246	ENSMUSG00000035246	X	89927831	90002667	4	5
233	X	24393259	24462463	ENSMUSG00000035232	ENSMUSG00000035232	ENSMUSG00000035232	X	90017326	90084894	4	5
234	X	23982986	24006007	ENSMUSG00000035150	ENSMUSG00000035150	ENSMUSG00000035150	X	90441422	90465370	4	5
235	X	23911758	23955224	ENSMUSG00000043929	ENSMUSG00000043929	ENSMUSG00000043929	X	90487649	90526234	4	5
236	X	51653438	51662188	ENSMUSG00000025151	ENSMUSG00000025151	ENSMUSG00000025151	X	90788189	90794793	Singleton	
237	X	62486674	62487423	ENSMUSG00000071722	ENSMUSG00000071722	ENSMUSG00000071722	X	91228164	91228913	5	6
238	X	62771572	62891718	ENSMUSG00000025656	ENSMUSG00000025656	ENSMUSG00000025656	X	91254692	91333740	5	6
239	X	63360801	63367212	ENSMUSG00000031204	ENSMUSG00000031204	ENSMUSG00000031204	X	91672918	91680848	5	6
240	X	64052986	64113089	ENSMUSG00000035062	ENSMUSG00000035062	ENSMUSG00000035062	X	91841912	91861151	5	6
241	X	64625440	64644491	ENSMUSG00000035045	ENSMUSG00000035045	ENSMUSG00000035045	X	92101815	92127402	5	6
242	X	64649187	64671361	ENSMUSG00000057421	ENSMUSG00000057421	ENSMUSG00000057421	X	92138030	92159678	5	6
243	X	64804236	64878517	ENSMUSG00000031207	ENSMUSG00000031207	ENSMUSG00000031207	X	92344584	92369353	5	6
244	X	65158305	65176674	ENSMUSG00000044206	ENSMUSG00000044206	ENSMUSG00000044206	X	92449922	92496157	5	6
245	X	65299388	65405434	ENSMUSG00000031209	ENSMUSG00000031209	ENSMUSG00000031209	X	92658180	92777204	5	6
246	X	65736051	65752587	ENSMUSG00000034457	ENSMUSG00000034457	ENSMUSG00000034457	X	93538638	93579891	5	6
247	X	66681190	66867186	ENSMUSG00000046532	ENSMUSG00000046532	ENSMUSG00000046532	X	94352469	94519866	5	6
248	X	67178911	67570372	ENSMUSG00000031214	ENSMUSG00000031214	ENSMUSG00000031214	X	94760234	95093704	5	6
249	X	67634890	67673852	ENSMUSG00000047694	ENSMUSG00000047694	ENSMUSG00000047694	X	95140500	95151740	5	6
250	X	67784335	67862402	ENSMUSG00000031216	ENSMUSG00000031216	ENSMUSG00000031216	X	95245384	95277449	5	6
251	X	67965556	67978727	ENSMUSG00000031217	ENSMUSG00000031217	ENSMUSG00000031217	X	95338849	95351710	5	6
252	X	68641803	68669072	ENSMUSG00000071719	ENSMUSG00000071719	ENSMUSG00000071719	X	96023788	96049064	5	6
253	X	68752878	69172184	ENSMUSG00000059327	ENSMUSG00000059327	ENSMUSG00000059327	X	96178500	96599875	5	6
254	X	69177117	69186513	ENSMUSG00000031220	ENSMUSG00000031220	ENSMUSG00000031220	X	96604940	96615281	5	6
255	X	69199066	69200754	ENSMUSG00000051582	ENSMUSG00000051582	ENSMUSG00000051582	X	96631732	96632604	5	6
256	X	69314061	69342278	ENSMUSG00000067597	ENSMUSG00000067597	ENSMUSG00000067597	X	96727557	96748612	5	6
257	X	69371271	69376865	ENSMUSG00000015665	ENSMUSG00000015665	ENSMUSG00000015665	X	96776548	96780607	5	6
258	X	69394745	69396339	ENSMUSG00000044359	ENSMUSG00000044359	ENSMUSG00000044359	X	96792873	96797588	5	6
259	X	69404927	69418415	ENSMUSG00000060890	ENSMUSG00000060890	ENSMUSG00000060890	X	96808275	96821106	5	6
260	X	69422684	69426597	ENSMUSG00000015668	ENSMUSG00000015668	ENSMUSG00000015668	X	96825605	96828657	5	6
261	X	69559716	69569959	ENSMUSG00000019359	ENSMUSG00000019359	ENSMUSG00000019359	X	96932659	96941614	5	6
262	X	69665516	70034121	ENSMUSG00000009670	ENSMUSG00000009670	ENSMUSG00000009670	X	97041367	97262358	5	6
263	X	70062157	70067671	ENSMUSG00000031297	ENSMUSG00000031297	ENSMUSG00000031297	X	97281940	97288071	5	6
264	X	70192907	70204898	ENSMUSG00000046032	ENSMUSG00000046032	ENSMUSG00000046032	X	97414680	97425211	5	6
265	X	70232772	70240110	ENSMUSG00000042903	ENSMUSG00000042903	ENSMUSG00000042903	X	97457540	97461957	5	6
266	X	70240562	70243179	ENSMUSG00000073048	ENSMUSG00000073048	ENSMUSG00000073048	X	97464096	97466711	5	6
267	X	70243984	70248128	ENSMUSG00000031304	ENSMUSG00000031304	ENSMUSG00000031304	X	97467097	97470925	5	6
268	X	70255296	70278872	ENSMUSG00000031302	ENSMUSG00000031302	ENSMUSG00000031302	X	97477612	97524067	5	6
269	X	70351769	70361775	ENSMUSG00000047797	ENSMUSG00000047797	ENSMUSG00000047797	X	97580056	97588350	5	6
270	X	70376201	70391635	ENSMUSG00000031310	ENSMUSG00000031310	ENSMUSG00000031310	X	97607104	97623568	5	6
271	X	70438309	70441946	ENSMUSG00000031312	ENSMUSG00000031312	ENSMUSG00000031312	X	97651884	97656498	5	6
272	X	70669658	70712472	ENSMUSG00000034160	ENSMUSG00000034160	ENSMUSG00000034160	X	97842783	97887068	5	6
273	X	70752491	70755085	ENSMUSG00000050232	ENSMUSG00000050232	ENSMUSG00000050232	X	97934255	97936866	5	6
274	X	71263685	71268476	ENSMUSG00000049191	ENSMUSG00000049191	ENSMUSG00000049191	X	98269263	98274023	5	6
275	X	71341232	71375583	ENSMUSG00000051220	ENSMUSG00000051220	ENSMUSG00000051220	X	98345677	98359752	5	6
276	X	71438222	71443762	ENSMUSG00000051159	ENSMUSG00000051159	ENSMUSG00000051159	X	98450100	98454882	5	6
277	X	71466092	71709378	ENSMUSG00000067567	ENSMUSG00000067567	ENSMUSG00000067567	X	98510356	98707783	5	6
278	X	71717202	71850831	ENSMUSG00000034055	ENSMUSG00000034055	ENSMUSG00000034055	X	98718393	98846917	5	6
279	X	72583813	72591806	ENSMUSG00000031326	ENSMUSG00000031326	ENSMUSG00000031326	X	99524117	99532943	5	6
280	X	72699769	72820971	ENSMUSG00000031327	ENSMUSG00000031327	ENSMUSG00000031327	X	99559222	99594954	5	6
281	X	73440750	73441591	ENSMUSG00000031330	ENSMUSG00000031330	ENSMUSG00000031330	X	99833305	99834383	5	6
282	X	73557810	73670468	ENSMUSG00000033965	ENSMUSG00000033965	ENSMUSG00000033965	X	99900133	100024643	5	6
283	X	73721777	73751177	ENSMUSG00000056537	ENSMUSG00000056537	ENSMUSG00000056537	X	100159886	100184003	5	6
284	X	73869409	74062045	ENSMUSG00000046449	ENSMUSG00000046449	ENSMUSG00000046449	X	100285912	100291678	5	6
285	X	74189834	74292857	ENSMUSG00000031333	ENSMUSG00000031333	ENSMUSG00000031333	X	100486618	100616553	5	6
286	X	74410648	74441160	ENSMUSG00000073016	ENSMUSG00000073016	ENSMUSG00000073016	X	100698465	100708981	5	6
287	X	74504987	74659815	ENSMUSG00000033906	ENSMUSG00000033906	ENSMUSG00000033906	X	100741269	100873522	5	6
288	X	74919548	74921796	ENSMUSG00000031224	ENSMUSG00000031224	ENSMUSG00000031224	X	101058191	101059762	5	6
289	X	75309173	75314442	ENSMUSG00000031226	ENSMUSG00000031226	ENSMUSG00000031226	X	101282494	101287905	5	6
290	X	75564521	75568148	ENSMUSG00000031227	ENSMUSG00000031227	ENSMUSG00000031227	X	101323115	101326628	5	6
291	X	76596303	76598669	ENSMUSG00000031230	ENSMUSG00000031230	ENSMUSG00000031230	X	101967260	101976899	5	6
292	X	76647034	76928358	ENSMUSG00000031229	ENSMUSG00000031229	ENSMUSG00000031229	X	102002894	102102714	5	6
293	X	76968517	77037657	ENSMUSG00000031232	ENSMUSG00000031232	ENSMUSG00000031232	X	102176891	102214482	5	6
294	X	77041591	77049526	ENSMUSG00000031231	ENSMUSG00000031231	ENSMUSG00000031231	X	102218419	102225167	5	6
295	X	77104778	77192548	ENSMUSG00000033792	ENSMUSG00000033792	ENSMUSG00000033792	X	102230043	102327470	5	6
296	X	77271901	77281859	ENSMUSG00000047242	ENSMUSG00000047242	ENSMUSG00000047242	X	102409593	102422352	5	6
297	X	77413617	77469636	ENSMUSG00000052821	ENSMUSG00000052821	ENSMUSG00000052821	X	102780539	102781594	5	6
298	X	77798222	77801481	ENSMUSG00000047686	ENSMUSG00000047686	ENSMUSG00000047686	X	103039801	103043362	5	6
299	X	77896957	77899247	ENSMUSG00000049929	ENSMUSG00000049929	ENSMUSG00000049929	X	103123354	103135153	5	6
300	X	78087485	78104093	ENSMUSG00000050921	ENSMUSG00000050921	ENSMUSG00000050921	X	103291211	103307689	5	6
301	X	78227089	78228101	ENSMUSG00000054293	ENSMUSG00000054293	ENSMUSG00000054293	X	103352334	103375437	5	6
302	X	78313125	78314382	ENSMUSG00000073008	ENSMUSG00000073008	ENSMUSG00000073008	X	103458745	103498026	5	6
303	X	78502541	78509703	ENSMUSG00000031239	ENSMUSG00000031239	ENSMUSG00000031239	X	103599914	103606079	5	6
304	X	79156911	79173528	ENSMUSG00000031241	ENSMUSG00000031241	ENSMUSG00000031241	X	103870683	103891694	5	6
305	X	79477659	79587461	ENSMUSG00000073007	ENSMUSG00000073007	ENSMUSG00000073007	X	104073048	104074190	5	6
306	X	79813009	79951843	ENSMUSG00000063663	ENSMUSG00000063663	ENSMUSG00000063663	X	104945738	105036961	5	6
307	X	80255856	80344097	ENSMUSG00000031245	ENSMUSG00000031245	ENSMUSG00000031245	X	105207828	105213608	5	6
308	X	80344098	80440702	ENSMUSG00000031246	ENSMUSG00000031246	ENSMUSG00000031246	X	105352634	105362881	5	6
309	X	82649941	82651431	ENSMUSG00000056854	ENSMUSG00000056854	ENSMUSG00000056854	X	107017184	107018269	5	6
310	X	83002810	83028361	ENSMUSG00000073001	ENSMUSG00000073001	ENSMUSG00000073001	X	107313177	107325813	5	6
311	X	83205640	83329571	ENSMUSG00000025665	ENSMUSG00000025665	ENSMUSG00000025665	X	107505533	107603726	5	6
312	X	83459541	83644117	ENSMUSG00000034551	ENSMUSG00000034551	ENSMUSG00000034551	X	107692148	107814011	5	6
313	X	84145559	84229615	ENSMUSG00000025525	ENSMUSG00000025525	ENSMUSG00000025525	X	108428302	108489698	5	6
314	X	84385694	84415022	ENSMUSG00000025529	ENSMUSG00000025529	ENSMUSG00000025529	X	108721238	108750497	5	6
315	X	84423777	84521378	ENSMUSG00000034607	ENSMUSG00000034607	ENSMUSG00000034607	X	108755371	108815595	5	6
316	X	85002841	85189222	ENSMUSG00000025531	ENSMUSG00000025531	ENSMUSG00000025531	X	109160388	109276515	5	6
317	X	85290281	85973814	ENSMUSG00000025592	ENSMUSG00000025592	ENSMUSG00000025592	X	109415200	109953330	5	6
318	X	86659473	86811252	ENSMUSG00000025597	ENSMUSG00000025597	ENSMUSG00000025597	X	110591464	110677642	5	6
319	X	87888882	87896442	ENSMUSG00000072995	ENSMUSG00000072995	ENSMUSG00000072995	X	112565888	112595727	5	6
320	X	90576253	90580238	ENSMUSG00000034732	ENSMUSG00000034732	ENSMUSG00000034732	X	116044140	116047109	5	6
321	X	92812585	92815223	ENSMUSG00000055733	ENSMUSG00000055733	ENSMUSG00000055733	X	118512329	118513963	5	6
322	X	92815861	92853917	ENSMUSG00000067420	ENSMUSG00000067420	ENSMUSG00000067420	X	118569312	118590904	5	6
323	X	95826367	96740972	ENSMUSG00000034480	ENSMUSG00000034480	ENSMUSG00000034480	X	125096143	125675204	5	6
324	X	99437576	99550251	ENSMUSG00000051323	ENSMUSG00000051323	ENSMUSG00000051323	X	128933966	129033614	5	6
325	X	99726455	99741538	ENSMUSG00000031250	ENSMUSG00000031250	ENSMUSG00000031250	X	129197358	129211927	5	6
326	X	99785871	99812952	ENSMUSG00000031253	ENSMUSG00000031253	ENSMUSG00000031253	X	129255046	129278164	5	6
327	X	99816147	99843759	ENSMUSG00000031255	ENSMUSG00000031255	ENSMUSG00000031255	X	129282733	129328133	5	6
328	X	99962004	99982577	ENSMUSG00000031256	ENSMUSG00000031256	ENSMUSG00000031256	X	129405699	129433171	5	6
329	X	99984969	100015990	ENSMUSG00000031257	ENSMUSG00000031257	ENSMUSG00000031257	X	129433496	129458058	5	6
330	X	100055092	100070554	ENSMUSG00000031258	ENSMUSG00000031258	ENSMUSG00000031258	X	129495395	129508278	5	6
331	X	100111369	100132357	ENSMUSG00000052549	ENSMUSG00000052549	ENSMUSG00000052549	X	129536186	129553773	5	6
332	X	100152023	100193501	ENSMUSG00000067369	ENSMUSG00000067369	ENSMUSG00000067369	X	129569305	129623290	5	6
333	X	100220365	100238009	ENSMUSG00000033578	ENSMUSG00000033578	ENSMUSG00000033578	X	129641575	129652319	5	6
334	X	100242082	100305326	ENSMUSG00000031262	ENSMUSG00000031262	ENSMUSG00000031262	X	129654505	129708986	5	6
335	X	100361641	100406142	ENSMUSG00000000223	ENSMUSG00000000223	ENSMUSG00000000223	X	129751187	129799749	5	6
336	X	100491094	100527839	ENSMUSG00000031264	ENSMUSG00000031264	ENSMUSG00000031264	X	129888686	129929418	5	6
337	X	100539453	100549607	ENSMUSG00000031266	ENSMUSG00000031266	ENSMUSG00000031266	X	129934521	129947287	5	6
338	X	100549847	100555762	ENSMUSG00000045427	ENSMUSG00000045427	ENSMUSG00000045427	X	129947609	129953405	5	6
339	X	100692170	100696339	ENSMUSG00000033460	ENSMUSG00000033460	ENSMUSG00000033460	X	130064334	130068274	5	6
340	X	100796923	100801485	ENSMUSG00000033436	ENSMUSG00000033436	ENSMUSG00000033436	X	130150511	130155585	5	6
341	X	101023918	101073393	ENSMUSG00000052676	ENSMUSG00000052676	ENSMUSG00000052676	X	130318990	130371656	5	6
342	X	101740752	101745743	ENSMUSG00000072969	ENSMUSG00000072969	ENSMUSG00000072969	X	131089056	131093684	5	6
343	X	101792973	101800041	ENSMUSG00000043384	ENSMUSG00000043384	ENSMUSG00000043384	X	131142503	131149838	5	6
344	X	101853760	101859316	ENSMUSG00000072966	ENSMUSG00000072966	ENSMUSG00000072966	X	131185420	131191090	5	6
345	X	101862310	101894022	ENSMUSG00000072964	ENSMUSG00000072964	ENSMUSG00000072964	X	131235266	131237439	5	6
346	X	102217394	102234709	ENSMUSG00000057000	ENSMUSG00000057000	ENSMUSG00000057000	X	131419624	131427488	5	6
347	X	102394579	102396787	ENSMUSG00000051579	ENSMUSG00000051579	ENSMUSG00000051579	X	131515346	131518542	5	6
348	X	102498029	102500053	ENSMUSG00000042712	ENSMUSG00000042712	ENSMUSG00000042712	X	131591484	131593488	5	6
349	X	102517910	102519652	ENSMUSG00000046432	ENSMUSG00000046432	ENSMUSG00000046432	X	131616691	131618322	5	6
350	X	102770288	102772031	ENSMUSG00000049536	ENSMUSG00000049536	ENSMUSG00000049536	X	132054396	132056235	5	6
351	X	102848808	102870200	ENSMUSG00000018595	ENSMUSG00000018595	ENSMUSG00000018595	X	132104038	132126085	5	6
352	X	102918410	102934201	ENSMUSG00000031425	ENSMUSG00000031425	ENSMUSG00000031425	X	132169200	132184124	5	6
353	X	102963914	102973838	ENSMUSG00000043463	ENSMUSG00000043463	ENSMUSG00000043463	X	132204515	132214930	5	6
354	X	103230554	103288364	ENSMUSG00000044348	ENSMUSG00000044348	ENSMUSG00000044348	X	132329034	132329954	5	6
355	X	103297661	103322781	ENSMUSG00000042595	ENSMUSG00000042595	ENSMUSG00000042595	X	132395993	132423866	5	6
356	X	103381380	103386270	ENSMUSG00000023443	ENSMUSG00000023443	ENSMUSG00000023443	X	132461762	132466690	5	6
357	X	103697652	104898478	ENSMUSG00000059203	ENSMUSG00000059203	ENSMUSG00000059203	X	132998084	134192988	5	6
358	X	104953192	105089257	ENSMUSG00000052854	ENSMUSG00000052854	ENSMUSG00000052854	X	134260869	134354403	5	6
359	X	105163853	105169385	ENSMUSG00000031271	ENSMUSG00000031271	ENSMUSG00000031271	X	134426520	134431558	5	6
360	X	105298954	105339605	ENSMUSG00000042515	ENSMUSG00000042515	ENSMUSG00000042515	X	134559535	134584696	5	6
361	X	105741858	105809328	ENSMUSG00000042498	ENSMUSG00000042498	ENSMUSG00000042498	X	134826807	134900944	5	6
362	X	105856550	105926900	ENSMUSG00000031438	ENSMUSG00000031438	ENSMUSG00000031438	X	134956984	135019499	5	6
363	X	105932566	106006031	ENSMUSG00000042473	ENSMUSG00000042473	ENSMUSG00000042473	X	135071544	135098202	5	6
364	X	106029951	106033203	ENSMUSG00000072945	ENSMUSG00000072945	ENSMUSG00000072945	X	135126076	135128675	5	6
365	X	106030050	106059459	ENSMUSG00000047230	ENSMUSG00000047230	ENSMUSG00000047230	X	135147192	135157748	5	6
366	X	106070625	106130130	ENSMUSG00000031434	ENSMUSG00000031434	ENSMUSG00000031434	X	135169039	135218018	5	6
367	X	106197024	106248690	ENSMUSG00000031433	ENSMUSG00000031433	ENSMUSG00000031433	X	135290594	135343072	5	6
368	X	106253344	106336306	ENSMUSG00000072944	ENSMUSG00000072944	ENSMUSG00000072944	X	135354039	135408916	5	6
369	X	106656532	106733259	ENSMUSG00000042425	ENSMUSG00000042425	ENSMUSG00000042425	X	135730352	135779973	5	6
370	X	106843110	106905858	ENSMUSG00000031431	ENSMUSG00000031431	ENSMUSG00000031431	X	135885901	135889549	5	6
371	X	107174856	107209069	ENSMUSG00000031430	ENSMUSG00000031430	ENSMUSG00000031430	X	136253984	136285322	5	6
372	X	107214093	107221504	ENSMUSG00000031429	ENSMUSG00000031429	ENSMUSG00000031429	X	136294899	136303045	5	6
373	X	107285502	107569367	ENSMUSG00000031273	ENSMUSG00000031273	ENSMUSG00000031273	X	136413086	136573853	5	6
374	X	107569810	107827431	ENSMUSG00000031274	ENSMUSG00000031274	ENSMUSG00000031274	X	136722103	136934489	5	6
375	X	107862368	107866295	ENSMUSG00000054667	ENSMUSG00000054667	ENSMUSG00000054667	X	136957366	136971585	5	6
376	X	108502791	108611957	ENSMUSG00000042282	ENSMUSG00000042282	ENSMUSG00000042282	X	137327528	137443299	5	6
377	X	108753585	108755057	ENSMUSG00000057263	ENSMUSG00000057263	ENSMUSG00000057263	X	137551962	137552393	5	6
378	X	108771222	108863277	ENSMUSG00000031278	ENSMUSG00000031278	ENSMUSG00000031278	X	137564361	137636903	5	6
379	X	109132979	109307672	ENSMUSG00000047045	ENSMUSG00000047045	ENSMUSG00000047045	X	137928245	138089716	5	6
380	X	109324070	109448123	ENSMUSG00000042225	ENSMUSG00000042225	ENSMUSG00000042225	X	138099842	138213096	5	6
381	X	109575114	109586210	ENSMUSG00000042217	ENSMUSG00000042217	ENSMUSG00000042217	X	138345962	138349981	5	6
382	X	109805133	109925649	ENSMUSG00000031283	ENSMUSG00000031283	ENSMUSG00000031283	X	138532043	138636467	5	6
383	X	110252988	110350331	ENSMUSG00000031284	ENSMUSG00000031284	ENSMUSG00000031284	X	138765079	139038221	5	6
384	X	110374987	110400407	ENSMUSG00000067276	ENSMUSG00000067276	ENSMUSG00000067276	X	139048604	139073663	5	6
385	X	110423663	110541030	ENSMUSG00000031285	ENSMUSG00000031285	ENSMUSG00000031285	X	139115578	139179418	5	6
386	X	110904198	111212660	ENSMUSG00000041710	ENSMUSG00000041710	ENSMUSG00000041710	X	139628039	139934548	5	6
387	X	111011781	111033867	ENSMUSG00000072934	ENSMUSG00000072934	ENSMUSG00000072934	X	139720253	139720585	5	6

388	X	111760532	111809938	ENSMUSG00000041700	ENSMUSG00000041700	ENSMUSG00000041700	X	140536727	140595262	5	6
389	X	111908451	111953010	ENSMUSG00000041688	ENSMUSG00000041688	ENSMUSG00000041688	X	140698237	140733462	5	6
390	X	113724807	114050880	ENSMUSG00000041380	ENSMUSG00000041380	ENSMUSG00000041380	X	142208881	142443644	5	6
391	X	114144794	114159792	ENSMUSG00000031289	ENSMUSG00000031289	ENSMUSG00000031289	X	142629920	142675560	5	6
392	X	55052213	55074136	ENSMUSG00000025270	ENSMUSG00000025270	ENSMUSG00000025270	X	145888161	145911335	6	7
393	X	54976119	55037236	ENSMUSG00000025271	ENSMUSG00000025271	ENSMUSG00000025271	X	145930637	145984576	6	7
394	X	54963969	54974589	ENSMUSG00000025272	ENSMUSG00000025272	ENSMUSG00000025272	X	145986421	145998115	6	7
395	X	54850757	54859165	ENSMUSG00000025268	ENSMUSG00000025268	ENSMUSG00000025268	X	146147420	146154998	6	7
396	X	54573369	54604229	ENSMUSG00000025266	ENSMUSG00000025266	ENSMUSG00000025266	X	146323851	146357960	6	7
397	X	54488612	54539324	ENSMUSG00000025265	ENSMUSG00000025265	ENSMUSG00000025265	X	146387949	146429815	6	7
398	X	54483559	54488645	ENSMUSG00000025264	ENSMUSG00000025264	ENSMUSG00000025264	X	146428025	146437234	6	7
399	X	54239580	54401163	ENSMUSG00000041245	ENSMUSG00000041245	ENSMUSG00000041245	X	146538856	146655841	6	7
400	X	54115310	54226416	ENSMUSG00000025262	ENSMUSG00000025262	ENSMUSG00000025262	X	146684939	146810626	6	7
401	X	53575782	53727839	ENSMUSG00000025261	ENSMUSG00000025261	ENSMUSG00000025261	X	147144043	147275608	6	7
402	X	53474931	53478045	ENSMUSG00000025260	ENSMUSG00000025260	ENSMUSG00000025260	X	147342597	147345155	6	7
403	X	53466615	53474784	ENSMUSG00000025257	ENSMUSG00000025257	ENSMUSG00000025257	X	147345445	147350357	6	7
404	X	53417795	53466343	ENSMUSG00000041133	ENSMUSG00000041133	ENSMUSG00000041133	X	147357144	147402683	6	7
405	X	53278783	53367247	ENSMUSG00000041115	ENSMUSG00000041115	ENSMUSG00000041115	X	147519781	147562547	6	7
406	X	53238059	53271329	ENSMUSG00000025332	ENSMUSG00000025332	ENSMUSG00000025332	X	147573988	147614512	6	7
407	X	53128274	53134447	ENSMUSG00000041096	ENSMUSG00000041096	ENSMUSG00000041096	X	147677568	147683200	6	7
408	X	53095231	53123967	ENSMUSG00000056679	ENSMUSG00000056679	ENSMUSG00000056679	X	147685362	147708827	6	7
409	X	55529687	55532356	ENSMUSG00000067215	ENSMUSG00000067215	ENSMUSG00000067215	X	148349229	148350113	7	8
410	X	55495263	55496724	ENSMUSG00000047238	ENSMUSG00000047238	ENSMUSG00000047238	X	148377398	148378054	7	8
411	X	55666558	55668482	ENSMUSG00000071665	ENSMUSG00000071665	ENSMUSG00000071665	X	148467613	148473577	7	8
412	X	55760904	55801931	ENSMUSG00000041658	ENSMUSG00000041658	ENSMUSG00000041658	X	148480697	148512658	7	8
413	X	56276053	56328379	ENSMUSG00000041649	ENSMUSG00000041649	ENSMUSG00000041649	X	148700329	148736511	7	8
414	X	56606797	56611026	ENSMUSG00000050148	ENSMUSG00000050148	ENSMUSG00000050148	X	148838948	148842286	7	8
415	X	23711235	23714212	ENSMUSG00000025283	ENSMUSG00000025283	ENSMUSG00000025283	X	150553852	150557125	8	9
416	X	23595484	23614437	ENSMUSG00000025289	ENSMUSG00000025289	ENSMUSG00000025289	X	150664637	150679183	8	9
417	X	23262054	23322340	ENSMUSG00000041552	ENSMUSG00000041552	ENSMUSG00000041552	X	150914368	150927747	8	9
418	X	21960480	22179348	ENSMUSG00000057457	ENSMUSG00000057457	ENSMUSG00000057457	X	152502791	152759383	8	9
419	X	21767675	21813463	ENSMUSG00000046873	ENSMUSG00000046873	ENSMUSG00000046873	X	152879524	152913110	8	9
420	X	21634011	21686202	ENSMUSG00000041476	ENSMUSG00000041476	ENSMUSG00000041476	X	153043223	153096694	8	9
421	X	21582729	21586369	ENSMUSG00000047485	ENSMUSG00000047485	ENSMUSG00000047485	X	153162567	153164354	8	9
422	X	21302901	21582734	ENSMUSG00000025658	ENSMUSG00000025658	ENSMUSG00000025658	X	153165673	153387215	8	9
423	X	20077950	20194671	ENSMUSG00000031309	ENSMUSG00000031309	ENSMUSG00000031309	X	154600225	154707590	8	9
424	X	19934906	20044956	ENSMUSG00000041020	ENSMUSG00000041020	ENSMUSG00000041020	X	154803480	154840386	8	9
425	X	19840899	19898337	ENSMUSG00000044150	ENSMUSG00000044150	ENSMUSG00000044150	X	154876811	154937185	8	9
426	X	19462014	19815640	ENSMUSG00000040990	ENSMUSG00000040990	ENSMUSG00000040990	X	154971513	155318528	8	9
427	X	19288097	19443363	ENSMUSG00000031303	ENSMUSG00000031303	ENSMUSG00000031303	X	155346131	155467450	8	9
428	X	19271972	19289724	ENSMUSG00000031299	ENSMUSG00000031299	ENSMUSG00000031299	X	155466324	155482441	8	9
429	X	18917348	19050676	ENSMUSG00000031298	ENSMUSG00000031298	ENSMUSG00000031298	X	155734830	155842175	8	9
430	X	18820339	18912637	ENSMUSG00000031295	ENSMUSG00000031295	ENSMUSG00000031295	X	155846795	155935827	8	9
431	X	18618967	18755960	ENSMUSG00000062168	ENSMUSG00000062168	ENSMUSG00000062168	X	155985935	156037717	8	9
432	X	18567733	18600150	ENSMUSG00000031293	ENSMUSG00000031293	ENSMUSG00000031293	X	156112118	156143768	8	9
433	X	18353624	18581585	ENSMUSG00000031292	ENSMUSG00000031292	ENSMUSG00000031292	X	156128584	156338782	8	9
434	X	17728092	17789277	ENSMUSG00000043518	ENSMUSG00000043518	ENSMUSG00000043518	X	157061732	157123601	8	9
435	X	17303464	17664035	ENSMUSG00000059493	ENSMUSG00000059493	ENSMUSG00000059493	X	157184575	157503493	8	9
436	X	16874735	17075656	ENSMUSG00000040855	ENSMUSG00000040855	ENSMUSG00000040855	X	157756059	157987706	8	9
437	X	16772698	16797896	ENSMUSG00000031353	ENSMUSG00000031353	ENSMUSG00000031353	X	158104564	158123195	8	9
438	X	16714471	16772563	ENSMUSG00000038344	ENSMUSG00000038344	ENSMUSG00000038344	X	158123024	158173504	8	9
439	X	16647676	16693380	ENSMUSG00000031357	ENSMUSG00000031357	ENSMUSG00000031357	X	158200957	158232536	8	9
440	X	16516350	16640980	ENSMUSG00000031360	ENSMUSG00000031360	ENSMUSG00000031360	X	158245764	158376613	8	9
441	X	16578202	16582714	ENSMUSG00000040808	ENSMUSG00000040808	ENSMUSG00000040808	X	158306097	158308704	8	9
442	X	16051345	16081562	ENSMUSG00000031364	ENSMUSG00000031364	ENSMUSG00000031364	X	158858009	158893752	8	9
443	X	15666313	15712579	ENSMUSG00000031373	ENSMUSG00000031373	ENSMUSG00000031373	X	159322486	159372066	8	9
444	X	15555362	15593075	ENSMUSG00000015401	ENSMUSG00000015401	ENSMUSG00000015401	X	159434292	159462964	8	9
445	X	15489077	15530199	ENSMUSG00000015405	ENSMUSG00000015405	ENSMUSG00000015405	X	159484737	159532523	8	9
446	X	15392290	15484572	ENSMUSG00000031377	ENSMUSG00000031377	ENSMUSG00000031377	X	159536967	159602298	8	9
447	X	15312842	15421608	ENSMUSG00000031379	ENSMUSG00000031379	ENSMUSG00000031379	X	159613542	159717010	8	9
448	X	15273640	15312498	ENSMUSG00000031380	ENSMUSG00000031380	ENSMUSG00000031380	X	159717653	159746755	8	9
449	X	15247499	15263566	ENSMUSG00000031381	ENSMUSG00000031381	ENSMUSG00000031381	X	159763921	159778021	8	9
450	X	15211548	15243699	ENSMUSG00000031382	ENSMUSG00000031382	ENSMUSG00000031382	X	159781099	159803266	8	9
451	X	15172030	15198191	ENSMUSG00000031384	ENSMUSG00000031384	ENSMUSG00000031384	X	159850473	159883857	8	9
452	X	14801484	14850370	ENSMUSG00000061778	ENSMUSG00000061778	ENSMUSG00000061778	X	160282314	160324440	8	9
453	X	14771450	14801112	ENSMUSG00000047757	ENSMUSG00000047757	ENSMUSG00000047757	X	160324807	160341377	8	9
454	X	14457565	14659852	ENSMUSG00000018589	ENSMUSG00000018589	ENSMUSG00000018589	X	160473122	160671086	8	9
455	X	13698983	13745235	ENSMUSG00000031342	ENSMUSG00000031342	ENSMUSG00000031342	X	161688797	161731206	8	9
456	X	13617165	13638546	ENSMUSG00000040577	ENSMUSG00000040577	ENSMUSG00000040577	X	161801364	161823799	8	9
457	X	13497662	13561615	ENSMUSG00000000402	ENSMUSG00000000402	ENSMUSG00000000402	X	161867112	161929821	8	9
458	X	12834679	12850343	ENSMUSG00000040522	ENSMUSG00000040522	ENSMUSG00000040522	X	162586799	162607911	8	9
459	X	12795123	12818420	ENSMUSG00000044583	ENSMUSG00000044583	ENSMUSG00000044583	X	162649036	162674633	8	9
460	X	12719452	12752262	ENSMUSG00000025742	ENSMUSG00000025742	ENSMUSG00000025742	X	162690438	162726804	8	9
461	X	12066841	12646832	ENSMUSG00000049176	ENSMUSG00000049176	ENSMUSG00000049176	X	162818078	163073243	8	9
462	X	11065585	11593742	ENSMUSG00000031355	ENSMUSG00000031355	ENSMUSG00000031355	X	164140326	164647116	8	9
463	X	11039342	11051119	ENSMUSG00000031352	ENSMUSG00000031352	ENSMUSG00000031352	X	164655640	164664385	8	9
464	X	10373596	10761694	ENSMUSG00000035299	ENSMUSG00000035299	ENSMUSG00000035299	X	165271056	165349841	8	9
465	8	56177603	56599340	ENSMUSG00000051951	ENSMUSG00000051951	ENSMUSG00000051951	1	3206103	3661429	9	10
466	8	55691180	55705947	ENSMUSG00000025900	ENSMUSG00000025900	ENSMUSG00000025900	1	4334224	4350473	9	10
467	8	55533048	55535484	ENSMUSG00000025902	ENSMUSG00000025902	ENSMUSG00000025902	1	4481009	4486494	9	10
468	8	55210334	55223011	ENSMUSG00000033845	ENSMUSG00000033845	ENSMUSG00000033845	1	4763292	4775790	9	10
469	8	55121492	55177130	ENSMUSG00000025903	ENSMUSG00000025903	ENSMUSG00000025903	1	4797943	4836817	9	10
470	8	55045401	55097561	ENSMUSG00000033813	ENSMUSG00000033813	ENSMUSG00000033813	1	4882025	4885092	9	10
471	8	54926921	55034416	ENSMUSG00000002459	ENSMUSG00000002459	ENSMUSG00000002459	1	4900326	5009507	9	10
472	8	54790669	54918403	ENSMUSG00000033793	ENSMUSG00000033793	ENSMUSG00000033793	1	5073254	5152630	9	10
473	8	54300829	54326747	ENSMUSG00000025905	ENSMUSG00000025905	ENSMUSG00000025905	1	5578574	5592947	9	10
474	8	54013546	54016230	ENSMUSG00000033774	ENSMUSG00000033774	ENSMUSG00000033774	1	5906388	5907374	9	10
475	8	53185952	53484848	ENSMUSG00000033740	ENSMUSG00000033740	ENSMUSG00000033740	1	6720132	6851021	9	10
476	8	51469326	51867978	ENSMUSG00000025909	ENSMUSG00000025909	ENSMUSG00000025909	1	8353117	8772831	9	10
477	8	67503817	67505520	ENSMUSG00000061024	ENSMUSG00000061024	ENSMUSG00000061024	1	9530650	9532664	10	11
478	8	67507288	67543596	ENSMUSG00000025911	ENSMUSG00000025911	ENSMUSG00000025911	1	9533294	9563182	10	11
479	8	67568321	67593293	ENSMUSG00000067879	ENSMUSG00000067879	ENSMUSG00000067879	1	9586481	9612356	10	11
480	8	67639486	67687729	ENSMUSG00000025912	ENSMUSG00000025912	ENSMUSG00000025912	1	9655098	9678608	10	11
481	8	67705044	67742006	ENSMUSG00000045210	ENSMUSG00000045210	ENSMUSG00000045210	1	9708406	9733596	10	11
482	8	67787445	67936800	ENSMUSG00000025915	ENSMUSG00000025915	ENSMUSG00000025915	1	9783344	9887784	10	11
483	8	67945538	67976212	ENSMUSG00000046101	ENSMUSG00000046101	ENSMUSG00000046101	1	9893852	9907741	10	11
484	8	68039015	68092536	ENSMUSG00000025916	ENSMUSG00000025916	ENSMUSG00000025916	1	9953846	9994350	10	11
485	8	68117871	68136785	ENSMUSG00000025917	ENSMUSG00000025917	ENSMUSG00000025917	1	10009816	10023160	10	11
486	8	68170454	68270382	ENSMUSG00000056763	ENSMUSG00000056763	ENSMUSG00000056763	1	10097612	10121980	10	11
487	8	68272451	68418466	ENSMUSG00000067851	ENSMUSG00000067851	ENSMUSG00000067851	1	10124049	10217708	10	11
488	8	68496963	68821134	ENSMUSG00000042501	ENSMUSG00000042501	ENSMUSG00000042501	1	10309941	10705157	10	11
489	8	69026907	69306451	ENSMUSG00000048960	ENSMUSG00000048960	ENSMUSG00000048960	1	10979007	11284089	10	11
490	8	69405511	69893344	ENSMUSG00000057715	ENSMUSG00000057715	ENSMUSG00000057715	1	11399783	11936272	10	11
491	8	70541427	70735701	ENSMUSG00000016918	ENSMUSG00000016918	ENSMUSG00000016918	1	12703743	12845591	10	11
492	8	70747129	70909762	ENSMUSG00000025938	ENSMUSG00000025938	ENSMUSG00000025938	1	12863809	12976349	10	11
493	8	71126574	71146116	ENSMUSG00000042414	ENSMUSG00000042414	ENSMUSG00000042414	1	13099474	13110902	10	11
494	8	71196006	71478574	ENSMUSG00000005886	ENSMUSG00000005886	ENSMUSG00000005886	1	13132089	13209908	10	11
495	8	71648227	71683158	ENSMUSG00000025935	ENSMUSG00000025935	ENSMUSG00000025935	1	13549916	13575078	10	11
496	8	71712045	71743946	ENSMUSG00000025937	ENSMUSG00000025937	ENSMUSG00000025937	1	13611130	13645749	10	11
497	8	71744154	71810730	ENSMUSG00000067813	ENSMUSG00000067813	ENSMUSG00000067813	1	13653985	13686937	10	11
498	8	72272222	72437021	ENSMUSG00000025932	ENSMUSG00000025932	ENSMUSG00000025932	1	14155857	14289786	10	11
499	8	72917413	72919259	ENSMUSG00000025930	ENSMUSG00000025930	ENSMUSG00000025930	1	14738560	14741180	10	11
500	8	73097695	73150198	ENSMUSG00000032769	ENSMUSG00000032769	ENSMUSG00000032769	1	14857862	14904076	10	11
501	8	74141809	74168061	ENSMUSG00000032719	ENSMUSG00000032719	ENSMUSG00000032719	1	15839076	15877936	10	11
502	8	74369891	74399345	ENSMUSG00000025921	ENSMUSG00000025921	ENSMUSG00000025921	1	16091096	16117764	10	11
503	8	74495160	74821629	ENSMUSG00000025920	ENSMUSG00000025920	ENSMUSG00000025920	1	16214024	16504450	10	11
504	8	74865396	74953664	ENSMUSG00000025939	ENSMUSG00000025939	ENSMUSG00000025939	1	16529148	16588929	10	11
505	8	75019928	75047076	ENSMUSG00000042730	ENSMUSG00000042730	ENSMUSG00000042730	1	16628502	16631044	10	11
506	8	75050998	75057565	ENSMUSG00000025940	ENSMUSG00000025940	ENSMUSG00000025940	1	16650421	16663489	10	11
507	8	75066141	75103859	ENSMUSG00000025779	ENSMUSG00000025779	ENSMUSG00000025779	1	16673647	16694819	10	11
508	8	75309493	75396117	ENSMUSG00000042686	ENSMUSG00000042686	ENSMUSG00000042686	1	16982678	17083012	10	11
509	8	75425248	75439157	ENSMUSG00000025777	ENSMUSG00000025777	ENSMUSG00000025777	1	17130611	17149485	10	11
510	8	75899327	75929819	ENSMUSG00000067780	ENSMUSG00000067780	ENSMUSG00000067780	1	17587118	17613034	10	11
511	6	49909929	49942171	ENSMUSG00000025774	ENSMUSG00000025774	ENSMUSG00000025774	1	18100405	18131110	11	12
512	6	50094516	50097653	ENSMUSG00000067773	ENSMUSG00000067773	ENSMUSG00000067773	1	18245738	18250294	11	12
513	6	50789216	50848705	ENSMUSG00000042596	ENSMUSG00000042596	ENSMUSG00000042596	1	19088234	19151562	11	12
514	6	50894542	50919634	ENSMUSG00000025927	ENSMUSG00000025927	ENSMUSG00000025927	1	19194128	19223948	11	12
515	6	51588057	52060382	ENSMUSG00000043760	ENSMUSG00000043760	ENSMUSG00000043760	1	20042930	20603208	11	12
516	6	52159144	52163395	ENSMUSG00000025929	ENSMUSG00000025929	ENSMUSG00000025929	1	20716056	20719647	11	12
517	6	52209443	52217257	ENSMUSG00000041872	ENSMUSG00000041872	ENSMUSG00000041872	1	20762302	20769421	11	12
518	6	52236766	52257541	ENSMUSG00000041859	ENSMUSG00000041859	ENSMUSG00000041859	1	20788124	20805411	11	12
519	6	52334904	52380532	ENSMUSG00000025931	ENSMUSG00000025931	ENSMUSG00000025931	1	20902997	20924208	11	12
520	6	52393065	52465303	ENSMUSG00000041809	ENSMUSG00000041809	ENSMUSG00000041809	1	20936839	20975990	11	12
521	6	52470163	52549821	ENSMUSG00000041779	ENSMUSG00000041779	ENSMUSG00000041779	1	20986548	21064376	11	12
522	6	52643864	52659345	ENSMUSG00000025933	ENSMUSG00000025933	ENSMUSG00000025933	1	21203813	21215315	11	12
523	6	73388639	73961858	ENSMUSG00000028033	ENSMUSG00000028033	ENSMUSG00000028033	1	21387314	21946732	12	13
524	6	72653448	73167137	ENSMUSG00000041670	ENSMUSG00000041670	ENSMUSG00000041670	1	22271402	22759999	12	13
525	6	72055198	72068692	ENSMUSG00000026158	ENSMUSG00000026158	ENSMUSG00000026158	1	23324066	23333333	12	13
526	6	71623103	71723462	ENSMUSG00000026156	ENSMUSG00000026156	ENSMUSG00000026156	1	23716293	23802868	12	13
527	6	71434200	71628439	ENSMUSG00000026155	ENSMUSG00000026155	ENSMUSG00000026155	1	23799900	23876564	12	13
528	6	71333341	71355993	ENSMUSG00000026154	ENSMUSG00000026154	ENSMUSG00000026154	1	23950221	23959915	12	13
529	6	71179910	71327595	ENSMUSG00000026153	ENSMUSG00000026153	ENSMUSG00000026153	1	23965954	24040849	12	13
530	6	70982529	71069494	ENSMUSG00000026147	ENSMUSG00000026147	ENSMUSG00000026147	1	24131975	24206620	12	13
531	6	70633169	70978878	ENSMUSG00000026141	ENSMUSG00000026141	ENSMUSG00000026141	1	24218269	24541686	12	13
532	6	70442415	70563724	ENSMUSG00000073725	ENSMUSG00000073725	ENSMUSG00000073725	1	24633032	24707573	12	13
533	6	69401980	70156124	ENSMUSG00000033569	ENSMUSG00000033569	ENSMUSG00000033569	1	25012714	25771306	12	13
534	6	64403700	64483336	ENSMUSG00000048874	ENSMUSG00000048874	ENSMUSG00000048874	1	30748348	30775377	12	13
535	6	64339879	64351447	ENSMUSG00000026064	ENSMUSG00000026064	ENSMUSG00000026064	1	30886138	30894291	12	13
536	6	64047247	64087841	ENSMUSG00000050217	ENSMUSG00000050217	ENSMUSG00000050217	1	31120981	31149273	12	13
537	6	62447826	63054091	ENSMUSG00000026058	ENSMUSG00000026058	ENSMUSG00000026058	1	32117273	32602829	12	13
538	6	57291203	57620646	ENSMUSG00000026134	ENSMUSG00000026134	ENSMUSG00000026134	1	33398361	33614326	12	13
539	6	57161567	57195039	ENSMUSG00000004768	ENSMUSG00000004768	ENSMUSG00000004768	1	33664442	33686043	12	13
540	6	57145083	57157694	ENSMUSG00000042215	ENSMUSG00000042215	ENSMUSG00000042215	1	33690030	33702317	12	13
541	6	57062795	57143064	ENSMUSG00000042197	ENSMUSG00000042197	ENSMUSG00000042197	1	33706299	33758973	12	13
542	6	56928047	56990116	ENSMUSG00000042182	ENSMUSG00000042182	ENSMUSG00000042182	1	33797334	33852167	12	13
543	6	56430755	56816422	ENSMUSG00000026131	ENSMUSG00000026131	ENSMUSG00000026131	1	33956371	34252174	12	13
544	2	130816453	130820667	ENSMUSG00000026127	ENSMUSG00000026127	ENSMUSG00000026127	1	34384587	34389016	13	14
545	2	130830088	130848613	ENSMUSG00000026126	ENSMUSG00000026126	ENSMUSG00000026126	1	34404304	34418332	13	14
546	2	131229583	131239129	ENSMUSG00000045174	ENSMUSG00000045174	ENSMUSG00000045174	1	34524273	34535490	13	14
547	2	131390694	131520232	ENSMUSG00000037509	ENSMUSG00000037509	ENSMUSG00000037509	1	34746268	34757300	13	14
548	2	131526949	131556689	ENSMUSG00000037503	ENSMUSG00000037503	ENSMUSG00000037503	1	34757767	34787596	13	14
549	2	131594985	131827569	ENSMUSG00000026123	ENSMUSG00000026123	ENSMUSG00000026123	1	34794538	34824123	13	14
550	2	128565402	128665444	ENSMUSG00000037470	ENSMUSG00000037470	ENSMUSG00000037470	1	35996441	36188795	14	15
551	2	128741502	128792722	ENSMUSG00000045216	ENSMUSG00000045216	ENSMUSG00000045216	1	36012946	36050990	14	15
552	2	96566191	96582097	ENSMUSG00000037447	ENSMUSG00000037447	ENSMUSG00000037447	1	36252300	36267521	15	16
553	2	96622666	96667810	ENSMUSG00000010453	ENSMUSG00000010453	ENSMUSG00000010453	1	36280276	36313727	15	16
554	2	96735396	96769517	ENSMUSG00000001143	ENSMUSG00000001143	ENSMUSG00000001143	1	36367732	36389785	15	16
555	2	96790366	96841351	ENSMUSG00000037408	ENSMUSG00000037408	ENSMUSG00000037408	1	36416166	36453304	15	16
556	2	96845718	96863357	ENSMUSG00000001138	ENSMUSG00000001138	ENSMUSG00000001138	1	36456422	36472783	15	16
557	2	96867379	96873485	ENSMUSG00000067653	ENSMUSG00000067653	ENSMUSG00000067653	1	36475080	36480275	15	16
558	2	96889206	96899435	ENSMUSG00000026121	ENSMUSG00000026121	ENSMUSG00000026121	1	36493188	36502105	15	16
559	2	97638863	97646893	ENSMUSG00000037351	ENSMUSG00000037351	ENSMUSG00000037351	1	36642662	36654487	15	16
560	2	97696461	97722755	ENSMUSG00000026117	ENSMUSG00000026117	ENSMUSG00000026117	1	36706371	36727362	15	16
561	2	97739235	97910336	ENSMUSG00000026116	ENSMUSG00000026116	ENSMUSG00000026116	1	36737467	36833872	15	16
562	2	98070085	98274976	ENSMUSG00000050122	ENSMUSG00000050122	ENSMUSG00000050122	1	36971142	37021541	15	16
563	2	98360183	98380527	ENSMUSG00000026114	ENSMUSG00000026114	ENSMUSG00000026114	1	37163807	37207932	15	16
564	2	98427753	98570554	ENSMUSG00000026113	ENSMUSG00000026113	ENSMUSG00000026113	1	37302465	37352805	15	16
565	2	98582298	98591387	ENSMUSG00000026112	ENSMUSG00000026112	ENSMUSG00000026112	1	37363735	37374524	15	16
566	2	98591472	98601406	ENSMUSG00000026111	ENSMUSG00000026111	ENSMUSG00000026111	1	37374718	37383670	15	16
567	2	98608089	98709362	ENSMUSG00000026110	ENSMUSG00000026110	ENSMUSG00000026110	1	37389102	37480678	15	16
568	2	98805327	98919110	ENSMUSG00000026090	ENSMUSG00000026090	ENSMUSG00000026090	1	37556223	37664357	15	16
569	2	98980773	99137812	ENSMUSG00000060771	ENSMUSG00000060771	ENSMUSG00000060771	1	37699320	37809625	15	16
570	2	99137850	99146043	ENSMUSG00000037216	ENSMUSG00000037216	ENSMUSG00000037216	1	37816752	37821933	15	16
571	2	99152160	99163924	ENSMUSG00000026088	ENSMUSG00000026088	ENSMUSG00000026088	1	37823436	37834891	15	16
572	2	99169069	99180318	ENSMUSG00000026087	ENSMUSG00000026087	ENSMUSG00000026087	1	37835099	37842881	15	16
573	2	99225149	99238002	ENSMUSG00000061584	ENSMUSG00000061584	ENSMUSG00000061584	1	37850469	37861039	15	16
574	2	99267155	99287637	ENSMUSG00000026085	ENSMUSG00000026085	ENSMUSG00000026085	1	37891284	37902305	15	16
575	2	99320300	99382673	ENSMUSG00000026083	ENSMUSG00000026083	ENSMUSG00000026083	1	37942556	38000125	15	16
576	2	99383846	99472912	ENSMUSG00000026082	ENSMUSG00000026082	ENSMUSG00000026082	1	37997330	38074208	15	16
577	2	99530150	100088477	ENSMUSG00000037138	ENSMUSG00000037138	ENSMUSG00000037138	1	38123998	38571687	15	16
578	2	100266436	100292399	ENSMUSG00000048814	ENSMUSG00000048814	ENSMUSG00000048814	1	38739055	38765761	15	16
579	2	100374762	100400550	ENSMUSG00000026080	ENSMUSG00000026080	ENSMUSG00000026080	1	38808413	38842706	15	16
580	2	100453376	100466174	ENSMUSG00000067604	ENSMUSG00000067604	ENSMUSG00000067604	1	38883695	38894824	15	16
581	2	100545859	100559633	ENSMUSG00000026078	ENSMUSG00000026078	ENSMUSG00000026078	1	38932360	38941782	15	16
582	2	100803045	100979719	ENSMUSG00000026077	ENSMUSG00000026077	ENSMUSG00000026077	1	39138750	39306732	15	16

583	2	100990123	101042444	ENSMUSG00000003134	ENSMUSG00000003134	ENSMUSG00000003134	1	39316046	39423281	15	16		
584	2	101235810	101253208	ENSMUSG00000003135	ENSMUSG00000003135	ENSMUSG00000003135	1	39480348	39491437	15	16		
585	2	101258985	101291584	ENSMUSG00000048234	ENSMUSG00000048234	ENSMUSG00000048234	1	39495847	39521951	15	16		
586	2	101331655	101370489	ENSMUSG00000050967	ENSMUSG00000050967	ENSMUSG00000050967	1	39562952	39595728	15	16		
587	2	101380255	101462828	ENSMUSG00000057173	ENSMUSG00000057173	ENSMUSG00000057173	1	39610030	39616759	15	16		
588	2	101680920	101877583	ENSMUSG00000026074	ENSMUSG00000026074	ENSMUSG00000026074	1	39845716	39969258	15	16		
589	2	101974738	102011312	ENSMUSG00000026073	ENSMUSG00000026073	ENSMUSG00000026073	1	40029314	40069773	15	16		
590	2	102125678	102159788	ENSMUSG00000026072	ENSMUSG00000026072	ENSMUSG00000026072	1	40169626	40260723	15	16		
591	2	102169865	102222243	ENSMUSG00000070942	ENSMUSG00000070942	ENSMUSG00000070942	1	40269236	40312097	15	16		
592	2	102294394	102334929	ENSMUSG00000026069	ENSMUSG00000026069	ENSMUSG00000026069	1	40385254	40409958	15	16		
593	2	102345529	102381650	ENSMUSG00000026070	ENSMUSG00000026070	ENSMUSG00000026070	1	40410552	40445379	15	16		
594	2	102401686	102435457	ENSMUSG00000026068	ENSMUSG00000026068	ENSMUSG00000026068	1	40459908	40493851	15	16		
595	2	102456194	102516859	ENSMUSG00000026065	ENSMUSG00000026065	ENSMUSG00000026065	1	40524747	40574199	15	16		
596	2	102602598	102694239	ENSMUSG00000026062	ENSMUSG00000026062	ENSMUSG00000026062	1	40626171	40713812	15	16		
597	2	102700106	102719745	ENSMUSG00000041945	ENSMUSG00000041945	ENSMUSG00000041945	1	40716586	40735209	15	16		
598	2	104838401	104839903	ENSMUSG00000045515	ENSMUSG00000045515	ENSMUSG00000045515	1	42641692	42643185	15	16		
599	2	105020938	105082849	ENSMUSG00000060679	ENSMUSG00000060679	ENSMUSG00000060679	1	42795829	42850226	15	16		
600	2	105224632	105226356	ENSMUSG00000041907	ENSMUSG00000041907	ENSMUSG00000041907	1	42897509	42979995	15	16		
601	2	105249974	105291210	ENSMUSG00000070939	ENSMUSG00000070939	ENSMUSG00000070939	1	42991815	43043168	15	16		
602	2	105320443	105331653	ENSMUSG00000010290	ENSMUSG00000010290	ENSMUSG00000010290	1	43043256	43060492	15	16		
603	2	105343719	105382113	ENSMUSG00000008136	ENSMUSG00000008136	ENSMUSG00000008136	1	43067620	43108415	15	16		
604	2	106048545	106061040	ENSMUSG00000026051	ENSMUSG00000026051	ENSMUSG00000026051	1	43675239	43687124	15	16		
605	2	106076203	106177189	ENSMUSG00000057363	ENSMUSG00000057363	ENSMUSG00000057363	1	43693833	43772294	15	16		
606	13	102047374	102129522	ENSMUSG00000041763	ENSMUSG00000041763	ENSMUSG00000041763	1	43878553	43947517	16	17		
607	13	102136098	102144855	ENSMUSG00000047343	ENSMUSG00000047343	ENSMUSG00000047343	1	43953954	43964552	16	17		
608	13	102184977	102187160	ENSMUSG00000070936	ENSMUSG00000070936	ENSMUSG00000070936	1	44004515	44006482	16	17		
609	13	102216236	102224129	ENSMUSG00000026049	ENSMUSG00000026049	ENSMUSG00000026049	1	44031412	44046969	16	17		
610	13	102234632	102249358	ENSMUSG00000026047	ENSMUSG00000026047	ENSMUSG00000026047	1	44051092	44063319	16	17		
611	13	102249400	102291882	ENSMUSG00000041684	ENSMUSG00000041684	ENSMUSG00000041684	1	44063708	44089056	16	17		
612	13	102296175	102326346	ENSMUSG00000026048	ENSMUSG00000026048	ENSMUSG00000026048	1	44092393	44125806	16	17		
613	13	102341779	102346384	ENSMUSG00000046828	ENSMUSG00000046828	ENSMUSG00000046828	1	44148616	44163477	16	17		
614	2	188864641	189168031	ENSMUSG00000056870	ENSMUSG00000056870	ENSMUSG00000056870	1	44496057	44741372	17	18		
615	2	189547344	189585717	ENSMUSG00000026043	ENSMUSG00000026043	ENSMUSG00000026043	1	45272979	45290601	17	18		
616	2	189605486	189752712	ENSMUSG00000026042	ENSMUSG00000026042	ENSMUSG00000026042	1	45318877	45447828	17	18		
617	2	190014404	190048509	ENSMUSG00000025995	ENSMUSG00000025995	ENSMUSG00000025995	1	45740012	45768161	17	18		
618	2	190133563	190153858	ENSMUSG00000025993	ENSMUSG00000025993	ENSMUSG00000025993	1	45852630	45870079	17	18		
619	2	196229927	196310663	ENSMUSG00000025986	ENSMUSG00000025986	ENSMUSG00000025986	1	46752090	46798055	18	19		
620	2	192407295	192420226	ENSMUSG00000045954	ENSMUSG00000045954	ENSMUSG00000045954	1	51233671	51247505	18	19		
621	2	192251107	192260531	ENSMUSG00000026107	ENSMUSG00000026107	ENSMUSG00000026107	1	51410407	51422460	18	19		
622	2	191818499	191998131	ENSMUSG00000018417	ENSMUSG00000018417	ENSMUSG00000018417	1	51694539	51860473	18	19		
623	2	191602553	191724539	ENSMUSG00000062939	ENSMUSG00000062939	ENSMUSG00000062939	1	51952788	52051729	18	19		
624	2	191542121	191587181	ENSMUSG00000026104	ENSMUSG00000026104	ENSMUSG00000026104	1	52067139	52100639	18	19		
625	2	191453842	191538517	ENSMUSG00000026103	ENSMUSG00000026103	ENSMUSG00000026103	1	52129826	52177777	18	19		
626	2	191222093	191265734	ENSMUSG00000002881	ENSMUSG00000002881	ENSMUSG00000002881	1	52402421	52444905	18	19		
627	2	191089289	191107626	ENSMUSG00000043015	ENSMUSG00000043015	ENSMUSG00000043015	1	52575303	52586957	18	19		
628	2	191008944	191075046	ENSMUSG00000041439	ENSMUSG00000041439	ENSMUSG00000041439	1	52604531	52671863	18	19		
629	2	190916680	190944552	ENSMUSG00000026102	ENSMUSG00000026102	ENSMUSG00000026102	1	52733967	52762120	18	19		
630	2	190777606	190892804	ENSMUSG00000041426	ENSMUSG00000041426	ENSMUSG00000041426	1	52789593	52865531	18	19		
631	2	190628674	190635700	ENSMUSG00000026100	ENSMUSG00000026100	ENSMUSG00000026100	1	53006208	53012624	18	19		
632	2	190357355	190450599	ENSMUSG00000026098	ENSMUSG00000026098	ENSMUSG00000026098	1	53133732	53241541	18	19		
633	2	190319631	190335738	ENSMUSG00000026096	ENSMUSG00000026096	ENSMUSG00000026096	1	53258206	53270885	18	19		
634	2	190234391	190243800	ENSMUSG00000026095	ENSMUSG00000026095	ENSMUSG00000026095	1	53289189	53297244	18	19		
635	2	196709913	196744544	ENSMUSG00000026094	ENSMUSG00000026094	ENSMUSG00000026094	1	53700057	53729699	19	20		
636	2	196772222	197165580	ENSMUSG00000042807	ENSMUSG00000042807	ENSMUSG00000042807	1	53757767	53985037	19	20		
637	2	197219304	197305528	ENSMUSG00000025983	ENSMUSG00000025983	ENSMUSG00000025983	1	54195228	54313272	19	20		
638	2	197336917	197372610	ENSMUSG00000041303	ENSMUSG00000041303	ENSMUSG00000041303	1	54342126	54383516	19	20		
639	2	197405973	197499699	ENSMUSG00000070889	ENSMUSG00000070889	ENSMUSG00000070889	1	54434766	54502229	19	20		
640	2	197964945	198008016	ENSMUSG00000025982	ENSMUSG00000025982	ENSMUSG00000025982	1	54929714	54972024	19	20		
641	2	198026476	198048096	ENSMUSG00000025981	ENSMUSG00000025981	ENSMUSG00000025981	1	54997414	55017246	19	20		
642	2	198089016	198125577	ENSMUSG00000025979	ENSMUSG00000025979	ENSMUSG00000025979	1	55075845	55099437	19	20		
643	2	198143773	198248829	ENSMUSG00000025978	ENSMUSG00000025978	ENSMUSG00000025978	1	55114707	55171327	19	20		
644	2	198278332	198281358	ENSMUSG00000046994	ENSMUSG00000046994	ENSMUSG00000046994	1	55181782	55184593	19	20		
645	2	198301218	198357979	ENSMUSG00000025977	ENSMUSG00000025977	ENSMUSG00000025977	1	55244614	55307859	19	20		
646	2	198656724	198721293	ENSMUSG00000038349	ENSMUSG00000038349	ENSMUSG00000038349	1	55640290	55696008	19	20		
647	2	199842476	200038076	ENSMUSG00000038331	ENSMUSG00000038331	ENSMUSG00000038331	1	56740812	56915879	19	20		
648	2	200502962	200528704	ENSMUSG00000048495	ENSMUSG00000048495	ENSMUSG00000048495	1	57332793	57350996	19	20		
649	2	200528285	200537076	ENSMUSG00000025971	ENSMUSG00000025971	ENSMUSG00000025971	1	57351304	57362490	19	20		
650	2	200985276	201051497	ENSMUSG00000038305	ENSMUSG00000038305	ENSMUSG00000038305	1	57824051	57891510	19	20		
651	2	201061933	201083037	ENSMUSG00000054770	ENSMUSG00000054770	ENSMUSG00000054770	1	57904600	57914598	19	20		
652	2	201158976	201244460	ENSMUSG00000063558	ENSMUSG00000063558	ENSMUSG00000063558	1	57974515	58050956	19	20		
653	2	201384558	201396811	ENSMUSG00000051223	ENSMUSG00000051223	ENSMUSG00000051223	1	58337673	58351098	19	20		
654	2	201425978	201437667	ENSMUSG00000026034	ENSMUSG00000026034	ENSMUSG00000026034	1	58356743	58366480	19	20		
655	2	201443925	201462244	ENSMUSG00000026035	ENSMUSG00000026035	ENSMUSG00000026035	1	58375750	58387145	19	20		
656	2	201462340	201476891	ENSMUSG00000026036	ENSMUSG00000026036	ENSMUSG00000026036	1	58389697	58406069	19	20		
657	2	201483140	201536655	ENSMUSG00000026037	ENSMUSG00000026037	ENSMUSG00000026037	1	58407317	58449473	19	20		
658	2	201551461	201644633	ENSMUSG00000038174	ENSMUSG00000038174	ENSMUSG00000038174	1	58471804	58530816	19	20		
659	2	201644759	201658716	ENSMUSG00000026032	ENSMUSG00000026032	ENSMUSG00000026032	1	58530989	58540468	19	20		
660	2	201861240	201924421	ENSMUSG00000047528	ENSMUSG00000047528	ENSMUSG00000047528	1	58603538	58636959	19	20		
661	2	201831024	201860677	ENSMUSG00000026029	ENSMUSG00000026029	ENSMUSG00000026029	1	58739978	58791553	19	20		
662	2	201950176	202024499	ENSMUSG00000026028	ENSMUSG00000026028	ENSMUSG00000026028	1	58844996	58891044	19	20		
663	2	202024637	202053695	ENSMUSG00000026027	ENSMUSG00000026027	ENSMUSG00000026027	1	58918146	58940261	19	20		
664	2	202060402	202192143	ENSMUSG00000072295	ENSMUSG00000072295	ENSMUSG00000072295	1	58995469	59022515	19	20		
665	2	202196668	202215688	ENSMUSG00000038079	ENSMUSG00000038079	ENSMUSG00000038079	1	59045136	59064905	19	20		
666	2	202273522	202353983	ENSMUSG00000026024	ENSMUSG00000026024	ENSMUSG00000026024	1	59107478	59181765	19	20		
667	2	202607555	202611405	ENSMUSG00000041075	ENSMUSG00000041075	ENSMUSG00000041075	1	59426725	59431505	19	20		
668	2	202646596	202682633	ENSMUSG00000047361	ENSMUSG00000047361	ENSMUSG00000047361	1	59460978	59574628	19	20		
669	2	202838702	202876596	ENSMUSG00000026020	ENSMUSG00000026020	ENSMUSG00000026020	1	59629555	59656670	19	20		
670	2	202949916	203140719	ENSMUSG00000067336	ENSMUSG00000067336	ENSMUSG00000067336	1	59709199	59815028	19	20		
671	2	203208456	203342725	ENSMUSG00000041040	ENSMUSG00000041040	ENSMUSG00000041040	1	59857552	59929892	19	20		
672	2	203348946	203444611	ENSMUSG00000026018	ENSMUSG00000026018	ENSMUSG00000026018	1	59933614	59987633	19	20		
673	2	203485175	203556948	ENSMUSG00000026017	ENSMUSG00000026017	ENSMUSG00000026017	1	60052627	60095580	19	20		
674	2	203708718	203790962	ENSMUSG00000073664	ENSMUSG00000073664	ENSMUSG00000073664	1	60223296	60276006	19	20		
675	2	203811977	203878579	ENSMUSG00000049439	ENSMUSG00000049439	ENSMUSG00000049439	1	60287918	60331933	19	20		
676	2	203901483	204000320	ENSMUSG00000026782	ENSMUSG00000026782	ENSMUSG00000026782	1	60354280	60424050	19	20		
677	2	204012405	204108303	ENSMUSG00000026014	ENSMUSG00000026014	ENSMUSG00000026014	1	60437871	60477952	19	20		
678	2	204279492	204310801	ENSMUSG00000026012	ENSMUSG00000026012	ENSMUSG00000026012	1	60691012	60717905	19	20		
679	2	204440756	204446928	ENSMUSG00000026011	ENSMUSG00000026011	ENSMUSG00000026011	1	60853571	60860376	19	20		
680	2	204509716	204534541	ENSMUSG00000026009	ENSMUSG00000026009	ENSMUSG00000026009	1	60922460	60940237	19	20		
681	2	205118968	206188782	ENSMUSG00000052062	ENSMUSG00000052062	ENSMUSG00000052062	1	61573519	62572199	19	20		
682	2	206255468	206371099	ENSMUSG00000025969	ENSMUSG00000025969	ENSMUSG00000025969	1	62638420	62752961	19	20		
683	2	206576811	206659151	ENSMUSG00000040865	ENSMUSG00000040865	ENSMUSG00000040865	1	62992259	63020600	19	20		
684	2	206695783	206732392	ENSMUSG00000025968	ENSMUSG00000025968	ENSMUSG00000025968	1	63077861	63110985	19	20		
685	2	206748289	206790880	ENSMUSG00000046856	ENSMUSG00000046856	ENSMUSG00000046856	1	63116967	63148485	19	20		
686	2	207016784	207191124	ENSMUSG00000025964	ENSMUSG00000025964	ENSMUSG00000025964	1	63380180	63530791	19	20		
687	2	207311300	207338272	ENSMUSG00000025963	ENSMUSG00000025963	ENSMUSG00000025963	1	63633103	63664594	19	20		
688	2	207338577	207365477	ENSMUSG00000025962	ENSMUSG00000025962	ENSMUSG00000025962	1	63664927	63687661	19	20		
689	2	207653323	207738677	ENSMUSG00000025959	ENSMUSG00000025959	ENSMUSG00000025959	1	63969951	64055694	19	20		
690	2	208102931	208171806	ENSMUSG00000025958	ENSMUSG00000025958	ENSMUSG00000025958	1	64467080	64538826	19	20		
691	2	208185086	208197952	ENSMUSG00000025956	ENSMUSG00000025956	ENSMUSG00000025956	1	64540756	64551444	19	20		
692	2	208284720	208328213	ENSMUSG00000070871	ENSMUSG00000070871	ENSMUSG00000070871	1	64635157	64657915	19	20		
693	2	208338962	208342363	ENSMUSG00000045005	ENSMUSG00000045005	ENSMUSG00000045005	1	64668689	64672026	19	20		
694	2	208401291	208574608	ENSMUSG00000051344	ENSMUSG00000051344	ENSMUSG00000051344	1	64720260	64891100	19	20		
695	2	208694577	208697458	ENSMUSG00000067299	ENSMUSG00000067299	ENSMUSG00000067299	1	64996115	64997697	19	20		
696	2	208701108	208702872	ENSMUSG00000025952	ENSMUSG00000025952	ENSMUSG00000025952	1	65005810	65007787	19	20		
697	2	208715542	208719125	ENSMUSG00000073658	ENSMUSG00000073658	ENSMUSG00000073658	1	65014503	65016543	19	20		
698	2	208733709	208736633	ENSMUSG00000044429	ENSMUSG00000044429	ENSMUSG00000044429	1	65034665	65037639	19	20		
699	2	208738327	208762973	ENSMUSG00000044816	ENSMUSG00000044816	ENSMUSG00000044816	1	65041589	65057469	19	20		
700	2	208809199	208828051	ENSMUSG00000025950	ENSMUSG00000025950	ENSMUSG00000025950	1	65092904	65113390	19	20		
701	2	208839236	208931720	ENSMUSG00000025949	ENSMUSG00000025949	ENSMUSG00000025949	1	65120978	65208034	19	20		
702	2	208979801	209067469	ENSMUSG00000025946	ENSMUSG00000025946	ENSMUSG00000025946	1	65245533	65323520	19	20		
703	2	210152648	210304223	ENSMUSG00000015222	ENSMUSG00000015222	ENSMUSG00000015222	1	66184870	66375789	19	20		
704	2	210345042	210367788	ENSMUSG00000047839	ENSMUSG00000047839	ENSMUSG00000047839	1	66401621	66443893	19	20		
705	2	210402198	210543283	ENSMUSG00000055567	ENSMUSG00000055567	ENSMUSG00000055567	1	66560404	66629857	19	20		
706	2	210593692	210744296	ENSMUSG00000026004	ENSMUSG00000026004	ENSMUSG00000026004	1	66652571	66750754	19	20		
707	2	210760963	210798405	ENSMUSG00000026003	ENSMUSG00000026003	ENSMUSG00000026003	1	66764061	66796457	19	20		
708	2	210863122	210888142	ENSMUSG00000061816	ENSMUSG00000061816	ENSMUSG00000061816	1	66857506	66878240	19	20		
709	2	211004223	211049676	ENSMUSG00000026000	ENSMUSG00000026000	ENSMUSG00000026000	1	66933725	66972073	19	20		
710	2	211050678	211252074	ENSMUSG00000025991	ENSMUSG00000025991	ENSMUSG00000025991	1	67085484	67164467	19	20		
711	2	211956585	213111499	ENSMUSG00000062209	ENSMUSG00000062209	ENSMUSG00000062209	1	67973387	69040964	19	20		
712	2	213579589	213723303	ENSMUSG00000025997	ENSMUSG00000025997	ENSMUSG00000025997	1	69470598	69619029	19	20		
713	2	213857453	214983284	ENSMUSG00000053153	ENSMUSG00000053153	ENSMUSG00000053153	1	69760212	70658150	19	20		
714	2	214987163	214987579	ENSMUSG00000045648	ENSMUSG00000045648	ENSMUSG00000045648	1	70658923	70818605	19	20		
715	2	215301539	215382673	ENSMUSG00000026196	ENSMUSG00000026196	ENSMUSG00000026196	1	70963573	71036126	19	20		
716	2	215504522	215711396	ENSMUSG00000050296	ENSMUSG00000050296	ENSMUSG00000050296	1	71176508	71347790	19	20		
717	2	215885036	215922724	ENSMUSG00000026192	ENSMUSG00000026192	ENSMUSG00000026192	1	71490367	71512612	19	20		
718	2	215933426	216009036	ENSMUSG00000026193	ENSMUSG00000026193	ENSMUSG00000026193	1	71518731	71586380	19	20		
719	2	216516366	216586591	ENSMUSG00000039395	ENSMUSG00000039395	ENSMUSG00000039395	1	72092441	72145319	19	20		
720	2	216612243	216654709	ENSMUSG00000026189	ENSMUSG00000026189	ENSMUSG00000026189	1	72192385	72217512	19	20		
721	2	216654887	216675739	ENSMUSG00000026188	ENSMUSG00000026188	ENSMUSG00000026188	1	72217603	72236311	19	20		
722	2	216682377	216779248	ENSMUSG00000026187	ENSMUSG00000026187	ENSMUSG00000026187	1	72243643	72327187	19	20		
723	2	216830834	216944995	ENSMUSG00000039372	ENSMUSG00000039372	ENSMUSG00000039372	1	72360321	72470138	19	20		
724	2	216985441	217055952	ENSMUSG00000039354	ENSMUSG00000039354	ENSMUSG00000039354	1	72516478	72566341	19	20		
725	2	190249675	190319615	ENSMUSG00000039342	ENSMUSG00000039342	ENSMUSG00000039342	1	72576187	72633771	out-of-place			
726	2	217206372	217237404	ENSMUSG00000039323	ENSMUSG00000039323	ENSMUSG00000039323	1	72757699	72785670	19	21	here	Presence of a singleton made AutoGRAPH to break the block
727	2	217245074	217268516	ENSMUSG00000026185	ENSMUSG00000026185	ENSMUSG00000026185	1	72791272	72808072	19	21	presence of singleton	1 -> 2
728	2	217432431	217433027	ENSMUSG00000026182	ENSMUSG00000026182	ENSMUSG00000026182	1	72948284	72949106	19	21		
729	2	218375757	218517006	ENSMUSG00000055322	ENSMUSG00000055322	ENSMUSG00000055322	1	73846153	73858909	19	21		
730	2	218642223	218663548	ENSMUSG00000061815	ENSMUSG00000061815	ENSMUSG00000061815	1	74058748	74081430	19	21		
731	2	218790119	218827315	ENSMUSG00000006304	ENSMUSG00000006304	ENSMUSG00000006304	1	74169784	74197664	19	21		
732	2	218832464	218836826	ENSMUSG00000064272	ENSMUSG00000064272	ENSMUSG00000064272	1	74211807	74212796	19	21		
733	2	218837097	218843137	ENSMUSG00000006299	ENSMUSG00000006299	ENSMUSG00000006299	1	74213054	74217873	19	21		
734	2	218843457	218919756	ENSMUSG00000026179	ENSMUSG00000026179	ENSMUSG00000026179	1	74218262	74286878	19	21		
735	2	218847161	218865524	ENSMUSG00000006301	ENSMUSG00000006301	ENSMUSG00000006301	1	74221461	74237367	19	21		
736	2	218929823	218941060	ENSMUSG00000073650	ENSMUSG00000073650	ENSMUSG00000073650	1	74295315	74302528	19	21		
737	2	218955203	218968994	ENSMUSG00000026177	ENSMUSG00000026177	ENSMUSG00000026177	1	74308410	74319259	19	21		
738	2	218972722	218978907	ENSMUSG00000026176	ENSMUSG00000026176	ENSMUSG00000026176	1	74324806	74330492	19	21		
739	2	218992089	219022491	ENSMUSG00000026175	ENSMUSG00000026175	ENSMUSG00000026175	1	74342615	74368752	19	21		
740	2	219023218	219141293	ENSMUSG00000033364	ENSMUSG00000033364	ENSMUSG00000033364	1	74372319	74477493	19	21		
741	2	219141775	219168717	ENSMUSG00000026174	ENSMUSG00000026174	ENSMUSG00000026174	1	74438458	74464048	19	21		
742	2	219180866	219210140	ENSMUSG00000026173	ENSMUSG00000026173	ENSMUSG00000026173	1	74477734	74499184	19	21		
743	2	219210883	219232599	ENSMUSG00000026135	ENSMUSG00000026135	ENSMUSG00000026135	1	74499649	74521213	19	21		
744	2	219231772	219236399	ENSMUSG00000026172	ENSMUSG00000026172	ENSMUSG00000026172	1	74521568	74525650	19	21		
745	2	219236832	219245025	ENSMUSG00000026171	ENSMUSG00000026171	ENSMUSG00000026171	1	74526960	74534604	19	21		
746	2	219245053	219275678	ENSMUSG00000033276	ENSMUSG00000033276	ENSMUSG00000033276	1	74534687	74570098	19	21		
747	2	219310541	219328380	ENSMUSG00000033257	ENSMUSG00000033257	ENSMUSG00000033257	1	74594961	74631182	19	21		
748	2	219354745	219388254	ENSMUSG00000026170	ENSMUSG00000026170	ENSMUSG00000026170	1	74646781	74671097	19	21		
749	2	219395350	219404758	ENSMUSG00000006542	ENSMUSG00000006542	ENSMUSG00000006542	1	74672706	74682162	19	21		
750	2	219432783	219447192	ENSMUSG00000033227	ENSMUSG00000033227	ENSMUSG00000033227	1	74705165	74718155	19	21		
751	2	219453488	219466889	ENSMUSG00000026167	ENSMUSG00000026167	ENSMUSG00000026167	1	74724723	74737386	19	21		
752	2	219554053	219558623	ENSMUSG00000055197	ENSMUSG00000055197	ENSMUSG00000055197	1	74814716	74818626	19	21		
753	2	219563156	219566371	ENSMUSG00000006546	ENSMUSG00000006546	ENSMUSG00000006546	1	74823151	74826342	19	21		
754	2	219575820	219614489	ENSMUSG00000047021	ENSMUSG00000047021	ENSMUSG00000047021	1	74835287	74868806	19	21		
755	2	219628173	219633433	ENSMUSG00000006538	ENSMUSG00000006538	ENSMUSG00000006538	1	74878522	74884858	19	21		
756	2	219734434	219742959	ENSMUSG00000026205	ENSMUSG00000026205	ENSMUSG00000026205	1	75008751	75017098	19	21		
757	2	219745140	219750976	ENSMUSG00000033159	ENSMUSG00000033159	ENSMUSG00000033159	1	75018424	75025579	19	21		
758	2	219751198	219756456	ENSMUSG00000049339	ENSMUSG00000049339	ENSMUSG00000049339	1	75026005	75030266	19	21		
759	2	219779759	219782272	ENSMUSG00000026197	ENSMUSG00000026197	ENSMUSG00000026197	1	75051857	75054769	19	21		
760	2	219782738	219791797	ENSMUSG00000026198	ENSMUSG00000026198	ENSMUSG00000026198	1	75054785	75063430	19	21		
761	2	219792347	219802595	ENSMUSG00000033124	ENSMUSG00000033124	ENSMUSG00000033124	1	75065736	75073552	19	21		
762	2	219802786	219809615	ENSMUSG00000026199	ENSMUSG00000026199	ENSMUSG00000026199	1	75078204	75082332	19	21		
763	2	219809577	219818395	ENSMUSG00000026200	ENSMUSG00000026200	ENSMUSG00000026200	1	75081391	75093926	19	21		
764	2	219818436	219821870	ENSMUSG00000026201	ENSMUSG00000026201	ENSMUSG00000026201	1	75094003	75098754	19	21		
765	2	219822677	219826882	ENSMUSG00000026202	ENSMUSG00000026202	ENSMUSG00000026202	1	75098770	75100582	19	21		
766	2	219852342	219859866	ENSMUSG00000026203	ENSMUSG00000026203	ENSMUSG00000026203	1	75119635	75128838	19	21		
767	2	219862591	219882387	ENSMUSG00000026204	ENSMUSG00000026204	ENSMUSG00000026204	1	75130198	75147299	19	21		
768	2	219900375	219905595	ENSMUSG00000033061	ENSMUSG00000033061	ENSMUSG00000033061	1	75155350	75161528	19	21		
769	2	219946512	219960981	ENSMUSG00000026209	ENSMUSG00000026209	ENSMUSG00000026209	1	75191807	75199951	19	21		
770	2	219991343	219999705	ENSMUSG00000026208	ENSMUSG00000026208	ENSMUSG00000026208	1	75243492	75250864	19	21		
771	2	220007812	220066598	ENSMUSG00000026207	ENSMUSG00000026207	ENSMUSG00000026207	1	75258588	75314596	19	21		
772	2	220071854	220079901	ENSMUSG00000033021	ENSMUSG00000033021	ENSMUSG00000033021	1	75319132	75326322	19	21		
773	2	220087296	220111730	ENSMUSG00000033007	ENSMUSG00000033007	ENSMUSG00000033007	1	75333847	75357488	19	21		
774	2	220111913	220116753	ENSMUSG00000032997	ENSMUSG00000032997	ENSMUSG00000032997	1	75357717	75362549	19	21		
775	2	220116629	220123545	ENSMUSG00000051703	ENSMUSG00000051703	ENSMUSG00000051703	1	75362728	75368853	19	21		
776	2	220123722	220144207	ENSMUSG00000026211	ENSMUSG00000026211	ENSMUSG00000026211	1	75373687	75388789	19	21		
777	2	220145161	220148679	ENSMUSG00000032968	ENSMUSG00000032968	ENSMUSG00000032968	1	75390225	75393502	19	21		

778	2	220170826	220189417	ENSMUSG00000026213	ENSMUSG00000026213	ENSMUSG00000026213	1	75404694	75420483	19	21
779	2	220200529	220214936	ENSMUSG00000006576	ENSMUSG00000006576	ENSMUSG00000006576	1	75429419	75442231	19	21
780	2	221990993	222145254	ENSMUSG00000026235	ENSMUSG00000026235	ENSMUSG00000026235	1	77250333	77398236	19	21
781	2	222774137	222871944	ENSMUSG00000004872	ENSMUSG00000004872	ENSMUSG00000004872	1	77985948	78080283	19	21
782	2	222997566	223131861	ENSMUSG00000032908	ENSMUSG00000032908	ENSMUSG00000032908	1	78193492	78303436	19	21
783	2	223143506	223229049	ENSMUSG00000026245	ENSMUSG00000026245	ENSMUSG00000026245	1	78301105	78372006	19	21
784	2	223244749	223282860	ENSMUSG00000012187	ENSMUSG00000012187	ENSMUSG00000012187	1	78395038	78421317	19	21
785	2	223625171	223626872	ENSMUSG00000047330	ENSMUSG00000047330	ENSMUSG00000047330	1	78695945	78699050	19	21
786	2	224169906	224175369	ENSMUSG00000050711	ENSMUSG00000050711	ENSMUSG00000050711	1	79313693	79319114	19	21
787	2	224448309	224518296	ENSMUSG00000004377	ENSMUSG00000004377	ENSMUSG00000004377	1	79605511	79640793	19	21
788	2	224530378	224540668	ENSMUSG00000026248	ENSMUSG00000026248	ENSMUSG00000026248	1	79655157	79660468	19	21
789	2	224548123	224612237	ENSMUSG00000026249	ENSMUSG00000026249	ENSMUSG00000026249	1	79673221	79737660	19	21
790	2	224806981	224974729	ENSMUSG00000043230	ENSMUSG00000043230	ENSMUSG00000043230	1	80077730	80092968	19	21
791	2	225043113	225158358	ENSMUSG00000004364	ENSMUSG00000004364	ENSMUSG00000004364	1	80145842	80219466	19	21
792	2	225338052	225615335	ENSMUSG00000038608	ENSMUSG00000038608	ENSMUSG00000038608	1	80380212	80471856	19	21
793	2	225981841	226417571	ENSMUSG00000054976	ENSMUSG00000054976	ENSMUSG00000054976	1	80956341	81150688	19	21
794	2	227308182	227372719	ENSMUSG00000055980	ENSMUSG00000055980	ENSMUSG00000055980	1	82115634	82170440	19	21
795	2	227409017	227569835	ENSMUSG00000026142	ENSMUSG00000026142	ENSMUSG00000026142	1	82195621	82323558	19	21
796	2	227898259	227930141	ENSMUSG00000026150	ENSMUSG00000026150	ENSMUSG00000026150	1	82603942	82631418	19	21
797	2	227935120	227952264	ENSMUSG00000026149	ENSMUSG00000026149	ENSMUSG00000026149	1	82635673	82647480	19	21
798	2	228045286	228130548	ENSMUSG00000026159	ENSMUSG00000026159	ENSMUSG00000026159	1	82718507	82775299	19	21
799	2	228386814	228390494	ENSMUSG00000026166	ENSMUSG00000026166	ENSMUSG00000026166	1	82995806	83005701	19	21
800	2	228444014	228497301	ENSMUSG00000053161	ENSMUSG00000053161	ENSMUSG00000053161	1	83038823	83089210	19	21
801	2	228552919	228754586	ENSMUSG00000026163	ENSMUSG00000026163	ENSMUSG00000026163	1	83136151	83241354	19	21
802	2	229596955	229805014	ENSMUSG00000045658	ENSMUSG00000045658	ENSMUSG00000045658	1	83915323	84163616	19	21
803	2	230340514	230453039	ENSMUSG00000026219	ENSMUSG00000026219	ENSMUSG00000026219	1	84603331	84693945	19	21
804	2	230495456	230584171	ENSMUSG00000073633	ENSMUSG00000073633	ENSMUSG00000073633	1	84718898	84779509	19	21
805	2	230607944	230641863	ENSMUSG00000026220	ENSMUSG00000026220	ENSMUSG00000026220	1	84784923	84814159	19	21
806	2	231437865	231452206	ENSMUSG00000026223	ENSMUSG00000026223	ENSMUSG00000026223	1	87725736	87739863	19	21
807	2	231480278	231498185	ENSMUSG00000049608	ENSMUSG00000049608	ENSMUSG00000049608	1	87770708	87792178	19	21
808	2	231569193	231575748	ENSMUSG00000026226	ENSMUSG00000026226	ENSMUSG00000026226	1	87853117	87861129	19	21
809	2	231610906	231619980	ENSMUSG00000026227	ENSMUSG00000026227	ENSMUSG00000026227	1	87877439	87886626	19	21
810	2	231629836	231745712	ENSMUSG00000026229	ENSMUSG00000026229	ENSMUSG00000026229	1	87895790	87970466	19	21
811	2	231681199	231698068	ENSMUSG00000026228	ENSMUSG00000026228	ENSMUSG00000026228	1	87930208	87943141	19	21
812	2	231771589	231918151	ENSMUSG00000062590	ENSMUSG00000062590	ENSMUSG00000062590	1	87985999	88165803	19	21
813	2	232096116	232103452	ENSMUSG00000026237	ENSMUSG00000026237	ENSMUSG00000026237	1	88217496	88219312	19	21
814	2	232165856	232167236	ENSMUSG00000036574	ENSMUSG00000036574	ENSMUSG00000036574	1	88257500	88259211	19	21
815	2	232305380	232354217	ENSMUSG00000026239	ENSMUSG00000026239	ENSMUSG00000026239	1	88374186	88413672	19	21
816	2	232359402	232381652	ENSMUSG00000026240	ENSMUSG00000026240	ENSMUSG00000026240	1	88418271	88437671	19	21
817	2	232498379	232499203	ENSMUSG00000026241	ENSMUSG00000026241	ENSMUSG00000026241	1	88497462	88501743	19	21
818	2	232587882	232909542	ENSMUSG00000053333	ENSMUSG00000053333	ENSMUSG00000053333	1	88534990	88881266	19	21
819	2	233052781	233060782	ENSMUSG00000026247	ENSMUSG00000026247	ENSMUSG00000026247	1	88978833	88987527	19	21
820	2	233099166	233108449	ENSMUSG00000026251	ENSMUSG00000026251	ENSMUSG00000026251	1	89022015	89031240	19	21
821	2	233112702	233120790	ENSMUSG00000026253	ENSMUSG00000026253	ENSMUSG00000026253	1	89036982	89043865	19	21
822	2	233206182	233255703	ENSMUSG00000026255	ENSMUSG00000026255	ENSMUSG00000026255	1	89095534	89142009	19	21
823	2	233270300	233431562	ENSMUSG00000048000	ENSMUSG00000048000	ENSMUSG00000048000	1	89158223	89281959	19	21
824	2	233443238	233449351	ENSMUSG00000026258	ENSMUSG00000026258	ENSMUSG00000026258	1	89301442	89306652	19	21
825	2	233451665	233586182	ENSMUSG00000026259	ENSMUSG00000026259	ENSMUSG00000026259	1	89308011	89405041	19	21
826	2	233633433	233778102	ENSMUSG00000026288	ENSMUSG00000026288	ENSMUSG00000026288	1	89451660	89549455	19	21
827	2	233825039	233869051	ENSMUSG00000026289	ENSMUSG00000026289	ENSMUSG00000026289	1	89587242	89623593	19	21
828	2	233881232	233920297	ENSMUSG00000056055	ENSMUSG00000056055	ENSMUSG00000056055	1	89634851	89676194	19	21
829	2	234050777	234134623	ENSMUSG00000005501	ENSMUSG00000005501	ENSMUSG00000005501	1	89776292	89839722	19	21
830	2	234316576	234317301	ENSMUSG00000048591	ENSMUSG00000048591	ENSMUSG00000048591	1	90036121	90036849	19	21
831	2	234490782	234592905	ENSMUSG00000036251	ENSMUSG00000036251	ENSMUSG00000036251	1	90150132	90220022	19	21
832	2	234624062	234650513	ENSMUSG00000026295	ENSMUSG00000026295	ENSMUSG00000026295	1	90238190	90257609	19	21
833	2	235066424	235069983	ENSMUSG00000049866	ENSMUSG00000049866	ENSMUSG00000049866	1	90504298	90533047	19	21
834	2	235552068	235629029	ENSMUSG00000036206	ENSMUSG00000036206	ENSMUSG00000036206	1	90901586	90984963	19	21
835	2	236739046	236741391	ENSMUSG00000034486	ENSMUSG00000034486	ENSMUSG00000034486	1	91759133	91762347	19	21
836	2	237143182	237155730	ENSMUSG00000044337	ENSMUSG00000044337	ENSMUSG00000044337	1	92035174	92047459	19	21
837	2	237658823	237672226	ENSMUSG00000034432	ENSMUSG00000034432	ENSMUSG00000034432	1	92433385	92443499	19	21
838	2	237897401	237987559	ENSMUSG00000048126	ENSMUSG00000048126	ENSMUSG00000048126	1	92596517	92674180	19	21
839	2	238060669	238127375	ENSMUSG00000026303	ENSMUSG00000026303	ENSMUSG00000026303	1	92745509	92781547	19	21
840	2	238139956	238140557	ENSMUSG00000034397	ENSMUSG00000034397	ENSMUSG00000034397	1	92783511	92784418	19	21
841	2	238147704	238164477	ENSMUSG00000026304	ENSMUSG00000026304	ENSMUSG00000026304	1	92788541	92800026	19	21
842	2	238265661	238338645	ENSMUSG00000026305	ENSMUSG00000026305	ENSMUSG00000026305	1	92884064	92946470	19	21
843	2	238372193	238416186	ENSMUSG00000070732	ENSMUSG00000070732	ENSMUSG00000070732	1	92975509	93001201	19	21
844	2	238634409	238671735	ENSMUSG00000026307	ENSMUSG00000026307	ENSMUSG00000026307	1	93128744	93151480	19	21
845	2	238673765	238706666	ENSMUSG00000049515	ENSMUSG00000049515	ENSMUSG00000049515	1	93152481	93178709	19	21
846	2	238714135	238724442	ENSMUSG00000026308	ENSMUSG00000026308	ENSMUSG00000026308	1	93181479	93192810	19	21
847	2	238732388	238740431	ENSMUSG00000047443	ENSMUSG00000047443	ENSMUSG00000047443	1	93196836	93204622	19	21
848	2	238743783	238777075	ENSMUSG00000026309	ENSMUSG00000026309	ENSMUSG00000026309	1	93206237	93229189	19	21
849	2	238812207	238813418	ENSMUSG00000067071	ENSMUSG00000067071	ENSMUSG00000067071	1	93241889	93243628	19	21
850	2	238817425	238861831	ENSMUSG00000055866	ENSMUSG00000055866	ENSMUSG00000055866	1	93246388	93289702	19	21
851	2	238893980	238972531	ENSMUSG00000034292	ENSMUSG00000034292	ENSMUSG00000034292	1	93325073	93358670	19	21
852	2	239000365	239020110	ENSMUSG00000026311	ENSMUSG00000026311	ENSMUSG00000026311	1	93371120	93389585	19	21
853	2	239635329	239987580	ENSMUSG00000026313	ENSMUSG00000026313	ENSMUSG00000026313	1	93763753	93885379	19	21
854	2	240571607	240613391	ENSMUSG00000026260	ENSMUSG00000026260	ENSMUSG00000026260	1	94269894	94304205	19	21
855	2	240714653	240724437	ENSMUSG00000073616	ENSMUSG00000073616	ENSMUSG00000073616	1	94467564	94472391	19	21
856	2	240727119	240728746	ENSMUSG00000044055	ENSMUSG00000044055	ENSMUSG00000044055	1	94474625	94479247	19	21
857	2	241023788	241056168	ENSMUSG00000034220	ENSMUSG00000034220	ENSMUSG00000034220	1	94662092	94690602	19	21
858	2	241067512	241146078	ENSMUSG00000034212	ENSMUSG00000034212	ENSMUSG00000034212	1	94700535	94733312	19	21
859	2	241148144	241152104	ENSMUSG00000047067	ENSMUSG00000047067	ENSMUSG00000047067	1	94737395	94739026	19	21
860	2	241156777	241166814	ENSMUSG00000026269	ENSMUSG00000026269	ENSMUSG00000026269	1	94741811	94750986	19	21
861	2	241174825	241187194	ENSMUSG00000026270	ENSMUSG00000026270	ENSMUSG00000026270	1	94764782	94778346	19	21
862	2	241456835	241467210	ENSMUSG00000026272	ENSMUSG00000026272	ENSMUSG00000026272	1	94965651	94975813	19	21
863	2	241475163	241484087	ENSMUSG00000034159	ENSMUSG00000034159	ENSMUSG00000034159	1	94981762	94991354	19	21
864	2	241618553	241681156	ENSMUSG00000047793	ENSMUSG00000047793	ENSMUSG00000047793	1	95066395	95126207	19	21
865	2	241675180	241690397	ENSMUSG00000026273	ENSMUSG00000026273	ENSMUSG00000026273	1	95131866	95135515	19	21
866	2	241694385	241737523	ENSMUSG00000026274	ENSMUSG00000026274	ENSMUSG00000026274	1	95139851	95173158	19	21
867	2	241738578	241771712	ENSMUSG00000026275	ENSMUSG00000026275	ENSMUSG00000026275	1	95174051	95198024	19	21
868	2	241776597	241812643	ENSMUSG00000034107	ENSMUSG00000034107	ENSMUSG00000034107	1	95204302	95233908	19	21
869	2	241816817	241903908	ENSMUSG00000034088	ENSMUSG00000034088	ENSMUSG00000034088	1	95236346	95309214	19	21
870	2	241903396	241942115	ENSMUSG00000026276	ENSMUSG00000026276	ENSMUSG00000026276	1	95309450	95340136	19	21
871	2	241944384	242082928	ENSMUSG00000034066	ENSMUSG00000034066	ENSMUSG00000034066	1	95358974	95452378	19	21
872	2	242083108	242096707	ENSMUSG00000026277	ENSMUSG00000026277	ENSMUSG00000026277	1	95452314	95466128	19	21
873	2	242146865	242162222	ENSMUSG00000026278	ENSMUSG00000026278	ENSMUSG00000026278	1	95516100	95526168	19	21
874	2	242172494	242225328	ENSMUSG00000026279	ENSMUSG00000026279	ENSMUSG00000026279	1	95535797	95585244	19	21
875	2	242263830	242274900	ENSMUSG00000026281	ENSMUSG00000026281	ENSMUSG00000026281	1	95622996	95632299	19	21
876	2	242290129	242317560	ENSMUSG00000026283	ENSMUSG00000026283	ENSMUSG00000026283	1	95634371	95652507	19	21
877	2	242322728	242356897	ENSMUSG00000073609	ENSMUSG00000073609	ENSMUSG00000073609	1	95655699	95681232	19	21
878	2	242400703	242407407	ENSMUSG00000034000	ENSMUSG00000034000	ENSMUSG00000034000	1	95852002	95855822	19	21
879	2	242440711	242449731	ENSMUSG00000026285	ENSMUSG00000026285	ENSMUSG00000026285	1	95868708	95882959	19	21
880	5	99898908	99950344	ENSMUSG00000051185	ENSMUSG00000051185	ENSMUSG00000051185	1	97144034	97158351	20	22
881	5	100170803	100266869	ENSMUSG00000040710	ENSMUSG00000040710	ENSMUSG00000040710	1	97418090	97497995	20	22
882	5	101597589	101660152	ENSMUSG00000040693	ENSMUSG00000040693	ENSMUSG00000040693	1	98648137	98702541	20	22
883	5	102622341	102642260	ENSMUSG00000044768	ENSMUSG00000044768	ENSMUSG00000044768	1	99474307	99492382	20	22
884	5	102483867	102566806	ENSMUSG00000040648	ENSMUSG00000040648	ENSMUSG00000040648	1	99536566	99600434	20	22
885	5	102460258	102483754	ENSMUSG00000026333	ENSMUSG00000026333	ENSMUSG00000026333	1	99600601	99624114	20	22
886	5	102229426	102393315	ENSMUSG00000026335	ENSMUSG00000026335	ENSMUSG00000026335	1	99651500	99925919	20	22
887	18	57308755	57373345	ENSMUSG00000050840	ENSMUSG00000050840	ENSMUSG00000050840	1	106596366	106823028	21	23
888	18	57631583	57711284	ENSMUSG00000047496	ENSMUSG00000047496	ENSMUSG00000047496	1	107107478	107184217	21	23
889	18	57862440	58005206	ENSMUSG00000056536	ENSMUSG00000056536	ENSMUSG00000056536	1	107348686	107486555	21	23
890	18	58005521	58125333	ENSMUSG00000026319	ENSMUSG00000026319	ENSMUSG00000026319	1	107491460	107581622	21	23
891	18	58143566	58204482	ENSMUSG00000026321	ENSMUSG00000026321	ENSMUSG00000026321	1	107608270	107672751	21	23
892	18	58342474	58404942	ENSMUSG00000038866	ENSMUSG00000038866	ENSMUSG00000038866	1	107818236	107859710	21	23
893	18	58533897	58798645	ENSMUSG00000044340	ENSMUSG00000044340	ENSMUSG00000044340	1	107999551	108221791	21	23
894	18	58941559	59137593	ENSMUSG00000057329	ENSMUSG00000057329	ENSMUSG00000057329	1	108365740	108541821	21	23
895	18	59148813	59185438	ENSMUSG00000009905	ENSMUSG00000009905	ENSMUSG00000009905	1	108551445	108587274	21	23
896	18	59207407	59240673	ENSMUSG00000009907	ENSMUSG00000009907	ENSMUSG00000009907	1	108596985	108623994	21	23
897	18	59295199	59323297	ENSMUSG00000067006	ENSMUSG00000067006	ENSMUSG00000067006	1	108688727	108710895	21	23
898	18	59374373	59385224	ENSMUSG00000059956	ENSMUSG00000059956	ENSMUSG00000059956	1	108761996	108784625	21	23
899	18	59406861	59415650	ENSMUSG00000048775	ENSMUSG00000048775	ENSMUSG00000048775	1	108808531	108828742	21	23
900	18	59528408	59541613	ENSMUSG00000026327	ENSMUSG00000026327	ENSMUSG00000026327	1	109189861	109208022	21	23
901	18	59571282	59623583	ENSMUSG00000067001	ENSMUSG00000067001	ENSMUSG00000067001	1	109250236	109280235	21	23
902	18	59705922	59722100	ENSMUSG00000062345	ENSMUSG00000062345	ENSMUSG00000062345	1	109338970	109353139	21	23
903	18	59788243	59807585	ENSMUSG00000026315	ENSMUSG00000026315	ENSMUSG00000026315	1	109417618	109436456	21	23
904	18	61568468	61699155	ENSMUSG00000026312	ENSMUSG00000026312	ENSMUSG00000026312	1	111810849	111966736	21	23
905	18	63324799	63334947	ENSMUSG00000038702	ENSMUSG00000038702	ENSMUSG00000038702	1	113688559	113692465	21	23
906	2	122229826	122239413	ENSMUSG00000026374	ENSMUSG00000026374	ENSMUSG00000026374	1	120128350	120137412	22	24
907	2	122201027	122210938	ENSMUSG00000026377	ENSMUSG00000026377	ENSMUSG00000026377	1	120149423	120161370	22	24
908	2	121811825	122123509	ENSMUSG00000064302	ENSMUSG00000064302	ENSMUSG00000064302	1	120247270	120434537	22	24
909	2	121695003	121759248	ENSMUSG00000026380	ENSMUSG00000026380	ENSMUSG00000026380	1	120455491	120504980	22	24
910	2	121271367	121464721	ENSMUSG00000048402	ENSMUSG00000048402	ENSMUSG00000048402	1	120665416	120763549	22	24
911	2	120819469	120825444	ENSMUSG00000037035	ENSMUSG00000037035	ENSMUSG00000037035	1	121243011	121249794	22	24
912	2	120752711	120768753	ENSMUSG00000004451	ENSMUSG00000004451	ENSMUSG00000004451	1	121297858	121332182	22	24
913	2	120487139	120653164	ENSMUSG00000026383	ENSMUSG00000026383	ENSMUSG00000026383	1	121375677	121476482	22	24
914	2	120233677	120451507	ENSMUSG00000026384	ENSMUSG00000026384	ENSMUSG00000026384	1	121487291	121630021	22	24
915	2	120153213	120156164	ENSMUSG00000036975	ENSMUSG00000036975	ENSMUSG00000036975	1	121735436	121740714	22	24
916	2	120018513	120130706	ENSMUSG00000036962	ENSMUSG00000036962	ENSMUSG00000036962	1	121751065	121781747	22	24
917	2	119913891	119998537	ENSMUSG00000026387	ENSMUSG00000026387	ENSMUSG00000026387	1	121834524	121890828	22	24
918	2	119905950	119911486	ENSMUSG00000050777	ENSMUSG00000050777	ENSMUSG00000050777	1	121894923	121901326	22	24
919	2	119776279	119840848	ENSMUSG00000026388	ENSMUSG00000026388	ENSMUSG00000026388	1	121948733	122015728	22	24
920	2	119697854	119739698	ENSMUSG00000026389	ENSMUSG00000026389	ENSMUSG00000026389	1	122054178	122097776	22	24
921	2	119630289	119632935	ENSMUSG00000036907	ENSMUSG00000036907	ENSMUSG00000036907	1	122168128	122170720	22	24
922	2	119416215	119468706	ENSMUSG00000026390	ENSMUSG00000026390	ENSMUSG00000026390	1	122302084	122332630	22	24
923	2	119316236	119322229	ENSMUSG00000058665	ENSMUSG00000058665	ENSMUSG00000058665	1	122430227	122435517	22	24
924	2	118562520	118584066	ENSMUSG00000003721	ENSMUSG00000003721	ENSMUSG00000003721	1	123131899	123155308	22	24
925	2	118389524	118488179	ENSMUSG00000026339	ENSMUSG00000026339	ENSMUSG00000026339	1	123258613	123329139	22	24
926	2	118333473	118377381	ENSMUSG00000050534	ENSMUSG00000050534	ENSMUSG00000050534	1	123337304	123355981	22	24
927	2	118288725	118306423	ENSMUSG00000001674	ENSMUSG00000001674	ENSMUSG00000001674	1	123381385	123395480	22	24
928	2	115636271	116318406	ENSMUSG00000036815	ENSMUSG00000036815	ENSMUSG00000036815	1	125159688	125873098	22	24
929	2	114364007	114432635	ENSMUSG00000026341	ENSMUSG00000026341	ENSMUSG00000026341	1	127220455	127263087	22	24
930	2	114188401	114230870	ENSMUSG00000026342	ENSMUSG00000026342	ENSMUSG00000026342	1	127388569	127423360	22	24
931	2	132891086	133119649	ENSMUSG00000026343	ENSMUSG00000026343	ENSMUSG00000026343	1	127504539	127701399	23	25
932	2	133120014	133144252	ENSMUSG00000026344	ENSMUSG00000026344	ENSMUSG00000026344	1	127699589	127739760	23	25
933	2	134725638	134922938	ENSMUSG00000036155	ENSMUSG00000036155	ENSMUSG00000036155	1	129032564	129310613	23	25
934	2	134930031	135193040	ENSMUSG00000026347	ENSMUSG00000026347	ENSMUSG00000026347	1	129314090	129505567	23	25
935	2	135312723	135375900	ENSMUSG00000026348	ENSMUSG00000026348	ENSMUSG00000026348	1	129556959	129595110	23	25
936	2	135392863	135428688	ENSMUSG00000026349	ENSMUSG00000026349	ENSMUSG00000026349	1	129601763	129631105	23	25
937	2	135526323	135644014	ENSMUSG00000036104	ENSMUSG00000036104	ENSMUSG00000036104	1	129696332	129771414	23	25
938	2	135674046	136005276	ENSMUSG00000036086	ENSMUSG00000036086	ENSMUSG00000036086	1	129781737	129919549	23	25
939	2	136005553	136199306	ENSMUSG00000056211	ENSMUSG00000056211	ENSMUSG00000056211	1	129930852	130065281	23	25
940	2	136215808	136259093	ENSMUSG00000026353	ENSMUSG00000026353	ENSMUSG00000026353	1	130071727	130106923	23	25
941	2	136261885	136311220	ENSMUSG00000026354	ENSMUSG00000026354	ENSMUSG00000026354	1	130112668	130155849	23	25
942	2	136313674	136350481	ENSMUSG00000026355	ENSMUSG00000026355	ENSMUSG00000026355	1	130159137	130187210	23	25
943	2	136380724	136459692	ENSMUSG00000026356	ENSMUSG00000026356	ENSMUSG00000026356	1	130191254	130244884	23	25
944	2	136588909	136589979	ENSMUSG00000045382	ENSMUSG00000045382	ENSMUSG00000045382	1	130415745	130419836	23	25
945	2	137530536	138151757	ENSMUSG00000042581	ENSMUSG00000042581	ENSMUSG00000042581	1	131101020	132046826	23	25
946	1	205344230	205384940	ENSMUSG00000026405	ENSMUSG00000026405	ENSMUSG00000026405	1	132462319	132489145	24	26
947	1	205293228	205317791	ENSMUSG00000026409	ENSMUSG00000026409	ENSMUSG00000026409	1	132516728	132543360	24	26
948	1	205283817	205291045	ENSMUSG00000046404	ENSMUSG00000046404	ENSMUSG00000046404	1	132544873	132549566	24	26
949	1	205258489	205272715	ENSMUSG00000042510	ENSMUSG00000042510	ENSMUSG00000042510	1	132559632	132572167	24	26
950	1	205198484	205210093	ENSMUSG00000026415	ENSMUSG00000026415	ENSMUSG00000026415	1	132628448	132642286	24	26
951	1	205168486	205186434	ENSMUSG00000026417	ENSMUSG00000026417	ENSMUSG00000026417	1	132654297	132866011	24	26
952	1	205144354	205161966	ENSMUSG00000042474	ENSMUSG00000042474	ENSMUSG00000042474	1	132693323	132708336	24	26
953	1	205137411	205143448	ENSMUSG00000026420	ENSMUSG00000026420	ENSMUSG00000026420	1	132709620	132713542	24	26
954	1	205105322	205109191	ENSMUSG00000026416	ENSMUSG00000026416	ENSMUSG00000026416	1	132734531	132738997	24	26
955	1	205038838	205082949	ENSMUSG00000016524	ENSMUSG00000016524	ENSMUSG00000016524	1	132760202	132766787	24	26
956	1	205007570	205012462	ENSMUSG00000016529	ENSMUSG00000016529	ENSMUSG00000016529	1	132847393	132852516	24	26
957	1	204925005	204974251	ENSMUSG00000016528	ENSMUSG00000016528	ENSMUSG00000016528	1	132881250	132925113	24	26
958	1	204875504	204889164	ENSMUSG00000016526	ENSMUSG00000016526	ENSMUSG00000016526	1	132955987	132965780	24	26
959	1	204831600	204852527	ENSMUSG00000026427	ENSMUSG00000026427	ENSMUSG00000026427	1	132980742	133007538	24	26
960	1	204747502	204827667	ENSMUSG00000026430	ENSMUSG00000026430	ENSMUSG00000026430	1	133003956	133072724	24	26
961	1	204710414	204736846	ENSMUSG00000042349	ENSMUSG00000042349	ENSMUSG00000042349	1	133082148	133107109	24	26
962	1	204391064	204398262	ENSMUSG00000026432	ENSMUSG00000026432	ENSMUSG00000026432	1	133427287	133439546	24	26
963	1	204484082	204498727	ENSMUSG00000004552	ENSMUSG00000004552	ENSMUSG00000004552	1	133465860	133503051	24	26
964	1	204148799	204171573	ENSMUSG00000042268	ENSMUSG00000042268	ENSMUSG00000042268	1	133571568	133597951	24	26
965	1	204063773	204085868	ENSMUSG00000042251	ENSMUSG00000042251	ENSMUSG00000042251	1	133624944	133649018	24	26
966	1	204024844	204048927	ENSMUSG00000013275	ENSMUSG00000013275	ENSMUSG00000013275	1	133655523	133676409	24	26
967	1	204003737	204011197	ENSMUSG00000026433	ENSMUSG00000026433	ENSMUSG00000026433	1	133694838	133700433	24	26
968	1	203948570	203986027	ENSMUSG00000026434	ENSMUSG00000026434	ENSMUSG00000026434	1	133738080	133760518	24	26
969	1	203893602	203916210	ENSMUSG00000026435	ENSMUSG00000026435	ENSMUSG00000026435	1	133790513	133809861	24	26
970	1	203833330	203867644	ENSMUSG00000026436	ENSMUSG00000026436	ENSMUSG00000026436	1	133835422	133851209	24	26
971	1	203804728	203838669	ENSMUSG00000059149	ENSMUSG00000059149	ENSMUSG00000059149	1	133850359	133895457	24	26
972	1	203740350	203768536	ENSMUSG00000026437	ENSMUSG00000026437	ENSMUSG00000026437	1	133941096	133967174	24	26

973	1	203572271	203579355	ENSMUSG00000042115	ENSMUSG00000042115	ENSMUSG00000042115	1	134126823	134134903	24	26
974	1	203537810	203557506	ENSMUSG00000009772	ENSMUSG00000009772	ENSMUSG00000009772	1	134143696	134160834	24	26
975	1	203463661	203509088	ENSMUSG00000042066	ENSMUSG00000042066	ENSMUSG00000042066	1	134183861	134218827	24	26
976	1	203378255	203447322	ENSMUSG00000042046	ENSMUSG00000042046	ENSMUSG00000042046	1	134245114	134294506	24	26
977	1	203321893	203357766	ENSMUSG00000026439	ENSMUSG00000026439	ENSMUSG00000026439	1	134304926	134332436	24	26
978	1	203318880	203320211	ENSMUSG00000048174	ENSMUSG00000048174	ENSMUSG00000048174	1	134333776	134336183	24	26
979	1	203278963	203313759	ENSMUSG00000053024	ENSMUSG00000053024	ENSMUSG00000053024	1	134336973	134370462	24	26
980	1	203064405	203258587	ENSMUSG00000026442	ENSMUSG00000026442	ENSMUSG00000026442	1	134396796	134534872	24	26
981	1	202852921	202921484	ENSMUSG00000026443	ENSMUSG00000026443	ENSMUSG00000026443	1	134707901	134767505	24	26
982	1	202658379	202726175	ENSMUSG00000026447	ENSMUSG00000026447	ENSMUSG00000026447	1	134873586	134933698	24	26
983	1	202639138	202647542	ENSMUSG00000046062	ENSMUSG00000046062	ENSMUSG00000046062	1	134958929	134967326	24	26
984	1	202454605	202595667	ENSMUSG00000041757	ENSMUSG00000041757	ENSMUSG00000041757	1	135073652	135129195	24	26
985	1	202433914	202449733	ENSMUSG00000041717	ENSMUSG00000041717	ENSMUSG00000041717	1	135137373	135157240	24	26
986	1	202390570	202402073	ENSMUSG00000070645	ENSMUSG00000070645	ENSMUSG00000070645	1	135178220	135187863	24	26
987	1	202366813	202387645	ENSMUSG00000070644	ENSMUSG00000070644	ENSMUSG00000070644	1	135191310	135202158	24	26
988	1	202348721	202362737	ENSMUSG00000070643	ENSMUSG00000070643	ENSMUSG00000070643	1	135210068	135220762	24	26
989	1	202031411	202089869	ENSMUSG00000026464	ENSMUSG00000026464	ENSMUSG00000026464	1	135449041	135482553	24	26
990	1	201862551	201979830	ENSMUSG00000026463	ENSMUSG00000026463	ENSMUSG00000026463	1	135523057	135569692	24	26
991	1	201729894	201744700	ENSMUSG00000010311	ENSMUSG00000010311	ENSMUSG00000010311	1	135713604	135723583	24	26
992	1	201711506	201727098	ENSMUSG00000041577	ENSMUSG00000041577	ENSMUSG00000041577	1	135726689	135737788	24	26
993	1	201576376	201584021	ENSMUSG00000041559	ENSMUSG00000041559	ENSMUSG00000041559	1	135853926	135864688	24	26
994	1	201541242	201545320	ENSMUSG00000020423	ENSMUSG00000020423	ENSMUSG00000020423	1	135891276	135895566	24	26
995	1	201451830	201465422	ENSMUSG00000026450	ENSMUSG00000026450	ENSMUSG00000026450	1	135955057	135967950	24	26
996	1	201414553	201422500	ENSMUSG00000064246	ENSMUSG00000064246	ENSMUSG00000064246	1	135998875	136006439	24	26
997	1	201403562	201411565	ENSMUSG00000042451	ENSMUSG00000042451	ENSMUSG00000042451	1	136009915	136016561	24	26
998	1	201326405	201403155	ENSMUSG00000042429	ENSMUSG00000042429	ENSMUSG00000042429	1	136015622	136051842	24	26
999	1	201318883	201321789	ENSMUSG00000026459	ENSMUSG00000026459	ENSMUSG00000026459	1	136106400	136108958	24	26
1000	1	201262249	201314487	ENSMUSG00000026458	ENSMUSG00000026458	ENSMUSG00000026458	1	136113195	136148892	24	26
1001	1	201222308	201224333	ENSMUSG00000050526	ENSMUSG00000050526	ENSMUSG00000050526	1	136202351	136207394	24	26
1002	1	201197620	201203031	ENSMUSG00000026456	ENSMUSG00000026456	ENSMUSG00000026456	1	136222192	136228136	24	26
1003	1	201176574	201194140	ENSMUSG00000026457	ENSMUSG00000026457	ENSMUSG00000026457	1	136231891	136248748	24	26
1004	1	201126851	201163013	ENSMUSG00000026455	ENSMUSG00000026455	ENSMUSG00000026455	1	136271942	136307429	24	26
1005	1	201114708	201124886	ENSMUSG00000042229	ENSMUSG00000042229	ENSMUSG00000042229	1	136311071	136324293	24	26
1006	1	200963156	201044466	ENSMUSG00000042207	ENSMUSG00000042207	ENSMUSG00000042207	1	136376606	136448030	24	26
1007	1	200826347	200946168	ENSMUSG00000026452	ENSMUSG00000026452	ENSMUSG00000026452	1	136525717	136565477	24	26
1008	1	200584459	200824320	ENSMUSG00000073557	ENSMUSG00000073557	ENSMUSG00000073557	1	136692379	136772339	24	26
1009	1	200567408	200577731	ENSMUSG00000026429	ENSMUSG00000026429	ENSMUSG00000026429	1	136778988	136790548	24	26
1010	1	200382764	200397332	ENSMUSG00000031506	ENSMUSG00000031506	ENSMUSG00000031506	1	136949136	136961731	24	26
1011	1	200368600	200380489	ENSMUSG00000026426	ENSMUSG00000026426	ENSMUSG00000026426	1	136963245	136972679	24	26
1012	1	200358609	200365263	ENSMUSG00000026424	ENSMUSG00000026424	ENSMUSG00000026424	1	136976661	136983915	24	26
1013	1	200243696	200251760	ENSMUSG00000003051	ENSMUSG00000003051	ENSMUSG00000003051	1	137069990	137074849	24	26
1014	1	200218375	200241898	ENSMUSG00000041926	ENSMUSG00000041926	ENSMUSG00000041926	1	137079120	137100445	24	26
1015	1	200191242	200206412	ENSMUSG00000062580	ENSMUSG00000062580	ENSMUSG00000062580	1	137117946	137130148	24	26
1016	1	200132203	200182338	ENSMUSG00000048096	ENSMUSG00000048096	ENSMUSG00000048096	1	137141421	137181583	24	26
1017	1	200124459	200128057	ENSMUSG00000041889	ENSMUSG00000041889	ENSMUSG00000041889	1	137187868	137191484	24	26
1018	1	200064892	200120045	ENSMUSG00000041879	ENSMUSG00000041879	ENSMUSG00000041879	1	137200893	137246902	24	26
1019	1	199884000	200062720	ENSMUSG00000009418	ENSMUSG00000009418	ENSMUSG00000009418	1	137255259	137423706	24	26
1020	1	199719281	199745207	ENSMUSG00000026421	ENSMUSG00000026421	ENSMUSG00000026421	1	137536472	137567864	24	26
1021	1	199701243	199704935	ENSMUSG00000041801	ENSMUSG00000041801	ENSMUSG00000041801	1	137582530	137585544	24	26
1022	1	199639519	199657497	ENSMUSG00000026418	ENSMUSG00000026418	ENSMUSG00000026418	1	137615850	137627398	24	26
1023	1	199616958	199635299	ENSMUSG00000041782	ENSMUSG00000041782	ENSMUSG00000041782	1	137635390	137648362	24	26
1024	1	199594765	199609005	ENSMUSG00000026414	ENSMUSG00000026414	ENSMUSG00000026414	1	137657286	137668671	24	26
1025	1	199519203	199568738	ENSMUSG00000026413	ENSMUSG00000026413	ENSMUSG00000026413	1	137687806	137735435	24	26
1026	1	199448393	199464693	ENSMUSG00000051985	ENSMUSG00000051985	ENSMUSG00000051985	1	137770985	137779660	24	26
1027	1	199370524	199407325	ENSMUSG00000026411	ENSMUSG00000026411	ENSMUSG00000026411	1	137834426	137851437	24	26
1028	1	199275265	199348317	ENSMUSG00000026407	ENSMUSG00000026407	ENSMUSG00000026407	1	137869195	137935939	24	26
1029	1	199205141	199259435	ENSMUSG00000041642	ENSMUSG00000041642	ENSMUSG00000041642	1	137947812	137994425	24	26
1030	1	199127092	199151486	ENSMUSG00000041605	ENSMUSG00000041605	ENSMUSG00000041605	1	138029942	138050675	24	26
1031	1	199108789	199109874	ENSMUSG00000052759	ENSMUSG00000052759	ENSMUSG00000052759	1	138074415	138077152	24	26
1032	1	198975309	199096452	ENSMUSG00000041570	ENSMUSG00000041570	ENSMUSG00000041570	1	138093601	138162515	24	26
1033	1	198879820	198905720	ENSMUSG00000026404	ENSMUSG00000026404	ENSMUSG00000026404	1	138231682	138256562	24	26
1034	1	198787248	198854474	ENSMUSG00000041498	ENSMUSG00000041498	ENSMUSG00000041498	1	138285245	138344024	24	26
1035	1	198642053	198645789	ENSMUSG00000041483	ENSMUSG00000041483	ENSMUSG00000041483	1	138441356	138444716	24	26
1036	1	198263353	198413175	ENSMUSG00000026398	ENSMUSG00000026398	ENSMUSG00000026398	1	138660687	138776859	24	26
1037	1	196874793	196993035	ENSMUSG00000026395	ENSMUSG00000026395	ENSMUSG00000026395	1	139879826	139991666	24	26
1038	1	196758979	196776698	ENSMUSG00000026394	ENSMUSG00000026394	ENSMUSG00000026394	1	140090149	140105873	24	26
1039	1	196392744	196558173	ENSMUSG00000026393	ENSMUSG00000026393	ENSMUSG00000026393	1	140301125	140436107	24	26
1040	1	196153140	196165896	ENSMUSG00000019230	ENSMUSG00000019230	ENSMUSG00000019230	1	140647778	140663614	24	26
1041	1	195788008	196011078	ENSMUSG00000056268	ENSMUSG00000056268	ENSMUSG00000056268	1	140780197	140986709	24	26
1042	1	195504031	195714208	ENSMUSG00000063681	ENSMUSG00000063681	ENSMUSG00000063681	1	141014754	141193452	24	26
1043	1	195389437	195436295	ENSMUSG00000033964	ENSMUSG00000033964	ENSMUSG00000033964	1	141239562	141263938	24	26
1044	1	195320077	195382190	ENSMUSG00000033952	ENSMUSG00000033952	ENSMUSG00000033952	1	141271184	141310501	24	26
1045	1	195274944	195303020	ENSMUSG00000026368	ENSMUSG00000026368	ENSMUSG00000026368	1	141318138	141340167	24	26
1046	1	194461536	194844122	ENSMUSG00000052726	ENSMUSG00000052726	ENSMUSG00000052726	1	142266604	142339672	24	26
1047	1	191357770	191489654	ENSMUSG00000026361	ENSMUSG00000026361	ENSMUSG00000026361	1	145369717	145464902	24	26
1048	1	191414798	191422407	ENSMUSG00000033849	ENSMUSG00000033849	ENSMUSG00000033849	1	145402961	145412048	24	26
1049	1	191332221	191341867	ENSMUSG00000018196	ENSMUSG00000018196	ENSMUSG00000018196	1	145501835	145511896	24	26
1050	1	191295787	191321738	ENSMUSG00000018199	ENSMUSG00000018199	ENSMUSG00000018199	1	145517790	145539196	24	26
1051	1	191251523	191295144	ENSMUSG00000018189	ENSMUSG00000018189	ENSMUSG00000018189	1	145539530	145569021	24	26
1052	1	191044794	191048029	ENSMUSG00000026360	ENSMUSG00000026360	ENSMUSG00000026360	1	145761556	145766367	24	26
1053	1	190871920	190896059	ENSMUSG00000051079	ENSMUSG00000051079	ENSMUSG00000051079	1	145900885	145939590	24	26
1054	1	190811480	190815784	ENSMUSG00000026358	ENSMUSG00000026358	ENSMUSG00000026358	1	146006813	146011277	24	26
1055	1	190394210	190421568	ENSMUSG00000026357	ENSMUSG00000026357	ENSMUSG00000026357	1	146515059	146537645	24	26
1056	1	188333415	188713382	ENSMUSG00000035131	ENSMUSG00000035131	ENSMUSG00000035131	1	148257884	148664687	24	26
1057	1	185064708	185224736	ENSMUSG00000056220	ENSMUSG00000056220	ENSMUSG00000056220	1	151591836	151723502	24	26
1058	1	184907546	184916178	ENSMUSG00000032487	ENSMUSG00000032487	ENSMUSG00000032487	1	151862342	151870203	24	26
1059	1	184679321	184696877	ENSMUSG00000006007	ENSMUSG00000006007	ENSMUSG00000006007	1	152081635	152096124	24	26
1060	1	184611624	184657122	ENSMUSG00000006010	ENSMUSG00000006010	ENSMUSG00000006010	1	152125038	152152554	24	26
1061	1	184549585	184610871	ENSMUSG00000006005	ENSMUSG00000006005	ENSMUSG00000006005	1	152155517	152212005	24	26
1062	1	184532601	184550317	ENSMUSG00000006014	ENSMUSG00000006014	ENSMUSG00000006014	1	152212087	152228383	24	26
1063	1	183532143	183553084	ENSMUSG00000023150	ENSMUSG00000023150	ENSMUSG00000023150	1	153106716	153126663	24	26
1064	1	183392835	183526762	ENSMUSG00000052748	ENSMUSG00000052748	ENSMUSG00000052748	1	153129917	153190599	24	26
1065	1	183353853	183392736	ENSMUSG00000053286	ENSMUSG00000053286	ENSMUSG00000053286	1	153190866	153220377	24	26
1066	1	183281119	183338363	ENSMUSG00000026484	ENSMUSG00000026484	ENSMUSG00000026484	1	153231624	153263041	24	26
1067	1	183026787	183210305	ENSMUSG00000026483	ENSMUSG00000026483	ENSMUSG00000026483	1	153333693	153481560	24	26
1068	1	182925988	182990670	ENSMUSG00000043019	ENSMUSG00000043019	ENSMUSG00000043019	1	153544681	153584274	24	26
1069	1	182622773	182864778	ENSMUSG00000032666	ENSMUSG00000032666	ENSMUSG00000032666	1	153677700	153768856	24	26
1070	1	182171618	182273486	ENSMUSG00000032649	ENSMUSG00000032649	ENSMUSG00000032649	1	154162139	154272913	24	26
1071	1	181871843	182164288	ENSMUSG00000026482	ENSMUSG00000026482	ENSMUSG00000026482	1	154279746	154387329	24	26
1072	1	181882164	181889071	ENSMUSG00000055547	ENSMUSG00000055547	ENSMUSG00000055547	1	154512795	154519762	24	26
1073	1	181791321	181826634	ENSMUSG00000026480	ENSMUSG00000026480	ENSMUSG00000026480	1	154562459	154598471	24	26
1074	1	181708261	181789944	ENSMUSG00000042772	ENSMUSG00000042772	ENSMUSG00000042772	1	154599214	154640386	24	26
1075	1	181483995	181654360	ENSMUSG00000042751	ENSMUSG00000042751	ENSMUSG00000042751	1	154717319	154881479	24	26
1076	1	181422022	181480662	ENSMUSG00000026479	ENSMUSG00000026479	ENSMUSG00000026479	1	154886248	154948205	24	26
1077	1	181259176	181381350	ENSMUSG00000026478	ENSMUSG00000026478	ENSMUSG00000026478	1	154981138	155095004	24	26
1078	1	181135624	181189108	ENSMUSG00000042708	ENSMUSG00000042708	ENSMUSG00000042708	1	155187427	155214792	24	26
1079	1	181075164	181123510	ENSMUSG00000042699	ENSMUSG00000042699	ENSMUSG00000042699	1	155218031	155249826	24	26
1080	1	181028017	181065890	ENSMUSG00000042684	ENSMUSG00000042684	ENSMUSG00000042684	1	155265591	155307548	24	26
1081	1	180882415	180908672	ENSMUSG00000042671	ENSMUSG00000042671	ENSMUSG00000042671	1	155415255	155459883	24	26
1082	1	180834381	180840166	ENSMUSG00000026475	ENSMUSG00000026475	ENSMUSG00000026475	1	155502566	155507686	24	26
1083	1	180809392	180825014	ENSMUSG00000066800	ENSMUSG00000066800	ENSMUSG00000066800	1	155515148	155524559	24	26
1084	1	180685915	180724506	ENSMUSG00000042634	ENSMUSG00000042634	ENSMUSG00000042634	1	155587491	155589824	24	26
1085	1	180617462	180627964	ENSMUSG00000026473	ENSMUSG00000026473	ENSMUSG00000026473	1	155662163	155671932	24	26
1086	1	179648918	180037339	ENSMUSG00000004110	ENSMUSG00000004110	ENSMUSG00000004110	1	156158170	156488137	24	26
1087	1	179324261	179326595	ENSMUSG00000056708	ENSMUSG00000056708	ENSMUSG00000056708	1	156860722	156861648	24	26
1088	1	179269762	179292312	ENSMUSG00000026471	ENSMUSG00000026471	ENSMUSG00000026471	1	156890096	156909001	24	26
1089	1	179208484	179258670	ENSMUSG00000026470	ENSMUSG00000026470	ENSMUSG00000026470	1	156920960	156965735	24	26
1090	1	179148948	179187373	ENSMUSG00000033722	ENSMUSG00000033722	ENSMUSG00000033722	1	156974691	156994958	24	26
1091	1	178867763	179126010	ENSMUSG00000026469	ENSMUSG00000026469	ENSMUSG00000026469	1	157037875	157179662	24	26
1092	1	178523988	178738712	ENSMUSG00000033701	ENSMUSG00000033701	ENSMUSG00000033701	1	157320340	157449450	24	26
1093	1	178466065	178510811	ENSMUSG00000026468	ENSMUSG00000026468	ENSMUSG00000026468	1	157463915	157504245	24	26
1094	1	178390592	178439788	ENSMUSG00000033684	ENSMUSG00000033684	ENSMUSG00000033684	1	157540373	157575117	24	26
1095	1	178190496	178350638	ENSMUSG00000033671	ENSMUSG00000033671	ENSMUSG00000033671	1	157625471	157686945	24	26
1096	1	178118000	178155834	ENSMUSG00000026466	ENSMUSG00000026466	ENSMUSG00000026466	1	157768999	157798513	24	26
1097	1	178075725	178113557	ENSMUSG00000050565	ENSMUSG00000050565	ENSMUSG00000050565	1	157797621	157827856	24	26
1098	1	177978921	178051956	ENSMUSG00000015484	ENSMUSG00000015484	ENSMUSG00000015484	1	157838184	158041702	24	26
1099	1	177827371	177927028	ENSMUSG00000060985	ENSMUSG00000060985	ENSMUSG00000060985	1	158096408	158122274	24	26
1100	1	177786297	177811691	ENSMUSG00000026602	ENSMUSG00000026602	ENSMUSG00000026602	1	158147412	158164712	24	26
1101	1	177601478	177790493	ENSMUSG00000026601	ENSMUSG00000026601	ENSMUSG00000026601	1	158201606	158236667	24	26
1102	1	177529640	177591075	ENSMUSG00000026600	ENSMUSG00000026600	ENSMUSG00000026600	1	158265149	158310920	24	26
1103	1	177343380	177465359	ENSMUSG00000026596	ENSMUSG00000026596	ENSMUSG00000026596	1	158395726	158479393	24	26
1104	1	177317135	177331754	ENSMUSG00000060519	ENSMUSG00000060519	ENSMUSG00000060519	1	158490297	158511002	24	26
1105	1	177261654	177312320	ENSMUSG00000033557	ENSMUSG00000033557	ENSMUSG00000033557	1	158517710	158555678	24	26
1106	1	176960923	177155861	ENSMUSG00000026594	ENSMUSG00000026594	ENSMUSG00000026594	1	158644799	158776257	24	26
1107	1	177085294	177106838	ENSMUSG00000033544	ENSMUSG00000033544	ENSMUSG00000033544	1	158675700	158697755	24	26
1108	1	176748833	176758407	ENSMUSG00000026592	ENSMUSG00000026592	ENSMUSG00000026592	1	158935825	158945322	24	26
1109	1	176329566	176709805	ENSMUSG00000070565	ENSMUSG00000070565	ENSMUSG00000070565	1	158977391	159248561	24	26
1110	1	176164546	176258302	ENSMUSG00000026589	ENSMUSG00000026589	ENSMUSG00000026589	1	159343487	159405102	24	26
1111	1	175465612	175517573	ENSMUSG00000004031	ENSMUSG00000004031	ENSMUSG00000004031	1	160081948	160193003	24	26
1112	1	175096827	175400647	ENSMUSG00000026587	ENSMUSG00000026587	ENSMUSG00000026587	1	160199033	160528465	24	26
1113	1	174698930	175081358	ENSMUSG00000026732	ENSMUSG00000026732	ENSMUSG00000026732	1	160675221	160793626	24	26
1114	1	173558558	173979529	ENSMUSG00000015829	ENSMUSG00000015829	ENSMUSG00000015829	1	161574187	161761152	24	26
1115	1	173303617	173383825	ENSMUSG00000026725	ENSMUSG00000026725	ENSMUSG00000026725	1	161921713	161990219	24	26
1116	1	173248717	173259184	ENSMUSG00000058267	ENSMUSG00000058267	ENSMUSG00000058267	1	162031937	162037863	24	26
1117	1	172395278	173193942	ENSMUSG00000026721	ENSMUSG00000026721	ENSMUSG00000026721	1	162093882	162629615	24	26
1118	1	172172540	172228825	ENSMUSG00000040423	ENSMUSG00000040423	ENSMUSG00000040423	1	162743142	162804772	24	26
1119	1	172139570	172153139	ENSMUSG00000026715	ENSMUSG00000026715	ENSMUSG00000026715	1	162815323	162839219	24	26
1120	1	172104109	172133117	ENSMUSG00000043467	ENSMUSG00000043467	ENSMUSG00000043467	1	162845477	162871512	24	26
1121	1	172060264	172094307	ENSMUSG00000026709	ENSMUSG00000026709	ENSMUSG00000026709	1	162877278	162907296	24	26
1122	1	172035311	172060083	ENSMUSG00000026708	ENSMUSG00000026708	ENSMUSG00000026708	1	162907618	162923003	24	26
1123	1	171950703	172022446	ENSMUSG00000026705	ENSMUSG00000026705	ENSMUSG00000026705	1	162927104	162946477	24	26
1124	1	171844098	171905624	ENSMUSG00000044835	ENSMUSG00000044835	ENSMUSG00000044835	1	162987670	163000099	24	26
1125	1	171736226	171838856	ENSMUSG00000026702	ENSMUSG00000026702	ENSMUSG00000026702	1	163031455	163053566	24	26
1126	1	171419494	171443094	ENSMUSG00000026700	ENSMUSG00000026700	ENSMUSG00000026700	1	163232129	163254883	24	26
1127	1	171275723	171286679	ENSMUSG00000066755	ENSMUSG00000066755	ENSMUSG00000066755	1	163331332	163343011	24	26
1128	1	170894777	170902637	ENSMUSG00000000817	ENSMUSG00000000817	ENSMUSG00000000817	1	163617366	163625172	24	26
1129	1	170768112	170847594	ENSMUSG00000040297	ENSMUSG00000040297	ENSMUSG00000040297	1	163655046	163713322	24	26
1130	1	170077261	170648479	ENSMUSG00000040265	ENSMUSG00000040265	ENSMUSG00000040265	1	163823979	164314705	24	26
1131	1	170017384	170033475	ENSMUSG00000026694	ENSMUSG00000026694	ENSMUSG00000026694	1	164368802	164385173	24	26
1132	1	169935923	169977823	ENSMUSG00000026696	ENSMUSG00000026696	ENSMUSG00000026696	1	164407398	164432040	24	26
1133	1	169871180	169888434	ENSMUSG00000026697	ENSMUSG00000026697	ENSMUSG00000026697	1	164475844	164486368	24	26
1134	1	169721290	169829273	ENSMUSG00000040225	ENSMUSG00000040225	ENSMUSG00000040225	1	164508469	164543004	24	26
1135	1	169549997	169577847	ENSMUSG00000026692	ENSMUSG00000026692	ENSMUSG00000026692	1	164630558	164649224	24	26
1136	1	169484262	169521741	ENSMUSG00000040181	ENSMUSG00000040181	ENSMUSG00000040181	1	164666232	164703223	24	26
1137	1	169420971	169448171	ENSMUSG00000040170	ENSMUSG00000040170	ENSMUSG00000040170	1	164711722	164735387	24	26
1138	1	169326642	169353583	ENSMUSG00000026691	ENSMUSG00000026691	ENSMUSG00000026691	1	164790475	164821166	24	26
1139	1	169145750	169300042	ENSMUSG00000071890	ENSMUSG00000071890	ENSMUSG00000071890	1	164861118	164916202	24	26
1140	1	168899937	168975165	ENSMUSG00000026586	ENSMUSG00000026586	ENSMUSG00000026586	1	165081791	165150325	24	26
1141	1	168767894	168789211	ENSMUSG00000040124	ENSMUSG00000040124	ENSMUSG00000040124	1	165221584	165240316	24	26
1142	1	168381873	168403501	ENSMUSG00000040113	ENSMUSG00000040113	ENSMUSG00000040113	1	165539710	165553916	24	26
1143	1	168157094	168310503	ENSMUSG00000026585	ENSMUSG00000026585	ENSMUSG00000026585	1	165616268	165753774	24	26
1144	1	168088839	168124653	ENSMUSG00000026584	ENSMUSG00000026584	ENSMUSG00000026584	1	165766465	165791785	24	26
1145	1	168030851	168088851	ENSMUSG00000041406	ENSMUSG00000041406	ENSMUSG00000041406	1	165783025	165831395	24	26
1146	1	168028294	168030731	ENSMUSG00000041396	ENSMUSG00000041396	ENSMUSG00000041396	1	165831588	165833916	24	26
1147	1	167958406	167969827	ENSMUSG00000026582	ENSMUSG00000026582	ENSMUSG00000026582	1	165884909	165894352	24	26
1148	1	167926432	167947463	ENSMUSG00000026581	ENSMUSG00000026581	ENSMUSG00000026581	1	165899728	165909011	24	26
1149	1	167824714	167866023	ENSMUSG00000026580	ENSMUSG00000026580	ENSMUSG00000026580	1	165960377	165985205	24	26
1150	1	167750033	167822345	ENSMUSG00000026579	ENSMUSG00000026579	ENSMUSG00000026579	1	165988633	166056227	24	26
1151	1	167699089	167721638	ENSMUSG00000040918	ENSMUSG00000040918	ENSMUSG00000040918	1	166085721	166102060	24	26
1152	1	167630732	167663279	ENSMUSG00000026578	ENSMUSG00000026578	ENSMUSG00000026578	1	166112273	166124517	24	26
1153	1	167603818	167632402	ENSMUSG00000026577	ENSMUSG00000026577	ENSMUSG00000026577	1	166126475	166144159	24	26
1154	1	167368393	167603829	ENSMUSG00000026575	ENSMUSG00000026575	ENSMUSG00000026575	1	166149554	166239755	24	26
1155	1	167342185	167367608	ENSMUSG00000026576	ENSMUSG00000026576	ENSMUSG00000026576	1	166273942	166295030	24	26
1156	1	166931321	166965126	ENSMUSG00000026574	ENSMUSG00000026574	ENSMUSG00000026574	1	166633389	166660944	24	26
1157	1	166516902	166550288	ENSMUSG00000026572	ENSMUSG00000026572	ENSMUSG00000026572	1	166974528	166997448	24	26
1158	1	166414817	166437949	ENSMUSG00000040843	ENSMUSG00000040843	ENSMUSG00000040843	1	167048961	167073618	24	26
1159	1	166320621	166372248	ENSMUSG00000040836	ENSMUSG00000040836	ENSMUSG00000040836	1	167142897	167147667	24	26
1160	1	166172680	166311696	ENSMUSG00000026571	ENSMUSG00000026571	ENSMUSG00000026571	1	167166365	167296962	24	26
1161	1	166152591	166171902	ENSMUSG00000026568	ENSMUSG00000026568	ENSMUSG00000026568	1	167297883	167317889	24	26
1162	1	166045507	166141002	ENSMUSG00000026567	ENSMUSG00000026567	ENSMUSG00000026567	1	167339845	167413448	24	26
1163	1	165957832	166026682	ENSMUSG00000026566	ENSMUSG00000026566	ENSMUSG00000026566	1	167441281	167471068	24	26
1164	1	165865954	165942109	ENSMUSG00000040723	ENSMUSG00000040723	ENSMUSG00000040723	1	167486853	167501728	24	26
1165	1	165776874	165789628	ENSMUSG00000040713	ENSMUSG00000040713	ENSMUSG00000040713	1	167600460	167607495	24	26
1166	1	165670755	165676632	ENSMUSG00000005763	ENSMUSG00000005763	ENSMUSG00000005763	1	167625398	167704109	24	26
1167	1	165564931	165663206	ENSMUSG00000026565	ENSMUSG00000026565	ENSMUSG00000026565	1	167712151	167839309	24	26

1168	1	165329906	165364990	ENSMUSG00000026564	ENSMUSG00000026564	ENSMUSG00000026564	1	167934828	167964573	24	26
1169	1	165288697	165326492	ENSMUSG00000000544	ENSMUSG00000000544	ENSMUSG00000000544	1	167967135	168003185	24	26
1170	1	165224970	165258075	ENSMUSG00000040629	ENSMUSG00000040629	ENSMUSG00000040629	1	168038047	168075415	24	26
1171	1	165154620	165211185	ENSMUSG00000040612	ENSMUSG00000040612	ENSMUSG00000040612	1	168105967	168131411	24	26
1172	1	165092371	165112188	ENSMUSG00000026563	ENSMUSG00000026563	ENSMUSG00000026563	1	168215975	168230296	24	26
1173	1	165075318	165092205	ENSMUSG00000040596	ENSMUSG00000040596	ENSMUSG00000040596	1	168221300	168246457	24	26
1174	1	164840618	164867261	ENSMUSG00000026560	ENSMUSG00000026560	ENSMUSG00000026560	1	168498730	168518520	24	26
1175	1	164293287	164402576	ENSMUSG00000060568	ENSMUSG00000060568	ENSMUSG00000060568	1	168838150	168927688	24	26
1176	1	164063392	164143970	ENSMUSG00000026558	ENSMUSG00000026558	ENSMUSG00000026558	1	169062754	169121802	24	26
1177	1	163962656	164005041	ENSMUSG00000052428	ENSMUSG00000052428	ENSMUSG00000052428	1	169145347	169170653	24	26
1178	1	163898077	163934724	ENSMUSG00000026687	ENSMUSG00000026687	ENSMUSG00000026687	1	169186888	169204961	24	26
1179	1	163867101	163891481	ENSMUSG00000026688	ENSMUSG00000026688	ENSMUSG00000026688	1	169209059	169230472	24	26
1180	1	163779868	163799822	ENSMUSG00000040485	ENSMUSG00000040485	ENSMUSG00000040485	1	169282350	169303455	24	26
1181	1	163636778	163681057	ENSMUSG00000015843	ENSMUSG00000015843	ENSMUSG00000015843	1	169435059	169476298	24	26
1182	1	163437729	163592576	ENSMUSG00000026686	ENSMUSG00000026686	ENSMUSG00000026686	1	169526233	169685408	24	26
1183	1	162795496	163087691	ENSMUSG00000052534	ENSMUSG00000052534	ENSMUSG00000052534	1	169995407	170268179	24	26
1184	1	161558349	161592178	ENSMUSG00000026683	ENSMUSG00000026683	ENSMUSG00000026683	1	171334986	171368103	24	26
1185	1	161383470	161454050	ENSMUSG00000026678	ENSMUSG00000026678	ENSMUSG00000026678	1	171492176	171532488	24	26
1186	1	161305673	161313004	ENSMUSG00000038530	ENSMUSG00000038530	ENSMUSG00000038530	1	171578985	171584317	24	26
1187	1	161090711	161105175	ENSMUSG00000070532	ENSMUSG00000070532	ENSMUSG00000070532	1	171765614	171771328	24	26
1188	1	160868852	161016861	ENSMUSG00000026674	ENSMUSG00000026674	ENSMUSG00000026674	1	171814321	171947236	24	26
1189	1	160797947	160836250	ENSMUSG00000026670	ENSMUSG00000026670	ENSMUSG00000026670	1	171978678	172011621	24	26
1190	1	160734257	160766024	ENSMUSG00000026667	ENSMUSG00000026667	ENSMUSG00000026667	1	172035931	172052068	24	26
1191	1	160610139	160613284	ENSMUSG00000042800	ENSMUSG00000042800	ENSMUSG00000042800	1	172145478	172148800	24	26
1192	1	160306190	160604868	ENSMUSG00000038473	ENSMUSG00000038473	ENSMUSG00000038473	1	172155147	172426524	24	26
1193	1	160219607	160260268	ENSMUSG00000038463	ENSMUSG00000038463	ENSMUSG00000038463	1	172481505	172519464	24	26
1194	1	160002708	160200484	ENSMUSG00000026663	ENSMUSG00000026663	ENSMUSG00000026663	1	172543889	172704443	24	26
1195	1	159986184	159993576	ENSMUSG00000026659	ENSMUSG00000026659	ENSMUSG00000026659	1	172710863	172722215	24	26
1196	1	159957958	159964556	ENSMUSG00000070524	ENSMUSG00000070524	ENSMUSG00000070524	1	172743948	172749616	24	26
1197	1	159943386	159950765	ENSMUSG00000038421	ENSMUSG00000038421	ENSMUSG00000038421	1	172754576	172764258	24	26
1198	1	159601145	159604288	ENSMUSG00000026649	ENSMUSG00000026649	ENSMUSG00000026649	1	172958336	172963625	24	26
1199	1	159541149	159546368	ENSMUSG00000056569	ENSMUSG00000056569	ENSMUSG00000056569	1	172987388	172997798	24	26
1200	1	159495141	159521863	ENSMUSG00000038370	ENSMUSG00000038370	ENSMUSG00000038370	1	173011058	173012308	24	26
1201	1	159466193	159473005	ENSMUSG00000005677	ENSMUSG00000005677	ENSMUSG00000005677	1	173050645	173055519	24	26
1202	1	159462417	159467021	ENSMUSG00000005674	ENSMUSG00000005674	ENSMUSG00000005674	1	173054478	173059189	24	26
1203	1	159458706	159460042	ENSMUSG00000005681	ENSMUSG00000005681	ENSMUSG00000005681	1	173061764	173063045	24	26
1204	1	159451693	159457113	ENSMUSG00000058715	ENSMUSG00000058715	ENSMUSG00000058715	1	173066249	173070964	24	26
1205	1	159435729	159450809	ENSMUSG00000013593	ENSMUSG00000013593	ENSMUSG00000013593	1	173071535	173083787	24	26
1206	1	159426162	159435470	ENSMUSG00000006403	ENSMUSG00000006403	ENSMUSG00000006403	1	173087096	173097312	24	26
1207	1	159407724	159413899	ENSMUSG00000052423	ENSMUSG00000052423	ENSMUSG00000052423	1	173107063	173113558	24	26
1208	1	159402818	159407634	ENSMUSG00000062729	ENSMUSG00000062729	ENSMUSG00000062729	1	173113667	173117835	24	26
1209	1	159395893	159402137	ENSMUSG00000053483	ENSMUSG00000053483	ENSMUSG00000053483	1	173118633	173124636	24	26
1210	1	159390166	159395270	ENSMUSG00000062963	ENSMUSG00000062963	ENSMUSG00000062963	1	173125239	173131657	24	26
1211	1	159358439	159367251	ENSMUSG00000013973	ENSMUSG00000013973	ENSMUSG00000013973	1	173167928	173178291	24	26
1212	1	159354496	159357478	ENSMUSG00000013997	ENSMUSG00000013997	ENSMUSG00000013997	1	173177253	173182281	24	26
1213	1	159334778	159336758	ENSMUSG00000045259	ENSMUSG00000045259	ENSMUSG00000045259	1	173195124	173197473	24	26
1214	1	159307409	159326013	ENSMUSG00000006411	ENSMUSG00000006411	ENSMUSG00000006411	1	173207073	173225256	24	26
1215	1	159284129	159306384	ENSMUSG00000048865	ENSMUSG00000048865	ENSMUSG00000048865	1	173225687	173246917	24	26
1216	1	159275665	159282391	ENSMUSG00000026641	ENSMUSG00000026641	ENSMUSG00000026641	1	173248059	173254796	24	26
1217	1	159231625	159257592	ENSMUSG00000038235	ENSMUSG00000038235	ENSMUSG00000038235	1	173274236	173301268	24	26
1218	1	159067597	159099174	ENSMUSG00000004709	ENSMUSG00000004709	ENSMUSG00000004709	1	173395868	173420260	24	26
1219	1	159032572	159064669	ENSMUSG00000004707	ENSMUSG00000004707	ENSMUSG00000004707	1	173425307	173444008	24	26
1220	1	158975679	158991235	ENSMUSG00000038179	ENSMUSG00000038179	ENSMUSG00000038179	1	173469078	173485678	24	26
1221	1	158915160	158948265	ENSMUSG00000015355	ENSMUSG00000015355	ENSMUSG00000015355	1	173518702	173541932	24	26
1222	1	158844514	158883709	ENSMUSG00000015316	ENSMUSG00000015316	ENSMUSG00000015316	1	173603806	173637851	24	26
1223	1	158784659	158815918	ENSMUSG00000038147	ENSMUSG00000038147	ENSMUSG00000038147	1	173677279	173723105	24	26
1224	1	158721453	158759657	ENSMUSG00000015314	ENSMUSG00000015314	ENSMUSG00000015314	1	173754190	173785627	24	26
1225	1	158636988	158665088	ENSMUSG00000026556	ENSMUSG00000026556	ENSMUSG00000026556	1	173839265	173865119	24	26
1226	1	158603481	158609262	ENSMUSG00000051251	ENSMUSG00000051251	ENSMUSG00000051251	1	173888970	173894246	24	26
1227	1	158579678	158595362	ENSMUSG00000003458	ENSMUSG00000003458	ENSMUSG00000003458	1	173902696	173919424	24	26
1228	1	158525687	158579662	ENSMUSG00000026553	ENSMUSG00000026553	ENSMUSG00000026553	1	173919478	173958999	24	26
1229	1	158513223	158521560	ENSMUSG00000003464	ENSMUSG00000003464	ENSMUSG00000003464	1	173963447	173972455	24	26
1230	1	158452129	158498915	ENSMUSG00000026554	ENSMUSG00000026554	ENSMUSG00000026554	1	173984791	174032141	24	26
1231	1	158426970	158438300	ENSMUSG00000007122	ENSMUSG00000007122	ENSMUSG00000007122	1	174046576	174056508	24	26
1232	1	158387984	158423391	ENSMUSG00000007107	ENSMUSG00000007107	ENSMUSG00000007107	1	174060473	174094709	24	26
1233	1	158352172	158379996	ENSMUSG00000007097	ENSMUSG00000007097	ENSMUSG00000007097	1	174111159	174134652	24	26
1234	1	158327754	158335103	ENSMUSG00000038034	ENSMUSG00000038034	ENSMUSG00000038034	1	174149082	174156512	24	26
1235	1	158317984	158326977	ENSMUSG00000038026	ENSMUSG00000038026	ENSMUSG00000038026	1	174157708	174165938	24	26
1236	1	158273881	158306662	ENSMUSG00000044708	ENSMUSG00000044708	ENSMUSG00000044708	1	174205596	174206735	24	26
1237	1	158261367	158268405	ENSMUSG00000050229	ENSMUSG00000050229	ENSMUSG00000050229	1	174213231	174217208	24	26
1238	1	158187906	158190668	ENSMUSG00000026548	ENSMUSG00000026548	ENSMUSG00000026548	1	174312049	174315250	24	26
1239	1	158163453	158182010	ENSMUSG00000037995	ENSMUSG00000037995	ENSMUSG00000037995	1	174318895	174335552	24	26
1240	1	158108778	158136577	ENSMUSG00000026546	ENSMUSG00000026546	ENSMUSG00000026546	1	174364465	174382428	24	26
1241	1	158090730	158099071	ENSMUSG00000049598	ENSMUSG00000049598	ENSMUSG00000049598	1	174392613	174400391	24	26
1242	1	158063225	158073903	ENSMUSG00000053318	ENSMUSG00000053318	ENSMUSG00000053318	1	174418433	174427163	24	26
1243	1	158038838	158052075	ENSMUSG00000070504	ENSMUSG00000070504	ENSMUSG00000070504	1	174434912	174439135	24	26
1244	1	158017346	158018957	ENSMUSG00000026544	ENSMUSG00000026544	ENSMUSG00000026544	1	174468045	174469637	24	26
1245	1	157948703	157951003	ENSMUSG00000037942	ENSMUSG00000037942	ENSMUSG00000037942	1	174534731	174535702	24	26
1246	1	157824240	157825284	ENSMUSG00000026542	ENSMUSG00000026542	ENSMUSG00000026542	1	174730637	174731718	24	26
1247	1	157771492	157772421	ENSMUSG00000037924	ENSMUSG00000037924	ENSMUSG00000037924	1	174793472	174794401	24	26
1248	1	157526128	157544638	ENSMUSG00000005339	ENSMUSG00000005339	ENSMUSG00000005339	1	175057959	175063889	24	26
1249	1	157515458	157516380	ENSMUSG00000049605	ENSMUSG00000049605	ENSMUSG00000049605	1	175106852	175107784	24	26
1250	1	157408023	157438426	ENSMUSG00000005338	ENSMUSG00000005338	ENSMUSG00000005338	1	175173422	175204325	24	26
1251	1	157044813	157045749	ENSMUSG00000045381	ENSMUSG00000045381	ENSMUSG00000045381	1	175878627	175879571	24	26
1252	1	157002158	157003096	ENSMUSG00000049528	ENSMUSG00000049528	ENSMUSG00000049528	1	175925717	175926655	24	26
1253	1	156991230	156992261	ENSMUSG00000046486	ENSMUSG00000046486	ENSMUSG00000046486	1	175953766	175954722	24	26
1254	1	156960542	156961450	ENSMUSG00000051528	ENSMUSG00000051528	ENSMUSG00000051528	1	175973421	175974368	24	26
1255	1	156936092	156937066	ENSMUSG00000055033	ENSMUSG00000055033	ENSMUSG00000055033	1	175995450	175996415	24	26
1256	1	156847644	156923112	ENSMUSG00000026532	ENSMUSG00000026532	ENSMUSG00000026532	1	176011002	176084132	24	26
1257	1	156842853	156843794	ENSMUSG00000050788	ENSMUSG00000050788	ENSMUSG00000050788	1	176086659	176087600	24	26
1258	1	156799065	156800018	ENSMUSG00000051509	ENSMUSG00000051509	ENSMUSG00000051509	1	176267105	176268058	24	26
1259	1	238321604	238705106	ENSMUSG00000028354	ENSMUSG00000028354	ENSMUSG00000028354	1	176338388	176578391	25	27
1260	1	238719498	238842085	ENSMUSG00000050069	ENSMUSG00000050069	ENSMUSG00000050069	1	176670460	176758494	25	27
1261	1	239030570	239585699	ENSMUSG00000026527	ENSMUSG00000026527	ENSMUSG00000026527	1	176903316	177327805	25	27
1262	1	239762330	239823322	ENSMUSG00000039783	ENSMUSG00000039783	ENSMUSG00000039783	1	177468868	177497528	25	27
1263	1	239823075	239870324	ENSMUSG00000026525	ENSMUSG00000026525	ENSMUSG00000026525	1	177499096	177529265	25	27
1264	1	239882203	240031580	ENSMUSG00000026523	ENSMUSG00000026523	ENSMUSG00000026523	1	177535462	177652038	25	27
1265	1	240078105	240119864	ENSMUSG00000039748	ENSMUSG00000039748	ENSMUSG00000039748	1	177717473	177745968	25	27
1266	1	240318312	240754621	ENSMUSG00000055214	ENSMUSG00000055214	ENSMUSG00000055214	1	177798981	178111973	25	27
1267	1	241486099	241729710	ENSMUSG00000026504	ENSMUSG00000026504	ENSMUSG00000026504	1	178651532	178856819	25	27
1268	1	241731688	242080053	ENSMUSG00000019699	ENSMUSG00000019699	ENSMUSG00000019699	1	178862039	178967772	25	27
1269	1	242281208	242287397	ENSMUSG00000063659	ENSMUSG00000063659	ENSMUSG00000063659	1	179279082	179287437	25	27
1270	1	242582560	242619016	ENSMUSG00000015962	ENSMUSG00000015962	ENSMUSG00000015962	1	179566489	179589980	25	27
1271	1	242638419	242682059	ENSMUSG00000015961	ENSMUSG00000015961	ENSMUSG00000015961	1	179599853	179633184	25	27
1272	1	242691301	242870429	ENSMUSG00000066667	ENSMUSG00000066667	ENSMUSG00000066667	1	179839668	179883678	25	27
1273	1	242882860	242938958	ENSMUSG00000026502	ENSMUSG00000026502	ENSMUSG00000026502	1	180074587	180086143	25	27
1274	1	243065262	243074980	ENSMUSG00000026500	ENSMUSG00000026500	ENSMUSG00000026500	1	180155828	180159368	25	27
1275	1	243081091	243094467	ENSMUSG00000039630	ENSMUSG00000039630	ENSMUSG00000039630	1	180165713	180174460	25	27
1276	1	243199907	243352916	ENSMUSG00000026495	ENSMUSG00000026495	ENSMUSG00000026495	1	180242773	180320716	25	27
1277	1	243385350	243386608	ENSMUSG00000054274	ENSMUSG00000054274	ENSMUSG00000054274	1	180365800	180553175	25	27
1278	1	243740927	243933051	ENSMUSG00000026494	ENSMUSG00000026494	ENSMUSG00000026494	1	180635113	180769530	25	27
1279	1	243979267	244737182	ENSMUSG00000055067	ENSMUSG00000055067	ENSMUSG00000055067	1	180794136	181354626	25	27
1280	1	244796371	244898509	ENSMUSG00000038949	ENSMUSG00000038949	ENSMUSG00000038949	1	181383204	181464153	25	27
1281	1	244953972	244998062	ENSMUSG00000038936	ENSMUSG00000038936	ENSMUSG00000038936	1	181504906	181523859	25	27
1282	1	225246676	225572449	ENSMUSG00000026490	ENSMUSG00000026490	ENSMUSG00000026490	1	181836036	181998838	26	28
1283	1	225151860	225241869	ENSMUSG00000026489	ENSMUSG00000026489	ENSMUSG00000026489	1	182001815	182032687	26	28
1284	1	225125592	225150422	ENSMUSG00000010609	ENSMUSG00000010609	ENSMUSG00000010609	1	182063681	182082576	26	28
1285	1	224886016	224991833	ENSMUSG00000038855	ENSMUSG00000038855	ENSMUSG00000038855	1	182168986	182258559	26	28
1286	1	224615015	224662397	ENSMUSG00000026496	ENSMUSG00000026496	ENSMUSG00000026496	1	182405648	182437928	26	28
1287	1	224485481	224564057	ENSMUSG00000058729	ENSMUSG00000058729	ENSMUSG00000058729	1	182478028	182518303	26	28
1288	1	224478006	224480687	ENSMUSG00000026497	ENSMUSG00000026497	ENSMUSG00000026497	1	182529718	182533709	26	28
1289	1	224399003	224441054	ENSMUSG00000026499	ENSMUSG00000026499	ENSMUSG00000026499	1	182562718	182590840	26	28
1290	1	224237026	224253655	ENSMUSG00000038806	ENSMUSG00000038806	ENSMUSG00000038806	1	182687826	182704788	26	28
1291	1	224174201	224178433	ENSMUSG00000026520	ENSMUSG00000026520	ENSMUSG00000026520	1	182740984	182744763	26	28
1292	1	224099860	224136692	ENSMUSG00000026519	ENSMUSG00000026519	ENSMUSG00000026519	1	182780757	182811778	26	28
1293	1	224064459	224099883	ENSMUSG00000038776	ENSMUSG00000038776	ENSMUSG00000038776	1	182826233	182854196	26	28
1294	1	222481659	222584503	ENSMUSG00000026516	ENSMUSG00000026516	ENSMUSG00000026516	1	182930098	182980862	27	29
1295	1	222611175	222633777	ENSMUSG00000062169	ENSMUSG00000062169	ENSMUSG00000062169	1	182987662	183003105	27	29
1296	1	222639728	222688624	ENSMUSG00000038733	ENSMUSG00000038733	ENSMUSG00000038733	1	183014036	183048653	27	29
1297	1	222870618	222994889	ENSMUSG00000026514	ENSMUSG00000026514	ENSMUSG00000026514	1	183189305	183297316	27	29
1298	1	223184007	223297258	ENSMUSG00000070467	ENSMUSG00000070467	ENSMUSG00000070467	1	183430103	183442610	27	29
1299	1	223495119	223652370	ENSMUSG00000047369	ENSMUSG00000047369	ENSMUSG00000047369	1	183577875	183600650	27	29
1300	1	223655827	223682360	ENSMUSG00000004880	ENSMUSG00000004880	ENSMUSG00000004880	1	183651991	183679029	27	29
1301	1	223751724	223907041	ENSMUSG00000022995	ENSMUSG00000022995	ENSMUSG00000022995	1	183742535	183798596	27	29
1302	1	222368436	222416372	ENSMUSG00000047539	ENSMUSG00000047539	ENSMUSG00000047539	1	184149777	184178281	28	30
1303	1	222034414	222075728	ENSMUSG00000026510	ENSMUSG00000026510	ENSMUSG00000026510	1	184265679	184299107	28	30
1304	1	221966657	222030343	ENSMUSG00000026509	ENSMUSG00000026509	ENSMUSG00000026509	1	184306854	184354029	28	30
1305	1	221871664	221920016	ENSMUSG00000038599	ENSMUSG00000038599	ENSMUSG00000038599	1	184401736	184466835	28	30
1306	1	221633382	221635435	ENSMUSG00000043429	ENSMUSG00000043429	ENSMUSG00000043429	1	184583932	184585797	28	30
1307	1	221460786	221604097	ENSMUSG00000038576	ENSMUSG00000038576	ENSMUSG00000038576	1	184601571	184732331	28	30
1308	1	219941389	219982141	ENSMUSG00000039384	ENSMUSG00000039384	ENSMUSG00000039384	1	185735717	185776892	28	30
1309	1	219119366	219125022	ENSMUSG00000039377	ENSMUSG00000039377	ENSMUSG00000039377	1	186427931	186433396	28	30
1310	1	219026724	219054353	ENSMUSG00000026621	ENSMUSG00000026621	ENSMUSG00000026621	1	186488076	186511985	28	30
1311	1	218988299	219024773	ENSMUSG00000073481	ENSMUSG00000073481	ENSMUSG00000073481	1	186513856	186546635	28	30
1312	1	218929810	218939120	ENSMUSG00000039349	ENSMUSG00000039349	ENSMUSG00000039349	1	186572714	186583802	28	30
1313	1	218768522	218902423	ENSMUSG00000026620	ENSMUSG00000026620	ENSMUSG00000026620	1	186599312	186645788	28	30
1314	1	218334078	218387999	ENSMUSG00000026618	ENSMUSG00000026618	ENSMUSG00000026618	1	186987428	187030189	28	30
1315	1	218297727	218329807	ENSMUSG00000026617	ENSMUSG00000026617	ENSMUSG00000026617	1	187032947	187058557	28	30
1316	1	218208566	218286623	ENSMUSG00000026615	ENSMUSG00000026615	ENSMUSG00000026615	1	187063883	187129143	28	30
1317	1	218154230	218168616	ENSMUSG00000026614	ENSMUSG00000026614	ENSMUSG00000026614	1	187155636	187169550	28	30
1318	1	217413809	217452830	ENSMUSG00000039246	ENSMUSG00000039246	ENSMUSG00000039246	1	187788668	187818098	28	30
1319	1	216586200	216684584	ENSMUSG00000039239	ENSMUSG00000039239	ENSMUSG00000039239	1	188324430	188406777	28	30
1320	1	216525252	216577948	ENSMUSG00000001305	ENSMUSG00000001305	ENSMUSG00000001305	1	188421766	188450123	28	30
1321	1	215871289	216111661	ENSMUSG00000026611	ENSMUSG00000026611	ENSMUSG00000026611	1	188749197	188916209	28	30
1322	1	215666957	215871047	ENSMUSG00000039210	ENSMUSG00000039210	ENSMUSG00000039210	1	188916327	189052233	28	30
1323	1	214743219	214963418	ENSMUSG00000026610	ENSMUSG00000026610	ENSMUSG00000026610	1	189698647	189915672	28	30
1324	1	213807358	213861770	ENSMUSG00000026608	ENSMUSG00000026608	ENSMUSG00000026608	1	190671886	190708628	28	30
1325	1	213323223	213477059	ENSMUSG00000037624	ENSMUSG00000037624	ENSMUSG00000037624	1	190910578	191043876	28	30
1326	1	212597474	212791189	ENSMUSG00000026604	ENSMUSG00000026604	ENSMUSG00000026604	1	191487431	191571095	28	30
1327	1	212521091	212577093	ENSMUSG00000026603	ENSMUSG00000026603	ENSMUSG00000026603	1	191581280	191623076	28	30
1328	1	212228483	212281219	ENSMUSG00000010175	ENSMUSG00000010175	ENSMUSG00000010175	1	191822557	191863034	28	30
1329	1	211291220	211514739	ENSMUSG00000037581	ENSMUSG00000037581	ENSMUSG00000037581	1	192499063	192612349	28	30
1330	1	211232147	211255791	ENSMUSG00000026634	ENSMUSG00000026634	ENSMUSG00000026634	1	192629160	192646353	28	30
1331	1	211191508	211230027	ENSMUSG00000037568	ENSMUSG00000037568	ENSMUSG00000037568	1	192648362	192679839	28	30
1332	1	211098220	211139328	ENSMUSG00000066595	ENSMUSG00000066595	ENSMUSG00000066595	1	192725968	192726708	28	30
1333	1	211070114	211087614	ENSMUSG00000057072	ENSMUSG00000057072	ENSMUSG00000057072	1	192737437	192743557	28	30
1334	1	211031882	211056589	ENSMUSG00000026632	ENSMUSG00000026632	ENSMUSG00000026632	1	192746723	192763519	28	30
1335	1	210976921	211031747	ENSMUSG00000062510	ENSMUSG00000062510	ENSMUSG00000062510	1	192763646	192785173	28	30
1336	1	210926383	210939950	ENSMUSG00000026630	ENSMUSG00000026630	ENSMUSG00000026630	1	192799029	192809558	28	30
1337	1	210864439	210866743	ENSMUSG00000073479	ENSMUSG00000073479	ENSMUSG00000073479	1	192863302	192865053	28	30
1338	1	210805374	210860740	ENSMUSG00000026628	ENSMUSG00000026628	ENSMUSG00000026628	1	192870911	192883955	28	30
1339	1	210672852	210686337	ENSMUSG00000037499	ENSMUSG00000037499	ENSMUSG00000037499	1	193007412	193018733	28	30
1340	1	210603896	210654860	ENSMUSG00000026627	ENSMUSG00000026627	ENSMUSG00000026627	1	193026580	193053540	28	30
1341	1	210275542	210344971	ENSMUSG00000037474	ENSMUSG00000037474	ENSMUSG00000037474	1	193239720	193276129	28	30
1342	1	210180364	210275501	ENSMUSG00000037461	ENSMUSG00000037461	ENSMUSG00000037461	1	193276378	193323287	28	30
1343	1	209983422	210070737	ENSMUSG00000026623	ENSMUSG00000026623	ENSMUSG00000026623	1	193418638	193480592	28	30
1344	1	209902737	209915583	ENSMUSG00000026622	ENSMUSG00000026622	ENSMUSG00000026622	1	193522148	193533663	28	30
1345	1	209811533	209818688	ENSMUSG00000037434	ENSMUSG00000037434	ENSMUSG00000037434	1	193607396	193613862	28	30
1346	1	209716487	209732882	ENSMUSG00000049353	ENSMUSG00000049353	ENSMUSG00000049353	1	193678140	193688706	28	30
1347	1	209566580	209614905	ENSMUSG00000026637	ENSMUSG00000026637	ENSMUSG00000026637	1	193697855	193771240	28	30
1348	1	209499940	209556350	ENSMUSG00000037395	ENSMUSG00000037395	ENSMUSG00000037395	1	193801013	193838754	28	30
1349	1	208923178	209374080	ENSMUSG00000058248	ENSMUSG00000058248	ENSMUSG00000058248	1	193891596	194210882	28	30
1350	1	208568920	208916261	ENSMUSG00000037375	ENSMUSG00000037375	ENSMUSG00000037375	1	194213552	194471745	28	30
1351	1	208472767	208486223	ENSMUSG00000016262	ENSMUSG00000016262	ENSMUSG00000016262	1	194545212	194556445	28	30
1352	1	208178199	208401010	ENSMUSG00000016200	ENSMUSG00000016200	ENSMUSG00000016200	1	194598031	194687607	28	30
1353	1	208067956	208097531	ENSMUSG00000016181	ENSMUSG00000016181	ENSMUSG00000016181	1	194805127	194830975	28	30
1354	1	208025659	208046012	ENSMUSG00000026638	ENSMUSG00000026638	ENSMUSG00000026638	1	194853886	194872730	28	30
1355	1	207996017	208022283	ENSMUSG00000037318	ENSMUSG00000037318	ENSMUSG00000037318	1	194876230	194902210	28	30
1356	1	207926133	207974832	ENSMUSG00000016194	ENSMUSG00000016194	ENSMUSG00000016194	1	194922413	194964769	28	30
1357	1	207915388	207916356	ENSMUSG00000009633	ENSMUSG00000009633	ENSMUSG00000009633	1	194972886	194973833	28	30
1358	1	207854843	207892443	ENSMUSG00000026639	ENSMUSG00000026639	ENSMUSG00000026639	1	195002750	195044602	28	30
1359	1	207823668	207853906	ENSMUSG00000016179	ENSMUSG00000016179	ENSMUSG00000016179	1	195047071	195070955	28	30
1360	1	206262210	206484288	ENSMUSG00000026640	ENSMUSG00000026640	ENSMUSG00000026640	1	196320645	196513303	28	30
1361	1	206126507	206151370	ENSMUSG00000016494	ENSMUSG00000016494	ENSMUSG00000016494	1	196639610	196662005	28	30
1362	1	205992025	206035481	ENSMUSG00000016493	ENSMUSG00000016493	ENSMUSG00000016493	1	196763073	196792949	28	30

1363	1	205909413	205978508	ENSMUSG00000016481	ENSMUSG00000016481	ENSMUSG00000016481	1	196804512	196832029	28	30
1364	1	205694198	205729863	ENSMUSG00000026616	ENSMUSG00000026616	ENSMUSG00000026616	1	196841897	196877439	28	30
1365	10	15293661	15453103	ENSMUSG00000050530	ENSMUSG00000050530	ENSMUSG00000050530	2	3035828	3143779	29	31
1366	10	15190962	15250633	ENSMUSG00000026643	ENSMUSG00000026643	ENSMUSG00000026643	2	3201484	3246149	29	31
1367	10	15179185	15186256	ENSMUSG00000049950	ENSMUSG00000049950	ENSMUSG00000049950	2	3246221	3249874	29	31
1368	10	15125968	15155857	ENSMUSG00000026645	ENSMUSG00000026645	ENSMUSG00000026645	2	3259267	3283863	29	31
1369	10	15048444	15054856	ENSMUSG00000026650	ENSMUSG00000026650	ENSMUSG00000026650	2	3326315	3339920	29	31
1370	10	14988903	15036100	ENSMUSG00000026648	ENSMUSG00000026648	ENSMUSG00000026648	2	3341422	3371364	29	31
1371	10	14960871	14985125	ENSMUSG00000026646	ENSMUSG00000026646	ENSMUSG00000026646	2	3373092	3392235	29	31
1372	10	14920169	14953746	ENSMUSG00000051396	ENSMUSG00000051396	ENSMUSG00000051396	2	3406126	3430086	29	31
1373	10	14901257	14919989	ENSMUSG00000039496	ENSMUSG00000039496	ENSMUSG00000039496	2	3430335	3443648	29	31
1374	10	14600565	14856902	ENSMUSG00000026655	ENSMUSG00000026655	ENSMUSG00000026655	2	3630730	3699406	29	31
1375	10	13725718	14412889	ENSMUSG00000026657	ENSMUSG00000026657	ENSMUSG00000026657	2	4348471	4528341	29	31
1376	10	13668933	13712874	ENSMUSG00000039449	ENSMUSG00000039449	ENSMUSG00000039449	2	4539439	4569358	29	31
1377	10	13520643	13584982	ENSMUSG00000048186	ENSMUSG00000048186	ENSMUSG00000048186	2	4635417	4718197	29	31
1378	10	13359849	13381554	ENSMUSG00000026664	ENSMUSG00000026664	ENSMUSG00000026664	2	4836291	4856001	29	31
1379	10	13303773	13316337	ENSMUSG00000026668	ENSMUSG00000026668	ENSMUSG00000026668	2	4893453	4903017	29	31
1380	10	13243587	13293106	ENSMUSG00000026669	ENSMUSG00000026669	ENSMUSG00000026669	2	4906986	4929616	29	31
1381	10	13181455	13220297	ENSMUSG00000026672	ENSMUSG00000026672	ENSMUSG00000026672	2	4937914	4981210	29	31
1382	10	12978666	13083744	ENSMUSG00000026676	ENSMUSG00000026676	ENSMUSG00000026676	2	5055048	5148150	29	31
1383	10	12431487	12917551	ENSMUSG00000039145	ENSMUSG00000039145	ENSMUSG00000039145	2	5210729	5631936	29	31
1384	10	12277971	12332545	ENSMUSG00000039128	ENSMUSG00000039128	ENSMUSG00000039128	2	5711566	5762232	29	31
1385	10	12249581	12277827	ENSMUSG00000025817	ENSMUSG00000025817	ENSMUSG00000025817	2	5762306	5786008	29	31
1386	10	12211704	12246832	ENSMUSG00000025816	ENSMUSG00000025816	ENSMUSG00000025816	2	5788259	5812704	29	31
1387	10	12150977	12205230	ENSMUSG00000025815	ENSMUSG00000025815	ENSMUSG00000025815	2	5815989	5859937	29	31
1388	10	12002027	12124839	ENSMUSG00000043241	ENSMUSG00000043241	ENSMUSG00000043241	2	5874761	5968902	29	31
1389	10	11905403	11954282	ENSMUSG00000045319	ENSMUSG00000045319	ENSMUSG00000045319	2	6017297	6047418	29	31
1390	10	11824371	11846075	ENSMUSG00000039063	ENSMUSG00000039063	ENSMUSG00000039063	2	6105737	6130266	29	31
1391	10	11693306	11694231	ENSMUSG00000048749	ENSMUSG00000048749	ENSMUSG00000048749	2	6239603	6240043	29	31
1392	10	11542517	11693759	ENSMUSG00000039046	ENSMUSG00000039046	ENSMUSG00000039046	2	6253630	6361065	29	31
1393	10	11247456	11418672	ENSMUSG00000002107	ENSMUSG00000002107	ENSMUSG00000002107	2	6459145	6638887	29	31
1394	10	8136662	8157167	ENSMUSG00000015619	ENSMUSG00000015619	ENSMUSG00000015619	2	9774921	9795442	29	31
1395	10	7900473	8098596	ENSMUSG00000025782	ENSMUSG00000025782	ENSMUSG00000025782	2	9833449	9966469	29	31
1396	10	7837198	7869950	ENSMUSG00000037262	ENSMUSG00000037262	ENSMUSG00000037262	2	9998465	10010557	29	31
1397	10	7785238	7831489	ENSMUSG00000037254	ENSMUSG00000037254	ENSMUSG00000037254	2	10012442	10048516	29	31
1398	10	7641238	7748940	ENSMUSG00000025780	ENSMUSG00000025780	ENSMUSG00000025780	2	10071424	10169624	29	31
1399	10	7240592	7491309	ENSMUSG00000061186	ENSMUSG00000061186	ENSMUSG00000061186	2	10290303	10508423	29	31
1400	10	6509111	6662269	ENSMUSG00000026778	ENSMUSG00000026778	ENSMUSG00000026778	2	11090235	11219079	29	31
1401	10	6284835	6315059	ENSMUSG00000026773	ENSMUSG00000026773	ENSMUSG00000026773	2	11389288	11419920	29	31
1402	10	6170956	6198849	ENSMUSG00000037197	ENSMUSG00000037197	ENSMUSG00000037197	2	11503292	11521052	29	31
1403	10	6092658	6144294	ENSMUSG00000026770	ENSMUSG00000026770	ENSMUSG00000026770	2	11560703	11611044	29	31
1404	10	6034340	6059559	ENSMUSG00000023206	ENSMUSG00000023206	ENSMUSG00000023206	2	11623664	11651254	29	31
1405	10	5976355	6019562	ENSMUSG00000058594	ENSMUSG00000058594	ENSMUSG00000058594	2	11660426	11695435	29	31
1406	10	5943695	5971867	ENSMUSG00000047909	ENSMUSG00000047909	ENSMUSG00000047909	2	11696442	11707729	29	31
1407	10	15595954	15802130	ENSMUSG00000026768	ENSMUSG00000026768	ENSMUSG00000026768	2	12024513	12219773	30	32
1408	10	15860175	15942525	ENSMUSG00000026767	ENSMUSG00000026767	ENSMUSG00000026767	2	12265117	12337313	30	32
1409	10	16518970	16595742	ENSMUSG00000026730	ENSMUSG00000026730	ENSMUSG00000026730	2	12841977	12919466	30	32
1410	10	16595748	16604010	ENSMUSG00000049630	ENSMUSG00000049630	ENSMUSG00000049630	2	12921293	12929600	30	32
1411	10	16672616	16899533	ENSMUSG00000026727	ENSMUSG00000026727	ENSMUSG00000026727	2	12994821	13189161	30	32
1412	10	16905969	17211836	ENSMUSG00000026726	ENSMUSG00000026726	ENSMUSG00000026726	2	13194192	13409666	30	32
1413	10	17224851	17283665	ENSMUSG00000026723	ENSMUSG00000026723	ENSMUSG00000026723	2	13428017	13462517	30	32
1414	10	17401879	17536335	ENSMUSG00000003418	ENSMUSG00000003418	ENSMUSG00000003418	2	13572782	13711274	30	32
1415	10	17726130	17797919	ENSMUSG00000026718	ENSMUSG00000026718	ENSMUSG00000026718	2	13991854	14066092	30	32
1416	10	18280898	18372221	ENSMUSG00000036949	ENSMUSG00000036949	ENSMUSG00000036949	2	14306158	14412731	30	32
1417	10	18469612	18870062	ENSMUSG00000057914	ENSMUSG00000057914	ENSMUSG00000057914	2	14522205	14903830	30	32
1418	10	18874496	18980557	ENSMUSG00000026707	ENSMUSG00000026707	ENSMUSG00000026707	2	14912869	14967034	30	32
1419	10	18988340	19010574	ENSMUSG00000017418	ENSMUSG00000017418	ENSMUSG00000017418	2	14973077	14996920	30	32
1420	10	19532671	19538621	ENSMUSG00000071017	ENSMUSG00000071017	ENSMUSG00000071017	2	15612955	15789335	30	32
1421	10	19827422	19896508	ENSMUSG00000075520	ENSMUSG00000075520	ENSMUSG00000075520	2	15959877	16019580	30	32
1422	10	20145174	20609292	ENSMUSG00000026748	ENSMUSG00000026748	ENSMUSG00000026748	2	16274058	16670059	30	32
1423	10	21110098	21226537	ENSMUSG00000053702	ENSMUSG00000053702	ENSMUSG00000053702	2	17266451	17378292	30	32
1424	10	21844031	21846757	ENSMUSG00000054074	ENSMUSG00000054074	ENSMUSG00000054074	2	17963364	17966093	30	32
1425	10	21863278	22072565	ENSMUSG00000026743	ENSMUSG00000026743	ENSMUSG00000026743	2	17982789	18128566	30	32
1426	10	22085472	22332660	ENSMUSG00000026740	ENSMUSG00000026740	ENSMUSG00000026740	2	18124088	18310565	30	32
1427	10	22644909	22649241	ENSMUSG00000051154	ENSMUSG00000051154	ENSMUSG00000051154	2	18590191	18593984	30	32
1428	10	22650146	22660419	ENSMUSG00000026739	ENSMUSG00000026739	ENSMUSG00000026739	2	18594772	18604385	30	32
1429	10	22863784	23043490	ENSMUSG00000026737	ENSMUSG00000026737	ENSMUSG00000026737	2	18760014	18915866	30	32
1430	10	23256966	23366501	ENSMUSG00000037683	ENSMUSG00000037683	ENSMUSG00000037683	2	19153236	19214737	30	32
1431	10	23424437	23449905	ENSMUSG00000023094	ENSMUSG00000023094	ENSMUSG00000023094	2	19289390	19312726	30	32
1432	10	23521262	23523187	ENSMUSG00000026735	ENSMUSG00000026735	ENSMUSG00000026735	2	19363392	19365251	30	32
1433	10	23596130	23672899	ENSMUSG00000026734	ENSMUSG00000026734	ENSMUSG00000026734	2	19380591	19471664	30	32
1434	10	24538099	24876777	ENSMUSG00000036617	ENSMUSG00000036617	ENSMUSG00000036617	2	20437612	20726961	30	32
1435	10	24912562	25052603	ENSMUSG00000036591	ENSMUSG00000036591	ENSMUSG00000036591	2	20765696	20885475	30	32
1436	10	25177560	25281539	ENSMUSG00000075513	ENSMUSG00000075513	ENSMUSG00000075513	2	20962562	21059708	30	32
1437	10	25310914	25345091	ENSMUSG00000026679	ENSMUSG00000026679	ENSMUSG00000026679	2	21098485	21123119	30	32
1438	10	25345593	25355599	ENSMUSG00000048550	ENSMUSG00000048550	ENSMUSG00000048550	2	21123478	21132763	30	32
1439	10	25503997	25931161	ENSMUSG00000045967	ENSMUSG00000045967	ENSMUSG00000045967	2	21285321	21748298	30	32
1440	10	26263202	26541462	ENSMUSG00000025716	ENSMUSG00000025716	ENSMUSG00000025716	2	22158822	22469894	30	32
1441	10	26545242	26633493	ENSMUSG00000026787	ENSMUSG00000026787	ENSMUSG00000026787	2	22473965	22542637	30	32
1442	10	26767138	26896738	ENSMUSG00000026786	ENSMUSG00000026786	ENSMUSG00000026786	2	22668600	22726968	30	32
1443	10	27026594	27075733	ENSMUSG00000026784	ENSMUSG00000026784	ENSMUSG00000026784	2	22747242	22767854	30	32
1444	10	27075531	27189965	ENSMUSG00000058835	ENSMUSG00000058835	ENSMUSG00000058835	2	22772194	22862074	30	32
1445	10	27526288	27569493	ENSMUSG00000026781	ENSMUSG00000026781	ENSMUSG00000026781	2	22891279	22932776	30	32
1446	10	27484305	27515854	ENSMUSG00000026779	ENSMUSG00000026779	ENSMUSG00000026779	2	22940074	22977925	30	32
1447	10	27439394	27483327	ENSMUSG00000026775	ENSMUSG00000026775	ENSMUSG00000026775	2	22978555	23020645	30	32
1448	2	139144962	139145717	ENSMUSG00000069132	ENSMUSG00000069132	ENSMUSG00000069132	2	23143255	23223982	31	33
1449	2	138975841	139047267	ENSMUSG00000026771	ENSMUSG00000026771	ENSMUSG00000026771	2	23332063	23394063	31	33
1450	2	138438278	138490403	ENSMUSG00000026986	ENSMUSG00000026986	ENSMUSG00000026986	2	23824918	23871385	31	33
1451	2	113496139	113526917	ENSMUSG00000026985	ENSMUSG00000026985	ENSMUSG00000026985	2	23975170	23982528	32	34
1452	2	113452077	113459698	ENSMUSG00000044103	ENSMUSG00000044103	ENSMUSG00000044103	2	24008491	24015576	32	34
1453	2	113480012	113482092	ENSMUSG00000026984	ENSMUSG00000026984	ENSMUSG00000026984	2	24037426	24047710	32	34
1454	2	113532686	113538787	ENSMUSG00000026983	ENSMUSG00000026983	ENSMUSG00000026983	2	24098963	24105435	32	34
1455	2	113542018	113549896	ENSMUSG00000046845	ENSMUSG00000046845	ENSMUSG00000046845	2	24113205	24115829	32	34
1456	2	113591941	113608063	ENSMUSG00000026981	ENSMUSG00000026981	ENSMUSG00000026981	2	24158869	24173490	32	34
1457	2	113648031	113677148	ENSMUSG00000026979	ENSMUSG00000026979	ENSMUSG00000026979	2	24189619	24231191	32	34
1458	2	113690046	113720991	ENSMUSG00000026976	ENSMUSG00000026976	ENSMUSG00000026976	2	24242565	24297583	32	34
1459	9	139892062	140138897	ENSMUSG00000004113	ENSMUSG00000004113	ENSMUSG00000004113	2	24428579	24585056	33	35
1460	9	139725238	139850397	ENSMUSG00000036893	ENSMUSG00000036893	ENSMUSG00000036893	2	24612778	24741618	33	35
1461	9	139619917	139629630	ENSMUSG00000026972	ENSMUSG00000026972	ENSMUSG00000026972	2	24747364	24757174	33	35
1462	9	139596352	139604763	ENSMUSG00000026974	ENSMUSG00000026974	ENSMUSG00000026974	2	24771791	24781534	33	35
1463	9	139569182	139593208	ENSMUSG00000026975	ENSMUSG00000026975	ENSMUSG00000026975	2	24784454	24795480	33	35
1464	9	139474226	139564657	ENSMUSG00000036833	ENSMUSG00000036833	ENSMUSG00000036833	2	24798071	24876064	33	35
1465	9	139462505	139473607	ENSMUSG00000006476	ENSMUSG00000006476	ENSMUSG00000006476	2	24876388	24884890	33	35
1466	9	139448638	139456089	ENSMUSG00000036813	ENSMUSG00000036813	ENSMUSG00000036813	2	24902331	24907724	33	35
1467	9	139437668	139448678	ENSMUSG00000036805	ENSMUSG00000036805	ENSMUSG00000036805	2	24907676	24917158	33	35
1468	9	139292099	139296913	ENSMUSG00000059555	ENSMUSG00000059555	ENSMUSG00000059555	2	25014729	25018622	33	35
1469	9	139269580	139287819	ENSMUSG00000013465	ENSMUSG00000013465	ENSMUSG00000013465	2	25021725	25033212	33	35
1470	9	139265551	139267736	ENSMUSG00000036770	ENSMUSG00000036770	ENSMUSG00000036770	2	25034217	25036640	33	35
1471	9	139257857	139262043	ENSMUSG00000026969	ENSMUSG00000026969	ENSMUSG00000026969	2	25040755	25044289	33	35
1472	9	139245191	139250827	ENSMUSG00000006469	ENSMUSG00000006469	ENSMUSG00000006469	2	25050906	25056212	33	35
1473	9	139238908	139240578	ENSMUSG00000036731	ENSMUSG00000036731	ENSMUSG00000036731	2	25060836	25061906	33	35
1474	9	139234700	139235242	ENSMUSG00000044628	ENSMUSG00000044628	ENSMUSG00000044628	2	25064947	25066271	33	35
1475	9	139219968	139231259	ENSMUSG00000006471	ENSMUSG00000006471	ENSMUSG00000006471	2	25066828	25077387	33	35
1476	9	139205890	139214378	ENSMUSG00000048707	ENSMUSG00000048707	ENSMUSG00000048707	2	25084627	25091891	33	35
1477	9	139202920	139204643	ENSMUSG00000026966	ENSMUSG00000026966	ENSMUSG00000026966	2	25093048	25094421	33	35
1478	9	139189057	139202878	ENSMUSG00000026965	ENSMUSG00000026965	ENSMUSG00000026965	2	25094508	25107924	33	35
1479	9	139185248	139186300	ENSMUSG00000026963	ENSMUSG00000026963	ENSMUSG00000026963	2	25110154	25111192	33	35
1480	9	139183031	139184324	ENSMUSG00000026961	ENSMUSG00000026961	ENSMUSG00000026961	2	25111925	25113186	33	35
1481	9	139152663	139183028	ENSMUSG00000026959	ENSMUSG00000026959	ENSMUSG00000026959	2	25114256	25140988	33	35
1482	9	139102641	139117583	ENSMUSG00000036646	ENSMUSG00000036646	ENSMUSG00000036646	2	25154765	25174221	33	35
1483	9	139124814	139129016	ENSMUSG00000026958	ENSMUSG00000026958	ENSMUSG00000026958	2	25174296	25178340	33	35
1484	9	139092414	139098812	ENSMUSG00000026956	ENSMUSG00000026956	ENSMUSG00000026956	2	25182365	25187633	33	35
1485	9	139076402	139084805	ENSMUSG00000026955	ENSMUSG00000026955	ENSMUSG00000026955	2	25194348	25198489	33	35
1486	9	139062374	139068326	ENSMUSG00000015085	ENSMUSG00000015085	ENSMUSG00000015085	2	25217912	25223324	33	35
1487	9	139053743	139058365	ENSMUSG00000015094	ENSMUSG00000015094	ENSMUSG00000015094	2	25225092	25231503	33	35
1488	9	139044447	139047283	ENSMUSG00000036587	ENSMUSG00000036587	ENSMUSG00000036587	2	25245498	25248380	33	35
1489	9	139021507	139043195	ENSMUSG00000026944	ENSMUSG00000026944	ENSMUSG00000026944	2	25250712	25270549	33	35
1490	9	139008908	139011076	ENSMUSG00000015093	ENSMUSG00000015093	ENSMUSG00000015093	2	25278847	25280783	33	35
1491	9	139006691	139008249	ENSMUSG00000047617	ENSMUSG00000047617	ENSMUSG00000047617	2	25281495	25282920	33	35
1492	9	138989367	138996014	ENSMUSG00000015090	ENSMUSG00000015090	ENSMUSG00000015090	2	25286029	25291745	33	35
1493	9	138963665	138969046	ENSMUSG00000026943	ENSMUSG00000026943	ENSMUSG00000026943	2	25312852	25315920	33	35
1494	9	138959534	138961240	ENSMUSG00000015083	ENSMUSG00000015083	ENSMUSG00000015083	2	25320665	25322515	33	35
1495	9	138954709	138958994	ENSMUSG00000015095	ENSMUSG00000015095	ENSMUSG00000015095	2	25323803	25327479	33	35
1496	9	138900786	138940887	ENSMUSG00000026942	ENSMUSG00000026942	ENSMUSG00000026942	2	25339991	25368903	33	35
1497	9	138876392	138880559	ENSMUSG00000015092	ENSMUSG00000015092	ENSMUSG00000015092	2	25379884	25384084	33	35
1498	9	138866640	138875070	ENSMUSG00000026941	ENSMUSG00000026941	ENSMUSG00000026941	2	25385340	25391820	33	35
1499	9	138858898	138861333	ENSMUSG00000029419	ENSMUSG00000029419	ENSMUSG00000029419	2	25398982	25401906	33	35
1500	9	138822195	138855460	ENSMUSG00000015087	ENSMUSG00000015087	ENSMUSG00000015087	2	25405027	25430455	33	35
1501	9	138768771	138772816	ENSMUSG00000036449	ENSMUSG00000036449	ENSMUSG00000036449	2	25475129	25478229	33	35
1502	9	138758293	138762801	ENSMUSG00000045684	ENSMUSG00000045684	ENSMUSG00000045684	2	25501275	25503614	33	35
1503	9	138753798	138757176	ENSMUSG00000047356	ENSMUSG00000047356	ENSMUSG00000047356	2	25504735	25508090	33	35
1504	9	137655323	137671207	ENSMUSG00000036401	ENSMUSG00000036401	ENSMUSG00000036401	2	25615868	25637852	34	36
1505	9	137694989	137697580	ENSMUSG00000023210	ENSMUSG00000023210	ENSMUSG00000023210	2	25645162	25647546	34	36
1506	9	137725074	137731195	ENSMUSG00000059625	ENSMUSG00000059625	ENSMUSG00000059625	2	25665004	25669257	34	36
1507	9	137733859	137826386	ENSMUSG00000058740	ENSMUSG00000058740	ENSMUSG00000058740	2	25699870	25740282	34	36
1508	9	138038204	138082247	ENSMUSG00000026932	ENSMUSG00000026932	ENSMUSG00000026932	2	25877545	25944820	34	36
1509	9	138227917	138234825	ENSMUSG00000026934	ENSMUSG00000026934	ENSMUSG00000026934	2	26022956	26030258	34	36
1510	9	138240395	138277470	ENSMUSG00000036327	ENSMUSG00000036327	ENSMUSG00000036327	2	26032143	26059361	34	36
1511	9	138352955	138373872	ENSMUSG00000026930	ENSMUSG00000026930	ENSMUSG00000026930	2	26137549	26170244	34	36
1512	9	138376177	138387917	ENSMUSG00000026928	ENSMUSG00000026928	ENSMUSG00000026928	2	26174324	26181556	34	36
1513	9	138389851	138413072	ENSMUSG00000036281	ENSMUSG00000036281	ENSMUSG00000036281	2	26184783	26202628	34	36
1514	9	138424933	138438034	ENSMUSG00000026926	ENSMUSG00000026926	ENSMUSG00000026926	2	26211357	26219130	34	36
1515	9	138442892	138454095	ENSMUSG00000026925	ENSMUSG00000026925	ENSMUSG00000026925	2	26218258	26231197	34	36
1516	9	138454380	138491962	ENSMUSG00000026924	ENSMUSG00000026924	ENSMUSG00000026924	2	26243386	26263431	34	36
1517	9	138508717	138560135	ENSMUSG00000026923	ENSMUSG00000026923	ENSMUSG00000026923	2	26279912	26325831	34	36
1518	9	138673129	138686951	ENSMUSG00000026921	ENSMUSG00000026921	ENSMUSG00000026921	2	26403092	26414691	34	36
1519	9	138687416	138701732	ENSMUSG00000026922	ENSMUSG00000026922	ENSMUSG00000026922	2	26415099	26426219	34	36
1520	9	138726843	138738323	ENSMUSG00000036186	ENSMUSG00000036186	ENSMUSG00000036186	2	26450466	26458503	34	36
1521	9	135120384	135140451	ENSMUSG00000015787	ENSMUSG00000015787	ENSMUSG00000015787	2	26664512	26686988	35	37
1522	9	135187373	135193056	ENSMUSG00000036160	ENSMUSG00000036160	ENSMUSG00000036160	2	26712781	26724830	35	37
1523	9	135194981	135204379	ENSMUSG00000015776	ENSMUSG00000015776	ENSMUSG00000015776	2	26727813	26732651	35	37
1524	9	135208431	135213182	ENSMUSG00000015790	ENSMUSG00000015790	ENSMUSG00000015790	2	26735397	26738539	35	37
1525	9	135213249	135217866	ENSMUSG00000014873	ENSMUSG00000014873	ENSMUSG00000014873	2	26738430	26742181	35	37
1526	9	135218146	135232791	ENSMUSG00000014867	ENSMUSG00000014867	ENSMUSG00000014867	2	26742048	26755937	35	37
1527	9	135233059	135261041	ENSMUSG00000049897	ENSMUSG00000049897	ENSMUSG00000049897	2	26756078	26775505	35	37
1528	9	135261007	135272985	ENSMUSG00000052406	ENSMUSG00000052406	ENSMUSG00000052406	2	26775572	26786395	35	37
1529	9	135276941	135314329	ENSMUSG00000014852	ENSMUSG00000014852	ENSMUSG00000014852	2	26795502	26831637	35	37
1530	9	135314930	135325791	ENSMUSG00000015488	ENSMUSG00000015488	ENSMUSG00000015488	2	26831959	26843098	35	37
1531	9	135326038	135334039	ENSMUSG00000036067	ENSMUSG00000036067	ENSMUSG00000036067	2	26843377	26849999	35	37
1532	9	135369579	135379787	ENSMUSG00000009214	ENSMUSG00000009214	ENSMUSG00000009214	2	26883682	26894170	35	37
1533	9	135387107	135430461	ENSMUSG00000036040	ENSMUSG00000036040	ENSMUSG00000036040	2	26901390	26930622	35	37
1534	9	135433965	135435166	ENSMUSG00000009216	ENSMUSG00000009216	ENSMUSG00000009216	2	26934012	26964483	35	37
1535	9	135491306	135514287	ENSMUSG00000000889	ENSMUSG00000000889	ENSMUSG00000000889	2	26987242	27004928	35	37
1536	9	135518503	135594863	ENSMUSG00000009614	ENSMUSG00000009614	ENSMUSG00000009614	2	27011391	27068821	35	37
1537	9	135616838	135847226	ENSMUSG00000009621	ENSMUSG00000009621	ENSMUSG00000009621	2	27084113	27249042	35	37
1538	9	135991031	136014914	ENSMUSG00000026917	ENSMUSG00000026917	ENSMUSG00000026917	2	27216188	27358544	35	37
1539	9	135886477	135908533	ENSMUSG00000026918	ENSMUSG00000026918	ENSMUSG00000026918	2	27269788	27297201	35	37
1540	9	136358137	136472250	ENSMUSG00000015846	ENSMUSG00000015846	ENSMUSG00000015846	2	27499210	27585328	35	37
1541	9	136673473	136874560	ENSMUSG00000026837	ENSMUSG00000026837	ENSMUSG00000026837	2	27708694	27861166	35	37
1542	9	136912479	136919187	ENSMUSG00000026835	ENSMUSG00000026835	ENSMUSG00000026835	2	27898389	27906866	35	37
1543	9	137119406	137152580	ENSMUSG00000026833	ENSMUSG00000026833	ENSMUSG00000026833	2	28027698	28052745	35	37
1544	9	137527094	137531518	ENSMUSG00000026831	ENSMUSG00000026831	ENSMUSG00000026831	2	28284015	28288341	35	37
1545	9	137531651	137536340	ENSMUSG00000035772	ENSMUSG00000035772	ENSMUSG00000035772	2	28287960	28292225	35	37
1546	9	135018161	135029153	ENSMUSG00000026829	ENSMUSG00000026829	ENSMUSG00000026829	2	28319755	28327424	36	38
1547	9	134962928	134986384	ENSMUSG00000026821	ENSMUSG00000026821	ENSMUSG00000026821	2	28355560	28375090	36	38
1548	9	134927186	134937069	ENSMUSG00000026818	ENSMUSG00000026818	ENSMUSG00000026818	2	28377831	28385375	36	38
1549	9	134896220	134923707	ENSMUSG00000026816	ENSMUSG00000026816	ENSMUSG00000026816	2	28388324	28405255	36	38
1550	9	134810753	134856892	ENSMUSG00000026815	ENSMUSG00000026815	ENSMUSG00000026815	2	28431464	28443954	36	38
1551	9	134756557	134809841	ENSMUSG00000026812	ENSMUSG00000026812	ENSMUSG00000026812	2	28463246	28513176	36	38
1552	9	134743235	134755239	ENSMUSG00000026809	ENSMUSG00000026809	ENSMUSG00000026809	2	28514089	28521660	36	38
1553	9	134590788	134744019	ENSMUSG00000026807	ENSMUSG00000026807	ENSMUSG00000026807	2	28522173	28635170	36	38
1554	9	134535243	134560163	ENSMUSG00000035666	ENSMUSG00000035666	ENSMUSG00000035666	2	28647584	28662295	36	38
1555	9	134458205	134535609	ENSMUSG00000026806	ENSMUSG00000026806	ENSMUSG00000026806	2	28662746	28727579	36	38
1556	9	134447814	134455461	ENSMUSG00000026805	ENSMUSG00000026805	ENSMUSG00000026805	2	28729682	28738431	36	38
1557	9	134240756	134272042	ENSMUSG00000026803	ENSMUSG00000026803	ENSMUSG00000026803	2	28886627	28909102	36	38

1558	9	134129104	134220193	ENSMUSG00000043535	ENSMUSG00000043535	ENSMUSG00000043535	2	28948896	29003746	36	38
1559	9	134027155	134108032	ENSMUSG00000035513	ENSMUSG00000035513	ENSMUSG00000035513	2	29016830	29070049	36	38
1560	9	133441979	133575050	ENSMUSG00000039844	ENSMUSG00000039844	ENSMUSG00000039844	2	29441729	29562372	36	38
1561	9	130111203	130124842	ENSMUSG00000039826	ENSMUSG00000039826	ENSMUSG00000039826	2	29598120	29610511	37	39
1562	9	130124636	130136172	ENSMUSG00000026798	ENSMUSG00000026798	ENSMUSG00000026798	2	29610272	29619752	37	39
1563	9	130142661	130163322	ENSMUSG00000059316	ENSMUSG00000059316	ENSMUSG00000059316	2	29624689	29639531	37	39
1564	9	130173419	130192834	ENSMUSG00000069020	ENSMUSG00000069020	ENSMUSG00000069020	2	29649398	29667005	37	39
1565	9	130222935	130239447	ENSMUSG00000039787	ENSMUSG00000039787	ENSMUSG00000039787	2	29691503	29704849	37	39
1566	9	130258613	130303036	ENSMUSG00000026790	ENSMUSG00000026790	ENSMUSG00000026790	2	29711904	29753754	37	39
1567	9	130354690	130435758	ENSMUSG00000057738	ENSMUSG00000057738	ENSMUSG00000057738	2	29800389	29853102	37	39
1568	9	130435761	130458950	ENSMUSG00000039715	ENSMUSG00000039715	ENSMUSG00000039715	2	29853560	29870839	37	39
1569	9	130504623	130523019	ENSMUSG00000026785	ENSMUSG00000026785	ENSMUSG00000026785	2	29900775	29913028	37	39
1570	9	130523281	130526222	ENSMUSG00000015335	ENSMUSG00000015335	ENSMUSG00000015335	2	29912953	29915644	37	39
1571	9	130531890	130574019	ENSMUSG00000039686	ENSMUSG00000039686	ENSMUSG00000039686	2	29919292	29946463	37	39
1572	9	130589304	130612532	ENSMUSG00000039678	ENSMUSG00000039678	ENSMUSG00000039678	2	29955875	29974022	37	39
1573	9	130620574	130624777	ENSMUSG00000015337	ENSMUSG00000015337	ENSMUSG00000015337	2	29993533	29996078	37	39
1574	9	130635044	130684153	ENSMUSG00000039648	ENSMUSG00000039648	ENSMUSG00000039648	2	30007137	30027856	37	39
1575	9	130684212	130720139	ENSMUSG00000007476	ENSMUSG00000007476	ENSMUSG00000007476	2	30059731	30104149	37	39
1576	9	130747630	130749719	ENSMUSG00000075419	ENSMUSG00000075419	ENSMUSG00000075419	2	30106436	30108040	37	39
1577	9	130749798	130809195	ENSMUSG00000052533	ENSMUSG00000052533	ENSMUSG00000052533	2	30108441	30166271	37	39
1578	9	130810139	130830379	ENSMUSG00000026860	ENSMUSG00000026860	ENSMUSG00000026860	2	30166818	30181240	37	39
1579	9	130839074	130874182	ENSMUSG00000026858	ENSMUSG00000026858	ENSMUSG00000026858	2	30186281	30207525	37	39
1580	9	130883203	130892535	ENSMUSG00000026856	ENSMUSG00000026856	ENSMUSG00000026856	2	30214425	30222538	37	39
1581	9	130896896	130912904	ENSMUSG00000026853	ENSMUSG00000026853	ENSMUSG00000026853	2	30222485	30237757	37	39
1582	9	130913434	130951044	ENSMUSG00000039515	ENSMUSG00000039515	ENSMUSG00000039515	2	30238059	30269813	37	39
1583	9	131414325	131422876	ENSMUSG00000044320	ENSMUSG00000044320	ENSMUSG00000044320	2	30617572	30623716	37	39
1584	9	131410984	131438030	ENSMUSG00000026857	ENSMUSG00000026857	ENSMUSG00000026857	2	30629977	30645028	37	39
1585	9	131436704	131444265	ENSMUSG00000039483	ENSMUSG00000039483	ENSMUSG00000039483	2	30645106	30652342	37	39
1586	9	131467741	131524774	ENSMUSG00000039476	ENSMUSG00000039476	ENSMUSG00000039476	2	30667290	30703262	37	39
1587	9	131540433	131555165	ENSMUSG00000050737	ENSMUSG00000050737	ENSMUSG00000050737	2	30711480	30725306	37	39
1588	9	131605253	131613381	ENSMUSG00000026848	ENSMUSG00000026848	ENSMUSG00000026848	2	30774981	30781020	37	39
1589	9	131615043	131626234	ENSMUSG00000026849	ENSMUSG00000026849	ENSMUSG00000026849	2	30782636	30789942	37	39
1590	9	131637546	131683928	ENSMUSG00000026854	ENSMUSG00000026854	ENSMUSG00000026854	2	30804288	30845595	37	39
1591	9	131689287	131845262	ENSMUSG00000075415	ENSMUSG00000075415	ENSMUSG00000075415	2	30855034	30964015	37	39
1592	9	131855526	131942264	ENSMUSG00000000194	ENSMUSG00000000194	ENSMUSG00000000194	2	30974343	31038576	37	39
1593	9	131974678	132039404	ENSMUSG00000062661	ENSMUSG00000062661	ENSMUSG00000062661	2	31067932	31117483	37	39
1594	9	132444814	132503560	ENSMUSG00000026843	ENSMUSG00000026843	ENSMUSG00000026843	2	31394826	31439528	37	39
1595	9	132579089	132752883	ENSMUSG00000026842	ENSMUSG00000026842	ENSMUSG00000026842	2	31581989	31626236	37	39
1596	9	132758636	132759046	ENSMUSG00000043102	ENSMUSG00000043102	ENSMUSG00000043102	2	31628177	31632527	37	39
1597	9	132767648	132804058	ENSMUSG00000026841	ENSMUSG00000026841	ENSMUSG00000026841	2	31635304	31668014	37	39
1598	9	132874325	132957301	ENSMUSG00000026840	ENSMUSG00000026840	ENSMUSG00000026840	2	31709290	31768544	37	39
1599	9	132961739	132988351	ENSMUSG00000001864	ENSMUSG00000001864	ENSMUSG00000001864	2	31772312	31795451	37	39
1600	9	132990858	133098909	ENSMUSG00000001855	ENSMUSG00000001855	ENSMUSG00000001855	2	31796490	31874378	37	39
1601	9	133123284	133141755	ENSMUSG00000050592	ENSMUSG00000050592	ENSMUSG00000050592	2	31888898	31905716	37	39
1602	9	133154902	133174470	ENSMUSG00000051373	ENSMUSG00000051373	ENSMUSG00000051373	2	31917660	31932829	37	39
1603	9	133312308	133361531	ENSMUSG00000039262	ENSMUSG00000039262	ENSMUSG00000039262	2	32004521	32056517	37	39
1604	9	133368133	133389014	ENSMUSG00000039254	ENSMUSG00000039254	ENSMUSG00000039254	2	32058632	32077014	37	39
1605	9	133389009	133396408	ENSMUSG00000002550	ENSMUSG00000002550	ENSMUSG00000002550	2	32077008	32082071	37	39
1606	9	130078322	130091088	ENSMUSG00000044627	ENSMUSG00000044627	ENSMUSG00000044627	2	32100825	32110045	38	40
1607	9	130057932	130078089	ENSMUSG00000002546	ENSMUSG00000002546	ENSMUSG00000002546	2	32110335	32129921	38	40
1608	9	130005484	130057348	ENSMUSG00000026825	ENSMUSG00000026825	ENSMUSG00000026825	2	32130482	32175292	38	40
1609	9	129968173	129993689	ENSMUSG00000039205	ENSMUSG00000039205	ENSMUSG00000039205	2	32185819	32200307	38	40
1610	9	129962360	129966028	ENSMUSG00000039195	ENSMUSG00000039195	ENSMUSG00000039195	2	32199106	32203779	38	40
1611	9	129951539	129955555	ENSMUSG00000026822	ENSMUSG00000026822	ENSMUSG00000026822	2	32206645	32209748	38	40
1612	9	129922793	129930562	ENSMUSG00000026820	ENSMUSG00000026820	ENSMUSG00000026820	2	32218267	32224748	38	40
1613	9	129870300	129911345	ENSMUSG00000026819	ENSMUSG00000026819	ENSMUSG00000026819	2	32236493	32273431	38	40
1614	9	129863333	129869421	ENSMUSG00000039164	ENSMUSG00000039164	ENSMUSG00000039164	2	32272466	32277485	38	40
1615	9	129742682	129782316	ENSMUSG00000039157	ENSMUSG00000039157	ENSMUSG00000039157	2	32357341	32391759	38	40
1616	9	129738134	129739943	ENSMUSG00000026810	ENSMUSG00000026810	ENSMUSG00000026810	2	32393215	32395026	38	40
1617	9	129723950	129731979	ENSMUSG00000046854	ENSMUSG00000046854	ENSMUSG00000046854	2	32397828	32405796	38	40
1618	9	129687422	129701746	ENSMUSG00000026811	ENSMUSG00000026811	ENSMUSG00000026811	2	32430080	32442813	38	40
1619	9	129669942	129675548	ENSMUSG00000026817	ENSMUSG00000026817	ENSMUSG00000026817	2	32451571	32457488	38	40
1620	9	129617112	129656856	ENSMUSG00000026814	ENSMUSG00000026814	ENSMUSG00000026814	2	32468604	32504127	38	40
1621	9	129604975	129616377	ENSMUSG00000009566	ENSMUSG00000009566	ENSMUSG00000009566	2	32504619	32516161	38	40
1622	9	129587898	129592177	ENSMUSG00000009555	ENSMUSG00000009555	ENSMUSG00000009555	2	32529447	32534794	38	40
1623	9	129540418	129580869	ENSMUSG00000059013	ENSMUSG00000059013	ENSMUSG00000059013	2	32543081	32577003	38	40
1624	9	129533624	129537417	ENSMUSG00000009563	ENSMUSG00000009563	ENSMUSG00000009563	2	32579269	32584251	38	40
1625	9	129518166	129533700	ENSMUSG00000039021	ENSMUSG00000039021	ENSMUSG00000039021	2	32584187	32597348	38	40
1626	9	129516044	129526973	ENSMUSG00000053746	ENSMUSG00000053746	ENSMUSG00000053746	2	32597795	32611798	38	40
1627	9	129509092	129516124	ENSMUSG00000038987	ENSMUSG00000038987	ENSMUSG00000038987	2	32599390	32606437	38	40
1628	9	129414365	129494816	ENSMUSG00000026797	ENSMUSG00000026797	ENSMUSG00000026797	2	32609971	32669173	38	40
1629	9	129307443	129371188	ENSMUSG00000026796	ENSMUSG00000026796	ENSMUSG00000026796	2	32698204	32747262	38	40
1630	9	129253613	129305599	ENSMUSG00000026792	ENSMUSG00000026792	ENSMUSG00000026792	2	32747229	32783260	38	40
1631	9	129199242	129209998	ENSMUSG00000026791	ENSMUSG00000026791	ENSMUSG00000026791	2	32794997	32804081	38	40
1632	9	129066965	129195649	ENSMUSG00000038860	ENSMUSG00000038860	ENSMUSG00000038860	2	32808375	32927004	38	40
1633	9	128889432	128924983	ENSMUSG00000004105	ENSMUSG00000004105	ENSMUSG00000004105	2	33038078	33068447	38	40
1634	9	128662765	128687978	ENSMUSG00000068966	ENSMUSG00000068966	ENSMUSG00000068966	2	33228120	33250196	38	40
1635	9	128607115	128640310	ENSMUSG00000026788	ENSMUSG00000026788	ENSMUSG00000026788	2	33272296	33284362	38	40
1636	9	128416543	128498857	ENSMUSG00000038765	ENSMUSG00000038765	ENSMUSG00000038765	2	33386645	33462520	38	40
1637	9	128128949	128309140	ENSMUSG00000038740	ENSMUSG00000038740	ENSMUSG00000038740	2	33551965	33709955	38	40
1638	9	127549474	127769477	ENSMUSG00000038718	ENSMUSG00000038718	ENSMUSG00000038718	2	33993768	34193945	38	40
1639	9	127239494	127509334	ENSMUSG00000038696	ENSMUSG00000038696	ENSMUSG00000038696	2	34228887	34445923	38	40
1640	9	127101022	127165428	ENSMUSG00000026867	ENSMUSG00000026867	ENSMUSG00000026867	2	34498976	34576570	38	40
1641	9	127036953	127043430	ENSMUSG00000026864	ENSMUSG00000026864	ENSMUSG00000026864	2	34594099	34598538	38	40
1642	9	127002708	127036225	ENSMUSG00000070953	ENSMUSG00000070953	ENSMUSG00000070953	2	34600675	34621800	38	40
1643	9	122566271	122595511	ENSMUSG00000035949	ENSMUSG00000035949	ENSMUSG00000035949	2	34627189	34648197	39	41
1644	9	122618155	122645027	ENSMUSG00000026869	ENSMUSG00000026869	ENSMUSG00000026869	2	34674097	34692948	39	41
1645	9	122657798	122679298	ENSMUSG00000026873	ENSMUSG00000026873	ENSMUSG00000026873	2	34715764	34735985	39	41
1646	9	122704492	122730868	ENSMUSG00000026875	ENSMUSG00000026875	ENSMUSG00000026875	2	34765267	34780621	39	41
1647	9	122754437	122852375	ENSMUSG00000026874	ENSMUSG00000026874	ENSMUSG00000026874	2	34805340	34883405	39	41
1648	9	122877111	122979707	ENSMUSG00000057110	ENSMUSG00000057110	ENSMUSG00000057110	2	34933595	34998322	39	41
1649	9	122980236	123003968	ENSMUSG00000026878	ENSMUSG00000026878	ENSMUSG00000026878	2	35002210	35023129	39	41
1650	9	123101892	123134941	ENSMUSG00000026879	ENSMUSG00000026879	ENSMUSG00000026879	2	35104449	35129901	39	41
1651	9	123141178	123172366	ENSMUSG00000026880	ENSMUSG00000026880	ENSMUSG00000026880	2	35135999	35159018	39	41
1652	9	123249008	123560547	ENSMUSG00000035778	ENSMUSG00000035778	ENSMUSG00000035778	2	35224118	35245336	39	41
1653	9	123453796	123587630	ENSMUSG00000026883	ENSMUSG00000026883	ENSMUSG00000026883	2	35404851	35552595	39	41
1654	9	123791010	123895706	ENSMUSG00000026885	ENSMUSG00000026885	ENSMUSG00000026885	2	35573250	35801305	39	41
1655	9	123946158	123961919	ENSMUSG00000026895	ENSMUSG00000026895	ENSMUSG00000026895	2	35858343	35871318	39	41
1656	9	123962011	124002188	ENSMUSG00000026894	ENSMUSG00000026894	ENSMUSG00000026894	2	35871482	35901725	39	41
1657	9	124004679	124030900	ENSMUSG00000026890	ENSMUSG00000026890	ENSMUSG00000026890	2	35909377	35927156	39	41
1658	9	124041655	124066939	ENSMUSG00000026889	ENSMUSG00000026889	ENSMUSG00000026889	2	35938090	35958620	39	41
1659	9	124173050	124197802	ENSMUSG00000047250	ENSMUSG00000047250	ENSMUSG00000047250	2	36052509	36074278	39	41
1660	9	124279058	124280026	ENSMUSG00000075384	ENSMUSG00000075384	ENSMUSG00000075384	2	36634158	36635099	39	41
1661	9	124355270	124356262	ENSMUSG00000055088	ENSMUSG00000055088	ENSMUSG00000055088	2	36728957	36729910	39	41
1662	9	124369648	124370577	ENSMUSG00000075380	ENSMUSG00000075380	ENSMUSG00000075380	2	36749189	36750121	39	41
1663	9	124416838	124417782	ENSMUSG00000055838	ENSMUSG00000055838	ENSMUSG00000055838	2	36818821	36819747	39	41
1664	9	124430679	124431635	ENSMUSG00000075377	ENSMUSG00000075377	ENSMUSG00000075377	2	36926704	36927657	39	41
1665	9	124590970	124591995	ENSMUSG00000049018	ENSMUSG00000049018	ENSMUSG00000049018	2	37153758	37154741	39	41
1666	9	124622043	124630731	ENSMUSG00000009030	ENSMUSG00000009030	ENSMUSG00000009030	2	37172083	37181241	39	41
1667	9	124646656	124700210	ENSMUSG00000075376	ENSMUSG00000075376	ENSMUSG00000075376	2	37197067	37236823	39	41
1668	9	124717666	124733600	ENSMUSG00000050714	ENSMUSG00000050714	ENSMUSG00000050714	2	37254179	37265130	39	41
1669	9	124743109	124906966	ENSMUSG00000035437	ENSMUSG00000035437	ENSMUSG00000035437	2	37265294	37388446	39	41
1670	9	124836627	124837796	ENSMUSG00000053164	ENSMUSG00000053164	ENSMUSG00000053164	2	37338635	37341292	39	41
1671	9	124926811	125070664	ENSMUSG00000026915	ENSMUSG00000026915	ENSMUSG00000026915	2	37406039	37467700	39	41
1672	9	125158270	125180853	ENSMUSG00000035403	ENSMUSG00000035403	ENSMUSG00000035403	2	37605205	37621110	39	41
1673	9	125181754	125732238	ENSMUSG00000035392	ENSMUSG00000035392	ENSMUSG00000035392	2	37621000	38109384	39	41
1674	9	125813710	125835263	ENSMUSG00000000247	ENSMUSG00000000247	ENSMUSG00000000247	2	38173317	38191737	39	41
1675	9	126060063	126155407	ENSMUSG00000026749	ENSMUSG00000026749	ENSMUSG00000026749	2	38333709	38410050	39	41
1676	9	126283337	126309530	ENSMUSG00000026751	ENSMUSG00000026751	ENSMUSG00000026751	2	38514636	38536513	39	41
1677	9	126324516	126573219	ENSMUSG00000063972	ENSMUSG00000063972	ENSMUSG00000063972	2	38545379	38748226	39	41
1678	9	126579371	126616978	ENSMUSG00000046618	ENSMUSG00000046618	ENSMUSG00000046618	2	38753989	38782594	39	41
1679	9	126655576	126659981	ENSMUSG00000035295	ENSMUSG00000035295	ENSMUSG00000035295	2	38819485	38828177	39	41
1680	9	126664230	126679824	ENSMUSG00000026755	ENSMUSG00000026755	ENSMUSG00000026755	2	38830205	38837885	39	41
1681	9	126680467	126743199	ENSMUSG00000026754	ENSMUSG00000026754	ENSMUSG00000026754	2	38838512	38887415	39	41
1682	9	126754201	126945596	ENSMUSG00000035236	ENSMUSG00000035236	ENSMUSG00000035236	2	38897019	38955097	39	41
1683	9	126948673	126991918	ENSMUSG00000026753	ENSMUSG00000026753	ENSMUSG00000026753	2	39018807	39048348	39	41
1684	2	140707225	142604768	ENSMUSG00000049252	ENSMUSG00000049252	ENSMUSG00000049252	2	40418943	42474825	40	42
1685	2	143351537	143516309	ENSMUSG00000026866	ENSMUSG00000026866	ENSMUSG00000026866	2	43377338	43504724	40	42
1686	2	143603433	144242385	ENSMUSG00000049744	ENSMUSG00000049744	ENSMUSG00000049744	2	43570836	44209636	40	42
1687	2	144862055	144994386	ENSMUSG00000026872	ENSMUSG00000026872	ENSMUSG00000026872	2	44809127	44935025	40	42
1688	2	148319067	148404863	ENSMUSG00000052155	ENSMUSG00000052155	ENSMUSG00000052155	2	48636166	48724172	40	42
1689	2	148408202	148495606	ENSMUSG00000026761	ENSMUSG00000026761	ENSMUSG00000026761	2	48724861	48771243	40	42
1690	2	148932508	148987512	ENSMUSG00000036792	ENSMUSG00000036792	ENSMUSG00000036792	2	49067978	49139078	40	42
1691	2	149118812	149261608	ENSMUSG00000069495	ENSMUSG00000069495	ENSMUSG00000069495	2	49273495	49373957	40	42
1692	2	149502042	149591519	ENSMUSG00000026764	ENSMUSG00000026764	ENSMUSG00000026764	2	49441323	49596787	40	42
1693	2	149603227	149780018	ENSMUSG00000026765	ENSMUSG00000026765	ENSMUSG00000026765	2	49609695	49770855	40	42
1694	2	149894745	150038383	ENSMUSG00000050447	ENSMUSG00000050447	ENSMUSG00000050447	2	49888582	50015573	40	42
1695	2	150134401	150152525	ENSMUSG00000026766	ENSMUSG00000026766	ENSMUSG00000026766	2	50101890	50118726	40	42
1696	2	151032955	151052426	ENSMUSG00000017144	ENSMUSG00000017144	ENSMUSG00000017144	2	50952448	50971120	40	42
1697	2	151814156	151826623	ENSMUSG00000036249	ENSMUSG00000036249	ENSMUSG00000036249	2	51746457	51757172	40	42
1698	2	151835231	151854620	ENSMUSG00000026946	ENSMUSG00000026946	ENSMUSG00000026946	2	51770514	51795105	40	42
1699	2	151922351	151944803	ENSMUSG00000053475	ENSMUSG00000053475	ENSMUSG00000053475	2	51860185	51877980	40	42
1700	2	151974674	152040665	ENSMUSG00000036202	ENSMUSG00000036202	ENSMUSG00000036202	2	51894851	51944392	40	42
1701	2	152365726	152393255	ENSMUSG00000036093	ENSMUSG00000036093	ENSMUSG00000036093	2	52219956	52246883	40	42
1702	2	152403879	152663771	ENSMUSG00000017412	ENSMUSG00000017412	ENSMUSG00000017412	2	52250329	52498591	40	42
1703	2	152683353	152740752	ENSMUSG00000055371	ENSMUSG00000055371	ENSMUSG00000055371	2	52513673	52564209	40	42
1704	2	153221066	153282167	ENSMUSG00000061136	ENSMUSG00000061136	ENSMUSG00000061136	2	52956713	53013234	40	42
1705	2	153282674	153325937	ENSMUSG00000026960	ENSMUSG00000026960	ENSMUSG00000026960	2	53014120	53041035	40	42
1706	2	154042996	154043325	ENSMUSG00000075334	ENSMUSG00000075334	ENSMUSG00000075334	2	53900361	53900690	40	42
1707	2	154436712	155015539	ENSMUSG00000060988	ENSMUSG00000060988	ENSMUSG00000060988	2	54331926	54928347	40	42
1708	2	155263339	155421260	ENSMUSG00000026824	ENSMUSG00000026824	ENSMUSG00000026824	2	55251356	55413523	40	42
1709	2	156889194	156897474	ENSMUSG00000026826	ENSMUSG00000026826	ENSMUSG00000026826	2	56922903	56939527	40	42
1710	2	157000442	157150910	ENSMUSG00000026827	ENSMUSG00000026827	ENSMUSG00000026827	2	57053857	57184007	40	42
1711	2	157822586	157876159	ENSMUSG00000026828	ENSMUSG00000026828	ENSMUSG00000026828	2	57813270	57861246	40	42
1712	2	157883372	157890662	ENSMUSG00000026830	ENSMUSG00000026830	ENSMUSG00000026830	2	57860501	57868138	40	42
1713	2	157979379	158008850	ENSMUSG00000026832	ENSMUSG00000026832	ENSMUSG00000026832	2	57947867	57975448	40	42
1714	2	158097152	158193645	ENSMUSG00000026834	ENSMUSG00000026834	ENSMUSG00000026834	2	58090307	58131446	40	42
1715	2	158301207	158439869	ENSMUSG00000026836	ENSMUSG00000026836	ENSMUSG00000026836	2	58261827	58382212	40	42
1716	2	158666618	158700724	ENSMUSG00000026839	ENSMUSG00000026839	ENSMUSG00000026839	2	58570571	58606054	40	42
1717	2	158736725	159021460	ENSMUSG00000036641	ENSMUSG00000036641	ENSMUSG00000036641	2	58637092	58961466	40	42
1718	2	159097943	159245432	ENSMUSG00000026991	ENSMUSG00000026991	ENSMUSG00000026991	2	59015522	59156047	40	42
1719	2	159360087	159380740	ENSMUSG00000026989	ENSMUSG00000026989	ENSMUSG00000026989	2	59285496	59305722	40	42
1720	2	159630669	159797412	ENSMUSG00000035168	ENSMUSG00000035168	ENSMUSG00000035168	2	59412883	59646988	40	42
1721	2	159800618	159851482	ENSMUSG00000026988	ENSMUSG00000026988	ENSMUSG00000026988	2	59653203	59683423	40	42
1722	2	159883738	160011735	ENSMUSG00000026987	ENSMUSG00000026987	ENSMUSG00000026987	2	59700200	59807026	40	42
1723	2	160277256	160333329	ENSMUSG00000026977	ENSMUSG00000026977	ENSMUSG00000026977	2	60010726	60050050	40	42
1724	2	160333610	160362999	ENSMUSG00000060703	ENSMUSG00000060703	ENSMUSG00000060703	2	60052832	60084963	40	42
1725	2	160368122	160469493	ENSMUSG00000026980	ENSMUSG00000026980	ENSMUSG00000026980	2	60093131	60184103	40	42
1726	2	160505506	160627367	ENSMUSG00000054580	ENSMUSG00000054580	ENSMUSG00000054580	2	60219878	60354100	40	42
1727	2	160664438	160765009	ENSMUSG00000026971	ENSMUSG00000026971	ENSMUSG00000026971	2	60399131	60474500	40	42
1728	2	161701712	161800928	ENSMUSG00000064289	ENSMUSG00000064289	ENSMUSG00000064289	2	61379422	61454950	40	42
1729	2	161872795	161976472	ENSMUSG00000026914	ENSMUSG00000026914	ENSMUSG00000026914	2	61512582	61601214	40	42
1730	2	161981168	161988984	ENSMUSG00000035033	ENSMUSG00000035033	ENSMUSG00000035033	2	61605292	61614955	40	42
1731	2	162189275	162542069	ENSMUSG00000026904	ENSMUSG00000026904	ENSMUSG00000026904	2	61847301	62127569	40	42
1732	2	162557005	162639298	ENSMUSG00000035000	ENSMUSG00000035000	ENSMUSG00000035000	2	62130912	62213070	40	42
1733	2	162708779	162713934	ENSMUSG00000000394	ENSMUSG00000000394	ENSMUSG00000000394	2	62275369	62284489	40	42
1734	2	162735446	162808291	ENSMUSG00000000392	ENSMUSG00000000392	ENSMUSG00000000392	2	62301782	62374914	40	42
1735	2	162831836	162883285	ENSMUSG00000026896	ENSMUSG00000026896	ENSMUSG00000026896	2	62398289	62446999	40	42
1736	2	162936585	163403274	ENSMUSG00000059742	ENSMUSG00000059742	ENSMUSG00000059742	2	62503785	62984916	40	42
1737	2	164172364	164300759	ENSMUSG00000075324	ENSMUSG00000075324	ENSMUSG00000075324	2	63778670	63898851	40	42
1738	2	165057579	165186606	ENSMUSG00000026888	ENSMUSG00000026888	ENSMUSG00000026888	2	64713313	64823605	40	42
1739	2	165249502	165406174	ENSMUSG00000034903	ENSMUSG00000034903	ENSMUSG00000034903	2	64890151	64972768	40	42
1740	2	165463058	165520281	ENSMUSG00000061171	ENSMUSG00000061171	ENSMUSG00000061171	2	65117661	65164873	40	42
1741	2	165652286	165768799	ENSMUSG00000057182	ENSMUSG00000057182	ENSMUSG00000057182	2	65295762	65337515	40	42
1742	2	166553919	166638377	ENSMUSG00000026993	ENSMUSG00000026993	ENSMUSG00000026993	2	65502826	65530956	40	42
1743	2	166034403	166244509	ENSMUSG00000044647	ENSMUSG00000044647	ENSMUSG00000044647	2	65646681	65826445	40	42
1744	2	166311691	166335456	ENSMUSG00000026994	ENSMUSG00000026994	ENSMUSG00000026994	2	65883605	65925632	40	42
1745	2	166439088	166518594	ENSMUSG00000034848	ENSMUSG00000034848	ENSMUSG00000034848	2	65985166	66057456	40	42
1746	2	166970135	167051727	ENSMUSG00000034810	ENSMUSG00000034810	ENSMUSG00000034810	2	66476151	66585757	40	42
1747	2	167700701	167816813	ENSMUSG00000027022	ENSMUSG00000027022	ENSMUSG00000027022	2	67246841	67327445	40	42
1748	2	168383428	168435612	ENSMUSG00000034780	ENSMUSG00000034780	ENSMUSG00000034780	2	67905511	67921205	40	42
1749	2	168518777	168812191	ENSMUSG00000027030	ENSMUSG00000027030	ENSMUSG00000027030	2	68011284	68273107	40	42
1750	2	169021081	169339398	ENSMUSG00000027035	ENSMUSG00000027035	ENSMUSG00000027035	2	68662396	68912129	40	42
1751	2	169367390	169429811	ENSMUSG00000034738	ENSMUSG00000034738	ENSMUSG00000034738	2	68936662	68990166	40	42
1752	2	169435648	169455190	ENSMUSG00000005233	ENSMUSG00000005233	ENSMUSG00000005233	2	68994735	69006996	40	42

1753	2	169466088	169472835	ENSMUSG00000005232	ENSMUSG00000005232	ENSMUSG00000005232	2	69020810	69028689	40	42
1754	2	169487694	169596079	ENSMUSG00000027048	ENSMUSG00000027048	ENSMUSG00000027048	2	69039121	69143453	40	42
1755	2	169646338	169660678	ENSMUSG00000027068	ENSMUSG00000027068	ENSMUSG00000027068	2	69181301	69203925	40	42
1756	2	169693107	169927259	ENSMUSG00000027070	ENSMUSG00000027070	ENSMUSG00000027070	2	69226487	69386732	40	42
1757	2	170044239	170071410	ENSMUSG00000063145	ENSMUSG00000063145	ENSMUSG00000063145	2	69448094	69468408	40	42
1758	2	170074458	170091018	ENSMUSG00000075307	ENSMUSG00000075307	ENSMUSG00000075307	2	69471450	69484435	40	42
1759	2	170094515	170138568	ENSMUSG00000027086	ENSMUSG00000027086	ENSMUSG00000027086	2	69487654	69513445	40	42
1760	2	170149096	170202491	ENSMUSG00000042133	ENSMUSG00000042133	ENSMUSG00000042133	2	69533053	69551218	40	42
1761	2	170259247	170314560	ENSMUSG00000042155	ENSMUSG00000042155	ENSMUSG00000042155	2	69623312	69637487	40	42
1762	2	170363605	170376797	ENSMUSG00000068882	ENSMUSG00000068882	ENSMUSG00000068882	2	69662419	69672625	40	42
1763	2	170376513	170389670	ENSMUSG00000051730	ENSMUSG00000051730	ENSMUSG00000051730	2	69679929	69686196	40	42
1764	2	170493514	170647122	ENSMUSG00000044308	ENSMUSG00000044308	ENSMUSG00000044308	2	69794531	69824848	40	42
1765	2	170742901	171219229	ENSMUSG00000042064	ENSMUSG00000042064	ENSMUSG00000042064	2	69895997	70227797	40	42
1766	2	171280107	171282743	ENSMUSG00000075304	ENSMUSG00000075304	ENSMUSG00000075304	2	70275762	70278565	40	42
1767	2	171348589	171363727	ENSMUSG00000075302	ENSMUSG00000075302	ENSMUSG00000075302	2	70309658	70324211	40	42
1768	2	171381318	171425891	ENSMUSG00000070880	ENSMUSG00000070880	ENSMUSG00000070880	2	70363052	70402856	40	42
1769	2	171493957	171531878	ENSMUSG00000014959	ENSMUSG00000014959	ENSMUSG00000014959	2	70462415	70492582	40	42
1770	2	171556816	171725289	ENSMUSG00000041997	ENSMUSG00000041997	ENSMUSG00000041997	2	70513221	70626319	40	42
1771	2	171888754	171998771	ENSMUSG00000041975	ENSMUSG00000041975	ENSMUSG00000041975	2	70765637	70856366	40	42
1772	2	171999071	172049806	ENSMUSG00000041966	ENSMUSG00000041966	ENSMUSG00000041966	2	70856620	70899978	40	42
1773	2	172087229	172122883	ENSMUSG00000027015	ENSMUSG00000027015	ENSMUSG00000027015	2	70918891	70942387	40	42
1774	2	172252228	172313165	ENSMUSG00000027012	ENSMUSG00000027012	ENSMUSG00000027012	2	71012815	71064131	40	42
1775	2	172349127	172458991	ENSMUSG00000027010	ENSMUSG00000027010	ENSMUSG00000027010	2	71071902	71168588	40	42
1776	2	172487204	172556845	ENSMUSG00000027018	ENSMUSG00000027018	ENSMUSG00000027018	2	71190096	71242461	40	42
1777	2	172573116	172654009	ENSMUSG00000041921	ENSMUSG00000041921	ENSMUSG00000041921	2	71254177	71326030	40	42
1778	2	172657723	172662645	ENSMUSG00000041911	ENSMUSG00000041911	ENSMUSG00000041911	2	71330284	71334819	40	42
1779	2	172672413	172675512	ENSMUSG00000023391	ENSMUSG00000023391	ENSMUSG00000023391	2	71344248	71347580	40	42
1780	2	173000616	173079256	ENSMUSG00000027111	ENSMUSG00000027111	ENSMUSG00000027111	2	71587779	71657597	40	42
1781	2	173129025	173172108	ENSMUSG00000006494	ENSMUSG00000006494	ENSMUSG00000006494	2	71674063	71702840	40	42
1782	2	173308842	173625864	ENSMUSG00000049044	ENSMUSG00000049044	ENSMUSG00000049044	2	71782054	72058308	40	42
1783	2	173648816	173840986	ENSMUSG00000004085	ENSMUSG00000004085	ENSMUSG00000004085	2	72086605	72243269	40	42
1784	2	174481505	174538676	ENSMUSG00000027109	ENSMUSG00000027109	ENSMUSG00000027109	2	72737266	72781285	40	42
1785	2	174645421	174821611	ENSMUSG00000027108	ENSMUSG00000027108	ENSMUSG00000027108	2	72894462	73013799	40	42
1786	2	174968713	175001971	ENSMUSG00000008226	ENSMUSG00000008226	ENSMUSG00000008226	2	73113491	73138646	40	42
1787	2	175004621	175060057	ENSMUSG00000041762	ENSMUSG00000041762	ENSMUSG00000041762	2	73144299	73183083	40	42
1788	2	175133744	175255873	ENSMUSG00000075284	ENSMUSG00000075284	ENSMUSG00000075284	2	73230449	73330573	40	42
1789	2	175320853	175337416	ENSMUSG00000027107	ENSMUSG00000027107	ENSMUSG00000027107	2	73364120	73381145	40	42
1790	2	175372338	175578361	ENSMUSG00000056486	ENSMUSG00000056486	ENSMUSG00000056486	2	73413867	73522287	40	42
1791	2	175647254	175741143	ENSMUSG00000027104	ENSMUSG00000027104	ENSMUSG00000027104	2	73617360	73664083	40	42
1792	2	175751120	175757581	ENSMUSG00000018770	ENSMUSG00000018770	ENSMUSG00000018770	2	73709288	73712142	40	42
1793	2	176496896	176575268	ENSMUSG00000009207	ENSMUSG00000009207	ENSMUSG00000009207	2	74315675	74379783	40	42
1794	2	176653081	176656750	ENSMUSG00000001815	ENSMUSG00000001815	ENSMUSG00000001815	2	74456455	74460396	40	42
1795	2	176665778	176668046	ENSMUSG00000001819	ENSMUSG00000001819	ENSMUSG00000001819	2	74469149	74470975	40	42
1796	2	176672776	176673734	ENSMUSG00000001823	ENSMUSG00000001823	ENSMUSG00000001823	2	74475926	74478544	40	42
1797	2	176680330	176682562	ENSMUSG00000042499	ENSMUSG00000042499	ENSMUSG00000042499	2	74482838	74485448	40	42
1798	2	176689746	176692672	ENSMUSG00000050368	ENSMUSG00000050368	ENSMUSG00000050368	2	74492809	74495942	40	42
1799	2	176695334	176697770	ENSMUSG00000043342	ENSMUSG00000043342	ENSMUSG00000043342	2	74498600	74501047	40	42
1800	2	176702714	176704977	ENSMUSG00000027102	ENSMUSG00000027102	ENSMUSG00000027102	2	74506387	74508093	40	42
1801	2	176737051	176746072	ENSMUSG00000042464	ENSMUSG00000042464	ENSMUSG00000042464	2	74527567	74548755	40	42
1802	2	176761776	176763880	ENSMUSG00000042448	ENSMUSG00000042448	ENSMUSG00000042448	2	74563819	74565982	40	42
1803	2	176842383	176910997	ENSMUSG00000027099	ENSMUSG00000027099	ENSMUSG00000027099	2	74626690	74679270	40	42
1804	2	177803285	177965671	ENSMUSG00000015839	ENSMUSG00000015839	ENSMUSG00000015839	2	75476352	75505480	40	42
1805	2	178201060	178645728	ENSMUSG00000075270	ENSMUSG00000075270	ENSMUSG00000075270	2	75791988	76138846	40	42
1806	2	178685520	178702626	ENSMUSG00000042369	ENSMUSG00000042369	ENSMUSG00000042369	2	76170823	76184604	40	42
1807	2	178893217	178969119	ENSMUSG00000042359	ENSMUSG00000042359	ENSMUSG00000042359	2	76207377	76401486	40	42
1808	2	179004396	179024110	ENSMUSG00000002731	ENSMUSG00000002731	ENSMUSG00000002731	2	76430778	76448841	40	42
1809	2	179026383	179026598	ENSMUSG00000075267	ENSMUSG00000075267	ENSMUSG00000075267	2	76451261	76459384	40	42
1810	2	179038743	179051472	ENSMUSG00000002732	ENSMUSG00000002732	ENSMUSG00000002732	2	76463883	76473909	40	42
1811	2	179053843	179076860	ENSMUSG00000002733	ENSMUSG00000002733	ENSMUSG00000002733	2	76476134	76497412	40	42
1812	2	179112356	179377614	ENSMUSG00000051747	ENSMUSG00000051747	ENSMUSG00000051747	2	76514471	76615759	40	42
1813	2	179408737	179457923	ENSMUSG00000075261	ENSMUSG00000075261	ENSMUSG00000075261	2	76812329	76820850	40	42
1814	2	179682871	179764813	ENSMUSG00000042272	ENSMUSG00000042272	ENSMUSG00000042272	2	76987392	77091945	40	42
1815	2	180016222	180342727	ENSMUSG00000027016	ENSMUSG00000027016	ENSMUSG00000027016	2	77212764	77520578	40	42
1816	2	182029864	182110711	ENSMUSG00000027009	ENSMUSG00000027009	ENSMUSG00000027009	2	79056428	79127617	40	42
1817	2	182111156	182229978	ENSMUSG00000075256	ENSMUSG00000075256	ENSMUSG00000075256	2	79132604	79229793	40	42
1818	2	182249440	182253499	ENSMUSG00000034701	ENSMUSG00000034701	ENSMUSG00000034701	2	79253480	79257479	40	42
1819	2	182464840	182503707	ENSMUSG00000027007	ENSMUSG00000027007	ENSMUSG00000027007	2	79436379	79471673	40	42
1820	2	182558849	182702985	ENSMUSG00000034683	ENSMUSG00000034683	ENSMUSG00000034683	2	79508619	79611152	40	42
1821	2	182758769	183095348	ENSMUSG00000059173	ENSMUSG00000059173	ENSMUSG00000059173	2	79639767	79930314	40	42
1822	2	183289242	183351733	ENSMUSG00000027006	ENSMUSG00000027006	ENSMUSG00000027006	2	80118235	80151581	40	42
1823	2	183406982	183439525	ENSMUSG00000027004	ENSMUSG00000027004	ENSMUSG00000027004	2	80212809	80248235	40	42
1824	2	183497857	183611831	ENSMUSG00000027002	ENSMUSG00000027002	ENSMUSG00000027002	2	80301361	80382000	40	42
1825	2	183651765	183673616	ENSMUSG00000027001	ENSMUSG00000027001	ENSMUSG00000027001	2	80418053	80432500	40	42
1826	2	183697328	183734650	ENSMUSG00000026999	ENSMUSG00000026999	ENSMUSG00000026999	2	80439653	80459741	40	42
1827	2	185171932	185511998	ENSMUSG00000070866	ENSMUSG00000070866	ENSMUSG00000070866	2	82036637	82058155	40	42
1828	2	186319669	186365310	ENSMUSG00000043394	ENSMUSG00000043394	ENSMUSG00000043394	2	82777349	82779355	40	42
1829	2	186368159	186373391	ENSMUSG00000075249	ENSMUSG00000075249	ENSMUSG00000075249	2	82782362	82785926	40	42
1830	2	186379964	186406261	ENSMUSG00000075248	ENSMUSG00000075248	ENSMUSG00000075248	2	82792437	82794836	40	42
1831	2	187163045	187253872	ENSMUSG00000027087	ENSMUSG00000027087	ENSMUSG00000027087	2	83525354	83604646	40	42
1832	2	187267034	187335795	ENSMUSG00000048388	ENSMUSG00000048388	ENSMUSG00000048388	2	83613567	83682197	40	42
1833	2	187400452	187422142	ENSMUSG00000034552	ENSMUSG00000034552	ENSMUSG00000034552	2	83715918	83742065	40	42
1834	2	187918077	188021223	ENSMUSG00000059588	ENSMUSG00000059588	ENSMUSG00000059588	2	84131465	84177826	40	42
1835	2	188039530	188127297	ENSMUSG00000027082	ENSMUSG00000027082	ENSMUSG00000027082	2	84233699	84275013	40	42
1836	11	57285845	57343226	ENSMUSG00000034101	ENSMUSG00000034101	ENSMUSG00000034101	2	84401622	84451512	41	43
1837	11	57265298	57267459	ENSMUSG00000076437	ENSMUSG00000076437	ENSMUSG00000076437	2	84470057	84471609	41	43
1838	11	57228086	57236249	ENSMUSG00000027080	ENSMUSG00000027080	ENSMUSG00000027080	2	84479241	84489054	41	43
1839	11	57192050	57225235	ENSMUSG00000034075	ENSMUSG00000034075	ENSMUSG00000034075	2	84488759	84515958	41	43
1840	11	57181206	57185910	ENSMUSG00000027079	ENSMUSG00000027079	ENSMUSG00000027079	2	84523961	84528107	41	43
1841	11	57169137	57173993	ENSMUSG00000034059	ENSMUSG00000034059	ENSMUSG00000034059	2	84535043	84538716	41	43
1842	11	57121603	57138902	ENSMUSG00000023224	ENSMUSG00000023224	ENSMUSG00000023224	2	84566224	84576243	41	43
1843	11	57075705	57091756	ENSMUSG00000027078	ENSMUSG00000027078	ENSMUSG00000027078	2	84599678	84611015	41	43
1844	11	57052537	57054790	ENSMUSG00000027076	ENSMUSG00000027076	ENSMUSG00000027076	2	84627860	84631050	41	43
1845	11	57008586	57039735	ENSMUSG00000027075	ENSMUSG00000027075	ENSMUSG00000027075	2	84640278	84664417	41	43
1846	11	56984915	57000960	ENSMUSG00000050896	ENSMUSG00000050896	ENSMUSG00000050896	2	84672785	84687531	41	43
1847	11	56931005	56951629	ENSMUSG00000027074	ENSMUSG00000027074	ENSMUSG00000027074	2	84737499	84758795	41	43
1848	11	56910832	56914684	ENSMUSG00000027073	ENSMUSG00000027073	ENSMUSG00000027073	2	84781300	84784471	41	43
1849	11	56900819	56905199	ENSMUSG00000027072	ENSMUSG00000027072	ENSMUSG00000027072	2	84789054	84794725	41	43
1850	11	56862525	56894125	ENSMUSG00000027071	ENSMUSG00000027071	ENSMUSG00000027071	2	84797391	84836673	41	43
1851	11	56850036	56859927	ENSMUSG00000027067	ENSMUSG00000027067	ENSMUSG00000027067	2	84838755	84847945	41	43
1852	11	56823694	56848969	ENSMUSG00000033955	ENSMUSG00000033955	ENSMUSG00000033955	2	84858998	84873887	41	43
1853	11	56757630	56761364	ENSMUSG00000044338	ENSMUSG00000044338	ENSMUSG00000044338	2	84937225	84940762	41	43
1854	11	56705797	56714154	ENSMUSG00000075224	ENSMUSG00000075224	ENSMUSG00000075224	2	84992749	84997517	41	43
1855	11	56187738	56188670	ENSMUSG00000075208	ENSMUSG00000075208	ENSMUSG00000075208	2	85641696	85643075	41	43
1856	11	56165541	56166491	ENSMUSG00000046975	ENSMUSG00000046975	ENSMUSG00000046975	2	85650293	85651246	41	43
1857	11	56050619	56051572	ENSMUSG00000044923	ENSMUSG00000044923	ENSMUSG00000044923	2	85784681	85785637	41	43
1858	11	55899676	55900605	ENSMUSG00000075200	ENSMUSG00000075200	ENSMUSG00000075200	2	85971710	85972654	41	43
1859	11	55870091	55871050	ENSMUSG00000075197	ENSMUSG00000075197	ENSMUSG00000075197	2	86017597	86018547	41	43
1860	11	55700670	55701608	ENSMUSG00000047149	ENSMUSG00000047149	ENSMUSG00000047149	2	86098657	86099595	41	43
1861	11	55617360	55618292	ENSMUSG00000075166	ENSMUSG00000075166	ENSMUSG00000075166	2	86822449	86823381	41	43
1862	11	55595082	55595852	ENSMUSG00000075164	ENSMUSG00000075164	ENSMUSG00000075164	2	86849135	86850073	41	43
1863	11	55554471	55555445	ENSMUSG00000075158	ENSMUSG00000075158	ENSMUSG00000075158	2	86950560	86951498	41	43
1864	11	55459508	55460452	ENSMUSG00000068816	ENSMUSG00000068816	ENSMUSG00000068816	2	87583375	87584325	41	43
1865	11	55362804	55363790	ENSMUSG00000075145	ENSMUSG00000075145	ENSMUSG00000075145	2	87743521	87744465	41	43
1866	11	55207563	55208324	ENSMUSG00000075126	ENSMUSG00000075126	ENSMUSG00000075126	2	88212566	88213495	41	43
1867	11	48343613	48344692	ENSMUSG00000042894	ENSMUSG00000042894	ENSMUSG00000042894	2	89778619	89779551	41	43
1868	11	48303069	48304058	ENSMUSG00000075069	ENSMUSG00000075069	ENSMUSG00000075069	2	89821977	89822903	41	43
1869	11	47756246	47826633	ENSMUSG00000051329	ENSMUSG00000051329	ENSMUSG00000051329	2	90478054	90537167	41	43
1870	11	47694648	47745605	ENSMUSG00000008200	ENSMUSG00000008200	ENSMUSG00000008200	2	90546209	90581860	41	43
1871	11	47638301	47693480	ENSMUSG00000040812	ENSMUSG00000040812	ENSMUSG00000040812	2	90584865	90616352	41	43
1872	11	47567792	47572479	ENSMUSG00000040794	ENSMUSG00000040794	ENSMUSG00000040794	2	90686697	90691358	41	43
1873	11	47550326	47557101	ENSMUSG00000005505	ENSMUSG00000005505	ENSMUSG00000005505	2	90705625	90711399	41	43
1874	11	47448459	47531245	ENSMUSG00000005506	ENSMUSG00000005506	ENSMUSG00000005506	2	90741297	90817894	41	43
1875	11	47415884	47427306	ENSMUSG00000002104	ENSMUSG00000002104	ENSMUSG00000002104	2	90836466	90846567	41	43
1876	11	47396896	47404565	ENSMUSG00000002102	ENSMUSG00000002102	ENSMUSG00000002102	2	90854921	90866938	41	43
1877	11	47350830	47393490	ENSMUSG00000002105	ENSMUSG00000002105	ENSMUSG00000002105	2	90862630	90871060	41	43
1878	11	47332988	47356674	ENSMUSG00000002111	ENSMUSG00000002111	ENSMUSG00000002111	2	90897636	90916595	41	43
1879	11	47309527	47330806	ENSMUSG00000002100	ENSMUSG00000002100	ENSMUSG00000002100	2	90918983	90937350	41	43
1880	11	47247775	47308158	ENSMUSG00000040687	ENSMUSG00000040687	ENSMUSG00000040687	2	90943595	90980088	41	43
1881	11	47236106	47246972	ENSMUSG00000002108	ENSMUSG00000002108	ENSMUSG00000002108	2	90984949	90995955	41	43
1882	11	47217429	47226939	ENSMUSG00000002103	ENSMUSG00000002103	ENSMUSG00000002103	2	91003760	91013613	41	43
1883	11	47193089	47217339	ENSMUSG00000002109	ENSMUSG00000002109	ENSMUSG00000002109	2	91012442	91037726	41	43
1884	11	47155664	47164565	ENSMUSG00000027257	ENSMUSG00000027257	ENSMUSG00000027257	2	91057001	91065518	41	43
1885	11	46914827	47139765	ENSMUSG00000040591	ENSMUSG00000040591	ENSMUSG00000040591	2	91078669	91245481	41	43
1886	11	46834995	46896749	ENSMUSG00000027253	ENSMUSG00000027253	ENSMUSG00000027253	2	91258540	91314618	41	43
1887	11	46721662	46824419	ENSMUSG00000040549	ENSMUSG00000040549	ENSMUSG00000040549	2	91347180	91421504	41	43
1888	11	46697331	46717631	ENSMUSG00000027249	ENSMUSG00000027249	ENSMUSG00000027249	2	91426157	91437253	41	43
1889	11	46678944	46684037	ENSMUSG00000075040	ENSMUSG00000075040	ENSMUSG00000075040	2	91445814	91450010	41	43
1890	11	46655209	46678698	ENSMUSG00000027247	ENSMUSG00000027247	ENSMUSG00000027247	2	91451043	91473152	41	43
1891	11	46595726	46650852	ENSMUSG00000027244	ENSMUSG00000027244	ENSMUSG00000027244	2	91475464	91511415	41	43
1892	11	46581124	46595353	ENSMUSG00000027243	ENSMUSG00000027243	ENSMUSG00000027243	2	91511789	91522404	41	43
1893	11	46374540	46569490	ENSMUSG00000040506	ENSMUSG00000040506	ENSMUSG00000040506	2	91531065	91719687	41	43
1894	11	46339721	46358680	ENSMUSG00000040479	ENSMUSG00000040479	ENSMUSG00000040479	2	91733665	91764438	41	43
1895	11	46256136	46299546	ENSMUSG00000027230	ENSMUSG00000027230	ENSMUSG00000027230	2	91783169	91824984	41	43
1896	11	45911610	46099146	ENSMUSG00000058318	ENSMUSG00000058318	ENSMUSG00000058318	2	92022312	92161262	41	43
1897	11	45901010	45907223	ENSMUSG00000040434	ENSMUSG00000040434	ENSMUSG00000040434	2	92165886	92171677	41	43
1898	11	45887797	45896182	ENSMUSG00000027222	ENSMUSG00000027222	ENSMUSG00000027222	2	92176337	92182056	41	43
1899	11	45825605	45861372	ENSMUSG00000068742	ENSMUSG00000068742	ENSMUSG00000068742	2	92204485	92234867	41	43
1900	11	45783217	45791142	ENSMUSG00000049922	ENSMUSG00000049922	ENSMUSG00000049922	2	92253594	92260380	41	43
1901	11	45627003	45643748	ENSMUSG00000027221	ENSMUSG00000027221	ENSMUSG00000027221	2	92412612	92416089	41	43
1902	11	45218428	45264446	ENSMUSG00000027220	ENSMUSG00000027220	ENSMUSG00000027220	2	92715940	92756890	41	43
1903	11	45073933	45203214	ENSMUSG00000075028	ENSMUSG00000075028	ENSMUSG00000075028	2	92776027	92814722	41	43
1904	11	44881900	44929416	ENSMUSG00000068735	ENSMUSG00000068735	ENSMUSG00000068735	2	92988423	93002596	41	43
1905	11	44838451	44909450	ENSMUSG00000027217	ENSMUSG00000027217	ENSMUSG00000027217	2	93005086	93028597	41	43
1906	11	44543717	44597889	ENSMUSG00000027215	ENSMUSG00000027215	ENSMUSG00000027215	2	93219950	93263250	41	43
1907	11	44238570	44288195	ENSMUSG00000040310	ENSMUSG00000040310	ENSMUSG00000040310	2	93443303	93478775	41	43
1908	11	44073843	44223057	ENSMUSG00000027198	ENSMUSG00000027198	ENSMUSG00000027198	2	93496472	93623384	41	43
1909	11	44044394	44062145	ENSMUSG00000040272	ENSMUSG00000040272	ENSMUSG00000040272	2	93636273	93650615	41	43
1910	11	44026107	44038103	ENSMUSG00000075023	ENSMUSG00000075023	ENSMUSG00000075023	2	93656202	93669882	41	43
1911	11	43858971	43898389	ENSMUSG00000040174	ENSMUSG00000040174	ENSMUSG00000040174	2	93781474	93809360	41	43
1912	11	43658835	43834736	ENSMUSG00000027195	ENSMUSG00000027195	ENSMUSG00000027195	2	93833536	93958748	41	43
1913	11	43337081	43472030	ENSMUSG00000027194	ENSMUSG00000027194	ENSMUSG00000027194	2	94101612	94167436	41	43
1914	11	43290109	43322655	ENSMUSG00000027193	ENSMUSG00000027193	ENSMUSG00000027193	2	94212571	94238912	41	43
1915	11	40092329	40272240	ENSMUSG00000050587	ENSMUSG00000050587	ENSMUSG00000050587	2	97268496	97432503	41	43
1916	11	36572646	36637397	ENSMUSG00000027165	ENSMUSG00000027165	ENSMUSG00000027165	2	101362963	101450371	41	43
1917	11	36570071	36576362	ENSMUSG00000032864	ENSMUSG00000032864	ENSMUSG00000032864	2	101425587	101433367	41	43
1918	11	36546139	36557862	ENSMUSG00000061311	ENSMUSG00000061311	ENSMUSG00000061311	2	101442512	101445634	41	43
1919	11	36274414	36441770	ENSMUSG00000032841	ENSMUSG00000032841	ENSMUSG00000032841	2	101517089	101598503	41	43
1920	11	35922204	36210262	ENSMUSG00000048058	ENSMUSG00000048058	ENSMUSG00000048058	2	101751042	101987199	41	43
1921	11	35640929	35786333	ENSMUSG00000027189	ENSMUSG00000027189	ENSMUSG00000027189	2	102100958	102201739	41	43
1922	11	35409956	35504155	ENSMUSG00000027188	ENSMUSG00000027188	ENSMUSG00000027188	2	102350889	102443880	41	43
1923	11	35229329	35397372	ENSMUSG00000005089	ENSMUSG00000005089	ENSMUSG00000005089	2	102459499	102582993	41	43
1924	11	35116993	35210522	ENSMUSG00000005087	ENSMUSG00000005087	ENSMUSG00000005087	2	102611980	102702504	41	43
1925	11	34894695	34974092	ENSMUSG00000010914	ENSMUSG00000010914	ENSMUSG00000010914	2	102821916	102874350	41	43
1926	11	34860421	34894484	ENSMUSG00000010911	ENSMUSG00000010911	ENSMUSG00000010911	2	102874514	102893460	41	43
1927	11	34599244	34639657	ENSMUSG00000012350	ENSMUSG00000012350	ENSMUSG00000012350	2	103064272	103104035	41	43
1928	11	34456918	34489922	ENSMUSG00000027186	ENSMUSG00000027186	ENSMUSG00000027186	2	103224916	103251828	41	43
1929	11	34417054	34450176	ENSMUSG00000027187	ENSMUSG00000027187	ENSMUSG00000027187	2	103254746	103285962	41	43
1930	11	34129111	34335378	ENSMUSG00000032724	ENSMUSG00000032724	ENSMUSG00000032724	2	103367149	103519262	41	43
1931	11	34083725	34125033	ENSMUSG00000027185	ENSMUSG00000027185	ENSMUSG00000027185	2	103522098	103562089	41	43
1932	11	34029806	34080733	ENSMUSG00000027184	ENSMUSG00000027184	ENSMUSG00000027184	2	103567638	103597779	41	43
1933	11	33836701	33870412	ENSMUSG00000032698	ENSMUSG00000032698	ENSMUSG00000032698	2	103771062	103782713	41	43
1934	11	33719066	33752647	ENSMUSG00000027180	ENSMUSG00000027180	ENSMUSG00000027180	2	103828638	103864076	41	43
1935	11	33676551	33678862	ENSMUSG00000032671	ENSMUSG00000032671	ENSMUSG00000032671	2	103923608	103925585	41	43
1936	11	33521069	33646273	ENSMUSG00000032662	ENSMUSG00000032662	ENSMUSG00000032662	2	103985826	104042022	41	43
1937	11	33264531	33331690	ENSMUSG00000027177	ENSMUSG00000027177	ENSMUSG00000027177	2	104230595	104295083	41	43
1938	11	33062713	33139613	ENSMUSG00000027176	ENSMUSG00000027176	ENSMUSG00000027176	2	104391362	104466264	41	43
1939	11	33017720	33051449	ENSMUSG00000027175	ENSMUSG00000027175	ENSMUSG00000027175	2	104480991	104513001	41	43
1940	11	32994304	33011700	ENSMUSG00000027173	ENSMUSG00000027173	ENSMUSG00000027173	2	104522623	104543640	41	43
1941	11	32905277	32962349	ENSMUSG00000074994	ENSMUSG00000074994	ENSMUSG00000074994	2	104587915	104588511	41	43
1942	11	32808065	32832679	ENSMUSG00000027171	ENSMUSG00000027171	ENSMUSG00000027171	2	104631580	104650513	41	43
1943	11	32580368	32772763	ENSMUSG00000045106	ENSMUSG00000045106	ENSMUSG00000045106	2	104752743	104800576	41	43
1944	11	32561935	32580588	ENSMUSG00000027170	ENSMUSG00000027170	ENSMUSG00000027170	2	104800498	104817884	41	43
1945	11	32365901	32413663	ENSMUSG00000016458	ENSMUSG00000016458	ENSMUSG00000016458	2	104927368	104974451	41	43
1946	11	32069026	32083649	ENSMUSG00000005973	ENSMUSG00000005973	ENSMUSG00000005973	2	105187772	105200078	41	43
1947	11	31767034	31796085	ENSMUSG00000027168	ENSMUSG00000027168	ENSMUSG00000027168	2	105480671	105497243	41	43

1948	11	31487901	31761885	ENSMUSG00000027167	ENSMUSG00000027167	ENSMUSG00000027167	2	105500948	105705339	41	43
1949	11	31348010	31410955	ENSMUSG00000027166	ENSMUSG00000027166	ENSMUSG00000027166	2	105767556	105804388	41	43
1950	11	30877702	30910027	ENSMUSG00000074981	ENSMUSG00000074981	ENSMUSG00000074981	2	106136850	106178750	41	43
1951	11	30388907	30565113	ENSMUSG00000016386	ENSMUSG00000016386	ENSMUSG00000016386	2	106496440	106668043	41	43
1952	11	30301232	30316344	ENSMUSG00000027122	ENSMUSG00000027122	ENSMUSG00000027122	2	106763368	106775236	41	43
1953	11	30209139	30213384	ENSMUSG00000027120	ENSMUSG00000027120	ENSMUSG00000027120	2	106856973	106860490	41	43
1954	11	29988341	29995064	ENSMUSG00000042604	ENSMUSG00000042604	ENSMUSG00000042604	2	107091493	107099343	41	43
1955	11	27998742	28086252	ENSMUSG00000027115	ENSMUSG00000027115	ENSMUSG00000027115	2	109081577	109142588	41	43
1956	11	27633022	27699872	ENSMUSG00000048482	ENSMUSG00000048482	ENSMUSG00000048482	2	109475539	109527845	41	43
1957	11	27344086	27450869	ENSMUSG00000050199	ENSMUSG00000050199	ENSMUSG00000050199	2	109771564	109813763	41	43
1958	11	27316944	27341371	ENSMUSG00000027160	ENSMUSG00000027160	ENSMUSG00000027160	2	109818687	109845240	41	43
1959	11	27019574	27105930	ENSMUSG00000041660	ENSMUSG00000041660	ENSMUSG00000041660	2	110063536	110106582	41	43
1960	11	26972204	26975206	ENSMUSG00000074971	ENSMUSG00000074971	ENSMUSG00000074971	2	110162981	110163634	41	43
1961	11	26669783	26700122	ENSMUSG00000041644	ENSMUSG00000041644	ENSMUSG00000041644	2	110398146	110448618	41	43
1962	11	26537156	26550244	ENSMUSG00000050808	ENSMUSG00000050808	ENSMUSG00000050808	2	110522447	110538802	41	43
1963	11	26309547	26641411	ENSMUSG00000074968	ENSMUSG00000074968	ENSMUSG00000074968	2	110539605	110595889	41	43
1964	15	100175913	100176851	ENSMUSG00000074951	ENSMUSG00000074951	ENSMUSG00000074951	2	111783973	111784935	42	44
1965	15	100163446	100164384	ENSMUSG00000074949	ENSMUSG00000074949	ENSMUSG00000074949	2	111809085	111810023	42	44
1966	15	32438419	32446707	ENSMUSG00000027134	ENSMUSG00000027134	ENSMUSG00000027134	2	112040680	112047950	43	45
1967	15	32425358	32437225	ENSMUSG00000041358	ENSMUSG00000041358	ENSMUSG00000041358	2	112048787	112060130	43	45
1968	15	32421217	32422654	ENSMUSG00000027133	ENSMUSG00000027133	ENSMUSG00000027133	2	112062808	112063739	43	45
1969	15	32312755	32417249	ENSMUSG00000027130	ENSMUSG00000027130	ENSMUSG00000027130	2	112067658	112163995	43	45
1970	15	32304511	32309642	ENSMUSG00000027131	ENSMUSG00000027131	ENSMUSG00000027131	2	112163848	112168866	43	45
1971	15	32220168	32289515	ENSMUSG00000027132	ENSMUSG00000027132	ENSMUSG00000027132	2	112180050	112215076	43	45
1972	15	32163513	32181362	ENSMUSG00000055943	ENSMUSG00000055943	ENSMUSG00000055943	2	112255883	112270037	43	45
1973	15	32142129	32144579	ENSMUSG00000074939	ENSMUSG00000074939	ENSMUSG00000074939	2	112280010	112281608	43	45
1974	15	31945721	32118595	ENSMUSG00000003604	ENSMUSG00000003604	ENSMUSG00000003604	2	112293830	112431879	43	45
1975	15	31545225	31945594	ENSMUSG00000057378	ENSMUSG00000057378	ENSMUSG00000057378	2	112432965	113017829	43	45
1976	15	30846106	31146321	ENSMUSG00000044042	ENSMUSG00000044042	ENSMUSG00000044042	2	113128621	113510694	43	45
1977	15	30797497	30814158	ENSMUSG00000074934	ENSMUSG00000074934	ENSMUSG00000074934	2	113549514	113559485	43	45
1978	15	30721223	30776584	ENSMUSG00000023236	ENSMUSG00000023236	ENSMUSG00000023236	2	113577206	113629888	43	45
1979	15	32831971	32833981	ENSMUSG00000068615	ENSMUSG00000068615	ENSMUSG00000068615	2	113701037	113705060	44	46
1980	15	32869724	32875181	ENSMUSG00000068614	ENSMUSG00000068614	ENSMUSG00000068614	2	113738727	113744252	44	46
1981	15	32935845	33049287	ENSMUSG00000040383	ENSMUSG00000040383	ENSMUSG00000040383	2	113792611	113866780	44	46
1982	15	33057839	33067761	ENSMUSG00000040321	ENSMUSG00000040321	ENSMUSG00000040321	2	113884904	113892873	44	46
1983	15	33450430	33622020	ENSMUSG00000057147	ENSMUSG00000057147	ENSMUSG00000057147	2	114207861	114346405	44	46
1984	15	34771180	34889724	ENSMUSG00000040282	ENSMUSG00000040282	ENSMUSG00000040282	2	115273217	115470209	44	46
1985	15	34970525	35179996	ENSMUSG00000027210	ENSMUSG00000027210	ENSMUSG00000027210	2	115555668	115754861	44	46
1986	15	36014749	36030912	ENSMUSG00000027355	ENSMUSG00000027355	ENSMUSG00000027355	2	116570149	116583935	44	46
1987	15	36331808	36433526	ENSMUSG00000027351	ENSMUSG00000027351	ENSMUSG00000027351	2	116812891	116870782	44	46
1988	15	36533647	36563158	ENSMUSG00000027349	ENSMUSG00000027349	ENSMUSG00000027349	2	116941180	116962981	44	46
1989	15	36567590	36644224	ENSMUSG00000027347	ENSMUSG00000027347	ENSMUSG00000027347	2	116971431	117034318	44	46
1990	15	37660572	37676957	ENSMUSG00000040152	ENSMUSG00000040152	ENSMUSG00000040152	2	117804084	117815705	44	46
1991	15	37679528	37862323	ENSMUSG00000027344	ENSMUSG00000027344	ENSMUSG00000027344	2	117896333	117948407	44	46
1992	15	37880223	38000385	ENSMUSG00000040133	ENSMUSG00000040133	ENSMUSG00000040133	2	117968551	118064752	44	46
1993	15	38013639	38115089	ENSMUSG00000005102	ENSMUSG00000005102	ENSMUSG00000005102	2	118080058	118166674	44	46
1994	15	38115255	38118622	ENSMUSG00000009549	ENSMUSG00000009549	ENSMUSG00000009549	2	118167292	118171137	44	46
1995	15	38167386	38188367	ENSMUSG00000040093	ENSMUSG00000040093	ENSMUSG00000040093	2	118220198	118241119	44	46
1996	15	38319361	38356979	ENSMUSG00000074923	ENSMUSG00000074923	ENSMUSG00000074923	2	118355211	118389459	44	46
1997	15	38367402	38387330	ENSMUSG00000040061	ENSMUSG00000040061	ENSMUSG00000040061	2	118398958	118419879	44	46
1998	15	38403549	38403764	ENSMUSG00000074918	ENSMUSG00000074918	ENSMUSG00000074918	2	118437556	118437771	44	46
1999	15	38411007	38420453	ENSMUSG00000045838	ENSMUSG00000045838	ENSMUSG00000045838	2	118445597	118454093	44	46
2000	15	38437723	38450548	ENSMUSG00000040035	ENSMUSG00000040035	ENSMUSG00000040035	2	118471359	118486616	44	46
2001	15	38462316	38473180	ENSMUSG00000027331	ENSMUSG00000027331	ENSMUSG00000027331	2	118505444	118526080	44	46
2002	15	38484978	38498355	ENSMUSG00000027332	ENSMUSG00000027332	ENSMUSG00000027332	2	118553423	118573288	44	46
2003	15	38520703	38547718	ENSMUSG00000040007	ENSMUSG00000040007	ENSMUSG00000040007	2	118592894	118615963	44	46
2004	15	38550489	38552645	ENSMUSG00000074916	ENSMUSG00000074916	ENSMUSG00000074916	2	118617937	118620026	44	46
2005	15	38610832	38644271	ENSMUSG00000039983	ENSMUSG00000039983	ENSMUSG00000039983	2	118709603	118720827	44	46
2006	15	38648823	38654183	ENSMUSG00000027324	ENSMUSG00000027324	ENSMUSG00000027324	2	118726231	118731210	44	46
2007	15	38673755	38734467	ENSMUSG00000027326	ENSMUSG00000027326	ENSMUSG00000027326	2	118738591	118764357	44	46
2008	15	38774661	38811646	ENSMUSG00000027323	ENSMUSG00000027323	ENSMUSG00000027323	2	118804258	118827511	44	46
2009	15	38815378	38834772	ENSMUSG00000070730	ENSMUSG00000070730	ENSMUSG00000070730	2	118828442	118848399	44	46
2010	15	38843558	38847198	ENSMUSG00000046814	ENSMUSG00000046814	ENSMUSG00000046814	2	118859229	118863830	44	46
2011	15	38847363	38886954	ENSMUSG00000034278	ENSMUSG00000034278	ENSMUSG00000034278	2	118863948	118900215	44	46
2012	15	38849470	38851935	ENSMUSG00000055926	ENSMUSG00000055926	ENSMUSG00000055926	2	118865950	118869005	44	46
2013	15	38886724	38894059	ENSMUSG00000068580	ENSMUSG00000068580	ENSMUSG00000068580	2	118900571	118908490	44	46
2014	15	38894943	38908227	ENSMUSG00000027317	ENSMUSG00000027317	ENSMUSG00000027317	2	118909562	118921293	44	46
2015	15	38923538	38937144	ENSMUSG00000027315	ENSMUSG00000027315	ENSMUSG00000027315	2	118928846	118940947	44	46
2016	15	38951733	38953779	ENSMUSG00000034226	ENSMUSG00000034226	ENSMUSG00000034226	2	118960642	118962670	44	46
2017	15	38973920	38983464	ENSMUSG00000034216	ENSMUSG00000034216	ENSMUSG00000034216	2	118980198	118989894	44	46
2018	15	39009159	39017019	ENSMUSG00000027314	ENSMUSG00000027314	ENSMUSG00000027314	2	119017225	119027403	44	46
2019	15	39032972	39036001	ENSMUSG00000027313	ENSMUSG00000027313	ENSMUSG00000027313	2	119042697	119045822	44	46
2020	15	39058373	39195632	ENSMUSG00000034154	ENSMUSG00000034154	ENSMUSG00000034154	2	119064478	119149709	44	46
2021	15	39262225	39310187	ENSMUSG00000048647	ENSMUSG00000048647	ENSMUSG00000048647	2	119210845	119239068	44	46
2022	15	39310729	39361369	ENSMUSG00000014077	ENSMUSG00000014077	ENSMUSG00000014077	2	119239155	119278461	44	46
2023	15	39583132	39593369	ENSMUSG00000027297	ENSMUSG00000027297	ENSMUSG00000027297	2	119307693	119451872	44	46
2024	15	39412361	39460535	ENSMUSG00000027306	ENSMUSG00000027306	ENSMUSG00000027306	2	119309739	119341220	44	46
2025	15	39466845	39481934	ENSMUSG00000027305	ENSMUSG00000027305	ENSMUSG00000027305	2	119346892	119354239	44	46
2026	15	39496608	39563043	ENSMUSG00000027304	ENSMUSG00000027304	ENSMUSG00000027304	2	119366525	119402466	44	46
2027	15	39573365	39583035	ENSMUSG00000027296	ENSMUSG00000027296	ENSMUSG00000027296	2	119433778	119442694	44	46
2028	15	39596669	39623756	ENSMUSG00000034032	ENSMUSG00000034032	ENSMUSG00000034032	2	119455405	119478941	44	46
2029	15	39638524	39658818	ENSMUSG00000027298	ENSMUSG00000027298	ENSMUSG00000027298	2	119490955	119509545	44	46
2030	15	39748385	39846767	ENSMUSG00000033943	ENSMUSG00000033943	ENSMUSG00000033943	2	119594114	119656406	44	46
2031	15	39853974	39906644	ENSMUSG00000033902	ENSMUSG00000033902	ENSMUSG00000033902	2	119664344	119716780	44	46
2032	15	39907606	39927633	ENSMUSG00000033852	ENSMUSG00000033852	ENSMUSG00000033852	2	119723326	119734471	44	46
2033	15	39978933	40052038	ENSMUSG00000027293	ENSMUSG00000027293	ENSMUSG00000027293	2	119780928	119846016	44	46
2034	15	40061074	40077012	ENSMUSG00000050211	ENSMUSG00000050211	ENSMUSG00000050211	2	119857853	119936776	44	46
2035	15	40147173	40174044	ENSMUSG00000070719	ENSMUSG00000070719	ENSMUSG00000070719	2	119957307	119980510	44	46
2036	15	40220691	40236102	ENSMUSG00000046971	ENSMUSG00000046971	ENSMUSG00000046971	2	119991407	120005606	44	46
2037	15	40240244	40287643	ENSMUSG00000027291	ENSMUSG00000027291	ENSMUSG00000027291	2	120008040	120044554	44	46
2038	15	40290041	40352923	ENSMUSG00000033808	ENSMUSG00000033808	ENSMUSG00000033808	2	120046753	120095530	44	46
2039	15	40353658	40433155	ENSMUSG00000062646	ENSMUSG00000062646	ENSMUSG00000062646	2	120096421	120152291	44	46
2040	15	40492314	40537022	ENSMUSG00000027288	ENSMUSG00000027288	ENSMUSG00000027288	2	120201214	120246725	44	46
2041	15	40575127	40612548	ENSMUSG00000027287	ENSMUSG00000027287	ENSMUSG00000027287	2	120259138	120291874	44	46
2042	15	40622014	40628292	ENSMUSG00000027286	ENSMUSG00000027286	ENSMUSG00000027286	2	120296323	120300794	44	46
2043	15	40628345	40647334	ENSMUSG00000027285	ENSMUSG00000027285	ENSMUSG00000027285	2	120300877	120312261	44	46
2044	15	40655149	40752587	ENSMUSG00000027279	ENSMUSG00000027279	ENSMUSG00000027279	2	120356321	120369421	44	46
2045	15	40764306	40766717	ENSMUSG00000074894	ENSMUSG00000074894	ENSMUSG00000074894	2	120384796	120390911	44	46
2046	15	40766990	40800348	ENSMUSG00000033705	ENSMUSG00000033705	ENSMUSG00000033705	2	120393983	120407523	44	46
2047	15	40825025	41000204	ENSMUSG00000027284	ENSMUSG00000027284	ENSMUSG00000027284	2	120430707	120542045	44	46
2048	15	41024819	41185512	ENSMUSG00000027272	ENSMUSG00000027272	ENSMUSG00000027272	2	120553087	120662156	44	46
2049	15	41213014	41264625	ENSMUSG00000054484	ENSMUSG00000054484	ENSMUSG00000054484	2	120668503	120699293	44	46
2050	15	41264793	41274680	ENSMUSG00000023572	ENSMUSG00000023572	ENSMUSG00000023572	2	120699951	120706172	44	46
2051	15	41276720	41300773	ENSMUSG00000023216	ENSMUSG00000023216	ENSMUSG00000023216	2	120709332	120728513	44	46
2052	15	41312648	41346347	ENSMUSG00000053675	ENSMUSG00000053675	ENSMUSG00000053675	2	120737552	120777282	44	46
2053	15	41407919	41409979	ENSMUSG00000074890	ENSMUSG00000074890	ENSMUSG00000074890	2	120829981	120832041	44	46
2054	15	41414601	41431429	ENSMUSG00000027259	ENSMUSG00000027259	ENSMUSG00000027259	2	120833582	120848109	44	46
2055	15	41440508	41449538	ENSMUSG00000050619	ENSMUSG00000050619	ENSMUSG00000050619	2	120857483	120861565	44	46
2056	15	41450585	41485530	ENSMUSG00000027263	ENSMUSG00000027263	ENSMUSG00000027263	2	120862682	120890210	44	46
2057	15	41486699	41590028	ENSMUSG00000043909	ENSMUSG00000043909	ENSMUSG00000043909	2	120889692	120962147	44	46
2058	15	41590448	41611110	ENSMUSG00000027254	ENSMUSG00000027254	ENSMUSG00000027254	2	120981076	120999525	44	46
2059	15	41612952	41728419	ENSMUSG00000033526	ENSMUSG00000033526	ENSMUSG00000033526	2	121002004	121042458	44	46
2060	15	41710052	41727551	ENSMUSG00000033486	ENSMUSG00000033486	ENSMUSG00000033486	2	121085800	121104976	44	46
2061	15	41825882	41851035	ENSMUSG00000027248	ENSMUSG00000027248	ENSMUSG00000027248	2	121105386	121129419	44	46
2062	15	41852095	41857033	ENSMUSG00000027246	ENSMUSG00000027246	ENSMUSG00000027246	2	121130468	121134042	44	46
2063	15	41873652	41879711	ENSMUSG00000046110	ENSMUSG00000046110	ENSMUSG00000046110	2	121142621	121148222	44	46
2064	15	41871844	41881362	ENSMUSG00000027245	ENSMUSG00000027245	ENSMUSG00000027245	2	121148537	121149948	44	46
2065	15	41906453	41947909	ENSMUSG00000027242	ENSMUSG00000027242	ENSMUSG00000027242	2	121198224	121236298	44	46
2066	15	41950254	42274721	ENSMUSG00000027238	ENSMUSG00000027238	ENSMUSG00000027238	2	121239390	121498272	44	46
2067	15	42368221	42495247	ENSMUSG00000060227	ENSMUSG00000060227	ENSMUSG00000060227	2	121558411	121627348	44	46
2068	15	42745910	42755092	ENSMUSG00000027233	ENSMUSG00000027233	ENSMUSG00000027233	2	121811549	121877630	44	46
2069	15	42790977	42797649	ENSMUSG00000060802	ENSMUSG00000060802	ENSMUSG00000060802	2	121839127	121844524	44	46
2070	15	42816015	42847317	ENSMUSG00000033368	ENSMUSG00000033368	ENSMUSG00000033368	2	121852141	121870468	44	46
2071	15	43036192	43058711	ENSMUSG00000027229	ENSMUSG00000027229	ENSMUSG00000027229	2	121877713	121897834	44	46
2072	15	43102637	43154330	ENSMUSG00000027227	ENSMUSG00000027227	ENSMUSG00000027227	2	121926280	121956778	44	46
2073	15	43172145	43192901	ENSMUSG00000068452	ENSMUSG00000068452	ENSMUSG00000068452	2	121974488	121989606	44	46
2074	15	43193964	43197591	ENSMUSG00000027225	ENSMUSG00000027225	ENSMUSG00000027225	2	121990341	121994326	44	46
2075	15	43196980	43209349	ENSMUSG00000027224	ENSMUSG00000027224	ENSMUSG00000027224	2	121995186	121998267	44	46
2076	15	43209484	43245066	ENSMUSG00000033268	ENSMUSG00000033268	ENSMUSG00000033268	2	122009371	122038830	44	46
2077	15	43246703	43280665	ENSMUSG00000033256	ENSMUSG00000033256	ENSMUSG00000033256	2	122040333	122058269	44	46
2078	15	43331726	43355425	ENSMUSG00000027219	ENSMUSG00000027219	ENSMUSG00000027219	2	122131284	122152571	44	46
2079	15	43440616	43457943	ENSMUSG00000027199	ENSMUSG00000027199	ENSMUSG00000027199	2	122285913	122302709	44	46
2080	15	43481871	43500903	ENSMUSG00000074876	ENSMUSG00000074876	ENSMUSG00000074876	2	122322127	122324151	44	46
2081	15	43510055	43512937	ENSMUSG00000033213	ENSMUSG00000033213	ENSMUSG00000033213	2	122329328	122332528	44	46
2082	15	43561974	43602294	ENSMUSG00000005802	ENSMUSG00000005802	ENSMUSG00000005802	2	122372680	122394104	44	46
2083	15	43666709	43689200	ENSMUSG00000005804	ENSMUSG00000005804	ENSMUSG00000005804	2	122429965	122439539	44	46
2084	15	43714501	43770747	ENSMUSG00000005803	ENSMUSG00000005803	ENSMUSG00000005803	2	122456800	122500986	44	46
2085	15	45797978	45853709	ENSMUSG00000027200	ENSMUSG00000027200	ENSMUSG00000027200	2	124345076	124356750	44	46
2086	15	46200461	46221881	ENSMUSG00000035183	ENSMUSG00000035183	ENSMUSG00000035183	2	124759568	124780118	44	46
2087	15	46287190	46382417	ENSMUSG00000027202	ENSMUSG00000027202	ENSMUSG00000027202	2	124844041	124921440	44	46
2088	15	46410913	46422862	ENSMUSG00000027203	ENSMUSG00000027203	ENSMUSG00000027203	2	124938699	124949170	44	46
2089	15	46489479	46724391	ENSMUSG00000027204	ENSMUSG00000027204	ENSMUSG00000027204	2	124992035	125197826	44	46
2090	15	46817428	46890562	ENSMUSG00000068394	ENSMUSG00000068394	ENSMUSG00000068394	2	125254531	125316554	44	46
2091	15	46903227	47042933	ENSMUSG00000035109	ENSMUSG00000035109	ENSMUSG00000035109	2	125318888	125415589	44	46
2092	15	47071619	47125987	ENSMUSG00000035093	ENSMUSG00000035093	ENSMUSG00000035093	2	125431706	125474311	44	46
2093	15	47206842	47235072	ENSMUSG00000027206	ENSMUSG00000027206	ENSMUSG00000027206	2	125521745	125550459	44	46
2094	15	47235268	47409294	ENSMUSG00000027207	ENSMUSG00000027207	ENSMUSG00000027207	2	125557694	125675026	44	46
2095	15	47407886	47700407	ENSMUSG00000027209	ENSMUSG00000027209	ENSMUSG00000027209	2	125694592	125843425	44	46
2096	15	47700576	47723151	ENSMUSG00000023330	ENSMUSG00000023330	ENSMUSG00000023330	2	125843635	125856717	44	46
2097	15	47937727	48198711	ENSMUSG00000060131	ENSMUSG00000060131	ENSMUSG00000060131	2	126014254	126172134	44	46
2098	15	48261716	48315873	ENSMUSG00000027359	ENSMUSG00000027359	ENSMUSG00000027359	2	126244496	126279682	44	46
2099	15	48321443	48345214	ENSMUSG00000027360	ENSMUSG00000027360	ENSMUSG00000027360	2	126285109	126310106	44	46
2100	15	48356681	48434687	ENSMUSG00000027361	ENSMUSG00000027361	ENSMUSG00000027361	2	126321671	126350018	44	46
2101	15	48503871	48579255	ENSMUSG00000027363	ENSMUSG00000027363	ENSMUSG00000027363	2	126398804	126450738	44	46
2102	15	48580258	48626014	ENSMUSG00000027364	ENSMUSG00000027364	ENSMUSG00000027364	2	126452863	126474885	44	46
2103	15	48639785	48766287	ENSMUSG00000027365	ENSMUSG00000027365	ENSMUSG00000027365	2	126483006	126567671	44	46
2104	15	48787057	48845191	ENSMUSG00000027366	ENSMUSG00000027366	ENSMUSG00000027366	2	126585573	126624676	44	46
2105	15	48988238	49085389	ENSMUSG00000001998	ENSMUSG00000001998	ENSMUSG00000001998	2	126700171	126758926	44	46
2106	2	96365211	96405001	ENSMUSG00000034906	ENSMUSG00000034906	ENSMUSG00000034906	2	126795272	126825384	45	47
2107	2	96355688	96357806	ENSMUSG00000074825	ENSMUSG00000074825	ENSMUSG00000074825	2	126825538	126833539	45	47
2108	2	96303804	96334988	ENSMUSG00000003660	ENSMUSG00000003660	ENSMUSG00000003660	2	126899840	126931892	45	47
2109	2	96295611	96303619	ENSMUSG00000003662	ENSMUSG00000003662	ENSMUSG00000003662	2	126932379	126939183	45	47
2110	2	96280041	96295459	ENSMUSG00000034850	ENSMUSG00000034850	ENSMUSG00000034850	2	126939416	126952205	45	47
2111	2	96214334	96238290	ENSMUSG00000027367	ENSMUSG00000027367	ENSMUSG00000027367	2	126961680	126990381	45	47
2112	2	96172644	96174906	ENSMUSG00000027368	ENSMUSG00000027368	ENSMUSG00000027368	2	127027600	127029818	45	47
2113	2	96153316	96167902	ENSMUSG00000050468	ENSMUSG00000050468	ENSMUSG00000050468	2	127033085	127049090	45	47
2114	2	96051422	96064390	ENSMUSG00000046338	ENSMUSG00000046338	ENSMUSG00000046338	2	127116640	127127531	45	47
2115	2	95303928	95320781	ENSMUSG00000027376	ENSMUSG00000027376	ENSMUSG00000027376	2	127217914	127232838	45	47
2116	2	95194910	95213792	ENSMUSG00000034800	ENSMUSG00000034800	ENSMUSG00000034800	2	127266981	127272949	45	47
2117	2	95116680	95151481	ENSMUSG00000027374	ENSMUSG00000027374	ENSMUSG00000027374	2	127278860	127295963	45	47
2118	2	95055156	95083462	ENSMUSG00000027375	ENSMUSG00000027375	ENSMUSG00000027375	2	127324671	127347724	45	47
2119	2	110198738	110231432	ENSMUSG00000027377	ENSMUSG00000027377	ENSMUSG00000027377	2	127395831	127421338	46	48
2120	2	110237195	110319883	ENSMUSG00000027378	ENSMUSG00000027378	ENSMUSG00000027378	2	127432173	127480295	46	48
2121	2	110327221	110327637	ENSMUSG00000051319	ENSMUSG00000051319	ENSMUSG00000051319	2	127482829	127483929	46	48
2122	2	111111883	111152135	ENSMUSG00000027379	ENSMUSG00000027379	ENSMUSG00000027379	2	127492567	127523301	46	48
2123	2	111247895	111591849	ENSMUSG00000027380	ENSMUSG00000027380	ENSMUSG00000027380	2	127546069	127815314	46	48
2124	2	111597794	111641054	ENSMUSG00000027381	ENSMUSG00000027381	ENSMUSG00000027381	2	127820074	127853613	46	48
2125	2	112372662	112503413	ENSMUSG00000014361	ENSMUSG00000014361	ENSMUSG00000014361	2	128390438	128493629	46	48
2126	2	112529285	112591101	ENSMUSG00000014353	ENSMUSG00000014353	ENSMUSG00000014353	2	128509752	128543233	46	48
2127	2	112612433	112662262	ENSMUSG00000027386	ENSMUSG00000027386	ENSMUSG00000027386	2	128555419	128587244	46	48
2128	2	112689913	112729121	ENSMUSG00000027387	ENSMUSG00000027387	ENSMUSG00000027387	2	128617709	128635518	46	48
2129	2	112773895	112808295	ENSMUSG00000042851	ENSMUSG00000042851	ENSMUSG00000042851	2	128682530	128710006	46	48
2130	2	112956369	113006452	ENSMUSG00000027394	ENSMUSG00000027394	ENSMUSG00000027394	2	128757425	128785196	46	48
2131	2	113016084	113051879	ENSMUSG00000027395	ENSMUSG00000027395	ENSMUSG00000027395	2	128792436	128818035	46	48
2132	2	113119998	113137867	ENSMUSG00000027397	ENSMUSG00000027397	ENSMUSG00000027397	2	128890241	128903050	46	48
2133	2	113211915	113238665	ENSMUSG00000048327	ENSMUSG00000048327	ENSMUSG00000048327	2	128960322	128988605	46	48
2134	2	113247966	113259442	ENSMUSG00000027399	ENSMUSG00000027399	ENSMUSG00000027399	2	128991051	129001413	46	48
2135	2	113303808	113310827	ENSMUSG00000027398	ENSMUSG00000027398	ENSMUSG00000027398	2	129056011	129062561	46	48
2136	20	1907403	1922702	ENSMUSG00000027400	ENSMUSG00000027400	ENSMUSG00000027400	2	129378007	129391274	47	49
2137	20	2030257	2077201	ENSMUSG00000037885	ENSMUSG00000037885	ENSMUSG00000037885	2	129492193	129519428	47	49
2138	20	2224647	2269724	ENSMUSG00000027401	ENSMUSG00000027401	ENSMUSG00000027401	2	129703790	129740002	47	49
2139	20	2309554	2361399	ENSMUSG00000027403	ENSMUSG00000027403	ENSMUSG00000027403	2	129814726	129845673	47	49
2140	20	2390402	2399499	ENSMUSG00000027404	ENSMUSG00000027404	ENSMUSG00000027404	2	129863076	129870844	47	49
2141	20	2465253	2570430	ENSMUSG00000060332	ENSMUSG00000060332	ENSMUSG00000060332	2	129886635	129955886	47	49
2142	20	2580791	2587039	ENSMUSG00000027405	ENSMUSG00000027405	ENSMUSG00000027405	2	129965871	129970754	47	49

2143	20	2587040	2592835	ENSMUSG00000027406	ENSMUSG00000027406	ENSMUSG00000027406	2	129970756	129975874	47	49
2144	20	2621480	2688747	ENSMUSG00000053552	ENSMUSG00000053552	ENSMUSG00000053552	2	129987380	130060949	47	49
2145	20	2722715	2729282	ENSMUSG00000027408	ENSMUSG00000027408	ENSMUSG00000027408	2	130082216	130089015	47	49
2146	20	2743614	2746712	ENSMUSG00000027409	ENSMUSG00000027409	ENSMUSG00000027409	2	130096737	130097514	47	49
2147	20	2763971	2769332	ENSMUSG00000037773	ENSMUSG00000037773	ENSMUSG00000037773	2	130109665	130114976	47	49
2148	20	2769366	2795378	ENSMUSG00000027411	ENSMUSG00000027411	ENSMUSG00000027411	2	130115806	130135171	47	49
2149	20	2974675	2976896	ENSMUSG00000037740	ENSMUSG00000037740	ENSMUSG00000037740	2	130255198	130256835	47	49
2150	20	3000266	3001162	ENSMUSG00000027301	ENSMUSG00000027301	ENSMUSG00000027301	2	130267614	130268429	47	49
2151	20	3011203	3014058	ENSMUSG00000037727	ENSMUSG00000037727	ENSMUSG00000037727	2	130272062	130273995	47	49
2152	20	3091273	3097207	ENSMUSG00000037703	ENSMUSG00000037703	ENSMUSG00000037703	2	130324213	130334285	47	49
2153	20	3118996	3133314	ENSMUSG00000068290	ENSMUSG00000068290	ENSMUSG00000068290	2	130345476	130356006	47	49
2154	20	3156063	3166812	ENSMUSG00000074796	ENSMUSG00000074796	ENSMUSG00000074796	2	130375555	130380006	47	49
2155	20	3177951	3336272	ENSMUSG00000027309	ENSMUSG00000027309	ENSMUSG00000027309	2	130397641	130531575	47	49
2156	20	3399676	3579760	ENSMUSG00000027312	ENSMUSG00000027312	ENSMUSG00000027312	2	130597937	130721770	47	49
2157	20	3587939	3592046	ENSMUSG00000027316	ENSMUSG00000027316	ENSMUSG00000027316	2	130731617	130734123	47	49
2158	20	3596621	3610750	ENSMUSG00000027318	ENSMUSG00000027318	ENSMUSG00000027318	2	130742266	130755010	47	49
2159	20	3615619	3635775	ENSMUSG00000027322	ENSMUSG00000027322	ENSMUSG00000027322	2	130760425	130778206	47	49
2160	20	3661356	3681758	ENSMUSG00000074793	ENSMUSG00000074793	ENSMUSG00000074793	2	130818853	130837431	47	49
2161	20	3706151	3710083	ENSMUSG00000027329	ENSMUSG00000027329	ENSMUSG00000027329	2	130861702	130878723	47	49
2162	20	3712498	3715256	ENSMUSG00000068267	ENSMUSG00000068267	ENSMUSG00000068267	2	130868911	130871317	47	49
2163	20	3724401	3734757	ENSMUSG00000027330	ENSMUSG00000027330	ENSMUSG00000027330	2	130878399	130889936	47	49
2164	20	3749203	3753954	ENSMUSG00000068264	ENSMUSG00000068264	ENSMUSG00000068264	2	130901881	130904955	47	49
2165	20	3775484	3796972	ENSMUSG00000037523	ENSMUSG00000037523	ENSMUSG00000037523	2	130925508	130939463	47	49
2166	20	3817742	3852502	ENSMUSG00000037514	ENSMUSG00000037514	ENSMUSG00000037514	2	130953941	130988132	47	49
2167	20	3860068	3944036	ENSMUSG00000048911	ENSMUSG00000048911	ENSMUSG00000048911	2	130989929	131044266	47	49
2168	20	4077450	4116369	ENSMUSG00000027333	ENSMUSG00000027333	ENSMUSG00000027333	2	131183384	131216613	47	49
2169	20	4149329	4177659	ENSMUSG00000027335	ENSMUSG00000027335	ENSMUSG00000027335	2	131237291	131253724	47	49
2170	20	4614996	4630236	ENSMUSG00000027338	ENSMUSG00000027338	ENSMUSG00000027338	2	131601398	131629868	47	49
2171	20	4708669	4752291	ENSMUSG00000027339	ENSMUSG00000027339	ENSMUSG00000027339	2	131684291	131721251	47	49
2172	20	4781002	4938939	ENSMUSG00000027340	ENSMUSG00000027340	ENSMUSG00000027340	2	131743937	131836549	47	49
2173	20	5028496	5041733	ENSMUSG00000027341	ENSMUSG00000027341	ENSMUSG00000027341	2	131930935	131939223	47	49
2174	20	5055432	5120000	ENSMUSG00000058793	ENSMUSG00000058793	ENSMUSG00000058793	2	131954589	132003491	47	49
2175	20	5230686	5243015	ENSMUSG00000050558	ENSMUSG00000050558	ENSMUSG00000050558	2	132062606	132076841	47	49
2176	20	5473085	5539672	ENSMUSG00000027346	ENSMUSG00000027346	ENSMUSG00000027346	2	132220525	132269602	47	49
2177	20	5685358	5792556	ENSMUSG00000044991	ENSMUSG00000044991	ENSMUSG00000044991	2	132378403	132442495	47	49
2178	20	5840168	5854007	ENSMUSG00000027350	ENSMUSG00000027350	ENSMUSG00000027350	2	132472719	132486513	47	49
2179	20	5866486	5879173	ENSMUSG00000037376	ENSMUSG00000037376	ENSMUSG00000037376	2	132495656	132507495	47	49
2180	20	5879298	5923827	ENSMUSG00000027353	ENSMUSG00000027353	ENSMUSG00000027353	2	132507635	132535097	47	49
2181	20	5934806	5968699	ENSMUSG00000027357	ENSMUSG00000027357	ENSMUSG00000027357	2	132538107	132558214	47	49
2182	20	5969425	5982677	ENSMUSG00000043110	ENSMUSG00000043110	ENSMUSG00000043110	2	132559957	132572303	47	49
2183	20	6003493	6052191	ENSMUSG00000027356	ENSMUSG00000027356	ENSMUSG00000027356	2	132595833	132637069	47	49
2184	20	6696311	6708927	ENSMUSG00000027358	ENSMUSG00000027358	ENSMUSG00000027358	2	133244640	133254326	47	49
2185	20	7811630	7869121	ENSMUSG00000027261	ENSMUSG00000027261	ENSMUSG00000027261	2	134188802	134245793	47	49
2186	20	7906024	7948476	ENSMUSG00000034723	ENSMUSG00000034723	ENSMUSG00000034723	2	134285949	134335562	47	49
2187	20	8060908	8813547	ENSMUSG00000051177	ENSMUSG00000051177	ENSMUSG00000051177	2	134477974	135163721	47	49
2188	20	9024932	9409460	ENSMUSG00000039943	ENSMUSG00000039943	ENSMUSG00000039943	2	135496989	135704509	47	49
2189	20	9443271	9459171	ENSMUSG00000027270	ENSMUSG00000027270	ENSMUSG00000027270	2	135749368	135761358	47	49
2190	20	9466036	9767689	ENSMUSG00000039913	ENSMUSG00000039913	ENSMUSG00000039913	2	135773338	136079379	47	49
2191	20	9963689	9984470	ENSMUSG00000074771	ENSMUSG00000074771	ENSMUSG00000074771	2	136223627	136253532	47	49
2192	20	10147477	10236065	ENSMUSG00000027273	ENSMUSG00000027273	ENSMUSG00000027273	2	136404894	136473860	47	49
2193	20	10363951	10552027	ENSMUSG00000027281	ENSMUSG00000027281	ENSMUSG00000027281	2	136451056	136761219	47	49
2194	20	10333839	10362866	ENSMUSG00000027274	ENSMUSG00000027274	ENSMUSG00000027274	2	136565224	136582823	47	49
2195	20	10566334	10602636	ENSMUSG00000027276	ENSMUSG00000027276	ENSMUSG00000027276	2	136772899	136807772	47	49
2196	20	12937642	13093118	ENSMUSG00000039092	ENSMUSG00000039092	ENSMUSG00000039092	2	139185361	139329115	47	49
2197	20	13199151	13229298	ENSMUSG00000074766	ENSMUSG00000074766	ENSMUSG00000074766	2	139369619	139450011	47	49
2198	20	13318039	13567550	ENSMUSG00000039033	ENSMUSG00000039033	ENSMUSG00000039033	2	139525027	139758201	47	49
2199	20	13642969	13713532	ENSMUSG00000045624	ENSMUSG00000045624	ENSMUSG00000045624	2	139812053	139859879	47	49
2200	20	13713596	13747067	ENSMUSG00000027384	ENSMUSG00000027384	ENSMUSG00000027384	2	139862220	139895073	47	49
2201	20	13778052	13919257	ENSMUSG00000074764	ENSMUSG00000074764	ENSMUSG00000074764	2	139921373	139996218	47	49
2202	20	13924015	15978696	ENSMUSG00000068205	ENSMUSG00000068205	ENSMUSG00000068205	2	140087117	142075829	47	49
2203	20	14251634	14266254	ENSMUSG00000051379	ENSMUSG00000051379	ENSMUSG00000051379	2	140349640	140362910	47	49
2204	20	16295488	16502078	ENSMUSG00000038844	ENSMUSG00000038844	ENSMUSG00000038844	2	142394046	142548868	47	49
2205	20	16677003	16680809	ENSMUSG00000027416	ENSMUSG00000027416	ENSMUSG00000027416	2	142769918	142773154	47	49
2206	20	17155631	17413223	ENSMUSG00000027419	ENSMUSG00000027419	ENSMUSG00000027419	2	143237620	143507727	47	49
2207	20	17422550	17460014	ENSMUSG00000027420	ENSMUSG00000027420	ENSMUSG00000027420	2	143517969	143554614	47	49
2208	20	17498697	17536887	ENSMUSG00000015932	ENSMUSG00000015932	ENSMUSG00000015932	2	143606761	143634765	47	49
2209	20	17542747	17589152	ENSMUSG00000027422	ENSMUSG00000027422	ENSMUSG00000027422	2	143638837	143681686	47	49
2210	20	17628593	17664516	ENSMUSG00000037307	ENSMUSG00000037307	ENSMUSG00000037307	2	143724543	143765418	47	49
2211	20	17870241	17897623	ENSMUSG00000027423	ENSMUSG00000027423	ENSMUSG00000027423	2	143941567	143961972	47	49
2212	20	17897556	17919765	ENSMUSG00000027424	ENSMUSG00000027424	ENSMUSG00000027424	2	143962476	143972670	47	49
2213	20	17946078	17986521	ENSMUSG00000037279	ENSMUSG00000037279	ENSMUSG00000037279	2	143996619	144023542	47	49
2214	20	18066527	18117016	ENSMUSG00000027425	ENSMUSG00000027425	ENSMUSG00000027425	2	144060476	144099074	47	49
2215	20	18226629	18245640	ENSMUSG00000074756	ENSMUSG00000074756	ENSMUSG00000074756	2	144110615	144158057	47	49
2216	20	18312721	18396531	ENSMUSG00000037259	ENSMUSG00000037259	ENSMUSG00000037259	2	144166365	144214017	47	49
2217	20	18395771	18413287	ENSMUSG00000027427	ENSMUSG00000027427	ENSMUSG00000027427	2	144219186	144233220	47	49
2218	20	18415184	18425887	ENSMUSG00000027428	ENSMUSG00000027428	ENSMUSG00000027428	2	144233706	144242300	47	49
2219	20	18436188	18490048	ENSMUSG00000027429	ENSMUSG00000027429	ENSMUSG00000027429	2	144247721	144282185	47	49
2220	20	18516537	18692561	ENSMUSG00000027430	ENSMUSG00000027430	ENSMUSG00000027430	2	144291426	144460162	47	49
2221	20	18742370	18831180	ENSMUSG00000027431	ENSMUSG00000027431	ENSMUSG00000027431	2	144515107	144515855	47	49
2222	20	19141290	19651581	ENSMUSG00000063873	ENSMUSG00000063873	ENSMUSG00000063873	2	144859500	145331802	47	49
2223	20	19815165	19931101	ENSMUSG00000001768	ENSMUSG00000001768	ENSMUSG00000001768	2	145513706	145577364	47	49
2224	20	19981196	20289346	ENSMUSG00000048201	ENSMUSG00000048201	ENSMUSG00000048201	2	145703546	145906233	47	49
2225	20	20296765	20299593	ENSMUSG00000068154	ENSMUSG00000068154	ENSMUSG00000068154	2	145913362	145916457	47	49
2226	20	20320856	20568542	ENSMUSG00000037110	ENSMUSG00000037110	ENSMUSG00000037110	2	146006360	146142006	47	49
2227	20	21054646	21175214	ENSMUSG00000074749	ENSMUSG00000074749	ENSMUSG00000074749	2	146547328	146661530	47	49
2228	20	21231942	21318463	ENSMUSG00000027433	ENSMUSG00000027433	ENSMUSG00000027433	2	146704460	146769436	47	49
2229	20	21324005	21326047	ENSMUSG00000054160	ENSMUSG00000054160	ENSMUSG00000054160	2	146774759	146776786	47	49
2230	20	21439648	21442699	ENSMUSG00000027434	ENSMUSG00000027434	ENSMUSG00000027434	2	146874593	146877800	47	49
2231	20	21634364	21644620	ENSMUSG00000037034	ENSMUSG00000037034	ENSMUSG00000037034	2	147056501	147066490	47	49
2232	20	22509643	22514093	ENSMUSG00000037025	ENSMUSG00000037025	ENSMUSG00000037025	2	147734364	147738410	47	49
2233	20	22964023	22965449	ENSMUSG00000037014	ENSMUSG00000037014	ENSMUSG00000037014	2	148086783	148092384	47	49
2234	20	22974270	22978287	ENSMUSG00000074743	ENSMUSG00000074743	ENSMUSG00000074743	2	148097654	148099387	47	49
2235	20	23007995	23014977	ENSMUSG00000027435	ENSMUSG00000027435	ENSMUSG00000027435	2	148128092	148134976	47	49
2236	20	23279373	23283408	ENSMUSG00000036992	ENSMUSG00000036992	ENSMUSG00000036992	2	148364083	148367464	47	49
2237	20	23290819	23301700	ENSMUSG00000027439	ENSMUSG00000027439	ENSMUSG00000027439	2	148372605	148384388	47	49
2238	20	23303159	23350109	ENSMUSG00000027438	ENSMUSG00000027438	ENSMUSG00000027438	2	148385426	148423908	47	49
2239	20	23368322	23373567	ENSMUSG00000055177	ENSMUSG00000055177	ENSMUSG00000055177	2	148441802	148446821	47	49
2240	20	23379040	23381482	ENSMUSG00000036958	ENSMUSG00000036958	ENSMUSG00000036958	2	148460059	148462938	47	49
2241	20	23419766	23424655	ENSMUSG00000027442	ENSMUSG00000027442	ENSMUSG00000027442	2	148490275	148497022	47	49
2242	20	23449513	23465607	ENSMUSG00000036924	ENSMUSG00000036924	ENSMUSG00000036924	2	148511522	148521848	47	49
2243	20	23531047	23534513	ENSMUSG00000027445	ENSMUSG00000027445	ENSMUSG00000027445	2	148526588	148530178	47	49
2244	20	24397835	24595252	ENSMUSG00000074736	ENSMUSG00000074736	ENSMUSG00000074736	2	149590937	149694776	47	49
2245	20	24877866	24888564	ENSMUSG00000068129	ENSMUSG00000068129	ENSMUSG00000068129	2	150261856	150270377	47	49
2246	20	24891581	24921425	ENSMUSG00000033096	ENSMUSG00000033096	ENSMUSG00000033096	2	150274525	150299983	47	49
2247	20	24934874	24987616	ENSMUSG00000027452	ENSMUSG00000027452	ENSMUSG00000027452	2	150309552	150359686	47	49
2248	20	25004102	25010996	ENSMUSG00000033080	ENSMUSG00000033080	ENSMUSG00000033080	2	150372140	150380578	47	49
2249	20	25124372	25155365	ENSMUSG00000033068	ENSMUSG00000033068	ENSMUSG00000033068	2	150440483	150463116	47	49
2250	20	25176706	25226648	ENSMUSG00000033059	ENSMUSG00000033059	ENSMUSG00000033059	2	150478229	150523191	47	49
2251	20	25223379	25319612	ENSMUSG00000032046	ENSMUSG00000032046	ENSMUSG00000032046	2	150523956	150596140	47	49
2252	20	25336363	25377191	ENSMUSG00000027454	ENSMUSG00000027454	ENSMUSG00000027454	2	150601042	150622719	47	49
2253	20	25381341	25514153	ENSMUSG00000068115	ENSMUSG00000068115	ENSMUSG00000068115	2	150626689	150671636	47	49
2254	20	1238619	1254513	ENSMUSG00000027456	ENSMUSG00000027456	ENSMUSG00000027456	2	151264073	151281442	48	50
2255	20	1194999	1237972	ENSMUSG00000027457	ENSMUSG00000027457	ENSMUSG00000027457	2	151281991	151323974	48	50
2256	20	1157098	1162834	ENSMUSG00000074704	ENSMUSG00000074704	ENSMUSG00000074704	2	151346414	151359974	48	50
2257	20	1109213	1113117	ENSMUSG00000044364	ENSMUSG00000044364	ENSMUSG00000044364	2	151393449	151398738	48	50
2258	20	1041939	1095971	ENSMUSG00000032869	ENSMUSG00000032869	ENSMUSG00000032869	2	151407503	151432734	48	50
2259	20	887098	931404	ENSMUSG00000032852	ENSMUSG00000032852	ENSMUSG00000032852	2	151534368	151566109	48	50
2260	20	801296	844960	ENSMUSG00000027460	ENSMUSG00000027460	ENSMUSG00000027460	2	151602773	151636095	48	50
2261	20	762371	774890	ENSMUSG00000027459	ENSMUSG00000027459	ENSMUSG00000027459	2	151660841	151671613	48	50
2262	20	688725	697131	ENSMUSG00000027463	ENSMUSG00000027463	ENSMUSG00000027463	2	151688069	151700697	48	50
2263	20	590241	604805	ENSMUSG00000060257	ENSMUSG00000060257	ENSMUSG00000060257	2	151773491	151785305	48	50
2264	20	532441	538953	ENSMUSG00000068079	ENSMUSG00000068079	ENSMUSG00000068079	2	151835013	151840538	48	50
2265	20	364124	391175	ENSMUSG00000027465	ENSMUSG00000027465	ENSMUSG00000027465	2	151985317	152005432	48	50
2266	20	336756	359610	ENSMUSG00000027466	ENSMUSG00000027466	ENSMUSG00000027466	2	152007769	152024076	48	50
2267	20	309273	326205	ENSMUSG00000032715	ENSMUSG00000032715	ENSMUSG00000032715	2	152029648	152034768	48	50
2268	20	275668	283512	ENSMUSG00000059361	ENSMUSG00000059361	ENSMUSG00000059361	2	152060202	152068039	48	50
2269	20	254045	257374	ENSMUSG00000051817	ENSMUSG00000051817	ENSMUSG00000051817	2	152085052	152089487	48	50
2270	20	225737	228965	ENSMUSG00000074682	ENSMUSG00000074682	ENSMUSG00000074682	2	152105016	152106218	48	50
2271	20	199524	219390	ENSMUSG00000032680	ENSMUSG00000032680	ENSMUSG00000032680	2	152107028	152135769	48	50
2272	20	155899	158527	ENSMUSG00000074681	ENSMUSG00000074681	ENSMUSG00000074681	2	152150591	152156033	48	50
2273	20	116527	118264	ENSMUSG00000049560	ENSMUSG00000049560	ENSMUSG00000049560	2	152168504	152171375	48	50
2274	20	16351	25296	ENSMUSG00000074680	ENSMUSG00000074680	ENSMUSG00000074680	2	152199239	152203133	48	50
2275	20	29420082	29425364	ENSMUSG00000056544	ENSMUSG00000056544	ENSMUSG00000056544	2	152264185	152266384	49	51
2276	20	29472974	29475362	ENSMUSG00000062124	ENSMUSG00000062124	ENSMUSG00000062124	2	152284664	152287926	49	51
2277	20	29492014	29501721	ENSMUSG00000044863	ENSMUSG00000044863	ENSMUSG00000044863	2	152295814	152304169	49	51
2278	20	29516970	29524477	ENSMUSG00000074678	ENSMUSG00000074678	ENSMUSG00000074678	2	152313797	152314494	49	51
2279	20	29526766	29536368	ENSMUSG00000000359	ENSMUSG00000000359	ENSMUSG00000000359	2	152318449	152326632	49	51
2280	20	29565902	29621029	ENSMUSG00000019188	ENSMUSG00000019188	ENSMUSG00000019188	2	152360929	152396477	49	51
2281	20	29656747	29657979	ENSMUSG00000042745	ENSMUSG00000042745	ENSMUSG00000042745	2	152427715	152428851	49	51
2282	20	29689352	29696470	ENSMUSG00000009876	ENSMUSG00000009876	ENSMUSG00000009876	2	152446596	152456322	49	51
2283	20	29715916	29774362	ENSMUSG00000007659	ENSMUSG00000007659	ENSMUSG00000007659	2	152458757	152523123	49	51
2284	20	29790565	29853264	ENSMUSG00000027469	ENSMUSG00000027469	ENSMUSG00000027469	2	152539504	152585459	49	51
2285	20	29895764	29897083	ENSMUSG00000074676	ENSMUSG00000074676	ENSMUSG00000074676	2	152623583	152624572	49	51
2286	20	29899102	29922128	ENSMUSG00000042662	ENSMUSG00000042662	ENSMUSG00000042662	2	152635652	152642846	49	51
2287	20	29922166	29996431	ENSMUSG00000074673	ENSMUSG00000074673	ENSMUSG00000074673	2	152653947	152694671	49	51
2288	20	29996430	30003556	ENSMUSG00000027472	ENSMUSG00000027472	ENSMUSG00000027472	2	152700331	152706824	49	51
2289	20	30019466	30049916	ENSMUSG00000042631	ENSMUSG00000042631	ENSMUSG00000042631	2	152723293	152747216	49	51
2290	20	30103718	30153316	ENSMUSG00000003283	ENSMUSG00000003283	ENSMUSG00000003283	2	152799949	152842877	49	51
2291	20	30161200	30218718	ENSMUSG00000068040	ENSMUSG00000068040	ENSMUSG00000068040	2	152852742	152901904	49	51
2292	20	30243968	30259192	ENSMUSG00000051413	ENSMUSG00000051413	ENSMUSG00000051413	2	152919210	152932799	49	51
2293	20	30259344	30290131	ENSMUSG00000046020	ENSMUSG00000046020	ENSMUSG00000046020	2	152932973	152961694	49	51
2294	20	30329128	30386475	ENSMUSG00000027475	ENSMUSG00000027475	ENSMUSG00000027475	2	152982896	153024822	49	51
2295	20	30409821	30490783	ENSMUSG00000042548	ENSMUSG00000042548	ENSMUSG00000042548	2	153037580	153093540	49	51
2296	20	30494523	30534922	ENSMUSG00000061411	ENSMUSG00000061411	ENSMUSG00000061411	2	153098907	153221203	49	51
2297	20	30754166	30795464	ENSMUSG00000056941	ENSMUSG00000056941	ENSMUSG00000056941	2	153310275	153324172	49	51
2298	20	30813852	30860822	ENSMUSG00000027478	ENSMUSG00000027478	ENSMUSG00000027478	2	153340895	153379171	49	51
2299	20	30871395	30901872	ENSMUSG00000027479	ENSMUSG00000027479	ENSMUSG00000027479	2	153432791	153458971	49	51
2300	20	31035240	31055900	ENSMUSG00000027480	ENSMUSG00000027480	ENSMUSG00000027480	2	153547631	153562525	49	51
2301	20	31059067	31075175	ENSMUSG00000027481	ENSMUSG00000027481	ENSMUSG00000027481	2	153566486	153586709	49	51
2302	20	31083115	31095514	ENSMUSG00000068009	ENSMUSG00000068009	ENSMUSG00000068009	2	153591829	153604236	49	51
2303	20	31106836	31125288	ENSMUSG00000068008	ENSMUSG00000068008	ENSMUSG00000068008	2	153609671	153624437	49	51
2304	20	31133029	31163218	ENSMUSG00000074665	ENSMUSG00000074665	ENSMUSG00000074665	2	153635364	153655293	49	51
2305	20	31213315	31232884	ENSMUSG00000042459	ENSMUSG00000042459	ENSMUSG00000042459	2	153699706	153707513	49	51
2306	20	31268777	31279218	ENSMUSG00000027482	ENSMUSG00000027482	ENSMUSG00000027482	2	153821788	153829792	49	51
2307	20	31334602	31361345	ENSMUSG00000027485	ENSMUSG00000027485	ENSMUSG00000027485	2	153882259	153911784	49	51
2308	20	31410308	31452998	ENSMUSG00000027487	ENSMUSG00000027487	ENSMUSG00000027487	2	154026821	154064168	49	51
2309	20	31459424	31495359	ENSMUSG00000027488	ENSMUSG00000027488	ENSMUSG00000027488	2	154067769	154099522	49	51
2310	20	31651842	31701498	ENSMUSG00000038533	ENSMUSG00000038533	ENSMUSG00000038533	2	154127959	154230797	49	51
2311	20	31708555	31725880	ENSMUSG00000027489	ENSMUSG00000027489	ENSMUSG00000027489	2	154235840	154250285	49	51
2312	20	31713753	31715374	ENSMUSG00000038523	ENSMUSG00000038523	ENSMUSG00000038523	2	154240345	154241441	49	51
2313	20	31727147	31737871	ENSMUSG00000027490	ENSMUSG00000027490	ENSMUSG00000027490	2	154251079	154261293	49	51
2314	20	31758176	31771764	ENSMUSG00000000876	ENSMUSG00000000876	ENSMUSG00000000876	2	154278485	154295114	49	51
2315	20	31783124	31843736	ENSMUSG00000059842	ENSMUSG00000059842	ENSMUSG00000059842	2	154304809	154338258	49	51
2316	20	31862780	31905830	ENSMUSG00000038467	ENSMUSG00000038467	ENSMUSG00000038467	2	154348565	154386226	49	51
2317	20	32311832	32320811	ENSMUSG00000027596	ENSMUSG00000027596	ENSMUSG00000027596	2	154704972	154742452	49	51
2318	20	32567921	32592423	ENSMUSG00000047459	ENSMUSG00000047459	ENSMUSG00000047459	2	154934172	154941405	49	51
2319	20	32598319	32611810	ENSMUSG00000027602	ENSMUSG00000027602	ENSMUSG00000027602	2	154967885	154969513	49	51
2320	20	32612007	32728750	ENSMUSG00000038383	ENSMUSG00000038383	ENSMUSG00000038383	2	154969687	155048836	49	51
2321	20	32755804	32764901	ENSMUSG00000038375	ENSMUSG00000038375	ENSMUSG00000038375	2	155073297	155081288	49	51
2322	20	32766239	32877094	ENSMUSG00000038369	ENSMUSG00000038369	ENSMUSG00000038369	2	155082112	155132224	49	51
2323	20	32896184	32924318	ENSMUSG00000027603	ENSMUSG00000027603	ENSMUSG00000027603	2	155181828	155206284	49	51
2324	20	32928078	32979424	ENSMUSG00000027605	ENSMUSG00000027605	ENSMUSG00000027605	2	155209498	155277165	49	51
2325	20	32979898	33007262	ENSMUSG00000027610	ENSMUSG00000027610	ENSMUSG00000027610	2	155254624	155284153	49	51
2326	20	33029094	33053897	ENSMUSG00000074652	ENSMUSG00000074652	ENSMUSG00000074652	2	155303099	155325492	49	51
2327	20	33053876	33144279	ENSMUSG00000038324	ENSMUSG00000038324	ENSMUSG00000038324	2	155325727	155383801	49	51
2328	20	33166842	33198828	ENSMUSG00000038312	ENSMUSG00000038312	ENSMUSG00000038312	2	155393118	155420866	49	51
2329	20	33212131	33228826	ENSMUSG00000027611	ENSMUSG00000027611	ENSMUSG00000027611	2	155442785	155446899	49	51
2330	20	33278095	33328218	ENSMUSG00000027612	ENSMUSG00000027612	ENSMUSG00000027612	2	155466840	155509807	49	51
2331	20	33330128	33336202	ENSMUSG00000027613	ENSMUSG00000027613	ENSMUSG00000027613	2	155511278	155518366	49	51
2332	20	33336950	33343639	ENSMUSG00000074647	ENSMUSG00000074647	ENSMUSG00000074647	2	155521609	155526295	49	51
2333	20	33353785	33463359	ENSMUSG00000005882	ENSMUSG00000005882	ENSMUSG00000005882	2	155539833	155613168	49	51
2334	20	33484559	33505982	ENSMUSG00000038259	ENSMUSG00000038259	ENSMUSG00000038259	2	155632468	155636808	49	51
2335	20	33506564	33563218	ENSMUSG00000038241	ENSMUSG00000038241	ENSMUSG00000038241	2	155653413	155690144	49	51
2336	20	33593192	33608818	ENSMUSG00000005881	ENSMUSG00000005881	ENSMUSG00000005881	2	155699547	155709718	49	51
2337	20	33652133	33658824	ENSMUSG00000074644	ENSMUSG00000074644	ENSMUSG00000074644	2	155737093	155739938	49	51

2338	20	33667228	33672385	ENSMUSG00000038180	ENSMUSG00000038180	ENSMUSG00000038180	2	155756803	155762060	49	51
2339	20	33700261	33716252	ENSMUSG00000038171	ENSMUSG00000038171	ENSMUSG00000038171	2	155786069	155803363	49	51
2340	20	33720024	33750695	ENSMUSG00000027618	ENSMUSG00000027618	ENSMUSG00000027618	2	155815090	155835626	49	51
2341	20	33754955	33793607	ENSMUSG00000027620	ENSMUSG00000027620	ENSMUSG00000027620	2	155838689	155871602	49	51
2342	20	34019926	34082035	ENSMUSG00000038085	ENSMUSG00000038085	ENSMUSG00000038085	2	156003890	156067078	49	51
2343	20	34206076	34284135	ENSMUSG00000027624	ENSMUSG00000027624	ENSMUSG00000027624	2	156112350	156234655	49	51
2344	20	34287814	34308267	ENSMUSG00000027628	ENSMUSG00000027628	ENSMUSG00000027628	2	156239040	156260413	49	51
2345	20	34357717	34590450	ENSMUSG00000061689	ENSMUSG00000061689	ENSMUSG00000061689	2	156392120	156455802	49	51
2346	20	34603311	34611640	ENSMUSG00000067818	ENSMUSG00000067818	ENSMUSG00000067818	2	156466870	156473098	49	51
2347	20	34667581	34674370	ENSMUSG00000027637	ENSMUSG00000027637	ENSMUSG00000027637	2	156554569	156565000	49	51
2348	20	34674135	34707996	ENSMUSG00000027636	ENSMUSG00000027636	ENSMUSG00000027636	2	156563898	156578519	49	51
2349	20	34713583	34807895	ENSMUSG00000027634	ENSMUSG00000027634	ENSMUSG00000027634	2	156618787	156683484	49	51
2350	20	34813612	34835614	ENSMUSG00000027635	ENSMUSG00000027635	ENSMUSG00000027635	2	156686718	156698595	49	51
2351	20	34844199	34924651	ENSMUSG00000055485	ENSMUSG00000055485	ENSMUSG00000055485	2	156707238	156724799	49	51
2352	20	34937984	34956033	ENSMUSG00000074628	ENSMUSG00000074628	ENSMUSG00000074628	2	156754333	156787922	49	51
2353	20	34953761	35013490	ENSMUSG00000027639	ENSMUSG00000027639	ENSMUSG00000027639	2	156788970	156826663	49	51
2354	20	35058166	35157812	ENSMUSG00000027641	ENSMUSG00000027641	ENSMUSG00000027641	2	156837339	156895960	49	51
2355	20	35163044	35241297	ENSMUSG00000074627	ENSMUSG00000074627	ENSMUSG00000074627	2	156903114	156963528	49	51
2356	20	35240888	35303434	ENSMUSG00000027642	ENSMUSG00000027642	ENSMUSG00000027642	2	156970500	157017760	49	51
2357	20	35312899	35323652	ENSMUSG00000027643	ENSMUSG00000027643	ENSMUSG00000027643	2	157020938	157038096	49	51
2358	20	35445971	35467239	ENSMUSG00000027646	ENSMUSG00000027646	ENSMUSG00000027646	2	157115730	157163278	49	51
2359	20	35579234	35589717	ENSMUSG00000067787	ENSMUSG00000067787	ENSMUSG00000067787	2	157247804	157257802	49	51
2360	20	35583148	35584979	ENSMUSG00000067786	ENSMUSG00000067786	ENSMUSG00000067786	2	157251551	157253958	49	51
2361	20	35755848	35933933	ENSMUSG00000027649	ENSMUSG00000027649	ENSMUSG00000027649	2	157428903	157583055	49	51
2362	20	35964920	36007166	ENSMUSG00000037843	ENSMUSG00000037843	ENSMUSG00000037843	2	157606206	157635821	49	51
2363	20	36044823	36095247	ENSMUSG00000027650	ENSMUSG00000027650	ENSMUSG00000027650	2	157673244	157719874	49	51
2364	20	36095467	36154632	ENSMUSG00000027651	ENSMUSG00000027651	ENSMUSG00000027651	2	157720162	157768754	49	51
2365	20	36190280	36227114	ENSMUSG00000037820	ENSMUSG00000037820	ENSMUSG00000037820	2	157807843	157837877	49	51
2366	20	36272321	36322588	ENSMUSG00000037813	ENSMUSG00000037813	ENSMUSG00000037813	2	157887192	157920663	49	51
2367	20	36365991	36399319	ENSMUSG00000052922	ENSMUSG00000052922	ENSMUSG00000052922	2	157949682	157975972	49	51
2368	20	36408299	36439067	ENSMUSG00000016024	ENSMUSG00000016024	ENSMUSG00000016024	2	157997986	158023737	49	51
2369	20	36534900	36640918	ENSMUSG00000027652	ENSMUSG00000027652	ENSMUSG00000027652	2	158112285	158190689	49	51
2370	20	36643252	36650518	ENSMUSG00000044405	ENSMUSG00000044405	ENSMUSG00000044405	2	158194049	158199639	49	51
2371	20	36696740	36708217	ENSMUSG00000074625	ENSMUSG00000074625	ENSMUSG00000074625	2	158226254	158242069	49	51
2372	20	36786519	36791429	ENSMUSG00000037771	ENSMUSG00000037771	ENSMUSG00000037771	2	158302199	158307188	49	51
2373	20	36810511	36834247	ENSMUSG00000037761	ENSMUSG00000037761	ENSMUSG00000037761	2	158316373	158330639	49	51
2374	20	36867762	36985081	ENSMUSG00000037754	ENSMUSG00000037754	ENSMUSG00000037754	2	158356839	158454314	49	51
2375	20	36988369	37015117	ENSMUSG00000027654	ENSMUSG00000027654	ENSMUSG00000027654	2	158459534	158478076	49	51
2376	20	37024409	37101777	ENSMUSG00000027655	ENSMUSG00000027655	ENSMUSG00000027655	2	158486261	158549657	49	51
2377	20	38747902	38751281	ENSMUSG00000074622	ENSMUSG00000074622	ENSMUSG00000074622	2	160057146	160058117	49	51
2378	20	39090876	39186541	ENSMUSG00000070544	ENSMUSG00000070544	ENSMUSG00000070544	2	160337671	160414202	49	51
2379	20	39199291	39237775	ENSMUSG00000016933	ENSMUSG00000016933	ENSMUSG00000016933	2	160422751	160467201	49	51
2380	20	39240502	39266970	ENSMUSG00000035877	ENSMUSG00000035877	ENSMUSG00000035877	2	160470666	160528770	49	51
2381	20	39402974	39422636	ENSMUSG00000027412	ENSMUSG00000027412	ENSMUSG00000027412	2	160572111	160597438	49	51
2382	20	39422020	39428912	ENSMUSG00000050700	ENSMUSG00000050700	ENSMUSG00000050700	2	160597879	160603769	49	51
2383	20	39464155	39680547	ENSMUSG00000057133	ENSMUSG00000057133	ENSMUSG00000057133	2	160638399	160800497	49	51
2384	20	40134806	41252023	ENSMUSG00000053141	ENSMUSG00000053141	ENSMUSG00000053141	2	161220957	162352340	49	51
2385	20	41519932	41525655	ENSMUSG00000016921	ENSMUSG00000016921	ENSMUSG00000016921	2	162622982	162628564	49	51
2386	20	41576467	41603949	ENSMUSG00000035576	ENSMUSG00000035576	ENSMUSG00000035576	2	162638630	162665966	49	51
2387	20	41628151	41647731	ENSMUSG00000017868	ENSMUSG00000017868	ENSMUSG00000017868	2	162678987	162705568	49	51
2388	20	41652985	41709350	ENSMUSG00000017858	ENSMUSG00000017858	ENSMUSG00000017858	2	162708913	162737576	49	51
2389	20	41729123	41778536	ENSMUSG00000017861	ENSMUSG00000017861	ENSMUSG00000017861	2	162746076	162776128	49	51
2390	20	42007759	42131666	ENSMUSG00000074607	ENSMUSG00000074607	ENSMUSG00000074607	2	162916909	163014221	49	51
2391	20	42173749	42249632	ENSMUSG00000017817	ENSMUSG00000017817	ENSMUSG00000017817	2	163029214	163089391	49	51
2392	20	42258550	42272845	ENSMUSG00000035399	ENSMUSG00000035399	ENSMUSG00000035399	2	163097270	163110929	49	51
2393	20	42309301	42342427	ENSMUSG00000017943	ENSMUSG00000017943	ENSMUSG00000017943	2	163129919	163145393	49	51
2394	20	42364894	42373505	ENSMUSG00000048486	ENSMUSG00000048486	ENSMUSG00000048486	2	163157829	163164069	49	51
2395	20	42463338	42493444	ENSMUSG00000017950	ENSMUSG00000017950	ENSMUSG00000017950	2	163238629	163264348	49	51
2396	20	42537942	42556658	ENSMUSG00000017679	ENSMUSG00000017679	ENSMUSG00000017679	2	163293767	163310454	49	51
2397	20	42558279	42584140	ENSMUSG00000017707	ENSMUSG00000017707	ENSMUSG00000017707	2	163314714	163336572	49	51
2398	20	42593840	42681092	ENSMUSG00000035268	ENSMUSG00000035268	ENSMUSG00000035268	2	163349845	163417591	49	51
2399	20	42681577	42713797	ENSMUSG00000017697	ENSMUSG00000017697	ENSMUSG00000017697	2	163418025	163441618	49	51
2400	20	42776889	42789866	ENSMUSG00000027656	ENSMUSG00000027656	ENSMUSG00000027656	2	163512340	163524587	49	51
2401	20	42807835	42813089	ENSMUSG00000035238	ENSMUSG00000035238	ENSMUSG00000035238	2	163545191	163550315	49	51
2402	20	42813863	42872393	ENSMUSG00000035226	ENSMUSG00000035226	ENSMUSG00000035226	2	163555322	163610124	49	51
2403	20	42947731	42970587	ENSMUSG00000018326	ENSMUSG00000018326	ENSMUSG00000018326	2	163686638	163710028	49	51
2404	20	42972199	43001371	ENSMUSG00000054582	ENSMUSG00000054582	ENSMUSG00000054582	2	163716922	163741972	49	51
2405	20	43004185	43022541	ENSMUSG00000018322	ENSMUSG00000018322	ENSMUSG00000018322	2	163744982	163762543	49	51
2406	20	43028529	43142014	ENSMUSG00000018209	ENSMUSG00000018209	ENSMUSG00000018209	2	163765593	163846964	49	51
2407	20	43154365	43163167	ENSMUSG00000040164	ENSMUSG00000040164	ENSMUSG00000040164	2	163855746	163862530	49	51
2408	20	43171507	43177217	ENSMUSG00000040154	ENSMUSG00000040154	ENSMUSG00000040154	2	163868802	163874182	49	51
2409	20	43185480	43186520	ENSMUSG00000042845	ENSMUSG00000042845	ENSMUSG00000042845	2	163880665	163881999	49	51
2410	20	43355821	43370583	ENSMUSG00000016995	ENSMUSG00000016995	ENSMUSG00000016995	2	164080834	164096365	49	51
2411	20	43368905	43379876	ENSMUSG00000017007	ENSMUSG00000017007	ENSMUSG00000017007	2	164094399	164106653	49	51
2412	20	43387342	43410478	ENSMUSG00000017009	ENSMUSG00000017009	ENSMUSG00000017009	2	164115452	164134393	49	51
2413	20	43423991	43429268	ENSMUSG00000045503	ENSMUSG00000045503	ENSMUSG00000045503	2	164152514	164165503	49	51
2414	20	43435940	43440447	ENSMUSG00000017720	ENSMUSG00000017720	ENSMUSG00000017720	2	164161509	164164929	49	51
2415	20	43470061	43472664	ENSMUSG00000017734	ENSMUSG00000017734	ENSMUSG00000017734	2	164177520	164181968	49	51
2416	20	43478192	43488295	ENSMUSG00000017721	ENSMUSG00000017721	ENSMUSG00000017721	2	164188738	164199504	49	51
2417	20	43531760	43543586	ENSMUSG00000017723	ENSMUSG00000017723	ENSMUSG00000017723	2	164253921	164259711	49	51
2418	20	43602679	43609442	ENSMUSG00000017733	ENSMUSG00000017733	ENSMUSG00000017733	2	164279548	164284799	49	51
2419	20	43613206	43641379	ENSMUSG00000070533	ENSMUSG00000070533	ENSMUSG00000070533	2	164290205	164304831	49	51
2420	20	43669992	43693321	ENSMUSG00000074594	ENSMUSG00000074594	ENSMUSG00000074594	2	164340992	164342968	49	51
2421	20	43691579	43693249	ENSMUSG00000070529	ENSMUSG00000070529	ENSMUSG00000070529	2	164347251	164348573	49	51
2422	20	43764069	43770870	ENSMUSG00000067704	ENSMUSG00000067704	ENSMUSG00000067704	2	164376312	164378911	49	51
2423	20	43784402	43787882	ENSMUSG00000017310	ENSMUSG00000017310	ENSMUSG00000017310	2	164389706	164393658	49	51
2424	20	43836254	43853954	ENSMUSG00000076434	ENSMUSG00000076434	ENSMUSG00000076434	2	164422385	164434517	49	51
2425	20	43853983	43873473	ENSMUSG00000017299	ENSMUSG00000017299	ENSMUSG00000017299	2	164437220	164459429	49	51
2426	20	43885260	43889387	ENSMUSG00000017300	ENSMUSG00000017300	ENSMUSG00000017300	2	164468366	164470946	49	51
2427	20	43895877	43905321	ENSMUSG00000050373	ENSMUSG00000050373	ENSMUSG00000050373	2	164477271	164485014	49	51
2428	20	43903768	43919442	ENSMUSG00000017307	ENSMUSG00000017307	ENSMUSG00000017307	2	164483973	164496413	49	51
2429	20	43919663	43941174	ENSMUSG00000045822	ENSMUSG00000045822	ENSMUSG00000045822	2	164496305	164513332	49	51
2430	20	43943273	43947312	ENSMUSG00000017764	ENSMUSG00000017764	ENSMUSG00000017764	2	164513891	164518072	49	51
2431	20	43948535	43949681	ENSMUSG00000017767	ENSMUSG00000017767	ENSMUSG00000017767	2	164518587	164519711	49	51
2432	20	43950674	43953333	ENSMUSG00000039873	ENSMUSG00000039873	ENSMUSG00000039873	2	164521938	164524661	49	51
2433	20	43952190	43960862	ENSMUSG00000017760	ENSMUSG00000017760	ENSMUSG00000017760	2	164524078	164532237	49	51
2434	20	43960719	43974169	ENSMUSG00000017754	ENSMUSG00000017754	ENSMUSG00000017754	2	164530723	164548913	49	51
2435	20	43996674	44010069	ENSMUSG00000039849	ENSMUSG00000039849	ENSMUSG00000039849	2	164570573	164582642	49	51
2436	20	44010700	44034240	ENSMUSG00000039834	ENSMUSG00000039834	ENSMUSG00000039834	2	164583098	164602955	49	51
2437	20	44070954	44078607	ENSMUSG00000017737	ENSMUSG00000017737	ENSMUSG00000017737	2	164639437	164647050	49	51
2438	20	44091245	44122196	ENSMUSG00000017740	ENSMUSG00000017740	ENSMUSG00000017740	2	164659524	164690936	49	51
2439	20	44123031	44151998	ENSMUSG00000039804	ENSMUSG00000039804	ENSMUSG00000039804	2	164691562	164725984	49	51
2440	20	44180318	44191909	ENSMUSG00000017652	ENSMUSG00000017652	ENSMUSG00000017652	2	164746841	164762859	49	51
2441	20	44235779	44313741	ENSMUSG00000053166	ENSMUSG00000053166	ENSMUSG00000053166	2	164802949	164925924	49	51
2442	20	44411574	44426441	ENSMUSG00000017664	ENSMUSG00000017664	ENSMUSG00000017664	2	164967759	164979032	49	51
2443	20	44428101	44468678	ENSMUSG00000017670	ENSMUSG00000017670	ENSMUSG00000017670	2	164982317	165008098	49	51
2444	20	44563116	44568389	ENSMUSG00000017667	ENSMUSG00000017667	ENSMUSG00000017667	2	165068641	165079464	49	51
2445	20	44603320	44608374	ENSMUSG00000027670	ENSMUSG00000027670	ENSMUSG00000027670	2	165086655	165091599	49	51
2446	20	44619870	44713505	ENSMUSG00000018459	ENSMUSG00000018459	ENSMUSG00000018459	2	165096492	165164402	49	51
2447	20	44771533	44798390	ENSMUSG00000027661	ENSMUSG00000027661	ENSMUSG00000027661	2	165195135	165211114	49	51
2448	20	44956670	45250899	ENSMUSG00000017897	ENSMUSG00000017897	ENSMUSG00000017897	2	165321550	165462835	49	51
2449	20	45645347	45715724	ENSMUSG00000027678	ENSMUSG00000027678	ENSMUSG00000027678	2	165683882	165762141	49	51
2450	20	45719063	45848737	ENSMUSG00000006800	ENSMUSG00000006800	ENSMUSG00000006800	2	165765120	165846868	49	51
2451	20	46674197	46877827	ENSMUSG00000039621	ENSMUSG00000039621	ENSMUSG00000039621	2	166257550	166405037	49	51
2452	20	46971834	47086637	ENSMUSG00000074583	ENSMUSG00000074583	ENSMUSG00000074583	2	166496895	166551603	49	51
2453	20	47096245	47146891	ENSMUSG00000002718	ENSMUSG00000002718	ENSMUSG00000002718	2	166597301	166637472	49	51
2454	20	47163285	47238311	ENSMUSG00000039536	ENSMUSG00000039536	ENSMUSG00000039536	2	166639352	166687441	49	51
2455	20	47269291	47294021	ENSMUSG00000017999	ENSMUSG00000017999	ENSMUSG00000017999	2	166706539	166726139	49	51
2456	20	47295841	47328009	ENSMUSG00000039501	ENSMUSG00000039501	ENSMUSG00000039501	2	166727933	166751297	49	51
2457	20	47418353	47532591	ENSMUSG00000050556	ENSMUSG00000050556	ENSMUSG00000050556	2	166794583	166880004	49	51
2458	20	47553818	47618114	ENSMUSG00000017969	ENSMUSG00000017969	ENSMUSG00000017969	2	166883022	166931749	49	51
2459	20	47682889	47763822	ENSMUSG00000017929	ENSMUSG00000017929	ENSMUSG00000017929	2	166992266	167040292	49	51
2460	20	47862657	47942179	ENSMUSG00000039463	ENSMUSG00000039463	ENSMUSG00000039463	2	167112926	167168203	49	51
2461	20	47953335	47965452	ENSMUSG00000047030	ENSMUSG00000047030	ENSMUSG00000047030	2	167172600	167184068	49	51
2462	20	47986321	48003827	ENSMUSG00000006418	ENSMUSG00000006418	ENSMUSG00000006418	2	167194641	167207376	49	51
2463	20	48240678	48242633	ENSMUSG00000056501	ENSMUSG00000056501	ENSMUSG00000056501	2	167380120	167381623	49	51
2464	20	48560294	48634706	ENSMUSG00000027540	ENSMUSG00000027540	ENSMUSG00000027540	2	167623614	167668115	49	51
2465	20	48781488	48803684	ENSMUSG00000044641	ENSMUSG00000044641	ENSMUSG00000044641	2	167772490	167790415	49	51
2466	20	48938992	48980919	ENSMUSG00000051149	ENSMUSG00000051149	ENSMUSG00000051149	2	167872194	167901826	49	51
2467	20	49008570	49010625	ENSMUSG00000074576	ENSMUSG00000074576	ENSMUSG00000074576	2	167921846	167923222	49	51
2468	20	49053600	49073051	ENSMUSG00000074575	ENSMUSG00000074575	ENSMUSG00000074575	2	167953585	167960249	49	51
2469	20	49441083	49592665	ENSMUSG00000027544	ENSMUSG00000027544	ENSMUSG00000027544	2	168167615	168281488	49	51
2470	20	49650835	49818580	ENSMUSG00000027546	ENSMUSG00000027546	ENSMUSG00000027546	2	168325668	168433169	49	51
2471	20	49833988	49852421	ENSMUSG00000027547	ENSMUSG00000027547	ENSMUSG00000027547	2	168440991	168458333	49	51
2472	20	50201224	50241932	ENSMUSG00000027551	ENSMUSG00000027551	ENSMUSG00000027551	2	168616868	168642987	49	51
2473	20	51022353	51541016	ENSMUSG00000047907	ENSMUSG00000047907	ENSMUSG00000047907	2	169324376	169604299	49	51
2474	20	51617011	51633043	ENSMUSG00000052056	ENSMUSG00000052056	ENSMUSG00000052056	2	169801284	169822425	49	51
2475	20	51993487	52120490	ENSMUSG00000013523	ENSMUSG00000013523	ENSMUSG00000013523	2	170038353	170119033	49	51
2476	20	52203395	52223931	ENSMUSG00000038567	ENSMUSG00000038567	ENSMUSG00000038567	2	170174170	170188343	49	51
2477	20	52257909	52269898	ENSMUSG00000052033	ENSMUSG00000052033	ENSMUSG00000052033	2	170202643	170210068	49	51
2478	20	52525588	52701117	ENSMUSG00000027560	ENSMUSG00000027560	ENSMUSG00000027560	2	170423145	170570973	49	51
2479	20	54005904	54013937	ENSMUSG00000067578	ENSMUSG00000067578	ENSMUSG00000067578	2	171727434	171734574	49	51
2480	20	54257195	54258278	ENSMUSG00000038537	ENSMUSG00000038537	ENSMUSG00000038537	2	171939697	171942319	49	51
2481	20	54367425	54377126	ENSMUSG00000027495	ENSMUSG00000027495	ENSMUSG00000027495	2	172036814	172046954	49	51
2482	20	54377852	54400758	ENSMUSG00000027496	ENSMUSG00000027496	ENSMUSG00000027496	2	172047401	172061711	49	51
2483	20	54400834	54412989	ENSMUSG00000027498	ENSMUSG00000027498	ENSMUSG00000027498	2	172062208	172072291	49	51
2484	20	54420575	54467210	ENSMUSG00000074570	ENSMUSG00000074570	ENSMUSG00000074570	2	172084999	172120759	49	51
2485	20	54477092	54527345	ENSMUSG00000027502	ENSMUSG00000027502	ENSMUSG00000027502	2	172131783	172161104	49	51
2486	20	54499955	54534388	ENSMUSG00000074569	ENSMUSG00000074569	ENSMUSG00000074569	2	172141518	172149801	49	51
2487	20	54532955	54534605	ENSMUSG00000027505	ENSMUSG00000027505	ENSMUSG00000027505	2	172163753	172165499	49	51
2488	20	54637765	54647743	ENSMUSG00000028640	ENSMUSG00000028640	ENSMUSG00000028640	2	172242262	172249031	49	51
2489	20	55177211	55275091	ENSMUSG00000008999	ENSMUSG00000008999	ENSMUSG00000008999	2	172512625	172583232	49	51
2490	20	55338222	55352450	ENSMUSG00000005883	ENSMUSG00000005883	ENSMUSG00000005883	2	172622784	172636506	49	51
2491	20	55399870	55417786	ENSMUSG00000027510	ENSMUSG00000027510	ENSMUSG00000027510	2	172664868	172677673	49	51
2492	20	55505634	55533591	ENSMUSG00000070495	ENSMUSG00000070495	ENSMUSG00000070495	2	172737606	172761727	49	51
2493	20	55569543	55574922	ENSMUSG00000027513	ENSMUSG00000027513	ENSMUSG00000027513	2	172796012	172802209	49	51
2494	20	55615515	55628935	ENSMUSG00000027514	ENSMUSG00000027514	ENSMUSG00000027514	2	172849551	172861829	49	51
2495	20	55656860	55718437	ENSMUSG00000038400	ENSMUSG00000038400	ENSMUSG00000038400	2	172870262	172919168	49	51
2496	20	56159388	56169612	ENSMUSG00000027518	ENSMUSG00000027518	ENSMUSG00000027518	2	173165539	173171435	49	51
2497	20	56226957	56237091	ENSMUSG00000027517	ENSMUSG00000027517	ENSMUSG00000027517	2	173211604	173221094	49	51
2498	20	56318177	56375966	ENSMUSG00000027519	ENSMUSG00000027519	ENSMUSG00000027519	2	173302800	173347611	49	51
2499	20	56467563	56523355	ENSMUSG00000070494	ENSMUSG00000070494	ENSMUSG00000070494	2	173436505	173442534	49	51
2500	20	56701363	56727701	ENSMUSG00000039263	ENSMUSG00000039263	ENSMUSG00000039263	2	173753304	173765641	49	51
2501	20	56862098	56919640	ENSMUSG00000027523	ENSMUSG00000027523	ENSMUSG00000027523	2	173940798	173989447	49	51
2502	20	57027704	57035104	ENSMUSG00000016255	ENSMUSG00000016255	ENSMUSG00000016255	2	174093684	174100803	49	51
2503	20	57043407	57051262	ENSMUSG00000016257	ENSMUSG00000016257	ENSMUSG00000016257	2	174108030	174115880	49	51
2504	20	57200603	57267563	ENSMUSG00000050600	ENSMUSG00000050600	ENSMUSG00000050600	2	174288219	174348851	49	51
2505	20	57308877	57334441	ENSMUSG00000027524	ENSMUSG00000027524	ENSMUSG00000027524	2	174403712	174426981	49	51
2506	20	57612998	57856161	ENSMUSG00000027525	ENSMUSG00000027525	ENSMUSG00000027525	2	178071341	178265480	49	51
2507	20	57945118	57948621	ENSMUSG00000049999	ENSMUSG00000049999	ENSMUSG00000049999	2	178342776	178343615	49	51
2508	20	57942214	57955954	ENSMUSG00000070476	ENSMUSG00000070476	ENSMUSG00000070476	2	178349680	178350816	49	51
2509	20	57966877	58042126	ENSMUSG00000039155	ENSMUSG00000039155	ENSMUSG00000039155	2	178360015	178416774	49	51
2510	20	59260954	59945672	ENSMUSG00000000305	ENSMUSG00000000305	ENSMUSG00000000305	2	179372030	179825326	49	51
2511	20	59983247	60074261	ENSMUSG00000039117	ENSMUSG00000039117	ENSMUSG00000039117	2	179842853	179869372	49	51
2512	20	60130928	60141912	ENSMUSG00000039108	ENSMUSG00000039108	ENSMUSG00000039108	2	179954395	179964867	49	51
2513	20	60145186	60151881	ENSMUSG00000027566	ENSMUSG00000027566	ENSMUSG00000027566	2	179965784	179971816	49	51
2514	20	60152233	60188733	ENSMUSG00000039086	ENSMUSG00000039086	ENSMUSG00000039086	2	179971917	179999609	49	51
2515	20	60191496	60211203	ENSMUSG00000039069	ENSMUSG00000039069	ENSMUSG00000039069	2	180008031	180015179	49	51
2516	20	60223421	60228706	ENSMUSG00000039059	ENSMUSG00000039059	ENSMUSG00000039059	2	180028881	180033632	49	51
2517	20	60246975	60304664	ENSMUSG00000039050	ENSMUSG00000039050	ENSMUSG00000039050	2	180048781	180091868	49	51
2518	20	60317516	60375763	ENSMUSG00000015647	ENSMUSG00000015647	ENSMUSG00000015647	2	180105853	180155210	49	51
2519	20	60397087	60415728	ENSMUSG00000038990	ENSMUSG00000038990	ENSMUSG00000038990	2	180188286	180202872	49	51
2520	20	60418688	60435984	ENSMUSG00000038980	ENSMUSG00000038980	ENSMUSG00000038980	2	180207054	180219287	49	51
2521	20	60471948	60483972	ENSMUSG00000015627	ENSMUSG00000015627	ENSMUSG00000015627	2	180254541	180264081	49	51
2522	20	60744313	60774079	ENSMUSG00000038963	ENSMUSG00000038963	ENSMUSG00000038963	2	180385742	180404258	49	51
2523	20	60810634	60864568	ENSMUSG00000027568	ENSMUSG00000027568	ENSMUSG00000027568	2	180429384	180474387	49	51
2524	20	60898250	60902390	ENSMUSG00000027569	ENSMUSG00000027569	ENSMUSG00000027569	2	180512216	180515698	49	51
2525	20	60906632	60915797	ENSMUSG00000049401	ENSMUSG00000049401	ENSMUSG00000049401	2	180518869	180525239	49	51
2526	20	60918832	60942955	ENSMUSG00000027570	ENSMUSG00000027570	ENSMUSG00000027570	2	180527630	180551593	49	51
2527	20	60942913	60963560	ENSMUSG00000038932	ENSMUSG00000038932	ENSMUSG00000038932	2	180551364	180571997	49	51
2528	20	60979535	61039719	ENSMUSG00000038914	ENSMUSG00000038914	ENSMUSG00000038914	2	180587372	180631508	49	51
2529	20	61039886	61050268	ENSMUSG00000027573	ENSMUSG00000027573	ENSMUSG00000027573	2	180639675	180648141	49	51
2530	20	61054497	61070394	ENSMUSG00000023393	ENSMUSG00000023393	ENSMUSG00000023393	2	180654792	180671686	49	51
2531	20	61107724	61108527	ENSMUSG00000045493	ENSMUSG00000045493	ENSMUSG00000045493	2	180705377	180706157	49	51
2532	20	61297226	61318023	ENSMUSG00000038848	ENSMUSG00000038848	ENSMUSG00000038848	2	180833785	180850344	49	51

2533	20	61337680	61342291	ENSMUSG00000038840	ENSMUSG00000038840	ENSMUSG00000038840	2	180858431	180864820	49	51
2534	20	61342581	61356337	ENSMUSG00000027574	ENSMUSG00000027574	ENSMUSG00000027574	2	180864180	180884107	49	51
2535	20	61374610	61391587	ENSMUSG00000027575	ENSMUSG00000027575	ENSMUSG00000027575	2	180896673	180911866	49	51
2536	20	61395021	61432728	ENSMUSG00000016356	ENSMUSG00000016356	ENSMUSG00000016356	2	180927990	180945240	49	51
2537	20	61445864	61463183	ENSMUSG00000027577	ENSMUSG00000027577	ENSMUSG00000027577	2	180951719	180968585	49	51
2538	20	61502013	61574437	ENSMUSG00000016346	ENSMUSG00000016346	ENSMUSG00000016346	2	181004987	181064500	49	51
2539	20	61589810	61600949	ENSMUSG00000016349	ENSMUSG00000016349	ENSMUSG00000016349	2	181077098	181086422	49	51
2540	20	61622572	61623735	ENSMUSG00000016344	ENSMUSG00000016344	ENSMUSG00000016344	2	181116712	181117907	49	51
2541	20	61630222	61639151	ENSMUSG00000038751	ENSMUSG00000038751	ENSMUSG00000038751	2	181124531	181132196	49	51
2542	20	61642607	61649301	ENSMUSG00000027579	ENSMUSG00000027579	ENSMUSG00000027579	2	181134970	181142578	49	51
2543	20	61654817	61658505	ENSMUSG00000047841	ENSMUSG00000047841	ENSMUSG00000047841	2	181149423	181152273	49	51
2544	20	61659884	61676033	ENSMUSG00000027580	ENSMUSG00000027580	ENSMUSG00000027580	2	181162845	181170405	49	51
2545	20	61689399	61728838	ENSMUSG00000038705	ENSMUSG00000038705	ENSMUSG00000038705	2	181180858	181217373	49	51
2546	20	61760091	61800495	ENSMUSG00000038685	ENSMUSG00000038685	ENSMUSG00000038685	2	181249169	181286023	49	51
2547	20	61800447	61809454	ENSMUSG00000038671	ENSMUSG00000038671	ENSMUSG00000038671	2	181287240	181294768	49	51
2548	20	61809835	61837938	ENSMUSG00000027582	ENSMUSG00000027582	ENSMUSG00000027582	2	181294832	181312570	49	51
2549	20	61845463	61933011	ENSMUSG00000027583	ENSMUSG00000027583	ENSMUSG00000027583	2	181320293	181353762	49	51
2550	20	61963010	61965103	ENSMUSG00000055882	ENSMUSG00000055882	ENSMUSG00000055882	2	181422617	181424384	49	51
2551	20	61967034	61993332	ENSMUSG00000000827	ENSMUSG00000000827	ENSMUSG00000000827	2	181426660	181446166	49	51
2552	20	61996979	62037828	ENSMUSG00000000826	ENSMUSG00000000826	ENSMUSG00000000826	2	181449912	181481656	49	51
2553	20	62041637	62058214	ENSMUSG00000010492	ENSMUSG00000010492	ENSMUSG00000010492	2	181498559	181511380	49	51
2554	20	62058499	62140382	ENSMUSG00000000823	ENSMUSG00000000823	ENSMUSG00000000823	2	181511542	181521868	49	51
2555	20	62075910	62081439	ENSMUSG00000038605	ENSMUSG00000038605	ENSMUSG00000038605	2	181525114	181528604	49	51
2556	20	62082901	62134893	ENSMUSG00000002455	ENSMUSG00000002455	ENSMUSG00000002455	2	181530792	181585066	49	51
2557	20	62149520	62151438	ENSMUSG00000046470	ENSMUSG00000046470	ENSMUSG00000046470	2	181599251	181601044	49	51
2558	20	62164454	62174143	ENSMUSG00000059540	ENSMUSG00000059540	ENSMUSG00000059540	2	181609723	181617465	49	51
2559	20	62174981	62181767	ENSMUSG00000002458	ENSMUSG00000002458	ENSMUSG00000002458	2	181617829	181623330	49	51
2560	20	62185177	62186156	ENSMUSG00000045794	ENSMUSG00000045794	ENSMUSG00000045794	2	181626201	181627848	49	51
2561	20	62181932	62202440	ENSMUSG00000027584	ENSMUSG00000027584	ENSMUSG00000027584	2	181644448	181650381	49	51
2562	20	62207617	62208968	ENSMUSG00000042892	ENSMUSG00000042892	ENSMUSG00000042892	2	181653460	181654396	49	51
2563	20	62266271	62344048	ENSMUSG00000010505	ENSMUSG00000010505	ENSMUSG00000010505	2	181718928	181755450	49	51
2564	20	62357565	62378023	ENSMUSG00000027589	ENSMUSG00000027589	ENSMUSG00000027589	2	181767309	181786858	49	51
2565	8	76482826	76639117	ENSMUSG00000017688	ENSMUSG00000017688	ENSMUSG00000017688	3	3493753	3643025	50	52
2566	8	77778835	77939356	ENSMUSG00000025255	ENSMUSG00000025255	ENSMUSG00000025255	3	5224789	5396220	50	52
2567	8	78057748	78074907	ENSMUSG00000040374	ENSMUSG00000040374	ENSMUSG00000040374	3	5543573	5559312	50	52
2568	8	79673149	79677999	ENSMUSG00000027499	ENSMUSG00000027499	ENSMUSG00000027499	3	7349743	7428439	50	52
2569	8	79740885	79792489	ENSMUSG00000043542	ENSMUSG00000043542	ENSMUSG00000043542	3	7486563	7536904	50	52
2570	8	79807564	79880313	ENSMUSG00000040329	ENSMUSG00000040329	ENSMUSG00000040329	3	7556913	7587247	50	52
2571	8	80711591	80740931	ENSMUSG00000027500	ENSMUSG00000027500	ENSMUSG00000027500	3	8492600	8544677	50	52
2572	8	80838801	80842560	ENSMUSG00000040289	ENSMUSG00000040289	ENSMUSG00000040289	3	8646435	8650112	50	52
2573	8	80993653	81105061	ENSMUSG00000040269	ENSMUSG00000040269	ENSMUSG00000040269	3	8785230	8906930	50	52
2574	8	81109662	81246425	ENSMUSG00000027506	ENSMUSG00000027506	ENSMUSG00000027506	3	8912509	8947127	50	52
2575	8	81561003	81595322	ENSMUSG00000069114	ENSMUSG00000069114	ENSMUSG00000069114	3	9247633	9264896	50	52
2576	8	81716159	81948834	ENSMUSG00000040209	ENSMUSG00000040209	ENSMUSG00000040209	3	9421364	9592792	50	52
2577	8	82042605	82186858	ENSMUSG00000027508	ENSMUSG00000027508	ENSMUSG00000027508	3	9674971	9816162	50	52
2578	8	82515121	82522274	ENSMUSG00000052468	ENSMUSG00000052468	ENSMUSG00000052468	3	10162380	10166417	50	52
2579	8	82533173	82536313	ENSMUSG00000027528	ENSMUSG00000027528	ENSMUSG00000027528	3	10176153	10179754	50	52
2580	8	82599863	82606105	ENSMUSG00000027530	ENSMUSG00000027530	ENSMUSG00000027530	3	10228451	10236784	50	52
2581	8	82732746	82761110	ENSMUSG00000027531	ENSMUSG00000027531	ENSMUSG00000027531	3	10296072	10313904	50	52
2582	8	82768446	82769762	ENSMUSG00000058921	ENSMUSG00000058921	ENSMUSG00000058921	3	10316826	10318130	50	52
2583	8	82807243	82834305	ENSMUSG00000027536	ENSMUSG00000027536	ENSMUSG00000027536	3	10349505	10373537	50	52
2584	8	82874377	82916990	ENSMUSG00000027534	ENSMUSG00000027534	ENSMUSG00000027534	3	10400679	10422619	50	52
2585	8	85604112	85962566	ENSMUSG00000039717	ENSMUSG00000039717	ENSMUSG00000039717	3	13759380	14120190	50	52
2586	8	86206702	86245562	ENSMUSG00000027550	ENSMUSG00000027550	ENSMUSG00000027550	3	14510604	14545045	50	52
2587	8	86276874	86314002	ENSMUSG00000027552	ENSMUSG00000027552	ENSMUSG00000027552	3	14555415	14583053	50	52
2588	8	86345259	86383554	ENSMUSG00000027555	ENSMUSG00000027555	ENSMUSG00000027555	3	14618518	14639140	50	52
2589	8	86427089	86478495	ENSMUSG00000027556	ENSMUSG00000027556	ENSMUSG00000027556	3	14639664	14755169	50	52
2590	8	86537710	86548526	ENSMUSG00000027559	ENSMUSG00000027559	ENSMUSG00000027559	3	14840253	14849276	50	52
2591	8	86563433	86580945	ENSMUSG00000027562	ENSMUSG00000027562	ENSMUSG00000027562	3	14863450	14877513	50	52
2592	8	64243991	64262863	ENSMUSG00000047213	ENSMUSG00000047213	ENSMUSG00000047213	3	16375648	16397581	51	53
2593	8	65655368	65658735	ENSMUSG00000025128	ENSMUSG00000025128	ENSMUSG00000025128	3	18246476	18249660	51	53
2594	8	65671246	65873902	ENSMUSG00000039519	ENSMUSG00000039519	ENSMUSG00000039519	3	18264101	18435480	51	53
2595	8	66677630	66708986	ENSMUSG00000027599	ENSMUSG00000027599	ENSMUSG00000027599	3	19324295	19355216	51	53
2596	8	66719528	66785339	ENSMUSG00000027601	ENSMUSG00000027601	ENSMUSG00000027601	3	19380395	19412956	51	53
2597	8	66793867	66863886	ENSMUSG00000069094	ENSMUSG00000069094	ENSMUSG00000069094	3	19418193	19503473	51	53
2598	8	67096349	67174920	ENSMUSG00000027606	ENSMUSG00000027606	ENSMUSG00000027606	3	19700777	19803012	51	53
2599	8	67201832	67250270	ENSMUSG00000060913	ENSMUSG00000060913	ENSMUSG00000060913	3	19836611	19854019	51	53
2600	8	67251166	67253380	ENSMUSG00000049796	ENSMUSG00000049796	ENSMUSG00000049796	3	19885565	19887480	51	53
2601	3	150374067	150422269	ENSMUSG00000003617	ENSMUSG00000003617	ENSMUSG00000003617	3	20149205	20181903	52	54
2602	3	150330061	150373995	ENSMUSG00000027615	ENSMUSG00000027615	ENSMUSG00000027615	3	20188102	20227435	52	54
2603	3	150230604	150287007	ENSMUSG00000002428	ENSMUSG00000002428	ENSMUSG00000002428	3	20249962	20310527	52	54
2604	3	150192024	150228074	ENSMUSG00000019528	ENSMUSG00000019528	ENSMUSG00000019528	3	20314728	20344903	52	54
2605	3	150065771	150097558	ENSMUSG00000001865	ENSMUSG00000001865	ENSMUSG00000001865	3	20407667	20434231	52	54
2606	3	150028131	150060633	ENSMUSG00000011463	ENSMUSG00000011463	ENSMUSG00000011463	3	20441534	20466802	52	54
2607	3	178226831	178397734	ENSMUSG00000027630	ENSMUSG00000027630	ENSMUSG00000027630	3	22267857	22402606	53	55
2608	3	176297261	177006120	ENSMUSG00000074666	ENSMUSG00000074666	ENSMUSG00000074666	3	24193970	24624288	53	55
2609	3	174805083	175481787	ENSMUSG00000063887	ENSMUSG00000063887	ENSMUSG00000063887	3	25624711	26324807	53	55
2610	3	174089842	174341700	ENSMUSG00000039335	ENSMUSG00000039335	ENSMUSG00000039335	3	26828704	27174287	53	55
2611	3	173951207	174021957	ENSMUSG00000027699	ENSMUSG00000027699	ENSMUSG00000027699	3	27288296	27344897	53	55
2612	3	173831130	173911702	ENSMUSG00000027698	ENSMUSG00000027698	ENSMUSG00000027698	3	27374077	27435984	53	55
2613	3	173706920	173723963	ENSMUSG00000039304	ENSMUSG00000039304	ENSMUSG00000039304	3	27508150	27530738	53	55
2614	3	173645645	173648897	ENSMUSG00000051136	ENSMUSG00000051136	ENSMUSG00000051136	3	27562424	27569083	53	55
2615	3	173241080	173601181	ENSMUSG00000039286	ENSMUSG00000039286	ENSMUSG00000039286	3	27607235	27901512	53	55
2616	3	173043844	173054352	ENSMUSG00000043164	ENSMUSG00000043164	ENSMUSG00000043164	3	28075866	28087440	53	55
2617	3	172801314	173010929	ENSMUSG00000027695	ENSMUSG00000027695	ENSMUSG00000027695	3	28129768	28323123	53	55
2618	3	172262986	172660812	ENSMUSG00000027692	ENSMUSG00000027692	ENSMUSG00000027692	3	28454287	28861657	53	55
2619	3	172196831	172227462	ENSMUSG00000027690	ENSMUSG00000027690	ENSMUSG00000027690	3	28894546	28919403	53	55
2620	3	172088898	172109120	ENSMUSG00000050192	ENSMUSG00000050192	ENSMUSG00000050192	3	28972384	28989921	53	55
2621	3	170285244	170346787	ENSMUSG00000027684	ENSMUSG00000027684	ENSMUSG00000027684	3	30142380	30331261	54	56
2622	3	170581663	170582025	ENSMUSG00000051636	ENSMUSG00000051636	ENSMUSG00000051636	3	30428810	30429157	54	56
2623	3	170967407	170970377	ENSMUSG00000037737	ENSMUSG00000037737	ENSMUSG00000037737	3	30787911	30790708	54	56
2624	3	170973547	170988027	ENSMUSG00000037730	ENSMUSG00000037730	ENSMUSG00000037730	3	30792925	30809345	54	56
2625	3	170993960	171013025	ENSMUSG00000027702	ENSMUSG00000027702	ENSMUSG00000027702	3	30815114	30838707	54	56
2626	3	171022395	171038092	ENSMUSG00000027703	ENSMUSG00000027703	ENSMUSG00000027703	3	30836168	30863269	54	56
2627	3	171039661	171070347	ENSMUSG00000074653	ENSMUSG00000074653	ENSMUSG00000074653	3	30869878	30886466	54	56
2628	3	171112054	171139640	ENSMUSG00000051860	ENSMUSG00000051860	ENSMUSG00000051860	3	30937131	30958004	54	56
2629	3	171167274	171198855	ENSMUSG00000027706	ENSMUSG00000027706	ENSMUSG00000027706	3	30983714	31012101	54	56
2630	3	171238432	171285504	ENSMUSG00000037661	ENSMUSG00000037661	ENSMUSG00000037661	3	31046827	31088036	54	56
2631	3	171297378	171382210	ENSMUSG00000037652	ENSMUSG00000037652	ENSMUSG00000037652	3	31097687	31160279	54	56
2632	3	171422919	171506458	ENSMUSG00000037643	ENSMUSG00000037643	ENSMUSG00000037643	3	31186625	31243615	54	56
2633	3	171560814	171593226	ENSMUSG00000027660	ENSMUSG00000027660	ENSMUSG00000027660	3	31288209	31313455	54	56
2634	3	171619359	171634577	ENSMUSG00000037625	ENSMUSG00000037625	ENSMUSG00000037625	3	31340824	31355199	54	56
2635	3	171667255	171786552	ENSMUSG00000069072	ENSMUSG00000069072	ENSMUSG00000069072	3	31393788	31501173	54	56
2636	3	180007823	180044899	ENSMUSG00000037610	ENSMUSG00000037610	ENSMUSG00000037610	3	32302720	32390643	55	57
2637	3	180224221	180272278	ENSMUSG00000027663	ENSMUSG00000027663	ENSMUSG00000027663	3	32533033	32552708	55	57
2638	3	180349005	180435189	ENSMUSG00000027665	ENSMUSG00000027665	ENSMUSG00000027665	3	32627755	32654380	55	57
2639	3	180443259	180451585	ENSMUSG00000037574	ENSMUSG00000037574	ENSMUSG00000037574	3	32663870	32674027	55	57
2640	3	180528776	180534904	ENSMUSG00000027667	ENSMUSG00000027667	ENSMUSG00000027667	3	32706844	32712234	55	57
2641	3	180548174	180593700	ENSMUSG00000027668	ENSMUSG00000027668	ENSMUSG00000027668	3	32721043	32770774	55	57
2642	3	180599696	180652065	ENSMUSG00000027669	ENSMUSG00000027669	ENSMUSG00000027669	3	32775075	32808084	55	57
2643	3	180763575	180788886	ENSMUSG00000027671	ENSMUSG00000027671	ENSMUSG00000027671	3	32900277	32918517	55	57
2644	3	180853635	180984675	ENSMUSG00000056900	ENSMUSG00000056900	ENSMUSG00000056900	3	33009175	33126811	55	57
2645	3	181000744	181237211	ENSMUSG00000027674	ENSMUSG00000027674	ENSMUSG00000027674	3	33142161	33274578	55	57
2646	3	181802654	181811453	ENSMUSG00000027677	ENSMUSG00000027677	ENSMUSG00000027677	3	33991758	33999423	55	57
2647	3	181831936	181909307	ENSMUSG00000027676	ENSMUSG00000027676	ENSMUSG00000027676	3	34003911	34035850	55	57
2648	3	182113146	182177642	ENSMUSG00000027680	ENSMUSG00000027680	ENSMUSG00000027680	3	34211657	34260894	55	57
2649	3	182912416	182914915	ENSMUSG00000074637	ENSMUSG00000074637	ENSMUSG00000074637	3	34841954	34842939	55	57
2650	3	183993985	184122113	ENSMUSG00000037400	ENSMUSG00000037400	ENSMUSG00000037400	3	35969574	36046735	55	57
2651	3	184215702	184300055	ENSMUSG00000027709	ENSMUSG00000027709	ENSMUSG00000027709	3	36150861	36192158	55	57
2652	4	122808598	122837626	ENSMUSG00000027712	ENSMUSG00000027712	ENSMUSG00000027712	3	36640893	36656884	56	58
2653	4	122941928	122957625	ENSMUSG00000027714	ENSMUSG00000027714	ENSMUSG00000027714	3	36744176	36757274	56	58
2654	4	122958002	122964505	ENSMUSG00000027715	ENSMUSG00000027715	ENSMUSG00000027715	3	36756415	36763547	56	58
2655	4	122965089	123011092	ENSMUSG00000037325	ENSMUSG00000037325	ENSMUSG00000037325	3	36764695	36804897	56	58
2656	4	123019633	123092359	ENSMUSG00000027716	ENSMUSG00000027716	ENSMUSG00000027716	3	36812031	36881716	56	58
2657	4	123326786	123380534	ENSMUSG00000037291	ENSMUSG00000037291	ENSMUSG00000037291	3	37054705	37139701	56	58
2658	4	123391011	123502849	ENSMUSG00000037270	ENSMUSG00000037270	ENSMUSG00000037270	3	37178518	37244086	56	58
2659	4	123519618	123570389	ENSMUSG00000027719	ENSMUSG00000027719	ENSMUSG00000027719	3	37255218	37303061	56	58
2660	4	123592080	123597339	ENSMUSG00000027720	ENSMUSG00000027720	ENSMUSG00000027720	3	37312271	37317502	56	58
2661	4	123753221	123761662	ENSMUSG00000027718	ENSMUSG00000027718	ENSMUSG00000027718	3	37414308	37424167	56	58
2662	4	123873307	123885547	ENSMUSG00000051444	ENSMUSG00000051444	ENSMUSG00000051444	3	37510615	37512735	56	58
2663	4	123967313	124038840	ENSMUSG00000037225	ENSMUSG00000037225	ENSMUSG00000037225	3	37540399	37596346	56	58
2664	4	124033256	124063177	ENSMUSG00000050174	ENSMUSG00000050174	ENSMUSG00000050174	3	37596531	37611119	56	58
2665	4	124063679	124455252	ENSMUSG00000027722	ENSMUSG00000027722	ENSMUSG00000027722	3	37611847	37770640	56	58
2666	4	124537406	124544357	ENSMUSG00000037211	ENSMUSG00000037211	ENSMUSG00000037211	3	37831507	37836147	56	58
2667	4	125804916	125851382	ENSMUSG00000044864	ENSMUSG00000044864	ENSMUSG00000044864	3	38641200	38647396	56	58
2668	4	128773562	128857374	ENSMUSG00000060798	ENSMUSG00000060798	ENSMUSG00000060798	3	40732336	40801289	56	58
2669	4	128871024	128914896	ENSMUSG00000069041	ENSMUSG00000069041	ENSMUSG00000069041	3	40805420	40822141	56	58
2670	4	128922903	128973974	ENSMUSG00000025757	ENSMUSG00000025757	ENSMUSG00000025757	3	40842162	40886931	56	58
2671	4	129021551	129039377	ENSMUSG00000025758	ENSMUSG00000025758	ENSMUSG00000025758	3	40896590	40913430	56	58
2672	4	129061027	129106554	ENSMUSG00000025759	ENSMUSG00000025759	ENSMUSG00000025759	3	40914619	40943365	56	58
2673	4	129123561	129176415	ENSMUSG00000037818	ENSMUSG00000037818	ENSMUSG00000037818	3	40990871	41033686	56	58
2674	4	129409861	129428395	ENSMUSG00000049940	ENSMUSG00000049940	ENSMUSG00000049940	3	41162890	41179536	56	58
2675	4	129972364	130012867	ENSMUSG00000025764	ENSMUSG00000025764	ENSMUSG00000025764	3	41652281	41711499	56	58
2676	4	130024837	130234212	ENSMUSG00000059834	ENSMUSG00000059834	ENSMUSG00000059834	3	41768003	41839030	56	58
2677	4	130234290	130253258	ENSMUSG00000025766	ENSMUSG00000025766	ENSMUSG00000025766	3	41843097	41852682	56	58
2678	4	134289920	134332181	ENSMUSG00000049100	ENSMUSG00000049100	ENSMUSG00000049100	3	45475274	45489074	56	58
2679	4	138660332	138672692	ENSMUSG00000037892	ENSMUSG00000037892	ENSMUSG00000037892	3	49839482	49853476	56	58
2680	4	139304698	139382953	ENSMUSG00000027737	ENSMUSG00000027737	ENSMUSG00000027737	3	50461131	50539808	56	58
2681	4	140156393	140186541	ENSMUSG00000023087	ENSMUSG00000023087	ENSMUSG00000023087	3	51312390	51339545	56	58
2682	4	140198321	140280056	ENSMUSG00000037174	ENSMUSG00000037174	ENSMUSG00000037174	3	51343594	51413916	56	58
2683	4	140407121	140420942	ENSMUSG00000037161	ENSMUSG00000037161	ENSMUSG00000037161	3	51476344	51484469	56	58
2684	4	140430542	140436408	ENSMUSG00000037152	ENSMUSG00000037152	ENSMUSG00000037152	3	51492606	51496867	56	58
2685	4	140474778	140531239	ENSMUSG00000063273	ENSMUSG00000063273	ENSMUSG00000063273	3	51504055	51561960	56	58
2686	4	140594411	140616511	ENSMUSG00000027739	ENSMUSG00000027739	ENSMUSG00000027739	3	51571895	51583833	56	58
2687	4	140646642	140697027	ENSMUSG00000037111	ENSMUSG00000037111	ENSMUSG00000037111	3	51603247	51648752	56	58
2688	4	140806372	140844857	ENSMUSG00000074604	ENSMUSG00000074604	ENSMUSG00000074604	3	51749178	51765566	56	58
2689	4	140859927	141031580	ENSMUSG00000061143	ENSMUSG00000061143	ENSMUSG00000061143	3	51777810	52192162	56	58
2690	13	40027801	40138734	ENSMUSG00000044167	ENSMUSG00000044167	ENSMUSG00000044167	3	52356303	52437467	57	59
2691	13	39127814	39224629	ENSMUSG00000027742	ENSMUSG00000027742	ENSMUSG00000027742	3	53070052	53105145	57	59
2692	13	38815029	39075308	ENSMUSG00000048332	ENSMUSG00000048332	ENSMUSG00000048332	3	53129476	53349608	57	59
2693	13	38159482	38352924	ENSMUSG00000037016	ENSMUSG00000037016	ENSMUSG00000037016	3	53512220	53745013	57	59
2694	13	38510455	38522246	ENSMUSG00000042997	ENSMUSG00000042997	ENSMUSG00000042997	3	53539925	53551187	57	59
2695	13	38482003	38510213	ENSMUSG00000049504	ENSMUSG00000049504	ENSMUSG00000049504	3	53551595	53569684	57	59
2696	13	38438062	38462983	ENSMUSG00000027744	ENSMUSG00000027744	ENSMUSG00000027744	3	53576676	53596431	57	59
2697	13	37821988	37833262	ENSMUSG00000027746	ENSMUSG00000027746	ENSMUSG00000027746	3	53943141	53951703	57	59
2698	13	37108980	37255470	ENSMUSG00000027748	ENSMUSG00000027748	ENSMUSG00000027748	3	54244033	54406085	57	59
2699	13	37034726	37070893	ENSMUSG00000027750	ENSMUSG00000027750	ENSMUSG00000027750	3	54449054	54478961	57	59
2700	13	36481637	36531823	ENSMUSG00000027751	ENSMUSG00000027751	ENSMUSG00000027751	3	54784413	54803629	57	59
2701	13	36472943	36481436	ENSMUSG00000027752	ENSMUSG00000027752	ENSMUSG00000027752	3	54816607	54823322	57	59
2702	13	36421967	36471477	ENSMUSG00000036632	ENSMUSG00000036632	ENSMUSG00000036632	3	54823468	54837724	57	59
2703	13	36320321	36351966	ENSMUSG00000027796	ENSMUSG00000027796	ENSMUSG00000027796	3	54843511	54889186	57	59
2704	13	36291339	36301740	ENSMUSG00000036615	ENSMUSG00000036615	ENSMUSG00000036615	3	54891052	54895720	57	59
2705	13	36146049	36169641	ENSMUSG00000056306	ENSMUSG00000056306	ENSMUSG00000056306	3	54985000	55003816	57	59
2706	13	35904495	35914972	ENSMUSG00000027793	ENSMUSG00000027793	ENSMUSG00000027793	3	55133406	55142971	57	59
2707	13	35775162	35818420	ENSMUSG00000036580	ENSMUSG00000036580	ENSMUSG00000036580	3	55200071	55225240	57	59
2708	13	35699311	35769961	ENSMUSG00000048655	ENSMUSG00000048655	ENSMUSG00000048655	3	55225268	55260864	57	59
2709	13	35640345	35686676	ENSMUSG00000027794	ENSMUSG00000027794	ENSMUSG00000027794	3	55269973	55297879	57	59
2710	13	35243478	35603443	ENSMUSG00000027797	ENSMUSG00000027797	ENSMUSG00000027797	3	55330557	55624607	57	59
2711	13	34414456	35144872	ENSMUSG00000027799	ENSMUSG00000027799	ENSMUSG00000027799	3	55713162	56271630	57	59
2712	13	34946319	34948830	ENSMUSG00000056947	ENSMUSG00000056947	ENSMUSG00000056947	3	55870441	55872930	57	59
2713	3	150570252	150578258	ENSMUSG00000027800	ENSMUSG00000027800	ENSMUSG00000027800	3	57377606	57382717	58	60
2714	3	150674451	150716260	ENSMUSG00000027801	ENSMUSG00000027801	ENSMUSG00000027801	3	57513388	57529606	58	60
2715	3	150721072	150903750	ENSMUSG00000027803	ENSMUSG00000027803	ENSMUSG00000027803	3	57543578	57663809	58	60
2716	3	150941222	150952968	ENSMUSG00000036513	ENSMUSG00000036513	ENSMUSG00000036513	3	57732851	57739620	58	60
2717	3	150971027	150992843	ENSMUSG00000074591	ENSMUSG00000074591	ENSMUSG00000074591	3	57755351	57780466	58	60
2718	3	151013194	151162613	ENSMUSG00000036503	ENSMUSG00000036503	ENSMUSG00000036503	3	57824042	57923048	58	60
2719	3	151165384	151171586	ENSMUSG00000027805	ENSMUSG00000027805	ENSMUSG00000027805	3	57929825	57935466	58	60
2720	3	151608812	151660303	ENSMUSG00000027806	ENSMUSG00000027806	ENSMUSG00000027806	3	58503618	58548194	58	60
2721	3	151742988	151803651	ENSMUSG00000027808	ENSMUSG00000027808	ENSMUSG00000027808	3	58609898	58613682	58	60
2722	3	151747206	151784717	ENSMUSG00000027810	ENSMUSG00000027810	ENSMUSG00000027810	3	58613945	58645430	58	60
2723	3	151803756	151830922	ENSMUSG00000075700	ENSMUSG00000075700	ENSMUSG00000075700	3	58664666	58678220	58	60
2724	3	151860367	151904432	ENSMUSG00000070471	ENSMUSG00000070471	ENSMUSG00000070471	3	58704239	58714633	58	60
2725	3	151941611	151963953	ENSMUSG00000036432	ENSMUSG00000036432	ENSMUSG00000036432	3	58762878	58780292	58	60
2726	3	152127044	152173476	ENSMUSG00000043850	ENSMUSG00000043850	ENSMUSG00000043850	3	58933144	58973141	58	60
2727	3	152287366	152634500	ENSMUSG00000056476	ENSMUSG00000056476	ENSMUSG00000056476	3	59094931	59402675	58	60

2728	3	152398904	152399863	ENSMUSG00000050075	ENSMUSG00000050075	ENSMUSG00000050075	3	59184636	59189701	58	60
2729	3	152412595	152478847	ENSMUSG00000036381	ENSMUSG00000036381	ENSMUSG00000036381	3	59202554	59218553	58	60
2730	3	152494587	152517326	ENSMUSG00000051431	ENSMUSG00000051431	ENSMUSG00000051431	3	59266852	59283033	58	60
2731	3	152526786	152530025	ENSMUSG00000036362	ENSMUSG00000036362	ENSMUSG00000036362	3	59295825	59298811	58	60
2732	3	152538066	152585234	ENSMUSG00000036353	ENSMUSG00000036353	ENSMUSG00000036353	3	59304198	59350754	58	60
2733	3	153014551	153028966	ENSMUSG00000027761	ENSMUSG00000027761	ENSMUSG00000027761	3	60119717	60128086	58	60
2734	3	153080371	153082026	ENSMUSG00000027762	ENSMUSG00000027762	ENSMUSG00000027762	3	60173985	60174935	58	60
2735	3	153499884	153666258	ENSMUSG00000027763	ENSMUSG00000027763	ENSMUSG00000027763	3	60589181	60717679	58	60
2736	3	154035426	154038531	ENSMUSG00000027765	ENSMUSG00000027765	ENSMUSG00000027765	3	61090724	61096908	58	60
2737	3	154362719	154368955	ENSMUSG00000036894	ENSMUSG00000036894	ENSMUSG00000036894	3	61452523	61456367	58	60
2738	3	155321482	155457031	ENSMUSG00000036885	ENSMUSG00000036885	ENSMUSG00000036885	3	62468823	62548275	58	60
2739	3	155476152	155524971	ENSMUSG00000027770	ENSMUSG00000027770	ENSMUSG00000027770	3	62558012	62594917	58	60
2740	3	155538188	155630098	ENSMUSG00000043441	ENSMUSG00000043441	ENSMUSG00000043441	3	62617892	62693069	58	60
2741	3	156284651	156384186	ENSMUSG00000027820	ENSMUSG00000027820	ENSMUSG00000027820	3	63383801	63470160	58	60
2742	3	156680365	156876522	ENSMUSG00000036834	ENSMUSG00000036834	ENSMUSG00000036834	3	63785362	63872067	58	60
2743	3	156963101	157006755	ENSMUSG00000048581	ENSMUSG00000048581	ENSMUSG00000048581	3	64002625	64017314	58	60
2744	3	157027032	157054889	ENSMUSG00000027822	ENSMUSG00000027822	ENSMUSG00000027822	3	64021440	64052595	58	60
2745	3	157219241	157238169	ENSMUSG00000043897	ENSMUSG00000043897	ENSMUSG00000043897	3	64204361	64206805	58	60
2746	3	157491520	157739237	ENSMUSG00000027827	ENSMUSG00000027827	ENSMUSG00000027827	3	65197313	65466155	58	60
2747	3	157741239	157755629	ENSMUSG00000027828	ENSMUSG00000027828	ENSMUSG00000027828	3	65467584	65480514	58	60
2748	3	157875092	157907249	ENSMUSG00000034640	ENSMUSG00000034640	ENSMUSG00000034640	3	65616376	65643447	58	60
2749	3	158193564	158246233	ENSMUSG00000057145	ENSMUSG00000057145	ENSMUSG00000057145	3	65871758	65919088	58	60
2750	3	158348279	158361176	ENSMUSG00000027829	ENSMUSG00000027829	ENSMUSG00000027829	3	66034067	66046148	58	60
2751	3	158460228	158703830	ENSMUSG00000027831	ENSMUSG00000027831	ENSMUSG00000027831	3	66141488	66384766	58	60
2752	3	158637301	158644070	ENSMUSG00000027832	ENSMUSG00000027832	ENSMUSG00000027832	3	66307825	66313734	58	60
2753	3	159298513	159306507	ENSMUSG00000027833	ENSMUSG00000027833	ENSMUSG00000027833	3	67061188	67069700	58	60
2754	3	159310586	159745268	ENSMUSG00000034544	ENSMUSG00000034544	ENSMUSG00000034544	3	67073898	67444242	58	60
2755	3	159783318	159805875	ENSMUSG00000048416	ENSMUSG00000048416	ENSMUSG00000048416	3	67462077	67487932	58	60
2756	3	159845011	159893055	ENSMUSG00000027774	ENSMUSG00000027774	ENSMUSG00000027774	3	67518051	67563010	58	60
2757	3	159866900	159873176	ENSMUSG00000047557	ENSMUSG00000047557	ENSMUSG00000047557	3	67545928	67551836	58	60
2758	3	159897597	159932969	ENSMUSG00000049404	ENSMUSG00000049404	ENSMUSG00000049404	3	67566901	67603452	58	60
2759	3	160002577	160030198	ENSMUSG00000027775	ENSMUSG00000027775	ENSMUSG00000027775	3	67670747	67689312	58	60
2760	3	160041482	160042567	ENSMUSG00000046887	ENSMUSG00000046887	ENSMUSG00000046887	3	67707725	67708648	58	60
2761	3	160474304	161097257	ENSMUSG00000027777	ENSMUSG00000027777	ENSMUSG00000027777	3	68556128	68714410	58	60
2762	3	161189323	161196499	ENSMUSG00000027776	ENSMUSG00000027776	ENSMUSG00000027776	3	68778573	68786454	58	60
2763	3	161426323	161428693	ENSMUSG00000046999	ENSMUSG00000046999	ENSMUSG00000046999	3	68957771	68960091	58	60
2764	3	161600124	161635433	ENSMUSG00000034349	ENSMUSG00000034349	ENSMUSG00000034349	3	69092901	69122558	58	60
2765	3	161700658	161766070	ENSMUSG00000027782	ENSMUSG00000027782	ENSMUSG00000027782	3	69160150	69215021	58	60
2766	3	161877641	161878927	ENSMUSG00000027783	ENSMUSG00000027783	ENSMUSG00000027783	3	69310348	69311545	58	60
2767	3	161956791	162269639	ENSMUSG00000027784	ENSMUSG00000027784	ENSMUSG00000027784	3	69404847	69643340	58	60
2768	3	162421793	162452489	ENSMUSG00000027787	ENSMUSG00000027787	ENSMUSG00000027787	3	69809972	69836971	58	60
2769	3	162546575	162546805	ENSMUSG00000043461	ENSMUSG00000043461	ENSMUSG00000043461	3	69907467	69947803	58	60
2770	3	162697290	162704424	ENSMUSG00000027788	ENSMUSG00000027788	ENSMUSG00000027788	3	70095542	70116633	58	60
2771	3	166179381	166278976	ENSMUSG00000027790	ENSMUSG00000027790	ENSMUSG00000027790	3	72976489	73054795	58	60
2772	3	166387227	166397163	ENSMUSG00000048304	ENSMUSG00000048304	ENSMUSG00000048304	3	73134051	73142869	58	60
2773	3	166973387	167037944	ENSMUSG00000027792	ENSMUSG00000027792	ENSMUSG00000027792	3	73721734	73794168	58	60
2774	3	168441116	168528470	ENSMUSG00000034151	ENSMUSG00000034151	ENSMUSG00000034151	3	75123833	75229649	58	60
2775	3	168642417	168674515	ENSMUSG00000034139	ENSMUSG00000034139	ENSMUSG00000034139	3	75328299	75356004	58	60
2776	3	168679169	168853983	ENSMUSG00000058653	ENSMUSG00000058653	ENSMUSG00000058653	3	75360923	75467966	58	60
2777	3	168936205	169026042	ENSMUSG00000027834	ENSMUSG00000027834	ENSMUSG00000027834	3	75643474	75729422	58	60
2778	3	169209925	169296363	ENSMUSG00000034109	ENSMUSG00000034109	ENSMUSG00000034109	3	75962110	76042876	58	60
2779	4	162524501	163304636	ENSMUSG00000034098	ENSMUSG00000034098	ENSMUSG00000034098	3	76160525	76795932	59	61
2780	4	160408696	160500749	ENSMUSG00000062232	ENSMUSG00000062232	ENSMUSG00000062232	3	79148463	79231802	59	61
2781	4	159812933	159849266	ENSMUSG00000027809	ENSMUSG00000027809	ENSMUSG00000027809	3	79689728	79714645	59	61
2782	4	159655440	159792814	ENSMUSG00000034009	ENSMUSG00000034009	ENSMUSG00000034009	3	79730643	79823721	59	61
2783	4	159351126	159395886	ENSMUSG00000027956	ENSMUSG00000027956	ENSMUSG00000027956	3	79899080	79928589	59	61
2784	4	159265459	159313652	ENSMUSG00000027955	ENSMUSG00000027955	ENSMUSG00000027955	3	79971915	80029312	59	61
2785	4	158361297	158506677	ENSMUSG00000033981	ENSMUSG00000033981	ENSMUSG00000033981	3	80770863	80888718	59	61
2786	4	158216788	158312299	ENSMUSG00000028020	ENSMUSG00000028020	ENSMUSG00000028020	3	80929531	80999544	59	61
2787	4	157902214	158111996	ENSMUSG00000028019	ENSMUSG00000028019	ENSMUSG00000028019	3	81122343	81299958	59	61
2788	4	157064728	157094498	ENSMUSG00000028015	ENSMUSG00000028015	ENSMUSG00000028015	3	82018543	82042652	59	61
2789	4	157044297	157061000	ENSMUSG00000028011	ENSMUSG00000028011	ENSMUSG00000028011	3	82044339	82061655	59	61
2790	4	156970331	157006875	ENSMUSG00000028008	ENSMUSG00000028008	ENSMUSG00000028008	3	82082861	82107160	59	61
2791	4	156899661	156951636	ENSMUSG00000028005	ENSMUSG00000028005	ENSMUSG00000028005	3	82119182	82159998	59	61
2792	4	156807800	156872926	ENSMUSG00000033910	ENSMUSG00000033910	ENSMUSG00000033910	3	82178350	82231000	59	61
2793	4	156488027	156517572	ENSMUSG00000033900	ENSMUSG00000033900	ENSMUSG00000033900	3	82444143	82469457	59	61
2794	4	156349231	156357677	ENSMUSG00000028004	ENSMUSG00000028004	ENSMUSG00000028004	3	82624310	82634013	59	61
2795	4	155921950	155969414	ENSMUSG00000033882	ENSMUSG00000033882	ENSMUSG00000033882	3	82950295	82952563	59	61
2796	4	155881440	155891637	ENSMUSG00000028003	ENSMUSG00000028003	ENSMUSG00000028003	3	82978509	82989901	59	61
2797	4	155745178	155753352	ENSMUSG00000033860	ENSMUSG00000033860	ENSMUSG00000033860	3	83093784	83100270	59	61
2798	4	155723730	155731347	ENSMUSG00000059807	ENSMUSG00000059807	ENSMUSG00000059807	3	83118630	83119337	59	61
2799	4	155703596	155711683	ENSMUSG00000033831	ENSMUSG00000033831	ENSMUSG00000033831	3	83128174	83135736	59	61
2800	4	155675629	155690998	ENSMUSG00000027998	ENSMUSG00000027998	ENSMUSG00000027998	3	83141465	83158218	59	61
2801	4	155374977	155531899	ENSMUSG00000068952	ENSMUSG00000068952	ENSMUSG00000068952	3	83434096	83442394	59	61
2802	4	154921194	154929678	ENSMUSG00000027996	ENSMUSG00000027996	ENSMUSG00000027996	3	83852248	83860242	59	61
2803	4	154842102	154846301	ENSMUSG00000027995	ENSMUSG00000027995	ENSMUSG00000027995	3	83922197	83927694	59	61
2804	4	154607002	154777310	ENSMUSG00000033767	ENSMUSG00000033767	ENSMUSG00000033767	3	83984136	84126064	59	61
2805	4	154485251	154555693	ENSMUSG00000033752	ENSMUSG00000033752	ENSMUSG00000033752	3	84173995	84227691	59	61
2806	4	154410988	154476949	ENSMUSG00000027993	ENSMUSG00000027993	ENSMUSG00000027993	3	84249649	84345650	59	61
2807	4	154083585	154120288	ENSMUSG00000041842	ENSMUSG00000041842	ENSMUSG00000041842	3	84529962	84565418	59	61
2808	4	153920562	154052514	ENSMUSG00000074513	ENSMUSG00000074513	ENSMUSG00000074513	3	84583663	84605591	59	61
2809	4	153909958	153920343	ENSMUSG00000047819	ENSMUSG00000047819	ENSMUSG00000047819	3	84679501	84682959	59	61
2810	4	153766727	153820641	ENSMUSG00000056498	ENSMUSG00000056498	ENSMUSG00000056498	3	84752119	84778512	59	61
2811	4	153461860	153675622	ENSMUSG00000028086	ENSMUSG00000028086	ENSMUSG00000028086	3	85038138	85063572	59	61
2812	4	152811462	152901609	ENSMUSG00000028085	ENSMUSG00000028085	ENSMUSG00000028085	3	85660056	85740397	59	61
2813	4	152417775	152432055	ENSMUSG00000049013	ENSMUSG00000049013	ENSMUSG00000049013	3	86079737	86088418	59	61
2814	4	152260889	152367022	ENSMUSG00000028082	ENSMUSG00000028082	ENSMUSG00000028082	3	86170815	86214396	59	61
2815	4	151405263	152156099	ENSMUSG00000028080	ENSMUSG00000028080	ENSMUSG00000028080	3	86310607	86868616	59	61
2816	4	151722699	151725262	ENSMUSG00000057777	ENSMUSG00000057777	ENSMUSG00000057777	3	86632539	86633618	59	61
2817	4	151219630	151398032	ENSMUSG00000028078	ENSMUSG00000028078	ENSMUSG00000028078	3	86873060	87006157	59	61
2818	1	156230091	156331534	ENSMUSG00000041734	ENSMUSG00000041734	ENSMUSG00000041734	3	87168739	87227036	60	62
2819	1	156067328	156078212	ENSMUSG00000015854	ENSMUSG00000015854	ENSMUSG00000015854	3	87443855	87456999	60	62
2820	1	156031275	156056519	ENSMUSG00000059994	ENSMUSG00000059994	ENSMUSG00000059994	3	87462375	87478060	60	62
2821	1	155357607	155374783	ENSMUSG00000003382	ENSMUSG00000003382	ENSMUSG00000003382	3	87611568	87625510	60	62
2822	1	155171256	155281786	ENSMUSG00000041977	ENSMUSG00000041977	ENSMUSG00000041977	3	87704678	87823960	60	62
2823	1	155157066	155169510	ENSMUSG00000023084	ENSMUSG00000023084	ENSMUSG00000023084	3	87822850	87834517	60	62
2824	1	155140277	155152845	ENSMUSG00000028073	ENSMUSG00000028073	ENSMUSG00000028073	3	87835024	87854880	60	62
2825	1	155097295	155118266	ENSMUSG00000028072	ENSMUSG00000028072	ENSMUSG00000028072	3	87864171	87881089	60	62
2826	1	155076479	155095434	ENSMUSG00000005640	ENSMUSG00000005640	ENSMUSG00000005640	3	87882989	87902028	60	62
2827	1	155042659	155053278	ENSMUSG00000028071	ENSMUSG00000028071	ENSMUSG00000028071	3	87932938	87941649	60	62
2828	1	155003898	155037226	ENSMUSG00000004895	ENSMUSG00000004895	ENSMUSG00000004895	3	87944839	87971507	60	62
2829	1	154973718	154977547	ENSMUSG00000019710	ENSMUSG00000019710	ENSMUSG00000019710	3	88005471	88009591	60	62
2830	1	154964891	154973365	ENSMUSG00000004896	ENSMUSG00000004896	ENSMUSG00000004896	3	88008832	88016073	60	62
2831	1	154958307	154964851	ENSMUSG00000048039	ENSMUSG00000048039	ENSMUSG00000048039	3	88016241	88026613	60	62
2832	1	154936022	154942017	ENSMUSG00000004885	ENSMUSG00000004885	ENSMUSG00000004885	3	88034620	88039299	60	62
2833	1	154905179	154913813	ENSMUSG00000004891	ENSMUSG00000004891	ENSMUSG00000004891	3	88057020	88066378	60	62
2834	1	154878357	154895939	ENSMUSG00000004892	ENSMUSG00000004892	ENSMUSG00000004892	3	88073458	88086157	60	62
2835	1	154855710	154862141	ENSMUSG00000004894	ENSMUSG00000004894	ENSMUSG00000004894	3	88107677	88110344	60	62
2836	1	154831403	154837894	ENSMUSG00000028069	ENSMUSG00000028069	ENSMUSG00000028069	3	88129051	88141656	60	62
2837	1	154828178	154830715	ENSMUSG00000028070	ENSMUSG00000028070	ENSMUSG00000028070	3	88142450	88144422	60	62
2838	1	154817781	154823186	ENSMUSG00000051036	ENSMUSG00000051036	ENSMUSG00000051036	3	88155337	88164231	60	62
2839	1	154761822	154809020	ENSMUSG00000028068	ENSMUSG00000028068	ENSMUSG00000028068	3	88167978	88206973	60	62
2840	1	154700143	154737244	ENSMUSG00000001419	ENSMUSG00000001419	ENSMUSG00000001419	3	88228354	88254650	60	62
2841	1	154605627	154621633	ENSMUSG00000001417	ENSMUSG00000001417	ENSMUSG00000001417	3	88328801	88340611	60	62
2842	1	154573729	154583410	ENSMUSG00000010538	ENSMUSG00000010538	ENSMUSG00000010538	3	88368687	88382765	60	62
2843	1	154545376	154574819	ENSMUSG00000001416	ENSMUSG00000001416	ENSMUSG00000001416	3	88383062	88407693	60	62
2844	1	154529103	154532087	ENSMUSG00000001418	ENSMUSG00000001418	ENSMUSG00000001418	3	88410987	88414599	60	62
2845	1	154519363	154528858	ENSMUSG00000001420	ENSMUSG00000001420	ENSMUSG00000001420	3	88414580	88420360	60	62
2846	1	154485639	154519240	ENSMUSG00000001415	ENSMUSG00000001415	ENSMUSG00000001415	3	88422208	88448264	60	62
2847	1	154479836	154484471	ENSMUSG00000041423	ENSMUSG00000041423	ENSMUSG00000041423	3	88450980	88454467	60	62
2848	1	154430502	154449210	ENSMUSG00000050144	ENSMUSG00000050144	ENSMUSG00000050144	3	88496425	88511045	60	62
2849	1	154386434	154414159	ENSMUSG00000028064	ENSMUSG00000028064	ENSMUSG00000028064	3	88521889	88541666	60	62
2850	1	154351122	154376504	ENSMUSG00000028063	ENSMUSG00000028063	ENSMUSG00000028063	3	88568249	88589234	60	62
2851	1	154312989	154318056	ENSMUSG00000074480	ENSMUSG00000074480	ENSMUSG00000074480	3	88621975	88627307	60	62
2852	1	154297589	154306920	ENSMUSG00000008601	ENSMUSG00000008601	ENSMUSG00000008601	3	88627956	88634206	60	62
2853	1	154291265	154294918	ENSMUSG00000028062	ENSMUSG00000028062	ENSMUSG00000028062	3	88635746	88638854	60	62
2854	1	154245463	154257374	ENSMUSG00000041355	ENSMUSG00000041355	ENSMUSG00000041355	3	88665807	88673918	60	62
2855	1	154183270	154208757	ENSMUSG00000028059	ENSMUSG00000028059	ENSMUSG00000028059	3	88707065	88732841	60	62
2856	1	154178125	154179364	ENSMUSG00000049741	ENSMUSG00000049741	ENSMUSG00000049741	3	88737825	88739069	60	62
2857	1	154149458	154170812	ENSMUSG00000028060	ENSMUSG00000028060	ENSMUSG00000028060	3	88771743	88798850	60	62
2858	1	154136689	154147330	ENSMUSG00000028057	ENSMUSG00000028057	ENSMUSG00000028057	3	88802834	88816976	60	62
2859	1	154095924	154121614	ENSMUSG00000068923	ENSMUSG00000068923	ENSMUSG00000068923	3	88831629	88858467	60	62
2860	1	153925473	153975425	ENSMUSG00000068921	ENSMUSG00000068921	ENSMUSG00000068921	3	89006730	89035880	60	62
2861	1	153571683	153799108	ENSMUSG00000028053	ENSMUSG00000028053	ENSMUSG00000028053	3	89051739	89165302	60	62
2862	1	153557342	153567529	ENSMUSG00000041263	ENSMUSG00000041263	ENSMUSG00000041263	3	89169908	89179238	60	62
2863	1	153526668	153537849	ENSMUSG00000041237	ENSMUSG00000041237	ENSMUSG00000041237	3	89222069	89231560	60	62
2864	1	153513998	153526263	ENSMUSG00000028051	ENSMUSG00000028051	ENSMUSG00000028051	3	89232001	89246123	60	62
2865	1	153492397	153498819	ENSMUSG00000028049	ENSMUSG00000028049	ENSMUSG00000028049	3	89263417	89268690	60	62
2866	1	153483621	153491898	ENSMUSG00000032657	ENSMUSG00000032657	ENSMUSG00000032657	3	89269152	89275216	60	62
2867	1	153445114	153450238	ENSMUSG00000064068	ENSMUSG00000064068	ENSMUSG00000064068	3	89295016	89299853	60	62
2868	1	153432003	153444312	ENSMUSG00000028047	ENSMUSG00000028047	ENSMUSG00000028047	3	89301134	89312746	60	62
2869	1	153425235	153428525	ENSMUSG00000042784	ENSMUSG00000042784	ENSMUSG00000042784	3	89314979	89319296	60	62
2870	1	153412984	153424069	ENSMUSG00000042766	ENSMUSG00000042766	ENSMUSG00000042766	3	89320104	89331824	60	62
2871	1	153408510	153412437	ENSMUSG00000042747	ENSMUSG00000042747	ENSMUSG00000042747	3	89331893	89335649	60	62
2872	1	153378991	153379507	ENSMUSG00000042737	ENSMUSG00000042737	ENSMUSG00000042737	3	89352479	89353004	60	62
2873	1	153374950	153377953	ENSMUSG00000027953	ENSMUSG00000027953	ENSMUSG00000027953	3	89354173	89356487	60	62
2874	1	153366560	153373957	ENSMUSG00000027954	ENSMUSG00000027954	ENSMUSG00000027954	3	89357663	89365568	60	62
2875	1	153317972	153326638	ENSMUSG00000028039	ENSMUSG00000028039	ENSMUSG00000028039	3	89401364	89402678	60	62
2876	1	153302831	153308653	ENSMUSG00000028040	ENSMUSG00000028040	ENSMUSG00000028040	3	89419665	89423955	60	62
2877	1	153290386	153301876	ENSMUSG00000028041	ENSMUSG00000028041	ENSMUSG00000028041	3	89424469	89435909	60	62
2878	1	153272924	153290030	ENSMUSG00000042672	ENSMUSG00000042672	ENSMUSG00000042672	3	89443435	89450189	60	62
2879	1	153257634	153272881	ENSMUSG00000042661	ENSMUSG00000042661	ENSMUSG00000042661	3	89451291	89458704	60	62
2880	1	153241757	153257622	ENSMUSG00000028042	ENSMUSG00000028042	ENSMUSG00000028042	3	89463571	89479130	60	62
2881	1	153222441	153232211	ENSMUSG00000042642	ENSMUSG00000042642	ENSMUSG00000042642	3	89486931	89497799	60	62
2882	1	153201398	153209847	ENSMUSG00000042626	ENSMUSG00000042626	ENSMUSG00000042626	3	89507778	89515941	60	62
2883	1	153196127	153200882	ENSMUSG00000047824	ENSMUSG00000047824	ENSMUSG00000047824	3	89516188	89521055	60	62
2884	1	153183180	153195204	ENSMUSG00000042613	ENSMUSG00000042613	ENSMUSG00000042613	3	89522633	89534872	60	62
2885	1	153163834	153176091	ENSMUSG00000027952	ENSMUSG00000027952	ENSMUSG00000027952	3	89545243	89554933	60	62
2886	1	152946541	153109378	ENSMUSG00000000794	ENSMUSG00000000794	ENSMUSG00000000794	3	89606348	89753332	60	62
2887	1	152821169	152867098	ENSMUSG00000027951	ENSMUSG00000027951	ENSMUSG00000027951	3	89816711	89837333	60	62
2888	1	152806881	152819126	ENSMUSG00000027950	ENSMUSG00000027950	ENSMUSG00000027950	3	89839375	89850512	60	62
2889	1	152787675	152797744	ENSMUSG00000042572	ENSMUSG00000042572	ENSMUSG00000042572	3	89859543	89869927	60	62
2890	1	152718581	152741213	ENSMUSG00000046280	ENSMUSG00000046280	ENSMUSG00000046280	3	89917297	89944763	60	62
2891	1	152644293	152708550	ENSMUSG00000027947	ENSMUSG00000027947	ENSMUSG00000027947	3	89955251	89999087	60	62
2892	1	152566900	152590403	ENSMUSG00000060671	ENSMUSG00000060671	ENSMUSG00000060671	3	90025416	90039292	60	62
2893	1	152464180	152509952	ENSMUSG00000042520	ENSMUSG00000042520	ENSMUSG00000042520	3	90086215	90138402	60	62
2894	1	152231785	152394216	ENSMUSG00000027939	ENSMUSG00000027939	ENSMUSG00000027939	3	90190061	90253913	60	62
2895	1	152213373	152217075	ENSMUSG00000027937	ENSMUSG00000027937	ENSMUSG00000027937	3	90317560	90321765	60	62
2896	1	152207003	152213463	ENSMUSG00000027938	ENSMUSG00000027938	ENSMUSG00000027938	3	90323427	90329439	60	62
2897	1	152186778	152197667	ENSMUSG00000027936	ENSMUSG00000027936	ENSMUSG00000027936	3	90340213	90350052	60	62
2898	1	152168601	152185796	ENSMUSG00000042404	ENSMUSG00000042404	ENSMUSG00000042404	3	90353333	90365909	60	62
2899	1	152043825	152162075	ENSMUSG00000042390	ENSMUSG00000042390	ENSMUSG00000042390	3	90379288	90444040	60	62
2900	1	152013454	152019257	ENSMUSG00000027932	ENSMUSG00000027932	ENSMUSG00000027932	3	90471167	90475865	60	62
2901	1	151967167	152013179	ENSMUSG00000027933	ENSMUSG00000027933	ENSMUSG00000027933	3	90477315	90519563	60	62
2902	1	151917737	151933092	ENSMUSG00000027931	ENSMUSG00000027931	ENSMUSG00000027931	3	90536519	90552265	60	62
2903	1	151901136	151910148	ENSMUSG00000001016	ENSMUSG00000001016	ENSMUSG00000001016	3	90562128	90574306	60	62
2904	1	151897754	151900930	ENSMUSG00000001018	ENSMUSG00000001018	ENSMUSG00000001018	3	90573961	90576931	60	62
2905	1	151867026	151871134	ENSMUSG00000044080	ENSMUSG00000044080	ENSMUSG00000044080	3	90596964	90600319	60	62
2906	1	151857899	151870460	ENSMUSG00000042312	ENSMUSG00000042312	ENSMUSG00000042312	3	90600362	90610510	60	62
2907	1	151853355	151856086	ENSMUSG00000042306	ENSMUSG00000042306	ENSMUSG00000042306	3	90612797	90614760	60	62
2908	1	151845986	151852268	ENSMUSG00000074457	ENSMUSG00000074457	ENSMUSG00000074457	3	90623300	90629075	60	62
2909	1	151786429	151788472	ENSMUSG00000001021	ENSMUSG00000001021	ENSMUSG00000001021	3	90672879	90688451	60	62
2910	1	151782713	151784985	ENSMUSG00000001020	ENSMUSG00000001020	ENSMUSG00000001020	3	90689694	90691967	60	62
2911	1	151776247	151780865	ENSMUSG00000001023	ENSMUSG00000001023	ENSMUSG00000001023	3	90695737	90697703	60	62
2912	1	151773699	151775344	ENSMUSG00000001025	ENSMUSG00000001025	ENSMUSG00000001025	3	90699108	90700341	60	62
2913	1	151629132	151630288	ENSMUSG00000056054	ENSMUSG00000056054	ENSMUSG00000056054	3	90755012	90755961	60	62
2914	1	151569220	151587646	ENSMUSG00000042250	ENSMUSG00000042250	ENSMUSG00000042250	3	90812879	90826441	60	62
2915	1	151536962	151549818	ENSMUSG00000042244	ENSMUSG00000042244	ENSMUSG00000042244	3	92100539	92117511	60	62
2916	1	151498800	151501222	ENSMUSG00000043165	ENSMUSG00000043165	ENSMUSG00000043165	3	92166200	92169058	60	62
2917	1	151456684	151458417	ENSMUSG00000056270	ENSMUSG00000056270	ENSMUSG00000056270	3	92208131	92209874	60	62
2918	1	151442543	151444220	ENSMUSG00000027927	ENSMUSG00000027927	ENSMUSG00000027927	3	92220922	92228671	60	62
2919	1	151240849	151242956	ENSMUSG00000045539	ENSMUSG00000045539	ENSMUSG00000045539	3	92542222	92542938	60	62
2920	1	151209752	151211693	ENSMUSG00000045566	ENSMUSG00000045566	ENSMUSG00000045566	3	92585666	92585896	60	62
2921	1	151117417	151124147	ENSMUSG00000074435	ENSMUSG00000074435	ENSMUSG00000074435	3	92669275	92674397	60	62
2922	1	150753602	150755110	ENSMUSG00000027913	ENSMUSG00000027913	ENSMUSG00000027913	3	93099610	93101089	60	62

2923	1	150222015	150233490	ENSMUSG00000041959	ENSMUSG00000041959	ENSMUSG00000041959	3	93640568	93650047	60	62
2924	1	150112684	150148908	ENSMUSG00000028145	ENSMUSG00000028145	ENSMUSG00000028145	3	94395621	94417936	60	62
2925	1	150086251	150091520	ENSMUSG00000028148	ENSMUSG00000028148	ENSMUSG00000028148	3	94427503	94432754	60	62
2926	1	150039364	150045254	ENSMUSG00000044505	ENSMUSG00000044505	ENSMUSG00000044505	3	94484623	94489905	60	62
2927	1	150010674	150029622	ENSMUSG00000041912	ENSMUSG00000041912	ENSMUSG00000041912	3	94498700	94518037	60	62
2928	1	150002069	150010429	ENSMUSG00000028141	ENSMUSG00000028141	ENSMUSG00000028141	3	94518794	94522020	60	62
2929	1	149941454	149955914	ENSMUSG00000028137	ENSMUSG00000028137	ENSMUSG00000028137	3	94563898	94577600	60	62
2930	1	149851182	149938174	ENSMUSG00000028136	ENSMUSG00000028136	ENSMUSG00000028136	3	94582954	94667953	60	62
2931	1	149779405	149822683	ENSMUSG00000005968	ENSMUSG00000005968	ENSMUSG00000005968	3	94698163	94744210	60	62
2932	1	149750497	149777789	ENSMUSG00000068876	ENSMUSG00000068876	ENSMUSG00000068876	3	94845487	94871896	60	62
2933	1	149641824	149698565	ENSMUSG00000038902	ENSMUSG00000038902	ENSMUSG00000038902	3	94939350	94966286	60	62
2934	1	149610623	149641036	ENSMUSG00000005779	ENSMUSG00000005779	ENSMUSG00000005779	3	94969728	94972336	60	62
2935	1	149579740	149586393	ENSMUSG00000005774	ENSMUSG00000005774	ENSMUSG00000005774	3	95039574	95044772	60	62
2936	1	149531037	149566536	ENSMUSG00000038861	ENSMUSG00000038861	ENSMUSG00000038861	3	95060151	95092025	60	62
2937	1	149521412	149531003	ENSMUSG00000019338	ENSMUSG00000019338	ENSMUSG00000019338	3	95092106	95100642	60	62
2938	1	149437675	149486988	ENSMUSG00000028126	ENSMUSG00000028126	ENSMUSG00000028126	3	95145888	95191855	60	62
2939	1	149415404	149429290	ENSMUSG00000008958	ENSMUSG00000008958	ENSMUSG00000008958	3	95196502	95208455	60	62
2940	1	149409087	149415116	ENSMUSG00000005628	ENSMUSG00000005628	ENSMUSG00000005628	3	95210918	95214612	60	62
2941	1	149405142	149409397	ENSMUSG00000005629	ENSMUSG00000005629	ENSMUSG00000005629	3	95215133	95219416	60	62
2942	1	149395729	149398849	ENSMUSG00000013707	ENSMUSG00000013707	ENSMUSG00000013707	3	95224925	95227764	60	62
2943	1	149370785	149385557	ENSMUSG00000038777	ENSMUSG00000038777	ENSMUSG00000038777	3	95245909	95259428	60	62
2944	1	149309704	149364642	ENSMUSG00000038766	ENSMUSG00000038766	ENSMUSG00000038766	3	95272854	95292007	60	62
2945	1	149290071	149298711	ENSMUSG00000046722	ENSMUSG00000046722	ENSMUSG00000046722	3	95314184	95321813	60	62
2946	1	149286840	149290520	ENSMUSG00000068860	ENSMUSG00000068860	ENSMUSG00000068860	3	95322509	95326610	60	62
2947	1	149275670	149286700	ENSMUSG00000028115	ENSMUSG00000028115	ENSMUSG00000028115	3	95326699	95336506	60	62
2948	1	149247577	149274813	ENSMUSG00000015711	ENSMUSG00000015711	ENSMUSG00000015711	3	95339078	95367480	60	62
2949	1	149235936	149247465	ENSMUSG00000038712	ENSMUSG00000038712	ENSMUSG00000038712	3	95368348	95381565	60	62
2950	1	149221178	149234738	ENSMUSG00000015702	ENSMUSG00000015702	ENSMUSG00000015702	3	95381500	95392580	60	62
2951	1	149204287	149211007	ENSMUSG00000015714	ENSMUSG00000015714	ENSMUSG00000015714	3	95400669	95408991	60	62
2952	1	149165512	149203837	ENSMUSG00000015697	ENSMUSG00000015697	ENSMUSG00000015697	3	95408930	95442562	60	62
2953	1	149048810	149115807	ENSMUSG00000015522	ENSMUSG00000015522	ENSMUSG00000015522	3	95519832	95580975	60	62
2954	1	149035311	149047436	ENSMUSG00000028111	ENSMUSG00000028111	ENSMUSG00000028111	3	95584660	95594766	60	62
2955	1	148969296	149005057	ENSMUSG00000038642	ENSMUSG00000038642	ENSMUSG00000038642	3	95612190	95641804	60	62
2956	1	148885330	148936257	ENSMUSG00000046519	ENSMUSG00000046519	ENSMUSG00000046519	3	95674363	95704645	60	62
2957	1	148813661	148818760	ENSMUSG00000038612	ENSMUSG00000038612	ENSMUSG00000038612	3	95744192	95748588	60	62
2958	1	148788510	148800037	ENSMUSG00000015850	ENSMUSG00000015850	ENSMUSG00000015850	3	95761609	95773263	60	62
2959	1	148747162	148752653	ENSMUSG00000028108	ENSMUSG00000028108	ENSMUSG00000028108	3	95819554	95824967	60	62
2960	1	148726517	148746702	ENSMUSG00000028107	ENSMUSG00000028107	ENSMUSG00000028107	3	95825381	95840267	60	62
2961	1	148603804	148712656	ENSMUSG00000028106	ENSMUSG00000028106	ENSMUSG00000028106	3	95847059	95904163	60	62
2962	1	148560549	148592295	ENSMUSG00000015748	ENSMUSG00000015748	ENSMUSG00000015748	3	95916022	95941193	60	62
2963	1	148507224	148519951	ENSMUSG00000038543	ENSMUSG00000038543	ENSMUSG00000038543	3	95970378	95977335	60	62
2964	1	148496793	148504102	ENSMUSG00000038526	ENSMUSG00000038526	ENSMUSG00000038526	3	95983175	95990026	60	62
2965	1	148457341	148475128	ENSMUSG00000015749	ENSMUSG00000015749	ENSMUSG00000015749	3	96014696	96032381	60	62
2966	1	148388248	148398416	ENSMUSG00000015745	ENSMUSG00000015745	ENSMUSG00000015745	3	96074206	96081375	60	62
2967	1	148181604	148216113	ENSMUSG00000038495	ENSMUSG00000038495	ENSMUSG00000038495	3	96189932	96246534	60	62
2968	1	148167663	148175396	ENSMUSG00000045934	ENSMUSG00000045934	ENSMUSG00000045934	3	96247442	96257123	60	62
2969	1	148141494	148156058	ENSMUSG00000038486	ENSMUSG00000038486	ENSMUSG00000038486	3	96266591	96280568	60	62
2970	1	148125110	148138668	ENSMUSG00000015943	ENSMUSG00000015943	ENSMUSG00000015943	3	96281993	96282991	60	62
2971	1	148125698	148126090	ENSMUSG00000063689	ENSMUSG00000063689	ENSMUSG00000063689	3	96305321	96305713	60	62
2972	1	145841560	145848017	ENSMUSG00000049908	ENSMUSG00000049908	ENSMUSG00000049908	3	97004427	97005749	60	62
2973	1	145695517	145712066	ENSMUSG00000057123	ENSMUSG00000057123	ENSMUSG00000057123	3	97117821	97139039	60	62
2974	1	145585807	145609258	ENSMUSG00000028093	ENSMUSG00000028093	ENSMUSG00000028093	3	97244196	97261973	60	62
2975	1	145479806	145564641	ENSMUSG00000038256	ENSMUSG00000038256	ENSMUSG00000038256	3	97290033	97301912	60	62
2976	1	145180958	145234067	ENSMUSG00000028089	ENSMUSG00000028089	ENSMUSG00000028089	3	97646147	97695608	60	62
2977	1	145124462	145163546	ENSMUSG00000028088	ENSMUSG00000028088	ENSMUSG00000028088	3	97714280	97737959	60	62
2978	1	145093314	145110753	ENSMUSG00000038205	ENSMUSG00000038205	ENSMUSG00000038205	3	97743617	97758488	60	62
2979	1	143562784	143706276	ENSMUSG00000038170	ENSMUSG00000038170	ENSMUSG00000038170	3	97775229	97974112	60	62
2980	1	120255701	120413799	ENSMUSG00000027878	ENSMUSG00000027878	ENSMUSG00000027878	3	98098943	98232854	60	62
2981	1	120237679	120240641	ENSMUSG00000043468	ENSMUSG00000043468	ENSMUSG00000043468	3	98246065	98249574	60	62
2982	1	120138164	120155726	ENSMUSG00000027876	ENSMUSG00000027876	ENSMUSG00000027876	3	98307558	98322153	60	62
2983	1	120092142	120113040	ENSMUSG00000027875	ENSMUSG00000027875	ENSMUSG00000027875	3	98365840	98396137	60	62
2984	1	119712955	119738276	ENSMUSG00000027870	ENSMUSG00000027870	ENSMUSG00000027870	3	99003638	99022276	60	62
2985	1	119375367	119484817	ENSMUSG00000004233	ENSMUSG00000004233	ENSMUSG00000004233	3	99270151	99349264	60	62
2986	1	119227193	119331951	ENSMUSG00000027868	ENSMUSG00000027868	ENSMUSG00000027868	3	99382888	99483320	60	62
2987	1	118273914	118304572	ENSMUSG00000033285	ENSMUSG00000033285	ENSMUSG00000033285	3	100267241	100291450	60	62
2988	1	118213560	118273776	ENSMUSG00000027865	ENSMUSG00000027865	ENSMUSG00000027865	3	100291579	100336042	60	62
2989	1	117950079	117972517	ENSMUSG00000044468	ENSMUSG00000044468	ENSMUSG00000044468	3	100600604	100618247	60	62
2990	1	117711594	117873017	ENSMUSG00000008763	ENSMUSG00000008763	ENSMUSG00000008763	3	100695478	100814288	60	62
2991	1	117487732	117517411	ENSMUSG00000051076	ENSMUSG00000051076	ENSMUSG00000051076	3	100954540	101024733	60	62
2992	1	117404448	117447015	ENSMUSG00000033222	ENSMUSG00000033222	ENSMUSG00000033222	3	101067917	101098722	60	62
2993	1	117254202	117334503	ENSMUSG00000027864	ENSMUSG00000027864	ENSMUSG00000027864	3	101169293	101239339	60	62
2994	1	117098530	117113373	ENSMUSG00000027863	ENSMUSG00000027863	ENSMUSG00000027863	3	101404969	101417000	60	62
2995	1	116918554	117011837	ENSMUSG00000042035	ENSMUSG00000042035	ENSMUSG00000042035	3	101506186	101589911	60	62
2996	1	116717359	116748917	ENSMUSG00000033161	ENSMUSG00000033161	ENSMUSG00000033161	3	101705286	101733745	60	62
2997	1	116455899	116479384	ENSMUSG00000044313	ENSMUSG00000044313	ENSMUSG00000044313	3	101942137	101965022	60	62
2998	1	116180528	116185270	ENSMUSG00000048540	ENSMUSG00000048540	ENSMUSG00000048540	3	102139207	102144553	60	62
2999	1	116044151	116112925	ENSMUSG00000027861	ENSMUSG00000027861	ENSMUSG00000027861	3	102215603	102275571	60	62
3000	1	115986120	116035547	ENSMUSG00000027860	ENSMUSG00000027860	ENSMUSG00000027860	3	102287257	102333669	60	62
3001	1	115630060	115682380	ENSMUSG00000027859	ENSMUSG00000027859	ENSMUSG00000027859	3	102598989	102650066	60	62
3002	1	115392155	115433638	ENSMUSG00000027858	ENSMUSG00000027858	ENSMUSG00000027858	3	102863831	102901353	60	62
3003	1	115373938	115378464	ENSMUSG00000027857	ENSMUSG00000027857	ENSMUSG00000027857	3	102906459	102911775	60	62
3004	1	115198947	115339514	ENSMUSG00000027855	ENSMUSG00000027855	ENSMUSG00000027855	3	102969923	103064712	60	62
3005	1	115113623	115124829	ENSMUSG00000027854	ENSMUSG00000027854	ENSMUSG00000027854	3	103124801	103132975	60	62
3006	1	115017245	115039762	ENSMUSG00000070385	ENSMUSG00000070385	ENSMUSG00000070385	3	103203157	103228775	60	62
3007	1	114929744	114970337	ENSMUSG00000007379	ENSMUSG00000007379	ENSMUSG00000007379	3	103256617	103296863	60	62
3008	1	114911701	114925783	ENSMUSG00000005687	ENSMUSG00000005687	ENSMUSG00000005687	3	103300772	103308227	60	62
3009	1	114736922	114855304	ENSMUSG00000033014	ENSMUSG00000033014	ENSMUSG00000033014	3	103408354	103484624	60	62
3010	1	114435912	114497995	ENSMUSG00000027849	ENSMUSG00000027849	ENSMUSG00000027849	3	103704439	103756193	60	62
3011	1	114323553	114326398	ENSMUSG00000027848	ENSMUSG00000027848	ENSMUSG00000027848	3	103864455	103867062	60	62
3012	1	114284529	114321945	ENSMUSG00000008730	ENSMUSG00000008730	ENSMUSG00000008730	3	103869218	103907358	60	62
3013	1	114249286	114258231	ENSMUSG00000027845	ENSMUSG00000027845	ENSMUSG00000027845	3	103929664	103938448	60	62
3014	1	114238893	114249215	ENSMUSG00000032952	ENSMUSG00000032952	ENSMUSG00000032952	3	103938581	103951084	60	62
3015	1	114225149	114231692	ENSMUSG00000044165	ENSMUSG00000044165	ENSMUSG00000044165	3	103961662	103967712	60	62
3016	1	114157956	114215904	ENSMUSG00000027843	ENSMUSG00000027843	ENSMUSG00000027843	3	103989353	104041308	60	62
3017	1	114109487	114156557	ENSMUSG00000044098	ENSMUSG00000044098	ENSMUSG00000044098	3	104043181	104095681	60	62
3018	1	114041350	114103621	ENSMUSG00000058388	ENSMUSG00000058388	ENSMUSG00000058388	3	104097171	104136551	60	62
3019	1	113735104	114030068	ENSMUSG00000052539	ENSMUSG00000052539	ENSMUSG00000052539	3	104144030	104190751	60	62
3020	1	113417354	113469347	ENSMUSG00000032913	ENSMUSG00000032913	ENSMUSG00000032913	3	104583044	104640917	60	62
3021	1	113054139	113059622	ENSMUSG00000002228	ENSMUSG00000002228	ENSMUSG00000002228	3	104909112	104914004	60	62
3022	1	113018566	113044891	ENSMUSG00000002227	ENSMUSG00000002227	ENSMUSG00000002227	3	104922892	104946298	60	62
3023	1	112867663	112963563	ENSMUSG00000045576	ENSMUSG00000045576	ENSMUSG00000045576	3	104993130	105054916	60	62
3024	1	112852923	112866811	ENSMUSG00000027840	ENSMUSG00000027840	ENSMUSG00000027840	3	105072861	105089765	60	62
3025	1	112740326	112805309	ENSMUSG00000062127	ENSMUSG00000062127	ENSMUSG00000062127	3	105129973	105181007	60	62
3026	1	112119954	112333300	ENSMUSG00000040896	ENSMUSG00000040896	ENSMUSG00000040896	3	105586872	105797265	60	62
3027	1	112099742	112111722	ENSMUSG00000027905	ENSMUSG00000027905	ENSMUSG00000027905	3	105806331	105815630	60	62
3028	1	112066209	112083545	ENSMUSG00000048458	ENSMUSG00000048458	ENSMUSG00000048458	3	105832733	105847777	60	62
3029	1	111827493	111847633	ENSMUSG00000074344	ENSMUSG00000074344	ENSMUSG00000074344	3	106042288	106050052	60	62
3030	1	111817937	111822657	ENSMUSG00000074342	ENSMUSG00000074342	ENSMUSG00000074342	3	106053947	106060722	60	62
3031	1	111793081	111806918	ENSMUSG00000000563	ENSMUSG00000000563	ENSMUSG00000000563	3	106070917	106088155	60	62
3032	1	111784035	111793521	ENSMUSG00000000561	ENSMUSG00000000561	ENSMUSG00000000561	3	106087554	106097816	60	62
3033	1	111758466	111771213	ENSMUSG00000074340	ENSMUSG00000074340	ENSMUSG00000074340	3	106101858	106115479	60	62
3034	1	111690433	111697158	ENSMUSG00000010136	ENSMUSG00000010136	ENSMUSG00000010136	3	106125013	106142702	60	62
3035	1	111531319	111544834	ENSMUSG00000027901	ENSMUSG00000027901	ENSMUSG00000027901	3	106614106	106629098	60	62
3036	1	111483772	111529247	ENSMUSG00000040774	ENSMUSG00000040774	ENSMUSG00000040774	3	106630316	106675817	60	62
3037	1	111461478	111484185	ENSMUSG00000027900	ENSMUSG00000027900	ENSMUSG00000027900	3	106675883	106702901	60	62
3038	1	111291335	111308089	ENSMUSG00000056260	ENSMUSG00000056260	ENSMUSG00000056260	3	106849738	106864635	60	62
3039	1	111217284	111244069	ENSMUSG00000040747	ENSMUSG00000040747	ENSMUSG00000040747	3	106887989	106918108	60	62
3040	1	111016610	111019178	ENSMUSG00000047959	ENSMUSG00000047959	ENSMUSG00000047959	3	107164479	107166065	60	62
3041	1	110947300	110949868	ENSMUSG00000040724	ENSMUSG00000040724	ENSMUSG00000040724	3	107229623	107234935	60	62
3042	1	110861396	110862983	ENSMUSG00000042861	ENSMUSG00000042861	ENSMUSG00000042861	3	107322111	107323646	60	62
3043	1	110795272	110801497	ENSMUSG00000070368	ENSMUSG00000070368	ENSMUSG00000070368	3	107363625	107365269	60	62
3044	1	110707005	110735171	ENSMUSG00000027896	ENSMUSG00000027896	ENSMUSG00000027896	3	107419348	107440171	60	62
3045	1	110682651	110690822	ENSMUSG00000048109	ENSMUSG00000048109	ENSMUSG00000048109	3	107454001	107461729	60	62
3046	1	110555488	110578189	ENSMUSG00000027895	ENSMUSG00000027895	ENSMUSG00000027895	3	107566357	107586954	60	62
3047	1	110494631	110546347	ENSMUSG00000027894	ENSMUSG00000027894	ENSMUSG00000027894	3	107595604	107646074	60	62
3048	1	110404139	110414757	ENSMUSG00000014603	ENSMUSG00000014603	ENSMUSG00000014603	3	107723087	107733825	60	62
3049	1	110378761	110398786	ENSMUSG00000014601	ENSMUSG00000014601	ENSMUSG00000014601	3	107740590	107759748	60	62
3050	1	110328831	110367880	ENSMUSG00000027893	ENSMUSG00000027893	ENSMUSG00000027893	3	107793568	107824333	60	62
3051	1	110254778	110275137	ENSMUSG00000014599	ENSMUSG00000014599	ENSMUSG00000014599	3	107869116	107888525	60	62
3052	1	110094226	110108172	ENSMUSG00000040600	ENSMUSG00000040600	ENSMUSG00000040600	3	108007427	108020431	60	62
3053	1	110078077	110084563	ENSMUSG00000004032	ENSMUSG00000004032	ENSMUSG00000004032	3	108023895	108026739	60	62
3054	1	110012227	110019072	ENSMUSG00000004035	ENSMUSG00000004035	ENSMUSG00000004035	3	108054391	108059801	60	62
3055	1	109964799	109976198	ENSMUSG00000027889	ENSMUSG00000027889	ENSMUSG00000027889	3	108202118	108214683	60	62
3056	1	109947298	109957228	ENSMUSG00000009108	ENSMUSG00000009108	ENSMUSG00000009108	3	108221124	108229342	60	62
3057	1	109892756	109938498	ENSMUSG00000000001	ENSMUSG00000000001	ENSMUSG00000000001	3	108235337	108274202	60	62
3058	1	109884032	109891405	ENSMUSG00000046793	ENSMUSG00000046793	ENSMUSG00000046793	3	108276377	108282938	60	62
3059	1	109848320	109853827	ENSMUSG00000050947	ENSMUSG00000050947	ENSMUSG00000050947	3	108314391	108319835	60	62
3060	1	109838244	109844578	ENSMUSG00000048796	ENSMUSG00000048796	ENSMUSG00000048796	3	108323835	108328864	60	62
3061	1	109828084	109836879	ENSMUSG00000048997	ENSMUSG00000048997	ENSMUSG00000048997	3	108330311	108338990	60	62
3062	1	109810773	109826282	ENSMUSG00000027887	ENSMUSG00000027887	ENSMUSG00000027887	3	108339528	108354704	60	62
3063	1	109653715	109742096	ENSMUSG00000068747	ENSMUSG00000068747	ENSMUSG00000068747	3	108412147	108489567	60	62
3064	1	109636510	109651186	ENSMUSG00000068745	ENSMUSG00000068745	ENSMUSG00000068745	3	108492967	108508113	60	62
3065	1	109623699	109627294	ENSMUSG00000068744	ENSMUSG00000068744	ENSMUSG00000068744	3	108511895	108516235	60	62
3066	1	109594164	109619895	ENSMUSG00000068740	ENSMUSG00000068740	ENSMUSG00000068740	3	108520500	108543550	60	62
3067	1	109558127	109582293	ENSMUSG00000068739	ENSMUSG00000068739	ENSMUSG00000068739	3	108554631	108573188	60	62
3068	1	109458056	109551120	ENSMUSG00000040412	ENSMUSG00000040412	ENSMUSG00000040412	3	108586125	108664578	60	62
3069	1	109450096	109456900	ENSMUSG00000027886	ENSMUSG00000027886	ENSMUSG00000027886	3	108665607	108672779	60	62
3070	1	109314359	109386258	ENSMUSG00000040389	ENSMUSG00000040389	ENSMUSG00000040389	3	108719335	108773775	60	62
3071	1	109273653	109307602	ENSMUSG00000027884	ENSMUSG00000027884	ENSMUSG00000027884	3	108782303	108806068	60	62
3072	1	109220896	109274566	ENSMUSG00000027883	ENSMUSG00000027883	ENSMUSG00000027883	3	108806694	108850199	60	62
3073	1	109160043	109201239	ENSMUSG00000049565	ENSMUSG00000049565	ENSMUSG00000049565	3	108879728	108885374	60	62
3074	1	109061922	109086888	ENSMUSG00000045326	ENSMUSG00000045326	ENSMUSG00000045326	3	108983377	109017762	60	62
3075	1	109036468	109045948	ENSMUSG00000027881	ENSMUSG00000027881	ENSMUSG00000027881	3	109030861	109039760	60	62
3076	1	108992440	109005200	ENSMUSG00000045662	ENSMUSG00000045662	ENSMUSG00000045662	3	109068140	109088732	60	62
3077	1	108904622	108983472	ENSMUSG00000040339	ENSMUSG00000040339	ENSMUSG00000040339	3	109101928	109155329	60	62
3078	1	108478965	108544421	ENSMUSG00000040322	ENSMUSG00000040322	ENSMUSG00000040322	3	109251223	109296509	60	62
3079	1	107915305	108309108	ENSMUSG00000033721	ENSMUSG00000033721	ENSMUSG00000033721	3	109468739	109813750	60	62
3080	1	107484268	107825973	ENSMUSG00000059857	ENSMUSG00000059857	ENSMUSG00000059857	3	109910738	110263564	60	62
3081	1	107400824	107403439	ENSMUSG00000049300	ENSMUSG00000049300	ENSMUSG00000049300	3	110374168	110379049	60	62
3082	1	103841281	103842952	ENSMUSG00000027981	ENSMUSG00000027981	ENSMUSG00000027981	3	113597071	113622153	60	62
3083	1	103115583	103346640	ENSMUSG00000027966	ENSMUSG00000027966	ENSMUSG00000027966	3	114022729	114212291	60	62
3084	1	102041630	102085117	ENSMUSG00000027965	ENSMUSG00000027965	ENSMUSG00000027965	3	115072969	115117260	60	62
3085	1	101475032	101479662	ENSMUSG00000045092	ENSMUSG00000045092	ENSMUSG00000045092	3	115702443	115707051	60	62
3086	1	101228207	101263916	ENSMUSG00000033554	ENSMUSG00000033554	ENSMUSG00000033554	3	115880187	115921336	60	62
3087	1	101134220	101219897	ENSMUSG00000054414	ENSMUSG00000054414	ENSMUSG00000054414	3	115937949	115999383	60	62
3088	1	101110531	101134142	ENSMUSG00000027963	ENSMUSG00000027963	ENSMUSG00000027963	3	115999481	116020876	60	62
3089	1	100957885	100977185	ENSMUSG00000027962	ENSMUSG00000027962	ENSMUSG00000027962	3	116102024	116121692	60	62
3090	1	100776281	100780162	ENSMUSG00000068696	ENSMUSG00000068696	ENSMUSG00000068696	3	116243510	116244664	60	62
3091	1	100590540	100737609	ENSMUSG00000033502	ENSMUSG00000033502	ENSMUSG00000033502	3	116265849	116416027	60	62
3092	1	100504351	100529915	ENSMUSG00000000339	ENSMUSG00000000339	ENSMUSG00000000339	3	116480970	116500150	60	62
3093	1	100432324	100487978	ENSMUSG00000000340	ENSMUSG00000000340	ENSMUSG00000000340	3	116505111	116541982	60	62
3094	1	100386997	100416359	ENSMUSG00000027961	ENSMUSG00000027961	ENSMUSG00000027961	3	116555026	116575138	60	62
3095	1	100371297	100388632	ENSMUSG00000033439	ENSMUSG00000033439	ENSMUSG00000033439	3	116573340	116606591	60	62
3096	1	100321707	100371099	ENSMUSG00000027959	ENSMUSG00000027959	ENSMUSG00000027959	3	116587012	116621151	60	62
3097	1	100276241	100321521	ENSMUSG00000027958	ENSMUSG00000027958	ENSMUSG00000027958	3	116623174	116654619	60	62
3098	1	100208681	100261594	ENSMUSG00000027957	ENSMUSG00000027957	ENSMUSG00000027957	3	116662802	116704284	60	62
3099	1	100088228	100162164	ENSMUSG00000033400	ENSMUSG00000033400	ENSMUSG00000033400	3	116735055	116750309	60	62
3100	1	99946847	100003937	ENSMUSG00000033386	ENSMUSG00000033386	ENSMUSG00000033386	3	116870231	116895646	60	62
3101	1	99884186	99932823	ENSMUSG00000033377	ENSMUSG00000033377	ENSMUSG00000033377	3	116910272	116960753	60	62
3102	1	99502097	99547734	ENSMUSG00000044667	ENSMUSG00000044667	ENSMUSG00000044667	3	117311141	117352880	60	62
3103	1	99128391	99243037	ENSMUSG00000033342	ENSMUSG00000033342	ENSMUSG00000033342	3	117567465	117681510	60	62
3104	1	98899801	98998642	ENSMUSG00000028007	ENSMUSG00000028007	ENSMUSG00000028007	3	117773501	117860896	60	62
3105	1	97315887	98159167	ENSMUSG00000033308	ENSMUSG00000033308	ENSMUSG00000033308	3	118554180	119424922	60	62
3106	1	96959954	97052937	ENSMUSG00000028134	ENSMUSG00000028134	ENSMUSG00000028134	3	119712332	119773546	60	62
3107	1	95472299	95485369	ENSMUSG00000028133	ENSMUSG00000028133	ENSMUSG00000028133	3	121147888	121163717	60	62
3108	1	95355482	95435751	ENSMUSG00000028132	ENSMUSG00000028132	ENSMUSG00000028132	3	121194031	121255339	60	62
3109	1	95220885	95311089	ENSMUSG00000039887	ENSMUSG00000039887	ENSMUSG00000039887	3	121283796	121354041	60	62
3110	1	95135095	95165422	ENSMUSG00000053931	ENSMUSG00000053931	ENSMUSG00000053931	3	121418564	121450228	60	62
3111	1	95058523	95133389	ENSMUSG00000039865	ENSMUSG00000039865	ENSMUSG00000039865	3	121451551	121524361	60	62
3112	1	94767369	94779944	ENSMUSG00000028128	ENSMUSG00000028128	ENSMUSG00000028128	3	121715560	121727071	60	62
3113	1	94656599	94756807	ENSMUSG00000028127	ENSMUSG00000028127	ENSMUSG00000028127	3	121750933	121807238	60	62
3114	1	94409895	94475709	ENSMUSG00000039831	ENSMUSG00000039831	ENSMUSG00000039831	3	121945349	122008176	60	62
3115	1	94230984	94359279	ENSMUSG00000028125	ENSMUSG00000028125	ENSMUSG00000028125	3	122036482	122172083	60	62
3116	1	94123349	94147554	ENSMUSG00000028124	ENSMUSG00000028124	ENSMUSG00000028124	3	122237614	122258670	60	62
3117	1	94104427	94117330	ENSMUSG00000039756	ENSMUSG00000039756	ENSMUSG00000039756	3	122266436	122277293	60	62

3118	1	93799936	94085294	ENSMUSG00000028121	ENSMUSG00000028121	ENSMUSG00000028121	3	122411820	122522202	60	62		
3119	1	93686313	93792806	ENSMUSG00000039735	ENSMUSG00000039735	ENSMUSG00000039735	3	122534130	122582265	60	62		
3120	4	120457854	120462766	ENSMUSG00000023057	ENSMUSG00000023057	ENSMUSG00000023057	3	122887398	122891525	61	63		
3121	4	120380363	120433814	ENSMUSG00000039701	ENSMUSG00000039701	ENSMUSG00000039701	3	122925623	122976469	61	63		
3122	4	120276469	120328383	ENSMUSG00000028116	ENSMUSG00000028116	ENSMUSG00000028116	3	122998230	123026971	61	63		
3123	4	120029444	120201841	ENSMUSG00000050315	ENSMUSG00000050315	ENSMUSG00000050315	3	123104424	123228029	61	63		
3124	4	119863427	119976714	ENSMUSG00000039234	ENSMUSG00000039234	ENSMUSG00000039234	3	123259518	123357658	61	63		
3125	4	119825995	119851523	ENSMUSG00000028114	ENSMUSG00000028114	ENSMUSG00000028114	3	123360321	123377970	61	63		
3126	4	119421865	119493370	ENSMUSG00000027978	ENSMUSG00000027978	ENSMUSG00000027978	3	123438935	123498625	61	63		
3127	4	119175060	119399235	ENSMUSG00000027977	ENSMUSG00000027977	ENSMUSG00000027977	3	123518186	123664414	61	63		
3128	4	118224888	118225997	ENSMUSG00000044528	ENSMUSG00000044528	ENSMUSG00000044528	3	124313109	124316765	61	63		
3129	4	115968378	116254481	ENSMUSG00000027971	ENSMUSG00000027971	ENSMUSG00000027971	3	125429702	125716801	61	63		
3130	4	115739060	115816934	ENSMUSG00000032854	ENSMUSG00000032854	ENSMUSG00000032854	3	125857361	125930568	61	63		
3131	4	114595014	114902177	ENSMUSG00000053819	ENSMUSG00000053819	ENSMUSG00000053819	3	126589462	126832391	61	63		
3132	4	114190319	114524334	ENSMUSG00000032826	ENSMUSG00000032826	ENSMUSG00000032826	3	126951029	127490761	61	63		
3133	4	113679949	113728256	ENSMUSG00000051278	ENSMUSG00000051278	ENSMUSG00000051278	3	127545511	127610038	61	63		
3134	4	113654125	113656777	ENSMUSG00000027967	ENSMUSG00000027967	ENSMUSG00000027967	3	127625167	127627653	61	63		
3135	4	113438006	113582216	ENSMUSG00000028028	ENSMUSG00000028028	ENSMUSG00000028028	3	127662954	127676462	61	63		
3136	4	113416232	113426508	ENSMUSG00000046688	ENSMUSG00000046688	ENSMUSG00000046688	3	127781941	127790411	61	63		
3137	4	113372392	113410652	ENSMUSG00000047396	ENSMUSG00000047396	ENSMUSG00000047396	3	127807675	127829247	61	63		
3138	4	113286127	113329678	ENSMUSG00000050549	ENSMUSG00000050549	ENSMUSG00000050549	3	127862563	127888312	61	63		
3139	4	111758033	111763703	ENSMUSG00000028023	ENSMUSG00000028023	ENSMUSG00000028023	3	129191965	129211613	61	63		
3140	4	111616697	111702812	ENSMUSG00000028024	ENSMUSG00000028024	ENSMUSG00000028024	3	129261642	129324742	61	63		
3141	4	111189992	111339220	ENSMUSG00000041220	ENSMUSG00000041220	ENSMUSG00000041220	3	129524427	129630513	61	63		
3142	4	111053499	111152860	ENSMUSG00000028017	ENSMUSG00000028017	ENSMUSG00000028017	3	129669600	129747338	61	63		
3143	4	110991935	111012919	ENSMUSG00000068627	ENSMUSG00000068627	ENSMUSG00000068627	3	129780313	129783583	61	63		
3144	4	110968599	110985312	ENSMUSG00000028012	ENSMUSG00000028012	ENSMUSG00000028012	3	129796430	129814515	61	63		
3145	4	110956115	110965340	ENSMUSG00000028010	ENSMUSG00000028010	ENSMUSG00000028010	3	129817568	129822863	61	63		
3146	4	110881301	110942590	ENSMUSG00000058952	ENSMUSG00000058952	ENSMUSG00000058952	3	129828759	129867350	61	63		
3147	4	110854140	110870660	ENSMUSG00000027999	ENSMUSG00000027999	ENSMUSG00000027999	3	129870628	129887841	61	63		
3148	4	110829234	110844078	ENSMUSG00000027997	ENSMUSG00000027997	ENSMUSG00000027997	3	129897501	129905595	61	63		
3149	4	110700897	110829312	ENSMUSG00000027994	ENSMUSG00000027994	ENSMUSG00000027994	3	129907001	129962228	61	63		
3150	4	110574320	110681058	ENSMUSG00000001052	ENSMUSG00000001052	ENSMUSG00000001052	3	129975205	130052929	61	63		
3151	4	109958002	110442624	ENSMUSG00000058897	ENSMUSG00000058897	ENSMUSG00000058897	3	130170639	130580987	61	63		
3152	4	109882657	109903658	ENSMUSG00000019232	ENSMUSG00000019232	ENSMUSG00000019232	3	130606730	130624903	61	63		
3153	4	109188150	109309027	ENSMUSG00000027985	ENSMUSG00000027985	ENSMUSG00000027985	3	131099626	131212499	61	63		
3154	4	109130319	109175466	ENSMUSG00000027984	ENSMUSG00000027984	ENSMUSG00000027984	3	131222609	131261198	61	63		
3155	4	109072166	109094060	ENSMUSG00000027983	ENSMUSG00000027983	ENSMUSG00000027983	3	131284541	131292282	61	63		
3156	4	108965267	109051504	ENSMUSG00000050931	ENSMUSG00000050931	ENSMUSG00000050931	3	131311893	131334090	61	63		
3157	4	108754272	108860868	ENSMUSG00000028032	ENSMUSG00000028032	ENSMUSG00000028032	3	131502197	131580977	61	63		
3158	4	108062418	108176903	ENSMUSG00000028031	ENSMUSG00000028031	ENSMUSG00000028031	3	132022607	132117616	61	63		
3159	4	107186689	107457244	ENSMUSG00000028030	ENSMUSG00000028030	ENSMUSG00000028030	3	132624106	132711902	61	63		
3160	4	107036054	107112273	ENSMUSG00000040998	ENSMUSG00000040998	ENSMUSG00000040998	3	132821736	132887349	61	63		
3161	4	106851419	106986355	ENSMUSG00000028018	ENSMUSG00000028018	ENSMUSG00000028018	3	132919059	133029084	61	63		
3162	4	106823236	106840622	ENSMUSG00000028016	ENSMUSG00000028016	ENSMUSG00000028016	3	133029259	133048294	61	63		
3163	4	106693226	106772285	ENSMUSG00000056559	ENSMUSG00000056559	ENSMUSG00000056559	3	133096815	133172195	61	63		
3164	4	106509684	106614676	ENSMUSG00000028013	ENSMUSG00000028013	ENSMUSG00000028013	3	133247422	133315377	61	63		
3165	4	106402364	106420407	ENSMUSG00000040943	ENSMUSG00000040943	ENSMUSG00000040943	3	133401145	133481674	61	63		
3166	4	105609015	105635500	ENSMUSG00000044365	ENSMUSG00000044365	ENSMUSG00000044365	3	134173884	134199367	61	63		
3167	4	104730074	104860422	ENSMUSG00000028172	ENSMUSG00000028172	ENSMUSG00000028172	3	134766394	134871959	61	63		
3168	4	104246412	104339015	ENSMUSG00000045328	ENSMUSG00000045328	ENSMUSG00000045328	3	135150046	135210626	61	63		
3169	4	104218232	104240473	ENSMUSG00000028167	ENSMUSG00000028167	ENSMUSG00000028167	3	135218679	135241813	61	63		
3170	4	104160840	104216909	ENSMUSG00000037994	ENSMUSG00000037994	ENSMUSG00000037994	3	135245134	135279927	61	63		
3171	4	103641518	103757506	ENSMUSG00000028163	ENSMUSG00000028163	ENSMUSG00000028163	3	135292997	135605172	61	63		
3172	4	104009639	104029833	ENSMUSG00000028165	ENSMUSG00000028165	ENSMUSG00000028165	3	135343799	135360820	61	63		
3173	4	103771696	103901186	ENSMUSG00000028164	ENSMUSG00000028164	ENSMUSG00000028164	3	135423055	135508791	61	63		
3174	4	103401847	103485654	ENSMUSG00000053897	ENSMUSG00000053897	ENSMUSG00000053897	3	135762878	135825773	61	63		
3175	4	102163610	102487376	ENSMUSG00000028161	ENSMUSG00000028161	ENSMUSG00000028161	3	136608220	136873264	61	63		
3176	4	101538009	101658202	ENSMUSG00000054690	ENSMUSG00000054690	ENSMUSG00000054690	3	137278498	137369572	61	63		
3177	4	101326052	101330648	ENSMUSG00000046818	ENSMUSG00000046818	ENSMUSG00000046818	3	137561123	137565720	61	63		
3178	4	101039591	101086760	ENSMUSG00000074212	ENSMUSG00000074212	ENSMUSG00000074212	3	137842159	137845856	61	63		
3179	4	100957013	101010334	ENSMUSG00000028159	ENSMUSG00000028159	ENSMUSG00000028159	3	137868992	137918911	61	63		
3180	4	100715004	100763649	ENSMUSG00000028158	ENSMUSG00000028158	ENSMUSG00000028158	3	138028273	138068711	61	63		
3181	4	100686910	100703848	ENSMUSG00000004127	ENSMUSG00000004127	ENSMUSG00000004127	3	138080925	138097208	61	63		
3182	4	100651197	100682463	ENSMUSG00000012042	ENSMUSG00000012042	ENSMUSG00000012042	3	138101522	138124100	61	63		
3183	4	100552441	100575548	ENSMUSG00000055301	ENSMUSG00000055301	ENSMUSG00000055301	3	138159100	138168593	61	63		
3184	4	100263855	100284472	ENSMUSG00000037797	ENSMUSG00000037797	ENSMUSG00000037797	3	138352896	138368183	61	63		
3185	4	100212327	100225399	ENSMUSG00000028138	ENSMUSG00000028138	ENSMUSG00000028138	3	138380551	138392886	61	63		
3186	4	100135903	100202976	ENSMUSG00000005813	ENSMUSG00000005813	ENSMUSG00000005813	3	138396347	138426769	61	63		
3187	4	99612435	99798750	ENSMUSG00000028152	ENSMUSG00000028152	ENSMUSG00000028152	3	138679582	138841811	61	63		
3188	4	99401692	99582508	ENSMUSG00000028149	ENSMUSG00000028149	ENSMUSG00000028149	3	138864972	138987976	61	63		
3189	4	98699050	99283414	ENSMUSG00000047940	ENSMUSG00000047940	ENSMUSG00000047940	3	139143280	139647686	61	63		
3190	4	96980266	96981645	ENSMUSG00000047674	ENSMUSG00000047674	ENSMUSG00000047674	3	141147957	141149132	61	63		
3191	4	96309408	96689031	ENSMUSG00000059921	ENSMUSG00000059921	ENSMUSG00000059921	3	141403101	141765959	61	63		
3192	4	95898151	96295099	ENSMUSG00000052430	ENSMUSG00000052430	ENSMUSG00000052430	3	141774523	141919638	61	63		
3193	4	95592061	95808400	ENSMUSG00000028273	ENSMUSG00000028273	ENSMUSG00000028273	3	142179594	142333029	61	63		
3194	1	89498853	89509134	ENSMUSG00000040264	ENSMUSG00000040264	ENSMUSG00000040264	3	142434320	142459728	62	64		
3195	1	89174044	89231104	ENSMUSG00000040213	ENSMUSG00000040213	ENSMUSG00000040213	3	142638466	142682296	62	64		
3196	1	88922493	89074526	ENSMUSG00000004591	ENSMUSG00000004591	ENSMUSG00000004591	3	142731197	142791100	62	64		
3197	1	87566739	87585376	ENSMUSG00000028266	ENSMUSG00000028266	ENSMUSG00000028266	3	144131276	144139434	62	64		
3198	1	87152950	87348269	ENSMUSG00000040151	ENSMUSG00000040151	ENSMUSG00000040151	3	144368489	144507525	62	64		
3199	1	87100720	87152636	ENSMUSG00000037072	ENSMUSG00000037072	ENSMUSG00000037072	3	144508111	144534340	62	64		
3200	1	86943166	86981539	ENSMUSG00000037062	ENSMUSG00000037062	ENSMUSG00000037062	3	144626230	144657709	62	64		
3201	1	86706639	86738532	ENSMUSG00000028255	ENSMUSG00000028255	ENSMUSG00000028255	3	144941923	144970158	62	64		
3202	1	86662357	86694829	ENSMUSG00000036960	ENSMUSG00000036960	ENSMUSG00000036960	3	145007653	145036490	62	64		
3203	1	86587009	86634591	ENSMUSG00000028256	ENSMUSG00000028256	ENSMUSG00000028256	3	145055994	145090911	62	64		
3204	1	85967504	86394709	ENSMUSG00000028197	ENSMUSG00000028197	ENSMUSG00000028197	3	145229858	145489391	62	64		
3205	1	85891079	85946689	ENSMUSG00000074182	ENSMUSG00000074182	ENSMUSG00000074182	3	145513754	145541361	62	64		
3206	1	85819032	85822233	ENSMUSG00000028195	ENSMUSG00000028195	ENSMUSG00000028195	3	145584362	145587367	62	64		
3207	1	85556756	85703411	ENSMUSG00000028194	ENSMUSG00000028194	ENSMUSG00000028194	3	145696113	145829059	62	64		
3208	1	85504519	85516359	ENSMUSG00000028191	ENSMUSG00000028191	ENSMUSG00000028191	3	145861736	145871669	62	64		
3209	1	85488227	85497943	ENSMUSG00000036873	ENSMUSG00000036873	ENSMUSG00000036873	3	145875428	145882213	62	64		
3210	1	85395944	85439043	ENSMUSG00000036863	ENSMUSG00000036863	ENSMUSG00000036863	3	145926039	145958429	62	64		
3211	1	85237418	85371408	ENSMUSG00000043020	ENSMUSG00000043020	ENSMUSG00000043020	3	145977913	146045422	62	64		
3212	1	85256353	85286717	ENSMUSG00000036853	ENSMUSG00000036853	ENSMUSG00000036853	3	146059064	146079192	62	64		
3213	1	85163855	85235384	ENSMUSG00000011008	ENSMUSG00000011008	ENSMUSG00000011008	3	146087229	146132898	62	64		
3214	1	85049873	85104430	ENSMUSG00000036832	ENSMUSG00000036832	ENSMUSG00000036832	3	146158371	146223572	62	64		
3215	1	84881978	84928816	ENSMUSG00000036825	ENSMUSG00000036825	ENSMUSG00000036825	3	146342041	146377521	62	64		
3216	1	84636803	84653289	ENSMUSG00000028185	ENSMUSG00000028185	ENSMUSG00000028185	3	146342199	146533998	62	64		
3217	1	84744601	84791355	ENSMUSG00000028188	ENSMUSG00000028188	ENSMUSG00000028188	3	146394589	146437139	62	64		
3218	1	84736596	84744836	ENSMUSG00000068523	ENSMUSG00000068523	ENSMUSG00000068523	3	146437222	146442929	62	64		
3219	1	84717538	84736061	ENSMUSG00000028187	ENSMUSG00000028187	ENSMUSG00000028187	3	146443734	146458794	62	64		
3220	1	84382542	84476769	ENSMUSG00000005034	ENSMUSG00000005034	ENSMUSG00000005034	3	146666960	146718719	62	64		
3221	1	84103299	84237403	ENSMUSG00000036745	ENSMUSG00000036745	ENSMUSG00000036745	3	146789793	146877642	62	64		
3222	1	81544439	82229640	ENSMUSG00000028184	ENSMUSG00000028184	ENSMUSG00000028184	3	148753242	148789342	62	64		
3223	1	79128037	79244991	ENSMUSG00000039167	ENSMUSG00000039167	ENSMUSG00000039167	3	151375273	151482474	62	64		
3224	1	78888104	78902351	ENSMUSG00000028037	ENSMUSG00000028037	ENSMUSG00000028037	3	151668310	151687235	62	64		
3225	1	78542156	78778022	ENSMUSG00000028036	ENSMUSG00000028036	ENSMUSG00000028036	3	151737371	151774818	62	64		
3226	1	78284174	78376721	ENSMUSG00000039131	ENSMUSG00000039131	ENSMUSG00000039131	3	152031229	152103336	62	64		
3227	1	78243126	78256236	ENSMUSG00000028035	ENSMUSG00000028035	ENSMUSG00000028035	3	152121482	152131189	62	64		
3228	1	78186678	78217364	ENSMUSG00000028034	ENSMUSG00000028034	ENSMUSG00000028034	3	152147884	152170157	62	64		
3229	1	78126901	78181497	ENSMUSG00000039103	ENSMUSG00000039103	ENSMUSG00000039103	3	152174368	152203648	62	64		
3230	1	78017897	78116694	ENSMUSG00000054942	ENSMUSG00000054942	ENSMUSG00000054942	3	152211235	152277776	62	64		
3231	1	77934264	77983735	ENSMUSG00000025437	ENSMUSG00000025437	ENSMUSG00000025437	3	152283875	152330580	62	64		
3232	1	77802773	77920931	ENSMUSG00000039068	ENSMUSG00000039068	ENSMUSG00000039068	3	152334080	152396867	62	64		
3233	1	77520324	77798238	ENSMUSG00000039058	ENSMUSG00000039058	ENSMUSG00000039058	3	152470727	152605245	62	64		
3234	1	77327263	77457699	ENSMUSG00000039047	ENSMUSG00000039047	ENSMUSG00000039047	3	152651500	152726397	62	64		
3235	1	77105714	77302307	ENSMUSG00000039037	ENSMUSG00000039037	ENSMUSG00000039037	3	152758897	152918698	62	64		
3236	1	76312992	76872874	ENSMUSG00000052544	ENSMUSG00000052544	ENSMUSG00000052544	3	153142791	153446901	62	64		
3237	1	76157146	76170704	ENSMUSG00000038997	ENSMUSG00000038997	ENSMUSG00000038997	3	153781628	153791001	62	64		
3238	1	76035259	76151511	ENSMUSG00000005493	ENSMUSG00000005493	ENSMUSG00000005493	3	153794533	153843370	62	64		
3239	1	76025769	76033058	ENSMUSG00000038975	ENSMUSG00000038975	ENSMUSG00000038975	3	153844840	153849445	62	64		
3240	1	75442748	75849357	ENSMUSG00000028360	ENSMUSG00000028360	ENSMUSG00000028360	3	153910822	154207000	62	64		
3241	1	75366707	75399806	ENSMUSG00000028201	ENSMUSG00000028201	ENSMUSG00000028201	3	154241591	154265822	62	64		
3242	1	74971428	75004946	ENSMUSG00000047583	ENSMUSG00000047583	ENSMUSG00000047583	3	154512492	154532388	62	64		
3243	1	74943776	74971347	ENSMUSG00000028199	ENSMUSG00000028199	ENSMUSG00000028199	3	154532492	154558475	62	64		
3244	1	74809389	74912010	ENSMUSG00000042943	ENSMUSG00000042943	ENSMUSG00000042943	3	154649179	154700116	62	64		
3245	1	74436551	74782696	ENSMUSG00000040086	ENSMUSG00000040086	ENSMUSG00000040086	3	154721595	154990700	62	64		
3246	1	74279673	74421956	ENSMUSG00000028182	ENSMUSG00000028182	ENSMUSG00000028182	3	155033998	155123683	62	64		
3247	1	71641213	72521005	ENSMUSG00000040037	ENSMUSG00000040037	ENSMUSG00000040037	3	156497488	157248203	62	64		
3248	1	71301563	71319333	ENSMUSG00000028180	ENSMUSG00000028180	ENSMUSG00000028180	3	157469680	157483633	62	64		
3249	1	71090624	71286079	ENSMUSG00000040016	ENSMUSG00000040016	ENSMUSG00000040016	3	157502311	157577106	62	64		
3250	1	70649543	70677841	ENSMUSG00000028179	ENSMUSG00000028179	ENSMUSG00000028179	3	157832479	157860323	62	64		
3251	1	70498859	70592993	ENSMUSG00000039988	ENSMUSG00000039988	ENSMUSG00000039988	3	157882544	157942128	62	64		
3252	1	70443995	70491323	ENSMUSG00000055436	ENSMUSG00000055436	ENSMUSG00000055436	3	157946214	157966926	62	64		
3253	1	70383076	70443891	ENSMUSG00000063052	ENSMUSG00000063052	ENSMUSG00000063052	3	157971996	158003771	62	64		
3254	1	68667033	68688230	ENSMUSG00000028174	ENSMUSG00000028174	ENSMUSG00000028174	3	159535602	159560092	62	64		
3255	8	56813874	56848439	ENSMUSG00000028232	ENSMUSG00000028232	ENSMUSG00000028232	4	3476188	3501915	63	65		
3256	8	56954948	57086493	ENSMUSG00000042228	ENSMUSG00000042228	ENSMUSG00000042228	4	3605272	3718759	63	65		
3257	8	57236037	57286392	ENSMUSG00000003282	ENSMUSG00000003282	ENSMUSG00000003282	4	3829756	3865529	63	65		
3258	8	57286869	57293728	ENSMUSG00000042198	ENSMUSG00000042198	ENSMUSG00000042198	4	3866061	3870670	63	65		
3259	8	57375122	57395795	ENSMUSG00000028236	ENSMUSG00000028236	ENSMUSG00000028236	4	3923089	3946810	63	65		
3260	8	57516071	57521143	ENSMUSG00000045573	ENSMUSG00000045573	ENSMUSG00000045573	4	4060680	4065592	63	65		
3261	8	58037822	58068957	ENSMUSG00000066324	ENSMUSG00000066324	ENSMUSG00000066324	4	4691503	4720502	63	65		
3262	8	59069667	59222812	ENSMUSG00000049119	ENSMUSG00000049119	ENSMUSG00000049119	4	5571326	5727091	63	65		
3263	8	59565292	59575275	ENSMUSG00000028240	ENSMUSG00000028240	ENSMUSG00000028240	4	6192759	6202778	63	65		
3264	8	59628282	59657972	ENSMUSG00000028249	ENSMUSG00000028249	ENSMUSG00000028249	4	6292830	6323431	63	65		
3265	8	59658624	59734940	ENSMUSG00000028245	ENSMUSG00000028245	ENSMUSG00000028245	4	6323371	6381408	63	65		
3266	8	59880531	60194321	ENSMUSG00000041272	ENSMUSG00000041272	ENSMUSG00000041272	4	6614605	6917870	63	65		
3267	8	61263977	61356508	ENSMUSG00000041261	ENSMUSG00000041261	ENSMUSG00000041261	4	8068640	8166188	63	65		
3268	8	61592316	61696850	ENSMUSG00000047187	ENSMUSG00000047187	ENSMUSG00000047187	4	8462824	8534925	63	65		
3269	8	62363104	62576756	ENSMUSG00000041216	ENSMUSG00000041216	ENSMUSG00000041216	4	9196509	9378838	63	65		
3270	8	62578374	62789560	ENSMUSG00000028207	ENSMUSG00000028207	ENSMUSG00000028207	4	9378438	9596231	63	65		
3271	8	97223736	97242196	ENSMUSG00000051279	ENSMUSG00000051279	ENSMUSG00000051279	4	9771519	9789492	64	66		
3272	8	96326369	96350594	ENSMUSG00000059482	ENSMUSG00000059482	ENSMUSG00000059482	4	10801755	10826572	64	66		
3273	8	96215208	96238087	ENSMUSG00000049969	ENSMUSG00000049969	ENSMUSG00000049969	4	10915810	10934717	64	66		
3274	8	96007377	96030770	ENSMUSG00000028211	ENSMUSG00000028211	ENSMUSG00000028211	4	11083588	11101524	64	66		
3275	8	95961631	95976660	ENSMUSG00000028212	ENSMUSG00000028212	ENSMUSG00000028212	4	11118501	11131829	64	66		
3276	8	95904710	95961896	ENSMUSG00000040738	ENSMUSG00000040738	ENSMUSG00000040738	4	11126305	11181376	64	66		
3277	8	95801325	95873238	ENSMUSG00000045205	ENSMUSG00000045205	ENSMUSG00000045205	4	11192928	11244328	64	66		
3278	8	95722589	95787843	ENSMUSG00000040728	ENSMUSG00000040728	ENSMUSG00000040728	4	11265904	11313583	64	66		
3279	8	95569776	95634864	ENSMUSG00000040720	ENSMUSG00000040720	ENSMUSG00000040720	4	11413190	11476843	64	66		
3280	8	95453365	95556486	ENSMUSG00000066307	ENSMUSG00000066307	ENSMUSG00000066307	4	11486119	11542954	64	66		
3281	8	95330663	95343733	ENSMUSG00000028214	ENSMUSG00000028214	ENSMUSG00000028214	4	11631604	11641899	64	66		
3282	8	95208566	95289986	ENSMUSG00000028217	ENSMUSG00000028217	ENSMUSG00000028217	4	11685362	11745042	64	66		
3283	8	94998338	95007470	ENSMUSG00000049225	ENSMUSG00000049225	ENSMUSG00000049225	4	11886958	11893567	64	66		
3284	8	94836306	94898322	ENSMUSG00000049488	ENSMUSG00000049488	ENSMUSG00000049488	4	11966792	12015104	64	66		
3285	8	93040328	93157540	ENSMUSG00000006586	ENSMUSG00000006586	ENSMUSG00000006586	4	13670583	13818712	64	66		
3286	8	92330692	92476122	ENSMUSG00000040569	ENSMUSG00000040569	ENSMUSG00000040569	4	14432342	14548904	64	66		
3287	8	92075680	92122226	ENSMUSG00000028221	ENSMUSG00000028221	ENSMUSG00000028221	4	14791366	14842407	64	66		
3288	8	91873291	92040806	ENSMUSG00000040536	ENSMUSG00000040536	ENSMUSG00000040536	4	14879394	15076278	64	66		
3289	8	91707078	91727309	ENSMUSG00000043252	ENSMUSG00000043252	ENSMUSG00000043252	4	15192967	15212517	64	66		
3290	8	91140014	91164283	ENSMUSG00000028222	ENSMUSG00000028222	ENSMUSG00000028222	4	15808411	15835211	64	66		
3291	8	91082756	91133403	ENSMUSG00000028223	ENSMUSG00000028223	ENSMUSG00000028223	4	15844384	15872654	64	66		
3292	8	91014740	91066075	ENSMUSG00000028224	ENSMUSG00000028224	ENSMUSG00000028224	4	15885115	15919736	64	66		
3293	8	90983269	91009271	ENSMUSG00000041153	ENSMUSG00000041153	ENSMUSG00000041153	4	15924268	15941024	64	66		
3294	8	90839173	90872432	ENSMUSG00000041135	ENSMUSG00000041135	ENSMUSG00000041135	4	16049889	16090790	64	66		
3295	8	89118580	89408892	ENSMUSG00000028226	ENSMUSG00000028226	ENSMUSG00000028226	4	17780747	18045881	64	66		
3296	8	87947840	88435135	ENSMUSG00000073991	ENSMUSG00000073991	ENSMUSG00000073991	4	18787596	18834288	64	66		
3297	8	87655280	87825017	ENSMUSG00000056494	ENSMUSG00000056494	ENSMUSG00000056494	4	19208080	19432497	64	66		
3298	8	87566186	87642842	ENSMUSG00000028228	ENSMUSG00000028228	ENSMUSG00000028228	4	19446399	19497251	64	66		
3299	8	87555454	87590037	ENSMUSG00000028229	ENSMUSG00000028229	ENSMUSG00000028229	4	19502354	19534079	64	66		
3300	8	87295413	87311720	ENSMUSG00000041052	ENSMUSG00000041052	ENSMUSG00000041052	4	19745891	19769365	64	66		
3301	8	87180249	87235573	ENSMUSG00000028238	ENSMUSG00000028238	ENSMUSG00000028238	4	19803985	19849705	64	66		
3302	8	64134926	64161152	ENSMUSG00000073988	ENSMUSG00000073988	ENSMUSG00000073988	4	19935575	19957932	65	66	here	Sub block(s) comes here, so ST did not merge
3303	8	63654652	63993625	ENSMUSG00000055761	ENSMUSG00000055761	ENSMUSG00000055761	4	20231103	20575856	65	66	sub blocks	2 -> 1
3304	6	100161632	100170175	ENSMUSG00000040478	ENSMUSG00000040478	ENSMUSG00000040478	4	21769059	21776798	66	67		
3305	6	99990313	100064817	ENSMUSG00000040455	ENSMUSG00000040455	ENSMUSG00000040455	4	21867133	21928707	66	67		
3306	6	99952648	99979905	ENSMUSG00000028248	ENSMUSG00000028248	ENSMUSG00000028248	4	21938883	21967310	66	67		
3307	6	99923997	99948801	ENSMUSG00000028247	ENSMUSG00000028247	ENSMUSG00000028247	4	21970510	22002994	66	67		
3308	6	99835761	99904014	ENSMUSG00000028246	ENSMUSG00000028246	ENSMUSG00000028246	4	22022192	22084422	66	67		
3309	6	99428055	99502523	ENSMUSG00000040410	ENSMUSG00000040410	ENSMUSG00000040410	4	22448400	22524926	66	67		
3310	6	99389301	99393381	ENSMUSG00000052621	ENSMUSG00000052621	ENSMUSG00000052621	4	22577629	22578966	66	67		
3311	6	97701132	97837773	ENSMUSG00000045751	ENSMUSG00000045751	ENSMUSG00000045751	4	24595792	24693579	66	67		
3312	6	97479329	97694980	ENSMUSG00000040387	ENSMUSG00000040387	ENSMUSG00000040387	4	24707903	24941687	66	67		

3313	6	97443910	97452478	ENSMUSG00000028261	ENSMUSG00000028261	ENSMUSG00000028261	4	24988731	24995631	66	67		
3314	6	97352645	97391921	ENSMUSG00000040372	ENSMUSG00000040372	ENSMUSG00000040372	4	25064049	25099863	66	67		
3315	6	97117156	97171233	ENSMUSG00000028259	ENSMUSG00000028259	ENSMUSG00000028259	4	25290533	25333506	66	67		
3316	6	97076451	97109870	ENSMUSG00000040359	ENSMUSG00000040359	ENSMUSG00000040359	4	25339231	25372390	66	67		
3317	6	96570590	96760477	ENSMUSG00000055373	ENSMUSG00000055373	ENSMUSG00000055373	4	25699963	25890633	66	67		
3318	6	96132140	96164054	ENSMUSG00000040520	ENSMUSG00000040520	ENSMUSG00000040520	4	26415136	26437282	66	67		
3319	6	94006459	94185965	ENSMUSG00000028289	ENSMUSG00000028289	ENSMUSG00000028289	4	28981940	29133870	66	67		
3320	6	91280013	91353485	ENSMUSG00000028284	ENSMUSG00000028284	ENSMUSG00000028284	4	32292729	32349408	66	67		
3321	6	90692969	91063182	ENSMUSG00000040270	ENSMUSG00000040270	ENSMUSG00000040270	4	32830238	32909894	66	67		
3322	6	90660909	90662560	ENSMUSG00000051056	ENSMUSG00000051056	ENSMUSG00000051056	4	32929478	32930995	66	67		
3323	6	90613030	90637843	ENSMUSG00000028282	ENSMUSG00000028282	ENSMUSG00000028282	4	32944126	32981873	66	67		
3324	6	90408939	90586163	ENSMUSG00000058006	ENSMUSG00000058006	ENSMUSG00000058006	4	32985864	33102746	66	67		
3325	6	90199651	90397935	ENSMUSG00000040183	ENSMUSG00000040183	ENSMUSG00000040183	4	33132648	33279439	66	67		
3326	6	90131074	90178708	ENSMUSG00000028278	ENSMUSG00000028278	ENSMUSG00000028278	4	33312076	33347408	66	67		
3327	6	90093066	90119338	ENSMUSG00000028277	ENSMUSG00000028277	ENSMUSG00000028277	4	33360038	33380977	66	67		
3328	6	90023958	90081676	ENSMUSG00000023267	ENSMUSG00000023267	ENSMUSG00000023267	4	33391612	33424478	66	67		
3329	6	89943939	89983871	ENSMUSG00000028280	ENSMUSG00000028280	ENSMUSG00000028280	4	33461169	33492201	66	67		
3330	6	89912488	89932003	ENSMUSG00000054659	ENSMUSG00000054659	ENSMUSG00000054659	4	33502951	33517987	66	67		
3331	6	89864858	89884453	ENSMUSG00000054679	ENSMUSG00000054679	ENSMUSG00000054679	4	33537604	33561953	66	67		
3332	6	89847216	89851598	ENSMUSG00000040128	ENSMUSG00000040128	ENSMUSG00000040128	4	33573848	33577123	66	67		
3333	6	89376704	89730034	ENSMUSG00000028274	ENSMUSG00000028274	ENSMUSG00000028274	4	33638989	33829251	66	67		
3334	6	88906302	88932385	ENSMUSG00000044288	ENSMUSG00000044288	ENSMUSG00000044288	4	34253206	34277434	66	67		
3335	6	88814226	88833269	ENSMUSG00000028264	ENSMUSG00000028264	ENSMUSG00000028264	4	34356927	34378680	66	67		
3336	6	88441509	88468646	ENSMUSG00000028291	ENSMUSG00000028291	ENSMUSG00000028291	4	34739502	34755795	66	67		
3337	6	88356562	88433883	ENSMUSG00000040044	ENSMUSG00000040044	ENSMUSG00000040044	4	34758190	34803831	66	67		
3338	6	88280815	88356440	ENSMUSG00000028292	ENSMUSG00000028292	ENSMUSG00000028292	4	34803865	34849054	66	67		
3339	6	88111537	88131830	ENSMUSG00000071015	ENSMUSG00000071015	ENSMUSG00000071015	4	34932675	34945146	66	67		
3340	6	88089023	88107891	ENSMUSG00000028295	ENSMUSG00000028295	ENSMUSG00000028295	4	34957288	34967246	66	67		
3341	6	87851935	87861569	ENSMUSG00000028298	ENSMUSG00000028298	ENSMUSG00000028298	4	35082666	35096261	66	67		
3342	9	27315207	27519850	ENSMUSG00000073910	ENSMUSG00000073910	ENSMUSG00000073910	4	35137961	35346371	67	68		
3343	9	27514302	27516491	ENSMUSG00000042993	ENSMUSG00000042993	ENSMUSG00000042993	4	35340942	35342908	67	68		
3344	9	27536544	27563476	ENSMUSG00000028300	ENSMUSG00000028300	ENSMUSG00000028300	4	35381769	35415010	67	68		
3345	9	27938076	28660283	ENSMUSG00000045083	ENSMUSG00000045083	ENSMUSG00000045083	4	35896291	36034083	67	68		
3346	9	32374618	32440834	ENSMUSG00000028405	ENSMUSG00000028405	ENSMUSG00000028405	4	40332103	40387005	67	68		
3347	9	32445300	32516322	ENSMUSG00000040296	ENSMUSG00000040296	ENSMUSG00000040296	4	40393530	40428431	67	68		
3348	9	32530545	32542601	ENSMUSG00000036822	ENSMUSG00000036822	ENSMUSG00000036822	4	40448852	40457172	67	68		
3349	9	32542997	32563160	ENSMUSG00000071014	ENSMUSG00000071014	ENSMUSG00000071014	4	40459334	40468039	67	68		
3350	9	32773996	32779074	ENSMUSG00000046593	ENSMUSG00000046593	ENSMUSG00000046593	4	40660851	40664324	67	68		
3351	9	32962747	32991602	ENSMUSG00000028411	ENSMUSG00000028411	ENSMUSG00000028411	4	40874913	40891858	67	68		
3352	9	33031762	33066657	ENSMUSG00000028409	ENSMUSG00000028409	ENSMUSG00000028409	4	40925230	40946515	67	68		
3353	9	33100635	33157354	ENSMUSG00000028413	ENSMUSG00000028413	ENSMUSG00000028413	4	40993273	41042669	67	68		
3354	9	33208363	33238550	ENSMUSG00000028415	ENSMUSG00000028415	ENSMUSG00000028415	4	41108766	41120061	67	68		
3355	9	33244163	33254672	ENSMUSG00000028416	ENSMUSG00000028416	ENSMUSG00000028416	4	41125070	41136927	67	68		
3356	9	33254167	33271482	ENSMUSG00000028419	ENSMUSG00000028419	ENSMUSG00000028419	4	41137224	41153973	67	68		
3357	9	33280509	33361155	ENSMUSG00000028423	ENSMUSG00000028423	ENSMUSG00000028423	4	41159652	41213846	67	68		
3358	9	33431152	33437609	ENSMUSG00000028435	ENSMUSG00000028435	ENSMUSG00000028435	4	41281395	41286844	67	68		
3359	9	33451354	33463941	ENSMUSG00000028430	ENSMUSG00000028430	ENSMUSG00000028430	4	41303099	41312938	67	68		
3360	9	33807182	33910401	ENSMUSG00000036241	ENSMUSG00000036241	ENSMUSG00000036241	4	41324692	41382041	67	68		
3361	9	33911691	34038947	ENSMUSG00000028433	ENSMUSG00000028433	ENSMUSG00000028433	4	41382986	41463806	67	68		
3362	9	34076386	34116736	ENSMUSG00000028436	ENSMUSG00000028436	ENSMUSG00000028436	4	41479971	41503487	67	68		
3363	9	34169003	34242520	ENSMUSG00000028437	ENSMUSG00000028437	ENSMUSG00000028437	4	41537719	41578436	67	68		
3364	9	34242379	34301369	ENSMUSG00000028438	ENSMUSG00000028438	ENSMUSG00000028438	4	41579416	41653511	67	68		
3365	9	34319504	34333709	ENSMUSG00000028443	ENSMUSG00000028443	ENSMUSG00000028443	4	41653819	41669597	67	68		
3366	9	34356668	34366851	ENSMUSG00000046312	ENSMUSG00000046312	ENSMUSG00000046312	4	41684276	41691728	67	68		
3367	9	34369020	34387849	ENSMUSG00000028441	ENSMUSG00000028441	ENSMUSG00000028441	4	41694239	41696255	67	68		
3368	9	34388182	34448550	ENSMUSG00000028439	ENSMUSG00000028439	ENSMUSG00000028439	4	41708911	41713088	67	68		
3369	9	34448811	34510981	ENSMUSG00000061322	ENSMUSG00000061322	ENSMUSG00000061322	4	41758446	41826829	67	68		
3370	9	34541430	34579735	ENSMUSG00000028444	ENSMUSG00000028444	ENSMUSG00000028444	4	41846167	41885710	67	68		
3371	9	34600483	34602101	ENSMUSG00000036114	ENSMUSG00000036114	ENSMUSG00000036114	4	41900703	41902182	67	68		
3372	9	34603549	34610515	ENSMUSG00000028447	ENSMUSG00000028447	ENSMUSG00000028447	4	41903468	41911828	67	68		
3373	9	34611455	34618011	ENSMUSG00000066224	ENSMUSG00000066224	ENSMUSG00000066224	4	41912903	41918863	67	68		
3374	9	34624717	34627806	ENSMUSG00000036078	ENSMUSG00000036078	ENSMUSG00000036078	4	41927007	41929824	67	68		
3375	9	34948192	34972541	ENSMUSG00000036062	ENSMUSG00000036062	ENSMUSG00000036062	4	42954720	42965248	67	68		
3376	9	34979742	34987421	ENSMUSG00000036052	ENSMUSG00000036052	ENSMUSG00000036052	4	42971018	42978694	67	68		
3377	9	35032226	35035987	ENSMUSG00000028451	ENSMUSG00000028451	ENSMUSG00000028451	4	42991046	42995320	67	68		
3378	9	35046061	35063246	ENSMUSG00000028452	ENSMUSG00000028452	ENSMUSG00000028452	4	43001064	43021534	67	68		
3379	9	35063835	35070013	ENSMUSG00000028453	ENSMUSG00000028453	ENSMUSG00000028453	4	43023444	43031588	67	68		
3380	9	35078688	35086545	ENSMUSG00000028454	ENSMUSG00000028454	ENSMUSG00000028454	4	43038738	43046874	67	68		
3381	9	35089593	35093113	ENSMUSG00000028455	ENSMUSG00000028455	ENSMUSG00000028455	4	43048790	43052484	67	68		
3382	9	35094109	35098271	ENSMUSG00000036002	ENSMUSG00000036002	ENSMUSG00000036002	4	43053610	43067303	67	68		
3383	9	35152006	35395331	ENSMUSG00000028456	ENSMUSG00000028456	ENSMUSG00000028456	4	43108981	43285970	67	68		
3384	9	35480124	35551889	ENSMUSG00000035969	ENSMUSG00000035969	ENSMUSG00000035969	4	43403082	43448186	67	68		
3385	9	35595281	35600037	ENSMUSG00000028458	ENSMUSG00000028458	ENSMUSG00000028458	4	43463039	43469161	67	68		
3386	9	35599981	35608753	ENSMUSG00000028459	ENSMUSG00000028459	ENSMUSG00000028459	4	43468835	43475663	67	68		
3387	9	35639300	35640947	ENSMUSG00000028460	ENSMUSG00000028460	ENSMUSG00000028460	4	43503184	43504822	67	68		
3388	9	35648311	35651508	ENSMUSG00000028461	ENSMUSG00000028461	ENSMUSG00000028461	4	43514477	43517020	67	68		
3389	9	35652406	35655284	ENSMUSG00000051517	ENSMUSG00000051517	ENSMUSG00000051517	4	43517242	43520730	67	68		
3390	9	35663853	35671156	ENSMUSG00000028463	ENSMUSG00000028463	ENSMUSG00000028463	4	43528107	43534829	67	68		
3391	9	35687338	35722367	ENSMUSG00000028465	ENSMUSG00000028465	ENSMUSG00000028465	4	43552618	43583355	67	68		
3392	9	35722317	35727004	ENSMUSG00000028466	ENSMUSG00000028466	ENSMUSG00000028466	4	43583765	43588021	67	68		
3393	9	35726863	35739225	ENSMUSG00000028467	ENSMUSG00000028467	ENSMUSG00000028467	4	43588029	43599950	67	68		
3394	9	35739315	35748572	ENSMUSG00000028468	ENSMUSG00000028468	ENSMUSG00000028468	4	43600029	43608587	67	68		
3395	9	35782151	35799729	ENSMUSG00000028469	ENSMUSG00000028469	ENSMUSG00000028469	4	43653035	43672344	67	68		
3396	9	35797782	35802259	ENSMUSG00000066196	ENSMUSG00000066196	ENSMUSG00000066196	4	43672560	43674652	67	68		
3397	9	35802957	35805018	ENSMUSG00000028470	ENSMUSG00000028470	ENSMUSG00000028470	4	43675329	43677550	67	68		
3398	9	35806407	35816158	ENSMUSG00000043633	ENSMUSG00000043633	ENSMUSG00000043633	4	43680755	43687709	67	68		
3399	9	35859260	35860598	ENSMUSG00000046450	ENSMUSG00000046450	ENSMUSG00000046450	4	43726728	43727666	67	68		
3400	9	35896189	35897138	ENSMUSG00000071001	ENSMUSG00000071001	ENSMUSG00000071001	4	43748298	43749739	67	68		
3401	9	36026430	36114448	ENSMUSG00000028476	ENSMUSG00000028476	ENSMUSG00000028476	4	43896630	43965845	67	68		
3402	9	36126695	36153887	ENSMUSG00000028480	ENSMUSG00000028480	ENSMUSG00000028480	4	43978802	44000218	67	68		
3403	9	36159391	36161331	ENSMUSG00000070999	ENSMUSG00000070999	ENSMUSG00000070999	4	44004604	44006633	67	68		
3404	9	36180934	36202056	ENSMUSG00000028478	ENSMUSG00000028478	ENSMUSG00000028478	4	44033892	44053946	67	68		
3405	9	36204430	36248450	ENSMUSG00000028479	ENSMUSG00000028479	ENSMUSG00000028479	4	44057926	44105266	67	68		
3406	9	36326393	36390296	ENSMUSG00000035696	ENSMUSG00000035696	ENSMUSG00000035696	4	44147310	44188708	67	68		
3407	9	36562873	36667678	ENSMUSG00000035683	ENSMUSG00000035683	ENSMUSG00000035683	4	44321976	44385775	67	68		
3408	9	36823272	37024103	ENSMUSG00000014030	ENSMUSG00000014030	ENSMUSG00000014030	4	44552606	44731762	67	68		
3409	9	37110536	37348146	ENSMUSG00000054444	ENSMUSG00000054444	ENSMUSG00000054444	4	44945253	44953302	67	68		
3410	9	37412663	37422011	ENSMUSG00000035637	ENSMUSG00000035637	ENSMUSG00000035637	4	45002504	45011797	67	68		
3411	9	37428111	37455396	ENSMUSG00000049657	ENSMUSG00000049657	ENSMUSG00000049657	4	45012338	45033370	67	68		
3412	9	37476150	37493694	ENSMUSG00000028318	ENSMUSG00000028318	ENSMUSG00000028318	4	45039725	45054792	67	68		
3413	9	37500899	37531765	ENSMUSG00000048232	ENSMUSG00000048232	ENSMUSG00000048232	4	45055354	45105694	67	68		
3414	9	37640997	37736901	ENSMUSG00000035615	ENSMUSG00000035615	ENSMUSG00000035615	4	45265731	45307036	67	68		
3415	9	37770308	37775065	ENSMUSG00000028322	ENSMUSG00000028322	ENSMUSG00000028322	4	45337713	45341698	67	68		
3416	9	37790864	37853322	ENSMUSG00000035572	ENSMUSG00000035572	ENSMUSG00000035572	4	45363268	45395374	67	68		
3417	9	37909614	38058901	ENSMUSG00000044813	ENSMUSG00000044813	ENSMUSG00000044813	4	45444379	45551430	67	68		
3418	9	38382661	38388658	ENSMUSG00000035561	ENSMUSG00000035561	ENSMUSG00000035561	4	45820149	45825699	67	68		
3419	9	38398991	38414444	ENSMUSG00000035551	ENSMUSG00000035551	ENSMUSG00000035551	4	45830568	45847915	67	68	here	Sub block(s) comes here, so ST did not merge
3420	9	99109790	99179033	ENSMUSG00000035539	ENSMUSG00000035539	ENSMUSG00000035539	4	45921301	45930667	68	68	sub blocks	2 -> 1
3421	9	99214129	99298222	ENSMUSG00000035517	ENSMUSG00000035517	ENSMUSG00000035517	4	46008560	46055861	68	68		
3422	9	99303742	99403357	ENSMUSG00000028328	ENSMUSG00000028328	ENSMUSG00000028328	4	46082066	46135904	68	68		
3423	9	99402186	99435600	ENSMUSG00000035495	ENSMUSG00000035495	ENSMUSG00000035495	4	46135846	46159510	68	68		
3424	9	99435729	99475851	ENSMUSG00000028330	ENSMUSG00000028330	ENSMUSG00000028330	4	46159953	46193500	68	68		
3425	9	99477012	99499460	ENSMUSG00000028329	ENSMUSG00000028329	ENSMUSG00000028329	4	46176448	46217388	68	68		
3426	9	99655511	99657116	ENSMUSG00000070990	ENSMUSG00000070990	ENSMUSG00000070990	4	46365294	46366409	68	68		
3427	9	99706592	99724673	ENSMUSG00000028331	ENSMUSG00000028331	ENSMUSG00000028331	4	46397605	46410493	68	68		
3428	9	99728894	99746959	ENSMUSG00000028332	ENSMUSG00000028332	ENSMUSG00000028332	4	46415593	46434578	68	68		
3429	9	99785464	99818046	ENSMUSG00000028333	ENSMUSG00000028333	ENSMUSG00000028333	4	46472227	46493509	68	68		
3430	9	99858834	99885178	ENSMUSG00000028334	ENSMUSG00000028334	ENSMUSG00000028334	4	46510419	46524732	68	68		
3431	9	99882940	99921305	ENSMUSG00000039853	ENSMUSG00000039853	ENSMUSG00000039853	4	46526179	46557220	68	68		
3432	9	99923078	99994743	ENSMUSG00000028337	ENSMUSG00000028337	ENSMUSG00000028337	4	46560236	46584195	68	68		
3433	9	100001132	100057736	ENSMUSG00000039813	ENSMUSG00000039813	ENSMUSG00000039813	4	46625490	46671299	68	68		
3434	9	100534112	100598615	ENSMUSG00000066191	ENSMUSG00000066191	ENSMUSG00000066191	4	47036769	47078406	68	68		
3435	9	100609802	100652184	ENSMUSG00000039774	ENSMUSG00000039774	ENSMUSG00000039774	4	47113053	47144142	68	68		
3436	9	100746011	100872879	ENSMUSG00000028339	ENSMUSG00000028339	ENSMUSG00000028339	4	47229315	47334265	68	68		
3437	9	100907233	100956406	ENSMUSG00000007613	ENSMUSG00000007613	ENSMUSG00000007613	4	47374405	47436024	68	68		
3438	9	101018529	101024057	ENSMUSG00000039740	ENSMUSG00000039740	ENSMUSG00000039740	4	47490934	47495425	68	68		
3439	9	101024380	101032715	ENSMUSG00000053317	ENSMUSG00000053317	ENSMUSG00000053317	4	47495761	47504333	68	68		
3440	9	101628830	101668994	ENSMUSG00000028341	ENSMUSG00000028341	ENSMUSG00000028341	4	48066409	48107545	68	68		
3441	9	101708787	101772442	ENSMUSG00000061455	ENSMUSG00000061455	ENSMUSG00000061455	4	48146031	48204233	68	68		
3442	9	101781282	101901143	ENSMUSG00000028343	ENSMUSG00000028343	ENSMUSG00000028343	4	48211812	48300631	68	68		
3443	9	101901332	102103247	ENSMUSG00000028344	ENSMUSG00000028344	ENSMUSG00000028344	4	48300922	48453054	68	68		
3444	9	102104187	102155042	ENSMUSG00000028345	ENSMUSG00000028345	ENSMUSG00000028345	4	48451964	48491163	68	68		
3445	9	102275216	102379739	ENSMUSG00000028347	ENSMUSG00000028347	ENSMUSG00000028347	4	48606293	48684231	68	68		
3446	9	102380157	102389992	ENSMUSG00000028348	ENSMUSG00000028348	ENSMUSG00000028348	4	48684614	48694592	68	68		
3447	9	102830812	103127238	ENSMUSG00000063446	ENSMUSG00000063446	ENSMUSG00000063446	4	49080562	49361361	68	68		
3448	9	103162890	103173578	ENSMUSG00000039653	ENSMUSG00000039653	ENSMUSG00000039653	4	49510522	49527606	68	68		
3449	9	103189736	103200717	ENSMUSG00000044018	ENSMUSG00000044018	ENSMUSG00000044018	4	49533697	49542183	68	68		
3450	9	103200984	103212763	ENSMUSG00000039634	ENSMUSG00000039634	ENSMUSG00000039634	4	49542663	49551825	68	68		
3451	9	103222681	103237926	ENSMUSG00000028307	ENSMUSG00000028307	ENSMUSG00000028307	4	49557095	49570583	68	68		
3452	9	103275274	103335551	ENSMUSG00000039611	ENSMUSG00000039611	ENSMUSG00000039611	4	49605677	49618903	68	68		
3453	9	103335954	103365447	ENSMUSG00000028309	ENSMUSG00000028309	ENSMUSG00000028309	4	49653221	49677986	68	68		
3454	9	103371456	103540683	ENSMUSG00000039579	ENSMUSG00000039579	ENSMUSG00000039579	4	49683873	49865870	68	68		
3455	9	103393718	103397104	ENSMUSG00000028310	ENSMUSG00000028310	ENSMUSG00000028310	4	49699847	49703083	68	68		
3456	9	104797414	104807781	ENSMUSG00000039555	ENSMUSG00000039555	ENSMUSG00000039555	4	51237778	51250725	68	68		
3457	9	105896428	105943519	ENSMUSG00000028312	ENSMUSG00000028312	ENSMUSG00000028312	4	52460349	52509360	68	68		
3458	9	106306365	106307324	ENSMUSG00000070984	ENSMUSG00000070984	ENSMUSG00000070984	4	52846499	52847458	68	68		
3459	9	106337872	106338915	ENSMUSG00000049648	ENSMUSG00000049648	ENSMUSG00000049648	4	52876658	52877611	68	68		
3460	9	106371270	106372232	ENSMUSG00000051593	ENSMUSG00000051593	ENSMUSG00000051593	4	52931933	52932892	68	68		
3461	9	106496524	106497564	ENSMUSG00000070983	ENSMUSG00000070983	ENSMUSG00000070983	4	52991723	52992667	68	68		
3462	9	106583104	106730339	ENSMUSG00000015243	ENSMUSG00000015243	ENSMUSG00000015243	4	53051888	53180992	68	68		
3463	9	107046724	107199448	ENSMUSG00000028412	ENSMUSG00000028412	ENSMUSG00000028412	4	53461513	53643578	68	68		
3464	9	107360232	107443220	ENSMUSG00000028414	ENSMUSG00000028414	ENSMUSG00000028414	4	53735282	53784371	68	68		
3465	9	107464599	107465214	ENSMUSG00000028417	ENSMUSG00000028417	ENSMUSG00000028417	4	53800805	53807985	68	68		
3466	9	107496646	107578714	ENSMUSG00000028420	ENSMUSG00000028420	ENSMUSG00000028420	4	53847162	53881691	68	68		
3467	9	108665199	108813584	ENSMUSG00000060206	ENSMUSG00000060206	ENSMUSG00000060206	4	55028663	55101970	68	68		
3468	9	109085365	109134291	ENSMUSG00000028426	ENSMUSG00000028426	ENSMUSG00000028426	4	55371157	55412312	68	68		
3469	9	109286956	109291576	ENSMUSG00000003032	ENSMUSG00000003032	ENSMUSG00000003032	4	55548365	55553566	68	68		
3470	9	110656692	110659068	ENSMUSG00000056979	ENSMUSG00000056979	ENSMUSG00000056979	4	56834154	56835410	68	68		
3471	9	110664367	110665855	ENSMUSG00000070979	ENSMUSG00000070979	ENSMUSG00000070979	4	56837466	56838979	68	68		
3472	9	110669618	110736217	ENSMUSG00000028431	ENSMUSG00000028431	ENSMUSG00000028431	4	56844828	56896351	68	68		
3473	9	110744672	110815630	ENSMUSG00000038816	ENSMUSG00000038816	ENSMUSG00000038816	4	56904989	56959242	68	68		
3474	9	110822468	110922046	ENSMUSG00000055296	ENSMUSG00000055296	ENSMUSG00000055296	4	56997939	57041465	68	68		
3475	9	110938988	110969392	ENSMUSG00000045589	ENSMUSG00000045589	ENSMUSG00000045589	4	57056920	57084148	68	68		
3476	9	110974076	111122508	ENSMUSG00000028434	ENSMUSG00000028434	ENSMUSG00000028434	4	57089765	57237157	68	68		
3477	9	111177800	111300407	ENSMUSG00000038764	ENSMUSG00000038764	ENSMUSG00000038764	4	57334879	57395891	68	68		
3478	9	111582410	111974613	ENSMUSG00000038729	ENSMUSG00000038729	ENSMUSG00000038729	4	57948214	57991038	68	68		
3479	9	112001662	112009735	ENSMUSG00000052117	ENSMUSG00000052117	ENSMUSG00000052117	4	58002438	58010318	68	68		
3480	9	112105622	112139897	ENSMUSG00000038709	ENSMUSG00000038709	ENSMUSG00000038709	4	58078083	58103161	68	68		
3481	9	112168391	112381807	ENSMUSG00000028369	ENSMUSG00000028369	ENSMUSG00000028369	4	58136496	58300650	68	68		
3482	9	112470872	112603099	ENSMUSG00000057280	ENSMUSG00000057280	ENSMUSG00000057280	4	58380016	58468357	68	68		
3483	9	112675364	112840144	ENSMUSG00000038668	ENSMUSG00000038668	ENSMUSG00000038668	4	58529314	58647308	68	68		
3484	9	113129584	113130621	ENSMUSG00000043385	ENSMUSG00000043385	ENSMUSG00000043385	4	58878833	58879774	68	68		
3485	9	113162793	113286458	ENSMUSG00000050812	ENSMUSG00000050812	ENSMUSG00000050812	4	58892965	59006803	68	68		
3486	9	113365068	113401508	ENSMUSG00000028378	ENSMUSG00000028378	ENSMUSG00000028378	4	59059644	59081132	68	68		
3487	9	113433470	113456445	ENSMUSG00000070972	ENSMUSG00000070972	ENSMUSG00000070972	4	59089116	59116737	68	68		
3488	9	113488274	113597109	ENSMUSG00000038598	ENSMUSG00000038598	ENSMUSG00000038598	4	59175184	59205167	68	68		
3489	9	113698867	113737470	ENSMUSG00000028381	ENSMUSG00000028381	ENSMUSG00000028381	4	59283604	59316887	68	68		
3490	9	113843181	113977368	ENSMUSG00000038578	ENSMUSG00000038578	ENSMUSG00000038578	4	59409800	59506293	68	68		
3491	9	114020917	114135726	ENSMUSG00000028382	ENSMUSG00000028382	ENSMUSG00000028382	4	59569120	59643350	68	68		
3492	9	114196641	114274505	ENSMUSG00000028383	ENSMUSG00000028383	ENSMUSG00000028383	4	59675649	59712737	68	68		
3493	9	114289116	114421315	ENSMUSG00000045071	ENSMUSG00000045071	ENSMUSG00000045071	4	59720297	59815457	68	68		
3494	9	114488618	114520211	ENSMUSG00000038544	ENSMUSG00000038544	ENSMUSG00000038544	4	59863696	59877909	68	68		
3495	9	114552939	114677088	ENSMUSG00000028385	ENSMUSG00000028385	ENSMUSG00000028385	4	59899704	59998794	68	68		
3496	9	114681021	114693014	ENSMUSG00000028386	ENSMUSG00000028386	ENSMUSG00000028386	4	59999180	60009110	68	68		
3497	9	114840481	114858817	ENSMUSG00000028389	ENSMUSG00000028389	ENSMUSG00000028389	4	61675904	61694722	68	68		
3498	9	114956660	114966223	ENSMUSG00000066152	ENSMUSG00000066152	ENSMUSG00000066152	4	61772806	61784772	68	68		
3499	9	114967621	115023419	ENSMUSG00000066151	ENSMUSG00000066151	ENSMUSG00000066151	4	61786704	61846823	68	68		
3500	9	115023663	115066593	ENSMUSG00000066150	ENSMUSG00000066150	ENSMUSG00000066150	4	61847110	61878123	68	68		
3501	9	115077444	115095287	ENSMUSG00000066148	ENSMUSG00000066148	ENSMUSG00000066148	4	61895144	61913351	68	68		
3502	9	115099707	115100285	ENSMUSG00000063851	ENSMUSG00000063851	ENSMUSG00000063851	4	61914348	61915065	68	68		
3503	9	115117772	115142383	ENSMUSG00000028391	ENSMUSG00000028391	ENSMUSG00000028391	4	61940095	61957238	68	68		
3504	9	115151642	115173334	ENSMUSG00000028392	ENSMUSG00000028392	ENSMUSG00000028392	4	61966428	61983659	68	68		
3505	9	115175520	115179100	ENSMUSG00000038422	ENSMUSG00000038422	ENSMUSG00000038422	4	61985373	61988575	68	68		
3506	9	115188420	115203412	ENSMUSG00000028393	ENSMUSG00000028393	ENSMUSG00000028393	4	61995531	62006424	68	68		
3507	9	115209336	115212773	ENSMUSG00000028394	ENSMUSG00000028394	ENSMUSG00000028394	4	62009017	62011429	68	68		

3508	9	115212787	115231785	ENSMUSG00000058046	ENSMUSG00000058046	ENSMUSG00000058046	4	62011730	62034354	68	68
3509	9	115678383	115852322	ENSMUSG00000028358	ENSMUSG00000028358	ENSMUSG00000028358	4	62452035	62620203	68	68
3510	9	115862231	115880536	ENSMUSG00000028356	ENSMUSG00000028356	ENSMUSG00000028356	4	62629637	62640516	68	68
3511	9	115893741	115901377	ENSMUSG00000028357	ENSMUSG00000028357	ENSMUSG00000028357	4	62651998	62658492	68	68
3512	9	115958052	116112796	ENSMUSG00000045672	ENSMUSG00000045672	ENSMUSG00000045672	4	62702187	62819669	68	68
3513	9	116138266	116196506	ENSMUSG00000039158	ENSMUSG00000039158	ENSMUSG00000039158	4	62847107	62889723	68	68
3514	9	116204181	116307526	ENSMUSG00000039137	ENSMUSG00000039137	ENSMUSG00000039137	4	62901271	62982312	68	68
3515	9	116413307	116448523	ENSMUSG00000045917	ENSMUSG00000045917	ENSMUSG00000045917	4	63045147	63071416	68	68
3516	9	116591421	116608227	ENSMUSG00000050395	ENSMUSG00000050395	ENSMUSG00000050395	4	63213443	63231448	68	68
3517	9	116704945	116732591	ENSMUSG00000028362	ENSMUSG00000028362	ENSMUSG00000028362	4	63319185	63347645	68	68
3518	9	116822634	116920260	ENSMUSG00000028364	ENSMUSG00000028364	ENSMUSG00000028364	4	63446239	63533177	68	68
3519	9	117955892	118204420	ENSMUSG00000028370	ENSMUSG00000028370	ENSMUSG00000028370	4	64610535	64843870	68	68
3520	9	118227690	119217138	ENSMUSG00000028373	ENSMUSG00000028373	ENSMUSG00000028373	4	64867164	65890844	68	68
3521	9	118489430	118503400	ENSMUSG00000051675	ENSMUSG00000051675	ENSMUSG00000051675	4	65091379	65102599	68	68
3522	9	119506471	119518970	ENSMUSG00000039005	ENSMUSG00000039005	ENSMUSG00000039005	4	66313972	66328954	68	68
3523	9	120968728	121171566	ENSMUSG00000028351	ENSMUSG00000028351	ENSMUSG00000028351	4	68247733	68440758	68	68
3524	9	122190968	122382258	ENSMUSG00000039298	ENSMUSG00000039298	ENSMUSG00000039298	4	69709385	69896728	68	68
3525	9	122402912	122516569	ENSMUSG00000039270	ENSMUSG00000039270	ENSMUSG00000039270	4	69915214	70021280	68	68
3526	9	84784320	84867863	ENSMUSG00000043003	ENSMUSG00000043003	ENSMUSG00000043003	4	73185810	73261833	69	69
3527	9	85049357	85343173	ENSMUSG00000049122	ENSMUSG00000049122	ENSMUSG00000049122	4	73484892	73661159	69	69
3528	9	6747656	7165648	ENSMUSG00000028397	ENSMUSG00000028397	ENSMUSG00000028397	4	73713728	73877092	70	70
3529	9	7786490	7789799	ENSMUSG00000028398	ENSMUSG00000028398	ENSMUSG00000028398	4	74748582	74749521	70	70
3530	9	8307277	8723949	ENSMUSG00000028399	ENSMUSG00000028399	ENSMUSG00000028399	4	75416028	75796579	70	70
3531	9	12683997	12699182	ENSMUSG00000005994	ENSMUSG00000005994	ENSMUSG00000005994	4	80305538	80322950	70	70
3532	9	12765012	12813058	ENSMUSG00000048706	ENSMUSG00000048706	ENSMUSG00000048706	4	80381917	80425532	70	70
3533	9	13095703	13240328	ENSMUSG00000028402	ENSMUSG00000028402	ENSMUSG00000028402	4	80749736	80914038	70	70
3534	9	14077599	14304518	ENSMUSG00000008575	ENSMUSG00000008575	ENSMUSG00000008575	4	81767308	81976036	70	70
3535	9	14605530	14683469	ENSMUSG00000028403	ENSMUSG00000028403	ENSMUSG00000028403	4	82273784	82330738	70	70
3536	9	14709722	14712715	ENSMUSG00000038192	ENSMUSG00000038192	ENSMUSG00000038192	4	82352982	82356382	70	70
3537	9	14727394	14858975	ENSMUSG00000059049	ENSMUSG00000059049	ENSMUSG00000059049	4	82371630	82492333	70	70
3538	9	15161561	15222408	ENSMUSG00000038172	ENSMUSG00000038172	ENSMUSG00000038172	4	82691531	82795486	70	70
3539	9	15412702	15454132	ENSMUSG00000028483	ENSMUSG00000028483	ENSMUSG00000028483	4	82888980	82938828	70	70
3540	9	15543084	15964037	ENSMUSG00000052407	ENSMUSG00000052407	ENSMUSG00000052407	4	82996827	83223139	70	70
3541	9	16406404	16860704	ENSMUSG00000028487	ENSMUSG00000028487	ENSMUSG00000028487	4	83746326	84027135	70	70
3542	9	17124980	17493921	ENSMUSG00000038070	ENSMUSG00000038070	ENSMUSG00000038070	4	84355558	84603149	70	70
3543	9	17569121	17787127	ENSMUSG00000028488	ENSMUSG00000028488	ENSMUSG00000028488	4	84676687	84860615	70	70
3544	9	18767615	18898543	ENSMUSG00000066113	ENSMUSG00000066113	ENSMUSG00000066113	4	85814015	85898984	70	70
3545	9	19105760	19117573	ENSMUSG00000028494	ENSMUSG00000028494	ENSMUSG00000028494	4	86127797	86141229	70	70
3546	9	19286072	19362856	ENSMUSG00000038024	ENSMUSG00000038024	ENSMUSG00000038024	4	86245486	86296798	70	70
3547	9	19505978	19776926	ENSMUSG00000037996	ENSMUSG00000037996	ENSMUSG00000037996	4	86461003	86701654	70	70
3548	9	20331663	20612542	ENSMUSG00000028496	ENSMUSG00000028496	ENSMUSG00000028496	4	87245249	87502938	70	70
3549	9	20648678	20985954	ENSMUSG00000038368	ENSMUSG00000038368	ENSMUSG00000038368	4	87700554	87881921	70	70
3550	9	20993620	21021635	ENSMUSG00000028497	ENSMUSG00000028497	ENSMUSG00000028497	4	87867375	87910146	70	70
3551	9	21067104	21067962	ENSMUSG00000048806	ENSMUSG00000048806	ENSMUSG00000048806	4	87993457	87994005	70	70
3552	9	21471067	21471693	ENSMUSG00000045364	ENSMUSG00000045364	ENSMUSG00000045364	4	88350832	88351410	70	70
3553	9	21792635	21857077	ENSMUSG00000062937	ENSMUSG00000062937	ENSMUSG00000062937	4	88608655	88652321	70	70
3554	9	21957751	21984490	ENSMUSG00000044303	ENSMUSG00000044303	ENSMUSG00000044303	4	88745772	88765770	70	70
3555	9	21992902	21999280	ENSMUSG00000073802	ENSMUSG00000073802	ENSMUSG00000073802	4	88777525	88782177	70	70
3556	9	22436840	22442472	ENSMUSG00000043753	ENSMUSG00000043753	ENSMUSG00000043753	4	89180216	89186784	70	70
3557	9	23681984	23755078	ENSMUSG00000008489	ENSMUSG00000008489	ENSMUSG00000008489	4	90742781	90892002	70	70
3558	9	24532958	24535909	ENSMUSG00000028533	ENSMUSG00000028533	ENSMUSG00000028533	4	91636356	91639252	70	70
3559	9	25667681	25668319	ENSMUSG00000054000	ENSMUSG00000054000	ENSMUSG00000054000	4	92826821	92827438	70	70
3560	9	26830683	26882802	ENSMUSG00000028578	ENSMUSG00000028578	ENSMUSG00000028578	4	93992090	94048802	70	70
3561	9	26895339	26937461	ENSMUSG00000028577	ENSMUSG00000028577	ENSMUSG00000028577	4	94061188	94095269	70	70
3562	9	26946410	27052928	ENSMUSG00000028576	ENSMUSG00000028576	ENSMUSG00000028576	4	94106562	94185087	70	70
3563	9	26983134	26995670	ENSMUSG00000049799	ENSMUSG00000049799	ENSMUSG00000049799	4	94128678	94142162	70	70
3564	9	27099236	27220165	ENSMUSG00000006386	ENSMUSG00000006386	ENSMUSG00000006386	4	94231318	94366957	70	70
3565	9	27274659	27287137	ENSMUSG00000028575	ENSMUSG00000028575	ENSMUSG00000028575	4	94399467	94420830	70	70
3566	1	58892999	58938352	ENSMUSG00000062627	ENSMUSG00000062627	ENSMUSG00000062627	4	94434059	94471118	71	71
3567	1	59019048	59022587	ENSMUSG00000052684	ENSMUSG00000052684	ENSMUSG00000052684	4	94542255	94544189	71	71
3568	1	59535241	60000984	ENSMUSG00000028573	ENSMUSG00000028573	ENSMUSG00000028573	4	95071479	95313579	71	71
3569	1	60053121	60114638	ENSMUSG00000028572	ENSMUSG00000028572	ENSMUSG00000028572	4	95459397	95516295	71	71
3570	1	61103519	61693785	ENSMUSG00000028565	ENSMUSG00000028565	ENSMUSG00000028565	4	97073995	97573834	71	71
3571	1	61919305	61963683	ENSMUSG00000028563	ENSMUSG00000028563	ENSMUSG00000028563	4	97847388	97875254	71	71
3572	1	61980737	62399195	ENSMUSG00000061859	ENSMUSG00000061859	ENSMUSG00000061859	4	97887844	98211621	71	71
3573	1	62475239	62557673	ENSMUSG00000035407	ENSMUSG00000035407	ENSMUSG00000035407	4	98246916	98309555	71	71
3574	1	62693157	62926541	ENSMUSG00000028556	ENSMUSG00000028556	ENSMUSG00000028556	4	98429276	98581641	71	71
3575	1	62835746	62844418	ENSMUSG00000028553	ENSMUSG00000028553	ENSMUSG00000028553	4	98522985	98530082	71	71
3576	1	63022394	63103411	ENSMUSG00000028550	ENSMUSG00000028550	ENSMUSG00000028550	4	98685952	98751809	71	71
3577	1	63561300	63563385	ENSMUSG00000067261	ENSMUSG00000067261	ENSMUSG00000067261	4	99148317	99150640	71	71
3578	1	63609029	63675436	ENSMUSG00000073792	ENSMUSG00000073792	ENSMUSG00000073792	4	99207683	99255475	71	71
3579	1	63679032	63761453	ENSMUSG00000028549	ENSMUSG00000028549	ENSMUSG00000028549	4	99257420	99321204	71	71
3580	1	63761649	63810952	ENSMUSG00000073791	ENSMUSG00000073791	ENSMUSG00000073791	4	99321366	99410542	71	71
3581	1	63861352	63898480	ENSMUSG00000025791	ENSMUSG00000025791	ENSMUSG00000025791	4	99427346	99485226	71	71
3582	1	64012302	64419769	ENSMUSG00000035305	ENSMUSG00000035305	ENSMUSG00000035305	4	99594151	99940177	71	71
3583	1	64442078	64505479	ENSMUSG00000069733	ENSMUSG00000069733	ENSMUSG00000069733	4	99988027	100047934	71	71
3584	1	64709063	64931329	ENSMUSG00000028532	ENSMUSG00000028532	ENSMUSG00000028532	4	100274611	100501696	71	71
3585	1	64983366	65071500	ENSMUSG00000035275	ENSMUSG00000035275	ENSMUSG00000035275	4	100566970	100650302	71	71
3586	1	65072346	65124605	ENSMUSG00000028530	ENSMUSG00000028530	ENSMUSG00000028530	4	100651427	100689608	71	71
3587	1	65547816	65653547	ENSMUSG00000028528	ENSMUSG00000028528	ENSMUSG00000028528	4	101005932	101140730	71	71
3588	1	65658923	65879830	ENSMUSG00000057722	ENSMUSG00000057722	ENSMUSG00000057722	4	101215840	101313489	71	71
3589	1	66030785	66612850	ENSMUSG00000028525	ENSMUSG00000028525	ENSMUSG00000028525	4	101753153	102103271	71	71
3590	1	66864108	66981911	ENSMUSG00000028524	ENSMUSG00000028524	ENSMUSG00000028524	4	102258295	102469109	71	71
3591	1	66990730	67017058	ENSMUSG00000028523	ENSMUSG00000028523	ENSMUSG00000028523	4	102486505	102502276	71	71
3592	1	67036012	67039527	ENSMUSG00000066090	ENSMUSG00000066090	ENSMUSG00000066090	4	102515804	102524660	71	71
3593	1	67051156	67163158	ENSMUSG00000035126	ENSMUSG00000035126	ENSMUSG00000035126	4	102536001	102612156	71	71
3594	1	67163230	67226301	ENSMUSG00000028522	ENSMUSG00000028522	ENSMUSG00000028522	4	102613570	102660701	71	71
3595	1	67237603	67292370	ENSMUSG00000028521	ENSMUSG00000028521	ENSMUSG00000028521	4	102668586	102712735	71	71
3596	1	67330447	67366808	ENSMUSG00000028520	ENSMUSG00000028520	ENSMUSG00000028520	4	102728380	102788790	71	71
3597	1	58718979	58785060	ENSMUSG00000035069	ENSMUSG00000035069	ENSMUSG00000035069	4	102811778	102864360	72	72
3598	1	57233043	57529428	ENSMUSG00000028519	ENSMUSG00000028519	ENSMUSG00000028519	4	103865503	104242774	72	72
3599	1	57167360	57204276	ENSMUSG00000029656	ENSMUSG00000029656	ENSMUSG00000029656	4	104264249	104302480	72	72
3600	1	57093065	57156482	ENSMUSG00000035031	ENSMUSG00000035031	ENSMUSG00000035031	4	104313604	104374315	72	72
3601	1	56732527	56817721	ENSMUSG00000028517	ENSMUSG00000028517	ENSMUSG00000028517	4	104655279	104730699	72	72
3602	1	55304620	55453627	ENSMUSG00000028514	ENSMUSG00000028514	ENSMUSG00000028514	4	105814404	105936768	72	72
3603	1	55277809	55303113	ENSMUSG00000044254	ENSMUSG00000044254	ENSMUSG00000044254	4	105940274	105962261	72	72
3604	1	55237194	55247244	ENSMUSG00000025418	ENSMUSG00000025418	ENSMUSG00000025418	4	105981388	105990172	72	72
3605	1	55087888	55125493	ENSMUSG00000034926	ENSMUSG00000034926	ENSMUSG00000034926	4	106058970	106087044	72	72
3606	1	55044324	55080513	ENSMUSG00000054362	ENSMUSG00000054362	ENSMUSG00000054362	4	106088841	106115170	72	72
3607	1	55017973	55039528	ENSMUSG00000034919	ENSMUSG00000034919	ENSMUSG00000034919	4	106120381	106138121	72	72
3608	1	54995159	55002775	ENSMUSG00000043572	ENSMUSG00000043572	ENSMUSG00000043572	4	106149038	106153214	72	72
3609	1	54890206	54954885	ENSMUSG00000047502	ENSMUSG00000047502	ENSMUSG00000047502	4	106188138	106219411	72	72
3610	1	54847443	54861817	ENSMUSG00000034871	ENSMUSG00000034871	ENSMUSG00000034871	4	106231838	106246220	72	72
3611	1	54786489	54877453	ENSMUSG00000034853	ENSMUSG00000034853	ENSMUSG00000034853	4	106242487	106297778	72	72
3612	1	54464893	54644680	ENSMUSG00000061887	ENSMUSG00000061887	ENSMUSG00000061887	4	106409402	106547626	72	72
3613	1	54438428	54456639	ENSMUSG00000028622	ENSMUSG00000028622	ENSMUSG00000028622	4	106553817	106564800	72	72
3614	1	54377256	54392031	ENSMUSG00000047636	ENSMUSG00000047636	ENSMUSG00000047636	4	106594823	106605680	72	72
3615	1	54292395	54334754	ENSMUSG00000028619	ENSMUSG00000028619	ENSMUSG00000028619	4	106636755	106676252	72	72
3616	1	54269937	54291699	ENSMUSG00000028618	ENSMUSG00000028618	ENSMUSG00000028618	4	106676611	106698924	72	72
3617	1	54247094	54256391	ENSMUSG00000070877	ENSMUSG00000070877	ENSMUSG00000070877	4	106710487	106715846	72	72
3618	1	54184625	54206429	ENSMUSG00000028617	ENSMUSG00000028617	ENSMUSG00000028617	4	106731446	106751465	72	72
3619	1	54159822	54184190	ENSMUSG00000063172	ENSMUSG00000063172	ENSMUSG00000063172	4	106751830	106777770	72	72
3620	1	54089980	54128073	ENSMUSG00000057375	ENSMUSG00000057375	ENSMUSG00000057375	4	106812309	106857753	72	72
3621	1	54003721	54077121	ENSMUSG00000028614	ENSMUSG00000028614	ENSMUSG00000028614	4	106865970	106912273	72	72
3622	1	53744494	53972465	ENSMUSG00000034762	ENSMUSG00000034762	ENSMUSG00000034762	4	106932651	107132991	72	72
3623	1	53697660	53705749	ENSMUSG00000028610	ENSMUSG00000028610	ENSMUSG00000028610	4	107178599	107182094	72	72
3624	1	53483806	53566314	ENSMUSG00000028613	ENSMUSG00000028613	ENSMUSG00000028613	4	107300193	107372099	72	72
3625	1	53465152	53476870	ENSMUSG00000028609	ENSMUSG00000028609	ENSMUSG00000028609	4	107377701	107385322	72	72
3626	1	53452357	53458879	ENSMUSG00000028608	ENSMUSG00000028608	ENSMUSG00000028608	4	107387831	107395734	72	72
3627	1	53434689	53452457	ENSMUSG00000028607	ENSMUSG00000028607	ENSMUSG00000028607	4	107401912	107421466	72	72
3628	1	53325443	53380837	ENSMUSG00000008932	ENSMUSG00000008932	ENSMUSG00000008932	4	107466264	107511461	72	72
3629	1	53300442	53323761	ENSMUSG00000028600	ENSMUSG00000028600	ENSMUSG00000028600	4	107512786	107526609	72	72
3630	1	53165536	53289870	ENSMUSG00000028603	ENSMUSG00000028603	ENSMUSG00000028603	4	107541771	107616433	72	72
3631	1	53134244	53160027	ENSMUSG00000028601	ENSMUSG00000028601	ENSMUSG00000028601	4	107663423	107677239	72	72
3632	1	53080996	53133258	ENSMUSG00000034645	ENSMUSG00000034645	ENSMUSG00000034645	4	107679867	107703076	72	72
3633	1	52964769	53065602	ENSMUSG00000034636	ENSMUSG00000034636	ENSMUSG00000034636	4	107727656	107799028	72	72
3634	1	52925096	52936626	ENSMUSG00000048351	ENSMUSG00000048351	ENSMUSG00000048351	4	107826084	107838650	72	72
3635	1	52840632	52847311	ENSMUSG00000028597	ENSMUSG00000028597	ENSMUSG00000028597	4	107898322	107904642	72	72
3636	1	52661538	52791331	ENSMUSG00000034610	ENSMUSG00000034610	ENSMUSG00000034610	4	107957358	108057347	72	72
3637	1	52642824	52656580	ENSMUSG00000063800	ENSMUSG00000063800	ENSMUSG00000063800	4	108062802	108077259	72	72
3638	1	52611089	52642719	ENSMUSG00000028587	ENSMUSG00000028587	ENSMUSG00000028587	4	108077386	108112765	72	72
3639	1	52588855	52604452	ENSMUSG00000028582	ENSMUSG00000028582	ENSMUSG00000028582	4	108117888	108132054	72	72
3640	1	52380634	52584945	ENSMUSG00000034557	ENSMUSG00000034557	ENSMUSG00000034557	4	108137194	108191444	72	72
3641	1	52294385	52328976	ENSMUSG00000028568	ENSMUSG00000028568	ENSMUSG00000028568	4	108314650	108331311	72	72
3642	1	52258391	52294431	ENSMUSG00000028567	ENSMUSG00000028567	ENSMUSG00000028567	4	108332610	108360054	72	72
3643	1	52270363	52272076	ENSMUSG00000073775	ENSMUSG00000073775	ENSMUSG00000073775	4	108345717	108347345	72	72
3644	1	52146216	52229024	ENSMUSG00000003411	ENSMUSG00000003411	ENSMUSG00000003411	4	108388240	108438745	72	72
3645	1	52027451	52117065	ENSMUSG00000053510	ENSMUSG00000053510	ENSMUSG00000053510	4	108498587	108559709	72	72
3646	1	51592523	51757588	ENSMUSG00000028552	ENSMUSG00000028552	ENSMUSG00000028552	4	108778200	108885749	72	72
3647	1	51525518	51560470	ENSMUSG00000028555	ENSMUSG00000028555	ENSMUSG00000028555	4	108905044	108942681	72	72
3648	1	51340494	51386340	ENSMUSG00000060491	ENSMUSG00000060491	ENSMUSG00000060491	4	109003269	109029229	72	72
3649	1	51199005	51212893	ENSMUSG00000028551	ENSMUSG00000028551	ENSMUSG00000028551	4	109158808	109163304	72	72
3650	1	50677738	51198523	ENSMUSG00000010517	ENSMUSG00000010517	ENSMUSG00000010517	4	109174520	109461892	72	72
3651	1	50655809	50659630	ENSMUSG00000047143	ENSMUSG00000047143	ENSMUSG00000047143	4	109475985	109481616	72	72
3652	1	50347885	50441313	ENSMUSG00000028546	ENSMUSG00000028546	ENSMUSG00000028546	4	109701669	109784821	72	72
3653	1	48771114	50262172	ENSMUSG00000061298	ENSMUSG00000061298	ENSMUSG00000061298	4	109976396	111154355	72	72
3654	1	48965782	49015177	ENSMUSG00000028545	ENSMUSG00000028545	ENSMUSG00000028545	4	110912938	110957857	72	72
3655	1	48533631	48710432	ENSMUSG00000034401	ENSMUSG00000034401	ENSMUSG00000034401	4	111217942	111326298	72	72
3656	1	48460987	48486902	ENSMUSG00000028544	ENSMUSG00000028544	ENSMUSG00000028544	4	111375188	111400728	72	72
3657	1	48016709	48039881	ENSMUSG00000066074	ENSMUSG00000066074	ENSMUSG00000066074	4	114078220	114097899	72	72
3658	1	47654331	47656311	ENSMUSG00000044518	ENSMUSG00000044518	ENSMUSG00000044518	4	114423079	114423945	72	72
3659	1	47572175	47617098	ENSMUSG00000028719	ENSMUSG00000028719	ENSMUSG00000028719	4	114458570	114485078	72	72
3660	1	47488401	47552382	ENSMUSG00000028718	ENSMUSG00000028718	ENSMUSG00000028718	4	114498135	114541128	72	72
3661	1	47454551	47468030	ENSMUSG00000028717	ENSMUSG00000028717	ENSMUSG00000028717	4	114554358	114569687	72	72
3662	1	47421848	47428358	ENSMUSG00000028716	ENSMUSG00000028716	ENSMUSG00000028716	4	114586655	114591825	72	72
3663	1	47261827	47289010	ENSMUSG00000047155	ENSMUSG00000047155	ENSMUSG00000047155	4	114606614	114631909	72	72
3664	1	47037305	47057608	ENSMUSG00000028713	ENSMUSG00000028713	ENSMUSG00000028713	4	115122657	115145641	72	72
3665	1	46913418	46957323	ENSMUSG00000034210	ENSMUSG00000034210	ENSMUSG00000034210	4	115236005	115275259	72	72
3666	1	46910087	46911836	ENSMUSG00000044556	ENSMUSG00000044556	ENSMUSG00000044556	4	115277931	115278536	72	72
3667	1	46845978	46853392	ENSMUSG00000028709	ENSMUSG00000028709	ENSMUSG00000028709	4	115326024	115334115	72	72
3668	1	46795678	46842496	ENSMUSG00000028708	ENSMUSG00000028708	ENSMUSG00000028708	4	115352924	115377180	72	72
3669	1	46745255	46752471	ENSMUSG00000028707	ENSMUSG00000028707	ENSMUSG00000028707	4	115413051	115437858	72	72
3670	1	46632524	46652104	ENSMUSG00000034171	ENSMUSG00000034171	ENSMUSG00000034171	4	115465077	115515607	72	72
3671	1	46578977	46603275	ENSMUSG00000028706	ENSMUSG00000028706	ENSMUSG00000028706	4	115530774	115551808	72	72
3672	1	46516964	46541625	ENSMUSG00000028703	ENSMUSG00000028703	ENSMUSG00000028703	4	115573341	115594975	72	72
3673	1	46485991	46516731	ENSMUSG00000028702	ENSMUSG00000028702	ENSMUSG00000028702	4	115594848	115621617	72	72
3674	1	46441593	46537834	ENSMUSG00000028701	ENSMUSG00000028701	ENSMUSG00000028701	4	115635231	115642548	72	72
3675	1	46426942	46458564	ENSMUSG00000028700	ENSMUSG00000028700	ENSMUSG00000028700	4	115648997	115657776	72	72
3676	1	46413346	46424217	ENSMUSG00000028699	ENSMUSG00000028699	ENSMUSG00000028699	4	115659813	115665530	72	72
3677	1	46278399	46371053	ENSMUSG00000028698	ENSMUSG00000028698	ENSMUSG00000028698	4	115719846	115800988	72	72
3678	1	46041872	46274383	ENSMUSG00000003810	ENSMUSG00000003810	ENSMUSG00000003810	4	115804694	115961780	72	72
3679	1	45936970	45988908	ENSMUSG00000028696	ENSMUSG00000028696	ENSMUSG00000028696	4	116008504	116035956	72	72
3680	1	45925473	45932702	ENSMUSG00000055900	ENSMUSG00000055900	ENSMUSG00000055900	4	116050966	116053859	72	72
3681	1	45865563	45924795	ENSMUSG00000034042	ENSMUSG00000034042	ENSMUSG00000034042	4	116055659	116091816	72	72
3682	1	45858304	45862316	ENSMUSG00000034035	ENSMUSG00000034035	ENSMUSG00000034035	4	116094662	116098198	72	72
3683	1	45788802	45808308	ENSMUSG00000028692	ENSMUSG00000028692	ENSMUSG00000028692	4	116134440	116149583	72	72
3684	1	45738312	45749326	ENSMUSG00000028690	ENSMUSG00000028690	ENSMUSG00000028690	4	116200367	116206256	72	72
3685	1	45582142	45729425	ENSMUSG00000033985	ENSMUSG00000033985	ENSMUSG00000033985	4	116218898	116302171	72	72
3686	1	45577929	45582234	ENSMUSG00000028688	ENSMUSG00000028688	ENSMUSG00000028688	4	116301935	116305491	72	72
3687	1	45567502	45578647	ENSMUSG00000028687	ENSMUSG00000028687	ENSMUSG00000028687	4	116305670	116317370	72	72
3688	1	45565132	45566934	ENSMUSG00000043155	ENSMUSG00000043155	ENSMUSG00000043155	4	116318079	116319194	72	72
3689	1	45254658	45444815	ENSMUSG00000033948	ENSMUSG00000033948	ENSMUSG00000033948	4	116375334	116487037	72	72
3690	1	45250417	45253928	ENSMUSG00000028684	ENSMUSG00000028684	ENSMUSG00000028684	4	116488150	116492307	72	72
3691	1	45240799	45249588	ENSMUSG00000046861	ENSMUSG00000046861	ENSMUSG00000046861	4	116493280	116503209	72	72
3692	1	45089037	45224869	ENSMUSG00000028683	ENSMUSG00000028683	ENSMUSG00000028683	4	116520097	116568855	72	72
3693	1	45060192	45081322	ENSMUSG00000028681	ENSMUSG00000028681	ENSMUSG00000028681	4	116594288	116612763	72	72
3694	1	45044172	45044934	ENSMUSG00000047671	ENSMUSG00000047671	ENSMUSG00000047671	4	116624745	116626662	72	72
3695	1	45038723	45049479	ENSMUSG00000028680	ENSMUSG00000028680	ENSMUSG00000028680	4	116626587	116631884	72	72
3696	1	45021844	45025964	ENSMUSG00000033872	ENSMUSG00000033872	ENSMUSG00000033872	4	116643189	116646449	72	72
3697	1	44912981	44963848	ENSMUSG00000060268	ENSMUSG00000060268	ENSMUSG00000060268	4	116713122	116749849	72	72
3698	1	44891506	44912814	ENSMUSG00000048772	ENSMUSG00000048772	ENSMUSG00000048772	4	116749921	116766512	72	72
3699	1	44650357	44889034	ENSMUSG00000028677	ENSMUSG00000028677	ENSMUSG00000028677	4	116769396	116994847	72	72
3700	1	44459373	44593492	ENSMUSG00000033423	ENSMUSG00000033423	ENSMUSG00000033423	4	117048426	117172215	72	72
3701	1	44451712	44458938	ENSMUSG00000009640	ENSMUSG00000009640	ENSMUSG00000009640	4	117172613	117180205	72	72
3702	1	44357109	44373399	ENSMUSG00000048626	ENSMUSG00000048626	ENSMUSG00000048626	4	117255768	117263564	72	72

3703	1	44234742	44255584	ENSMUSG00000028542	ENSMUSG00000028542	ENSMUSG00000028542	4	117332438	117367230	72	72
3704	1	44217453	44229426	ENSMUSG00000028541	ENSMUSG00000028541	ENSMUSG00000028541	4	117367196	117381087	72	72
3705	1	44213198	44216553	ENSMUSG00000033379	ENSMUSG00000033379	ENSMUSG00000033379	4	117382262	117385261	72	72
3706	1	44208246	44211632	ENSMUSG00000028540	ENSMUSG00000028540	ENSMUSG00000028540	4	117386575	117389935	72	72
3707	1	44185198	44206281	ENSMUSG00000033365	ENSMUSG00000033365	ENSMUSG00000033365	4	117392425	117412931	72	72
3708	1	44172071	44175498	ENSMUSG00000028539	ENSMUSG00000028539	ENSMUSG00000028539	4	117424093	117427402	72	72
3709	1	43945805	44169417	ENSMUSG00000028538	ENSMUSG00000028538	ENSMUSG00000028538	4	117430086	117632822	72	72
3710	1	43888416	43943773	ENSMUSG00000033326	ENSMUSG00000033326	ENSMUSG00000033326	4	117634891	117677552	72	72
3711	1	43769134	43861924	ENSMUSG00000033295	ENSMUSG00000033295	ENSMUSG00000033295	4	117707733	117775378	72	72
3712	1	43689416	43692221	ENSMUSG00000006395	ENSMUSG00000006395	ENSMUSG00000006395	4	117857946	117860676	72	72
3713	1	43661384	43690891	ENSMUSG00000033253	ENSMUSG00000033253	ENSMUSG00000033253	4	117860675	117881347	72	72
3714	1	43622176	43628070	ENSMUSG00000006392	ENSMUSG00000006392	ENSMUSG00000006392	4	117908811	117913041	72	72
3715	1	43601428	43606263	ENSMUSG00000006390	ENSMUSG00000006390	ENSMUSG00000006390	4	117926025	117930848	72	72
3716	1	43576065	43591030	ENSMUSG00000006389	ENSMUSG00000006389	ENSMUSG00000006389	4	117940347	117955416	72	72
3717	1	43539251	43561366	ENSMUSG00000033191	ENSMUSG00000033191	ENSMUSG00000033191	4	117969123	117987993	72	72
3718	1	43520149	43523826	ENSMUSG00000028536	ENSMUSG00000028536	ENSMUSG00000028536	4	118025207	118028149	72	72
3719	1	43508283	43512259	ENSMUSG00000050854	ENSMUSG00000050854	ENSMUSG00000050854	4	118038874	118041660	72	72
3720	1	43410613	43449136	ENSMUSG00000028730	ENSMUSG00000028730	ENSMUSG00000028730	4	118090768	118118337	72	72
3721	1	43402440	43410518	ENSMUSG00000028729	ENSMUSG00000028729	ENSMUSG00000028729	4	118118748	118125708	72	72
3722	1	43164106	43197088	ENSMUSG00000028645	ENSMUSG00000028645	ENSMUSG00000028645	4	118606700	118635259	72	72
3723	1	43087813	43090731	ENSMUSG00000045268	ENSMUSG00000045268	ENSMUSG00000045268	4	118667452	118671788	72	72
3724	1	43055363	43083247	ENSMUSG00000028644	ENSMUSG00000028644	ENSMUSG00000028644	4	118673389	118687943	72	72
3725	1	43045310	43055062	ENSMUSG00000028643	ENSMUSG00000028643	ENSMUSG00000028643	4	118693471	118699023	72	72
3726	1	43028035	43036462	ENSMUSG00000028642	ENSMUSG00000028642	ENSMUSG00000028642	4	118702987	118716149	72	72
3727	1	42984633	43005270	ENSMUSG00000028641	ENSMUSG00000028641	ENSMUSG00000028641	4	118730878	118746904	72	72
3728	1	42971351	42978512	ENSMUSG00000066058	ENSMUSG00000066058	ENSMUSG00000066058	4	118753401	118757787	72	72
3729	1	42896635	42915015	ENSMUSG00000060288	ENSMUSG00000060288	ENSMUSG00000060288	4	118797943	118818433	72	72
3730	1	42772129	42892444	ENSMUSG00000028637	ENSMUSG00000028637	ENSMUSG00000028637	4	118821773	118913380	72	72
3731	1	42694789	42698707	ENSMUSG00000028636	ENSMUSG00000028636	ENSMUSG00000028636	4	118916465	118920352	72	72
3732	1	42668587	42694525	ENSMUSG00000070806	ENSMUSG00000070806	ENSMUSG00000070806	4	118920805	118951831	72	72
3733	1	42619064	42653437	ENSMUSG00000048899	ENSMUSG00000048899	ENSMUSG00000048899	4	118963215	118990511	72	72
3734	1	42414797	42574135	ENSMUSG00000032998	ENSMUSG00000032998	ENSMUSG00000032998	4	119037620	119127051	72	72
3735	1	42400949	42402982	ENSMUSG00000023247	ENSMUSG00000023247	ENSMUSG00000023247	4	119135647	119137395	72	72
3736	1	42391679	42394082	ENSMUSG00000032978	ENSMUSG00000032978	ENSMUSG00000032978	4	119154547	119156877	72	72
3737	1	41744623	42156756	ENSMUSG00000028634	ENSMUSG00000028634	ENSMUSG00000028634	4	119312363	119633046	72	72
3738	1	41717033	41722929	ENSMUSG00000028635	ENSMUSG00000028635	ENSMUSG00000028635	4	119658836	119664993	72	72
3739	1	41600181	41621849	ENSMUSG00000052135	ENSMUSG00000052135	ENSMUSG00000052135	4	119764721	119784984	72	72
3740	1	41265464	41399161	ENSMUSG00000000085	ENSMUSG00000000085	ENSMUSG00000000085	4	119950878	120027525	72	72
3741	1	41253856	41261496	ENSMUSG00000047518	ENSMUSG00000047518	ENSMUSG00000047518	4	120029866	120034296	72	72
3742	1	41217594	41250822	ENSMUSG00000028633	ENSMUSG00000028633	ENSMUSG00000028633	4	120037501	120067899	72	72
3743	1	41099310	41100625	ENSMUSG00000070803	ENSMUSG00000070803	ENSMUSG00000070803	4	120164207	120165453	72	72
3744	1	41022271	41078711	ENSMUSG00000028631	ENSMUSG00000028631	ENSMUSG00000028631	4	120195532	120244811	72	72
3745	1	40858938	40903916	ENSMUSG00000032890	ENSMUSG00000032890	ENSMUSG00000032890	4	120352466	120389437	72	72
3746	1	40747000	40754295	ENSMUSG00000028629	ENSMUSG00000028629	ENSMUSG00000028629	4	120418837	120422640	72	72
3747	1	40715474	40734602	ENSMUSG00000064141	ENSMUSG00000064141	ENSMUSG00000064141	4	120427988	120432735	72	72
3748	1	40611956	40661585	ENSMUSG00000032870	ENSMUSG00000032870	ENSMUSG00000032870	4	120465957	120514882	72	72
3749	1	40538746	40555553	ENSMUSG00000028626	ENSMUSG00000028626	ENSMUSG00000028626	4	120536967	120552936	72	72
3750	1	40496366	40532443	ENSMUSG00000043207	ENSMUSG00000043207	ENSMUSG00000043207	4	120556873	120595878	72	72
3751	1	40399632	40479180	ENSMUSG00000049878	ENSMUSG00000049878	ENSMUSG00000049878	4	120643993	120680373	72	72
3752	1	40310972	40335555	ENSMUSG00000028657	ENSMUSG00000028657	ENSMUSG00000028657	4	122338549	122361381	72	72
3753	1	40278492	40310908	ENSMUSG00000028656	ENSMUSG00000028656	ENSMUSG00000028656	4	122361365	122388166	72	72
3754	1	40193409	40208225	ENSMUSG00000028655	ENSMUSG00000028655	ENSMUSG00000028655	4	122449158	122463490	72	72
3755	1	40079295	40121763	ENSMUSG00000028653	ENSMUSG00000028653	ENSMUSG00000028653	4	122518901	122557239	72	72
3756	1	39977117	40000883	ENSMUSG00000028651	ENSMUSG00000028651	ENSMUSG00000028651	4	122629419	122642239	72	72
3757	1	39916907	39929948	ENSMUSG00000046093	ENSMUSG00000046093	ENSMUSG00000046093	4	122685807	122697002	72	72
3758	1	39897380	39910297	ENSMUSG00000054958	ENSMUSG00000054958	ENSMUSG00000054958	4	122703861	122718510	72	72
3759	1	39862412	39878204	ENSMUSG00000032744	ENSMUSG00000032744	ENSMUSG00000032744	4	122735859	122751023	72	72
3760	1	39569397	39725376	ENSMUSG00000028649	ENSMUSG00000028649	ENSMUSG00000028649	4	122852021	123186663	72	72
3761	1	39229547	39241831	ENSMUSG00000023075	ENSMUSG00000023075	ENSMUSG00000023075	4	123232456	123252648	72	72
3762	1	39076457	39098082	ENSMUSG00000028646	ENSMUSG00000028646	ENSMUSG00000028646	4	123419777	123439293	72	72
3763	1	38251001	38263083	ENSMUSG00000028907	ENSMUSG00000028907	ENSMUSG00000028907	4	124181068	124195858	72	72
3764	1	38235025	38243830	ENSMUSG00000032643	ENSMUSG00000032643	ENSMUSG00000032643	4	124203024	124210404	72	72
3765	1	38100327	38184568	ENSMUSG00000028894	ENSMUSG00000028894	ENSMUSG00000028894	4	124244838	124303815	72	72
3766	1	38052438	38097879	ENSMUSG00000028890	ENSMUSG00000028890	ENSMUSG00000028890	4	124304876	124349954	72	72
3767	1	38041201	38046452	ENSMUSG00000028889	ENSMUSG00000028889	ENSMUSG00000028889	4	124353079	124357546	72	72
3768	1	38032129	38039392	ENSMUSG00000042763	ENSMUSG00000042763	ENSMUSG00000042763	4	124357519	124364482	72	72
3769	1	37930677	37947978	ENSMUSG00000028873	ENSMUSG00000028873	ENSMUSG00000028873	4	124421415	124439177	72	72
3770	1	37919829	37928779	ENSMUSG00000044730	ENSMUSG00000044730	ENSMUSG00000044730	4	124439474	124446959	72	72
3771	1	37849538	37872924	ENSMUSG00000028871	ENSMUSG00000028871	ENSMUSG00000028871	4	124488734	124511403	72	72
3772	1	37805004	37834109	ENSMUSG00000028869	ENSMUSG00000028869	ENSMUSG00000028869	4	124532342	124557676	72	72
3773	1	37795107	37805045	ENSMUSG00000042707	ENSMUSG00000042707	ENSMUSG00000042707	4	124557643	124567961	72	72
3774	1	37730763	37752951	ENSMUSG00000028863	ENSMUSG00000028863	ENSMUSG00000028863	4	124587451	124612193	72	72
3775	1	37712740	37722565	ENSMUSG00000042677	ENSMUSG00000042677	ENSMUSG00000042677	4	124620727	124630144	72	72
3776	1	37033715	37272313	ENSMUSG00000001985	ENSMUSG00000001985	ENSMUSG00000001985	4	125125784	125210392	72	72
3777	1	36704231	36721466	ENSMUSG00000028859	ENSMUSG00000028859	ENSMUSG00000028859	4	125529618	125546743	72	72
3778	1	36693906	36702625	ENSMUSG00000028861	ENSMUSG00000028861	ENSMUSG00000028861	4	125549232	125557843	72	72
3779	1	36656094	36688639	ENSMUSG00000042616	ENSMUSG00000042616	ENSMUSG00000042616	4	125560905	125591638	72	72
3780	1	36631618	36636080	ENSMUSG00000050188	ENSMUSG00000050188	ENSMUSG00000050188	4	125599028	125600885	72	72
3781	1	36577812	36624076	ENSMUSG00000042608	ENSMUSG00000042608	ENSMUSG00000042608	4	125620611	125643183	72	72
3782	1	36560219	36562342	ENSMUSG00000050212	ENSMUSG00000050212	ENSMUSG00000050212	4	125650048	125652178	72	72
3783	1	36545305	36563074	ENSMUSG00000073758	ENSMUSG00000073758	ENSMUSG00000073758	4	125653029	125665431	72	72
3784	1	36394386	36419035	ENSMUSG00000028849	ENSMUSG00000028849	ENSMUSG00000028849	4	125734471	125758587	72	72
3785	1	36374760	36387657	ENSMUSG00000028847	ENSMUSG00000028847	ENSMUSG00000028847	4	125764709	125778187	72	72
3786	1	36333424	36338437	ENSMUSG00000056174	ENSMUSG00000056174	ENSMUSG00000056174	4	125789098	125816634	72	72
3787	1	36327063	36332120	ENSMUSG00000042558	ENSMUSG00000042558	ENSMUSG00000042558	4	125818359	125823984	72	72
3788	1	36322263	36326462	ENSMUSG00000028845	ENSMUSG00000028845	ENSMUSG00000028845	4	125824425	125827992	72	72
3789	1	36169266	36294650	ENSMUSG00000028842	ENSMUSG00000028842	ENSMUSG00000028842	4	125835036	125931860	72	72
3790	1	36121397	36162487	ENSMUSG00000041530	ENSMUSG00000041530	ENSMUSG00000041530	4	125941807	125970725	72	72
3791	1	36046360	36096078	ENSMUSG00000042500	ENSMUSG00000042500	ENSMUSG00000042500	4	125999037	126028471	72	72
3792	1	35974405	36008138	ENSMUSG00000042489	ENSMUSG00000042489	ENSMUSG00000042489	4	126059239	126096207	72	72
3793	1	35952064	35957377	ENSMUSG00000073755	ENSMUSG00000073755	ENSMUSG00000073755	4	126113985	126114677	72	72
3794	1	35808000	35879718	ENSMUSG00000028837	ENSMUSG00000028837	ENSMUSG00000028837	4	126179970	126212010	72	72
3795	1	35811558	35833516	ENSMUSG00000042477	ENSMUSG00000042477	ENSMUSG00000042477	4	126218302	126238412	72	72
3796	1	35795980	35804967	ENSMUSG00000028833	ENSMUSG00000028833	ENSMUSG00000028833	4	126246059	126255652	72	72
3797	1	35671678	35795601	ENSMUSG00000028830	ENSMUSG00000028830	ENSMUSG00000028830	4	126255859	126372374	72	72
3798	1	35507155	35660132	ENSMUSG00000042446	ENSMUSG00000042446	ENSMUSG00000042446	4	126365932	126470482	72	72
3799	1	35317555	35354043	ENSMUSG00000043872	ENSMUSG00000043872	ENSMUSG00000043872	4	126549398	126561351	72	72
3800	1	35225168	35270156	ENSMUSG00000042408	ENSMUSG00000042408	ENSMUSG00000042408	4	126579687	126626598	72	72
3801	1	35103624	35167773	ENSMUSG00000042388	ENSMUSG00000042388	ENSMUSG00000042388	4	126671575	126739332	72	72
3802	1	35031186	35033935	ENSMUSG00000050234	ENSMUSG00000050234	ENSMUSG00000050234	4	126813725	126816282	72	72
3803	1	35019377	35024554	ENSMUSG00000042367	ENSMUSG00000042367	ENSMUSG00000042367	4	126827543	126832897	72	72
3804	1	34997929	35001912	ENSMUSG00000046623	ENSMUSG00000046623	ENSMUSG00000046623	4	126853390	126856385	72	72
3805	1	34993235	34996700	ENSMUSG00000042357	ENSMUSG00000042357	ENSMUSG00000042357	4	126857113	126860468	72	72
3806	1	34405071	34457319	ENSMUSG00000028813	ENSMUSG00000028813	ENSMUSG00000028813	4	127430307	127453688	72	72
3807	1	33752200	34403462	ENSMUSG00000028804	ENSMUSG00000028804	ENSMUSG00000028804	4	127490374	128066469	72	72
3808	1	34098663	34102978	ENSMUSG00000048686	ENSMUSG00000048686	ENSMUSG00000048686	4	127762532	127763077	72	72
3809	1	33717477	33734578	ENSMUSG00000061894	ENSMUSG00000061894	ENSMUSG00000061894	4	128087680	128112318	72	72
3810	1	33561814	33613781	ENSMUSG00000028796	ENSMUSG00000028796	ENSMUSG00000028796	4	128207300	128255178	72	72
3811	1	33544954	33551077	ENSMUSG00000028794	ENSMUSG00000028794	ENSMUSG00000028794	4	128261562	128271602	72	72
3812	1	33508744	33538907	ENSMUSG00000028799	ENSMUSG00000028799	ENSMUSG00000028799	4	128275399	128292643	72	72
3813	1	33383590	33419854	ENSMUSG00000041000	ENSMUSG00000041000	ENSMUSG00000041000	4	128386444	128413634	72	72
3814	1	33320438	33358370	ENSMUSG00000028789	ENSMUSG00000028789	ENSMUSG00000028789	4	128434450	128464743	72	72
3815	1	33251232	33275180	ENSMUSG00000028792	ENSMUSG00000028792	ENSMUSG00000028792	4	128495569	128510702	72	72
3816	1	33132781	33139590	ENSMUSG00000028786	ENSMUSG00000028786	ENSMUSG00000028786	4	128607905	128613927	72	72
3817	1	33124623	33132834	ENSMUSG00000028785	ENSMUSG00000028785	ENSMUSG00000028785	4	128613880	128623607	72	72
3818	1	33100482	33108934	ENSMUSG00000001334	ENSMUSG00000001334	ENSMUSG00000001334	4	128639303	128646897	72	72
3819	1	33055630	33096042	ENSMUSG00000040928	ENSMUSG00000040928	ENSMUSG00000040928	4	128651676	128684834	72	72
3820	1	33013427	33056341	ENSMUSG00000028811	ENSMUSG00000028811	ENSMUSG00000028811	4	128692255	128721911	72	72
3821	1	32992209	33013158	ENSMUSG00000050390	ENSMUSG00000050390	ENSMUSG00000050390	4	128721882	128750650	72	72
3822	1	32918548	32933460	ENSMUSG00000001333	ENSMUSG00000001333	ENSMUSG00000001333	4	128789921	128810863	72	72
3823	1	32703245	32838980	ENSMUSG00000048485	ENSMUSG00000048485	ENSMUSG00000048485	4	128928574	128943095	72	72
3824	1	32603359	32632614	ENSMUSG00000040859	ENSMUSG00000040859	ENSMUSG00000040859	4	128963988	128990702	72	72
3825	1	32600385	32602500	ENSMUSG00000000411	ENSMUSG00000000411	ENSMUSG00000000411	4	128991312	128993074	72	72
3826	1	32572020	32574567	ENSMUSG00000047945	ENSMUSG00000047945	ENSMUSG00000047945	4	129015935	129018275	72	72
3827	1	32512320	32524350	ENSMUSG00000000409	ENSMUSG00000000409	ENSMUSG00000000409	4	129050653	129075945	72	72
3828	1	32485421	32487044	ENSMUSG00000050493	ENSMUSG00000050493	ENSMUSG00000050493	4	129079168	129080851	72	72
3829	1	32454385	32460531	ENSMUSG00000028797	ENSMUSG00000028797	ENSMUSG00000028797	4	129102984	129110832	72	72
3830	1	32443849	32446875	ENSMUSG00000040795	ENSMUSG00000040795	ENSMUSG00000040795	4	129117569	129121374	72	72
3831	1	32438821	32443575	ENSMUSG00000028795	ENSMUSG00000028795	ENSMUSG00000028795	4	129121578	129126212	72	72
3832	1	32346244	32411361	ENSMUSG00000003731	ENSMUSG00000003731	ENSMUSG00000003731	4	129146284	129175071	72	72
3833	1	32311090	32341049	ENSMUSG00000053730	ENSMUSG00000053730	ENSMUSG00000053730	4	129178659	129199142	72	72
3834	1	32252017	32282070	ENSMUSG00000028790	ENSMUSG00000028790	ENSMUSG00000028790	4	129206041	129244601	72	72
3835	1	32144609	32176578	ENSMUSG00000028788	ENSMUSG00000028788	ENSMUSG00000028788	4	129322782	129350676	72	72
3836	1	32028612	32054167	ENSMUSG00000028784	ENSMUSG00000028784	ENSMUSG00000028784	4	129404785	129458848	72	72
3837	1	31965306	32002223	ENSMUSG00000028782	ENSMUSG00000028782	ENSMUSG00000028782	4	129494372	129524652	72	72
3838	1	31890435	31942507	ENSMUSG00000040690	ENSMUSG00000040690	ENSMUSG00000040690	4	129550192	129601579	72	72
3839	1	31868050	31883075	ENSMUSG00000028779	ENSMUSG00000028779	ENSMUSG00000028779	4	129609860	129630438	72	72
3840	1	31857228	31865379	ENSMUSG00000028778	ENSMUSG00000028778	ENSMUSG00000028778	4	129633145	129639932	72	72
3841	1	31814700	31825872	ENSMUSG00000028776	ENSMUSG00000028776	ENSMUSG00000028776	4	129666758	129677106	72	72
3842	1	31654999	31680111	ENSMUSG00000023232	ENSMUSG00000023232	ENSMUSG00000023232	4	129755801	129777871	72	72
3843	1	31542429	31610370	ENSMUSG00000028772	ENSMUSG00000028772	ENSMUSG00000028772	4	129818951	129840889	72	72
3844	1	31178284	31311350	ENSMUSG00000028580	ENSMUSG00000028580	ENSMUSG00000028580	4	129935455	130053638	72	72
3845	1	31118569	31154195	ENSMUSG00000025743	ENSMUSG00000025743	ENSMUSG00000025743	4	130064613	130098394	72	72
3846	1	30977903	31003254	ENSMUSG00000028581	ENSMUSG00000028581	ENSMUSG00000028581	4	130185392	130208215	72	72
3847	1	30956711	30969021	ENSMUSG00000040533	ENSMUSG00000040533	ENSMUSG00000040533	4	130216461	130227092	72	72
3848	1	29435615	29525898	ENSMUSG00000028909	ENSMUSG00000028909	ENSMUSG00000028909	4	131040533	131110364	72	72
3849	1	29391972	29430041	ENSMUSG00000028910	ENSMUSG00000028910	ENSMUSG00000028910	4	131115547	131139843	72	72
3850	1	29346842	29381086	ENSMUSG00000028911	ENSMUSG00000028911	ENSMUSG00000028911	4	131145732	131173776	72	72
3851	1	29318526	29323008	ENSMUSG00000070720	ENSMUSG00000070720	ENSMUSG00000070720	4	131193847	131194701	72	72
3852	1	29092840	29319140	ENSMUSG00000028906	ENSMUSG00000028906	ENSMUSG00000028906	4	131196332	131344173	72	72
3853	1	29011241	29062795	ENSMUSG00000050511	ENSMUSG00000050511	ENSMUSG00000050511	4	131382802	131416562	72	72
3854	1	28935858	28968874	ENSMUSG00000040025	ENSMUSG00000040025	ENSMUSG00000040025	4	131458133	131484332	72	72
3855	1	28867831	28913972	ENSMUSG00000028901	ENSMUSG00000028901	ENSMUSG00000028901	4	131497095	131523928	72	72
3856	1	28801949	28842165	ENSMUSG00000028899	ENSMUSG00000028899	ENSMUSG00000028899	4	131546489	131565403	72	72
3857	1	28752184	28777638	ENSMUSG00000028898	ENSMUSG00000028898	ENSMUSG00000028898	4	131583841	131601592	72	72
3858	1	28717367	28737263	ENSMUSG00000028896	ENSMUSG00000028896	ENSMUSG00000028896	4	131604002	131617781	72	72
3859	1	28637269	28699407	ENSMUSG00000066043	ENSMUSG00000066043	ENSMUSG00000066043	4	131632547	131694499	72	72
3860	1	28528136	28535063	ENSMUSG00000066042	ENSMUSG00000066042	ENSMUSG00000066042	4	131730807	131735997	72	72
3861	1	28458593	28481586	ENSMUSG00000028893	ENSMUSG00000028893	ENSMUSG00000028893	4	131764872	131782532	72	72
3862	1	28435231	28437198	ENSMUSG00000054428	ENSMUSG00000054428	ENSMUSG00000054428	4	131802638	131805724	72	72
3863	1	28399657	28432126	ENSMUSG00000054405	ENSMUSG00000054405	ENSMUSG00000054405	4	131807632	131825811	72	72
3864	1	28346264	28376286	ENSMUSG00000056529	ENSMUSG00000056529	ENSMUSG00000056529	4	131836299	131852935	72	72
3865	1	28169442	28287794	ENSMUSG00000028886	ENSMUSG00000028886	ENSMUSG00000028886	4	131911122	131996841	72	72
3866	1	28158560	28167194	ENSMUSG00000037752	ENSMUSG00000037752	ENSMUSG00000037752	4	131996980	132004622	72	72
3867	1	28091171	28113611	ENSMUSG00000028884	ENSMUSG00000028884	ENSMUSG00000028884	4	132040411	132050828	72	72
3868	1	28071666	28085771	ENSMUSG00000037731	ENSMUSG00000037731	ENSMUSG00000037731	4	132053926	132068434	72	72
3869	1	28029876	28050774	ENSMUSG00000028882	ENSMUSG00000028882	ENSMUSG00000028882	4	132099005	132115196	72	72
3870	1	27972370	28023401	ENSMUSG00000028879	ENSMUSG00000028879	ENSMUSG00000028879	4	132125579	132156519	72	72
3871	1	27811162	27834375	ENSMUSG00000028874	ENSMUSG00000028874	ENSMUSG00000028874	4	132246266	132273953	72	72
3872	1	27733133	27803275	ENSMUSG00000037692	ENSMUSG00000037692	ENSMUSG00000037692	4	132283582	132349324	72	72
3873	1	27591735	27594905	ENSMUSG00000049649	ENSMUSG00000049649	ENSMUSG00000049649	4	132481412	132484583	72	72
3874	1	27578253	27582457	ENSMUSG00000028865	ENSMUSG00000028865	ENSMUSG00000028865	4	132484452	132496086	72	72
3875	1	27554257	27565924	ENSMUSG00000028862	ENSMUSG00000028862	ENSMUSG00000028862	4	132512894	132525004	72	72
3876	1	27541100	27553008	ENSMUSG00000028860	ENSMUSG00000028860	ENSMUSG00000028860	4	132525175	132535163	72	72
3877	1	27521309	27535477	ENSMUSG00000028857	ENSMUSG00000028857	ENSMUSG00000028857	4	132538123	132549863	72	72
3878	1	27433746	27507322	ENSMUSG00000037622	ENSMUSG00000037622	ENSMUSG00000037622	4	132564542	132611181	72	72
3879	1	27297894	27353990	ENSMUSG00000028854	ENSMUSG00000028854	ENSMUSG00000028854	4	132642305	132695015	72	72
3880	1	27204098	27211914	ENSMUSG00000046694	ENSMUSG00000046694	ENSMUSG00000046694	4	132752213	132760012	72	72
3881	1	27192785	27199731	ENSMUSG00000056596	ENSMUSG00000056596	ENSMUSG00000056596	4	132763180	132770582	72	72
3882	1	27148640	27159484	ENSMUSG00000037600	ENSMUSG00000037600	ENSMUSG00000037600	4	132791111	132802868	72	72
3883	1	27110566	27113047	ENSMUSG00000037583	ENSMUSG00000037583	ENSMUSG00000037583	4	132825456	132828611	72	72
3884	1	27089567	27099549	ENSMUSG00000028850	ENSMUSG00000028850	ENSMUSG00000028850	4	132846821	132856318	72	72
3885	1	27078360	27089375	ENSMUSG00000028848	ENSMUSG00000028848	ENSMUSG00000028848	4	132856456	132863813	72	72
3886	1	27062216	27063535	ENSMUSG00000047281	ENSMUSG00000047281	ENSMUSG00000047281	4	132873099	132873845	72	72
3887	1	27025788	27054789	ENSMUSG00000037553	ENSMUSG00000037553	ENSMUSG00000037553	4	132880732	132905198	72	72
3888	1	26987042	26997363	ENSMUSG00000043257	ENSMUSG00000043257	ENSMUSG00000043257	4	132932463	132944696	72	72
3889	1	26895111	26981182	ENSMUSG00000007880	ENSMUSG00000007880	ENSMUSG00000007880	4	132952036	133028845	72	72
3890	1	26744930	26774107	ENSMUSG00000003644	ENSMUSG00000003644	ENSMUSG00000003644	4	133119370	133159860	72	72
3891	1	26631389	26670380	ENSMUSG00000012117	ENSMUSG00000012117	ENSMUSG00000012117	4	133241133	133272940	72	72
3892	1	26609856	26628800	ENSMUSG00000050966	ENSMUSG00000050966	ENSMUSG00000050966	4	133275406	133290892	72	72
3893	1	26521046	26541900	ENSMUSG00000012123	ENSMUSG00000012123	ENSMUSG00000012123	4	133351011	133364577	72	72
3894	1	26481362	26517441	ENSMUSG00000012126	ENSMUSG00000012126	ENSMUSG00000012126	4	133374675	133398854	72	72
3895	1	26478254	26480594	ENSMUSG00000028843	ENSMUSG00000028843	ENSMUSG00000028843	4	133399482	133400829	72	72
3896	1	26433280	26477884	ENSMUSG00000037443	ENSMUSG00000037443	ENSMUSG00000037443	4	133403355	133459113	72	72
3897	1	26389706	26401620	ENSMUSG00000048003	ENSMUSG00000048003	ENSMUSG00000048003	4	133484046	133499444	72	72

3898	1	26376587	26388962	ENSMUSG00000028841	ENSMUSG00000028841	ENSMUSG00000028841	4	133500119	133510384	72	72
3899	1	26368979	26370390	ENSMUSG00000028840	ENSMUSG00000028840	ENSMUSG00000028840	4	133515382	133517604	72	72
3900	1	26358098	26361706	ENSMUSG00000050105	ENSMUSG00000050105	ENSMUSG00000050105	4	133523188	133526178	72	72
3901	1	26310915	26324621	ENSMUSG00000050890	ENSMUSG00000050890	ENSMUSG00000050890	4	133547078	133559908	72	72
3902	1	26250382	26266711	ENSMUSG00000028834	ENSMUSG00000028834	ENSMUSG00000028834	4	133587300	133599857	72	72
3903	1	26237100	26245211	ENSMUSG00000028836	ENSMUSG00000028836	ENSMUSG00000028836	4	133615785	133625260	72	72
3904	1	26220858	26235542	ENSMUSG00000028838	ENSMUSG00000028838	ENSMUSG00000028838	4	133628454	133644623	72	72
3905	1	26158845	26197235	ENSMUSG00000037366	ENSMUSG00000037366	ENSMUSG00000037366	4	133669545	133699488	72	72
3906	1	26060288	26070331	ENSMUSG00000037348	ENSMUSG00000037348	ENSMUSG00000037348	4	133769054	133780886	72	72
3907	1	26019271	26032016	ENSMUSG00000046671	ENSMUSG00000046671	ENSMUSG00000046671	4	133797626	133807344	72	72
3908	1	25816546	25983519	ENSMUSG00000037306	ENSMUSG00000037306	ENSMUSG00000037306	4	133833766	133976366	72	72
3909	1	25742658	25767964	ENSMUSG00000037295	ENSMUSG00000037295	ENSMUSG00000037295	4	134016811	134040041	72	72
3910	1	25629998	25699287	ENSMUSG00000028826	ENSMUSG00000028826	ENSMUSG00000028826	4	134074835	134125206	72	72
3911	1	25536995	25561439	ENSMUSG00000028822	ENSMUSG00000028822	ENSMUSG00000028822	4	134169925	134186992	72	72
3912	1	25441327	25446572	ENSMUSG00000037266	ENSMUSG00000037266	ENSMUSG00000037266	4	134195699	134199445	72	72
3913	1	25421759	25431589	ENSMUSG00000028821	ENSMUSG00000028821	ENSMUSG00000028821	4	134203056	134209613	72	72
3914	1	25100107	25164062	ENSMUSG00000070691	ENSMUSG00000070691	ENSMUSG00000070691	4	134392721	134450066	72	72
3915	1	24944435	25043402	ENSMUSG00000037242	ENSMUSG00000037242	ENSMUSG00000037242	4	134486045	134544836	72	72
3916	1	24842377	24872340	ENSMUSG00000028809	ENSMUSG00000028809	ENSMUSG00000028809	4	134592567	134625221	72	72
3917	1	24755189	24808403	ENSMUSG00000043924	ENSMUSG00000043924	ENSMUSG00000043924	4	134641971	134670297	72	72
3918	1	24701974	24736093	ENSMUSG00000059713	ENSMUSG00000059713	ENSMUSG00000059713	4	134684384	134697497	72	72
3919	1	24618625	24671985	ENSMUSG00000028803	ENSMUSG00000028803	ENSMUSG00000028803	4	134717496	134763113	72	72
3920	1	24556076	24614174	ENSMUSG00000028801	ENSMUSG00000028801	ENSMUSG00000028801	4	134768063	134809881	72	72
3921	1	24518502	24563555	ENSMUSG00000037188	ENSMUSG00000037188	ENSMUSG00000037188	4	134814735	134833445	72	72
3922	1	24353234	24386338	ENSMUSG00000062157	ENSMUSG00000062157	ENSMUSG00000062157	4	134958600	134980257	72	72
3923	1	24318848	24342198	ENSMUSG00000037157	ENSMUSG00000037157	ENSMUSG00000037157	4	135000248	135024216	72	72
3924	1	24256441	24311252	ENSMUSG00000037139	ENSMUSG00000037139	ENSMUSG00000037139	4	135037677	135086646	72	72
3925	1	24158889	24162539	ENSMUSG00000028675	ENSMUSG00000028675	ENSMUSG00000028675	4	135143005	135145903	72	72
3926	1	24069603	24112439	ENSMUSG00000062585	ENSMUSG00000062585	ENSMUSG00000062585	4	135167470	135190954	72	72
3927	1	24044161	24067363	ENSMUSG00000028673	ENSMUSG00000028673	ENSMUSG00000028673	4	135192833	135212389	72	72
3928	1	24000909	24024493	ENSMUSG00000028672	ENSMUSG00000028672	ENSMUSG00000028672	4	135218541	135234684	72	72
3929	1	23994676	23999881	ENSMUSG00000028671	ENSMUSG00000028671	ENSMUSG00000028671	4	135235803	135240254	72	72
3930	1	23977470	23987307	ENSMUSG00000028669	ENSMUSG00000028669	ENSMUSG00000028669	4	135247681	135259321	72	72
3931	1	23890856	23895502	ENSMUSG00000059291	ENSMUSG00000059291	ENSMUSG00000059291	4	135321807	135325469	72	72
3932	1	23756996	23758872	ENSMUSG00000007872	ENSMUSG00000007872	ENSMUSG00000007872	4	135415900	135417466	72	72
3933	1	23627642	23683259	ENSMUSG00000036995	ENSMUSG00000036995	ENSMUSG00000036995	4	135478500	135516985	72	72
3934	1	23580141	23623820	ENSMUSG00000001604	ENSMUSG00000001604	ENSMUSG00000001604	4	135520028	135546974	72	72
3935	1	23558528	23568522	ENSMUSG00000051351	ENSMUSG00000051351	ENSMUSG00000051351	4	135558145	135566018	72	72
3936	1	23508863	23543390	ENSMUSG00000066037	ENSMUSG00000066037	ENSMUSG00000066037	4	135583048	135612748	72	72
3937	1	23389580	23393342	ENSMUSG00000070687	ENSMUSG00000070687	ENSMUSG00000070687	4	135695602	135715840	72	72
3938	1	23284038	23308210	ENSMUSG00000001089	ENSMUSG00000001089	ENSMUSG00000001089	4	135812430	135821391	72	72
3939	1	23218573	23282768	ENSMUSG00000036940	ENSMUSG00000036940	ENSMUSG00000036940	4	135822620	135874786	72	72
3940	1	22910045	23114405	ENSMUSG00000028664	ENSMUSG00000028664	ENSMUSG00000028664	4	135925688	136108032	72	72
3941	1	22852061	22860618	ENSMUSG00000036905	ENSMUSG00000036905	ENSMUSG00000036905	4	136152221	136158253	72	72
3942	1	22842710	22847190	ENSMUSG00000036896	ENSMUSG00000036896	ENSMUSG00000036896	4	136161885	136164970	72	72
3943	1	22762591	22802674	ENSMUSG00000028661	ENSMUSG00000028661	ENSMUSG00000028661	4	136201495	136228894	72	72
3944	1	22650947	22730234	ENSMUSG00000060862	ENSMUSG00000060862	ENSMUSG00000060862	4	136251808	136320771	72	72
3945	1	22318177	22342038	ENSMUSG00000036856	ENSMUSG00000036856	ENSMUSG00000036856	4	136549711	136568855	72	72
3946	1	22176005	22211620	ENSMUSG00000023433	ENSMUSG00000023433	ENSMUSG00000023433	4	136693075	136702616	72	72
3947	1	22021325	22136377	ENSMUSG00000028763	ENSMUSG00000028763	ENSMUSG00000028763	4	136740938	136842705	72	72
3948	1	22011345	22024301	ENSMUSG00000070666	ENSMUSG00000070666	ENSMUSG00000070666	4	136842952	136846719	72	72
3949	1	21877659	21982686	ENSMUSG00000043411	ENSMUSG00000043411	ENSMUSG00000043411	4	136866378	136927327	72	72
3950	1	21795302	21868381	ENSMUSG00000041351	ENSMUSG00000041351	ENSMUSG00000041351	4	136936844	137001936	72	72
3951	1	21708452	21777492	ENSMUSG00000028766	ENSMUSG00000028766	ENSMUSG00000028766	4	137013809	137068395	72	72
3952	1	21418939	21489494	ENSMUSG00000057530	ENSMUSG00000057530	ENSMUSG00000057530	4	137185658	137237301	72	72
3953	1	21005563	21250088	ENSMUSG00000028760	ENSMUSG00000028760	ENSMUSG00000028760	4	137265546	137478564	72	72
3954	1	20941766	20985768	ENSMUSG00000028759	ENSMUSG00000028759	ENSMUSG00000028759	4	137488711	137515224	72	72
3955	1	20918812	20927166	ENSMUSG00000045349	ENSMUSG00000045349	ENSMUSG00000045349	4	137522536	137532231	72	72
3956	1	20863096	20917097	ENSMUSG00000028758	ENSMUSG00000028758	ENSMUSG00000028758	4	137534327	137573568	72	72
3957	1	20850857	20860587	ENSMUSG00000028757	ENSMUSG00000028757	ENSMUSG00000028757	4	137576814	137584687	72	72
3958	1	20832535	20850591	ENSMUSG00000028756	ENSMUSG00000028756	ENSMUSG00000028756	4	137585488	137598350	72	72
3959	1	20788028	20817988	ENSMUSG00000028755	ENSMUSG00000028755	ENSMUSG00000028755	4	137610606	137640036	72	72
3960	1	20698530	20707241	ENSMUSG00000041241	ENSMUSG00000041241	ENSMUSG00000041241	4	137706748	137714341	72	72
3961	1	20681471	20685300	ENSMUSG00000046447	ENSMUSG00000046447	ENSMUSG00000046447	4	137727262	137730219	72	72
3962	1	20509682	20553343	ENSMUSG00000028753	ENSMUSG00000028753	ENSMUSG00000028753	4	137841163	137896088	72	72
3963	1	20385165	20395128	ENSMUSG00000043621	ENSMUSG00000043621	ENSMUSG00000043621	4	137990614	138008275	72	72
3964	1	20363071	20374247	ENSMUSG00000028750	ENSMUSG00000028750	ENSMUSG00000028750	4	137997804	138016649	72	72
3965	1	20338406	20349466	ENSMUSG00000028749	ENSMUSG00000028749	ENSMUSG00000028749	4	138022609	138029663	72	72
3966	1	20311019	20318637	ENSMUSG00000041202	ENSMUSG00000041202	ENSMUSG00000041202	4	138047811	138054219	72	72
3967	1	20269288	20290248	ENSMUSG00000041193	ENSMUSG00000041193	ENSMUSG00000041193	4	138071320	138135541	72	72
3968	1	20174518	20179496	ENSMUSG00000058908	ENSMUSG00000058908	ENSMUSG00000058908	4	138103952	138107263	72	72
3969	1	20119089	20122697	ENSMUSG00000028751	ENSMUSG00000028751	ENSMUSG00000028751	4	138150018	138154895	72	72
3970	1	20081498	20112016	ENSMUSG00000041161	ENSMUSG00000041161	ENSMUSG00000041161	4	138167817	138170214	72	72
3971	1	20013109	20014358	ENSMUSG00000070661	ENSMUSG00000070661	ENSMUSG00000070661	4	138239193	138240436	72	72
3972	1	19881293	19998997	ENSMUSG00000041143	ENSMUSG00000041143	ENSMUSG00000041143	4	138244968	138331242	72	72
3973	1	19864367	19878642	ENSMUSG00000028747	ENSMUSG00000028747	ENSMUSG00000028747	4	138333485	138346865	72	72
3974	1	19842313	19857532	ENSMUSG00000041120	ENSMUSG00000041120	ENSMUSG00000041120	4	138354368	138357725	72	72
3975	1	19796054	19827761	ENSMUSG00000050608	ENSMUSG00000050608	ENSMUSG00000050608	4	138377060	138403061	72	72
3976	1	19537859	19683360	ENSMUSG00000028745	ENSMUSG00000028745	ENSMUSG00000028745	4	138465000	138563834	72	72
3977	1	19511407	19533562	ENSMUSG00000028744	ENSMUSG00000028744	ENSMUSG00000028744	4	138566106	138582761	72	72
3978	1	19273587	19409333	ENSMUSG00000066036	ENSMUSG00000066036	ENSMUSG00000066036	4	138652763	138761304	72	72
3979	1	19103362	19154930	ENSMUSG00000041025	ENSMUSG00000041025	ENSMUSG00000041025	4	138803765	138889146	72	72
3980	1	19070513	19101862	ENSMUSG00000028737	ENSMUSG00000028737	ENSMUSG00000028737	4	138894970	138921757	72	72
3981	1	19038680	19058763	ENSMUSG00000028738	ENSMUSG00000028738	ENSMUSG00000028738	4	138925614	138942360	72	72
3982	1	18830087	18935219	ENSMUSG00000028736	ENSMUSG00000028736	ENSMUSG00000028736	4	139010410	139105044	72	72
3983	1	18306827	18577563	ENSMUSG00000040972	ENSMUSG00000040972	ENSMUSG00000040972	4	139298928	139518860	72	72
3984	1	17738917	17896956	ENSMUSG00000040964	ENSMUSG00000040964	ENSMUSG00000040964	4	139786561	139920826	72	72
3985	1	17605843	17638807	ENSMUSG00000040945	ENSMUSG00000040945	ENSMUSG00000040945	4	139972617	139993493	72	72
3986	1	17571278	17600782	ENSMUSG00000040935	ENSMUSG00000040935	ENSMUSG00000040935	4	139999436	140014714	72	72
3987	1	17507279	17563086	ENSMUSG00000025330	ENSMUSG00000025330	ENSMUSG00000025330	4	140017941	140046261	72	72
3988	1	17448180	17483315	ENSMUSG00000025328	ENSMUSG00000025328	ENSMUSG00000025328	4	140057445	140082724	72	72
3989	1	17404208	17445087	ENSMUSG00000025329	ENSMUSG00000025329	ENSMUSG00000025329	4	140085057	140117854	72	72
3990	1	17265843	17318517	ENSMUSG00000028927	ENSMUSG00000028927	ENSMUSG00000028927	4	140178494	140224656	72	72
3991	1	17217804	17253252	ENSMUSG00000009863	ENSMUSG00000009863	ENSMUSG00000009863	4	140233347	140251271	72	72
3992	1	17185040	17210854	ENSMUSG00000036622	ENSMUSG00000036622	ENSMUSG00000036622	4	140258976	140279405	72	72
3993	1	17173584	17179917	ENSMUSG00000060572	ENSMUSG00000060572	ENSMUSG00000060572	4	140282779	140288045	72	72
3994	1	17121032	17172046	ENSMUSG00000040860	ENSMUSG00000040860	ENSMUSG00000040860	4	140288713	140325737	72	72
3995	1	16639806	16659159	ENSMUSG00000028923	ENSMUSG00000028923	ENSMUSG00000028923	4	140338593	140350409	72	72
3996	1	16597850	16630453	ENSMUSG00000045004	ENSMUSG00000045004	ENSMUSG00000045004	4	140360421	140384835	72	72
3997	1	16566325	16597224	ENSMUSG00000040842	ENSMUSG00000040842	ENSMUSG00000040842	4	140385077	140411842	72	72
3998	1	16445921	16551536	ENSMUSG00000028920	ENSMUSG00000028920	ENSMUSG00000028920	4	140420007	140476142	72	72
3999	1	16430782	16436178	ENSMUSG00000073733	ENSMUSG00000073733	ENSMUSG00000073733	4	140486437	140492162	72	72
4000	1	16397186	16411691	ENSMUSG00000028919	ENSMUSG00000028919	ENSMUSG00000028919	4	140514960	140529638	72	72
4001	1	16323419	16355169	ENSMUSG00000006445	ENSMUSG00000006445	ENSMUSG00000006445	4	140573316	140601454	72	72
4002	1	16256851	16272708	ENSMUSG00000006218	ENSMUSG00000006218	ENSMUSG00000006218	4	140651741	140655723	72	72
4003	1	16213110	16217872	ENSMUSG00000006221	ENSMUSG00000006221	ENSMUSG00000006221	4	140693861	140697386	72	72
4004	1	16203318	16207889	ENSMUSG00000070637	ENSMUSG00000070637	ENSMUSG00000070637	4	140704979	140708181	72	72
4005	1	16140953	16175191	ENSMUSG00000006215	ENSMUSG00000006215	ENSMUSG00000006215	4	140716760	140740006	72	72
4006	1	16046946	16139542	ENSMUSG00000040761	ENSMUSG00000040761	ENSMUSG00000040761	4	140740934	140810322	72	72
4007	1	15955741	15985676	ENSMUSG00000006219	ENSMUSG00000006219	ENSMUSG00000006219	4	140848801	140870300	72	72
4008	1	15941504	15947064	ENSMUSG00000043085	ENSMUSG00000043085	ENSMUSG00000043085	4	140886309	140890709	72	72
4009	1	15935396	15940471	ENSMUSG00000040740	ENSMUSG00000040740	ENSMUSG00000040740	4	140890900	140895910	72	72
4010	1	15883414	15933851	ENSMUSG00000028917	ENSMUSG00000028917	ENSMUSG00000028917	4	140898646	140936203	72	72
4011	1	15858951	15860804	ENSMUSG00000040715	ENSMUSG00000040715	ENSMUSG00000040715	4	140955639	140995495	72	72
4012	1	15771435	15784192	ENSMUSG00000040706	ENSMUSG00000040706	ENSMUSG00000040706	4	141018886	141031144	72	72
4013	1	15725895	15770815	ENSMUSG00000040697	ENSMUSG00000040697	ENSMUSG00000040697	4	141032266	141062967	72	72
4014	1	15691352	15723377	ENSMUSG00000028914	ENSMUSG00000028914	ENSMUSG00000028914	4	141065688	141088051	72	72
4015	1	15655811	15690444	ENSMUSG00000058579	ENSMUSG00000058579	ENSMUSG00000058579	4	141087038	141098236	72	72
4016	1	15637525	15645740	ENSMUSG00000062478	ENSMUSG00000062478	ENSMUSG00000062478	4	141110729	141118344	72	72
4017	1	15435808	15548149	ENSMUSG00000051435	ENSMUSG00000051435	ENSMUSG00000051435	4	141234949	141287158	72	72
4018	1	15351649	15419422	ENSMUSG00000040616	ENSMUSG00000040616	ENSMUSG00000040616	4	141303069	141356380	72	72
4019	1	14797787	15317125	ENSMUSG00000040606	ENSMUSG00000040606	ENSMUSG00000040606	4	141374467	141482288	72	72
4020	1	13903937	14024162	ENSMUSG00000057637	ENSMUSG00000057637	ENSMUSG00000057637	4	142398399	142447681	72	72
4021	1	13782839	13817035	ENSMUSG00000028583	ENSMUSG00000028583	ENSMUSG00000028583	4	142534123	142566141	72	72
4022	1	13674032	13713130	ENSMUSG00000028584	ENSMUSG00000028584	ENSMUSG00000028584	4	142616466	142637603	72	72
4023	1	12728750	12746434	ENSMUSG00000028589	ENSMUSG00000028589	ENSMUSG00000028589	4	143684878	143705459	72	72
4024	1	12550526	12600381	ENSMUSG00000066026	ENSMUSG00000066026	ENSMUSG00000066026	4	144159764	144194896	72	72
4025	1	12212700	12494683	ENSMUSG00000020220	ENSMUSG00000020220	ENSMUSG00000020220	4	144241827	144457279	72	72
4026	1	12149647	12191872	ENSMUSG00000028599	ENSMUSG00000028599	ENSMUSG00000028599	4	144479055	144513557	72	72
4027	1	12046021	12126851	ENSMUSG00000028602	ENSMUSG00000028602	ENSMUSG00000028602	4	144535663	144581834	72	72
4028	1	12002099	12014693	ENSMUSG00000029022	ENSMUSG00000029022	ENSMUSG00000029022	4	146704592	146712301	72	72
4029	1	11962956	11996152	ENSMUSG00000029020	ENSMUSG00000029020	ENSMUSG00000029020	4	146717399	146748504	72	72
4030	1	11917333	11958181	ENSMUSG00000019055	ENSMUSG00000019055	ENSMUSG00000019055	4	146753861	146780504	72	72
4031	1	11840108	11841575	ENSMUSG00000029019	ENSMUSG00000029019	ENSMUSG00000029019	4	146829588	146831005	72	72
4032	1	11828353	11830427	ENSMUSG00000041616	ENSMUSG00000041616	ENSMUSG00000041616	4	146844522	146845879	72	72
4033	1	11788904	11821694	ENSMUSG00000029016	ENSMUSG00000029016	ENSMUSG00000029016	4	146850284	146882567	72	72
4034	1	11770938	11788057	ENSMUSG00000029009	ENSMUSG00000029009	ENSMUSG00000029009	4	146885012	146903353	72	72
4035	1	11674373	11708501	ENSMUSG00000029005	ENSMUSG00000029005	ENSMUSG00000029005	4	146942238	146974479	72	72
4036	1	11657132	11674294	ENSMUSG00000029003	ENSMUSG00000029003	ENSMUSG00000029003	4	146974720	146989478	72	72
4037	1	11646768	11656998	ENSMUSG00000055401	ENSMUSG00000055401	ENSMUSG00000055401	4	146989587	146995624	72	72
4038	1	11637525	11644035	ENSMUSG00000029001	ENSMUSG00000029001	ENSMUSG00000029001	4	146996601	147003680	72	72
4039	1	11631011	11637326	ENSMUSG00000041556	ENSMUSG00000041556	ENSMUSG00000041556	4	147004468	147010220	72	72
4040	1	11483910	11519327	ENSMUSG00000041544	ENSMUSG00000041544	ENSMUSG00000041544	4	147096576	147131697	72	72
4041	1	11255850	11271078	ENSMUSG00000047719	ENSMUSG00000047719	ENSMUSG00000047719	4	147278297	147288551	72	72
4042	1	11089180	11245176	ENSMUSG00000028991	ENSMUSG00000028991	ENSMUSG00000028991	4	147292411	147401483	72	72
4043	1	11171985	11178625	ENSMUSG00000028989	ENSMUSG00000028989	ENSMUSG00000028989	4	147338983	147344249	72	72
4044	1	11049262	11082525	ENSMUSG00000017264	ENSMUSG00000017264	ENSMUSG00000017264	4	147402258	147426203	72	72
4045	1	11037236	11042678	ENSMUSG00000006442	ENSMUSG00000006442	ENSMUSG00000006442	4	147435319	147438419	72	72
4046	1	11009168	11029872	ENSMUSG00000028979	ENSMUSG00000028979	ENSMUSG00000028979	4	147446346	147459282	72	72
4047	1	10930307	10946845	ENSMUSG00000070577	ENSMUSG00000070577	ENSMUSG00000070577	4	147502801	147520096	72	72
4048	1	10629857	10779294	ENSMUSG00000028977	ENSMUSG00000028977	ENSMUSG00000028977	4	147648192	147783406	72	72
4049	1	10457531	10613402	ENSMUSG00000028975	ENSMUSG00000028975	ENSMUSG00000028975	4	147804335	147943676	72	72
4050	1	10439166	10455170	ENSMUSG00000028974	ENSMUSG00000028974	ENSMUSG00000028974	4	147947953	147964450	72	72
4051	1	10412746	10434797	ENSMUSG00000028971	ENSMUSG00000028971	ENSMUSG00000028971	4	147968991	147970541	72	72
4052	1	10381721	10402773	ENSMUSG00000028961	ENSMUSG00000028961	ENSMUSG00000028961	4	147994444	148009884	72	72
4053	1	10214893	10364241	ENSMUSG00000063077	ENSMUSG00000063077	ENSMUSG00000063077	4	148024568	148151423	72	72
4054	1	10016241	10163884	ENSMUSG00000028960	ENSMUSG00000028960	ENSMUSG00000028960	4	148172216	148259909	72	72
4055	1	9979851	9998665	ENSMUSG00000028996	ENSMUSG00000028996	ENSMUSG00000028996	4	148293487	148298778	72	72
4056	1	9926073	9968143	ENSMUSG00000028992	ENSMUSG00000028992	ENSMUSG00000028992	4	148312409	148329096	72	72
4057	1	9911584	9925974	ENSMUSG00000028990	ENSMUSG00000028990	ENSMUSG00000028990	4	148329133	148340468	72	72
4058	1	9711671	9807171	ENSMUSG00000039953	ENSMUSG00000039953	ENSMUSG00000039953	4	148430228	148492698	72	72
4059	1	9692843	9709877	ENSMUSG00000039936	ENSMUSG00000039936	ENSMUSG00000039936	4	148492970	148542498	72	72
4060	1	9571576	9597515	ENSMUSG00000044700	ENSMUSG00000044700	ENSMUSG00000044700	4	148559183	148581834	72	72
4061	1	9275528	9352175	ENSMUSG00000039911	ENSMUSG00000039911	ENSMUSG00000039911	4	148740086	148798837	72	72
4062	1	9222490	9250540	ENSMUSG00000028980	ENSMUSG00000028980	ENSMUSG00000028980	4	148823275	148852823	72	72
4063	1	9082951	9111816	ENSMUSG00000047875	ENSMUSG00000047875	ENSMUSG00000047875	4	148931165	148948167	72	72
4064	1	9017753	9052362	ENSMUSG00000028976	ENSMUSG00000028976	ENSMUSG00000028976	4	148963144	148987968	72	72
4065	1	8985946	9008991	ENSMUSG00000062064	ENSMUSG00000062064	ENSMUSG00000062064	4	148993296	149012177	72	72
4066	1	8928533	8957090	ENSMUSG00000028972	ENSMUSG00000028972	ENSMUSG00000028972	4	149030818	149044961	72	72
4067	1	8335044	8800111	ENSMUSG00000039852	ENSMUSG00000039852	ENSMUSG00000039852	4	149250140	149463289	72	72
4068	1	8300756	8326804	ENSMUSG00000039838	ENSMUSG00000039838	ENSMUSG00000039838	4	149472372	149495965	72	72
4069	1	7994366	8008955	ENSMUSG00000028967	ENSMUSG00000028967	ENSMUSG00000028967	4	149697719	149712687	72	72
4070	1	7944310	7967926	ENSMUSG00000028964	ENSMUSG00000028964	ENSMUSG00000028964	4	149740942	149753650	72	72
4071	1	7902494	7923513	ENSMUSG00000028965	ENSMUSG00000028965	ENSMUSG00000028965	4	149763990	149789893	72	72
4072	1	7825731	7836161	ENSMUSG00000028963	ENSMUSG00000028963	ENSMUSG00000028963	4	149840897	149845610	72	72
4073	1	7767301	7827824	ENSMUSG00000028957	ENSMUSG00000028957	ENSMUSG00000028957	4	149847452	149888465	72	72
4074	1	7753916	7764079	ENSMUSG00000028955	ENSMUSG00000028955	ENSMUSG00000028955	4	149891105	149901753	72	72
4075	1	6767971	7752351	ENSMUSG00000014592	ENSMUSG00000014592	ENSMUSG00000014592	4	149906623	150007772	72	72
4076	1	6616815	6684571	ENSMUSG00000039768	ENSMUSG00000039768	ENSMUSG00000039768	4	150777539	150825759	72	72
4077	1	6607513	6616212	ENSMUSG00000039759	ENSMUSG00000039759	ENSMUSG00000039759	4	150826439	150832797	72	72
4078	1	6596332	6606680	ENSMUSG00000047777	ENSMUSG00000047777	ENSMUSG00000047777	4	150833432	150839664	72	72
4079	1	6573371	6585516	ENSMUSG00000073700	ENSMUSG00000073700	ENSMUSG00000073700	4	150852691	150861477	72	72
4080	1	6562695	6571927	ENSMUSG00000028952	ENSMUSG00000028952	ENSMUSG00000028952	4	150863574	150871465	72	72
4081	1	6537828	6562404	ENSMUSG00000028950	ENSMUSG00000028950	ENSMUSG00000028950	4	150871714	150882368	72	72
4082	1	6503994	6537182	ENSMUSG00000028948	ENSMUSG00000028948	ENSMUSG00000028948	4	150883155	150903824	72	72
4083	1	6448739	6502656	ENSMUSG00000039713	ENSMUSG00000039713	ENSMUSG00000039713	4	150940519	150959200	72	72
4084	1	6397879	6402566	ENSMUSG00000028940	ENSMUSG00000028940	ENSMUSG00000028940	4	151002667	151006269	72	72
4085	1	6231443	6243622	ENSMUSG00000042804	ENSMUSG00000042804	ENSMUSG00000042804	4	151122810	151129141	72	72
4086	1	6226232	6228682	ENSMUSG00000028946	ENSMUSG00000028946	ENSMUSG00000028946	4	151129772	151131860	72	72
4087	1	6203840	6218587	ENSMUSG00000039662	ENSMUSG00000039662	ENSMUSG00000039662	4	151141104	151150919	72	72
4088	1	6084440	6162770	ENSMUSG00000005045	ENSMUSG00000005045	ENSMUSG00000005045	4	151220373	151229637	72	72
4089	1	6028568	6083826	ENSMUSG00000028931	ENSMUSG00000028931	ENSMUSG00000028931	4	151234544	151321280	72	72
4090	1	5845457	5975118	ENSMUSG00000039577	ENSMUSG00000039577	ENSMUSG00000039577	4	151321942	151406982	72	72
4091	1	4614652	4743711	ENSMUSG00000039546	ENSMUSG00000039546	ENSMUSG00000039546	4	152226065	152325957	72	72
4092	1	3763705	3791853	ENSMUSG00000029027	ENSMUSG00000029027	ENSMUSG00000029027	4	152808255	152818926	72	72

4093	1	3718505	3763623	ENSMUSG00000039523	ENSMUSG00000039523	ENSMUSG00000039523	4	152819361	152851025	72	72
4094	1	3686644	3702928	ENSMUSG00000029028	ENSMUSG00000029028	ENSMUSG00000029028	4	152855603	152865312	72	72
4095	1	3658822	3678069	ENSMUSG00000039492	ENSMUSG00000039492	ENSMUSG00000039492	4	152870444	152886477	72	72
4096	1	3558944	3642625	ENSMUSG00000029026	ENSMUSG00000029026	ENSMUSG00000029026	4	152902644	152984008	72	72
4097	1	3537191	3556497	ENSMUSG00000029029	ENSMUSG00000029029	ENSMUSG00000029029	4	152986172	153000618	72	72
4098	1	3531416	3536549	ENSMUSG00000029030	ENSMUSG00000029030	ENSMUSG00000029030	4	153001285	153004484	72	72
4099	1	3396350	3517919	ENSMUSG00000057751	ENSMUSG00000057751	ENSMUSG00000057751	4	153106330	153119513	72	72
4100	1	3360850	3387537	ENSMUSG00000029032	ENSMUSG00000029032	ENSMUSG00000029032	4	153122270	153143852	72	72
4101	1	2975621	3340717	ENSMUSG00000039410	ENSMUSG00000039410	ENSMUSG00000039410	4	153164092	153374379	72	72
4102	1	2927907	2929327	ENSMUSG00000051276	ENSMUSG00000051276	ENSMUSG00000051276	4	153510344	153511477	72	72
4103	1	2511941	2554289	ENSMUSG00000058183	ENSMUSG00000058183	ENSMUSG00000058183	4	153713385	153739321	72	72
4104	1	2508118	2512766	ENSMUSG00000029059	ENSMUSG00000029059	ENSMUSG00000029059	4	153740205	153742843	72	72
4105	1	2479150	2486613	ENSMUSG00000042333	ENSMUSG00000042333	ENSMUSG00000042333	4	153766010	153771877	72	72
4106	1	2450044	2451544	ENSMUSG00000048001	ENSMUSG00000048001	ENSMUSG00000048001	4	153804723	153806171	72	72
4107	1	2429832	2447899	ENSMUSG00000029056	ENSMUSG00000029056	ENSMUSG00000029056	4	153807933	153824737	72	72
4108	1	2397614	2426824	ENSMUSG00000029055	ENSMUSG00000029055	ENSMUSG00000029055	4	153827177	153854784	72	72
4109	1	2326103	2333870	ENSMUSG00000029047	ENSMUSG00000029047	ENSMUSG00000029047	4	153910857	153915981	72	72
4110	1	2313132	2325048	ENSMUSG00000029048	ENSMUSG00000029048	ENSMUSG00000029048	4	153917912	153930097	72	72
4111	1	2242552	2313006	ENSMUSG00000029049	ENSMUSG00000029049	ENSMUSG00000029049	4	153930403	153955981	72	72
4112	1	2149994	2231418	ENSMUSG00000029050	ENSMUSG00000029050	ENSMUSG00000029050	4	153999286	154066320	72	72
4113	1	2110850	2134019	ENSMUSG00000073684	ENSMUSG00000073684	ENSMUSG00000073684	4	154093796	154100483	72	72
4114	1	1971769	2106694	ENSMUSG00000029053	ENSMUSG00000029053	ENSMUSG00000029053	4	154103920	154205191	72	72
4115	1	1940640	1952052	ENSMUSG00000029054	ENSMUSG00000029054	ENSMUSG00000029054	4	154228780	154241862	72	72
4116	1	1876877	1909932	ENSMUSG00000042233	ENSMUSG00000042233	ENSMUSG00000042233	4	154259487	154285945	72	72
4117	1	1838890	1840572	ENSMUSG00000023153	ENSMUSG00000023153	ENSMUSG00000023153	4	154312934	154314657	72	72
4118	1	1706589	1812355	ENSMUSG00000029064	ENSMUSG00000029064	ENSMUSG00000029064	4	154335170	154401618	72	72
4119	1	1672531	1701368	ENSMUSG00000029063	ENSMUSG00000029063	ENSMUSG00000029063	4	154406204	154434797	72	72
4120	1	1582802	1613943	ENSMUSG00000042202	ENSMUSG00000042202	ENSMUSG00000042202	4	154445411	154463890	72	72
4121	1	1557337	1559893	ENSMUSG00000029061	ENSMUSG00000029061	ENSMUSG00000029061	4	154494455	154497184	72	72
4122	1	1541261	1555847	ENSMUSG00000029060	ENSMUSG00000029060	ENSMUSG00000029060	4	154498555	154505152	72	72
4123	1	1466917	1500125	ENSMUSG00000029038	ENSMUSG00000029038	ENSMUSG00000029038	4	154548924	154577582	72	72
4124	1	1360914	1368125	ENSMUSG00000042116	ENSMUSG00000042116	ENSMUSG00000042116	4	154612295	154618314	72	72
4125	1	1351371	1353030	ENSMUSG00000073680	ENSMUSG00000073680	ENSMUSG00000073680	4	154626874	154629674	72	72
4126	1	1327151	1332556	ENSMUSG00000029066	ENSMUSG00000029066	ENSMUSG00000029066	4	154647444	154653775	72	72
4127	1	1311738	1324555	ENSMUSG00000029068	ENSMUSG00000029068	ENSMUSG00000029068	4	154656289	154668343	72	72
4128	1	1277935	1283786	ENSMUSG00000029070	ENSMUSG00000029070	ENSMUSG00000029070	4	154683560	154687893	72	72
4129	1	1260522	1274593	ENSMUSG00000029071	ENSMUSG00000029071	ENSMUSG00000029071	4	154691212	154703103	72	72
4130	1	1256557	1260549	ENSMUSG00000029072	ENSMUSG00000029072	ENSMUSG00000029072	4	154703073	154707162	72	72
4131	1	1236841	1249909	ENSMUSG00000029034	ENSMUSG00000029034	ENSMUSG00000029034	4	154713367	154732903	72	72
4132	1	1233810	1236920	ENSMUSG00000051557	ENSMUSG00000051557	ENSMUSG00000051557	4	154732955	154735069	72	72
4133	1	1217619	1233261	ENSMUSG00000029033	ENSMUSG00000029033	ENSMUSG00000029033	4	154735675	154751051	72	72
4134	1	1167696	1171965	ENSMUSG00000023571	ENSMUSG00000023571	ENSMUSG00000023571	4	154806118	154810429	72	72
4135	1	1157508	1160284	ENSMUSG00000050796	ENSMUSG00000050796	ENSMUSG00000050796	4	154835439	154836416	72	72
4136	1	1142151	1157244	ENSMUSG00000029076	ENSMUSG00000029076	ENSMUSG00000029076	4	154836714	154857410	72	72
4137	1	1136569	1139375	ENSMUSG00000029075	ENSMUSG00000029075	ENSMUSG00000029075	4	154857495	154860389	72	72
4138	1	1128834	1131952	ENSMUSG00000041954	ENSMUSG00000041954	ENSMUSG00000041954	4	154870122	154872689	72	72
4139	1	1099149	1123176	ENSMUSG00000029074	ENSMUSG00000029074	ENSMUSG00000029074	4	154878637	154892425	72	72
4140	1	1007061	1041324	ENSMUSG00000059939	ENSMUSG00000059939	ENSMUSG00000059939	4	154953780	154971065	72	72
4141	1	945366	981355	ENSMUSG00000041936	ENSMUSG00000041936	ENSMUSG00000041936	4	155009094	155028900	72	72
4142	7	92072175	92301148	ENSMUSG00000040274	ENSMUSG00000040274	ENSMUSG00000040274	5	3350318	3528231	73	73
4143	7	91996045	92004187	ENSMUSG00000040302	ENSMUSG00000040302	ENSMUSG00000040302	5	3589984	3602414	73	73
4144	7	91914824	91926085	ENSMUSG00000007415	ENSMUSG00000007415	ENSMUSG00000007415	5	3645975	3653923	73	73
4145	7	91713484	91866583	ENSMUSG00000040351	ENSMUSG00000040351	ENSMUSG00000040351	5	3696006	3808931	73	73
4146	7	91666219	91713350	ENSMUSG00000000600	ENSMUSG00000000600	ENSMUSG00000000600	5	3809195	3847363	73	73
4147	7	91612144	91632071	ENSMUSG00000040367	ENSMUSG00000040367	ENSMUSG00000040367	5	3851179	3872602	73	73
4148	7	91437720	91576295	ENSMUSG00000040407	ENSMUSG00000040407	ENSMUSG00000040407	5	3934296	4086210	73	73
4149	7	91579420	91601676	ENSMUSG00000001467	ENSMUSG00000001467	ENSMUSG00000001467	5	4086680	4110703	73	73
4150	7	90731719	90736059	ENSMUSG00000044674	ENSMUSG00000044674	ENSMUSG00000044674	5	4761658	4763586	73	73
4151	7	90063747	90674881	ENSMUSG00000028926	ENSMUSG00000028926	ENSMUSG00000028926	5	4809739	5386209	73	73
4152	7	89870698	89883200	ENSMUSG00000046798	ENSMUSG00000046798	ENSMUSG00000046798	5	5511115	5520879	73	73
4153	7	89722676	89777485	ENSMUSG00000040473	ENSMUSG00000040473	ENSMUSG00000040473	5	5585286	5670187	73	73
4154	7	89678936	89704865	ENSMUSG00000015653	ENSMUSG00000015653	ENSMUSG00000015653	5	5676944	5699767	73	73
4155	7	88261357	88262967	ENSMUSG00000040514	ENSMUSG00000040514	ENSMUSG00000040514	5	7310133	7317497	73	73
4156	7	87746545	87774142	ENSMUSG00000012428	ENSMUSG00000012428	ENSMUSG00000012428	5	7966478	7988219	73	73
4157	7	87672371	87687329	ENSMUSG00000003161	ENSMUSG00000003161	ENSMUSG00000003161	5	8062581	8073784	73	73
4158	7	87401638	87664383	ENSMUSG00000040537	ENSMUSG00000040537	ENSMUSG00000040537	5	8098266	8374087	73	73
4159	7	87343480	87376792	ENSMUSG00000002297	ENSMUSG00000002297	ENSMUSG00000002297	5	8403001	8428612	73	73
4160	7	87095665	87297691	ENSMUSG00000040570	ENSMUSG00000040570	ENSMUSG00000040570	5	8496340	8628958	73	73
4161	7	86869302	86942991	ENSMUSG00000042476	ENSMUSG00000042476	ENSMUSG00000042476	5	8899723	8965232	73	73
4162	7	86812947	86867045	ENSMUSG00000003623	ENSMUSG00000003623	ENSMUSG00000003623	5	8972045	9003167	73	73
4163	7	86347174	86408624	ENSMUSG00000056004	ENSMUSG00000056004	ENSMUSG00000056004	5	9272166	9486535	73	73
4164	7	86111160	86332127	ENSMUSG00000003974	ENSMUSG00000003974	ENSMUSG00000003974	5	9491242	9731358	73	73
4165	7	84462812	84589183	ENSMUSG00000040254	ENSMUSG00000040254	ENSMUSG00000040254	5	12389562	12593064	73	73
4166	7	83428426	83661845	ENSMUSG00000028883	ENSMUSG00000028883	ENSMUSG00000028883	5	13402790	13606980	73	73
4167	7	82831158	83116260	ENSMUSG00000063531	ENSMUSG00000063531	ENSMUSG00000063531	5	14031384	14258853	73	73
4168	7	82225830	82629844	ENSMUSG00000061601	ENSMUSG00000061601	ENSMUSG00000061601	5	14521228	14688402	73	73
4169	7	81417354	81910967	ENSMUSG00000040118	ENSMUSG00000040118	ENSMUSG00000040118	5	15446612	15882873	73	73
4170	7	81166258	81237357	ENSMUSG00000028864	ENSMUSG00000028864	ENSMUSG00000028864	5	16065374	16131263	73	73
4171	7	80209791	80386603	ENSMUSG00000028780	ENSMUSG00000028780	ENSMUSG00000028780	5	17086640	17242094	73	73
4172	7	80069459	80141668	ENSMUSG00000002944	ENSMUSG00000002944	ENSMUSG00000002944	5	17297546	17340718	73	73
4173	7	79602076	79686655	ENSMUSG00000057614	ENSMUSG00000057614	ENSMUSG00000057614	5	17776959	17872237	73	73
4174	7	77486568	78920572	ENSMUSG00000040003	ENSMUSG00000040003	ENSMUSG00000040003	5	18739113	20173176	73	73
4175	7	77307509	77424475	ENSMUSG00000039987	ENSMUSG00000039987	ENSMUSG00000039987	5	20273470	20325120	73	73
4176	7	77260982	77265683	ENSMUSG00000045435	ENSMUSG00000045435	ENSMUSG00000045435	5	20398042	20398694	73	73
4177	7	77163696	77246939	ENSMUSG00000039968	ENSMUSG00000039968	ENSMUSG00000039968	5	20407902	20463609	73	73
4178	7	77004351	77107324	ENSMUSG00000028771	ENSMUSG00000028771	ENSMUSG00000028771	5	20498465	20567621	73	73
4179	7	76778611	76790998	ENSMUSG00000039934	ENSMUSG00000039934	ENSMUSG00000039934	5	20698113	20803540	73	73
4180	7	76589870	76762455	ENSMUSG00000064280	ENSMUSG00000064280	ENSMUSG00000064280	5	20805119	20911602	73	73
4181	7	76663363	76667059	ENSMUSG00000039899	ENSMUSG00000039899	ENSMUSG00000039899	5	20884527	20890197	73	73
4182	7	76588938	76589369	ENSMUSG00000047221	ENSMUSG00000047221	ENSMUSG00000047221	5	20936992	20993948	73	73
4183	7	102240916	102502251	ENSMUSG00000048520	ENSMUSG00000048520	ENSMUSG00000048520	5	20995672	21157429	74	74
4184	7	102340688	102372632	ENSMUSG00000039883	ENSMUSG00000039883	ENSMUSG00000039883	5	21055387	21087722	74	74
4185	7	102527467	102576749	ENSMUSG00000044968	ENSMUSG00000044968	ENSMUSG00000044968	5	21174727	21213169	74	74
4186	7	102725143	102740077	ENSMUSG00000029017	ENSMUSG00000029017	ENSMUSG00000029017	5	21248984	21268976	74	74
4187	7	102740237	102772485	ENSMUSG00000029014	ENSMUSG00000029014	ENSMUSG00000029014	5	21269106	21297016	74	74
4188	7	102775325	102795891	ENSMUSG00000028932	ENSMUSG00000028932	ENSMUSG00000028932	5	21297107	21315608	74	74
4189	7	102801895	102873834	ENSMUSG00000029015	ENSMUSG00000029015	ENSMUSG00000029015	5	21322479	21377428	74	74
4190	7	102900495	103417039	ENSMUSG00000042453	ENSMUSG00000042453	ENSMUSG00000042453	5	21397297	21856514	74	74
4191	7	103554026	103635731	ENSMUSG00000029012	ENSMUSG00000029012	ENSMUSG00000029012	5	21998314	22062182	74	74
4192	7	103756464	104165503	ENSMUSG00000058361	ENSMUSG00000058361	ENSMUSG00000058361	5	22258047	22614498	74	74
4193	7	104441873	104542044	ENSMUSG00000029004	ENSMUSG00000029004	ENSMUSG00000029004	5	22962090	23014868	74	74
4194	7	104544060	104816577	ENSMUSG00000062604	ENSMUSG00000062604	ENSMUSG00000062604	5	23019360	23077091	74	74
4195	7	104884194	104949921	ENSMUSG00000057541	ENSMUSG00000057541	ENSMUSG00000057541	5	23252735	23293730	74	74
4196	7	104959768	104995360	ENSMUSG00000028999	ENSMUSG00000028999	ENSMUSG00000028999	5	23297809	23330439	74	74
4197	7	22818782	22828946	ENSMUSG00000028998	ENSMUSG00000028998	ENSMUSG00000028998	5	23349719	23354188	75	75
4198	7	22949785	23020273	ENSMUSG00000028995	ENSMUSG00000028995	ENSMUSG00000028995	5	23471899	23540687	75	75
4199	7	23111944	23181561	ENSMUSG00000028986	ENSMUSG00000028986	ENSMUSG00000028986	5	23610670	23670850	75	75
4200	7	23188011	23207149	ENSMUSG00000048439	ENSMUSG00000048439	ENSMUSG00000048439	5	23675028	23694073	75	75
4201	7	150272982	150306121	ENSMUSG00000038319	ENSMUSG00000038319	ENSMUSG00000038319	5	23829654	23861669	76	76
4202	7	150319080	150342608	ENSMUSG00000028978	ENSMUSG00000028978	ENSMUSG00000028978	5	23874884	23894536	76	76
4203	7	150340268	150352519	ENSMUSG00000038295	ENSMUSG00000038295	ENSMUSG00000038295	5	23894246	23902208	76	76
4204	7	150356470	150373582	ENSMUSG00000028973	ENSMUSG00000028973	ENSMUSG00000028973	5	23904220	23920012	76	76
4205	7	150381832	150385929	ENSMUSG00000028969	ENSMUSG00000028969	ENSMUSG00000028969	5	23928307	23933595	76	76
4206	7	150386232	150404546	ENSMUSG00000028962	ENSMUSG00000028962	ENSMUSG00000028962	5	23935384	23951010	76	76
4207	7	150404641	150408884	ENSMUSG00000028959	ENSMUSG00000028959	ENSMUSG00000028959	5	23951105	23955300	76	76
4208	7	150409106	150411553	ENSMUSG00000028958	ENSMUSG00000028958	ENSMUSG00000028958	5	23955528	23957870	76	76
4209	7	150414867	150471963	ENSMUSG00000023353	ENSMUSG00000023353	ENSMUSG00000023353	5	23962242	24012112	76	76
4210	7	150476609	150477028	ENSMUSG00000067724	ENSMUSG00000067724	ENSMUSG00000067724	5	24014654	24037267	76	76
4211	7	150503718	150515852	ENSMUSG00000038204	ENSMUSG00000038204	ENSMUSG00000038204	5	24043719	24050414	76	76
4212	7	150519000	150520849	ENSMUSG00000038199	ENSMUSG00000038199	ENSMUSG00000038199	5	24053497	24058379	76	76
4213	7	150535856	150555250	ENSMUSG00000028953	ENSMUSG00000028953	ENSMUSG00000028953	5	24075408	24087433	76	76
4214	7	150560518	150566838	ENSMUSG00000038181	ENSMUSG00000038181	ENSMUSG00000038181	5	24096806	24102429	76	76
4215	7	150566995	150576986	ENSMUSG00000028949	ENSMUSG00000028949	ENSMUSG00000028949	5	24102695	24112077	76	76
4216	7	150669832	150706463	ENSMUSG00000028954	ENSMUSG00000028954	ENSMUSG00000028954	5	24195880	24220441	76	76
4217	7	150709147	150738673	ENSMUSG00000055235	ENSMUSG00000055235	ENSMUSG00000055235	5	24222334	24240745	76	76
4218	7	150757990	150768032	ENSMUSG00000038135	ENSMUSG00000038135	ENSMUSG00000038135	5	24261068	24267908	76	76
4219	7	150794032	150847940	ENSMUSG00000028945	ENSMUSG00000028945	ENSMUSG00000028945	5	24313729	24327900	76	76
4220	7	150884960	151204728	ENSMUSG00000028944	ENSMUSG00000028944	ENSMUSG00000028944	5	24372822	24610495	76	76
4221	7	151284444	151347946	ENSMUSG00000028938	ENSMUSG00000028938	ENSMUSG00000028938	5	24691545	24730354	76	76
4222	7	151353797	151450357	ENSMUSG00000038072	ENSMUSG00000038072	ENSMUSG00000038072	5	24732958	24775983	76	76
4223	7	151464789	151763984	ENSMUSG00000038056	ENSMUSG00000038056	ENSMUSG00000038056	5	24783702	25008767	76	76
4224	7	151974522	152004183	ENSMUSG00000028933	ENSMUSG00000028933	ENSMUSG00000028933	5	25199877	25215862	76	76
4225	7	153380839	154315123	ENSMUSG00000061576	ENSMUSG00000061576	ENSMUSG00000061576	5	27380376	28056538	76	76
4226	7	154366334	154425591	ENSMUSG00000002221	ENSMUSG00000002221	ENSMUSG00000002221	5	28074889	28122066	76	76
4227	7	154492967	154508392	ENSMUSG00000039106	ENSMUSG00000039106	ENSMUSG00000039106	5	28172734	28185873	76	76
4228	7	154720476	154732877	ENSMUSG00000045294	ENSMUSG00000045294	ENSMUSG00000045294	5	28402154	28409448	76	76
4229	7	154942176	154950263	ENSMUSG00000039095	ENSMUSG00000039095	ENSMUSG00000039095	5	28497267	28502960	76	76
4230	7	154649830	154994496	ENSMUSG00000044681	ENSMUSG00000044681	ENSMUSG00000044681	5	28536250	28568660	76	76
4231	7	155158335	155231173	ENSMUSG00000048271	ENSMUSG00000048271	ENSMUSG00000048271	5	28718616	28746891	76	76
4232	7	155288319	155297728	ENSMUSG00000002633	ENSMUSG00000002633	ENSMUSG00000002633	5	28787602	28797888	76	76
4233	7	156126162	156162579	ENSMUSG00000029130	ENSMUSG00000029130	ENSMUSG00000029130	5	29529361	29556078	76	76
4234	7	156167667	156378442	ENSMUSG00000010721	ENSMUSG00000010721	ENSMUSG00000010721	5	29562906	29709089	76	76
4235	7	156435178	156458634	ENSMUSG00000001569	ENSMUSG00000001569	ENSMUSG00000001569	5	29765451	29782262	76	76
4236	7	156490326	156495805	ENSMUSG00000001566	ENSMUSG00000001566	ENSMUSG00000001566	5	29804656	29809062	76	76
4237	7	156624425	156754823	ENSMUSG00000039000	ENSMUSG00000039000	ENSMUSG00000039000	5	29900029	30006864	76	76
4238	7	22732028	22738091	ENSMUSG00000025746	ENSMUSG00000025746	ENSMUSG00000025746	5	30343948	30350755	Singleton	
4239	2	26249464	26266017	ENSMUSG00000044576	ENSMUSG00000044576	ENSMUSG00000044576	5	30439047	30449157	77	77
4240	2	26267008	26321098	ENSMUSG00000025745	ENSMUSG00000025745	ENSMUSG00000025745	5	30449091	30485767	77	77
4241	2	26386316	26395421	ENSMUSG00000067642	ENSMUSG00000067642	ENSMUSG00000067642	5	30524211	30536509	77	77
4242	2	26422605	26465439	ENSMUSG00000075703	ENSMUSG00000075703	ENSMUSG00000075703	5	30563557	30597564	77	77
4243	2	26478288	26533083	ENSMUSG00000073102	ENSMUSG00000073102	ENSMUSG00000073102	5	30611997	30612155	77	77
4244	2	26533575	26635070	ENSMUSG00000062372	ENSMUSG00000062372	ENSMUSG00000062372	5	30646678	30738513	77	77
4245	2	26638985	26655810	ENSMUSG00000029182	ENSMUSG00000029182	ENSMUSG00000029182	5	30742655	30760668	77	77
4246	2	26657577	26717715	ENSMUSG00000053194	ENSMUSG00000053194	ENSMUSG00000053194	5	30762165	30822417	77	77
4247	2	26769123	26806207	ENSMUSG00000049265	ENSMUSG00000049265	ENSMUSG00000049265	5	30864668	30900062	77	77
4248	2	26840660	26857603	ENSMUSG00000029175	ENSMUSG00000029175	ENSMUSG00000029175	5	30924544	30936310	77	77
4249	2	26974872	27023801	ENSMUSG00000029168	ENSMUSG00000029168	ENSMUSG00000029168	5	30988476	31075950	77	77
4250	2	27046984	27103568	ENSMUSG00000029166	ENSMUSG00000029166	ENSMUSG00000029166	5	31091337	31142687	77	77
4251	2	27109342	27118040	ENSMUSG00000038828	ENSMUSG00000038828	ENSMUSG00000038828	5	31146214	31154945	77	77
4252	2	27128025	27146985	ENSMUSG00000029165	ENSMUSG00000029165	ENSMUSG00000029165	5	31165938	31180067	77	77
4253	2	27154939	27162780	ENSMUSG00000029163	ENSMUSG00000029163	ENSMUSG00000029163	5	31190287	31197850	77	77
4254	2	27163119	27177123	ENSMUSG00000029162	ENSMUSG00000029162	ENSMUSG00000029162	5	31198476	31207827	77	77
4255	2	27177646	27180752	ENSMUSG00000029161	ENSMUSG00000029161	ENSMUSG00000029161	5	31209725	31222061	77	77
4256	2	27200161	27207182	ENSMUSG00000006638	ENSMUSG00000006638	ENSMUSG00000006638	5	31226653	31231672	77	77
4257	2	27207128	27211047	ENSMUSG00000045302	ENSMUSG00000045302	ENSMUSG00000045302	5	31232224	31348848	77	77
4258	2	27225506	27229239	ENSMUSG00000006642	ENSMUSG00000006642	ENSMUSG00000006642	5	31245243	31253595	77	77
4259	2	27275959	27288658	ENSMUSG00000006641	ENSMUSG00000006641	ENSMUSG00000006641	5	31312617	31325505	77	77
4260	2	27288399	27293528	ENSMUSG00000013622	ENSMUSG00000013622	ENSMUSG00000013622	5	31325248	31331204	77	77
4261	2	27293762	27320164	ENSMUSG00000013629	ENSMUSG00000013629	ENSMUSG00000013629	5	31331392	31355060	77	77
4262	2	27330975	27339631	ENSMUSG00000029151	ENSMUSG00000029151	ENSMUSG00000029151	5	31362688	31370066	77	77
4263	2	27351793	27357802	ENSMUSG00000053856	ENSMUSG00000053856	ENSMUSG00000053856	5	31384900	31389105	77	77
4264	2	27358834	27383811	ENSMUSG00000062077	ENSMUSG00000062077	ENSMUSG00000062077	5	31393293	31414207	77	77
4265	2	27383769	27384634	ENSMUSG00000038676	ENSMUSG00000038676	ENSMUSG00000038676	5	31414570	31415476	77	77
4266	2	27402220	27432863	ENSMUSG00000029144	ENSMUSG00000029144	ENSMUSG00000029144	5	31428171	31456725	77	77
4267	2	27440723	27446828	ENSMUSG00000029145	ENSMUSG00000029145	ENSMUSG00000029145	5	31464149	31469674	77	77
4268	2	27446893	27453499	ENSMUSG00000029146	ENSMUSG00000029146	ENSMUSG00000029146	5	31469876	31475480	77	77
4269	2	27453602	27457096	ENSMUSG00000043059	ENSMUSG00000043059	ENSMUSG00000043059	5	31475563	31478814	77	77
4270	2	27457565	27486058	ENSMUSG00000029147	ENSMUSG00000029147	ENSMUSG00000029147	5	31479249	31497126	77	77
4271	2	27504161	27518630	ENSMUSG00000029148	ENSMUSG00000029148	ENSMUSG00000029148	5	31517506	31528141	77	77
4272	2	27518752	27520668	ENSMUSG00000029149	ENSMUSG00000029149	ENSMUSG00000029149	5	31528341	31529583	77	77
4273	2	27520742	27566128	ENSMUSG00000038564	ENSMUSG00000038564	ENSMUSG00000038564	5	31529858	31567695	77	77
4274	2	27568254	27571616	ENSMUSG00000038552	ENSMUSG00000038552	ENSMUSG00000038552	5	31568827	31572458	77	77
4275	2	27573210	27600052	ENSMUSG00000059434	ENSMUSG00000059434	ENSMUSG00000059434	5	31574162	31603883	77	77
4276	2	27659397	27699460	ENSMUSG00000062761	ENSMUSG00000062761	ENSMUSG00000062761	5	31729042	31758332	77	77
4277	2	27705407	27727879	ENSMUSG00000064037	ENSMUSG00000064037	ENSMUSG00000064037	5	31771342	31788208	77	77
4278	2	27729423	27740011	ENSMUSG00000053134	ENSMUSG00000053134	ENSMUSG00000053134	5	31792245	31803312	77	77
4279	2	27739842	27771335	ENSMUSG00000029141	ENSMUSG00000029141	ENSMUSG00000029141	5	31803592	31830624	77	77
4280	2	27848088	27856112	ENSMUSG00000029142	ENSMUSG00000029142	ENSMUSG00000029142	5	31890566	31940933	77	77
4281	2	27857735	27967469	ENSMUSG00000029136	ENSMUSG00000029136	ENSMUSG00000029136	5	31901033	31974208	77	77
4282	2	27967061	28415269	ENSMUSG00000052139	ENSMUSG00000052139	ENSMUSG00000052139	5	31974631	32361320	77	77
4283	2	28469229	28493683	ENSMUSG00000029135	ENSMUSG00000029135	ENSMUSG00000029135	5	32413115	32434423	77	77
4284	2	28572486	28720117	ENSMUSG00000029134	ENSMUSG00000029134	ENSMUSG00000029134	5	32541433	32640746	77	77
4285	2	28828118	28879300	ENSMUSG00000014956	ENSMUSG00000014956	ENSMUSG00000014956	5	32735733	32794014	77	77
4286	22	30345380	30388195	ENSMUSG00000023452	ENSMUSG00000023452	ENSMUSG00000023452	5	33053158	33102482	78	78
4287	22	30407336	30464846	ENSMUSG00000054280	ENSMUSG00000054280	ENSMUSG00000054280	5	33106132	33144532	78	78

4288	22	30480069	30633000	ENSMUSG00000037426	ENSMUSG00000037426	ENSMUSG00000037426	5	33180578	33308417	78	78		
4289	22	30670447	30683587	ENSMUSG00000018965	ENSMUSG00000018965	ENSMUSG00000018965	5	33335738	33344822	78	78		
4290	22	30769259	30836645	ENSMUSG00000011034	ENSMUSG00000011034	ENSMUSG00000011034	5	33421091	33479554	78	78		
4291	4	1150720	1156617	ENSMUSG00000037379	ENSMUSG00000037379	ENSMUSG00000037379	5	33530375	33535040	79	79		
4292	4	1195228	1232908	ENSMUSG00000037373	ENSMUSG00000037373	ENSMUSG00000037373	5	33564581	33591839	79	79		
4293	4	1331880	1369749	ENSMUSG00000037355	ENSMUSG00000037355	ENSMUSG00000037355	5	33695553	33734721	79	79		
4294	4	1387002	1387181	ENSMUSG00000029112	ENSMUSG00000029112	ENSMUSG00000029112	5	33747873	33748052	79	79		
4295	4	1612836	1640266	ENSMUSG00000037339	ENSMUSG00000037339	ENSMUSG00000037339	5	33917211	33946429	79	79		
4296	4	1688708	1692362	ENSMUSG00000019295	ENSMUSG00000019295	ENSMUSG00000019295	5	33970073	33974689	79	79		
4297	4	1693062	1716693	ENSMUSG00000037313	ENSMUSG00000037313	ENSMUSG00000037313	5	33974985	33995854	79	79		
4298	4	1764832	1780390	ENSMUSG00000054252	ENSMUSG00000054252	ENSMUSG00000054252	5	34038581	34053924	79	79		
4299	4	1783004	1827772	ENSMUSG00000005299	ENSMUSG00000005299	ENSMUSG00000005299	5	34056537	34099561	79	79		
4300	4	1864307	1953728	ENSMUSG00000057406	ENSMUSG00000057406	ENSMUSG00000057406	5	34159998	34212031	79	79		
4301	4	1954248	1981635	ENSMUSG00000029111	ENSMUSG00000029111	ENSMUSG00000029111	5	34214779	34253179	79	79		
4302	4	2013352	2015493	ENSMUSG00000070858	ENSMUSG00000070858	ENSMUSG00000070858	5	34300348	34301865	79	79		
4303	4	2043443	2200756	ENSMUSG00000045102	ENSMUSG00000045102	ENSMUSG00000045102	5	34324055	34486307	79	79		
4304	4	2218957	2233819	ENSMUSG00000037235	ENSMUSG00000037235	ENSMUSG00000037235	5	34492821	34504537	79	79		
4305	4	2241123	2390167	ENSMUSG00000037224	ENSMUSG00000037224	ENSMUSG00000037224	5	34512826	34560173	79	79		
4306	4	2440605	2487379	ENSMUSG00000029110	ENSMUSG00000029110	ENSMUSG00000029110	5	34663632	34668237	79	79		
4307	4	2596957	2704097	ENSMUSG00000037210	ENSMUSG00000037210	ENSMUSG00000037210	5	34737708	34803315	79	79		
4308	4	2713185	2727901	ENSMUSG00000059866	ENSMUSG00000059866	ENSMUSG00000059866	5	34812944	34830820	79	79		
4309	4	2764552	2805818	ENSMUSG00000054520	ENSMUSG00000054520	ENSMUSG00000054520	5	34860312	34880483	79	79		
4310	4	2815382	2901587	ENSMUSG00000029106	ENSMUSG00000029106	ENSMUSG00000029106	5	34890720	34949162	79	79		
4311	4	2902090	2906384	ENSMUSG00000001082	ENSMUSG00000001082	ENSMUSG00000001082	5	34950506	34953988	79	79		
4312	4	2909464	2934916	ENSMUSG00000036693	ENSMUSG00000036693	ENSMUSG00000036693	5	34955394	34976948	79	79		
4313	4	2935141	3012270	ENSMUSG00000052783	ENSMUSG00000052783	ENSMUSG00000052783	5	34977810	35072160	79	79		
4314	4	3046206	3215484	ENSMUSG00000029104	ENSMUSG00000029104	ENSMUSG00000029104	5	35078597	35226253	79	79		
4315	4	3220748	3235484	ENSMUSG00000051246	ENSMUSG00000051246	ENSMUSG00000051246	5	35232772	35240696	79	79		
4316	4	3413412	3421009	ENSMUSG00000029102	ENSMUSG00000029102	ENSMUSG00000029102	5	35358417	35365269	79	79		
4317	4	3444942	3472344	ENSMUSG00000044716	ENSMUSG00000044716	ENSMUSG00000044716	5	35373945	35404692	79	79		
4318	4	3484088	3503947	ENSMUSG00000029103	ENSMUSG00000029103	ENSMUSG00000029103	5	35408367	35422558	79	79		
4319	4	8645287	8672379	ENSMUSG00000036596	ENSMUSG00000036596	ENSMUSG00000036596	5	35819196	35842396	80	80		
4320	4	8493450	8528619	ENSMUSG00000029097	ENSMUSG00000029097	ENSMUSG00000029097	5	35874199	35891921	80	80		
4321	4	8419265	8493338	ENSMUSG00000029098	ENSMUSG00000029098	ENSMUSG00000029098	5	35899921	35930662	80	80		
4322	4	8322392	8359738	ENSMUSG00000029096	ENSMUSG00000029096	ENSMUSG00000029096	5	35968905	35996643	80	80		
4323	4	8251994	8293730	ENSMUSG00000036553	ENSMUSG00000036553	ENSMUSG00000036553	5	36014042	36040958	80	80		
4324	4	8018929	8211381	ENSMUSG00000029095	ENSMUSG00000029095	ENSMUSG00000029095	5	36074792	36201831	80	80		
4325	4	7811341	7924940	ENSMUSG00000029094	ENSMUSG00000029094	ENSMUSG00000029094	5	36210180	36320783	80	80		
4326	4	7245275	7792891	ENSMUSG00000029093	ENSMUSG00000029093	ENSMUSG00000029093	5	36334044	36715219	80	80		
4327	4	7112682	7120704	ENSMUSG00000029198	ENSMUSG00000029198	ENSMUSG00000029198	5	36782280	36791172	80	80		
4328	4	7096208	7107679	ENSMUSG00000029196	ENSMUSG00000029196	ENSMUSG00000029196	5	36793065	36794057	80	80		
4329	4	7093477	7095567	ENSMUSG00000050677	ENSMUSG00000050677	ENSMUSG00000050677	5	36801683	36805266	80	80		
4330	4	6962473	7085742	ENSMUSG00000029192	ENSMUSG00000029192	ENSMUSG00000029192	5	36807699	36899468	80	80		
4331	4	6835368	6936791	ENSMUSG00000029190	ENSMUSG00000029190	ENSMUSG00000029190	5	36917587	36961167	80	80		
4332	4	6768683	6770299	ENSMUSG00000060708	ENSMUSG00000060708	ENSMUSG00000060708	5	37036247	37036894	80	80		
4333	4	6627803	6675089	ENSMUSG00000029119	ENSMUSG00000029119	ENSMUSG00000029119	5	37095134	37118851	80	80		
4334	4	6373209	6525074	ENSMUSG00000029120	ENSMUSG00000029120	ENSMUSG00000029120	5	37156819	37243329	80	80		
4335	4	6322478	6355893	ENSMUSG00000039474	ENSMUSG00000039474	ENSMUSG00000039474	5	37254356	37277167	80	80		
4336	4	6106383	6253183	ENSMUSG00000063646	ENSMUSG00000063646	ENSMUSG00000063646	5	37339165	37413544	80	80		
4337	4	6009746	6041067	ENSMUSG00000070823	ENSMUSG00000070823	ENSMUSG00000070823	5	37475304	37481272	80	80		
4338	4	5873393	5945686	ENSMUSG00000029121	ENSMUSG00000029121	ENSMUSG00000029121	5	37534041	37580382	80	80		
4339	4	5763825	5866933	ENSMUSG00000029122	ENSMUSG00000029122	ENSMUSG00000029122	5	37587422	37625110	80	80		
4340	4	5615316	5761195	ENSMUSG00000050248	ENSMUSG00000050248	ENSMUSG00000050248	5	37626736	37713303	80	80		
4341	4	5104428	5553626	ENSMUSG00000029123	ENSMUSG00000029123	ENSMUSG00000029123	5	37736902	37937396	80	80		
4342	4	5067214	5072100	ENSMUSG00000062329	ENSMUSG00000062329	ENSMUSG00000062329	5	38023808	38028065	80	80		
4343	4	4912307	4916564	ENSMUSG00000048450	ENSMUSG00000048450	ENSMUSG00000048450	5	38108918	38112832	80	80		
4344	4	4471596	4594656	ENSMUSG00000029125	ENSMUSG00000029125	ENSMUSG00000029125	5	38327484	38425067	80	80		
4345	4	4400768	4471686	ENSMUSG00000029126	ENSMUSG00000029126	ENSMUSG00000029126	5	38425442	38447716	80	80		
4346	4	4342879	4374410	ENSMUSG00000029127	ENSMUSG00000029127	ENSMUSG00000029127	5	38488293	38508677	80	80		
4347	4	4320329	4342794	ENSMUSG00000067367	ENSMUSG00000067367	ENSMUSG00000067367	5	38508799	38522546	80	80		
4348	4	4288181	4300870	ENSMUSG00000067365	ENSMUSG00000067365	ENSMUSG00000067365	5	38548624	38557875	80	80		
4349	4	4241431	4279522	ENSMUSG00000051596	ENSMUSG00000051596	ENSMUSG00000051596	5	38564186	38592458	80	80		
4350	4	9392402	9394731	ENSMUSG00000039358	ENSMUSG00000039358	ENSMUSG00000039358	5	38607758	38610559	81	81		
4351	4	9436946	9632205	ENSMUSG00000005107	ENSMUSG00000005107	ENSMUSG00000005107	5	38639130	38768555	81	81		
4352	4	9685062	9727671	ENSMUSG00000005103	ENSMUSG00000005103	ENSMUSG00000005103	5	38815063	38849915	81	81		
4353	4	10050602	10068130	ENSMUSG00000046572	ENSMUSG00000046572	ENSMUSG00000046572	5	38959661	38962591	81	81		
4354	4	10100424	10295484	ENSMUSG00000039315	ENSMUSG00000039315	ENSMUSG00000039315	5	38994711	39072826	81	81		
4355	4	11009474	11040487	ENSMUSG00000051022	ENSMUSG00000051022	ENSMUSG00000051022	5	39902192	39932858	81	81		
4356	4	12978446	13095054	ENSMUSG00000029128	ENSMUSG00000029128	ENSMUSG00000029128	5	41913224	41996403	81	81		
4357	4	13151552	13155136	ENSMUSG00000049691	ENSMUSG00000049691	ENSMUSG00000049691	5	42049890	42052748	81	81		
4358	4	13179460	13238445	ENSMUSG00000061755	ENSMUSG00000061755	ENSMUSG00000061755	5	42105033	42132416	81	81		
4359	4	14614620	14680872	ENSMUSG00000039782	ENSMUSG00000039782	ENSMUSG00000039782	5	43522207	43574438	81	81		
4360	4	14950658	15056885	ENSMUSG00000061535	ENSMUSG00000061535	ENSMUSG00000061535	5	43803836	43907065	81	81		
4361	4	15086655	15212143	ENSMUSG00000039765	ENSMUSG00000039765	ENSMUSG00000039765	5	43950692	44029220	81	81		
4362	4	15215252	15266111	ENSMUSG00000039753	ENSMUSG00000039753	ENSMUSG00000039753	5	44032866	44070369	81	81		
4363	4	15313817	15343508	ENSMUSG00000029082	ENSMUSG00000029082	ENSMUSG00000029082	5	44107163	44131495	81	81		
4364	4	15388999	15460167	ENSMUSG00000029084	ENSMUSG00000029084	ENSMUSG00000029084	5	44157098	44200622	81	81		
4365	4	15546290	15549461	ENSMUSG00000048373	ENSMUSG00000048373	ENSMUSG00000048373	5	44267106	44270010	81	81		
4366	4	15578955	15686664	ENSMUSG00000029086	ENSMUSG00000029086	ENSMUSG00000029086	5	44281869	44389964	81	81		
4367	4	15773472	15837197	ENSMUSG00000046985	ENSMUSG00000046985	ENSMUSG00000046985	5	44463407	44514854	81	81		
4368	4	16112262	16509425	ENSMUSG00000039706	ENSMUSG00000039706	ENSMUSG00000039706	5	44760927	45087826	81	81		
4369	4	17097119	17122955	ENSMUSG00000015806	ENSMUSG00000015806	ENSMUSG00000015806	5	45722300	45738429	81	81		
4370	4	17125988	17137803	ENSMUSG00000049530	ENSMUSG00000049530	ENSMUSG00000049530	5	45742059	45752212	81	81		
4371	4	17187913	17218689	ENSMUSG00000039682	ENSMUSG00000039682	ENSMUSG00000039682	5	45781657	45800920	81	81		
4372	4	17225371	17235258	ENSMUSG00000015804	ENSMUSG00000015804	ENSMUSG00000015804	5	45808500	45817532	81	81		
4373	4	17242809	17392046	ENSMUSG00000015879	ENSMUSG00000015879	ENSMUSG00000015879	5	45817953	45927556	81	81		
4374	4	17421623	17455583	ENSMUSG00000015880	ENSMUSG00000015880	ENSMUSG00000015880	5	45964163	45975673	81	81		
4375	4	17491316	17631455	ENSMUSG00000015882	ENSMUSG00000015882	ENSMUSG00000015882	5	46021704	46145746	81	81		
4376	4	19864333	20229900	ENSMUSG00000031558	ENSMUSG00000031558	ENSMUSG00000031558	5	48273041	48593759	81	81		
4377	4	20307003	20363628	ENSMUSG00000029089	ENSMUSG00000029089	ENSMUSG00000029089	5	48660578	48676987	81	81		
4378	4	20339328	21559402	ENSMUSG00000029088	ENSMUSG00000029088	ENSMUSG00000029088	5	48679157	49812949	81	81		
4379	4	21998097	22126770	ENSMUSG00000029090	ENSMUSG00000029090	ENSMUSG00000029090	5	50248204	50347244	81	81		
4380	4	22303686	22430289	ENSMUSG00000067313	ENSMUSG00000067313	ENSMUSG00000067313	5	50570263	50586785	81	81		
4381	4	23402742	23500798	ENSMUSG00000029167	ENSMUSG00000029167	ENSMUSG00000029167	5	51745885	51842109	81	81		
4382	4	24138188	24195282	ENSMUSG00000029169	ENSMUSG00000029169	ENSMUSG00000029169	5	52438464	52478736	81	81		
4383	4	24405153	24411562	ENSMUSG00000072941	ENSMUSG00000072941	ENSMUSG00000072941	5	52656189	52656991	81	81		
4384	4	24419112	24463899	ENSMUSG00000051719	ENSMUSG00000051719	ENSMUSG00000051719	5	52664496	52723983	81	81		
4385	4	24609567	24641599	ENSMUSG00000039252	ENSMUSG00000039252	ENSMUSG00000039252	5	52826112	52854551	81	81		
4386	4	24731722	24771162	ENSMUSG00000029173	ENSMUSG00000029173	ENSMUSG00000029173	5	52929429	52957955	81	81		
4387	4	24844695	24889812	ENSMUSG00000029186	ENSMUSG00000029186	ENSMUSG00000029186	5	53029839	53057592	81	81		
4388	4	24923504	24980764	ENSMUSG00000029179	ENSMUSG00000029179	ENSMUSG00000029179	5	53071315	53108013	81	81		
4389	4	24987933	25029218	ENSMUSG00000029176	ENSMUSG00000029176	ENSMUSG00000029176	5	53122383	53154982	81	81		
4390	4	25266564	25289468	ENSMUSG00000029188	ENSMUSG00000029188	ENSMUSG00000029188	5	53337605	53359909	81	81		
4391	4	25358154	25474315	ENSMUSG00000029189	ENSMUSG00000029189	ENSMUSG00000029189	5	53395333	53501700	81	81		
4392	4	25524917	25540531	ENSMUSG00000061461	ENSMUSG00000061461	ENSMUSG00000061461	5	53555418	53566787	81	81		
4393	4	25930430	26042376	ENSMUSG00000039191	ENSMUSG00000039191	ENSMUSG00000039191	5	53878598	53944383	81	81		
4394	4	26092116	26101140	ENSMUSG00000029193	ENSMUSG00000029193	ENSMUSG00000029193	5	53986735	53996350	81	81		
4395	4	26194804	26365900	ENSMUSG00000039178	ENSMUSG00000039178	ENSMUSG00000039178	5	54097927	54191973	81	81		
4396	4	26471561	26634686	ENSMUSG00000039156	ENSMUSG00000039156	ENSMUSG00000039156	5	54286771	54407954	81	81		
4397	4	30332143	30753569	ENSMUSG00000029108	ENSMUSG00000029108	ENSMUSG00000029108	5	58006337	58017692	81	81		
4398	4	35744017	35922374	ENSMUSG00000037999	ENSMUSG00000037999	ENSMUSG00000037999	5	62890685	63037911	81	81		
4399	4	35959639	36021811	ENSMUSG00000070787	ENSMUSG00000070787	ENSMUSG00000070787	5	63106759	63176489	81	81		
4400	4	37034446	37125231	ENSMUSG00000043636	ENSMUSG00000043636	ENSMUSG00000043636	5	63929369	64088668	81	81		
4401	4	37131958	37269843	ENSMUSG00000060512	ENSMUSG00000060512	ENSMUSG00000060512	5	64091499	64178754	81	81		
4402	4	37268817	37364392	ENSMUSG00000047881	ENSMUSG00000047881	ENSMUSG00000047881	5	64188662	64248001	81	81		
4403	4	37504723	37540107	ENSMUSG00000029171	ENSMUSG00000029171	ENSMUSG00000029171	5	64372085	64407313	81	81		
4404	4	37569115	37817189	ENSMUSG00000029174	ENSMUSG00000029174	ENSMUSG00000029174	5	64438586	64630615	81	81		
4405	4	38342212	38379058	ENSMUSG00000029178	ENSMUSG00000029178	ENSMUSG00000029178	5	65082658	65109264	81	81		
4406	4	38474275	38482807	ENSMUSG00000044827	ENSMUSG00000044827	ENSMUSG00000044827	5	65203969	65211834	81	81		
4407	4	38504618	38507555	ENSMUSG00000051498	ENSMUSG00000051498	ENSMUSG00000051498	5	65232241	65239170	81	81		
4408	4	38545832	38623755	ENSMUSG00000029185	ENSMUSG00000029185	ENSMUSG00000029185	5	65249210	65321006	81	81		
4409	4	38644760	38710422	ENSMUSG00000037913	ENSMUSG00000037913	ENSMUSG00000037913	5	65344264	65370690	81	81		
4410	4	38740455	38804810	ENSMUSG00000054920	ENSMUSG00000054920	ENSMUSG00000054920	5	65410366	65447111	81	81		
4411	4	38860419	38963822	ENSMUSG00000037890	ENSMUSG00000037890	ENSMUSG00000037890	5	65478848	65539552	81	81		
4412	4	38965471	39044390	ENSMUSG00000029191	ENSMUSG00000029191	ENSMUSG00000029191	5	65542198	65598665	81	81		
4413	4	39084868	39129551	ENSMUSG00000029195	ENSMUSG00000029195	ENSMUSG00000029195	5	65627546	65663138	81	81		
4414	4	39137015	39155668	ENSMUSG00000029199	ENSMUSG00000029199	ENSMUSG00000029199	5	65670688	65689558	81	81		
4415	4	39176770	39205613	ENSMUSG00000029201	ENSMUSG00000029201	ENSMUSG00000029201	5	65692354	65714977	81	81		
4416	4	39228930	39316908	ENSMUSG00000037822	ENSMUSG00000037822	ENSMUSG00000037822	5	65727699	65772073	81	81		
4417	4	39376343	39456449	ENSMUSG00000029203	ENSMUSG00000029203	ENSMUSG00000029203	5	65816406	65878063	81	81		
4418	4	39500879	39655887	ENSMUSG00000029202	ENSMUSG00000029202	ENSMUSG00000029202	5	65902020	65976062	81	81		
4419	4	39734919	39836267	ENSMUSG00000037795	ENSMUSG00000037795	ENSMUSG00000037795	5	66069151	66105919	81	81		
4420	4	39874965	39922663	ENSMUSG00000029204	ENSMUSG00000029204	ENSMUSG00000029204	5	66142036	66172579	81	81		
4421	4	40032103	40051991	ENSMUSG00000029205	ENSMUSG00000029205	ENSMUSG00000029205	5	66246583	66256379	81	81		
4422	4	40120041	40211451	ENSMUSG00000070780	ENSMUSG00000070780	ENSMUSG00000070780	5	66297781	66430064	81	81		
4423	4	40446671	40506759	ENSMUSG00000029206	ENSMUSG00000029206	ENSMUSG00000029206	5	66539260	66577151	81	81		
4424	4	40512167	40711176	ENSMUSG00000029207	ENSMUSG00000029207	ENSMUSG00000029207	5	66581023	66731440	81	81		
4425	4	40953607	40965203	ENSMUSG00000029223	ENSMUSG00000029223	ENSMUSG00000029223	5	66955376	66966364	81	81		
4426	4	41235078	41394587	ENSMUSG00000037736	ENSMUSG00000037736	ENSMUSG00000037736	5	67248208	67325938	81	81		
4427	4	41440856	41445744	ENSMUSG00000012520	ENSMUSG00000012520	ENSMUSG00000012520	5	67373532	67378261	81	81		
4428	4	41631903	41652588	ENSMUSG00000037720	ENSMUSG00000037720	ENSMUSG00000037720	5	67539787	67570596	81	81		
4429	4	41687280	41784295	ENSMUSG00000029221	ENSMUSG00000029221	ENSMUSG00000029221	5	67586092	67635277	81	81		
4430	4	41807714	41849652	ENSMUSG00000070752	ENSMUSG00000070752	ENSMUSG00000070752	5	67677371	67696924	81	81		
4431	4	42094613	42098413	ENSMUSG00000050010	ENSMUSG00000050010	ENSMUSG00000050010	5	67887543	67893112	81	81		
4432	4	42105147	42353867	ENSMUSG00000037685	ENSMUSG00000037685	ENSMUSG00000037685	5	67901734	68093859	81	81		
4433	4	43870683	44145581	ENSMUSG00000037653	ENSMUSG00000037653	ENSMUSG00000037653	5	69389470	69620436	81	81		
4434	4	44319191	44346874	ENSMUSG00000029158	ENSMUSG00000029158	ENSMUSG00000029158	5	69795810	69821767	81	81		
4435	4	44375203	44397700	ENSMUSG00000029208	ENSMUSG00000029208	ENSMUSG00000029208	5	69836097	69851025	81	81		
4436	4	44398913	44423369	ENSMUSG00000029209	ENSMUSG00000029209	ENSMUSG00000029209	5	69854133	69871420	81	81		
4437	4	45732543	45820855	ENSMUSG00000001260	ENSMUSG00000001260	ENSMUSG00000001260	5	71030182	71121752	81	81		
4438	4	45946341	46086153	ENSMUSG00000000560	ENSMUSG00000000560	ENSMUSG00000000560	5	71240192	71374984	81	81		
4439	4	46431601	46606005	ENSMUSG00000049387	ENSMUSG00000049387	ENSMUSG00000049387	5	71721959	71827340	81	81		
4440	4	46615674	46691181	ENSMUSG00000029211	ENSMUSG00000029211	ENSMUSG00000029211	5	71848869	71937443	81	81		
4441	4	46728134	47123218	ENSMUSG00000029212	ENSMUSG00000029212	ENSMUSG00000029212	5	71979151	72416379	81	81		
4442	4	47147570	47160433	ENSMUSG00000029213	ENSMUSG00000029213	ENSMUSG00000029213	5	72440025	72447279	81	81		
4443	4	47182062	47290260	ENSMUSG00000046808	ENSMUSG00000046808	ENSMUSG00000046808	5	72506804	72576372	81	81		
4444	4	47290772	47534816	ENSMUSG00000005220	ENSMUSG00000005220	ENSMUSG00000005220	5	72579161	72783343	81	81		
4445	4	47544014	47611390	ENSMUSG00000072889	ENSMUSG00000072889	ENSMUSG00000072889	5	72792439	72803364	81	81		
4446	4	48187066	48191181	ENSMUSG00000063935	ENSMUSG00000063935	ENSMUSG00000063935	5	72856249	72860219	81	82	here	
4447	4	47632788	47678188	ENSMUSG00000067220	ENSMUSG00000067220	ENSMUSG00000067220	5	72883250	72916981	81	82	Single marker moved to different place with in a HSB	1 -> 2
4448	4	47713548	47736947	ENSMUSG00000067219	ENSMUSG00000067219	ENSMUSG00000067219	5	72927023	72947330	81	82		
4449	4	47762988	47831030	ENSMUSG00000054892	ENSMUSG00000054892	ENSMUSG00000054892	5	72975117	73015737	81	82		
4450	4	47832557	47966638	ENSMUSG00000029217	ENSMUSG00000029217	ENSMUSG00000029217	5	73034875	73147570	81	82		
4451	4	48038448	48118848	ENSMUSG00000036087	ENSMUSG00000036087	ENSMUSG00000036087	5	73193508	73257515	81	82		
4452	4	48211983	48261758	ENSMUSG00000070733	ENSMUSG00000070733	ENSMUSG00000070733	5	73300346	73355080	81	82		
4453	4	48527839	48558022	ENSMUSG00000029152	ENSMUSG00000029152	ENSMUSG00000029152	5	73571959	73593219	81	82		
4454	4	48582164	48603572	ENSMUSG00000029153	ENSMUSG00000029153	ENSMUSG00000029153	5	73601916	73615082	81	82		
4455	4	48683022	48758853	ENSMUSG00000029154	ENSMUSG00000029154	ENSMUSG00000029154	5	73685213	73732560	81	82		
4456	4	52404097	52477760	ENSMUSG00000051674	ENSMUSG00000051674	ENSMUSG00000051674	5	73760190	73837124	81	82		
4457	4	52555302	52578543	ENSMUSG00000067206	ENSMUSG00000067206	ENSMUSG00000067206	5	73885778	73911563	81	82		
4458	4	52581629	52599222	ENSMUSG00000029156	ENSMUSG00000029156	ENSMUSG00000029156	5	73914393	73923616	81	82		
4459	4	52612350	52658215	ENSMUSG00000029155	ENSMUSG00000029155	ENSMUSG00000029155	5	73930517	73954555	81	82		
4460	4	53155967	53220197	ENSMUSG00000054814	ENSMUSG00000054814	ENSMUSG00000054814	5	74283732	74318810	81	82		
4461	4	53423214	53427759	ENSMUSG00000049907	ENSMUSG00000049907	ENSMUSG00000049907	5	74476993	74481167	81	82		
4462	4	53938620	54020599	ENSMUSG00000029227	ENSMUSG00000029227	ENSMUSG00000029227	5	74817214	74878821	81	82		
4463	4	54021380	54152481	ENSMUSG00000029228	ENSMUSG00000029228	ENSMUSG00000029228	5	74878801	74984593	81	82		
4464	4	54570709	54625570	ENSMUSG00000029229	ENSMUSG00000029229	ENSMUSG00000029229	5	75288121	75326323	81	82		
4465	4	54660955	54663429	ENSMUSG00000035946	ENSMUSG00000035946	ENSMUSG00000035946	5	75357298	75359590	81	82		
4466	4	54790204	54859168	ENSMUSG00000029231	ENSMUSG00000029231	ENSMUSG00000029231	5	75434033	75479895	81	82		
4467	4	55218842	55301638	ENSMUSG00000005672	ENSMUSG00000005672	ENSMUSG00000005672	5	75856705	75938416	81	82		
4468	4	55639401	55686513	ENSMUSG00000062960	ENSMUSG00000062960	ENSMUSG00000062960	5	76214954	76260125	81	82		
4469	4	55907145	55934020	ENSMUSG00000029233	ENSMUSG00000029233	ENSMUSG00000029233	5	77215052	77230246	81	82		
4470	4	55956837	55987099	ENSMUSG00000029234	ENSMUSG00000029234	ENSMUSG00000029234	5	77258725	77284019	81	82		
4471	4	55988827	56107753	ENSMUSG00000029238	ENSMUSG00000029238	ENSMUSG00000029238	5	77285742	77379567	81	82		
4472	4	56117453	56153136	ENSMUSG00000029235	ENSMUSG00000029235	ENSMUSG00000029235	5	77387222	77399888	81	82		
4473	4	56156155	56197222	ENSMUSG00000029236	ENSMUSG00000029236	ENSMUSG00000029236	5	77408271	77438563	81	82		
4474	4	56414573	56466001	ENSMUSG00000036435	ENSMUSG00000036435	ENSMUSG00000036435	5	77604189	77645060	81	82		
4475	4	56509794	56594283	ENSMUSG00000036403	ENSMUSG00000036403	ENSMUSG00000036403	5	77666489	77721241	81	82		
4476	4	56899219	56948395	ENSMUSG00000055923	ENSMUSG00000055923	ENSMUSG00000055923	5	77950710	77976962	81	82		
4477	4	56954288	56996602	ENSMUSG00000029246	ENSMUSG00000029246	ENSMUSG00000029246	5	77989918	77993045	81	82		
4478	4	56971439	56971888	ENSMUSG00000072852	ENSMUSG00000072852	ENSMUSG00000072852	5	78003143	78003652	81	82		
4479	4	56996698	57022282	ENSMUSG00000029247	ENSMUSG00000029247	ENSMUSG00000029247	5	78026186	78042284	81	82		
4480	4	57028549	57062833	ENSMUSG00000036323	ENSMUSG00000036323	ENSMUSG00000036323	5	78049483	78070696	81	82		
4481	4	57066132	57084815	ENSMUSG00000063820	ENSMUSG00000063820	ENSMUSG00000063820	5	78078830	78084253	81	82		
4482	4	57091600	57164246	ENSMUSG00000029248	ENSMUSG00000029248	ENSMUSG00000029248	5	78090798	78136297	81	82		

4483	4	57208921	57242627	ENSMUSG00000059325	ENSMUSG00000059325	ENSMUSG00000059325	5	78161763	78189898	81	82
4484	4	57468832	57496767	ENSMUSG00000029249	ENSMUSG00000029249	ENSMUSG00000029249	5	78342716	78357759	81	82
4485	4	57524273	57539746	ENSMUSG00000036285	ENSMUSG00000036285	ENSMUSG00000036285	5	78368957	78384851	81	82
4486	4	57538665	57592081	ENSMUSG00000029250	ENSMUSG00000029250	ENSMUSG00000029250	5	78385259	78424099	81	82
4487	4	57591696	57671308	ENSMUSG00000036256	ENSMUSG00000036256	ENSMUSG00000036256	5	78424021	78482820	81	82
4488	4	62045434	62620762	ENSMUSG00000037605	ENSMUSG00000037605	ENSMUSG00000037605	5	82096154	82870291	81	82
4489	4	64828385	64957769	ENSMUSG00000049537	ENSMUSG00000049537	ENSMUSG00000049537	5	84352709	84429756	81	82
4490	4	65872241	66218104	ENSMUSG00000029245	ENSMUSG00000029245	ENSMUSG00000029245	5	85132352	85491482	81	82
4491	4	68020584	68093851	ENSMUSG00000029253	ENSMUSG00000029253	ENSMUSG00000029253	5	87086818	87136620	81	82
4492	4	68107041	68155206	ENSMUSG00000029254	ENSMUSG00000029254	ENSMUSG00000029254	5	87146389	87178554	81	82
4493	4	68165123	68249453	ENSMUSG00000035898	ENSMUSG00000035898	ENSMUSG00000035898	5	87185278	87247304	81	82
4494	4	68285701	68304399	ENSMUSG00000029255	ENSMUSG00000029255	ENSMUSG00000029255	5	87256547	87272431	81	82
4495	4	68369196	68432311	ENSMUSG00000061259	ENSMUSG00000061259	ENSMUSG00000061259	5	87378172	87414167	81	82
4496	4	68458614	68511827	ENSMUSG00000072845	ENSMUSG00000072845	ENSMUSG00000072845	5	87486323	87520447	81	82
4497	4	68601511	68678182	ENSMUSG00000048764	ENSMUSG00000048764	ENSMUSG00000048764	5	87598573	87631615	81	82
4498	4	68774967	68794004	ENSMUSG00000035861	ENSMUSG00000035861	ENSMUSG00000035861	5	87735026	87746479	81	82
4499	4	68861639	68898349	ENSMUSG00000035851	ENSMUSG00000035851	ENSMUSG00000035851	5	87895836	87911218	81	82
4500	4	69828756	69852093	ENSMUSG00000035780	ENSMUSG00000035780	ENSMUSG00000035780	5	88399533	88411737	81	82
4501	4	70489562	70548007	ENSMUSG00000029268	ENSMUSG00000029268	ENSMUSG00000029268	5	88534051	88556819	81	82
4502	4	70627277	70661019	ENSMUSG00000029269	ENSMUSG00000029269	ENSMUSG00000029269	5	88587900	88612756	81	82
4503	4	70741520	70760459	ENSMUSG00000029272	ENSMUSG00000029272	ENSMUSG00000029272	5	88650522	88666155	81	82
4504	4	70831388	70846877	ENSMUSG00000070702	ENSMUSG00000070702	ENSMUSG00000070702	5	88740769	88755521	81	82
4505	4	70855564	70861315	ENSMUSG00000063157	ENSMUSG00000063157	ENSMUSG00000063157	5	88769035	88773960	81	82
4506	4	71098421	71104750	ENSMUSG00000009580	ENSMUSG00000009580	ENSMUSG00000009580	5	88962501	88967273	81	82
4507	4	71126377	71135553	ENSMUSG00000070701	ENSMUSG00000070701	ENSMUSG00000070701	5	88983340	88988731	81	82
4508	4	71142894	71151732	ENSMUSG00000001622	ENSMUSG00000001622	ENSMUSG00000001622	5	89000168	89007089	81	82
4509	4	71235287	71237421	ENSMUSG00000007907	ENSMUSG00000007907	ENSMUSG00000007907	5	89054012	89056104	81	82
4510	4	71418887	71433048	ENSMUSG00000029282	ENSMUSG00000029282	ENSMUSG00000029282	5	89450669	89460477	81	82
4511	4	71492582	71507591	ENSMUSG00000029288	ENSMUSG00000029288	ENSMUSG00000029288	5	89531446	89542543	81	82
4512	4	71714082	71731400	ENSMUSG00000029286	ENSMUSG00000029286	ENSMUSG00000029286	5	89562536	89579289	81	82
4513	4	71740548	71751128	ENSMUSG00000067149	ENSMUSG00000067149	ENSMUSG00000067149	5	89595076	89602343	81	82
4514	4	71773098	71775132	ENSMUSG00000070697	ENSMUSG00000070697	ENSMUSG00000070697	5	89629019	89630643	81	82
4515	4	71806576	71891789	ENSMUSG00000029291	ENSMUSG00000029291	ENSMUSG00000029291	5	89639710	89724536	81	82
4516	4	71904356	71924408	ENSMUSG00000044221	ENSMUSG00000044221	ENSMUSG00000044221	5	89734019	89750732	81	82
4517	4	71986928	72072755	ENSMUSG00000006262	ENSMUSG00000006262	ENSMUSG00000006262	5	89815709	89832550	81	82
4518	4	72078256	72115477	ENSMUSG00000029366	ENSMUSG00000029366	ENSMUSG00000029366	5	89839588	89857838	81	82
4519	4	72271867	72654298	ENSMUSG00000060961	ENSMUSG00000060961	ENSMUSG00000060961	5	90009311	90309422	81	82
4520	4	72826296	72868798	ENSMUSG00000035540	ENSMUSG00000035540	ENSMUSG00000035540	5	90492721	90532964	81	82
4521	4	73116385	73232642	ENSMUSG00000035528	ENSMUSG00000035528	ENSMUSG00000035528	5	90602628	90658939	81	82
4522	4	73365551	73653380	ENSMUSG00000050676	ENSMUSG00000050676	ENSMUSG00000050676	5	90752563	90766565	81	82
4523	4	74139279	74154336	ENSMUSG00000035505	ENSMUSG00000035505	ENSMUSG00000035505	5	91289927	91299163	81	82
4524	4	74159369	74343249	ENSMUSG00000055204	ENSMUSG00000055204	ENSMUSG00000055204	5	91302365	91441247	81	82
4525	4	74488870	74505996	ENSMUSG00000029368	ENSMUSG00000029368	ENSMUSG00000029368	5	91536101	91551800	81	82
4526	4	74520797	74540356	ENSMUSG00000054932	ENSMUSG00000054932	ENSMUSG00000054932	5	91565937	91584107	81	82
4527	4	74566326	74588582	ENSMUSG00000029369	ENSMUSG00000029369	ENSMUSG00000029369	5	91594149	91628735	81	82
4528	4	74657024	74705081	ENSMUSG00000029370	ENSMUSG00000029370	ENSMUSG00000029370	5	91679073	91692987	81	82
4529	4	75071622	75072764	ENSMUSG00000029372	ENSMUSG00000029372	ENSMUSG00000029372	5	91843718	91845263	81	82
4530	4	75393068	75398162	ENSMUSG00000035020	ENSMUSG00000035020	ENSMUSG00000035020	5	92102717	92110417	81	82
4531	4	75449724	75473341	ENSMUSG00000029377	ENSMUSG00000029377	ENSMUSG00000029377	5	92149822	92168846	81	82
4532	4	75889001	75938711	ENSMUSG00000029391	ENSMUSG00000029391	ENSMUSG00000029391	5	92432461	92478113	81	82
4533	4	76156658	76194347	ENSMUSG00000034981	ENSMUSG00000034981	ENSMUSG00000034981	5	92669017	92699211	81	82
4534	4	76623381	76658652	ENSMUSG00000029397	ENSMUSG00000029397	ENSMUSG00000029397	5	93024102	93038268	81	82
4535	4	76658707	76674258	ENSMUSG00000060466	ENSMUSG00000060466	ENSMUSG00000060466	5	93038533	93047261	81	82
4536	4	76723375	76774595	ENSMUSG00000029403	ENSMUSG00000029403	ENSMUSG00000029403	5	93081352	93118218	81	82
4537	4	76786991	76817629	ENSMUSG00000029405	ENSMUSG00000029405	ENSMUSG00000029405	5	93127364	93158905	81	82
4538	4	77000052	77042705	ENSMUSG00000029410	ENSMUSG00000029410	ENSMUSG00000029410	5	93301876	93331478	81	82
4539	4	77055081	77081126	ENSMUSG00000029413	ENSMUSG00000029413	ENSMUSG00000029413	5	93333459	93353381	81	82
4540	4	77090083	77131115	ENSMUSG00000029415	ENSMUSG00000029415	ENSMUSG00000029415	5	93359210	93385224	81	82
4541	4	77141523	77147648	ENSMUSG00000029417	ENSMUSG00000029417	ENSMUSG00000029417	5	93398190	93403317	81	82
4542	4	77161297	77163674	ENSMUSG00000034855	ENSMUSG00000034855	ENSMUSG00000034855	5	93423001	93424014	81	82
4543	4	77173975	77176376	ENSMUSG00000060183	ENSMUSG00000060183	ENSMUSG00000060183	5	93435400	93440685	81	82
4544	4	77214887	77252905	ENSMUSG00000034842	ENSMUSG00000034842	ENSMUSG00000034842	5	93463343	93489822	81	82
4545	4	77254842	77288679	ENSMUSG00000034826	ENSMUSG00000034826	ENSMUSG00000034826	5	93490746	93510323	81	82
4546	4	77298918	77354059	ENSMUSG00000029426	ENSMUSG00000029426	ENSMUSG00000029426	5	93519073	93580808	81	82
4547	4	77357785	77423931	ENSMUSG00000057068	ENSMUSG00000057068	ENSMUSG00000057068	5	93660487	93665818	81	82
4548	4	77446203	77450763	ENSMUSG00000047963	ENSMUSG00000047963	ENSMUSG00000047963	5	93678255	93681783	81	82
4549	4	77453350	77543384	ENSMUSG00000050050	ENSMUSG00000050050	ENSMUSG00000050050	5	93683499	93750331	81	82
4550	4	77575277	77919966	ENSMUSG00000029381	ENSMUSG00000029381	ENSMUSG00000029381	5	93758639	94040095	81	82
4551	4	78035645	78038026	ENSMUSG00000045314	ENSMUSG00000045314	ENSMUSG00000045314	5	94117780	94120062	81	82
4552	4	78089919	78178791	ENSMUSG00000058013	ENSMUSG00000058013	ENSMUSG00000058013	5	94168655	94251641	81	82
4553	4	78297551	78319319	ENSMUSG00000029385	ENSMUSG00000029385	ENSMUSG00000029385	5	94343826	94350201	81	82
4554	4	78745998	78752006	ENSMUSG00000023078	ENSMUSG00000023078	ENSMUSG00000023078	5	96197241	96201370	81	82
4555	4	79198158	79684446	ENSMUSG00000034687	ENSMUSG00000034687	ENSMUSG00000034687	5	96614257	97025030	81	82
4556	4	79694613	79750606	ENSMUSG00000029484	ENSMUSG00000029484	ENSMUSG00000029484	5	97042796	97086270	81	82
4557	4	80027331	80079572	ENSMUSG00000055725	ENSMUSG00000055725	ENSMUSG00000055725	5	97322631	97351898	81	82
4558	4	80546717	80548378	ENSMUSG00000050553	ENSMUSG00000050553	ENSMUSG00000050553	5	97695615	97697279	81	82
4559	4	81117757	81212741	ENSMUSG00000029338	ENSMUSG00000029338	ENSMUSG00000029338	5	98178389	98270851	81	82
4560	4	81324463	81343710	ENSMUSG00000035456	ENSMUSG00000035456	ENSMUSG00000035456	5	98421324	98429293	81	82
4561	4	81406766	81431194	ENSMUSG00000029337	ENSMUSG00000029337	ENSMUSG00000029337	5	98494486	98517332	81	82
4562	4	81475947	82103790	ENSMUSG00000057816	ENSMUSG00000057816	ENSMUSG00000057816	5	98569656	99042212	81	82
4563	4	82171143	82193749	ENSMUSG00000029335	ENSMUSG00000029335	ENSMUSG00000029335	5	99094741	99120430	81	82
4564	4	82228861	82345239	ENSMUSG00000029334	ENSMUSG00000029334	ENSMUSG00000029334	5	99171570	99277381	81	82
4565	4	82567243	82612085	ENSMUSG00000029333	ENSMUSG00000029333	ENSMUSG00000029333	5	99461659	99483542	81	82
4566	4	83493491	83514173	ENSMUSG00000000568	ENSMUSG00000000568	ENSMUSG00000000568	5	100201309	100218926	81	82
4567	4	83563388	83570447	ENSMUSG00000029328	ENSMUSG00000029328	ENSMUSG00000029328	5	100276430	100279464	81	82
4568	4	83570787	83601263	ENSMUSG00000029326	ENSMUSG00000029326	ENSMUSG00000029326	5	100280303	100309062	81	82
4569	4	83959199	84040715	ENSMUSG00000035325	ENSMUSG00000035325	ENSMUSG00000035325	5	100602356	100648414	81	82
4570	4	84065167	84151006	ENSMUSG00000035310	ENSMUSG00000035310	ENSMUSG00000035310	5	100682343	100740928	81	82
4571	4	84175263	84215994	ENSMUSG00000035297	ENSMUSG00000035297	ENSMUSG00000035297	5	100758746	100788082	81	82
4572	4	84230239	84254935	ENSMUSG00000029322	ENSMUSG00000029322	ENSMUSG00000029322	5	100794035	100812527	81	82
4573	4	84404003	84425091	ENSMUSG00000029319	ENSMUSG00000029319	ENSMUSG00000029319	5	100895023	100914553	81	82
4574	4	84432639	84475329	ENSMUSG00000035273	ENSMUSG00000035273	ENSMUSG00000035273	5	100920698	100959985	81	82
4575	4	84547527	84596020	ENSMUSG00000035266	ENSMUSG00000035266	ENSMUSG00000035266	5	101002454	101038866	81	82
4576	4	84596196	84601900	ENSMUSG00000016833	ENSMUSG00000016833	ENSMUSG00000016833	5	101039049	101044756	81	82
4577	4	84601263	84625277	ENSMUSG00000035234	ENSMUSG00000035234	ENSMUSG00000035234	5	101045498	101061264	81	82
4578	4	84676588	84746049	ENSMUSG00000029314	ENSMUSG00000029314	ENSMUSG00000029314	5	101086598	101139401	81	82
4579	4	85633460	85638411	ENSMUSG00000035187	ENSMUSG00000035187	ENSMUSG00000035187	5	101899479	101905013	81	82
4580	4	85723081	85789363	ENSMUSG00000029330	ENSMUSG00000029330	ENSMUSG00000029330	5	102005432	102064154	81	82
4581	4	85809719	86106568	ENSMUSG00000043940	ENSMUSG00000043940	ENSMUSG00000043940	5	102075887	102310223	81	82
4582	4	86710719	87140899	ENSMUSG00000057315	ENSMUSG00000057315	ENSMUSG00000057315	5	102721693	103138131	81	82
4583	4	87156657	87500242	ENSMUSG00000046709	ENSMUSG00000046709	ENSMUSG00000046709	5	103152671	103451575	81	82
4584	4	87734909	87955326	ENSMUSG00000034573	ENSMUSG00000034573	ENSMUSG00000034573	5	103665481	103798319	81	82
4585	4	87963865	87989292	ENSMUSG00000029321	ENSMUSG00000029321	ENSMUSG00000029321	5	103846013	103869705	81	82
4586	4	88016437	88032572	ENSMUSG00000029320	ENSMUSG00000029320	ENSMUSG00000029320	5	103888895	103896026	81	82
4587	4	88147164	88281214	ENSMUSG00000029313	ENSMUSG00000029313	ENSMUSG00000029313	5	104024342	104091122	81	82
4588	4	88300749	88360698	ENSMUSG00000029312	ENSMUSG00000029312	ENSMUSG00000029312	5	104102352	104151531	81	82
4589	4	88443965	88463062	ENSMUSG00000034528	ENSMUSG00000034528	ENSMUSG00000034528	5	104195742	104217666	81	82
4590	4	88476790	88531369	ENSMUSG00000029311	ENSMUSG00000029311	ENSMUSG00000029311	5	104230067	104262098	81	82
4591	4	88562759	88598519	ENSMUSG00000029310	ENSMUSG00000029310	ENSMUSG00000029310	5	104287299	104305669	81	82
4592	4	88613524	88669410	ENSMUSG00000029309	ENSMUSG00000029309	ENSMUSG00000029309	5	104319413	104354035	81	82
4593	4	88751085	88756744	ENSMUSG00000053268	ENSMUSG00000053268	ENSMUSG00000053268	5	104411005	104418912	81	82
4594	4	88790483	88804529	ENSMUSG00000029307	ENSMUSG00000029307	ENSMUSG00000029307	5	104442915	104454404	81	82
4595	4	88939757	88952098	ENSMUSG00000029306	ENSMUSG00000029306	ENSMUSG00000029306	5	104539604	104551767	81	82
4596	4	88961574	88986990	ENSMUSG00000053863	ENSMUSG00000053863	ENSMUSG00000053863	5	104565631	104578913	81	82
4597	4	89115890	89123502	ENSMUSG00000029304	ENSMUSG00000029304	ENSMUSG00000029304	5	104675436	104681350	81	82
4598	4	89147844	89217952	ENSMUSG00000034462	ENSMUSG00000034462	ENSMUSG00000034462	5	104699752	104746120	81	82
4599	1	89763050	89836011	ENSMUSG00000070639	ENSMUSG00000070639	ENSMUSG00000070639	5	105720092	105726491	82	83
4600	1	89871219	89957680	ENSMUSG00000054720	ENSMUSG00000054720	ENSMUSG00000054720	5	105759706	105849258	82	83
4601	1	90059192	90174147	ENSMUSG00000046079	ENSMUSG00000046079	ENSMUSG00000046079	5	105940338	106055354	82	83
4602	1	90233262	90266369	ENSMUSG00000029290	ENSMUSG00000029290	ENSMUSG00000029290	5	106116870	106156114	82	83
4603	1	90949747	90955382	ENSMUSG00000034384	ENSMUSG00000034384	ENSMUSG00000034384	5	106692816	106698468	82	83
4604	1	91153447	91260259	ENSMUSG00000049606	ENSMUSG00000049606	ENSMUSG00000049606	5	106858538	106878939	82	83
4605	1	91500331	91581665	ENSMUSG00000043410	ENSMUSG00000043410	ENSMUSG00000043410	5	107081912	107126843	82	83
4606	1	91615588	91642954	ENSMUSG00000048367	ENSMUSG00000048367	ENSMUSG00000048367	5	107142191	107161388	82	83
4607	1	91739021	91763901	ENSMUSG00000029283	ENSMUSG00000029283	ENSMUSG00000029283	5	107204624	107224733	82	83
4608	1	91920575	92124243	ENSMUSG00000029287	ENSMUSG00000029287	ENSMUSG00000029287	5	107346872	107529822	82	83
4609	1	92187516	92252563	ENSMUSG00000029279	ENSMUSG00000029279	ENSMUSG00000029279	5	107571461	107626009	82	83
4610	1	92268127	92301681	ENSMUSG00000033805	ENSMUSG00000033805	ENSMUSG00000033805	5	107643853	107670193	82	83
4611	1	92318481	92385983	ENSMUSG00000070632	ENSMUSG00000070632	ENSMUSG00000070632	5	107678299	107731898	82	83
4612	1	92407069	92422868	ENSMUSG00000044060	ENSMUSG00000044060	ENSMUSG00000044060	5	107748014	107751120	82	83
4613	1	92456085	92483958	ENSMUSG00000070631	ENSMUSG00000070631	ENSMUSG00000070631	5	107775059	107788562	82	83
4614	1	92484547	92537121	ENSMUSG00000029276	ENSMUSG00000029276	ENSMUSG00000029276	5	107789325	107838190	82	83
4615	1	92537110	92626318	ENSMUSG00000033773	ENSMUSG00000033773	ENSMUSG00000033773	5	107837998	107902140	82	83
4616	1	92712907	92722099	ENSMUSG00000029275	ENSMUSG00000029275	ENSMUSG00000029275	5	107956968	107966108	82	83
4617	1	92746841	93030549	ENSMUSG00000011831	ENSMUSG00000011831	ENSMUSG00000011831	5	107988448	108082602	82	83
4618	1	93080312	93199645	ENSMUSG00000029270	ENSMUSG00000029270	ENSMUSG00000029270	5	108149706	108165027	82	83
4619	1	93317383	93377226	ENSMUSG00000029267	ENSMUSG00000029267	ENSMUSG00000029267	5	108321125	108348571	82	83
4620	1	93387887	93418873	ENSMUSG00000063406	ENSMUSG00000063406	ENSMUSG00000063406	5	108361949	108372893	82	83
4621	1	93418869	93516875	ENSMUSG00000056531	ENSMUSG00000056531	ENSMUSG00000056531	5	108375727	108469349	82	83
4622	1	93584033	93600737	ENSMUSG00000029265	ENSMUSG00000029265	ENSMUSG00000029265	5	108509199	108520823	82	83
4623	4	483019	523315	ENSMUSG00000029263	ENSMUSG00000029263	ENSMUSG00000029263	5	108553227	108576867	83	84
4624	4	609373	653896	ENSMUSG00000029491	ENSMUSG00000029491	ENSMUSG00000029491	5	108628694	108672034	83	84
4625	4	656227	658127	ENSMUSG00000050856	ENSMUSG00000050856	ENSMUSG00000050856	5	108673556	108674688	83	84
4626	4	665618	673230	ENSMUSG00000029490	ENSMUSG00000029490	ENSMUSG00000029490	5	108681356	108689193	83	84
4627	4	689573	754428	ENSMUSG00000033623	ENSMUSG00000033623	ENSMUSG00000033623	5	108701634	108743401	83	84
4628	4	833066	915975	ENSMUSG00000062234	ENSMUSG00000062234	ENSMUSG00000062234	5	108809716	108870041	83	84
4629	4	916222	942444	ENSMUSG00000013495	ENSMUSG00000013495	ENSMUSG00000013495	5	108870304	108887565	83	84
4630	4	942675	957344	ENSMUSG00000004815	ENSMUSG00000004815	ENSMUSG00000004815	5	108886995	108901113	83	84
4631	4	970785	988316	ENSMUSG00000033540	ENSMUSG00000033540	ENSMUSG00000033540	5	108909667	108924647	83	84
4632	4	971447	977224	ENSMUSG00000046959	ENSMUSG00000046959	ENSMUSG00000046959	5	108910180	108915871	83	84
4633	4	996239	1010685	ENSMUSG00000008090	ENSMUSG00000008090	ENSMUSG00000008090	5	108934804	108947200	83	84
4634	4	1056544	1097314	ENSMUSG00000055385	ENSMUSG00000055385	ENSMUSG00000055385	5	108971963	108989750	83	84
4635	X	1274890	1291527	ENSMUSG00000033467	ENSMUSG00000033467	ENSMUSG00000033467	5	109795011	109799277	84	85
4636	X	132989	160023	ENSMUSG00000064247	ENSMUSG00000064247	ENSMUSG00000064247	5	110340271	110344119	84	85
4637	X	160025	170886	ENSMUSG00000033434	ENSMUSG00000033434	ENSMUSG00000033434	5	110344281	110348473	84	85
4638	12	131927641	131974267	ENSMUSG00000014668	ENSMUSG00000014668	ENSMUSG00000014668	5	110376162	110412271	85	86
4639	12	131855568	131915361	ENSMUSG00000029502	ENSMUSG00000029502	ENSMUSG00000029502	5	110417003	110463457	85	86
4640	12	131813778	131848490	ENSMUSG00000029501	ENSMUSG00000029501	ENSMUSG00000029501	5	110471343	110496952	85	86
4641	12	131797509	131805819	ENSMUSG00000029500	ENSMUSG00000029500	ENSMUSG00000029500	5	110499457	110510184	85	86
4642	12	131774265	131791649	ENSMUSG00000029499	ENSMUSG00000029499	ENSMUSG00000029499	5	110514597	110526323	85	86
4643	12	131710421	131923460	ENSMUSG00000007080	ENSMUSG00000007080	ENSMUSG00000007080	5	110526686	110577755	85	86
4644	12	131705476	131708631	ENSMUSG00000029503	ENSMUSG00000029503	ENSMUSG00000029503	5	110580495	110583257	85	86
4645	12	131690913	131691478	ENSMUSG00000072754	ENSMUSG00000072754	ENSMUSG00000072754	5	110594482	110595152	85	86
4646	12	131657121	131670437	ENSMUSG00000043323	ENSMUSG00000043323	ENSMUSG00000043323	5	110603089	110627398	85	86
4647	12	131247605	131415862	ENSMUSG00000033316	ENSMUSG00000033316	ENSMUSG00000033316	5	110785004	110860953	85	86
4648	12	131194946	131202939	ENSMUSG00000033294	ENSMUSG00000033294	ENSMUSG00000033294	5	110888721	110893684	85	86
4649	12	131190033	131194806	ENSMUSG00000029504	ENSMUSG00000029504	ENSMUSG00000029504	5	110893776	110900614	85	86
4650	12	131000461	131130958	ENSMUSG00000029505	ENSMUSG00000029505	ENSMUSG00000029505	5	110904680	111010829	85	86
4651	12	130979742	130994355	ENSMUSG00000029507	ENSMUSG00000029507	ENSMUSG00000029507	5	111013969	111020917	85	86
4652	12	130945232	130973649	ENSMUSG00000029512	ENSMUSG00000029512	ENSMUSG00000029512	5	111024789	111050383	85	86
4653	22	27468019	27483488	ENSMUSG00000043510	ENSMUSG00000043510	ENSMUSG00000043510	5	111069372	111080043	86	87
4654	22	27413731	27467822	ENSMUSG00000029521	ENSMUSG00000029521	ENSMUSG00000029521	5	111080319	111114435	86	87
4655	22	26708209	27405711	ENSMUSG00000033209	ENSMUSG00000033209	ENSMUSG00000033209	5	111133134	111526847	86	87
4656	22	26577658	26645255	ENSMUSG00000050017	ENSMUSG00000050017	ENSMUSG00000050017	5	111571066	111628662	86	87
4657	22	26474266	26526531	ENSMUSG00000070576	ENSMUSG00000070576	ENSMUSG00000070576	5	111658469	111695094	86	87
4658	22	25347928	25356632	ENSMUSG00000066975	ENSMUSG00000066975	ENSMUSG00000066975	5	112486797	112492105	86	87
4659	22	25325362	25344038	ENSMUSG00000029343	ENSMUSG00000029343	ENSMUSG00000029343	5	112496124	112509885	86	87
4660	22	25217898	25238343	ENSMUSG00000029345	ENSMUSG00000029345	ENSMUSG00000029345	5	112566672	112578376	86	87
4661	22	25209846	25217897	ENSMUSG00000029346	ENSMUSG00000029346	ENSMUSG00000029346	5	112578275	112583308	86	87
4662	22	25177446	25209820	ENSMUSG00000042328	ENSMUSG00000042328	ENSMUSG00000042328	5	112583398	112618715	86	87
4663	22	25155239	25169557	ENSMUSG00000029348	ENSMUSG00000029348	ENSMUSG00000029348	5	112625748	112632516	86	87
4664	22	24895480	25106434	ENSMUSG00000058153	ENSMUSG00000058153	ENSMUSG00000058153	5	112659454	112817278	86	87
4665	22	24487060	24757007	ENSMUSG00000072720	ENSMUSG00000072720	ENSMUSG00000072720	5	113057527	113112813	86	87
4666	22	24290946	24449916	ENSMUSG00000042249	ENSMUSG00000042249	ENSMUSG00000042249	5	113150781	113255817	86	87
4667	22	23925825	23933324	ENSMUSG00000029352	ENSMUSG00000029352	ENSMUSG00000029352	5	113316142	113320956	86	87
4668	22	23753967	23923405	ENSMUSG00000051339	ENSMUSG00000051339	ENSMUSG00000051339	5	113332722	113341880	86	87
4669	22	23661208	23665314	ENSMUSG00000066964	ENSMUSG00000066964	ENSMUSG00000066964	5	113467212	113479566	86	87
4670	22	23532286	23650241	ENSMUSG00000042216	ENSMUSG00000042216	ENSMUSG00000042216	5	113485308	113529205	86	87
4671	12	107047641	107168441	ENSMUSG00000063430	ENSMUSG00000063430	ENSMUSG00000063430	5	113811698	113849086	87	88
4672	12	107208800	107257218	ENSMUSG00000042190	ENSMUSG00000042190	ENSMUSG00000042190	5	113873360	113911389	87	88
4673	12	107433181	107437510	ENSMUSG00000053334	ENSMUSG00000053334	ENSMUSG00000053334	5	113996802	114001599	87	88
4674	12	107440487	107479067	ENSMUSG00000018974	ENSMUSG00000018974	ENSMUSG00000018974	5	114003445	114032648	87	88
4675	12	107507756	107516023	ENSMUSG00000054675	ENSMUSG00000054675	ENSMUSG00000054675	5	114054729	114061365	87	88
4676	12	107540974	107542212	ENSMUSG00000048163	ENSMUSG00000048163	ENSMUSG00000048163	5	114079845	114091474	87	88
4677	12	107563020	107649424	ENSMUSG00000004530	ENSMUSG00000004530	ENSMUSG00000004530	5	114103438	114169713	87	88

4678	12	107705100	107775480	ENSMUSG00000042121	ENSMUSG00000042121	ENSMUSG00000042121	5	114202650	114254758	87	88		
4679	12	107797996	107818826	ENSMUSG00000042096	ENSMUSG00000042096	ENSMUSG00000042096	5	114265807	114286445	87	88		
4680	12	107828787	107856860	ENSMUSG00000042078	ENSMUSG00000042078	ENSMUSG00000042078	5	114287909	114352379	87	88		
4681	12	107974774	108008560	ENSMUSG00000029592	ENSMUSG00000029592	ENSMUSG00000029592	5	114361332	114383923	87	88		
4682	12	108010379	108015660	ENSMUSG00000044339	ENSMUSG00000044339	ENSMUSG00000044339	5	114384933	114389175	87	88		
4683	12	108019798	108033181	ENSMUSG00000029591	ENSMUSG00000029591	ENSMUSG00000029591	5	114391376	114400320	87	88		
4684	12	108038783	108188550	ENSMUSG00000042010	ENSMUSG00000042010	ENSMUSG00000042010	5	114426517	114510733	87	88		
4685	12	108200167	108231408	ENSMUSG00000042002	ENSMUSG00000042002	ENSMUSG00000042002	5	114515164	114534057	87	88		
4686	12	108362755	108370561	ENSMUSG00000066952	ENSMUSG00000066952	ENSMUSG00000066952	5	114617814	114625575	87	88		
4687	12	108370843	108399538	ENSMUSG00000001098	ENSMUSG00000001098	ENSMUSG00000001098	5	114624566	114641514	87	88		
4688	12	108399822	108458887	ENSMUSG00000029577	ENSMUSG00000029577	ENSMUSG00000029577	5	114641606	114682165	87	88		
4689	12	108477662	108495685	ENSMUSG00000029575	ENSMUSG00000029575	ENSMUSG00000029575	5	114693081	114705026	87	88		
4690	12	108496995	108519450	ENSMUSG00000041939	ENSMUSG00000041939	ENSMUSG00000041939	5	114705305	114721590	87	88		
4691	12	108636573	108692694	ENSMUSG00000041930	ENSMUSG00000041930	ENSMUSG00000041930	5	114829250	114874219	87	88		
4692	12	108705277	108755595	ENSMUSG00000014158	ENSMUSG00000014158	ENSMUSG00000014158	5	114883154	114919404	87	88		
4693	12	108822462	108875457	ENSMUSG00000002486	ENSMUSG00000002486	ENSMUSG00000002486	5	114969653	114983325	87	88		
4694	12	108851992	108918483	ENSMUSG00000041890	ENSMUSG00000041890	ENSMUSG00000041890	5	114991181	115034364	87	88		
4695	12	108921618	108961614	ENSMUSG00000041870	ENSMUSG00000041870	ENSMUSG00000041870	5	115035676	115066821	87	88		
4696	12	119942478	119961164	ENSMUSG00000041827	ENSMUSG00000041827	ENSMUSG00000041827	5	115184239	115198910	88	89		
4697	12	119925231	119938683	ENSMUSG00000029559	ENSMUSG00000029559	ENSMUSG00000029559	5	115203201	115209539	88	89		
4698	12	119900932	119924695	ENSMUSG00000029556	ENSMUSG00000029556	ENSMUSG00000029556	5	115209360	115232037	88	89		
4699	12	119685419	119826534	ENSMUSG00000029550	ENSMUSG00000029550	ENSMUSG00000029550	5	115272523	115359789	88	89		
4700	12	119648025	119662193	ENSMUSG00000029545	ENSMUSG00000029545	ENSMUSG00000029545	5	115371298	115380312	88	89		
4701	12	119632644	119645825	ENSMUSG00000046562	ENSMUSG00000046562	ENSMUSG00000046562	5	115383565	115395974	88	89		
4702	12	119609332	119624050	ENSMUSG00000048578	ENSMUSG00000048578	ENSMUSG00000048578	5	115403981	115419078	88	89		
4703	12	119562805	119589508	ENSMUSG00000029544	ENSMUSG00000029544	ENSMUSG00000029544	5	115429945	115447120	88	89		
4704	12	119501232	119503583	ENSMUSG00000060152	ENSMUSG00000060152	ENSMUSG00000060152	5	115496826	115501971	88	89		
4705	12	119456538	119499740	ENSMUSG00000041740	ENSMUSG00000041740	ENSMUSG00000041740	5	115502770	115533893	88	89		
4706	12	119425466	119451337	ENSMUSG00000041733	ENSMUSG00000041733	ENSMUSG00000041733	5	115540694	115557964	88	89		
4707	12	119418309	119420679	ENSMUSG00000009013	ENSMUSG00000009013	ENSMUSG00000009013	5	115558125	115561933	88	89		
4708	12	119383855	119391941	ENSMUSG00000029538	ENSMUSG00000029538	ENSMUSG00000029538	5	115588176	115594079	88	89		
4709	12	119368630	119383772	ENSMUSG00000029536	ENSMUSG00000029536	ENSMUSG00000029536	5	115594241	115602160	88	89		
4710	12	119366148	119368598	ENSMUSG00000029535	ENSMUSG00000029535	ENSMUSG00000029535	5	115602246	115604551	88	89		
4711	12	119263516	119291341	ENSMUSG00000054256	ENSMUSG00000054256	ENSMUSG00000054256	5	115690684	115715201	88	89		
4712	12	119244298	119249975	ENSMUSG00000029522	ENSMUSG00000029522	ENSMUSG00000029522	5	115727265	115735716	88	89		
4713	12	119224546	119235430	ENSMUSG00000029524	ENSMUSG00000029524	ENSMUSG00000029524	5	115740596	115744110	88	89		
4714	12	119132637	119187904	ENSMUSG00000029528	ENSMUSG00000029528	ENSMUSG00000029528	5	115805341	115815134	88	89		
4715	12	119049399	119116896	ENSMUSG00000041638	ENSMUSG00000041638	ENSMUSG00000041638	5	115826263	115883649	88	89		
4716	12	119017289	119038982	ENSMUSG00000029518	ENSMUSG00000029518	ENSMUSG00000029518	5	115892928	115908735	88	89		
4717	12	118912031	119016680	ENSMUSG00000041609	ENSMUSG00000041609	ENSMUSG00000041609	5	115909177	115992551	88	89		
4718	12	118607981	118799475	ENSMUSG00000029516	ENSMUSG00000029516	ENSMUSG00000029516	5	116106643	116269946	88	89		
4719	12	118590144	118603799	ENSMUSG00000029513	ENSMUSG00000029513	ENSMUSG00000029513	5	116274585	116285427	88	89		
4720	12	118256900	118463234	ENSMUSG00000043913	ENSMUSG00000043913	ENSMUSG00000043913	5	116386580	116549984	88	89		
4721	12	118100978	118116933	ENSMUSG00000041548	ENSMUSG00000041548	ENSMUSG00000041548	5	116669494	116683863	88	89		
4722	12	117903779	118085240	ENSMUSG00000063919	ENSMUSG00000063919	ENSMUSG00000063919	5	116700274	116852463	88	89		
4723	12	117073165	117295044	ENSMUSG00000061288	ENSMUSG00000061288	ENSMUSG00000061288	5	117381159	117534864	88	89		
4724	12	116985783	117026223	ENSMUSG00000066894	ENSMUSG00000066894	ENSMUSG00000066894	5	117580348	117600078	88	89		
4725	12	116954882	116983334	ENSMUSG00000029364	ENSMUSG00000029364	ENSMUSG00000029364	5	117618335	117639631	88	89		
4726	12	116938893	116954421	ENSMUSG00000029363	ENSMUSG00000029363	ENSMUSG00000029363	5	117640177	117650063	88	89		
4727	12	116391846	116777724	ENSMUSG00000061578	ENSMUSG00000061578	ENSMUSG00000061578	5	117675030	117816972	88	89		
4728	12	116135362	116283965	ENSMUSG00000029361	ENSMUSG00000029361	ENSMUSG00000029361	5	118103174	118219870	88	89		
4729	12	116065969	116112644	ENSMUSG00000032898	ENSMUSG00000032898	ENSMUSG00000032898	5	118237800	118271221	88	89		
4730	12	115833144	115953336	ENSMUSG00000032867	ENSMUSG00000032867	ENSMUSG00000032867	5	118326029	118416415	88	89		
4731	12	115783411	115803615	ENSMUSG00000046607	ENSMUSG00000046607	ENSMUSG00000046607	5	118430794	118450508	88	89		
4732	12	115660526	115775129	ENSMUSG00000032850	ENSMUSG00000032850	ENSMUSG00000032850	5	118451776	118506017	88	89		
4733	12	115637981	115660226	ENSMUSG00000032840	ENSMUSG00000032840	ENSMUSG00000032840	5	118506299	118524134	88	89		
4734	12	114880766	115199526	ENSMUSG00000018076	ENSMUSG00000018076	ENSMUSG00000018076	5	118821773	119024010	88	89		
4735	12	113592442	113606352	ENSMUSG00000018604	ENSMUSG00000018604	ENSMUSG00000018604	5	119931286	119945218	88	89		
4736	12	113276119	113330630	ENSMUSG00000018263	ENSMUSG00000018263	ENSMUSG00000018263	5	120095280	120145102	88	89		
4737	12	112738930	112888494	ENSMUSG00000029594	ENSMUSG00000029594	ENSMUSG00000029594	5	120377130	120459588	88	89		
4738	12	112385077	112394260	ENSMUSG00000029595	ENSMUSG00000029595	ENSMUSG00000029595	5	120692518	120702074	88	89		
4739	12	112344601	112360463	ENSMUSG00000029596	ENSMUSG00000029596	ENSMUSG00000029596	5	120718820	120733427	88	89		
4740	12	112314640	112326075	ENSMUSG00000029597	ENSMUSG00000029597	ENSMUSG00000029597	5	120737164	120744546	88	89		
4741	12	112280761	112311584	ENSMUSG00000029598	ENSMUSG00000029598	ENSMUSG00000029598	5	120743899	120764235	88	89		
4742	12	112220955	112257308	ENSMUSG00000032754	ENSMUSG00000032754	ENSMUSG00000032754	5	120771809	120794636	88	89		
4743	12	112143659	112220770	ENSMUSG00000032741	ENSMUSG00000032741	ENSMUSG00000032741	5	120796725	120820970	88	89		
4744	12	112117631	112143263	ENSMUSG00000029601	ENSMUSG00000029601	ENSMUSG00000029601	5	120849633	120867733	88	89		
4745	12	112107924	112114333	ENSMUSG00000029600	ENSMUSG00000029600	ENSMUSG00000029600	5	120869680	120873186	88	89		
4746	12	112081065	112107667	ENSMUSG00000029599	ENSMUSG00000029599	ENSMUSG00000029599	5	120873747	120889209	88	89		
4747	12	112021007	112058412	ENSMUSG00000029602	ENSMUSG00000029602	ENSMUSG00000029602	5	120909439	120940210	88	89		
4748	12	111980045	112020216	ENSMUSG00000029603	ENSMUSG00000029603	ENSMUSG00000029603	5	120940820	120972241	88	89		
4749	12	111900657	111933911	ENSMUSG00000032690	ENSMUSG00000032690	ENSMUSG00000032690	5	120993754	121010390	88	89		
4750	12	111860540	111895433	ENSMUSG00000032661	ENSMUSG00000032661	ENSMUSG00000032661	5	121013715	121038276	88	89		
4751	12	111750507	111821065	ENSMUSG00000029608	ENSMUSG00000029608	ENSMUSG00000029608	5	121201119	121270697	88	89		
4752	12	111340919	111432099	ENSMUSG00000043733	ENSMUSG00000043733	ENSMUSG00000043733	5	121395374	121432734	88	89		
4753	12	111171744	111195940	ENSMUSG00000053294	ENSMUSG00000053294	ENSMUSG00000053294	5	121546903	121564292	88	89		
4754	12	111082384	111169738	ENSMUSG00000042744	ENSMUSG00000042744	ENSMUSG00000042744	5	121609335	121629180	88	89		
4755	12	111047747	111075790	ENSMUSG00000042726	ENSMUSG00000042726	ENSMUSG00000042726	5	121632347	121646188	88	89		
4756	12	110948877	111030980	ENSMUSG00000042719	ENSMUSG00000042719	ENSMUSG00000042719	5	121658599	121700730	88	89		
4757	12	110853486	110935326	ENSMUSG00000029452	ENSMUSG00000029452	ENSMUSG00000029452	5	121685638	121755805	88	89		
4758	12	110935535	110945407	ENSMUSG00000029616	ENSMUSG00000029616	ENSMUSG00000029616	5	121705368	121713091	88	89		
4759	12	110787435	110815543	ENSMUSG00000029454	ENSMUSG00000029454	ENSMUSG00000029454	5	121785668	121806509	88	89		
4760	12	110688729	110732165	ENSMUSG00000029455	ENSMUSG00000029455	ENSMUSG00000029455	5	121828319	121854203	88	89		
4761	12	110565881	110608138	ENSMUSG00000029458	ENSMUSG00000029458	ENSMUSG00000029458	5	121921203	121947867	88	89		
4762	12	110374455	110521863	ENSMUSG00000042605	ENSMUSG00000042605	ENSMUSG00000042605	5	121972226	122075325	88	89		
4763	12	110328135	110373809	ENSMUSG00000042594	ENSMUSG00000042594	ENSMUSG00000042594	5	122076265	122097476	88	89		
4764	12	110282868	110291209	ENSMUSG00000044134	ENSMUSG00000044134	ENSMUSG00000044134	5	122109620	122115249	88	89		
4765	12	109956212	110272739	ENSMUSG00000042589	ENSMUSG00000042589	ENSMUSG00000042589	5	122121212	122158972	88	89		
4766	12	109833009	109842766	ENSMUSG00000013936	ENSMUSG00000013936	ENSMUSG00000013936	5	122362366	122367386	88	89		
4767	12	109769194	109829721	ENSMUSG00000043036	ENSMUSG00000043036	ENSMUSG00000043036	5	122368673	122390797	88	89		
4768	12	109570995	109610897	ENSMUSG00000064267	ENSMUSG00000064267	ENSMUSG00000064267	5	122470515	122502913	88	89		
4769	12	109456326	109505506	ENSMUSG00000038582	ENSMUSG00000038582	ENSMUSG00000038582	5	122545015	122584898	88	89		
4770	12	109424519	109453366	ENSMUSG00000038569	ENSMUSG00000038569	ENSMUSG00000038569	5	122586125	122614812	88	89		
4771	12	109414117	109421734	ENSMUSG00000029462	ENSMUSG00000029462	ENSMUSG00000029462	5	122620630	122623539	88	89		
4772	12	109390621	109412553	ENSMUSG00000029463	ENSMUSG00000029463	ENSMUSG00000029463	5	122625203	122632972	88	89		
4773	12	109374675	109390447	ENSMUSG00000029464	ENSMUSG00000029464	ENSMUSG00000029464	5	122632493	122643458	88	89		
4774	12	109296332	109325815	ENSMUSG00000029466	ENSMUSG00000029466	ENSMUSG00000029466	5	122683098	122705196	88	89		
4775	12	109203815	109269504	ENSMUSG00000029467	ENSMUSG00000029467	ENSMUSG00000029467	5	122714131	122762501	88	89		
4776	12	109046687	109140414	ENSMUSG00000029469	ENSMUSG00000029469	ENSMUSG00000029469	5	122810823	122871703	88	89		
4777	12	120055061	120108239	ENSMUSG00000029468	ENSMUSG00000029468	ENSMUSG00000029468	5	122904686	122941921	89	90		
4778	12	120132043	120156290	ENSMUSG00000029470	ENSMUSG00000029470	ENSMUSG00000029470	5	122968201	122989656	89	90		
4779	12	120159880	120220494	ENSMUSG00000029471	ENSMUSG00000029471	ENSMUSG00000029471	5	122993796	123039999	89	90		
4780	12	120230544	120274585	ENSMUSG00000029472	ENSMUSG00000029472	ENSMUSG00000029472	5	123048086	123081882	89	90		
4781	12	120322285	120346538	ENSMUSG00000029474	ENSMUSG00000029474	ENSMUSG00000029474	5	123110810	123129573	89	90		
4782	12	120351285	120503272	ENSMUSG00000029475	ENSMUSG00000029475	ENSMUSG00000029475	5	123131285	123249709	89	90		
4783	12	120548838	120564321	ENSMUSG00000049686	ENSMUSG00000049686	ENSMUSG00000049686	5	123275691	123290773	89	90		
4784	12	120573680	120591943	ENSMUSG00000029477	ENSMUSG00000029477	ENSMUSG00000029477	5	123297750	123307437	89	90		
4785	12	120635041	120700739	ENSMUSG00000054434	ENSMUSG00000054434	ENSMUSG00000054434	5	123336941	123377948	89	90		
4786	12	120700045	120715977	ENSMUSG00000029449	ENSMUSG00000029449	ENSMUSG00000029449	5	123363661	123393013	89	90		
4787	12	120741760	120754944	ENSMUSG00000038384	ENSMUSG00000038384	ENSMUSG00000038384	5	123413235	123428028	89	90		
4788	12	120761818	120781152	ENSMUSG00000029445	ENSMUSG00000029445	ENSMUSG00000029445	5	123432596	123443190	89	90		
4789	12	120811029	120840153	ENSMUSG00000029440	ENSMUSG00000029440	ENSMUSG00000029440	5	123488923	123510734	89	90		
4790	12	120840863	120926201	ENSMUSG00000029442	ENSMUSG00000029442	ENSMUSG00000029442	5	123534362	123587236	89	90		
4791	12	120944244	120984333	ENSMUSG00000029438	ENSMUSG00000029438	ENSMUSG00000029438	5	123605070	123634671	89	90		
4792	12	121001143	121192312	ENSMUSG00000038342	ENSMUSG00000038342	ENSMUSG00000038342	5	123655548	123714131	89	90		
4793	12	121222530	121224699	ENSMUSG00000029437	ENSMUSG00000029437	ENSMUSG00000029437	5	123740928	123742719	89	90		
4794	12	121235017	121253970	ENSMUSG00000063409	ENSMUSG00000063409	ENSMUSG00000063409	5	123749943	123768822	89	90		
4795	12	121254043	121258037	ENSMUSG00000029431	ENSMUSG00000029431	ENSMUSG00000029431	5	123771078	123772500	89	90		
4796	12	121258276	121277973	ENSMUSG00000029433	ENSMUSG00000029433	ENSMUSG00000029433	5	123773129	123784782	89	90		
4797	12	121282049	121316970	ENSMUSG00000029434	ENSMUSG00000029434	ENSMUSG00000029434	5	123789720	123833624	89	90		
4798	12	121321934	121473069	ENSMUSG00000049550	ENSMUSG00000049550	ENSMUSG00000049550	5	123839688	123944909	89	90		
4799	12	121523390	121551340	ENSMUSG00000029427	ENSMUSG00000029427	ENSMUSG00000029427	5	123959771	123981548	89	90		
4800	12	121555226	121577500	ENSMUSG00000029422	ENSMUSG00000029422	ENSMUSG00000029422	5	123989795	124005729	89	90		
4801	12	121577762	121675212	ENSMUSG00000029414	ENSMUSG00000029414	ENSMUSG00000029414	5	124014487	124080578	89	90		
4802	12	121766074	121767237	ENSMUSG00000045502	ENSMUSG00000045502	ENSMUSG00000045502	5	124124974	124126056	89	90		
4803	12	121776684	121781082	ENSMUSG00000049241	ENSMUSG00000049241	ENSMUSG00000049241	5	124139189	124140220	89	90		
4804	12	121804202	121819946	ENSMUSG00000023106	ENSMUSG00000023106	ENSMUSG00000023106	5	124167893	124189450	89	90		
4805	12	121825026	121877704	ENSMUSG00000061882	ENSMUSG00000061882	ENSMUSG00000061882	5	124204851	124227507	89	90		
4806	12	121886051	121913460	ENSMUSG00000000915	ENSMUSG00000000915	ENSMUSG00000000915	5	124234381	124263818	89	90		
4807	12	121915835	121946665	ENSMUSG00000066278	ENSMUSG00000066278	ENSMUSG00000066278	5	124265259	124292878	89	90		
4808	12	121979500	122032149	ENSMUSG00000029408	ENSMUSG00000029408	ENSMUSG00000029408	5	124322151	124356393	89	90		
4809	12	122025720	122030541	ENSMUSG00000023707	ENSMUSG00000023707	ENSMUSG00000023707	5	124372956	124376101	89	90		
4810	12	122030560	122033407	ENSMUSG00000029404	ENSMUSG00000029404	ENSMUSG00000029404	5	124376726	124378813	89	90		
4811	12	122033981	122201238	ENSMUSG00000029406	ENSMUSG00000029406	ENSMUSG00000029406	5	124380365	124447800	89	90		
4812	12	122206901	122272394	ENSMUSG00000038126	ENSMUSG00000038126	ENSMUSG00000038126	5	124515638	124559697	89	90		
4813	12	122283416	122308459	ENSMUSG00000047635	ENSMUSG00000047635	ENSMUSG00000047635	5	124588755	124602454	89	90		
4814	12	122346408	122400941	ENSMUSG00000038095	ENSMUSG00000038095	ENSMUSG00000038095	5	124635147	124675145	89	90		
4815	12	122465541	122486996	ENSMUSG00000029401	ENSMUSG00000029401	ENSMUSG00000029401	5	124723878	124738853	89	90		
4816	12	122508144	122516889	ENSMUSG00000029402	ENSMUSG00000029402	ENSMUSG00000029402	5	124743773	124751742	89	90		
4817	12	122522316	122584020	ENSMUSG00000029392	ENSMUSG00000029392	ENSMUSG00000029392	5	124753698	124792009	89	90		
4818	12	122652625	122671433	ENSMUSG00000029389	ENSMUSG00000029389	ENSMUSG00000029389	5	124813496	124829591	89	90		
4819	12	122672256	122684200	ENSMUSG00000029388	ENSMUSG00000029388	ENSMUSG00000029388	5	124831808	124839157	89	90		
4820	12	122684333	122711573	ENSMUSG00000029387	ENSMUSG00000029387	ENSMUSG00000029387	5	124839783	124858298	89	90		
4821	12	122721615	122758895	ENSMUSG00000029386	ENSMUSG00000029386	ENSMUSG00000029386	5	124859367	124888267	89	90		
4822	12	122762818	122810394	ENSMUSG00000038023	ENSMUSG00000038023	ENSMUSG00000038023	5	124890266	125011627	89	90		
4823	12	122948326	122985978	ENSMUSG00000038011	ENSMUSG00000038011	ENSMUSG00000038011	5	125097789	125111955	89	90		
4824	12	122986936	123023317	ENSMUSG00000037979	ENSMUSG00000037979	ENSMUSG00000037979	5	125123427	125151176	89	90		
4825	12	123345929	123365021	ENSMUSG00000037962	ENSMUSG00000037962	ENSMUSG00000037962	5	125292426	125301525	89	90		
4826	12	123374915	123545750	ENSMUSG00000029478	ENSMUSG00000029478	ENSMUSG00000029478	5	125306858	125413879	89	90		
4827	12	123833952	123914303	ENSMUSG00000037936	ENSMUSG00000037936	ENSMUSG00000037936	5	125570084	125629861	89	90		
4828	12	123997326	124039620	ENSMUSG00000029480	ENSMUSG00000029480	ENSMUSG00000029480	5	125703780	125723424	89	90		
4829	12	124044204	124076998	ENSMUSG00000037905	ENSMUSG00000037905	ENSMUSG00000037905	5	125730944	125831366	89	90		
4830	12	124115878	124193814	ENSMUSG00000029482	ENSMUSG00000029482	ENSMUSG00000029482	5	125765311	125806864	89	90		
4831	12	124672995	124705316	ENSMUSG00000029483	ENSMUSG00000029483	ENSMUSG00000029483	5	126068137	126077526	89	90		
4832	12	127666660	127758407	ENSMUSG00000034324	ENSMUSG00000034324	ENSMUSG00000034324	5	127531010	127853681	89	90		
4833	12	127844527	127874494	ENSMUSG00000029416	ENSMUSG00000029416	ENSMUSG00000029416	5	127884861	127906595	89	90		
4834	12	127904034	128035462	ENSMUSG00000049971	ENSMUSG00000049971	ENSMUSG00000049971	5	127921465	127981105	89	90		
4835	12	128124373	128953837	ENSMUSG00000034310	ENSMUSG00000034310	ENSMUSG00000034310	5	128072694	128747770	89	90		
4836	12	129388567	129422825	ENSMUSG00000029423	ENSMUSG00000029423	ENSMUSG00000029423	5	129050954	129070182	89	90		
4837	12	129446635	129568363	ENSMUSG00000029420	ENSMUSG00000029420	ENSMUSG00000029420	5	129075531	129268194	89	90		
4838	12	129840102	129889764	ENSMUSG00000029428	ENSMUSG00000029428	ENSMUSG00000029428	5	129299275	129323280	89	90		
4839	12	129922521	129927316	ENSMUSG00000029430	ENSMUSG00000029430	ENSMUSG00000029430	5	129334926	129339029	89	90		
4840	12	130004790	130189786	ENSMUSG00000044017	ENSMUSG00000044017	ENSMUSG00000044017	5	129493848	129517264	89	90		
4841	12	130761588	130850235	ENSMUSG00000029439	ENSMUSG00000029439	ENSMUSG00000029439	5	129815939	129886092	89	90		
4842	12	130878891	130902269	ENSMUSG00000029436	ENSMUSG00000029436	ENSMUSG00000029436	5	129898922	129922921	89	90		
4843	7	55831115	55881827	ENSMUSG00000034219	ENSMUSG00000034219	ENSMUSG00000034219	5	129982979	130013770	90	91		
4844	7	55999790	56035365	ENSMUSG00000029432	ENSMUSG00000029432	ENSMUSG00000029432	5	130039807	130072959	90	91		
4845	7	56046240	56086762	ENSMUSG00000029446	ENSMUSG00000029446	ENSMUSG00000029446	5	130080273	130101913	90	91		
4846	7	56099452	56115851	ENSMUSG00000025538	ENSMUSG00000025538	ENSMUSG00000025538	5	130131699	130148660	90	91		
4847	7	56115471	56128121	ENSMUSG00000025537	ENSMUSG00000025537	ENSMUSG00000025537	5	130148130	130163787	90	91		
4848	7	56149869	56151584	ENSMUSG00000025535	ENSMUSG00000025535	ENSMUSG00000025535	5	130193295	130195655	90	91		
4849	7	64975759	65060252	ENSMUSG00000066735	ENSMUSG00000066735	ENSMUSG00000066735	5	130226816	130267594	90	91		
4850	7	65063110	65084635	ENSMUSG00000025534	ENSMUSG00000025534	ENSMUSG00000025534	5	130273725	130287552	90	91		
4851	7	65178211	65195756	ENSMUSG00000025533	ENSMUSG00000025533	ENSMUSG00000025533	5	130296259	130304568	90	91		
4852	7	65217324	65255319	ENSMUSG00000025532	ENSMUSG00000025532	ENSMUSG00000025532	5	130314009	130345492	90	91		
4853	7	65307749	65462864	ENSMUSG00000034118	ENSMUSG00000034118	ENSMUSG00000034118	5	130364337	130414866	90	91		
4854	7	65731487	65913881	ENSMUSG00000025340	ENSMUSG00000025340	ENSMUSG00000025340	5	130471883	130499040	90	91		
4855	7	66023647	66058574	ENSMUSG00000053094	ENSMUSG00000053094	ENSMUSG00000053094	5	130507128	130528367	90	91		
4856	7	66090125	66098023	ENSMUSG00000025337	ENSMUSG00000025337	ENSMUSG00000025337	5	130530438	130540169	90	91		
4857	7	70890696	71381724	ENSMUSG00000060371	ENSMUSG00000060371	ENSMUSG00000060371	5	130654179	131124877	91	91	here	This inversion was ignored by AG
4858	7	70235725	70815067	ENSMUSG00000034040	ENSMUSG00000034040	ENSMUSG00000034040	5	131159663	131592225	91	91	Inversion ignored by AG	2 -> 1
4859	7	68702576	69893918	ENSMUSG00000029673	ENSMUSG00000029673	ENSMUSG00000029673	5	131724142	132826526	91	91		
4860	7	74094229	74127573	ENSMUSG00000061979	ENSMUSG00000061979	ENSMUSG00000061979	5	134432685	134461392	92	92		
4861	7	73709966	73812956	ENSMUSG00000060261	ENSMUSG00000060261	ENSMUSG00000060261	5	134522470	134599357	92	92		
4862	7	73506056	73654846	ENSMUSG00000023079	ENSMUSG00000023079	ENSMUSG00000023079	5	134643280	134741319	92	92		
4863	7	73341741	73458200	ENSMUSG00000063146	ENSMUSG00000063146	ENSMUSG00000063146	5	134776520	134819797	92	92		
4864	7	73283770	73306674	ENSMUSG00000023104	ENSMUSG00000023104	ENSMUSG00000023104	5	134867317	134882955	92	92		
4865	7	73262211	73282093	ENSMUSG00000040751	ENSMUSG00000040751	ENSMUSG00000040751	5	134884892	134899650	92	92		
4866	7	73136092	73174790	ENSMUSG00000029674	ENSMUSG00000029674	ENSMUSG00000029674	5	134940680	134973184	92	92		
4867	7	73080454	73120966	ENSMUSG00000029675	ENSMUSG00000029675	ENSMUSG00000029675	5	134988408	135031868	92	92		
4868	7	72913425	72918156	ENSMUSG00000040576	ENSMUSG00000040576	ENSMUSG00000040576	5	135186221	135191360	92	92		
4869	7	72880010	72884950	ENSMUSG00000008843	ENSMUSG00000008843	ENSMUSG00000008843	5	135199324	135199956	92	92		
4870	7	72886856	72894800	ENSMUSG00000040557	ENSMUSG00000040557	ENSMUSG00000040557	5	135217000	135227264	92	92		
4871	7	72821653	72822315	ENSMUSG00000070473	ENSMUSG00000070473	ENSMUSG00000070473	5	135270841	135272099	92	92		
4872	7	72788363	72791120	ENSMUSG00000040532	ENSMUSG00000040532	ENSMUSG00000040532	5	135293926	135296797	92	92		

4873	7	72751477	72771924	ENSMUSG00000007207	ENSMUSG00000007207	ENSMUSG00000007207	5	135308164	135335725	92	92		
4874	7	72735834	72750477	ENSMUSG00000005378	ENSMUSG00000005378	ENSMUSG00000005378	5	135337586	135348717	92	92		
4875	7	72720110	72724375	ENSMUSG00000043614	ENSMUSG00000043614	ENSMUSG00000043614	5	135357530	135362893	92	92		
4876	7	72645460	72676806	ENSMUSG00000005373	ENSMUSG00000005373	ENSMUSG00000005373	5	135391540	135423011	92	92		
4877	7	72621938	72630908	ENSMUSG00000005374	ENSMUSG00000005374	ENSMUSG00000005374	5	135434317	135447294	92	92		
4878	7	72588622	72609960	ENSMUSG00000029681	ENSMUSG00000029681	ENSMUSG00000029681	5	135453022	135466456	92	92		
4879	7	72492669	72574551	ENSMUSG00000002748	ENSMUSG00000002748	ENSMUSG00000002748	5	135471996	135529194	92	92		
4880	7	72486045	72488386	ENSMUSG00000049551	ENSMUSG00000049551	ENSMUSG00000049551	5	135533878	135535656	92	92		
4881	7	72380299	72410568	ENSMUSG00000040013	ENSMUSG00000040013	ENSMUSG00000040013	5	135576331	135634618	92	92		
4882	7	75001345	75206215	ENSMUSG00000039959	ENSMUSG00000039959	ENSMUSG00000039959	5	135695844	135739772	93	93		
4883	7	75236788	75257150	ENSMUSG00000070464	ENSMUSG00000070464	ENSMUSG00000070464	5	135845075	135848196	93	93		
4884	7	75279050	75280969	ENSMUSG00000004814	ENSMUSG00000004814	ENSMUSG00000004814	5	135855212	135857676	93	93		
4885	7	75346202	75356174	ENSMUSG00000039917	ENSMUSG00000039917	ENSMUSG00000039917	5	135917281	135931003	93	93		
4886	7	75382409	75454108	ENSMUSG00000005514	ENSMUSG00000005514	ENSMUSG00000005514	5	135973785	136019952	93	93		
4887	7	75454238	75461913	ENSMUSG00000039886	ENSMUSG00000039886	ENSMUSG00000039886	5	136020117	136028799	93	93		
4888	7	75463592	75515257	ENSMUSG00000019178	ENSMUSG00000019178	ENSMUSG00000019178	5	136031848	136062865	93	93		
4889	7	75702321	75753093	ENSMUSG00000039860	ENSMUSG00000039860	ENSMUSG00000039860	5	136119677	136157935	93	93		
4890	7	75794053	75826252	ENSMUSG00000051391	ENSMUSG00000051391	ENSMUSG00000051391	5	136193038	136219180	93	93		
4891	7	75856582	75876948	ENSMUSG00000029699	ENSMUSG00000029699	ENSMUSG00000029699	5	136244851	136246896	93	93		
4892	7	75864804	75909320	ENSMUSG00000004948	ENSMUSG00000004948	ENSMUSG00000004948	5	136264732	136273251	93	93		
4893	7	75928954	75973241	ENSMUSG00000004947	ENSMUSG00000004947	ENSMUSG00000004947	5	136279432	136317491	93	93		
4894	7	75977906	75982504	ENSMUSG00000042985	ENSMUSG00000042985	ENSMUSG00000042985	5	136323326	136329926	93	93		
4895	7	101967441	101969172	ENSMUSG00000006143	ENSMUSG00000006143	ENSMUSG00000006143	5	136341938	136348946	94	94		
4896	7	101892385	101900613	ENSMUSG00000029703	ENSMUSG00000029703	ENSMUSG00000029703	5	136407695	136420701	94	94		
4897	7	101883702	101892293	ENSMUSG00000039754	ENSMUSG00000039754	ENSMUSG00000039754	5	136420781	136426239	94	94		
4898	7	101861022	101876168	ENSMUSG00000039747	ENSMUSG00000039747	ENSMUSG00000039747	5	136432091	136455283	94	94		
4899	7	101730555	101748817	ENSMUSG00000005057	ENSMUSG00000005057	ENSMUSG00000005057	5	136502776	136529530	94	94		
4900	7	101246011	101679620	ENSMUSG00000029705	ENSMUSG00000029705	ENSMUSG00000029705	5	136558881	136851889	94	94		
4901	7	101043475	101059118	ENSMUSG00000005474	ENSMUSG00000005474	ENSMUSG00000005474	5	136978805	136985721	94	94		
4902	7	100793034	100987530	ENSMUSG00000004415	ENSMUSG00000004415	ENSMUSG00000004415	5	137026386	137167748	94	94		
4903	7	100743369	100751750	ENSMUSG00000007987	ENSMUSG00000007987	ENSMUSG00000007987	5	137192777	137197871	94	94		
4904	7	100662095	100668821	ENSMUSG00000001739	ENSMUSG00000001739	ENSMUSG00000001739	5	137252496	137260467	94	94		
4905	7	100647705	100654189	ENSMUSG00000059518	ENSMUSG00000059518	ENSMUSG00000059518	5	137267023	137269695	94	94		
4906	7	100635979	100647731	ENSMUSG00000004846	ENSMUSG00000004846	ENSMUSG00000004846	5	137271646	137281164	94	94		
4907	7	100631814	100633276	ENSMUSG00000066703	ENSMUSG00000066703	ENSMUSG00000066703	5	137282792	137283921	94	94		
4908	7	100592517	100595594	ENSMUSG00000037428	ENSMUSG00000037428	ENSMUSG00000037428	5	137314922	137317978	94	94		
4909	7	100584398	100591240	ENSMUSG00000004849	ENSMUSG00000004849	ENSMUSG00000004849	5	137320371	137327869	94	94		
4910	7	100557172	100569026	ENSMUSG00000037411	ENSMUSG00000037411	ENSMUSG00000037411	5	137346135	137356886	94	94		
4911	7	100515440	100521746	ENSMUSG00000043279	ENSMUSG00000043279	ENSMUSG00000043279	5	137395911	137400824	94	94		
4912	7	100325692	100331651	ENSMUSG00000023328	ENSMUSG00000023328	ENSMUSG00000023328	5	137518065	137524251	94	94		
4913	7	100324439	100325065	ENSMUSG00000051502	ENSMUSG00000051502	ENSMUSG00000051502	5	137524436	137525452	94	94		
4914	7	100310671	100324221	ENSMUSG00000037364	ENSMUSG00000037364	ENSMUSG00000037364	5	137525503	137537421	94	94		
4915	7	100302910	100309004	ENSMUSG00000023348	ENSMUSG00000023348	ENSMUSG00000023348	5	137539687	137544029	94	94		
4916	7	100288294	100302567	ENSMUSG00000037344	ENSMUSG00000037344	ENSMUSG00000037344	5	137544346	137563290	94	94		
4917	7	100238123	100263079	ENSMUSG00000029710	ENSMUSG00000029710	ENSMUSG00000029710	5	137579897	137604298	94	94		
4918	7	100156359	100159257	ENSMUSG00000029711	ENSMUSG00000029711	ENSMUSG00000029711	5	137712873	137714972	94	94		
4919	7	100141705	100143053	ENSMUSG00000029715	ENSMUSG00000029715	ENSMUSG00000029715	5	137731227	137732219	94	94		
4920	7	100116108	100125007	ENSMUSG00000029714	ENSMUSG00000029714	ENSMUSG00000029714	5	137748656	137755862	94	94		
4921	7	100109311	100114727	ENSMUSG00000029713	ENSMUSG00000029713	ENSMUSG00000029713	5	137757915	137763022	94	94		
4922	7	100078678	100092007	ENSMUSG00000029712	ENSMUSG00000029712	ENSMUSG00000029712	5	137783343	137799370	94	94		
4923	7	100055975	100077095	ENSMUSG00000029716	ENSMUSG00000029716	ENSMUSG00000029716	5	137799657	137817267	94	94		
4924	7	100047661	100050870	ENSMUSG00000037221	ENSMUSG00000037221	ENSMUSG00000037221	5	137826426	137830828	94	94		
4925	7	100037924	100043730	ENSMUSG00000029718	ENSMUSG00000029718	ENSMUSG00000029718	5	137834897	137841156	94	94		
4926	7	100021947	100036674	ENSMUSG00000029719	ENSMUSG00000029719	ENSMUSG00000029719	5	137842545	137854376	94	94		
4927	7	100010219	100021712	ENSMUSG00000029720	ENSMUSG00000029720	ENSMUSG00000029720	5	137858971	137870290	94	94		
4928	7	99974818	100001205	ENSMUSG00000029722	ENSMUSG00000029722	ENSMUSG00000029722	5	137880626	137914405	94	94		
4929	7	99919486	99930358	ENSMUSG00000045348	ENSMUSG00000045348	ENSMUSG00000045348	5	137960736	137969786	94	94		
4930	7	99902080	99914838	ENSMUSG00000029723	ENSMUSG00000029723	ENSMUSG00000029723	5	137975784	137997991	94	94		
4931	7	99870848	99872030	ENSMUSG00000029725	ENSMUSG00000029725	ENSMUSG00000029725	5	138008706	138009895	94	94		
4932	7	99865190	99869676	ENSMUSG00000029726	ENSMUSG00000029726	ENSMUSG00000029726	5	138011709	138015961	94	94		
4933	7	99837927	99864238	ENSMUSG00000037108	ENSMUSG00000037108	ENSMUSG00000037108	5	138017663	138052409	94	94		
4934	7	99359104	99365179	ENSMUSG00000056966	ENSMUSG00000056966	ENSMUSG00000056966	5	138183597	138192747	94	95	here	This inversion was ignored by our tool
4935	7	99402290	99411623	ENSMUSG00000037053	ENSMUSG00000037053	ENSMUSG00000037053	5	138211324	138220018	94	95	Inversion ignored by ST	1 -> 2
4936	7	99451158	99473368	ENSMUSG00000029729	ENSMUSG00000029729	ENSMUSG00000029729	5	138314872	138337610	94	95		
4937	7	99492566	99500596	ENSMUSG00000037017	ENSMUSG00000037017	ENSMUSG00000037017	5	138346743	138364001	94	95		
4938	7	99505547	99517299	ENSMUSG00000037007	ENSMUSG00000037007	ENSMUSG00000037007	5	138374455	138382687	94	95		
4939	7	99524519	99527756	ENSMUSG00000019494	ENSMUSG00000019494	ENSMUSG00000019494	5	138390885	138393768	94	95		
4940	7	99528340	99536952	ENSMUSG00000029730	ENSMUSG00000029730	ENSMUSG00000029730	5	138394377	138401650	94	95		
4941	7	99537223	99545877	ENSMUSG00000019518	ENSMUSG00000019518	ENSMUSG00000019518	5	138401809	138408471	94	95		
4942	7	99542630	99554915	ENSMUSG00000036980	ENSMUSG00000036980	ENSMUSG00000036980	5	138408405	138416974	94	95		
4943	7	99555197	99561063	ENSMUSG00000036968	ENSMUSG00000036968	ENSMUSG00000036968	5	138417267	138423730	94	95		
4944	7	99584478	99589761	ENSMUSG00000050552	ENSMUSG00000050552	ENSMUSG00000050552	5	138485519	138489172	94	95		
4945	7	99589983	99594244	ENSMUSG00000036948	ENSMUSG00000036948	ENSMUSG00000036948	5	138489448	138493620	94	95		
4946	7	99605166	99612926	ENSMUSG00000029510	ENSMUSG00000029510	ENSMUSG00000029510	5	138503451	138509732	94	95		
4947	7	504489	525557	ENSMUSG00000025856	ENSMUSG00000025856	ENSMUSG00000025856	5	139230621	139248584	95	96		
4948	7	555905	733813	ENSMUSG00000025855	ENSMUSG00000025855	ENSMUSG00000025855	5	139270948	139383333	95	96		
4949	7	732864	792636	ENSMUSG00000025857	ENSMUSG00000025857	ENSMUSG00000025857	5	139423816	139439285	95	96		
4950	7	838688	881065	ENSMUSG00000036817	ENSMUSG00000036817	ENSMUSG00000036817	5	139454310	139503437	95	96		
4951	7	882717	902597	ENSMUSG00000025858	ENSMUSG00000025858	ENSMUSG00000025858	5	139506017	139523692	95	96		
4952	7	904066	960811	ENSMUSG00000056413	ENSMUSG00000056413	ENSMUSG00000056413	5	139526500	139559804	95	96		
4953	7	972328	981696	ENSMUSG00000045438	ENSMUSG00000045438	ENSMUSG00000045438	5	139591458	139598851	95	96		
4954	7	989361	995778	ENSMUSG00000029541	ENSMUSG00000029541	ENSMUSG00000029541	5	139606283	139610669	95	96		
4955	7	1003169	1144419	ENSMUSG00000053553	ENSMUSG00000053553	ENSMUSG00000053553	5	139613382	139714155	95	96		
4956	7	1050769	1065410	ENSMUSG00000044197	ENSMUSG00000044197	ENSMUSG00000044197	5	139634305	139650053	95	96		
4957	7	1097891	1099399	ENSMUSG00000053647	ENSMUSG00000053647	ENSMUSG00000053647	5	139676821	139681430	95	96		
4958	7	1159070	1166324	ENSMUSG00000053581	ENSMUSG00000053581	ENSMUSG00000053581	5	139724858	139738059	95	96		
4959	7	1239180	1243139	ENSMUSG00000029546	ENSMUSG00000029546	ENSMUSG00000029546	5	139797551	139801821	95	96		
4960	7	1440758	1465527	ENSMUSG00000036718	ENSMUSG00000036718	ENSMUSG00000036718	5	139959310	139988634	95	96		
4961	7	1476742	1510122	ENSMUSG00000029547	ENSMUSG00000029547	ENSMUSG00000029547	5	140003757	140028127	95	96		
4962	7	1536894	1549205	ENSMUSG00000018143	ENSMUSG00000018143	ENSMUSG00000018143	5	140044010	140055126	95	96		
4963	7	1573498	1576155	ENSMUSG00000029551	ENSMUSG00000029551	ENSMUSG00000029551	5	140076093	140079359	95	96		
4964	7	1750759	1753245	ENSMUSG00000048988	ENSMUSG00000048988	ENSMUSG00000048988	5	140160417	140227196	95	96		
4965	7	1821956	2236304	ENSMUSG00000029554	ENSMUSG00000029554	ENSMUSG00000029554	5	140261163	140574024	95	96		
4966	7	2240392	2248330	ENSMUSG00000029557	ENSMUSG00000029557	ENSMUSG00000029557	5	140580812	140583748	95	96		
4967	7	2249153	2257306	ENSMUSG00000036639	ENSMUSG00000036639	ENSMUSG00000036639	5	140584385	140590609	95	96		
4968	7	2261167	2320625	ENSMUSG00000029560	ENSMUSG00000029560	ENSMUSG00000029560	5	140592773	140641736	95	96		
4969	7	2361027	2386603	ENSMUSG00000056076	ENSMUSG00000056076	ENSMUSG00000056076	5	140671793	140695834	95	96		
4970	7	2409733	2440219	ENSMUSG00000036599	ENSMUSG00000036599	ENSMUSG00000036599	5	140758102	140777712	95	96		
4971	7	2526022	2534587	ENSMUSG00000029570	ENSMUSG00000029570	ENSMUSG00000029570	5	140859815	140868017	95	96		
4972	7	2638129	2670953	ENSMUSG00000036565	ENSMUSG00000036565	ENSMUSG00000036565	5	140873060	140901505	95	96		
4973	7	2565158	2617062	ENSMUSG00000036555	ENSMUSG00000036555	ENSMUSG00000036555	5	140915979	140954852	95	96		
4974	7	2685689	2721587	ENSMUSG00000050022	ENSMUSG00000050022	ENSMUSG00000050022	5	140976601	141005784	95	96		
4975	7	2734271	2850485	ENSMUSG00000000149	ENSMUSG00000000149	ENSMUSG00000000149	5	141012418	141082905	95	96		
4976	7	2912309	3050025	ENSMUSG00000036526	ENSMUSG00000036526	ENSMUSG00000036526	5	141125799	141165891	95	96		
4977	7	3307745	4271542	ENSMUSG00000039683	ENSMUSG00000039683	ENSMUSG00000039683	5	141494323	142464649	95	96		
4978	7	4688456	4777600	ENSMUSG00000056493	ENSMUSG00000056493	ENSMUSG00000056493	5	142653991	142714484	95	96		
4979	7	4781809	4800552	ENSMUSG00000039623	ENSMUSG00000039623	ENSMUSG00000039623	5	142716405	142731189	95	96		
4980	7	4803212	4889811	ENSMUSG00000029576	ENSMUSG00000029576	ENSMUSG00000029576	5	142737316	142797503	95	96		
4981	7	4912147	4965316	ENSMUSG00000039533	ENSMUSG00000039533	ENSMUSG00000039533	5	142817212	142832738	95	96		
4982	7	5196361	5240012	ENSMUSG00000029578	ENSMUSG00000029578	ENSMUSG00000029578	5	142882057	142921857	95	96		
4983	7	5289101	5310216	ENSMUSG00000050822	ENSMUSG00000050822	ENSMUSG00000050822	5	142958132	142976132	95	96		
4984	7	5495406	5519908	ENSMUSG00000066640	ENSMUSG00000066640	ENSMUSG00000066640	5	143136929	143160357	95	96		
4985	7	5533313	5535814	ENSMUSG00000029580	ENSMUSG00000029580	ENSMUSG00000029580	5	143168256	143171864	95	96		
4986	7	5598980	5611631	ENSMUSG00000029581	ENSMUSG00000029581	ENSMUSG00000029581	5	143225496	143238322	95	96		
4987	7	5629017	5787818	ENSMUSG00000045078	ENSMUSG00000045078	ENSMUSG00000045078	5	143257789	143378134	95	96		
4988	7	5052079	5073323	ENSMUSG00000061898	ENSMUSG00000061898	ENSMUSG00000061898	5	143437326	143445915	95	96		
4989	7	6696844	6704186	ENSMUSG00000029587	ENSMUSG00000029587	ENSMUSG00000029587	5	143500392	143513090	95	97	here	This inversion was ignored by our tool
4990	7	6648566	6660767	ENSMUSG00000046658	ENSMUSG00000046658	ENSMUSG00000046658	5	143516867	143529793	95	97	Inversion ignored by ST	1 -> 2
4991	7	6596301	6614880	ENSMUSG00000039244	ENSMUSG00000039244	ENSMUSG00000039244	5	143566348	143580500	95	97		
4992	7	6583590	6595087	ENSMUSG00000001844	ENSMUSG00000001844	ENSMUSG00000001844	5	143581631	143594366	95	97		
4993	7	6415286	6454108	ENSMUSG00000039206	ENSMUSG00000039206	ENSMUSG00000039206	5	143728564	143761560	95	97		
4994	7	6167939	6278800	ENSMUSG00000018001	ENSMUSG00000018001	ENSMUSG00000018001	5	143878086	143965865	95	97		
4995	7	6111102	6167717	ENSMUSG00000051306	ENSMUSG00000051306	ENSMUSG00000051306	5	143969150	143983636	95	97		
4996	7	6037533	6042537	ENSMUSG00000029607	ENSMUSG00000029607	ENSMUSG00000029607	5	144144270	144148557	95	97		
4997	7	6015408	6029991	ENSMUSG00000029610	ENSMUSG00000029610	ENSMUSG00000029610	5	144156232	144163319	95	97		
4998	7	97574133	97672983	ENSMUSG00000038970	ENSMUSG00000038970	ENSMUSG00000038970	5	144427737	144441726	96	98		
4999	7	97679540	97680127	ENSMUSG00000052271	ENSMUSG00000052271	ENSMUSG00000052271	5	144443813	144447968	96	98		
5000	7	97683993	97719404	ENSMUSG00000066621	ENSMUSG00000066621	ENSMUSG00000066621	5	144448878	144477105	96	98		
5001	7	97748923	97758775	ENSMUSG00000047843	ENSMUSG00000047843	ENSMUSG00000047843	5	144508763	144518100	96	98		
5002	7	97760007	97868316	ENSMUSG00000038859	ENSMUSG00000038859	ENSMUSG00000038859	5	144519484	144578159	96	98		
5003	7	98084540	98097117	ENSMUSG00000059991	ENSMUSG00000059991	ENSMUSG00000059991	5	144799523	144810158	96	98		
5004	7	98282050	98305579	ENSMUSG00000043388	ENSMUSG00000043388	ENSMUSG00000043388	5	144989442	145015105	96	98		
5005	7	98314129	98448800	ENSMUSG00000045482	ENSMUSG00000045482	ENSMUSG00000045482	5	145024677	145112482	96	98		
5006	7	98463000	98579659	ENSMUSG00000038780	ENSMUSG00000038780	ENSMUSG00000038780	5	145132884	145156109	96	98		
5007	7	98844537	98855175	ENSMUSG00000038722	ENSMUSG00000038722	ENSMUSG00000038722	5	145393924	145401601	96	98		
5008	7	98852298	98874306	ENSMUSG00000029624	ENSMUSG00000029624	ENSMUSG00000029624	5	145401053	145420582	96	98		
5009	7	98874632	98892930	ENSMUSG00000029625	ENSMUSG00000029625	ENSMUSG00000029625	5	145420751	145435567	96	98		
5010	7	98928800	98935652	ENSMUSG00000029627	ENSMUSG00000029627	ENSMUSG00000029627	5	145448476	145455394	96	98		
5011	7	98940232	98969380	ENSMUSG00000055991	ENSMUSG00000055991	ENSMUSG00000055991	5	145458089	145474676	96	98		
5012	7	98993981	99012008	ENSMUSG00000007812	ENSMUSG00000007812	ENSMUSG00000007812	5	145485250	145500829	96	98		
5013	13	25684905	25694508	ENSMUSG00000029634	ENSMUSG00000029634	ENSMUSG00000029634	5	146513438	146525671	97	99		
5014	13	25726276	25877375	ENSMUSG00000029635	ENSMUSG00000029635	ENSMUSG00000029635	5	146535913	146607053	97	99		
5015	13	26029840	26161080	ENSMUSG00000029636	ENSMUSG00000029636	ENSMUSG00000029636	5	146689240	146774650	97	99		
5016	13	26230447	26232108	ENSMUSG00000041468	ENSMUSG00000041468	ENSMUSG00000041468	5	146892499	146895588	97	99		
5017	13	26742464	26745827	ENSMUSG00000029641	ENSMUSG00000029641	ENSMUSG00000029641	5	147155467	147158075	97	99		
5018	13	26896681	26907823	ENSMUSG00000016503	ENSMUSG00000016503	ENSMUSG00000016503	5	147259030	147265952	97	99		
5019	13	26907780	26922739	ENSMUSG00000016510	ENSMUSG00000016510	ENSMUSG00000016510	5	147261923	147274100	97	99		
5020	13	27018048	27053940	ENSMUSG00000016520	ENSMUSG00000016520	ENSMUSG00000016520	5	147327452	147386878	97	99		
5021	13	27092903	27095589	ENSMUSG00000029642	ENSMUSG00000029642	ENSMUSG00000029642	5	147387890	147389429	97	99		
5022	13	27264780	27266089	ENSMUSG00000053129	ENSMUSG00000053129	ENSMUSG00000053129	5	147499045	147501296	97	99		
5023	13	27392157	27397394	ENSMUSG00000029644	ENSMUSG00000029644	ENSMUSG00000029644	5	147580588	147585171	97	99		
5024	13	27434273	27441317	ENSMUSG00000029646	ENSMUSG00000029646	ENSMUSG00000029646	5	147611249	147617598	97	99		
5025	13	27450252	27460774	ENSMUSG00000075543	ENSMUSG00000075543	ENSMUSG00000075543	5	147625453	147632680	97	99		
5026	13	27475757	27572729	ENSMUSG00000042817	ENSMUSG00000042817	ENSMUSG00000042817	5	147641520	147710644	97	99		
5027	13	27646417	27764659	ENSMUSG00000029647	ENSMUSG00000029647	ENSMUSG00000029647	5	147761010	147856086	97	99		
5028	13	27773790	27967232	ENSMUSG00000029648	ENSMUSG00000029648	ENSMUSG00000029648	5	147872545	148036337	97	99		
5029	13	28131251	28151059	ENSMUSG00000029649	ENSMUSG00000029649	ENSMUSG00000029649	5	148172902	148185880	97	99		
5030	13	28172854	28191107	ENSMUSG00000029650	ENSMUSG00000029650	ENSMUSG00000029650	5	148188801	148205164	97	99		
5031	13	28496748	28975870	ENSMUSG00000029651	ENSMUSG00000029651	ENSMUSG00000029651	5	148267669	148624431	97	99		
5032	13	28981547	29067721	ENSMUSG00000041313	ENSMUSG00000041313	ENSMUSG00000041313	5	148638675	148710228	97	99		
5033	13	29236508	29322821	ENSMUSG00000001687	ENSMUSG00000001687	ENSMUSG00000001687	5	148816369	148822351	97	99		
5034	13	29674768	29779584	ENSMUSG00000041298	ENSMUSG00000041298	ENSMUSG00000041298	5	149186415	149238972	97	99		
5035	13	30089920	30131686	ENSMUSG00000041264	ENSMUSG00000041264	ENSMUSG00000041264	5	149495015	149525610	97	99		
5036	13	30207645	30236556	ENSMUSG00000060063	ENSMUSG00000060063	ENSMUSG00000060063	5	149575459	149596995	97	99		
5037	13	30378328	30397709	ENSMUSG00000029659	ENSMUSG00000029659	ENSMUSG00000029659	5	149680185	149700082	97	99		
5038	13	30404840	30447639	ENSMUSG00000029660	ENSMUSG00000029660	ENSMUSG00000029660	5	149708079	149739358	97	99		
5039	13	30608765	30634117	ENSMUSG00000029657	ENSMUSG00000029657	ENSMUSG00000029657	5	149882666	149904552	97	99		
5040	13	30672132	30803657	ENSMUSG00000051950	ENSMUSG00000051950	ENSMUSG00000051950	5	149973176	150027982	97	99		
5041	13	31211674	31275009	ENSMUSG00000053368	ENSMUSG00000053368	ENSMUSG00000053368	5	150287054	150349715	97	99		
5042	13	31503437	31768749	ENSMUSG00000056602	ENSMUSG00000056602	ENSMUSG00000056602	5	150578645	150764801	97	99		
5043	13	31776016	31783894	ENSMUSG00000056586	ENSMUSG00000056586	ENSMUSG00000056586	5	150775448	150786367	97	99		
5044	13	31787617	31871806	ENSMUSG00000041147	ENSMUSG00000041147	ENSMUSG00000041147	5	150791023	150838107	97	99		
5045	13	31873810	31900270	ENSMUSG00000041132	ENSMUSG00000041132	ENSMUSG00000041132	5	150840023	150862879	97	99		
5046	13	32058624	32250157	ENSMUSG00000034021	ENSMUSG00000034021	ENSMUSG00000034021	5	150942206	151077056	97	99		
5047	13	32488571	32538282	ENSMUSG00000058488	ENSMUSG00000058488	ENSMUSG00000058488	5	151221078	151262196	97	99		
5048	13	32575307	32757892	ENSMUSG00000016128	ENSMUSG00000016128	ENSMUSG00000016128	5	151306007	151458573	97	99		
5049	13	33290203	33309633	ENSMUSG00000033970	ENSMUSG00000033970	ENSMUSG00000033970	5	151911205	151919558	97	99		
5050	7	92597304	92615616	ENSMUSG00000047735	ENSMUSG00000047735	ENSMUSG00000047735	6	3322520	3327268	98	100		
5051	7	92655836	92686806	ENSMUSG00000044156	ENSMUSG00000044156	ENSMUSG00000044156	6	3413257	3444478	98	100		
5052	7	92699640	92826119	ENSMUSG00000001376	ENSMUSG00000001376	ENSMUSG00000001376	6	3448405	3553307	98	100		
5053	7	92891740	93041686	ENSMUSG00000023964	ENSMUSG00000023964	ENSMUSG00000023964	6	3637395	3672973	98	100		
5054	7	93352663	93358001	ENSMUSG00000029664	ENSMUSG00000029664	ENSMUSG00000029664	6	3912585	3918357	98	100		
5055	7	93373756	93378421	ENSMUSG00000029663	ENSMUSG00000029663	ENSMUSG00000029663	6	3944012	3947436	98	100		
5056	7	93388974	93393762	ENSMUSG00000032766	ENSMUSG00000032766	ENSMUSG00000032766	6	3953987	3958445	98	100		
5057	7	93430021	93471581	ENSMUSG00000032757	ENSMUSG00000032757	ENSMUSG00000032757	6	4026904	4036927	98	100		
5058	7	93861809	93898480	ENSMUSG00000029661	ENSMUSG00000029661	ENSMUSG00000029661	6	4455840	4490729	98	100		
5059	7	93977120	94024215	ENSMUSG00000015189	ENSMUSG00000015189	ENSMUSG00000015189	6	4551066	4593381	98	100		
5060	7	94052480	94123414	ENSMUSG00000004631	ENSMUSG00000004631	ENSMUSG00000004631	6	4624582	4669506	98	100		
5061	7	94377362	94760677	ENSMUSG00000032827	ENSMUSG00000032827	ENSMUSG00000032827	6	4853320	5115661	98	100		
5062	7	94764924	94791780	ENSMUSG00000002588	ENSMUSG00000002588	ENSMUSG00000002588	6	5118105	5143824	98	100		
5063	7	94827209	94863609	ENSMUSG00000029759	ENSMUSG00000029759	ENSMUSG00000029759	6	5170852	5206233	98	100		
5064	7	94872111	94902320	ENSMUSG00000032667	ENSMUSG00000032667	ENSMUSG00000032667	6	5214624	5248373	98	100		
5065	7	94953220	95005007	ENSMUSG00000042607	ENSMUSG00000042607	ENSMUSG00000042607	6	5333386	5383022	98	100		
5066	7	95050753	95063739	ENSMUSG00000019577	ENSMUSG00000019577	ENSMUSG00000019577	6	5433351	5446309	98	100		
5067	7	95239838	95565660	ENSMUSG00000029757	ENSMUSG00000029757	ENSMUSG00000029757	6	5675808	5978039	98	100		

5068	7	95587469	95789341	ENSMUSG00000015112	ENSMUSG00000015112	ENSMUSG00000015112	6	5992146	6141317	98	100
5069	7	96156019	96177139	ENSMUSG00000042541	ENSMUSG00000042541	ENSMUSG00000042541	6	6508283	6528624	98	100
5070	7	96473226	96478288	ENSMUSG00000029754	ENSMUSG00000029754	ENSMUSG00000029754	6	6813797	6817970	98	100
5071	7	96487648	96492079	ENSMUSG00000029755	ENSMUSG00000029755	ENSMUSG00000029755	6	6827801	6832068	98	100
5072	7	96584972	96649007	ENSMUSG00000042505	ENSMUSG00000042505	ENSMUSG00000042505	6	6906027	6991770	98	100
5073	7	97199311	97207696	ENSMUSG00000061762	ENSMUSG00000061762	ENSMUSG00000061762	6	7505071	7512973	98	100
5074	7	97319379	97339732	ENSMUSG00000029752	ENSMUSG00000029752	ENSMUSG00000029752	6	7625170	7643199	98	100
5075	7	7240414	7250505	ENSMUSG00000042460	ENSMUSG00000042460	ENSMUSG00000042460	6	7795333	7822047	99	101
5076	7	7578632	7613376	ENSMUSG00000042447	ENSMUSG00000042447	ENSMUSG00000042447	6	8164806	8186274	99	101
5077	7	8119940	8268693	ENSMUSG00000062995	ENSMUSG00000062995	ENSMUSG00000062995	6	8580530	8728107	99	101
5078	7	8757163	8757924	ENSMUSG00000046178	ENSMUSG00000046178	ENSMUSG00000046178	6	9197085	9197846	99	101
5079	7	10980041	11175767	ENSMUSG00000029629	ENSMUSG00000029629	ENSMUSG00000029629	6	11875915	11958074	99	101
5080	7	11380787	11642839	ENSMUSG00000032625	ENSMUSG00000032625	ENSMUSG00000032625	6	12267367	12699022	99	101
5081	7	12220953	12243410	ENSMUSG00000029571	ENSMUSG00000029571	ENSMUSG00000029571	6	13019842	13035060	99	101
5082	7	12337036	12376543	ENSMUSG00000044476	ENSMUSG00000044476	ENSMUSG00000044476	6	13108049	13137260	99	101
5083	7	112193032	112217684	ENSMUSG00000029569	ENSMUSG00000029569	ENSMUSG00000029569	6	13530687	13558063	100	102
5084	7	112246439	112366963	ENSMUSG00000042742	ENSMUSG00000042742	ENSMUSG00000042742	6	13575937	13627966	100	102
5085	7	112507704	112515015	ENSMUSG00000048216	ENSMUSG00000048216	ENSMUSG00000048216	6	13785071	13789848	100	102
5086	7	113301622	113346300	ENSMUSG00000042717	ENSMUSG00000042717	ENSMUSG00000042717	6	14663582	14705246	100	102
5087	7	113842288	114117219	ENSMUSG00000029563	ENSMUSG00000029563	ENSMUSG00000029563	6	15135506	15391977	100	102
5088	7	114349445	114446492	ENSMUSG00000041390	ENSMUSG00000041390	ENSMUSG00000041390	6	15670694	15752165	100	102
5089	7	115362438	115458034	ENSMUSG00000029553	ENSMUSG00000029553	ENSMUSG00000029553	6	16783373	16848441	100	102
5090	7	115926680	115935830	ENSMUSG00000000058	ENSMUSG00000000058	ENSMUSG00000000058	6	17231310	17239004	100	102
5091	7	115952075	115988466	ENSMUSG00000007655	ENSMUSG00000007655	ENSMUSG00000007655	6	17256370	17291324	100	102
5092	7	116099695	116223632	ENSMUSG00000009376	ENSMUSG00000009376	ENSMUSG00000009376	6	17441241	17521823	100	102
5093	7	116289799	116346548	ENSMUSG00000015733	ENSMUSG00000015733	ENSMUSG00000015733	6	17587098	17616534	100	102
5094	7	116380617	116650270	ENSMUSG00000029534	ENSMUSG00000029534	ENSMUSG00000029534	6	17644032	17892966	100	102
5095	7	116704518	116750579	ENSMUSG00000010797	ENSMUSG00000010797	ENSMUSG00000010797	6	17938940	17980356	100	102
5096	7	116790512	116854779	ENSMUSG00000010796	ENSMUSG00000010796	ENSMUSG00000010796	6	18000972	18059055	100	102
5097	7	116907253	117095951	ENSMUSG00000041301	ENSMUSG00000041301	ENSMUSG00000041301	6	18120687	18272767	100	102
5098	7	117611455	117620114	ENSMUSG00000044155	ENSMUSG00000044155	ENSMUSG00000044155	6	18798636	18804053	100	102
5099	7	117652172	117664214	ENSMUSG00000029517	ENSMUSG00000029517	ENSMUSG00000029517	6	18816319	18829583	100	102
5100	7	119701923	120175148	ENSMUSG00000060882	ENSMUSG00000060882	ENSMUSG00000060882	6	21166300	21677343	100	102
5101	7	120214612	120285413	ENSMUSG00000029669	ENSMUSG00000029669	ENSMUSG00000029669	6	21721396	21802516	100	102
5102	7	120378053	120402938	ENSMUSG00000029670	ENSMUSG00000029670	ENSMUSG00000029670	6	21899647	21926038	100	102
5103	7	120416032	120724730	ENSMUSG00000062980	ENSMUSG00000062980	ENSMUSG00000062980	6	21967067	22190003	100	102
5104	7	120756326	120768393	ENSMUSG00000029671	ENSMUSG00000029671	ENSMUSG00000029671	6	22238231	22248523	100	102
5105	7	121300643	121489324	ENSMUSG00000068748	ENSMUSG00000068748	ENSMUSG00000068748	6	22825828	23002110	100	102
5106	7	121503532	121571504	ENSMUSG00000029695	ENSMUSG00000029695	ENSMUSG00000029695	6	23022171	23082975	100	102
5107	7	121729287	121731835	ENSMUSG00000029697	ENSMUSG00000029697	ENSMUSG00000029697	6	23195051	23198075	100	102
5108	7	121746687	122313627	ENSMUSG00000017978	ENSMUSG00000017978	ENSMUSG00000017978	6	23212836	23789139	100	102
5109	7	122125078	122126208	ENSMUSG00000051956	ENSMUSG00000051956	ENSMUSG00000051956	6	23598871	23600307	100	102
5110	7	122422049	122422924	ENSMUSG00000043865	ENSMUSG00000043865	ENSMUSG00000043865	6	23919163	23920062	100	102
5111	7	122540825	122627261	ENSMUSG00000029700	ENSMUSG00000029700	ENSMUSG00000029700	6	24038296	24118094	100	102
5112	7	122879950	122961879	ENSMUSG00000046192	ENSMUSG00000046192	ENSMUSG00000046192	6	24394870	24465066	100	102
5113	7	123036313	123064322	ENSMUSG00000029685	ENSMUSG00000029685	ENSMUSG00000029685	6	24506082	24523174	100	102
5114	7	123109237	123176352	ENSMUSG00000029684	ENSMUSG00000029684	ENSMUSG00000029684	6	24563813	24614981	100	102
5115	7	123295564	123304445	ENSMUSG00000029680	ENSMUSG00000029680	ENSMUSG00000029680	6	24698356	24717672	100	102
5116	7	124173352	124192917	ENSMUSG00000039904	ENSMUSG00000039904	ENSMUSG00000039904	6	25618118	25640739	100	102
5117	7	125866409	126670494	ENSMUSG00000024211	ENSMUSG00000024211	ENSMUSG00000024211	6	27225655	28076135	100	102
5118	7	126797592	126820003	ENSMUSG00000039841	ENSMUSG00000039841	ENSMUSG00000039841	6	28190250	28347985	100	102
5119	7	127007919	127012890	ENSMUSG00000029708	ENSMUSG00000029708	ENSMUSG00000029708	6	28366701	28371734	100	102
5120	7	127015659	127018995	ENSMUSG00000020440	ENSMUSG00000020440	ENSMUSG00000020440	6	28373650	28376509	100	102
5121	7	127020925	127029079	ENSMUSG00000029707	ENSMUSG00000029707	ENSMUSG00000029707	6	28378026	28386213	100	102
5122	7	127037582	127043016	ENSMUSG00000029706	ENSMUSG00000029706	ENSMUSG00000029706	6	28392388	28396789	100	102
5123	7	127079438	127519894	ENSMUSG00000001424	ENSMUSG00000001424	ENSMUSG00000001424	6	28430358	28838842	100	102
5124	7	127454360	127458238	ENSMUSG00000049939	ENSMUSG00000049939	ENSMUSG00000049939	6	28779666	28781624	100	102
5125	7	127668567	127684917	ENSMUSG00000059201	ENSMUSG00000059201	ENSMUSG00000059201	6	29010231	29023886	100	102
5126	7	127737673	127771198	ENSMUSG00000029701	ENSMUSG00000029701	ENSMUSG00000029701	6	29075168	29114679	100	102
5127	7	127820297	127837191	ENSMUSG00000003500	ENSMUSG00000003500	ENSMUSG00000003500	6	29150447	29166360	100	102
5128	7	127883181	127885706	ENSMUSG00000043421	ENSMUSG00000043421	ENSMUSG00000043421	6	29222680	29225454	100	102
5129	7	128142679	128158897	ENSMUSG00000039742	ENSMUSG00000039742	ENSMUSG00000039742	6	29269209	29284186	100	102
5130	7	128166653	128197866	ENSMUSG00000029767	ENSMUSG00000029767	ENSMUSG00000029767	6	29298191	29326677	100	102
5131	7	128199783	128203087	ENSMUSG00000058831	ENSMUSG00000058831	ENSMUSG00000058831	6	29326667	29330490	100	102
5132	7	128219335	128249419	ENSMUSG00000029769	ENSMUSG00000029769	ENSMUSG00000029769	6	29349153	29372099	100	102
5133	7	128257719	128286568	ENSMUSG00000068699	ENSMUSG00000068699	ENSMUSG00000068699	6	29383469	29411216	100	102
5134	7	128317523	128337966	ENSMUSG00000059022	ENSMUSG00000059022	ENSMUSG00000059022	6	29432046	29457947	100	102
5135	7	128365230	128377320	ENSMUSG00000029771	ENSMUSG00000029771	ENSMUSG00000029771	6	29476743	29487329	100	102
5136	7	128384510	128482173	ENSMUSG00000012535	ENSMUSG00000012535	ENSMUSG00000012535	6	29490835	29559617	100	102
5137	7	128571946	128595907	ENSMUSG00000001763	ENSMUSG00000001763	ENSMUSG00000001763	6	29644266	29668572	100	102
5138	7	128615949	128640617	ENSMUSG00000001761	ENSMUSG00000001761	ENSMUSG00000001761	6	29685513	29711369	100	102
5139	7	128652100	128857287	ENSMUSG00000029772	ENSMUSG00000029772	ENSMUSG00000029772	6	29718502	29862320	100	102
5140	7	128861536	128915473	ENSMUSG00000039629	ENSMUSG00000039629	ENSMUSG00000039629	6	29867023	29909691	100	102
5141	7	129038833	129183090	ENSMUSG00000058440	ENSMUSG00000058440	ENSMUSG00000058440	6	30039899	30094865	100	102
5142	7	129445740	129478442	ENSMUSG00000039130	ENSMUSG00000039130	ENSMUSG00000039130	6	30316398	30341020	100	102
5143	7	129591790	129632519	ENSMUSG00000029782	ENSMUSG00000029782	ENSMUSG00000029782	6	30431243	30458812	100	102
5144	7	129634954	129644733	ENSMUSG00000029784	ENSMUSG00000029784	ENSMUSG00000029784	6	30462314	30469973	100	102
5145	7	129693939	129716870	ENSMUSG00000071553	ENSMUSG00000071553	ENSMUSG00000071553	6	30491652	30514486	100	102
5146	7	129720229	129751249	ENSMUSG00000039070	ENSMUSG00000039070	ENSMUSG00000039070	6	30518379	30541757	100	102
5147	7	129771892	129795807	ENSMUSG00000029788	ENSMUSG00000029788	ENSMUSG00000029788	6	30561020	30581531	100	102
5148	7	129807468	129815165	ENSMUSG00000054446	ENSMUSG00000054446	ENSMUSG00000054446	6	30589223	30595365	100	102
5149	7	129823611	129868133	ENSMUSG00000029790	ENSMUSG00000029790	ENSMUSG00000029790	6	30603467	30643692	100	102
5150	7	129919167	129933365	ENSMUSG00000051855	ENSMUSG00000051855	ENSMUSG00000051855	6	30688063	30698465	100	102
5151	7	130004028	130022808	ENSMUSG00000039032	ENSMUSG00000039032	ENSMUSG00000039032	6	30827142	30845736	100	102
5152	7	130068429	130069400	ENSMUSG00000073209	ENSMUSG00000073209	ENSMUSG00000073209	6	30887970	30888860	100	102
5153	7	130663175	130831930	ENSMUSG00000025609	ENSMUSG00000025609	ENSMUSG00000025609	6	31328898	31446974	100	102
5154	7	130835563	130891908	ENSMUSG00000025608	ENSMUSG00000025608	ENSMUSG00000025608	6	31449656	31494074	100	102
5155	7	131465778	131824668	ENSMUSG00000029765	ENSMUSG00000029765	ENSMUSG00000029765	6	32080285	32518350	100	102
5156	7	132120170	132417390	ENSMUSG00000053768	ENSMUSG00000053768	ENSMUSG00000053768	6	32722385	32990381	100	102
5157	7	132588363	133401051	ENSMUSG00000029763	ENSMUSG00000029763	ENSMUSG00000029763	6	33179308	33903101	100	102
5158	7	133462645	133599473	ENSMUSG00000056215	ENSMUSG00000056215	ENSMUSG00000056215	6	33959606	34064192	100	102
5159	7	133624632	133652343	ENSMUSG00000018999	ENSMUSG00000018999	ENSMUSG00000018999	6	34079089	34107212	100	102
5160	7	133982095	134015105	ENSMUSG00000038871	ENSMUSG00000038871	ENSMUSG00000038871	6	34406514	34435768	100	102
5161	7	134114711	134306010	ENSMUSG00000029761	ENSMUSG00000029761	ENSMUSG00000029761	6	34616156	34701283	100	102
5162	7	134321802	134451161	ENSMUSG00000038836	ENSMUSG00000038836	ENSMUSG00000038836	6	34711027	34735783	100	102
5163	7	134483364	134501190	ENSMUSG00000057137	ENSMUSG00000057137	ENSMUSG00000057137	6	34793298	34805098	100	102
5164	7	134501121	134504130	ENSMUSG00000046806	ENSMUSG00000046806	ENSMUSG00000046806	6	34801923	34808173	100	102
5165	7	134520525	134546811	ENSMUSG00000058486	ENSMUSG00000058486	ENSMUSG00000058486	6	34810578	34840983	100	102
5166	7	134567271	134593784	ENSMUSG00000029848	ENSMUSG00000029848	ENSMUSG00000029848	6	34850315	34869494	100	102
5167	7	134697975	134845365	ENSMUSG00000038784	ENSMUSG00000038784	ENSMUSG00000038784	6	34975105	35063856	100	102
5168	7	134893228	134984029	ENSMUSG00000038759	ENSMUSG00000038759	ENSMUSG00000038759	6	35107573	35177742	100	102
5169	7	135016529	135063492	ENSMUSG00000029843	ENSMUSG00000029843	ENSMUSG00000029843	6	35198110	35238278	100	102
5170	7	135064895	135083987	ENSMUSG00000047420	ENSMUSG00000047420	ENSMUSG00000047420	6	35242898	35256293	100	102
5171	7	135262051	135312647	ENSMUSG00000029840	ENSMUSG00000029840	ENSMUSG00000029840	6	35439058	35469939	100	102
5172	7	136203956	136355539	ENSMUSG00000045613	ENSMUSG00000045613	ENSMUSG00000045613	6	36453362	36454762	100	102
5173	7	136562637	136679086	ENSMUSG00000029838	ENSMUSG00000029838	ENSMUSG00000029838	6	36645815	36741513	100	102
5174	7	136724103	137182148	ENSMUSG00000038665	ENSMUSG00000038665	ENSMUSG00000038665	6	36946756	37229554	100	102
5175	7	137210267	137337113	ENSMUSG00000038648	ENSMUSG00000038648	ENSMUSG00000038648	6	37260627	37371747	100	102
5176	7	137411736	137453590	ENSMUSG00000038641	ENSMUSG00000038641	ENSMUSG00000038641	6	37459781	37498016	100	102
5177	7	137795619	137920851	ENSMUSG00000029833	ENSMUSG00000029833	ENSMUSG00000029833	6	37800419	37895904	100	102
5178	7	137931702	138014330	ENSMUSG00000029830	ENSMUSG00000029830	ENSMUSG00000029830	6	37916097	37971223	100	102
5179	7	138041580	138133466	ENSMUSG00000038600	ENSMUSG00000038600	ENSMUSG00000038600	6	37978091	38054182	100	102
5180	7	138172394	138254704	ENSMUSG00000063455	ENSMUSG00000063455	ENSMUSG00000063455	6	38055628	38126201	100	102
5181	7	138360992	138371315	ENSMUSG00000047749	ENSMUSG00000047749	ENSMUSG00000047749	6	38221480	38228867	100	102
5182	7	138378808	138445005	ENSMUSG00000029826	ENSMUSG00000029826	ENSMUSG00000029826	6	38240667	38283890	100	102
5183	7	138469064	138525086	ENSMUSG00000056832	ENSMUSG00000056832	ENSMUSG00000056832	6	38311684	38354847	100	102
5184	7	138566991	138633529	ENSMUSG00000038538	ENSMUSG00000038538	ENSMUSG00000038538	6	38363558	38432208	100	102
5185	7	138676612	138681079	ENSMUSG00000019689	ENSMUSG00000019689	ENSMUSG00000019689	6	38464449	38469053	100	102
5186	7	138695167	138758738	ENSMUSG00000029823	ENSMUSG00000029823	ENSMUSG00000029823	6	38481074	38539029	100	102
5187	7	138786805	138818998	ENSMUSG00000071537	ENSMUSG00000071537	ENSMUSG00000071537	6	38556268	38566841	100	102
5188	7	138907915	139123984	ENSMUSG00000061436	ENSMUSG00000061436	ENSMUSG00000061436	6	38627513	38749116	100	102
5189	7	139124668	139366560	ENSMUSG00000029925	ENSMUSG00000029925	ENSMUSG00000029925	6	38848593	39014194	100	102
5190	7	139370020	139409990	ENSMUSG00000038507	ENSMUSG00000038507	ENSMUSG00000038507	6	39016020	39047854	100	102
5191	7	139680023	139744780	ENSMUSG00000029924	ENSMUSG00000029924	ENSMUSG00000029924	6	39265578	39307256	100	102
5192	7	139753916	139772419	ENSMUSG00000029923	ENSMUSG00000029923	ENSMUSG00000029923	6	39311002	39319981	100	102
5193	7	139864699	139987045	ENSMUSG00000038456	ENSMUSG00000038456	ENSMUSG00000038456	6	39391985	39487441	100	102
5194	7	140019422	140041378	ENSMUSG00000046947	ENSMUSG00000046947	ENSMUSG00000046947	6	39503483	39518376	100	102
5195	7	140042979	140069059	ENSMUSG00000002416	ENSMUSG00000002416	ENSMUSG00000002416	6	39522181	39532989	100	102
5196	7	140080754	140271033	ENSMUSG00000002413	ENSMUSG00000002413	ENSMUSG00000002413	6	39543731	39654902	100	102
5197	7	140897679	141000532	ENSMUSG00000029916	ENSMUSG00000029916	ENSMUSG00000029916	6	40255102	40326369	100	102
5198	7	141002999	141048411	ENSMUSG00000037172	ENSMUSG00000037172	ENSMUSG00000037172	6	40336346	40365740	100	102
5199	7	141084628	141096725	ENSMUSG00000029911	ENSMUSG00000029911	ENSMUSG00000029911	6	40401022	40408194	100	102
5200	7	141110366	141111466	ENSMUSG00000052850	ENSMUSG00000052850	ENSMUSG00000052850	6	40420845	40421846	100	102
5201	7	141124758	141125657	ENSMUSG00000037140	ENSMUSG00000037140	ENSMUSG00000037140	6	40423199	40424092	100	102
5202	7	141182573	141187756	ENSMUSG00000029909	ENSMUSG00000029909	ENSMUSG00000029909	6	40444431	40449115	100	102
5203	7	141273626	141293252	ENSMUSG00000029915	ENSMUSG00000029915	ENSMUSG00000029915	6	40504505	40515412	100	102
5204	7	141318957	141319958	ENSMUSG00000058250	ENSMUSG00000058250	ENSMUSG00000058250	6	40541922	40542917	100	102
5205	7	141587032	141593361	ENSMUSG00000029885	ENSMUSG00000029885	ENSMUSG00000029885	6	40808401	40817101	100	102
5206	7	141598442	141604355	ENSMUSG00000051936	ENSMUSG00000051936	ENSMUSG00000051936	6	40824869	40829994	100	102
5207	7	141614585	141618537	ENSMUSG00000029883	ENSMUSG00000029883	ENSMUSG00000029883	6	40850067	40870164	100	102
5208	7	142270629	142278960	ENSMUSG00000029869	ENSMUSG00000029869	ENSMUSG00000029869	6	41535175	41550109	100	102
5209	7	142279083	142293629	ENSMUSG00000029868	ENSMUSG00000029868	ENSMUSG00000029868	6	41550232	41565942	100	102
5210	7	142315389	142341027	ENSMUSG00000036899	ENSMUSG00000036899	ENSMUSG00000036899	6	41581780	41610376	100	102
5211	7	142346721	142348070	ENSMUSG00000029867	ENSMUSG00000029867	ENSMUSG00000029867	6	41614038	41615324	100	102
5212	7	142348325	142369625	ENSMUSG00000029866	ENSMUSG00000029866	ENSMUSG00000029866	6	41615942	41633905	100	102
5213	7	142433409	142434341	ENSMUSG00000045479	ENSMUSG00000045479	ENSMUSG00000045479	6	41700960	41701904	100	102
5214	7	142539288	142546960	ENSMUSG00000058499	ENSMUSG00000058499	ENSMUSG00000058499	6	41777161	41781671	100	102
5215	7	142695524	142714906	ENSMUSG00000029863	ENSMUSG00000029863	ENSMUSG00000029863	6	42194648	42212114	100	102
5216	7	142723341	142759219	ENSMUSG00000029862	ENSMUSG00000029862	ENSMUSG00000029862	6	42216292	42244263	100	102
5217	7	142760616	142769967	ENSMUSG00000029861	ENSMUSG00000029861	ENSMUSG00000029861	6	42247391	42254187	100	102
5218	7	142788482	142798322	ENSMUSG00000029860	ENSMUSG00000029860	ENSMUSG00000029860	6	42279665	42289753	100	102
5219	7	142798331	142816107	ENSMUSG00000029859	ENSMUSG00000029859	ENSMUSG00000029859	6	42288094	42302875	100	102
5220	7	142850668	142851624	ENSMUSG00000056203	ENSMUSG00000056203	ENSMUSG00000056203	6	42335136	42336101	100	102
5221	7	142918148	142919126	ENSMUSG00000071494	ENSMUSG00000071494	ENSMUSG00000071494	6	42467674	42468627	100	102
5222	7	143180984	143230181	ENSMUSG00000036667	ENSMUSG00000036667	ENSMUSG00000036667	6	42597609	42639674	100	102
5223	7	143725275	143729156	ENSMUSG00000029736	ENSMUSG00000029736	ENSMUSG00000029736	6	43233279	43239169	100	102
5224	7	143779976	144164066	ENSMUSG00000029735	ENSMUSG00000029735	ENSMUSG00000029735	6	43276389	43540993	100	102
5225	7	145444902	147743641	ENSMUSG00000039419	ENSMUSG00000039419	ENSMUSG00000039419	6	44989897	47228200	100	102
5226	7	148058024	148128732	ENSMUSG00000029686	ENSMUSG00000029686	ENSMUSG00000029686	6	47383910	47455725	100	102
5227	7	148135408	148212347	ENSMUSG00000029687	ENSMUSG00000029687	ENSMUSG00000029687	6	47459867	47524606	100	102
5228	7	148331098	148356675	ENSMUSG00000025823	ENSMUSG00000025823	ENSMUSG00000025823	6	47725727	47743098	100	102
5229	7	148398448	148409232	ENSMUSG00000051499	ENSMUSG00000051499	ENSMUSG00000051499	6	47748852	47760091	100	102
5230	7	148475751	148511052	ENSMUSG00000062519	ENSMUSG00000062519	ENSMUSG00000062519	6	47765283	47797091	100	102
5231	7	148523487	148554260	ENSMUSG00000025821	ENSMUSG00000025821	ENSMUSG00000025821	6	47807142	47836553	100	102
5232	7	148567675	148583619	ENSMUSG00000052763	ENSMUSG00000052763	ENSMUSG00000052763	6	47850272	47862221	100	102
5233	7	148800820	148825727	ENSMUSG00000057691	ENSMUSG00000057691	ENSMUSG00000057691	6	47993418	48016179	100	102
5234	7	149043081	149062595	ENSMUSG00000042810	ENSMUSG00000042810	ENSMUSG00000042810	6	48325242	48346485	100	102
5235	7	149092388	149101228	ENSMUSG00000068551	ENSMUSG00000068551	ENSMUSG00000068551	6	48367226	48375279	100	102
5236	7	149651313	149666171	ENSMUSG00000073096	ENSMUSG00000073096	ENSMUSG00000073096	6	48497831	48498610	100	102
5237	7	149666351	149669696	ENSMUSG00000009281	ENSMUSG00000009281	ENSMUSG00000009281	6	48499284	48502276	100	102
5238	7	149707383	149726650	ENSMUSG00000007216	ENSMUSG00000007216	ENSMUSG00000007216	6	48542766	48552813	100	102
5239	7	149778895	149807413	ENSMUSG00000064262	ENSMUSG00000064262	ENSMUSG00000064262	6	48576947	48590461	100	102
5240	7	149895391	149901973	ENSMUSG00000054435	ENSMUSG00000054435	ENSMUSG00000054435	6	48614164	48620885	100	102
5241	7	149953399	149960406	ENSMUSG00000047867	ENSMUSG00000047867	ENSMUSG00000047867	6	48631169	48637830	100	102
5242	7	150119323	150129383	ENSMUSG00000029810	ENSMUSG00000029810	ENSMUSG00000029810	6	48763404	48770614	100	102
5243	7	150128771	150133138	ENSMUSG00000023367	ENSMUSG00000023367	ENSMUSG00000023367	6	48771108	48776634	100	102
5244	7	23252841	23281248	ENSMUSG00000029816	ENSMUSG00000029816	ENSMUSG00000029816	6	48966211	48987694	101	103
5245	7	23305485	23317894	ENSMUSG00000029815	ENSMUSG00000029815	ENSMUSG00000029815	6	49003431	49014276	101	103
5246	7	23316354	23476520	ENSMUSG00000029814	ENSMUSG00000029814	ENSMUSG00000029814	6	49016928	49144299	101	103
5247	7	23510928	23538124	ENSMUSG00000029817	ENSMUSG00000029817	ENSMUSG00000029817	6	49173510	49193596	101	103
5248	7	23603556	23650851	ENSMUSG00000050786	ENSMUSG00000050786	ENSMUSG00000050786	6	49248939	49271167	101	103
5249	7	23686347	23708793	ENSMUSG00000047115	ENSMUSG00000047115	ENSMUSG00000047115	6	49297412	49318863	101	103
5250	7	23716404	23838651	ENSMUSG00000023403	ENSMUSG00000023403	ENSMUSG00000023403	6	49325190	49399092	101	103
5251	7	24290332	24298002	ENSMUSG00000029819	ENSMUSG00000029819	ENSMUSG00000029819	6	49753184	49758913	101	103
5252	7	24579526	24694022	ENSMUSG00000038388	ENSMUSG00000038388	ENSMUSG00000038388	6	50039835	50127953	101	103
5253	7	24704500	24763888	ENSMUSG00000029821	ENSMUSG00000029821	ENSMUSG00000029821	6	50136991	50193448	101	103
5254	7	24802691	24986285	ENSMUSG00000029822	ENSMUSG00000029822	ENSMUSG00000029822	6	50229800	50312423	101	103
5255	7	25140841	25186342	ENSMUSG00000029828	ENSMUSG00000029828	ENSMUSG00000029828	6	50502890	50526176	101	103
5256	7	25230714	25234630	ENSMUSG00000029831	ENSMUSG00000029831	ENSMUSG00000029831	6	50580457	50583979	101	103
5257	7	26158385	26192430	ENSMUSG00000029832	ENSMUSG00000029832	ENSMUSG00000029832	6	51362253	51388351	101	103
5258	7	26196459	26204014	ENSMUSG00000004980	ENSMUSG00000004980	ENSMUSG00000004980	6	51392343	51397012	101	103
5259	7	26673215	26870866	ENSMUSG00000059182	ENSMUSG00000059182	ENSMUSG00000059182	6	51788744	51942132	101	103
5260	7	27099137	27102056	ENSMUSG00000029844	ENSMUSG00000029844	ENSMUSG00000029844	6	52084950	52087900	101	103
5261	7	27106499	27108919	ENSMUSG00000014704	ENSMUSG00000014704	ENSMUSG00000014704	6	52092094	52094414	101	103
5262	7	27112334	27133164	ENSMUSG00000059723	ENSMUSG00000059723	ENSMUSG00000059723	6	52098645	52130525	101	103

5263	7	27134535	27136924	ENSMUSG00000000942	ENSMUSG00000000942	ENSMUSG00000000942	6	52119270	52121286	101	103		
5264	7	27147521	27149812	ENSMUSG00000038253	ENSMUSG00000038253	ENSMUSG00000038253	6	52131337	52134170	101	103		
5265	7	27151641	27153893	ENSMUSG00000043219	ENSMUSG00000043219	ENSMUSG00000043219	6	52135947	52138207	101	103		
5266	7	27160815	27162821	ENSMUSG00000038236	ENSMUSG00000038236	ENSMUSG00000038236	6	52144081	52147057	101	103		
5267	7	27168583	27171601	ENSMUSG00000038227	ENSMUSG00000038227	ENSMUSG00000038227	6	52153637	52156953	101	103		
5268	7	27177382	27180399	ENSMUSG00000000938	ENSMUSG00000000938	ENSMUSG00000000938	6	52160780	52164522	101	103		
5269	7	27187654	27191355	ENSMUSG00000038210	ENSMUSG00000038210	ENSMUSG00000038210	6	52171690	52175371	101	103		
5270	7	27203024	27206221	ENSMUSG00000038203	ENSMUSG00000038203	ENSMUSG00000038203	6	52188454	52190352	101	103		
5271	7	27248946	27252717	ENSMUSG00000005503	ENSMUSG00000005503	ENSMUSG00000005503	6	52243076	52247956	101	103		
5272	7	27746333	27835399	ENSMUSG00000004535	ENSMUSG00000004535	ENSMUSG00000004535	6	52643326	52695652	101	103		
5273	7	27836726	28186887	ENSMUSG00000063568	ENSMUSG00000063568	ENSMUSG00000063568	6	52698375	52998173	101	103		
5274	7	28418669	28832036	ENSMUSG00000053007	ENSMUSG00000053007	ENSMUSG00000053007	6	53299362	53623662	101	103		
5275	7	29001374	29152606	ENSMUSG00000052955	ENSMUSG00000052955	ENSMUSG00000052955	6	53802857	53908251	101	103		
5276	7	29200851	29519454	ENSMUSG00000004633	ENSMUSG00000004633	ENSMUSG00000004633	6	54145240	54229871	101	103		
5277	7	29569952	29573436	ENSMUSG00000045725	ENSMUSG00000045725	ENSMUSG00000045725	6	54256461	54259778	101	103		
5278	7	29881782	29910868	ENSMUSG00000068499	ENSMUSG00000068499	ENSMUSG00000068499	6	54374799	54418686	101	103		
5279	7	29926273	29995902	ENSMUSG00000019124	ENSMUSG00000019124	ENSMUSG00000019124	6	54435472	54495947	101	103		
5280	7	30019409	30032793	ENSMUSG00000038074	ENSMUSG00000038074	ENSMUSG00000038074	6	54507189	54522728	101	103		
5281	7	30290448	30373832	ENSMUSG00000058446	ENSMUSG00000058446	ENSMUSG00000058446	6	54746494	54819314	101	103		
5282	7	30430672	30484833	ENSMUSG00000038058	ENSMUSG00000038058	ENSMUSG00000038058	6	54853525	54901991	101	103		
5283	7	30502764	30510977	ENSMUSG00000002797	ENSMUSG00000002797	ENSMUSG00000002797	6	54913680	54922445	101	103		
5284	7	30600706	30640169	ENSMUSG00000029777	ENSMUSG00000029777	ENSMUSG00000029777	6	54967598	55009078	101	103		
5285	7	30659388	30706244	ENSMUSG00000003476	ENSMUSG00000003476	ENSMUSG00000003476	6	55019665	55062550	101	103		
5286	7	30758276	30763743	ENSMUSG00000003477	ENSMUSG00000003477	ENSMUSG00000003477	6	55100205	55104616	101	103		
5287	7	30777558	30898145	ENSMUSG00000038022	ENSMUSG00000038022	ENSMUSG00000038022	6	55132961	55247889	101	103		
5288	7	30917993	30931656	ENSMUSG00000004655	ENSMUSG00000004655	ENSMUSG00000004655	6	55266010	55278133	101	103		
5289	7	30970161	30985666	ENSMUSG00000004654	ENSMUSG00000004654	ENSMUSG00000004654	6	55305921	55318108	101	103		
5290	7	31058667	31112836	ENSMUSG00000029778	ENSMUSG00000029778	ENSMUSG00000029778	6	55381558	55431033	101	103		
5291	7	31343607	31347063	ENSMUSG00000037984	ENSMUSG00000037984	ENSMUSG00000037984	6	55607401	55610647	101	103		
5292	7	31523503	31659828	ENSMUSG00000037973	ENSMUSG00000037973	ENSMUSG00000037973	6	55816403	55908313	101	103		
5293	7	31693372	31714593	ENSMUSG00000002930	ENSMUSG00000002930	ENSMUSG00000002930	6	55947075	55962263	101	103		
5294	7	31759523	32304872	ENSMUSG00000004347	ENSMUSG00000004347	ENSMUSG00000004347	6	55999382	56291970	101	103		
5295	7	32501701	32590304	ENSMUSG00000029787	ENSMUSG00000029787	ENSMUSG00000029787	6	56644505	56691489	101	103		
5296	7	32874311	32897897	ENSMUSG00000059486	ENSMUSG00000059486	ENSMUSG00000059486	6	56707103	56727301	101	103		
5297	7	33020267	33068934	ENSMUSG00000029780	ENSMUSG00000029780	ENSMUSG00000029780	6	56811983	56853407	101	103		
5298	4	89402077	89424771	ENSMUSG00000037826	ENSMUSG00000037826	ENSMUSG00000037826	6	57436080	57465004	102	104		
5299	4	89518915	89583271	ENSMUSG00000029798	ENSMUSG00000029798	ENSMUSG00000029798	6	57510615	57592004	102	104		
5300	4	89745968	89848709	ENSMUSG00000029804	ENSMUSG00000029804	ENSMUSG00000029804	6	58763278	58849978	102	104		
5301	4	89837380	89837928	ENSMUSG00000055430	ENSMUSG00000055430	ENSMUSG00000055430	6	58836075	58836545	102	104		
5302	4	89866129	90197346	ENSMUSG00000037709	ENSMUSG00000037709	ENSMUSG00000037709	6	58865163	58933086	102	104		
5303	4	90252991	90255062	ENSMUSG00000049232	ENSMUSG00000049232	ENSMUSG00000049232	6	59138555	59141306	102	104		
5304	4	90387287	90447967	ENSMUSG00000045441	ENSMUSG00000045441	ENSMUSG00000045441	6	59277774	59355872	102	104		
5305	4	90866479	90978470	ENSMUSG00000025889	ENSMUSG00000025889	ENSMUSG00000025889	6	60661224	60759433	102	104		
5306	4	91035075	91094801	ENSMUSG00000054641	ENSMUSG00000054641	ENSMUSG00000054641	6	60874058	60918597	102	104		
5307	4	91448453	92739231	ENSMUSG00000039578	ENSMUSG00000039578	ENSMUSG00000039578	6	61241393	62309845	102	104		
5308	4	93444831	94912672	ENSMUSG00000071424	ENSMUSG00000071424	ENSMUSG00000071424	6	63186435	64595857	102	104		
5309	4	94969100	94970165	ENSMUSG00000073043	ENSMUSG00000073043	ENSMUSG00000073043	6	64658703	64660810	102	104		
5310	4	95347896	95431464	ENSMUSG00000029920	ENSMUSG00000029920	ENSMUSG00000029920	6	64972245	65045627	102	104		
5311	4	95438730	95483050	ENSMUSG00000029919	ENSMUSG00000029919	ENSMUSG00000029919	6	65048942	65074308	102	104		
5312	4	122272017	122304926	ENSMUSG00000044162	ENSMUSG00000044162	ENSMUSG00000044162	6	65520186	65544375	103	104	here	Sub block(s) comes here, so our tool did not merge
5313	4	122176232	122213019	ENSMUSG00000049001	ENSMUSG00000049001	ENSMUSG00000049001	6	65601189	65636508	103	104	sub blocks	2 -> 1
5314	4	121835380	122063463	ENSMUSG00000029913	ENSMUSG00000029913	ENSMUSG00000029913	6	65708617	65865951	103	104		
5315	1	67939737	68071738	ENSMUSG00000036402	ENSMUSG00000036402	ENSMUSG00000036402	6	66945329	66950872	104	105		
5316	1	67923332	67926609	ENSMUSG00000036390	ENSMUSG00000036390	ENSMUSG00000036390	6	66964674	66966985	104	105		
5317	1	67646081	67668711	ENSMUSG00000036371	ENSMUSG00000036371	ENSMUSG00000036371	6	67196557	67215541	104	105		
5318	1	67545635	67635171	ENSMUSG00000018341	ENSMUSG00000018341	ENSMUSG00000018341	6	67221596	67305715	104	105		
5319	1	67404671	67498250	ENSMUSG00000049093	ENSMUSG00000049093	ENSMUSG00000049093	6	67352943	67396002	104	105		
5320	2	88937809	89013011	ENSMUSG00000061260	ENSMUSG00000061260	ENSMUSG00000061260	6	70301036	70656336	105	106		
5321	2	88772314	88831566	ENSMUSG00000053604	ENSMUSG00000053604	ENSMUSG00000053604	6	70695280	70721734	105	106		
5322	2	88637376	88707907	ENSMUSG00000031668	ENSMUSG00000031668	ENSMUSG00000031668	6	70774094	70834818	105	106		
5323	2	88605284	88610217	ENSMUSG00000051896	ENSMUSG00000051896	ENSMUSG00000051896	6	70842666	70848501	105	106		
5324	2	88252094	88267260	ENSMUSG00000054474	ENSMUSG00000054474	ENSMUSG00000054474	6	71057977	71073955	105	106		
5325	2	88203625	88208693	ENSMUSG00000054422	ENSMUSG00000054422	ENSMUSG00000054422	6	71129471	71134602	105	106		
5326	2	88148496	88194015	ENSMUSG00000055027	ENSMUSG00000055027	ENSMUSG00000055027	6	71143520	71191838	105	106		
5327	2	88107840	88136387	ENSMUSG00000053012	ENSMUSG00000053012	ENSMUSG00000053012	6	71201297	71214895	105	106		
5328	2	86865245	86871578	ENSMUSG00000053977	ENSMUSG00000053977	ENSMUSG00000053977	6	71303062	71307116	105	106		
5329	2	86684028	86703520	ENSMUSG00000052656	ENSMUSG00000052656	ENSMUSG00000052656	6	71424172	71440390	105	106		
5330	2	86521954	86573350	ENSMUSG00000053470	ENSMUSG00000053470	ENSMUSG00000053470	6	71518554	71562414	105	106		
5331	2	86294633	86418288	ENSMUSG00000052852	ENSMUSG00000052852	ENSMUSG00000052852	6	71637479	71724841	105	106		
5332	2	86280091	86293480	ENSMUSG00000052962	ENSMUSG00000052962	ENSMUSG00000052962	6	71742512	71753272	105	106		
5333	2	86224574	86276108	ENSMUSG00000052337	ENSMUSG00000052337	ENSMUSG00000052337	6	71760840	71804775	105	106		
5334	2	86186849	86222791	ENSMUSG00000063884	ENSMUSG00000063884	ENSMUSG00000063884	6	71810143	71838271	105	106		
5335	2	86107891	86186479	ENSMUSG00000049553	ENSMUSG00000049553	ENSMUSG00000049553	6	71838582	71908875	105	106		
5336	2	85919782	85969648	ENSMUSG00000056091	ENSMUSG00000056091	ENSMUSG00000056091	6	72027122	72084079	105	106		
5337	2	85834528	85868696	ENSMUSG00000037621	ENSMUSG00000037621	ENSMUSG00000037621	6	72153248	72164673	105	106		
5338	2	85737954	85748823	ENSMUSG00000056370	ENSMUSG00000056370	ENSMUSG00000056370	6	72234134	72242441	105	106		
5339	2	85696830	85729911	ENSMUSG00000056305	ENSMUSG00000056305	ENSMUSG00000056305	6	72248185	72274684	105	106		
5340	2	85692144	85692618	ENSMUSG00000058706	ENSMUSG00000058706	ENSMUSG00000058706	6	72276852	72282656	105	106		
5341	2	85679183	85683293	ENSMUSG00000055912	ENSMUSG00000055912	ENSMUSG00000055912	6	72284992	72289271	105	106		
5342	2	85676348	85678342	ENSMUSG00000055850	ENSMUSG00000055850	ENSMUSG00000055850	6	72289230	72291902	105	106		
5343	2	85665042	85674020	ENSMUSG00000073002	ENSMUSG00000073002	ENSMUSG00000073002	6	72298812	72299840	105	106		
5344	2	85658228	85662664	ENSMUSG00000050732	ENSMUSG00000050732	ENSMUSG00000050732	6	72315019	72317929	105	106		
5345	2	85629718	85642090	ENSMUSG00000053460	ENSMUSG00000053460	ENSMUSG00000053460	6	72343843	72360221	105	106		
5346	2	85619799	85625912	ENSMUSG00000053907	ENSMUSG00000053907	ENSMUSG00000053907	6	72362308	72369048	105	106		
5347	2	85511130	85517213	ENSMUSG00000052631	ENSMUSG00000052631	ENSMUSG00000052631	6	72443163	72450136	105	106		
5348	2	85475462	85494676	ENSMUSG00000056737	ENSMUSG00000056737	ENSMUSG00000056737	6	72478981	72492484	105	106		
5349	2	85435380	85472380	ENSMUSG00000056698	ENSMUSG00000056698	ENSMUSG00000056698	6	72495443	72527852	105	106		
5350	2	85422733	85435166	ENSMUSG00000056666	ENSMUSG00000056666	ENSMUSG00000056666	6	72528137	72536997	105	106		
5351	2	85214245	85391012	ENSMUSG00000055799	ENSMUSG00000055799	ENSMUSG00000055799	6	72555889	72718465	105	106		
5352	2	84784885	84900040	ENSMUSG00000052861	ENSMUSG00000052861	ENSMUSG00000052861	6	72947118	72989812	105	106		
5353	2	84504194	84539931	ENSMUSG00000052738	ENSMUSG00000052738	ENSMUSG00000052738	6	73178158	73206414	105	106		
5354	2	80368994	80385029	ENSMUSG00000060780	ENSMUSG00000060780	ENSMUSG00000060780	6	77172199	77187302	105	106		
5355	2	76829329	77600502	ENSMUSG00000052581	ENSMUSG00000052581	ENSMUSG00000052581	6	79951119	80738698	105	106		
5356	2	75742802	75791830	ENSMUSG00000035125	ENSMUSG00000035125	ENSMUSG00000035125	6	81889327	81924756	105	106		
5357	2	75727437	75751387	ENSMUSG00000030045	ENSMUSG00000030045	ENSMUSG00000030045	6	81923484	81931607	105	106		
5358	2	75572952	75650356	ENSMUSG00000035104	ENSMUSG00000035104	ENSMUSG00000035104	6	82007331	82058756	105	106		
5359	2	75129738	75279781	ENSMUSG00000030043	ENSMUSG00000030043	ENSMUSG00000030043	6	82368133	82525757	105	106		
5360	2	75039283	75050366	ENSMUSG00000030042	ENSMUSG00000030042	ENSMUSG00000030042	6	82617084	82618497	105	106		
5361	2	74913290	74973982	ENSMUSG00000000628	ENSMUSG00000000628	ENSMUSG00000000628	6	82690705	82740117	105	106		
5362	2	74734901	74762693	ENSMUSG00000000627	ENSMUSG00000000627	ENSMUSG00000000627	6	82877544	82905386	105	106		
5363	2	74639351	74728672	ENSMUSG00000030041	ENSMUSG00000030041	ENSMUSG00000030041	6	82922067	82995825	105	106		
5364	2	74634795	74638181	ENSMUSG00000068335	ENSMUSG00000068335	ENSMUSG00000068335	6	82996592	82999123	105	106		
5365	2	74613454	74634570	ENSMUSG00000000693	ENSMUSG00000000693	ENSMUSG00000000693	6	82999882	83018222	105	106		
5366	2	74610040	74614191	ENSMUSG00000068329	ENSMUSG00000068329	ENSMUSG00000068329	6	83016938	83020229	105	106		
5367	2	74607284	74610395	ENSMUSG00000068328	ENSMUSG00000068328	ENSMUSG00000068328	6	83020354	83023340	105	106		
5368	2	74598768	74606826	ENSMUSG00000009145	ENSMUSG00000009145	ENSMUSG00000009145	6	83023502	83032726	105	106		
5369	2	74595442	74598256	ENSMUSG00000068327	ENSMUSG00000068327	ENSMUSG00000068327	6	83033983	83035883	105	106		
5370	2	74585700	74589215	ENSMUSG00000069678	ENSMUSG00000069678	ENSMUSG00000069678	6	83044048	83046505	105	106		
5371	2	74578153	74580351	ENSMUSG00000034968	ENSMUSG00000034968	ENSMUSG00000034968	6	83052023	83053899	105	106		
5372	2	74554737	74563631	ENSMUSG00000030037	ENSMUSG00000030037	ENSMUSG00000030037	6	83068783	83074029	105	106		
5373	2	74541712	74546026	ENSMUSG00000030036	ENSMUSG00000030036	ENSMUSG00000030036	6	83081164	83084556	105	106		
5374	2	74539085	74541518	ENSMUSG00000030035	ENSMUSG00000030035	ENSMUSG00000030035	6	83084706	83087217	105	106		
5375	2	74535741	74538499	ENSMUSG00000030034	ENSMUSG00000030034	ENSMUSG00000030034	6	83087486	83090451	105	106		
5376	2	74506497	74521218	ENSMUSG00000034930	ENSMUSG00000034930	ENSMUSG00000034930	6	83101545	83118234	105	106		
5377	2	74502365	74506389	ENSMUSG00000030032	ENSMUSG00000030032	ENSMUSG00000030032	6	83115020	83121953	105	106		
5378	2	74494964	74496885	ENSMUSG00000030030	ENSMUSG00000030030	ENSMUSG00000030030	6	83122123	83128601	105	106		
5379	2	74441790	74461472	ENSMUSG00000031865	ENSMUSG00000031865	ENSMUSG00000031865	6	83148200	83165469	105	106		
5380	2	74302102	74395658	ENSMUSG00000068323	ENSMUSG00000068323	ENSMUSG00000068323	6	83190329	83269410	105	106		
5381	2	74235495	74253452	ENSMUSG00000043131	ENSMUSG00000043131	ENSMUSG00000043131	6	83295113	83308454	105	106		
5382	2	74216036	74228547	ENSMUSG00000045160	ENSMUSG00000045160	ENSMUSG00000045160	6	83315142	83324050	105	106		
5383	2	74154234	74183203	ENSMUSG00000034832	ENSMUSG00000034832	ENSMUSG00000034832	6	83333822	83342513	105	106		
5384	2	74007461	74039596	ENSMUSG00000014554	ENSMUSG00000014554	ENSMUSG00000014554	6	83445877	83472571	105	106		
5385	2	73973601	74000287	ENSMUSG00000059430	ENSMUSG00000059430	ENSMUSG00000059430	6	83478582	83501890	105	106		
5386	2	73909597	73954268	ENSMUSG00000006906	ENSMUSG00000006906	ENSMUSG00000006906	6	83508864	83538109	105	106		
5387	2	70889290	70901240	ENSMUSG00000014542	ENSMUSG00000014542	ENSMUSG00000014542	6	83610200	83621774	106	107		
5388	2	70910856	70916414	ENSMUSG00000034783	ENSMUSG00000034783	ENSMUSG00000034783	6	83636873	83643457	106	107		
5389	2	70981228	71014081	ENSMUSG00000034777	ENSMUSG00000034777	ENSMUSG00000034777	6	83676922	83703962	106	107		
5390	2	71016529	71046063	ENSMUSG00000006269	ENSMUSG00000006269	ENSMUSG00000006269	6	83708675	83724468	106	107		
5391	2	71059083	71065977	ENSMUSG00000014747	ENSMUSG00000014747	ENSMUSG00000014747	6	83728302	83733984	106	107		
5392	2	71066578	71075509	ENSMUSG00000014748	ENSMUSG00000014748	ENSMUSG00000014748	6	83736074	83741417	106	107		
5393	2	71148955	71159279	ENSMUSG00000034744	ENSMUSG00000034744	ENSMUSG00000034744	6	83760702	83768214	106	107		
5394	2	71263377	71307721	ENSMUSG00000045896	ENSMUSG00000045896	ENSMUSG00000045896	6	83771719	83797341	106	107		
5395	2	71412397	71515695	ENSMUSG00000030016	ENSMUSG00000030016	ENSMUSG00000030016	6	83880025	83952519	106	107		
5396	2	71534268	71767398	ENSMUSG00000033788	ENSMUSG00000033788	ENSMUSG00000033788	6	83985047	84176717	106	107		
5397	2	72209875	72228471	ENSMUSG00000063415	ENSMUSG00000063415	ENSMUSG00000063415	6	84537602	84559566	106	107		
5398	2	72545760	72906662	ENSMUSG00000033769	ENSMUSG00000033769	ENSMUSG00000033769	6	84800639	85035160	106	107		
5399	2	72998112	73014984	ENSMUSG00000033726	ENSMUSG00000033726	ENSMUSG00000033726	6	85153589	85170016	106	107		
5400	2	73022674	73152473	ENSMUSG00000033720	ENSMUSG00000033720	ENSMUSG00000033720	6	85178712	85299077	106	107		
5401	2	73156160	73193419	ENSMUSG00000051343	ENSMUSG00000051343	ENSMUSG00000051343	6	85300620	85340292	106	107		
5402	2	73294915	73306579	ENSMUSG00000033706	ENSMUSG00000033706	ENSMUSG00000033706	6	85397647	85411443	106	107		
5403	2	73308644	73313864	ENSMUSG00000030008	ENSMUSG00000030008	ENSMUSG00000030008	6	85412469	85416926	106	107		
5404	2	73314913	73333655	ENSMUSG00000030007	ENSMUSG00000030007	ENSMUSG00000030007	6	85417731	85434130	106	107		
5405	2	73336457	73350266	ENSMUSG00000047013	ENSMUSG00000047013	ENSMUSG00000047013	6	85436183	85468652	106	107		
5406	2	73371574	73374118	ENSMUSG00000071341	ENSMUSG00000071341	ENSMUSG00000071341	6	85476780	85479200	106	107		
5407	2	73810509	73818021	ENSMUSG00000054226	ENSMUSG00000054226	ENSMUSG00000054226	6	85881391	85895918	106	107		
5408	2	73842841	73860730	ENSMUSG00000030002	ENSMUSG00000030002	ENSMUSG00000030002	6	85907900	85927301	106	107		
5409	2	70858273	70871283	ENSMUSG00000030001	ENSMUSG00000030001	ENSMUSG00000030001	6	85982825	85986630	107	108		
5410	2	70742773	70848837	ENSMUSG00000030000	ENSMUSG00000030000	ENSMUSG00000030000	6	86043718	86085189	107	108		
5411	2	70527927	70634438	ENSMUSG00000029999	ENSMUSG00000029999	ENSMUSG00000029999	6	86228789	86237128	107	108		
5412	2	70362012	70374373	ENSMUSG00000057278	ENSMUSG00000057278	ENSMUSG00000057278	6	86337138	86344536	107	108		
5413	2	70338760	70360418	ENSMUSG00000029998	ENSMUSG00000029998	ENSMUSG00000029998	6	86352491	86362754	107	108		
5414	2	70230523	70271655	ENSMUSG00000046679	ENSMUSG00000046679	ENSMUSG00000046679	6	86404032	86436019	107	108		
5415	2	70168090	70169766	ENSMUSG00000051695	ENSMUSG00000051695	ENSMUSG00000051695	6	86490479	86491549	107	108		
5416	2	70041293	70042324	ENSMUSG00000033508	ENSMUSG00000033508	ENSMUSG00000033508	6	86593810	86595336	107	108		
5417	2	69995707	70023581	ENSMUSG00000001156	ENSMUSG00000001156	ENSMUSG00000001156	6	86615467	86634730	107	108		
5418	2	69974594	69985852	ENSMUSG00000001158	ENSMUSG00000001158	ENSMUSG00000001158	6	86640803	86650125	107	108		
5419	2	69801427	69906410	ENSMUSG00000029994	ENSMUSG00000029994	ENSMUSG00000029994	6	86703398	86726395	107	108		
5420	2	69561706	69723771	ENSMUSG00000057230	ENSMUSG00000057230	ENSMUSG00000057230	6	86815474	86957498	107	108		
5421	2	69539918	69547168	ENSMUSG00000062792	ENSMUSG00000062792	ENSMUSG00000062792	6	86962130	86967703	107	108		
5422	2	69476403	69518257	ENSMUSG00000029993	ENSMUSG00000029993	ENSMUSG00000029993	6	86975523	86994094	107	108		
5423	2	69405907	69467886	ENSMUSG00000029992	ENSMUSG00000029992	ENSMUSG00000029992	6	87008525	87053873	107	108		
5424	2	69093993	69329804	ENSMUSG00000033420	ENSMUSG00000033420	ENSMUSG00000033420	6	87099487	87301349	107	108		
5425	2	69055209	69061616	ENSMUSG00000030050	ENSMUSG00000030050	ENSMUSG00000030050	6	87311287	87316549	107	108		
5426	2	69025871	69033606	ENSMUSG00000030049	ENSMUSG00000030049	ENSMUSG00000030049	6	87340926	87344937	107	108		
5427	2	68946117	68952153	ENSMUSG00000030046	ENSMUSG00000030046	ENSMUSG00000030046	6	87394674	87400133	107	108		
5428	2	68815472	68907452	ENSMUSG00000030047	ENSMUSG00000030047	ENSMUSG00000030047	6	87425026	87498876	107	108		
5429	2	68726458	68736212	ENSMUSG00000049409	ENSMUSG00000049409	ENSMUSG00000049409	6	87544233	87556342	107	108		
5430	2	68548246	68660797	ENSMUSG00000030051	ENSMUSG00000030051	ENSMUSG00000030051	6	87595390	87637809	107	108		
5431	3	130231990	130242273	ENSMUSG00000068263	ENSMUSG00000068263	ENSMUSG00000068263	6	87717433	87720500	108	109	here	This inversion was ignored by our tool
5432	3	130262300	130263941	ENSMUSG00000030054	ENSMUSG00000030054	ENSMUSG00000030054	6	87743777	87745403	108	109	Inversion ignored by ST	1 -> 2
5433	3	130289108	130362569	ENSMUSG00000030056	ENSMUSG00000030056	ENSMUSG00000030056	6	87779921	87804368	108	109		
5434	3	130371020	130385432	ENSMUSG00000030057	ENSMUSG00000030057	ENSMUSG00000030057	6	87808723	87816711	108	109		
5435	3	130451143	130479304	ENSMUSG00000030058	ENSMUSG00000030058	ENSMUSG00000030058	6	87853581	87879236	108	109		
5436	3	130480374	130506823	ENSMUSG00000030060	ENSMUSG00000030060	ENSMUSG00000030060	6	87879617	87902255	108	109		
5437	3	130516794	130517435	ENSMUSG00000044927	ENSMUSG00000044927	ENSMUSG00000044927	6	87946431	87946997	108	109		
5438	3	129821503	129852409	ENSMUSG00000030062	ENSMUSG00000030062	ENSMUSG00000030062	6	88050157	88070945	108	110		
5439	3	129680962	129694718	ENSMUSG00000015053	ENSMUSG00000015053	ENSMUSG00000015053	6	88159532	88172671	108	110		
5440	3	129663972	129668781	ENSMUSG00000048206	ENSMUSG00000048206	ENSMUSG00000048206	6	88187909	88188897	108	110		
5441	3	129355003	129610178	ENSMUSG00000033216	ENSMUSG00000033216	ENSMUSG00000033216	6	88222975	88412161	108	110		
5442	3	129282493	129325350	ENSMUSG00000030079	ENSMUSG00000030079	ENSMUSG00000030079	6	88431098	88463206	108	110		
5443	3	129253902	129273215	ENSMUSG00000030082	ENSMUSG00000030082	ENSMUSG00000030082	6	88470748	88484086	108	110		
5444	3	128893731	129023381	ENSMUSG00000033174	ENSMUSG00000033174	ENSMUSG00000033174	6	88690125	88793998	108	110		
5445	3	128874471	128882452	ENSMUSG00000030083	ENSMUSG00000030083	ENSMUSG00000030083	6	88801565	88807541	108	110		
5446	3	128830729	128874334	ENSMUSG00000033152	ENSMUSG00000033152	ENSMUSG00000033152	6	88808206	88840238	108	110		
5447	3	128799943	128823964	ENSMUSG00000002870	ENSMUSG00000002870	ENSMUSG00000002870	6	88849641	88863163	108	110		
5448	3	128774679	128792621	ENSMUSG00000002871	ENSMUSG00000002871	ENSMUSG00000002871	6	88867959	88877871	108	110		
5449	3	128190192	128238918	ENSMUSG00000030084	ENSMUSG00000030084	ENSMUSG00000030084	6	89284498	89328239	108	110		
5450	3	127905808	128161934	ENSMUSG00000030086	ENSMUSG00000030086	ENSMUSG00000030086	6	89350242	89561186	108	110		
5451	3	127809028	127856644	ENSMUSG00000000811	ENSMUSG00000000811	ENSMUSG00000000811	6	89609639	89641155	108	110		
5452	3	127751210	127760448	ENSMUSG00000049694	ENSMUSG00000049694	ENSMUSG00000049694	6	90266774	90270952	108	110		
5453	3	127725866	127744823	ENSMUSG00000056643	ENSMUSG00000056643	ENSMUSG00000056643	6	90274478	90275284	108	110		
5454	3	127683390	127719286	ENSMUSG00000034456	ENSMUSG00000034456	ENSMUSG00000034456	6	90298810	90330059	108	110		
5455	3	127639911	127677398	ENSMUSG00000034430	ENSMUSG00000034430	ENSMUSG00000034430	6	90335107	90368990	108	110		
5456	3	127596472	127638088	ENSMUSG00000048794	ENSMUSG00000048794	ENSMUSG00000048794	6	90369248	90393936	108	110		
5457	3	127544191	127558929	ENSMUSG00000030087	ENSMUSG00000030087	ENSMUSG00000030087	6	90428130	90440713	108	110		

5458	3	127305102	127382719	ENSMUSG00000030088	ENSMUSG00000030088	ENSMUSG00000030088	6	90516352	90564663	108	110		
5459	3	127207940	127285824	ENSMUSG00000030089	ENSMUSG00000030089	ENSMUSG00000030089	6	90570384	90611916	108	110		
5460	3	13332737	13436809	ENSMUSG00000030091	ENSMUSG00000030091	ENSMUSG00000030091	6	90978574	91082298	109	111		
5461	3	13496824	13521834	ENSMUSG00000034245	ENSMUSG00000034245	ENSMUSG00000034245	6	91122308	91140192	109	111		
5462	3	13565625	13654922	ENSMUSG00000064080	ENSMUSG00000064080	ENSMUSG00000064080	6	91178368	91238044	109	111		
5463	3	13835085	13896619	ENSMUSG00000030093	ENSMUSG00000030093	ENSMUSG00000030093	6	91329487	91376873	109	111		
5464	3	14141546	14160178	ENSMUSG00000030095	ENSMUSG00000030095	ENSMUSG00000030095	6	91439217	91453962	109	111		
5465	3	14161651	14195143	ENSMUSG00000030094	ENSMUSG00000030094	ENSMUSG00000030094	6	91454814	91481329	109	111		
5466	3	14195337	14214858	ENSMUSG00000034192	ENSMUSG00000034192	ENSMUSG00000034192	6	91481539	91488124	109	111		
5467	3	14419110	14503971	ENSMUSG00000030096	ENSMUSG00000030096	ENSMUSG00000030096	6	91649599	91724570	109	111		
5468	3	14505623	14556840	ENSMUSG00000030098	ENSMUSG00000030098	ENSMUSG00000030098	6	91727016	91748413	109	111		
5469	3	14668273	14689155	ENSMUSG00000034083	ENSMUSG00000034083	ENSMUSG00000034083	6	91843563	91865341	109	111		
5470	3	14691658	14789542	ENSMUSG00000034063	ENSMUSG00000034063	ENSMUSG00000034063	6	91869737	91916229	109	111		
5471	3	14835583	14950899	ENSMUSG00000034037	ENSMUSG00000034037	ENSMUSG00000034037	6	91944382	92041508	109	111		
5472	3	15020395	15059704	ENSMUSG00000005893	ENSMUSG00000005893	ENSMUSG00000005893	6	92104498	92133463	109	111		
5473	3	15065024	15081820	ENSMUSG00000014551	ENSMUSG00000014551	ENSMUSG00000014551	6	92135016	92149527	109	111		
5474	3	15086584	15115659	ENSMUSG00000014550	ENSMUSG00000014550	ENSMUSG00000014550	6	92152216	92180315	109	111		
5475	3	131176253	131179470	ENSMUSG00000005892	ENSMUSG00000005892	ENSMUSG00000005892	6	92207566	92210152	Singleton			
5476	3	64054594	64186171	ENSMUSG00000030020	ENSMUSG00000030020	ENSMUSG00000030020	6	92341392	92532870	110	112		
5477	3	64476373	64648405	ENSMUSG00000030022	ENSMUSG00000030022	ENSMUSG00000030022	6	92835770	92908994	110	112		
5478	3	65317093	65999023	ENSMUSG00000045095	ENSMUSG00000045095	ENSMUSG00000045095	6	93643711	94248785	110	112		
5479	3	66376317	66521084	ENSMUSG00000045100	ENSMUSG00000045100	ENSMUSG00000045100	6	94465811	94570138	110	112		
5480	3	66511912	66634041	ENSMUSG00000030029	ENSMUSG00000030029	ENSMUSG00000030029	6	94569992	94665608	110	112		
5481	3	67131421	67144320	ENSMUSG00000030031	ENSMUSG00000030031	ENSMUSG00000030031	6	95083422	95095186	110	112		
5482	3	68138460	68689160	ENSMUSG00000059187	ENSMUSG00000059187	ENSMUSG00000059187	6	96081100	96614652	110	112		
5483	3	68863608	69064401	ENSMUSG00000046500	ENSMUSG00000046500	ENSMUSG00000046500	6	96809205	96979949	110	112		
5484	3	69107056	69145509	ENSMUSG00000035245	ENSMUSG00000035245	ENSMUSG00000035245	6	97076407	97114346	110	112		
5485	3	69154462	69184144	ENSMUSG00000030059	ENSMUSG00000030059	ENSMUSG00000030059	6	97133550	97144486	110	112		
5486	3	69187278	69212194	ENSMUSG00000030061	ENSMUSG00000030061	ENSMUSG00000030061	6	97149282	97171023	110	112		
5487	3	69216780	69237927	ENSMUSG00000035199	ENSMUSG00000035199	ENSMUSG00000035199	6	97176258	97198837	110	112		
5488	3	69250392	69254407	ENSMUSG00000044086	ENSMUSG00000044086	ENSMUSG00000044086	6	97212609	97218222	110	112		
5489	3	69302109	69517914	ENSMUSG00000030064	ENSMUSG00000030064	ENSMUSG00000030064	6	97253823	97388990	110	112		
5490	3	69871323	70100174	ENSMUSG00000035158	ENSMUSG00000035158	ENSMUSG00000035158	6	97772647	97984170	110	112		
5491	3	71087426	71715830	ENSMUSG00000030067	ENSMUSG00000030067	ENSMUSG00000030067	6	98895553	99128481	110	112		
5492	3	71816526	71857208	ENSMUSG00000030068	ENSMUSG00000030068	ENSMUSG00000030068	6	99590600	99632234	110	112		
5493	3	71885891	71887018	ENSMUSG00000072875	ENSMUSG00000072875	ENSMUSG00000072875	6	99658142	99659281	110	112		
5494	3	71903497	71916902	ENSMUSG00000030069	ENSMUSG00000030069	ENSMUSG00000030069	6	99676762	99691855	110	112		
5495	3	72882125	72980181	ENSMUSG00000035378	ENSMUSG00000035378	ENSMUSG00000035378	6	100539165	100637159	110	112		
5496	3	73020075	73106994	ENSMUSG00000030074	ENSMUSG00000030074	ENSMUSG00000030074	6	100670832	100771181	110	112		
5497	3	73128651	73197827	ENSMUSG00000052144	ENSMUSG00000052144	ENSMUSG00000052144	6	100799736	100834815	110	112		
5498	3	73514342	73756762	ENSMUSG00000035357	ENSMUSG00000035357	ENSMUSG00000035357	6	101115712	101343995	110	112		
5499	3	74394412	74653033	ENSMUSG00000030075	ENSMUSG00000030075	ENSMUSG00000030075	6	102131416	102404031	110	112		
5500	3	213650	426098	ENSMUSG00000030077	ENSMUSG00000030077	ENSMUSG00000030077	6	103476776	103695605	111	113		
5501	3	1109629	1420278	ENSMUSG00000030092	ENSMUSG00000030092	ENSMUSG00000030092	6	104458817	104829181	111	113		
5502	3	2752877	3074645	ENSMUSG00000064293	ENSMUSG00000064293	ENSMUSG00000064293	6	106310505	106665082	111	113		
5503	3	3086421	3127031	ENSMUSG00000005364	ENSMUSG00000005364	ENSMUSG00000005364	6	106678159	106710113	111	113		
5504	3	3143620	3167542	ENSMUSG00000013736	ENSMUSG00000013736	ENSMUSG00000013736	6	106734949	106748246	111	113		
5505	3	3166695	3196394	ENSMUSG00000005362	ENSMUSG00000005362	ENSMUSG00000005362	6	106744026	106765846	111	113		
5506	3	3816550	3864300	ENSMUSG00000034648	ENSMUSG00000034648	ENSMUSG00000034648	6	107495560	107535987	111	113		
5507	3	3844104	4483929	ENSMUSG00000030101	ENSMUSG00000030101	ENSMUSG00000030101	6	108072805	108151357	111	113		
5508	3	4996208	5001861	ENSMUSG00000030103	ENSMUSG00000030103	ENSMUSG00000030103	6	108626406	108632702	111	113		
5509	3	5138665	5197701	ENSMUSG00000030105	ENSMUSG00000030105	ENSMUSG00000030105	6	108748876	108789494	111	113		
5510	3	5204433	5236642	ENSMUSG00000030104	ENSMUSG00000030104	ENSMUSG00000030104	6	108794429	108825128	111	113		
5511	3	6878076	7707906	ENSMUSG00000056755	ENSMUSG00000056755	ENSMUSG00000056755	6	110611645	111461678	111	113		
5512	3	8518511	8584805	ENSMUSG00000057604	ENSMUSG00000057604	ENSMUSG00000057604	6	112239535	112296199	111	113		
5513	3	8636086	8668760	ENSMUSG00000034387	ENSMUSG00000034387	ENSMUSG00000034387	6	112325101	112353800	111	113		
5514	3	8750253	8763451	ENSMUSG00000062694	ENSMUSG00000062694	ENSMUSG00000062694	6	112425278	112438649	111	113		
5515	3	8767114	8786300	ENSMUSG00000049112	ENSMUSG00000049112	ENSMUSG00000049112	6	112442851	112455574	111	113		
5516	3	8893760	8980186	ENSMUSG00000030254	ENSMUSG00000030254	ENSMUSG00000030254	6	112585662	112662424	111	113		
5517	3	8997278	9266311	ENSMUSG00000030257	ENSMUSG00000030257	ENSMUSG00000030257	6	112688565	112912273	111	113		
5518	3	9518481	9570486	ENSMUSG00000042873	ENSMUSG00000042873	ENSMUSG00000042873	6	112980188	113161108	111	113		
5519	3	9379736	9403475	ENSMUSG00000030264	ENSMUSG00000030264	ENSMUSG00000030264	6	113012104	113034050	111	113		
5520	3	9446634	9493143	ENSMUSG00000034269	ENSMUSG00000034269	ENSMUSG00000034269	6	113043416	113119201	111	113		
5521	3	9666146	9719076	ENSMUSG00000030269	ENSMUSG00000030269	ENSMUSG00000030269	6	113203670	113246979	111	113		
5522	3	9720510	9744125	ENSMUSG00000030270	ENSMUSG00000030270	ENSMUSG00000030270	6	113248084	113271348	111	113		
5523	3	9748429	9764702	ENSMUSG00000001632	ENSMUSG00000001632	ENSMUSG00000001632	6	113272974	113290494	111	113		
5524	3	9766628	9783421	ENSMUSG00000030271	ENSMUSG00000030271	ENSMUSG00000030271	6	113292779	113299963	111	113		
5525	3	9774032	9786661	ENSMUSG00000030272	ENSMUSG00000030272	ENSMUSG00000030272	6	113299901	113309699	111	113		
5526	3	9854533	9860700	ENSMUSG00000051169	ENSMUSG00000051169	ENSMUSG00000051169	6	113381109	113385054	111	113		
5527	3	9883398	9895740	ENSMUSG00000030278	ENSMUSG00000030278	ENSMUSG00000030278	6	113390413	113401532	111	113		
5528	3	9907238	9911033	ENSMUSG00000051256	ENSMUSG00000051256	ENSMUSG00000051256	6	113408380	113413979	111	113		
5529	3	9919303	9933079	ENSMUSG00000043088	ENSMUSG00000043088	ENSMUSG00000043088	6	113424601	113436528	111	113		
5530	3	9933782	9950313	ENSMUSG00000030281	ENSMUSG00000030281	ENSMUSG00000030281	6	113437351	113448915	111	113		
5531	3	9950540	9961712	ENSMUSG00000030284	ENSMUSG00000030284	ENSMUSG00000030284	6	113449203	113459115	111	113		
5532	3	9962226	9969078	ENSMUSG00000045009	ENSMUSG00000045009	ENSMUSG00000045009	6	113459700	113467708	111	113		
5533	3	9979221	10003804	ENSMUSG00000030286	ENSMUSG00000030286	ENSMUSG00000030286	6	113480654	113497411	111	113		
5534	3	10043113	10116344	ENSMUSG00000034023	ENSMUSG00000034023	ENSMUSG00000034023	6	113497462	113562062	111	113		
5535	3	10132333	10143871	ENSMUSG00000033940	ENSMUSG00000033940	ENSMUSG00000033940	6	113570547	113581914	111	113		
5536	3	10181563	10260427	ENSMUSG00000060477	ENSMUSG00000060477	ENSMUSG00000060477	6	113604280	113660528	111	113		
5537	3	10302434	10307535	ENSMUSG00000064177	ENSMUSG00000064177	ENSMUSG00000064177	6	113681897	113685657	111	113		
5538	3	10317215	10337766	ENSMUSG00000030298	ENSMUSG00000030298	ENSMUSG00000030298	6	113694127	113703775	111	113		
5539	3	10340707	10466549	ENSMUSG00000030302	ENSMUSG00000030302	ENSMUSG00000030302	6	113711445	113857153	111	113		
5540	3	11009440	11055927	ENSMUSG00000030310	ENSMUSG00000030310	ENSMUSG00000030310	6	114248369	114283117	111	113		
5541	3	11275687	11279415	ENSMUSG00000053004	ENSMUSG00000053004	ENSMUSG00000053004	6	114363531	114448891	111	113		
5542	3	11289083	11571556	ENSMUSG00000030314	ENSMUSG00000030314	ENSMUSG00000030314	6	114638616	114742701	111	113		
5543	3	11572544	11737037	ENSMUSG00000030315	ENSMUSG00000030315	ENSMUSG00000030315	6	114827692	114887371	111	113		
5544	3	11806920	11863355	ENSMUSG00000030316	ENSMUSG00000030316	ENSMUSG00000030316	6	114970000	115003493	111	113		
5545	3	12020862	12207907	ENSMUSG00000009394	ENSMUSG00000009394	ENSMUSG00000009394	6	115100601	115247615	111	113		
5546	3	12169578	12175851	ENSMUSG00000030317	ENSMUSG00000030317	ENSMUSG00000030317	6	115211438	115217467	111	113		
5547	3	12367959	12450840	ENSMUSG00000000440	ENSMUSG00000000440	ENSMUSG00000000440	6	115387685	115456020	111	113		
5548	3	12501025	12549809	ENSMUSG00000042389	ENSMUSG00000042389	ENSMUSG00000042389	6	115510323	115543955	111	113		
5549	3	12573571	12600205	ENSMUSG00000000439	ENSMUSG00000000439	ENSMUSG00000000439	6	115567557	115584289	111	113		
5550	3	12600108	12680678	ENSMUSG00000000441	ENSMUSG00000000441	ENSMUSG00000000441	6	115584217	115642173	111	113		
5551	3	12750061	12775719	ENSMUSG00000059900	ENSMUSG00000059900	ENSMUSG00000059900	6	115696053	115707998	111	113		
5552	3	12813220	12851301	ENSMUSG00000030319	ENSMUSG00000030319	ENSMUSG00000030319	6	115740194	115770488	111	113		
5553	3	130602855	130630184	ENSMUSG00000030321	ENSMUSG00000030321	ENSMUSG00000030321	6	115783396	115803751	112	114		
5554	3	130632483	130641542	ENSMUSG00000030322	ENSMUSG00000030322	ENSMUSG00000030322	6	115807963	115818829	112	114		
5555	3	130641658	130721880	ENSMUSG00000030323	ENSMUSG00000030323	ENSMUSG00000030323	6	115819147	115892309	112	114		
5556	3	130730172	130736867	ENSMUSG00000030324	ENSMUSG00000030324	ENSMUSG00000030324	6	115897546	115904449	112	114		
5557	3	130744747	130752894	ENSMUSG00000042279	ENSMUSG00000042279	ENSMUSG00000042279	6	115910558	115915852	112	114		
5558	3	130756708	130808351	ENSMUSG00000030123	ENSMUSG00000030123	ENSMUSG00000030123	6	115921359	115960583	112	114		
5559	3	130849327	131081999	ENSMUSG00000030126	ENSMUSG00000030126	ENSMUSG00000030126	6	115986965	116099939	112	114		
5560	10	45431047	45488267	ENSMUSG00000042213	ENSMUSG00000042213	ENSMUSG00000042213	6	116250397	116295917	113	115		
5561	10	45270047	45279339	ENSMUSG00000025702	ENSMUSG00000025702	ENSMUSG00000025702	6	116367068	116371942	113	115		
5562	10	45189635	45261567	ENSMUSG00000025701	ENSMUSG00000025701	ENSMUSG00000025701	6	116375696	116426797	113	115		
5563	10	45118108	45123282	ENSMUSG00000053391	ENSMUSG00000053391	ENSMUSG00000053391	6	116459230	116460159	113	115		
5564	10	44774861	44810161	ENSMUSG00000042129	ENSMUSG00000042129	ENSMUSG00000042129	6	116598627	116639410	113	115		
5565	10	44791583	44794264	ENSMUSG00000048489	ENSMUSG00000048489	ENSMUSG00000048489	6	116616303	116618407	113	115		
5566	10	44726967	44750585	ENSMUSG00000048108	ENSMUSG00000048108	ENSMUSG00000048108	6	116658344	116682377	113	115		
5567	10	44191371	44200548	ENSMUSG00000061353	ENSMUSG00000061353	ENSMUSG00000061353	6	117134182	117146986	113	115		
5568	10	43459314	43464332	ENSMUSG00000059689	ENSMUSG00000059689	ENSMUSG00000059689	6	117806873	117811574	113	115		
5569	10	43187096	43191786	ENSMUSG00000004988	ENSMUSG00000004988	ENSMUSG00000004988	6	117899348	117902954	113	115		
5570	10	43009989	43045193	ENSMUSG00000030134	ENSMUSG00000030134	ENSMUSG00000030134	6	118050130	118055130	113	115		
5571	10	42953940	43000762	ENSMUSG00000042042	ENSMUSG00000042042	ENSMUSG00000042042	6	118073065	118104759	113	115		
5572	10	42892533	42944955	ENSMUSG00000030110	ENSMUSG00000030110	ENSMUSG00000030110	6	118119472	118162752	113	115		
5573	10	42598255	42646862	ENSMUSG00000030138	ENSMUSG00000030138	ENSMUSG00000030138	6	118349000	118385036	113	115		
5574	10	38157699	38186544	ENSMUSG00000030145	ENSMUSG00000030145	ENSMUSG00000030145	6	118394515	118399053	113	115		
5575	12	2094650	2670626	ENSMUSG00000051331	ENSMUSG00000051331	ENSMUSG00000051331	6	118559511	119023135	114	116		
5576	12	1925477	1983938	ENSMUSG00000041477	ENSMUSG00000041477	ENSMUSG00000041477	6	119140912	119186560	114	116		
5577	12	1771384	1898131	ENSMUSG00000041460	ENSMUSG00000041460	ENSMUSG00000041460	6	119202145	119316176	114	116		
5578	12	1799956	1816179	ENSMUSG00000055003	ENSMUSG00000055003	ENSMUSG00000055003	6	119280752	119296352	114	116		
5579	12	1670408	1768098	ENSMUSG00000030168	ENSMUSG00000030168	ENSMUSG00000030168	6	119318769	119383102	114	116		
5580	12	1596483	1626640	ENSMUSG00000030170	ENSMUSG00000030170	ENSMUSG00000030170	6	119398153	119509937	114	116		
5581	12	1545421	1573592	ENSMUSG00000030019	ENSMUSG00000030019	ENSMUSG00000030019	6	119445390	119448503	114	116		
5582	12	1007174	1472958	ENSMUSG00000030172	ENSMUSG00000030172	ENSMUSG00000030172	6	119540864	119790837	114	116		
5583	12	891972	969480	ENSMUSG00000030166	ENSMUSG00000030166	ENSMUSG00000030166	6	119868333	119888441	114	116		
5584	12	732993	888219	ENSMUSG00000045962	ENSMUSG00000045962	ENSMUSG00000045962	6	119889957	120003398	114	116		
5585	12	543724	643016	ENSMUSG00000041377	ENSMUSG00000041377	ENSMUSG00000041377	6	120163522	120164428	114	116		
5586	12	439804	541319	ENSMUSG00000041372	ENSMUSG00000041372	ENSMUSG00000041372	6	120169429	120260178	114	116		
5587	12	381084	422065	ENSMUSG00000030177	ENSMUSG00000030177	ENSMUSG00000030177	6	120290416	120322979	114	116		
5588	12	263249	368643	ENSMUSG00000030180	ENSMUSG00000030180	ENSMUSG00000030180	6	120330114	120397845	114	116		
5589	22	15945858	15971387	ENSMUSG00000002897	ENSMUSG00000002897	ENSMUSG00000002897	6	120428866	120449348	115	117		
5590	22	15998411	16020169	ENSMUSG00000058979	ENSMUSG00000058979	ENSMUSG00000058979	6	120475114	120496924	115	117		
5591	22	16336627	16413845	ENSMUSG00000071226	ENSMUSG00000071226	ENSMUSG00000071226	6	120720333	120731005	115	117		
5592	22	16423183	16453647	ENSMUSG00000004902	ENSMUSG00000004902	ENSMUSG00000004902	6	120751661	120759786	115	117		
5593	22	16501485	16591991	ENSMUSG00000009112	ENSMUSG00000009112	ENSMUSG00000009112	6	120801849	120858461	115	117		
5594	22	16596908	16636782	ENSMUSG00000004446	ENSMUSG00000004446	ENSMUSG00000004446	6	120858471	120882453	115	117		
5595	22	16650418	16694635	ENSMUSG00000051586	ENSMUSG00000051586	ENSMUSG00000051586	6	120897331	120948338	115	117		
5596	22	16704441	16769593	ENSMUSG00000003178	ENSMUSG00000003178	ENSMUSG00000003178	6	120972859	121047209	115	117		
5597	22	16973242	16993903	ENSMUSG00000030137	ENSMUSG00000030137	ENSMUSG00000030137	6	121186003	121191698	115	117		
5598	12	200142	239479	ENSMUSG00000030108	ENSMUSG00000030108	ENSMUSG00000030108	6	121265915	121303340	116	118		
5599	12	46310	154457	ENSMUSG00000040797	ENSMUSG00000040797	ENSMUSG00000040797	6	121338552	121439297	116	118		
5600	12	9102453	9159825	ENSMUSG00000030111	ENSMUSG00000030111	ENSMUSG00000030111	6	121601908	121644181	117	119		
5601	12	9033484	9054605	ENSMUSG00000030114	ENSMUSG00000030114	ENSMUSG00000030114	6	122236213	122248452	117	119		
5602	12	8984243	8993519	ENSMUSG00000007458	ENSMUSG00000007458	ENSMUSG00000007458	6	122274629	122283299	117	119		
5603	12	8689807	8706700	ENSMUSG00000030116	ENSMUSG00000030116	ENSMUSG00000030116	6	122479962	122494253	117	119		
5604	12	8648147	8650880	ENSMUSG00000040627	ENSMUSG00000040627	ENSMUSG00000040627	6	122519432	122529799	117	119		
5605	12	7693264	7709769	ENSMUSG00000040613	ENSMUSG00000040613	ENSMUSG00000040613	6	122543411	122565746	117	120	here	This block size is less than the threshold size, so merged with the neighboring block
5606	12	7733649	7739656	ENSMUSG00000030117	ENSMUSG00000030117	ENSMUSG00000030117	6	122571022	122575690	117	120	small blocks merged with neighboring block	1 -> 2
5607	12	8076626	8099384	ENSMUSG00000003154	ENSMUSG00000003154	ENSMUSG00000003154	6	122786311	122810985	117	120		
5608	12	8102179	8110280	ENSMUSG00000040552	ENSMUSG00000040552	ENSMUSG00000040552	6	122813577	122821624	117	120		
5609	12	8499975	8521327	ENSMUSG00000023349	ENSMUSG00000023349	ENSMUSG00000023349	6	123195476	123212638	117	120		
5610	12	8557403	8566225	ENSMUSG00000030144	ENSMUSG00000030144	ENSMUSG00000030144	6	123227736	123240884	117	120		
5611	12	8577168	8584811	ENSMUSG00000030142	ENSMUSG00000030142	ENSMUSG00000030142	6	123247408	123255490	117	120		
5612	12	7515021	7547553	ENSMUSG00000008845	ENSMUSG00000008845	ENSMUSG00000008845	6	124270275	124296146	118	121		
5613	12	7234224	7255342	ENSMUSG00000005069	ENSMUSG00000005069	ENSMUSG00000005069	6	124362460	124380656	118	121		
5614	12	7176249	7202795	ENSMUSG00000008153	ENSMUSG00000008153	ENSMUSG00000008153	6	124396386	124430343	118	121		
5615	12	7138294	7153069	ENSMUSG00000038527	ENSMUSG00000038527	ENSMUSG00000038527	6	124458753	124476262	118	121		
5616	12	7058091	7135416	ENSMUSG00000055172	ENSMUSG00000055172	ENSMUSG00000055172	6	124478024	124488747	118	121		
5617	12	7038278	7048594	ENSMUSG00000038521	ENSMUSG00000038521	ENSMUSG00000038521	6	124495970	124602563	118	121		
5618	12	6955608	6996103	ENSMUSG00000004270	ENSMUSG00000004270	ENSMUSG00000004270	6	124628752	124669990	118	121		
5619	12	6950254	6955397	ENSMUSG00000004268	ENSMUSG00000004268	ENSMUSG00000004268	6	124670000	124677774	118	121		
5620	12	6930763	6940740	ENSMUSG00000004266	ENSMUSG00000004266	ENSMUSG00000004266	6	124686727	124698484	118	121		
5621	12	6923546	6925425	ENSMUSG00000072772	ENSMUSG00000072772	ENSMUSG00000072772	6	124704802	124706698	118	121		
5622	12	6903887	6921743	ENSMUSG00000004263	ENSMUSG00000004263	ENSMUSG00000004263	6	124708161	124722106	118	121		
5623	12	6893875	6903120	ENSMUSG00000004267	ENSMUSG00000004267	ENSMUSG00000004267	6	124725675	124735128	118	121		
5624	12	6884213	6893666	ENSMUSG00000030125	ENSMUSG00000030125	ENSMUSG00000030125	6	124735482	124745337	118	121		
5625	12	6850365	6852713	ENSMUSG00000038451	ENSMUSG00000038451	ENSMUSG00000038451	6	124774294	124776234	118	121		
5626	12	6831553	6846054	ENSMUSG00000038429	ENSMUSG00000038429	ENSMUSG00000038429	6	124780638	124795066	118	121		
5627	12	6828262	6830377	ENSMUSG00000023505	ENSMUSG00000023505	ENSMUSG00000023505	6	124795854	124799317	118	121		
5628	12	6820713	6826819	ENSMUSG00000023439	ENSMUSG00000023439	ENSMUSG00000023439	6	124799859	124805894	118	121		
5629	12	6807465	6819271	ENSMUSG00000023191	ENSMUSG00000023191	ENSMUSG00000023191	6	124806714	124823306	118	121		
5630	12	6769005	6800233	ENSMUSG00000023274	ENSMUSG00000023274	ENSMUSG00000023274	6	124830325	124853807	118	121		
5631	12	6751931	6757880	ENSMUSG00000030124	ENSMUSG00000030124	ENSMUSG00000030124	6	124869980	124877324	118	121		
5632	12	6746019	6749866	ENSMUSG00000030122	ENSMUSG00000030122	ENSMUSG00000030122	6	124879801	124883334	118	121		
5633	12	6727434	6732561	ENSMUSG00000030120	ENSMUSG00000030120	ENSMUSG00000030120	6	124897095	124901763	118	121		
5634	12	6703473	6711283	ENSMUSG00000030127	ENSMUSG00000030127	ENSMUSG00000030127	6	124924435	124930994	118	121		
5635	12	6673221	6677263	ENSMUSG00000030329	ENSMUSG00000030329	ENSMUSG00000030329	6	124962339	124968713	118	121		
5636	12	6646078	6668751	ENSMUSG00000038346	ENSMUSG00000038346	ENSMUSG00000038346	6	124974858	125003256	118	121		
5637	12	6629710	6642565	ENSMUSG00000030330	ENSMUSG00000030330	ENSMUSG00000030330	6	125005462	125014620	118	121		
5638	12	6617503	6626841	ENSMUSG00000072770	ENSMUSG00000072770	ENSMUSG00000072770	6	125015594	125028862	118	121		
5639	12	6598262	6615548	ENSMUSG00000067714	ENSMUSG00000067714	ENSMUSG00000067714	6	125033540	125047698	118	121		
5640	12	6549510	6586812	ENSMUSG00000063870	ENSMUSG00000063870	ENSMUSG00000063870	6	125061782	125096133	118	121		
5641	12	6536290	6547389	ENSMUSG00000038279	ENSMUSG00000038279	ENSMUSG00000038279	6	125097528	125110371	118	121		
5642	12	6519004	6535456	ENSMUSG00000038271	ENSMUSG00000038271	ENSMUSG00000038271	6	125110863	125127393	118	121		
5643	12	6472806	6511381	ENSMUSG00000038252	ENSMUSG00000038252	ENSMUSG00000038252	6	125133628	125157214	118	121		
5644	12	6471579	6472718	ENSMUSG00000030335	ENSMUSG00000030335	ENSMUSG00000030335	6	125157819	125160011	118	121		
5645	12	6441667	6450326	ENSMUSG00000030337	ENSMUSG00000030337	ENSMUSG00000030337	6	125181200	125206163	118	121		
5646	12	6431676	6445876	ENSMUSG00000038213	ENSMUSG00000038213	ENSMUSG00000038213	6	125189820	125197479	118	121		
5647	12	6424327	6431144	ENSMUSG00000030336	ENSMUSG00000030336	ENSMUSG00000030336	6	125198242	125202629	118	121		
5648	12	6363595	6370994	ENSMUSG00000030339	ENSMUSG00000030339	ENSMUSG00000030339	6	125272195	125279473	118	121		
5649	12	6326276	6354651	ENSMUSG00000030340	ENSMUSG00000030340	ENSMUSG00000030340	6	125287329	125310559	118	121		
5650	12	6308185	6321522	ENSMUSG00000030341	ENSMUSG00000030341	ENSMUSG00000030341	6	125315374	125328103	118	121		
5651	12	6289863	6307910	ENSMUSG00000038167	ENSMUSG00000038167	ENSMUSG00000038167	6	125328657	125344458	118	121		
5652	12	6179134	6217664	ENSMUSG00000030342	ENSMUSG00000030342	ENSMUSG00000030342	6	125425885	125460379	118	121		

5653	12	5928308	6103924	ENSMUSG00000001930	ENSMUSG00000001930	ENSMUSG00000001930	6	125520072	125652158	118	121		
5654	12	5542081	5925659	ENSMUSG00000038115	ENSMUSG00000038115	ENSMUSG00000038115	6	125676245	126005745	118	121		
5655	12	5473527	5474725	ENSMUSG00000049107	ENSMUSG00000049107	ENSMUSG00000049107	6	126067031	126130540	118	121		
5656	12	5023346	5026210	ENSMUSG00000045534	ENSMUSG00000045534	ENSMUSG00000045534	6	126498974	126500782	118	121		
5657	12	4890806	4892293	ENSMUSG00000047976	ENSMUSG00000047976	ENSMUSG00000047976	6	126606016	126610415	118	121		
5658	12	4789372	4791132	ENSMUSG00000038077	ENSMUSG00000038077	ENSMUSG00000038077	6	126673953	126705813	118	121		
5659	12	4628553	4666934	ENSMUSG00000000399	ENSMUSG00000000399	ENSMUSG00000000399	6	126787574	126814744	118	121		
5660	12	4594938	4624604	ENSMUSG00000030344	ENSMUSG00000030344	ENSMUSG00000030344	6	126825874	126839734	118	121		
5661	12	4569747	4593291	ENSMUSG00000030345	ENSMUSG00000030345	ENSMUSG00000030345	6	126841829	126887458	118	121		
5662	12	4518323	4539473	ENSMUSG00000030346	ENSMUSG00000030346	ENSMUSG00000030346	6	126889814	126901629	118	121		
5663	12	4467195	4517898	ENSMUSG00000030347	ENSMUSG00000030347	ENSMUSG00000030347	6	126905585	126941323	118	121		
5664	12	4413569	4425041	ENSMUSG00000000183	ENSMUSG00000000183	ENSMUSG00000000183	6	126981205	126990337	118	121		
5665	12	4347654	4359155	ENSMUSG00000000182	ENSMUSG00000000182	ENSMUSG00000000182	6	127038593	127046844	118	121		
5666	12	4300632	4332599	ENSMUSG00000038028	ENSMUSG00000038028	ENSMUSG00000038028	6	127052982	127075160	118	121		
5667	12	4253199	4284777	ENSMUSG00000000184	ENSMUSG00000000184	ENSMUSG00000000184	6	127091327	127116667	118	121		
5668	12	3788288	3852863	ENSMUSG00000037997	ENSMUSG00000037997	ENSMUSG00000037997	6	127419342	127459858	118	121		
5669	12	3627370	3732524	ENSMUSG00000061414	ENSMUSG00000061414	ENSMUSG00000061414	6	127543594	127595557	118	121		
5670	12	3596086	3613157	ENSMUSG00000037976	ENSMUSG00000037976	ENSMUSG00000037976	6	127615452	127633279	118	121		
5671	12	3470686	3573397	ENSMUSG00000030350	ENSMUSG00000030350	ENSMUSG00000030350	6	127654626	127734366	118	121		
5672	12	30998193	31036117	ENSMUSG00000030351	ENSMUSG00000030351	ENSMUSG00000030351	6	127853241	127918650	out-of-place		here	Presence of a singleton made AutoGRAPH to break the block
5673	12	3056806	3265985	ENSMUSG00000030352	ENSMUSG00000030352	ENSMUSG00000030352	6	127927019	128109197	118	122	Presence of singletons	1 -> 2
5674	12	2938757	3020103	ENSMUSG00000030353	ENSMUSG00000030353	ENSMUSG00000030353	6	128192762	128266427	118	122		
5675	12	2870294	2920558	ENSMUSG00000001521	ENSMUSG00000001521	ENSMUSG00000001521	6	128288698	128303409	118	122		
5676	12	2856650	2868885	ENSMUSG00000048668	ENSMUSG00000048668	ENSMUSG00000048668	6	128322979	128328402	118	122		
5677	12	2837113	2856564	ENSMUSG00000001517	ENSMUSG00000001517	ENSMUSG00000001517	6	128328595	128341501	118	122		
5678	12	2804773	2814451	ENSMUSG00000001520	ENSMUSG00000001520	ENSMUSG00000001520	6	128365385	128373870	118	122		
5679	12	2792124	2804367	ENSMUSG00000001518	ENSMUSG00000001518	ENSMUSG00000001518	6	128375064	128390529	118	122		
5680	12	2774414	2783381	ENSMUSG00000030357	ENSMUSG00000030357	ENSMUSG00000030357	6	128395157	128404275	118	122		
5681	12	31158951	31245435	ENSMUSG00000047228	ENSMUSG00000047228	ENSMUSG00000047228	6	128505901	128547225	out-of-place			
5682	12	9796353	9804764	ENSMUSG00000030156	ENSMUSG00000030156	ENSMUSG00000030156	6	129233017	129241062	119	123		
5683	12	10015281	10029459	ENSMUSG00000053063	ENSMUSG00000053063	ENSMUSG00000053063	6	129315979	129330996	119	123		
5684	12	10054493	10062431	ENSMUSG00000030158	ENSMUSG00000030158	ENSMUSG00000030158	6	129341208	129351196	119	123		
5685	12	10036931	10043146	ENSMUSG00000030159	ENSMUSG00000030159	ENSMUSG00000030159	6	129362990	129371026	119	123		
5686	12	10074543	10109832	ENSMUSG00000046080	ENSMUSG00000046080	ENSMUSG00000046080	6	129374556	129388262	119	123		
5687	12	10113421	10142851	ENSMUSG00000033082	ENSMUSG00000033082	ENSMUSG00000033082	6	129392381	129417514	119	123		
5688	12	10202171	10216004	ENSMUSG00000030162	ENSMUSG00000030162	ENSMUSG00000030162	6	129453359	129472800	119	123		
5689	12	10223355	10235651	ENSMUSG00000030160	ENSMUSG00000030160	ENSMUSG00000030160	6	129479874	129484920	119	123		
5690	12	10353250	10358867	ENSMUSG00000030165	ENSMUSG00000030165	ENSMUSG00000030165	6	129559176	129564465	119	123		
5691	12	10416857	10454012	ENSMUSG00000030149	ENSMUSG00000030149	ENSMUSG00000030149	6	129577264	129588327	119	123		
5692	12	10663049	10718158	ENSMUSG00000032899	ENSMUSG00000032899	ENSMUSG00000032899	6	131264837	131279520	119	123		
5693	12	10742956	10767171	ENSMUSG00000030189	ENSMUSG00000030189	ENSMUSG00000030189	6	131330551	131354143	119	123		
5694	12	10845480	10846436	ENSMUSG00000054497	ENSMUSG00000054497	ENSMUSG00000054497	6	131595585	131596523	119	123		
5695	12	11229866	11230810	ENSMUSG00000057699	ENSMUSG00000057699	ENSMUSG00000057699	6	132922605	132923537	119	123		
5696	12	11694055	11939588	ENSMUSG00000030199	ENSMUSG00000030199	ENSMUSG00000030199	6	134091327	134235851	119	123		
5697	12	12115145	12143892	ENSMUSG00000030200	ENSMUSG00000030200	ENSMUSG00000030200	6	134362022	134404417	119	123		
5698	12	12164958	12311013	ENSMUSG00000030201	ENSMUSG00000030201	ENSMUSG00000030201	6	134416479	134507739	119	123		
5699	12	12373466	12387515	ENSMUSG00000032718	ENSMUSG00000032718	ENSMUSG00000032718	6	134574903	134598181	119	123		
5700	12	12401322	12511105	ENSMUSG00000042992	ENSMUSG00000042992	ENSMUSG00000042992	6	134606674	134676863	119	123		
5701	12	12520098	12606584	ENSMUSG00000030203	ENSMUSG00000030203	ENSMUSG00000030203	6	134681161	134758168	119	123		
5702	12	12656098	12689308	ENSMUSG00000032652	ENSMUSG00000032652	ENSMUSG00000032652	6	134795892	134823576	119	123		
5703	12	12705092	12728722	ENSMUSG00000032641	ENSMUSG00000032641	ENSMUSG00000032641	6	134834853	134863553	119	123		
5704	12	12761576	12766569	ENSMUSG00000003031	ENSMUSG00000003031	ENSMUSG00000003031	6	134886110	134889751	119	123		
5705	12	12770130	12874176	ENSMUSG00000030204	ENSMUSG00000030204	ENSMUSG00000030204	6	134977313	134989464	119	123		
5706	12	12934983	12957833	ENSMUSG00000046733	ENSMUSG00000046733	ENSMUSG00000046733	6	135031383	135050402	119	123		
5707	12	12984976	12994585	ENSMUSG00000030205	ENSMUSG00000030205	ENSMUSG00000030205	6	135071684	135082600	119	123		
5708	12	13019070	13044488	ENSMUSG00000042770	ENSMUSG00000042770	ENSMUSG00000042770	6	135103221	135133891	119	123		
5709	12	13088582	13127648	ENSMUSG00000030207	ENSMUSG00000030207	ENSMUSG00000030207	6	135163753	135209662	119	123		
5710	12	13240900	13260971	ENSMUSG00000030208	ENSMUSG00000030208	ENSMUSG00000030208	6	135328624	135348868	119	123		
5711	12	13605411	14024319	ENSMUSG00000030209	ENSMUSG00000030209	ENSMUSG00000030209	6	135697791	136009994	119	123		
5712	12	14409883	14542964	ENSMUSG00000030213	ENSMUSG00000030213	ENSMUSG00000030213	6	136483047	136571591	119	123		
5713	12	14547863	14612058	ENSMUSG00000030214	ENSMUSG00000030214	ENSMUSG00000030214	6	136576267	136626041	119	123		
5714	12	14656843	14740696	ENSMUSG00000042638	ENSMUSG00000042638	ENSMUSG00000042638	6	136661481	136745938	119	123		
5715	12	14818672	14819612	ENSMUSG00000060032	ENSMUSG00000060032	ENSMUSG00000060032	6	136772520	136774270	119	123		
5716	12	14847773	14868053	ENSMUSG00000047515	ENSMUSG00000047515	ENSMUSG00000047515	6	136803960	136804703	119	123		
5717	12	14873512	14887680	ENSMUSG00000030217	ENSMUSG00000030217	ENSMUSG00000030217	6	136812647	136821796	119	123		
5718	12	14925119	14930025	ENSMUSG00000030218	ENSMUSG00000030218	ENSMUSG00000030218	6	136836632	136840001	119	123		
5719	12	14958241	14982750	ENSMUSG00000030219	ENSMUSG00000030219	ENSMUSG00000030219	6	136871583	136886376	119	123		
5720	12	14986232	15005870	ENSMUSG00000030220	ENSMUSG00000030220	ENSMUSG00000030220	6	136887905	136905913	119	123		
5721	12	15017245	15026066	ENSMUSG00000064330	ENSMUSG00000064330	ENSMUSG00000064330	6	136918747	136933052	119	123		
5722	12	15151985	15265571	ENSMUSG00000030222	ENSMUSG00000030222	ENSMUSG00000030222	6	137019021	137134695	119	123		
5723	12	15366754	15641602	ENSMUSG00000030223	ENSMUSG00000030223	ENSMUSG00000030223	6	137216661	137427429	119	123		
5724	12	15664343	15833601	ENSMUSG00000015766	ENSMUSG00000015766	ENSMUSG00000015766	6	137442857	137535213	119	123		
5725	12	15926600	15947670	ENSMUSG00000030224	ENSMUSG00000030224	ENSMUSG00000030224	6	137699320	137714808	119	123		
5726	12	16233869	16316967	ENSMUSG00000044378	ENSMUSG00000044378	ENSMUSG00000044378	6	137947786	138037394	119	123		
5727	12	16391343	16408610	ENSMUSG00000008540	ENSMUSG00000008540	ENSMUSG00000008540	6	138104733	138120948	119	123		
5728	12	16592626	16649348	ENSMUSG00000030226	ENSMUSG00000030226	ENSMUSG00000030226	6	138328677	138546164	119	123		
5729	12	18125073	18134381	ENSMUSG00000071118	ENSMUSG00000071118	ENSMUSG00000071118	6	139457410	139465280	119	123		
5730	12	18305741	18692615	ENSMUSG00000030228	ENSMUSG00000030228	ENSMUSG00000030228	6	139586084	139931690	119	123		
5731	12	18727379	18782216	ENSMUSG00000030230	ENSMUSG00000030230	ENSMUSG00000030230	6	139952128	140003837	119	123		
5732	12	18782308	18783388	ENSMUSG00000041791	ENSMUSG00000041791	ENSMUSG00000041791	6	140003963	140005184	119	123		
5733	12	19173995	19420598	ENSMUSG00000030231	ENSMUSG00000030231	ENSMUSG00000030231	6	140388924	140557312	119	123		
5734	12	19448267	19562921	ENSMUSG00000030232	ENSMUSG00000030232	ENSMUSG00000030232	6	140586101	140615231	119	123		
5735	12	20413486	20725646	ENSMUSG00000041741	ENSMUSG00000041741	ENSMUSG00000041741	6	141211675	141461757	119	123		
5736	12	20739666	20797585	ENSMUSG00000030235	ENSMUSG00000030235	ENSMUSG00000030235	6	141486956	141532126	119	123		
5737	12	21417085	21423683	ENSMUSG00000041681	ENSMUSG00000041681	ENSMUSG00000041681	6	142255650	142261039	119	123		
5738	12	21481878	21514549	ENSMUSG00000041671	ENSMUSG00000041671	ENSMUSG00000041671	6	142302894	142319273	119	123		
5739	12	21513983	21545796	ENSMUSG00000030243	ENSMUSG00000030243	ENSMUSG00000030243	6	142319942	142335588	119	123		
5740	12	21546016	21562358	ENSMUSG00000030245	ENSMUSG00000030245	ENSMUSG00000030245	6	142344465	142361080	119	123		
5741	12	21570508	21576724	ENSMUSG00000071112	ENSMUSG00000071112	ENSMUSG00000071112	6	142371055	142376952	119	123		
5742	12	21580392	21648821	ENSMUSG00000030244	ENSMUSG00000030244	ENSMUSG00000030244	6	142379835	142430179	119	123		
5743	12	21809156	21819014	ENSMUSG00000030247	ENSMUSG00000030247	ENSMUSG00000030247	6	142522146	142528581	119	123		
5744	12	21845245	21980875	ENSMUSG00000030249	ENSMUSG00000030249	ENSMUSG00000030249	6	142546356	142659440	119	123		
5745	12	22090426	22109869	ENSMUSG00000030282	ENSMUSG00000030282	ENSMUSG00000030282	6	142713979	142732929	119	123		
5746	12	22245204	22378915	ENSMUSG00000030283	ENSMUSG00000030283	ENSMUSG00000030283	6	142778763	142921674	119	123		
5747	12	22492808	22588719	ENSMUSG00000030279	ENSMUSG00000030279	ENSMUSG00000030279	6	142968159	143057011	119	123		
5748	12	22669276	22729910	ENSMUSG00000030275	ENSMUSG00000030275	ENSMUSG00000030275	6	143124452	143163842	119	123		
5749	12	23576499	23993904	ENSMUSG00000041540	ENSMUSG00000041540	ENSMUSG00000041540	6	143790486	144112384	119	123		
5750	12	24862209	24946092	ENSMUSG00000030268	ENSMUSG00000030268	ENSMUSG00000030268	6	144956319	145005971	119	123		
5751	12	25096508	25152535	ENSMUSG00000030263	ENSMUSG00000030263	ENSMUSG00000030263	6	145078973	145132087	119	123		
5752	12	25152488	25239361	ENSMUSG00000043541	ENSMUSG00000043541	ENSMUSG00000043541	6	145132211	145162506	119	123		
5753	12	25239473	25250293	ENSMUSG00000040370	ENSMUSG00000040370	ENSMUSG00000040370	6	145168315	145174098	119	123		
5754	12	25249449	25295121	ENSMUSG00000030265	ENSMUSG00000030265	ENSMUSG00000030265	6	145182237	145207378	119	123		
5755	12	25520283	25597445	ENSMUSG00000054966	ENSMUSG00000054966	ENSMUSG00000054966	6	145354394	145391161	119	123		
5756	12	26003265	26117068	ENSMUSG00000030259	ENSMUSG00000030259	ENSMUSG00000030259	6	145765542	145774743	119	123		
5757	12	26164228	26169113	ENSMUSG00000030256	ENSMUSG00000030256	ENSMUSG00000030256	6	145819896	145822499	119	123		
5758	12	26239789	26277134	ENSMUSG00000030255	ENSMUSG00000030255	ENSMUSG00000030255	6	145891306	145922386	119	123		
5759	12	26381609	26877347	ENSMUSG00000030287	ENSMUSG00000030287	ENSMUSG00000030287	6	146068634	146459082	119	123		
5760	12	26949391	26982210	ENSMUSG00000040250	ENSMUSG00000040250	ENSMUSG00000040250	6	146506802	146534929	119	123		
5761	12	26982583	27010124	ENSMUSG00000040242	ENSMUSG00000040242	ENSMUSG00000040242	6	146542854	146554602	119	123		
5762	12	27017395	27058594	ENSMUSG00000040234	ENSMUSG00000040234	ENSMUSG00000040234	6	146560659	146591700	119	123		
5763	12	27066746	27073947	ENSMUSG00000030291	ENSMUSG00000030291	ENSMUSG00000030291	6	146599749	146607770	119	123		
5764	12	27341921	27366655	ENSMUSG00000001630	ENSMUSG00000001630	ENSMUSG00000001630	6	146682178	146735977	119	123		
5765	12	27377255	27464733	ENSMUSG00000040187	ENSMUSG00000040187	ENSMUSG00000040187	6	146762700	146789816	119	123		
5766	12	27514832	27546321	ENSMUSG00000030292	ENSMUSG00000030292	ENSMUSG00000030292	6	146807274	146828574	119	123		
5767	12	27568406	27739761	ENSMUSG00000016487	ENSMUSG00000016487	ENSMUSG00000016487	6	146845684	146989193	119	123		
5768	12	27740695	27741833	ENSMUSG00000040121	ENSMUSG00000040121	ENSMUSG00000040121	6	146989707	146990775	119	123		
5769	12	27754996	27800494	ENSMUSG00000040112	ENSMUSG00000040112	ENSMUSG00000040112	6	146999940	147028072	119	123		
5770	12	27806944	27815458	ENSMUSG00000072662	ENSMUSG00000072662	ENSMUSG00000072662	6	147032232	147044202	119	123		
5771	12	27824454	27847239	ENSMUSG00000040102	ENSMUSG00000040102	ENSMUSG00000040102	6	147048672	147067377	119	123		
5772	12	28006092	28015124	ENSMUSG00000048776	ENSMUSG00000048776	ENSMUSG00000048776	6	147214106	147220540	119	123		
5773	12	28301400	28594365	ENSMUSG00000030301	ENSMUSG00000030301	ENSMUSG00000030301	6	147433041	147589782	119	123		
5774	12	29193400	29378272	ENSMUSG00000030303	ENSMUSG00000030303	ENSMUSG00000030303	6	148004608	148133316	119	123		
5775	12	29381556	29415861	ENSMUSG00000030304	ENSMUSG00000030304	ENSMUSG00000030304	6	148136488	148169192	119	123		
5776	12	29550185	29828959	ENSMUSG00000030306	ENSMUSG00000030306	ENSMUSG00000030306	6	148193842	148401522	119	123		
5777	12	30673190	30740018	ENSMUSG00000040029	ENSMUSG00000040029	ENSMUSG00000040029	6	148727861	148788417	119	123		
5778	12	30753755	30798715	ENSMUSG00000030309	ENSMUSG00000030309	ENSMUSG00000030309	6	148800000	148853341	119	123		
5779	12	31431594	31544759	ENSMUSG00000030313	ENSMUSG00000030313	ENSMUSG00000030313	6	148971158	149058708	119	123		
5780	12	31703889	31717299	ENSMUSG00000039958	ENSMUSG00000039958	ENSMUSG00000039958	6	149098767	149108348	119	123		
5781	12	31715345	31773319	ENSMUSG00000068250	ENSMUSG00000068250	ENSMUSG00000068250	6	149114748	149145852	119	123		
5782	12	32003620	32037298	ENSMUSG00000032712	ENSMUSG00000032712	ENSMUSG00000032712	6	149266601	149292826	119	123		
5783	12	32151452	32422408	ENSMUSG00000003452	ENSMUSG00000003452	ENSMUSG00000003452	6	149366599	149476286	119	123		
5784	19	59289815	59297806	ENSMUSG00000054594	ENSMUSG00000054594	ENSMUSG00000054594	7	3212900	3219220	120	124		
5785	19	59302142	59310848	ENSMUSG00000006335	ENSMUSG00000006335	ENSMUSG00000006335	7	3223409	3232971	120	124		
5786	19	59310649	59326952	ENSMUSG00000008373	ENSMUSG00000008373	ENSMUSG00000008373	7	3233114	3245335	120	124		
5787	19	59333261	59351239	ENSMUSG00000035632	ENSMUSG00000035632	ENSMUSG00000035632	7	3248439	3264187	120	124		
5788	19	59355714	59368756	ENSMUSG00000019734	ENSMUSG00000019734	ENSMUSG00000019734	7	3268877	3280599	120	124		
5789	19	59368921	59385478	ENSMUSG00000035596	ENSMUSG00000035596	ENSMUSG00000035596	7	3280876	3296612	120	124		
5790	19	59386006	59389333	ENSMUSG00000035585	ENSMUSG00000035585	ENSMUSG00000035585	7	3296844	3304091	120	124		
5791	19	59618417	59639864	ENSMUSG00000030428	ENSMUSG00000030428	ENSMUSG00000030428	7	3722673	3737743	120	124		
5792	19	59652212	59665006	ENSMUSG00000035545	ENSMUSG00000035545	ENSMUSG00000035545	7	3740143	3751260	120	124		
5793	19	59665082	59666521	ENSMUSG00000043432	ENSMUSG00000043432	ENSMUSG00000043432	7	3751305	3752762	120	124		
5794	19	59668022	59676234	ENSMUSG00000063838	ENSMUSG00000063838	ENSMUSG00000063838	7	3754347	3767789	120	124		
5795	19	60109351	60116153	ENSMUSG00000062524	ENSMUSG00000062524	ENSMUSG00000062524	7	3940811	3948251	120	124		
5796	19	60247523	60267013	ENSMUSG00000008435	ENSMUSG00000008435	ENSMUSG00000008435	7	4028750	4048696	120	124		
5797	19	60279081	60291102	ENSMUSG00000006154	ENSMUSG00000006154	ENSMUSG00000006154	7	4067829	4082329	120	124		
5798	19	60294095	60320739	ENSMUSG00000019254	ENSMUSG00000019254	ENSMUSG00000019254	7	4084608	4104736	120	124		
5799	19	60336009	60352386	ENSMUSG00000064179	ENSMUSG00000064179	ENSMUSG00000064179	7	4107750	4119062	120	124		
5800	19	60355014	60360496	ENSMUSG00000035458	ENSMUSG00000035458	ENSMUSG00000035458	7	4121455	4125177	120	124		
5801	19	60361848	60369830	ENSMUSG00000055809	ENSMUSG00000055809	ENSMUSG00000055809	7	4126044	4135529	120	124		
5802	19	60376281	60383458	ENSMUSG00000004961	ENSMUSG00000004961	ENSMUSG00000004961	7	4142852	4149654	120	124		
5803	19	60384428	60412654	ENSMUSG00000035429	ENSMUSG00000035429	ENSMUSG00000035429	7	4151701	4206228	120	124		
5804	19	60429928	60432444	ENSMUSG00000045282	ENSMUSG00000045282	ENSMUSG00000045282	7	4231125	4233569	120	124		
5805	19	60432960	60462175	ENSMUSG00000052296	ENSMUSG00000052296	ENSMUSG00000052296	7	4235424	4250021	120	124		
5806	19	60465411	60483540	ENSMUSG00000063802	ENSMUSG00000063802	ENSMUSG00000063802	7	4263608	4288050	120	124		
5807	19	60382212	60515713	ENSMUSG00000035390	ENSMUSG00000035390	ENSMUSG00000035390	7	4294015	4319086	120	124		
5808	19	60516039	60537228	ENSMUSG00000046456	ENSMUSG00000046456	ENSMUSG00000046456	7	4319090	4328169	120	124		
5809	19	60543076	60551300	ENSMUSG00000059851	ENSMUSG00000059851	ENSMUSG00000059851	7	4343202	4350600	120	124		
5810	19	60558480	60563006	ENSMUSG00000049952	ENSMUSG00000049952	ENSMUSG00000049952	7	4356316	4374389	120	124		
5811	19	60567569	60573626	ENSMUSG00000004371	ENSMUSG00000004371	ENSMUSG00000004371	7	4376654	4379588	120	124		
5812	19	60580034	60581383	ENSMUSG00000013091	ENSMUSG00000013091	ENSMUSG00000013091	7	4386035	4387423	120	124		
5813	19	60582425	60587415	ENSMUSG00000030431	ENSMUSG00000030431	ENSMUSG00000030431	7	4387870	4392647	120	124		
5814	19	60636335	60645583	ENSMUSG00000053550	ENSMUSG00000053550	ENSMUSG00000053550	7	4431561	4439261	120	124		
5815	19	60656168	60664824	ENSMUSG00000052605	ENSMUSG00000052605	ENSMUSG00000052605	7	4448047	4469266	120	124		
5816	19	60688385	60690737	ENSMUSG00000035285	ENSMUSG00000035285	ENSMUSG00000035285	7	4525702	4528089	120	124		
5817	19	60691769	60722174	ENSMUSG00000035279	ENSMUSG00000035279	ENSMUSG00000035279	7	4528931	4547908	120	124		
5818	19	60733344	60739323	ENSMUSG00000030433	ENSMUSG00000030433	ENSMUSG00000030433	7	4560270	4566150	120	124		
5819	19	60794558	60802705	ENSMUSG00000061374	ENSMUSG00000061374	ENSMUSG00000061374	7	4610146	4617718	120	124		
5820	19	60803542	60806316	ENSMUSG00000051184	ENSMUSG00000051184	ENSMUSG00000051184	7	4618592	4621573	120	124		
5821	19	60816797	60819976	ENSMUSG00000074405	ENSMUSG00000074405	ENSMUSG00000074405	7	4623463	4636310	120	124		
5822	19	60823919	60827753	ENSMUSG00000043290	ENSMUSG00000043290	ENSMUSG00000043290	7	4637533	4641521	120	124		
5823	19	60851213	60856333	ENSMUSG00000035228	ENSMUSG00000035228	ENSMUSG00000035228	7	4659825	4663610	120	124		
5824	19	60878404	60898944	ENSMUSG00000035203	ENSMUSG00000035203	ENSMUSG00000035203	7	4683380	4701262	120	124		
5825	19	61291201	61324458	ENSMUSG00000046792	ENSMUSG00000046792	ENSMUSG00000046792	7	5734576	5759058	120	124		
5826	19	61344368	61364073	ENSMUSG00000044876	ENSMUSG00000044876	ENSMUSG00000044876	7	5775633	5794332	120	124		
5827	19	61379201	61388956	ENSMUSG00000034660	ENSMUSG00000034660	ENSMUSG00000034660	7	5800177	5819224	120	124		
5828	19	61643020	61673236	ENSMUSG00000054893	ENSMUSG00000054893	ENSMUSG00000054893	7	5889667	5909911	120	124		
5829	19	61607530	61628212	ENSMUSG00000030443	ENSMUSG00000030443	ENSMUSG00000030443	7	5918753	5928672	120	124		
5830	19	61742129	61759981	ENSMUSG00000062861	ENSMUSG00000062861	ENSMUSG00000062861	7	5987396	5999058	120	124		
5831	19	62015615	62043876	ENSMUSG00000002265	ENSMUSG00000002265	ENSMUSG00000002265	7	6310046	6334505	120	124		
5832	19	62774747	62782055	ENSMUSG00000034538	ENSMUSG00000034538	ENSMUSG00000034538	7	6775618	6787084	120	124		
5833	19	62787440	62795570	ENSMUSG00000030393	ENSMUSG00000030393	ENSMUSG00000030393	7	9362115	9370232	120	124		
5834	19	62885189	62891468	ENSMUSG00000034071	ENSMUSG00000034071	ENSMUSG00000034071	7	11321015	11328355	120	124		
5835	19	63180529	63208120	ENSMUSG00000030386	ENSMUSG00000030386	ENSMUSG00000030386	7	11384167	11400202	120	124		
5836	19	63161619	63177716	ENSMUSG00000030385	ENSMUSG00000030385	ENSMUSG00000030385	7	11418400	11462180	120	124		
5837	19	63287022	63301435	ENSMUSG00000070822	ENSMUSG00000070822	ENSMUSG00000070822	7	11668269	11671295	120	124		
5838	19	63329444	63345843	ENSMUSG00000057894	ENSMUSG00000057894	ENSMUSG00000057894	7	11705161	11719037	120	124		
5839	19	63482130	63499066	ENSMUSG00000060397	ENSMUSG00000060397	ENSMUSG00000060397	7	11781474	11793603	120	124		
5840	19	63530197	63545504	ENSMUSG00000054715	ENSMUSG00000054715	ENSMUSG00000054715	7	11797994	11808418	120	124		
5841	19	63679564	63684403	ENSMUSG00000033961	ENSMUSG00000033961	ENSMUSG00000033961	7	11878027	11884488	120	124		
5842	19	63701516	63715244	ENSMUSG00000030382	ENSMUSG00000030382	ENSMUSG00000030382	7	11888525	11898329	120	124		
5843	19	63716711	63723000	ENSMUSG00000049600	ENSMUSG00000049600	ENSMUSG00000049600	7	11906303	11908508	120	124		
5844	19	63553889	63753894	ENSMUSG00000005566	ENSMUSG00000005566	ENSMUSG00000005566	7	11924331	11931210	120	124		
5845	19	63754746	63758298	ENSMUSG00000033916	ENSMUSG00000033916	ENSMUSG00000033916	7	11932198	11934923	120	124		
5846	19	63765097	63776754	ENSMUSG00000030380	ENSMUSG00000030380	ENSMUSG00000030380	7	11943329	11953863	120	124		
5847	19	53365761	53392328	ENSMUSG00000070814	ENSMUSG00000070814	ENSMUSG00000070814	7	12159146	12177659	121	125		

5848	19	53310515	53365372	ENSMUSG00000056394	ENSMUSG00000056394	ENSMUSG00000056394	7	12177462	12211570	121	125			
5849	19	53242916	53305865	ENSMUSG00000033847	ENSMUSG00000033847	ENSMUSG00000033847	7	12229819	12260122	121	125			
5850	19	53016976	53038392	ENSMUSG00000041578	ENSMUSG00000041578	ENSMUSG00000041578	7	15024471	15038416	121	125			
5851	19	52973654	52979753	ENSMUSG00000041571	ENSMUSG00000041571	ENSMUSG00000041571	7	15075737	15080868	121	125			
5852	19	52940613	52952126	ENSMUSG00000041560	ENSMUSG00000041560	ENSMUSG00000041560	7	15096692	15104575	121	125			
5853	19	52908502	52938202	ENSMUSG00000074364	ENSMUSG00000074364	ENSMUSG00000074364	7	15105488	15125998	121	125			
5854	19	52682703	52710309	ENSMUSG00000006024	ENSMUSG00000006024	ENSMUSG00000006024	7	15257165	15276497	121	125			
5855	19	52670304	52679288	ENSMUSG00000006021	ENSMUSG00000006021	ENSMUSG00000006021	7	15278417	15286035	121	125			
5856	19	52623735	52666934	ENSMUSG00000030376	ENSMUSG00000030376	ENSMUSG00000030376	7	15288682	15319029	121	125			
5857	19	52544386	52577795	ENSMUSG00000006019	ENSMUSG00000006019	ENSMUSG00000006019	7	15355819	15380554	121	125			
5858	19	52535880	52537112	ENSMUSG00000074361	ENSMUSG00000074361	ENSMUSG00000074361	7	15395446	15400840	121	125			
5859	19	52504971	52517172	ENSMUSG00000049130	ENSMUSG00000049130	ENSMUSG00000049130	7	15405265	15417773	121	125			
5860	19	52451632	52467048	ENSMUSG00000041375	ENSMUSG00000041375	ENSMUSG00000041375	7	15434171	15442897	121	125			
5861	19	52415921	52427863	ENSMUSG00000002083	ENSMUSG00000002083	ENSMUSG00000002083	7	15468160	15476856	121	125			
5862	19	52325960	52405287	ENSMUSG00000052833	ENSMUSG00000052833	ENSMUSG00000052833	7	15485575	15546309	121	125			
5863	19	52259289	52308849	ENSMUSG00000059273	ENSMUSG00000059273	ENSMUSG00000059273	7	15559388	15594029	121	125			
5864	19	52241008	52243739	ENSMUSG00000019158	ENSMUSG00000019158	ENSMUSG00000019158	7	15611301	15614012	121	125			
5865	19	52214941	52240873	ENSMUSG00000001988	ENSMUSG00000001988	ENSMUSG00000001988	7	15614243	15634210	121	125			
5866	19	52113773	52196472	ENSMUSG00000058230	ENSMUSG00000058230	ENSMUSG00000058230	7	15653561	15773515	121	125			
5867	19	51969982	51983062	ENSMUSG00000001918	ENSMUSG00000001918	ENSMUSG00000001918	7	15939891	15956764	121	125			
5868	19	51943121	51953581	ENSMUSG00000048920	ENSMUSG00000048920	ENSMUSG00000048920	7	15967789	15973651	121	125			
5869	19	51914610	51941527	ENSMUSG00000030374	ENSMUSG00000030374	ENSMUSG00000030374	7	15974411	15999425	121	125			
5870	19	51869417	51911597	ENSMUSG00000041187	ENSMUSG00000041187	ENSMUSG00000041187	7	16001456	16028881	121	125			
5871	19	51829173	51829782	ENSMUSG00000063594	ENSMUSG00000063594	ENSMUSG00000063594	7	16050308	16053957	121	125			
5872	19	51815566	51820194	ENSMUSG00000043017	ENSMUSG00000043017	ENSMUSG00000043017	7	16065301	16069427	121	125			
5873	19	51686293	51690562	ENSMUSG00000070802	ENSMUSG00000070802	ENSMUSG00000070802	7	16103615	16104760	121	125			
5874	19	51661602	51666132	ENSMUSG00000041141	ENSMUSG00000041141	ENSMUSG00000041141	7	16118317	16120845	121	125			
5875	19	51606291	51607907	ENSMUSG00000041117	ENSMUSG00000041117	ENSMUSG00000041117	7	16153110	16155167	121	125			
5876	19	51542134	51585943	ENSMUSG00000003099	ENSMUSG00000003099	ENSMUSG00000003099	7	16163174	16186407	121	125			
5877	19	51492172	51535006	ENSMUSG00000004328	ENSMUSG00000004328	ENSMUSG00000004328	7	16194256	16215201	121	125			
5878	19	51214255	51218144	ENSMUSG00000030413	ENSMUSG00000030413	ENSMUSG00000030413	7	18043202	18048960	121	125			
5879	19	51198108	51213485	ENSMUSG00000074358	ENSMUSG00000074358	ENSMUSG00000074358	7	18049406	18068926	121	125			
5880	19	51134631	51168497	ENSMUSG00000030411	ENSMUSG00000030411	ENSMUSG00000030411	7	18083871	18117322	121	125			
5881	19	51109375	51109791	ENSMUSG00000051965	ENSMUSG00000051965	ENSMUSG00000051965	7	18146046	18147484	121	125			
5882	19	51085125	51097702	ENSMUSG00000048481	ENSMUSG00000048481	ENSMUSG00000048481	7	18149841	18160288	121	125			
5883	19	51079118	51080872	ENSMUSG00000044030	ENSMUSG00000044030	ENSMUSG00000044030	7	18162575	18165296	121	125			
5884	19	51059358	51068894	ENSMUSG00000040891	ENSMUSG00000040891	ENSMUSG00000040891	7	18171805	18182061	121	125			
5885	19	50990809	51010417	ENSMUSG00000040866	ENSMUSG00000040866	ENSMUSG00000040866	7	18213216	18232969	121	125			
5886	19	50979324	50987866	ENSMUSG00000030410	ENSMUSG00000030410	ENSMUSG00000030410	7	18234837	18241297	121	125			
5887	19	50965579	50975268	ENSMUSG00000030409	ENSMUSG00000030409	ENSMUSG00000030409	7	18242463	18252340	121	125			
5888	19	50959884	50964152	ENSMUSG00000040841	ENSMUSG00000040841	ENSMUSG00000040841	7	18253066	18256867	121	125			
5889	19	50887772	50898425	ENSMUSG00000030407	ENSMUSG00000030407	ENSMUSG00000030407	7	18298739	18307718	121	125			
5890	19	50863342	50877544	ENSMUSG00000030406	ENSMUSG00000030406	ENSMUSG00000030406	7	18321065	18323144	121	125			
5891	19	50804500	50834509	ENSMUSG00000040811	ENSMUSG00000040811	ENSMUSG00000040811	7	18339726	18365001	121	125			
5892	19	50784875	50797294	ENSMUSG00000044317	ENSMUSG00000044317	ENSMUSG00000044317	7	18371060	18382698	121	125			
5893	19	50722525	50779940	ENSMUSG00000052214	ENSMUSG00000052214	ENSMUSG00000052214	7	18386875	18405158	121	125			
5894	19	50702528	50722076	ENSMUSG00000030403	ENSMUSG00000030403	ENSMUSG00000030403	7	18416239	18423349	121	125			
5895	19	50693705	50697406	ENSMUSG00000030402	ENSMUSG00000030402	ENSMUSG00000030402	7	18435328	18438571	121	125			
5896	19	50680390	50692151	ENSMUSG00000030401	ENSMUSG00000030401	ENSMUSG00000030401	7	18441189	18454682	121	125			
5897	19	50663093	50670254	ENSMUSG00000003545	ENSMUSG00000003545	ENSMUSG00000003545	7	18463363	18467953	121	125			
5898	19	50608533	50618642	ENSMUSG00000003549	ENSMUSG00000003549	ENSMUSG00000003549	7	18503622	18515046	121	125			
5899	19	50601307	50605864	ENSMUSG00000047649	ENSMUSG00000047649	ENSMUSG00000047649	7	18515371	18517941	121	125			
5900	19	50574741	50601410	ENSMUSG00000040734	ENSMUSG00000040734	ENSMUSG00000040734	7	18519768	18536530	121	125			
5901	19	50546686	50565669	ENSMUSG00000030400	ENSMUSG00000030400	ENSMUSG00000030400	7	18540561	18554214	121	125			
5902	19	50540498	50546618	ENSMUSG00000040714	ENSMUSG00000040714	ENSMUSG00000040714	7	18552965	18558400	121	125			
5903	19	50501512	50517974	ENSMUSG00000030399	ENSMUSG00000030399	ENSMUSG00000030399	7	18569616	18580105	121	125			
5904	19	50446682	50500381	ENSMUSG00000030397	ENSMUSG00000030397	ENSMUSG00000030397	7	18584597	18617016	121	125			
5905	19	50407719	50429309	ENSMUSG00000011263	ENSMUSG00000011263	ENSMUSG00000011263	7	18647578	18655282	121	125			
5906	19	50374395	50375003	ENSMUSG00000057667	ENSMUSG00000057667	ENSMUSG00000057667	7	18664326	18666853	121	125			
5907	19	50358027	50373325	ENSMUSG00000002043	ENSMUSG00000002043	ENSMUSG00000002043	7	18667247	18674666	121	125			
5908	19	50347036	50348868	ENSMUSG00000060621	ENSMUSG00000060621	ENSMUSG00000060621	7	18682200	18683572	121	125			
5909	19	50333614	50342377	ENSMUSG00000051403	ENSMUSG00000051403	ENSMUSG00000051403	7	18689323	18720768	121	125			
5910	19	50274358	50286622	ENSMUSG00000044709	ENSMUSG00000044709	ENSMUSG00000044709	7	18723471	18731865	121	125			
5911	19	50266599	50271528	ENSMUSG00000011267	ENSMUSG00000011267	ENSMUSG00000011267	7	18735844	18739177	121	125			
5912	19	50234138	50266054	ENSMUSG00000061028	ENSMUSG00000061028	ENSMUSG00000061028	7	18739561	18762981	121	125			
5913	19	50196539	50233292	ENSMUSG00000002983	ENSMUSG00000002983	ENSMUSG00000002983	7	18764744	18787960	121	125			
5914	19	50150477	50188257	ENSMUSG00000002981	ENSMUSG00000002981	ENSMUSG00000002981	7	18791765	18823534	121	125			
5915	19	50137335	50144660	ENSMUSG00000074336	ENSMUSG00000074336	ENSMUSG00000074336	7	18836616	18839938	121	125			
5916	19	50109419	50114423	ENSMUSG00000040564	ENSMUSG00000040564	ENSMUSG00000040564	7	18848006	18851169	121	125			
5917	19	50100879	50104489	ENSMUSG00000002985	ENSMUSG00000002985	ENSMUSG00000002985	7	18854795	18857574	121	125			
5918	19	50086328	50098266	ENSMUSG00000002984	ENSMUSG00000002984	ENSMUSG00000002984	7	18859887	18873939	121	125			
5919	19	50041390	50083476	ENSMUSG00000062300	ENSMUSG00000062300	ENSMUSG00000062300	7	18875186	18908047	121	125			
5920	19	50004178	50016517	ENSMUSG00000002980	ENSMUSG00000002980	ENSMUSG00000002980	7	18914905	18929017	121	125			
5921	19	49972966	49995736	ENSMUSG00000040525	ENSMUSG00000040525	ENSMUSG00000040525	7	18939488	18955304	121	125			
5922	19	49943820	49955138	ENSMUSG00000053175	ENSMUSG00000053175	ENSMUSG00000053175	7	18966984	18981237	121	125			
5923	19	49867044	49878602	ENSMUSG00000049848	ENSMUSG00000049848	ENSMUSG00000049848	7	19035585	19046473	121	125			
5924	19	49839066	49858689	ENSMUSG00000040511	ENSMUSG00000040511	ENSMUSG00000040511	7	19062101	19079622	121	125			
5925	19	49706916	49725651	ENSMUSG00000070777	ENSMUSG00000070777	ENSMUSG00000070777	7	19123934	19149631	121	125			
5926	19	61206892	61264706	ENSMUSG00000015721	ENSMUSG00000015721	ENSMUSG00000015721	7	23094657	23150681	out-of-place		here	Presence of a singleton made AutoGRAPH to break the block	
5927	19	49671701	49696395	ENSMUSG00000057101	ENSMUSG00000057101	ENSMUSG00000057101	7	23790684	23816458	121	126	Presence of singletons	1 -> 2	
5928	19	49522546	49537172	ENSMUSG00000052675	ENSMUSG00000052675	ENSMUSG00000052675	7	23821074	23836712	121	126			
5929	19	48992235	48998439	ENSMUSG00000030484	ENSMUSG00000030484	ENSMUSG00000030484	7	24057982	24063009	121	126			
5930	19	48962578	48976944	ENSMUSG00000054342	ENSMUSG00000054342	ENSMUSG00000054342	7	24079023	24093969	121	126			
5931	19	48927588	48946239	ENSMUSG00000002210	ENSMUSG00000002210	ENSMUSG00000002210	7	24108388	24131537	121	126			
5932	19	48844572	48866342	ENSMUSG00000046223	ENSMUSG00000046223	ENSMUSG00000046223	7	24171260	24184727	121	126			
5933	19	48818362	48835831	ENSMUSG00000054793	ENSMUSG00000054793	ENSMUSG00000054793	7	24190859	24213187	121	126			
5934	19	48803217	48815846	ENSMUSG00000064264	ENSMUSG00000064264	ENSMUSG00000064264	7	24215941	24224424	121	126			
5935	19	48787866	48791516	ENSMUSG00000041037	ENSMUSG00000041037	ENSMUSG00000041037	7	24239449	24247360	121	126			
5936	19	48776594	48778085	ENSMUSG00000011632	ENSMUSG00000011632	ENSMUSG00000011632	7	24250418	24254765	121	126			
5937	19	48739032	48771998	ENSMUSG00000051768	ENSMUSG00000051768	ENSMUSG00000051768	7	24255049	24282200	121	126			
5938	19	48729169	48732122	ENSMUSG00000066721	ENSMUSG00000066721	ENSMUSG00000066721	7	24292598	24296401	121	126			
5939	19	48702712	48723236	ENSMUSG00000064254	ENSMUSG00000064254	ENSMUSG00000064254	7	24296303	24317685	121	126			
5940	19	48692026	48700927	ENSMUSG00000061511	ENSMUSG00000061511	ENSMUSG00000061511	7	24320105	24321743	121	126			
5941	19	48671106	48682642	ENSMUSG00000074277	ENSMUSG00000074277	ENSMUSG00000074277	7	24325592	24337739	121	126			
5942	19	48656779	48661648	ENSMUSG00000057454	ENSMUSG00000057454	ENSMUSG00000057454	7	24345293	24349876	121	126			
5943	19	48602214	48614588	ENSMUSG00000062773	ENSMUSG00000062773	ENSMUSG00000062773	7	24376767	24380810	121	126			
5944	19	47032990	47040348	ENSMUSG00000062732	ENSMUSG00000062732	ENSMUSG00000062732	7	24573380	24578451	121	127	here	This inversion was ignored by our tool	
5945	19	47040864	47047604	ENSMUSG00000011349	ENSMUSG00000011349	ENSMUSG00000011349	7	24578817	24586409	121	127	Inversion ignored by ST	0 -> 2	
5946	19	47073030	47077275	ENSMUSG00000003379	ENSMUSG00000003379	ENSMUSG00000003379	7	24606271	24610957	121	127			
5947	19	47083952	47103437	ENSMUSG00000040940	ENSMUSG00000040940	ENSMUSG00000040940	7	24616494	24634891	121	127			
5948	19	47105358	47106090	ENSMUSG00000042789	ENSMUSG00000042789	ENSMUSG00000042789	7	24637553	24638129	121	127			
5949	19	47152675	47155325	ENSMUSG00000003380	ENSMUSG00000003380	ENSMUSG00000003380	7	24678508	24681415	121	127			
5950	19	47162578	47190222	ENSMUSG00000040907	ENSMUSG00000040907	ENSMUSG00000040907	7	24686927	24714655	121	127			
5951	19	47198107	47261797	ENSMUSG00000003378	ENSMUSG00000003378	ENSMUSG00000003378	7	24718613	24781017	121	127			
5952	19	47264469	47277543	ENSMUSG00000045252	ENSMUSG00000045252	ENSMUSG00000045252	7	24786025	24791240	121	127			
5953	19	47287136	47328419	ENSMUSG00000008496	ENSMUSG00000008496	ENSMUSG00000008496	7	24801890	24829982	121	127			
5954	19	47394605	47416115	ENSMUSG00000054499	ENSMUSG00000054499	ENSMUSG00000054499	7	24911600	24928619	121	127			
5955	19	47416332	47424193	ENSMUSG00000046541	ENSMUSG00000046541	ENSMUSG00000046541	7	24930211	24936267	121	127			
5956	19	47426186	47438591	ENSMUSG00000057177	ENSMUSG00000057177	ENSMUSG00000057177	7	24937019	24946611	121	127			
5957	19	47443561	47451113	ENSMUSG00000040857	ENSMUSG00000040857	ENSMUSG00000040857	7	24951314	24959425	121	127			
5958	19	47480657	47491789	ENSMUSG00000005442	ENSMUSG00000005442	ENSMUSG00000005442	7	24979606	25002906	121	127			
5959	19	47493025	47498563	ENSMUSG00000005447	ENSMUSG00000005447	ENSMUSG00000005447	7	25003809	25006715	121	127			
5960	19	47509369	47521036	ENSMUSG00000043843	ENSMUSG00000043843	ENSMUSG00000043843	7	25014868	25024955	121	127			
5961	19	47532798	47573470	ENSMUSG00000045039	ENSMUSG00000045039	ENSMUSG00000045039	7	25039579	25074670	121	127			
5962	19	47583013	47586279	ENSMUSG00000063651	ENSMUSG00000063651	ENSMUSG00000063651	7	25076382	25078484	121	127			
5963	19	47597499	47623418	ENSMUSG00000003123	ENSMUSG00000003123	ENSMUSG00000003123	7	25088287	25104747	121	127			
5964	19	47624280	47639040	ENSMUSG00000060188	ENSMUSG00000060188	ENSMUSG00000060188	7	25108813	25121646	121	127			
5965	19	46640903	46641969	ENSMUSG00000074261	ENSMUSG00000074261	ENSMUSG00000074261	7	25323381	25324652	121	128			
5966	19	46629093	46637714	ENSMUSG00000057229	ENSMUSG00000057229	ENSMUSG00000057229	7	25328194	25334310	121	128			
5967	19	46623105	46626475	ENSMUSG00000059479	ENSMUSG00000059479	ENSMUSG00000059479	7	25336384	25338252	121	128			
5968	19	46574502	46622746	ENSMUSG00000060376	ENSMUSG00000060376	ENSMUSG00000060376	7	25338707	25367507	121	128			
5969	19	46584121	46595224	ENSMUSG00000061286	ENSMUSG00000061286	ENSMUSG00000061286	7	25367924	25376783	121	128			
5970	19	46552167	46561877	ENSMUSG00000063439	ENSMUSG00000063439	ENSMUSG00000063439	7	25389918	25395322	121	128			
5971	19	46528254	46551628	ENSMUSG00000002603	ENSMUSG00000002603	ENSMUSG00000002603	7	25395762	25413756	121	128			
5972	19	46507934	46522625	ENSMUSG00000002608	ENSMUSG00000002608	ENSMUSG00000002608	7	25419877	25427813	121	128			
5973	19	46462076	46505438	ENSMUSG00000040725	ENSMUSG00000040725	ENSMUSG00000040725	7	25430244	25463450	121	128			
5974	19	46416663	46459510	ENSMUSG00000002602	ENSMUSG00000002602	ENSMUSG00000002602	7	25466034	25497330	121	128			
5975	19	46390955	46404515	ENSMUSG00000040703	ENSMUSG00000040703	ENSMUSG00000040703	7	25511235	25525307	121	128			
5976	19	46312193	46326121	ENSMUSG00000052974	ENSMUSG00000052974	ENSMUSG00000052974	7	26828735	26842420	121	128			
5977	19	45996932	46006176	ENSMUSG00000058709	ENSMUSG00000058709	ENSMUSG00000058709	7	26867419	26875533	121	128			
5978	19	45976790	45994680	ENSMUSG00000053291	ENSMUSG00000053291	ENSMUSG00000053291	7	26877193	26887643	121	128			
5979	19	45914848	45938603	ENSMUSG00000003752	ENSMUSG00000003752	ENSMUSG00000003752	7	26915930	26937357	121	128			
5980	19	45889274	45914440	ENSMUSG00000003762	ENSMUSG00000003762	ENSMUSG00000003762	7	26941783	26966705	121	128			
5981	19	45864440	45888374	ENSMUSG00000063160	ENSMUSG00000063160	ENSMUSG00000063160	7	26967521	26990910	121	128			
5982	19	45790912	45827562	ENSMUSG00000040488	ENSMUSG00000040488	ENSMUSG00000040488	7	27013902	27046452	121	128			
5983	19	45774598	45789141	ENSMUSG00000003759	ENSMUSG00000003759	ENSMUSG00000003759	7	27050897	27064779	121	128			
5984	19	45663988	45774205	ENSMUSG00000011751	ENSMUSG00000011751	ENSMUSG00000011751	7	27065143	27155349	121	128			
5985	19	45645541	45663435	ENSMUSG00000040466	ENSMUSG00000040466	ENSMUSG00000040466	7	27156878	27174741	121	128			
5986	19	45638591	45642122	ENSMUSG00000055200	ENSMUSG00000055200	ENSMUSG00000055200	7	27182600	27186124	121	128			
5987	19	45619339	45623772	ENSMUSG00000008384	ENSMUSG00000008384	ENSMUSG00000008384	7	27195670	27199067	121	128			
5988	19	45591516	45611113	ENSMUSG00000053198	ENSMUSG00000053198	ENSMUSG00000053198	7	27208084	27228594	121	128			
5989	19	45577019	45587934	ENSMUSG00000040424	ENSMUSG00000040424	ENSMUSG00000040424	7	27235819	27239932	121	128			
5990	19	45546425	45576222	ENSMUSG00000003363	ENSMUSG00000003363	ENSMUSG00000003363	7	27240761	27261869	121	128			
5991	19	45517302	45546274	ENSMUSG00000049643	ENSMUSG00000049643	ENSMUSG00000049643	7	27265250	27290859	121	128			
5992	19	45413805	45416146	ENSMUSG00000007944	ENSMUSG00000007944	ENSMUSG00000007944	7	27362683	27364968	121	128			
5993	19	45389491	45413314	ENSMUSG00000040390	ENSMUSG00000040390	ENSMUSG00000040390	7	27365137	27382380	121	128			
5994	19	45168913	45179191	ENSMUSG00000030603	ENSMUSG00000030603	ENSMUSG00000030603	7	27750473	27758829	121	128			
5995	19	45045811	45132373	ENSMUSG00000047730	ENSMUSG00000047730	ENSMUSG00000047730	7	27782042	27826092	121	128			
5996	19	45007833	45016681	ENSMUSG00000002409	ENSMUSG00000002409	ENSMUSG00000002409	7	27889076	27895703	121	128			
5997	19	44721849	44722559	ENSMUSG00000046058	ENSMUSG00000046058	ENSMUSG00000046058	7	27976641	27977932	121	128			
5998	19	44714797	44715282	ENSMUSG00000070705	ENSMUSG00000070705	ENSMUSG00000070705	7	27986499	27988249	121	128			
5999	19	44697593	44703166	ENSMUSG00000046750	ENSMUSG00000046750	ENSMUSG00000046750	7	27993413	27999946	121	128			
6000	19	44681427	44690948	ENSMUSG00000003436	ENSMUSG00000003436	ENSMUSG00000003436	7	28000414	28010544	121	128			
6001	19	44662892	44673368	ENSMUSG00000003438	ENSMUSG00000003438	ENSMUSG00000003438	7	28014277	28020805	121	128			
6002	19	44628052	44659150	ENSMUSG00000003435	ENSMUSG00000003435	ENSMUSG00000003435	7	28023658	28047494	121	128			
6003	19	44595590	44607994	ENSMUSG00000037552	ENSMUSG00000037552	ENSMUSG00000037552	7	28068364	28081337	121	128			
6004	19	44589293	44591853	ENSMUSG00000044786	ENSMUSG00000044786	ENSMUSG00000044786	7	28085544	28087988	121	128			
6005	19	44573803	44583041	ENSMUSG00000003444	ENSMUSG00000003444	ENSMUSG00000003444	7	28094904	28101433	121	128			
6006	19	44568115	44573519	ENSMUSG00000003437	ENSMUSG00000003437	ENSMUSG00000003437	7	28101775	28108283	121	128			
6007	19	44524979	44566007	ENSMUSG00000037513	ENSMUSG00000037513	ENSMUSG00000037513	7	28109022	28132598	121	128			
6008	19	44386333	44386764	ENSMUSG00000047314	ENSMUSG00000047314	ENSMUSG00000047314	7	28249638	28250965	121	128			
6009	19	44379441	44384362	ENSMUSG00000047586	ENSMUSG00000047586	ENSMUSG00000047586	7	28252362	28255664	121	128			
6010	19	44308260	44361886	ENSMUSG00000030602	ENSMUSG00000030602	ENSMUSG00000030602	7	28267579	28277068	121	128			
6011	19	44266785	44293318	ENSMUSG00000037469	ENSMUSG00000037469	ENSMUSG00000037469	7	28316397	28339775	121	128			
6012	19	44206533	44215076	ENSMUSG00000037463	ENSMUSG00000037463	ENSMUSG00000037463	7	28401609	28408094	121	128			
6013	19	44123882	44158220	ENSMUSG00000030598	ENSMUSG00000030598	ENSMUSG00000030598	7	28441260	28446242	121	128			
6014	19	44113028	44115493	ENSMUSG00000045948	ENSMUSG00000045948	ENSMUSG00000045948	7	28448401	28450550	121	128			
6015	19	44097779	44113238	ENSMUSG00000070699	ENSMUSG00000070699	ENSMUSG00000070699	7	28450750	28462609	121	128			
6016	19	44082455	44091371	ENSMUSG00000030595	ENSMUSG00000030595	ENSMUSG00000030595	7	28467011	28475284	121	128			
6017	19	44061061	44082304	ENSMUSG00000015149	ENSMUSG00000015149	ENSMUSG00000015149	7	28475527	28497418	121	128			
6018	19	44050314	44060734	ENSMUSG00000051735	ENSMUSG00000051735	ENSMUSG00000051735	7	28497737	28507723	121	128			
6019	19	44018869	44032457	ENSMUSG00000015165	ENSMUSG00000015165	ENSMUSG00000015165	7	28519673	28531009	121	128			
6020	19	43997908	44014337	ENSMUSG00000053898	ENSMUSG00000053898	ENSMUSG00000053898	7	28534037	28541006	121	128			
6021	19	43984155	43995422	ENSMUSG00000053964	ENSMUSG00000053964	ENSMUSG00000053964	7	28542750	28550463	121	128			
6022	19	43912672	43926954	ENSMUSG00000054083	ENSMUSG00000054083	ENSMUSG00000054083	7	28590416	28602345	121	128			
6023	19	43830167	43913010	ENSMUSG00000054808	ENSMUSG00000054808	ENSMUSG00000054808	7	28602011	28671040	121	128			
6024	19	43771653	43800471	ENSMUSG00000037337	ENSMUSG00000037337	ENSMUSG00000037337	7	28691621	28712036	121	128			
6025	19	43616180	43770012	ENSMUSG00000030592	ENSMUSG00000030592	ENSMUSG00000030592	7	28712104	28833911	121	128			
6026	19	43591538	43608785	ENSMUSG00000030589	ENSMUSG00000030589	ENSMUSG00000030589	7	28843747	28862713	121	128			
6027	19	43572783	43578625	ENSMUSG00000037239	ENSMUSG00000037239	ENSMUSG00000037239	7	28867589	28877407	121	128			
6028	19	43566833	43569741	ENSMUSG00000031493	ENSMUSG00000031493	ENSMUSG00000031493	7	28879957	28882736	121	128			
6029	19	43557061	43566303	ENSMUSG00000030591	ENSMUSG00000030591	ENSMUSG00000030591	7	28883015	28889291	121	128			
6030	19	43502322	43511480	ENSMUSG00000046410	ENSMUSG00000046410	ENSMUSG00000046410	7	28930688	28941253	121	128			
6031	19	43487601	43498420	ENSMUSG00000030588	ENSMUSG00000030588	ENSMUSG00000030588	7	28947113	28956289	121	128			
6032	19	43486641	43487489	ENSMUSG00000030587	ENSMUSG00000030587	ENSMUSG00000030587	7	28955321	28957152	121	128			
6033	19	43447007	43474927	ENSMUSG00000074227	ENSMUSG00000074227	ENSMUSG00000074227	7	28965097	28990672	121	128			
6034	19	43433717	43439071	ENSMUSG00000037166	ENSMUSG00000037166	ENSMUSG00000037166	7	28998072	29002151	121	128			
6035	19	43393523	43412194	ENSMUSG00000030584	ENSMUSG00000030584	ENSMUSG00000030584	7	29012765	29026346	121	128			
6036	19	43089708	43390849	ENSMUSG00000030583	ENSMUSG00000030583	ENSMUSG00000030583	7	29030907	29109602	121	128			
6037	19	42815229	42838153	ENSMUSG00000047473	ENSMUSG00000047473	ENSMUSG00000047473	7	29493549	29503300	121	128			
6038	19	42261222	42312489	ENSMUSG00000058402	ENSMUSG00000058402	ENSMUSG00000058402	7	29583252	29624386	121	128			
6039	19	42593902	42650179	ENSMUSG00000059975	ENSMUSG00000059975	ENSMUSG00000059975	7	29641559	29646840	121	128			
6040	19	41519002	41561918	ENSMUSG00000053985	ENSMUSG00000053985	ENSMUSG00000053985	7	29746813	29752431	121	128			
6041	19	41574701	41601390	ENSMUSG00000058447	ENSMUSG00000058447	ENSMUSG00000058447	7	29764913	29775136	121	128			
6042	19	41696742	41697980	ENSMUSG00000049421	ENSMUSG00000049421	ENSMUSG00000049421	7	29803836	29816374	121	128			

6043	19	41788061	41811338	ENSMUSG00000074220	ENSMUSG00000074220	ENSMUSG00000074220	7	29830794	29843419	121	128			
6044	19	41397875	41421506	ENSMUSG00000037029	ENSMUSG00000037029	ENSMUSG00000037029	7	29870034	29878487	121	128			
6045	19	41333664	41335611	ENSMUSG00000074218	ENSMUSG00000074218	ENSMUSG00000074218	7	29892931	29894790	121	128			
6046	19	41322757	41333095	ENSMUSG00000001794	ENSMUSG00000001794	ENSMUSG00000001794	7	29895703	29903808	121	128			
6047	19	41297728	41308689	ENSMUSG00000006095	ENSMUSG00000006095	ENSMUSG00000006095	7	29932898	29940546	121	128			
6048	19	41296452	41298088	ENSMUSG00000019738	ENSMUSG00000019738	ENSMUSG00000019738	7	29940834	29942146	121	128			
6049	19	41237660	41287845	ENSMUSG00000037020	ENSMUSG00000037020	ENSMUSG00000037020	7	29948900	29989156	121	128			
6050	19	41198474	41215099	ENSMUSG00000013921	ENSMUSG00000013921	ENSMUSG00000013921	7	30010603	30015617	121	128			
6051	19	41191883	41196981	ENSMUSG00000042831	ENSMUSG00000042831	ENSMUSG00000042831	7	30017513	30023063	121	128			
6052	19	41186050	41191509	ENSMUSG00000019737	ENSMUSG00000019737	ENSMUSG00000019737	7	30023589	30027806	121	128			
6053	19	41177941	41179053	ENSMUSG00000074210	ENSMUSG00000074210	ENSMUSG00000074210	7	30030733	30031089	121	128			
6054	19	41119590	41127924	ENSMUSG00000036957	ENSMUSG00000036957	ENSMUSG00000036957	7	30064244	30071532	121	128			
6055	19	41087152	41091026	ENSMUSG00000030579	ENSMUSG00000030579	ENSMUSG00000030579	7	30122548	30126345	121	128			
6056	19	41085222	41087015	ENSMUSG00000064109	ENSMUSG00000064109	ENSMUSG00000064109	7	30126473	30128613	121	128			
6057	19	41070983	41085025	ENSMUSG00000036931	ENSMUSG00000036931	ENSMUSG00000036931	7	30132064	30137508	121	128			
6058	19	41051241	41062539	ENSMUSG00000006651	ENSMUSG00000006651	ENSMUSG00000006651	7	30143740	30154321	121	128			
6059	19	41038602	41049875	ENSMUSG00000036915	ENSMUSG00000036915	ENSMUSG00000036915	7	30156526	30166275	121	128			
6060	19	41008706	41034579	ENSMUSG00000006649	ENSMUSG00000006649	ENSMUSG00000006649	7	30168485	30195968	121	128			
6061	19	40958316	40971564	ENSMUSG00000036882	ENSMUSG00000036882	ENSMUSG00000036882	7	30230986	30242940	121	128			
6062	19	40940925	41019682	ENSMUSG00000036864	ENSMUSG00000036864	ENSMUSG00000036864	7	30247896	30258467	121	128			
6063	19	40937336	40939799	ENSMUSG00000036854	ENSMUSG00000036854	ENSMUSG00000036854	7	30262062	30264203	121	128			
6064	19	40921993	40925191	ENSMUSG00000036826	ENSMUSG00000036826	ENSMUSG00000036826	7	30274840	30276719	121	128			
6065	19	40900761	40921619	ENSMUSG00000006307	ENSMUSG00000006307	ENSMUSG00000006307	7	30277619	30297477	121	128			
6066	19	40895670	40899780	ENSMUSG00000006310	ENSMUSG00000006310	ENSMUSG00000006310	7	30298441	30300663	121	128			
6067	19	40849555	40861207	ENSMUSG00000006313	ENSMUSG00000006313	ENSMUSG00000006313	7	30311852	30321494	121	128			
6068	19	40830995	40841523	ENSMUSG00000036751	ENSMUSG00000036751	ENSMUSG00000036751	7	30325734	30334911	121	128			
6069	19	40824499	40827613	ENSMUSG00000006311	ENSMUSG00000006311	ENSMUSG00000006311	7	30342378	30344907	121	128			
6070	19	40795513	40808091	ENSMUSG00000036733	ENSMUSG00000036733	ENSMUSG00000036733	7	30362474	30373733	121	128			
6071	19	40732936	40746400	ENSMUSG00000005553	ENSMUSG00000005553	ENSMUSG00000005553	7	30420990	30434293	121	128			
6072	19	40728376	40730268	ENSMUSG00000006315	ENSMUSG00000006315	ENSMUSG00000006315	7	30436461	30438283	121	128			
6073	19	40716204	40728058	ENSMUSG00000061099	ENSMUSG00000061099	ENSMUSG00000061099	7	30438540	30452441	121	128			
6074	19	40679963	40696394	ENSMUSG00000060962	ENSMUSG00000060962	ENSMUSG00000060962	7	30472684	30488640	121	128			
6075	19	40670068	40673196	ENSMUSG00000074199	ENSMUSG00000074199	ENSMUSG00000074199	7	30496665	30499843	121	128			
6076	19	40632457	40633449	ENSMUSG00000051314	ENSMUSG00000051314	ENSMUSG00000051314	7	30527117	30532535	121	128			
6077	19	40534295	40535197	ENSMUSG00000044453	ENSMUSG00000044453	ENSMUSG00000044453	7	30569328	30570230	121	128			
6078	19	40511944	40530098	ENSMUSG00000030577	ENSMUSG00000030577	ENSMUSG00000030577	7	30574589	30589029	121	128			
6079	19	40474904	40496547	ENSMUSG00000036634	ENSMUSG00000036634	ENSMUSG00000036634	7	30607943	30623592	121	128			
6080	19	40451736	40462558	ENSMUSG00000058239	ENSMUSG00000058239	ENSMUSG00000058239	7	30654756	30665563	121	128			
6081	19	40431399	40450703	ENSMUSG00000001247	ENSMUSG00000001247	ENSMUSG00000001247	7	30666539	30682077	121	128			
6082	19	40407544	40411468	ENSMUSG00000046826	ENSMUSG00000046826	ENSMUSG00000046826	7	30685803	30698225	121	128			
6083	19	40337467	40352625	ENSMUSG00000009687	ENSMUSG00000009687	ENSMUSG00000009687	7	30741490	30750226	121	128			
6084	19	40325994	40337045	ENSMUSG00000036578	ENSMUSG00000036578	ENSMUSG00000036578	7	30751275	30760227	121	128			
6085	19	40321572	40325793	ENSMUSG00000036570	ENSMUSG00000036570	ENSMUSG00000036570	7	30760440	30764415	121	128			
6086	19	40307257	40317944	ENSMUSG00000036560	ENSMUSG00000036560	ENSMUSG00000036560	7	30768695	30779695	121	128			
6087	19	40298623	40307067	ENSMUSG00000057092	ENSMUSG00000057092	ENSMUSG00000057092	7	30779297	30785438	121	128			
6088	19	40223250	40249315	ENSMUSG00000001249	ENSMUSG00000001249	ENSMUSG00000001249	7	30807485	30819662	121	128			
6089	19	40213374	40223192	ENSMUSG00000019194	ENSMUSG00000019194	ENSMUSG00000019194	7	30825284	30835705	121	128			
6090	19	40191858	40209213	ENSMUSG00000001248	ENSMUSG00000001248	ENSMUSG00000001248	7	30839252	30851663	121	128			
6091	19	39665173	39683914	ENSMUSG00000036459	ENSMUSG00000036459	ENSMUSG00000036459	7	33818307	33842028	121	128			
6092	19	39611108	39652635	ENSMUSG00000052997	ENSMUSG00000052997	ENSMUSG00000052997	7	33849448	33877315	121	128			
6093	19	39587143	39608910	ENSMUSG00000002635	ENSMUSG00000002635	ENSMUSG00000002635	7	33893257	33905407	121	128			
6094	19	39437317	39538307	ENSMUSG00000066571	ENSMUSG00000066571	ENSMUSG00000066571	7	33945474	33994369	121	128			
6095	19	39355283	39410838	ENSMUSG00000066568	ENSMUSG00000066568	ENSMUSG00000066568	7	34053466	34098300	121	128			
6096	19	38979607	38996984	ENSMUSG00000030499	ENSMUSG00000030499	ENSMUSG00000030499	7	34347779	34364269	121	128			
6097	19	38804717	38956248	ENSMUSG00000060402	ENSMUSG00000060402	ENSMUSG00000060402	7	34383919	34456951	121	128			
6098	19	38569705	38704540	ENSMUSG00000063931	ENSMUSG00000063931	ENSMUSG00000063931	7	34621153	34753468	121	128			
6099	19	38556449	38565431	ENSMUSG00000056216	ENSMUSG00000056216	ENSMUSG00000056216	7	34755537	34765263	121	128			
6100	19	38391410	38408596	ENSMUSG00000030495	ENSMUSG00000030495	ENSMUSG00000030495	7	34895178	34909870	121	128			
6101	19	38377364	38390383	ENSMUSG00000001802	ENSMUSG00000001802	ENSMUSG00000001802	7	34909047	34923688	121	128			
6102	19	38263626	38313158	ENSMUSG00000063808	ENSMUSG00000063808	ENSMUSG00000063808	7	34985304	35027200	121	128			
6103	19	38161339	38247634	ENSMUSG00000030494	ENSMUSG00000030494	ENSMUSG00000030494	7	35043074	35101041	121	128			
6104	19	38154996	38160968	ENSMUSG00000030493	ENSMUSG00000030493	ENSMUSG00000030493	7	35100912	35105527	121	128			
6105	19	38061908	38154620	ENSMUSG00000023072	ENSMUSG00000023072	ENSMUSG00000023072	7	35105795	35147444	121	128			
6106	19	38013262	38052512	ENSMUSG00000030492	ENSMUSG00000030492	ENSMUSG00000030492	7	35157939	35174763	121	128			
6107	19	37902519	37998370	ENSMUSG00000030491	ENSMUSG00000030491	ENSMUSG00000030491	7	35202370	35246442	121	128			
6108	19	37858153	37861046	ENSMUSG00000056043	ENSMUSG00000056043	ENSMUSG00000056043	7	35293267	35293980	121	128			
6109	19	37779754	37857945	ENSMUSG00000034867	ENSMUSG00000034867	ENSMUSG00000034867	7	35295018	35347053	121	128			
6110	19	37763945	37770046	ENSMUSG00000030417	ENSMUSG00000030417	ENSMUSG00000030417	7	35350747	35356131	121	128			
6111	19	37588520	37668635	ENSMUSG00000043671	ENSMUSG00000043671	ENSMUSG00000043671	7	35397572	35463158	121	128			
6112	19	37528393	37570411	ENSMUSG00000044452	ENSMUSG00000044452	ENSMUSG00000044452	7	35481112	35511717	121	128			
6113	19	36458840	36531984	ENSMUSG00000021217	ENSMUSG00000021217	ENSMUSG00000021217	7	36477381	36480593	121	128			
6114	19	35555168	35740805	ENSMUSG00000043456	ENSMUSG00000043456	ENSMUSG00000043456	7	37187882	37479740	121	128			
6115	19	35125265	35198451	ENSMUSG00000030421	ENSMUSG00000030421	ENSMUSG00000030421	7	37668762	37728302	121	128			
6116	19	34994741	35007056	ENSMUSG00000002068	ENSMUSG00000002068	ENSMUSG00000002068	7	37806746	37816294	121	128			
6117	19	34789041	34798546	ENSMUSG00000030423	ENSMUSG00000030423	ENSMUSG00000030423	7	37972261	37978046	121	128			
6118	19	34709331	34713045	ENSMUSG00000039257	ENSMUSG00000039257	ENSMUSG00000039257	7	40672431	40703129	121	128			
6119	19	39275285	39276055	ENSMUSG00000044609	ENSMUSG00000044609	ENSMUSG00000044609	7	42726750	42727541	out-of-place				
6120	19	56574976	56583009	ENSMUSG00000004613	ENSMUSG00000004613	ENSMUSG00000004613	7	43298008	43303911	122	129			
6121	19	56566688	56567781	ENSMUSG00000004612	ENSMUSG00000004612	ENSMUSG00000004612	7	43304993	43306166	122	129			
6122	19	56562164	56564069	ENSMUSG00000038973	ENSMUSG00000038973	ENSMUSG00000038973	7	43308702	43311248	122	129			
6123	19	56540243	56552178	ENSMUSG00000004610	ENSMUSG00000004610	ENSMUSG00000004610	7	43312028	43325704	122	129			
6124	19	56526884	56535219	ENSMUSG00000070604	ENSMUSG00000070604	ENSMUSG00000070604	7	43333287	43338792	122	129			
6125	19	56517096	56525401	ENSMUSG00000013367	ENSMUSG00000013367	ENSMUSG00000013367	7	43340827	43357910	122	129			
6126	19	56362397	56368508	ENSMUSG00000004609	ENSMUSG00000004609	ENSMUSG00000004609	7	43396416	43401556	122	129			
6127	19	56293588	56299659	ENSMUSG00000038888	ENSMUSG00000038888	ENSMUSG00000038888	7	43540073	43546342	122	129			
6128	19	56272386	56279314	ENSMUSG00000044737	ENSMUSG00000044737	ENSMUSG00000044737	7	43558461	43563090	122	129			
6129	19	56251275	56260179	ENSMUSG00000054046	ENSMUSG00000054046	ENSMUSG00000054046	7	43580609	43767378	122	129			
6130	19	56224223	56229960	ENSMUSG00000044430	ENSMUSG00000044430	ENSMUSG00000044430	7	43637693	43641620	122	129			
6131	19	56217301	56222697	ENSMUSG00000067616	ENSMUSG00000067616	ENSMUSG00000067616	7	43642659	43647300	122	129			
6132	19	56209338	56215094	ENSMUSG00000030693	ENSMUSG00000030693	ENSMUSG00000030693	7	43649109	43653451	122	129			
6133	19	56191082	56197779	ENSMUSG00000064023	ENSMUSG00000064023	ENSMUSG00000064023	7	43665619	43671864	122	129			
6134	19	56171541	56178962	ENSMUSG00000030713	ENSMUSG00000030713	ENSMUSG00000030713	7	43679486	43684401	122	129			
6135	19	56153700	56164741	ENSMUSG00000050063	ENSMUSG00000050063	ENSMUSG00000050063	7	43692600	43699828	122	129			
6136	19	56138373	56148156	ENSMUSG00000074155	ENSMUSG00000074155	ENSMUSG00000074155	7	43710311	43719223	122	129			
6137	19	56101420	56105806	ENSMUSG00000006948	ENSMUSG00000006948	ENSMUSG00000006948	7	43749202	43753846	122	129			
6138	19	55985484	55990293	ENSMUSG00000012777	ENSMUSG00000012777	ENSMUSG00000012777	7	44121372	44125060	122	129			
6139	19	55918456	55920536	ENSMUSG00000004473	ENSMUSG00000004473	ENSMUSG00000004473	7	44172299	44174944	122	129			
6140	19	55856896	55912007	ENSMUSG00000038738	ENSMUSG00000038738	ENSMUSG00000038738	7	44180749	44219402	122	129			
6141	19	55712643	55743913	ENSMUSG00000047085	ENSMUSG00000047085	ENSMUSG00000047085	7	44310529	44331393	122	129			
6142	19	55706802	55708877	ENSMUSG00000038704	ENSMUSG00000038704	ENSMUSG00000038704	7	44333477	44335793	122	129			
6143	19	55701067	55706247	ENSMUSG00000038695	ENSMUSG00000038695	ENSMUSG00000038695	7	44336098	44339693	122	129			
6144	19	55670405	55678172	ENSMUSG00000008140	ENSMUSG00000008140	ENSMUSG00000008140	7	44358169	44364450	122	129			
6145	19	55628004	55662502	ENSMUSG00000038670	ENSMUSG00000038670	ENSMUSG00000038670	7	44371671	44390951	122	129			
6146	19	55614028	55624058	ENSMUSG00000008193	ENSMUSG00000008193	ENSMUSG00000008193	7	44394037	44400113	122	129			
6147	19	55579408	55613082	ENSMUSG00000038644	ENSMUSG00000038644	ENSMUSG00000038644	7	44400831	44411478	122	129			
6148	19	55571515	55578030	ENSMUSG00000060601	ENSMUSG00000060601	ENSMUSG00000060601	7	44417659	44421965	122	129			
6149	19	55510577	55524446	ENSMUSG00000062785	ENSMUSG00000062785	ENSMUSG00000062785	7	44458928	44470047	122	129			
6150	19	55398697	55505610	ENSMUSG00000030739	ENSMUSG00000030739	ENSMUSG00000030739	7	44473872	44538885	122	129			
6151	19	55347618	55358350	ENSMUSG00000066500	ENSMUSG00000066500	ENSMUSG00000066500	7	44576814	44587876	122	129			
6152	19	55171538	55220617	ENSMUSG00000002205	ENSMUSG00000002205	ENSMUSG00000002205	7	44616671	44645557	122	129			
6153	19	55124272	55129003	ENSMUSG00000038539	ENSMUSG00000038539	ENSMUSG00000038539	7	44680299	44684641	122	129			
6154	19	55084728	55124574	ENSMUSG00000074141	ENSMUSG00000074141	ENSMUSG00000074141	7	44704331	44708851	122	129			
6155	19	55072641	55083812	ENSMUSG00000038520	ENSMUSG00000038520	ENSMUSG00000038520	7	44709271	44716576	122	129			
6156	19	55064113	55068364	ENSMUSG00000011096	ENSMUSG00000011096	ENSMUSG00000011096	7	44717269	44723452	122	129			
6157	19	55056274	55062630	ENSMUSG00000002963	ENSMUSG00000002963	ENSMUSG00000002963	7	44725386	44730967	122	129			
6158	19	55046260	55055802	ENSMUSG00000038502	ENSMUSG00000038502	ENSMUSG00000038502	7	44731110	44737830	122	129			
6159	19	55013358	55033802	ENSMUSG00000002968	ENSMUSG00000002968	ENSMUSG00000002968	7	44747433	44760451	122	129			
6160	19	55002220	55008353	ENSMUSG00000011658	ENSMUSG00000011658	ENSMUSG00000011658	7	44764135	44768665	122	129			
6161	19	54961992	55002181	ENSMUSG00000060279	ENSMUSG00000060279	ENSMUSG00000060279	7	44768423	44797511	122	129			
6162	19	54934830	54958355	ENSMUSG00000059891	ENSMUSG00000059891	ENSMUSG00000059891	7	44811303	44826077	122	129			
6163	19	54886219	54908800	ENSMUSG00000007783	ENSMUSG00000007783	ENSMUSG00000007783	7	44827415	44842856	122	129			
6164	19	54874941	54883516	ENSMUSG00000052429	ENSMUSG00000052429	ENSMUSG00000052429	7	44844804	44854398	122	129			
6165	19	54860211	54868984	ENSMUSG00000003190	ENSMUSG00000003190	ENSMUSG00000003190	7	44859267	44866754	122	129			
6166	19	54854642	54860926	ENSMUSG00000003184	ENSMUSG00000003184	ENSMUSG00000003184	7	44865702	44870890	122	129			
6167	19	54837194	54853710	ENSMUSG00000038406	ENSMUSG00000038406	ENSMUSG00000038406	7	44870993	44884309	122	129			
6168	19	54830364	54835212	ENSMUSG00000038387	ENSMUSG00000038387	ENSMUSG00000038387	7	44886032	44889683	122	129			
6169	19	54778289	54786072	ENSMUSG00000007837	ENSMUSG00000007837	ENSMUSG00000007837	7	44921655	44930464	122	129			
6170	19	54750782	54775626	ENSMUSG00000003421	ENSMUSG00000003421	ENSMUSG00000003421	7	44930546	44945870	122	129			
6171	19	54722687	54738701	ENSMUSG00000019539	ENSMUSG00000019539	ENSMUSG00000019539	7	44950955	44960196	122	129			
6172	19	54708437	54721400	ENSMUSG00000003420	ENSMUSG00000003420	ENSMUSG00000003420	7	44961106	44971787	122	129			
6173	19	54669298	54681299	ENSMUSG00000074129	ENSMUSG00000074129	ENSMUSG00000074129	7	45000255	45004092	122	129			
6174	19	54648325	54665657	ENSMUSG00000007833	ENSMUSG00000007833	ENSMUSG00000007833	7	45009882	45022580	122	129			
6175	19	54641371	54646899	ENSMUSG00000003423	ENSMUSG00000003423	ENSMUSG00000003423	7	45022396	45028106	122	129			
6176	19	54624470	54636596	ENSMUSG00000070570	ENSMUSG00000070570	ENSMUSG00000070570	7	45032065	45043086	122	129			
6177	19	54617483	54618510	ENSMUSG00000038300	ENSMUSG00000038300	ENSMUSG00000038300	7	45049224	45049880	122	129			
6178	19	54583318	54612579	ENSMUSG00000038292	ENSMUSG00000038292	ENSMUSG00000038292	7	45051718	45072934	122	129			
6179	19	54558854	54570183	ENSMUSG00000030792	ENSMUSG00000030792	ENSMUSG00000030792	7	45075567	45079893	122	129			
6180	19	54535669	54557476	ENSMUSG00000030796	ENSMUSG00000030796	ENSMUSG00000030796	7	45083795	45101661	122	129			
6181	19	54530240	54535671	ENSMUSG00000030798	ENSMUSG00000030798	ENSMUSG00000030798	7	45101674	45107061	122	129			
6182	19	54484709	54506416	ENSMUSG00000045228	ENSMUSG00000045228	ENSMUSG00000045228	7	45127224	45140686	122	129			
6183	19	54352864	54406903	ENSMUSG00000038260	ENSMUSG00000038260	ENSMUSG00000038260	7	45172649	45201752	122	129			
6184	19	54346270	54350493	ENSMUSG00000038239	ENSMUSG00000038239	ENSMUSG00000038239	7	45203332	45207012	122	129			
6185	19	54314475	54346090	ENSMUSG00000003863	ENSMUSG00000003863	ENSMUSG00000003863	7	45207315	45234968	122	129			
6186	19	54309430	54313529	ENSMUSG00000003872	ENSMUSG00000003872	ENSMUSG00000003872	7	45235934	45238625	122	129			
6187	19	54280277	54303681	ENSMUSG00000063511	ENSMUSG00000063511	ENSMUSG00000063511	7	45244502	45263735	122	129			
6188	19	54262487	54268010	ENSMUSG00000038201	ENSMUSG00000038201	ENSMUSG00000038201	7	45274002	45279249	122	129			
6189	19	54256216	54258936	ENSMUSG00000074121	ENSMUSG00000074121	ENSMUSG00000074121	7	45281737	45285221	122	129			
6190	19	54007256	54210952	ENSMUSG00000003868	ENSMUSG00000003868	ENSMUSG00000003868	7	45289939	45306138	122			here	
6191	19	54163195	54188361	ENSMUSG00000003865	ENSMUSG00000003865	ENSMUSG00000003865	7	45303088	45324144	122	130		Single marker moved to different place with in a HSB	1 -> 2
6192	19	54149929	54156331	ENSMUSG00000003873	ENSMUSG00000003873	ENSMUSG00000003873	7	45329742	45334871	122	130			
6193	19	54128751	54140030	ENSMUSG00000011382	ENSMUSG00000011382	ENSMUSG00000011382	7	45341605	45356838	122	130			
6194	19	54095412	54118009	ENSMUSG00000030824	ENSMUSG00000030824	ENSMUSG00000030824	7	45360918	45378450	122	130			
6195	19	54076041	54093802	ENSMUSG00000023467	ENSMUSG00000023467	ENSMUSG00000023467	7	45381795	45390429	122	130			
6196	19	54067490	54071126	ENSMUSG00000040435	ENSMUSG00000040435	ENSMUSG00000040435	7	45390962	45394255	122	130			
6197	19	54032167	54114458	ENSMUSG00000040428	ENSMUSG00000040428	ENSMUSG00000040428	7	45394372	45417610	122	130			
6198	19	54008091	54031746	ENSMUSG00000030825	ENSMUSG00000030825	ENSMUSG00000030825	7	45424086	45435360	122	130			
6199	19	53990134	54006113	ENSMUSG00000030826	ENSMUSG00000030826	ENSMUSG00000030826	7	45438405	45457754	122	130			
6200	19	53950628	53953395	ENSMUSG00000030827	ENSMUSG00000030827	ENSMUSG00000030827	7	45481928	45483532	122	130			
6201	19	53943080	53948612	ENSMUSG00000008461	ENSMUSG00000008461	ENSMUSG00000008461	7	45485648	45489101	122	130			
6202	19	53935959	53941978	ENSMUSG00000064158	ENSMUSG00000064158	ENSMUSG00000064158	7	45489853	45495284	122	130			
6203	19	53915655	53935782	ENSMUSG00000044562	ENSMUSG00000044562	ENSMUSG00000044562	7	45495579	45507134	122	130			
6204	19	53908067	53914790	ENSMUSG00000042918	ENSMUSG00000042918	ENSMUSG00000042918	7	45508019	45513810	122	130			
6205	19	53856478	53868076	ENSMUSG00000070564	ENSMUSG00000070564	ENSMUSG00000070564	7	45552507	45562596	122	130			
6206	19	53833084	53841263	ENSMUSG00000003273	ENSMUSG00000003273	ENSMUSG00000003273	7	45568009	45572713	122	130			
6207	19	53825659	53832435	ENSMUSG00000059824	ENSMUSG00000059824	ENSMUSG00000059824	7	45573274	45578193	122	130			
6208	19	53814575	53825473	ENSMUSG00000057342	ENSMUSG00000057342	ENSMUSG00000057342	7	45578327	45583249	122	130			
6209	19	53795879	53808631	ENSMUSG00000054161	ENSMUSG00000054161	ENSMUSG00000054161	7	45590415	45597533	122	130			
6210	19	53801812	53802782	ENSMUSG00000070563	ENSMUSG00000070563	ENSMUSG00000070563	7	45593235	45593618	122	130			
6211	19	53747241	53794495	ENSMUSG00000003271	ENSMUSG00000003271	ENSMUSG00000003271	7	45598025	45627564	122	130			
6212	19	53680342	53708231	ENSMUSG00000062044	ENSMUSG00000062044	ENSMUSG00000062044	7	45653648	45669105	122	130			
6213	19	53664424	53674457	ENSMUSG00000003269	ENSMUSG00000003269	ENSMUSG00000003269	7	45674679	45682328	122	130			
6214	19	53650578	53661179	ENSMUSG00000058743	ENSMUSG00000058743	ENSMUSG00000058743	7	45684503	45692798	122	130			
6215	19	53589944	53639205	ENSMUSG00000002771	ENSMUSG00000002771	ENSMUSG00000002771	7	45700822	45734609	122	130			
6216	19	53577639	53586622	ENSMUSG00000002778	ENSMUSG00000002778	ENSMUSG00000002778	7	45740814	45751766	122	130			
6217	19	53559469	53571439	ENSMUSG00000040231	ENSMUSG00000040231	ENSMUSG00000040231	7	45754892	45764753	122	130			
6218	19	53527425	53559009	ENSMUSG00000002781	ENSMUSG00000002781	ENSMUSG00000002781	7	45765117	45785455	122	130			
6219	19	53520441	53525623	ENSMUSG00000040212	ENSMUSG00000040212	ENSMUSG00000040212	7	45786106	45789446	122	130			
6220	19	53491534	53515656	ENSMUSG00000040189	ENSMUSG00000040189	ENSMUSG00000040189	7	45796440	45816998	122	130			
6221	11	17365042	17366214	ENSMUSG00000070561	ENSMUSG00000070561	ENSMUSG00000070561	7	45966767	45967939	123	131			
6222	11	17371009	17455025	ENSMUSG00000040136	ENSMUSG00000040136	ENSMUSG00000040136	7	45972565	46048075	123	131			
6223	11	17472018	17522539	ENSMUSG00000030838	ENSMUSG00000030838	ENSMUSG00000030838	7	46063399	46106532	123	131			
6224	11	17556387	17617057	ENSMUSG00000009487	ENSMUSG00000009487	ENSMUSG00000009487	7	46109029	46179476	123	131			
6225	11	17697686	17700251	ENSMUSG00000009471	ENSMUSG00000009471	ENSMUSG00000009471	7	46244516	46247134	123	131			
6226	11	17712735	17750753	ENSMUSG00000058975	ENSMUSG00000058975	ENSMUSG00000058975	7	46264970	46296353	123	131			
6227	11	17766175	17991265	ENSMUSG00000030839	ENSMUSG00000030839	ENSMUSG00000030839	7	46311201	46507828	123	131			
6228	11	17997772	18018885	ENSMUSG00000040046	ENSMUSG00000040046	ENSMUSG00000040046	7	46515203	46540579	123	131			
6229	11	18058496	18084210	ENSMUSG00000006763	ENSMUSG00000006763	ENSMUSG00000006763	7	46555855	46578698	123	131			
6230	11	18209479	18214910	ENSMUSG00000040017	ENSMUSG00000040017	ENSMUSG00000040017	7	46596038	46600586	123	131			
6231	11	18300719	18345152	ENSMUSG00000006599	ENSMUSG00000006599	ENSMUSG00000006599	7	46664145	46691839	123	131			
6232	11	18390429	18429366	ENSMUSG00000030851	ENSMUSG00000030851	ENSMUSG00000030851	7	46729305	46746184	123	131			
6233	11	18458438	18505065	ENSMUSG00000014402	ENSMUSG00000014402	ENSMUSG00000014402	7	46757396	46787915	123	131			
6234	11	18509821	18566828	ENSMUSG00000043262	ENSMUSG00000043262	ENSMUSG00000043262	7	46791564	46826549	123	131			
6235	11	18584527	18612596	ENSMUSG00000049516	ENSMUSG00000049516	ENSMUSG00000049516	7	46858718	46876231	123	131			
6236	11	18676927	18682908	ENSMUSG00000010307	ENSMUSG00000010307	ENSMUSG00000010307	7	46920872	46921874	123	131			
6237	11	18706053	18769965	ENSMUSG00000030854	ENSMUSG00000030854	ENSMUSG00000030854	7	46945824	47000700	123	131			

6238	11	19095268	19154542	ENSMUSG00000030471	ENSMUSG00000030471	ENSMUSG00000030471	7	48657024	48695458	123	131		
6239	11	19160154	19180177	ENSMUSG00000030470	ENSMUSG00000030470	ENSMUSG00000030470	7	48698421	48716054	123	131		
6240	11	19202194	19219083	ENSMUSG00000046179	ENSMUSG00000046179	ENSMUSG00000046179	7	48735068	48746736	123	131		
6241	11	20134336	20138446	ENSMUSG00000030507	ENSMUSG00000030507	ENSMUSG00000030507	7	49499520	49504856	123	131		
6242	11	20341924	20361904	ENSMUSG00000039745	ENSMUSG00000039745	ENSMUSG00000039745	7	49627174	49642016	123	131		
6243	11	20365682	20487404	ENSMUSG00000030505	ENSMUSG00000030505	ENSMUSG00000030505	7	49646367	49726283	123	131		
6244	11	20577520	20633041	ENSMUSG00000039728	ENSMUSG00000039728	ENSMUSG00000039728	7	49778167	49831877	123	131		
6245	11	20647693	21553803	ENSMUSG00000055409	ENSMUSG00000055409	ENSMUSG00000055409	7	49843371	50731308	123	131		
6246	11	22171298	22261476	ENSMUSG00000055489	ENSMUSG00000055489	ENSMUSG00000055489	7	51379048	51466728	123	131		
6247	11	22316243	22357619	ENSMUSG00000030500	ENSMUSG00000030500	ENSMUSG00000030500	7	51490086	51539138	123	131		
6248	11	22602808	22603932	ENSMUSG00000043480	ENSMUSG00000043480	ENSMUSG00000043480	7	51729257	51730273	123	131		
6249	11	22646230	22791113	ENSMUSG00000030498	ENSMUSG00000030498	ENSMUSG00000030498	7	51746986	51862465	123	131		
6250	11	24475366	25056780	ENSMUSG00000063297	ENSMUSG00000063297	ENSMUSG00000063297	7	54703978	55133385	123	131		
6251	15	20384869	20425331	ENSMUSG00000033790	ENSMUSG00000033790	ENSMUSG00000033790	7	55662173	55699698	124	132		
6252	15	20444104	20555043	ENSMUSG00000030447	ENSMUSG00000030447	ENSMUSG00000030447	7	55710301	55798717	124	132		
6253	15	20556790	20585849	ENSMUSG00000030452	ENSMUSG00000030452	ENSMUSG00000030452	7	55800935	55830487	124	132		
6254	15	20594720	20637877	ENSMUSG00000047037	ENSMUSG00000047037	ENSMUSG00000047037	7	55846486	55887606	124	132		
6255	15	25673628	26018061	ENSMUSG00000030450	ENSMUSG00000030450	ENSMUSG00000030450	7	56107790	56404536	125	133		
6256	15	24799429	25451622	ENSMUSG00000055026	ENSMUSG00000055026	ENSMUSG00000055026	7	56592432	57254792	125	133		
6257	15	24663151	24776749	ENSMUSG00000055078	ENSMUSG00000055078	ENSMUSG00000055078	7	57275688	57377873	125	133		
6258	15	24339793	24767432	ENSMUSG00000033676	ENSMUSG00000033676	ENSMUSG00000033676	7	57458398	57696666	125	133		
6259	15	23473513	23659963	ENSMUSG00000025324	ENSMUSG00000025324	ENSMUSG00000025324	7	58526285	58697289	125	133		
6260	15	23133489	23235221	ENSMUSG00000025326	ENSMUSG00000025326	ENSMUSG00000025326	7	59096621	59174596	125	133		
6261	15	22771598	22774684	ENSMUSG00000000948	ENSMUSG00000000948	ENSMUSG00000000948	7	59862190	59866513	125	133		
6262	15	21482492	21483457	ENSMUSG00000033585	ENSMUSG00000033585	ENSMUSG00000033585	7	62227270	62228875	125	133		
6263	15	21361547	21364653	ENSMUSG00000070527	ENSMUSG00000070527	ENSMUSG00000070527	7	62297357	62298991	125	133		
6264	15	29562621	29734834	ENSMUSG00000033510	ENSMUSG00000033510	ENSMUSG00000033510	7	63323722	63637977	126	134		
6265	15	29406375	29457393	ENSMUSG00000052040	ENSMUSG00000052040	ENSMUSG00000052040	7	63770327	63817865	126	134		
6266	15	29080845	29181216	ENSMUSG00000030523	ENSMUSG00000030523	ENSMUSG00000030523	7	64080837	64148080	126	134		
6267	15	29025706	29070910	ENSMUSG00000030522	ENSMUSG00000030522	ENSMUSG00000030522	7	64176518	64218248	126	134		
6268	15	28983399	29022600	ENSMUSG00000033458	ENSMUSG00000033458	ENSMUSG00000033458	7	64240442	64252323	126	134		
6269	2	71210952	71230738	ENSMUSG00000030521	ENSMUSG00000030521	ENSMUSG00000030521	7	64255491	64271186	out-of-place	135	here	out of place markers were called HSBs by AG
6270	2	71190322	71210875	ENSMUSG00000033429	ENSMUSG00000033429	ENSMUSG00000033429	7	64271721	64291071	out-of-place	135	out of place markers were called HSBs by AG	0 -> 1
6271	15	27001141	27197806	ENSMUSG00000030519	ENSMUSG00000030519	ENSMUSG00000030519	7	64380656	64632828	127	136		
6272	15	27199749	27276047	ENSMUSG00000030518	ENSMUSG00000030518	ENSMUSG00000030518	7	64635063	64735178	127	136		
6273	15	27348287	27349201	ENSMUSG00000070520	ENSMUSG00000070520	ENSMUSG00000070520	7	64751029	64751868	127	136		
6274	15	27779656	27902010	ENSMUSG00000030516	ENSMUSG00000030516	ENSMUSG00000030516	7	65175115	65250189	127	136		
6275	15	100011479	100082168	ENSMUSG00000030515	ENSMUSG00000030515	ENSMUSG00000030515	7	65523864	65571041	128	137		
6276	15	99661657	99847396	ENSMUSG00000030513	ENSMUSG00000030513	ENSMUSG00000030513	7	65741163	65929284	128	137		
6277	15	99628737	99635223	ENSMUSG00000075701	ENSMUSG00000075701	ENSMUSG00000075701	7	65958664	65967802	128	137		
6278	15	99533456	99609660	ENSMUSG00000032640	ENSMUSG00000032640	ENSMUSG00000032640	7	65988465	66052738	128	137		
6279	15	99237580	99274349	ENSMUSG00000015134	ENSMUSG00000015134	ENSMUSG00000015134	7	66269840	66306401	128	137		
6280	15	98960358	99007174	ENSMUSG00000030509	ENSMUSG00000030509	ENSMUSG00000030509	7	66523534	66567834	128	137		
6281	15	98926957	98959902	ENSMUSG00000053091	ENSMUSG00000053091	ENSMUSG00000053091	7	66568908	66593694	128	137		
6282	15	98758124	98902448	ENSMUSG00000030510	ENSMUSG00000030510	ENSMUSG00000030510	7	66643126	66700792	128	137		
6283	15	98331993	98699650	ENSMUSG00000074076	ENSMUSG00000074076	ENSMUSG00000074076	7	66926468	67028971	128	137		
6284	15	98085137	98091149	ENSMUSG00000043831	ENSMUSG00000043831	ENSMUSG00000043831	7	67101558	67107406	128	137		
6285	15	97923738	98071374	ENSMUSG00000030557	ENSMUSG00000030557	ENSMUSG00000030557	7	67113818	67174528	128	137		
6286	15	97609175	97744019	ENSMUSG00000030556	ENSMUSG00000030556	ENSMUSG00000030556	7	67386924	67518744	128	137		
6287	15	97494052	97599290	ENSMUSG00000030555	ENSMUSG00000030555	ENSMUSG00000030555	7	67520954	67600087	128	137		
6288	15	97462809	97490786	ENSMUSG00000030554	ENSMUSG00000030554	ENSMUSG00000030554	7	67606141	67633141	128	137		
6289	15	97010302	97319034	ENSMUSG00000005533	ENSMUSG00000005533	ENSMUSG00000005533	7	67826372	68100293	128	137		
6290	15	97328982	97368547	ENSMUSG00000030553	ENSMUSG00000030553	ENSMUSG00000030553	7	68110123	68137746	128	137		
6291	15	96800383	96841532	ENSMUSG00000074071	ENSMUSG00000074071	ENSMUSG00000074071	7	68174341	68236602	128	137		
6292	15	96304947	96318062	ENSMUSG00000042659	ENSMUSG00000042659	ENSMUSG00000042659	7	68612993	68622585	128	137		
6293	15	94674950	94683045	ENSMUSG00000030551	ENSMUSG00000030551	ENSMUSG00000030551	7	70226952	70234106	128	137		
6294	15	92575955	92824636	ENSMUSG00000032776	ENSMUSG00000032776	ENSMUSG00000032776	7	71951343	72180108	128	137		
6295	15	91387651	91417381	ENSMUSG00000070509	ENSMUSG00000070509	ENSMUSG00000070509	7	73249033	73293411	128	137		
6296	15	91244423	91366396	ENSMUSG00000025788	ENSMUSG00000025788	ENSMUSG00000025788	7	73325212	73414346	128	137		
6297	15	90738144	90812962	ENSMUSG00000025789	ENSMUSG00000025789	ENSMUSG00000025789	7	73812635	73887187	128	137		
6298	15	90197950	90507783	ENSMUSG00000025790	ENSMUSG00000025790	ENSMUSG00000025790	7	74155429	74428293	128	137		
6299	15	89570104	89639514	ENSMUSG00000053025	ENSMUSG00000053025	ENSMUSG00000053025	7	74989842	75181825	128	137		
6300	15	83724875	84093590	ENSMUSG00000066406	ENSMUSG00000066406	ENSMUSG00000066406	7	75435164	75610217	129	138		
6301	15	84103561	84139175	ENSMUSG00000055652	ENSMUSG00000055652	ENSMUSG00000055652	7	75721882	75741004	129	138		
6302	15	84601109	84867192	ENSMUSG00000025754	ENSMUSG00000025754	ENSMUSG00000025754	7	76284575	76573636	129	138		
6303	15	86221026	86600665	ENSMUSG00000059146	ENSMUSG00000059146	ENSMUSG00000059146	7	78066072	78451296	129	138		
6304	15	86803713	86811623	ENSMUSG00000030612	ENSMUSG00000030612	ENSMUSG00000030612	7	78648749	78656579	129	138		
6305	15	86811944	86823219	ENSMUSG00000030611	ENSMUSG00000030611	ENSMUSG00000030611	7	78656823	78666501	129	138		
6306	15	86855794	86875952	ENSMUSG00000030610	ENSMUSG00000030610	ENSMUSG00000030610	7	78700987	78720724	129	138		
6307	15	86970445	86975411	ENSMUSG00000030609	ENSMUSG00000030609	ENSMUSG00000030609	7	78769440	78782346	129	138		
6308	15	86983198	86999851	ENSMUSG00000039236	ENSMUSG00000039236	ENSMUSG00000039236	7	78786971	78793820	129	138		
6309	15	87147709	87218716	ENSMUSG00000030607	ENSMUSG00000030607	ENSMUSG00000030607	7	78926996	78988014	129	138		
6310	15	87221538	87239832	ENSMUSG00000030606	ENSMUSG00000030606	ENSMUSG00000030606	7	78990540	79004531	129	138		
6311	15	87242949	87257646	ENSMUSG00000030605	ENSMUSG00000030605	ENSMUSG00000030605	7	79007282	79022526	129	138		
6312	15	87432628	87541199	ENSMUSG00000039202	ENSMUSG00000039202	ENSMUSG00000039202	7	79146779	79235114	129	138		
6313	15	87554105	87565926	ENSMUSG00000039194	ENSMUSG00000039194	ENSMUSG00000039194	7	79249073	79257265	129	138		
6314	15	87588198	87661366	ENSMUSG00000039187	ENSMUSG00000039187	ENSMUSG00000039187	7	79272647	79309206	129	138		
6315	15	87660577	87679030	ENSMUSG00000039176	ENSMUSG00000039176	ENSMUSG00000039176	7	79322896	79339786	129	138		
6316	15	87815646	87840818	ENSMUSG00000030549	ENSMUSG00000030549	ENSMUSG00000030549	7	79466882	79491170	129	138		
6317	15	87919822	87972254	ENSMUSG00000046591	ENSMUSG00000046591	ENSMUSG00000046591	7	79533709	79571661	129	138		
6318	15	87972211	87992578	ENSMUSG00000050382	ENSMUSG00000050382	ENSMUSG00000050382	7	79571612	79582174	129	138		
6319	15	88008607	88023595	ENSMUSG00000030546	ENSMUSG00000030546	ENSMUSG00000030546	7	79594677	79605684	129	138		
6320	15	88027318	88035012	ENSMUSG00000030545	ENSMUSG00000030545	ENSMUSG00000030545	7	79610777	79616538	129	138		
6321	15	88035032	88087870	ENSMUSG00000039099	ENSMUSG00000039099	ENSMUSG00000039099	7	79616676	79659463	129	138		
6322	15	88094111	88095547	ENSMUSG00000030544	ENSMUSG00000030544	ENSMUSG00000030544	7	79665755	79667301	129	138		
6323	15	88120593	88121516	ENSMUSG00000030543	ENSMUSG00000030543	ENSMUSG00000030543	7	79684304	79686952	129	138		
6324	15	88129131	88159072	ENSMUSG00000039062	ENSMUSG00000039062	ENSMUSG00000039062	7	79695323	79715831	129	138		
6325	15	88345756	88425029	ENSMUSG00000048897	ENSMUSG00000048897	ENSMUSG00000048897	7	79899847	79966258	129	138		
6326	15	88428220	88446712	ENSMUSG00000030541	ENSMUSG00000030541	ENSMUSG00000030541	7	79968363	79988851	129	138		
6327	15	88529156	88572879	ENSMUSG00000030539	ENSMUSG00000030539	ENSMUSG00000030539	7	80060360	80099907	129	138		
6328	15	88574481	88578168	ENSMUSG00000030538	ENSMUSG00000030538	ENSMUSG00000030538	7	80100673	80106129	129	138		
6329	15	88585145	88586302	ENSMUSG00000050973	ENSMUSG00000050973	ENSMUSG00000050973	7	80106421	80114518	129	138		
6330	15	88597484	88608308	ENSMUSG00000045467	ENSMUSG00000045467	ENSMUSG00000045467	7	80119889	80134328	129	138		
6331	15	89342909	89366817	ENSMUSG00000030534	ENSMUSG00000030534	ENSMUSG00000030534	7	80143192	80165086	129	139	here	This inversion was ignored by our tool
6332	15	89310279	89338808	ENSMUSG00000038943	ENSMUSG00000038943	ENSMUSG00000038943	7	80168019	80189771	129	139	Inversion ignored by ST	1 -> 2
6333	15	89299110	89307357	ENSMUSG00000038930	ENSMUSG00000038930	ENSMUSG00000038930	7	80190147	80197937	129	139		
6334	15	89279536	89298326	ENSMUSG00000030533	ENSMUSG00000030533	ENSMUSG00000030533	7	80198805	80213721	129	139		
6335	15	89275161	89276780	ENSMUSG00000030532	ENSMUSG00000030532	ENSMUSG00000030532	7	80216637	80219610	129	139		
6336	15	89248442	89265435	ENSMUSG00000038886	ENSMUSG00000038886	ENSMUSG00000038886	7	80225215	80243237	129	139		
6337	15	89228713	89240010	ENSMUSG00000053158	ENSMUSG00000053158	ENSMUSG00000053158	7	80251277	80261384	129	139		
6338	15	89212889	89227691	ENSMUSG00000030530	ENSMUSG00000030530	ENSMUSG00000030530	7	80262709	80276275	129	139		
6339	15	89061639	89159688	ENSMUSG00000030528	ENSMUSG00000030528	ENSMUSG00000030528	7	80328605	80408610	129	139		
6340	15	88874304	88986729	ENSMUSG00000030527	ENSMUSG00000030527	ENSMUSG00000030527	7	80461672	80562225	129	139		
6341	15	88732477	88846479	ENSMUSG00000030536	ENSMUSG00000030536	ENSMUSG00000030536	7	80586294	80676807	129	139		
6342	15	82948163	82967951	ENSMUSG00000038797	ENSMUSG00000038797	ENSMUSG00000038797	7	80735621	80750029	130	140	here	Blocks 140 and 141 defined by AG do not have orientation, and so are merged with the next block by ST
6343	15	82987676	82998515	ENSMUSG00000025722	ENSMUSG00000025722	ENSMUSG00000025722	7	80764778	80774751	130	140	Blocks without orientation merged with the neighboring block	1 -> 3
6344	15	82999368	83002806	ENSMUSG00000025723	ENSMUSG00000025723	ENSMUSG00000025723	7	80775749	80778549	130	140		
6345	15	83126889	83146719	ENSMUSG00000005621	ENSMUSG00000005621	ENSMUSG00000005621	7	80882945	80916231	130	140		
6346	15	83160915	83217714	ENSMUSG00000038763	ENSMUSG00000038763	ENSMUSG00000038763	7	80931113	80979125	130	140		
6347	15	83228913	83290030	ENSMUSG00000025726	ENSMUSG00000025726	ENSMUSG00000025726	7	80988312	81043929	130	140		
6348	15	83324675	83483371	ENSMUSG00000025584	ENSMUSG00000025584	ENSMUSG00000025584	7	81087251	81208046	130	140		
6349	15	81125089	81175689	ENSMUSG00000062444	ENSMUSG00000062444	ENSMUSG00000062444	7	81333912	81367219	130	141		
6350	15	81221173	81271875	ENSMUSG00000038663	ENSMUSG00000038663	ENSMUSG00000038663	7	81406821	81440457	130	141		
6351	15	81275434	81300665	ENSMUSG00000045795	ENSMUSG00000045795	ENSMUSG00000045795	7	81444863	81470349	130	141		
6352	15	81314792	81352599	ENSMUSG00000025813	ENSMUSG00000025813	ENSMUSG00000025813	7	81483766	81523225	130	141		
6353	15	81465054	81471282	ENSMUSG00000025102	ENSMUSG00000025102	ENSMUSG00000025102	7	81655507	81662897	130	141		
6354	15	81567392	81596902	ENSMUSG00000038623	ENSMUSG00000038623	ENSMUSG00000038623	7	81732514	81757584	130	141		
6355	15	81597934	81667774	ENSMUSG00000025104	ENSMUSG00000025104	ENSMUSG00000025104	7	81765487	81807893	130	141		
6356	15	81715659	81744472	ENSMUSG00000025105	ENSMUSG00000025105	ENSMUSG00000025105	7	81841856	81852039	130	141		
6357	15	81907287	82078495	ENSMUSG00000030638	ENSMUSG00000030638	ENSMUSG00000030638	7	82048309	82183594	130	141		
6358	15	80342195	80364317	ENSMUSG00000038570	ENSMUSG00000038570	ENSMUSG00000038570	7	82509145	82524708	130			
6359	15	82540911	82561920	ENSMUSG00000038563	ENSMUSG00000038563	ENSMUSG00000038563	7	82524826	82654037	out-of-place			
6360	15	80121183	80125416	ENSMUSG00000057706	ENSMUSG00000057706	ENSMUSG00000057706	7	82743533	82746394	130	142		
6361	15	79417038	79453552	ENSMUSG00000038540	ENSMUSG00000038540	ENSMUSG00000038540	7	83461116	83501790	130	142		
6362	15	79392069	79403579	ENSMUSG00000046027	ENSMUSG00000046027	ENSMUSG00000046027	7	83508185	83518503	130	142		
6363	15	79262256	79388491	ENSMUSG00000001741	ENSMUSG00000001741	ENSMUSG00000001741	7	83519249	83611708	130	142		
6364	15	79213699	79228570	ENSMUSG00000011154	ENSMUSG00000011154	ENSMUSG00000011154	7	83650287	83671004	130	142		
6365	15	79081028	79083400	ENSMUSG00000070462	ENSMUSG00000070462	ENSMUSG00000070462	7	83758318	83759406	130	142		
6366	15	79026725	79069224	ENSMUSG00000038503	ENSMUSG00000038503	ENSMUSG00000038503	7	83768442	83777717	130	142		
6367	15	78858767	79031054	ENSMUSG00000052353	ENSMUSG00000052353	ENSMUSG00000052353	7	83810968	83962687	130	142		
6368	15	78483747	78677324	ENSMUSG00000015709	ENSMUSG00000015709	ENSMUSG00000015709	7	84122466	84247087	130	142		
6369	15	78232191	78265740	ENSMUSG00000030630	ENSMUSG00000030630	ENSMUSG00000030630	7	84461344	84481937	130	142		
6370	15	78139076	78217767	ENSMUSG00000030629	ENSMUSG00000030629	ENSMUSG00000030629	7	84491239	84555575	130	142		
6371	1	245902043	245902966	ENSMUSG00000054054	ENSMUSG00000054054	ENSMUSG00000054054	7	86181946	86182872	out-of-place	143	here	out of place markers were called HSBs by AG
6372	1	245941754	245942680	ENSMUSG00000054498	ENSMUSG00000054498	ENSMUSG00000054498	7	86196785	86197711	out-of-place	143	out of place markers were called HSBs by AG	0 -> 1
6373	11	88699163	88864301	ENSMUSG00000030562	ENSMUSG00000030562	ENSMUSG00000030562	7	87122556	87271624	131	144		
6374	11	88550268	88668474	ENSMUSG00000004651	ENSMUSG00000004651	ENSMUSG00000004651	7	87303166	87369172	131	144		
6375	11	87881006	88420838	ENSMUSG00000049583	ENSMUSG00000049583	ENSMUSG00000049583	7	87477812	88006270	131	144		
6376	11	87666421	87710586	ENSMUSG00000030560	ENSMUSG00000030560	ENSMUSG00000030560	7	88153327	88186080	131	144		
6377	11	87486096	87548283	ENSMUSG00000030559	ENSMUSG00000030559	ENSMUSG00000030559	7	88305465	88396563	131	144		
6378	11	86426713	86712216	ENSMUSG00000039428	ENSMUSG00000039428	ENSMUSG00000039428	7	89014913	89213788	131	144		
6379	11	86334370	86344081	ENSMUSG00000049791	ENSMUSG00000049791	ENSMUSG00000049791	7	89279586	89285277	131	144		
6380	11	86189211	86199917	ENSMUSG00000039405	ENSMUSG00000039405	ENSMUSG00000039405	7	89382977	89392744	131	144		
6381	11	85829798	86061075	ENSMUSG00000030621	ENSMUSG00000030621	ENSMUSG00000030621	7	89508010	89729551	131	144		
6382	11	85763426	85811798	ENSMUSG00000039391	ENSMUSG00000039391	ENSMUSG00000039391	7	89741588	89778803	131	144		
6383	11	85690936	85734632	ENSMUSG00000062797	ENSMUSG00000062797	ENSMUSG00000062797	7	89793881	89816377	131	144		
6384	11	85633463	85667215	ENSMUSG00000030619	ENSMUSG00000030619	ENSMUSG00000030619	7	89829868	89856162	131	144		
6385	11	85346134	85457756	ENSMUSG00000039361	ENSMUSG00000039361	ENSMUSG00000039361	7	90005765	90072224	131	144		
6386	11	85243792	85308699	ENSMUSG00000030617	ENSMUSG00000030617	ENSMUSG00000030617	7	90110953	90140624	131	144		
6387	11	85082982	85116719	ENSMUSG00000030616	ENSMUSG00000030616	ENSMUSG00000030616	7	90223891	90285630	131	144		
6388	11	85073697	85074821	ENSMUSG00000044362	ENSMUSG00000044362	ENSMUSG00000044362	7	90301769	90303854	131	144		
6389	11	85048415	85053348	ENSMUSG00000051451	ENSMUSG00000051451	ENSMUSG00000051451	7	90318301	90319524	131	144		
6390	11	85036669	85045227	ENSMUSG00000030615	ENSMUSG00000030615	ENSMUSG00000030615	7	90325904	90332400	131	144		
6391	11	85017296	85025210	ENSMUSG00000030614	ENSMUSG00000030614	ENSMUSG00000030614	7	90344015	90351187	131	144		
6392	11	82650154	82675045	ENSMUSG00000030613	ENSMUSG00000030613	ENSMUSG00000030613	7	92436707	92457485	131	144		
6393	11	82582939	82636691	ENSMUSG00000041343	ENSMUSG00000041343	ENSMUSG00000041343	7	92457204	92512295	131	144		
6394	11	82545833	82574481	ENSMUSG00000041328	ENSMUSG00000041328	ENSMUSG00000041328	7	92519447	92545076	131	144		
6395	11	82370127	82460532	ENSMUSG00000030643	ENSMUSG00000030643	ENSMUSG00000030643	7	92616906	92712309	131	144		
6396	11	82303402	82323345	ENSMUSG00000030641	ENSMUSG00000030641	ENSMUSG00000030641	7	92732721	92749397	131	144		
6397	11	82213058	82289205	ENSMUSG00000061119	ENSMUSG00000061119	ENSMUSG00000061119	7	92750489	92809191	131	144		
6398	11	78046754	78458637	ENSMUSG00000048078	ENSMUSG00000048078	ENSMUSG00000048078	7	96425197	96783746	131	144		
6399	11	77824895	77963474	ENSMUSG00000018995	ENSMUSG00000018995	ENSMUSG00000018995	7	96826719	96939947	131	144		
6400	11	77603990	77730574	ENSMUSG00000004508	ENSMUSG00000004508	ENSMUSG00000004508	7	96956943	97180228	131	144		
6401	11	77584940	77602507	ENSMUSG00000035713	ENSMUSG00000035713	ENSMUSG00000035713	7	97195159	97199542	131	144		
6402	11	77559951	77577312	ENSMUSG00000044952	ENSMUSG00000044952	ENSMUSG00000044952	7	97207519	97225401	131	144		
6403	11	77489636	77528282	ENSMUSG00000035704	ENSMUSG00000035704	ENSMUSG00000035704	7	97246820	97267358	131	144		
6404	11	77457051	77468579	ENSMUSG00000030647	ENSMUSG00000030647	ENSMUSG00000030647	7	97275269	97282991	131	144		
6405	11	77452555	77457044	ENSMUSG00000035686	ENSMUSG00000035686	ENSMUSG00000035686	7	97288136	97292702	131	144		
6406	11	77404412	77411934	ENSMUSG00000051727	ENSMUSG00000051727	ENSMUSG00000051727	7	97328396	97333677	131	144		
6407	11	77209849	77261045	ENSMUSG00000035642	ENSMUSG00000035642	ENSMUSG00000035642	7	97425763	97454640	131	144		
6408	11	77054923	77209528	ENSMUSG00000035623	ENSMUSG00000035623	ENSMUSG00000035623	7	97455114	97561176	131	144		
6409	11	76978328	76998339	ENSMUSG00000042797	ENSMUSG00000042797	ENSMUSG00000042797	7	97601571	97613439	131	144		
6410	11	76710709	76862581	ENSMUSG00000030774	ENSMUSG00000030774	ENSMUSG00000030774	7	97718145	97787562	131	144		
6411	11	76605630	76676111	ENSMUSG00000035582	ENSMUSG00000035582	ENSMUSG00000035582	7	97833013	97923803	131	144		
6412	11	76531241	76603931	ENSMUSG00000030761	ENSMUSG00000030761	ENSMUSG00000030761	7	97926593	97982312	131	144		
6413	11	76455666	76514844	ENSMUSG00000035547	ENSMUSG00000035547	ENSMUSG00000035547	7	97996757	98053385	131	144		
6414	11	76423108	76430651	ENSMUSG00000074004	ENSMUSG00000074004	ENSMUSG00000074004	7	98067615	98074673	131	144		
6415	11	76249601	76411592	ENSMUSG00000030760	ENSMUSG00000030760	ENSMUSG00000030760	7	98088852	98184719	131	144		
6416	11	76171933	76186845	ENSMUSG00000049580	ENSMUSG00000049580	ENSMUSG00000049580	7	98225869	98236480	131	144		
6417	11	75833717	75940234	ENSMUSG00000035401	ENSMUSG00000035401	ENSMUSG00000035401	7	98465807	98531761	131	144		
6418	11	75738652	75769528	ENSMUSG00000030753	ENSMUSG00000030753	ENSMUSG00000030753	7	98578555	98593254	131	144		
6419	11	75575018	75595222	ENSMUSG00000015957	ENSMUSG00000015957	ENSMUSG00000015957	7	98711321	98729939	131	144		
6420	11	75203923	75531342	ENSMUSG00000035354	ENSMUSG00000035354	ENSMUSG00000035354	7	98761945	99016313	131	144		
6421	11	75157456	75190225	ENSMUSG00000030747	ENSMUSG00000030747	ENSMUSG00000030747	7	99028850	99057909	131	144		
6422	11	75106582	75119979	ENSMUSG00000052396	ENSMUSG00000052396	ENSMUSG00000052396	7	99094276	99113795	131	144		
6423	11	74975612	75057127	ENSMUSG00000055407	ENSMUSG00000055407	ENSMUSG00000055407	7	99141577	99211219	131	144		
6424	11	74955043	74961473	ENSMUSG00000070436	ENSMUSG00000070436	ENSMUSG00000070436	7	99219453	99227179	131	144		
6425	11	74823334	74914515	ENSMUSG00000035314	ENSMUSG00000035314	ENSMUSG00000035314	7	99255632	99335067	131	144		
6426	11	74811127	74818884	ENSMUSG00000035298	ENSMUSG00000035298	ENSMUSG00000035298	7	99340110	99348100	131	144		
6427	11	74788223	74810972	ENSMUSG00000030744	ENSMUSG00000030744	ENSMUSG00000030744	7	99352967	99357734	131	144		
6428	11	74654130	74740521	ENSMUSG00000018909	ENSMUSG00000018909	ENSMUSG00000018909	7	99409569	99480854	131	144		
6429	11	74630211	74630891	ENSMUSG00000060814	ENSMUSG00000060814	ENSMUSG00000060814	7	99499577	99500257	131	144		
6430	11	74539809	74594945	ENSMUSG00000030737	ENSMUSG00000030737	ENSMUSG00000030737	7	99531887	99585423	131	144		
6431	11	74377251	74395777	ENSMUSG00000035239	ENSMUSG00000035239	ENSMUSG00000035239	7	99685520	99702500	131	144		
6432	11	74231664	74337809	ENSMUSG00000035211	ENSMUSG00000035211	ENSMUSG00000035211	7	99741434	99791722	131	144		

6433	11	74137561	74231095	ENSMUSG00000058761	ENSMUSG00000058761	ENSMUSG00000058761	7	99794340	99829614	131	144		
6434	11	74085122	74119875	ENSMUSG00000030732	ENSMUSG00000030732	ENSMUSG00000030732	7	99880522	99908809	131	144		
6435	11	73981277	74031413	ENSMUSG00000030726	ENSMUSG00000030726	ENSMUSG00000030726	7	99956195	99995586	131	144		
6436	11	73880828	73882396	ENSMUSG00000030725	ENSMUSG00000030725	ENSMUSG00000030725	7	100033360	100035448	131	144		
6437	11	73843536	73856186	ENSMUSG00000035165	ENSMUSG00000035165	ENSMUSG00000035165	7	100052780	100058947	131	144		
6438	11	73719009	73787150	ENSMUSG00000030729	ENSMUSG00000030729	ENSMUSG00000030729	7	100101707	100149441	131	144		
6439	11	73655350	73700347	ENSMUSG00000051048	ENSMUSG00000051048	ENSMUSG00000051048	7	100159616	100193775	131	144		
6440	11	73423188	73559526	ENSMUSG00000047248	ENSMUSG00000047248	ENSMUSG00000047248	7	100248501	100344234	131	144		
6441	11	73388985	73397778	ENSMUSG00000032942	ENSMUSG00000032942	ENSMUSG00000032942	7	100347073	100360514	131	144		
6442	11	73363360	73372000	ENSMUSG00000033685	ENSMUSG00000033685	ENSMUSG00000033685	7	100367440	100376255	131	144		
6443	11	73339012	73358980	ENSMUSG00000030708	ENSMUSG00000030708	ENSMUSG00000030708	7	100377101	100389042	131	144		
6444	11	73261360	73265550	ENSMUSG00000044881	ENSMUSG00000044881	ENSMUSG00000044881	7	100411216	100415431	131	144		
6445	11	73176009	73253304	ENSMUSG00000030706	ENSMUSG00000030706	ENSMUSG00000030706	7	100422882	100482388	131	144		
6446	11	73037671	73051510	ENSMUSG00000030701	ENSMUSG00000030701	ENSMUSG00000030701	7	100517983	100536452	131	144		
6447	11	72789493	72986739	ENSMUSG00000029461	ENSMUSG00000029461	ENSMUSG00000029461	7	100580738	100715420	131	144		
6448	11	72765053	72786166	ENSMUSG00000008318	ENSMUSG00000008318	ENSMUSG00000008318	7	100719935	100737482	131	144		
6449	11	72697317	72757783	ENSMUSG00000032875	ENSMUSG00000032875	ENSMUSG00000032875	7	100746222	100768777	131	144		
6450	11	72653218	72686497	ENSMUSG00000048779	ENSMUSG00000048779	ENSMUSG00000048779	7	100811721	100838449	131	144		
6451	11	72606992	72625041	ENSMUSG00000032860	ENSMUSG00000032860	ENSMUSG00000032860	7	100870659	100886078	131	144		
6452	11	72225460	72530739	ENSMUSG00000030691	ENSMUSG00000030691	ENSMUSG00000030691	7	100982900	101158492	131	144		
6453	11	72203099	72218326	ENSMUSG00000047767	ENSMUSG00000047767	ENSMUSG00000047767	7	101163714	101176338	131	144		
6454	11	72073768	72182292	ENSMUSG00000032812	ENSMUSG00000032812	ENSMUSG00000032812	7	101246814	101285142	131	144		
6455	11	71964834	72063113	ENSMUSG00000030653	ENSMUSG00000030653	ENSMUSG00000030653	7	101324449	101386635	131	144		
6456	11	71681122	71823216	ENSMUSG00000001829	ENSMUSG00000001829	ENSMUSG00000001829	7	101537589	101663989	131	144		
6457	11	71627772	71632852	ENSMUSG00000007946	ENSMUSG00000007946	ENSMUSG00000007946	7	101692311	101696550	131	144		
6458	11	71613473	71627797	ENSMUSG00000032737	ENSMUSG00000032737	ENSMUSG00000032737	7	101696482	101711897	131	144		
6459	11	71578250	71584992	ENSMUSG00000001827	ENSMUSG00000001827	ENSMUSG00000001827	7	101732171	101744602	131	144		
6460	11	71469553	71485584	ENSMUSG00000064307	ENSMUSG00000064307	ENSMUSG00000064307	7	101786814	101807627	131	144		
6461	11	71391559	71469221	ENSMUSG00000066306	ENSMUSG00000066306	ENSMUSG00000066306	7	101851230	101887774	131	144		
6462	11	71387757	71392447	ENSMUSG00000070427	ENSMUSG00000070427	ENSMUSG00000070427	7	101888895	101891976	131	144		
6463	11	71317731	71386289	ENSMUSG00000070426	ENSMUSG00000070426	ENSMUSG00000070426	7	101892965	101939288	131	144		
6464	11	3606503	3615365	ENSMUSG00000070425	ENSMUSG00000070425	ENSMUSG00000070425	7	101949697	101970155	132	145		
6465	11	3616320	3620122	ENSMUSG00000070424	ENSMUSG00000070424	ENSMUSG00000070424	7	101970880	101973314	132	145		
6466	11	3622937	3642222	ENSMUSG00000030996	ENSMUSG00000030996	ENSMUSG00000030996	7	101975564	101987754	132	145		
6467	11	3643394	3649190	ENSMUSG00000066279	ENSMUSG00000066279	ENSMUSG00000066279	7	101985087	101990534	132	145		
6468	11	3652817	3775468	ENSMUSG00000063550	ENSMUSG00000063550	ENSMUSG00000063550	7	101993222	102030950	132	145		
6469	11	3786360	3804156	ENSMUSG00000030990	ENSMUSG00000030990	ENSMUSG00000030990	7	102096895	102111570	132	145		
6470	11	3804788	3818760	ENSMUSG00000073982	ENSMUSG00000073982	ENSMUSG00000073982	7	102113490	102114065	132	145		
6471	11	3833509	4071013	ENSMUSG00000030987	ENSMUSG00000030987	ENSMUSG00000030987	7	102141627	102310669	132	145		
6472	11	4072587	4116681	ENSMUSG00000030978	ENSMUSG00000030978	ENSMUSG00000030978	7	102315638	102342746	132	145		
6473	11	4362734	4371502	ENSMUSG00000030966	ENSMUSG00000030966	ENSMUSG00000030966	7	102432657	102439262	132	145		
6474	11	4409136	4410035	ENSMUSG00000073974	ENSMUSG00000073974	ENSMUSG00000073974	7	102461615	102462562	132	145		
6475	11	4522997	4523950	ENSMUSG00000073971	ENSMUSG00000073971	ENSMUSG00000073971	7	102514069	102515022	132	145		
6476	11	4576586	4586010	ENSMUSG00000073968	ENSMUSG00000073968	ENSMUSG00000073968	7	102551398	102561075	132	145		
6477	11	4617597	4618571	ENSMUSG00000073967	ENSMUSG00000073967	ENSMUSG00000073967	7	102572061	102573029	132	145		
6478	11	4621788	4633288	ENSMUSG00000070423	ENSMUSG00000070423	ENSMUSG00000070423	7	102576144	102585875	132	145		
6479	11	4657977	4675648	ENSMUSG00000043366	ENSMUSG00000043366	ENSMUSG00000043366	7	102614552	102633216	132	145		
6480	11	4687411	4688274	ENSMUSG00000073966	ENSMUSG00000073966	ENSMUSG00000073966	7	102648347	102649291	132	145		
6481	11	4799192	4800220	ENSMUSG00000073965	ENSMUSG00000073965	ENSMUSG00000073965	7	102750943	102751884	132	145		
6482	11	4826043	4827014	ENSMUSG00000043310	ENSMUSG00000043310	ENSMUSG00000043310	7	102782690	102783658	132	145		
6483	11	4859625	4860689	ENSMUSG00000045824	ENSMUSG00000045824	ENSMUSG00000045824	7	102822288	102823328	132	145		
6484	11	4867405	4868402	ENSMUSG00000066269	ENSMUSG00000066269	ENSMUSG00000066269	7	102828485	102829441	132	145		
6485	11	4885176	4886114	ENSMUSG00000073962	ENSMUSG00000073962	ENSMUSG00000073962	7	102838923	102848981	132	145		
6486	11	4892525	4893469	ENSMUSG00000043354	ENSMUSG00000043354	ENSMUSG00000043354	7	102846873	102847811	132	145		
6487	11	4901180	4902145	ENSMUSG00000045792	ENSMUSG00000045792	ENSMUSG00000045792	7	102858042	102858983	132	145		
6488	11	5024332	5025267	ENSMUSG00000073956	ENSMUSG00000073956	ENSMUSG00000073956	7	103060424	103061362	132	145		
6489	11	5155520	5156359	ENSMUSG00000047535	ENSMUSG00000047535	ENSMUSG00000047535	7	103661033	103665651	132	145		
6490	11	5226130	5227693	ENSMUSG00000052217	ENSMUSG00000052217	ENSMUSG00000052217	7	103715459	103716983	132	145		
6491	11	5367205	5368185	ENSMUSG00000042219	ENSMUSG00000042219	ENSMUSG00000042219	7	103788883	103803732	132	145		
6492	11	5418376	5419320	ENSMUSG00000066262	ENSMUSG00000066262	ENSMUSG00000066262	7	103895193	103896137	132	145		
6493	11	5431295	5432233	ENSMUSG00000073932	ENSMUSG00000073932	ENSMUSG00000073932	7	103913619	103914557	132	145		
6494	11	5466513	5467469	ENSMUSG00000073931	ENSMUSG00000073931	ENSMUSG00000073931	7	103980102	103981040	132	145		
6495	11	5485106	5487791	ENSMUSG00000051618	ENSMUSG00000051618	ENSMUSG00000051618	7	104014444	104017093	132	145		
6496	11	5492199	5494511	ENSMUSG00000051437	ENSMUSG00000051437	ENSMUSG00000051437	7	104022277	104024109	132	145		
6497	11	5522367	5523329	ENSMUSG00000042909	ENSMUSG00000042909	ENSMUSG00000042909	7	104053277	104054227	132	145		
6498	11	5765660	5766622	ENSMUSG00000051885	ENSMUSG00000051885	ENSMUSG00000051885	7	104607263	104608183	132	145		
6499	11	5798142	5799107	ENSMUSG00000063582	ENSMUSG00000063582	ENSMUSG00000063582	7	104766491	104767447	132	145		
6500	11	5755466	5756440	ENSMUSG00000073916	ENSMUSG00000073916	ENSMUSG00000073916	7	104812349	104813302	132	145		
6501	11	5862099	5863037	ENSMUSG00000073914	ENSMUSG00000073914	ENSMUSG00000073914	7	104930069	104931007	132	145		
6502	11	5878583	5879896	ENSMUSG00000073913	ENSMUSG00000073913	ENSMUSG00000073913	7	104943290	104944231	132	145		
6503	11	5944983	5946096	ENSMUSG00000044120	ENSMUSG00000044120	ENSMUSG00000044120	7	105017171	105018130	132	145		
6504	11	5979857	5980954	ENSMUSG00000047225	ENSMUSG00000047225	ENSMUSG00000047225	7	105030563	105031501	132	145		
6505	11	6129582	6130394	ENSMUSG00000050266	ENSMUSG00000050266	ENSMUSG00000050266	7	105203064	105204026	132	145		
6506	11	6177030	6177992	ENSMUSG00000073906	ENSMUSG00000073906	ENSMUSG00000073906	7	105242149	105243138	132	145		
6507	11	6189153	6212451	ENSMUSG00000044465	ENSMUSG00000044465	ENSMUSG00000044465	7	105252432	105273832	132	145		
6508	11	6216906	6222283	ENSMUSG00000030897	ENSMUSG00000030897	ENSMUSG00000030897	7	105278389	105282559	132	145		
6509	11	6237542	6249932	ENSMUSG00000030898	ENSMUSG00000030898	ENSMUSG00000030898	7	105299641	105310159	132	145		
6510	11	6296752	6298316	ENSMUSG00000037060	ENSMUSG00000037060	ENSMUSG00000037060	7	105354428	105356018	132	145		
6511	11	6368231	6372801	ENSMUSG00000037049	ENSMUSG00000037049	ENSMUSG00000037049	7	105428213	105432204	132	145		
6512	11	6372964	6389153	ENSMUSG00000037032	ENSMUSG00000037032	ENSMUSG00000037032	7	105432305	105442536	132	145		
6513	11	6408858	6418830	ENSMUSG00000030895	ENSMUSG00000030895	ENSMUSG00000030895	7	105465432	105473917	132	145		
6514	11	6426423	6451790	ENSMUSG00000036989	ENSMUSG00000036989	ENSMUSG00000036989	7	105484291	105507320	132	145		
6515	11	6453504	6459171	ENSMUSG00000030881	ENSMUSG00000030881	ENSMUSG00000030881	7	105508030	105513244	132	145		
6516	11	6517670	6526841	ENSMUSG00000030882	ENSMUSG00000030882	ENSMUSG00000030882	7	105530709	105595375	132	145		
6517	11	6572881	6581332	ENSMUSG00000030888	ENSMUSG00000030888	ENSMUSG00000030888	7	105605835	105611122	132	145		
6518	11	6581540	6588673	ENSMUSG00000030890	ENSMUSG00000030890	ENSMUSG00000030890	7	105610473	105616738	132	145		
6519	11	6590576	6597236	ENSMUSG00000030894	ENSMUSG00000030894	ENSMUSG00000030894	7	105618668	105626028	132	145		
6520	11	6599134	6633656	ENSMUSG00000036862	ENSMUSG00000036862	ENSMUSG00000036862	7	105626812	105630302	132	145		
6521	11	6658589	6661150	ENSMUSG00000030879	ENSMUSG00000030879	ENSMUSG00000030879	7	105677603	105684908	132	145		
6522	11	6772532	6773515	ENSMUSG00000070417	ENSMUSG00000070417	ENSMUSG00000070417	7	106794524	106795507	132	145		
6523	11	6854428	6855402	ENSMUSG00000073897	ENSMUSG00000073897	ENSMUSG00000073897	7	106888626	106889591	132	145		
6524	11	6898809	6899801	ENSMUSG00000073896	ENSMUSG00000073896	ENSMUSG00000073896	7	106938495	106939439	132	145		
6525	11	7016373	7049333	ENSMUSG00000016626	ENSMUSG00000016626	ENSMUSG00000016626	7	106958167	106989279	132	145		
6526	11	7066859	7068955	ENSMUSG00000073894	ENSMUSG00000073894	ENSMUSG00000073894	7	107000622	107002093	132	145		
6527	11	7229757	7446844	ENSMUSG00000062542	ENSMUSG00000062542	ENSMUSG00000062542	7	107162168	107297801	132	145		
6528	11	7463289	7489178	ENSMUSG00000051041	ENSMUSG00000051041	ENSMUSG00000051041	7	107358943	107382113	132	145		
6529	11	7491627	7631558	ENSMUSG00000036528	ENSMUSG00000036528	ENSMUSG00000036528	7	107398021	107539759	132	145		
6530	11	7642909	7651397	ENSMUSG00000048065	ENSMUSG00000048065	ENSMUSG00000048065	7	107539632	107549173	132	145		
6531	11	7669020	7684517	ENSMUSG00000048236	ENSMUSG00000048236	ENSMUSG00000048236	7	107572815	107592356	132	145		
6532	11	7937547	7941780	ENSMUSG00000049709	ENSMUSG00000049709	ENSMUSG00000049709	7	108713030	108721335	132	145		
6533	11	8016756	8084228	ENSMUSG00000031028	ENSMUSG00000031028	ENSMUSG00000031028	7	108802076	108825502	132	145		
6534	11	8087072	8147148	ENSMUSG00000048330	ENSMUSG00000048330	ENSMUSG00000048330	7	108825462	108874501	132	145		
6535	11	8202437	8246758	ENSMUSG00000036111	ENSMUSG00000036111	ENSMUSG00000036111	7	108930048	108934847	132	145		
6536	11	8369994	8572079	ENSMUSG00000031027	ENSMUSG00000031027	ENSMUSG00000031027	7	109111974	109132678	132	145		
6537	11	8593662	8636959	ENSMUSG00000031026	ENSMUSG00000031026	ENSMUSG00000031026	7	109244959	109299311	132	145		
6538	11	8671478	8817478	ENSMUSG00000031024	ENSMUSG00000031024	ENSMUSG00000031024	7	109315088	109408324	132	145		
6539	11	8889277	8898200	ENSMUSG00000031023	ENSMUSG00000031023	ENSMUSG00000031023	7	109494914	109503358	132	145		
6540	11	8898200	8911103	ENSMUSG00000031022	ENSMUSG00000031022	ENSMUSG00000031022	7	109503359	109515055	132	145		
6541	11	8915695	8921156	ENSMUSG00000035951	ENSMUSG00000035951	ENSMUSG00000035951	7	109518642	109522909	132	145		
6542	11	8925417	8942578	ENSMUSG00000031021	ENSMUSG00000031021	ENSMUSG00000031021	7	109527013	109543440	132	145		
6543	11	8960970	8982124	ENSMUSG00000034825	ENSMUSG00000034825	ENSMUSG00000034825	7	109549234	109573202	132	145		
6544	11	8998511	9069731	ENSMUSG00000007279	ENSMUSG00000007279	ENSMUSG00000007279	7	109589826	109656831	132	145		
6545	11	9116957	9243447	ENSMUSG00000035901	ENSMUSG00000035901	ENSMUSG00000035901	7	109684957	109751599	132	145		
6546	11	9362780	9423651	ENSMUSG00000066232	ENSMUSG00000066232	ENSMUSG00000066232	7	109809602	109846291	132	145		
6547	11	9439089	9506187	ENSMUSG00000061079	ENSMUSG00000061079	ENSMUSG00000061079	7	109852887	109886564	132	145		
6548	11	9552057	9566725	ENSMUSG00000031016	ENSMUSG00000031016	ENSMUSG00000031016	7	109913527	109933289	132	145		
6549	11	9642248	9731081	ENSMUSG00000031015	ENSMUSG00000031015	ENSMUSG00000031015	7	110012938	110074683	132	145		
6550	11	9756790	10272296	ENSMUSG00000038371	ENSMUSG00000038371	ENSMUSG00000038371	7	110100578	110405938	132	145		
6551	11	10283172	10285491	ENSMUSG00000030790	ENSMUSG00000030790	ENSMUSG00000030790	7	110418838	110420996	132	145		
6552	11	10428800	10485702	ENSMUSG00000005686	ENSMUSG00000005686	ENSMUSG00000005686	7	110563781	110603586	132	145		
6553	11	10489801	10519350	ENSMUSG00000030788	ENSMUSG00000030788	ENSMUSG00000030788	7	110604708	110635623	132	145		
6554	11	10535990	10546855	ENSMUSG00000030787	ENSMUSG00000030787	ENSMUSG00000030787	7	110641788	110654134	132	145		
6555	11	10553885	10630499	ENSMUSG00000005611	ENSMUSG00000005611	ENSMUSG00000005611	7	110659453	110773642	132	145		
6556	11	10729379	10757863	ENSMUSG00000005609	ENSMUSG00000005609	ENSMUSG00000005609	7	110820202	110847517	132	145		
6557	11	10775178	10787078	ENSMUSG00000005610	ENSMUSG00000005610	ENSMUSG00000005610	7	110863113	110874211	132	145		
6558	11	11248997	11600125	ENSMUSG00000038296	ENSMUSG00000038296	ENSMUSG00000038296	7	111262837	111571158	132	145		
6559	11	11851923	11934528	ENSMUSG00000059263	ENSMUSG00000059263	ENSMUSG00000059263	7	111838108	111902561	132	145		
6560	11	11941123	11987493	ENSMUSG00000030772	ENSMUSG00000030772	ENSMUSG00000030772	7	111907200	111950238	132	145		
6561	11	12088714	12241903	ENSMUSG00000038244	ENSMUSG00000038244	ENSMUSG00000038244	7	112062478	112145156	132	145		
6562	11	12265023	12297967	ENSMUSG00000030771	ENSMUSG00000030771	ENSMUSG00000030771	7	112165514	112186528	132	145		
6563	11	12355679	12508914	ENSMUSG00000030770	ENSMUSG00000030770	ENSMUSG00000030770	7	112218887	112382873	132	145		
6564	11	12723164	12922066	ENSMUSG00000055320	ENSMUSG00000055320	ENSMUSG00000055320	7	112470774	112691171	132	145		
6565	11	13255921	13365345	ENSMUSG00000055116	ENSMUSG00000055116	ENSMUSG00000055116	7	112998646	113105303	132	145		
6566	11	13470178	13474143	ENSMUSG00000059077	ENSMUSG00000059077	ENSMUSG00000059077	7	113176759	113179753	132	145		
6567	11	13646847	13710467	ENSMUSG00000030759	ENSMUSG00000030759	ENSMUSG00000030759	7	113330459	113359438	132	145		
6568	11	13940814	14245926	ENSMUSG00000038156	ENSMUSG00000038156	ENSMUSG00000038156	7	113557321	113832556	132	145		
6569	11	14256889	14337289	ENSMUSG00000055723	ENSMUSG00000055723	ENSMUSG00000055723	7	113837969	113908962	132	145		
6570	11	14435631	14477974	ENSMUSG00000030754	ENSMUSG00000030754	ENSMUSG00000030754	7	114006740	114045861	132	145		
6571	11	14482999	14621739	ENSMUSG00000030751	ENSMUSG00000030751	ENSMUSG00000030751	7	114056081	114065641	132	145		
6572	11	14621913	14848926	ENSMUSG00000030671	ENSMUSG00000030671	ENSMUSG00000030671	7	114206462	114327893	132	145		
6573	11	14856142	14870327	ENSMUSG00000030670	ENSMUSG00000030670	ENSMUSG00000030670	7	114341345	114354153	132	145		
6574	11	14945162	14950392	ENSMUSG00000030669	ENSMUSG00000030669	ENSMUSG00000030669	7	114424440	114427437	132	145		
6575	11	15051719	15056754	ENSMUSG00000030666	ENSMUSG00000030666	ENSMUSG00000030666	7	114509786	114514372	132	145		
6576	11	15127230	15224198	ENSMUSG00000048782	ENSMUSG00000048782	ENSMUSG00000048782	7	114559882	114641521	132	145		
6577	11	15950833	16380985	ENSMUSG00000051910	ENSMUSG00000051910	ENSMUSG00000051910	7	115262493	115786235	132	145		
6578	11	16716770	16734149	ENSMUSG00000030663	ENSMUSG00000030663	ENSMUSG00000030663	7	115884489	115896356	132	145		
6579	11	16765791	16992535	ENSMUSG00000045659	ENSMUSG00000045659	ENSMUSG00000045659	7	115917196	116099482	132	145		
6580	11	17067861	17147864	ENSMUSG00000030660	ENSMUSG00000030660	ENSMUSG00000030660	7	116131091	116209669	132	145		
6581	11	17273447	17309114	ENSMUSG00000030659	ENSMUSG00000030659	ENSMUSG00000030659	7	116312986	116331183	132	145		
6582	16	17108840	17472239	ENSMUSG00000030657	ENSMUSG00000030657	ENSMUSG00000030657	7	117172128	117458779	Singleton	146		
6583	16	18710492	18720367	ENSMUSG00000030654	ENSMUSG00000030654	ENSMUSG00000030654	7	117910048	117920760	133	146		
6584	16	18728395	18844864	ENSMUSG00000030655	ENSMUSG00000030655	ENSMUSG00000030655	7	117922461	118034786	133	146		
6585	16	19087139	19186055	ENSMUSG00000058420	ENSMUSG00000058420	ENSMUSG00000058420	7	118173005	118233648	133	146		
6586	16	18986428	18998851	ENSMUSG00000030652	ENSMUSG00000030652	ENSMUSG00000030652	7	118300959	118324462	133	146		
6587	16	18902828	18982000	ENSMUSG00000042246	ENSMUSG00000042246	ENSMUSG00000042246	7	118326993	118375835	133	146		
6588	16	19329558	19417038	ENSMUSG00000030650	ENSMUSG00000030650	ENSMUSG00000030650	7	118414228	118466216	133	146		
6589	16	19420518	19440951	ENSMUSG00000033917	ENSMUSG00000033917	ENSMUSG00000033917	7	118479707	118496899	133	146		
6590	16	19442780	19472223	ENSMUSG00000033904	ENSMUSG00000033904	ENSMUSG00000033904	7	118506499	118528173	133	146		
6591	16	19474541	19619943	ENSMUSG00000030982	ENSMUSG00000030982	ENSMUSG00000030982	7	118531890	118632634	133	146		
6592	16	19625736	19637131	ENSMUSG00000030980	ENSMUSG00000030980	ENSMUSG00000030980	7	118636909	118647408	133	146		
6593	16	19635279	19776358	ENSMUSG00000073856	ENSMUSG00000073856	ENSMUSG00000073856	7	118646925	118748980	133	146		
6594	16	19777796	19803652	ENSMUSG00000008734	ENSMUSG00000008734	ENSMUSG00000008734	7	118763196	118786310	133	146		
6595	16	19950544	19992530	ENSMUSG00000066197	ENSMUSG00000066197	ENSMUSG00000066197	7	118935472	118975523	133	146		
6596	16	20228398	20246403	ENSMUSG00000030954	ENSMUSG00000030954	ENSMUSG00000030954	7	119233779	119248445	133	146		
6597	16	20251875	20271533	ENSMUSG00000030963	ENSMUSG00000030963	ENSMUSG00000030963	7	119254013	119270404	133	146		
6598	16	20277998	20323560	ENSMUSG00000030968	ENSMUSG00000030968	ENSMUSG00000030968	7	119277736	119314631	133	146		
6599	16	20328357	20360149	ENSMUSG00000030972	ENSMUSG00000030972	ENSMUSG00000030972	7	119317453	119334942	133	146		
6600	16	20542179	20610079	ENSMUSG00000033533	ENSMUSG00000033533	ENSMUSG00000033533	7	119408972	119453664	133	146		
6601	16	20652492	20660907	ENSMUSG00000030942	ENSMUSG00000030942	ENSMUSG00000030942	7	119506242	119511914	133	146		
6602	16	20682813	20715979	ENSMUSG00000030935	ENSMUSG00000030935	ENSMUSG00000030935	7	119552072	119576282	133	146		
6603	16	20716550	20722501	ENSMUSG00000030929	ENSMUSG00000030929	ENSMUSG00000030929	7	119573718	119583670	133	146		
6604	16	20725322	20768485	ENSMUSG00000030924	ENSMUSG00000030924	ENSMUSG00000030924	7	119590714	119639953	133	146		
6605	16	20776918	20819161	ENSMUSG00000048787	ENSMUSG00000048787	ENSMUSG00000048787	7	119646960	119686825	133	146		
6606	16	20819637	20843797	ENSMUSG00000030922	ENSMUSG00000030922	ENSMUSG00000030922	7	119700953	119707406	133	146		
6607	16	20851977	21078263	ENSMUSG00000052273	ENSMUSG00000052273	ENSMUSG00000052273	7	119717128	119743306	133	146		
6608	16	21077501	21099431	ENSMUSG00000030917	ENSMUSG00000030917	ENSMUSG00000030917	7	119893575	119912135	133	146		
6609	16	21116274	21130369	ENSMUSG00000030911	ENSMUSG00000030911	ENSMUSG00000030911	7	119923510	119936439	133	146		
6610	16	21152560	21169642	ENSMUSG00000030909	ENSMUSG00000030909	ENSMUSG00000030909	7	119965025	119974863	133	146		
6611	16	21177344	21197782	ENSMUSG00000030905	ENSMUSG00000030905	ENSMUSG00000030905	7	119977533	119993137	133	146		
6612	16	21871482	21902174	ENSMUSG00000030884	ENSMUSG00000030884	ENSMUSG00000030884	7	120426395	120450672	133	146		
6613	16	21903089	21914084	ENSMUSG00000030887	ENSMUSG00000030887	ENSMUSG00000030887	7	120449880	120461399	133	146		
6614	16	21983224	22002523	ENSMUSG00000046096	ENSMUSG00000046096	ENSMUSG00000046096	7	120468895	120523626	133	146		
6615	16	22016392	22074715	ENSMUSG00000030889	ENSMUSG00000030889	ENSMUSG00000030889	7	120543682	120595674	133	146		
6616	16	22085092	22109167	ENSMUSG00000035077	ENSMUSG00000035077	ENSMUSG00000035077	7	120604153	120622192	133	146		
6617	16	22125248	22205450	ENSMUSG00000035064	ENSMUSG00000035064	ENSMUSG00000035064	7	120634032	120695150	133	146		
6618	16	22216231	22252840	ENSMUSG00000030880	ENSMUSG00000030880	ENSMUSG00000030880	7	120708966	120737398	133	146		
6619	16	22264758	22293439	ENSMUSG00000030878	ENSMUSG00000030878	ENSMUSG00000030878	7	120748185	120773433	133	146		
6620	16	21518413	21576292	ENSMUSG00000030876	ENSMUSG00000030876	ENSMUSG00000030876	7	120825594	120867984	133	147	here	
6621	16	21559426	21571430	ENSMUSG00000035004	ENSMUSG00000035004	ENSMUSG00000035004	7	120855216	120865679	133	147	Markers moved to different place with in a HSB	1 -> 3
6622	16	21597336	21679550	ENSMUSG00000034990	ENSMUSG00000034990	ENSMUSG00000034990	7	120874587	120954241	133	147		
6623	16	22732999	22835572	ENSMUSG00000046321	ENSMUSG00000046321	ENSMUSG00000046321	7	121183878	121292184	133	148		
6624	16	22986868	23068092	ENSMUSG00000063317	ENSMUSG00000063317	ENSMUSG00000063317	7	121439335	121459015	133	148		
6625	16	23101541	23135701	ENSMUSG00000000216	ENSMUSG00000000216	ENSMUSG00000000216	7	121525627	121559623	133	148		
6626	16	23222822	23300120	ENSMUSG00000030873	ENSMUSG00000030873	ENSMUSG00000030873	7	121656228	121709876	133	148		
6627	16	23307465	23372000	ENSMUSG00000034951	ENSMUSG00000034951	ENSMUSG00000034951	7	121713991	121772807	133	148		

6628	16	23383145	23429309	ENSMUSG00000030872	ENSMUSG00000030872	ENSMUSG00000030872	7	121777870	121812315	133	148		
6629	16	23441551	23476165	ENSMUSG00000030871	ENSMUSG00000030871	ENSMUSG00000030871	7	121828361	121858192	133	148		
6630	16	23475893	23493211	ENSMUSG00000030870	ENSMUSG00000030870	ENSMUSG00000030870	7	121858346	121873347	133	148		
6631	16	23521984	23560128	ENSMUSG00000044702	ENSMUSG00000044702	ENSMUSG00000044702	7	121898723	121924036	133	148		
6632	16	23560308	23588683	ENSMUSG00000030868	ENSMUSG00000030868	ENSMUSG00000030868	7	121924220	121940192	133	148		
6633	16	23597755	23609188	ENSMUSG00000030867	ENSMUSG00000030867	ENSMUSG00000030867	7	121950586	121960987	133	148		
6634	16	23609198	23632322	ENSMUSG00000030866	ENSMUSG00000030866	ENSMUSG00000030866	7	121961086	121977323	133	148		
6635	16	23673449	23677757	ENSMUSG00000030865	ENSMUSG00000030865	ENSMUSG00000030865	7	122010811	122013972	133	148		
6636	16	23754823	24134803	ENSMUSG00000052889	ENSMUSG00000052889	ENSMUSG00000052889	7	122080445	122419803	133	148		
6637	16	24174382	24281238	ENSMUSG00000066189	ENSMUSG00000066189	ENSMUSG00000066189	7	122462922	122559994	133	148		
6638	16	24458409	24491682	ENSMUSG00000030779	ENSMUSG00000030779	ENSMUSG00000030779	7	122776879	122793701	133	148		
6639	16	24649071	24742629	ENSMUSG00000052707	ENSMUSG00000052707	ENSMUSG00000052707	7	122915811	122984878	133	148		
6640	16	24765053	24830444	ENSMUSG00000030769	ENSMUSG00000030769	ENSMUSG00000030769	7	123026768	123064351	133	148		
6641	16	24838211	24934176	ENSMUSG00000030766	ENSMUSG00000030766	ENSMUSG00000030766	7	123071134	123122415	133	148		
6642	16	25044785	25097053	ENSMUSG00000030763	ENSMUSG00000030763	ENSMUSG00000030763	7	123169297	123219359	133	148		
6643	16	25135784	25147752	ENSMUSG00000030762	ENSMUSG00000030762	ENSMUSG00000030762	7	123253460	123259134	133	148		
6644	16	25154827	25176343	ENSMUSG00000030757	ENSMUSG00000030757	ENSMUSG00000030757	7	123271001	123291115	133	148		
6645	16	27122317	27140544	ENSMUSG00000030752	ENSMUSG00000030752	ENSMUSG00000030752	7	125235824	125253407	133	148		
6646	16	27143817	27187586	ENSMUSG00000030750	ENSMUSG00000030750	ENSMUSG00000030750	7	125258792	125282683	133	148		
6647	16	27259005	27283599	ENSMUSG00000030748	ENSMUSG00000030748	ENSMUSG00000030748	7	125356803	125369129	133	148		
6648	16	27321224	27369616	ENSMUSG00000030745	ENSMUSG00000030745	ENSMUSG00000030745	7	125394685	125424414	133	148		
6649	16	27379436	27468735	ENSMUSG00000032777	ENSMUSG00000032777	ENSMUSG00000032777	7	125432102	125498836	133	148		
6650	16	27468971	27699190	ENSMUSG00000032743	ENSMUSG00000032743	ENSMUSG00000032743	7	125577403	125653144	133	148		
6651	16	27709713	27982323	ENSMUSG00000046182	ENSMUSG00000046182	ENSMUSG00000046182	7	125671851	125873559	133	148		
6652	16	28016817	28130691	ENSMUSG00000000131	ENSMUSG00000000131	ENSMUSG00000000131	7	125893415	125965397	133	148		
6653	16	28211341	28242661	ENSMUSG00000042978	ENSMUSG00000042978	ENSMUSG00000042978	7	126063767	126086163	133	148		
6654	16	28893581	28903362	ENSMUSG00000030741	ENSMUSG00000030741	ENSMUSG00000030741	7	126161215	126168559	133	149	here	This inversion was ignored by our tool
6655	16	28869819	28885266	ENSMUSG00000030722	ENSMUSG00000030722	ENSMUSG00000030722	7	126176129	126187863	133	149	Inversion ignored by ST	0 -> 1
6656	16	28850779	28858164	ENSMUSG00000030724	ENSMUSG00000030724	ENSMUSG00000030724	7	126199599	126205984	133	149		
6657	16	28823244	28844574	ENSMUSG00000030727	ENSMUSG00000030727	ENSMUSG00000030727	7	126219907	126237048	133	149		
6658	16	28797310	28823331	ENSMUSG00000030730	ENSMUSG00000030730	ENSMUSG00000030730	7	126238017	126254070	133	149		
6659	16	28765511	28793027	ENSMUSG00000030733	ENSMUSG00000030733	ENSMUSG00000030733	7	126258154	126266198	133	149		
6660	16	28741857	28755302	ENSMUSG00000032637	ENSMUSG00000032637	ENSMUSG00000032637	7	126282856	126294450	133	149		
6661	16	28396102	28413398	ENSMUSG00000030720	ENSMUSG00000030720	ENSMUSG00000030720	7	126362548	126374368	133	149		
6662	16	28413504	28417791	ENSMUSG00000042759	ENSMUSG00000042759	ENSMUSG00000042759	7	126376148	126380272	133	149		
6663	16	28418184	28425656	ENSMUSG00000044701	ENSMUSG00000044701	ENSMUSG00000044701	7	126380443	126386058	133	149		
6664	16	28456163	28457831	ENSMUSG00000030717	ENSMUSG00000030717	ENSMUSG00000030717	7	126414397	126416637	133	149		
6665	16	28472758	28510610	ENSMUSG00000030714	ENSMUSG00000030714	ENSMUSG00000030714	7	126440519	126463927	133	149		
6666	16	30102427	30107898	ENSMUSG00000030707	ENSMUSG00000030707	ENSMUSG00000030707	7	126490923	126495905	133	150	here	This inversion was ignored by our tool
6667	16	30032951	30042116	ENSMUSG00000063065	ENSMUSG00000063065	ENSMUSG00000063065	7	126550780	126556964	133	150	Inversion ignored by ST	0 -> 2
6668	16	30023633	30032359	ENSMUSG00000030703	ENSMUSG00000030703	ENSMUSG00000030703	7	126557566	126566791	133	150		
6669	16	30011139	30014437	ENSMUSG00000042675	ENSMUSG00000042675	ENSMUSG00000042675	7	126568123	126571657	133	150		
6670	16	30004583	30010709	ENSMUSG00000030699	ENSMUSG00000030699	ENSMUSG00000030699	7	126572631	126576696	133	150		
6671	16	29994839	30004193	ENSMUSG00000030697	ENSMUSG00000030697	ENSMUSG00000030697	7	126577014	126583619	133	150		
6672	16	29943250	29949638	ENSMUSG00000058966	ENSMUSG00000058966	ENSMUSG00000058966	7	126615141	126621362	133	150		
6673	16	29942156	29943524	ENSMUSG00000045989	ENSMUSG00000045989	ENSMUSG00000045989	7	126621622	126622644	133	150		
6674	16	29924694	29931126	ENSMUSG00000052301	ENSMUSG00000052301	ENSMUSG00000052301	7	126638701	126643853	133	150		
6675	16	29914532	29924527	ENSMUSG00000030689	ENSMUSG00000030689	ENSMUSG00000030689	7	126643581	126652611	133	150		
6676	16	29911818	29914888	ENSMUSG00000042606	ENSMUSG00000042606	ENSMUSG00000042606	7	126653120	126656191	133	150		
6677	16	29892723	29907220	ENSMUSG00000059981	ENSMUSG00000059981	ENSMUSG00000059981	7	126656826	126675223	133	150		
6678	16	29825166	29845046	ENSMUSG00000030685	ENSMUSG00000030685	ENSMUSG00000030685	7	126720041	126736779	133	150		
6679	16	29819648	29838679	ENSMUSG00000046378	ENSMUSG00000046378	ENSMUSG00000046378	7	126737135	126740730	133	150		
6680	16	29790332	29818074	ENSMUSG00000030683	ENSMUSG00000030683	ENSMUSG00000030683	7	126741711	126761751	133	150		
6681	16	29777180	29782556	ENSMUSG00000030682	ENSMUSG00000030682	ENSMUSG00000030682	7	126767062	126771310	133	150		
6682	16	29739281	29766842	ENSMUSG00000030681	ENSMUSG00000030681	ENSMUSG00000030681	7	126778012	126805742	133	150		
6683	16	29734786	29738903	ENSMUSG00000030680	ENSMUSG00000030680	ENSMUSG00000030680	7	126806199	126808500	133	150		
6684	16	29731091	29733460	ENSMUSG00000045114	ENSMUSG00000045114	ENSMUSG00000045114	7	126809868	126811439	133	150		
6685	16	29725359	29729206	ENSMUSG00000030678	ENSMUSG00000030678	ENSMUSG00000030678	7	126813291	126817567	133	150		
6686	16	29697112	29700597	ENSMUSG00000049350	ENSMUSG00000049350	ENSMUSG00000049350	7	126841304	126843125	133	150		
6687	16	29661289	29664828	ENSMUSG00000045165	ENSMUSG00000045165	ENSMUSG00000045165	7	126882584	126885197	133	150		
6688	16	29597942	29616806	ENSMUSG00000030674	ENSMUSG00000030674	ENSMUSG00000030674	7	126899171	126900402	133	150		
6689	16	29582077	29589688	ENSMUSG00000051457	ENSMUSG00000051457	ENSMUSG00000051457	7	126924612	126928965	133	150		
6690	16	30271670	30274128	ENSMUSG00000042502	ENSMUSG00000042502	ENSMUSG00000042502	7	126982808	126987148	133	151		
6691	16	30275925	30288587	ENSMUSG00000042492	ENSMUSG00000042492	ENSMUSG00000042492	7	126988613	126999616	133	151		
6692	16	30293622	30296813	ENSMUSG00000030672	ENSMUSG00000030672	ENSMUSG00000030672	7	127004009	127005343	133	151		
6693	16	30296958	30301672	ENSMUSG00000000486	ENSMUSG00000000486	ENSMUSG00000000486	7	127005906	127009379	133	151		
6694	16	30314556	30318928	ENSMUSG00000045598	ENSMUSG00000045598	ENSMUSG00000045598	7	127024591	127029025	133	151		
6695	16	30326437	30337416	ENSMUSG00000054716	ENSMUSG00000054716	ENSMUSG00000054716	7	127035674	127045949	133	151		
6696	16	30342522	30348874	ENSMUSG00000042462	ENSMUSG00000042462	ENSMUSG00000042462	7	127048112	127051842	133	151		
6697	16	30363203	30364549	ENSMUSG00000049091	ENSMUSG00000049091	ENSMUSG00000049091	7	127063709	127065067	133	151		
6698	16	30391551	30441772	ENSMUSG00000030830	ENSMUSG00000030830	ENSMUSG00000030830	7	127087558	127124876	133	151		
6699	16	30442829	30445411	ENSMUSG00000047371	ENSMUSG00000047371	ENSMUSG00000047371	7	127133945	127136532	133	151		
6700	16	30497795	30504591	ENSMUSG00000045757	ENSMUSG00000045757	ENSMUSG00000045757	7	127194816	127197970	133	151		
6701	16	30488554	30491556	ENSMUSG00000045251	ENSMUSG00000045251	ENSMUSG00000045251	7	127210115	127213175	133	151		
6702	16	30522203	30529183	ENSMUSG00000048921	ENSMUSG00000048921	ENSMUSG00000048921	7	127233286	127240269	133	151		
6703	16	30569742	30575234	ENSMUSG00000030822	ENSMUSG00000030822	ENSMUSG00000030822	7	127262762	127267906	133	151		
6704	16	30578744	30589631	ENSMUSG00000042423	ENSMUSG00000042423	ENSMUSG00000042423	7	127276804	127282032	133	151		
6705	16	30622886	30663100	ENSMUSG00000053877	ENSMUSG00000053877	ENSMUSG00000053877	7	127310729	127351914	133	151		
6706	16	30697279	30705990	ENSMUSG00000045639	ENSMUSG00000045639	ENSMUSG00000045639	7	127322795	127405581	133	151		
6707	16	30667238	30676183	ENSMUSG00000030815	ENSMUSG00000030815	ENSMUSG00000030815	7	127364552	127374453	133	151		
6708	16	30676254	30681043	ENSMUSG00000057176	ENSMUSG00000057176	ENSMUSG00000057176	7	127373532	127379324	133	151		
6709	16	30681131	30694037	ENSMUSG00000030816	ENSMUSG00000030816	ENSMUSG00000030816	7	127379928	127394740	133	151		
6710	16	30815429	30822381	ENSMUSG00000042340	ENSMUSG00000042340	ENSMUSG00000042340	7	127503884	127509333	133	151		
6711	16	30841893	30867598	ENSMUSG00000030811	ENSMUSG00000030811	ENSMUSG00000030811	7	127537923	127560076	133	151		
6712	16	30867916	30873757	ENSMUSG00000043964	ENSMUSG00000043964	ENSMUSG00000043964	7	127560965	127566153	133	151		
6713	16	30876116	30903482	ENSMUSG00000042308	ENSMUSG00000042308	ENSMUSG00000042308	7	127577245	127590624	133	151		
6714	16	30904033	30907972	ENSMUSG00000042289	ENSMUSG00000042289	ENSMUSG00000042289	7	127591777	127594949	133	151		
6715	16	30910869	30929276	ENSMUSG00000030806	ENSMUSG00000030806	ENSMUSG00000030806	7	127598364	127615447	133	151		
6716	16	30979673	30993005	ENSMUSG00000049728	ENSMUSG00000049728	ENSMUSG00000049728	7	127656318	127667964	133	151		
6717	16	30993263	31002327	ENSMUSG00000049739	ENSMUSG00000049739	ENSMUSG00000049739	7	127668849	127677144	133	151		
6718	16	31009956	31013551	ENSMUSG00000030804	ENSMUSG00000030804	ENSMUSG00000030804	7	127684211	127686765	133	151		
6719	16	31027251	31031611	ENSMUSG00000030802	ENSMUSG00000030802	ENSMUSG00000030802	7	127695251	127699757	133	151		
6720	16	31036494	31050181	ENSMUSG00000030801	ENSMUSG00000030801	ENSMUSG00000030801	7	127703670	127716980	133	151		
6721	16	31050284	31054320	ENSMUSG00000030800	ENSMUSG00000030800	ENSMUSG00000030800	7	127716871	127721030	133	151		
6722	16	31057750	31068888	ENSMUSG00000070371	ENSMUSG00000070371	ENSMUSG00000070371	7	127723787	127737596	133	151		
6723	16	31120310	31121512	ENSMUSG00000030793	ENSMUSG00000030793	ENSMUSG00000030793	7	127783251	127784895	133	151		
6724	16	31132916	31144011	ENSMUSG00000042828	ENSMUSG00000042828	ENSMUSG00000042828	7	127795960	127802540	133	151		
6725	16	31180512	31251207	ENSMUSG00000030786	ENSMUSG00000030786	ENSMUSG00000030786	7	127853827	127918264	133	151		
6726	16	31274010	31301819	ENSMUSG00000030789	ENSMUSG00000030789	ENSMUSG00000030789	7	127920710	127941804	133	151		
6727	16	31312134	31345327	ENSMUSG00000070369	ENSMUSG00000070369	ENSMUSG00000070369	7	127965115	127996645	133	151		
6728	16	31346556	31347222	ENSMUSG00000030785	ENSMUSG00000030785	ENSMUSG00000030785	7	127996583	127997514	133	151		
6729	16	31377818	31385711	ENSMUSG00000042178	ENSMUSG00000042178	ENSMUSG00000042178	7	128028505	128036248	133	151		
6730	16	31390993	31396780	ENSMUSG00000030782	ENSMUSG00000030782	ENSMUSG00000030782	7	128038019	128044859	133	151		
6731	16	31401940	31409590	ENSMUSG00000030781	ENSMUSG00000030781	ENSMUSG00000030781	7	128056831	128063577	133	151		
6732	16	31408316	31427207	ENSMUSG00000030780	ENSMUSG00000030780	ENSMUSG00000030780	7	128062525	128089260	133	151		
6733	10	121249330	121292212	ENSMUSG00000030844	ENSMUSG00000030844	ENSMUSG00000030844	7	128164773	128209320	134	152		
6734	10	121322970	121346531	ENSMUSG00000030846	ENSMUSG00000030846	ENSMUSG00000030846	7	128233450	128252437	134	152		
6735	10	121400872	121427321	ENSMUSG00000030847	ENSMUSG00000030847	ENSMUSG00000030847	7	128314764	128338129	134	152		
6736	10	121475599	121578648	ENSMUSG00000042105	ENSMUSG00000042105	ENSMUSG00000042105	7	128402513	128487584	134	152		
6737	10	121578962	121622256	ENSMUSG00000048170	ENSMUSG00000048170	ENSMUSG00000048170	7	128487589	128531577	134	152		
6738	10	121642213	121691648	ENSMUSG00000055319	ENSMUSG00000055319	ENSMUSG00000055319	7	128536129	128574939	134	152		
6739	10	122253081	122339353	ENSMUSG00000070366	ENSMUSG00000070366	ENSMUSG00000070366	7	129098710	129170679	134	152		
6740	10	122600685	122659026	ENSMUSG00000042055	ENSMUSG00000042055	ENSMUSG00000042055	7	129383077	129426123	134	152		
6741	10	123227846	123347907	ENSMUSG00000030849	ENSMUSG00000030849	ENSMUSG00000030849	7	129954867	130057386	134	152		
6742	10	123489929	123677541	ENSMUSG00000030850	ENSMUSG00000030850	ENSMUSG00000030850	7	130182641	130310687	134	152		
6743	10	123706593	123724722	ENSMUSG00000040331	ENSMUSG00000040331	ENSMUSG00000040331	7	130323675	130338529	134	152		
6744	10	123738699	124004047	ENSMUSG00000030852	ENSMUSG00000030852	ENSMUSG00000030852	7	130368632	130555930	134	152		
6745	10	124020811	124087666	ENSMUSG00000040298	ENSMUSG00000040298	ENSMUSG00000040298	7	130565217	130581614	134	152		
6746	10	124124202	124181857	ENSMUSG00000040268	ENSMUSG00000040268	ENSMUSG00000040268	7	130657046	130702631	134	152		
6747	10	124211031	124264414	ENSMUSG00000006205	ENSMUSG00000006205	ENSMUSG00000006205	7	130727378	130776801	134	152		
6748	10	124310171	124393242	ENSMUSG00000047517	ENSMUSG00000047517	ENSMUSG00000047517	7	130823224	130912776	134	152		
6749	10	124447218	124449332	ENSMUSG00000013668	ENSMUSG00000013668	ENSMUSG00000013668	7	130928861	130937462	134	152		
6750	10	124470811	124541947	ENSMUSG00000006204	ENSMUSG00000006204	ENSMUSG00000006204	7	130965550	131042095	134	152		
6751	10	124581655	124600299	ENSMUSG00000040205	ENSMUSG00000040205	ENSMUSG00000040205	7	131099702	131113440	134	152		
6752	10	124729546	124739896	ENSMUSG00000063179	ENSMUSG00000063179	ENSMUSG00000063179	7	131162294	131178987	134	152		
6753	10	124758522	124807817	ENSMUSG00000030861	ENSMUSG00000030861	ENSMUSG00000030861	7	131201807	131237354	134	152		
6754	10	124885557	124887237	ENSMUSG00000040148	ENSMUSG00000040148	ENSMUSG00000040148	7	131334111	131336073	134	152		
6755	10	124897628	124900178	ENSMUSG00000050100	ENSMUSG00000050100	ENSMUSG00000050100	7	131339921	131347272	134	152		
6756	10	124903783	124914876	ENSMUSG00000066979	ENSMUSG00000066979	ENSMUSG00000066979	7	131351858	131359873	134	152		
6757	10	125415861	125444113	ENSMUSG00000040125	ENSMUSG00000040125	ENSMUSG00000040125	7	131804803	131823976	134	152		
6758	10	125495142	125641490	ENSMUSG00000030862	ENSMUSG00000030862	ENSMUSG00000030862	7	131881159	131993082	134	152		
6759	10	125757174	125841960	ENSMUSG00000030930	ENSMUSG00000030930	ENSMUSG00000030930	7	132074123	132155057	134	152		
6760	10	126075866	126097495	ENSMUSG00000030934	ENSMUSG00000030934	ENSMUSG00000030934	7	132395969	132414732	134	152		
6761	10	126140394	126292700	ENSMUSG00000030946	ENSMUSG00000030946	ENSMUSG00000030946	7	132448986	132544762	134	152		
6762	10	126297853	126422920	ENSMUSG00000030956	ENSMUSG00000030956	ENSMUSG00000030956	7	132550427	132651539	134	152		
6763	10	126437396	126470429	ENSMUSG00000030960	ENSMUSG00000030960	ENSMUSG00000030960	7	132665800	132690990	134	152		
6764	10	126480375	126515226	ENSMUSG00000030965	ENSMUSG00000030965	ENSMUSG00000030965	7	132697587	132721847	134	152		
6765	10	126620682	126666748	ENSMUSG00000030967	ENSMUSG00000030967	ENSMUSG00000030967	7	132787965	132822294	134	152		
6766	10	127334253	127361703	ENSMUSG00000030976	ENSMUSG00000030976	ENSMUSG00000030976	7	133425367	133440501	134	152		
6767	10	127398074	127442702	ENSMUSG00000039990	ENSMUSG00000039990	ENSMUSG00000039990	7	133476049	133511316	134	152		
6768	10	127445012	127454380	ENSMUSG00000030981	ENSMUSG00000030981	ENSMUSG00000030981	7	133512613	133518404	134	152		
6769	10	127467175	127501760	ENSMUSG00000030979	ENSMUSG00000030979	ENSMUSG00000030979	7	133524673	133547638	134	152		
6770	10	127502105	127532254	ENSMUSG00000030983	ENSMUSG00000030983	ENSMUSG00000030983	7	133547680	133559487	134	152		
6771	10	127514899	127559874	ENSMUSG00000030986	ENSMUSG00000030986	ENSMUSG00000030986	7	133559355	133615146	134	152		
6772	10	127575120	127688149	ENSMUSG00000053111	ENSMUSG00000053111	ENSMUSG00000053111	7	133615204	133719875	134	152		
6773	10	127690940	128067014	ENSMUSG00000054555	ENSMUSG00000054555	ENSMUSG00000054555	7	133721547	134063220	134	152		
6774	10	128103568	128200000	ENSMUSG00000030994	ENSMUSG00000030994	ENSMUSG00000030994	7	134104618	134188144	134	152		
6775	10	128584013	129140764	ENSMUSG00000058325	ENSMUSG00000058325	ENSMUSG00000058325	7	134562672	135011989	134	152		
6776	10	128823680	128884412	ENSMUSG00000073805	ENSMUSG00000073805	ENSMUSG00000073805	7	134737449	134757142	134	152		
6777	10	129425504	129427216	ENSMUSG00000048377	ENSMUSG00000048377	ENSMUSG00000048377	7	135248651	135251959	134	152		
6778	10	129566095	129581201	ENSMUSG00000050866	ENSMUSG00000050866	ENSMUSG00000050866	7	135351067	135366985	134	152		
6779	10	129595315	129774155	ENSMUSG00000041836	ENSMUSG00000041836	ENSMUSG00000041836	7	135376167	135524637	134	152		
6780	10	131155481	131455352	ENSMUSG00000054612	ENSMUSG00000054612	ENSMUSG00000054612	7	136732967	136968609	134	152		
6781	10	131523537	131652095	ENSMUSG00000010476	ENSMUSG00000010476	ENSMUSG00000010476	7	137032064	137152788	134	152		
6782	10	131824653	131872775	ENSMUSG00000031068	ENSMUSG00000031068	ENSMUSG00000031068	7	137275991	137306937	134	152		
6783	10	132780644	132999974	ENSMUSG00000045309	ENSMUSG00000045309	ENSMUSG00000045309	7	138098133	138232643	134	152		
6784	10	133597950	133620027	ENSMUSG00000041769	ENSMUSG00000041769	ENSMUSG00000041769	7	138684702	138721397	134	152		
6785	10	133768303	133848302	ENSMUSG00000056856	ENSMUSG00000056856	ENSMUSG00000056856	7	138779073	138865185	134	152		
6786	10	133850394	133869256	ENSMUSG00000025478	ENSMUSG00000025478	ENSMUSG00000025478	7	138937347	138953085	134	152		
6787	10	133870986	133971467	ENSMUSG00000015981	ENSMUSG00000015981	ENSMUSG00000015981	7	138954959	139039793	134	152		
6788	10	133995694	134043412	ENSMUSG00000015980	ENSMUSG00000015980	ENSMUSG00000015980	7	139064308	139094299	134	152		
6789	10	134060694	134081324	ENSMUSG00000060260	ENSMUSG00000060260	ENSMUSG00000060260	7	139099839	139107491	134	152		
6790	10	134201314	134446969	ENSMUSG00000025477	ENSMUSG00000025477	ENSMUSG00000025477	7	139240430	139430965	134	152		
6791	10	134448290	134449546	ENSMUSG00000041309	ENSMUSG00000041309	ENSMUSG00000041309	7	139430619	139434113	134	152		
6792	10	134471887	134521226	ENSMUSG00000070357	ENSMUSG00000070357	ENSMUSG00000070357	7	139454516	139458432	134	152		
6793	10	134582822	134586108	ENSMUSG00000049571	ENSMUSG00000049571	ENSMUSG00000049571	7	139490220	139535135	134	152		
6794	10	134734423	134795169	ENSMUSG00000025475	ENSMUSG00000025475	ENSMUSG00000025475	7	139685616	139728271	134	152		
6795	10	134823961	134889906	ENSMUSG00000066129	ENSMUSG00000066129	ENSMUSG00000066129	7	139746158	139781318	134	152		
6796	10	134893768	134895052	ENSMUSG00000047751	ENSMUSG00000047751	ENSMUSG00000047751	7	139795142	139796426	134	152		
6797	10	134925898	134940362	ENSMUSG00000025473	ENSMUSG00000025473	ENSMUSG00000025473	7	139830258	139843855	134	152		
6798	10	134943125	134966475	ENSMUSG00000025474	ENSMUSG00000025474	ENSMUSG00000025474	7	139847316	139887586	134	152		
6799	10	134972435	134976656	ENSMUSG00000025470	ENSMUSG00000025470	ENSMUSG00000025470	7	139887774	139890769	134	152		
6800	10	134988922	135000408	ENSMUSG00000025468	ENSMUSG00000025468	ENSMUSG00000025468	7	139921207	139933868	134	152		
6801	10	135010640	135016177	ENSMUSG00000025467	ENSMUSG00000025467	ENSMUSG00000025467	7	139944728	139948523	134	152		
6802	10	135018654	135021519	ENSMUSG00000025466	ENSMUSG00000025466	ENSMUSG00000025466	7	139949831	139953726	134	152		
6803	10	135025974	135037183	ENSMUSG00000025465	ENSMUSG00000025465	ENSMUSG00000025465	7	139957032	139967776	134	152		
6804	10	135057690	135084813	ENSMUSG00000039018	ENSMUSG00000039018	ENSMUSG00000039018	7	139988975	140001935	134	152		
6805	10	135119724	135161789	ENSMUSG00000025461	ENSMUSG00000025461	ENSMUSG00000025461	7	140069601	140082456	134	152		
6806	10	135190857	135202610	ENSMUSG00000025479	ENSMUSG00000025479	ENSMUSG00000025479	7	140615152	140626301	134	152		
6807	10	135218364	135229036	ENSMUSG00000025480	ENSMUSG00000025480	ENSMUSG00000025480	7	140628553	140639173	134	152		
6808	11	183080	184573	ENSMUSG00000038801	ENSMUSG00000038801	ENSMUSG00000038801	7	140696897	140697952	135	153		
6809	11	186738	190258	ENSMUSG00000025482	ENSMUSG00000025482	ENSMUSG00000025482	7	140699125	140702334	135	153		
6810	11	192926	197428	ENSMUSG00000025484	ENSMUSG00000025484	ENSMUSG00000025484	7	140704731	140707577	135	153		
6811	11	198530	205113	ENSMUSG00000025485	ENSMUSG00000025485	ENSMUSG00000025485	7	140708717	140715051	135	153		
6812	11	205031	226361	ENSMUSG00000025486	ENSMUSG00000025486	ENSMUSG00000025486	7	140714990	140733629	135	153		
6813	11	226975	242983	ENSMUSG00000025487	ENSMUSG00000025487	ENSMUSG00000025487	7	140733714	140749962	135	153		
6814	11	268570	275304	ENSMUSG00000038745	ENSMUSG00000038745	ENSMUSG00000038745	7	140772378	140779520	135	153		
6815	11	280739	285678	ENSMUSG00000062031	ENSMUSG00000062031	ENSMUSG00000062031	7	140792901	140798978	135	153		
6816	11	288203	289526	ENSMUSG00000025489	ENSMUSG00000025489	ENSMUSG00000025489	7	140800283	140801589	135	153		
6817	11	359804	372117	ENSMUSG00000055629	ENSMUSG00000055629	ENSMUSG00000055629	7	140912594	140923439	135	153		
6818	11	384217	394908	ENSMUSG00000054065	ENSMUSG00000054065	ENSMUSG00000054065	7	140931098	140941829	135	153		
6819	11	395903	407397	ENSMUSG00000025494	ENSMUSG00000025494	ENSMUSG00000025494	7	140942658	140945249	135	153		
6820	11	407938	431996	ENSMUSG00000054662	ENSMUSG00000054662	ENSMUSG00000054662	7	140952545	140959994	135	153		
6821	11	440280	481399	ENSMUSG00000025495	ENSMUSG00000025495	ENSMUSG00000025495	7	140982606	141007474	135	153		
6822	11	484513	497273	ENSMUSG00000038650	ENSMUSG00000038650	ENSMUSG00000038650	7	141011642	141020009	135	153		

6823	11	523286	525572	ENSMUSG00000025499	ENSMUSG00000025499	ENSMUSG00000025499	7	141042250	141045314	135	153		
6824	11	530668	544911	ENSMUSG00000038637	ENSMUSG00000038637	ENSMUSG00000038637	7	141045496	141061369	135	153		
6825	11	550404	553645	ENSMUSG00000038618	ENSMUSG00000038618	ENSMUSG00000038618	7	141067153	141069978	135	153		
6826	11	566486	602220	ENSMUSG00000038611	ENSMUSG00000038611	ENSMUSG00000038611	7	141108182	141114071	135	153		
6827	11	602591	605652	ENSMUSG00000025498	ENSMUSG00000025498	ENSMUSG00000025498	7	141114504	141117743	135	153		
6828	11	607253	616078	ENSMUSG00000025497	ENSMUSG00000025497	ENSMUSG00000025497	7	141120406	141128106	135	153		
6829	11	616313	617173	ENSMUSG00000038580	ENSMUSG00000038580	ENSMUSG00000038580	7	141129651	141130453	135	153		
6830	11	627305	630703	ENSMUSG00000025496	ENSMUSG00000025496	ENSMUSG00000025496	7	141143350	141147783	135	153		
6831	11	634225	685047	ENSMUSG00000058886	ENSMUSG00000058886	ENSMUSG00000058886	7	141148505	141178890	135	153		
6832	11	685643	694127	ENSMUSG00000025505	ENSMUSG00000025505	ENSMUSG00000025505	7	141184634	141188133	135	153		
6833	11	696113	717726	ENSMUSG00000025504	ENSMUSG00000025504	ENSMUSG00000025504	7	141190276	141214338	135	153		
6834	11	737432	755023	ENSMUSG00000025503	ENSMUSG00000025503	ENSMUSG00000025503	7	141243519	141254287	135	153		
6835	11	777104	780123	ENSMUSG00000060240	ENSMUSG00000060240	ENSMUSG00000060240	7	141277769	141280740	135	153		
6836	11	780478	786221	ENSMUSG00000019082	ENSMUSG00000019082	ENSMUSG00000019082	7	141281070	141289147	135	153		
6837	11	789185	794388	ENSMUSG00000025507	ENSMUSG00000025507	ENSMUSG00000025507	7	141289437	141295312	135	153		
6838	11	799936	802876	ENSMUSG00000025508	ENSMUSG00000025508	ENSMUSG00000025508	7	141298976	141302905	135	153		
6839	11	808902	815216	ENSMUSG00000025509	ENSMUSG00000025509	ENSMUSG00000025509	7	141306526	141311412	135	153		
6840	11	819297	821985	ENSMUSG00000048200	ENSMUSG00000048200	ENSMUSG00000048200	7	141312414	141317932	135	153		
6841	11	822961	828830	ENSMUSG00000025510	ENSMUSG00000025510	ENSMUSG00000025510	7	141320142	141322788	135	153		
6842	11	832824	857116	ENSMUSG00000025511	ENSMUSG00000025511	ENSMUSG00000025511	7	141326560	141344742	135	153		
6843	11	858417	901461	ENSMUSG00000025512	ENSMUSG00000025512	ENSMUSG00000025512	7	141344671	141391119	135	153		
6844	11	915841	1002240	ENSMUSG00000002957	ENSMUSG00000002957	ENSMUSG00000002957	7	141413558	141484333	135	153		
6845	11	1008091	1026706	ENSMUSG00000048191	ENSMUSG00000048191	ENSMUSG00000048191	7	141485369	141506628	135	153		
6846	11	1064902	1083653	ENSMUSG00000025515	ENSMUSG00000025515	ENSMUSG00000025515	7	141541684	141555936	135	153		
6847	11	1141627	1152360	ENSMUSG00000037974	ENSMUSG00000037974	ENSMUSG00000037974	7	141600511	141619634	135	153		
6848	11	1254468	1287415	ENSMUSG00000025139	ENSMUSG00000025139	ENSMUSG00000025139	7	141690975	141711796	135	153		
6849	11	1388958	1439551	ENSMUSG00000053046	ENSMUSG00000053046	ENSMUSG00000053046	7	141759476	141811622	135	153		
6850	11	1447264	1458701	ENSMUSG00000025147	ENSMUSG00000025147	ENSMUSG00000025147	7	141817943	141870424	135	153		
6851	11	1533971	1543742	ENSMUSG00000037887	ENSMUSG00000037887	ENSMUSG00000037887	7	141890884	141904432	135	153		
6852	11	1710216	1728397	ENSMUSG00000045777	ENSMUSG00000045777	ENSMUSG00000045777	7	142135500	142181649	135	153		
6853	11	1730558	1741798	ENSMUSG00000007891	ENSMUSG00000007891	ENSMUSG00000007891	7	142185307	142197290	135	153		
6854	11	1812250	1815327	ENSMUSG00000031098	ENSMUSG00000031098	ENSMUSG00000031098	7	142244367	142249788	135	153		
6855	11	1816795	1819484	ENSMUSG00000031097	ENSMUSG00000031097	ENSMUSG00000031097	7	142251220	142253795	135	153		
6856	11	1830776	1870069	ENSMUSG00000018819	ENSMUSG00000018819	ENSMUSG00000018819	7	142270247	142303989	135	153		
6857	11	1866951	1867775	ENSMUSG00000043795	ENSMUSG00000043795	ENSMUSG00000043795	7	142300470	142316161	135	153		
6858	11	1900669	1916510	ENSMUSG00000061723	ENSMUSG00000061723	ENSMUSG00000061723	7	142308226	142325398	135	153		
6859	11	1972984	1975648	ENSMUSG00000073782	ENSMUSG00000073782	ENSMUSG00000073782	7	142386451	142386849	135	153		
6860	11	2106918	2116780	ENSMUSG00000048583	ENSMUSG00000048583	ENSMUSG00000048583	7	142460158	142468879	135	153		
6861	11	2141735	2149611	ENSMUSG00000000214	ENSMUSG00000000214	ENSMUSG00000000214	7	142702166	142709356	135	153		
6862	11	2279819	2295948	ENSMUSG00000000244	ENSMUSG00000000244	ENSMUSG00000000244	7	142814436	142828876	135	153		
6863	11	2363863	2374722	ENSMUSG00000037706	ENSMUSG00000037706	ENSMUSG00000037706	7	142862185	142877314	135	153		
6864	11	2378294	2381682	ENSMUSG00000045752	ENSMUSG00000045752	ENSMUSG00000045752	7	142878758	142880477	135	153		
6865	11	2382321	2400851	ENSMUSG00000009246	ENSMUSG00000009246	ENSMUSG00000009246	7	142881577	142904032	135	153		
6866	11	2422797	2826915	ENSMUSG00000009545	ENSMUSG00000009545	ENSMUSG00000009545	7	142916644	143236440	135	153		
6867	11	2861019	2863546	ENSMUSG00000037664	ENSMUSG00000037664	ENSMUSG00000037664	7	143267730	143270386	135	153		
6868	11	2877527	2903052	ENSMUSG00000000154	ENSMUSG00000000154	ENSMUSG00000000154	7	143283179	143308712	135	153		
6869	11	2906079	2907261	ENSMUSG00000010760	ENSMUSG00000010760	ENSMUSG00000010760	7	143310938	143311914	135	153		
6870	11	2922237	2970183	ENSMUSG00000059119	ENSMUSG00000059119	ENSMUSG00000059119	7	143322982	143349638	135	153		
6871	11	2978736	3025808	ENSMUSG00000010755	ENSMUSG00000010755	ENSMUSG00000010755	7	143367262	143402051	135	153		
6872	11	3064922	3143116	ENSMUSG00000037606	ENSMUSG00000037606	ENSMUSG00000037606	7	143498152	143551353	135	153		
6873	11	3205676	3206560	ENSMUSG00000048965	ENSMUSG00000048965	ENSMUSG00000048965	7	143587755	143593708	135	153		
6874	11	70841865	70890227	ENSMUSG00000031090	ENSMUSG00000031090	ENSMUSG00000031090	7	143604984	143632231	136	154		
6875	11	70823111	70837050	ENSMUSG00000058454	ENSMUSG00000058454	ENSMUSG00000058454	7	143632557	143657800	136	154		
6876	11	69991609	70185520	ENSMUSG00000037541	ENSMUSG00000037541	ENSMUSG00000037541	7	143985283	144230525	136	154		
6877	11	69922292	69960162	ENSMUSG00000031078	ENSMUSG00000031078	ENSMUSG00000031078	7	144245125	144280399	136	154		
6878	11	69726917	69731134	ENSMUSG00000031077	ENSMUSG00000031077	ENSMUSG00000031077	7	144387713	144391826	136	154		
6879	11	69602056	69713281	ENSMUSG00000031075	ENSMUSG00000031075	ENSMUSG00000031075	7	144398033	144547933	136	154		
6880	11	69333917	69343129	ENSMUSG00000031074	ENSMUSG00000031074	ENSMUSG00000031074	7	144648098	144652734	136	154		
6881	11	69296978	69299352	ENSMUSG00000050917	ENSMUSG00000050917	ENSMUSG00000050917	7	144670776	144674633	136	154		
6882	11	69222188	69228287	ENSMUSG00000031073	ENSMUSG00000031073	ENSMUSG00000031073	7	144705922	144710343	136	154		
6883	11	69189515	69199346	ENSMUSG00000031072	ENSMUSG00000031072	ENSMUSG00000031072	7	144724525	144730523	136	154		
6884	11	69165054	69178422	ENSMUSG00000070348	ENSMUSG00000070348	ENSMUSG00000070348	7	144739321	144749220	136	154		
6885	11	68572941	68612568	ENSMUSG00000048677	ENSMUSG00000048677	ENSMUSG00000048677	7	145063313	145093317	136	154		
6886	11	68529322	68537296	ENSMUSG00000031070	ENSMUSG00000031070	ENSMUSG00000031070	7	145110299	145118947	136	154		
6887	11	68504066	68505031	ENSMUSG00000051207	ENSMUSG00000051207	ENSMUSG00000051207	7	145124225	145133476	136	154		
6888	19	7067653	7245045	ENSMUSG00000005534	ENSMUSG00000005534	ENSMUSG00000005534	8	3155401	3279128	137	155		
6889	19	7410574	7443363	ENSMUSG00000004568	ENSMUSG00000004568	ENSMUSG00000004568	8	3393008	3456600	137	155		
6890	19	7447761	7459905	ENSMUSG00000069633	ENSMUSG00000069633	ENSMUSG00000069633	8	3457108	3467680	137	155		
6891	19	7468460	7479336	ENSMUSG00000040340	ENSMUSG00000040340	ENSMUSG00000040340	8	3470862	3487178	137	155		
6892	19	7490088	7491911	ENSMUSG00000047264	ENSMUSG00000047264	ENSMUSG00000047264	8	3493171	3497208	137	155		
6893	19	7493514	7504868	ENSMUSG00000004567	ENSMUSG00000004567	ENSMUSG00000004567	8	3500506	3515227	137	155		
6894	19	7505075	7532647	ENSMUSG00000004565	ENSMUSG00000004565	ENSMUSG00000004565	8	3515384	3544267	137	155		
6895	19	7566788	7588981	ENSMUSG00000044433	ENSMUSG00000044433	ENSMUSG00000044433	8	3587676	3609047	137	155		
6896	19	7590417	7600451	ENSMUSG00000019470	ENSMUSG00000019470	ENSMUSG00000019470	8	3608421	3621305	137	155		
6897	19	7602509	7604636	ENSMUSG00000004630	ENSMUSG00000004630	ENSMUSG00000004630	8	3623377	3625532	137	155		
6898	19	7608010	7618758	ENSMUSG00000004626	ENSMUSG00000004626	ENSMUSG00000004626	8	3631139	3642794	137	155		
6899	19	7639972	7641340	ENSMUSG00000012705	ENSMUSG00000012705	ENSMUSG00000012705	8	3655771	3659817	137	155		
6900	19	7647514	7650708	ENSMUSG00000013974	ENSMUSG00000013974	ENSMUSG00000013974	8	3665756	3669259	137	155		
6901	19	7651761	7653742	ENSMUSG00000040236	ENSMUSG00000040236	ENSMUSG00000040236	8	3676512	3681255	137	155		
6902	19	7659667	7673032	ENSMUSG00000005540	ENSMUSG00000005540	ENSMUSG00000005540	8	3681801	3694174	137	155		
6903	19	7801243	7835861	ENSMUSG00000011832	ENSMUSG00000011832	ENSMUSG00000011832	8	4183534	4206780	137	155		
6904	19	7859390	7872906	ENSMUSG00000046589	ENSMUSG00000046589	ENSMUSG00000046589	8	4226827	4236898	137	155		
6905	19	7874728	7885362	ENSMUSG00000002948	ENSMUSG00000002948	ENSMUSG00000002948	8	4238865	4251420	137	155		
6906	19	7895372	7897051	ENSMUSG00000048644	ENSMUSG00000048644	ENSMUSG00000048644	8	4257658	4259210	137	155		
6907	19	7897604	7914538	ENSMUSG00000002949	ENSMUSG00000002949	ENSMUSG00000002949	8	4259888	4275895	137	155		
6908	19	7933159	8415925	ENSMUSG00000040028	ENSMUSG00000040028	ENSMUSG00000040028	8	4288343	4324906	137	155		
6909	19	8023934	8028809	ENSMUSG00000023235	ENSMUSG00000023235	ENSMUSG00000023235	8	4349822	4359515	137	155		
6910	19	8180253	8233302	ENSMUSG00000008206	ENSMUSG00000008206	ENSMUSG00000008206	8	4493504	4525757	137	155		
6911	13	102494351	102517197	ENSMUSG00000023073	ENSMUSG00000023073	ENSMUSG00000023073	8	5085595	5105204	138	156		
6912	13	105940099	105985338	ENSMUSG00000001300	ENSMUSG00000001300	ENSMUSG00000001300	8	8620282	8660745	138	156		
6913	13	105992022	106018513	ENSMUSG00000040459	ENSMUSG00000040459	ENSMUSG00000040459	8	8681855	8690289	138	156		
6914	13	107657791	107665131	ENSMUSG00000049717	ENSMUSG00000049717	ENSMUSG00000049717	8	9969992	9976295	138	156		
6915	13	107668728	107684604	ENSMUSG00000040396	ENSMUSG00000040396	ENSMUSG00000040396	8	9977836	9990193	138	156		
6916	13	107720067	107758826	ENSMUSG00000031497	ENSMUSG00000031497	ENSMUSG00000031497	8	10006815	10035413	138	156		
6917	13	108046501	108658356	ENSMUSG00000039057	ENSMUSG00000039057	ENSMUSG00000039057	8	10272544	10569502	138	156		
6918	13	109204185	109236916	ENSMUSG00000038894	ENSMUSG00000038894	ENSMUSG00000038894	8	11004440	11008402	138	156		
6919	13	109599312	109757459	ENSMUSG00000031502	ENSMUSG00000031502	ENSMUSG00000031502	8	11198403	11312702	138	156		
6920	13	109757632	109963372	ENSMUSG00000031503	ENSMUSG00000031503	ENSMUSG00000031503	8	11354249	11448288	138	156		
6921	13	109973420	110012081	ENSMUSG00000031504	ENSMUSG00000031504	ENSMUSG00000031504	8	11453494	11478596	138	156		
6922	13	110066009	110090341	ENSMUSG00000031505	ENSMUSG00000031505	ENSMUSG00000031505	8	11497574	11513256	138	156		
6923	13	110091760	110156464	ENSMUSG00000056228	ENSMUSG00000056228	ENSMUSG00000056228	8	11513993	11550755	138	156		
6924	13	110165330	110171422	ENSMUSG00000045969	ENSMUSG00000045969	ENSMUSG00000045969	8	11556038	11563222	138	156		
6925	13	110328888	110365417	ENSMUSG00000031508	ENSMUSG00000031508	ENSMUSG00000031508	8	11611555	11635726	138	156		
6926	13	110766559	110794595	ENSMUSG00000031512	ENSMUSG00000031512	ENSMUSG00000031512	8	11840891	11855677	138	156		
6927	13	112078634	112137004	ENSMUSG00000010435	ENSMUSG00000010435	ENSMUSG00000010435	8	12573026	12600715	138	156		
6928	13	112187327	112290482	ENSMUSG00000000759	ENSMUSG00000000759	ENSMUSG00000000759	8	12614254	12672077	138	156		
6929	13	112392644	112589470	ENSMUSG00000031441	ENSMUSG00000031441	ENSMUSG00000031441	8	12806627	12865124	138	156		
6930	13	112604510	112799176	ENSMUSG00000031442	ENSMUSG00000031442	ENSMUSG00000031442	8	12915935	13020479	138	156		
6931	13	112808122	112822996	ENSMUSG00000031443	ENSMUSG00000031443	ENSMUSG00000031443	8	13026011	13035782	138	156		
6932	13	112825114	112851844	ENSMUSG00000031444	ENSMUSG00000031444	ENSMUSG00000031444	8	13037299	13055859	138	156		
6933	13	112860969	112874695	ENSMUSG00000031445	ENSMUSG00000031445	ENSMUSG00000031445	8	13060942	13075999	138	156		
6934	13	112879927	112910984	ENSMUSG00000038542	ENSMUSG00000038542	ENSMUSG00000038542	8	13077167	13104810	138	156		
6935	13	112999557	113025742	ENSMUSG00000031447	ENSMUSG00000031447	ENSMUSG00000031447	8	13159138	13175307	138	156		
6936	13	113026513	113066421	ENSMUSG00000038515	ENSMUSG00000038515	ENSMUSG00000038515	8	13176847	13200512	138	156		
6937	13	113158135	113193024	ENSMUSG00000038506	ENSMUSG00000038506	ENSMUSG00000038506	8	13255941	13281102	138	156		
6938	13	113193311	113252507	ENSMUSG00000038497	ENSMUSG00000038497	ENSMUSG00000038497	8	13287990	13322901	138	156		
6939	13	113351125	113360502	ENSMUSG00000031449	ENSMUSG00000031449	ENSMUSG00000031449	8	13386189	13396802	138	156		
6940	13	113369703	113373974	ENSMUSG00000031450	ENSMUSG00000031450	ENSMUSG00000031450	8	13405058	13417557	138	156		
6941	13	113599044	113651885	ENSMUSG00000038457	ENSMUSG00000038457	ENSMUSG00000038457	8	13435460	13461424	138	156		
6942	13	113546919	113590407	ENSMUSG00000031451	ENSMUSG00000031451	ENSMUSG00000031451	8	13465353	13494505	138	156		
6943	13	113765297	113916066	ENSMUSG00000031453	ENSMUSG00000031453	ENSMUSG00000031453	8	13566927	13677560	138	156		
6944	13	114018476	114056300	ENSMUSG00000038416	ENSMUSG00000038416	ENSMUSG00000038416	8	13757667	13781859	138	156		
6945	13	114065161	114089385	ENSMUSG00000038398	ENSMUSG00000038398	ENSMUSG00000038398	8	13785615	13798698	138	156		
6946	13	114098090	114110898	ENSMUSG00000047710	ENSMUSG00000047710	ENSMUSG00000047710	8	13869706	13880546	138	156		
6947	8	346808	409871	ENSMUSG00000038365	ENSMUSG00000038365	ENSMUSG00000038365	8	13907783	13940498	139	157		
6948	8	431647	484974	ENSMUSG00000050052	ENSMUSG00000050052	ENSMUSG00000050052	8	13951986	13974667	139	157		
6949	8	604201	671226	ENSMUSG00000051978	ENSMUSG00000051978	ENSMUSG00000051978	8	14028100	14078912	139	157		
6950	8	1436941	1637558	ENSMUSG00000047495	ENSMUSG00000047495	ENSMUSG00000047495	8	14531001	14847657	139	157		
6951	8	1759549	1894206	ENSMUSG00000071176	ENSMUSG00000071176	ENSMUSG00000071176	8	14911720	15001051	139	157		
6952	8	1909451	1942509	ENSMUSG00000055675	ENSMUSG00000055675	ENSMUSG00000055675	8	15027367	15033307	139	157		
6953	8	1980612	2080779	ENSMUSG00000031461	ENSMUSG00000031461	ENSMUSG00000031461	8	15057630	15133518	139	157		
6954	8	2782790	3254536	ENSMUSG00000060924	ENSMUSG00000060924	ENSMUSG00000060924	8	15895694	17534951	139	157		
6955	8	6251530	6488550	ENSMUSG00000039842	ENSMUSG00000039842	ENSMUSG00000039842	8	18595150	18803178	139	157		
6956	8	6344580	6408338	ENSMUSG00000031465	ENSMUSG00000031465	ENSMUSG00000031465	8	18691314	18741528	139	157		
6957	8	6553286	6604592	ENSMUSG00000031467	ENSMUSG00000031467	ENSMUSG00000031467	8	18846481	18884358	139	157		
6958	8	6656129	6680576	ENSMUSG00000039814	ENSMUSG00000039814	ENSMUSG00000039814	8	18933798	18950910	139	157		
6959	13	51334118	51338367	ENSMUSG00000017049	ENSMUSG00000017049	ENSMUSG00000017049	8	23435320	23439562	140	158		
6960	13	51406893	51447305	ENSMUSG00000006567	ENSMUSG00000006567	ENSMUSG00000006567	8	23459893	23525621	140	158		
6961	13	51537544	51599802	ENSMUSG00000037738	ENSMUSG00000037738	ENSMUSG00000037738	8	23539161	23590598	140	158		
6962	13	51604780	51631933	ENSMUSG00000031478	ENSMUSG00000031478	ENSMUSG00000031478	8	23593830	23631957	140	158		
6963	13	51927596	51948759	ENSMUSG00000037725	ENSMUSG00000037725	ENSMUSG00000037725	8	23633994	23651364	140	158		
6964	13	51884743	51922799	ENSMUSG00000031479	ENSMUSG00000031479	ENSMUSG00000031479	8	23658405	23684142	140	158		
6965	13	51849306	51878321	ENSMUSG00000031480	ENSMUSG00000031480	ENSMUSG00000031480	8	23692885	23726867	140	158		
6966	13	40261632	40284596	ENSMUSG00000031482	ENSMUSG00000031482	ENSMUSG00000031482	8	23841552	23864098	141	158	here	Sub blocks comes here
6967	13	40201432	40243309	ENSMUSG00000031533	ENSMUSG00000031533	ENSMUSG00000031533	8	23876927	23895210	141	158	sub blocks	2 -> 1
6968	8	42515913	42527272	ENSMUSG00000031534	ENSMUSG00000031534	ENSMUSG00000031534	8	23928159	23942400	142	159		
6969	8	42393173	42516225	ENSMUSG00000037656	ENSMUSG00000037656	ENSMUSG00000037656	8	23942333	24035157	142	159		
6970	8	42350744	42353832	ENSMUSG00000031535	ENSMUSG00000031535	ENSMUSG00000031535	8	24089588	24093092	142	159		
6971	8	42315131	42348482	ENSMUSG00000031536	ENSMUSG00000031536	ENSMUSG00000031536	8	24093675	24118966	142	159		
6972	8	42247986	42309122	ENSMUSG00000031537	ENSMUSG00000031537	ENSMUSG00000031537	8	24124758	24172077	142	159		
6973	8	42151912	42184351	ENSMUSG00000031538	ENSMUSG00000031538	ENSMUSG00000031538	8	24223292	24248389	142	159		
6974	8	42129748	42147857	ENSMUSG00000031539	ENSMUSG00000031539	ENSMUSG00000031539	8	24252900	24271172	142	159		
6975	8	41907430	42028635	ENSMUSG00000031540	ENSMUSG00000031540	ENSMUSG00000031540	8	24325084	24406187	142	159		
6976	8	41632000	41873437	ENSMUSG00000031543	ENSMUSG00000031543	ENSMUSG00000031543	8	24440681	24616041	142	159		
6977	8	41623128	41627168	ENSMUSG00000063672	ENSMUSG00000063672	ENSMUSG00000063672	8	24618816	24624183	142	159		
6978	8	41505925	41521427	ENSMUSG00000031546	ENSMUSG00000031546	ENSMUSG00000031546	8	24692170	24703204	142	159		
6979	8	41467238	41487650	ENSMUSG00000015341	ENSMUSG00000015341	ENSMUSG00000015341	8	24706898	24722600	142	159		
6980	8	41238640	41286149	ENSMUSG00000031548	ENSMUSG00000031548	ENSMUSG00000031548	8	24877063	24915179	142	159		
6981	8	40507270	40874500	ENSMUSG00000037492	ENSMUSG00000037492	ENSMUSG00000037492	8	25135208	25528648	142	159		
6982	8	40130176	40131950	ENSMUSG00000056313	ENSMUSG00000056313	ENSMUSG00000056313	8	25904121	25904441	142	159		
6983	8	39925875	39992269	ENSMUSG00000031549	ENSMUSG00000031549	ENSMUSG00000031549	8	25997999	26041878	142	159		
6984	8	39890545	39905120	ENSMUSG00000031551	ENSMUSG00000031551	ENSMUSG00000031551	8	26049686	26062554	142	159		
6985	8	39561299	39706644	ENSMUSG00000031552	ENSMUSG00000031552	ENSMUSG00000031552	8	26067792	26140300	142	159		
6986	8	39084325	39261585	ENSMUSG00000037437	ENSMUSG00000037437	ENSMUSG00000037437	8	26317781	26414328	142	159		
6987	8	38973662	39081934	ENSMUSG00000031555	ENSMUSG00000031555	ENSMUSG00000031555	8	26415178	26482450	142	159		
6988	8	38965484	38973198	ENSMUSG00000031556	ENSMUSG00000031556	ENSMUSG00000031556	8	26482798	26488803	142	159		
6989	8	38950862	38964868	ENSMUSG00000037406	ENSMUSG00000037406	ENSMUSG00000037406	8	26490929	26504426	142	159		
6990	8	38894605	38946456	ENSMUSG00000031557	ENSMUSG00000031557	ENSMUSG00000031557	8	26506385	26567277	142	159		
6991	8	38764258	38829702	ENSMUSG00000065954	ENSMUSG00000065954	ENSMUSG00000065954	8	26620097	26666830	142	159		
6992	8	38389406	38445296	ENSMUSG00000031565	ENSMUSG00000031565	ENSMUSG00000031565	8	26997826	27039466	142	159		
6993	8	38363182	38386197	ENSMUSG00000037363	ENSMUSG00000037363	ENSMUSG00000037363	8	27044040	27063032	142	159		
6994	8	38251717	38358947	ENSMUSG00000054823	ENSMUSG00000054823	ENSMUSG00000054823	8	27106166	27179942	142	159		
6995	8	38239813	38245815	ENSMUSG00000031570	ENSMUSG00000031570	ENSMUSG00000031570	8	27185629	27190223	142	159		
6996	8	38208628	38239436	ENSMUSG00000061313	ENSMUSG00000061313	ENSMUSG00000061313	8	27193204	27218712	142	159		
6997	8	38153469	38189966	ENSMUSG00000037316	ENSMUSG00000037316	ENSMUSG00000037316	8	27230081	27250754	142	159		
6998	8	38140034	38152995	ENSMUSG00000037296	ENSMUSG00000037296	ENSMUSG00000037296	8	27251136	27269520	142	159		
6999	8	38119383	38127757	ENSMUSG00000031574	ENSMUSG00000031574	ENSMUSG00000031574	8	27274058	27281527	142	159		
7000	8	38082223	38116351	ENSMUSG00000031575	ENSMUSG00000031575	ENSMUSG00000031575	8	27281764	27306308	142	159		
7001	8	43114803	43173866	ENSMUSG00000037260	ENSMUSG00000037260	ENSMUSG00000037260	8	27409999	27442256	143	160	here	Our tool has ignored this small block and merged the neighboring blocks.
7002	8	43077849	43097177	ENSMUSG00000037251	ENSMUSG00000037251	ENSMUSG00000037251	8	27446149	27459666	143	160	Merge blocks seperated by smaller blocks	2 -> 5
7003	8	43030599	43060080	ENSMUSG00000015994	ENSMUSG00000015994	ENSMUSG00000015994	8	27464267	27481146	143	160		
7004	8	42871190	42994084	ENSMUSG00000037234	ENSMUSG00000037234	ENSMUSG00000037234	8	27496216	27584679	143	160		
7005	8	42810975	42817631	ENSMUSG00000037214	ENSMUSG00000037214	ENSMUSG00000037214	8	27623757	27628579	143	160		
7006	8	37672471	37675350	ENSMUSG00000065951	ENSMUSG00000065951	ENSMUSG00000065951	8	28363721	28446030	142	161		
7007	8	37713307	37734476	ENSMUSG00000031483	ENSMUSG00000031483	ENSMUSG00000031483	8	28489795	28505373	142	161		
7008	8	37738833	37756443	ENSMUSG00000031485	ENSMUSG00000031485	ENSMUSG00000031485	8	28508535	28522235	142	161		
7009	8	37773932	37820648	ENSMUSG00000031486	ENSMUSG00000031486	ENSMUSG00000031486	8	28551777	28589358	142	161		
7010	8	37820432	37826512	ENSMUSG00000031487	ENSMUSG00000031487	ENSMUSG00000031487	8	28589300	28594570	142	161		
7011	8	37910962	37916804	ENSMUSG00000039720	ENSMUSG00000039720	ENSMUSG00000039720	8	28663395	28668483	142	161		
7012	8	37939673	37943341	ENSMUSG00000031489	ENSMUSG00000031489	ENSMUSG00000031489	8	28691712	28695524	142	161		
7013	8	38007185	38037033	ENSMUSG00000031490	ENSMUSG00000031490	ENSMUSG00000031490	8	28726338	28742604	142	161		
7014	8	42671719	42711366	ENSMUSG00000031492	ENSMUSG00000031492	ENSMUSG00000031492	8	28834647	28865665	143	162		
7015	8	42726939	42742776	ENSMUSG00000031491	ENSMUSG00000031491	ENSMUSG00000031491	8	28869148	28879638	143	162		
7016	8	35212845	35767623	ENSMUSG00000063626	ENSMUSG00000063626	ENSMUSG00000063626	8	30118704	30685274	out-of-place	163		
7017	8	33568398	33577043	ENSMUSG00000039661	ENSMUSG00000039661	ENSMUSG00000039661	8	32555597	32562982	142	163		

7018	8	33524815	33544185	ENSMUSG00000039328	ENSMUSG00000039328	ENSMUSG00000039328	8	32577781	32597417	142	163		
7019	8	33476025	33490198	ENSMUSG00000031577	ENSMUSG00000031577	ENSMUSG00000031577	8	32616262	32628598	142	163		
7020	8	33462247	33478317	ENSMUSG00000031578	ENSMUSG00000031578	ENSMUSG00000031578	8	32625403	32634659	142	163		
7021	8	33347890	33438512	ENSMUSG00000046152	ENSMUSG00000046152	ENSMUSG00000046152	8	32653313	32727418	142	163		
7022	8	32525371	32741531	ENSMUSG00000062991	ENSMUSG00000062991	ENSMUSG00000062991	8	33283963	33384138	142	163		
7023	8	31010320	31150818	ENSMUSG00000031583	ENSMUSG00000031583	ENSMUSG00000031583	8	34701594	34851407	142	163		
7024	8	30972863	31010773	ENSMUSG00000049184	ENSMUSG00000049184	ENSMUSG00000049184	8	34851559	34853468	142	163		
7025	8	30808604	30826075	ENSMUSG00000009628	ENSMUSG00000009628	ENSMUSG00000009628	8	35036479	35051520	142	163		
7026	8	30762683	30789894	ENSMUSG00000009630	ENSMUSG00000009630	ENSMUSG00000009630	8	35065560	35085738	142	163		
7027	8	30721241	30744064	ENSMUSG00000052906	ENSMUSG00000052906	ENSMUSG00000052906	8	35085521	35107911	142	163		
7028	8	30656549	30704894	ENSMUSG00000031584	ENSMUSG00000031584	ENSMUSG00000031584	8	35119173	35164097	142	163		
7029	8	30555422	30635260	ENSMUSG00000031585	ENSMUSG00000031585	ENSMUSG00000031585	8	35197977	35243099	142	163		
7030	8	30361486	30524569	ENSMUSG00000031586	ENSMUSG00000031586	ENSMUSG00000031586	8	35269875	35395740	142	163		
7031	8	30133355	30160601	ENSMUSG00000031516	ENSMUSG00000031516	ENSMUSG00000031516	8	35558940	35576975	142	163		
7032	8	30109001	30109990	ENSMUSG00000071113	ENSMUSG00000071113	ENSMUSG00000071113	8	35592261	35593235	142	163		
7033	8	30072492	30085128	ENSMUSG00000031513	ENSMUSG00000031513	ENSMUSG00000031513	8	35604089	35615256	142	163		
7034	8	30040184	30060201	ENSMUSG00000031532	ENSMUSG00000031532	ENSMUSG00000031532	8	35623093	35639336	142	163		
7035	8	29249539	29264104	ENSMUSG00000031530	ENSMUSG00000031530	ENSMUSG00000031530	8	36276127	36288411	142	163		
7036	8	9450832	9671804	ENSMUSG00000031529	ENSMUSG00000031529	ENSMUSG00000031529	8	36297696	36434207	144	164		
7037	8	9032916	9045616	ENSMUSG00000046794	ENSMUSG00000046794	ENSMUSG00000046794	8	36845196	36856632	144	164		
7038	8	8897764	8928139	ENSMUSG00000031527	ENSMUSG00000031527	ENSMUSG00000031527	8	36935756	36964052	144	164		
7039	8	8680942	8787978	ENSMUSG00000070056	ENSMUSG00000070056	ENSMUSG00000070056	8	37056315	37147964	144	164		
7040	8	8597319	8598197	ENSMUSG00000055976	ENSMUSG00000055976	ENSMUSG00000055976	8	37293959	37294849	144	164		
7041	8	8212676	8276599	ENSMUSG00000050271	ENSMUSG00000050271	ENSMUSG00000050271	8	37563378	37616061	144	164		
7042	8	12623790	12640008	ENSMUSG00000039633	ENSMUSG00000039633	ENSMUSG00000039633	8	37685960	37697772	145	165		
7043	8	12914144	12928045	ENSMUSG00000039620	ENSMUSG00000039620	ENSMUSG00000039620	8	37926065	37981473	145	165		
7044	8	12985243	13416766	ENSMUSG00000031523	ENSMUSG00000031523	ENSMUSG00000031523	8	38038050	38201243	145	165		
7045	8	13468723	13470167	ENSMUSG00000074384	ENSMUSG00000074384	ENSMUSG00000074384	8	38462099	38463251	145	165		
7046	8	13991744	15140219	ENSMUSG00000039539	ENSMUSG00000039539	ENSMUSG00000039539	8	38991779	39421574	145	165		
7047	8	15442147	15666366	ENSMUSG00000039530	ENSMUSG00000039530	ENSMUSG00000039530	8	40474409	40665143	145	165		
7048	8	16009761	16094595	ENSMUSG00000025044	ENSMUSG00000025044	ENSMUSG00000025044	8	41080502	41131646	145	165		
7049	8	16894049	16904061	ENSMUSG00000031603	ENSMUSG00000031603	ENSMUSG00000031603	8	41777983	41785770	145	165		
7050	8	16929118	17024524	ENSMUSG00000039478	ENSMUSG00000039478	ENSMUSG00000039478	8	41834732	41853220	145	165		
7051	8	17086667	17122305	ENSMUSG00000039470	ENSMUSG00000039470	ENSMUSG00000039470	8	41922635	41984533	145	165		
7052	8	17148851	17197438	ENSMUSG00000031600	ENSMUSG00000031600	ENSMUSG00000031600	8	42010926	42043841	145	165		
7053	8	17199923	17315207	ENSMUSG00000039431	ENSMUSG00000039431	ENSMUSG00000039431	8	42050368	42107833	145	165		
7054	8	17445206	17472296	ENSMUSG00000031596	ENSMUSG00000031596	ENSMUSG00000031596	8	42397761	42415357	145	165		
7055	8	17478986	17544896	ENSMUSG00000031595	ENSMUSG00000031595	ENSMUSG00000031595	8	42425058	42489599	145	165		
7056	8	17545584	17702666	ENSMUSG00000045636	ENSMUSG00000045636	ENSMUSG00000045636	8	42489736	42613484	145	165		
7057	8	17766169	17797693	ENSMUSG00000031594	ENSMUSG00000031594	ENSMUSG00000031594	8	42690254	42713970	145	165		
7058	8	17824845	17929534	ENSMUSG00000031592	ENSMUSG00000031592	ENSMUSG00000031592	8	42738590	42831161	145	165		
7059	8	17958215	17986757	ENSMUSG00000031591	ENSMUSG00000031591	ENSMUSG00000031591	8	42839468	42873466	145	165		
7060	4	189297592	189305643	ENSMUSG00000031651	ENSMUSG00000031651	ENSMUSG00000031651	8	44628624	44640303	146	166		
7061	4	189160856	189163193	ENSMUSG00000051176	ENSMUSG00000051176	ENSMUSG00000051176	8	44793885	44805797	146	166		
7062	4	187745932	187881981	ENSMUSG00000070047	ENSMUSG00000070047	ENSMUSG00000070047	8	46449043	46526497	146	166		
7063	4	187691803	187713531	ENSMUSG00000054764	ENSMUSG00000054764	ENSMUSG00000054764	8	46568027	46587323	146	166		
7064	4	187424272	187446928	ENSMUSG00000031645	ENSMUSG00000031645	ENSMUSG00000031645	8	46739987	46760848	146	166		
7065	4	187385660	187416618	ENSMUSG00000031640	ENSMUSG00000031640	ENSMUSG00000031640	8	46768488	46807283	146	166		
7066	4	187263225	187330132	ENSMUSG00000070044	ENSMUSG00000070044	ENSMUSG00000070044	8	46837897	46881001	146	166		
7067	4	187227303	187243244	ENSMUSG00000031639	ENSMUSG00000031639	ENSMUSG00000031639	8	46894482	46909356	146	166		
7068	4	186743592	187114516	ENSMUSG00000031626	ENSMUSG00000031626	ENSMUSG00000031626	8	47261925	47326721	146	166		
7069	4	186603336	186629879	ENSMUSG00000071104	ENSMUSG00000071104	ENSMUSG00000071104	8	47433466	47442962	146	166		
7070	4	186590139	186594261	ENSMUSG00000071103	ENSMUSG00000071103	ENSMUSG00000071103	8	47452423	47474069	146	166		
7071	4	186558482	186584117	ENSMUSG00000031634	ENSMUSG00000031634	ENSMUSG00000031634	8	47474345	47495771	146	166		
7072	4	186554834	186558383	ENSMUSG00000050914	ENSMUSG00000050914	ENSMUSG00000050914	8	47495719	47498667	146	166		
7073	4	186525212	186554047	ENSMUSG00000031637	ENSMUSG00000031637	ENSMUSG00000031637	8	47498125	47526203	146	166		
7074	4	186362385	186522103	ENSMUSG00000038291	ENSMUSG00000038291	ENSMUSG00000038291	8	47532089	47615519	146	166		
7075	4	186327175	186362176	ENSMUSG00000031631	ENSMUSG00000031631	ENSMUSG00000031631	8	47662506	47694401	146	166		
7076	4	186301392	186305418	ENSMUSG00000031633	ENSMUSG00000031633	ENSMUSG00000031633	8	47705991	47709847	146	166		
7077	4	186177077	186178920	ENSMUSG00000047171	ENSMUSG00000047171	ENSMUSG00000047171	8	47790856	47793488	146	166		
7078	4	185913744	185984209	ENSMUSG00000018796	ENSMUSG00000018796	ENSMUSG00000018796	8	47969859	48034867	146	166		
7079	4	185852229	185904203	ENSMUSG00000031629	ENSMUSG00000031629	ENSMUSG00000031629	8	48050872	48078824	146	166		
7080	4	185807896	185853106	ENSMUSG00000038225	ENSMUSG00000038225	ENSMUSG00000038225	8	48074396	48116017	146	166		
7081	4	185785845	185807623	ENSMUSG00000031628	ENSMUSG00000031628	ENSMUSG00000031628	8	48116235	48137523	146	166		
7082	4	185545909	185632697	ENSMUSG00000031627	ENSMUSG00000031627	ENSMUSG00000031627	8	48238594	48346283	146	166		
7083	4	185246854	185376013	ENSMUSG00000038173	ENSMUSG00000038173	ENSMUSG00000038173	8	48485742	48593712	146	166		
7084	4	185063503	185175835	ENSMUSG00000038143	ENSMUSG00000038143	ENSMUSG00000038143	8	48684773	48788179	146	166		
7085	4	184817435	184871738	ENSMUSG00000038102	ENSMUSG00000038102	ENSMUSG00000038102	8	48988943	49032287	146	166		
7086	4	184663214	184669243	ENSMUSG00000063049	ENSMUSG00000063049	ENSMUSG00000063049	8	49165995	49173975	146	166		
7087	4	184602794	184606340	ENSMUSG00000038069	ENSMUSG00000038069	ENSMUSG00000038069	8	49208265	49212716	146	166		
7088	4	184351762	184476893	ENSMUSG00000031563	ENSMUSG00000031563	ENSMUSG00000031563	8	49326412	49489389	146	166		
7089	4	184048238	184075624	ENSMUSG00000031562	ENSMUSG00000031562	ENSMUSG00000031562	8	49608814	49640477	146	166		
7090	4	183482168	183958495	ENSMUSG00000031561	ENSMUSG00000031561	ENSMUSG00000031561	8	49727263	50173458	146	166		
7091	4	178588918	178600585	ENSMUSG00000031521	ENSMUSG00000031521	ENSMUSG00000031521	8	55010424	55022120	146	166		
7092	4	178468022	178520994	ENSMUSG00000039396	ENSMUSG00000039396	ENSMUSG00000039396	8	55085565	55137694	146	166		
7093	4	177841685	177950889	ENSMUSG00000031520	ENSMUSG00000031520	ENSMUSG00000031520	8	55576304	55685794	146	166		
7094	4	177478109	177487338	ENSMUSG00000054408	ENSMUSG00000054408	ENSMUSG00000054408	8	56019131	56028680	146	166		
7095	4	177371825	177427367	ENSMUSG00000031519	ENSMUSG00000031519	ENSMUSG00000031519	8	56049028	56086533	146	166		
7096	4	177342719	177353816	ENSMUSG00000031518	ENSMUSG00000031518	ENSMUSG00000031518	8	56099478	56108797	146	166		
7097	4	177224126	177340959	ENSMUSG00000039375	ENSMUSG00000039375	ENSMUSG00000039375	8	56128319	56223065	146	166		
7098	4	176791083	177160642	ENSMUSG00000031517	ENSMUSG00000031517	ENSMUSG00000031517	8	56453541	56559569	146	166		
7099	4	176076134	176135905	ENSMUSG00000046258	ENSMUSG00000046258	ENSMUSG00000046258	8	58763054	58799090	146	166		
7100	4	175800505	175987040	ENSMUSG00000038257	ENSMUSG00000038257	ENSMUSG00000038257	8	58833029	59017513	146	166		
7101	4	175647958	175680213	ENSMUSG00000031613	ENSMUSG00000031613	ENSMUSG00000031613	8	59186735	59213185	146	166		
7102	4	175456796	175478056	ENSMUSG00000038215	ENSMUSG00000038215	ENSMUSG00000038215	8	59423664	59442708	146	166		
7103	4	175394698	175420818	ENSMUSG00000038206	ENSMUSG00000038206	ENSMUSG00000038206	8	59443276	59486081	146	166		
7104	4	174684918	174687015	ENSMUSG00000038193	ENSMUSG00000038193	ENSMUSG00000038193	8	60213125	60216659	146	166		
7105	4	174545875	174557192	ENSMUSG00000031610	ENSMUSG00000031610	ENSMUSG00000031610	8	60348065	60369727	146	166		
7106	4	174528669	174535257	ENSMUSG00000031609	ENSMUSG00000031609	ENSMUSG00000031609	8	60374849	60380004	146	166		
7107	4	174326505	174481692	ENSMUSG00000031608	ENSMUSG00000031608	ENSMUSG00000031608	8	60416711	60545145	146	166		
7108	4	172972307	174197826	ENSMUSG00000074341	ENSMUSG00000074341	ENSMUSG00000074341	8	60668367	60850472	146	166		
7109	4	171217951	171247940	ENSMUSG00000057228	ENSMUSG00000057228	ENSMUSG00000057228	8	63398672	63437819	146	166		
7110	4	171144328	171184004	ENSMUSG00000031647	ENSMUSG00000031647	ENSMUSG00000031647	8	63525021	63564891	146	166		
7111	4	170887191	170915679	ENSMUSG00000038005	ENSMUSG00000038005	ENSMUSG00000038005	8	63782593	63799713	146	166		
7112	4	170778297	170878731	ENSMUSG00000004319	ENSMUSG00000004319	ENSMUSG00000004319	8	63805159	63868033	146	166		
7113	4	170551121	170770307	ENSMUSG00000031644	ENSMUSG00000031644	ENSMUSG00000031644	8	63885337	64023481	146	166		
7114	4	170251985	170428740	ENSMUSG00000031642	ENSMUSG00000031642	ENSMUSG00000031642	8	64116351	64288213	146	166		
7115	4	170145319	170167997	ENSMUSG00000031641	ENSMUSG00000031641	ENSMUSG00000031641	8	64379926	64395628	146	166		
7116	4	169374022	169476478	ENSMUSG00000037921	ENSMUSG00000037921	ENSMUSG00000037921	8	64832743	64929273	146	166		
7117	4	169250310	169407564	ENSMUSG00000031635	ENSMUSG00000031635	ENSMUSG00000031635	8	64949884	64988951	146	166		
7118	4	167891167	168392275	ENSMUSG00000054162	ENSMUSG00000054162	ENSMUSG00000054162	8	65843992	66249295	146	166		
7119	4	167013860	167244443	ENSMUSG00000053626	ENSMUSG00000053626	ENSMUSG00000053626	8	66907123	67098185	146	166		
7120	4	166468268	166483674	ENSMUSG00000031604	ENSMUSG00000031604	ENSMUSG00000031604	8	67610337	67625770	146	166		
7121	4	166348238	166463748	ENSMUSG00000031605	ENSMUSG00000031605	ENSMUSG00000031605	8	67635088	67778664	146	166		
7122	4	166218550	166253431	ENSMUSG00000025521	ENSMUSG00000025521	ENSMUSG00000025521	8	67876174	67898022	146	166		
7123	4	166199711	166201156	ENSMUSG00000071089	ENSMUSG00000071089	ENSMUSG00000071089	8	67910640	67916635	146	166		
7124	4	166180040	166182115	ENSMUSG00000053490	ENSMUSG00000053490	ENSMUSG00000053490	8	67929042	67947566	146	166		
7125	4	164668499	164994733	ENSMUSG00000036469	ENSMUSG00000036469	ENSMUSG00000036469	8	68808303	69398949	146	166		
7126	4	164635063	164661107	ENSMUSG00000025591	ENSMUSG00000025591	ENSMUSG00000025591	8	69405664	69413159	146	166		
7127	4	164612456	164614336	ENSMUSG00000025519	ENSMUSG00000025519	ENSMUSG00000025519	8	69440748	69447324	146	166		
7128	4	164484541	164492534	ENSMUSG00000044014	ENSMUSG00000044014	ENSMUSG00000044014	8	69608954	69617083	146	166		
7129	4	164464567	164473198	ENSMUSG00000036437	ENSMUSG00000036437	ENSMUSG00000036437	8	69632837	69635097	146	166		
7130	8	18700155	18915476	ENSMUSG00000030465	ENSMUSG00000030465	ENSMUSG00000030465	8	70625036	70903563	147	167		
7131	8	19215408	19298009	ENSMUSG00000053886	ENSMUSG00000053886	ENSMUSG00000053886	8	71205732	71276567	147	167		
7132	8	19305953	19584552	ENSMUSG00000036356	ENSMUSG00000036356	ENSMUSG00000036356	8	71285783	71664124	147	167		
7133	8	19719277	19753864	ENSMUSG00000031864	ENSMUSG00000031864	ENSMUSG00000031864	8	71723003	71758397	147	167		
7134	8	19841232	19867912	ENSMUSG00000015568	ENSMUSG00000015568	ENSMUSG00000015568	8	71809547	71836437	147	167		
7135	8	20046646	20084997	ENSMUSG00000036330	ENSMUSG00000036330	ENSMUSG00000036330	8	71966697	72018211	147	167		
7136	8	20098984	20123485	ENSMUSG00000006273	ENSMUSG00000006273	ENSMUSG00000006273	8	72017755	72042699	147	167		
7137	8	20147956	20205754	ENSMUSG00000036306	ENSMUSG00000036306	ENSMUSG00000036306	8	72064492	72069942	147	167		
7138	19	19617012	19635479	ENSMUSG00000031862	ENSMUSG00000031862	ENSMUSG00000031862	8	72720223	72736737	148	168		
7139	19	19601285	19615417	ENSMUSG00000036246	ENSMUSG00000036246	ENSMUSG00000036246	8	72737676	72750861	148	168		
7140	19	19595478	19600739	ENSMUSG00000031861	ENSMUSG00000031861	ENSMUSG00000031861	8	72751418	72760091	148	168		
7141	19	19533524	19590462	ENSMUSG00000031860	ENSMUSG00000031860	ENSMUSG00000031860	8	72761684	72801280	148	168		
7142	19	19510057	19518466	ENSMUSG00000044006	ENSMUSG00000044006	ENSMUSG00000044006	8	72810264	72816340	148	168		
7143	19	19500720	19509176	ENSMUSG00000048967	ENSMUSG00000048967	ENSMUSG00000048967	8	72816777	72831701	148	168		
7144	19	19487639	19500007	ENSMUSG00000036199	ENSMUSG00000036199	ENSMUSG00000036199	8	72823169	72831547	148	168		
7145	19	19484230	19487838	ENSMUSG00000047654	ENSMUSG00000047654	ENSMUSG00000047654	8	72831173	72832505	148	168		
7146	19	19237469	19478787	ENSMUSG00000036180	ENSMUSG00000036180	ENSMUSG00000036180	8	72837459	72925353	148	168		
7147	19	19300882	19339992	ENSMUSG00000031858	ENSMUSG00000031858	ENSMUSG00000031858	8	72947571	72971674	148	168		
7148	19	19248323	19292307	ENSMUSG00000011306	ENSMUSG00000011306	ENSMUSG00000011306	8	72972033	73001318	148	168		
7149	19	19226557	19245178	ENSMUSG00000007594	ENSMUSG00000007594	ENSMUSG00000007594	8	73013409	73017510	148	168		
7150	19	19183838	19224040	ENSMUSG00000002341	ENSMUSG00000002341	ENSMUSG00000002341	8	73022071	73049799	148	168		
7151	19	19164008	19173678	ENSMUSG00000036120	ENSMUSG00000036120	ENSMUSG00000036120	8	73059795	73068186	148	168		
7152	19	19173734	19175155	ENSMUSG00000071078	ENSMUSG00000071078	ENSMUSG00000071078	8	73060322	73062205	148	168		
7153	19	19117379	19163937	ENSMUSG00000002345	ENSMUSG00000002345	ENSMUSG00000002345	8	73068698	73096477	148	168		
7154	19	19091430	19110287	ENSMUSG00000002342	ENSMUSG00000002342	ENSMUSG00000002342	8	73103292	73111128	148	168		
7155	19	19067934	19084697	ENSMUSG00000002346	ENSMUSG00000002346	ENSMUSG00000002346	8	73113329	73123193	148	168		
7156	19	19005538	19029985	ENSMUSG00000002343	ENSMUSG00000002343	ENSMUSG00000002343	8	73149184	73163455	148	168		
7157	19	18966175	19005625	ENSMUSG00000036054	ENSMUSG00000036054	ENSMUSG00000036054	8	73165672	73189578	148	168		
7158	19	18901252	18910791	ENSMUSG00000003573	ENSMUSG00000003573	ENSMUSG00000003573	8	73212039	73223346	148	168		
7159	19	18891494	18900436	ENSMUSG00000057788	ENSMUSG00000057788	ENSMUSG00000057788	8	73221855	73231441	148	168		
7160	19	18871323	18891206	ENSMUSG00000055681	ENSMUSG00000055681	ENSMUSG00000055681	8	73231758	73241979	148	168		
7161	19	18803747	18840038	ENSMUSG00000058301	ENSMUSG00000058301	ENSMUSG00000058301	8	73260513	73282262	148	168		
7162	19	18754584	18763114	ENSMUSG00000031849	ENSMUSG00000031849	ENSMUSG00000031849	8	73302547	73311055	148	168		
7163	19	18655488	18749762	ENSMUSG00000003575	ENSMUSG00000003575	ENSMUSG00000003575	8	73311344	73368568	148	168		
7164	19	18608838	18642302	ENSMUSG00000055707	ENSMUSG00000055707	ENSMUSG00000055707	8	73380427	73384645	148	168		
7165	19	18584691	18592834	ENSMUSG00000035964	ENSMUSG00000035964	ENSMUSG00000035964	8	73412854	73416347	148	168		
7166	19	18565047	18578660	ENSMUSG00000007888	ENSMUSG00000007888	ENSMUSG00000007888	8	73422145	73433070	148	168		
7167	19	18560608	18564146	ENSMUSG00000058833	ENSMUSG00000058833	ENSMUSG00000058833	8	73433285	73435734	148	168		
7168	19	18529598	18541176	ENSMUSG00000055553	ENSMUSG00000055553	ENSMUSG00000055553	8	73442387	73629768	148	168		
7169	19	18503568	18515383	ENSMUSG00000019428	ENSMUSG00000019428	ENSMUSG00000019428	8	73456794	73464316	148	168		
7170	19	18414475	18493918	ENSMUSG00000070002	ENSMUSG00000070002	ENSMUSG00000070002	8	73468694	73521845	148	168		
7171	19	18406620	18409941	ENSMUSG00000019139	ENSMUSG00000019139	ENSMUSG00000019139	8	73523470	73526279	148	168		
7172	19	18391136	18406459	ENSMUSG00000070003	ENSMUSG00000070003	ENSMUSG00000070003	8	73526479	73537262	148	168		
7173	19	18362955	18369415	ENSMUSG00000049988	ENSMUSG00000049988	ENSMUSG00000049988	8	73545833	73549839	148	168		
7174	19	18357968	18360987	ENSMUSG00000038508	ENSMUSG00000038508	ENSMUSG00000038508	8	73558380	73561084	148	168		
7175	19	18312450	18335428	ENSMUSG00000056204	ENSMUSG00000056204	ENSMUSG00000056204	8	73578066	73588637	148	168		
7176	19	18278720	18294958	ENSMUSG00000031848	ENSMUSG00000031848	ENSMUSG00000031848	8	73605544	73607440	148	168		
7177	19	18252251	18253294	ENSMUSG00000071076	ENSMUSG00000071076	ENSMUSG00000071076	8	73628047	73629072	148	168		
7178	19	18228909	18246235	ENSMUSG00000035586	ENSMUSG00000035586	ENSMUSG00000035586	8	73638301	73646556	148	168		
7179	19	18182010	18227229	ENSMUSG00000031842	ENSMUSG00000031842	ENSMUSG00000031842	8	73653515	73679803	148	168		
7180	19	18168611	18175839	ENSMUSG00000031840	ENSMUSG00000031840	ENSMUSG00000031840	8	73684845	73687077	148	168		
7181	19	18165092	18168756	ENSMUSG00000035559	ENSMUSG00000035559	ENSMUSG00000035559	8	73687640	73689919	148	168		
7182	19	18145579	18149927	ENSMUSG00000031838	ENSMUSG00000031838	ENSMUSG00000031838	8	73691763	73695652	148	168		
7183	19	18125016	18142343	ENSMUSG00000031834	ENSMUSG00000031834	ENSMUSG00000031834	8	73697168	73705691	148	168		
7184	19	18069605	18123494	ENSMUSG00000031833	ENSMUSG00000031833	ENSMUSG00000031833	8	73711091	73725935	148	168		
7185	19	18031701	18058697	ENSMUSG00000000791	ENSMUSG00000000791	ENSMUSG00000000791	8	73737483	73750411	148	168		
7186	19	17980120	17985909	ENSMUSG00000002910	ENSMUSG00000002910	ENSMUSG00000002910	8	73764127	73768709	148	168		
7187	19	17945647	17970254	ENSMUSG00000002908	ENSMUSG00000002908	ENSMUSG00000002908	8	73772145	73784574	148	168		
7188	19	17908217	17915783	ENSMUSG00000007721	ENSMUSG00000007721	ENSMUSG00000007721	8	73797217	73802486	148	168		
7189	19	17843782	17865897	ENSMUSG00000000792	ENSMUSG00000000792	ENSMUSG00000000792	8	73811881	73821746	148	168		
7190	19	17691303	17706324	ENSMUSG00000019261	ENSMUSG00000019261	ENSMUSG00000019261	8	73834971	73846515	148	168		
7191	19	17021573	17047343	ENSMUSG00000035439	ENSMUSG00000035439	ENSMUSG00000035439	8	74180114	74201365	148	169	here	This inversion was ignored by our tool
7192	19	17073466	17185093	ENSMUSG00000004677	ENSMUSG00000004677	ENSMUSG00000004677	8	74219286	74288962	148	169	inversion ignored by ST	1 -> 2
7193	19	17187168	17191638	ENSMUSG00000002395	ENSMUSG00000002395	ENSMUSG00000002395	8	74296224	74298698	148	169		
7194	19	17198013	17201023	ENSMUSG00000002396	ENSMUSG00000002396	ENSMUSG00000002396	8	74300342	74302147	148	169		
7195	19	17203694	17217151	ENSMUSG00000002393	ENSMUSG00000002393	ENSMUSG00000002393	8	74303116	74310656	148	169		
7196	19	17221849	17236544	ENSMUSG00000034911	ENSMUSG00000034911	ENSMUSG00000034911	8	74314359	74324774	148	169		
7197	19	17239232	17251162	ENSMUSG00000031820	ENSMUSG00000031820	ENSMUSG00000031820	8	74325857	74333534	148	169		
7198	19	17253681	17259454	ENSMUSG00000046295	ENSMUSG00000046295	ENSMUSG00000046295	8	74335018	74339531	148	169		
7199	19	17263941	17275234	ENSMUSG00000007950	ENSMUSG00000007950	ENSMUSG00000007950	8	74386172	74390971	148	169		
7200	19	17277477	17278652	ENSMUSG00000034880	ENSMUSG00000034880	ENSMUSG00000034880	8	74393948	74394734	148	169		
7201	19	17281350	17292098	ENSMUSG00000074247	ENSMUSG00000074247	ENSMUSG00000074247	8	74401026	74403587	148	169		
7202	19	17295034	17306638	ENSMUSG00000034863	ENSMUSG00000034863	ENSMUSG00000034863	8	74405012	74415022	148	169		
7203	19	17309395	17313803	ENSMUSG00000007610	ENSMUSG00000007610	ENSMUSG00000007610	8	74417092	74422389	148	169		
7204	19	17323264	17349159	ENSMUSG00000034845	ENSMUSG00000034845	ENSMUSG00000034845	8	74426756	74440741	148	169		
7205	19	17374761	17377457	ENSMUSG00000046718	ENSMUSG00000046718	ENSMUSG00000046718	8	74463253	74466426	148	169		
7206	19	17391853	17397141	ENSMUSG00000031813	ENSMUSG00000031813	ENSMUSG00000031813	8	74471919	74477015	148	169		
7207	19	17408453	17420118	ENSMUSG00000043664	ENSMUSG00000043664	ENSMUSG00000043664	8	74483228	74487567	148	169		
7208	19	17427236	17432725	ENSMUSG00000034829	ENSMUSG00000034829	ENSMUSG00000034829	8	74489544	74495590	148	169		
7209	19	17442350	17477977	ENSMUSG00000031808	ENSMUSG00000031808	ENSMUSG00000031808	8	74497916	74515697	148	169		
7210	19	17483432	17493095	ENSMUSG00000031807	ENSMUSG00000031807	ENSMUSG00000031807	8	74521173	74525256	148	169		
7211	19	17495110	17525454	ENSMUSG00000043243	ENSMUSG00000043243	ENSMUSG00000043243	8	74531340	74535738	148	169		
7212	19	17527511	17554965	ENSMUSG00000034807	ENSMUSG00000034807	ENSMUSG00000034807	8	74539987	74553900	148	169		

7213	19	17573167	17647872	ENSMUSG00000034799	ENSMUSG00000034799	ENSMUSG00000034799	8	74558412	74600636	148	169		
7214	19	17788680	17819800	ENSMUSG00000031805	ENSMUSG00000031805	ENSMUSG00000031805	8	74607312	74619563	148	169		
7215	19	17766658	17784860	ENSMUSG00000031803	ENSMUSG00000031803	ENSMUSG00000031803	8	74620727	74630781	148	169		
7216	19	17726920	17760371	ENSMUSG00000070000	ENSMUSG00000070000	ENSMUSG00000070000	8	74637625	74650296	148	169		
7217	19	16083467	16106037	ENSMUSG00000003037	ENSMUSG00000003037	ENSMUSG00000003037	8	75090189	75109679	148	170	here	This is also a no orientation block as the previous block, and so is merged with the top block by ST.
7218	19	16115501	16130344	ENSMUSG00000062007	ENSMUSG00000062007	ENSMUSG00000062007	8	75118657	75129947	148	170	Blocks without orientation merged with the neighboring block	0 -> 1
7219	19	16133179	16145286	ENSMUSG00000074240	ENSMUSG00000074240	ENSMUSG00000074240	8	75134451	75141963	148	170		
7220	19	16169731	16207149	ENSMUSG00000003033	ENSMUSG00000003033	ENSMUSG00000003033	8	75169121	75186368	148	170		
7221	19	16296648	16299337	ENSMUSG00000055148	ENSMUSG00000055148	ENSMUSG00000055148	8	75248022	75250338	148	170		
7222	19	16333408	16443762	ENSMUSG00000006276	ENSMUSG00000006276	ENSMUSG00000006276	8	75270370	75329222	148	170		
7223	19	16450888	16468003	ENSMUSG00000019732	ENSMUSG00000019732	ENSMUSG00000019732	8	75353541	75372767	148	170		
7224	19	16468224	16493160	ENSMUSG00000052794	ENSMUSG00000052794	ENSMUSG00000052794	8	75372930	75389990	148	170		
7225	19	16489710	16514248	ENSMUSG00000052488	ENSMUSG00000052488	ENSMUSG00000052488	8	75389473	75404130	148	170		
7226	19	16523584	16544193	ENSMUSG00000019731	ENSMUSG00000019731	ENSMUSG00000019731	8	75410672	75421351	148	170		
7227	19	16546718	16600015	ENSMUSG00000045248	ENSMUSG00000045248	ENSMUSG00000045248	8	75423550	75477259	148	170		
7228	19	16632938	16660814	ENSMUSG00000031791	ENSMUSG00000031791	ENSMUSG00000031791	8	75501092	75516273	148	170		
7229	19	16703001	16789756	ENSMUSG00000048148	ENSMUSG00000048148	ENSMUSG00000048148	8	75549555	75604144	148	170		
7230	19	16801218	16852164	ENSMUSG00000031622	ENSMUSG00000031622	ENSMUSG00000031622	8	75619370	75653477	148	170		
7231	19	16860826	16870516	ENSMUSG00000050147	ENSMUSG00000050147	ENSMUSG00000050147	8	75657952	75659957	148	170		
7232	22	31998847	32646326	ENSMUSG00000004383	ENSMUSG00000004383	ENSMUSG00000004383	8	75710671	76248913	149	171		
7233	22	33792129	33816014	ENSMUSG00000031621	ENSMUSG00000031621	ENSMUSG00000031621	8	77769246	77789578	149	171		
7234	22	33983489	34021799	ENSMUSG00000034518	ENSMUSG00000034518	ENSMUSG00000034518	8	77889774	77928049	149	171		
7235	22	34107057	34120172	ENSMUSG00000005413	ENSMUSG00000005413	ENSMUSG00000005413	8	77989721	77996661	149	171		
7236	22	34126128	34150494	ENSMUSG00000005410	ENSMUSG00000005410	ENSMUSG00000005410	8	78005641	78024177	149	171		
7237	22	34267296	34279987	ENSMUSG00000034472	ENSMUSG00000034472	ENSMUSG00000034472	8	78110016	78120185	149	171		
7238	4	149219370	149582973	ENSMUSG00000031618	ENSMUSG00000031618	ENSMUSG00000031618	8	79804344	80138602	150	172		
7239	4	148872903	149213376	ENSMUSG00000037148	ENSMUSG00000037148	ENSMUSG00000037148	8	80146991	80413860	150	172		
7240	4	148778986	148824730	ENSMUSG00000037134	ENSMUSG00000037134	ENSMUSG00000037134	8	80445482	80477410	150	172		
7241	4	148758046	148776120	ENSMUSG00000031617	ENSMUSG00000031617	ENSMUSG00000031617	8	80492435	80506297	150	172		
7242	4	148621575	148685555	ENSMUSG00000031616	ENSMUSG00000031616	ENSMUSG00000031616	8	80559103	80620420	150	172		
7243	4	147847636	148086434	ENSMUSG00000037101	ENSMUSG00000037101	ENSMUSG00000037101	8	81109414	81290398	150	172		
7244	4	147779538	147783073	ENSMUSG00000031688	ENSMUSG00000031688	ENSMUSG00000031688	8	81329081	81332724	150	172		
7245	4	147395013	147662429	ENSMUSG00000031684	ENSMUSG00000031684	ENSMUSG00000031684	8	81405522	81629651	150	172		
7246	4	147316331	147330646	ENSMUSG00000031683	ENSMUSG00000031683	ENSMUSG00000031683	8	81702634	81717173	150	172		
7247	4	146901340	147079032	ENSMUSG00000071064	ENSMUSG00000071064	ENSMUSG00000071064	8	81956312	82082426	150	172		
7248	4	146622401	146699773	ENSMUSG00000031681	ENSMUSG00000031681	ENSMUSG00000031681	8	82234467	82295540	150	172		
7249	4	146135765	146238815	ENSMUSG00000036977	ENSMUSG00000036977	ENSMUSG00000036977	8	82607892	82673395	150	172		
7250	4	145786623	145879337	ENSMUSG00000064325	ENSMUSG00000064325	ENSMUSG00000064325	8	82866417	82954078	150	172		
7251	4	144718214	144841278	ENSMUSG00000042353	ENSMUSG00000042353	ENSMUSG00000042353	8	83506826	83591102	150	172		
7252	4	144477500	144610728	ENSMUSG00000031714	ENSMUSG00000031714	ENSMUSG00000031714	8	83660180	83776225	150	172		
7253	4	144325548	144362087	ENSMUSG00000038250	ENSMUSG00000038250	ENSMUSG00000038250	8	83876479	83910652	150	172		
7254	4	143168636	143603330	ENSMUSG00000037940	ENSMUSG00000037940	ENSMUSG00000037940	8	84610947	85018308	150	172		
7255	4	142777204	142874061	ENSMUSG00000031712	ENSMUSG00000031712	ENSMUSG00000031712	8	85227661	85240228	150	172		
7256	4	142006175	142274066	ENSMUSG00000047747	ENSMUSG00000047747	ENSMUSG00000047747	8	85753577	85884741	150	172		
7257	4	141762799	141896724	ENSMUSG00000031709	ENSMUSG00000031709	ENSMUSG00000031709	8	86056180	86163044	150	172		
7258	4	141700500	141709457	ENSMUSG00000031710	ENSMUSG00000031710	ENSMUSG00000031710	8	86180457	86188557	150	172		
7259	4	141664772	141694374	ENSMUSG00000035151	ENSMUSG00000035151	ENSMUSG00000035151	8	86202737	86222530	150	172		
7260	4	141592014	141638826	ENSMUSG00000035057	ENSMUSG00000035057	ENSMUSG00000035057	8	86238574	86269507	150	172		
7261	4	141529059	141568232	ENSMUSG00000002190	ENSMUSG00000002190	ENSMUSG00000002190	8	86279972	86318146	150	172		
7262	19	14537892	14543886	ENSMUSG00000033938	ENSMUSG00000033938	ENSMUSG00000033938	8	86456863	86461728	151	173		
7263	19	14486582	14490201	ENSMUSG00000005483	ENSMUSG00000005483	ENSMUSG00000005483	8	86498298	86502008	151	173		
7264	19	14449572	14467944	ENSMUSG00000019433	ENSMUSG00000019433	ENSMUSG00000019433	8	86542783	86554894	151	173		
7265	19	14444279	14447174	ENSMUSG00000019464	ENSMUSG00000019464	ENSMUSG00000019464	8	86556973	86562860	151	173		
7266	19	14412072	14443678	ENSMUSG00000057672	ENSMUSG00000057672	ENSMUSG00000057672	8	86560204	86584100	151	173		
7267	19	14380632	14391171	ENSMUSG00000005481	ENSMUSG00000005481	ENSMUSG00000005481	8	86605304	86613453	151	173		
7268	19	14353213	14380531	ENSMUSG00000002885	ENSMUSG00000002885	ENSMUSG00000002885	8	86613467	86631274	151	173		
7269	19	14119547	14177972	ENSMUSG00000013033	ENSMUSG00000013033	ENSMUSG00000013033	8	86809033	86829706	151	173		
7270	19	14091322	14108440	ENSMUSG00000005470	ENSMUSG00000005470	ENSMUSG00000005470	8	86845767	86860298	151	173		
7271	19	14053646	14057181	ENSMUSG00000046408	ENSMUSG00000046408	ENSMUSG00000046408	8	86891577	86894875	151	173		
7272	19	14044821	14046873	ENSMUSG00000074217	ENSMUSG00000074217	ENSMUSG00000074217	8	86900816	86901546	151	173		
7273	19	14025945	14027264	ENSMUSG00000047986	ENSMUSG00000047986	ENSMUSG00000047986	8	86911576	86919294	151	173		
7274	19	14003262	14025026	ENSMUSG00000005465	ENSMUSG00000005465	ENSMUSG00000005465	8	86920411	86932680	151	173		
7275	19	14000017	14002783	ENSMUSG00000045232	ENSMUSG00000045232	ENSMUSG00000045232	8	86933172	86935084	151	173		
7276	19	13933353	13978097	ENSMUSG00000031706	ENSMUSG00000031706	ENSMUSG00000031706	8	86956971	86987107	151	173		
7277	19	13924304	13933254	ENSMUSG00000037103	ENSMUSG00000037103	ENSMUSG00000037103	8	86987180	86994878	151	173		
7278	19	13904167	13910289	ENSMUSG00000012889	ENSMUSG00000012889	ENSMUSG00000012889	8	87016094	87022672	151	173		
7279	19	13878014	13902691	ENSMUSG00000036686	ENSMUSG00000036686	ENSMUSG00000036686	8	87022933	87037858	151	173		
7280	19	13854168	13877909	ENSMUSG00000008129	ENSMUSG00000008129	ENSMUSG00000008129	8	87038264	87062695	151	173		
7281	19	13849061	13852317	ENSMUSG00000056155	ENSMUSG00000056155	ENSMUSG00000056155	8	87063838	87066657	151	173		
7282	19	13767274	13804044	ENSMUSG00000035671	ENSMUSG00000035671	ENSMUSG00000035671	8	87100783	87127160	151	173		
7283	19	13746282	13750275	ENSMUSG00000019362	ENSMUSG00000019362	ENSMUSG00000019362	8	87136338	87139815	151	173		
7284	19	13736360	13746096	ENSMUSG00000004996	ENSMUSG00000004996	ENSMUSG00000004996	8	87140011	87147431	151	173		
7285	19	13703574	13735046	ENSMUSG00000004994	ENSMUSG00000004994	ENSMUSG00000004994	8	87148586	87154467	151	173		
7286	19	13179088	13478038	ENSMUSG00000034656	ENSMUSG00000034656	ENSMUSG00000034656	8	87305750	87530351	151	173		
7287	19	13122282	13126716	ENSMUSG00000053560	ENSMUSG00000053560	ENSMUSG00000053560	8	87552191	87552856	151	173		
7288	19	13090109	13112955	ENSMUSG00000001910	ENSMUSG00000001910	ENSMUSG00000001910	8	87560588	87577967	151	173		
7289	19	13076721	13088522	ENSMUSG00000001909	ENSMUSG00000001909	ENSMUSG00000001909	8	87579383	87589892	151	173		
7290	19	13070848	13074681	ENSMUSG00000034041	ENSMUSG00000034041	ENSMUSG00000034041	8	87591649	87594797	151	173		
7291	19	12996811	13066482	ENSMUSG00000001911	ENSMUSG00000001911	ENSMUSG00000001911	8	87603454	87663501	151	173		
7292	19	12941432	12946567	ENSMUSG00000053226	ENSMUSG00000053226	ENSMUSG00000053226	8	87705510	87712928	151	173		
7293	19	12925972	12929050	ENSMUSG00000033751	ENSMUSG00000033751	ENSMUSG00000033751	8	87722387	87725587	151	173		
7294	19	12917654	12925448	ENSMUSG00000003813	ENSMUSG00000003813	ENSMUSG00000003813	8	87724757	87730746	151	173		
7295	19	12910392	12916274	ENSMUSG00000003814	ENSMUSG00000003814	ENSMUSG00000003814	8	87731955	87736972	151	173		
7296	19	12894294	12905529	ENSMUSG00000003808	ENSMUSG00000003808	ENSMUSG00000003808	8	87747096	87759361	151	173		
7297	19	12870894	12890151	ENSMUSG00000003824	ENSMUSG00000003824	ENSMUSG00000003824	8	87762216	87777500	151	173		
7298	19	12862974	12871782	ENSMUSG00000003809	ENSMUSG00000003809	ENSMUSG00000003809	8	87776714	87783856	151	173		
7299	19	12856241	12859017	ENSMUSG00000054191	ENSMUSG00000054191	ENSMUSG00000054191	8	87792033	87795396	151	173		
7300	19	12847023	12853335	ENSMUSG00000003812	ENSMUSG00000003812	ENSMUSG00000003812	8	87798729	87801566	151	173		
7301	19	12810348	12846765	ENSMUSG00000053693	ENSMUSG00000053693	ENSMUSG00000053693	8	87802011	87827458	151	173		
7302	19	12797520	12807242	ENSMUSG00000048617	ENSMUSG00000048617	ENSMUSG00000048617	8	87837096	87846708	151	173		
7303	19	12778488	12785452	ENSMUSG00000052926	ENSMUSG00000052926	ENSMUSG00000052926	8	87847772	87856190	151	173		
7304	19	12763286	12765129	ENSMUSG00000052837	ENSMUSG00000052837	ENSMUSG00000052837	8	87867500	87868534	151	173		
7305	19	12734817	12747337	ENSMUSG00000052566	ENSMUSG00000052566	ENSMUSG00000052566	8	87880708	87893462	151	173		
7306	19	12724407	12730269	ENSMUSG00000052819	ENSMUSG00000052819	ENSMUSG00000052819	8	87897310	87904513	151	173		
7307	19	12709306	12720137	ENSMUSG00000052456	ENSMUSG00000052456	ENSMUSG00000052456	8	87908036	87915383	151	173		
7308	19	12702487	12706589	ENSMUSG00000041203	ENSMUSG00000041203	ENSMUSG00000041203	8	87916928	87920384	151	173		
7309	19	12672967	12694386	ENSMUSG00000031691	ENSMUSG00000031691	ENSMUSG00000031691	8	87927020	87947685	151	173		
7310	19	12661030	12668395	ENSMUSG00000008167	ENSMUSG00000008167	ENSMUSG00000008167	8	87950233	87954776	151	173		
7311	19	12647565	12653716	ENSMUSG00000060038	ENSMUSG00000060038	ENSMUSG00000060038	8	87961862	87965266	151	173		
7312	19	12641567	12647640	ENSMUSG00000005150	ENSMUSG00000005150	ENSMUSG00000005150	8	87965140	87970851	151	173		
7313	19	12639885	12643170	ENSMUSG00000059355	ENSMUSG00000059355	ENSMUSG00000059355	8	87970879	87972433	151	173		
7314	19	12618322	12638828	ENSMUSG00000005142	ENSMUSG00000005142	ENSMUSG00000005142	8	87973420	87988387	151	173		
7315	19	12582753	12601647	ENSMUSG00000074194	ENSMUSG00000074194	ENSMUSG00000074194	8	87998666	88003738	151	173		
7316	16	45251095	45280593	ENSMUSG00000031696	ENSMUSG00000031696	ENSMUSG00000031696	8	88151235	88185062	152	174		
7317	16	45281059	45289806	ENSMUSG00000031697	ENSMUSG00000031697	ENSMUSG00000031697	8	88189896	88198175	152	174		
7318	16	45298960	45339722	ENSMUSG00000031698	ENSMUSG00000031698	ENSMUSG00000031698	8	88214406	88255279	152	174		
7319	16	45393460	45422575	ENSMUSG00000036934	ENSMUSG00000036934	ENSMUSG00000036934	8	88298864	88322934	152	174		
7320	16	45475809	45522699	ENSMUSG00000031700	ENSMUSG00000031700	ENSMUSG00000031700	8	88382690	88417664	152	174		
7321	16	45672943	45735409	ENSMUSG00000036902	ENSMUSG00000036902	ENSMUSG00000036902	8	88530641	88564236	152	174		
7322	16	45746815	46052519	ENSMUSG00000031703	ENSMUSG00000031703	ENSMUSG00000031703	8	88608645	88730877	152	174		
7323	16	46052739	46291657	ENSMUSG00000036879	ENSMUSG00000036879	ENSMUSG00000036879	8	88731107	88950754	152	174		
7324	16	46674385	46738182	ENSMUSG00000036872	ENSMUSG00000036872	ENSMUSG00000036872	8	89394946	89456695	152	174		
7325	16	46835712	46944908	ENSMUSG00000047866	ENSMUSG00000047866	ENSMUSG00000047866	8	89514212	89606741	152	174		
7326	16	47130140	47201621	ENSMUSG00000031652	ENSMUSG00000031652	ENSMUSG00000031652	8	89734441	89775345	152	174		
7327	16	47870202	47873193	ENSMUSG00000031654	ENSMUSG00000031654	ENSMUSG00000031654	8	90358972	90362401	152	174		
7328	16	47965309	47990816	ENSMUSG00000069971	ENSMUSG00000069971	ENSMUSG00000069971	8	90454012	90479302	152	174		
7329	16	48082023	48418419	ENSMUSG00000045333	ENSMUSG00000045333	ENSMUSG00000045333	8	90576534	90794663	152	174		
7330	16	48616728	48624943	ENSMUSG00000036810	ENSMUSG00000036810	ENSMUSG00000036810	8	91008905	91024585	152	174		
7331	16	48657417	48696874	ENSMUSG00000031657	ENSMUSG00000031657	ENSMUSG00000031657	8	91027990	91062103	152	174		
7332	16	48745280	48820909	ENSMUSG00000036779	ENSMUSG00000036779	ENSMUSG00000036779	8	91089319	91149821	152	174		
7333	16	48879323	48909536	ENSMUSG00000031659	ENSMUSG00000031659	ENSMUSG00000031659	8	91162508	91219144	152	174		
7334	16	48910450	48960362	ENSMUSG00000031660	ENSMUSG00000031660	ENSMUSG00000031660	8	91222416	91252261	152	174		
7335	16	49139742	49226142	ENSMUSG00000031661	ENSMUSG00000031661	ENSMUSG00000031661	8	91411459	91483156	152	174		
7336	16	49264387	49272667	ENSMUSG00000031662	ENSMUSG00000031662	ENSMUSG00000031662	8	91516890	91526207	152	174		
7337	16	49288551	49324488	ENSMUSG00000055994	ENSMUSG00000055994	ENSMUSG00000055994	8	91537452	91578579	152	174		
7338	16	49333486	49390029	ENSMUSG00000036712	ENSMUSG00000036712	ENSMUSG00000036712	8	91587180	91638573	152	174		
7339	16	49727830	49742653	ENSMUSG00000031665	ENSMUSG00000031665	ENSMUSG00000031665	8	91917351	91934267	152	174		
7340	16	51030641	51138136	ENSMUSG00000043668	ENSMUSG00000043668	ENSMUSG00000043668	8	93137145	93238347	152	174		
7341	16	51747339	51918283	ENSMUSG00000056608	ENSMUSG00000056608	ENSMUSG00000056608	8	93718942	93944613	152	174		
7342	16	52025862	52083060	ENSMUSG00000031666	ENSMUSG00000031666	ENSMUSG00000031666	8	93960214	94013949	152	174		
7343	16	52082693	52094671	ENSMUSG00000031667	ENSMUSG00000031667	ENSMUSG00000031667	8	94013604	94024570	152	174		
7344	16	52192101	52292136	ENSMUSG00000033282	ENSMUSG00000033282	ENSMUSG00000033282	8	94107127	94203367	152	174		
7345	16	52295569	52705845	ENSMUSG00000055932	ENSMUSG00000055932	ENSMUSG00000055932	8	94203637	94558542	152	174		
7346	16	52874713	52877879	ENSMUSG00000031734	ENSMUSG00000031734	ENSMUSG00000031734	8	94687657	94690782	152	174		
7347	16	53522612	53525898	ENSMUSG00000031737	ENSMUSG00000031737	ENSMUSG00000031737	8	95246924	95250584	152	174		
7348	16	53915173	53922168	ENSMUSG00000031738	ENSMUSG00000031738	ENSMUSG00000031738	8	95564163	95567950	152	174		
7349	16	54070589	54098101	ENSMUSG00000031740	ENSMUSG00000031740	ENSMUSG00000031740	8	95716456	95742548	152	174		
7350	16	54100455	54174650	ENSMUSG00000033192	ENSMUSG00000033192	ENSMUSG00000033192	8	95744478	95808407	152	174		
7351	16	54248057	54296685	ENSMUSG00000055368	ENSMUSG00000055368	ENSMUSG00000055368	8	95850358	95890795	152	174		
7352	16	54437665	54466754	ENSMUSG00000058019	ENSMUSG00000058019	ENSMUSG00000058019	8	96403325	96424836	152	174		
7353	16	54952865	55016804	ENSMUSG00000031751	ENSMUSG00000031751	ENSMUSG00000031751	8	96861055	96901971	152	174		
7354	16	55043002	55070513	ENSMUSG00000033009	ENSMUSG00000033009	ENSMUSG00000033009	8	96926420	96957051	152	174		
7355	16	55075801	55111696	ENSMUSG00000031755	ENSMUSG00000031755	ENSMUSG00000031755	8	96957083	96987940	152	174		
7356	16	55156542	55160345	ENSMUSG00000031757	ENSMUSG00000031757	ENSMUSG00000031757	8	97026371	97027996	152	174		
7357	16	55180768	55182500	ENSMUSG00000031760	ENSMUSG00000031760	ENSMUSG00000031760	8	97041838	97043275	152	174		
7358	16	55273894	55275608	ENSMUSG00000031762	ENSMUSG00000031762	ENSMUSG00000031762	8	97061913	97062691	152	174		
7359	16	55321573	55436177	ENSMUSG00000032939	ENSMUSG00000032939	ENSMUSG00000032939	8	97103726	97203986	152	174		
7360	16	55456643	55504850	ENSMUSG00000031766	ENSMUSG00000031766	ENSMUSG00000031766	8	97218330	97255342	152	174		
7361	16	55523249	55535294	ENSMUSG00000031770	ENSMUSG00000031770	ENSMUSG00000031770	8	97275629	97284487	152	174		
7362	16	55612126	55673941	ENSMUSG00000031773	ENSMUSG00000031773	ENSMUSG00000031773	8	97365252	97395907	152	174		
7363	16	55684007	55739377	ENSMUSG00000034361	ENSMUSG00000034361	ENSMUSG00000034361	8	97422157	97459658	152	174		
7364	16	55777755	55831887	ENSMUSG00000050079	ENSMUSG00000050079	ENSMUSG00000050079	8	97491451	97549404	152	174		
7365	16	55836539	55845046	ENSMUSG00000031776	ENSMUSG00000031776	ENSMUSG00000031776	8	97555780	97563546	152	174		
7366	16	55847505	55876077	ENSMUSG00000031775	ENSMUSG00000031775	ENSMUSG00000031775	8	97564024	97585371	152	174		
7367	16	55950219	55957600	ENSMUSG00000031779	ENSMUSG00000031779	ENSMUSG00000031779	8	97634719	97640698	152	174		
7368	16	55963900	55976455	ENSMUSG00000031778	ENSMUSG00000031778	ENSMUSG00000031778	8	97661333	97671552	152	174		
7369	16	56005294	56007475	ENSMUSG00000031780	ENSMUSG00000031780	ENSMUSG00000031780	8	97699582	97701164	152	174		
7370	16	56038903	56052679	ENSMUSG00000031782	ENSMUSG00000031782	ENSMUSG00000031782	8	97727546	97744024	152	174		
7371	16	56054080	56063422	ENSMUSG00000031783	ENSMUSG00000031783	ENSMUSG00000031783	8	97746685	97759818	152	174		
7372	16	56063381	56077828	ENSMUSG00000040631	ENSMUSG00000040631	ENSMUSG00000040631	8	97754258	97765431	152	174		
7373	16	56103596	56127978	ENSMUSG00000063605	ENSMUSG00000063605	ENSMUSG00000063605	8	97792000	97807184	152	174		
7374	16	56153507	56183094	ENSMUSG00000061577	ENSMUSG00000061577	ENSMUSG00000061577	8	97812823	97832409	152	174		
7375	16	56241701	56256445	ENSMUSG00000031785	ENSMUSG00000031785	ENSMUSG00000031785	8	97866238	97903324	152	174		
7376	16	56259658	56281476	ENSMUSG00000060470	ENSMUSG00000060470	ENSMUSG00000060470	8	97906861	97934379	152	174		
7377	16	56286225	56322866	ENSMUSG00000031786	ENSMUSG00000031786	ENSMUSG00000031786	8	97944232	97967269	152	174		
7378	16	56328618	56348657	ENSMUSG00000031787	ENSMUSG00000031787	ENSMUSG00000031787	8	97970330	97989003	152	174		
7379	16	56349634	56393909	ENSMUSG00000031788	ENSMUSG00000031788	ENSMUSG00000031788	8	97990536	98027116	152	174		
7380	16	56475348	56562513	ENSMUSG00000031789	ENSMUSG00000031789	ENSMUSG00000031789	8	98128177	98173310	152	174		
7381	16	56567840	56578853	ENSMUSG00000031794	ENSMUSG00000031794	ENSMUSG00000031794	8	98200644	98210457	152	174		
7382	16	56586074	56591263	ENSMUSG00000046556	ENSMUSG00000046556	ENSMUSG00000046556	8	98214862	98220938	152	174		
7383	16	56592806	56613022	ENSMUSG00000031792	ENSMUSG00000031792	ENSMUSG00000031792	8	98221469	98236638	152	174		
7384	16	56616783	56638303	ENSMUSG00000031790	ENSMUSG00000031790	ENSMUSG00000031790	8	98241466	98263422	152	174		
7385	16	56705000	56720797	ENSMUSG00000031796	ENSMUSG00000031796	ENSMUSG00000031796	8	98310262	98323700	152	174		
7386	16	56749320	56789283	ENSMUSG00000046707	ENSMUSG00000046707	ENSMUSG00000046707	8	98337108	98377956	152	174		
7387	16	56841364	56875233	ENSMUSG00000036598	ENSMUSG00000036598	ENSMUSG00000036598	8	98423229	98448017	152	174		
7388	16	56871403	56886444	ENSMUSG00000048400	ENSMUSG00000048400	ENSMUSG00000048400	8	98448421	98464326	152	174		
7389	16	56983908	56997502	ENSMUSG00000031669	ENSMUSG00000031669	ENSMUSG00000031669	8	98522688	98534188	152	174		
7390	16	57055118	57105023	ENSMUSG00000036564	ENSMUSG00000036564	ENSMUSG00000036564	8	98567215	98604248	152	174		
7391	16	57111359	57221251	ENSMUSG00000036550	ENSMUSG00000036550	ENSMUSG00000036550	8	98609539	98677860	152	174		
7392	16	57257799	57276175	ENSMUSG00000036534	ENSMUSG00000036534	ENSMUSG00000036534	8	98725052	98742620	152	174		
7393	16	64958064	64996186	ENSMUSG00000031871	ENSMUSG00000031871	ENSMUSG00000031871	8	106990828	107033630	152	174		
7394	16	65018741	65074230	ENSMUSG00000031872	ENSMUSG00000031872	ENSMUSG00000031872	8	107059569	107107385	152	174		
7395	16	65102151	65141816	ENSMUSG00000035824	ENSMUSG00000035824	ENSMUSG00000035824	8	107115833	107137687	152	174		
7396	16	65143971	65157471	ENSMUSG00000054400	ENSMUSG00000054400	ENSMUSG00000054400	8	107140032	107152572	152	174		
7397	16	65157851	65170535	ENSMUSG00000031876	ENSMUSG00000031876	ENSMUSG00000031876	8	107198330	107199124	152	174		
7398	16	65195436	65205284	ENSMUSG00000031875	ENSMUSG00000031875	ENSMUSG00000031875	8	107229755	107236798	152	174		
7399	16	65208681	65288111	ENSMUSG00000051578	ENSMUSG00000051578	ENSMUSG00000051578	8	107243646	107284825	152	174		
7400	16	65312301	65343026	ENSMUSG00000035770	ENSMUSG00000035770	ENSMUSG00000035770	8	107308258	107332134	152	174		
7401	16	65349703	65393024	ENSMUSG00000052616	ENSMUSG00000052616	ENSMUSG00000052616	8	107335848	107399016	152	174		
7402	16	65394284	65422380	ENSMUSG00000031878	ENSMUSG00000031878	ENSMUSG00000031878	8	107400153	107423737	152	174		
7403	16	65435781	65445545	ENSMUSG00000031883	ENSMUSG00000031883	ENSMUSG00000031883	8	107429936	107439470	152	174		
7404	16	65471937	65479356	ENSMUSG00000048371	ENSMUSG00000048371	ENSMUSG00000048371	8	107480597	107485994	152	174		
7405	16	65499526	65510267	ENSMUSG00000031881	ENSMUSG00000031881	ENSMUSG00000031881	8	107503270	107513392	152	174		
7406	16	65513092	65516939	ENSMUSG00000031880	ENSMUSG00000031880	ENSMUSG00000031880	8	107517201	107520445	152	174		
7407	16	65591956	65601156	ENSMUSG00000060560	ENSMUSG00000060560	ENSMUSG00000060560	8	108020929	108039238	152	174		

7408	16	65620551	65692457	ENSMUSG00000031885	ENSMUSG00000031885	ENSMUSG00000031885	8	108059803	108107115	152	174		
7409	16	65701416	65739941	ENSMUSG00000031889	ENSMUSG00000031889	ENSMUSG00000031889	8	108114328	108142178	152	174		
7410	16	65745605	65751306	ENSMUSG00000031887	ENSMUSG00000031887	ENSMUSG00000031887	8	108147415	108153738	152	174		
7411	16	65751392	65755578	ENSMUSG00000033313	ENSMUSG00000033313	ENSMUSG00000033313	8	108153859	108158454	152	174		
7412	16	65756216	65761225	ENSMUSG00000033249	ENSMUSG00000033249	ENSMUSG00000033249	8	108158998	108164974	152	174		
7413	16	65765574	65767125	ENSMUSG00000014776	ENSMUSG00000014776	ENSMUSG00000014776	8	108168105	108168909	152	174		
7414	16	65768134	65774977	ENSMUSG00000014837	ENSMUSG00000014837	ENSMUSG00000014837	8	108169543	108178592	152	174		
7415	16	65775784	65781500	ENSMUSG00000043251	ENSMUSG00000043251	ENSMUSG00000043251	8	108179053	108185227	152	174		
7416	16	65783569	65790322	ENSMUSG00000014859	ENSMUSG00000014859	ENSMUSG00000014859	8	108186792	108194499	152	174		
7417	16	65790515	65795433	ENSMUSG00000014791	ENSMUSG00000014791	ENSMUSG00000014791	8	108194881	108199006	152	174		
7418	16	65798544	65818405	ENSMUSG00000041679	ENSMUSG00000041679	ENSMUSG00000041679	8	108201477	108215407	152	174		
7419	16	65818515	65820682	ENSMUSG00000014856	ENSMUSG00000014856	ENSMUSG00000014856	8	108215506	108224003	152	174		
7420	16	65820800	65838896	ENSMUSG00000014778	ENSMUSG00000014778	ENSMUSG00000014778	8	108218293	108237082	152	174		
7421	16	65840354	65863594	ENSMUSG00000014786	ENSMUSG00000014786	ENSMUSG00000014786	8	108238506	108257601	152	174		
7422	16	65871449	65880883	ENSMUSG00000014782	ENSMUSG00000014782	ENSMUSG00000014782	8	108265075	108270989	152	174		
7423	16	65880979	65912289	ENSMUSG00000051648	ENSMUSG00000051648	ENSMUSG00000051648	8	108272085	108297753	152	174		
7424	16	65918248	65976604	ENSMUSG00000054320	ENSMUSG00000054320	ENSMUSG00000054320	8	108316769	108353208	152	174		
7425	16	65981215	65984922	ENSMUSG00000014846	ENSMUSG00000014846	ENSMUSG00000014846	8	108356626	108360518	152	174		
7426	16	65985829	66007878	ENSMUSG00000039199	ENSMUSG00000039199	ENSMUSG00000039199	8	108361730	108372912	152	174		
7427	16	66022518	66028953	ENSMUSG00000031891	ENSMUSG00000031891	ENSMUSG00000031891	8	108407884	108413115	152	174		
7428	16	66029426	66072589	ENSMUSG00000013160	ENSMUSG00000013160	ENSMUSG00000013160	8	108413599	108455169	152	174		
7429	16	66073978	66075217	ENSMUSG00000005705	ENSMUSG00000005705	ENSMUSG00000005705	8	108455827	108457427	152	174		
7430	16	66120221	66138189	ENSMUSG00000038604	ENSMUSG00000038604	ENSMUSG00000038604	8	108494410	108511209	152	174		
7431	16	66153965	66230584	ENSMUSG00000005698	ENSMUSG00000005698	ENSMUSG00000005698	8	108525670	108572051	152	174		
7432	16	66236531	66248957	ENSMUSG00000050357	ENSMUSG00000050357	ENSMUSG00000050357	8	108579536	108587269	152	174		
7433	16	66248934	66252214	ENSMUSG00000038000	ENSMUSG00000038000	ENSMUSG00000038000	8	108584989	108590214	152	174		
7434	16	66252352	66254181	ENSMUSG00000005699	ENSMUSG00000005699	ENSMUSG00000005699	8	108590812	108592594	152	174		
7435	16	66254365	66258168	ENSMUSG00000013155	ENSMUSG00000013155	ENSMUSG00000013155	8	108592784	108597319	152	174		
7436	16	66258220	66260156	ENSMUSG00000013158	ENSMUSG00000013158	ENSMUSG00000013158	8	108597420	108599373	152	174		
7437	16	66265948	66310720	ENSMUSG00000013150	ENSMUSG00000013150	ENSMUSG00000013150	8	108605240	108647748	152	174		
7438	16	66314506	66398056	ENSMUSG00000037415	ENSMUSG00000037415	ENSMUSG00000037415	8	108657437	108716479	152	174		
7439	16	66405755	66419394	ENSMUSG00000031893	ENSMUSG00000031893	ENSMUSG00000031893	8	108716873	108733805	152	174		
7440	16	66521349	66522423	ENSMUSG00000031896	ENSMUSG00000031896	ENSMUSG00000031896	8	108717667	108822976	152	175	here	
7441	16	66419563	66426706	ENSMUSG00000036672	ENSMUSG00000036672	ENSMUSG00000036672	8	108733806	108741183	152	175	Markers moved to different place with in a HSB	1 -> 2
7442	16	66433714	66435598	ENSMUSG00000036442	ENSMUSG00000036442	ENSMUSG00000036442	8	108744272	108746080	152	175		
7443	16	66464500	66475906	ENSMUSG00000036270	ENSMUSG00000036270	ENSMUSG00000036270	8	108770080	108782337	152	175		
7444	16	66476282	66477772	ENSMUSG00000044287	ENSMUSG00000044287	ENSMUSG00000044287	8	108782696	108784147	152	175		
7445	16	66484705	66521078	ENSMUSG00000048310	ENSMUSG00000048310	ENSMUSG00000048310	8	108789570	108820907	152	175		
7446	16	66525910	66528251	ENSMUSG00000031897	ENSMUSG00000031897	ENSMUSG00000031897	8	108824877	108827244	152	175		
7447	16	66535731	66560026	ENSMUSG00000017765	ENSMUSG00000017765	ENSMUSG00000017765	8	108832721	108855173	152	175		
7448	16	66567068	66571953	ENSMUSG00000031898	ENSMUSG00000031898	ENSMUSG00000031898	8	108862644	108868558	152	175		
7449	16	66578803	66584668	ENSMUSG00000053687	ENSMUSG00000053687	ENSMUSG00000053687	8	108874186	108885552	152	175		
7450	16	66612984	66614606	ENSMUSG00000045538	ENSMUSG00000045538	ENSMUSG00000045538	8	108898931	108900553	152	175		
7451	16	66614701	66670727	ENSMUSG00000031901	ENSMUSG00000031901	ENSMUSG00000031901	8	108900636	108942948	152	175		
7452	16	66676876	66818301	ENSMUSG00000031902	ENSMUSG00000031902	ENSMUSG00000031902	8	108948972	109017574	152	175		
7453	16	66819951	66827637	ENSMUSG00000033824	ENSMUSG00000033824	ENSMUSG00000033824	8	109019531	109025971	152	175		
7454	16	66836748	66852460	ENSMUSG00000031903	ENSMUSG00000031903	ENSMUSG00000031903	8	109039528	109053844	152	175		
7455	16	66855934	66893222	ENSMUSG00000031904	ENSMUSG00000031904	ENSMUSG00000031904	8	109058004	109087833	152	175		
7456	16	66889570	66902330	ENSMUSG00000033106	ENSMUSG00000033106	ENSMUSG00000033106	8	109089846	109100052	152	175		
7457	16	66906803	66948663	ENSMUSG00000060098	ENSMUSG00000060098	ENSMUSG00000060098	8	109100159	109140821	152	175		
7458	16	66949733	67039905	ENSMUSG00000031906	ENSMUSG00000031906	ENSMUSG00000031906	8	109141677	109227117	152	175		
7459	16	67236240	67290440	ENSMUSG00000061048	ENSMUSG00000061048	ENSMUSG00000061048	8	109400042	109446037	152	175		
7460	16	67328756	67424940	ENSMUSG00000000303	ENSMUSG00000000303	ENSMUSG00000000303	8	109492497	109559375	152	175		
7461	16	67435010	67676584	ENSMUSG00000041949	ENSMUSG00000041949	ENSMUSG00000041949	8	109572197	109740568	152	175		
7462	16	67698944	67709072	ENSMUSG00000031910	ENSMUSG00000031910	ENSMUSG00000031910	8	109763037	109767957	152	175		
7463	16	67709414	67723982	ENSMUSG00000046691	ENSMUSG00000046691	ENSMUSG00000046691	8	109772992	109782722	152	175		
7464	16	67724034	67760424	ENSMUSG00000041438	ENSMUSG00000041438	ENSMUSG00000041438	8	109782780	109812216	152	175		
7465	16	67778533	67892379	ENSMUSG00000041308	ENSMUSG00000041308	ENSMUSG00000041308	8	109824879	109903321	152	175		
7466	16	67907407	67915718	ENSMUSG00000031913	ENSMUSG00000031913	ENSMUSG00000031913	8	109925745	109934885	152	175		
7467	16	67920025	67931014	ENSMUSG00000031916	ENSMUSG00000031916	ENSMUSG00000031916	8	109935418	109945802	152	175		
7468	16	67931047	67934510	ENSMUSG00000031917	ENSMUSG00000031917	ENSMUSG00000031917	8	109946006	109948374	152	175		
7469	16	67934661	67943190	ENSMUSG00000031919	ENSMUSG00000031919	ENSMUSG00000031919	8	109950613	109954773	152	175		
7470	16	67947036	67977374	ENSMUSG00000031921	ENSMUSG00000031921	ENSMUSG00000031921	8	109959033	109985558	152	175		
7471	16	68016082	68055357	ENSMUSG00000031924	ENSMUSG00000031924	ENSMUSG00000031924	8	110039769	110076599	152	175		
7472	16	68156498	68296054	ENSMUSG00000003847	ENSMUSG00000003847	ENSMUSG00000003847	8	110182991	110261358	152	175		
7473	16	68300808	68318034	ENSMUSG00000003849	ENSMUSG00000003849	ENSMUSG00000003849	8	110277948	110282145	152	175		
7474	16	68333448	68346344	ENSMUSG00000003848	ENSMUSG00000003848	ENSMUSG00000003848	8	110301618	110314168	152	175		
7475	16	68353749	68533144	ENSMUSG00000031930	ENSMUSG00000031930	ENSMUSG00000031930	8	110325527	110447724	152	175		
7476	16	72888182	72897685	ENSMUSG00000039067	ENSMUSG00000039067	ENSMUSG00000039067	8	110469510	110477611	153	176		
7477	16	71378464	71639775	ENSMUSG00000038872	ENSMUSG00000038872	ENSMUSG00000038872	8	111603773	111846367	153	176		
7478	16	70710500	70763552	ENSMUSG00000031727	ENSMUSG00000031727	ENSMUSG00000031727	8	112384034	112431017	153	176		
7479	16	70685116	70704311	ENSMUSG00000037993	ENSMUSG00000037993	ENSMUSG00000037993	8	112437144	112454730	153	176		
7480	16	70676259	70685015	ENSMUSG00000031723	ENSMUSG00000031723	ENSMUSG00000031723	8	112455021	112463180	153	176		
7481	16	70600144	70616455	ENSMUSG00000031730	ENSMUSG00000031730	ENSMUSG00000031730	8	112482377	112497802	153	176		
7482	16	70520942	70591378	ENSMUSG00000048827	ENSMUSG00000048827	ENSMUSG00000048827	8	112503646	112559850	153	176		
7483	16	70486940	70520407	ENSMUSG00000031729	ENSMUSG00000031729	ENSMUSG00000031729	8	112560454	112582389	153	176		
7484	16	70451090	70475551	ENSMUSG00000031728	ENSMUSG00000031728	ENSMUSG00000031728	8	112594788	112614061	153	176		
7485	16	70236355	70316105	ENSMUSG00000031732	ENSMUSG00000031732	ENSMUSG00000031732	8	112757775	112830037	153	176		
7486	16	70217571	70233367	ENSMUSG00000001672	ENSMUSG00000001672	ENSMUSG00000001672	8	112837034	112851297	153	176		
7487	16	70158255	70168496	ENSMUSG00000001670	ENSMUSG00000001670	ENSMUSG00000001670	8	112879586	112888930	153	176		
7488	16	70117560	70129910	ENSMUSG00000035930	ENSMUSG00000035930	ENSMUSG00000035930	8	112918282	112928869	153	176		
7489	16	70039014	70053651	ENSMUSG00000044676	ENSMUSG00000044676	ENSMUSG00000044676	8	112971848	112979310	153	176		
7490	16	69950127	69981836	ENSMUSG00000003657	ENSMUSG00000003657	ENSMUSG00000003657	8	113031665	113057335	153	176		
7491	16	69873709	69881013	ENSMUSG00000046441	ENSMUSG00000046441	ENSMUSG00000046441	8	113107118	113113615	153	176		
7492	16	69402732	69454585	ENSMUSG00000059854	ENSMUSG00000059854	ENSMUSG00000059854	8	113156465	113496731	153	176		
7493	16	69278845	69392572	ENSMUSG00000010936	ENSMUSG00000010936	ENSMUSG00000010936	8	113505315	113607079	153	176		
7494	16	69237969	69252085	ENSMUSG00000031750	ENSMUSG00000031750	ENSMUSG00000031750	8	113628517	113692580	153	176		
7495	16	69115241	69163921	ENSMUSG00000033732	ENSMUSG00000033732	ENSMUSG00000033732	8	113696916	113733453	153	176		
7496	16	69071978	69114938	ENSMUSG00000031753	ENSMUSG00000031753	ENSMUSG00000031753	8	113733724	113768904	153	176		
7497	16	69045999	69071677	ENSMUSG00000033703	ENSMUSG00000033703	ENSMUSG00000033703	8	113769140	113789136	153	176		
7498	16	68972810	69030492	ENSMUSG00000031749	ENSMUSG00000031749	ENSMUSG00000031749	8	113806599	113859157	153	176		
7499	16	68843791	68880910	ENSMUSG00000031960	ENSMUSG00000031960	ENSMUSG00000031960	8	113920642	113943545	153	176		
7500	16	68705030	68752685	ENSMUSG00000033624	ENSMUSG00000033624	ENSMUSG00000033624	8	113981314	114023747	153	176		
7501	16	73043357	73198518	ENSMUSG00000003316	ENSMUSG00000003316	ENSMUSG00000003316	8	114044214	114145875	154	177		
7502	16	73263262	73287156	ENSMUSG00000012519	ENSMUSG00000012519	ENSMUSG00000012519	8	114198473	114224850	154	177		
7503	16	73304359	73366224	ENSMUSG00000033579	ENSMUSG00000033579	ENSMUSG00000033579	8	114231814	114280488	154	177		
7504	16	73464975	73576518	ENSMUSG00000031959	ENSMUSG00000031959	ENSMUSG00000031959	8	114335692	114408743	154	177		
7505	16	73591092	73700099	ENSMUSG00000033545	ENSMUSG00000033545	ENSMUSG00000033545	8	114423515	114510453	154	177		
7506	16	73703260	73708166	ENSMUSG00000031958	ENSMUSG00000031958	ENSMUSG00000031958	8	114512176	114517008	154	177		
7507	16	73758157	73763480	ENSMUSG00000055835	ENSMUSG00000055835	ENSMUSG00000055835	8	114530116	114557681	154	177		
7508	16	73820430	73843004	ENSMUSG00000031955	ENSMUSG00000031955	ENSMUSG00000031955	8	114597147	114608054	154	177		
7509	16	73885109	74024888	ENSMUSG00000031954	ENSMUSG00000031954	ENSMUSG00000031954	8	114655146	114740983	154	177		
7510	16	74038426	74056085	ENSMUSG00000031953	ENSMUSG00000031953	ENSMUSG00000031953	8	114751571	114763348	154	177		
7511	16	74129555	74147667	ENSMUSG00000031951	ENSMUSG00000031951	ENSMUSG00000031951	8	114800605	114820464	154	177		
7512	16	74190499	74214655	ENSMUSG00000031949	ENSMUSG00000031949	ENSMUSG00000031949	8	114853581	114878975	154	177		
7513	16	74219294	74239064	ENSMUSG00000031948	ENSMUSG00000031948	ENSMUSG00000031948	8	114880117	114897939	154	177		
7514	16	74239144	74248830	ENSMUSG00000033430	ENSMUSG00000033430	ENSMUSG00000033430	8	114898055	114905872	154	177		
7515	16	74907578	75150461	ENSMUSG00000031772	ENSMUSG00000031772	ENSMUSG00000031772	8	115456020	115768696	154	177		
7516	16	75782337	75791042	ENSMUSG00000033409	ENSMUSG00000033409	ENSMUSG00000033409	8	116521812	116529475	154	177		
7517	16	75873929	76026512	ENSMUSG00000053399	ENSMUSG00000053399	ENSMUSG00000053399	8	116583904	116734471	154	177		
7518	16	76313981	76333348	ENSMUSG00000031767	ENSMUSG00000031767	ENSMUSG00000031767	8	117021311	117038282	154	177		
7519	16	76379984	76571504	ENSMUSG00000046844	ENSMUSG00000046844	ENSMUSG00000046844	8	117091607	117260047	154	177		
7520	16	76613992	76623495	ENSMUSG00000008874	ENSMUSG00000008874	ENSMUSG00000008874	8	117304063	117311823	154	177		
7521	16	76691177	77803532	ENSMUSG00000004637	ENSMUSG00000004637	ENSMUSG00000004637	8	117325749	118237803	154	177		
7522	16	78185736	78192112	ENSMUSG00000055435	ENSMUSG00000055435	ENSMUSG00000055435	8	118579136	118592840	154	177		
7523	16	79132355	79142040	ENSMUSG00000034467	ENSMUSG00000034467	ENSMUSG00000034467	8	119393479	119401700	154	177		
7524	16	79195179	79395676	ENSMUSG00000031758	ENSMUSG00000031758	ENSMUSG00000031758	8	119461106	119510346	154	177		
7525	16	79567206	79597997	ENSMUSG00000014633	ENSMUSG00000014633	ENSMUSG00000014633	8	119775219	119807407	154	177		
7526	16	79597604	79620428	ENSMUSG00000031756	ENSMUSG00000031756	ENSMUSG00000031756	8	119807717	119827480	154	177		
7527	16	79626959	79638452	ENSMUSG00000047388	ENSMUSG00000047388	ENSMUSG00000047388	8	119829385	119846422	154	177		
7528	16	79652093	79668305	ENSMUSG00000031847	ENSMUSG00000031847	ENSMUSG00000031847	8	119855576	119864920	154	177		
7529	16	79692235	79811467	ENSMUSG00000034416	ENSMUSG00000034416	ENSMUSG00000034416	8	119881656	119968426	154	177		
7530	16	79829797	79882248	ENSMUSG00000031845	ENSMUSG00000031845	ENSMUSG00000031845	8	119981831	120019474	154	177		
7531	16	79906076	79971441	ENSMUSG00000052557	ENSMUSG00000052557	ENSMUSG00000052557	8	120044112	120091161	154	177		
7532	16	80036396	80300905	ENSMUSG00000034390	ENSMUSG00000034390	ENSMUSG00000034390	8	120235147	120345390	154	177		
7533	16	80370408	80549399	ENSMUSG00000034330	ENSMUSG00000034330	ENSMUSG00000034330	8	120384268	120521119	154	177		
7534	16	80588759	80602571	ENSMUSG00000034308	ENSMUSG00000034308	ENSMUSG00000034308	8	120547376	120557492	154	177		
7535	16	80626364	80689638	ENSMUSG00000031844	ENSMUSG00000031844	ENSMUSG00000031844	8	120587923	120645004	154	177		
7536	16	81449460	82387705	ENSMUSG00000031841	ENSMUSG00000031841	ENSMUSG00000031841	8	121391578	122200176	154	177		
7537	16	82490231	82507286	ENSMUSG00000074064	ENSMUSG00000074064	ENSMUSG00000074064	8	122280869	122297067	154	177		
7538	16	82540173	82557437	ENSMUSG00000074063	ENSMUSG00000074063	ENSMUSG00000074063	8	122323139	122332233	154	177		
7539	16	82559738	82593878	ENSMUSG00000031837	ENSMUSG00000031837	ENSMUSG00000031837	8	122332696	122358612	154	177		
7540	16	82600890	82633263	ENSMUSG00000034224	ENSMUSG00000034224	ENSMUSG00000034224	8	122365579	122387675	154	177		
7541	16	82644872	82708018	ENSMUSG00000031835	ENSMUSG00000031835	ENSMUSG00000031835	8	122394128	122444704	154	177		
7542	16	82713389	82736265	ENSMUSG00000034189	ENSMUSG00000034189	ENSMUSG00000034189	8	122447966	122461160	154	177		
7543	16	82736393	82769024	ENSMUSG00000031831	ENSMUSG00000031831	ENSMUSG00000031831	8	122461212	122484431	154	177		
7544	16	82770048	82776095	ENSMUSG00000031832	ENSMUSG00000031832	ENSMUSG00000031832	8	122483951	122491196	154	177		
7545	16	82782245	82788273	ENSMUSG00000024266	ENSMUSG00000024266	ENSMUSG00000024266	8	122498724	122502901	154	177		
7546	16	82813324	82830857	ENSMUSG00000045246	ENSMUSG00000045246	ENSMUSG00000045246	8	122509831	122521657	154	177		
7547	16	82885822	82920888	ENSMUSG00000023336	ENSMUSG00000023336	ENSMUSG00000023336	8	122552500	122574199	154	177		
7548	16	82995534	83055107	ENSMUSG00000034112	ENSMUSG00000034112	ENSMUSG00000034112	8	122607208	122643693	154	177		
7549	16	83239632	83253416	ENSMUSG00000031828	ENSMUSG00000031828	ENSMUSG00000031828	8	122748282	122762970	154	177		
7550	16	83291119	83371026	ENSMUSG00000031826	ENSMUSG00000031826	ENSMUSG00000031826	8	122796806	122843534	154	177		
7551	16	83411113	83500614	ENSMUSG00000031825	ENSMUSG00000031825	ENSMUSG00000031825	8	122878486	122937553	154	177		
7552	16	83565687	83602629	ENSMUSG00000031823	ENSMUSG00000031823	ENSMUSG00000031823	8	122967072	122987449	154	177		
7553	16	83618911	83685327	ENSMUSG00000031824	ENSMUSG00000031824	ENSMUSG00000031824	8	123000143	123051275	154	177		
7554	16	83689466	83703615	ENSMUSG00000042269	ENSMUSG00000042269	ENSMUSG00000042269	8	123052375	123063446	154	177		
7555	16	84204425	84267311	ENSMUSG00000031822	ENSMUSG00000031822	ENSMUSG00000031822	8	123374843	123467367	154	177		
7556	16	84268782	84280089	ENSMUSG00000031821	ENSMUSG00000031821	ENSMUSG00000031821	8	123464610	123475067	154	177		
7557	16	84298630	84301957	ENSMUSG00000043687	ENSMUSG00000043687	ENSMUSG00000043687	8	123494579	123520359	154	177		
7558	16	84369737	84390601	ENSMUSG00000031819	ENSMUSG00000031819	ENSMUSG00000031819	8	123542970	123553956	154	177		
7559	16	84390697	84398109	ENSMUSG00000031818	ENSMUSG00000031818	ENSMUSG00000031818	8	123554267	123560184	154	177		
7560	16	84490275	84513710	ENSMUSG00000041515	ENSMUSG00000041515	ENSMUSG00000041515	8	123622335	123642667	154	177		
7561	16	85121988	85157509	ENSMUSG00000031816	ENSMUSG00000031816	ENSMUSG00000031816	8	123983919	123994356	154	177		
7562	16	85158443	85159948	ENSMUSG00000046714	ENSMUSG00000046714	ENSMUSG00000046714	8	124002148	124004867	154	177		
7563	16	85893924	85908349	ENSMUSG00000031809	ENSMUSG00000031809	ENSMUSG00000031809	8	124416761	124427931	154	177		
7564	16	85920445	85974877	ENSMUSG00000031811	ENSMUSG00000031811	ENSMUSG00000031811	8	124437991	124464843	154	177		
7565	16	85997378	86082966	ENSMUSG00000061410	ENSMUSG00000061410	ENSMUSG00000061410	8	124485528	124538023	154	177		
7566	16	86194000	86289263	ENSMUSG00000025318	ENSMUSG00000025318	ENSMUSG00000025318	8	124616678	124680366	154	177		
7567	16	86298920	86357056	ENSMUSG00000040263	ENSMUSG00000040263	ENSMUSG00000040263	8	124682420	124715659	154	177		
7568	16	86421131	86460615	ENSMUSG00000040010	ENSMUSG00000040010	ENSMUSG00000040010	8	124767240	124793773	154	177		
7569	16	86479126	86527613	ENSMUSG00000025317	ENSMUSG00000025317	ENSMUSG00000025317	8	124802236	124830957	154	177		
7570	16	87047226	87128890	ENSMUSG00000049577	ENSMUSG00000049577	ENSMUSG00000049577	8	125168229	125223341	154	177		
7571	16	87164343	87225756	ENSMUSG00000017478	ENSMUSG00000017478	ENSMUSG00000017478	8	125262706	125303450	154	177		
7572	16	87232502	87234031	ENSMUSG00000046108	ENSMUSG00000046108	ENSMUSG00000046108	8	125308175	125309658	154	177		
7573	16	87237199	87244958	ENSMUSG00000006519	ENSMUSG00000006519	ENSMUSG00000006519	8	125310866	125319017	154	177		
7574	16	87245849	87257019	ENSMUSG00000006517	ENSMUSG00000006517	ENSMUSG00000006517	8	125319691	125329512	154	177		
7575	16	87271677	87280383	ENSMUSG00000006587	ENSMUSG00000006587	ENSMUSG00000006587	8	125340296	125346782	154	177		
7576	16	87290411	87300312	ENSMUSG00000014470	ENSMUSG00000014470	ENSMUSG00000014470	8	125352237	125362154	154	177		
7577	16	87300431	87309285	ENSMUSG00000049482	ENSMUSG00000049482	ENSMUSG00000049482	8	125362280	125367837	154	177		
7578	16	87309267	87330158	ENSMUSG00000014444	ENSMUSG00000014444	ENSMUSG00000014444	8	125367809	125381093	154	177		
7579	16	87397687	87403166	ENSMUSG00000006585	ENSMUSG00000006585	ENSMUSG00000006585	8	125454116	125459644	154	177		
7580	16	87407644	87450885	ENSMUSG00000015027	ENSMUSG00000015027	ENSMUSG00000015027	8	125464328	125497479	154	177		
7581	16	87457665	87460515	ENSMUSG00000069867	ENSMUSG00000069867	ENSMUSG00000069867	8	125505561	125508825	154	177		
7582	16	87468768	87570902	ENSMUSG00000006362	ENSMUSG00000006362	ENSMUSG00000006362	8	125516160	125585070	154	177		
7583	16	87687825	87749670	ENSMUSG00000015016	ENSMUSG00000015016	ENSMUSG00000015016	8	125666060	125703596	154	177		
7584	16	87765664	87789400	ENSMUSG00000031962	ENSMUSG00000031962	ENSMUSG00000031962	8	125734056	125753487	154	177		
7585	16	87861536	88084470	ENSMUSG00000035569	ENSMUSG00000035569	ENSMUSG00000035569	8	125770244	125786243	154	177		
7586	16	88102306	88151674	ENSMUSG00000000738	ENSMUSG00000000738	ENSMUSG00000000738	8	125951269	125983845	154	177		
7587	16	88169677	88191155	ENSMUSG00000034796	ENSMUSG00000034796	ENSMUSG00000034796	8	126003537	126021272	154	177		
7588	16	88207582	88232307	ENSMUSG00000019278	ENSMUSG00000019278	ENSMUSG00000019278	8	126072332	126087314	154	177		
7589	16	88251711	88265176	ENSMUSG00000048478	ENSMUSG00000048478	ENSMUSG00000048478	8	126098974	126108134	154	177		
7590	16	88290258	88295604	ENSMUSG00000033594	ENSMUSG00000033594	ENSMUSG00000033594	8	126118345	126122323	154	177		
7591	16	88301773	88314770	ENSMUSG00000001062	ENSMUSG00000001062	ENSMUSG00000001062	8	126128450	126140312	154	177		
7592	16	88314934	88333811	ENSMUSG00000001065	ENSMUSG00000001065	ENSMUSG00000001065	8	126140333	126155823	154	177		
7593	16	88332510	88410524	ENSMUSG00000032815	ENSMUSG00000032815	ENSMUSG00000032815	8	126154390	126204666	154	177		
7594	16	88422408	88465228	ENSMUSG00000010154	ENSMUSG00000010154	ENSMUSG00000010154	8	126218803	126255612	154	177		
7595	16	88467521	88505293	ENSMUSG00000001472	ENSMUSG00000001472	ENSMUSG00000001472	8	126259914	126287982	154	177		
7596	16	88513168	88529713	ENSMUSG00000062380	ENSMUSG00000062380	ENSMUSG00000062380	8	126297639	126308095	154	177		
7597	16	88566549	88594558	ENSMUSG00000031967	ENSMUSG00000031967	ENSMUSG00000031967	8	126363993	126390006	154	177		
7598	16	88598804	88613382	ENSMUSG00000031970	ENSMUSG00000031970	ENSMUSG00000031970	8	126391807	126401481	154	177		
7599	16	88616509	88638880	ENSMUSG00000040220	ENSMUSG00000040220	ENSMUSG00000040220	8	126404924	126422740	154	177		
7600	1	226937447	226949039	ENSMUSG00000039960	ENSMUSG00000039960	ENSMUSG00000039960	8	126540019	126549972	155	178		
7601	1	227473516	227507000	ENSMUSG00000019478	ENSMUSG00000019478	ENSMUSG00000019478	8	126692115	126721377	155	178		
7602	1	227523381	227544887	ENSMUSG00000031971	ENSMUSG00000031971	ENSMUSG00000031971	8	126726935	126746299	155	178		

7603	1	227633615	227636464	ENSMUSG00000031972	ENSMUSG00000031972	ENSMUSG00000031972	8	126777859	126780841	155	178
7604	1	227643667	227710711	ENSMUSG00000039509	ENSMUSG00000039509	ENSMUSG00000039509	8	126785390	126835355	155	178
7605	1	227718952	227761065	ENSMUSG00000031974	ENSMUSG00000031974	ENSMUSG00000031974	8	126838549	126869212	155	178
7606	1	227795481	227816852	ENSMUSG00000038697	ENSMUSG00000038697	ENSMUSG00000038697	8	126882400	126907399	155	178
7607	1	227828604	227862569	ENSMUSG00000031976	ENSMUSG00000031976	ENSMUSG00000031976	8	126909563	126934594	155	178
7608	1	228269579	228484493	ENSMUSG00000031977	ENSMUSG00000031977	ENSMUSG00000031977	8	127117484	127231812	155	178
7609	1	228524016	228579990	ENSMUSG00000050751	ENSMUSG00000050751	ENSMUSG00000050751	8	127255139	127325748	155	178
7610	1	228844861	228896351	ENSMUSG00000031979	ENSMUSG00000031979	ENSMUSG00000031979	8	127406876	127438093	155	178
7611	1	228904892	228916666	ENSMUSG00000031980	ENSMUSG00000031980	ENSMUSG00000031980	8	127442678	127462983	155	178
7612	1	228949753	229004141	ENSMUSG00000031981	ENSMUSG00000031981	ENSMUSG00000031981	8	127462310	127502367	155	178
7613	1	229039488	229070992	ENSMUSG00000031983	ENSMUSG00000031983	ENSMUSG00000031983	8	127521846	127549459	155	178
7614	1	229108612	229181207	ENSMUSG00000037300	ENSMUSG00000037300	ENSMUSG00000037300	8	127557425	127608054	155	178
7615	1	229181446	229203094	ENSMUSG00000031982	ENSMUSG00000031982	ENSMUSG00000031982	8	127608229	127620213	155	178
7616	1	229221331	229242633	ENSMUSG00000043068	ENSMUSG00000043068	ENSMUSG00000043068	8	127627137	127637893	155	178
7617	1	229365297	229422321	ENSMUSG00000036913	ENSMUSG00000036913	ENSMUSG00000036913	8	127679182	127720792	155	178
7618	1	229426135	229443547	ENSMUSG00000031984	ENSMUSG00000031984	ENSMUSG00000031984	8	127725447	127749208	155	178
7619	1	229443579	229480342	ENSMUSG00000031985	ENSMUSG00000031985	ENSMUSG00000031985	8	127749143	127776139	155	178
7620	1	229537937	229540114	ENSMUSG00000074030	ENSMUSG00000074030	ENSMUSG00000074030	8	127781566	127783716	155	178
7621	1	229540417	229557392	ENSMUSG00000031986	ENSMUSG00000031986	ENSMUSG00000031986	8	127784248	127788017	155	178
7622	1	229730910	229768893	ENSMUSG00000056820	ENSMUSG00000056820	ENSMUSG00000056820	8	127899087	127920282	155	178
7623	1	229829184	230243641	ENSMUSG00000043051	ENSMUSG00000043051	ENSMUSG00000043051	8	127940285	128147238	155	178
7624	1	230600334	230763927	ENSMUSG00000001995	ENSMUSG00000001995	ENSMUSG00000001995	8	128304166	128455846	155	178
7625	1	231153009	231179897	ENSMUSG00000031851	ENSMUSG00000031851	ENSMUSG00000031851	8	128620319	128634300	155	178
7626	1	231530137	231587517	ENSMUSG00000031853	ENSMUSG00000031853	ENSMUSG00000031853	8	128796540	128833529	155	178
7627	1	231816373	231874881	ENSMUSG00000033998	ENSMUSG00000033998	ENSMUSG00000033998	8	128881260	128916775	155	178
7628	1	232107302	232526885	ENSMUSG00000057060	ENSMUSG00000057060	ENSMUSG00000057060	8	129184689	129281572	155	178
7629	1	232575825	232586418	ENSMUSG00000051671	ENSMUSG00000051671	ENSMUSG00000051671	8	129308605	129311523	155	178
7630	1	232806638	232811894	ENSMUSG00000051495	ENSMUSG00000051495	ENSMUSG00000051495	8	129477206	129479526	155	178
7631	1	233361121	233391395	ENSMUSG00000033931	ENSMUSG00000033931	ENSMUSG00000033931	8	129833260	129857169	155	178
7632	10	34438495	35144255	ENSMUSG00000025812	ENSMUSG00000025812	ENSMUSG00000025812	8	129950335	130496920	156	179
7633	10	33506426	33665196	ENSMUSG00000025810	ENSMUSG00000025810	ENSMUSG00000025810	8	131245328	131389437	156	179
7634	10	33229326	33287204	ENSMUSG00000025809	ENSMUSG00000025809	ENSMUSG00000025809	8	131591503	131618178	156	179
7635	11	106880600	106936829	ENSMUSG00000025899	ENSMUSG00000025899	ENSMUSG00000025899	9	3335478	3386929	157	180
7636	11	106063120	106394381	ENSMUSG00000041624	ENSMUSG00000041624	ENSMUSG00000041624	9	3532803	3797381	157	180
7637	11	105453495	105474629	ENSMUSG00000025894	ENSMUSG00000025894	ENSMUSG00000025894	9	4294817	4309469	157	180
7638	11	105427036	105453396	ENSMUSG00000025893	ENSMUSG00000025893	ENSMUSG00000025893	9	4309833	4331732	157	180
7639	11	105383840	105398164	ENSMUSG00000041124	ENSMUSG00000041124	ENSMUSG00000041124	9	4376612	4386870	157	180
7640	11	104986935	105355676	ENSMUSG00000025892	ENSMUSG00000025892	ENSMUSG00000025892	9	4417889	4796142	157	180
7641	11	104261876	104274372	ENSMUSG00000025887	ENSMUSG00000025887	ENSMUSG00000025887	9	5345502	5373034	157	180
7642	11	103283131	103540317	ENSMUSG00000032006	ENSMUSG00000032006	ENSMUSG00000032006	9	6168612	6377519	157	180
7643	11	103412538	103415126	ENSMUSG00000047619	ENSMUSG00000047619	ENSMUSG00000047619	9	6265141	6266367	157	180
7644	11	102585842	102855521	ENSMUSG00000047193	ENSMUSG00000047193	ENSMUSG00000047193	9	6928503	7177046	157	180
7645	11	102438037	102467811	ENSMUSG00000032002	ENSMUSG00000032002	ENSMUSG00000032002	9	7184524	7207031	157	180
7646	11	102318937	102331672	ENSMUSG00000050578	ENSMUSG00000050578	ENSMUSG00000050578	9	7272545	7283333	157	180
7647	11	102238686	102250922	ENSMUSG00000049723	ENSMUSG00000049723	ENSMUSG00000049723	9	7347406	7360461	157	180
7648	11	102088599	102100868	ENSMUSG00000005800	ENSMUSG00000005800	ENSMUSG00000005800	9	7558456	7568486	157	180
7649	11	102067625	102081678	ENSMUSG00000070323	ENSMUSG00000070323	ENSMUSG00000070323	9	7571458	7581393	157	180
7650	11	101952776	102001273	ENSMUSG00000018620	ENSMUSG00000018620	ENSMUSG00000018620	9	7628240	7674979	157	180
7651	11	101896450	101906688	ENSMUSG00000018623	ENSMUSG00000018623	ENSMUSG00000018623	9	7692146	7699125	157	180
7652	11	101723176	101754604	ENSMUSG00000057367	ENSMUSG00000057367	ENSMUSG00000057367	9	7818749	7835790	157	180
7653	11	101693404	101713658	ENSMUSG00000032000	ENSMUSG00000032000	ENSMUSG00000032000	9	7849220	7873545	157	180
7654	11	101486497	101606394	ENSMUSG00000053110	ENSMUSG00000053110	ENSMUSG00000053110	9	7935018	8005113	157	180
7655	11	101423399	101460501	ENSMUSG00000053070	ENSMUSG00000053070	ENSMUSG00000053070	9	8022193	8043344	157	180
7656	11	101290963	101377000	ENSMUSG00000040729	ENSMUSG00000040729	ENSMUSG00000040729	9	8076982	8121246	157	180
7657	11	101266616	101292465	ENSMUSG00000074514	ENSMUSG00000074514	ENSMUSG00000074514	9	8143116	8185647	157	180
7658	11	100827577	100959869	ENSMUSG00000031997	ENSMUSG00000031997	ENSMUSG00000031997	9	8544711	8642583	157	180
7659	11	100289393	100364739	ENSMUSG00000050730	ENSMUSG00000050730	ENSMUSG00000050730	9	8959439	9021001	157	180
7660	11	99195473	99732683	ENSMUSG00000039488	ENSMUSG00000039488	ENSMUSG00000039488	9	9623184	10133944	157	180
7661	11	95351088	95466332	ENSMUSG00000031925	ENSMUSG00000031925	ENSMUSG00000031925	9	13370387	13456998	157	180
7662	11	95205694	95297107	ENSMUSG00000031918	ENSMUSG00000031918	ENSMUSG00000031918	9	13498807	13556194	157	180
7663	11	95163290	95205502	ENSMUSG00000031922	ENSMUSG00000031922	ENSMUSG00000031922	9	13558180	13577386	157	180
7664	11	94545781	94603894	ENSMUSG00000032009	ENSMUSG00000032009	ENSMUSG00000032009	9	14026802	14076531	157	180
7665	11	94462671	94505455	ENSMUSG00000037419	ENSMUSG00000037419	ENSMUSG00000037419	9	14104403	14131639	157	180
7666	11	94141185	94250990	ENSMUSG00000013076	ENSMUSG00000013076	ENSMUSG00000013076	9	14297902	14393922	157	180
7667	11	93940162	93994234	ENSMUSG00000036912	ENSMUSG00000036912	ENSMUSG00000036912	9	14452751	14491126	157	180
7668	11	93916775	93922712	ENSMUSG00000049307	ENSMUSG00000049307	ENSMUSG00000049307	9	14501088	14502389	157	180
7669	11	93866801	93872385	ENSMUSG00000031931	ENSMUSG00000031931	ENSMUSG00000031931	9	14530602	14534954	157	180
7670	11	93790123	93866688	ENSMUSG00000031928	ENSMUSG00000031928	ENSMUSG00000031928	9	14535100	14584812	157	180
7671	11	93750131	93774066	ENSMUSG00000031932	ENSMUSG00000031932	ENSMUSG00000031932	9	14610647	14619892	157	180
7672	11	93678445	93680506	ENSMUSG00000031933	ENSMUSG00000031933	ENSMUSG00000031933	9	14650440	14654325	157	180
7673	11	93501742	93554781	ENSMUSG00000031934	ENSMUSG00000031934	ENSMUSG00000031934	9	14755554	14795816	157	180
7674	11	93394192	93483872	ENSMUSG00000031936	ENSMUSG00000031936	ENSMUSG00000031936	9	14811306	14862500	157	180
7675	11	93157096	93186102	ENSMUSG00000031935	ENSMUSG00000031935	ENSMUSG00000031935	9	15010744	15030260	157	180
7676	11	93114483	93135099	ENSMUSG00000031938	ENSMUSG00000031938	ENSMUSG00000031938	9	15033733	15056523	157	180
7677	11	93034525	93103170	ENSMUSG00000046111	ENSMUSG00000046111	ENSMUSG00000046111	9	15084129	15105133	157	180
7678	11	92702804	92788015	ENSMUSG00000039977	ENSMUSG00000039977	ENSMUSG00000039977	9	15310263	15377124	157	180
7679	11	92520500	92570743	ENSMUSG00000043885	ENSMUSG00000043885	ENSMUSG00000043885	9	15460162	15490027	157	180
7680	11	92342437	92355596	ENSMUSG00000050901	ENSMUSG00000050901	ENSMUSG00000050901	9	15613060	15624853	157	180
7681	11	89573832	89595827	ENSMUSG00000001774	ENSMUSG00000001774	ENSMUSG00000001774	9	18042669	18064402	157	180
7682	11	89507491	89565416	ENSMUSG00000043943	ENSMUSG00000043943	ENSMUSG00000043943	9	18073423	18138540	157	180
7683	19	8781382	8794565	ENSMUSG00000056712	ENSMUSG00000056712	ENSMUSG00000056712	9	18206594	18207967	158	181
7684	19	8814332	8814995	ENSMUSG00000038691	ENSMUSG00000038691	ENSMUSG00000038691	9	18228781	18235694	158	181
7685	19	8820521	8887324	ENSMUSG00000032134	ENSMUSG00000032134	ENSMUSG00000032134	9	18245920	18260952	158	181
7686	19	9064921	9065862	ENSMUSG00000054141	ENSMUSG00000054141	ENSMUSG00000054141	9	18505094	18506035	158	181
7687	19	9112073	9135089	ENSMUSG00000057551	ENSMUSG00000057551	ENSMUSG00000057551	9	19391626	19398450	158	181
7688	19	9499683	9510303	ENSMUSG00000059475	ENSMUSG00000059475	ENSMUSG00000059475	9	20220397	20236772	158	181
7689	19	9781948	9790755	ENSMUSG00000066892	ENSMUSG00000066892	ENSMUSG00000066892	9	20388188	20393208	158	181
7690	19	9825438	9908228	ENSMUSG00000032172	ENSMUSG00000032172	ENSMUSG00000032172	9	20418509	20426128	158	181
7691	19	9931237	9982147	ENSMUSG00000004098	ENSMUSG00000004098	ENSMUSG00000004098	9	20520452	20565436	158	181
7692	19	9984925	9993954	ENSMUSG00000053773	ENSMUSG00000053773	ENSMUSG00000053773	9	20568906	20576565	158	181
7693	19	10058528	10064918	ENSMUSG00000038884	ENSMUSG00000038884	ENSMUSG00000038884	9	20619103	20624709	158	181
7694	19	10064015	10074472	ENSMUSG00000038742	ENSMUSG00000038742	ENSMUSG00000038742	9	20624211	20630112	158	181
7695	19	10077965	10082975	ENSMUSG00000004100	ENSMUSG00000004100	ENSMUSG00000004100	9	20638659	20642580	158	181
7696	19	10086691	10091585	ENSMUSG00000070319	ENSMUSG00000070319	ENSMUSG00000070319	9	20645035	20648965	158	181
7697	19	10105023	10166811	ENSMUSG00000004099	ENSMUSG00000004099	ENSMUSG00000004099	9	20657611	20703267	158	181
7698	19	10195520	10202929	ENSMUSG00000043895	ENSMUSG00000043895	ENSMUSG00000043895	9	20716360	20727183	158	181
7699	19	10223640	10231489	ENSMUSG00000003299	ENSMUSG00000003299	ENSMUSG00000003299	9	20753140	20759227	158	181
7700	19	10242765	10258291	ENSMUSG00000037405	ENSMUSG00000037405	ENSMUSG00000037405	9	20766362	20779199	158	181
7701	19	10258650	10260176	ENSMUSG00000001014	ENSMUSG00000001014	ENSMUSG00000001014	9	20779856	20780896	158	181
7702	19	10261655	10268453	ENSMUSG00000032174	ENSMUSG00000032174	ENSMUSG00000032174	9	20782492	20789438	158	181
7703	19	10289150	10305183	ENSMUSG00000010205	ENSMUSG00000010205	ENSMUSG00000010205	9	20817916	20842399	158	181
7704	19	10322205	10350114	ENSMUSG00000032175	ENSMUSG00000032175	ENSMUSG00000032175	9	20854476	20881612	158	181
7705	19	10362809	10375271	ENSMUSG00000019471	ENSMUSG00000019471	ENSMUSG00000019471	9	20888943	20900308	158	181
7706	19	10392331	10440169	ENSMUSG00000032177	ENSMUSG00000032177	ENSMUSG00000032177	9	20916193	20962003	158	181
7707	19	10457802	10475243	ENSMUSG00000003308	ENSMUSG00000003308	ENSMUSG00000003308	9	20980133	20989389	158	181
7708	19	10484623	10489607	ENSMUSG00000045087	ENSMUSG00000045087	ENSMUSG00000045087	9	20993314	20996611	158	181
7709	19	10515593	10525093	ENSMUSG00000002820	ENSMUSG00000002820	ENSMUSG00000002820	9	21015701	21025239	158	181
7710	19	10524761	10537702	ENSMUSG00000035047	ENSMUSG00000035047	ENSMUSG00000035047	9	21023864	21038363	158	181
7711	19	10538139	10540655	ENSMUSG00000066860	ENSMUSG00000066860	ENSMUSG00000066860	9	21038861	21041429	158	181
7712	19	10544348	10558991	ENSMUSG00000003309	ENSMUSG00000003309	ENSMUSG00000003309	9	21045863	21062713	158	181
7713	19	10597229	10616233	ENSMUSG00000057193	ENSMUSG00000057193	ENSMUSG00000057193	9	21088246	21105427	158	181
7714	19	10625988	10664095	ENSMUSG00000032178	ENSMUSG00000032178	ENSMUSG00000032178	9	21138514	21155752	158	181
7715	19	10689755	10803579	ENSMUSG00000033335	ENSMUSG00000033335	ENSMUSG00000033335	9	21175373	21257528	158	181
7716	19	10804115	10807983	ENSMUSG00000032180	ENSMUSG00000032180	ENSMUSG00000032180	9	21257782	21260588	158	181
7717	19	10843253	10894448	ENSMUSG00000032185	ENSMUSG00000032185	ENSMUSG00000032185	9	21297380	21339862	158	181
7718	19	10894446	10900357	ENSMUSG00000032182	ENSMUSG00000032182	ENSMUSG00000032182	9	21339084	21343148	158	181
7719	19	10900466	10905191	ENSMUSG00000048429	ENSMUSG00000048429	ENSMUSG00000048429	9	21343124	21346356	158	181
7720	19	10932606	11033953	ENSMUSG00000032187	ENSMUSG00000032187	ENSMUSG00000032187	9	21366571	21453769	158	181
7721	19	11061155	11103838	ENSMUSG00000032193	ENSMUSG00000032193	ENSMUSG00000032193	9	21474033	21500318	158	181
7722	19	11118859	11127452	ENSMUSG00000074476	ENSMUSG00000074476	ENSMUSG00000074476	9	21507520	21510680	158	181
7723	19	11137266	11169197	ENSMUSG00000032194	ENSMUSG00000032194	ENSMUSG00000032194	9	21517191	21548937	158	181
7724	19	11170973	11234128	ENSMUSG00000032198	ENSMUSG00000032198	ENSMUSG00000032198	9	21551699	21597151	158	181
7725	19	11209819	11213618	ENSMUSG00000047822	ENSMUSG00000047822	ENSMUSG00000047822	9	21585962	21587748	158	181
7726	19	11296094	11311321	ENSMUSG00000019066	ENSMUSG00000019066	ENSMUSG00000019066	9	21657912	21668523	158	181
7727	19	11321625	11326422	ENSMUSG00000006241	ENSMUSG00000006241	ENSMUSG00000006241	9	21678110	21686032	158	181
7728	19	11327107	11337372	ENSMUSG00000040563	ENSMUSG00000040563	ENSMUSG00000040563	9	21687435	21699302	158	181
7729	19	11346383	11348627	ENSMUSG00000051238	ENSMUSG00000051238	ENSMUSG00000051238	9	21706164	21708665	158	181
7730	19	11348883	11355883	ENSMUSG00000006235	ENSMUSG00000006235	ENSMUSG00000006235	9	21709306	21713908	158	181
7731	19	11365735	11391006	ENSMUSG00000040146	ENSMUSG00000040146	ENSMUSG00000040146	9	21721929	21739855	158	181
7732	19	11392273	11406901	ENSMUSG00000039632	ENSMUSG00000039632	ENSMUSG00000039632	9	21740274	21753036	158	181
7733	19	11407269	11422783	ENSMUSG00000003402	ENSMUSG00000003402	ENSMUSG00000003402	9	21753437	21764624	158	181
7734	19	11423143	11452803	ENSMUSG00000003410	ENSMUSG00000003410	ENSMUSG00000003410	9	21768905	21787271	158	181
7735	19	11455246	11477654	ENSMUSG00000038895	ENSMUSG00000038895	ENSMUSG00000038895	9	21805862	21821747	158	181
7736	19	11477744	11500972	ENSMUSG00000066839	ENSMUSG00000066839	ENSMUSG00000066839	9	21822648	21835806	158	181
7737	19	11510579	11522138	ENSMUSG00000001349	ENSMUSG00000001349	ENSMUSG00000001349	9	21849683	21860032	158	181
7738	19	11546506	11550801	ENSMUSG00000001348	ENSMUSG00000001348	ENSMUSG00000001348	9	21877422	21880531	158	181
7739	7	36395957	36459923	ENSMUSG00000036777	ENSMUSG00000036777	ENSMUSG00000036777	9	22082414	22139625	159	182
7740	7	36330364	36373289	ENSMUSG00000036411	ENSMUSG00000036411	ENSMUSG00000036411	9	22174490	22195083	159	182
7741	7	33100937	33115527	ENSMUSG00000032239	ENSMUSG00000032239	ENSMUSG00000032239	9	22198729	22218739	160	183
7742	7	33135677	33612205	ENSMUSG00000035919	ENSMUSG00000035919	ENSMUSG00000035919	9	22226108	22638685	160	183
7743	7	33911048	34162009	ENSMUSG00000031963	ENSMUSG00000031963	ENSMUSG00000031963	9	22973478	23235604	160	183
7744	7	34664546	34855805	ENSMUSG00000043659	ENSMUSG00000043659	ENSMUSG00000043659	9	23848425	24066805	160	183
7745	7	34937713	35019749	ENSMUSG00000043067	ENSMUSG00000043067	ENSMUSG00000043067	9	24162190	24225821	160	183
7746	7	35208570	35259756	ENSMUSG00000031965	ENSMUSG00000031965	ENSMUSG00000031965	9	24473611	24524709	160	183
7747	7	35638797	35701254	ENSMUSG00000008429	ENSMUSG00000008429	ENSMUSG00000008429	9	24858533	24901918	160	183
7748	7	35807190	35911440	ENSMUSG00000001833	ENSMUSG00000001833	ENSMUSG00000001833	9	25002201	25058011	160	183
7749	7	36159361	36307677	ENSMUSG00000036611	ENSMUSG00000036611	ENSMUSG00000036611	9	25230993	25353547	160	183
7750	11	133753610	133787022	ENSMUSG00000045994	ENSMUSG00000045994	ENSMUSG00000045994	9	26483270	26512541	161	184
7751	11	133707166	133751055	ENSMUSG00000036395	ENSMUSG00000036395	ENSMUSG00000036395	9	26512490	26555863	161	184
7752	11	133652916	133694052	ENSMUSG00000031966	ENSMUSG00000031966	ENSMUSG00000031966	9	26567476	26610101	161	184
7753	11	133628617	133640954	ENSMUSG00000031969	ENSMUSG00000031969	ENSMUSG00000031969	9	26723581	26748992	161	184
7754	11	133623383	133628470	ENSMUSG00000035443	ENSMUSG00000035443	ENSMUSG00000035443	9	26749168	26756780	161	184
7755	11	133599709	133622894	ENSMUSG00000031988	ENSMUSG00000031988	ENSMUSG00000031988	9	26757213	26779503	161	184
7756	11	133527547	133599636	ENSMUSG00000035024	ENSMUSG00000035024	ENSMUSG00000035024	9	26780216	26844753	161	184
7757	11	133444030	133526846	ENSMUSG00000031990	ENSMUSG00000031990	ENSMUSG00000031990	9	26846831	26904839	161	184
7758	11	133294144	133302069	ENSMUSG00000034275	ENSMUSG00000034275	ENSMUSG00000034275	9	27076386	27084209	161	184
7759	11	133215727	133220602	ENSMUSG00000031991	ENSMUSG00000031991	ENSMUSG00000031991	9	27146267	27151157	161	184
7760	11	131795297	132318197	ENSMUSG00000062257	ENSMUSG00000062257	ENSMUSG00000062257	9	27540832	28669633	161	184
7761	11	130745583	131711926	ENSMUSG00000059974	ENSMUSG00000059974	ENSMUSG00000059974	9	28745980	29712372	161	184
7762	11	130250985	130291572	ENSMUSG00000031993	ENSMUSG00000031993	ENSMUSG00000031993	9	30176675	30216285	161	184
7763	11	129824079	129848926	ENSMUSG00000033453	ENSMUSG00000033453	ENSMUSG00000033453	9	30648718	30672017	161	184
7764	11	129780030	129803623	ENSMUSG00000031994	ENSMUSG00000031994	ENSMUSG00000031994	9	30692127	30712421	161	184
7765	11	129606412	129689696	ENSMUSG00000047412	ENSMUSG00000047412	ENSMUSG00000047412	9	30802861	30818436	161	184
7766	11	129534892	129585466	ENSMUSG00000031995	ENSMUSG00000031995	ENSMUSG00000031995	9	30838155	30881364	161	184
7767	11	129445011	129519910	ENSMUSG00000031996	ENSMUSG00000031996	ENSMUSG00000031996	9	30899122	30961356	161	184
7768	11	129239573	129268116	ENSMUSG00000042185	ENSMUSG00000042185	ENSMUSG00000042185	9	31135770	31170896	161	184
7769	11	129190951	129235108	ENSMUSG00000041737	ENSMUSG00000041737	ENSMUSG00000041737	9	31175761	31213803	161	184
7770	11	128344012	128539450	ENSMUSG00000041444	ENSMUSG00000041444	ENSMUSG00000041444	9	31957765	32010849	161	184
7771	11	128266517	128293159	ENSMUSG00000032034	ENSMUSG00000032034	ENSMUSG00000032034	9	32064348	32093802	161	184
7772	11	128213125	128217573	ENSMUSG00000041248	ENSMUSG00000041248	ENSMUSG00000041248	9	32122162	32147120	161	184
7773	11	128069199	128186093	ENSMUSG00000016087	ENSMUSG00000016087	ENSMUSG00000016087	9	32173341	32226353	161	184
7774	11	127833874	127897371	ENSMUSG00000032035	ENSMUSG00000032035	ENSMUSG00000032035	9	32445607	32507385	161	184
7775	11	125781603	125815962	ENSMUSG00000032038	ENSMUSG00000032038	ENSMUSG00000032038	9	34796247	34866481	161	184
7776	11	125678857	125720854	ENSMUSG00000032040	ENSMUSG00000032040	ENSMUSG00000032040	9	34874083	34925686	161	184
7777	11	125658212	125672683	ENSMUSG00000032041	ENSMUSG00000032041	ENSMUSG00000032041	9	34938328	34940755	161	184
7778	11	125644251	125653229	ENSMUSG00000039048	ENSMUSG00000039048	ENSMUSG00000039048	9	34953879	34960596	161	184
7779	11	125638052	125643993	ENSMUSG00000032042	ENSMUSG00000032042	ENSMUSG00000032042	9	34960874	34966674	161	184
7780	11	125586894	125637702	ENSMUSG00000050471	ENSMUSG00000050471	ENSMUSG00000050471	9	34966691	35017448	161	184
7781	11	125577534	125586758	ENSMUSG00000032044	ENSMUSG00000032044	ENSMUSG00000032044	9	35017536	35025633	161	184
7782	11	125331926	125438397	ENSMUSG00000038119	ENSMUSG00000038119	ENSMUSG00000038119	9	35201747	35254014	161	184
7783	11	125279550	125298213	ENSMUSG00000032101	ENSMUSG00000032101	ENSMUSG00000032101	9	35291496	35308135	161	184
7784	11	125268591	125278315	ENSMUSG00000032103	ENSMUSG00000032103	ENSMUSG00000032103	9	35309137	35316854	161	184
7785	11	125151268	125153924	ENSMUSG00000074452	ENSMUSG00000074452	ENSMUSG00000074452	9	35419310	35439648	161	184
7786	11	125047440	125056152	ENSMUSG00000032110	ENSMUSG00000032110	ENSMUSG00000032110	9	36442891	36448508	161	184
7787	11	125001547	125030847	ENSMUSG00000032113	ENSMUSG00000032113	ENSMUSG00000032113	9	36458153	36476298	161	184
7788	11	124967963	124996478	ENSMUSG00000032116	ENSMUSG00000032116	ENSMUSG00000032116	9	36481044	36517325	161	184
7789	11	124944508	124959784	ENSMUSG00000062762	ENSMUSG00000062762	ENSMUSG00000062762	9	36528824	36546739	161	184
7790	11	124820867	124871373	ENSMUSG00000032118	ENSMUSG00000032118	ENSMUSG00000032118	9	36582480	36640423	161	184
7791	11	124539812	124808492	ENSMUSG00000035934	ENSMUSG00000035934	ENSMUSG00000035934	9	36640651	36896985	161	184
7792	11	124472173	124486719	ENSMUSG00000032121	ENSMUSG00000032121	ENSMUSG00000032121	9	36958004	36972882	161	184
7793	11	124438442	124464341	ENSMUSG00000032122	ENSMUSG00000032122	ENSMUSG00000032122	9	36979987	37005085	161	184
7794	11	124329839	124413678	ENSMUSG00000034303	ENSMUSG00000034303	ENSMUSG00000034303	9	37029279	37098054	161	184
7795	11	124294299	124311156	ENSMUSG00000046240	ENSMUSG00000046240	ENSMUSG00000046240	9	37117277	37136246	161	184
7796	11	124258797	124272939	ENSMUSG00000032125	ENSMUSG00000032125	ENSMUSG00000032125	9	37151717	37163693	161	184
7797	11	124240492	124261676	ENSMUSG00000032128	ENSMUSG00000032128	ENSMUSG00000032128	9	37165833	37182672	161	184

7798	11	124141634	124175495	ENSMUSG00000042138	ENSMUSG00000042138	ENSMUSG00000042138	9	37238992	37273836	161	184
7799	11	124128240	124137396	ENSMUSG00000001946	ENSMUSG00000001946	ENSMUSG00000001946	9	37277775	37287983	161	184
7800	11	124122581	124127378	ENSMUSG00000001943	ENSMUSG00000001943	ENSMUSG00000001943	9	37288965	37293813	161	184
7801	11	124115051	124122314	ENSMUSG00000053310	ENSMUSG00000053310	ENSMUSG00000053310	9	37294164	37302480	161	184
7802	11	124048950	124069895	ENSMUSG00000001948	ENSMUSG00000001948	ENSMUSG00000001948	9	37352969	37363285	161	184
7803	11	124010904	124048973	ENSMUSG00000001942	ENSMUSG00000001942	ENSMUSG00000001942	9	37363518	37397989	161	184
7804	11	123997986	124007529	ENSMUSG00000011114	ENSMUSG00000011114	ENSMUSG00000011114	9	37398853	37406983	161	184
7805	11	123986663	123995462	ENSMUSG00000011118	ENSMUSG00000011118	ENSMUSG00000011118	9	37409573	37418893	161	184
7806	11	123945175	123946155	ENSMUSG00000061165	ENSMUSG00000061165	ENSMUSG00000061165	9	37461019	37480652	161	184
7807	11	123815256	123816191	ENSMUSG00000066748	ENSMUSG00000066748	ENSMUSG00000066748	9	37647077	37648009	161	184
7808	11	123799048	123799977	ENSMUSG00000066747	ENSMUSG00000066747	ENSMUSG00000066747	9	37668315	37669259	161	184
7809	11	123561187	123562125	ENSMUSG00000050853	ENSMUSG00000050853	ENSMUSG00000050853	9	39300502	39301440	161	184
7810	11	123370080	123371078	ENSMUSG00000046678	ENSMUSG00000046678	ENSMUSG00000046678	9	39772966	39773898	161	184
7811	11	123352613	123353608	ENSMUSG00000049010	ENSMUSG00000049010	ENSMUSG00000049010	9	39824868	39825833	161	184
7812	11	123315534	123316490	ENSMUSG00000045812	ENSMUSG00000045812	ENSMUSG00000045812	9	39851115	39852059	161	184
7813	11	123282349	123283293	ENSMUSG00000049073	ENSMUSG00000049073	ENSMUSG00000049073	9	39877595	39878530	161	184
7814	11	123258843	123261550	ENSMUSG00000040541	ENSMUSG00000040541	ENSMUSG00000040541	9	39898693	39901445	161	184
7815	11	123129498	123130436	ENSMUSG00000051095	ENSMUSG00000051095	ENSMUSG00000051095	9	39937688	39938626	161	184
7816	11	123100207	123117573	ENSMUSG00000025602	ENSMUSG00000025602	ENSMUSG00000025602	9	39942897	39963762	161	184
7817	11	123005107	123030165	ENSMUSG00000049281	ENSMUSG00000049281	ENSMUSG00000049281	9	40019788	40042188	161	184
7818	11	122901554	122998567	ENSMUSG00000040111	ENSMUSG00000040111	ENSMUSG00000040111	9	40048478	40206335	161	184
7819	11	122448245	122571217	ENSMUSG00000032024	ENSMUSG00000032024	ENSMUSG00000032024	9	40437063	40536231	161	184
7820	11	122353567	122357589	ENSMUSG00000054360	ENSMUSG00000054360	ENSMUSG00000054360	9	40625196	40629041	161	184
7821	11	122258683	122335639	ENSMUSG00000032023	ENSMUSG00000032023	ENSMUSG00000032023	9	40645943	40668608	161	184
7822	11	122214416	122248544	ENSMUSG00000032021	ENSMUSG00000032021	ENSMUSG00000032021	9	40723865	40755655	161	184
7823	11	122031640	122190390	ENSMUSG00000032020	ENSMUSG00000032020	ENSMUSG00000032020	9	40764710	40908563	161	184
7824	11	120828159	121005597	ENSMUSG00000049313	ENSMUSG00000049313	ENSMUSG00000049313	9	41719598	41875108	161	184
7825	11	120668690	120683431	ENSMUSG00000032018	ENSMUSG00000032018	ENSMUSG00000032018	9	42005172	42015251	161	184
7826	11	120478585	120566725	ENSMUSG00000037705	ENSMUSG00000037705	ENSMUSG00000037705	9	42080617	42150924	161	184
7827	11	120400040	120463333	ENSMUSG00000037287	ENSMUSG00000037287	ENSMUSG00000037287	9	42164413	42212397	161	184
7828	11	120036238	120362179	ENSMUSG00000032017	ENSMUSG00000032017	ENSMUSG00000032017	9	42271407	42559632	161	184
7829	11	119713156	119865855	ENSMUSG00000059495	ENSMUSG00000059495	ENSMUSG00000059495	9	42720709	42856205	161	184
7830	11	119701226	119706434	ENSMUSG00000048503	ENSMUSG00000048503	ENSMUSG00000048503	9	42859648	42867565	161	184
7831	11	119616256	119695863	ENSMUSG00000032015	ENSMUSG00000032015	ENSMUSG00000032015	9	42875866	42956750	161	184
7832	11	119586957	119605859	ENSMUSG00000032014	ENSMUSG00000032014	ENSMUSG00000032014	9	42972273	42990811	161	184
7833	11	119487205	119513949	ENSMUSG00000032013	ENSMUSG00000032013	ENSMUSG00000032013	9	43061843	43087109	161	184
7834	11	119040667	119104645	ENSMUSG00000032012	ENSMUSG00000032012	ENSMUSG00000032012	9	43495571	43558456	161	184
7835	11	118794096	118799181	ENSMUSG00000032011	ENSMUSG00000032011	ENSMUSG00000032011	9	43796949	43799574	161	184
7836	11	118732163	118757575	ENSMUSG00000032010	ENSMUSG00000032010	ENSMUSG00000032010	9	43818134	43844928	161	184
7837	11	118714862	118722593	ENSMUSG00000034739	ENSMUSG00000034739	ENSMUSG00000034739	9	43852837	43860182	161	184
7838	11	118684444	118693050	ENSMUSG00000032135	ENSMUSG00000032135	ENSMUSG00000032135	9	43885666	43893719	161	184
7839	11	118582200	118684066	ENSMUSG00000034342	ENSMUSG00000034342	ENSMUSG00000034342	9	43900257	43985041	161	184
7840	11	118566175	118572689	ENSMUSG00000070306	ENSMUSG00000070306	ENSMUSG00000070306	9	43991829	43997880	161	184
7841	11	118561419	118566142	ENSMUSG00000032105	ENSMUSG00000032105	ENSMUSG00000032105	9	43998313	44002459	161	184
7842	11	118544648	118559933	ENSMUSG00000032109	ENSMUSG00000032109	ENSMUSG00000032109	9	44003712	44016866	161	184
7843	11	118524960	118538582	ENSMUSG00000032131	ENSMUSG00000032131	ENSMUSG00000032131	9	44024185	44039239	161	184
7844	11	118497498	118510974	ENSMUSG00000032119	ENSMUSG00000032119	ENSMUSG00000032119	9	44045635	44056629	161	184
7845	11	118483303	118493036	ENSMUSG00000032120	ENSMUSG00000032120	ENSMUSG00000032120	9	44061211	44071280	161	184
7846	11	118472427	118484251	ENSMUSG00000032123	ENSMUSG00000032123	ENSMUSG00000032123	9	44077840	44084595	161	184
7847	11	118469799	118471369	ENSMUSG00000049932	ENSMUSG00000049932	ENSMUSG00000049932	9	44085689	44087066	161	184
7848	11	118443745	118457896	ENSMUSG00000032127	ENSMUSG00000032127	ENSMUSG00000032127	9	44099087	44112626	161	184
7849	11	118420110	118433122	ENSMUSG00000032115	ENSMUSG00000032115	ENSMUSG00000032115	9	44130538	44143356	161	184
7850	11	118400276	118405289	ENSMUSG00000032114	ENSMUSG00000032114	ENSMUSG00000032114	9	44149171	44153963	161	184
7851	11	118394352	118399585	ENSMUSG00000032112	ENSMUSG00000032112	ENSMUSG00000032112	9	44154754	44158543	161	184
7852	11	118374062	118391665	ENSMUSG00000043923	ENSMUSG00000043923	ENSMUSG00000043923	9	44161152	44168998	161	184
7853	11	118332236	118334478	ENSMUSG00000041523	ENSMUSG00000041523	ENSMUSG00000041523	9	44203720	44205762	161	184
7854	11	118272263	118286823	ENSMUSG00000063382	ENSMUSG00000063382	ENSMUSG00000063382	9	44250131	44261405	161	184
7855	11	118259777	118272180	ENSMUSG00000047880	ENSMUSG00000047880	ENSMUSG00000047880	9	44262778	44277416	161	184
7856	11	118034209	118055592	ENSMUSG00000032098	ENSMUSG00000032098	ENSMUSG00000032098	9	44424233	44437299	161	184
7857	11	117982423	118033951	ENSMUSG00000048537	ENSMUSG00000048537	ENSMUSG00000048537	9	44437304	44479074	161	184
7858	11	117920471	117941960	ENSMUSG00000002031	ENSMUSG00000002031	ENSMUSG00000002031	9	44524004	44543709	161	184
7859	11	117907134	117911757	ENSMUSG00000002032	ENSMUSG00000002032	ENSMUSG00000002032	9	44544774	44549777	161	184
7860	11	117903420	117906841	ENSMUSG00000039438	ENSMUSG00000039438	ENSMUSG00000039438	9	44550395	44553952	161	184
7861	11	117812415	117901146	ENSMUSG00000002028	ENSMUSG00000002028	ENSMUSG00000002028	9	44555652	44631971	161	184
7862	11	117735512	117775132	ENSMUSG00000059890	ENSMUSG00000059890	ENSMUSG00000059890	9	44674126	44716555	161	184
7863	11	117720317	117729979	ENSMUSG00000002033	ENSMUSG00000002033	ENSMUSG00000002033	9	44720683	44731421	161	184
7864	11	117715001	117718669	ENSMUSG00000032094	ENSMUSG00000032094	ENSMUSG00000032094	9	44732876	44737418	161	184
7865	11	117680662	117692096	ENSMUSG00000032093	ENSMUSG00000032093	ENSMUSG00000032093	9	44749738	44760585	161	184
7866	11	117629347	117640219	ENSMUSG00000032092	ENSMUSG00000032092	ENSMUSG00000032092	9	44793561	44805020	161	184
7867	11	117602619	117628245	ENSMUSG00000070305	ENSMUSG00000070305	ENSMUSG00000070305	9	44806185	44816410	161	184
7868	11	117569891	117600939	ENSMUSG00000048534	ENSMUSG00000048534	ENSMUSG00000048534	9	44830178	44858846	161	184
7869	11	117538729	117552546	ENSMUSG00000070304	ENSMUSG00000070304	ENSMUSG00000070304	9	44868876	44881067	161	184
7870	11	117509302	117528747	ENSMUSG00000046480	ENSMUSG00000046480	ENSMUSG00000046480	9	44890037	44905056	161	184
7871	11	117452976	117494380	ENSMUSG00000032091	ENSMUSG00000032091	ENSMUSG00000032091	9	44924392	44941157	161	184
7872	11	117362319	117377404	ENSMUSG00000032089	ENSMUSG00000032089	ENSMUSG00000032089	9	45004833	45020131	161	184
7873	11	117278987	117305306	ENSMUSG00000037129	ENSMUSG00000037129	ENSMUSG00000037129	9	45070095	45098579	161	184
7874	11	117198230	117200644	ENSMUSG00000059412	ENSMUSG00000059412	ENSMUSG00000059412	9	45150213	45161273	161	184
7875	11	116804254	117173184	ENSMUSG00000032087	ENSMUSG00000032087	ENSMUSG00000032087	9	45181462	45453611	161	184
7876	11	116661627	116692182	ENSMUSG00000032086	ENSMUSG00000032086	ENSMUSG00000032086	9	45589578	45613436	161	184
7877	11	116610190	116661263	ENSMUSG00000042790	ENSMUSG00000042790	ENSMUSG00000042790	9	45614686	45657872	161	184
7878	11	116581020	116608021	ENSMUSG00000035382	ENSMUSG00000035382	ENSMUSG00000035382	9	45657981	45680652	161	184
7879	11	116575250	116580717	ENSMUSG00000032085	ENSMUSG00000032085	ENSMUSG00000032085	9	45680623	45686893	161	184
7880	11	116555107	116573370	ENSMUSG00000034908	ENSMUSG00000034908	ENSMUSG00000034908	9	45688868	45706242	161	184
7881	11	116528374	116543625	ENSMUSG00000003131	ENSMUSG00000003131	ENSMUSG00000003131	9	45716306	45735866	161	184
7882	11	116219328	116474347	ENSMUSG00000034135	ENSMUSG00000034135	ENSMUSG00000034135	9	45764019	45975181	161	184
7883	11	116211677	116213876	ENSMUSG00000032083	ENSMUSG00000032083	ENSMUSG00000032083	9	45979952	45981463	161	184
7884	11	116205841	116208998	ENSMUSG00000032081	ENSMUSG00000032081	ENSMUSG00000032081	9	45984046	45986292	161	184
7885	11	116196628	116199221	ENSMUSG00000032080	ENSMUSG00000032080	ENSMUSG00000032080	9	45992057	45994453	161	184
7886	11	116165293	116167821	ENSMUSG00000032079	ENSMUSG00000032079	ENSMUSG00000032079	9	46020073	46022914	161	184
7887	11	116153646	116163976	ENSMUSG00000032078	ENSMUSG00000032078	ENSMUSG00000032078	9	46024176	46032599	161	184
7888	11	116124096	116148914	ENSMUSG00000032077	ENSMUSG00000032077	ENSMUSG00000032077	9	46034003	46049774	161	184
7889	11	114552404	114880322	ENSMUSG00000032076	ENSMUSG00000032076	ENSMUSG00000032076	9	47281368	47601384	161	184
7890	11	113946864	113971610	ENSMUSG00000044229	ENSMUSG00000044229	ENSMUSG00000044229	9	47913968	48151015	161	184
7891	11	114055563	114082859	ENSMUSG00000032028	ENSMUSG00000032028	ENSMUSG00000032028	9	48069951	48092742	161	184
7892	11	113897648	113935790	ENSMUSG00000074362	ENSMUSG00000074362	ENSMUSG00000074362	9	48172157	48183906	161	184
7893	11	113815362	113826211	ENSMUSG00000032026	ENSMUSG00000032026	ENSMUSG00000032026	9	48220481	48232578	161	184
7894	11	113776566	113783739	ENSMUSG00000042396	ENSMUSG00000042396	ENSMUSG00000042396	9	48240676	48247272	161	184
7895	11	113767380	113776349	ENSMUSG00000042293	ENSMUSG00000042293	ENSMUSG00000042293	9	48247409	48247938	161	184
7896	11	113633763	113688448	ENSMUSG00000032271	ENSMUSG00000032271	ENSMUSG00000032271	9	48343844	48357120	161	184
7897	11	113435525	113626562	ENSMUSG00000066687	ENSMUSG00000066687	ENSMUSG00000066687	9	48406264	48587864	161	184
7898	11	113351040	113366241	ENSMUSG00000032269	ENSMUSG00000032269	ENSMUSG00000032269	9	48651624	48662908	161	184
7899	11	113280729	113322492	ENSMUSG00000008590	ENSMUSG00000008590	ENSMUSG00000008590	9	48686940	48716922	161	184
7900	11	113173808	113251466	ENSMUSG00000032267	ENSMUSG00000032267	ENSMUSG00000032267	9	48737317	48794449	161	184
7901	11	113109123	113149635	ENSMUSG00000032264	ENSMUSG00000032264	ENSMUSG00000032264	9	48807564	48830707	161	184
7902	11	113063483	113082305	ENSMUSG00000032268	ENSMUSG00000032268	ENSMUSG00000032268	9	48858046	48867555	161	184
7903	11	112785528	112851103	ENSMUSG00000032259	ENSMUSG00000032259	ENSMUSG00000032259	9	49092857	49160407	161	184
7904	11	112763723	112776199	ENSMUSG00000032257	ENSMUSG00000032257	ENSMUSG00000032257	9	49167870	49179251	161	184
7905	11	112692171	112749226	ENSMUSG00000040219	ENSMUSG00000040219	ENSMUSG00000040219	9	49189191	49238455	161	184
7906	11	111628293	111636522	ENSMUSG00000032068	ENSMUSG00000032068	ENSMUSG00000032068	9	50246755	50257363	161	184
7907	11	111602309	111609903	ENSMUSG00000032067	ENSMUSG00000032067	ENSMUSG00000032067	9	50273848	50280880	161	184
7908	11	111551443	111594853	ENSMUSG00000032066	ENSMUSG00000032066	ENSMUSG00000032066	9	50285382	50302932	161	184
7909	11	111543305	111547849	ENSMUSG00000032065	ENSMUSG00000032065	ENSMUSG00000032065	9	50309577	50313555	161	184
7910	11	111519186	111540050	ENSMUSG00000039217	ENSMUSG00000039217	ENSMUSG00000039217	9	50327503	50334067	161	184
7911	11	111462832	111471727	ENSMUSG00000000171	ENSMUSG00000000171	ENSMUSG00000000171	9	50348576	50356047	161	184
7912	11	111461083	111462690	ENSMUSG00000039016	ENSMUSG00000039016	ENSMUSG00000039016	9	50356143	50366422	161	184
7913	11	111443774	111449914	ENSMUSG00000000167	ENSMUSG00000000167	ENSMUSG00000000167	9	50369566	50377230	161	184
7914	11	111401381	111439798	ENSMUSG00000000168	ENSMUSG00000000168	ENSMUSG00000000168	9	50386865	50412010	161	184
7915	11	111313434	111394967	ENSMUSG00000032064	ENSMUSG00000032064	ENSMUSG00000032064	9	50414982	50480205	161	184
7916	11	111294847	111302798	ENSMUSG00000032062	ENSMUSG00000032062	ENSMUSG00000032062	9	50491921	50498751	161	184
7917	11	111288176	111290026	ENSMUSG00000038086	ENSMUSG00000038086	ENSMUSG00000038086	9	50503308	50504523	161	184
7918	11	111284560	111287704	ENSMUSG00000032060	ENSMUSG00000032060	ENSMUSG00000032060	9	50503555	50508860	161	184
7919	11	111255475	111261774	ENSMUSG00000037971	ENSMUSG00000037971	ENSMUSG00000037971	9	50515933	50518163	161	184
7920	11	111250134	111255070	ENSMUSG00000037845	ENSMUSG00000037845	ENSMUSG00000037845	9	50520468	50524900	161	184
7921	11	111162220	111247416	ENSMUSG00000032059	ENSMUSG00000032059	ENSMUSG00000032059	9	50527516	50595741	161	184
7922	11	111102848	111142345	ENSMUSG00000032058	ENSMUSG00000032058	ENSMUSG00000032058	9	50609165	50646459	161	184
7923	11	110978380	111102840	ENSMUSG00000037112	ENSMUSG00000037112	ENSMUSG00000037112	9	50645029	50761303	161	184
7924	11	110916644	110936393	ENSMUSG00000060594	ENSMUSG00000060594	ENSMUSG00000060594	9	50814414	50829200	161	184
7925	11	110890720	110912964	ENSMUSG00000032057	ENSMUSG00000032057	ENSMUSG00000032057	9	50833345	50854196	161	184
7926	11	110843465	110888274	ENSMUSG00000032056	ENSMUSG00000032056	ENSMUSG00000032056	9	50868231	50871937	161	184
7927	11	110631917	110662180	ENSMUSG00000036027	ENSMUSG00000036027	ENSMUSG00000036027	9	51042277	51044079	161	184
7928	11	109952976	110088661	ENSMUSG00000053199	ENSMUSG00000053199	ENSMUSG00000053199	9	51517717	51605424	161	184
7929	11	109805804	109840814	ENSMUSG00000032051	ENSMUSG00000032051	ENSMUSG00000032051	9	51695672	51715911	161	184
7930	11	109605378	109672647	ENSMUSG00000032050	ENSMUSG00000032050	ENSMUSG00000032050	9	51799393	51841094	161	184
7931	11	109511789	109547774	ENSMUSG00000035164	ENSMUSG00000035164	ENSMUSG00000035164	9	51867750	51879030	161	184
7932	11	108798136	108801618	ENSMUSG00000050570	ENSMUSG00000050570	ENSMUSG00000050570	9	52438679	52441832	161	184
7933	11	108041014	108316866	ENSMUSG00000053289	ENSMUSG00000053289	ENSMUSG00000053289	9	52861036	53010486	161	184
7934	11	107884893	107969570	ENSMUSG00000034584	ENSMUSG00000034584	ENSMUSG00000034584	9	53064065	53139909	161	184
7935	11	107848062	107874323	ENSMUSG00000034487	ENSMUSG00000034487	ENSMUSG00000034487	9	53146418	53164262	161	184
7936	11	107707140	107843438	ENSMUSG00000005131	ENSMUSG00000005131	ENSMUSG00000005131	9	53167681	53195198	161	184
7937	11	107598769	107745036	ENSMUSG00000034218	ENSMUSG00000034218	ENSMUSG00000034218	9	53201763	53296362	161	184
7938	11	107534879	107598539	ENSMUSG00000033054	ENSMUSG00000033054	ENSMUSG00000033054	9	53299442	53335351	161	184
7939	11	107497468	107523485	ENSMUSG00000032047	ENSMUSG00000032047	ENSMUSG00000032047	9	53342917	53372745	161	184
7940	11	107384618	107483676	ENSMUSG00000032030	ENSMUSG00000032030	ENSMUSG00000032030	9	53379265	53421096	161	184
7941	11	107304487	107339416	ENSMUSG00000055069	ENSMUSG00000055069	ENSMUSG00000055069	9	53446505	53468627	161	184
7942	11	107166930	107234864	ENSMUSG00000042195	ENSMUSG00000042195	ENSMUSG00000042195	9	53569675	53616308	161	184
7943	11	106967284	107042714	ENSMUSG00000041986	ENSMUSG00000041986	ENSMUSG00000041986	9	53709597	53773438	161	184
7944	15	49136093	49184765	ENSMUSG00000074345	ENSMUSG00000074345	ENSMUSG00000074345	9	53825212	53825775	162	185
7945	15	49288964	49403413	ENSMUSG00000032274	ENSMUSG00000032274	ENSMUSG00000032274	9	53964894	53991538	162	185
7946	15	49421169	49485509	ENSMUSG00000046167	ENSMUSG00000046167	ENSMUSG00000046167	9	54084623	54139914	162	185
7947	15	49528181	49702241	ENSMUSG00000041268	ENSMUSG00000041268	ENSMUSG00000041268	9	54167345	54299574	162	185
7948	15	76177874	76182912	ENSMUSG00000046460	ENSMUSG00000046460	ENSMUSG00000046460	9	54337121	54343157	163	186
7949	15	76184046	76210933	ENSMUSG00000037493	ENSMUSG00000037493	ENSMUSG00000037493	9	54344007	54358155	163	186
7950	15	76228774	76249938	ENSMUSG00000032279	ENSMUSG00000032279	ENSMUSG00000032279	9	54384615	54402797	163	186
7951	15	76246865	76313913	ENSMUSG00000032281	ENSMUSG00000032281	ENSMUSG00000032281	9	54403134	54460022	163	186
7952	15	76343551	76361591	ENSMUSG00000032285	ENSMUSG00000032285	ENSMUSG00000032285	9	54497696	54514449	163	186
7953	15	76362635	76378995	ENSMUSG00000061559	ENSMUSG00000061559	ENSMUSG00000061559	9	54515292	54532665	163	186
7954	15	76419744	76427620	ENSMUSG00000032291	ENSMUSG00000032291	ENSMUSG00000032291	9	54562944	54571246	163	186
7955	15	76517639	76579935	ENSMUSG00000032293	ENSMUSG00000032293	ENSMUSG00000032293	9	54661926	54708169	163	186
7956	15	76592486	76613064	ENSMUSG00000035878	ENSMUSG00000035878	ENSMUSG00000035878	9	54715420	54745619	163	186
7957	15	76644961	76673493	ENSMUSG00000035594	ENSMUSG00000035594	ENSMUSG00000035594	9	54779234	54804712	163	186
7958	15	76674707	76700377	ENSMUSG00000032303	ENSMUSG00000032303	ENSMUSG00000032303	9	54809480	54824696	163	186
7959	15	76703691	76720646	ENSMUSG00000035200	ENSMUSG00000035200	ENSMUSG00000035200	9	54826293	54846727	163	186
7960	15	73983255	74013999	ENSMUSG00000032309	ENSMUSG00000032309	ENSMUSG00000032309	9	55007132	55022570	164	187
7961	15	74213660	74283708	ENSMUSG00000032313	ENSMUSG00000032313	ENSMUSG00000032313	9	55125086	55236481	164	187
7962	15	74295646	74390844	ENSMUSG00000032314	ENSMUSG00000032314	ENSMUSG00000032314	9	55252660	55310380	164	187
7963	15	74416201	74421870	ENSMUSG00000032318	ENSMUSG00000032318	ENSMUSG00000032318	9	55339344	55344316	164	187
7964	15	74427593	74963247	ENSMUSG00000034007	ENSMUSG00000034007	ENSMUSG00000034007	9	55348908	55714241	164	187
7965	15	75011174	75029349	ENSMUSG00000032320	ENSMUSG00000032320	ENSMUSG00000032320	9	55840154	55857214	164	187
7966	15	75074609	75116726	ENSMUSG00000032322	ENSMUSG00000032322	ENSMUSG00000032322	9	55888113	55927013	164	187
7967	15	75537798	75564954	ENSMUSG00000032329	ENSMUSG00000032329	ENSMUSG00000032329	9	56216781	56295021	164	187
7968	15	75693441	75695303	ENSMUSG00000049556	ENSMUSG00000049556	ENSMUSG00000049556	9	56416612	56483390	164	187
7969	15	73803374	73807082	ENSMUSG00000045620	ENSMUSG00000045620	ENSMUSG00000045620	9	56646796	56650100	165	188
7970	15	73728499	73738026	ENSMUSG00000032733	ENSMUSG00000032733	ENSMUSG00000032733	9	56715339	56725182	165	188
7971	15	73677519	73705202	ENSMUSG00000055334	ENSMUSG00000055334	ENSMUSG00000055334	9	56749045	56781338	165	188
7972	15	73546515	73658680	ENSMUSG00000032290	ENSMUSG00000032290	ENSMUSG00000032290	9	56793105	56860945	165	188
7973	15	73450330	73535177	ENSMUSG00000042557	ENSMUSG00000042557	ENSMUSG00000042557	9	56874536	56926495	165	188
7974	15	73435186	73447994	ENSMUSG00000032295	ENSMUSG00000032295	ENSMUSG00000032295	9	56928894	56940343	165	188
7975	15	73428300	73434639	ENSMUSG00000032298	ENSMUSG00000032298	ENSMUSG00000032298	9	56940937	56946266	165	188
7976	15	73415470	73421321	ENSMUSG00000032299	ENSMUSG00000032299	ENSMUSG00000032299	9	56953377	56955275	165	188
7977	15	73278286	73291561	ENSMUSG00000032300	ENSMUSG00000032300	ENSMUSG00000032300	9	57049280	57060736	165	188
7978	15	73102980	73130119	ENSMUSG00000063849	ENSMUSG00000063849	ENSMUSG00000063849	9	57210790	57238251	165	188
7979	15	73074958	73100887	ENSMUSG00000040722	ENSMUSG00000040722	ENSMUSG00000040722	9	57241807	57249625	165	188
7980	15	73035378	73035977	ENSMUSG00000062309	ENSMUSG00000062309	ENSMUSG00000062309	9	57302170	57303576	165	188
7981	15	72999674	73017522	ENSMUSG00000000088	ENSMUSG00000000088	ENSMUSG00000000088	9	57319416	57330561	165	188
7982	15	72982013	72986231	ENSMUSG00000032305	ENSMUSG00000032305	ENSMUSG00000032305	9	57335758	57337029	165	188
7983	15	72969450	72977622	ENSMUSG00000032306	ENSMUSG00000032306	ENSMUSG00000032306	9	57342857	57350421	165	188
7984	15	72924249	72952728	ENSMUSG00000040188	ENSMUSG00000040188	ENSMUSG00000040188	9	57359117	57386932	165	188
7985	15	72915513	72922740	ENSMUSG00000032308	ENSMUSG00000032308	ENSMUSG00000032308	9	57387605	57394363	165	188
7986	15	72892237	72905148	ENSMUSG00000056271	ENSMUSG00000056271	ENSMUSG00000056271	9	57405218	57418832	165	188
7987	15	72861489	72882524	ENSMUSG00000032312	ENSMUSG00000032312	ENSMUSG00000032312	9	57424784	57443375	165	188
7988	15	72799883	72802491	ENSMUSG00000032315	ENSMUSG00000032315	ENSMUSG00000032315	9	57449522	57501958	165	188
7989	15	72828237	72835994	ENSMUSG00000032310	ENSMUSG00000032310	ENSMUSG00000032310	9	57475074	57481792	165	188
7990	15	72709953	72775413	ENSMUSG00000038957	ENSMUSG00000038957	ENSMUSG00000038957	9	57506699	57548297	165	188
7991	15	72698591	72709510	ENSMUSG00000032316	ENSMUSG00000032316	ENSMUSG00000032316	9	57548848	57563997	165	188
7992	15	72620600	72677521	ENSMUSG00000004661	ENSMUSG00000004661	ENSMUSG00000004661	9	57588633	57632373	165	188

7993	15	72525371	72540582	ENSMUSG00000055720	ENSMUSG00000055720	ENSMUSG00000055720	9	57709123	57728107	165	188
7994	15	72489376	72513329	ENSMUSG00000038264	ENSMUSG00000038264	ENSMUSG00000038264	9	57738272	57761002	165	188
7995	15	72417193	72447134	ENSMUSG00000032323	ENSMUSG00000032323	ENSMUSG00000032323	9	57813150	57825158	165	188
7996	15	72346112	72415864	ENSMUSG00000037716	ENSMUSG00000037716	ENSMUSG00000037716	9	57826814	57916881	165	188
7997	15	72258863	72288419	ENSMUSG00000032327	ENSMUSG00000032327	ENSMUSG00000032327	9	57927225	57952090	165	188
7998	15	72253115	72256265	ENSMUSG00000037206	ENSMUSG00000037206	ENSMUSG00000037206	9	57954404	57957279	165	188
7999	15	72208752	72216194	ENSMUSG00000051243	ENSMUSG00000051243	ENSMUSG00000051243	9	57994441	58000275	165	188
8000	15	72062614	72071744	ENSMUSG00000032333	ENSMUSG00000032333	ENSMUSG00000032333	9	58051329	58060655	165	188
8001	15	72005842	72031531	ENSMUSG00000032334	ENSMUSG00000032334	ENSMUSG00000032334	9	58088888	58111023	165	188
8002	15	71953021	71968603	ENSMUSG00000036244	ENSMUSG00000036244	ENSMUSG00000036244	9	58157853	58168592	165	188
8003	15	71819311	71830524	ENSMUSG00000066607	ENSMUSG00000066607	ENSMUSG00000066607	9	58286740	58297879	165	188
8004	15	71763675	71793903	ENSMUSG00000035914	ENSMUSG00000035914	ENSMUSG00000035914	9	58322260	58338882	165	188
8005	15	71639418	71712747	ENSMUSG00000032336	ENSMUSG00000032336	ENSMUSG00000032336	9	58380573	58449452	165	188
8006	15	71522572	71639404	ENSMUSG00000074269	ENSMUSG00000074269	ENSMUSG00000074269	9	58451016	58539694	165	188
8007	15	71400988	71448230	ENSMUSG00000032338	ENSMUSG00000032338	ENSMUSG00000032338	9	58621649	58658900	165	188
8008	15	71131928	71382835	ENSMUSG00000032340	ENSMUSG00000032340	ENSMUSG00000032340	9	58674694	58834562	165	188
8009	15	70831651	70863121	ENSMUSG00000025236	ENSMUSG00000025236	ENSMUSG00000025236	9	59089578	59114206	165	188
8010	15	70765588	70817869	ENSMUSG00000025235	ENSMUSG00000025235	ENSMUSG00000025235	9	59120876	59143857	165	188
8011	15	70554035	70663149	ENSMUSG00000025234	ENSMUSG00000025234	ENSMUSG00000025234	9	59190614	59284620	165	188
8012	15	70477704	70487424	ENSMUSG00000049526	ENSMUSG00000049526	ENSMUSG00000049526	9	59316673	59323507	165	188
8013	15	70423288	70455868	ENSMUSG00000025232	ENSMUSG00000025232	ENSMUSG00000025232	9	59337673	59363111	165	188
8014	15	70320594	70350446	ENSMUSG00000025237	ENSMUSG00000025237	ENSMUSG00000025237	9	59421129	59448032	165	188
8015	15	70278424	70310738	ENSMUSG00000032294	ENSMUSG00000032294	ENSMUSG00000032294	9	59454614	59477381	165	188
8016	15	70239202	70277180	ENSMUSG00000074259	ENSMUSG00000074259	ENSMUSG00000074259	9	59505722	59514037	165	188
8017	15	70193653	70220357	ENSMUSG00000051705	ENSMUSG00000051705	ENSMUSG00000051705	9	59532269	59548644	165	188
8018	15	69905975	70197107	ENSMUSG00000074258	ENSMUSG00000074258	ENSMUSG00000074258	9	59577620	59625329	165	188
8019	15	68972041	69129488	ENSMUSG00000047766	ENSMUSG00000047766	ENSMUSG00000047766	9	60385246	60485917	165	188
8020	15	68910931	68933552	ENSMUSG00000034839	ENSMUSG00000034839	ENSMUSG00000034839	9	60511127	60536807	165	188
8021	15	68733949	68842904	ENSMUSG00000034485	ENSMUSG00000034485	ENSMUSG00000034485	9	60592554	60678376	165	188
8022	15	68129473	68176212	ENSMUSG00000032280	ENSMUSG00000032280	ENSMUSG00000032280	9	61170864	61216487	165	188
8023	15	67493742	67527810	ENSMUSG00000032254	ENSMUSG00000032254	ENSMUSG00000032254	9	61715670	61743436	165	188
8024	15	67439358	67486096	ENSMUSG00000032278	ENSMUSG00000032278	ENSMUSG00000032278	9	61751744	61824796	165	188
8025	15	67240027	67351592	ENSMUSG00000032252	ENSMUSG00000032252	ENSMUSG00000032252	9	61855254	61868612	165	188
8026	15	67009910	67026203	ENSMUSG00000046846	ENSMUSG00000046846	ENSMUSG00000046846	9	62068735	62080185	165	188
8027	15	66658646	66807193	ENSMUSG00000041729	ENSMUSG00000041729	ENSMUSG00000041729	9	62219288	62287447	165	188
8028	15	66381469	66511531	ENSMUSG00000032243	ENSMUSG00000032243	ENSMUSG00000032243	9	62475867	62581975	165	188
8029	15	66357810	66375247	ENSMUSG00000032244	ENSMUSG00000032244	ENSMUSG00000032244	9	62589827	62609608	165	188
8030	15	66286386	66309102	ENSMUSG00000032245	ENSMUSG00000032245	ENSMUSG00000032245	9	62636793	62650008	165	188
8031	15	66273195	66285441	ENSMUSG00000032246	ENSMUSG00000032246	ENSMUSG00000032246	9	62656248	62673580	165	188
8032	15	66165695	66267392	ENSMUSG00000032405	ENSMUSG00000032405	ENSMUSG00000032405	9	62679974	62750209	165	188
8033	15	65904995	65913228	ENSMUSG00000022245	ENSMUSG00000022245	ENSMUSG00000022245	9	62936180	62944986	165	188
8034	15	65622075	65886351	ENSMUSG00000058444	ENSMUSG00000058444	ENSMUSG00000058444	9	62961776	63175858	165	188
8035	15	65334221	65580574	ENSMUSG00000037801	ENSMUSG00000037801	ENSMUSG00000037801	9	63219463	63400486	165	188
8036	15	65280425	65334038	ENSMUSG00000037257	ENSMUSG00000037257	ENSMUSG00000037257	9	63400666	63439897	165	188
8037	15	65145249	65274586	ENSMUSG00000032402	ENSMUSG00000032402	ENSMUSG00000032402	9	63444773	63556000	165	188
8038	15	64781722	64861377	ENSMUSG00000036867	ENSMUSG00000036867	ENSMUSG00000036867	9	63751152	63820065	165	188
8039	15	64627702	64644889	ENSMUSG00000032401	ENSMUSG00000032401	ENSMUSG00000032401	9	63915153	63936124	165	188
8040	15	64584509	64628866	ENSMUSG00000032400	ENSMUSG00000032400	ENSMUSG00000032400	9	63935152	63970937	165	188
8041	15	64573236	64577123	ENSMUSG00000032398	ENSMUSG00000032398	ENSMUSG00000032398	9	63977314	63980694	165	188
8042	15	64466674	64570935	ENSMUSG00000004936	ENSMUSG00000004936	ENSMUSG00000004936	9	63983787	64051430	165	188
8043	15	64416230	64436083	ENSMUSG00000032397	ENSMUSG00000032397	ENSMUSG00000032397	9	64086120	64102476	165	188
8044	15	64372714	64413289	ENSMUSG00000032396	ENSMUSG00000032396	ENSMUSG00000032396	9	64104764	64139172	165	188
8045	15	63974693	64333129	ENSMUSG00000036466	ENSMUSG00000036466	ENSMUSG00000036466	9	64183696	64498960	165	188
8046	15	63948850	63968846	ENSMUSG00000004771	ENSMUSG00000004771	ENSMUSG00000004771	9	64513391	64535665	165	188
8047	15	63740596	63871640	ENSMUSG00000035492	ENSMUSG00000035492	ENSMUSG00000035492	9	64694816	64715594	165	188
8048	15	63703472	63733471	ENSMUSG00000034452	ENSMUSG00000034452	ENSMUSG00000034452	9	64720865	64749613	165	188
8049	15	63658173	63690522	ENSMUSG00000034263	ENSMUSG00000034263	ENSMUSG00000034263	9	64758943	64784714	165	188
8050	15	63609857	63657736	ENSMUSG00000033629	ENSMUSG00000033629	ENSMUSG00000033629	9	64784992	64819719	165	188
8051	15	63526028	63596664	ENSMUSG00000032393	ENSMUSG00000032393	ENSMUSG00000032393	9	64830464	64880661	165	188
8052	15	63460878	63502463	ENSMUSG00000032816	ENSMUSG00000032816	ENSMUSG00000032816	9	64899492	64935946	165	188
8053	15	63407842	63457379	ENSMUSG00000032394	ENSMUSG00000032394	ENSMUSG00000032394	9	64939160	64983863	165	188
8054	15	63337491	63366071	ENSMUSG00000032392	ENSMUSG00000032392	ENSMUSG00000032392	9	64993359	65036436	165	188
8055	15	63275398	63290864	ENSMUSG00000042254	ENSMUSG00000042254	ENSMUSG00000042254	9	65063186	65078611	165	188
8056	15	63229837	63264616	ENSMUSG00000015357	ENSMUSG00000015357	ENSMUSG00000015357	9	65092314	65128663	165	188
8057	15	63196771	63213172	ENSMUSG00000041837	ENSMUSG00000041837	ENSMUSG00000041837	9	65144074	65157649	165	188
8058	15	63156324	63157583	ENSMUSG00000054978	ENSMUSG00000054978	ENSMUSG00000054978	9	65188282	65189541	165	188
8059	15	63132732	63147441	ENSMUSG00000041696	ENSMUSG00000041696	ENSMUSG00000041696	9	65196531	65210711	165	188
8060	15	63124858	63132787	ENSMUSG00000053862	ENSMUSG00000053862	ENSMUSG00000053862	9	65210759	65220779	165	188
8061	15	63081904	63109018	ENSMUSG00000059183	ENSMUSG00000059183	ENSMUSG00000059183	9	65233794	65250420	165	188
8062	15	63042417	63069304	ENSMUSG00000032388	ENSMUSG00000032388	ENSMUSG00000032388	9	65258977	65285461	165	188
8063	15	63001179	63038083	ENSMUSG00000066510	ENSMUSG00000066510	ENSMUSG00000066510	9	65299459	65310755	165	188
8064	15	62921186	62947252	ENSMUSG00000050721	ENSMUSG00000050721	ENSMUSG00000050721	9	65352392	65378046	165	188
8065	15	62894889	62904891	ENSMUSG00000041064	ENSMUSG00000041064	ENSMUSG00000041064	9	65385166	65393970	165	188
8066	15	62766829	62770860	ENSMUSG00000040652	ENSMUSG00000040652	ENSMUSG00000040652	9	65474589	65488303	165	188
8067	15	62578672	62765320	ENSMUSG00000040524	ENSMUSG00000040524	ENSMUSG00000040524	9	65493468	65593333	165	188
8068	15	62467073	62534530	ENSMUSG00000032386	ENSMUSG00000032386	ENSMUSG00000032386	9	65630231	65706792	165	188
8069	15	62244771	62435423	ENSMUSG00000032384	ENSMUSG00000032384	ENSMUSG00000032384	9	65707016	65843020	165	188
8070	15	62235070	62242407	ENSMUSG00000032383	ENSMUSG00000032383	ENSMUSG00000032383	9	65858175	65864635	165	188
8071	15	62230969	62236733	ENSMUSG00000039452	ENSMUSG00000039452	ENSMUSG00000039452	9	65863182	65867737	165	188
8072	15	62175266	62216870	ENSMUSG00000032382	ENSMUSG00000032382	ENSMUSG00000032382	9	65886133	65922892	165	188
8073	15	62151818	62173260	ENSMUSG00000032381	ENSMUSG00000032381	ENSMUSG00000032381	9	65924617	65936974	165	188
8074	15	61986288	62119519	ENSMUSG00000032380	ENSMUSG00000032380	ENSMUSG00000032380	9	65956242	66070246	165	188
8075	15	61687871	61913143	ENSMUSG00000038664	ENSMUSG00000038664	ENSMUSG00000038664	9	66169818	66306327	165	188
8076	15	61676649	61681671	ENSMUSG00000050503	ENSMUSG00000050503	ENSMUSG00000050503	9	66306465	66312582	165	188
8077	15	61583986	61673891	ENSMUSG00000032376	ENSMUSG00000032376	ENSMUSG00000032376	9	66315887	66390986	165	188
8078	15	61402784	61461128	ENSMUSG00000032373	ENSMUSG00000032373	ENSMUSG00000032373	9	66512886	66565991	165	188
8079	15	61268781	61347028	ENSMUSG00000036943	ENSMUSG00000036943	ENSMUSG00000036943	9	66642808	66718831	165	188
8080	15	61233108	61236729	ENSMUSG00000036781	ENSMUSG00000036781	ENSMUSG00000036781	9	66745262	66748655	165	188
8081	15	61201072	61221305	ENSMUSG00000032370	ENSMUSG00000032370	ENSMUSG00000032370	9	66754537	66774628	165	188
8082	15	61122003	61151164	ENSMUSG00000032366	ENSMUSG00000032366	ENSMUSG00000032366	9	66821737	66848357	165	188
8083	15	60726802	60920733	ENSMUSG00000052698	ENSMUSG00000052698	ENSMUSG00000052698	9	67023227	67196791	165	188
8084	15	59931884	60139939	ENSMUSG00000035284	ENSMUSG00000035284	ENSMUSG00000035284	9	67639591	67794550	165	188
8085	15	58576755	58707024	ENSMUSG00000032238	ENSMUSG00000032238	ENSMUSG00000032238	9	68452971	69178125	165	188
8086	15	58499100	58558636	ENSMUSG00000032235	ENSMUSG00000032235	ENSMUSG00000032235	9	69197162	69232242	165	188
8087	15	58084427	58085434	ENSMUSG00000059246	ENSMUSG00000059246	ENSMUSG00000059246	9	69556916	69559991	165	188
8088	15	57742356	57768810	ENSMUSG00000011958	ENSMUSG00000011958	ENSMUSG00000011958	9	69788665	69807481	165	188
8089	15	57718358	57736991	ENSMUSG00000033543	ENSMUSG00000033543	ENSMUSG00000033543	9	69811714	69822011	165	188
8090	15	57691415	57699501	ENSMUSG00000032226	ENSMUSG00000032226	ENSMUSG00000032226	9	69832709	69835863	165	188
8091	15	57215461	57452362	ENSMUSG00000032220	ENSMUSG00000032220	ENSMUSG00000032220	9	70086076	70197962	165	188
8092	15	57184612	57204535	ENSMUSG00000032218	ENSMUSG00000032218	ENSMUSG00000032218	9	70206853	70220718	165	188
8093	15	57067637	57175922	ENSMUSG00000032217	ENSMUSG00000032217	ENSMUSG00000032217	9	70224593	70302889	165	188
8094	15	56958537	57013144	ENSMUSG00000032212	ENSMUSG00000032212	ENSMUSG00000032212	9	70342084	70391395	165	188
8095	15	56850806	56934051	ENSMUSG00000042444	ENSMUSG00000042444	ENSMUSG00000042444	9	70403733	70456322	165	188
8096	15	56675802	56829469	ENSMUSG00000054693	ENSMUSG00000054693	ENSMUSG00000054693	9	70478203	70579439	165	188
8097	15	56511467	56648364	ENSMUSG00000032207	ENSMUSG00000032207	ENSMUSG00000032207	9	70597343	70734025	165	188
8098	15	56217771	56265401	ENSMUSG00000032204	ENSMUSG00000032204	ENSMUSG00000032204	9	70909221	70961849	165	188
8099	15	56032920	56145908	ENSMUSG00000013584	ENSMUSG00000013584	ENSMUSG00000013584	9	71014382	71094795	165	188
8100	15	55671406	55797044	ENSMUSG00000041361	ENSMUSG00000041361	ENSMUSG00000041361	9	71302907	71390920	165	188
8101	15	55455997	55630212	ENSMUSG00000032232	ENSMUSG00000032232	ENSMUSG00000032232	9	71425069	71570162	165	188
8102	15	54998125	55368004	ENSMUSG00000032228	ENSMUSG00000032228	ENSMUSG00000032228	9	71644313	71910179	165	188
8103	15	54709671	54813079	ENSMUSG00000038535	ENSMUSG00000038535	ENSMUSG00000038535	9	72094535	72161068	165	188
8104	15	54508299	54544627	ENSMUSG00000032221	ENSMUSG00000032221	ENSMUSG00000032221	9	72237807	72256960	165	188
8105	15	54444936	54525363	ENSMUSG00000032219	ENSMUSG00000032219	ENSMUSG00000032219	9	72257146	72290519	165	188
8106	15	54166958	54258802	ENSMUSG00000037674	ENSMUSG00000037674	ENSMUSG00000037674	9	72391818	72418188	165	188
8107	15	53906423	53996621	ENSMUSG00000032216	ENSMUSG00000032216	ENSMUSG00000032216	9	72461124	72548414	165	188
8108	15	53699411	53761992	ENSMUSG00000036030	ENSMUSG00000036030	ENSMUSG00000036030	9	72606024	72710423	165	188
8109	15	53625513	53668342	ENSMUSG00000034910	ENSMUSG00000034910	ENSMUSG00000034910	9	72722780	72743147	165	188
8110	15	53435135	53487796	ENSMUSG00000034563	ENSMUSG00000034563	ENSMUSG00000034563	9	72782710	72811068	165	188
8111	15	53398741	53435137	ENSMUSG00000032203	ENSMUSG00000032203	ENSMUSG00000032203	9	72811799	72836783	165	188
8112	15	53283094	53369293	ENSMUSG00000032202	ENSMUSG00000032202	ENSMUSG00000032202	9	72841997	72894724	165	188
8113	15	52314521	52708094	ENSMUSG00000070294	ENSMUSG00000070294	ENSMUSG00000070294	9	73278008	73375628	165	188
8114	15	51594673	51842367	ENSMUSG00000044976	ENSMUSG00000044976	ENSMUSG00000044976	9	73931560	74078852	165	188
8115	15	50836645	50869501	ENSMUSG00000043013	ENSMUSG00000043013	ENSMUSG00000043013	9	74648524	74676264	165	188
8116	15	50392602	50608539	ENSMUSG00000034593	ENSMUSG00000034593	ENSMUSG00000034593	9	74902095	75003902	165	188
8117	15	50271814	50375144	ENSMUSG00000033590	ENSMUSG00000033590	ENSMUSG00000033590	9	75018058	75089988	165	188
8118	15	50189114	50192264	ENSMUSG00000032191	ENSMUSG00000032191	ENSMUSG00000032191	9	75133676	75137549	165	188
8119	15	50017516	50051271	ENSMUSG00000042487	ENSMUSG00000042487	ENSMUSG00000042487	9	75227433	75252341	165	188
8120	15	49909155	49989134	ENSMUSG00000058587	ENSMUSG00000058587	ENSMUSG00000058587	9	75283693	75345566	165	188
8121	15	49831102	49889634	ENSMUSG00000032186	ENSMUSG00000032186	ENSMUSG00000032186	9	75351530	75397234	165	188
8122	15	49802554	49817608	ENSMUSG00000032184	ENSMUSG00000032184	ENSMUSG00000032184	9	75411650	75423680	165	188
8123	15	49760842	49800514	ENSMUSG00000032181	ENSMUSG00000032181	ENSMUSG00000032181	9	75429283	75469926	165	188
8124	6	55726402	55848321	ENSMUSG00000032179	ENSMUSG00000032179	ENSMUSG00000032179	9	75561274	75684926	166	189
8125	6	55407132	55551944	ENSMUSG00000007908	ENSMUSG00000007908	ENSMUSG00000007908	9	75800886	75922259	166	189
8126	6	55300226	55375250	ENSMUSG00000059383	ENSMUSG00000059383	ENSMUSG00000059383	9	75950011	75994685	166	189
8127	6	55147009	55255377	ENSMUSG00000032360	ENSMUSG00000032360	ENSMUSG00000032360	9	76011789	76109487	166	189
8128	6	54819528	54914779	ENSMUSG00000032358	ENSMUSG00000032358	ENSMUSG00000032358	9	76277167	76331692	166	189
8129	6	54281162	54362870	ENSMUSG00000032357	ENSMUSG00000032357	ENSMUSG00000032357	9	76737608	76831658	166	189
8130	6	53991748	54239035	ENSMUSG00000032355	ENSMUSG00000032355	ENSMUSG00000032355	9	76950674	77133652	166	189
8131	6	53767737	53896878	ENSMUSG00000032352	ENSMUSG00000032352	ENSMUSG00000032352	9	77216518	77330563	166	189
8132	6	53624355	53628029	ENSMUSG00000048853	ENSMUSG00000048853	ENSMUSG00000048853	9	77422425	77442040	166	189
8133	6	53470098	53517790	ENSMUSG00000032350	ENSMUSG00000032350	ENSMUSG00000032350	9	77540462	77579335	166	189
8134	6	53240155	53321906	ENSMUSG00000032349	ENSMUSG00000032349	ENSMUSG00000032349	9	77703052	77770197	166	189
8135	6	53099721	53121586	ENSMUSG00000023333	ENSMUSG00000023333	ENSMUSG00000023333	9	77837642	77851322	166	189
8136	6	53043781	53072335	ENSMUSG00000001366	ENSMUSG00000001366	ENSMUSG00000001366	9	77867190	77890041	166	189
8137	6	52974068	53034549	ENSMUSG00000009828	ENSMUSG00000009828	ENSMUSG00000009828	9	77898999	77953622	166	189
8138	6	74134999	74161537	ENSMUSG00000032346	ENSMUSG00000032346	ENSMUSG00000032346	9	78163089	78164272	167	190
8139	6	74161192	74184013	ENSMUSG00000070291	ENSMUSG00000070291	ENSMUSG00000070291	9	78186584	78207495	167	190
8140	6	74191314	74218720	ENSMUSG00000032344	ENSMUSG00000032344	ENSMUSG00000032344	9	78217040	78228854	167	190
8141	6	74228209	74267896	ENSMUSG00000032342	ENSMUSG00000032342	ENSMUSG00000032342	9	78233895	78259836	167	190
8142	6	74359823	74420599	ENSMUSG00000049624	ENSMUSG00000049624	ENSMUSG00000049624	9	78322193	78373711	167	190
8143	6	74462548	74591509	ENSMUSG00000046186	ENSMUSG00000046186	ENSMUSG00000046186	9	78401461	78501940	167	190
8144	6	75850762	75972290	ENSMUSG00000032332	ENSMUSG00000032332	ENSMUSG00000032332	9	79385516	79504149	167	190
8145	6	76019360	76051221	ENSMUSG00000032328	ENSMUSG00000032328	ENSMUSG00000032328	9	79554633	79579114	167	190
8146	6	76074522	76260216	ENSMUSG00000034898	ENSMUSG00000034898	ENSMUSG00000034898	9	79601378	79684154	167	190
8147	6	76367945	76482315	ENSMUSG00000034252	ENSMUSG00000034252	ENSMUSG00000034252	9	79852657	79929852	167	190
8148	6	76687782	76839055	ENSMUSG00000032343	ENSMUSG00000032343	ENSMUSG00000032343	9	80100055	80190491	167	190
8149	6	78228641	78229900	ENSMUSG00000049511	ENSMUSG00000049511	ENSMUSG00000049511	9	81428110	81429270	167	190
8150	6	79633908	79665039	ENSMUSG00000032251	ENSMUSG00000032251	ENSMUSG00000032251	9	82626717	82644404	167	190
8151	6	79707008	79844708	ENSMUSG00000032253	ENSMUSG00000032253	ENSMUSG00000032253	9	82668070	82772104	167	190
8152	6	79967685	80001174	ENSMUSG00000066456	ENSMUSG00000066456	ENSMUSG00000066456	9	82907109	82917141	167	190
8153	6	80251427	80303833	ENSMUSG00000032258	ENSMUSG00000032258	ENSMUSG00000032258	9	83190137	83237745	167	190
8154	6	80397719	80470091	ENSMUSG00000032261	ENSMUSG00000032261	ENSMUSG00000032261	9	83362706	83391456	167	190
8155	6	80681248	80714016	ENSMUSG00000032262	ENSMUSG00000032262	ENSMUSG00000032262	9	83575418	83602883	167	190
8156	6	80771078	80808956	ENSMUSG00000038379	ENSMUSG00000038379	ENSMUSG00000038379	9	83631436	83669100	167	190
8157	6	80873083	81112706	ENSMUSG00000032263	ENSMUSG00000032263	ENSMUSG00000032263	9	83721868	83920957	167	190
8158	6	82512166	82519210	ENSMUSG00000032265	ENSMUSG00000032265	ENSMUSG00000032265	9	85121175	85123486	167	190
8159	6	82936675	83014167	ENSMUSG00000035941	ENSMUSG00000035941	ENSMUSG00000035941	9	85484079	85540162	167	190
8160	6	83130067	83137264	ENSMUSG00000035274	ENSMUSG00000035274	ENSMUSG00000035274	9	85639128	85642507	167	190
8161	6	83658908	83832269	ENSMUSG00000032415	ENSMUSG00000032415	ENSMUSG00000032415	9	86169056	86261535	167	190
8162	6	83834104	83934846	ENSMUSG00000034973	ENSMUSG00000034973	ENSMUSG00000034973	9	86282184	86351036	167	190
8163	6	83935349	83959690	ENSMUSG00000056131	ENSMUSG00000056131	ENSMUSG00000056131	9	86349194	86368545	167	190
8164	6	83959817	83962975	ENSMUSG00000032417	ENSMUSG00000032417	ENSMUSG00000032417	9	86368771	86371614	167	190
8165	6	84278976	84292142	ENSMUSG00000033491	ENSMUSG00000033491	ENSMUSG00000033491	9	86551462	86552691	167	190
8166	6	84319326	84475738	ENSMUSG00000033419	ENSMUSG00000033419	ENSMUSG00000033419	9	86571458	86677497	167	190
8167	6	84408769	84623833	ENSMUSG00000047897	ENSMUSG00000047897	ENSMUSG00000047897	9	86813579	86817862	167	190
8168	6	84626125	84726862	ENSMUSG00000032872	ENSMUSG00000032872	ENSMUSG00000032872	9	86819975	86875393	167	190
8169	6	84800139	84857318	ENSMUSG00000042761	ENSMUSG00000042761	ENSMUSG00000042761	9	86967478	86973840	167	190
8170	6	84890691	84994033	ENSMUSG00000056919	ENSMUSG00000056919	ENSMUSG00000056919	9	87016450	87044652	167	190
8171	6	85500876	85530956	ENSMUSG00000032419	ENSMUSG00000032419	ENSMUSG00000032419	9	87502145	87528790	167	190
8172	6	86216528	86262215	ENSMUSG00000032420	ENSMUSG00000032420	ENSMUSG00000032420	9	88125533	88169982	167	190
8173	6	86271942	86360348	ENSMUSG00000032422	ENSMUSG00000032422	ENSMUSG00000032422	9	88174646	88236829	167	190
8174	6	86380543	86409722	ENSMUSG00000032423	ENSMUSG00000032423	ENSMUSG00000032423	9	88250166	88280101	167	190
8175	15	77924375	77976398	ENSMUSG00000066442	ENSMUSG00000066442	ENSMUSG00000066442	9	89009083	89038118	168	191
8176	15	77511913	77551687	ENSMUSG00000039313	ENSMUSG00000039313	ENSMUSG00000039313	9	89387922	89420879	168	191
8177	15	77390504	77402234	ENSMUSG00000032353	ENSMUSG00000032353	ENSMUSG00000032353	9	89497098	89502884	168	191
8178	15	77372682	77374289	ENSMUSG00000047606	ENSMUSG00000047606	ENSMUSG00000047606	9	89526142	89536368	168	191
8179	15	77041541	77170161	ENSMUSG00000032356	ENSMUSG00000032356	ENSMUSG00000032356	9	89707668	89824819	168	191
8180	15	77001163	77024473	ENSMUSG00000032359	ENSMUSG00000032359	ENSMUSG00000032359	9	89852402	89873982	168	191
8181	15	76838606	76890830	ENSMUSG00000032363	ENSMUSG00000032363	ENSMUSG00000032363	9	89961138	89997904	168	191
8182	15	76074383	76156912	ENSMUSG00000037410	ENSMUSG00000037410	ENSMUSG00000037410	9	89999940	90068662	168	
8183	3	148609871	148617196	ENSMUSG00000032368	ENSMUSG00000032368	ENSMUSG00000032368	9	91158250	91163704	169	192
8184	3	148588023	148603274	ENSMUSG00000036972	ENSMUSG00000036972	ENSMUSG00000036972	9	91166877	91187253	169	192
8185	3	147392882	147451656	ENSMUSG00000032377	ENSMUSG00000032377	ENSMUSG00000032377	9	92261149	92296287	169	192
8186	3	147269922	147361972	ENSMUSG00000032374	ENSMUSG00000032374	ENSMUSG00000032374	9	92346223	92412201	169	192
8187	3	145173603	145193893	ENSMUSG00000045414	ENSMUSG00000045414	ENSMUSG00000045414	9	94329590	94347194	169	192

8188	3	144468234	145049854	ENSMUSG00000031129	ENSMUSG00000031129	ENSMUSG00000031129	9	94479270	95039797	169	192		
8189	3	144320863	144324500	ENSMUSG00000033350	ENSMUSG00000033350	ENSMUSG00000033350	9	95213460	95216009	169	192		
8190	3	144203154	144259182	ENSMUSG00000032407	ENSMUSG00000032407	ENSMUSG00000032407	9	95269754	95321246	169	192		
8191	3	144163735	144164868	ENSMUSG00000064225	ENSMUSG00000064225	ENSMUSG00000064225	9	95369169	95371473	169	192		
8192	3	144019407	144090731	ENSMUSG00000015354	ENSMUSG00000015354	ENSMUSG00000015354	9	95446980	95504903	169	192		
8193	3	143925603	144007960	ENSMUSG00000032839	ENSMUSG00000032839	ENSMUSG00000032839	9	95515979	95559710	169	192		
8194	3	143650773	143780341	ENSMUSG00000032409	ENSMUSG00000032409	ENSMUSG00000032409	9	95667077	95761000	169	192		
8195	3	143508143	143649543	ENSMUSG00000032410	ENSMUSG00000032410	ENSMUSG00000032410	9	95764211	95862379	169	192		
8196	3	143365445	143427080	ENSMUSG00000041440	ENSMUSG00000041440	ENSMUSG00000041440	9	95928754	95989550	169	192		
8197	3	143154035	143351008	ENSMUSG00000032411	ENSMUSG00000032411	ENSMUSG00000032411	9	96005656	96128193	169	192		
8198	3	142688616	142813887	ENSMUSG00000032413	ENSMUSG00000032413	ENSMUSG00000032413	9	96350434	96440855	169	192		
8199	3	142643921	142647508	ENSMUSG00000040433	ENSMUSG00000040433	ENSMUSG00000040433	9	96495619	96541027	169	192		
8200	3	142433389	142496435	ENSMUSG00000043587	ENSMUSG00000043587	ENSMUSG00000043587	9	96632696	96698781	169	192		
8201	3	142253433	142350142	ENSMUSG00000046997	ENSMUSG00000046997	ENSMUSG00000046997	9	96752835	96828170	169	192		
8202	3	141879571	141902681	ENSMUSG00000032451	ENSMUSG00000032451	ENSMUSG00000032451	9	97158918	97179310	169	192		
8203	3	141136907	141767785	ENSMUSG00000032452	ENSMUSG00000032452	ENSMUSG00000032452	9	97255005	97842327	169	192		
8204	3	140761717	140879549	ENSMUSG00000032456	ENSMUSG00000032456	ENSMUSG00000032456	9	98105935	98220786	169	192		
8205	3	140718970	140741180	ENSMUSG00000046402	ENSMUSG00000046402	ENSMUSG00000046402	9	98232313	98255907	169	192		
8206	3	140654417	140678042	ENSMUSG00000032454	ENSMUSG00000032454	ENSMUSG00000032454	9	98299905	98319089	169	192		
8207	3	140559125	140591158	ENSMUSG00000032458	ENSMUSG00000032458	ENSMUSG00000032458	9	98377345	98397492	169	192		
8208	3	140545559	140558583	ENSMUSG00000032459	ENSMUSG00000032459	ENSMUSG00000032459	9	98398089	98411011	169	192		
8209	3	140147124	140148254	ENSMUSG00000050397	ENSMUSG00000050397	ENSMUSG00000050397	9	98765013	98766140	169	192		
8210	3	139810232	139834901	ENSMUSG00000032463	ENSMUSG00000032463	ENSMUSG00000032463	9	98795725	98811370	169	192		
8211	3	139856921	139960875	ENSMUSG00000032462	ENSMUSG00000032462	ENSMUSG00000032462	9	98847754	98949439	169	192		
8212	3	139695879	139795819	ENSMUSG00000056267	ENSMUSG00000056267	ENSMUSG00000056267	9	99052794	99109756	169	192		
8213	3	139636331	139675873	ENSMUSG00000037681	ENSMUSG00000037681	ENSMUSG00000037681	9	99119322	99167870	169	192		
8214	3	139549229	139604299	ENSMUSG00000032470	ENSMUSG00000032470	ENSMUSG00000032470	9	99197332	99235248	169	192		
8215	3	139411348	139498909	ENSMUSG00000032468	ENSMUSG00000032468	ENSMUSG00000032468	9	99289447	99378251	169	192		
8216	3	139362547	139376463	ENSMUSG00000032469	ENSMUSG00000032469	ENSMUSG00000032469	9	99385158	99393806	169	192		
8217	3	139325757	139333897	ENSMUSG00000037953	ENSMUSG00000037953	ENSMUSG00000037953	9	99423018	99430124	169	192		
8218	3	139263524	139317141	ENSMUSG00000037784	ENSMUSG00000037784	ENSMUSG00000037784	9	99438947	99478608	169	192		
8219	3	139200348	139235184	ENSMUSG00000032473	ENSMUSG00000032473	ENSMUSG00000032473	9	99500151	99519367	169	192		
8220	3	138966269	138967086	ENSMUSG00000053747	ENSMUSG00000053747	ENSMUSG00000053747	9	99683577	99685009	169	192		
8221	3	138159397	138212610	ENSMUSG00000044244	ENSMUSG00000044244	ENSMUSG00000044244	9	100275060	100295737	169	192		
8222	3	138063763	138150657	ENSMUSG00000032475	ENSMUSG00000032475	ENSMUSG00000032475	9	100304355	100355405	169	192		
8223	3	138020551	138057414	ENSMUSG00000070287	ENSMUSG00000070287	ENSMUSG00000070287	9	100361730	100362968	169	192		
8224	3	137538689	137953935	ENSMUSG00000037286	ENSMUSG00000037286	ENSMUSG00000037286	9	100452975	100767896	169	192		
8225	3	137451838	137531695	ENSMUSG00000032527	ENSMUSG00000032527	ENSMUSG00000032527	9	100791390	100844227	169	192		
8226	3	137350450	137397378	ENSMUSG00000066415	ENSMUSG00000066415	ENSMUSG00000066415	9	100887852	100958425	169	192		
8227	3	137167257	137349423	ENSMUSG00000043154	ENSMUSG00000043154	ENSMUSG00000043154	9	100964109	101069385	169	192		
8228	3	135996950	136461995	ENSMUSG00000032537	ENSMUSG00000032537	ENSMUSG00000032537	9	101779749	102079716	169	192		
8229	3	135805111	135852554	ENSMUSG00000035606	ENSMUSG00000035606	ENSMUSG00000035606	9	102362011	102399321	169	192		
8230	3	135687642	135763298	ENSMUSG00000032534	ENSMUSG00000032534	ENSMUSG00000032534	9	102443123	102474823	169	192		
8231	3	135556880	135576096	ENSMUSG00000032531	ENSMUSG00000032531	ENSMUSG00000032531	9	102578293	102591561	169	192		
8232	3	135358668	135452184	ENSMUSG00000032547	ENSMUSG00000032547	ENSMUSG00000032547	9	102693067	102765653	169	192		
8233	3	135134236	135231439	ENSMUSG00000032548	ENSMUSG00000032548	ENSMUSG00000032548	9	102866637	102945999	169	192		
8234	3	135025777	135097129	ENSMUSG00000032549	ENSMUSG00000032549	ENSMUSG00000032549	9	102970101	103043418	169	192		
8235	3	135007329	135022213	ENSMUSG00000032553	ENSMUSG00000032553	ENSMUSG00000032553	9	103046183	103060215	169	192		
8236	3	134947925	134980325	ENSMUSG00000032554	ENSMUSG00000032554	ENSMUSG00000032554	9	103067024	103088436	169	192		
8237	3	134802140	134863380	ENSMUSG00000032555	ENSMUSG00000032555	ENSMUSG00000032555	9	103163365	103208582	169	192		
8238	3	134601480	134676746	ENSMUSG00000032556	ENSMUSG00000032556	ENSMUSG00000032556	9	103284706	103338376	169	192		
8239	3	134239936	134597745	ENSMUSG00000042757	ENSMUSG00000042757	ENSMUSG00000042757	9	103342589	103619967	169	192		
8240	3	133882144	133923966	ENSMUSG00000032558	ENSMUSG00000032558	ENSMUSG00000032558	9	103861126	103902075	169	192		
8241	3	133862072	133878314	ENSMUSG00000032557	ENSMUSG00000032557	ENSMUSG00000032557	9	103905494	103921447	169	192		
8242	3	133798784	133804072	ENSMUSG00000041748	ENSMUSG00000041748	ENSMUSG00000041748	9	103956617	103985278	169	192		
8243	3	133636085	133740563	ENSMUSG00000032560	ENSMUSG00000032560	ENSMUSG00000032560	9	104009945	104046002	169	192		
8244	3	133518941	133569831	ENSMUSG00000032561	ENSMUSG00000032561	ENSMUSG00000032561	9	104146585	104196067	169	192		
8245	3	132736274	133241855	ENSMUSG00000032564	ENSMUSG00000032564	ENSMUSG00000032564	9	104427888	104892644	169	192		
8246	3	132663736	132704519	ENSMUSG00000032563	ENSMUSG00000032563	ENSMUSG00000032563	9	104911368	104935576	169	192		
8247	3	132231147	132551957	ENSMUSG00000035032	ENSMUSG00000035032	ENSMUSG00000035032	9	105020567	105253343	169	192		
8248	3	132215411	132228336	ENSMUSG00000032567	ENSMUSG00000032567	ENSMUSG00000032567	9	105253622	105263561	169	192		
8249	3	132096124	132218243	ENSMUSG00000032570	ENSMUSG00000032570	ENSMUSG00000032570	9	105269458	105379247	169	192		
8250	3	131880468	131948340	ENSMUSG00000032571	ENSMUSG00000032571	ENSMUSG00000032571	9	105501130	105545631	169	192		
8251	3	131761902	131776061	ENSMUSG00000043719	ENSMUSG00000043719	ENSMUSG00000043719	9	105632212	105667748	169	192	here	Sub blocks comes here. So this was made into two blocks by our tool.
8252	3	52296876	52302533	ENSMUSG00000020258	ENSMUSG00000020258	ENSMUSG00000020258	9	106011060	106016200	170	192	sub blocks	2 -> 1
8253	3	52255743	52258867	ENSMUSG00000020253	ENSMUSG00000020253	ENSMUSG00000020253	9	106053050	106057043	170	192		
8254	3	52230273	52238999	ENSMUSG00000045322	ENSMUSG00000045322	ENSMUSG00000045322	9	106081616	106084711	170	192		
8255	3	52207156	52223383	ENSMUSG00000032786	ENSMUSG00000032786	ENSMUSG00000032786	9	106092028	106105957	170	192		
8256	3	52084310	52163460	ENSMUSG00000023345	ENSMUSG00000023345	ENSMUSG00000023345	9	106139502	106207982	170	192		
8257	3	52059871	52065269	ENSMUSG00000053716	ENSMUSG00000053716	ENSMUSG00000053716	9	106226733	106233822	170	192		
8258	3	51992535	51998223	ENSMUSG00000023262	ENSMUSG00000023262	ENSMUSG00000023262	9	106291097	106296337	170	192		
8259	3	51984124	51990250	ENSMUSG00000042210	ENSMUSG00000042210	ENSMUSG00000042210	9	106298160	106305737	170	192		
8260	3	51977567	51992465	ENSMUSG00000042073	ENSMUSG00000042073	ENSMUSG00000042073	9	106306762	106311019	170	192		
8261	3	51966518	51972312	ENSMUSG00000023495	ENSMUSG00000023495	ENSMUSG00000023495	9	106311991	106322110	170	192		
8262	3	51964437	51966549	ENSMUSG00000053970	ENSMUSG00000053970	ENSMUSG00000053970	9	106322785	106323828	170	192		
8263	3	51951401	51957921	ENSMUSG00000023249	ENSMUSG00000023249	ENSMUSG00000023249	9	106328454	106334752	170	192		
8264	3	51942487	51950980	ENSMUSG00000041506	ENSMUSG00000041506	ENSMUSG00000041506	9	106335410	106343518	170	192		
8265	3	51903933	51912391	ENSMUSG00000066383	ENSMUSG00000066383	ENSMUSG00000066383	9	106358599	106360338	170	192		
8266	3	51716118	51727663	ENSMUSG00000023192	ENSMUSG00000023192	ENSMUSG00000023192	9	106503119	106508542	170	192		
8267	3	51680288	51713372	ENSMUSG00000040813	ENSMUSG00000040813	ENSMUSG00000040813	9	106516854	106544016	170	192		
8268	3	51636716	51672650	ENSMUSG00000040661	ENSMUSG00000040661	ENSMUSG00000040661	9	106550342	106647186	170	192		
8269	3	51410313	51505823	ENSMUSG00000040325	ENSMUSG00000040325	ENSMUSG00000040325	9	106680077	106738544	170	192		
8270	3	51403771	51410370	ENSMUSG00000045365	ENSMUSG00000045365	ENSMUSG00000045365	9	106742408	106744597	170	192		
8271	3	51397538	51401815	ENSMUSG00000032575	ENSMUSG00000032575	ENSMUSG00000032575	9	106746862	106750047	170	192		
8272	3	50791106	51394030	ENSMUSG00000039716	ENSMUSG00000039716	ENSMUSG00000039716	9	106753481	107089960	170	192		
8273	3	50624343	50661724	ENSMUSG00000032577	ENSMUSG00000032577	ENSMUSG00000032577	9	107113028	107147978	170	192		
8274	3	50618925	50624196	ENSMUSG00000032578	ENSMUSG00000032578	ENSMUSG00000032578	9	107143623	107160062	170	192		
8275	3	50581913	50597426	ENSMUSG00000032579	ENSMUSG00000032579	ENSMUSG00000032579	9	107185804	107196396	170	192		
8276	3	50570467	50583426	ENSMUSG00000037977	ENSMUSG00000037977	ENSMUSG00000037977	9	107198741	107207784	170	192		
8277	3	50375237	50516032	ENSMUSG00000010066	ENSMUSG00000010066	ENSMUSG00000010066	9	107257834	107387159	170	192		
8278	3	50367186	50371943	ENSMUSG00000010045	ENSMUSG00000010045	ENSMUSG00000010045	9	107392046	107396757	170	192		
8279	3	50363280	50366477	ENSMUSG00000037190	ENSMUSG00000037190	ENSMUSG00000037190	9	107397112	107399966	170	192		
8280	3	50359923	50363490	ENSMUSG00000010057	ENSMUSG00000010057	ENSMUSG00000010057	9	107400327	107403796	170	192		
8281	3	50353550	50359287	ENSMUSG00000010044	ENSMUSG00000010044	ENSMUSG00000010044	9	107405411	107409424	170	192		
8282	3	50342221	50353371	ENSMUSG00000010067	ENSMUSG00000010067	ENSMUSG00000010067	9	107409698	107420361	170	192		
8283	3	50337345	50340534	ENSMUSG00000010054	ENSMUSG00000010054	ENSMUSG00000010054	9	107421356	107424209	170	192		
8284	3	50330225	50335217	ENSMUSG00000010047	ENSMUSG00000010047	ENSMUSG00000010047	9	107427227	107430872	170	192		
8285	3	50312325	50316008	ENSMUSG00000010051	ENSMUSG00000010051	ENSMUSG00000010051	9	107435053	107438248	170	192		
8286	3	50300178	50305353	ENSMUSG00000010048	ENSMUSG00000010048	ENSMUSG00000010048	9	107445819	107451139	170	192		
8287	3	50291522	50300548	ENSMUSG00000070285	ENSMUSG00000070285	ENSMUSG00000070285	9	107451637	107455141	170	192		
8288	3	50280044	50289574	ENSMUSG00000057969	ENSMUSG00000057969	ENSMUSG00000057969	9	107456318	107467330	170	192		
8289	3	50248651	50271775	ENSMUSG00000032562	ENSMUSG00000032562	ENSMUSG00000032562	9	107472231	107493318	170	192		
8290	3	50226637	50233404	ENSMUSG00000010064	ENSMUSG00000010064	ENSMUSG00000010064	9	107509262	107527069	170	192		
8291	3	50204047	50208953	ENSMUSG00000034837	ENSMUSG00000034837	ENSMUSG00000034837	9	107532576	107537716	170	192		
8292	3	50167852	50201509	ENSMUSG00000034684	ENSMUSG00000034684	ENSMUSG00000034684	9	107540519	107563657	170	192		
8293	3	50101372	50131392	ENSMUSG00000032580	ENSMUSG00000032580	ENSMUSG00000032580	9	107598474	107629040	170	192		
8294	3	49952525	50089685	ENSMUSG00000032582	ENSMUSG00000032582	ENSMUSG00000032582	9	107631666	107730808	170	192		
8295	3	49921312	49942261	ENSMUSG00000032583	ENSMUSG00000032583	ENSMUSG00000032583	9	107746283	107761226	170	192		
8296	3	49899444	49916074	ENSMUSG00000032584	ENSMUSG00000032584	ENSMUSG00000032584	9	107764990	107778488	170	192		
8297	3	49871755	49882354	ENSMUSG00000032936	ENSMUSG00000032936	ENSMUSG00000032936	9	107794021	107807792	170	192		
8298	3	49817643	49826395	ENSMUSG00000032596	ENSMUSG00000032596	ENSMUSG00000032596	9	107833662	107842151	170	192		
8299	3	49815691	49817465	ENSMUSG00000042106	ENSMUSG00000042106	ENSMUSG00000042106	9	107842327	107843980	170	192		
8300	3	49736732	49798977	ENSMUSG00000032594	ENSMUSG00000032594	ENSMUSG00000032594	9	107860690	107906881	170	192		
8301	3	49734189	49736388	ENSMUSG00000070284	ENSMUSG00000070284	ENSMUSG00000070284	9	107907410	107909802	170	192		
8302	3	49701974	49733961	ENSMUSG00000041528	ENSMUSG00000041528	ENSMUSG00000041528	9	107909778	107937402	170	192		
8303	3	49730388	49731902	ENSMUSG00000032593	ENSMUSG00000032593	ENSMUSG00000032593	9	107911260	107913802	170	192		
8304	3	49696465	49701086	ENSMUSG00000032591	ENSMUSG00000032591	ENSMUSG00000032591	9	107938553	107943101	170	192		
8305	3	49686439	49695935	ENSMUSG00000032590	ENSMUSG00000032590	ENSMUSG00000032590	9	107943516	107952567	170	192		
8306	3	49566926	49683974	ENSMUSG00000032589	ENSMUSG00000032589	ENSMUSG00000032589	9	107954124	108048359	170	192		
8307	3	49482595	49548048	ENSMUSG00000039952	ENSMUSG00000039952	ENSMUSG00000039952	9	108064056	108122019	170	192		
8308	3	49435384	49441725	ENSMUSG00000032606	ENSMUSG00000032606	ENSMUSG00000032606	9	108148541	108154588	170	192		
8309	3	49429216	49435016	ENSMUSG00000032607	ENSMUSG00000032607	ENSMUSG00000032607	9	108155015	108159690	170	192		
8310	3	49424643	49428912	ENSMUSG00000039461	ENSMUSG00000039461	ENSMUSG00000039461	9	108161051	108164044	170	192		
8311	3	49290003	49352519	ENSMUSG00000032612	ENSMUSG00000032612	ENSMUSG00000032612	9	108205963	108250621	170	192		
8312	3	49281223	49289456	ENSMUSG00000032611	ENSMUSG00000032611	ENSMUSG00000032611	9	108250929	108255860	170	192		
8313	3	49084173	49270159	ENSMUSG00000047220	ENSMUSG00000047220	ENSMUSG00000047220	9	108261737	108286587	170	193	here	
8314	3	49184072	49188922	ENSMUSG00000032609	ENSMUSG00000032609	ENSMUSG00000032609	9	108305742	108310480	170	193	Markers moved to different place with in a HSB	1 -> 2
8315	3	49174973	49178760	ENSMUSG00000049305	ENSMUSG00000049305	ENSMUSG00000049305	9	108318630	108324041	170	193		
8316	3	49133663	49145603	ENSMUSG00000052911	ENSMUSG00000052911	ENSMUSG00000052911	9	108338048	108348632	170	193		
8317	3	49121112	49133217	ENSMUSG00000006676	ENSMUSG00000006676	ENSMUSG00000006676	9	108348851	108359802	170	193		
8318	3	49108377	49117194	ENSMUSG00000032604	ENSMUSG00000032604	ENSMUSG00000032604	9	108366162	108374040	170	193		
8319	3	49042148	49106508	ENSMUSG00000006673	ENSMUSG00000006673	ENSMUSG00000006673	9	108375213	108418094	170	193		
8320	3	49034582	49035926	ENSMUSG00000070283	ENSMUSG00000070283	ENSMUSG00000070283	9	108423969	108425485	170	193		
8321	3	49027928	49031014	ENSMUSG00000019039	ENSMUSG00000019039	ENSMUSG00000019039	9	108427996	108430874	170	193		
8322	3	49019830	49028390	ENSMUSG00000066357	ENSMUSG00000066357	ENSMUSG00000066357	9	108430421	108436938	170	193		
8323	3	49002345	49019583	ENSMUSG00000006675	ENSMUSG00000006675	ENSMUSG00000006675	9	108437025	108454937	170	193		
8324	3	48931297	48996041	ENSMUSG00000064145	ENSMUSG00000064145	ENSMUSG00000064145	9	108460279	108506785	170	193		
8325	3	48869370	48911333	ENSMUSG00000032602	ENSMUSG00000032602	ENSMUSG00000032602	9	108519502	108542045	170	193		
8326	3	48763097	48860274	ENSMUSG00000032601	ENSMUSG00000032601	ENSMUSG00000032601	9	108549547	108606747	170	193		
8327	3	48700441	48729710	ENSMUSG00000032599	ENSMUSG00000032599	ENSMUSG00000032599	9	108653217	108663560	170	193		
8328	3	48637835	48684985	ENSMUSG00000023473	ENSMUSG00000023473	ENSMUSG00000023473	9	108683543	108710186	170	193		
8329	3	48633279	48634193	ENSMUSG00000025652	ENSMUSG00000025652	ENSMUSG00000025652	9	108771842	108772786	170	193		
8330	3	48611440	48622474	ENSMUSG00000025651	ENSMUSG00000025651	ENSMUSG00000025651	9	108793553	108806581	170	193		
8331	3	48576510	48607704	ENSMUSG00000025650	ENSMUSG00000025650	ENSMUSG00000025650	9	108810682	108841157	170	193		
8332	3	48574157	48576205	ENSMUSG00000049699	ENSMUSG00000049699	ENSMUSG00000049699	9	108843111	108843962	170	193		
8333	3	48530130	48569231	ENSMUSG00000025648	ENSMUSG00000025648	ENSMUSG00000025648	9	108848901	108889603	170	193		
8334	3	48484201	48516665	ENSMUSG00000025647	ENSMUSG00000025647	ENSMUSG00000025647	9	108895991	108915130	170	193		
8335	3	48463141	48482026	ENSMUSG00000025646	ENSMUSG00000025646	ENSMUSG00000025646	9	108917155	108931517	170	193		
8336	3	48448591	48456523	ENSMUSG00000025645	ENSMUSG00000025645	ENSMUSG00000025645	9	108939906	108949896	170	193		
8337	3	48420897	48441024	ENSMUSG00000053646	ENSMUSG00000053646	ENSMUSG00000053646	9	108952998	108977323	170	193		
8338	3	48239866	48241979	ENSMUSG00000038357	ENSMUSG00000038357	ENSMUSG00000038357	9	109704793	109706855	170	193		
8339	3	48173674	48204805	ENSMUSG00000032477	ENSMUSG00000032477	ENSMUSG00000032477	9	109733366	109751296	170	193		
8340	3	47868054	48105318	ENSMUSG00000032479	ENSMUSG00000032479	ENSMUSG00000032479	9	109824045	109926791	170	193		
8341	3	47819625	47866686	ENSMUSG00000032480	ENSMUSG00000032480	ENSMUSG00000032480	9	109929497	109946209	170	193		
8342	3	47602391	47798410	ENSMUSG00000032481	ENSMUSG00000032481	ENSMUSG00000032481	9	109977231	110084487	170	193		
8343	3	47578741	47597286	ENSMUSG00000032482	ENSMUSG00000032482	ENSMUSG00000032482	9	110088959	110107751	170	193		
8344	3	47512558	47530107	ENSMUSG00000054836	ENSMUSG00000054836	ENSMUSG00000054836	9	110150363	110166492	170	193		
8345	3	47430188	47492449	ENSMUSG00000032485	ENSMUSG00000032485	ENSMUSG00000032485	9	110178494	110230110	170	193		
8346	3	47397528	47429929	ENSMUSG00000036057	ENSMUSG00000036057	ENSMUSG00000036057	9	110230256	110253284	170	193		
8347	3	47299444	47363306	ENSMUSG00000054792	ENSMUSG00000054792	ENSMUSG00000054792	9	110271094	110321510	170	193		
8348	3	47244890	47299092	ENSMUSG00000032489	ENSMUSG00000032489	ENSMUSG00000032489	9	110321913	110369050	170	193		
8349	3	47032926	47140084	ENSMUSG00000044791	ENSMUSG00000044791	ENSMUSG00000044791	9	110418504	110463417	170	193		
8350	3	46996337	47026042	ENSMUSG00000056724	ENSMUSG00000056724	ENSMUSG00000056724	9	110469944	110498781	170	193		
8351	3	46938225	46993242	ENSMUSG00000019659	ENSMUSG00000019659	ENSMUSG00000019659	9	110501319	110556405	170	193		
8352	3	46905279	46926585	ENSMUSG00000032492	ENSMUSG00000032492	ENSMUSG00000032492	9	110566974	110591938	170	193		
8353	3	46874371	46879938	ENSMUSG00000059741	ENSMUSG00000059741	ENSMUSG00000059741	9	110608494	110614613	170	193		
8354	3	46758844	46761249	ENSMUSG00000047257	ENSMUSG00000047257	ENSMUSG00000047257	9	110679404	110686126	170	193		
8355	3	46728610	46734318	ENSMUSG00000048752	ENSMUSG00000048752	ENSMUSG00000048752	9	110702783	110709444	170	193		
8356	3	46717827	46727205	ENSMUSG00000049555	ENSMUSG00000049555	ENSMUSG00000049555	9	110710527	110724889	170	193		
8357	3	46685683	46710195	ENSMUSG00000044037	ENSMUSG00000044037	ENSMUSG00000044037	9	110724990	110745340	170	193		
8358	3	46534874	46596576	ENSMUSG00000032495	ENSMUSG00000032495	ENSMUSG00000032495	9	110796361	110828878	170	193		
8359	3	46514489	46517417	ENSMUSG00000066319	ENSMUSG00000066319	ENSMUSG00000066319	9	110830687	110834427	170	193		
8360	3	46452508	46481657	ENSMUSG00000032496	ENSMUSG00000032496	ENSMUSG00000032496	9	110864108	110887582	170	193		
8361	3	46424119	46426017	ENSMUSG00000043953	ENSMUSG00000043953	ENSMUSG00000043953	9	110899651	110902322	170	193		
8362	3	37070181	37191059	ENSMUSG00000032497	ENSMUSG00000032497	ENSMUSG00000032497	9	110986059	111068873	171	194		
8363	3	37009983	37067341	ENSMUSG00000032498	ENSMUSG00000032498	ENSMUSG00000032498	9	111073071	111116378	171	194		
8364	3	36847363	36875378	ENSMUSG00000062296	ENSMUSG00000062296	ENSMUSG00000062296	9	111219636	111240590	171	194		
8365	3	36728925	36755035	ENSMUSG00000032500	ENSMUSG00000032500	ENSMUSG00000032500	9	111283929	111333929	171	194		
8366	3	36397101	36564500	ENSMUSG00000032502	ENSMUSG00000032502	ENSMUSG00000032502	9	111406250	111535105	171	194		
8367	3	35658853	35810992	ENSMUSG00000032503	ENSMUSG00000032503	ENSMUSG00000032503	9	111910147	112032615	171	194		
8368	3	33814561	33886198	ENSMUSG00000032504	ENSMUSG00000032504	ENSMUSG00000032504	9	113501953	113556927	171	194		
8369	3	33404835	33456874	ENSMUSG00000009741	ENSMUSG00000009741	ENSMUSG00000009741	9	113780352	113824081	171	194		
8370	3	33293938	33403121	ENSMUSG00000032507	ENSMUSG00000032507	ENSMUSG00000032507	9	113827531	113866748	171	194		
8371	3	33130574	33159660	ENSMUSG00000032431	ENSMUSG00000032431	ENSMUSG00000032431	9	114223836	114239368	171	194		
8372	3	33013114	33113638	ENSMUSG00000045594	ENSMUSG00000045594	ENSMUSG00000045594	9	114249846	114313988	171	194		
8373	3	32968070	32972840	ENSMUSG00000047898	ENSMUSG00000047898	ENSMUSG00000047898	9	114339014	114345242	171	194		
8374	3	32701702	32790358	ENSMUSG00000056167	ENSMUSG00000056167	ENSMUSG00000056167	9	114440236	114481473	171	194		
8375	3	32542473	32587354	ENSMUSG00000032435	ENSMUSG00000032435	ENSMUSG00000032435	9	114537529	114572486	171	194		
8376	3	32497808	32519869	ENSMUSG00000032434	ENSMUSG00000032434	ENSMUSG00000032434	9	114579900	114598041	171	194		
8377	3	32255175	32386815	ENSMUSG00000041012	ENSMUSG00000041012	ENSMUSG00000041012	9	114638043	114692850	171	194		
8378	3	31677324	31998242	ENSMUSG00000040875	ENSMUSG00000040875	ENSMUSG00000040875	9	114958098	115080854	171	194		
8379	3	31549495	31652560	ENSMUSG00000032437	ENSMUSG00000032437	ENSMUSG00000032437	9	115091279	115159119	171	194		
8380	3	30742696	30867341	ENSMUSG00000056880	ENSMUSG00000056880	ENSMUSG00000056880	9	115758153	115889913	171	194		
8381	3	30622998	30710635	ENSMUSG00000032440	ENSMUSG00000032440	ENSMUSG00000032440	9	115932995	116023963	171	194		
8382	3	29297947	30021623	ENSMUSG00000039607	ENSMUSG00000039607	ENSMUSG00000039607	9	116431432	117201263	171	194		

8383	3	28365664	28541634	ENSMUSG00000032443	ENSMUSG00000032443	ENSMUSG00000032443	9	117796498	117862696	171	194
8384	3	28340071	28365538	ENSMUSG00000039285	ENSMUSG00000039285	ENSMUSG00000039285	9	117889390	117912535	171	194
8385	3	28258101	28341620	ENSMUSG00000039163	ENSMUSG00000039163	ENSMUSG00000039163	9	117913926	117964140	171	194
8386	3	27733565	27738807	ENSMUSG00000032446	ENSMUSG00000032446	ENSMUSG00000032446	9	118327042	118334830	171	194
8387	3	37259742	37383240	ENSMUSG00000038708	ENSMUSG00000038708	ENSMUSG00000038708	9	118355016	118437734	172	195
8388	3	37468817	37836285	ENSMUSG00000039115	ENSMUSG00000039115	ENSMUSG00000039115	9	118455407	118747637	172	195
8389	3	37878673	38000964	ENSMUSG00000047409	ENSMUSG00000047409	ENSMUSG00000047409	9	118775509	118892696	172	195
8390	3	38007292	38023677	ENSMUSG00000038775	ENSMUSG00000038775	ENSMUSG00000038775	9	118901476	118920023	172	195
8391	3	38023993	38041071	ENSMUSG00000010660	ENSMUSG00000010660	ENSMUSG00000010660	9	118920228	118942200	172	195
8392	3	38055700	38139233	ENSMUSG00000038060	ENSMUSG00000038060	ENSMUSG00000038060	9	118951176	118973993	172	195
8393	3	38322431	38335069	ENSMUSG00000070280	ENSMUSG00000070280	ENSMUSG00000070280	9	119018341	119029725	172	195
8394	3	38182030	38271983	ENSMUSG00000036737	ENSMUSG00000036737	ENSMUSG00000036737	9	119087139	119171108	172	195
8395	3	38155157	38159514	ENSMUSG00000032508	ENSMUSG00000032508	ENSMUSG00000032508	9	119184642	119188700	172	195
8396	3	38363244	38431471	ENSMUSG00000035769	ENSMUSG00000035769	ENSMUSG00000035769	9	119206079	119242018	172	195
8397	3	38470814	38499868	ENSMUSG00000061393	ENSMUSG00000061393	ENSMUSG00000061393	9	119251215	119282213	172	195
8398	3	38512860	38542438	ENSMUSG00000042787	ENSMUSG00000042787	ENSMUSG00000042787	9	119293658	119312928	172	195
8399	3	38564558	38666167	ENSMUSG00000032511	ENSMUSG00000032511	ENSMUSG00000032511	9	119332109	119427714	172	195
8400	3	38713841	38810505	ENSMUSG00000034533	ENSMUSG00000034533	ENSMUSG00000034533	9	119457165	119543058	172	195
8401	3	38862264	38967056	ENSMUSG00000034115	ENSMUSG00000034115	ENSMUSG00000034115	9	119602457	119672558	172	195
8402	3	39068510	39113163	ENSMUSG00000032512	ENSMUSG00000032512	ENSMUSG00000032512	9	119743584	119775283	172	195
8403	3	39279989	39298190	ENSMUSG00000052336	ENSMUSG00000052336	ENSMUSG00000052336	9	119750314	119916981	172	195
8404	3	39113154	39124858	ENSMUSG00000032513	ENSMUSG00000032513	ENSMUSG00000032513	9	119774371	119786256	172	195
8405	3	39124199	39155394	ENSMUSG00000032514	ENSMUSG00000032514	ENSMUSG00000032514	9	119786304	119816491	172	195
8406	3	39158351	39170105	ENSMUSG00000032515	ENSMUSG00000032515	ENSMUSG00000032515	9	119819861	119833356	172	195
8407	3	39346219	39351077	ENSMUSG00000042262	ENSMUSG00000042262	ENSMUSG00000042262	9	119940831	119943604	172	195
8408	3	39399843	39413722	ENSMUSG00000032519	ENSMUSG00000032519	ENSMUSG00000032519	9	119959082	119973020	172	195
8409	3	39484175	39519375	ENSMUSG00000032517	ENSMUSG00000032517	ENSMUSG00000032517	9	119998440	120017357	172	195
8410	3	39917312	40274661	ENSMUSG00000041794	ENSMUSG00000041794	ENSMUSG00000041794	9	120152771	120323532	172	195
8411	3	40326177	40328919	ENSMUSG00000006941	ENSMUSG00000006941	ENSMUSG00000006941	9	120341339	120344025	172	195
8412	3	40403689	40445113	ENSMUSG00000041608	ENSMUSG00000041608	ENSMUSG00000041608	9	120388541	120417015	172	195
8413	3	41216004	41256938	ENSMUSG00000006932	ENSMUSG00000006932	ENSMUSG00000006932	9	120782173	120809205	172	195
8414	3	41898646	41978465	ENSMUSG00000040936	ENSMUSG00000040936	ENSMUSG00000040936	9	120922553	121125895	172	195
8415	3	42107750	42242264	ENSMUSG00000032536	ENSMUSG00000032536	ENSMUSG00000032536	9	121215714	121323614	172	195
8416	3	42274322	42281399	ENSMUSG00000032532	ENSMUSG00000032532	ENSMUSG00000032532	9	121338872	121343228	172	195
8417	3	42413581	42427069	ENSMUSG00000032530	ENSMUSG00000032530	ENSMUSG00000032530	9	121426548	121490794	172	195
8418	3	42519121	42554064	ENSMUSG00000032528	ENSMUSG00000032528	ENSMUSG00000032528	9	121491414	121519332	172	195
8419	3	42569403	42598432	ENSMUSG00000061536	ENSMUSG00000061536	ENSMUSG00000061536	9	121531768	121553727	172	195
8420	3	42607296	42611490	ENSMUSG00000032526	ENSMUSG00000032526	ENSMUSG00000032526	9	121559142	121562086	172	195
8421	3	42617151	42665236	ENSMUSG00000032525	ENSMUSG00000032525	ENSMUSG00000032525	9	121567874	121605539	172	195
8422	3	42674852	42684076	ENSMUSG00000013419	ENSMUSG00000013419	ENSMUSG00000013419	9	121611365	121616710	172	195
8423	3	42702015	42708942	ENSMUSG00000074001	ENSMUSG00000074001	ENSMUSG00000074001	9	121626393	121632517	172	195
8424	3	42709165	42719130	ENSMUSG00000032523	ENSMUSG00000032523	ENSMUSG00000032523	9	121632720	121641082	172	195
8425	3	42724884	42789748	ENSMUSG00000061181	ENSMUSG00000061181	ENSMUSG00000061181	9	121646781	121679734	172	195
8426	3	42836115	42883778	ENSMUSG00000044534	ENSMUSG00000044534	ENSMUSG00000044534	9	121747162	121759762	172	195
8427	3	42890807	42892312	ENSMUSG00000050445	ENSMUSG00000050445	ENSMUSG00000050445	9	121763460	121764962	172	195
8428	3	42995833	43076707	ENSMUSG00000038233	ENSMUSG00000038233	ENSMUSG00000038233	9	121801432	121827385	172	195
8429	3	43095735	43122569	ENSMUSG00000066235	ENSMUSG00000066235	ENSMUSG00000066235	9	121830304	121844711	172	195
8430	3	43303034	43367631	ENSMUSG00000038145	ENSMUSG00000038145	ENSMUSG00000038145	9	121966037	122018393	172	195
8431	3	43382834	43638564	ENSMUSG00000037949	ENSMUSG00000037949	ENSMUSG00000037949	9	122024577	122143072	172	195
8432	3	43707379	43735295	ENSMUSG00000032540	ENSMUSG00000032540	ENSMUSG00000032540	9	122200428	122230221	172	195
8433	3	44258382	44261091	ENSMUSG00000066234	ENSMUSG00000066234	ENSMUSG00000066234	9	122596041	122598647	172	195
8434	3	44355272	44425941	ENSMUSG00000046603	ENSMUSG00000046603	ENSMUSG00000046603	9	122654296	122685032	172	195
8435	3	44457428	44472196	ENSMUSG00000047036	ENSMUSG00000047036	ENSMUSG00000047036	9	122700612	122710826	172	195
8436	3	44665259	44677279	ENSMUSG00000057895	ENSMUSG00000057895	ENSMUSG00000057895	9	122771776	122779717	172	195
8437	3	44778289	44869745	ENSMUSG00000036768	ENSMUSG00000036768	ENSMUSG00000036768	9	122799762	122867423	172	195
8438	3	44878421	44882162	ENSMUSG00000066233	ENSMUSG00000066233	ENSMUSG00000066233	9	122870024	122872185	172	195
8439	3	44891107	44931090	ENSMUSG00000025787	ENSMUSG00000025787	ENSMUSG00000025787	9	122883439	122916256	172	195
8440	3	44992760	45029129	ENSMUSG00000025785	ENSMUSG00000025785	ENSMUSG00000025785	9	122961943	122984825	172	195
8441	3	45042774	45052566	ENSMUSG00000025784	ENSMUSG00000025784	ENSMUSG00000025784	9	122999547	123006125	172	195
8442	3	45098774	45162918	ENSMUSG00000035498	ENSMUSG00000035498	ENSMUSG00000035498	9	123020971	123064736	172	195
8443	3	45405072	45565332	ENSMUSG00000035202	ENSMUSG00000035202	ENSMUSG00000035202	9	123215638	123311362	172	195
8444	3	45611355	45696569	ENSMUSG00000025239	ENSMUSG00000025239	ENSMUSG00000025239	9	123327404	123370215	172	195
8445	3	45705962	45760165	ENSMUSG00000025240	ENSMUSG00000025240	ENSMUSG00000025240	9	123378515	123441296	172	195
8446	3	45903023	45919671	ENSMUSG00000029530	ENSMUSG00000029530	ENSMUSG00000029530	9	123527137	123630663	172	195
8447	3	45934400	46012304	ENSMUSG00000025241	ENSMUSG00000025241	ENSMUSG00000025241	9	123638208	123700267	172	195
8448	3	45957429	45964833	ENSMUSG00000048521	ENSMUSG00000048521	ENSMUSG00000048521	9	123655197	123660458	172	195
8449	3	46037295	46043983	ENSMUSG00000060509	ENSMUSG00000060509	ENSMUSG00000060509	9	123701016	123710826	172	195
8450	3	46227186	46283103	ENSMUSG00000035448	ENSMUSG00000035448	ENSMUSG00000035448	9	123870898	123879738	172	195
8451	6	154768003	154873445	ENSMUSG00000015202	ENSMUSG00000015202	ENSMUSG00000015202	10	3134304	3227479	173	196
8452	6	154520902	154692907	ENSMUSG00000064065	ENSMUSG00000064065	ENSMUSG00000064065	10	3366830	3456102	173	196
8453	6	154402136	154456348	ENSMUSG00000000766	ENSMUSG00000000766	ENSMUSG00000000766	10	3513554	3557732	173	196
8454	6	153373550	153494077	ENSMUSG00000019775	ENSMUSG00000019775	ENSMUSG00000019775	10	4424141	4514341	173	196
8455	6	153333353	153345867	ENSMUSG00000019773	ENSMUSG00000019773	ENSMUSG00000019773	10	4541152	4547381	173	196
8456	6	153113626	153122593	ENSMUSG00000019772	ENSMUSG00000019772	ENSMUSG00000019772	10	4698927	4707323	173	196
8457	6	153060731	153087546	ENSMUSG00000046916	ENSMUSG00000046916	ENSMUSG00000046916	10	4739637	4752711	173	196
8458	6	152484608	153000227	ENSMUSG00000019769	ENSMUSG00000019769	ENSMUSG00000019769	10	4804527	5324531	173	196
8459	6	152170379	152466099	ENSMUSG00000019768	ENSMUSG00000019768	ENSMUSG00000019768	10	5342780	5734495	173	196
8460	6	151856920	151984020	ENSMUSG00000019767	ENSMUSG00000019767	ENSMUSG00000019767	10	5785430	5833846	173	196
8461	6	151815115	151832929	ENSMUSG00000061759	ENSMUSG00000061759	ENSMUSG00000061759	10	5891406	5914028	173	196
8462	6	151603202	151719601	ENSMUSG00000038587	ENSMUSG00000038587	ENSMUSG00000038587	10	5987073	6080178	173	196
8463	6	151228378	151464716	ENSMUSG00000040675	ENSMUSG00000040675	ENSMUSG00000040675	10	6190430	6373423	173	196
8464	6	150962692	151206492	ENSMUSG00000040624	ENSMUSG00000040624	ENSMUSG00000040624	10	6379246	6473507	173	196
8465	6	150731721	150767457	ENSMUSG00000019762	ENSMUSG00000019762	ENSMUSG00000019762	10	6791664	6806285	173	196
8466	6	150505905	150613186	ENSMUSG00000040653	ENSMUSG00000040653	ENSMUSG00000040653	10	6881570	6980428	173	196
8467	6	150181627	150227173	ENSMUSG00000019796	ENSMUSG00000019796	ENSMUSG00000019796	10	7280007	7315665	173	196
8468	6	150112273	150174249	ENSMUSG00000019795	ENSMUSG00000019795	ENSMUSG00000019795	10	7320388	7353463	173	196
8469	6	150087144	150109507	ENSMUSG00000040034	ENSMUSG00000040034	ENSMUSG00000040034	10	7357703	7369067	173	196
8470	6	150023744	150081085	ENSMUSG00000040021	ENSMUSG00000040021	ENSMUSG00000040021	10	7381657	7403525	173	196
8471	6	149957702	150001421	ENSMUSG00000019794	ENSMUSG00000019794	ENSMUSG00000019794	10	7416196	7453339	173	196
8472	6	149929123	149954577	ENSMUSG00000040006	ENSMUSG00000040006	ENSMUSG00000040006	10	7458137	7471107	173	196
8473	6	149867324	149908864	ENSMUSG00000015757	ENSMUSG00000015757	ENSMUSG00000015757	10	7483111	7513019	173	196
8474	6	149813515	149837372	ENSMUSG00000039981	ENSMUSG00000039981	ENSMUSG00000039981	10	7522660	7560586	173	196
8475	6	149680756	149774440	ENSMUSG00000015755	ENSMUSG00000015755	ENSMUSG00000015755	10	7596309	7615052	173	196
8476	6	149110157	149439819	ENSMUSG00000047712	ENSMUSG00000047712	ENSMUSG00000047712	10	7897457	8208962	173	196
8477	6	148705422	148914879	ENSMUSG00000015305	ENSMUSG00000015305	ENSMUSG00000015305	10	8412409	8576075	173	196
8478	6	147871756	147932850	ENSMUSG00000060487	ENSMUSG00000060487	ENSMUSG00000060487	10	9317452	9365398	173	196
8479	6	147567201	147748559	ENSMUSG00000019790	ENSMUSG00000019790	ENSMUSG00000019790	10	9451103	9590873	173	196
8480	6	146906522	146917794	ENSMUSG00000019832	ENSMUSG00000019832	ENSMUSG00000019832	10	10235229	10248397	173	196
8481	6	146392112	146800427	ENSMUSG00000019828	ENSMUSG00000019828	ENSMUSG00000019828	10	10378793	10772495	173	196
8482	6	146160969	146177614	ENSMUSG00000047648	ENSMUSG00000047648	ENSMUSG00000047648	10	10971564	10987155	173	196
8483	6	145988134	146098853	ENSMUSG00000055493	ENSMUSG00000055493	ENSMUSG00000055493	10	11033594	11147912	173	196
8484	6	144654658	145215863	ENSMUSG00000019820	ENSMUSG00000019820	ENSMUSG00000019820	10	12075702	12551836	173	196
8485	6	144513356	144551200	ENSMUSG00000039232	ENSMUSG00000039232	ENSMUSG00000039232	10	12630179	12654280	173	196
8486	6	144457711	144458403	ENSMUSG00000061029	ENSMUSG00000061029	ENSMUSG00000061029	10	12695541	12699250	173	196
8487	6	144303130	144331808	ENSMUSG00000019817	ENSMUSG00000019817	ENSMUSG00000019817	10	12780986	12819853	173	196
8488	6	144247136	144261521	ENSMUSG00000019815	ENSMUSG00000019815	ENSMUSG00000019815	10	12840338	12861536	173	196
8489	6	144206174	144226642	ENSMUSG00000019814	ENSMUSG00000019814	ENSMUSG00000019814	10	12869084	12882437	173	196
8490	6	143971093	144188885	ENSMUSG00000062866	ENSMUSG00000062866	ENSMUSG00000062866	10	12914978	13164594	173	196
8491	6	143857642	143874530	ENSMUSG00000019810	ENSMUSG00000019810	ENSMUSG00000019810	10	13191226	13207473	173	196
8492	6	143813810	143852840	ENSMUSG00000019809	ENSMUSG00000019809	ENSMUSG00000019809	10	13214314	13243292	173	196
8493	6	143789819	143813503	ENSMUSG00000019808	ENSMUSG00000019808	ENSMUSG00000019808	10	13243427	13253578	173	196
8494	6	143423731	143698360	ENSMUSG00000019806	ENSMUSG00000019806	ENSMUSG00000019806	10	13342907	13559028	173	196
8495	6	143115159	143308031	ENSMUSG00000015501	ENSMUSG00000015501	ENSMUSG00000015501	10	13656577	13841576	173	196
8496	6	142664759	142809078	ENSMUSG00000039116	ENSMUSG00000039116	ENSMUSG00000039116	10	14092783	14235234	173	196
8497	6	142510060	142583778	ENSMUSG00000019868	ENSMUSG00000019868	ENSMUSG00000019868	10	14345529	14395758	173	196
8498	6	142495989	142497752	ENSMUSG00000019867	ENSMUSG00000019867	ENSMUSG00000019867	10	14405824	14408412	173	196
8499	6	142438438	142451629	ENSMUSG00000019865	ENSMUSG00000019865	ENSMUSG00000019865	10	14450487	14460754	173	196
8500	6	139735089	139737478	ENSMUSG00000039910	ENSMUSG00000039910	ENSMUSG00000039910	10	17412662	17415072	173	196
8501	6	139602891	139654969	ENSMUSG00000039891	ENSMUSG00000039891	ENSMUSG00000039891	10	17488517	17535079	173	196
8502	6	139497942	139543639	ENSMUSG00000039879	ENSMUSG00000039879	ENSMUSG00000039879	10	17591632	17708387	173	196
8503	6	139267691	139350789	ENSMUSG00000019854	ENSMUSG00000019854	ENSMUSG00000019854	10	17778867	17814549	173	196
8504	6	139212198	139231108	ENSMUSG00000071392	ENSMUSG00000071392	ENSMUSG00000071392	10	17849094	17857775	173	196
8505	6	139136339	139156147	ENSMUSG00000059554	ENSMUSG00000059554	ENSMUSG00000059554	10	17906343	17924394	173	196
8506	6	138784873	138934741	ENSMUSG00000039835	ENSMUSG00000039835	ENSMUSG00000039835	10	18097729	18216469	173	196
8507	6	138767029	138776275	ENSMUSG00000019853	ENSMUSG00000019853	ENSMUSG00000019853	10	18229539	18235492	173	196
8508	6	138569924	138701629	ENSMUSG00000019852	ENSMUSG00000019852	ENSMUSG00000019852	10	18277427	18433174	173	196
8509	6	138230151	138246135	ENSMUSG00000019850	ENSMUSG00000019850	ENSMUSG00000019850	10	18690335	18704826	173	196
8510	6	137855032	137857080	ENSMUSG00000045591	ENSMUSG00000045591	ENSMUSG00000045591	10	19045949	19048020	173	196
8511	6	137560314	137582279	ENSMUSG00000020009	ENSMUSG00000020009	ENSMUSG00000020009	10	19281386	19299641	173	196
8512	6	137506651	137536478	ENSMUSG00000039760	ENSMUSG00000039760	ENSMUSG00000039760	10	19311456	19322568	173	196
8513	6	137362801	137407991	ENSMUSG00000020007	ENSMUSG00000020007	ENSMUSG00000020007	10	19402003	19449469	173	196
8514	6	137285095	137288467	ENSMUSG00000050473	ENSMUSG00000050473	ENSMUSG00000050473	10	19537333	19540875	173	196
8515	6	137185416	137276752	ENSMUSG00000020003	ENSMUSG00000020003	ENSMUSG00000020003	10	19549944	19597078	173	196
8516	6	136919878	137155349	ENSMUSG00000071369	ENSMUSG00000071369	ENSMUSG00000071369	10	19623888	19831139	173	196
8517	6	136705566	136913485	ENSMUSG00000019996	ENSMUSG00000019996	ENSMUSG00000019996	10	19838430	19967769	173	196
8518	6	136621419	136652682	ENSMUSG00000037608	ENSMUSG00000037608	ENSMUSG00000037608	10	20011429	20029340	173	196
8519	6	136593861	136613142	ENSMUSG00000019992	ENSMUSG00000019992	ENSMUSG00000019992	10	20037327	20047185	173	196
8520	6	136400869	136558403	ENSMUSG00000019990	ENSMUSG00000019990	ENSMUSG00000019990	10	20089834	20308624	173	196
8521	6	135653254	135860571	ENSMUSG00000019986	ENSMUSG00000019986	ENSMUSG00000019986	10	20649186	20763893	173	196
8522	6	135544146	135581998	ENSMUSG00000019982	ENSMUSG00000019982	ENSMUSG00000019982	10	20815589	20845875	173	196
8523	6	135323209	135417715	ENSMUSG00000019977	ENSMUSG00000019977	ENSMUSG00000019977	10	20985550	21057211	173	196
8524	6	135280222	135312937	ENSMUSG00000037542	ENSMUSG00000037542	ENSMUSG00000037542	10	21066707	21085849	173	196
8525	6	134532083	134680889	ENSMUSG00000019970	ENSMUSG00000019970	ENSMUSG00000019970	10	21617758	21688243	173	196
8526	6	134351528	134415467	ENSMUSG00000037490	ENSMUSG00000037490	ENSMUSG00000037490	10	22334547	22393701	173	196
8527	6	134251969	134258384	ENSMUSG00000045680	ENSMUSG00000045680	ENSMUSG00000045680	10	22506679	22509544	173	196
8528	6	133604206	133894951	ENSMUSG00000010461	ENSMUSG00000010461	ENSMUSG00000010461	10	22795624	22862541	173	196
8529	6	133132200	133161440	ENSMUSG00000037455	ENSMUSG00000037455	ENSMUSG00000037455	10	23486616	23516076	173	196
8530	6	133085621	133097596	ENSMUSG00000020010	ENSMUSG00000020010	ENSMUSG00000020010	10	23540888	23559257	173	196
8531	6	133044422	133076881	ENSMUSG00000037440	ENSMUSG00000037440	ENSMUSG00000037440	10	23584273	23594756	173	196
8532	6	133007816	133008835	ENSMUSG00000056379	ENSMUSG00000056379	ENSMUSG00000056379	10	23609822	23610820	173	196
8533	6	132979982	132987107	ENSMUSG00000059763	ENSMUSG00000059763	ENSMUSG00000059763	10	23630004	23630999	173	196
8534	6	132951505	132952518	ENSMUSG00000069706	ENSMUSG00000069706	ENSMUSG00000069706	10	23660122	23661135	173	196
8535	6	132933154	132934191	ENSMUSG00000045111	ENSMUSG00000045111	ENSMUSG00000045111	10	23674025	23675062	173	196
8536	6	132901120	132902168	ENSMUSG00000037424	ENSMUSG00000037424	ENSMUSG00000037424	10	23797904	23798950	173	196
8537	6	132820747	132866422	ENSMUSG00000019998	ENSMUSG00000019998	ENSMUSG00000019998	10	23838776	23878375	173	196
8538	6	132658887	132764377	ENSMUSG00000020000	ENSMUSG00000020000	ENSMUSG00000020000	10	23912933	23992199	173	196
8539	6	132311009	132314206	ENSMUSG00000019997	ENSMUSG00000019997	ENSMUSG00000019997	10	24284923	24288096	173	196
8540	6	132170853	132254043	ENSMUSG00000037370	ENSMUSG00000037370	ENSMUSG00000037370	10	24330827	24401518	173	196
8541	6	132000135	132110243	ENSMUSG00000019989	ENSMUSG00000019989	ENSMUSG00000019989	10	24463318	24525509	173	196
8542	6	131950512	131991056	ENSMUSG00000019984	ENSMUSG00000019984	ENSMUSG00000019984	10	24559463	24602876	173	196
8543	6	131936058	131947161	ENSMUSG00000019987	ENSMUSG00000019987	ENSMUSG00000019987	10	24604637	24616856	173	196
8544	6	131508154	131646368	ENSMUSG00000039166	ENSMUSG00000039166	ENSMUSG00000039166	10	24859538	24988582	173	196
8545	6	131202180	131333172	ENSMUSG00000019978	ENSMUSG00000019978	ENSMUSG00000019978	10	25131003	25202496	173	196
8546	6	130728572	130805901	ENSMUSG00000049420	ENSMUSG00000049420	ENSMUSG00000049420	10	25680602	25768468	173	196
8547	6	130507156	130585792	ENSMUSG00000051354	ENSMUSG00000051354	ENSMUSG00000051354	10	25919172	25941341	173	196
8548	6	130381427	130504275	ENSMUSG00000039089	ENSMUSG00000039089	ENSMUSG00000039089	10	25963887	26065347	173	196
8549	6	129939934	130073063	ENSMUSG00000039031	ENSMUSG00000039031	ENSMUSG00000039031	10	26461928	26608068	173	196
8550	6	129246035	129879401	ENSMUSG00000019899	ENSMUSG00000019899	ENSMUSG00000019899	10	26670818	27306267	173	196
8551	6	128332679	128883451	ENSMUSG00000019889	ENSMUSG00000019889	ENSMUSG00000019889	10	27764273	28285271	173	196
8552	6	128065879	128281435	ENSMUSG00000049109	ENSMUSG00000049109	ENSMUSG00000049109	10	28357811	28572165	173	196
8553	6	127940012	127954655	ENSMUSG00000015519	ENSMUSG00000015519	ENSMUSG00000015519	10	28661782	28675724	173	196
8554	6	127810381	127881771	ENSMUSG00000038916	ENSMUSG00000038916	ENSMUSG00000038916	10	28836075	28886859	173	196
8555	6	127651550	127705245	ENSMUSG00000019883	ENSMUSG00000019883	ENSMUSG00000019883	10	29003022	29036079	173	196
8556	6	127629448	127651396	ENSMUSG00000038876	ENSMUSG00000038876	ENSMUSG00000038876	10	29036225	29037301	173	196
8557	6	127481876	127560603	ENSMUSG00000019880	ENSMUSG00000019880	ENSMUSG00000019880	10	29141834	29225229	173	196
8558	6	126703013	126711447	ENSMUSG00000075266	ENSMUSG00000075266	ENSMUSG00000075266	10	29886616	29889908	173	196
8559	6	126349304	126402111	ENSMUSG00000019792	ENSMUSG00000019792	ENSMUSG00000019792	10	30223641	30290108	173	196
8560	6	126319564	126343056	ENSMUSG00000019791	ENSMUSG00000019791	ENSMUSG00000019791	10	30297623	30307782	173	196
8561	6	126144000	126293950	ENSMUSG00000039697	ENSMUSG00000039697	ENSMUSG00000039697	10	30335743	30492354	173	196
8562	6	126112419	126124108	ENSMUSG00000019789	ENSMUSG00000019789	ENSMUSG00000019789	10	30521775	30532199	173	196
8563	6	125638195	125664826	ENSMUSG00000000295	ENSMUSG00000000295	ENSMUSG00000000295	10	31002831	31017619	173	196
8564	6	125516578	125626341	ENSMUSG00000000296	ENSMUSG00000000296	ENSMUSG00000000296	10	31022257	31068765	173	196
8565	6	125346213	125446351	ENSMUSG00000063760	ENSMUSG00000063760	ENSMUSG00000063760	10	31192114	31224318	173	196
8566	6	124166985	125188502	ENSMUSG00000069671	ENSMUSG00000069671	ENSMUSG00000069671	10	32019181	32091789	173	196
8567	6	123358813	123426925	ENSMUSG00000019785	ENSMUSG00000019785	ENSMUSG00000019785	10	33201750	33314016	173	196
8568	6	117044343	117060834	ENSMUSG00000039552	ENSMUSG00000039552	ENSMUSG00000039552	10	33594901	33605299	174	197
8569	6	117063474	117096650	ENSMUSG00000039531	ENSMUSG00000039531	ENSMUSG00000039531	10	33608563	33640675	174	197
8570	6	116999360	117023635	ENSMUSG00000019782	ENSMUSG00000019782	ENSMUSG00000019782	10	33685971	33709032	174	197
8571	6	116924339	116973466	ENSMUSG00000071340	ENSMUSG00000071340	ENSMUSG00000071340	10	33727013	33799229	174	197
8572	6	116981606	116986431	ENSMUSG00000039508	ENSMUSG00000039508	ENSMUSG00000039508	10	33728595	33733722	174	197
8573	6	116939502	116952648	ENSMUSG00000049872	ENSMUSG00000049872	ENSMUSG00000049872	10	33778663	33785902	174	197
8574	6	116889249	116891627	ENSMUSG00000046031	ENSMUSG00000046031	ENSMUSG00000046031	10	33815485	33817398	174	197
8575	6	116707976	116866138	ENSMUSG00000039497	ENSMUSG00000039497	ENSMUSG00000039497	10	33840809	33896967	174	197
8576	6	116704434	116707759	ENSMUSG00000047514	ENSMUSG00000047514	ENSMUSG00000047514	10	33971655	33974301	174	197
8577	6	116677817	116682014	ENSMUSG00000039485	ENSMUSG00000039485	ENSMUSG00000039485	10	33986889	33990738	174	197

8578	6	116528692	116673545	ENSMUSG00000039480	ENSMUSG00000039480	ENSMUSG00000039480	10	33993225	34107944	174	197		
8579	6	116546791	116553989	ENSMUSG00000039462	ENSMUSG00000039462	ENSMUSG00000039462	10	34079251	34087720	174	197		
8580	6	116369386	116488614	ENSMUSG00000019779	ENSMUSG00000019779	ENSMUSG00000019779	10	34172815	34299745	174	197		
8581	6	114485071	114490735	ENSMUSG00000044499	ENSMUSG00000044499	ENSMUSG00000044499	10	36196235	36523805	174	197		
8582	6	114368571	114399029	ENSMUSG00000019777	ENSMUSG00000019777	ENSMUSG00000019777	10	36663960	36691304	174	197		
8583	6	114285234	114291341	ENSMUSG00000069662	ENSMUSG00000069662	ENSMUSG00000069662	10	36824237	36828330	174	197		
8584	6	112536654	112682503	ENSMUSG00000019846	ENSMUSG00000019846	ENSMUSG00000019846	10	38654993	38799597	174	197		
8585	6	112515367	112530685	ENSMUSG00000051736	ENSMUSG00000051736	ENSMUSG00000051736	10	38808224	38823311	174	197		
8586	6	112498673	112515409	ENSMUSG00000019845	ENSMUSG00000019845	ENSMUSG00000019845	10	38823475	38839818	174	197		
8587	6	112482155	112498864	ENSMUSG00000062074	ENSMUSG00000062074	ENSMUSG00000062074	10	38840586	38853056	174	197		
8588	6	112089180	112301348	ENSMUSG00000019843	ENSMUSG00000019843	ENSMUSG00000019843	10	39059514	39254797	174	197		
8589	6	111986836	112033755	ENSMUSG00000019842	ENSMUSG00000019842	ENSMUSG00000019842	10	39302350	39344723	174	197		
8590	6	111726927	111911125	ENSMUSG00000019841	ENSMUSG00000019841	ENSMUSG00000019841	10	39421891	39564619	174	197		
8591	6	111515474	111651838	ENSMUSG00000019838	ENSMUSG00000019838	ENSMUSG00000019838	10	39725887	39831670	174	197		
8592	6	111409911	111453996	ENSMUSG00000038510	ENSMUSG00000038510	ENSMUSG00000038510	10	39912662	39936395	174	197		
8593	6	111386456	111395786	ENSMUSG00000019837	ENSMUSG00000019837	ENSMUSG00000019837	10	39938145	39947080	174	197		
8594	6	111037874	111243212	ENSMUSG00000038481	ENSMUSG00000038481	ENSMUSG00000038481	10	40038724	40173234	174	197		
8595	6	110852583	110904537	ENSMUSG00000019834	ENSMUSG00000019834	ENSMUSG00000019834	10	40260060	40293544	174	197		
8596	6	110819667	110843458	ENSMUSG00000063428	ENSMUSG00000063428	ENSMUSG00000063428	10	40319451	40338387	174	197		
8597	6	110672405	110786168	ENSMUSG00000045555	ENSMUSG00000045555	ENSMUSG00000045555	10	40371739	40499498	174	197		
8598	6	110608037	110660113	ENSMUSG00000038446	ENSMUSG00000038446	ENSMUSG00000038446	10	40521594	40571507	174	197		
8599	6	110528382	110607556	ENSMUSG00000019831	ENSMUSG00000019831	ENSMUSG00000019831	10	40571988	40626982	174	197		
8600	6	110406991	110408614	ENSMUSG00000046922	ENSMUSG00000046922	ENSMUSG00000046922	10	40758908	40759999	174	197		
8601	6	110007373	110119113	ENSMUSG00000071331	ENSMUSG00000071331	ENSMUSG00000071331	10	41004662	41047057	174	197		
8602	6	109955525	109978433	ENSMUSG00000038407	ENSMUSG00000038407	ENSMUSG00000038407	10	41077379	41097296	174	197		
8603	6	109921265	109934268	ENSMUSG00000071330	ENSMUSG00000071330	ENSMUSG00000071330	10	41111472	41121703	174	197		
8604	6	109890412	109911133	ENSMUSG00000019826	ENSMUSG00000019826	ENSMUSG00000019826	10	41138811	41153991	174	197		
8605	6	109871958	109883883	ENSMUSG00000019823	ENSMUSG00000019823	ENSMUSG00000019823	10	41164792	41175447	174	197		
8606	6	109868659	109871814	ENSMUSG00000019822	ENSMUSG00000019822	ENSMUSG00000019822	10	41175595	41178767	174	197		
8607	6	109794416	109810340	ENSMUSG00000019818	ENSMUSG00000019818	ENSMUSG00000019818	10	41207915	41219463	174	197		
8608	6	109722331	109743314	ENSMUSG00000019816	ENSMUSG00000019816	ENSMUSG00000019816	10	41268637	41274825	174	197		
8609	6	109664557	109698267	ENSMUSG00000071327	ENSMUSG00000071327	ENSMUSG00000071327	10	41311784	41346211	174	197		
8610	6	109523416	109591059	ENSMUSG00000019813	ENSMUSG00000019813	ENSMUSG00000019813	10	41407256	41498290	174	197		
8611	6	109414340	109521970	ENSMUSG00000038332	ENSMUSG00000038332	ENSMUSG00000038332	10	41498989	41596838	174	197		
8612	6	109276318	109401877	ENSMUSG00000071324	ENSMUSG00000071324	ENSMUSG00000071324	10	41605480	41696124	174	197		
8613	6	108987731	109108661	ENSMUSG00000048756	ENSMUSG00000048756	ENSMUSG00000048756	10	41874201	41965157	174	197		
8614	6	108722791	108950951	ENSMUSG00000038302	ENSMUSG00000038302	ENSMUSG00000038302	10	42001000	42166829	174	197		
8615	6	108639119	108689157	ENSMUSG00000019804	ENSMUSG00000019804	ENSMUSG00000019804	10	42190444	42223782	174	197		
8616	6	108595920	108616699	ENSMUSG00000019803	ENSMUSG00000019803	ENSMUSG00000019803	10	42250385	42272047	174	197		
8617	6	108469306	108502634	ENSMUSG00000038280	ENSMUSG00000038280	ENSMUSG00000038280	10	42367332	42390870	174	197		
8618	6	108295653	108386086	ENSMUSG00000019802	ENSMUSG00000019802	ENSMUSG00000019802	10	42449911	42520925	174	197		
8619	6	108132108	108252214	ENSMUSG00000044770	ENSMUSG00000044770	ENSMUSG00000044770	10	42549132	42649208	174	197		
8620	6	107918261	108088067	ENSMUSG00000038248	ENSMUSG00000038248	ENSMUSG00000038248	10	42709397	42849265	174	197		
8621	6	107580454	107887460	ENSMUSG00000038240	ENSMUSG00000038240	ENSMUSG00000038240	10	42909932	43128372	174	197		
8622	6	107493079	107543166	ENSMUSG00000038214	ENSMUSG00000038214	ENSMUSG00000038214	10	43167555	43203826	174	197		
8623	6	107456110	107479237	ENSMUSG00000019797	ENSMUSG00000019797	ENSMUSG00000019797	10	43213536	43229409	174	197		
8624	6	107184146	107222985	ENSMUSG00000019863	ENSMUSG00000019863	ENSMUSG00000019863	10	43562605	43590151	174	197		
8625	6	107126539	107184055	ENSMUSG00000019864	ENSMUSG00000019864	ENSMUSG00000019864	10	43590222	43636277	174	197		
8626	6	107066423	107125022	ENSMUSG00000019866	ENSMUSG00000019866	ENSMUSG00000019866	10	43640281	43686474	174	197		
8627	6	106739044	106880359	ENSMUSG00000038160	ENSMUSG00000038160	ENSMUSG00000038160	10	43956773	44052706	174	197		
8628	6	106640888	106664507	ENSMUSG00000038151	ENSMUSG00000038151	ENSMUSG00000038151	10	44125592	44147163	174	197		
8629	6	105832133	105957652	ENSMUSG00000019849	ENSMUSG00000019849	ENSMUSG00000019849	10	44755629	44847410	174	197		
8630	6	105712476	105734563	ENSMUSG00000019848	ENSMUSG00000019848	ENSMUSG00000019848	10	45003210	45006395	174	197		
8631	6	105651396	105691742	ENSMUSG00000071317	ENSMUSG00000071317	ENSMUSG00000071317	10	45024187	45058123	174	197		
8632	6	105511616	105637899	ENSMUSG00000063804	ENSMUSG00000063804	ENSMUSG00000063804	10	45069480	45157640	174	197		
8633	6	105282663	105414451	ENSMUSG00000038822	ENSMUSG00000038822	ENSMUSG00000038822	10	45266304	45400685	174	197		
8634	6	101953385	102624651	ENSMUSG00000056073	ENSMUSG00000056073	ENSMUSG00000056073	10	48789583	49471674	174	197		
8635	6	101063333	101435945	ENSMUSG00000038774	ENSMUSG00000038774	ENSMUSG00000038774	10	50293208	50538996	174	197		
8636	6	100939606	101018272	ENSMUSG00000019913	ENSMUSG00000019913	ENSMUSG00000019913	10	50584066	50677567	174	197		
8637	6	117180056	117193579	ENSMUSG00000019909	ENSMUSG00000019909	ENSMUSG00000019909	10	51273835	51278895	175	198		
8638	6	117219941	117256891	ENSMUSG00000019905	ENSMUSG00000019905	ENSMUSG00000019905	10	51303238	51319873	175	198		
8639	6	117305069	117359993	ENSMUSG00000019900	ENSMUSG00000019900	ENSMUSG00000019900	10	51366225	51418218	175	198		
8640	6	117693430	117701418	ENSMUSG00000049641	ENSMUSG00000049641	ENSMUSG00000049641	10	51710946	51716763	175	198		
8641	6	117716156	117853711	ENSMUSG00000019893	ENSMUSG00000019893	ENSMUSG00000019893	10	51734341	51883659	175	198		
8642	6	117910590	117976650	ENSMUSG00000019891	ENSMUSG00000019891	ENSMUSG00000019891	10	51922034	52008690	175	198		
8643	6	117988125	118030374	ENSMUSG00000019861	ENSMUSG00000019861	ENSMUSG00000019861	10	52025492	52070490	175	198		
8644	6	118103358	118138496	ENSMUSG00000023068	ENSMUSG00000023068	ENSMUSG00000023068	10	52105962	52125180	175	198		
8645	6	118335583	118742108	ENSMUSG00000038602	ENSMUSG00000038602	ENSMUSG00000038602	10	52379412	52796787	175	198		
8646	6	118888628	119079481	ENSMUSG00000038594	ENSMUSG00000038594	ENSMUSG00000038594	10	52984631	53037681	175	198		
8647	6	118976154	118988586	ENSMUSG00000038583	ENSMUSG00000038583	ENSMUSG00000038583	10	53026122	53034396	175	198		
8648	6	119254628	119297978	ENSMUSG00000058298	ENSMUSG00000058298	ENSMUSG00000058298	10	53225739	53318854	175	198		
8649	6	119263629	119270478	ENSMUSG00000019857	ENSMUSG00000019857	ENSMUSG00000019857	10	53285376	53297640	175	198		
8650	6	119322693	119441258	ENSMUSG00000019856	ENSMUSG00000019856	ENSMUSG00000019856	10	53322052	53439295	175	198		
8651	6	119540968	119712625	ENSMUSG00000003746	ENSMUSG00000003746	ENSMUSG00000003746	10	53594448	53764211	175	198		
8652	6	121443616	121697325	ENSMUSG00000038122	ENSMUSG00000038122	ENSMUSG00000038122	10	55737278	55917017	175	198		
8653	6	122762390	122795810	ENSMUSG00000019878	ENSMUSG00000019878	ENSMUSG00000019878	10	57174901	57201550	175	198		
8654	6	122806195	122834725	ENSMUSG00000019877	ENSMUSG00000019877	ENSMUSG00000019877	10	57204197	57220919	175	198		
8655	6	123080406	123088125	ENSMUSG00000019876	ENSMUSG00000019876	ENSMUSG00000019876	10	57320496	57429527	175	198		
8656	6	123142603	123145740	ENSMUSG00000019874	ENSMUSG00000019874	ENSMUSG00000019874	10	57473338	57476865	175	198		
8657	6	123152014	123172564	ENSMUSG00000019872	ENSMUSG00000019872	ENSMUSG00000019872	10	57483015	57500236	175	198		
8658	2	108432068	108492286	ENSMUSG00000038039	ENSMUSG00000038039	ENSMUSG00000038039	10	57650883	57700951	176	199		
8659	2	108702369	108767683	ENSMUSG00000003226	ENSMUSG00000003226	ENSMUSG00000003226	10	57842209	57889512	176	199		
8660	2	108769651	108859279	ENSMUSG00000038010	ENSMUSG00000038010	ENSMUSG00000038010	10	57933654	57971599	176	199		
8661	2	108877366	108972260	ENSMUSG00000003227	ENSMUSG00000003227	ENSMUSG00000003227	10	57996146	58071053	176	199		
8662	2	109381463	109432377	ENSMUSG00000037990	ENSMUSG00000037990	ENSMUSG00000037990	10	58209460	58534273	176	199		
8663	2	109657852	109726388	ENSMUSG00000019917	ENSMUSG00000019917	ENSMUSG00000019917	10	58537007	58588175	176	199		
8664	2	109729181	109733852	ENSMUSG00000071286	ENSMUSG00000071286	ENSMUSG00000071286	10	58617564	58618939	176	199		
8665	10	74436981	74526614	ENSMUSG00000019916	ENSMUSG00000019916	ENSMUSG00000019916	10	58718730	58767701	177	200		
8666	10	74364934	74384542	ENSMUSG00000009646	ENSMUSG00000009646	ENSMUSG00000009646	10	58799042	58817347	177	200		
8667	10	74323345	74362797	ENSMUSG00000009654	ENSMUSG00000009654	ENSMUSG00000009654	10	58818317	58837136	177	200		
8668	10	74121895	74317458	ENSMUSG00000009647	ENSMUSG00000009647	ENSMUSG00000009647	10	58843954	59012027	177	200		
8669	10	73797104	74055869	ENSMUSG00000020111	ENSMUSG00000020111	ENSMUSG00000020111	10	59097924	59259487	177	200		
8670	10	73762594	73784875	ENSMUSG00000020109	ENSMUSG00000020109	ENSMUSG00000020109	10	59274948	59293372	177	200		
8671	10	73703683	73705803	ENSMUSG00000020108	ENSMUSG00000020108	ENSMUSG00000020108	10	59345032	59347127	177	200		
8672	10	73645812	73665622	ENSMUSG00000020107	ENSMUSG00000020107	ENSMUSG00000020107	10	59383266	59398469	177	200		
8673	10	73526285	73646052	ENSMUSG00000044475	ENSMUSG00000044475	ENSMUSG00000044475	10	59398196	59495343	177	200		
8674	10	73488799	73518773	ENSMUSG00000058297	ENSMUSG00000058297	ENSMUSG00000058297	10	59501825	59529277	177	200		
8675	10	73394129	73443328	ENSMUSG00000057337	ENSMUSG00000057337	ENSMUSG00000057337	10	59576884	59584064	177	200		
8676	10	73246062	73281034	ENSMUSG00000004207	ENSMUSG00000004207	ENSMUSG00000004207	10	59673071	59697954	177	200		
8677	10	72826700	73245659	ENSMUSG00000012819	ENSMUSG00000012819	ENSMUSG00000012819	10	59698656	60052690	177	200		
8678	10	73177322	73203261	ENSMUSG00000020101	ENSMUSG00000020101	ENSMUSG00000020101	10	59742261	59768041	177	200		
8679	10	72749021	72793148	ENSMUSG00000020100	ENSMUSG00000020100	ENSMUSG00000020100	10	60111193	60146042	177	200		
8680	10	72642333	72732627	ENSMUSG00000020099	ENSMUSG00000020099	ENSMUSG00000020099	10	60157952	60226938	177	200		
8681	10	72313273	72318547	ENSMUSG00000020098	ENSMUSG00000020098	ENSMUSG00000020098	10	60487421	60489262	177	200		
8682	10	72245732	72310936	ENSMUSG00000020097	ENSMUSG00000020097	ENSMUSG00000020097	10	60494001	60518891	177	200		
8683	10	72201001	72215163	ENSMUSG00000020096	ENSMUSG00000020096	ENSMUSG00000020096	10	60567343	60584198	177	200		
8684	10	72102565	72192203	ENSMUSG00000059901	ENSMUSG00000059901	ENSMUSG00000059901	10	60593838	60666615	177	200		
8685	10	72027110	72032521	ENSMUSG00000037202	ENSMUSG00000037202	ENSMUSG00000037202	10	60693191	60699667	177	200		
8686	10	71908570	71998211	ENSMUSG00000020092	ENSMUSG00000020092	ENSMUSG00000020092	10	60715020	60778715	177	200		
8687	10	71862077	71871429	ENSMUSG00000037171	ENSMUSG00000037171	ENSMUSG00000037171	10	60813645	60820694	177	200		
8688	10	71834141	71858380	ENSMUSG00000020091	ENSMUSG00000020091	ENSMUSG00000020091	10	60827852	60848130	177	200		
8689	10	71728735	71812351	ENSMUSG00000037151	ENSMUSG00000037151	ENSMUSG00000037151	10	60871190	60977599	177	200		
8690	10	71632592	71663196	ENSMUSG00000020089	ENSMUSG00000020089	ENSMUSG00000020089	10	61043978	61069522	177	200		
8691	10	71579966	71600285	ENSMUSG00000020088	ENSMUSG00000020088	ENSMUSG00000020088	10	61075678	61088654	177	200		
8692	10	71567743	71576348	ENSMUSG00000020087	ENSMUSG00000020087	ENSMUSG00000020087	10	61090871	61098123	177	200		
8693	10	71527985	71553864	ENSMUSG00000020085	ENSMUSG00000020085	ENSMUSG00000020085	10	61116006	61134012	177	200		
8694	10	71482558	71542021	ENSMUSG00000020086	ENSMUSG00000020086	ENSMUSG00000020086	10	61134000	61179303	177	200		
8695	10	71231694	71388910	ENSMUSG00000058806	ENSMUSG00000058806	ENSMUSG00000058806	10	61235435	61373993	177	200		
8696	10	71060020	71063358	ENSMUSG00000020083	ENSMUSG00000020083	ENSMUSG00000020083	10	61503013	61505380	177	200		
8697	10	71001460	71003000	ENSMUSG00000044312	ENSMUSG00000044312	ENSMUSG00000044312	10	61528447	61530120	177	200		
8698	10	70881232	70937429	ENSMUSG00000037031	ENSMUSG00000037031	ENSMUSG00000037031	10	61582713	61626583	177	200		
8699	10	70834246	70846629	ENSMUSG00000020081	ENSMUSG00000020081	ENSMUSG00000020081	10	61648175	61661347	177	200		
8700	10	70699762	70831641	ENSMUSG00000037012	ENSMUSG00000037012	ENSMUSG00000037012	10	61664465	61757594	177	200		
8701	10	70650065	70697321	ENSMUSG00000020080	ENSMUSG00000020080	ENSMUSG00000020080	10	61778494	61817814	177	200		
8702	10	70609994	70638860	ENSMUSG00000020079	ENSMUSG00000020079	ENSMUSG00000020079	10	61824697	61845077	177	200		
8703	10	70553274	70602623	ENSMUSG00000020078	ENSMUSG00000020078	ENSMUSG00000020078	10	61850592	61881955	177	200		
8704	10	70517834	70534571	ENSMUSG00000020077	ENSMUSG00000020077	ENSMUSG00000020077	10	61889785	61903112	177	200		
8705	10	70418493	70446744	ENSMUSG00000036955	ENSMUSG00000036955	ENSMUSG00000036955	10	61953827	61973814	177	200		
8706	10	70331040	70376609	ENSMUSG00000020076	ENSMUSG00000020076	ENSMUSG00000020076	10	62011380	62046575	177	200		
8707	10	70314699	70322822	ENSMUSG00000036923	ENSMUSG00000036923	ENSMUSG00000036923	10	62054547	62121220	177	200		
8708	10	70150977	70222140	ENSMUSG00000020074	ENSMUSG00000020074	ENSMUSG00000020074	10	62139819	62187613	177	200		
8709	10	69990419	70124245	ENSMUSG00000075011	ENSMUSG00000075011	ENSMUSG00000075011	10	62208966	62228986	177	200		
8710	10	69912104	69957590	ENSMUSG00000071253	ENSMUSG00000071253	ENSMUSG00000071253	10	62315990	62341851	177	200		
8711	10	69843828	69901678	ENSMUSG00000036875	ENSMUSG00000036875	ENSMUSG00000036875	10	62344541	62367350	177	200		
8712	10	69775531	69836819	ENSMUSG00000020070	ENSMUSG00000020070	ENSMUSG00000020070	10	62378027	62407127	177	200		
8713	10	69761838	69772950	ENSMUSG00000020069	ENSMUSG00000020069	ENSMUSG00000020069	10	62411157	62419204	177	200		
8714	10	69660387	69661866	ENSMUSG00000036816	ENSMUSG00000036816	ENSMUSG00000036816	10	62495513	62495962	177	200		
8715	10	69535886	69641464	ENSMUSG00000020067	ENSMUSG00000020067	ENSMUSG00000020067	10	62512217	62588639	177	200		
8716	10	69351671	69505046	ENSMUSG00000020064	ENSMUSG00000020064	ENSMUSG00000020064	10	62641196	62713113	177	200		
8717	10	69314433	69348153	ENSMUSG00000020063	ENSMUSG00000020063	ENSMUSG00000020063	10	62714364	62734366	177	200		
8718	10	69226434	69267943	ENSMUSG00000036764	ENSMUSG00000036764	ENSMUSG00000036764	10	62781928	62804143	177	200		
8719	10	68355800	68529594	ENSMUSG00000042846	ENSMUSG00000042846	ENSMUSG00000042846	10	63325415	63484740	177	200		
8720	10	64951179	65054887	ENSMUSG00000019873	ENSMUSG00000019873	ENSMUSG00000019873	10	66406865	66492302	177	200		
8721	10	64596987	64644799	ENSMUSG00000037876	ENSMUSG00000037876	ENSMUSG00000037876	10	66585093	66650894	177	200		
8722	10	64241763	64246133	ENSMUSG00000037868	ENSMUSG00000037868	ENSMUSG00000037868	10	66933461	66937538	177	200		
8723	10	64234623	64238249	ENSMUSG00000057134	ENSMUSG00000057134	ENSMUSG00000057134	10	66943360	66944130	177	200		
8724	10	63803957	64101777	ENSMUSG00000037855	ENSMUSG00000037855	ENSMUSG00000037855	10	67281460	67308019	177	200		
8725	10	63622959	63698472	ENSMUSG00000037846	ENSMUSG00000037846	ENSMUSG00000037846	10	67375072	67437655	177	200		
8726	10	63331065	63526709	ENSMUSG00000019947	ENSMUSG00000019947	ENSMUSG00000019947	10	67491861	67673547	177	200		
8727	10	63001770	63195774	ENSMUSG00000019945	ENSMUSG00000019945	ENSMUSG00000019945	10	67826310	67937232	177	200		
8728	10	62840092	62882845	ENSMUSG00000060044	ENSMUSG00000060044	ENSMUSG00000060044	10	68119103	68175968	177	200		
8729	10	62299202	62431204	ENSMUSG00000019944	ENSMUSG00000019944	ENSMUSG00000019944	10	68603909	68685257	177	200		
8730	10	62208107	62224616	ENSMUSG00000019942	ENSMUSG00000019942	ENSMUSG00000019942	10	68731992	68748269	177	200		
8731	10	61458165	61819494	ENSMUSG00000069601	ENSMUSG00000069601	ENSMUSG00000069601	10	69102169	69410987	177	200		
8732	10	61218527	61336314	ENSMUSG00000048701	ENSMUSG00000048701	ENSMUSG00000048701	10	69492657	69584635	177	200		
8733	10	61080529	61114093	ENSMUSG00000037762	ENSMUSG00000037762	ENSMUSG00000037762	10	69640633	69681308	177	200		
8734	10	60675902	60792358	ENSMUSG00000043259	ENSMUSG00000043259	ENSMUSG00000043259	10	69836272	69940306	177	200		
8735	10	60606356	60677540	ENSMUSG00000037747	ENSMUSG00000037747	ENSMUSG00000037747	10	69952986	69994577	177	200		
8736	10	59942906	60261201	ENSMUSG00000014329	ENSMUSG00000014329	ENSMUSG00000014329	10	70320402	70554990	177	200		
8737	10	59814788	59828987	ENSMUSG00000003923	ENSMUSG00000003923	ENSMUSG00000003923	10	70620777	70633344	177	200		
8738	10	59764745	59800515	ENSMUSG00000019927	ENSMUSG00000019927	ENSMUSG00000019927	10	70650280	70657583	177	200		
8739	10	59621284	59697700	ENSMUSG00000060733	ENSMUSG00000060733	ENSMUSG00000060733	10	70743093	70781185	177	200		
8740	10	57786995	57791042	ENSMUSG00000019923	ENSMUSG00000019923	ENSMUSG00000019923	10	72050189	72070263	177	200		
8741	10	55250866	56231057	ENSMUSG00000052613	ENSMUSG00000052613	ENSMUSG00000052613	10	73342356	74076383	177	200		
8742	22	21731594	21814241	ENSMUSG00000009070	ENSMUSG00000009070	ENSMUSG00000009070	10	74401193	74476281	178	201	here	Blocks 201, 202 and 203 defined by AG do not have orientation and so were made a single block by ST
8743	22	21742534	21797221	ENSMUSG00000040009	ENSMUSG00000040009	ENSMUSG00000040009	10	74410947	74459593	178	201	Blocks without orientation merged with the neighboring block	1 -> 3
8744	22	21817513	21836531	ENSMUSG00000020175	ENSMUSG00000020175	ENSMUSG00000020175	10	74480805	74497816	178	201		
8745	22	22996866	23143212	ENSMUSG00000033444	ENSMUSG00000033444	ENSMUSG00000033444	10	74674387	74756089	178	202		
8746	22	23153537	23168309	ENSMUSG00000020178	ENSMUSG00000020178	ENSMUSG00000020178	10	74769509	74778497	178	202		
8747	22	23221251	23252552	ENSMUSG00000033427	ENSMUSG00000033427	ENSMUSG00000033427	10	74850749	74883913	178	202		
8748	22	23266410	23281275	ENSMUSG00000033416	ENSMUSG00000033416	ENSMUSG00000033416	10	74949999	74960921	178	202		
8749	22	23281618	23298509	ENSMUSG00000020180	ENSMUSG00000020180	ENSMUSG00000020180	10	74961737	74979181	178	202		
8750	22	23311603	23319040	ENSMUSG00000046807	ENSMUSG00000046807	ENSMUSG00000046807	10	74993845	75003836	178	202		
8751	22	22945628	22971100	ENSMUSG00000006344	ENSMUSG00000006344	ENSMUSG00000006344	10	75033097	75060916	178	203		
8752	22	22907425	22915069	ENSMUSG00000006342	ENSMUSG00000006342	ENSMUSG00000006342	10	75080339	75087705	178	203		
8753	22	22737765	22904596	ENSMUSG00000020196	ENSMUSG00000020196	ENSMUSG00000020196	10	75090260	75198620	178	203		
8754	22	22696752	22700882	ENSMUSG00000001665	ENSMUSG00000001665	ENSMUSG00000001665	10	75217838	75225130	178	203		
8755	22	22706142	22714271	ENSMUSG00000001663	ENSMUSG00000001663	ENSMUSG00000001663	10	75227531	75242300	178	203		
8756	22	22507014	22511201	ENSMUSG00000009092	ENSMUSG00000009092	ENSMUSG00000009092	10	75337114	75339657	178	203		
8757	22	22459150	22506692	ENSMUSG00000000902	ENSMUSG00000000902	ENSMUSG00000000902	10	75340491	75365312	178	203		
8758	22	22445036	22456502	ENSMUSG00000000901	ENSMUSG00000000901	ENSMUSG00000000901	10	75366940	75376185	178	203		
8759	22	22438024	22440141	ENSMUSG00000049422	ENSMUSG00000049422	ENSMUSG00000049422	10	75377183	75381460	178	203		
8760	22	22435208	22437919	ENSMUSG00000050157	ENSMUSG00000050157	ENSMUSG00000050157	10	75381437	75384388	178	203		
8761	22	22424937	22426655	ENSMUSG00000000903	ENSMUSG00000000903	ENSMUSG00000000903	10	75392028	75393362	178	203		
8762	21	46879507	46909280	ENSMUSG00000020230	ENSMUSG00000020230	ENSMUSG00000020230	10	75650945	75681491	179	204		
8763	21	46842968	46849424	ENSMUSG00000033208	ENSMUSG00000033208	ENSMUSG00000033208	10	75697569	75703788	179	204		
8764	21	46703318	46813028	ENSMUSG00000020231	ENSMUSG00000020231	ENSMUSG00000020231	10	75706767	75788913	179	204		
8765	21	46568483	46690106	ENSMUSG00000001151	ENSMUSG00000001151	ENSMUSG00000001151	10	75794980	75886622	179	204		
8766	21	46545474	46568212	ENSMUSG00000009114	ENSMUSG00000009114	ENSMUSG00000009114	10	75892860	75904831	179	204		
8767	21	46530695	46542092	ENSMUSG00000033126	ENSMUSG00000033126	ENSMUSG00000033126	10	75903285	75912830	179	204		
8768	21	46479476	46529664	ENSMUSG00000001150	ENSMUSG00000001150	ENSMUSG00000001150	10	75912643	75959569	179	204		
8769	21	46432793	46473119	ENSMUSG00000033105	ENSMUSG00000033105	ENSMUSG00000033105	10	75975334	75999197	179	204		
8770	21	46405498	46427158	ENSMUSG00000009115	ENSMUSG00000009115	ENSMUSG00000009115	10	76005988	76013916	179	204		
8771	21	46380610	46399909	ENSMUSG00000001155	ENSMUSG00000001155	ENSMUSG00000001155	10	76019383	76033275	179	204		
8772	21	46342470	46377189	ENSMUSG00000020241	ENSMUSG00000020241	ENSMUSG00000020241	10	76039478	76067043	179	204		

8773	21	46226111	46248443	ENSMUSG00000001119	ENSMUSG00000001119	ENSMUSG00000001119	10	76152508	76169760	179	204
8774	21	46140636	46186795	ENSMUSG00000001120	ENSMUSG00000001120	ENSMUSG00000001120	10	76205573	76244544	179	204
8775	21	45759057	45786690	ENSMUSG00000001436	ENSMUSG00000001436	ENSMUSG00000001436	10	76476445	76486338	179	204
8776	21	45699873	45758061	ENSMUSG00000001435	ENSMUSG00000001435	ENSMUSG00000001435	10	76496736	76557392	179	204
8777	21	45509825	45532235	ENSMUSG00000020260	ENSMUSG00000020260	ENSMUSG00000020260	10	76703016	76713302	179	204
8778	21	45416000	45466540	ENSMUSG00000020262	ENSMUSG00000020262	ENSMUSG00000020262	10	76734109	76861923	179	204
8779	21	45184359	45221297	ENSMUSG00000032977	ENSMUSG00000032977	ENSMUSG00000032977	10	76930377	76959501	179	204
8780	21	45093941	45118169	ENSMUSG00000009291	ENSMUSG00000009291	ENSMUSG00000009291	10	77025436	77042447	179	204
8781	21	45049960	45062472	ENSMUSG00000020265	ENSMUSG00000020265	ENSMUSG00000020265	10	77049986	77061092	179	204
8782	21	45013384	45046166	ENSMUSG00000009293	ENSMUSG00000009293	ENSMUSG00000009293	10	77066037	77089706	179	204
8783	21	44742205	44955923	ENSMUSG00000069581	ENSMUSG00000069581	ENSMUSG00000069581	10	77232563	77284382	179	204
8784	21	44699797	44703158	ENSMUSG00000051652	ENSMUSG00000051652	ENSMUSG00000051652	10	77302976	77305639	179	204
8785	21	44594474	44687392	ENSMUSG00000009292	ENSMUSG00000009292	ENSMUSG00000009292	10	77312056	77372983	179	204
8786	21	44574202	44583704	ENSMUSG00000020284	ENSMUSG00000020284	ENSMUSG00000020284	10	77381771	77388563	179	204
8787	21	44544358	44571681	ENSMUSG00000020277	ENSMUSG00000020277	ENSMUSG00000020277	10	77390400	77412878	179	204
8788	21	44530191	44542486	ENSMUSG00000000731	ENSMUSG00000000731	ENSMUSG00000000731	10	77433160	77446709	179	204
8789	21	44490651	44506527	ENSMUSG00000000730	ENSMUSG00000000730	ENSMUSG00000000730	10	77452971	77466744	179	204
8790	21	44467313	44485151	ENSMUSG00000000732	ENSMUSG00000000732	ENSMUSG00000000732	10	77474279	77480310	179	204
8791	21	44377924	44389259	ENSMUSG00000053329	ENSMUSG00000053329	ENSMUSG00000053329	10	77565719	77572897	179	204
8792	21	44351636	44375490	ENSMUSG00000032834	ENSMUSG00000032834	ENSMUSG00000032834	10	77575089	77587314	179	204
8793	21	44256634	44350852	ENSMUSG00000000374	ENSMUSG00000000374	ENSMUSG00000000374	10	77590988	77647631	179	204
8794	21	44209238	44226701	ENSMUSG00000001211	ENSMUSG00000001211	ENSMUSG00000001211	10	77674692	77754829	179	204
8795	21	44033859	44049585	ENSMUSG00000061032	ENSMUSG00000061032	ENSMUSG00000061032	10	77803513	77816124	179	204
8796	21	44016826	44020585	ENSMUSG00000005054	ENSMUSG00000005054	ENSMUSG00000005054	10	77828799	77830755	179	204
8797	19	15086789	15097577	ENSMUSG00000032763	ENSMUSG00000032763	ENSMUSG00000032763	10	77977671	77987622	180	205
8798	19	15079214	15086789	ENSMUSG00000032714	ENSMUSG00000032714	ENSMUSG00000032714	10	77987637	77995075	180	205
8799	19	15058877	15059944	ENSMUSG00000071185	ENSMUSG00000071185	ENSMUSG00000071185	10	78014827	78015768	180	205
8800	19	15024015	15027900	ENSMUSG00000005355	ENSMUSG00000005355	ENSMUSG00000005355	10	78115126	78121371	180	205
8801	19	14921991	14944730	ENSMUSG00000005357	ENSMUSG00000005357	ENSMUSG00000005357	10	78183631	78217894	180	205
8802	19	232048	242169	ENSMUSG00000052151	ENSMUSG00000052151	ENSMUSG00000052151	10	78929564	78936892	181	206
8803	19	256575	295791	ENSMUSG00000042570	ENSMUSG00000042570	ENSMUSG00000042570	10	78944639	78953541	181	206
8804	19	313059	327009	ENSMUSG00000020317	ENSMUSG00000020317	ENSMUSG00000020317	10	78979606	78990427	181	206
8805	19	356508	360145	ENSMUSG00000045912	ENSMUSG00000045912	ENSMUSG00000045912	10	79009983	79017129	181	206
8806	19	367593	411903	ENSMUSG00000020312	ENSMUSG00000020312	ENSMUSG00000020312	10	79022632	79040904	181	206
8807	19	414360	425983	ENSMUSG00000035963	ENSMUSG00000035963	ENSMUSG00000035963	10	79042655	79048867	181	206
8808	19	447490	456342	ENSMUSG00000020310	ENSMUSG00000020310	ENSMUSG00000020310	10	79067712	79071665	181	206
8809	19	458507	470423	ENSMUSG00000020308	ENSMUSG00000020308	ENSMUSG00000020308	10	79072539	79079066	181	206
8810	19	495027	500919	ENSMUSG00000054206	ENSMUSG00000054206	ENSMUSG00000054206	10	79092149	79098389	181	206
8811	19	523537	534492	ENSMUSG00000023175	ENSMUSG00000023175	ENSMUSG00000023175	10	79107635	79115097	181	206
8812	19	540893	568157	ENSMUSG00000020331	ENSMUSG00000020331	ENSMUSG00000020331	10	79119763	79139237	181	206
8813	19	568228	584568	ENSMUSG00000020329	ENSMUSG00000020329	ENSMUSG00000020329	10	79139252	79149702	181	206
8814	19	590920	594605	ENSMUSG00000020327	ENSMUSG00000020327	ENSMUSG00000020327	10	79158243	79161725	181	206
8815	19	627403	634385	ENSMUSG00000020325	ENSMUSG00000020325	ENSMUSG00000020325	10	79180423	79184604	181	206
8816	19	636548	646483	ENSMUSG00000020323	ENSMUSG00000020323	ENSMUSG00000020323	10	79186440	79190659	181	206
8817	19	660002	699328	ENSMUSG00000035863	ENSMUSG00000035863	ENSMUSG00000035863	10	79196845	79224025	181	206
8818	19	702126	715318	ENSMUSG00000035852	ENSMUSG00000035852	ENSMUSG00000035852	10	79224172	79233619	181	206
8819	19	763435	772967	ENSMUSG00000035835	ENSMUSG00000035835	ENSMUSG00000035835	10	79267988	79277223	181	206
8820	19	791985	799175	ENSMUSG00000057729	ENSMUSG00000057729	ENSMUSG00000057729	10	79282796	79286303	181	206
8821	19	803291	807242	ENSMUSG00000020125	ENSMUSG00000020125	ENSMUSG00000020125	10	79289464	79291246	181	206
8822	19	810665	814606	ENSMUSG00000061780	ENSMUSG00000061780	ENSMUSG00000061780	10	79294041	79295783	181	206
8823	19	818964	844218	ENSMUSG00000013833	ENSMUSG00000013833	ENSMUSG00000013833	10	79297838	79312021	181	206
8824	19	847532	864231	ENSMUSG00000035781	ENSMUSG00000035781	ENSMUSG00000035781	10	79313182	79320059	181	206
8825	19	868358	872013	ENSMUSG00000035773	ENSMUSG00000035773	ENSMUSG00000035773	10	79320100	79325312	181	206
8826	19	877037	923781	ENSMUSG00000019564	ENSMUSG00000019564	ENSMUSG00000019564	10	79330201	79358141	181	206
8827	19	935328	945569	ENSMUSG00000035754	ENSMUSG00000035754	ENSMUSG00000035754	10	79363281	79372375	181	206
8828	19	951437	960721	ENSMUSG00000035745	ENSMUSG00000035745	ENSMUSG00000035745	10	79373844	79380319	181	206
8829	19	960650	972141	ENSMUSG00000013858	ENSMUSG00000013858	ENSMUSG00000013858	10	79380249	79387291	181	206
8830	19	992361	1016424	ENSMUSG00000035722	ENSMUSG00000035722	ENSMUSG00000035722	10	79400744	79418701	181	206
8831	19	1018174	1037627	ENSMUSG00000035697	ENSMUSG00000035697	ENSMUSG00000035697	10	79419955	79434601	181	206
8832	19	1039166	1046314	ENSMUSG00000004667	ENSMUSG00000004667	ENSMUSG00000004667	10	79439079	79442839	181	206
8833	19	1058658	1125259	ENSMUSG00000035673	ENSMUSG00000035673	ENSMUSG00000035673	10	79460141	79505831	181	206
8834	19	1156798	1179434	ENSMUSG00000003068	ENSMUSG00000003068	ENSMUSG00000003068	10	79519331	79533808	181	206
8835	19	1180178	1188841	ENSMUSG00000035640	ENSMUSG00000035640	ENSMUSG00000035640	10	79533681	79540516	181	206
8836	19	1192749	1195823	ENSMUSG00000003072	ENSMUSG00000003072	ENSMUSG00000003072	10	79545494	79548940	181	206
8837	19	1199552	1210139	ENSMUSG00000035621	ENSMUSG00000035621	ENSMUSG00000035621	10	79551419	79561496	181	206
8838	19	1220336	1224169	ENSMUSG00000045193	ENSMUSG00000045193	ENSMUSG00000045193	10	79572943	79573907	181	206
8839	19	1226520	1230227	ENSMUSG00000035595	ENSMUSG00000035595	ENSMUSG00000035595	10	79576073	79578248	181	206
8840	19	1237168	1250944	ENSMUSG00000003070	ENSMUSG00000003070	ENSMUSG00000003070	10	79582611	79592774	181	206
8841	19	1306010	1329427	ENSMUSG00000020156	ENSMUSG00000020156	ENSMUSG00000020156	10	79631455	79647032	181	206
8842	19	1334903	1346583	ENSMUSG00000020153	ENSMUSG00000020153	ENSMUSG00000020153	10	79652581	79659921	181	206
8843	19	1348089	1352552	ENSMUSG00000020150	ENSMUSG00000020150	ENSMUSG00000020150	10	79661328	79664141	181	206
8844	19	1358584	1386683	ENSMUSG00000069565	ENSMUSG00000069565	ENSMUSG00000069565	10	79668203	79691535	181	206
8845	19	1401148	1424243	ENSMUSG00000020135	ENSMUSG00000020135	ENSMUSG00000020135	10	79704967	79719154	181	206
8846	19	1425814	1430211	ENSMUSG00000020133	ENSMUSG00000020133	ENSMUSG00000020133	10	79721383	79723644	181	206
8847	19	1432427	1441407	ENSMUSG00000020131	ENSMUSG00000020131	ENSMUSG00000020131	10	79724431	79732602	181	206
8848	19	1442165	1448923	ENSMUSG00000035504	ENSMUSG00000035504	ENSMUSG00000035504	10	79733360	79737930	181	206
8849	19	1456023	1464019	ENSMUSG00000043822	ENSMUSG00000043822	ENSMUSG00000043822	10	79744290	79748542	181	206
8850	19	1506330	1518523	ENSMUSG00000048696	ENSMUSG00000048696	ENSMUSG00000048696	10	79783609	79790228	181	206
8851	19	1527678	1543652	ENSMUSG00000035478	ENSMUSG00000035478	ENSMUSG00000035478	10	79795670	79802608	181	206
8852	19	1561964	1603328	ENSMUSG00000020167	ENSMUSG00000020167	ENSMUSG00000020167	10	79812737	79836750	181	206
8853	19	1726154	1726444	ENSMUSG00000045518	ENSMUSG00000045518	ENSMUSG00000045518	10	79898035	79920405	181	206
8854	19	1733076	1763270	ENSMUSG00000003341	ENSMUSG00000003341	ENSMUSG00000003341	10	79922714	79942253	181	206
8855	19	1803399	1814496	ENSMUSG00000035397	ENSMUSG00000035397	ENSMUSG00000035397	10	79970264	79980423	181	206
8856	19	1856417	1864444	ENSMUSG00000035370	ENSMUSG00000035370	ENSMUSG00000035370	10	80009150	80018912	181	206
8857	19	1892188	1931455	ENSMUSG00000003345	ENSMUSG00000003345	ENSMUSG00000003345	10	80025967	80043870	181	206
8858	19	1937487	1966702	ENSMUSG00000003344	ENSMUSG00000003344	ENSMUSG00000003344	10	80045754	80051813	181	206
8859	19	1988481	2002233	ENSMUSG00000020190	ENSMUSG00000020190	ENSMUSG00000020190	10	80070236	80079003	181	206
8860	19	2022037	2047304	ENSMUSG00000003348	ENSMUSG00000003348	ENSMUSG00000003348	10	80088383	80104949	181	206
8861	19	2035101	2050581	ENSMUSG00000055862	ENSMUSG00000055862	ENSMUSG00000055862	10	80105313	80108501	181	206
8862	19	2051993	2102540	ENSMUSG00000020198	ENSMUSG00000020198	ENSMUSG00000020198	10	80110086	80145356	181	206
8863	19	2115148	2179346	ENSMUSG00000061589	ENSMUSG00000061589	ENSMUSG00000061589	10	80158335	80195867	181	206
8864	19	2184155	2187319	ENSMUSG00000035278	ENSMUSG00000035278	ENSMUSG00000035278	10	80199230	80201716	181	206
8865	19	2200122	2203071	ENSMUSG00000035262	ENSMUSG00000035262	ENSMUSG00000035262	10	80208377	80210777	181	206
8866	19	2203252	2207416	ENSMUSG00000020216	ENSMUSG00000020216	ENSMUSG00000020216	10	80211630	80216624	181	206
8867	19	2220520	2224487	ENSMUSG00000035242	ENSMUSG00000035242	ENSMUSG00000035242	10	80229785	80232414	181	206
8868	19	2240997	2242841	ENSMUSG00000051067	ENSMUSG00000051067	ENSMUSG00000051067	10	80235930	80247159	181	206
8869	19	2279680	2305799	ENSMUSG00000035206	ENSMUSG00000035206	ENSMUSG00000035206	10	80258386	80271834	181	206
8870	19	2340784	2377086	ENSMUSG00000059406	ENSMUSG00000059406	ENSMUSG00000059406	10	80282945	80302469	181	206
8871	19	2376630	2378875	ENSMUSG00000020219	ENSMUSG00000020219	ENSMUSG00000020219	10	80302579	80303895	181	206
8872	19	2379165	2407958	ENSMUSG00000062075	ENSMUSG00000062075	ENSMUSG00000062075	10	80304493	80321362	181	206
8873	19	2427135	2429257	ENSMUSG00000015312	ENSMUSG00000015312	ENSMUSG00000015312	10	80333216	80335333	181	206
8874	19	2665566	2672369	ENSMUSG00000043670	ENSMUSG00000043670	ENSMUSG00000043670	10	80422719	80428506	181	206
8875	19	2683525	2691074	ENSMUSG00000046822	ENSMUSG00000046822	ENSMUSG00000046822	10	80431671	80440379	181	206
8876	19	2705712	2734354	ENSMUSG00000004937	ENSMUSG00000004937	ENSMUSG00000004937	10	80447204	80463291	181	206
8877	19	2736506	2764588	ENSMUSG00000004929	ENSMUSG00000004929	ENSMUSG00000004929	10	80473279	80485687	181	206
8878	19	4104629	4124050	ENSMUSG00000035041	ENSMUSG00000035041	ENSMUSG00000035041	10	80487462	80502001	182	207
8879	19	4041331	4075126	ENSMUSG00000035027	ENSMUSG00000035027	ENSMUSG00000035027	10	80509092	80527465	182	207
8880	19	3996217	4017816	ENSMUSG00000035011	ENSMUSG00000035011	ENSMUSG00000035011	10	80547087	80551582	182	207
8881	19	3958748	3990383	ENSMUSG00000004934	ENSMUSG00000004934	ENSMUSG00000004934	10	80557102	80567322	182	207
8882	19	3909454	3922038	ENSMUSG00000034974	ENSMUSG00000034974	ENSMUSG00000034974	10	80587134	80596127	182	207
8883	19	3884101	3893412	ENSMUSG00000004939	ENSMUSG00000004939	ENSMUSG00000004939	10	80602322	80605180	182	207
8884	19	3831672	3873184	ENSMUSG00000034958	ENSMUSG00000034958	ENSMUSG00000034958	10	80607642	80633908	182	207
8885	19	3728968	3740345	ENSMUSG00000004933	ENSMUSG00000004933	ENSMUSG00000004933	10	80660674	80666110	182	207
8886	19	3713672	3718562	ENSMUSG00000034932	ENSMUSG00000034932	ENSMUSG00000034932	10	80667851	80670055	182	207
8887	19	3701771	3712673	ENSMUSG00000004931	ENSMUSG00000004931	ENSMUSG00000004931	10	80671301	80676356	182	207
8888	19	3679374	3701682	ENSMUSG00000034917	ENSMUSG00000034917	ENSMUSG00000034917	10	80676336	80694342	182	207
8889	19	3581182	3651445	ENSMUSG00000034902	ENSMUSG00000034902	ENSMUSG00000034902	10	80696188	80720532	182	207
8890	19	3561665	3574961	ENSMUSG00000034889	ENSMUSG00000034889	ENSMUSG00000034889	10	80726781	80729368	182	207
8891	19	3545778	3557658	ENSMUSG00000034881	ENSMUSG00000034881	ENSMUSG00000034881	10	80731957	80738300	182	207
8892	19	3536569	3541434	ENSMUSG00000034872	ENSMUSG00000034872	ENSMUSG00000034872	10	80740891	80746395	182	207
8893	19	3495198	3508571	ENSMUSG00000034854	ENSMUSG00000034854	ENSMUSG00000034854	10	80760727	80767285	182	207
8894	19	3490176	3495002	ENSMUSG00000020234	ENSMUSG00000020234	ENSMUSG00000020234	10	80767178	80768919	182	207
8895	19	3457368	3487191	ENSMUSG00000020235	ENSMUSG00000020235	ENSMUSG00000020235	10	80770008	80781499	182	207
8896	19	3441824	3451621	ENSMUSG00000020236	ENSMUSG00000020236	ENSMUSG00000020236	10	80787592	80791477	182	207
8897	19	3317635	3414603	ENSMUSG00000055053	ENSMUSG00000055053	ENSMUSG00000055053	10	80799320	80830302	182	207
8898	19	3175701	3248071	ENSMUSG00000034818	ENSMUSG00000034818	ENSMUSG00000034818	10	80871075	80874486	182	207
8899	19	3136875	3160572	ENSMUSG00000020238	ENSMUSG00000020238	ENSMUSG00000020238	10	80889378	80899485	182	207
8900	19	3129766	3131329	ENSMUSG00000044199	ENSMUSG00000044199	ENSMUSG00000044199	10	80901607	80902767	182	207
8901	19	3087230	3114741	ENSMUSG00000034792	ENSMUSG00000034792	ENSMUSG00000034792	10	80905439	80927354	182	207
8902	19	3045408	3072452	ENSMUSG00000034781	ENSMUSG00000034781	ENSMUSG00000034781	10	80931861	80948175	182	207
8903	19	3003908	3014010	ENSMUSG00000054452	ENSMUSG00000054452	ENSMUSG00000054452	10	80962732	80969293	182	207
8904	19	2948637	2980022	ENSMUSG00000034771	ENSMUSG00000034771	ENSMUSG00000034771	10	80978661	80993974	182	207
8905	19	2928562	2946176	ENSMUSG00000034758	ENSMUSG00000034758	ENSMUSG00000034758	10	80994034	81004029	182	207
8906	19	4125106	4133596	ENSMUSG00000034748	ENSMUSG00000034748	ENSMUSG00000034748	10	81024914	81030687	183	208
8907	19	4149434	4168773	ENSMUSG00000054708	ENSMUSG00000054708	ENSMUSG00000054708	10	81032676	81050743	183	208
8908	12	102868116	102875101	ENSMUSG00000063320	ENSMUSG00000063320	ENSMUSG00000063320	10	82049657	82052910	184	209
8909	12	102907204	102982091	ENSMUSG00000020251	ENSMUSG00000020251	ENSMUSG00000020251	10	82082342	82120416	184	209
8910	12	102982457	103021373	ENSMUSG00000020246	ENSMUSG00000020246	ENSMUSG00000020246	10	82128943	82169090	184	209
8911	12	103034988	103056170	ENSMUSG00000020248	ENSMUSG00000020248	ENSMUSG00000020248	10	82180214	82186594	184	209
8912	12	103374906	103676013	ENSMUSG00000034612	ENSMUSG00000034612	ENSMUSG00000034612	10	82415296	82625699	184	209
8913	12	103722495	103846563	ENSMUSG00000034591	ENSMUSG00000034591	ENSMUSG00000034591	10	82660938	82767616	184	209
8914	12	103904245	103912620	ENSMUSG00000020255	ENSMUSG00000020255	ENSMUSG00000020255	10	82790037	82798634	184	209
8915	12	103942332	104002366	ENSMUSG00000020256	ENSMUSG00000020256	ENSMUSG00000020256	10	82917249	82963861	184	209
8916	12	104025622	104087034	ENSMUSG00000034560	ENSMUSG00000034560	ENSMUSG00000034560	10	82973771	83021584	184	209
8917	12	104091205	104154138	ENSMUSG00000020263	ENSMUSG00000020263	ENSMUSG00000020263	10	83029832	83078463	184	209
8918	12	104981255	105057941	ENSMUSG00000020032	ENSMUSG00000020032	ENSMUSG00000020032	10	83801104	83870270	184	209
8919	12	105156585	105165759	ENSMUSG00000046841	ENSMUSG00000046841	ENSMUSG00000046841	10	83956104	83963687	184	209
8920	12	105220711	105264920	ENSMUSG00000020034	ENSMUSG00000020034	ENSMUSG00000020034	10	84006746	84044158	184	209
8921	12	105275619	105428104	ENSMUSG00000034453	ENSMUSG00000034453	ENSMUSG00000034453	10	84052236	84156977	184	209
8922	12	105501163	105680710	ENSMUSG00000020037	ENSMUSG00000020037	ENSMUSG00000020037	10	84185847	84336335	184	209
8923	12	105692579	105805190	ENSMUSG00000035620	ENSMUSG00000035620	ENSMUSG00000035620	10	84347415	84446246	184	209
8924	12	105873815	105891889	ENSMUSG00000060935	ENSMUSG00000060935	ENSMUSG00000060935	10	84532542	84550417	184	209
8925	12	105895199	105905059	ENSMUSG00000049038	ENSMUSG00000049038	ENSMUSG00000049038	10	84549235	84557800	184	209
8926	12	105909289	106011737	ENSMUSG00000020038	ENSMUSG00000020038	ENSMUSG00000020038	10	84561499	84614853	184	209
8927	12	106236848	106575625	ENSMUSG00000020042	ENSMUSG00000020042	ENSMUSG00000020042	10	84816613	85090088	184	209
8928	12	106603720	106630387	ENSMUSG00000001785	ENSMUSG00000001785	ENSMUSG00000001785	10	85301664	85318895	184	209
8929	12	106650774	106679044	ENSMUSG00000035529	ENSMUSG00000035529	ENSMUSG00000035529	10	85321763	85346744	184	209
8930	12	106692292	106694525	ENSMUSG00000057741	ENSMUSG00000057741	ENSMUSG00000057741	10	85358263	85358724	184	209
8931	22	31113569	31138233	ENSMUSG00000001783	ENSMUSG00000001783	ENSMUSG00000001783	10	85368436	85387592	185	210
8932	22	31140290	31183373	ENSMUSG00000050108	ENSMUSG00000050108	ENSMUSG00000050108	10	85390218	85430438	185	210
8933	22	31201244	31224788	ENSMUSG00000001786	ENSMUSG00000001786	ENSMUSG00000001786	10	85451796	85478130	185	210
8934	22	31239679	31732647	ENSMUSG00000059602	ENSMUSG00000059602	ENSMUSG00000059602	10	85485331	85897088	185	210
8935	22	31526802	31589025	ENSMUSG00000020044	ENSMUSG00000020044	ENSMUSG00000020044	10	85730336	85779307	185	210
8936	12	102505385	102684244	ENSMUSG00000035459	ENSMUSG00000035459	ENSMUSG00000035459	10	86270997	86437741	186	211
8937	12	102219851	102413876	ENSMUSG00000047129	ENSMUSG00000047129	ENSMUSG00000047129	10	86659804	86660241	186	211
8938	12	101875594	101878417	ENSMUSG00000020052	ENSMUSG00000020052	ENSMUSG00000020052	10	86778011	86922980	186	211
8939	12	101756234	101835511	ENSMUSG00000020051	ENSMUSG00000020051	ENSMUSG00000020051	10	86951736	87013930	186	211
8940	12	101335404	101398471	ENSMUSG00000020053	ENSMUSG00000020053	ENSMUSG00000020053	10	87289829	87345569	186	211
8941	12	101038155	101115421	ENSMUSG00000035365	ENSMUSG00000035365	ENSMUSG00000035365	10	87521232	87576740	186	211
8942	12	100992105	101036491	ENSMUSG00000035351	ENSMUSG00000035351	ENSMUSG00000035351	10	87576791	87608184	186	211
8943	12	100930851	100979977	ENSMUSG00000020056	ENSMUSG00000020056	ENSMUSG00000020056	10	87630892	87675957	186	211
8944	12	100795606	100841520	ENSMUSG00000020057	ENSMUSG00000020057	ENSMUSG00000020057	10	87752603	87786874	186	211
8945	12	100663409	100748763	ENSMUSG00000035311	ENSMUSG00000035311	ENSMUSG00000035311	10	87809218	87877120	186	211
8946	12	100615548	100646977	ENSMUSG00000060002	ENSMUSG00000060002	ENSMUSG00000060002	10	87903329	87933664	186	211
8947	12	100512878	100603789	ENSMUSG00000020061	ENSMUSG00000020061	ENSMUSG00000020061	10	87952755	88015495	186	211
8948	12	100393330	100404900	ENSMUSG00000004359	ENSMUSG00000004359	ENSMUSG00000004359	10	88105463	88109754	186	211
8949	12	100312565	100325645	ENSMUSG00000060904	ENSMUSG00000060904	ENSMUSG00000060904	10	88161165	88173856	186	211
8950	12	100198048	100304524	ENSMUSG00000004356	ENSMUSG00000004356	ENSMUSG00000004356	10	88176407	88256449	186	211
8951	12	100073697	100128120	ENSMUSG00000020062	ENSMUSG00000020062	ENSMUSG00000020062	10	88315791	88359313	186	211
8952	12	99712716	100046539	ENSMUSG00000035189	ENSMUSG00000035189	ENSMUSG00000035189	10	88378793	88687589	186	211
8953	12	99512769	99542812	ENSMUSG00000074802	ENSMUSG00000074802	ENSMUSG00000074802	10	88843002	88864127	186	211
8954	12	99421269	99481398	ENSMUSG00000047638	ENSMUSG00000047638	ENSMUSG00000047638	10	88884177	88936426	186	211
8955	12	99274988	99339939	ENSMUSG00000019935	ENSMUSG00000019935	ENSMUSG00000019935	10	89003819	89051052	186	211
8956	12	99185680	99258043	ENSMUSG00000069539	ENSMUSG00000069539	ENSMUSG00000069539	10	89069906	89116084	186	211
8957	12	99117996	99142332	ENSMUSG00000019948	ENSMUSG00000019948	ENSMUSG00000019948	10	89141772	89162094	186	211
8958	12	98954996	99060773	ENSMUSG00000019951	ENSMUSG00000019951	ENSMUSG00000019951	10	89174825	89248199	186	211
8959	12	97653469	98902146	ENSMUSG00000058589	ENSMUSG00000058589	ENSMUSG00000058589	10	89500854	90396191	186	211
8960	12	97563209	97653335	ENSMUSG00000019979	ENSMUSG00000019979	ENSMUSG00000019979	10	90419117	90512538	186	211
8961	12	97542167	97562720	ENSMUSG00000019975	ENSMUSG00000019975	ENSMUSG00000019975	10	90512889	90528456	186	211
8962	12	97511534	97519906	ENSMUSG00000061904	ENSMUSG00000061904	ENSMUSG00000061904	10	90546377	90553762	186	211
8963	12	97433573	97452452	ENSMUSG00000019961	ENSMUSG00000019961	ENSMUSG00000019961	10	90591557	90601334	186	211
8964	12	95825147	95871259	ENSMUSG00000019988	ENSMUSG00000019988	ENSMUSG00000019988	10	92114546	92152204	186	211
8965	12	95565884	95683179	ENSMUSG00000054579	ENSMUSG00000054579	ENSMUSG00000054579	10	92347089	92370096	186	211
8966	12	95407516	95458876	ENSMUSG00000020014	ENSMUSG00000020014	ENSMUSG00000020014	10	92464495	92511314	186	211
8967	12	95196171	95318354	ENSMUSG00000020015	ENSMUSG00000020015	ENSMUSG00000020015	10	92590689	92670642	186	211

8968	12	95112338	95185739	ENSMUSG00000008398	ENSMUSG00000008398	ENSMUSG00000008398	10	92678159	92740634	186	211
8969	12	94918742	94953496	ENSMUSG00000015889	ENSMUSG00000015889	ENSMUSG00000015889	10	92883228	92914653	186	211
8970	12	94892141	94914202	ENSMUSG00000020017	ENSMUSG00000020017	ENSMUSG00000020017	10	92918567	92946542	186	211
8971	12	94861202	94886500	ENSMUSG00000015890	ENSMUSG00000015890	ENSMUSG00000015890	10	92953137	92969820	186	211
8972	12	94784958	94860559	ENSMUSG00000036168	ENSMUSG00000036168	ENSMUSG00000036168	10	92975268	93012860	186	211
8973	12	94575715	94708518	ENSMUSG00000020019	ENSMUSG00000020019	ENSMUSG00000020019	10	93071000	93175685	186	211
8974	12	94392053	94432250	ENSMUSG00000036112	ENSMUSG00000036112	ENSMUSG00000036112	10	93288288	93321001	186	211
8975	12	94135736	94219075	ENSMUSG00000036099	ENSMUSG00000036099	ENSMUSG00000036099	10	93399992	93436899	186	211
8976	12	93998948	94135371	ENSMUSG00000020021	ENSMUSG00000020021	ENSMUSG00000020021	10	93465960	93575137	186	211
8977	12	93939802	93991339	ENSMUSG00000005897	ENSMUSG00000005897	ENSMUSG00000005897	10	93577812	93625483	186	211
8978	12	93889240	93921642	ENSMUSG00000020022	ENSMUSG00000020022	ENSMUSG00000020022	10	93628803	93650747	186	211
8979	12	93486184	93568455	ENSMUSG00000020023	ENSMUSG00000020023	ENSMUSG00000020023	10	93944678	94020753	186	211
8980	12	93226189	93377851	ENSMUSG00000020024	ENSMUSG00000020024	ENSMUSG00000020024	10	94118589	94220135	186	211
8981	12	93066630	93223356	ENSMUSG00000074785	ENSMUSG00000074785	ENSMUSG00000074785	10	94222985	94374266	186	211
8982	12	92595282	92768658	ENSMUSG00000045867	ENSMUSG00000045867	ENSMUSG00000045867	10	94604434	94753785	186	211
8983	12	92295833	92324182	ENSMUSG00000020029	ENSMUSG00000020029	ENSMUSG00000020029	10	94976699	94993608	186	211
8984	12	91693257	91847102	ENSMUSG00000036499	ENSMUSG00000036499	ENSMUSG00000036499	10	95403650	95474831	186	211
8985	12	91061030	91063751	ENSMUSG00000036478	ENSMUSG00000036478	ENSMUSG00000036478	10	96046703	96049313	186	211
8986	12	90063167	90100937	ENSMUSG00000019929	ENSMUSG00000019929	ENSMUSG00000019929	10	96912055	96947839	186	211
8987	12	90021363	90029673	ENSMUSG00000036446	ENSMUSG00000036446	ENSMUSG00000036446	10	96995491	97002391	186	211
8988	12	89968407	89976262	ENSMUSG00000019932	ENSMUSG00000019932	ENSMUSG00000019932	10	97036871	97043375	186	211
8989	12	89881591	89922934	ENSMUSG00000019936	ENSMUSG00000019936	ENSMUSG00000019936	10	97073756	97111588	186	211
8990	12	89871430	89872650	ENSMUSG00000047025	ENSMUSG00000047025	ENSMUSG00000047025	10	97122747	97124614	186	211
8991	12	88505959	88573975	ENSMUSG00000019943	ENSMUSG00000019943	ENSMUSG00000019943	10	98344858	98453480	186	211
8992	12	88337636	88443908	ENSMUSG00000019952	ENSMUSG00000019952	ENSMUSG00000019952	10	98537301	98627676	186	211
8993	12	88265970	88270427	ENSMUSG00000019960	ENSMUSG00000019960	ENSMUSG00000019960	10	98692919	98697172	186	211
8994	12	87410697	87498369	ENSMUSG00000019966	ENSMUSG00000019966	ENSMUSG00000019966	10	99481534	99518923	186	211
8995	12	87060218	87116936	ENSMUSG00000036676	ENSMUSG00000036676	ENSMUSG00000036676	10	99877120	99916991	186	211
8996	12	86966925	87059215	ENSMUSG00000019971	ENSMUSG00000019971	ENSMUSG00000019971	10	99917995	100002792	186	211
8997	12	86953399	86968067	ENSMUSG00000046567	ENSMUSG00000046567	ENSMUSG00000046567	10	100001962	100018903	186	211
8998	12	86897947	86947307	ENSMUSG00000056912	ENSMUSG00000056912	ENSMUSG00000056912	10	100022103	100047997	186	211
8999	12	84896647	84967646	ENSMUSG00000019888	ENSMUSG00000019888	ENSMUSG00000019888	10	101588767	101821004	186	211
9000	12	84792206	84800894	ENSMUSG00000019890	ENSMUSG00000019890	ENSMUSG00000019890	10	101911446	101920123	186	211
9001	12	84722611	84723873	ENSMUSG00000044921	ENSMUSG00000044921	ENSMUSG00000044921	10	101974492	101975754	186	211
9002	12	84198016	84219690	ENSMUSG00000036602	ENSMUSG00000036602	ENSMUSG00000036602	10	102437535	102458465	186	211
9003	12	83954230	84042767	ENSMUSG00000019892	ENSMUSG00000019892	ENSMUSG00000019892	10	102557794	102665994	186	211
9004	12	83777402	83830705	ENSMUSG00000019894	ENSMUSG00000019894	ENSMUSG00000019894	10	102797496	102849057	186	211
9005	12	81605065	82052194	ENSMUSG00000036019	ENSMUSG00000036019	ENSMUSG00000036019	10	104591771	104978561	186	211
9006	12	81276455	81397069	ENSMUSG00000036009	ENSMUSG00000036009	ENSMUSG00000036009	10	105167357	105245464	186	211
9007	12	81270755	81276297	ENSMUSG00000019897	ENSMUSG00000019897	ENSMUSG00000019897	10	105245172	105251460	186	211
9008	12	80179892	80672131	ENSMUSG00000053825	ENSMUSG00000053825	ENSMUSG00000053825	10	105874449	106336628	186	211
9009	12	79995940	80173713	ENSMUSG00000035948	ENSMUSG00000035948	ENSMUSG00000035948	10	106369192	106527778	186	211
9010	12	79715306	79855825	ENSMUSG00000019906	ENSMUSG00000019906	ENSMUSG00000019906	10	106675971	106816281	186	211
9011	12	79634932	79637578	ENSMUSG00000000435	ENSMUSG00000000435	ENSMUSG00000000435	10	106887018	106890244	186	211
9012	12	79625583	79627382	ENSMUSG00000035923	ENSMUSG00000035923	ENSMUSG00000035923	10	106896963	106898847	186	211
9013	12	79274265	79297001	ENSMUSG00000053575	ENSMUSG00000053575	ENSMUSG00000053575	10	107166420	107183362	186	211
9014	12	78692317	78852842	ENSMUSG00000019907	ENSMUSG00000019907	ENSMUSG00000019907	10	107566510	107681685	186	211
9015	12	78509878	78608921	ENSMUSG00000035873	ENSMUSG00000035873	ENSMUSG00000035873	10	107736299	107817864	186	211
9016	12	77782685	78369917	ENSMUSG00000035864	ENSMUSG00000035864	ENSMUSG00000035864	10	107901762	108415063	186	211
9017	12	76749328	77130071	ENSMUSG00000020181	ENSMUSG00000020181	ENSMUSG00000020181	10	109088233	109307982	186	211
9018	12	75939157	75983478	ENSMUSG00000020185	ENSMUSG00000020185	ENSMUSG00000020185	10	110150442	110188948	186	211
9019	12	75776647	75796971	ENSMUSG00000020186	ENSMUSG00000020186	ENSMUSG00000020186	10	110324289	110343732	186	211
9020	12	75681499	75771605	ENSMUSG00000035798	ENSMUSG00000035798	ENSMUSG00000035798	10	110345892	110414250	186	211
9021	12	75269720	75477720	ENSMUSG00000020189	ENSMUSG00000020189	ENSMUSG00000020189	10	110568912	110701357	186	211
9022	12	75262643	75266338	ENSMUSG00000035759	ENSMUSG00000035759	ENSMUSG00000035759	10	110702789	110705835	186	211
9023	12	74710583	74711788	ENSMUSG00000020205	ENSMUSG00000020205	ENSMUSG00000020205	10	110910396	110912758	186	211
9024	12	74160801	74179158	ENSMUSG00000056888	ENSMUSG00000056888	ENSMUSG00000056888	10	111389572	111401339	186	211
9025	12	74014686	74050286	ENSMUSG00000020213	ENSMUSG00000020213	ENSMUSG00000020213	10	111464299	111482616	186	211
9026	12	74071148	74111922	ENSMUSG00000020214	ENSMUSG00000020214	ENSMUSG00000020214	10	111487464	111512208	186	211
9027	12	73956027	74010103	ENSMUSG00000035694	ENSMUSG00000035694	ENSMUSG00000035694	10	111567731	111620665	186	211
9028	12	73720127	73889494	ENSMUSG00000035681	ENSMUSG00000035681	ENSMUSG00000035681	10	111675233	111870414	186	211
9029	12	70952730	71345688	ENSMUSG00000050663	ENSMUSG00000050663	ENSMUSG00000050663	10	113803133	114205474	186	211
9030	12	70618893	70712488	ENSMUSG00000006764	ENSMUSG00000006764	ENSMUSG00000006764	10	114482749	114589132	186	211
9031	12	70519806	70604364	ENSMUSG00000020130	ENSMUSG00000020130	ENSMUSG00000020130	10	114602568	114655577	186	211
9032	12	70434925	70467417	ENSMUSG00000020132	ENSMUSG00000020132	ENSMUSG00000020132	10	114693972	114719701	186	211
9033	12	70366145	70382568	ENSMUSG00000069520	ENSMUSG00000069520	ENSMUSG00000069520	10	114744852	114766357	186	211
9034	12	70344051	70360684	ENSMUSG00000020137	ENSMUSG00000020137	ENSMUSG00000020137	10	114774076	114788545	186	211
9035	12	70289745	70343866	ENSMUSG00000034163	ENSMUSG00000034163	ENSMUSG00000034163	10	114802155	114836881	186	211
9036	12	70120031	70264781	ENSMUSG00000020140	ENSMUSG00000020140	ENSMUSG00000020140	10	114855736	114991603	186	211
9037	12	69805144	69838046	ENSMUSG00000034127	ENSMUSG00000034127	ENSMUSG00000034127	10	115221381	115253730	186	211
9038	12	69319191	69600853	ENSMUSG00000020151	ENSMUSG00000020151	ENSMUSG00000020151	10	115422509	115679039	186	211
9039	12	69201231	69289891	ENSMUSG00000020154	ENSMUSG00000020154	ENSMUSG00000020154	10	115705606	115785122	186	211
9040	12	69046329	69111245	ENSMUSG00000054934	ENSMUSG00000054934	ENSMUSG00000054934	10	115821980	115877911	186	211
9041	12	68923489	69033962	ENSMUSG00000020166	ENSMUSG00000020166	ENSMUSG00000020166	10	115889276	115985610	186	211
9042	12	68334689	68379463	ENSMUSG00000020169	ENSMUSG00000020169	ENSMUSG00000020169	10	116390424	116429150	186	211
9043	12	68290052	68290885	ENSMUSG00000060187	ENSMUSG00000060187	ENSMUSG00000060187	10	116449465	116450874	186	211
9044	12	68265475	68281624	ENSMUSG00000034024	ENSMUSG00000034024	ENSMUSG00000034024	10	116455356	116467160	186	211
9045	12	68150453	68255011	ENSMUSG00000020170	ENSMUSG00000020170	ENSMUSG00000020170	10	116474239	116552620	186	211
9046	12	68039799	68070842	ENSMUSG00000020171	ENSMUSG00000020171	ENSMUSG00000020171	10	116619252	116628583	186	211
9047	12	67919663	67949986	ENSMUSG00000055531	ENSMUSG00000055531	ENSMUSG00000055531	10	116751624	116781080	186	211
9048	12	67531227	67612891	ENSMUSG00000020183	ENSMUSG00000020183	ENSMUSG00000020183	10	117032498	117088295	186	211
9049	12	67488247	67520481	ENSMUSG00000020184	ENSMUSG00000020184	ENSMUSG00000020184	10	117091888	117113704	186	211
9050	12	67426203	67446120	ENSMUSG00000060181	ENSMUSG00000060181	ENSMUSG00000060181	10	117136667	117149351	186	211
9051	12	67366998	67422739	ENSMUSG00000052798	ENSMUSG00000052798	ENSMUSG00000052798	10	117153853	117193556	186	211
9052	12	67290919	67340641	ENSMUSG00000052681	ENSMUSG00000052681	ENSMUSG00000052681	10	117217464	117248907	186	211
9053	12	66974613	67012379	ENSMUSG00000020212	ENSMUSG00000020212	ENSMUSG00000020212	10	117532014	117571990	186	211
9054	12	66834816	66839790	ENSMUSG00000055170	ENSMUSG00000055170	ENSMUSG00000055170	10	117844040	117848885	186	211
9055	12	66329021	66340410	ENSMUSG00000028630	ENSMUSG00000028630	ENSMUSG00000028630	10	118262338	118271896	186	211
9056	12	65949426	65994658	ENSMUSG00000020114	ENSMUSG00000020114	ENSMUSG00000020114	10	118605706	118643076	186	211
9057	12	65029066	65359192	ENSMUSG00000034813	ENSMUSG00000034813	ENSMUSG00000034813	10	119222572	119478662	186	211
9058	12	64982623	65018225	ENSMUSG00000020228	ENSMUSG00000020228	ENSMUSG00000020228	10	119486675	119516047	186	211
9059	12	64869270	64928684	ENSMUSG00000020227	ENSMUSG00000020227	ENSMUSG00000020227	10	119544714	119604597	186	211
9060	12	64817459	64850031	ENSMUSG00000020225	ENSMUSG00000020225	ENSMUSG00000020225	10	119611886	119627957	186	211
9061	12	64504507	64595566	ENSMUSG00000056758	ENSMUSG00000056758	ENSMUSG00000056758	10	119865724	119879123	186	211
9062	12	63958755	64146945	ENSMUSG00000051236	ENSMUSG00000051236	ENSMUSG00000051236	10	120184160	120301912	186	211
9063	12	63730674	63801383	ENSMUSG00000020218	ENSMUSG00000020218	ENSMUSG00000020218	10	120437064	120503703	186	211
9064	12	63466058	63558350	ENSMUSG00000052302	ENSMUSG00000052302	ENSMUSG00000052302	10	120667417	120714012	186	211
9065	12	63393491	63439479	ENSMUSG00000034707	ENSMUSG00000034707	ENSMUSG00000034707	10	120768150	120800305	186	211
9066	12	63290560	63375459	ENSMUSG00000025795	ENSMUSG00000025795	ENSMUSG00000025795	10	120813415	120879318	186	211
9067	12	63132167	63182153	ENSMUSG00000020115	ENSMUSG00000020115	ENSMUSG00000020115	10	120949520	120989807	186	211
9068	12	63084500	63128728	ENSMUSG00000034667	ENSMUSG00000034667	ENSMUSG00000034667	10	120992687	121018287	186	211
9069	12	62947034	63070558	ENSMUSG00000047642	ENSMUSG00000047642	ENSMUSG00000047642	10	121044761	121095952	186	211
9070	12	62866366	62902312	ENSMUSG00000053684	ENSMUSG00000053684	ENSMUSG00000053684	10	121142997	121155929	186	211
9071	12	62524808	62823751	ENSMUSG00000020121	ENSMUSG00000020121	ENSMUSG00000020121	10	121188461	121334573	186	211
9072	12	62459931	62489567	ENSMUSG00000034620	ENSMUSG00000034620	ENSMUSG00000034620	10	121481174	121500195	186	211
9073	12	61826483	61832857	ENSMUSG00000020123	ENSMUSG00000020123	ENSMUSG00000020123	10	121851559	121856513	186	211
9074	12	61328469	61615197	ENSMUSG00000034613	ENSMUSG00000034613	ENSMUSG00000034613	10	122081865	122345599	186	211
9075	12	61146896	61273667	ENSMUSG00000034602	ENSMUSG00000034602	ENSMUSG00000034602	10	122398223	122479565	186	211
9076	12	60940405	61084689	ENSMUSG00000020124	ENSMUSG00000020124	ENSMUSG00000020124	10	122516304	122599995	186	211
9077	12	60388322	60703829	ENSMUSG00000044071	ENSMUSG00000044071	ENSMUSG00000044071	10	122996543	123108893	186	211
9078	12	58276115	58460250	ENSMUSG00000020102	ENSMUSG00000020102	ENSMUSG00000020102	10	124629524	124732023	186	211
9079	12	57552205	57600529	ENSMUSG00000020105	ENSMUSG00000020105	ENSMUSG00000020105	10	125369225	125418184	186	211
9080	12	56621627	56637318	ENSMUSG00000025436	ENSMUSG00000025436	ENSMUSG00000025436	10	126271379	126304304	186	211
9081	12	56477704	56496119	ENSMUSG00000025432	ENSMUSG00000025432	ENSMUSG00000025432	10	126403688	126423937	186	211
9082	12	56462851	56477601	ENSMUSG00000040521	ENSMUSG00000040521	ENSMUSG00000040521	10	126425281	126433763	186	211
9083	12	56452650	56462588	ENSMUSG00000069491	ENSMUSG00000069491	ENSMUSG00000069491	10	126435875	126444343	186	211
9084	12	56448523	56452522	ENSMUSG00000006732	ENSMUSG00000006732	ENSMUSG00000006732	10	126444881	126448410	186	211
9085	12	56442385	56447243	ENSMUSG00000006724	ENSMUSG00000006724	ENSMUSG00000006724	10	126451199	126455954	186	211
9086	12	56435167	56439956	ENSMUSG00000040502	ENSMUSG00000040502	ENSMUSG00000040502	10	126458999	126463133	186	211
9087	12	56428272	56432431	ENSMUSG00000006728	ENSMUSG00000006728	ENSMUSG00000006728	10	126466564	126470344	186	211
9088	12	56425051	56428271	ENSMUSG00000006736	ENSMUSG00000006736	ENSMUSG00000006736	10	126470229	126473213	186	211
9089	12	56406602	56418296	ENSMUSG00000025422	ENSMUSG00000025422	ENSMUSG00000025422	10	126481856	126496118	186	211
9090	12	56374153	56401605	ENSMUSG00000040462	ENSMUSG00000040462	ENSMUSG00000040462	10	126498601	126524080	186	211
9091	12	56303460	56313201	ENSMUSG00000006731	ENSMUSG00000006731	ENSMUSG00000006731	10	126568174	126575279	186	211
9092	12	56291485	56297287	ENSMUSG00000019467	ENSMUSG00000019467	ENSMUSG00000019467	10	126585475	126592985	186	211
9093	12	56284874	56289853	ENSMUSG00000040415	ENSMUSG00000040415	ENSMUSG00000040415	10	126594065	126596307	186	211
9094	12	56271253	56283465	ENSMUSG00000025417	ENSMUSG00000025417	ENSMUSG00000025417	10	126600007	126614504	186	211
9095	12	56230289	56266682	ENSMUSG00000074657	ENSMUSG00000074657	ENSMUSG00000074657	10	126628645	126666297	186	211
9096	12	56210202	56227204	ENSMUSG00000025410	ENSMUSG00000025410	ENSMUSG00000025410	10	126669348	126684756	186	211
9097	12	56202892	56210198	ENSMUSG00000025409	ENSMUSG00000025409	ENSMUSG00000025409	10	126684905	126691720	186	211
9098	12	56196640	56200567	ENSMUSG00000025408	ENSMUSG00000025408	ENSMUSG00000025408	10	126693757	126699237	186	211
9099	12	56168106	56196699	ENSMUSG00000040354	ENSMUSG00000040354	ENSMUSG00000040354	10	126699177	126714627	186	211
9100	12	56152314	56164047	ENSMUSG00000040345	ENSMUSG00000040345	ENSMUSG00000040345	10	126725792	126732737	186	211
9101	12	56140201	56152312	ENSMUSG00000025407	ENSMUSG00000025407	ENSMUSG00000025407	10	126732838	126744538	186	211
9102	12	56135363	56138056	ENSMUSG00000047492	ENSMUSG00000047492	ENSMUSG00000047492	10	126752505	126754721	186	211
9103	12	56114810	56130876	ENSMUSG00000025405	ENSMUSG00000025405	ENSMUSG00000025405	10	126759269	126773380	186	211
9104	12	55923509	55931236	ENSMUSG00000040287	ENSMUSG00000040287	ENSMUSG00000040287	10	126904666	126911764	186	211
9105	12	55914961	55920794	ENSMUSG00000040280	ENSMUSG00000040280	ENSMUSG00000040280	10	126917927	126920101	186	211
9106	12	55896870	55906498	ENSMUSG00000040258	ENSMUSG00000040258	ENSMUSG00000040258	10	126929058	126937381	186	211
9107	12	55808549	55893390	ENSMUSG00000040249	ENSMUSG00000040249	ENSMUSG00000040249	10	126941128	127024075	186	211
9108	12	55775462	55791428	ENSMUSG00000002147	ENSMUSG00000002147	ENSMUSG00000002147	10	127046117	127063352	186	211
9109	12	55769157	55775525	ENSMUSG00000025402	ENSMUSG00000025402	ENSMUSG00000025402	10	127063868	127069627	186	211
9110	12	55735698	55758819	ENSMUSG00000040195	ENSMUSG00000040195	ENSMUSG00000040195	10	127080017	127103996	186	211
9111	12	55708569	55730160	ENSMUSG00000025401	ENSMUSG00000025401	ENSMUSG00000025401	10	127108196	127123884	186	211
9112	12	55690053	55696592	ENSMUSG00000025400	ENSMUSG00000025400	ENSMUSG00000025400	10	127127427	127134716	186	211
9113	12	55678885	55686497	ENSMUSG00000044617	ENSMUSG00000044617	ENSMUSG00000044617	10	127142487	127150304	186	211
9114	12	55603205	55614456	ENSMUSG00000040127	ENSMUSG00000040127	ENSMUSG00000040127	10	127301484	127314710	186	211
9115	12	55443375	55467841	ENSMUSG00000025396	ENSMUSG00000025396	ENSMUSG00000025396	10	127393882	127403627	186	211
9116	12	55411647	55432349	ENSMUSG00000025395	ENSMUSG00000025395	ENSMUSG00000025395	10	127418141	127432979	186	211
9117	12	55275647	55316421	ENSMUSG00000040054	ENSMUSG00000040054	ENSMUSG00000040054	10	127511956	127529702	186	211
9118	12	55201993	55269875	ENSMUSG00000040043	ENSMUSG00000040043	ENSMUSG00000040043	10	127532419	127579517	186	211
9119	12	55151004	55168448	ENSMUSG00000044005	ENSMUSG00000044005	ENSMUSG00000044005	10	127597632	127612886	186	211
9120	12	55148618	55151030	ENSMUSG00000051346	ENSMUSG00000051346	ENSMUSG00000051346	10	127612855	127614743	186	211
9121	12	55130151	55134696	ENSMUSG00000025389	ENSMUSG00000025389	ENSMUSG00000025389	10	127628787	127634760	186	211
9122	12	55097177	55129458	ENSMUSG00000039994	ENSMUSG00000039994	ENSMUSG00000039994	10	127635041	127655884	186	211
9123	12	55040653	55057759	ENSMUSG00000047631	ENSMUSG00000047631	ENSMUSG00000047631	10	127670946	127673100	186	211
9124	12	55021651	55040176	ENSMUSG00000040033	ENSMUSG00000040033	ENSMUSG00000040033	10	127673525	127695798	186	211
9125	12	55018926	55020460	ENSMUSG00000025383	ENSMUSG00000025383	ENSMUSG00000025383	10	127699089	127701033	186	211
9126	12	54996998	55013796	ENSMUSG00000005682	ENSMUSG00000005682	ENSMUSG00000005682	10	127706284	127724296	186	211
9127	12	54990484	54996387	ENSMUSG00000025381	ENSMUSG00000025381	ENSMUSG00000025381	10	127725538	127730502	186	211
9128	12	54947334	54951015	ENSMUSG00000039914	ENSMUSG00000039914	ENSMUSG00000039914	10	127766205	127771186	186	211
9129	12	54923196	54938320	ENSMUSG00000014498	ENSMUSG00000014498	ENSMUSG00000014498	10	127780073	127796955	186	211
9130	12	54910805	54917894	ENSMUSG00000039878	ENSMUSG00000039878	ENSMUSG00000039878	10	127798883	127804134	186	211
9131	12	54904418	54909904	ENSMUSG00000025374	ENSMUSG00000025374	ENSMUSG00000025374	10	127804344	127812745	186	211
9132	12	54884555	54902002	ENSMUSG00000025373	ENSMUSG00000025373	ENSMUSG00000025373	10	127814606	127844390	186	211
9133	12	54843034	54869618	ENSMUSG00000025369	ENSMUSG00000025369	ENSMUSG00000025369	10	127862243	127893123	186	211
9134	12	54832471	54838027	ENSMUSG00000039824	ENSMUSG00000039824	ENSMUSG00000039824	10	127897106	127901634	186	211
9135	12	54808302	54824719	ENSMUSG00000025366	ENSMUSG00000025366	ENSMUSG00000025366	10	127913205	127928819	186	211
9136	12	54798297	54802545	ENSMUSG00000039810	ENSMUSG00000039810	ENSMUSG00000039810	10	127944912	127950723	186	211
9137	12	54784628	54793912	ENSMUSG00000025364	ENSMUSG00000025364	ENSMUSG00000025364	10	127960715	127968887	186	211
9138	12	54760159	54783395	ENSMUSG00000018166	ENSMUSG00000018166	ENSMUSG00000018166	10	127972317	127992450	186	211
9139	12	54704791	54717887	ENSMUSG00000002578	ENSMUSG00000002578	ENSMUSG00000002578	10	128035364	128048775	186	211
9140	12	54677310	54685572	ENSMUSG00000049858	ENSMUSG00000049858	ENSMUSG00000049858	10	128072843	128075552	186	211
9141	12	54634157	54646765	ENSMUSG00000025359	ENSMUSG00000025359	ENSMUSG00000025359	10	128109207	128123073	186	211
9142	12	54611213	54634072	ENSMUSG00000025357	ENSMUSG00000025357	ENSMUSG00000025357	10	128123083	128147005	186	211
9143	12	54515511	54523002	ENSMUSG00000025355	ENSMUSG00000025355	ENSMUSG00000025355	10	128193922	128202419	186	211
9144	12	54501011	54509603	ENSMUSG00000025354	ENSMUSG00000025354	ENSMUSG00000025354	10	128208625	128222395	186	211
9145	12	54498101	54501736	ENSMUSG00000025353	ENSMUSG00000025353	ENSMUSG00000025353	10	128220851	128224543	186	211
9146	12	54423331	54430332	ENSMUSG00000025352	ENSMUSG00000025352	ENSMUSG00000025352	10	128288055	128294634	186	211
9147	12	54400464	54404754	ENSMUSG00000025350	ENSMUSG00000025350	ENSMUSG00000025350	10	128316537	128322222	186	211
9148	12	54364640	54387752	ENSMUSG00000025348	ENSMUSG00000025348	ENSMUSG00000025348	10	128336767	128361229	186	211
9149	12	54361686	54364659	ENSMUSG00000025347	ENSMUSG00000025347	ENSMUSG00000025347	10	128361226	128363937	186	211
9150	12	54172414	54173367	ENSMUSG00000049573	ENSMUSG00000049573	ENSMUSG00000049573	10	128724574	128725512	186	211
9151	12	54011752	54012687	ENSMUSG00000049217	ENSMUSG00000049217	ENSMUSG00000049217	10	128875643	128876578	186	211
9152	12	54045162	54046100	ENSMUSG00000044025	ENSMUSG00000044025	ENSMUSG00000044025	10	128903835	128904803	186	211
9153	12	54132265	54133203	ENSMUSG00000047626	ENSMUSG00000047626	ENSMUSG00000047626	10	128929178	128930116	186	211
9154	12	54316943	54317884	ENSMUSG00000052012	ENSMUSG00000052012	ENSMUSG00000052012	10	129010496	129011428	186	211
9155	12	54080580	54081518	ENSMUSG00000049894	ENSMUSG00000049894	ENSMUSG00000049894	10	129170447	129171385	186	211
9156	12	53927339	53928277	ENSMUSG00000044897	ENSMUSG00000044897	ENSMUSG00000044897	10	129436577	129437509	186	211
9157	12	53699889	53707486	ENSMUSG00000048015	ENSMUSG00000048015	ENSMUSG00000048015	10	129671102	129683189	186	211
9158	12	53628069	53653981	ENSMUSG00000034833	ENSMUSG00000034833	ENSMUSG00000034833	10	129750137	129765586	186	211
9159	22	30222261	30344533	ENSMUSG00000023764	ENSMUSG00000023764	ENSMUSG00000023764	11	3032030	3087415	187	212
9160	22	30125609	30160438	ENSMUSG00000020457	ENSMUSG00000020457	ENSMUSG00000020457	11	3149925	3166396	187	212
9161	22	30051790	30072249	ENSMUSG00000020453	ENSMUSG00000020453	ENSMUSG00000020453	11	3190460	3208539	187	212
9162	22	30007599	30018474	ENSMUSG00000034614	ENSMUSG00000034614	ENSMUSG00000034614	11	3230404	3242941	187	212

9163	22	29974348	30003624	ENSMUSG00000020451	ENSMUSG00000020451	ENSMUSG00000020451	11	3244259	3309177	187	212
9164	22	29886179	29932998	ENSMUSG00000020448	ENSMUSG00000020448	ENSMUSG00000020448	11	3315986	3352328	187	212
9165	22	29860795	29866593	ENSMUSG00000034579	ENSMUSG00000034579	ENSMUSG00000034579	11	3388355	3394166	187	212
9166	22	29848961	29860683	ENSMUSG00000034570	ENSMUSG00000034570	ENSMUSG00000034570	11	3394386	3404824	187	212
9167	22	29830765	29833491	ENSMUSG00000075702	ENSMUSG00000075702	ENSMUSG00000075702	11	3414948	3417163	187	212
9168	22	29807305	29830609	ENSMUSG00000020439	ENSMUSG00000020439	ENSMUSG00000020439	11	3417530	3439295	187	212
9169	22	29420793	29632326	ENSMUSG00000020435	ENSMUSG00000020435	ENSMUSG00000020435	11	3603734	3763906	187	212
9170	22	29388039	29393872	ENSMUSG00000047205	ENSMUSG00000047205	ENSMUSG00000047205	11	3795284	3801299	187	212
9171	22	29361799	29373879	ENSMUSG00000048807	ENSMUSG00000048807	ENSMUSG00000048807	11	3807025	3814667	187	212
9172	22	29333161	29353047	ENSMUSG00000020432	ENSMUSG00000020432	ENSMUSG00000020432	11	3817193	3831586	187	212
9173	22	29280624	29286748	ENSMUSG00000049721	ENSMUSG00000049721	ENSMUSG00000049721	11	3883639	3899329	187	212
9174	22	29216094	29231698	ENSMUSG00000019368	ENSMUSG00000019368	ENSMUSG00000019368	11	3931785	3948026	187	212
9175	22	29185218	29198034	ENSMUSG00000054986	ENSMUSG00000054986	ENSMUSG00000054986	11	3964955	3977041	187	212
9176	22	29151652	29155040	ENSMUSG00000004748	ENSMUSG00000004748	ENSMUSG00000004748	11	3991484	3995434	187	212
9177	22	29123031	29151276	ENSMUSG00000003585	ENSMUSG00000003585	ENSMUSG00000003585	11	3997034	4018732	187	212
9178	22	29104801	29113302	ENSMUSG00000003581	ENSMUSG00000003581	ENSMUSG00000003581	11	4035205	4041175	187	212
9179	22	29083036	29102816	ENSMUSG00000051427	ENSMUSG00000051427	ENSMUSG00000051427	11	4042003	4060277	187	212
9180	22	29057979	29082913	ENSMUSG00000002129	ENSMUSG00000002129	ENSMUSG00000002129	11	4060371	4080632	187	212
9181	22	29017980	29052921	ENSMUSG00000034412	ENSMUSG00000034412	ENSMUSG00000034412	11	4086792	4117403	187	212
9182	22	29011108	29015522	ENSMUSG00000020424	ENSMUSG00000020424	ENSMUSG00000020424	11	4118254	4122412	187	212
9183	22	28988821	28992840	ENSMUSG00000058755	ENSMUSG00000058755	ENSMUSG00000058755	11	4136423	4141029	187	212
9184	22	28966441	28972748	ENSMUSG00000034394	ENSMUSG00000034394	ENSMUSG00000034394	11	4157571	4172517	187	212
9185	22	28806453	28903058	ENSMUSG00000020419	ENSMUSG00000020419	ENSMUSG00000020419	11	4245817	4341108	187	212
9186	22	28609199	28753869	ENSMUSG00000034354	ENSMUSG00000034354	ENSMUSG00000034354	11	4382771	4428409	187	212
9187	22	28514603	28564254	ENSMUSG00000020412	ENSMUSG00000020412	ENSMUSG00000020412	11	4537796	4583388	187	212
9188	22	28456948	28492969	ENSMUSG00000009076	ENSMUSG00000009076	ENSMUSG00000009076	11	4604681	4637669	187	212
9189	22	28446344	28457818	ENSMUSG00000009075	ENSMUSG00000009075	ENSMUSG00000009075	11	4638824	4646781	187	212
9190	22	28329565	28409903	ENSMUSG00000009073	ENSMUSG00000009073	ENSMUSG00000009073	11	4668058	4749553	187	212
9191	22	28280800	28307244	ENSMUSG00000034285	ENSMUSG00000034285	ENSMUSG00000034285	11	4782972	4793956	187	212
9192	22	28231868	28279703	ENSMUSG00000034274	ENSMUSG00000034274	ENSMUSG00000034274	11	4795346	4828868	187	212
9193	22	28196907	28217275	ENSMUSG00000020396	ENSMUSG00000020396	ENSMUSG00000020396	11	4839348	4847937	187	212
9194	22	28053669	28114569	ENSMUSG00000009090	ENSMUSG00000009090	ENSMUSG00000009090	11	4847524	4941795	187	212
9195	22	28039290	28041748	ENSMUSG00000034209	ENSMUSG00000034209	ENSMUSG00000034209	11	4958131	4960388	187	212
9196	22	28034096	28038486	ENSMUSG00000034201	ENSMUSG00000034201	ENSMUSG00000034201	11	4960794	4965330	187	212
9197	22	27985887	27993967	ENSMUSG00000034175	ENSMUSG00000034175	ENSMUSG00000034175	11	4999441	5006094	187	212
9198	22	27931912	27985547	ENSMUSG00000034164	ENSMUSG00000034164	ENSMUSG00000034164	11	5006269	5052225	187	212
9199	22	27799106	27894321	ENSMUSG00000020393	ENSMUSG00000020393	ENSMUSG00000020393	11	5091555	5161613	187	212
9200	22	27609890	27783475	ENSMUSG00000041961	ENSMUSG00000041961	ENSMUSG00000041961	11	5179727	5344681	187	212
9201	22	27498702	27515282	ENSMUSG00000020482	ENSMUSG00000020482	ENSMUSG00000020482	11	5428893	5442190	187	212
9202	7	43872685	43875633	ENSMUSG00000020477	ENSMUSG00000020477	ENSMUSG00000020477	11	5603986	5607688	188	213
9203	7	43882039	43912750	ENSMUSG00000049680	ENSMUSG00000049680	ENSMUSG00000049680	11	5614188	5641113	188	213
9204	7	44050835	44067761	ENSMUSG00000020476	ENSMUSG00000020476	ENSMUSG00000020476	11	5688533	5700965	188	213
9205	7	44068855	44071709	ENSMUSG00000020475	ENSMUSG00000020475	ENSMUSG00000020475	11	5701647	5703736	188	213
9206	7	44078374	44088607	ENSMUSG00000020474	ENSMUSG00000020474	ENSMUSG00000020474	11	5727864	5737799	188	213
9207	7	44110485	44120683	ENSMUSG00000020473	ENSMUSG00000020473	ENSMUSG00000020473	11	5761877	5772090	188	213
9208	7	44120812	44128238	ENSMUSG00000020471	ENSMUSG00000020471	ENSMUSG00000020471	11	5772183	5778295	188	213
9209	7	44144990	44147441	ENSMUSG00000020469	ENSMUSG00000020469	ENSMUSG00000020469	11	5796640	5798785	188	213
9210	7	44150395	44165412	ENSMUSG00000041798	ENSMUSG00000041798	ENSMUSG00000041798	11	5800826	5850084	188	213
9211	7	44225422	44331749	ENSMUSG00000057897	ENSMUSG00000057897	ENSMUSG00000057897	11	5869675	5965751	188	213
9212	7	44389716	44496808	ENSMUSG00000053838	ENSMUSG00000053838	ENSMUSG00000053838	11	6005709	6100165	188	213
9213	7	44519571	44547383	ENSMUSG00000020447	ENSMUSG00000020447	ENSMUSG00000020447	11	6111016	6130146	188	213
9214	7	44571929	44581175	ENSMUSG00000004393	ENSMUSG00000004393	ENSMUSG00000004393	11	6157548	6167732	188	213
9215	7	44584021	44588342	ENSMUSG00000004394	ENSMUSG00000004394	ENSMUSG00000004394	11	6170717	6174850	188	213
9216	7	44612696	44715193	ENSMUSG00000020456	ENSMUSG00000020456	ENSMUSG00000020456	11	6191671	6256564	188	213
9217	7	44762303	44776002	ENSMUSG00000041164	ENSMUSG00000041164	ENSMUSG00000041164	11	6289367	6306165	188	213
9218	7	44839998	44854234	ENSMUSG00000041126	ENSMUSG00000041126	ENSMUSG00000041126	11	6328147	6339462	188	213
9219	7	44890534	44891472	ENSMUSG00000049647	ENSMUSG00000049647	ENSMUSG00000049647	11	6374915	6375889	188	213
9220	7	44968786	44985203	ENSMUSG00000020437	ENSMUSG00000020437	ENSMUSG00000020437	11	6406555	6420961	188	213
9221	7	45033150	45082575	ENSMUSG00000000378	ENSMUSG00000000378	ENSMUSG00000000378	11	6446959	6496747	188	213
9222	7	45086562	45092185	ENSMUSG00000041073	ENSMUSG00000041073	ENSMUSG00000041073	11	6497827	6506057	188	213
9223	7	45106225	45117842	ENSMUSG00000000384	ENSMUSG00000000384	ENSMUSG00000000384	11	6516616	6524299	188	213
9224	7	45163924	45190372	ENSMUSG00000041046	ENSMUSG00000041046	ENSMUSG00000041046	11	6558536	6577478	188	213
9225	7	45580646	45729237	ENSMUSG00000020431	ENSMUSG00000020431	ENSMUSG00000020431	11	6963492	7078509	188	213
9226	7	45894484	45899791	ENSMUSG00000020429	ENSMUSG00000020429	ENSMUSG00000020429	11	7097782	7102541	188	213
9227	7	45920944	45927264	ENSMUSG00000020427	ENSMUSG00000020427	ENSMUSG00000020427	11	7106098	7113909	188	213
9228	7	47281279	47588216	ENSMUSG00000020422	ENSMUSG00000020422	ENSMUSG00000020422	11	8334497	8564531	188	213
9229	7	47780815	47954562	ENSMUSG00000046634	ENSMUSG00000046634	ENSMUSG00000046634	11	8726778	8873268	188	213
9230	7	47969508	47985651	ENSMUSG00000020413	ENSMUSG00000020413	ENSMUSG00000020413	11	8893141	8911148	188	213
9231	7	47993273	48035241	ENSMUSG00000040985	ENSMUSG00000040985	ENSMUSG00000040985	11	8916057	8938320	188	213
9232	7	48041642	48067416	ENSMUSG00000040978	ENSMUSG00000040978	ENSMUSG00000040978	11	8948595	8969357	188	213
9233	7	48094766	48114839	ENSMUSG00000020407	ENSMUSG00000020407	ENSMUSG00000020407	11	9018106	9036167	188	213
9234	7	48208389	48657637	ENSMUSG00000004668	ENSMUSG00000004668	ENSMUSG00000004668	11	9091969	9583635	188	213
9235	7	49783798	49922684	ENSMUSG00000050830	ENSMUSG00000050830	ENSMUSG00000050830	11	11014747	11167726	188	213
9236	7	49947585	50103372	ENSMUSG00000020193	ENSMUSG00000020193	ENSMUSG00000020193	11	11180043	11362367	188	213
9237	7	50106348	50106788	ENSMUSG00000047886	ENSMUSG00000047886	ENSMUSG00000047886	11	11364008	11366924	188	213
9238	7	50146240	50169311	ENSMUSG00000020191	ENSMUSG00000020191	ENSMUSG00000020191	11	11390224	11415103	188	213
9239	7	50314924	50438053	ENSMUSG00000018654	ENSMUSG00000018654	ENSMUSG00000018654	11	11586216	11672929	188	213
9240	7	50480455	50482479	ENSMUSG00000035455	ENSMUSG00000035455	ENSMUSG00000035455	11	11700291	11708935	188	213
9241	7	50493657	50600537	ENSMUSG00000020182	ENSMUSG00000020182	ENSMUSG00000020182	11	11714107	11798103	188	213
9242	7	50625260	50828652	ENSMUSG00000020176	ENSMUSG00000020176	ENSMUSG00000020176	11	11830511	11937390	188	213
9243	7	51051404	51351990	ENSMUSG00000020173	ENSMUSG00000020173	ENSMUSG00000020173	11	12149638	12286768	188	213
9244	7	54577635	54587714	ENSMUSG00000048834	ENSMUSG00000048834	ENSMUSG00000048834	11	16157775	16268377	188	213
9245	2	68543011	68547894	ENSMUSG00000044966	ENSMUSG00000044966	ENSMUSG00000044966	11	16851413	16854775	189	214
9246	2	68445920	68478088	ENSMUSG00000020120	ENSMUSG00000020120	ENSMUSG00000020120	11	16871209	16908705	189	214
9247	2	68373647	68400347	ENSMUSG00000044629	ENSMUSG00000044629	ENSMUSG00000044629	11	16951587	16955009	189	214
9248	2	68238509	68256593	ENSMUSG00000020116	ENSMUSG00000020116	ENSMUSG00000020116	11	17103205	17111576	189	214
9249	2	68122844	68143634	ENSMUSG00000000581	ENSMUSG00000000581	ENSMUSG00000000581	11	17157621	17169179	189	214
9250	2	66516036	66653394	ENSMUSG00000020160	ENSMUSG00000020160	ENSMUSG00000020160	11	18781632	18918717	189	214
9251	2	65391492	65512815	ENSMUSG00000045671	ENSMUSG00000045671	ENSMUSG00000045671	11	19824445	19922600	189	214
9252	2	65308406	65351889	ENSMUSG00000020152	ENSMUSG00000020152	ENSMUSG00000020152	11	19962307	20012916	189	214
9253	2	65136999	65167618	ENSMUSG00000044066	ENSMUSG00000044066	ENSMUSG00000044066	11	20128509	20149420	189	214
9254	2	65070098	65104501	ENSMUSG00000020142	ENSMUSG00000020142	ENSMUSG00000020142	11	20202208	20232674	189	214
9255	2	64712260	64734550	ENSMUSG00000049800	ENSMUSG00000049800	ENSMUSG00000049800	11	20443256	20553026	189	214
9256	2	64605165	64672753	ENSMUSG00000049659	ENSMUSG00000049659	ENSMUSG00000049659	11	20585095	20641502	189	214
9257	2	64173505	64225062	ENSMUSG00000020134	ENSMUSG00000020134	ENSMUSG00000020134	11	20991327	21049428	189	214
9258	2	63973171	64099718	ENSMUSG00000020128	ENSMUSG00000020128	ENSMUSG00000020128	11	21139284	21221139	189	214
9259	2	63922518	63972196	ENSMUSG00000001891	ENSMUSG00000001891	ENSMUSG00000001891	11	21221141	21270480	189	214
9260	2	63669626	63687832	ENSMUSG00000020321	ENSMUSG00000020321	ENSMUSG00000020321	11	21456790	21472196	189	214
9261	2	63202041	63669156	ENSMUSG00000020319	ENSMUSG00000020319	ENSMUSG00000020319	11	21472284	21798689	189	214
9262	2	63131469	63137807	ENSMUSG00000005917	ENSMUSG00000005917	ENSMUSG00000005917	11	21894767	21901654	189	214
9263	2	62786625	63127123	ENSMUSG00000042302	ENSMUSG00000042302	ENSMUSG00000042302	11	21905831	22186798	189	214
9264	2	62581848	62587108	ENSMUSG00000049904	ENSMUSG00000049904	ENSMUSG00000049904	11	22412286	22419234	189	214
9265	2	62276766	62305368	ENSMUSG00000051650	ENSMUSG00000051650	ENSMUSG00000051650	11	22734734	22759688	189	214
9266	2	61986292	62216698	ENSMUSG00000051355	ENSMUSG00000051355	ENSMUSG00000051355	11	22799764	22878996	189	214
9267	2	61948769	61969295	ENSMUSG00000007739	ENSMUSG00000007739	ENSMUSG00000007739	11	22890593	22903336	189	214
9268	2	61905494	61934692	ENSMUSG00000049811	ENSMUSG00000049811	ENSMUSG00000049811	11	22913387	22923731	189	214
9269	2	61559459	61614536	ENSMUSG00000020290	ENSMUSG00000020290	ENSMUSG00000020290	11	23156041	23197597	189	214
9270	2	61268190	61486444	ENSMUSG00000056342	ENSMUSG00000056342	ENSMUSG00000056342	11	23243320	23388864	189	214
9271	2	61258178	61267419	ENSMUSG00000020288	ENSMUSG00000020288	ENSMUSG00000020288	11	23387882	23397946	189	214
9272	2	61150965	61214871	ENSMUSG00000042208	ENSMUSG00000042208	ENSMUSG00000042208	11	23473776	23529033	189	214
9273	2	61098374	61129895	ENSMUSG00000020283	ENSMUSG00000020283	ENSMUSG00000020283	11	23546479	23565935	189	214
9274	2	61022608	61098712	ENSMUSG00000020280	ENSMUSG00000020280	ENSMUSG00000020280	11	23565674	23632876	189	214
9275	2	60962254	61003174	ENSMUSG00000020275	ENSMUSG00000020275	ENSMUSG00000020275	11	23641514	23670970	189	214
9276	2	60836887	60879600	ENSMUSG00000020273	ENSMUSG00000020273	ENSMUSG00000020273	11	23762646	23795253	189	214
9277	2	60537838	60634121	ENSMUSG00000000861	ENSMUSG00000000861	ENSMUSG00000000861	11	23978117	24073194	189	214
9278	2	58239884	58322018	ENSMUSG00000004018	ENSMUSG00000004018	ENSMUSG00000004018	11	26287084	26371876	189	214
9279	2	58127224	58240505	ENSMUSG00000064090	ENSMUSG00000064090	ENSMUSG00000064090	11	26371404	26493611	189	214
9280	2	56264762	56466812	ENSMUSG00000032878	ENSMUSG00000032878	ENSMUSG00000032878	11	28288815	28484296	189	214
9281	2	55947243	56004426	ENSMUSG00000020467	ENSMUSG00000020467	ENSMUSG00000020467	11	28753204	28826739	189	214
9282	2	55715971	55774463	ENSMUSG00000020464	ENSMUSG00000020464	ENSMUSG00000020464	11	29030751	29061828	189	214
9283	2	55629019	55698227	ENSMUSG00000020463	ENSMUSG00000020463	ENSMUSG00000020463	11	29072967	29119680	189	214
9284	2	55600244	55625718	ENSMUSG00000020462	ENSMUSG00000020462	ENSMUSG00000020462	11	29121547	29147306	189	214
9285	2	55368482	55500467	ENSMUSG00000032740	ENSMUSG00000032740	ENSMUSG00000032740	11	29273775	29410812	189	214
9286	2	55362991	55365065	ENSMUSG00000032673	ENSMUSG00000032673	ENSMUSG00000032673	11	29412883	29415031	189	214
9287	2	55317288	55344498	ENSMUSG00000020459	ENSMUSG00000020459	ENSMUSG00000020459	11	29426465	29445279	189	214
9288	2	55256290	55312953	ENSMUSG00000020461	ENSMUSG00000020461	ENSMUSG00000020461	11	29447950	29478350	189	214
9289	2	55052833	55131238	ENSMUSG00000020458	ENSMUSG00000020458	ENSMUSG00000020458	11	29593189	29642026	189	214
9290	2	54537143	54749366	ENSMUSG00000020315	ENSMUSG00000020315	ENSMUSG00000020315	11	29999722	30119726	189	214
9291	2	54411612	54441365	ENSMUSG00000040919	ENSMUSG00000040919	ENSMUSG00000040919	11	30326004	30371829	189	214
9292	2	54195914	54385777	ENSMUSG00000060923	ENSMUSG00000060923	ENSMUSG00000060923	11	30405992	30549396	189	214
9293	2	53945075	54051354	ENSMUSG00000040850	ENSMUSG00000040850	ENSMUSG00000040850	11	30671983	30779734	189	214
9294	2	53933775	53935397	ENSMUSG00000043999	ENSMUSG00000043999	ENSMUSG00000043999	11	30791097	30792719	189	214
9295	2	53867721	53899437	ENSMUSG00000020311	ENSMUSG00000020311	ENSMUSG00000020311	11	30831419	30854123	189	214
9296	2	53750622	53867583	ENSMUSG00000020305	ENSMUSG00000020305	ENSMUSG00000020305	11	30854463	31001470	189	214
9297	2	53848456	53855835	ENSMUSG00000020309	ENSMUSG00000020309	ENSMUSG00000020309	11	30876724	30886334	189	214
9298	5	172674332	172689112	ENSMUSG00000020303	ENSMUSG00000020303	ENSMUSG00000020303	11	31259441	31270061	190	215
9299	5	172967155	172976262	ENSMUSG00000044502	ENSMUSG00000044502	ENSMUSG00000044502	11	31565145	31571862	190	215
9300	5	173248795	173319916	ENSMUSG00000020300	ENSMUSG00000020300	ENSMUSG00000020300	11	31772211	31831577	190	215
9301	5	173348779	173365748	ENSMUSG00000020299	ENSMUSG00000020299	ENSMUSG00000020299	11	31865592	31879651	190	215
9302	5	173405330	173468785	ENSMUSG00000020297	ENSMUSG00000020297	ENSMUSG00000020297	11	31900428	31959200	190	215
9303	16	43010	47443	ENSMUSG00000040767	ENSMUSG00000040767	ENSMUSG00000040767	11	32105415	32108984	191	216
9304	16	48059	62568	ENSMUSG00000020282	ENSMUSG00000020282	ENSMUSG00000020282	11	32109585	32122300	191	216
9305	16	68169	75839	ENSMUSG00000020287	ENSMUSG00000020287	ENSMUSG00000020287	11	32126511	32132702	191	216
9306	16	74273	128859	ENSMUSG00000020289	ENSMUSG00000020289	ENSMUSG00000020289	11	32132705	32167615	191	216
9307	5	171693108	171814132	ENSMUSG00000040711	ENSMUSG00000040711	ENSMUSG00000040711	11	32247811	32328183	192	217
9308	5	171571040	171643286	ENSMUSG00000044949	ENSMUSG00000044949	ENSMUSG00000044949	11	32355372	32418709	192	217
9309	5	171403230	171547865	ENSMUSG00000020272	ENSMUSG00000020272	ENSMUSG00000020272	11	32433305	32524587	192	217
9310	5	171221161	171366482	ENSMUSG00000020271	ENSMUSG00000020271	ENSMUSG00000020271	11	32542875	32646816	192	217
9311	5	171145481	171150598	ENSMUSG00000020270	ENSMUSG00000020270	ENSMUSG00000020270	11	32720376	32724573	192	217
9312	5	170779272	170816767	ENSMUSG00000057967	ENSMUSG00000057967	ENSMUSG00000057967	11	33017430	33047400	192	217
9313	5	170668893	170671742	ENSMUSG00000040610	ENSMUSG00000040610	ENSMUSG00000040610	11	33101279	33103459	192	217
9314	5	170143343	170173626	ENSMUSG00000020159	ENSMUSG00000020159	ENSMUSG00000020159	11	33450781	33478957	192	217
9315	5	169864155	170095388	ENSMUSG00000053519	ENSMUSG00000053519	ENSMUSG00000053519	11	33529790	33743585	192	217
9316	5	169737745	169749216	ENSMUSG00000020155	ENSMUSG00000020155	ENSMUSG00000020155	11	33863286	33871275	192	217
9317	5	169607667	169657400	ENSMUSG00000002699	ENSMUSG00000002699	ENSMUSG00000002699	11	33947144	33992281	192	217
9318	5	169465495	169469291	ENSMUSG00000047861	ENSMUSG00000047861	ENSMUSG00000047861	11	34104341	34108089	192	217
9319	5	168996871	169442959	ENSMUSG00000020143	ENSMUSG00000020143	ENSMUSG00000020143	11	34127668	34627246	192	217
9320	5	169223855	169340322	ENSMUSG00000069911	ENSMUSG00000069911	ENSMUSG00000069911	11	34214822	34322640	192	217
9321	5	168026037	168660291	ENSMUSG00000056427	ENSMUSG00000056427	ENSMUSG00000056427	11	34964938	35551565	192	217
9322	5	167915208	167939166	ENSMUSG00000018846	ENSMUSG00000018846	ENSMUSG00000018846	11	35612898	35629937	192	217
9323	5	167889088	167890086	ENSMUSG00000051062	ENSMUSG00000051062	ENSMUSG00000051062	11	35640913	35641857	192	217
9324	5	167846041	167878885	ENSMUSG00000018848	ENSMUSG00000018848	ENSMUSG00000018848	11	35651806	35677929	192	217
9325	5	167651670	167829334	ENSMUSG00000018849	ENSMUSG00000018849	ENSMUSG00000018849	11	35681827	35823890	192	217
9326	5	166644421	167622390	ENSMUSG00000049336	ENSMUSG00000049336	ENSMUSG00000049336	11	35851458	36787589	192	217
9327	5	162865163	162878904	ENSMUSG00000042032	ENSMUSG00000042032	ENSMUSG00000042032	11	40522739	40536068	192	217
9328	5	162820241	162851523	ENSMUSG00000020330	ENSMUSG00000020330	ENSMUSG00000020330	11	40546196	40576809	192	217
9329	5	162806112	162819721	ENSMUSG00000020328	ENSMUSG00000020328	ENSMUSG00000020328	11	40577091	40583467	192	217
9330	5	161427295	161515122	ENSMUSG00000020436	ENSMUSG00000020436	ENSMUSG00000020436	11	41755280	41844101	192	217
9331	5	161207772	161257006	ENSMUSG00000010803	ENSMUSG00000010803	ENSMUSG00000010803	11	41974903	42026353	192	217
9332	5	161045548	161061689	ENSMUSG00000020428	ENSMUSG00000020428	ENSMUSG00000020428	11	42149861	42164431	192	217
9333	5	160653449	160907708	ENSMUSG00000007653	ENSMUSG00000007653	ENSMUSG00000007653	11	42263180	42472451	192	217
9334	5	159922718	160211624	ENSMUSG00000055415	ENSMUSG00000055415	ENSMUSG00000055415	11	42995038	43103329	192	217
9335	5	159762737	159778672	ENSMUSG00000020409	ENSMUSG00000020409	ENSMUSG00000020409	11	43277154	43291404	192	217
9336	5	159707365	159730207	ENSMUSG00000046491	ENSMUSG00000046491	ENSMUSG00000046491	11	43317729	43334932	192	217
9337	5	159611238	159640351	ENSMUSG00000044707	ENSMUSG00000044707	ENSMUSG00000044707	11	43372820	43429138	192	217
9338	5	159573319	159598307	ENSMUSG00000020405	ENSMUSG00000020405	ENSMUSG00000020405	11	43439535	43444934	192	217
9339	5	159424152	159479008	ENSMUSG00000044950	ENSMUSG00000044950	ENSMUSG00000044950	11	43525429	43550614	192	217
9340	5	159368708	159425096	ENSMUSG00000041278	ENSMUSG00000041278	ENSMUSG00000041278	11	43572935	43591408	192	217
9341	5	159276318	159332595	ENSMUSG00000050541	ENSMUSG00000050541	ENSMUSG00000050541	11	43618029	43679755	192	217
9342	5	158674369	158690059	ENSMUSG00000004296	ENSMUSG00000004296	ENSMUSG00000004296	11	44243486	44257456	192	217
9343	5	158058697	158459064	ENSMUSG00000057098	ENSMUSG00000057098	ENSMUSG00000057098	11	44461602	44848171	192	217
9344	5	157146500	157218731	ENSMUSG00000006169	ENSMUSG00000006169	ENSMUSG00000006169	11	45727131	45754046	192	217
9345	5	157103333	157116324	ENSMUSG00000044847	ENSMUSG00000044847	ENSMUSG00000044847	11	45774503	45788358	192	217
9346	5	157091024	157100488	ENSMUSG00000011254	ENSMUSG00000011254	ENSMUSG00000011254	11	45791286	45798906	192	217
9347	5	156985265	157012006	ENSMUSG00000040489	ENSMUSG00000040489	ENSMUSG00000040489	11	45823733	45861417	192	217
9348	5	156836890	156935346	ENSMUSG00000011256	ENSMUSG00000011256	ENSMUSG00000011256	11	45899475	45990766	192	217
9349	5	156822307	156834302	ENSMUSG00000020411	ENSMUSG00000020411	ENSMUSG00000020411	11	45991578	46009782	192	217
9350	5	156625669	156755184	ENSMUSG00000020340	ENSMUSG00000020340	ENSMUSG00000020340	11	46037281	46155773	192	217
9351	5	156701619	156705257	ENSMUSG00000048721	ENSMUSG00000048721	ENSMUSG00000048721	11	46078980	46083294	192	217
9352	5	156540432	156614687	ENSMUSG00000020395	ENSMUSG00000020395	ENSMUSG00000020395	11	46168576	46232916	192	217
9353	5	156521929	156525856	ENSMUSG00000020401	ENSMUSG00000020401	ENSMUSG00000020401	11	46248153	46251405	192	217
9354	5	156494937	156502453	ENSMUSG00000020397	ENSMUSG00000020397	ENSMUSG00000020397	11	46280397	46285031	192	217
9355	5	156445421	156468716	ENSMUSG00000020399	ENSMUSG00000020399	ENSMUSG00000020399	11	46298358	46324678	192	217
9356	5	156278949	156322844	ENSMUSG00000055546	ENSMUSG00000055546	ENSMUSG00000055546	11	46654223	46687756	192	217
9357	5	155686334	156125623	ENSMUSG00000020354	ENSMUSG00000020354	ENSMUSG00000020354	11	46822686	47198796	192	217

9358	5	180596583	180603406	ENSMUSG00000020372	ENSMUSG00000020372	ENSMUSG00000020372	11	48643783	48649857	193	218		
9359	5	180582912	180595414	ENSMUSG00000040365	ENSMUSG00000040365	ENSMUSG00000040365	11	48649827	48660776	193	218		
9360	5	180553531	180564783	ENSMUSG00000040350	ENSMUSG00000040350	ENSMUSG00000040350	11	48680950	48693590	193	218		
9361	5	180514549	180515496	ENSMUSG00000047511	ENSMUSG00000047511	ENSMUSG00000047511	11	48956200	48957180	193	218		
9362	5	180399854	180421121	ENSMUSG00000040283	ENSMUSG00000040283	ENSMUSG00000040283	11	49012477	49030468	193	218		
9363	5	180208335	180211076	ENSMUSG00000046311	ENSMUSG00000046311	ENSMUSG00000046311	11	49057700	49062205	193	218		
9364	5	180150154	180175147	ENSMUSG00000020346	ENSMUSG00000020346	ENSMUSG00000020346	11	49087669	49106453	193	218		
9365	5	179962143	180009171	ENSMUSG00000020357	ENSMUSG00000020357	ENSMUSG00000020357	11	49453150	49495652	193	218		
9366	5	179949712	179951146	ENSMUSG00000064057	ENSMUSG00000064057	ENSMUSG00000064057	11	49507033	49508541	193	218		
9367	5	179854023	179937959	ENSMUSG00000020362	ENSMUSG00000020362	ENSMUSG00000020362	11	49518121	49556133	193	218		
9368	5	179660296	179712931	ENSMUSG00000020363	ENSMUSG00000020363	ENSMUSG00000020363	11	49637610	49682036	193	218		
9369	5	179595388	179640218	ENSMUSG00000020366	ENSMUSG00000020366	ENSMUSG00000020366	11	49690177	49729521	193	218		
9370	5	179460402	179568751	ENSMUSG00000020374	ENSMUSG00000020374	ENSMUSG00000020374	11	49745258	49823648	193	218		
9371	5	179315111	179431715	ENSMUSG00000020376	ENSMUSG00000020376	ENSMUSG00000020376	11	49868771	49948176	193	218		
9372	5	179221679	179267464	ENSMUSG00000036644	ENSMUSG00000036644	ENSMUSG00000036644	11	49974843	50015448	193	218		
9373	5	179196873	179218446	ENSMUSG00000020381	ENSMUSG00000020381	ENSMUSG00000020381	11	50018272	50044730	193	218		
9374	5	179180503	179196199	ENSMUSG00000015837	ENSMUSG00000015837	ENSMUSG00000015837	11	50044213	50054213	193	218		
9375	5	179157216	179162481	ENSMUSG00000036620	ENSMUSG00000036620	ENSMUSG00000036620	11	50069056	50078073	193	218		
9376	5	179092457	179136887	ENSMUSG00000050567	ENSMUSG00000050567	ENSMUSG00000050567	11	50099060	50135739	193	218		
9377	5	179058536	179091243	ENSMUSG00000020368	ENSMUSG00000020368	ENSMUSG00000020368	11	50137886	50169014	193	218		
9378	5	179038190	179038849	ENSMUSG00000050087	ENSMUSG00000050087	ENSMUSG00000050087	11	50201184	50203122	193	218		
9379	5	178973795	178984276	ENSMUSG00000007850	ENSMUSG00000007850	ENSMUSG00000007850	11	50221174	50229318	193	218		
9380	5	178910177	178969618	ENSMUSG00000020375	ENSMUSG00000020375	ENSMUSG00000020375	11	50232726	50274534	193	218		
9381	5	178473474	178704935	ENSMUSG00000036545	ENSMUSG00000036545	ENSMUSG00000036545	11	50445508	50647478	193	218		
9382	5	178420213	178440295	ENSMUSG00000044807	ENSMUSG00000044807	ENSMUSG00000044807	11	50654509	50669881	193	218		
9383	5	178383412	178394647	ENSMUSG00000044296	ENSMUSG00000044296	ENSMUSG00000044296	11	50675458	50684975	193	218		
9384	5	178300830	178326040	ENSMUSG00000048728	ENSMUSG00000048728	ENSMUSG00000048728	11	50716143	50730924	193	218		
9385	5	178219560	178244614	ENSMUSG00000020335	ENSMUSG00000020335	ENSMUSG00000020335	11	50765246	50774356	193	218		
9386	5	177351842	177355849	ENSMUSG00000044542	ENSMUSG00000044542	ENSMUSG00000044542	11	50794229	50797055	193			
9387	5	178071137	178090309	ENSMUSG00000020364	ENSMUSG00000020364	ENSMUSG00000020364	11	50903545	50914685	193	219	here	This inversion was ignored by our tool.
9388	5	177962272	177986660	ENSMUSG00000020385	ENSMUSG00000020385	ENSMUSG00000020385	11	51110594	51124840	193	219	Inversion ignored by ST	1 -> 2
9389	5	177598748	177949804	ENSMUSG00000063564	ENSMUSG00000063564	ENSMUSG00000063564	11	51133166	51427341	193	219		
9390	5	177568191	177592157	ENSMUSG00000020359	ENSMUSG00000020359	ENSMUSG00000020359	11	51428226	51444581	193	219		
9391	5	177564114	177570787	ENSMUSG00000020358	ENSMUSG00000020358	ENSMUSG00000020358	11	51444831	51450258	193	219		
9392	5	177509074	177513567	ENSMUSG00000001056	ENSMUSG00000001056	ENSMUSG00000001056	11	51463196	51467137	193	219		
9393	5	177490603	177508147	ENSMUSG00000001054	ENSMUSG00000001054	ENSMUSG00000001054	11	51467096	51479319	193	219		
9394	5	177473162	177485713	ENSMUSG00000001053	ENSMUSG00000001053	ENSMUSG00000001053	11	51486512	51494496	193	219		
9395	5	134012710	134091398	ENSMUSG00000036391	ENSMUSG00000036391	ENSMUSG00000036391	11	51538243	51600008	194	220		
9396	5	133969443	133996386	ENSMUSG00000020386	ENSMUSG00000020386	ENSMUSG00000020386	11	51607110	51635348	194	220		
9397	5	133889272	133942906	ENSMUSG00000020387	ENSMUSG00000020387	ENSMUSG00000020387	11	51660318	51692474	194	220		
9398	5	133765704	133775488	ENSMUSG00000020392	ENSMUSG00000020392	ENSMUSG00000020392	11	51811084	51820757	194	220		
9399	5	133734769	133755698	ENSMUSG00000020390	ENSMUSG00000020390	ENSMUSG00000020390	11	51828985	51843810	194	220		
9400	5	133662010	133730664	ENSMUSG00000020389	ENSMUSG00000020389	ENSMUSG00000020389	11	51847745	51881588	194	220		
9401	5	133560047	133589849	ENSMUSG00000020349	ENSMUSG00000020349	ENSMUSG00000020349	11	51942247	51966172	194	220		
9402	5	133478301	133511818	ENSMUSG00000000782	ENSMUSG00000000782	ENSMUSG00000000782	11	52096027	52126602	194	220		
9403	5	133319101	133332377	ENSMUSG00000036275	ENSMUSG00000036275	ENSMUSG00000036275	11	52239851	52252146	194	220		
9404	5	132560051	132976122	ENSMUSG00000036264	ENSMUSG00000036264	ENSMUSG00000036264	11	52608129	53030770	194	220		
9405	5	132415561	132468608	ENSMUSG00000020361	ENSMUSG00000020361	ENSMUSG00000020361	11	53103239	53143853	194	220		
9406	5	132238970	132327225	ENSMUSG00000049470	ENSMUSG00000049470	ENSMUSG00000049470	11	53194256	53265263	194	220		
9407	5	132235913	132238103	ENSMUSG00000036216	ENSMUSG00000036216	ENSMUSG00000036216	11	53265606	53266552	194	220		
9408	5	132230241	132231277	ENSMUSG00000044894	ENSMUSG00000044894	ENSMUSG00000044894	11	53271345	53274237	194	220		
9409	5	132224772	132228824	ENSMUSG00000018238	ENSMUSG00000018238	ENSMUSG00000018238	11	53276791	53281323	194	220		
9410	5	132186387	132189882	ENSMUSG00000018387	ENSMUSG00000018387	ENSMUSG00000018387	11	53300672	53310924	194	220		
9411	5	132177178	132180388	ENSMUSG00000044352	ENSMUSG00000044352	ENSMUSG00000044352	11	53321684	53322688	194	220		
9412	5	132114416	132140966	ENSMUSG00000018398	ENSMUSG00000018398	ENSMUSG00000018398	11	53363433	53381342	194	220		
9413	5	132059221	132101163	ENSMUSG00000018395	ENSMUSG00000018395	ENSMUSG00000018395	11	53410826	53445390	194	220		
9414	5	132037272	132046267	ENSMUSG00000000869	ENSMUSG00000000869	ENSMUSG00000000869	11	53455891	53462067	194	220		
9415	5	132021764	132024701	ENSMUSG00000020383	ENSMUSG00000020383	ENSMUSG00000020383	11	53474747	53478125	194	220		
9416	5	131920529	132007651	ENSMUSG00000020380	ENSMUSG00000020380	ENSMUSG00000020380	11	53492942	53550742	194	220		
9417	5	131905035	131907113	ENSMUSG00000036117	ENSMUSG00000036117	ENSMUSG00000036117	11	53564217	53568526	194	220		
9418	5	131845200	131854389	ENSMUSG00000018899	ENSMUSG00000018899	ENSMUSG00000018899	11	53613918	53620744	194	220		
9419	5	131658035	131707798	ENSMUSG00000020334	ENSMUSG00000020334	ENSMUSG00000020334	11	53826549	53871513	194	220		
9420	5	131621263	131637046	ENSMUSG00000020388	ENSMUSG00000020388	ENSMUSG00000020388	11	53898355	53912391	194	220		
9421	5	131555430	131590850	ENSMUSG00000018906	ENSMUSG00000018906	ENSMUSG00000018906	11	53944939	53975071	194	220		
9422	5	131437382	131439758	ENSMUSG00000018916	ENSMUSG00000018916	ENSMUSG00000018916	11	54090687	54093065	194	220		
9423	5	131313565	131375254	ENSMUSG00000020333	ENSMUSG00000020333	ENSMUSG00000020333	11	54147627	54204962	194	220		
9424	5	130789500	130998828	ENSMUSG00000037533	ENSMUSG00000037533	ENSMUSG00000037533	11	54366270	54542708	194	220		
9425	5	130627692	130758257	ENSMUSG00000052298	ENSMUSG00000052298	ENSMUSG00000052298	11	54560880	54631059	194	220		
9426	5	130534535	130564905	ENSMUSG00000020268	ENSMUSG00000020268	ENSMUSG00000020268	11	54670291	54704339	194	220		
9427	5	150380112	150388741	ENSMUSG00000018339	ENSMUSG00000018339	ENSMUSG00000018339	11	54746389	54753081	195	221		
9428	5	150389733	150440762	ENSMUSG00000020400	ENSMUSG00000020400	ENSMUSG00000020400	11	54754208	54806335	195	221		
9429	5	150460461	150500006	ENSMUSG00000018340	ENSMUSG00000018340	ENSMUSG00000018340	11	54822378	54857356	195	221		
9430	5	150540807	150583847	ENSMUSG00000049588	ENSMUSG00000049588	ENSMUSG00000049588	11	54893161	54921512	195	221		
9431	5	150612837	150629000	ENSMUSG00000000594	ENSMUSG00000000594	ENSMUSG00000000594	11	54941534	54954321	195	221		
9432	5	150636516	150663520	ENSMUSG00000049491	ENSMUSG00000049491	ENSMUSG00000049491	11	54968246	54995129	195	221		
9433	5	150674736	150707312	ENSMUSG00000020264	ENSMUSG00000020264	ENSMUSG00000020264	11	55001893	55028491	195	221		
9434	5	150807356	150852132	ENSMUSG00000020261	ENSMUSG00000020261	ENSMUSG00000020261	11	55047793	55077329	195	221		
9435	5	150865319	150928685	ENSMUSG00000055333	ENSMUSG00000055333	ENSMUSG00000055333	11	55094033	55179971	195	221		
9436	5	151021212	151046710	ENSMUSG00000018593	ENSMUSG00000018593	ENSMUSG00000018593	11	55237924	55263289	195	221		
9437	5	151106079	151111539	ENSMUSG00000018585	ENSMUSG00000018585	ENSMUSG00000018585	11	55293924	55298385	195	221		
9438	5	151182361	151284596	ENSMUSG00000000263	ENSMUSG00000000263	ENSMUSG00000000263	11	55357661	55451621	195	221		
9439	5	151751303	151765017	ENSMUSG00000037393	ENSMUSG00000037393	ENSMUSG00000037393	11	55868413	55884410	195	221		
9440	5	153351519	153398656	ENSMUSG00000020523	ENSMUSG00000020523	ENSMUSG00000020523	11	57299195	57334810	195	221		
9441	5	153398752	153414783	ENSMUSG00000020522	ENSMUSG00000020522	ENSMUSG00000020522	11	57334860	57350012	195	221		
9442	5	153550488	153780003	ENSMUSG00000020520	ENSMUSG00000020520	ENSMUSG00000020520	11	57461637	57603696	195	221		
9443	5	153805766	153816059	ENSMUSG00000020519	ENSMUSG00000020519	ENSMUSG00000020519	11	57617832	57626409	195	221		
9444	5	153834726	153838017	ENSMUSG00000037335	ENSMUSG00000037335	ENSMUSG00000037335	11	57644908	57648342	195	221		
9445	5	154178247	154210406	ENSMUSG00000037290	ENSMUSG00000037290	ENSMUSG00000037290	11	57882247	57889132	195	221		
9446	5	154218299	154236544	ENSMUSG00000020515	ENSMUSG00000020515	ENSMUSG00000020515	11	57920348	57934789	195	221		
9447	5	154247169	154297941	ENSMUSG00000037275	ENSMUSG00000037275	ENSMUSG00000037275	11	57937485	57984734	195	221		
9448	5	154300856	154326721	ENSMUSG00000020514	ENSMUSG00000020514	ENSMUSG00000020514	11	57987856	57995760	195	221		
9449	1	247110828	247119894	ENSMUSG00000037243	ENSMUSG00000037243	ENSMUSG00000037243	11	58123298	58130805	196	222		
9450	1	247099153	247110336	ENSMUSG00000049755	ENSMUSG00000049755	ENSMUSG00000049755	11	58131326	58139507	196	222		
9451	1	247071273	247087455	ENSMUSG00000013646	ENSMUSG00000013646	ENSMUSG00000013646	11	58146945	58163920	196	222		
9452	1	246070853	246071821	ENSMUSG00000043880	ENSMUSG00000043880	ENSMUSG00000043880	11	58441268	58442239	196	222		
9453	1	246087124	246108130	ENSMUSG00000037124	ENSMUSG00000037124	ENSMUSG00000037124	11	58456660	58468599	196	222		
9454	1	226741691	226750090	ENSMUSG00000020496	ENSMUSG00000020496	ENSMUSG00000020496	11	58750202	58754652	197	222	here	Sub blocks comes here
9455	1	226712431	226714794	ENSMUSG00000069311	ENSMUSG00000069311	ENSMUSG00000069311	11	58770243	58770707	197	222	sub blocks	2 -> 1
9456	1	226662264	226670977	ENSMUSG00000036964	ENSMUSG00000036964	ENSMUSG00000036964	11	58779976	58787928	197	222		
9457	1	226648004	226661140	ENSMUSG00000020455	ENSMUSG00000020455	ENSMUSG00000020455	11	58794311	58807653	197	222		
9458	1	226462454	226615574	ENSMUSG00000061462	ENSMUSG00000061462	ENSMUSG00000061462	11	58836653	58891359	197	222		
9459	1	226420139	226436581	ENSMUSG00000049287	ENSMUSG00000049287	ENSMUSG00000049287	11	58973628	58979940	197	222		
9460	1	226404038	226414146	ENSMUSG00000043448	ENSMUSG00000043448	ENSMUSG00000043448	11	58991761	58999408	197	222		
9461	1	226395958	226403274	ENSMUSG00000020444	ENSMUSG00000020444	ENSMUSG00000020444	11	59000070	59008011	197	222		
9462	1	226361003	226363349	ENSMUSG00000036860	ENSMUSG00000036860	ENSMUSG00000036860	11	59018687	59022331	197	222		
9463	1	226355057	226359735	ENSMUSG00000020441	ENSMUSG00000020441	ENSMUSG00000020441	11	59024556	59026931	197	222		
9464	1	226261375	226314628	ENSMUSG00000009900	ENSMUSG00000009900	ENSMUSG00000009900	11	59064228	59106947	197	222		
9465	1	226172980	226202222	ENSMUSG00000000126	ENSMUSG00000000126	ENSMUSG00000000126	11	59123123	59147570	197	222		
9466	1	226070017	226100794	ENSMUSG00000049291	ENSMUSG00000049291	ENSMUSG00000049291	11	59189111	59191825	197	222		
9467	1	225989320	226035550	ENSMUSG00000009894	ENSMUSG00000009894	ENSMUSG00000009894	11	59223329	59266194	197	222		
9468	1	225985513	225989735	ENSMUSG00000036819	ENSMUSG00000036819	ENSMUSG00000036819	11	59266283	59274263	197	222		
9469	1	245527337	245561668	ENSMUSG00000020472	ENSMUSG00000020472	ENSMUSG00000020472	11	59301739	59322111	196	223	here	ST is correct as it merged blocks separated by small block (197)
9470	1	245647974	245679027	ENSMUSG00000032691	ENSMUSG00000032691	ENSMUSG00000032691	11	59357780	59383151	196	223	Merge blocks seperated by smaller blocks	0 -> 1
9471	1	245680954	245681907	ENSMUSG00000059610	ENSMUSG00000059610	ENSMUSG00000059610	11	59386977	59387933	196	223		
9472	17	17008699	17029595	ENSMUSG00000005417	ENSMUSG00000005417	ENSMUSG00000005417	11	59478727	59592417	198	224		
9473	17	17045036	17050357	ENSMUSG00000043648	ENSMUSG00000043648	ENSMUSG00000043648	11	59600093	59603830	198	224		
9474	17	17056254	17072176	ENSMUSG00000032633	ENSMUSG00000032633	ENSMUSG00000032633	11	59607603	59620964	198	224		
9475	17	17090876	17125339	ENSMUSG00000019373	ENSMUSG00000019373	ENSMUSG00000019373	11	59633992	59655962	198	224		
9476	17	17147421	17191696	ENSMUSG00000032615	ENSMUSG00000032615	ENSMUSG00000032615	11	59664268	59692728	198	224		
9477	17	17321025	17337259	ENSMUSG00000061650	ENSMUSG00000061650	ENSMUSG00000061650	11	59764409	59779501	198	224		
9478	17	17338480	17340432	ENSMUSG00000049892	ENSMUSG00000049892	ENSMUSG00000049892	11	59779376	59781137	198	224		
9479	17	17349604	17435719	ENSMUSG00000000301	ENSMUSG00000000301	ENSMUSG00000000301	11	59786812	59862630	198	224		
9480	17	17525512	17655492	ENSMUSG00000062115	ENSMUSG00000062115	ENSMUSG00000062115	11	59992074	60015392	198	224		
9481	17	17656111	17681050	ENSMUSG00000020538	ENSMUSG00000020538	ENSMUSG00000020538	11	60015284	60036799	198	224		
9482	17	17690888	17816436	ENSMUSG00000000538	ENSMUSG00000000538	ENSMUSG00000000538	11	60042910	60169100	198	224		
9483	17	17816942	17860895	ENSMUSG00000056598	ENSMUSG00000056598	ENSMUSG00000056598	11	60169548	60193468	198	224		
9484	17	17862104	17883184	ENSMUSG00000042709	ENSMUSG00000042709	ENSMUSG00000042709	11	60216971	60233200	198	224		
9485	17	17883336	17912443	ENSMUSG00000018415	ENSMUSG00000018415	ENSMUSG00000018415	11	60233498	60261472	198	224		
9486	17	17932008	17952017	ENSMUSG00000020537	ENSMUSG00000020537	ENSMUSG00000020537	11	60270812	60284900	198	224		
9487	17	17952745	18023838	ENSMUSG00000042678	ENSMUSG00000042678	ENSMUSG00000042678	11	60285534	60344564	198	224		
9488	17	18028278	18052676	ENSMUSG00000042650	ENSMUSG00000042650	ENSMUSG00000042650	11	60354173	60372092	198	224		
9489	17	18069661	18088914	ENSMUSG00000020536	ENSMUSG00000020536	ENSMUSG00000020536	11	60515918	60530375	198	224		
9490	17	18088880	18102780	ENSMUSG00000002812	ENSMUSG00000002812	ENSMUSG00000002812	11	60530344	60543416	198	224		
9491	17	18104594	18109818	ENSMUSG00000018599	ENSMUSG00000018599	ENSMUSG00000018599	11	60544593	60549146	198	224		
9492	17	18117960	18159046	ENSMUSG00000002814	ENSMUSG00000002814	ENSMUSG00000002814	11	60556253	60593560	198	224		
9493	17	18159441	18167109	ENSMUSG00000049323	ENSMUSG00000049323	ENSMUSG00000049323	11	60594098	60600482	198	224		
9494	17	18171920	18207581	ENSMUSG00000020534	ENSMUSG00000020534	ENSMUSG00000020534	11	60604300	60627305	198	224		
9495	17	20970850	21035428	ENSMUSG00000042569	ENSMUSG00000042569	ENSMUSG00000042569	11	60646882	60674347	199	225		
9496	17	21041861	21058500	ENSMUSG00000043284	ENSMUSG00000043284	ENSMUSG00000043284	11	60680649	60695227	199	225		
9497	17	21087218	21097169	ENSMUSG00000018931	ENSMUSG00000018931	ENSMUSG00000018931	11	60718441	60729986	199	225		
9498	17	21142253	21159118	ENSMUSG00000018932	ENSMUSG00000018932	ENSMUSG00000018932	11	60748250	60769002	199	225		
9499	17	21220292	21260983	ENSMUSG00000042529	ENSMUSG00000042529	ENSMUSG00000042529	11	60881999	60887324	199	225		
9500	17	16783124	16816127	ENSMUSG00000010142	ENSMUSG00000010142	ENSMUSG00000010142	11	60942950	60965567	Singleton			
9501	17	20843502	20886944	ENSMUSG00000042506	ENSMUSG00000042506	ENSMUSG00000042506	11	60968136	60991215	199	226	here	Blocks 225, 226 and 227 defined by AG do not have orientation, and therefore ST merged them into sinlge block (199)
9502	17	19581895	19587280	ENSMUSG00000019102	ENSMUSG00000019102	ENSMUSG00000019102	11	61024940	61034609	199	226	Blocks without orientation merged with the neighboring block	1 -> 3
9503	17	19492656	19521496	ENSMUSG00000010025	ENSMUSG00000010025	ENSMUSG00000010025	11	61039612	61083342	199	226		
9504	17	19377759	19422938	ENSMUSG00000010122	ENSMUSG00000010122	ENSMUSG00000010122	11	61159595	61194259	199	226		
9505	17	19255315	19261179	ENSMUSG00000010086	ENSMUSG00000010086	ENSMUSG00000010086	11	61264637	61270044	199	226		
9506	17	19227349	19231086	ENSMUSG00000042436	ENSMUSG00000042436	ENSMUSG00000042436	11	61301639	61304899	199	226		
9507	17	19221659	19227445	ENSMUSG00000001034	ENSMUSG00000001034	ENSMUSG00000001034	11	61305007	61310410	199	226		
9508	17	19187082	19206619	ENSMUSG00000001039	ENSMUSG00000001039	ENSMUSG00000001039	11	61321339	61329126	199	226		
9509	17	19081303	19180620	ENSMUSG00000001036	ENSMUSG00000001036	ENSMUSG00000001036	11	61333445	61395858	199	226		
9510	17	18864716	18891061	ENSMUSG00000004837	ENSMUSG00000004837	ENSMUSG00000004837	11	61469516	61488972	199	226		
9511	17	18794708	18864720	ENSMUSG00000042371	ENSMUSG00000042371	ENSMUSG00000042371	11	61488977	61536994	199	226		
9512	17	18815400	18848079	ENSMUSG00000042377	ENSMUSG00000042377	ENSMUSG00000042377	11	61500656	61524350	199	226		
9513	17	19614736	19711841	ENSMUSG00000004798	ENSMUSG00000004798	ENSMUSG00000004798	11	61597766	61671268	199	227		
9514	17	19749344	19821721	ENSMUSG00000047804	ENSMUSG00000047804	ENSMUSG00000047804	11	61687502	61746447	199	227		
9515	17	19930927	20158660	ENSMUSG00000042331	ENSMUSG00000042331	ENSMUSG00000042331	11	61933894	62035569	199	227		
9516	17	15788956	15819935	ENSMUSG00000018500	ENSMUSG00000018500	ENSMUSG00000018500	11	62065179	62082647	200	228		
9517	17	15843507	15872821	ENSMUSG00000042298	ENSMUSG00000042298	ENSMUSG00000042298	11	62097679	62132619	200	228		
9518	17	15875984	16059570	ENSMUSG00000018501	ENSMUSG00000018501	ENSMUSG00000018501	11	62132885	62273527	200	228		
9519	17	16061259	16170286	ENSMUSG00000014245	ENSMUSG00000014245	ENSMUSG00000014245	11	62274655	62330095	200	228		
9520	17	16186574	16197538	ENSMUSG00000018509	ENSMUSG00000018509	ENSMUSG00000018509	11	62341141	62355456	200	228		
9521	17	16259613	16281040	ENSMUSG00000018507	ENSMUSG00000018507	ENSMUSG00000018507	11	62390681	62416494	200	228		
9522	17	16395436	16413189	ENSMUSG00000005267	ENSMUSG00000005267	ENSMUSG00000005267	11	62527458	62544861	200	228		
9523	17	18588283	18623336	ENSMUSG00000047821	ENSMUSG00000047821	ENSMUSG00000047821	11	62663264	62693660	Singleton			
9524	17	15280063	15311650	ENSMUSG00000042200	ENSMUSG00000042200	ENSMUSG00000042200	11	62767388	62809290	200	229	here	Presence of a singleton made AutoGRAPH to break the block
9525	17	15147856	15185624	ENSMUSG00000042189	ENSMUSG00000042189	ENSMUSG00000042189	11	62877854	62911155	200	229	presence of singletons	1 -> 2
9526	17	15073822	15109369	ENSMUSG00000018217	ENSMUSG00000018217	ENSMUSG00000018217	11	62947705	62975741	200	229		
9527	17	13913444	14052712	ENSMUSG00000042148	ENSMUSG00000042148	ENSMUSG00000042148	11	63780063	63895609	200	229		
9528	17	12836435	12862049	ENSMUSG00000020549	ENSMUSG00000020549	ENSMUSG00000020549	11	64795292	64818264	200	229		
9529	17	12633581	12835684	ENSMUSG00000033389	ENSMUSG00000033389	ENSMUSG00000033389	11	64819506	64891407	200	229		
9530	17	12509939	12607724	ENSMUSG00000020542	ENSMUSG00000020542	ENSMUSG00000020542	11	64992756	65086136	200	229		
9531	17	11864866	11987865	ENSMUSG00000033352	ENSMUSG00000033352	ENSMUSG00000033352	11	65504439	65604492	200	229		
9532	17	11821487	11841510	ENSMUSG00000018347	ENSMUSG00000018347	ENSMUSG00000018347	11	65623370	65645368	200	229		
9533	17	11442473	11813856	ENSMUSG00000056752	ENSMUSG00000056752	ENSMUSG00000056752	11	65647522	65984662	200	229		
9534	17	10669274	10669681	ENSMUSG00000048070	ENSMUSG00000048070	ENSMUSG00000048070	11	66728186	66744895	200	229		
9535	17	10557337	10574371	ENSMUSG00000050270	ENSMUSG00000050270	ENSMUSG00000050270	11	66841349	66850985	200	229		
9536	17	10548969	10555273	ENSMUSG00000020910	ENSMUSG00000020910	ENSMUSG00000020910	11	66854100	66858276	200	229		
9537	17	10524380	10541570	ENSMUSG00000069844	ENSMUSG00000069844	ENSMUSG00000069844	11	66868865	66881526	200	229		
9538	17	10472576	10500192	ENSMUSG00000020908	ENSMUSG00000020908	ENSMUSG00000020908	11	66896194	66918361	200	229		
9539	17	10365198	10393704	ENSMUSG00000033196	ENSMUSG00000033196	ENSMUSG00000033196	11	66987415	67013709	200	229		
9540	17	10336353	10360684	ENSMUSG00000056328	ENSMUSG00000056328	ENSMUSG00000056328	11	67016297	67040664	200	229		
9541	17	10287332	10313601	ENSMUSG00000057003	ENSMUSG00000057003	ENSMUSG00000057003	11	67054224	67076641	200	229		
9542	17	10234367	10265747	ENSMUSG00000055775	ENSMUSG00000055775	ENSMUSG00000055775	11	67093319	67124829	200	229		
9543	17	10145530	10217047	ENSMUSG00000060180	ENSMUSG00000060180	ENSMUSG00000060180	11	67143298	67187261	200	229		
9544	17	9754651	10042593	ENSMUSG00000033066	ENSMUSG00000033066	ENSMUSG00000033066	11	67363349	67501169	200	229		
9545	17	9741752	9749409	ENSMUSG00000020907	ENSMUSG00000020907	ENSMUSG00000020907	11	67511521	67519528	200	229		
9546	17	9670106	9733747	ENSMUSG00000049928	ENSMUSG00000049928	ENSMUSG00000049928	11	67522625	67587348	200	229		
9547	17	9615536	9635371	ENSMUSG00000033044	ENSMUSG00000033044	ENSMUSG00000033044	11	67614483	67632200	200	229		
9548	17	9489675	9573032	ENSMUSG00000020905	ENSMUSG00000020905	ENSMUSG00000020905	11	67670721	67738185	200	229		
9549	17	9420684	9487489	ENSMUSG00000020904	ENSMUSG00000020904	ENSMUSG00000020904	11	67741007	67781837	200	229		
9550	17	9094514	9420000	ENSMUSG00000020903	ENSMUSG00000020903	ENSMUSG00000020903	11	67782678	68023348	200	229		
9551	17	8865584	9084078	ENSMUSG00000020902	ENSMUSG00000020902	ENSMUSG00000020902	11	68029187	68202331	200	229		
9552	17	8722953	8756559	ENSMUSG00000020901	ENSMUSG00000020901	ENSMUSG00000020901	11	68248320	68314041	200	229		

9553	17	8647316	8683749	ENSMUSG00000046207	ENSMUSG00000046207	ENSMUSG00000046207	11	68319293	68368890	200	229
9554	17	8641403	8643163	ENSMUSG00000048329	ENSMUSG00000048329	ENSMUSG00000048329	11	68372381	68374440	200	229
9555	17	8573977	8588879	ENSMUSG00000045915	ENSMUSG00000045915	ENSMUSG00000045915	11	68403422	68414149	200	229
9556	17	8318248	8474804	ENSMUSG00000020900	ENSMUSG00000020900	ENSMUSG00000020900	11	68508675	68632739	200	229
9557	17	8279895	8324667	ENSMUSG00000018736	ENSMUSG00000018736	ENSMUSG00000018736	11	68637629	68669257	200	229
9558	17	8236842	8237504	ENSMUSG00000046490	ENSMUSG00000046490	ENSMUSG00000046490	11	68704748	68711201	200	229
9559	17	8183907	8190089	ENSMUSG00000032921	ENSMUSG00000032921	ENSMUSG00000032921	11	68738030	68743244	200	229
9560	17	8154284	8166559	ENSMUSG00000052921	ENSMUSG00000052921	ENSMUSG00000052921	11	68759349	68773053	200	229
9561	17	8131807	8139386	ENSMUSG00000018740	ENSMUSG00000018740	ENSMUSG00000018740	11	68784326	68788711	200	229
9562	17	8093338	8114534	ENSMUSG00000020899	ENSMUSG00000020899	ENSMUSG00000020899	11	68801895	68820032	200	229
9563	17	8070918	8092085	ENSMUSG00000020898	ENSMUSG00000020898	ENSMUSG00000020898	11	68832291	68852665	200	229
9564	17	8048776	8054661	ENSMUSG00000020897	ENSMUSG00000020897	ENSMUSG00000020897	11	68861838	68867857	200	229
9565	17	8033113	8034183	ENSMUSG00000045176	ENSMUSG00000045176	ENSMUSG00000045176	11	68875929	68877771	200	229
9566	17	8017785	8020329	ENSMUSG00000020895	ENSMUSG00000020895	ENSMUSG00000020895	11	68887008	68888793	200	229
9567	17	8003189	8007017	ENSMUSG00000020894	ENSMUSG00000020894	ENSMUSG00000020894	11	68904705	68908573	200	229
9568	17	7984534	7996427	ENSMUSG00000020893	ENSMUSG00000020893	ENSMUSG00000020893	11	68915129	68926155	200	229
9569	17	7965614	7968127	ENSMUSG00000023781	ENSMUSG00000023781	ENSMUSG00000023781	11	68936648	68939454	200	229
9570	17	7939943	7962532	ENSMUSG00000020892	ENSMUSG00000020892	ENSMUSG00000020892	11	68942572	68965309	200	229
9571	17	7916679	7931747	ENSMUSG00000032807	ENSMUSG00000032807	ENSMUSG00000032807	11	68973267	68985986	200	229
9572	17	7883086	7893177	ENSMUSG00000020891	ENSMUSG00000020891	ENSMUSG00000020891	11	69000080	69014034	200	229
9573	17	7846713	7864383	ENSMUSG00000020890	ENSMUSG00000020890	ENSMUSG00000020890	11	69039230	69053217	200	229
9574	17	7776198	7793619	ENSMUSG00000032782	ENSMUSG00000032782	ENSMUSG00000032782	11	69116069	69136163	200	229
9575	17	7774388	7775991	ENSMUSG00000049299	ENSMUSG00000049299	ENSMUSG00000049299	11	69140353	69141941	200	229
9576	17	7765902	7773478	ENSMUSG00000018470	ENSMUSG00000018470	ENSMUSG00000018470	11	69142453	69149236	200	229
9577	17	7757383	7759985	ENSMUSG00000043419	ENSMUSG00000043419	ENSMUSG00000043419	11	69157012	69158836	200	229
9578	17	7728849	7756246	ENSMUSG00000018474	ENSMUSG00000018474	ENSMUSG00000018474	11	69160546	69185589	200	229
9579	17	7700730	7701466	ENSMUSG00000059278	ENSMUSG00000059278	ENSMUSG00000059278	11	69211986	69212866	200	229
9580	17	7699101	7700142	ENSMUSG00000045377	ENSMUSG00000045377	ENSMUSG00000045377	11	69212714	69214429	200	229
9581	17	7683947	7698831	ENSMUSG00000018476	ENSMUSG00000018476	ENSMUSG00000018476	11	69214703	69229870	200	229
9582	17	7563778	7677576	ENSMUSG00000005237	ENSMUSG00000005237	ENSMUSG00000005237	11	69237006	69360737	200	229
9583	17	7549245	7555421	ENSMUSG00000003934	ENSMUSG00000003934	ENSMUSG00000003934	11	69370320	69376373	200	229
9584	17	7530723	7547542	ENSMUSG00000041346	ENSMUSG00000041346	ENSMUSG00000041346	11	69377957	69396451	200	229
9585	17	7512464	7531642	ENSMUSG00000059552	ENSMUSG00000059552	ENSMUSG00000059552	11	69396600	69407992	200	229
9586	17	7494979	7501812	ENSMUSG00000041329	ENSMUSG00000041329	ENSMUSG00000041329	11	69415937	69422137	200	229
9587	17	7474193	7477426	ENSMUSG00000005202	ENSMUSG00000005202	ENSMUSG00000005202	11	69431028	69434100	200	229
9588	17	7470277	7471912	ENSMUSG00000069835	ENSMUSG00000069835	ENSMUSG00000069835	11	69438247	69440065	200	229
9589	17	7435272	7458885	ENSMUSG00000018765	ENSMUSG00000018765	ENSMUSG00000018765	11	69449166	69469492	200	229
9590	17	7432222	7434212	ENSMUSG00000041287	ENSMUSG00000041287	ENSMUSG00000041287	11	69471568	69472816	200	229
9591	17	7427698	7432247	ENSMUSG00000018761	ENSMUSG00000018761	ENSMUSG00000018761	11	69472909	69478785	200	229
9592	17	7423646	7426155	ENSMUSG00000018774	ENSMUSG00000018774	ENSMUSG00000018774	11	69480409	69482322	200	229
9593	17	7416748	7423048	ENSMUSG00000059796	ENSMUSG00000059796	ENSMUSG00000059796	11	69483131	69488618	200	229
9594	17	7405996	7415912	ENSMUSG00000005204	ENSMUSG00000005204	ENSMUSG00000005204	11	69489313	69498041	200	229
9595	17	7393140	7405649	ENSMUSG00000018752	ENSMUSG00000018752	ENSMUSG00000018752	11	69498772	69512293	200	229
9596	17	7328422	7358653	ENSMUSG00000005198	ENSMUSG00000005198	ENSMUSG00000005198	11	69550305	69574488	200	229
9597	17	7303409	7328241	ENSMUSG00000018750	ENSMUSG00000018750	ENSMUSG00000018750	11	69592066	69595596	200	229
9598	17	7289104	7301750	ENSMUSG00000041189	ENSMUSG00000041189	ENSMUSG00000041189	11	69600231	69612138	200	229
9599	17	7283413	7288980	ENSMUSG00000042826	ENSMUSG00000042826	ENSMUSG00000042826	11	69612268	69618052	200	229
9600	17	7279486	7281722	ENSMUSG00000070397	ENSMUSG00000070397	ENSMUSG00000070397	11	69619798	69621819	200	229
9601	17	7269659	7271608	ENSMUSG00000044084	ENSMUSG00000044084	ENSMUSG00000044084	11	69632761	69634662	200	229
9602	17	7264403	7265674	ENSMUSG00000041165	ENSMUSG00000041165	ENSMUSG00000041165	11	69637067	69638360	200	229
9603	17	7252226	7263903	ENSMUSG00000051790	ENSMUSG00000051790	ENSMUSG00000051790	11	69639317	69651044	200	229
9604	17	7247038	7248142	ENSMUSG00000070394	ENSMUSG00000070394	ENSMUSG00000070394	11	69654746	69655807	200	229
9605	17	7233773	7238565	ENSMUSG00000019461	ENSMUSG00000019461	ENSMUSG00000019461	11	69662871	69668253	200	229
9606	17	7225134	7233780	ENSMUSG00000001583	ENSMUSG00000001583	ENSMUSG00000001583	11	69667200	69674925	200	229
9607	17	7180572	7198987	ENSMUSG00000001588	ENSMUSG00000001588	ENSMUSG00000001588	11	69697762	69711734	200	229
9608	17	7159677	7173362	ENSMUSG00000047284	ENSMUSG00000047284	ENSMUSG00000047284	11	69718086	69730015	200	229
9609	17	7151042	7156498	ENSMUSG00000041070	ENSMUSG00000041070	ENSMUSG00000041070	11	69732917	69738153	200	229
9610	17	7132322	7138543	ENSMUSG00000018554	ENSMUSG00000018554	ENSMUSG00000018554	11	69752170	69757792	200	229
9611	17	7125777	7132300	ENSMUSG00000018566	ENSMUSG00000018566	ENSMUSG00000018566	11	69758738	69764329	200	229
9612	17	7096096	7103974	ENSMUSG00000018565	ENSMUSG00000018565	ENSMUSG00000018565	11	69784419	69796705	200	229
9613	17	7087634	7096004	ENSMUSG00000018559	ENSMUSG00000018559	ENSMUSG00000018559	11	69797363	69806796	200	229
9614	17	7084464	7086481	ENSMUSG00000018567	ENSMUSG00000018567	ENSMUSG00000018567	11	69807559	69811141	200	229
9615	17	7079669	7083322	ENSMUSG00000018572	ENSMUSG00000018572	ENSMUSG00000018572	11	69811972	69816111	200	229
9616	17	7069384	7078592	ENSMUSG00000020888	ENSMUSG00000020888	ENSMUSG00000020888	11	69816790	69828496	200	229
9617	17	7063855	7069311	ENSMUSG00000018574	ENSMUSG00000018574	ENSMUSG00000018574	11	69826378	69831519	200	229
9618	17	7033936	7063754	ENSMUSG00000020886	ENSMUSG00000020886	ENSMUSG00000020886	11	69835059	69860970	200	229
9619	17	7017474	7023607	ENSMUSG00000020884	ENSMUSG00000020884	ENSMUSG00000020884	11	69870590	69874072	200	229
9620	17	6945365	6958283	ENSMUSG00000040963	ENSMUSG00000040963	ENSMUSG00000040963	11	69909265	69922382	200	229
9621	17	6885673	6887966	ENSMUSG00000040938	ENSMUSG00000040938	ENSMUSG00000040938	11	70030706	70032561	200	229
9622	17	6880426	6884005	ENSMUSG00000044367	ENSMUSG00000044367	ENSMUSG00000044367	11	70032987	70037189	200	229
9623	17	6867113	6872088	ENSMUSG00000000317	ENSMUSG00000000317	ENSMUSG00000000317	11	70040323	70045367	200	229
9624	17	6858901	6861545	ENSMUSG00000020831	ENSMUSG00000020831	ENSMUSG00000020831	11	70051889	70053999	200	229
9625	17	6840108	6856220	ENSMUSG00000000320	ENSMUSG00000000320	ENSMUSG00000000320	11	70057650	70071536	200	229
9626	17	6737825	6744072	ENSMUSG00000018907	ENSMUSG00000018907	ENSMUSG00000018907	11	70131808	70138713	200	229
9627	17	4480963	4491707	ENSMUSG00000018924	ENSMUSG00000018924	ENSMUSG00000018924	11	70160347	70168210	201	230
9628	17	4521437	4554155	ENSMUSG00000018921	ENSMUSG00000018921	ENSMUSG00000018921	11	70209079	70226226	201	230
9629	17	4560533	4571544	ENSMUSG00000060216	ENSMUSG00000060216	ENSMUSG00000060216	11	70248840	70257023	201	230
9630	17	4581457	4583637	ENSMUSG00000018923	ENSMUSG00000018923	ENSMUSG00000018923	11	70268126	70269922	201	230
9631	17	4583577	4589972	ENSMUSG00000018920	ENSMUSG00000018920	ENSMUSG00000018920	11	70270429	70276179	201	230
9632	17	4590557	4596159	ENSMUSG00000040829	ENSMUSG00000040829	ENSMUSG00000040829	11	70275817	70282394	201	230
9633	17	4621936	4633255	ENSMUSG00000018919	ENSMUSG00000018919	ENSMUSG00000018919	11	70321472	70327367	201	230
9634	17	4635321	4636471	ENSMUSG00000020830	ENSMUSG00000020830	ENSMUSG00000020830	11	70329711	70330811	201	230
9635	17	4639047	4640331	ENSMUSG00000046811	ENSMUSG00000046811	ENSMUSG00000046811	11	70335391	70336930	201	230
9636	17	4646397	4648756	ENSMUSG00000018286	ENSMUSG00000018286	ENSMUSG00000018286	11	70341581	70343840	201	230
9637	17	4657348	4673694	ENSMUSG00000020828	ENSMUSG00000020828	ENSMUSG00000020828	11	70356359	70374305	201	230
9638	17	4728437	4741358	ENSMUSG00000020827	ENSMUSG00000020827	ENSMUSG00000020827	11	70413363	70429907	201	230
9639	17	4741840	4747148	ENSMUSG00000014609	ENSMUSG00000014609	ENSMUSG00000014609	11	70431078	70435389	201	230
9640	17	4776680	4778599	ENSMUSG00000050675	ENSMUSG00000050675	ENSMUSG00000050675	11	70455317	70458253	201	230
9641	17	4781169	4784208	ENSMUSG00000014606	ENSMUSG00000014606	ENSMUSG00000014606	11	70460391	70463188	201	230
9642	17	4784048	4789231	ENSMUSG00000040746	ENSMUSG00000040746	ENSMUSG00000040746	11	70463784	70467616	201	230
9643	17	4789692	4793054	ENSMUSG00000018293	ENSMUSG00000018293	ENSMUSG00000018293	11	70468044	70470830	201	230
9644	17	4795135	4801150	ENSMUSG00000060600	ENSMUSG00000060600	ENSMUSG00000060600	11	70473399	70478705	201	230
9645	17	4803353	4811832	ENSMUSG00000018287	ENSMUSG00000018287	ENSMUSG00000018287	11	70479985	70485488	201	230
9646	17	4812017	4831655	ENSMUSG00000040712	ENSMUSG00000040712	ENSMUSG00000040712	11	70485662	70504129	201	230
9647	17	4832149	4841629	ENSMUSG00000057054	ENSMUSG00000057054	ENSMUSG00000057054	11	70504556	70516350	201	230
9648	17	4841967	4872420	ENSMUSG00000020821	ENSMUSG00000020821	ENSMUSG00000020821	11	70516856	70546619	201	230
9649	17	4922478	4940395	ENSMUSG00000043602	ENSMUSG00000043602	ENSMUSG00000043602	11	70580642	70589123	201	230
9650	17	5052980	5067496	ENSMUSG00000057135	ENSMUSG00000057135	ENSMUSG00000057135	11	70607631	70616996	201	230
9651	17	5126506	5227242	ENSMUSG00000020817	ENSMUSG00000020817	ENSMUSG00000020817	11	70660977	70757498	201	230
9652	17	5230070	5263724	ENSMUSG00000040667	ENSMUSG00000040667	ENSMUSG00000040667	11	70759253	70786156	201	230
9653	17	5263685	5276920	ENSMUSG00000018449	ENSMUSG00000018449	ENSMUSG00000018449	11	70786408	70793999	201	230
9654	17	5276676	5283125	ENSMUSG00000018446	ENSMUSG00000018446	ENSMUSG00000018446	11	70794002	70799169	201	230
9655	17	5284956	5312905	ENSMUSG00000040620	ENSMUSG00000040620	ENSMUSG00000040620	11	70800286	70820627	201	230
9656	17	5315295	5330261	ENSMUSG00000018442	ENSMUSG00000018442	ENSMUSG00000018442	11	70823640	70835458	201	230
9657	17	5330440	5334858	ENSMUSG00000040599	ENSMUSG00000040599	ENSMUSG00000040599	11	70835806	70843329	201	230
9658	17	5914658	5968469	ENSMUSG00000020811	ENSMUSG00000020811	ENSMUSG00000020811	11	71566898	71605844	201	230
9659	17	6267781	6279209	ENSMUSG00000040554	ENSMUSG00000040554	ENSMUSG00000040554	11	71845360	71853653	201	230
9660	17	6295307	6400601	ENSMUSG00000040543	ENSMUSG00000040543	ENSMUSG00000040543	11	71863723	71951973	201	230
9661	17	6422375	6484716	ENSMUSG00000020807	ENSMUSG00000020807	ENSMUSG00000020807	11	71970125	72023654	201	230
9662	17	6484802	6488644	ENSMUSG00000020803	ENSMUSG00000020803	ENSMUSG00000020803	11	72023749	72026682	201	230
9663	17	6487359	6495678	ENSMUSG00000020801	ENSMUSG00000020801	ENSMUSG00000020801	11	72027919	72031787	201	230
9664	17	6528756	6557411	ENSMUSG00000020805	ENSMUSG00000020805	ENSMUSG00000020805	11	72059700	72082760	201	230
9665	17	6599518	6619685	ENSMUSG00000040483	ENSMUSG00000040483	ENSMUSG00000040483	11	72117873	72123087	201	230
9666	17	6620275	6631689	ENSMUSG00000070388	ENSMUSG00000070388	ENSMUSG00000070388	11	72133019	72135406	201	230
9667	17	6642036	6675804	ENSMUSG00000020799	ENSMUSG00000020799	ENSMUSG00000020799	11	72160915	72178068	201	230
9668	17	4434650	4457528	ENSMUSG00000045667	ENSMUSG00000045667	ENSMUSG00000045667	11	72206309	72227907	202	231
9669	17	4407376	4410610	ENSMUSG00000040471	ENSMUSG00000040471	ENSMUSG00000040471	11	72251721	72254758	202	231
9670	17	4388984	4405393	ENSMUSG00000040463	ENSMUSG00000040463	ENSMUSG00000040463	11	72257550	72267963	202	231
9671	17	4348882	4389078	ENSMUSG00000040447	ENSMUSG00000040447	ENSMUSG00000040447	11	72267833	72306115	202	231
9672	17	4283968	4338247	ENSMUSG00000020798	ENSMUSG00000020798	ENSMUSG00000020798	11	72314351	72366441	202	231
9673	17	4013950	4114000	ENSMUSG00000020790	ENSMUSG00000020790	ENSMUSG00000020790	11	72506201	72588341	202	231
9674	17	3993299	4007738	ENSMUSG00000057778	ENSMUSG00000057778	ENSMUSG00000057778	11	72593444	72611957	202	231
9675	17	3854488	3993063	ENSMUSG00000055670	ENSMUSG00000055670	ENSMUSG00000055670	11	72612492	72743315	202	231
9676	17	3773918	3814485	ENSMUSG00000020788	ENSMUSG00000020788	ENSMUSG00000020788	11	72777450	72809234	202	231
9677	17	3746640	3766709	ENSMUSG00000020787	ENSMUSG00000020787	ENSMUSG00000020787	11	72815337	72831395	202	231
9678	17	3710358	3740787	ENSMUSG00000020785	ENSMUSG00000020785	ENSMUSG00000020785	11	72835238	72858258	202	231
9679	17	3663090	3696266	ENSMUSG00000020783	ENSMUSG00000020783	ENSMUSG00000020783	11	72864075	72896411	202	231
9680	17	3564672	3651293	ENSMUSG00000005947	ENSMUSG00000005947	ENSMUSG00000005947	11	72906778	72963636	202	231
9681	17	3573946	3576742	ENSMUSG00000050107	ENSMUSG00000050107	ENSMUSG00000050107	11	72952192	72954456	202	231
9682	17	3513106	3546237	ENSMUSG00000005950	ENSMUSG00000005950	ENSMUSG00000005950	11	72976720	72988880	202	231
9683	17	3518835	3519709	ENSMUSG00000047260	ENSMUSG00000047260	ENSMUSG00000047260	11	72991728	72993232	202	231
9684	17	3486522	3511585	ENSMUSG00000005949	ENSMUSG00000005949	ENSMUSG00000005949	11	72999328	73015214	202	231
9685	17	3458305	3486365	ENSMUSG00000005951	ENSMUSG00000005951	ENSMUSG00000005951	11	73015677	73040701	202	231
9686	17	3415493	3447141	ENSMUSG00000005952	ENSMUSG00000005952	ENSMUSG00000005952	11	73050516	73077434	202	231
9687	17	3360546	3408039	ENSMUSG00000043029	ENSMUSG00000043029	ENSMUSG00000043029	11	73083815	73113395	202	231
9688	17	3326046	3353463	ENSMUSG00000020774	ENSMUSG00000020774	ENSMUSG00000020774	11	73121183	73140829	202	231
9689	17	3290058	3321839	ENSMUSG00000069825	ENSMUSG00000069825	ENSMUSG00000069825	11	73147347	73162141	202	231
9690	17	3160355	3161401	ENSMUSG00000043692	ENSMUSG00000043692	ENSMUSG00000043692	11	73870017	73870952	202	231
9691	17	2646482	2884500	ENSMUSG00000038807	ENSMUSG00000038807	ENSMUSG00000038807	11	74199551	74406381	202	231
9692	17	2572895	2585219	ENSMUSG00000069814	ENSMUSG00000069814	ENSMUSG00000069814	11	74435800	74454983	202	231
9693	17	2539432	2554594	ENSMUSG00000020741	ENSMUSG00000020741	ENSMUSG00000020741	11	74465735	74487042	202	231
9694	17	2443686	2535638	ENSMUSG00000020745	ENSMUSG00000020745	ENSMUSG00000020745	11	74490147	74540865	202	231
9695	17	2268539	2361930	ENSMUSG00000010554	ENSMUSG00000010554	ENSMUSG00000010554	11	74589717	74633953	202	231
9696	17	2234104	2251162	ENSMUSG00000000282	ENSMUSG00000000282	ENSMUSG00000000282	11	74647119	74661905	202	231
9697	17	2187556	2231094	ENSMUSG00000038351	ENSMUSG00000038351	ENSMUSG00000038351	11	74665459	74713266	202	231
9698	17	2172742	2187428	ENSMUSG00000038335	ENSMUSG00000038335	ENSMUSG00000038335	11	74714302	74725535	202	231
9699	17	2153998	2175304	ENSMUSG00000001323	ENSMUSG00000001323	ENSMUSG00000001323	11	74722991	74741898	202	231
9700	17	1909888	2220160	ENSMUSG00000038290	ENSMUSG00000038290	ENSMUSG00000038290	11	74742056	74980635	202	231
9701	17	1906354	1909070	ENSMUSG00000043099	ENSMUSG00000043099	ENSMUSG00000043099	11	74980763	74985714	202	231
9702	17	1880156	1893469	ENSMUSG00000038268	ENSMUSG00000038268	ENSMUSG00000038268	11	74992299	75006669	202	231
9703	17	1786540	1787865	ENSMUSG00000045287	ENSMUSG00000045287	ENSMUSG00000045287	11	75009978	75083964	202	231
9704	17	1680016	1749598	ENSMUSG00000000751	ENSMUSG00000000751	ENSMUSG00000000751	11	75116456	75164505	202	231
9705	17	1629579	1679894	ENSMUSG00000018809	ENSMUSG00000018809	ENSMUSG00000018809	11	75164693	75221900	202	231
9706	17	1612003	1627609	ENSMUSG00000000753	ENSMUSG00000000753	ENSMUSG00000000753	11	75225964	75238709	202	231
9707	17	1593070	1605310	ENSMUSG00000038224	ENSMUSG00000038224	ENSMUSG00000038224	11	75247932	75255698	202	231
9708	17	1577618	1588628	ENSMUSG00000045374	ENSMUSG00000045374	ENSMUSG00000045374	11	75257148	75267493	202	231
9709	17	1557874	1560292	ENSMUSG00000038217	ENSMUSG00000038217	ENSMUSG00000038217	11	75284400	75286758	202	231
9710	17	1500673	1534904	ENSMUSG00000020850	ENSMUSG00000020850	ENSMUSG00000020850	11	75303015	75325644	202	231
9711	17	1496194	1500102	ENSMUSG00000038195	ENSMUSG00000038195	ENSMUSG00000038195	11	75326289	75329363	202	231
9712	17	1483902	1495792	ENSMUSG00000038188	ENSMUSG00000038188	ENSMUSG00000038188	11	75329736	75342775	202	231
9713	17	1424448	1478880	ENSMUSG00000038178	ENSMUSG00000038178	ENSMUSG00000038178	11	75347898	75391236	202	231
9714	17	1368037	1412835	ENSMUSG00000017781	ENSMUSG00000017781	ENSMUSG00000017781	11	75404292	75444999	202	231
9715	17	1344622	1366719	ENSMUSG00000006127	ENSMUSG00000006127	ENSMUSG00000006127	11	75447183	75465066	202	231
9716	17	1314230	1342745	ENSMUSG00000017774	ENSMUSG00000017774	ENSMUSG00000017774	11	75467705	75490105	202	231
9717	17	1270733	1306302	ENSMUSG00000017776	ENSMUSG00000017776	ENSMUSG00000017776	11	75495505	75522289	202	231
9718	17	62293	202888	ENSMUSG00000020847	ENSMUSG00000020847	ENSMUSG00000020847	11	75648332	75727257	203	232
9719	17	293183	295958	ENSMUSG00000020846	ENSMUSG00000020846	ENSMUSG00000020846	11	75837453	75843977	203	232
9720	17	369121	564699	ENSMUSG00000017288	ENSMUSG00000017288	ENSMUSG00000017288	11	75862421	75995825	203	232
9721	17	582548	591560	ENSMUSG00000069808	ENSMUSG00000069808	ENSMUSG00000069808	11	76018292	76025611	203	232
9722	17	594856	598032	ENSMUSG00000049396	ENSMUSG00000049396	ENSMUSG00000049396	11	76026766	76033859	203	232
9723	17	609300	633363	ENSMUSG00000017286	ENSMUSG00000017286	ENSMUSG00000017286	11	76037718	76059886	203	232
9724	17	632263	642499	ENSMUSG00000038046	ENSMUSG00000038046	ENSMUSG00000038046	11	76059965	76066817	203	232
9725	17	649331	829760	ENSMUSG00000020844	ENSMUSG00000020844	ENSMUSG00000020844	11	76073437	76215335	203	232
9726	17	847107	852138	ENSMUSG00000020843	ENSMUSG00000020843	ENSMUSG00000020843	11	76223385	76232487	203	232
9727	17	853508	1029881	ENSMUSG00000017631	ENSMUSG00000017631	ENSMUSG00000017631	11	76232930	76387286	203	232
9728	17	1120155	1121315	ENSMUSG00000044243	ENSMUSG00000044243	ENSMUSG00000044243	11	76488665	76489871	203	232
9729	17	25828552	25877957	ENSMUSG00000010392	ENSMUSG00000010392	ENSMUSG00000010392	11	76542797	76579750	204	233
9730	17	25730110	25819825	ENSMUSG00000020841	ENSMUSG00000020841	ENSMUSG00000020841	11	76598184	76663191	204	233
9731	17	25667492	25685191	ENSMUSG00000020839	ENSMUSG00000020839	ENSMUSG00000020839	11	76718354	76732782	204	233
9732	17	25599349	25643200	ENSMUSG00000020840	ENSMUSG00000020840	ENSMUSG00000020840	11	76761939	76803573	204	233
9733	17	25549032	25586831	ENSMUSG00000020838	ENSMUSG00000020838	ENSMUSG00000020838	11	76814792	76848536	204	233
9734	17	25467960	25537617	ENSMUSG00000037958	ENSMUSG00000037958	ENSMUSG00000037958	11	76860487	76894630	204	233
9735	17	25320327	25459595	ENSMUSG00000050944	ENSMUSG00000050944	ENSMUSG00000050944	11	76906110	76968174	204	233
9736	17	24977091	25281144	ENSMUSG00000037926	ENSMUSG00000037926	ENSMUSG00000037926	11	77032620	77271743	204	233
9737	17	24966879	24972565	ENSMUSG00000020836	ENSMUSG00000020836	ENSMUSG00000020836	11	77280108	77285696	204	233
9738	17	24944612	24965904	ENSMUSG00000037907	ENSMUSG00000037907	ENSMUSG00000037907	11	77286683	77305747	204	233
9739	17	24924620	24940735	ENSMUSG00000011877	ENSMUSG00000011877	ENSMUSG00000011877	11	77309607	77323969	204	233
9740	17	24919788	24924301	ENSMUSG00000044328	ENSMUSG00000044328	ENSMUSG00000044328	11	77324304	77329426	204	233
9741	17	24911817	24918168	ENSMUSG00000000686	ENSMUSG00000000686	ENSMUSG00000000686	11	77331316	77336240	204	233
9742	17	24742069	24895628	ENSMUSG00000017291	ENSMUSG00000017291	ENSMUSG00000017291	11	77353793	77401644	204	233
9743	17	24612772	24645262	ENSMUSG00000037857	ENSMUSG00000037857	ENSMUSG00000037857	11	77502350	77557987	204	233
9744	17	24598001	24605626	ENSMUSG00000000724	ENSMUSG00000000724	ENSMUSG00000000724	11	77534927	77541488	204	233
9745	17	24424663	24531556	ENSMUSG00000000631	ENSMUSG00000000631	ENSMUSG00000000631	11	77593460	77682180	204	233
9746	17	24394044	24408362	ENSMUSG00000017453	ENSMUSG00000017453	ENSMUSG00000017453	11	77696815	77710028	204	233
9747	17	24306075	24357584	ENSMUSG00000000632	ENSMUSG00000000632	ENSMUSG00000000632	11	77747138	77795235	204	233

9748	17	24256417	24302634	ENSMUSG00000037791	ENSMUSG00000037791	ENSMUSG00000037791	11	77799408	77846731	204	233		
9749	17	24248928	24254215	ENSMUSG00000020834	ENSMUSG00000020834	ENSMUSG00000020834	11	77848534	77854057	204	233		
9750	17	24230484	24248696	ENSMUSG00000061981	ENSMUSG00000061981	ENSMUSG00000061981	11	77854174	77876626	204	233		
9751	17	24206153	24212201	ENSMUSG00000020832	ENSMUSG00000020832	ENSMUSG00000020832	11	77889575	77896567	204	233		
9752	17	24109415	24193934	ENSMUSG00000037750	ENSMUSG00000037750	ENSMUSG00000037750	11	77910972	77972251	204	233		
9753	17	24095166	24101223	ENSMUSG00000017386	ENSMUSG00000017386	ENSMUSG00000017386	11	77973506	77981722	204	233		
9754	17	24079959	24093911	ENSMUSG00000017405	ENSMUSG00000017405	ENSMUSG00000017405	11	77982301	77992870	204	233		
9755	17	24075504	24077353	ENSMUSG00000019437	ENSMUSG00000019437	ENSMUSG00000019437	11	77994961	77996967	204	233		
9756	17	24065430	24069328	ENSMUSG00000002059	ENSMUSG00000002059	ENSMUSG00000002059	11	78004635	78008382	204	233		
9757	17	24054661	24062999	ENSMUSG00000044122	ENSMUSG00000044122	ENSMUSG00000044122	11	78011068	78021662	204	233		
9758	17	24013429	24053375	ENSMUSG00000002052	ENSMUSG00000002052	ENSMUSG00000002052	11	78023219	78061860	204	233		
9759	17	23999734	24013060	ENSMUSG00000002064	ENSMUSG00000002064	ENSMUSG00000002064	11	78061941	78071681	204	233		
9760	17	23965599	23996256	ENSMUSG00000010277	ENSMUSG00000010277	ENSMUSG00000010277	11	78077951	78106818	204	233		
9761	17	23959109	23965338	ENSMUSG00000037593	ENSMUSG00000037593	ENSMUSG00000037593	11	78107078	78110000	204	233		
9762	17	23928735	23950424	ENSMUSG00000002055	ENSMUSG00000002055	ENSMUSG00000002055	11	78117792	78138652	204	233		
9763	17	23924261	23928078	ENSMUSG00000017390	ENSMUSG00000017390	ENSMUSG00000017390	11	78139163	78143976	204	233		
9764	17	23904535	23922664	ENSMUSG00000041958	ENSMUSG00000041958	ENSMUSG00000041958	11	78144617	78158973	204	233		
9765	17	23897852	23903770	ENSMUSG00000002058	ENSMUSG00000002058	ENSMUSG00000002058	11	78159717	78165351	204	233		
9766	17	23875086	23889302	ENSMUSG00000002057	ENSMUSG00000002057	ENSMUSG00000002057	11	78174483	78202753	204	233		
9767	17	23824791	23848514	ENSMUSG00000001095	ENSMUSG00000001095	ENSMUSG00000001095	11	78213471	78238380	204	233		
9768	17	23750179	23757355	ENSMUSG00000020829	ENSMUSG00000020829	ENSMUSG00000020829	11	78281911	78288138	204	233		
9769	17	23723181	23747450	ENSMUSG00000050132	ENSMUSG00000050132	ENSMUSG00000050132	11	78289488	78313949	204	233		
9770	17	23718425	23721844	ENSMUSG00000017344	ENSMUSG00000017344	ENSMUSG00000017344	11	78315315	78318518	204	233		
9771	17	23715417	23716392	ENSMUSG00000001103	ENSMUSG00000001103	ENSMUSG00000001103	11	78319708	78321276	204	233		
9772	17	23708814	23713215	ENSMUSG00000051232	ENSMUSG00000051232	ENSMUSG00000051232	11	78323250	78328363	204	233		
9773	17	23697787	23708600	ENSMUSG00000001100	ENSMUSG00000001100	ENSMUSG00000001100	11	78328479	78338931	204	233		
9774	17	23686913	23698160	ENSMUSG00000017615	ENSMUSG00000017615	ENSMUSG00000017615	11	78339043	78352389	204	233		
9775	17	23679490	23686602	ENSMUSG00000001105	ENSMUSG00000001105	ENSMUSG00000001105	11	78352631	78357668	204	233		
9776	17	23393309	23547529	ENSMUSG00000017376	ENSMUSG00000017376	ENSMUSG00000017376	11	78383361	78513568	204	233		
9777	17	23107922	23151682	ENSMUSG00000020826	ENSMUSG00000020826	ENSMUSG00000020826	11	78737046	78776416	204	233		
9778	17	22807797	22974545	ENSMUSG00000018334	ENSMUSG00000018334	ENSMUSG00000018334	11	78829635	78962602	204	233		
9779	17	22645233	22664772	ENSMUSG00000017677	ENSMUSG00000017677	ENSMUSG00000017677	11	79055577	79070890	204	233		
9780	17	26446275	26725609	ENSMUSG00000020716	ENSMUSG00000020716	ENSMUSG00000020716	11	79156087	79397804	205	234		
9781	17	26645795	26648481	ENSMUSG00000049612	ENSMUSG00000049612	ENSMUSG00000049612	11	79317547	79320214	205	234		
9782	17	26654917	26665217	ENSMUSG00000070354	ENSMUSG00000070354	ENSMUSG00000070354	11	79329580	79346754	205	234		
9783	17	26742768	26889352	ENSMUSG00000017639	ENSMUSG00000017639	ENSMUSG00000017639	11	79407407	79510207	205	234		
9784	17	27214303	27252842	ENSMUSG00000035575	ENSMUSG00000035575	ENSMUSG00000035575	11	79750151	79778582	205	234		
9785	17	27288185	27352162	ENSMUSG00000017548	ENSMUSG00000017548	ENSMUSG00000017548	11	79809301	79850318	205	234		
9786	17	26135331	26175795	ENSMUSG00000017561	ENSMUSG00000017561	ENSMUSG00000017561	11	79862694	79897152	205	235	here	This inversion was ignored by our tool.
9787	17	26183146	26246421	ENSMUSG00000017550	ENSMUSG00000017550	ENSMUSG00000017550	11	79905607	79951986	205	235	Inversion ignored by ST	1 -> 2
9788	17	26250127	26257379	ENSMUSG00000046909	ENSMUSG00000046909	ENSMUSG00000046909	11	79952877	79958314	205	235		
9789	17	26273059	26310335	ENSMUSG00000020709	ENSMUSG00000020709	ENSMUSG00000020709	11	79970357	79995035	205	235		
9790	17	26322218	26351051	ENSMUSG00000020707	ENSMUSG00000020707	ENSMUSG00000020707	11	80000067	80015948	205	235		
9791	17	27493586	27576859	ENSMUSG00000017686	ENSMUSG00000017686	ENSMUSG00000017686	11	80025314	80082958	206	236		
9792	17	27617308	27675793	ENSMUSG00000017692	ENSMUSG00000017692	ENSMUSG00000017692	11	80117271	80172150	206	236		
9793	17	27682505	27693302	ENSMUSG00000057181	ENSMUSG00000057181	ENSMUSG00000057181	11	80177650	80194229	206	236		
9794	17	27795615	27832154	ENSMUSG00000017428	ENSMUSG00000017428	ENSMUSG00000017428	11	80244860	80287789	206	236		
9795	17	27838214	27842383	ENSMUSG00000048895	ENSMUSG00000048895	ENSMUSG00000048895	11	80293218	80297379	206	236		
9796	17	27843741	28228015	ENSMUSG00000035441	ENSMUSG00000035441	ENSMUSG00000035441	11	80400022	80596220	206	236		
9797	17	28279041	28292778	ENSMUSG00000035413	ENSMUSG00000035413	ENSMUSG00000035413	11	80626610	80638228	206	236		
9798	17	28342995	28349005	ENSMUSG00000053184	ENSMUSG00000053184	ENSMUSG00000053184	11	80678288	80684007	206	236		
9799	17	28364221	28644119	ENSMUSG00000020704	ENSMUSG00000020704	ENSMUSG00000020704	11	80696375	80969098	206	236		
9800	17	29711512	29714365	ENSMUSG00000020702	ENSMUSG00000020702	ENSMUSG00000020702	11	81992854	81996007	206	236		
9801	17	29931881	29990450	ENSMUSG00000020701	ENSMUSG00000020701	ENSMUSG00000020701	11	82205095	82262522	206	236		
9802	17	30279053	30312619	ENSMUSG00000020698	ENSMUSG00000020698	ENSMUSG00000020698	11	82535445	82580501	206	236		
9803	17	30312689	30314316	ENSMUSG00000046010	ENSMUSG00000046010	ENSMUSG00000046010	11	82580546	82582985	206	236		
9804	17	30331651	30356201	ENSMUSG00000020697	ENSMUSG00000020697	ENSMUSG00000020697	11	82597330	82620469	206	236		
9805	17	30360244	30440407	ENSMUSG00000020696	ENSMUSG00000020696	ENSMUSG00000020696	11	82620017	82662043	206	236		
9806	17	30450929	30470948	ENSMUSG00000018841	ENSMUSG00000018841	ENSMUSG00000018841	11	82690902	82706784	206	236		
9807	17	30472744	30481863	ENSMUSG00000018844	ENSMUSG00000018844	ENSMUSG00000018844	11	82708319	82716996	206	236		
9808	17	30479885	30493383	ENSMUSG00000020692	ENSMUSG00000020692	ENSMUSG00000020692	11	82716963	82724606	206	236		
9809	17	30498949	30540473	ENSMUSG00000018845	ENSMUSG00000018845	ENSMUSG00000018845	11	82727875	82759598	206	236		
9810	17	30594203	30618521	ENSMUSG00000054404	ENSMUSG00000054404	ENSMUSG00000054404	11	82768297	82779136	206	236		
9811	17	30925928	30929695	ENSMUSG00000018733	ENSMUSG00000018733	ENSMUSG00000018733	11	83110840	83115172	206	236		
9812	17	30938395	31077547	ENSMUSG00000035152	ENSMUSG00000035152	ENSMUSG00000035152	11	83118892	83221234	206	236		
9813	17	31082792	31094652	ENSMUSG00000020684	ENSMUSG00000020684	ENSMUSG00000020684	11	83225883	83235025	206	236		
9814	17	31095955	31104010	ENSMUSG00000020686	ENSMUSG00000020686	ENSMUSG00000020686	11	83238097	83245650	206	236		
9815	17	31112046	31116210	ENSMUSG00000035085	ENSMUSG00000035085	ENSMUSG00000035085	11	83253889	83257427	206	236		
9816	17	31116989	31146753	ENSMUSG00000020682	ENSMUSG00000020682	ENSMUSG00000020682	11	83258071	83279156	206	236		
9817	17	31160601	31198350	ENSMUSG00000020680	ENSMUSG00000020680	ENSMUSG00000020680	11	83289303	83322935	206	236		
9818	17	31206073	31220008	ENSMUSG00000018925	ENSMUSG00000018925	ENSMUSG00000018925	11	83327957	83338231	206	236		
9819	17	31222613	31231490	ENSMUSG00000035042	ENSMUSG00000035042	ENSMUSG00000035042	11	83341978	83346713	206	236		
9820	17	55475344	55511064	ENSMUSG00000000976	ENSMUSG00000000976	ENSMUSG00000000976	11	83569909	83598216	Singleton			
9821	17	33120548	33179182	ENSMUSG00000020679	ENSMUSG00000020679	ENSMUSG00000020679	11	83667064	83722010	207	237		
9822	17	33046526	33077600	ENSMUSG00000020677	ENSMUSG00000020677	ENSMUSG00000020677	11	83758373	83778389	207	237		
9823	17	32952053	33043559	ENSMUSG00000034940	ENSMUSG00000034940	ENSMUSG00000034940	11	83780671	83858289	207	237		
9824	17	32924064	32947701	ENSMUSG00000018648	ENSMUSG00000018648	ENSMUSG00000018648	11	83864240	83884552	207	237		
9825	17	32841425	32911337	ENSMUSG00000018651	ENSMUSG00000018651	ENSMUSG00000018651	11	83895115	83943292	207	237		
9826	17	32807098	32823773	ENSMUSG00000051452	ENSMUSG00000051452	ENSMUSG00000051452	11	83964546	83983671	207	237		
9827	17	32380374	32488253	ENSMUSG00000018697	ENSMUSG00000018697	ENSMUSG00000018697	11	84239051	84329696	207	237		
9828	17	32368612	32374596	ENSMUSG00000018698	ENSMUSG00000018698	ENSMUSG00000018698	11	84334172	84340370	207	237		
9829	17	32032353	32039510	ENSMUSG00000018405	ENSMUSG00000018405	ENSMUSG00000018405	11	84629256	84635567	207	237		
9830	17	32022442	32031324	ENSMUSG00000034449	ENSMUSG00000034449	ENSMUSG00000034449	11	84636921	84645159	207	237		
9831	17	31974917	32020387	ENSMUSG00000020530	ENSMUSG00000020530	ENSMUSG00000020530	11	84648924	84686933	207	237		
9832	17	31965516	31969263	ENSMUSG00000045140	ENSMUSG00000045140	ENSMUSG00000045140	11	84692508	84696390	207	237		
9833	17	31925601	31965376	ENSMUSG00000020527	ENSMUSG00000020527	ENSMUSG00000020527	11	84698781	84727308	207	237		
9834	17	31916586	31925775	ENSMUSG00000020526	ENSMUSG00000020526	ENSMUSG00000020526	11	84727145	84732561	207	237		
9835	17	55582079	55591683	ENSMUSG00000000805	ENSMUSG00000000805	ENSMUSG00000000805	11	84774030	84782234	208	238		
9836	17	55609473	55824368	ENSMUSG00000000804	ENSMUSG00000000804	ENSMUSG00000000804	11	84800685	84920156	208	238		
9837	17	55854736	55863400	ENSMUSG00000018479	ENSMUSG00000018479	ENSMUSG00000018479	11	84987291	84997309	208	238		
9838	17	55875302	55958362	ENSMUSG00000018481	ENSMUSG00000018481	ENSMUSG00000018481	11	85007501	85051315	208	238		
9839	17	56032336	56096818	ENSMUSG00000020525	ENSMUSG00000020525	ENSMUSG00000020525	11	85127449	85163261	208	238		
9840	17	56832039	56841607	ENSMUSG00000000093	ENSMUSG00000000093	ENSMUSG00000000093	11	85649273	85657325	208	238		
9841	17	56888589	56916446	ENSMUSG00000000094	ENSMUSG00000000094	ENSMUSG00000000094	11	85706258	85730940	208	238		
9842	17	57114767	57295537	ENSMUSG00000034329	ENSMUSG00000034329	ENSMUSG00000034329	11	85874331	86017388	208	238		
9843	17	57297517	57365737	ENSMUSG00000018068	ENSMUSG00000018068	ENSMUSG00000018068	11	86026878	86073763	208	238		
9844	17	57377824	57497425	ENSMUSG00000034297	ENSMUSG00000034297	ENSMUSG00000034297	11	86087181	86173720	208	239		
9845	17	55384506	55396862	ENSMUSG00000020521	ENSMUSG00000020521	ENSMUSG00000020521	11	86300852	86315202	209	239		
9846	17	55325225	55382564	ENSMUSG00000020516	ENSMUSG00000020516	ENSMUSG00000020516	11	86318784	86360963	209	239		
9847	17	55291633	55325078	ENSMUSG00000020513	ENSMUSG00000020513	ENSMUSG00000020513	11	86361186	86383554	209	239		
9848	17	55139811	55273235	ENSMUSG00000018171	ENSMUSG00000018171	ENSMUSG00000018171	11	86400060	86500017	209	239		
9849	17	55052038	55127254	ENSMUSG00000047126	ENSMUSG00000047126	ENSMUSG00000047126	11	86510846	86573687	209	239		
9850	17	54997668	55040484	ENSMUSG00000018425	ENSMUSG00000018425	ENSMUSG00000018425	11	86585949	86623832	209	239		
9851	17	54763835	54833877	ENSMUSG00000018427	ENSMUSG00000018427	ENSMUSG00000018427	11	86756305	86809902	209	239		
9852	17	54652616	54706655	ENSMUSG00000061666	ENSMUSG00000061666	ENSMUSG00000061666	11	86850083	86890251	209	239		
9853	17	54642180	54647390	ENSMUSG00000020495	ENSMUSG00000020495	ENSMUSG00000020495	11	86893925	86902972	209	239		
9854	17	54587900	54635977	ENSMUSG00000020493	ENSMUSG00000020493	ENSMUSG00000020493	11	86905609	86924884	209	239		
9855	17	54430351	54539011	ENSMUSG00000018548	ENSMUSG00000018548	ENSMUSG00000018548	11	86943272	87036877	209	239		
9856	17	54188231	54417314	ENSMUSG00000046442	ENSMUSG00000046442	ENSMUSG00000046442	11	87043101	87175189	209	239		
9857	17	54124962	54166689	ENSMUSG00000007646	ENSMUSG00000007646	ENSMUSG00000007646	11	87194752	87221149	209	239		
9858	17	53989047	54124383	ENSMUSG00000010342	ENSMUSG00000010342	ENSMUSG00000010342	11	87250005	87372018	209	239		
9859	17	53973947	53976682	ENSMUSG00000020486	ENSMUSG00000020486	ENSMUSG00000020486	11	87382997	87385233	209	239		
9860	17	53921897	53950211	ENSMUSG00000018401	ENSMUSG00000018401	ENSMUSG00000018401	11	87408412	87432497	209	239		
9861	17	53852530	53920744	ENSMUSG00000070345	ENSMUSG00000070345	ENSMUSG00000070345	11	87433467	87473562	209	239		
9862	17	53784860	53847937	ENSMUSG00000034177	ENSMUSG00000034177	ENSMUSG00000034177	11	87480811	87551733	209	239		
9863	17	53733597	53760477	ENSMUSG00000034156	ENSMUSG00000034156	ENSMUSG00000034156	11	87577597	87602122	209	239		
9864	17	53702201	53713281	ENSMUSG00000009350	ENSMUSG00000009350	ENSMUSG00000009350	11	87609776	87620592	209	239		
9865	17	53675340	53700354	ENSMUSG00000009356	ENSMUSG00000009356	ENSMUSG00000009356	11	87622623	87642308	209	239		
9866	17	53637802	53648606	ENSMUSG00000034121	ENSMUSG00000034121	ENSMUSG00000034121	11	87669445	87679220	209	239		
9867	17	53625088	53636783	ENSMUSG00000052234	ENSMUSG00000052234	ENSMUSG00000052234	11	87680196	87691726	209	239		
9868	17	53587688	53588446	ENSMUSG00000060787	ENSMUSG00000060787	ENSMUSG00000060787	11	87730158	87731099	209	239		
9869	17	53515805	53521810	ENSMUSG00000020483	ENSMUSG00000020483	ENSMUSG00000020483	11	87795728	87803700	209	239		
9870	17	53403909	53420614	ENSMUSG00000018377	ENSMUSG00000018377	ENSMUSG00000018377	11	87884596	87900918	209	239		
9871	17	53295336	53387683	ENSMUSG00000018378	ENSMUSG00000018378	ENSMUSG00000018378	11	87915444	88009126	209	239		
9872	17	53271847	53282407	ENSMUSG00000023723	ENSMUSG00000023723	ENSMUSG00000023723	11	88020613	88027702	209	239		
9873	17	53177171	53177632	ENSMUSG00000034031	ENSMUSG00000034031	ENSMUSG00000034031	11	88110253	88110889	209	239		
9874	17	52688930	53112298	ENSMUSG00000069769	ENSMUSG00000069769	ENSMUSG00000069769	11	88155655	88534319	209	239		
9875	17	52517620	52553608	ENSMUSG00000018428	ENSMUSG00000018428	ENSMUSG00000018428	11	88646885	88680674	209	239		
9876	17	52410488	52439123	ENSMUSG00000000278	ENSMUSG00000000278	ENSMUSG00000000278	11	88740110	88771522	209	239		
9877	17	52370562	52393410	ENSMUSG00000033983	ENSMUSG00000033983	ENSMUSG00000033983	11	88790039	88807703	209	239		
9878	17	52320269	52346408	ENSMUSG00000000275	ENSMUSG00000000275	ENSMUSG00000000275	11	88815570	88836383	209	239		
9879	17	52266552	52295922	ENSMUSG00000000276	ENSMUSG00000000276	ENSMUSG00000000276	11	88854224	88876635	209	239		
9880	17	52026274	52027542	ENSMUSG00000048616	ENSMUSG00000048616	ENSMUSG00000048616	11	89117412	89118110	209	239		
9881	17	51585841	51915006	ENSMUSG00000047773	ENSMUSG00000047773	ENSMUSG00000047773	11	89237176	89354643	209	239		
9882	17	51183413	51209733	ENSMUSG00000020553	ENSMUSG00000020553	ENSMUSG00000020553	11	89800687	89818955	209	239		
9883	17	51152278	51164444	ENSMUSG00000069763	ENSMUSG00000069763	ENSMUSG00000069763	11	89846438	89852595	209	239		
9884	17	50824973	50854340	ENSMUSG00000003948	ENSMUSG00000003948	ENSMUSG00000003948	11	90065566	90094663	209	239		
9885	17	50697370	50755863	ENSMUSG00000003949	ENSMUSG00000003949	ENSMUSG00000003949	11	90154655	90206985	209	239		
9886	17	50401125	50596448	ENSMUSG00000020546	ENSMUSG00000020546	ENSMUSG00000020546	11	90296007	90454174	209	239		
9887	17	50333203	50394309	ENSMUSG00000020541	ENSMUSG00000020541	ENSMUSG00000020541	11	90459553	90503669	209	239		
9888	17	49255261	49257572	ENSMUSG00000046755	ENSMUSG00000046755	ENSMUSG00000046755	11	91391539	91393545	209	239		
9889	17	47062673	47567316	ENSMUSG00000056158	ENSMUSG00000056158	ENSMUSG00000056158	11	92914500	93417363	209	239		
9890	17	46692945	46730289	ENSMUSG00000054079	ENSMUSG00000054079	ENSMUSG00000054079	11	93675333	93701856	209	239		
9891	17	46612939	46657521	ENSMUSG00000059474	ENSMUSG00000059474	ENSMUSG00000059474	11	93702307	93759983	209	239		
9892	17	46397988	46553094	ENSMUSG00000020859	ENSMUSG00000020859	ENSMUSG00000020859	11	93812261	93938989	209	239		
9893	17	38404294	38408482	ENSMUSG00000069756	ENSMUSG00000069756	ENSMUSG00000069756	11	93853308	93853718	out-of-place		here	Presence of a singleton made AutoGRAPH to break the block
9894	17	46295340	46296377	ENSMUSG00000037573	ENSMUSG00000037573	ENSMUSG00000037573	11	94027544	94031585	209	240	presence of singletons	1 -> 2
9895	17	46267770	46274706	ENSMUSG00000044177	ENSMUSG00000044177	ENSMUSG00000044177	11	94053688	94058639	209	240		
9896	17	46151963	46185069	ENSMUSG00000020863	ENSMUSG00000020863	ENSMUSG00000020863	11	94107227	94138001	209	240		
9897	17	46125552	46140040	ENSMUSG00000020864	ENSMUSG00000020864	ENSMUSG00000020864	11	94144344	94155599	209	240		
9898	17	46067227	46124061	ENSMUSG00000020865	ENSMUSG00000020865	ENSMUSG00000020865	11	94159385	94209066	209	240		
9899	17	45993820	46059430	ENSMUSG00000020866	ENSMUSG00000020866	ENSMUSG00000020866	11	94224606	94289707	209	240		
9900	17	45979519	45988210	ENSMUSG00000020867	ENSMUSG00000020867	ENSMUSG00000020867	11	94294994	94301400	209	240		
9901	17	45965098	45975160	ENSMUSG00000010080	ENSMUSG00000010080	ENSMUSG00000010080	11	94307081	94316013	209	240		
9902	17	45940943	45963857	ENSMUSG00000039110	ENSMUSG00000039110	ENSMUSG00000039110	11	94317438	94337535	209	240		
9903	17	45911189	45918335	ENSMUSG00000039096	ENSMUSG00000039096	ENSMUSG00000039096	11	94355888	94365289	209	240		
9904	17	45896860	45901322	ENSMUSG00000039084	ENSMUSG00000039084	ENSMUSG00000039084	11	94381157	94385217	209	240		
9905	17	45813598	45957115	ENSMUSG00000020869	ENSMUSG00000020869	ENSMUSG00000020869	11	94445888	94461303	209	240		
9906	17	45805589	45813817	ENSMUSG00000039055	ENSMUSG00000039055	ENSMUSG00000039055	11	94461092	94469844	209	240		
9907	17	45778392	45793511	ENSMUSG00000020868	ENSMUSG00000020868	ENSMUSG00000020868	11	94479889	94493550	209	240		
9908	17	45616456	45633992	ENSMUSG00000001506	ENSMUSG00000001506	ENSMUSG00000001506	11	94752360	94767959	209	240		
9909	17	45598401	45608291	ENSMUSG00000001508	ENSMUSG00000001508	ENSMUSG00000001508	11	94779298	94789528	209	240		
9910	17	45603873	45604836	ENSMUSG00000038994	ENSMUSG00000038994	ENSMUSG00000038994	11	94783688	94784544	209	240		
9911	17	45567695	45582873	ENSMUSG00000038976	ENSMUSG00000038976	ENSMUSG00000038976	11	94807302	94822974	209	240		
9912	17	45545035	45562166	ENSMUSG00000047181	ENSMUSG00000047181	ENSMUSG00000047181	11	94825969	94842171	209	240		
9913	17	45527695	45543419	ENSMUSG00000038967	ENSMUSG00000038967	ENSMUSG00000038967	11	94843272	94857346	209	240		
9914	17	45488431	45522843	ENSMUSG00000001507	ENSMUSG00000001507	ENSMUSG00000001507	11	94860572	94892804	209	240		
9915	17	45422388	45427587	ENSMUSG00000001510	ENSMUSG00000001510	ENSMUSG00000001510	11	94936412	94941360	209	240		
9916	17	45404678	45406306	ENSMUSG00000020871	ENSMUSG00000020871	ENSMUSG00000020871	11	94956546	94962353	209	240		
9917	17	45270670	45280378	ENSMUSG00000020872	ENSMUSG00000020872	ENSMUSG00000020872	11	95077619	95085352	209	240		
9918	17	45221057	45261455	ENSMUSG00000038909	ENSMUSG00000038909	ENSMUSG00000038909	11	95091876	95122304	209	240		
9919	17	45142688	45196518	ENSMUSG00000038893	ENSMUSG00000038893	ENSMUSG00000038893	11	95153108	95197960	209	240		
9920	17	45031247	45110524	ENSMUSG00000057522	ENSMUSG00000057522	ENSMUSG00000057522	11	95230204	95309103	209	240		
9921	17	45008219	45012718	ENSMUSG00000046719	ENSMUSG00000046719	ENSMUSG00000046719	11	95325940	95330361	209	240		
9922	17	44927654	44947356	ENSMUSG00000000120	ENSMUSG00000000120	ENSMUSG00000000120	11	95384908	95403788	209	240		
9923	17	44727485	44794834	ENSMUSG00000075595	ENSMUSG00000075595	ENSMUSG00000075595	11	95565157	95567036	209	240		
9924	17	44656607	44663108	ENSMUSG00000050860	ENSMUSG00000050860	ENSMUSG00000050860	11	95640590	95647399	209	240		
9925	17	44642588	44655586	ENSMUSG00000018381	ENSMUSG00000018381	ENSMUSG00000018381	11	95646162	95658250	209	240		
9926	17	44638596	44641742	ENSMUSG00000038811	ENSMUSG00000038811	ENSMUSG00000038811	11	95658760	95661819	209	240		
9927	17	44565328	44602235	ENSMUSG00000013418	ENSMUSG00000013418	ENSMUSG00000013418	11	95682033	95730981	209	240		
9928	17	44429773	44488506	ENSMUSG00000013415	ENSMUSG00000013415	ENSMUSG00000013415	11	95778822	95822002	209	240		
9929	17	44390917	44400954	ENSMUSG00000014351	ENSMUSG00000014351	ENSMUSG00000014351	11	95840635	95846921	209	240		
9930	17	44362460	44377153	ENSMUSG00000006058	ENSMUSG00000006058	ENSMUSG00000006058	11	95851007	95863495	209	240		
9931	17	44340884	44359492	ENSMUSG00000014349	ENSMUSG00000014349	ENSMUSG00000014349	11	95863520	95881451	209	240		
9932	17	44194602	44226685	ENSMUSG00000038756	ENSMUSG00000038756	ENSMUSG00000038756	11	95950309	95981541	209	240		
9933	17	44157132	44161119	ENSMUSG00000049604	ENSMUSG00000049604	ENSMUSG00000049604	11	96010473	96013535	209	240		
9934	17	44053525	44058630	ENSMUSG00000020875	ENSMUSG00000020875	ENSMUSG00000020875	11	96087599	96091983	209	240		
9935	17	44044707	44047300	ENSMUSG00000056648	ENSMUSG00000056648	ENSMUSG00000056648	11	96099053	96100560	209	240		
9936	17	44039596	44043378	ENSMUSG00000038721	ENSMUSG00000038721	ENSMUSG00000038721	11	96102736	96106253	209	240		
9937	17	44028113	44037333	ENSMUSG00000000690	ENSMUSG00000000690	ENSMUSG00000000690	11	96115164	96117656	209	240		
9938	17	44023619	44026322	ENSMUSG00000038700	ENSMUSG00000038700	ENSMUSG00000038700	11	96119602	96122210	209	240		
9939	17	44007875	44010742	ENSMUSG00000038692	ENSMUSG00000038692	ENSMUSG00000038692	11	96134357	96136703	209	240		
9940	17	43982246	44006848	ENSMUSG00000048763	ENSMUSG00000048763	ENSMUSG00000048763	11	96159859	96162597	209	240		
9941	17	43975020	43977391	ENSMUSG00000075588	ENSMUSG00000075588	ENSMUSG00000075588	11	96167901	96169716	209	240		
9942	17	43961813	43963271	ENSMUSG00000018973	ENSMUSG00000018973	ENSMUSG00000018973	11	96181848	96183766	209	240		

9943	17	43565804	43862551	ENSMUSG00000057058	ENSMUSG00000057058	ENSMUSG00000057058	11	96280683	96575031	209	240		
9944	17	43539910	43555104	ENSMUSG00000020876	ENSMUSG00000020876	ENSMUSG00000020876	11	96583645	96593621	209	240		
9945	17	43502414	43533806	ENSMUSG00000018666	ENSMUSG00000018666	ENSMUSG00000018666	11	96605249	96624730	209	240		
9946	17	43480720	43493836	ENSMUSG00000038615	ENSMUSG00000038615	ENSMUSG00000038615	11	96633522	96645995	209	240		
9947	17	43458535	43470138	ENSMUSG00000018672	ENSMUSG00000018672	ENSMUSG00000018672	11	96665966	96677295	209	240		
9948	17	43403521	43414099	ENSMUSG00000018669	ENSMUSG00000018669	ENSMUSG00000018669	11	96723516	96732586	209	240		
9949	17	43384335	43390109	ENSMUSG00000047040	ENSMUSG00000047040	ENSMUSG00000047040	11	96745559	96753922	209	240		
9950	17	43373936	43380653	ENSMUSG00000018659	ENSMUSG00000018659	ENSMUSG00000018659	11	96753920	96760076	209	240		
9951	17	43347691	43361320	ENSMUSG00000018678	ENSMUSG00000018678	ENSMUSG00000018678	11	96770309	96779541	209	240		
9952	17	43277279	43288239	ENSMUSG00000038560	ENSMUSG00000038560	ENSMUSG00000038560	11	96829659	96840824	209	240		
9953	17	43270209	43273641	ENSMUSG00000020877	ENSMUSG00000020877	ENSMUSG00000020877	11	96846506	96849914	209	240		
9954	17	43264099	43269689	ENSMUSG00000020878	ENSMUSG00000020878	ENSMUSG00000020878	11	96850692	96857459	209	240		
9955	17	43255638	43261585	ENSMUSG00000001445	ENSMUSG00000001445	ENSMUSG00000001445	11	96857676	96865303	209	240		
9956	17	43239738	43254199	ENSMUSG00000038534	ENSMUSG00000038534	ENSMUSG00000038534	11	96868392	96872252	209	240		
9957	17	43165609	43178484	ENSMUSG00000001444	ENSMUSG00000001444	ENSMUSG00000001444	11	96914097	96931418	209	240		
9958	17	43127629	43144415	ENSMUSG00000038517	ENSMUSG00000038517	ENSMUSG00000038517	11	96952261	96965676	209	240		
9959	17	43082274	43116003	ENSMUSG00000001440	ENSMUSG00000001440	ENSMUSG00000001440	11	96975800	97003982	209	240		
9960	17	42963603	43055634	ENSMUSG00000001441	ENSMUSG00000001441	ENSMUSG00000001441	11	97021944	97096666	209	240		
9961	17	33706517	33732627	ENSMUSG00000018882	ENSMUSG00000018882	ENSMUSG00000018882	11	97131946	97146010	210	241		
9962	17	33735019	33753219	ENSMUSG00000070337	ENSMUSG00000070337	ENSMUSG00000070337	11	97152363	97168106	210	241		
9963	17	33761531	33809541	ENSMUSG00000038485	ENSMUSG00000038485	ENSMUSG00000038485	11	97178641	97214632	210	241		
9964	17	33919745	33920734	ENSMUSG00000049807	ENSMUSG00000049807	ENSMUSG00000049807	11	97282114	97318490	210	241		
9965	17	33941350	33988592	ENSMUSG00000038453	ENSMUSG00000038453	ENSMUSG00000038453	11	97327759	97390009	210	241		
9966	17	34083135	34084274	ENSMUSG00000043439	ENSMUSG00000043439	ENSMUSG00000043439	11	97444262	97445371	210	241		
9967	17	34115401	34136964	ENSMUSG00000038437	ENSMUSG00000038437	ENSMUSG00000038437	11	97479567	97498621	210	241		
9968	17	34145002	34150181	ENSMUSG00000018537	ENSMUSG00000018537	ENSMUSG00000018537	11	97505910	97516587	210	241		
9969	17	34177324	34209684	ENSMUSG00000018547	ENSMUSG00000018547	ENSMUSG00000018547	11	97531247	97560794	210	241		
9970	17	34210973	34242007	ENSMUSG00000018541	ENSMUSG00000018541	ENSMUSG00000018541	11	97561705	97582646	210	241		
9971	17	34346244	34377181	ENSMUSG00000070336	ENSMUSG00000070336	ENSMUSG00000070336	11	97669916	97671642	210	241		
9972	17	34473083	34561298	ENSMUSG00000017417	ENSMUSG00000017417	ENSMUSG00000017417	11	97739328	97802470	210	241		
9973	17	34566673	34575539	ENSMUSG00000038352	ENSMUSG00000038352	ENSMUSG00000038352	11	97805668	97812263	210	241		
9974	17	34583232	34607427	ENSMUSG00000020882	ENSMUSG00000020882	ENSMUSG00000020882	11	97817598	97838724	210	241		
9975	17	34610990	34614506	ENSMUSG00000017404	ENSMUSG00000017404	ENSMUSG00000017404	11	97843048	97846582	210	241		
9976	17	34620316	34635500	ENSMUSG00000017400	ENSMUSG00000017400	ENSMUSG00000017400	11	97852714	97869552	210	241		
9977	17	34670374	34811424	ENSMUSG00000020883	ENSMUSG00000020883	ENSMUSG00000020883	11	97904787	97965702	210	241		
9978	17	34816380	34861030	ENSMUSG00000018160	ENSMUSG00000018160	ENSMUSG00000018160	11	97970338	98008996	210	241		
9979	17	34871818	34942044	ENSMUSG00000003119	ENSMUSG00000003119	ENSMUSG00000003119	11	98019404	98062796	210	241		
9980	17	35014577	35017699	ENSMUSG00000038255	ENSMUSG00000038255	ENSMUSG00000038255	11	98142680	98145738	210	241		
9981	17	35036705	35046403	ENSMUSG00000061718	ENSMUSG00000061718	ENSMUSG00000061718	11	98164828	98173885	210	241		
9982	17	35046844	35073214	ENSMUSG00000018167	ENSMUSG00000018167	ENSMUSG00000018167	11	98174474	98197189	210	241		
9983	17	35073966	35076326	ENSMUSG00000007877	ENSMUSG00000007877	ENSMUSG00000007877	11	98199901	98201302	210	241		
9984	17	35078033	35080254	ENSMUSG00000038216	ENSMUSG00000038216	ENSMUSG00000038216	11	98202722	98204187	210	241		
9985	17	35080902	35097836	ENSMUSG00000038208	ENSMUSG00000038208	ENSMUSG00000038208	11	98204768	98216503	210	241		
9986	17	35109780	35138441	ENSMUSG00000062312	ENSMUSG00000062312	ENSMUSG00000062312	11	98228574	98253806	210	241		
9987	17	35138939	35140566	ENSMUSG00000002580	ENSMUSG00000002580	ENSMUSG00000002580	11	98253813	98255062	210	241		
9988	17	35147713	35157061	ENSMUSG00000019312	ENSMUSG00000019312	ENSMUSG00000019312	11	98262929	98271460	210	241		
9989	17	35174724	35273967	ENSMUSG00000018168	ENSMUSG00000018168	ENSMUSG00000018168	11	98280992	98361890	210	241		
9990	17	35428881	35464175	ENSMUSG00000017210	ENSMUSG00000017210	ENSMUSG00000017210	11	98352628	98545004	210	241		
9991	17	35277998	35286861	ENSMUSG00000017195	ENSMUSG00000017195	ENSMUSG00000017195	11	98367242	98374746	210	241		
9992	17	35353259	35354471	ENSMUSG00000069726	ENSMUSG00000069726	ENSMUSG00000069726	11	98414981	98415631	210	241		
9993	17	35390750	35407738	ENSMUSG00000017221	ENSMUSG00000017221	ENSMUSG00000017221	11	98498644	98512068	210	241		
9994	17	35425214	35427592	ENSMUSG00000038067	ENSMUSG00000038067	ENSMUSG00000038067	11	98517403	98519719	210	241		
9995	17	35472686	35503644	ENSMUSG00000058756	ENSMUSG00000058756	ENSMUSG00000058756	11	98557991	98585094	210	241		
9996	17	35502567	35510499	ENSMUSG00000020889	ENSMUSG00000020889	ENSMUSG00000020889	11	98584238	98591322	210	241		
9997	17	35550110	35581950	ENSMUSG00000052915	ENSMUSG00000052915	ENSMUSG00000052915	11	98620995	98649897	210	241		
9998	17	35588133	35605426	ENSMUSG00000038020	ENSMUSG00000038020	ENSMUSG00000038020	11	98656394	98669093	210	241		
9999	17	35629100	35691965	ENSMUSG00000038013	ENSMUSG00000038013	ENSMUSG00000038013	11	98679899	98719610	210	241		
10000	17	35697672	35712939	ENSMUSG00000017499	ENSMUSG00000017499	ENSMUSG00000017499	11	98724241	98740032	210	241		
10001	17	35751868	35767420	ENSMUSG00000037992	ENSMUSG00000037992	ENSMUSG00000037992	11	98753808	98791028	210	241		
10002	17	35770761	35773593	ENSMUSG00000047197	ENSMUSG00000047197	ENSMUSG00000047197	11	98798270	98799106	210	241		
10003	17	35798321	35827695	ENSMUSG00000020914	ENSMUSG00000020914	ENSMUSG00000020914	11	98809033	98840279	210	241		
10004	17	35853202	35867507	ENSMUSG00000017493	ENSMUSG00000017493	ENSMUSG00000017493	11	98857334	98868725	210	241		
10005	17	35885639	35911341	ENSMUSG00000017607	ENSMUSG00000017607	ENSMUSG00000017607	11	98881769	98905392	210	241		
10006	17	35963550	35975250	ENSMUSG00000037944	ENSMUSG00000037944	ENSMUSG00000037944	11	98961048	98965187	210	241		
10007	17	36107771	36113528	ENSMUSG00000020913	ENSMUSG00000020913	ENSMUSG00000020913	11	99096273	99101352	210	241		
10008	17	36157800	36165091	ENSMUSG00000035831	ENSMUSG00000035831	ENSMUSG00000035831	11	99131936	99139041	210	241		
10009	17	36176018	36181937	ENSMUSG00000075570	ENSMUSG00000075570	ENSMUSG00000075570	11	99144572	99154057	210	241		
10010	17	36186587	36192312	ENSMUSG00000017588	ENSMUSG00000017588	ENSMUSG00000017588	11	99161656	99167194	210	241		
10011	17	36201981	36209737	ENSMUSG00000055937	ENSMUSG00000055937	ENSMUSG00000055937	11	99180962	99190993	210	241		
10012	17	36227896	36232373	ENSMUSG00000019761	ENSMUSG00000019761	ENSMUSG00000019761	11	99201344	99205454	210	241		
10013	17	36271081	36276988	ENSMUSG00000020912	ENSMUSG00000020912	ENSMUSG00000020912	11	99231756	99238346	210	241		
10014	17	36285721	36295005	ENSMUSG00000035775	ENSMUSG00000035775	ENSMUSG00000035775	11	99244493	99254243	210	241		
10015	17	36332478	36347362	ENSMUSG00000006777	ENSMUSG00000006777	ENSMUSG00000006777	11	99294064	99309214	210	241		
10016	17	36368195	36376670	ENSMUSG00000064165	ENSMUSG00000064165	ENSMUSG00000064165	11	99330714	99337348	210	241		
10017	17	36387494	36394056	ENSMUSG00000059169	ENSMUSG00000059169	ENSMUSG00000059169	11	99353575	99359248	210	241		
10018	17	36418556	36418852	ENSMUSG00000047564	ENSMUSG00000047564	ENSMUSG00000047564	11	99382377	99382673	210	241		
10019	17	36869588	36877164	ENSMUSG00000046095	ENSMUSG00000046095	ENSMUSG00000046095	11	99896947	99904205	210	241		
10020	17	36886467	36891194	ENSMUSG00000048013	ENSMUSG00000048013	ENSMUSG00000048013	11	99908282	99912276	210	241		
10021	17	36910762	36915391	ENSMUSG00000044041	ENSMUSG00000044041	ENSMUSG00000044041	11	99933420	99937656	210	241		
10022	17	36923524	36932191	ENSMUSG00000054146	ENSMUSG00000054146	ENSMUSG00000054146	11	99948107	99952018	210	241		
10023	17	36933396	36938160	ENSMUSG00000020911	ENSMUSG00000020911	ENSMUSG00000020911	11	99956900	99962009	210	241		
10024	17	36975622	36981836	ENSMUSG00000051617	ENSMUSG00000051617	ENSMUSG00000051617	11	100002871	100009336	210	241		
10025	17	36992070	36996699	ENSMUSG00000045545	ENSMUSG00000045545	ENSMUSG00000045545	11	100019252	100023600	210	241		
10026	17	37019559	37022545	ENSMUSG00000053797	ENSMUSG00000053797	ENSMUSG00000053797	11	100062181	100064992	210	241		
10027	17	37122139	37125746	ENSMUSG00000017165	ENSMUSG00000017165	ENSMUSG00000017165	11	100150497	100153086	210	241		
10028	17	37132418	37144422	ENSMUSG00000006930	ENSMUSG00000006930	ENSMUSG00000006930	11	100163417	100172218	210	241		
10029	17	37164412	37196476	ENSMUSG00000001552	ENSMUSG00000001552	ENSMUSG00000001552	11	100186709	100213829	210	241		
10030	17	37212073	37222381	ENSMUSG00000006931	ENSMUSG00000006931	ENSMUSG00000006931	11	100224546	100230787	210	241		
10031	17	37222727	37232995	ENSMUSG00000001555	ENSMUSG00000001555	ENSMUSG00000001555	11	100231787	100240915	210	241		
10032	17	37234998	37246017	ENSMUSG00000017176	ENSMUSG00000017176	ENSMUSG00000017176	11	100245449	100257145	210	241		
10033	17	37247587	37258139	ENSMUSG00000001558	ENSMUSG00000001558	ENSMUSG00000001558	11	100258014	100273114	210	241		
10034	17	37263325	37275176	ENSMUSG00000048732	ENSMUSG00000048732	ENSMUSG00000048732	11	100278702	100288872	210	241		
10035	17	37276710	37328798	ENSMUSG00000020917	ENSMUSG00000020917	ENSMUSG00000020917	11	100292448	100339688	210	241		
10036	17	37340424	37371174	ENSMUSG00000006784	ENSMUSG00000006784	ENSMUSG00000006784	11	100361792	100388050	210	241		
10037	17	37372285	37383280	ENSMUSG00000006782	ENSMUSG00000006782	ENSMUSG00000006782	11	100390994	100397818	210	241		
10038	17	37381983	37423197	ENSMUSG00000014195	ENSMUSG00000014195	ENSMUSG00000014195	11	100398921	100436258	210	241		
10039	17	37425641	37429437	ENSMUSG00000017837	ENSMUSG00000017837	ENSMUSG00000017837	11	100436374	100442506	210	241		
10040	17	37431122	37434233	ENSMUSG00000014198	ENSMUSG00000014198	ENSMUSG00000014198	11	100443633	100466783	210	241		
10041	17	37506979	37518277	ENSMUSG00000017830	ENSMUSG00000017830	ENSMUSG00000017830	11	100510974	100520361	210	241		
10042	17	37518655	37526901	ENSMUSG00000020918	ENSMUSG00000020918	ENSMUSG00000020918	11	100520836	100528555	210	241		
10043	17	37526975	37528895	ENSMUSG00000017832	ENSMUSG00000017832	ENSMUSG00000017832	11	100530019	100530665	210	241		
10044	17	37530526	37560557	ENSMUSG00000019173	ENSMUSG00000019173	ENSMUSG00000019173	11	100531101	100554280	210	241		
10045	17	37562439	37586822	ENSMUSG00000035355	ENSMUSG00000035355	ENSMUSG00000035355	11	100561701	100575695	210	241		
10046	17	37589604	37590996	ENSMUSG00000045471	ENSMUSG00000045471	ENSMUSG00000045471	11	100577783	100579021	210	241		
10047	17	37594632	37599125	ENSMUSG00000017747	ENSMUSG00000017747	ENSMUSG00000017747	11	100582422	100587047	210	241		
10048	17	37604722	37681950	ENSMUSG00000020919	ENSMUSG00000020919	ENSMUSG00000020919	11	100596902	100666816	210	241		
10049	17	37693091	37717484	ENSMUSG00000004043	ENSMUSG00000004043	ENSMUSG00000004043	11	100675493	100701259	210	241		
10050	17	37718869	37794039	ENSMUSG00000004040	ENSMUSG00000004040	ENSMUSG00000004040	11	100702900	100755601	210	241		
10051	17	37809180	37828641	ENSMUSG00000004044	ENSMUSG00000004044	ENSMUSG00000004044	11	100772826	100786707	210	241		
10052	17	37864388	37928122	ENSMUSG00000019302	ENSMUSG00000019302	ENSMUSG00000019302	11	100827893	100878514	210	241		
10053	17	37941477	37949990	ENSMUSG00000001751	ENSMUSG00000001751	ENSMUSG00000001751	11	100886210	100893759	210	241		
10054	17	37967663	37971821	ENSMUSG00000001755	ENSMUSG00000001755	ENSMUSG00000001755	11	100898696	100902707	210	241		
10055	17	37972616	37978783	ENSMUSG00000017801	ENSMUSG00000017801	ENSMUSG00000017801	11	100903400	100908297	210	241		
10056	17	37977881	37983375	ENSMUSG00000019303	ENSMUSG00000019303	ENSMUSG00000019303	11	100908247	100911493	210	241		
10057	17	37985066	38014928	ENSMUSG00000017802	ENSMUSG00000017802	ENSMUSG00000017802	11	100912620	100935929	210	241		
10058	17	38015220	38020773	ENSMUSG00000035198	ENSMUSG00000035198	ENSMUSG00000035198	11	100936221	100942509	210	241		
10059	17	38064824	38072523	ENSMUSG00000045007	ENSMUSG00000045007	ENSMUSG00000045007	11	100972003	100977877	210	241		
10060	17	38073463	38082495	ENSMUSG00000035172	ENSMUSG00000035172	ENSMUSG00000035172	11	100978769	100987392	210	241		
10061	17	38084962	38087371	ENSMUSG00000044052	ENSMUSG00000044052	ENSMUSG00000044052	11	100989088	100991533	210	241		
10062	17	38088158	38105358	ENSMUSG00000017167	ENSMUSG00000017167	ENSMUSG00000017167	11	100992207	101005937	210	241		
10063	17	38105820	38150574	ENSMUSG00000006920	ENSMUSG00000006920	ENSMUSG00000006920	11	101009029	101033455	210	241		
10064	17	38166731	38168585	ENSMUSG00000001240	ENSMUSG00000001240	ENSMUSG00000001240	11	101062424	101064340	210	241		
10065	17	38186222	38202587	ENSMUSG00000035112	ENSMUSG00000035112	ENSMUSG00000035112	11	101069788	101103647	210	241		
10066	17	38203178	38204269	ENSMUSG00000017188	ENSMUSG00000017188	ENSMUSG00000017188	11	101094060	101095038	210	241		
10067	17	38215681	38229816	ENSMUSG00000035086	ENSMUSG00000035086	ENSMUSG00000035086	11	101102042	101118357	210	241		
10068	17	38238949	38249301	ENSMUSG00000019326	ENSMUSG00000019326	ENSMUSG00000019326	11	101132537	101192826	210	241		
10069	17	38356097	38386033	ENSMUSG00000057537	ENSMUSG00000057537	ENSMUSG00000057537	11	101222927	101233523	210	241		
10070	17	38386108	38399233	ENSMUSG00000035007	ENSMUSG00000035007	ENSMUSG00000035007	11	101241175	101251550	210	241		
10071	17	38412351	38419998	ENSMUSG00000010358	ENSMUSG00000010358	ENSMUSG00000010358	11	101264571	101274552	210	241		
10072	17	38420150	38427865	ENSMUSG00000034993	ENSMUSG00000034993	ENSMUSG00000034993	11	101274838	101282289	210	241		
10073	17	38430791	38434767	ENSMUSG00000001313	ENSMUSG00000001313	ENSMUSG00000001313	11	101284428	101287396	210	241		
10074	17	38449840	38530994	ENSMUSG00000017146	ENSMUSG00000017146	ENSMUSG00000017146	11	101305657	101367902	210	241		
10075	17	38581343	38717616	ENSMUSG00000017119	ENSMUSG00000017119	ENSMUSG00000017119	11	101368239	101398041	210	241		
10076	17	38719420	38727115	ENSMUSG00000034947	ENSMUSG00000034947	ENSMUSG00000034947	11	101398359	101406886	210	241		
10077	17	31269197	31281893	ENSMUSG00000010362	ENSMUSG00000010362	ENSMUSG00000010362	11	101444039	101452171	Singleton			
10078	17	38831879	38834029	ENSMUSG00000034936	ENSMUSG00000034936	ENSMUSG00000034936	11	101481651	101483919	210	242	here	Presence of a singleton made AutoGRAPH to break the block
10079	17	38916860	38957206	ENSMUSG00000034931	ENSMUSG00000034931	ENSMUSG00000034931	11	101549046	101583451	210	242	presence of singletons	1 -> 2
10080	17	38960738	38979230	ENSMUSG00000017724	ENSMUSG00000017724	ENSMUSG00000017724	11	101585841	101601353	210	242		
10081	17	39073296	39094457	ENSMUSG00000001493	ENSMUSG00000001493	ENSMUSG00000001493	11	101693600	101710444	210	242		
10082	17	39186629	39191682	ENSMUSG00000001494	ENSMUSG00000001494	ENSMUSG00000001494	11	101778626	101783105	210	242		
10083	17	39199016	39211872	ENSMUSG00000003518	ENSMUSG00000003518	ENSMUSG00000003518	11	101790249	101800860	210	242		
10084	17	39213329	39217721	ENSMUSG00000010841	ENSMUSG00000010841	ENSMUSG00000010841	11	101803204	101807602	210	242		
10085	17	39234315	39266064	ENSMUSG00000052373	ENSMUSG00000052373	ENSMUSG00000052373	11	101815752	101842995	210	242		
10086	17	39280070	39296520	ENSMUSG00000017309	ENSMUSG00000017309	ENSMUSG00000017309	11	101857638	101871707	210	242		
10087	17	39308253	39340639	ENSMUSG00000017314	ENSMUSG00000017314	ENSMUSG00000017314	11	101873109	101898500	210	242		
10088	17	39373698	39375359	ENSMUSG00000017316	ENSMUSG00000017316	ENSMUSG00000017316	11	101916262	101916801	210	242		
10089	17	39437558	39441959	ENSMUSG00000048217	ENSMUSG00000048217	ENSMUSG00000048217	11	101961603	101965566	210	242		
10090	17	39444087	39447875	ENSMUSG00000020921	ENSMUSG00000020921	ENSMUSG00000020921	11	101968643	101972478	210	242		
10091	17	39503644	39509238	ENSMUSG00000034793	ENSMUSG00000034793	ENSMUSG00000034793	11	102005710	102010173	210	242		
10092	17	39509647	39556540	ENSMUSG00000008855	ENSMUSG00000008855	ENSMUSG00000008855	11	102011839	102046214	210	242		
10093	17	39574852	39595370	ENSMUSG00000034773	ENSMUSG00000034773	ENSMUSG00000034773	11	102066769	102080786	210	242		
10094	17	39603600	39611977	ENSMUSG00000034768	ENSMUSG00000034768	ENSMUSG00000034768	11	102084913	102094151	210	242		
10095	17	39621941	39624619	ENSMUSG00000034757	ENSMUSG00000034757	ENSMUSG00000034757	11	102101057	102105325	210	242		
10096	17	39627277	39630964	ENSMUSG00000059995	ENSMUSG00000059995	ENSMUSG00000059995	11	102107865	102112721	210	242		
10097	17	39639488	39651190	ENSMUSG00000020923	ENSMUSG00000020923	ENSMUSG00000020923	11	102122082	102135052	210	242		
10098	17	39682566	39700993	ENSMUSG00000006574	ENSMUSG00000006574	ENSMUSG00000006574	11	102164917	102181211	210	242		
10099	17	39741469	39751565	ENSMUSG00000006575	ENSMUSG00000006575	ENSMUSG00000006575	11	102209766	102218645	210	242		
10100	17	39778017	39785996	ENSMUSG00000034708	ENSMUSG00000034708	ENSMUSG00000034708	11	102246571	102252895	210	242		
10101	17	39805258	39822367	ENSMUSG00000034664	ENSMUSG00000034664	ENSMUSG00000034664	11	102269387	102285973	210	242		
10102	17	39828178	39936328	ENSMUSG00000034621	ENSMUSG00000034621	ENSMUSG00000034621	11	102293311	102324537	210	242		
10103	17	39990353	39992382	ENSMUSG00000050288	ENSMUSG00000050288	ENSMUSG00000050288	11	102420623	102424144	210	242		
10104	17	40098012	40104114	ENSMUSG00000051455	ENSMUSG00000051455	ENSMUSG00000051455	11	102482528	102491398	210	242		
10105	17	40110334	40122652	ENSMUSG00000020925	ENSMUSG00000020925	ENSMUSG00000020925	11	102500774	102513824	210	242		
10106	17	40192094	40214740	ENSMUSG00000020926	ENSMUSG00000020926	ENSMUSG00000020926	11	102577669	102596350	210	242		
10107	17	40237146	40263707	ENSMUSG00000034520	ENSMUSG00000034520	ENSMUSG00000034520	11	102615675	102635776	210	242		
10108	17	40280805	40283366	ENSMUSG00000020928	ENSMUSG00000020928	ENSMUSG00000020928	11	102652386	102654130	210	242		
10109	17	40283363	40332318	ENSMUSG00000020929	ENSMUSG00000020929	ENSMUSG00000020929	11	102654562	102697031	210	242		
10110	17	40332661	40338284	ENSMUSG00000075510	ENSMUSG00000075510	ENSMUSG00000075510	11	102701462	102702712	210	242		
10111	17	40338521	40348380	ENSMUSG00000020932	ENSMUSG00000020932	ENSMUSG00000020932	11	102703437	102713221	210	242		
10112	17	40392588	40401170	ENSMUSG00000045532	ENSMUSG00000045532	ENSMUSG00000045532	11	102755823	102762551	210	242		
10113	17	40456240	40493912	ENSMUSG00000020935	ENSMUSG00000020935	ENSMUSG00000020935	11	102810146	102833237	210	242		
10114	17	40494206	40541903	ENSMUSG00000020936	ENSMUSG00000020936	ENSMUSG00000020936	11	102844662	102880972	210	242		
10115	17	40544544	40565407	ENSMUSG00000020937	ENSMUSG00000020937	ENSMUSG00000020937	11	102886398	102917716	210	242		
10116	17	40565524	40577322	ENSMUSG00000056938	ENSMUSG00000056938	ENSMUSG00000056938	11	102917864	102928290	210	242		
10117	17	40580467	40585251	ENSMUSG00000048878	ENSMUSG00000048878	ENSMUSG00000048878	11	102932415	102935815	210	242		
10118	17	40594054	40603189	ENSMUSG00000043372	ENSMUSG00000043372	ENSMUSG00000043372	11	102949453	102955960	210	242		
10119	17	40655075	40680464	ENSMUSG00000055805	ENSMUSG00000055805	ENSMUSG00000055805	11	102987403	103014676	210	242		
10120	17	40687543	40695262	ENSMUSG00000044787	ENSMUSG00000044787	ENSMUSG00000044787	11	103024217	103034522	210	242		
10121	17	40696278	40750148	ENSMUSG00000020941	ENSMUSG00000020941	ENSMUSG00000020941	11	103035854	103083491	210	242		
10122	17	40827058	40858780	ENSMUSG00000034255	ENSMUSG00000034255	ENSMUSG00000034255	11	103147590	103160792	210	242		
10123	17	31285662	31294787	ENSMUSG00000020945	ENSMUSG00000020945	ENSMUSG00000020945	11	103447150	103454964	Singleton			
10124	17	42411010	42411372	ENSMUSG00000046215	ENSMUSG00000046215	ENSMUSG00000046215	11	103465599	103466670	211	243		
10125	17	42355485	42372978	ENSMUSG00000020946	ENSMUSG00000020946	ENSMUSG00000020946	11	103492939	103513784	211	243		
10126	17	42283967	42309436	ENSMUSG00000018486	ENSMUSG00000018486	ENSMUSG00000018486	11	103543453	103565911	211	243		
10127	17	42196863	42251081	ENSMUSG00000000125	ENSMUSG00000000125	ENSMUSG00000000125	11	103590314	103634047	211	243		
10128	17	42056760	42189993	ENSMUSG00000034187	ENSMUSG00000034187	ENSMUSG00000034187	11	103637872	103770146	211	243		
10129	17	41239997	41267911	ENSMUSG00000018634	ENSMUSG00000018634	ENSMUSG00000018634	11	103948972	103991608	211	243		
10130	17	41278034	41280216	ENSMUSG00000049506	ENSMUSG00000049506	ENSMUSG00000049506	11	104002443	104007253	211	243		
10131	17	41327624	41461547	ENSMUSG00000018411	ENSMUSG00000018411	ENSMUSG00000018411	11	104047526	104148186	211	243		
10132	17	41463181	41626049	ENSMUSG00000018412	ENSMUSG00000018412	ENSMUSG00000018412	11	104149645	104284951	211	243		
10133	17	42552625	42621664	ENSMUSG00000020687	ENSMUSG00000020687	ENSMUSG00000020687	11	104321691	104366479	212	244		
10134	17	42641427	42656043	ENSMUSG00000061086	ENSMUSG00000061086	ENSMUSG00000061086	11	104366753	104403309	212	244		
10135	17	42686207	42745076	ENSMUSG00000020689	ENSMUSG00000020689	ENSMUSG00000020689	11	104424146	104483465	212	244		
10136	17	42756346	42873674	ENSMUSG00000040838	ENSMUSG00000040838	ENSMUSG00000040838	11	104506921	104554191	212	244		
10137	17	57811646	57847569	ENSMUSG00000020690	ENSMUSG00000020690	ENSMUSG00000020690	11	104908311	104933627	213	245		

10138	17	57889751	58044757	ENSMUSG00000020694	ENSMUSG00000020694	ENSMUSG00000020694	11	104998089	105098266	213	245		
10139	17	58058494	58124629	ENSMUSG00000020695	ENSMUSG00000020695	ENSMUSG00000020695	11	105108773	105166059	213	245		
10140	17	58132408	58239437	ENSMUSG00000050879	ENSMUSG00000050879	ENSMUSG00000050879	11	105176889	105206528	213	245		
10141	17	58750076	58853045	ENSMUSG00000053580	ENSMUSG00000053580	ENSMUSG00000053580	11	105656733	105740431	213	245		
10142	17	58863400	58877454	ENSMUSG00000019590	ENSMUSG00000019590	ENSMUSG00000019590	11	105749794	105760277	213	245		
10143	17	58908166	58928711	ENSMUSG00000020681	ENSMUSG00000020681	ENSMUSG00000020681	11	105784052	105805352	213	245		
10144	17	58981555	59025373	ENSMUSG00000049354	ENSMUSG00000049354	ENSMUSG00000049354	11	105852962	105875414	213	245		
10145	17	59031973	59039456	ENSMUSG00000001983	ENSMUSG00000001983	ENSMUSG00000001983	11	105882177	105889685	213	245		
10146	17	59053533	59127399	ENSMUSG00000020700	ENSMUSG00000020700	ENSMUSG00000020700	11	105900698	105971522	213	245		
10147	17	59176343	59204668	ENSMUSG00000069631	ENSMUSG00000069631	ENSMUSG00000069631	11	106016575	106032434	213	245		
10148	17	59205299	59250408	ENSMUSG00000020705	ENSMUSG00000020705	ENSMUSG00000020705	11	106033016	106065229	213	245		
10149	17	59250527	59258188	ENSMUSG00000020706	ENSMUSG00000020706	ENSMUSG00000020706	11	106065234	106071892	213	245		
10150	17	59258519	59263098	ENSMUSG00000020708	ENSMUSG00000020708	ENSMUSG00000020708	11	106072244	106079207	213	245		
10151	17	59263176	59273661	ENSMUSG00000040699	ENSMUSG00000040699	ENSMUSG00000040699	11	106079276	106088798	213	245		
10152	17	59359832	59363446	ENSMUSG00000040592	ENSMUSG00000040592	ENSMUSG00000040592	11	106127431	106130760	213	245		
10153	17	59369646	59404010	ENSMUSG00000001027	ENSMUSG00000001027	ENSMUSG00000001027	11	106135394	106165480	213	245		
10154	17	59433708	59451710	ENSMUSG00000001029	ENSMUSG00000001029	ENSMUSG00000001029	11	106193746	106198731	213	245		
10155	17	59475080	59561208	ENSMUSG00000020715	ENSMUSG00000020715	ENSMUSG00000020715	11	106213710	106303886	213	245		
10156	17	59578528	59694385	ENSMUSG00000040548	ENSMUSG00000040548	ENSMUSG00000040548	11	106318224	106429020	213	245		
10157	17	59754404	59794504	ENSMUSG00000020717	ENSMUSG00000020717	ENSMUSG00000020717	11	106477887	106566718	213	245		
10158	17	59875901	59895221	ENSMUSG00000040528	ENSMUSG00000040528	ENSMUSG00000040528	11	106567358	106583658	213	245		
10159	17	59904366	59923631	ENSMUSG00000020718	ENSMUSG00000020718	ENSMUSG00000020718	11	106584396	106595532	213	245		
10160	17	59933620	59964522	ENSMUSG00000018372	ENSMUSG00000018372	ENSMUSG00000018372	11	106605365	106634943	213	245		
10161	17	59971197	60088848	ENSMUSG00000018363	ENSMUSG00000018363	ENSMUSG00000018363	11	106638115	106736560	213	245		
10162	17	63252242	63410954	ENSMUSG00000040481	ENSMUSG00000040481	ENSMUSG00000040481	11	106851610	106888823	214	246		
10163	17	63144521	63170772	ENSMUSG00000018433	ENSMUSG00000018433	ENSMUSG00000018433	11	106982741	107005350	214	246		
10164	17	62764495	62793183	ENSMUSG00000020720	ENSMUSG00000020720	ENSMUSG00000020720	11	107295583	107314223	214	246		
10165	17	62504528	62671774	ENSMUSG00000020721	ENSMUSG00000020721	ENSMUSG00000020721	11	107364050	107503033	214	246		
10166	17	62471168	62483375	ENSMUSG00000020722	ENSMUSG00000020722	ENSMUSG00000020722	11	107519308	107532566	214	246		
10167	17	62391475	62459980	ENSMUSG00000020723	ENSMUSG00000020723	ENSMUSG00000020723	11	107550870	107610554	214	246		
10168	17	62303913	62311561	ENSMUSG00000040373	ENSMUSG00000040373	ENSMUSG00000040373	11	107690978	107731145	214	246		
10169	17	61729388	62237324	ENSMUSG00000050965	ENSMUSG00000050965	ENSMUSG00000050965	11	107754338	108159844	214	246		
10170	17	61638577	61655974	ENSMUSG00000000049	ENSMUSG00000000049	ENSMUSG00000000049	11	108211381	108230485	214	246		
10171	17	61062121	61618435	ENSMUSG00000020728	ENSMUSG00000020728	ENSMUSG00000020728	11	108314340	108675498	214	246		
10172	17	60955143	60988227	ENSMUSG00000000142	ENSMUSG00000000142	ENSMUSG00000000142	11	108736456	108766873	214	246		
10173	17	60564034	60654258	ENSMUSG00000020599	ENSMUSG00000020599	ENSMUSG00000020599	11	109041445	109114219	214	246		
10174	17	60437296	60483216	ENSMUSG00000020611	ENSMUSG00000020611	ENSMUSG00000020611	11	109178921	109212576	214	246		
10175	17	63755310	63764892	ENSMUSG00000020610	ENSMUSG00000020610	ENSMUSG00000020610	11	109242036	109253078	215	247		
10176	17	63775933	63799002	ENSMUSG00000041920	ENSMUSG00000041920	ENSMUSG00000041920	11	109266945	109289686	215	247		
10177	17	63766918	63928549	ENSMUSG00000020604	ENSMUSG00000020604	ENSMUSG00000020604	11	109305970	109389420	215	247		
10178	17	63929018	63965210	ENSMUSG00000041895	ENSMUSG00000041895	ENSMUSG00000041895	11	109389421	109427522	215	247		
10179	17	64019705	64040503	ENSMUSG00000020612	ENSMUSG00000020612	ENSMUSG00000020612	11	109466334	109485746	215	247		
10180	17	64044607	64109125	ENSMUSG00000020614	ENSMUSG00000020614	ENSMUSG00000020614	11	109489013	109538346	215	247		
10181	17	64375028	64463128	ENSMUSG00000020620	ENSMUSG00000020620	ENSMUSG00000020620	11	109750357	109811906	215	247		
10182	17	64482580	64568731	ENSMUSG00000041797	ENSMUSG00000041797	ENSMUSG00000041797	11	109916912	109984243	215	247		
10183	17	64586442	64649610	ENSMUSG00000044749	ENSMUSG00000044749	ENSMUSG00000044749	11	109992910	110067818	215	247		
10184	17	64754387	64834885	ENSMUSG00000018800	ENSMUSG00000018800	ENSMUSG00000018800	11	110088340	110153765	215	247		
10185	17	64922434	65051067	ENSMUSG00000020623	ENSMUSG00000020623	ENSMUSG00000020623	11	110215212	110329727	215	247		
10186	17	65583021	65643339	ENSMUSG00000051497	ENSMUSG00000051497	ENSMUSG00000051497	11	110840488	110844058	215	247		
10187	17	65677271	65687755	ENSMUSG00000041695	ENSMUSG00000041695	ENSMUSG00000041695	11	110882254	110891741	215	247		
10188	17	67628756	67634147	ENSMUSG00000000567	ENSMUSG00000000567	ENSMUSG00000000567	11	112598314	112603839	215	247		
10189	17	68153684	68600427	ENSMUSG00000041654	ENSMUSG00000041654	ENSMUSG00000041654	11	113060944	113381858	215	247		
10190	17	68672755	68679655	ENSMUSG00000047904	ENSMUSG00000047904	ENSMUSG00000047904	11	113440347	113441726	215	247		
10191	17	68700366	68716237	ENSMUSG00000018661	ENSMUSG00000018661	ENSMUSG00000018661	11	113465619	113478490	215	247		
10192	17	68740953	68755257	ENSMUSG00000041623	ENSMUSG00000041623	ENSMUSG00000041623	11	113500500	113515408	215	247		
10193	17	68761642	68769552	ENSMUSG00000018727	ENSMUSG00000018727	ENSMUSG00000018727	11	113514262	113526107	215	247		
10194	17	68791359	68819738	ENSMUSG00000041598	ENSMUSG00000041598	ENSMUSG00000041598	11	113542946	113567897	215	247		
10195	17	68845407	68945808	ENSMUSG00000041592	ENSMUSG00000041592	ENSMUSG00000041592	11	113592464	113882308	215	247		
10196	17	69721291	69769750	ENSMUSG00000034714	ENSMUSG00000034714	ENSMUSG00000034714	11	114491558	114537074	215	247		
10197	17	69782024	69822352	ENSMUSG00000034706	ENSMUSG00000034706	ENSMUSG00000034706	11	114549003	114552077	215	247		
10198	17	69864391	69869553	ENSMUSG00000000202	ENSMUSG00000000202	ENSMUSG00000000202	11	114606759	114611982	215	247		
10199	17	69833546	69882691	ENSMUSG00000034677	ENSMUSG00000034677	ENSMUSG00000034677	11	114615014	114622835	215	247		
10200	17	69938647	69955161	ENSMUSG00000051043	ENSMUSG00000051043	ENSMUSG00000051043	11	114667631	114685185	215	247		
10201	17	70028909	70039200	ENSMUSG00000063193	ENSMUSG00000063193	ENSMUSG00000063193	11	114738871	114750476	215	247		
10202	17	70117621	70131474	ENSMUSG00000048498	ENSMUSG00000048498	ENSMUSG00000048498	11	114868007	114878129	215	247		
10203	17	70244956	70255068	ENSMUSG00000020732	ENSMUSG00000020732	ENSMUSG00000020732	11	114970268	114978323	215	247		
10204	17	70256581	70277084	ENSMUSG00000020733	ENSMUSG00000020733	ENSMUSG00000020733	11	114979431	114997268	215	247		
10205	17	70278289	70284095	ENSMUSG00000015542	ENSMUSG00000015542	ENSMUSG00000015542	11	114998922	115003934	215	247		
10206	17	70284254	70347484	ENSMUSG00000045980	ENSMUSG00000045980	ENSMUSG00000045980	11	115003615	115061911	215	247		
10207	17	70349757	70367593	ENSMUSG00000020734	ENSMUSG00000020734	ENSMUSG00000020734	11	115065661	115077222	215	247		
10208	17	70370218	70380692	ENSMUSG00000018861	ENSMUSG00000018861	ENSMUSG00000018861	11	115084115	115093059	215	247		
10209	17	70385076	70401310	ENSMUSG00000044788	ENSMUSG00000044788	ENSMUSG00000044788	11	115096743	115113566	215	247		
10210	17	70431965	70441235	ENSMUSG00000050201	ENSMUSG00000050201	ENSMUSG00000050201	11	115123253	115148391	215	247		
10211	17	70423771	70430946	ENSMUSG00000045288	ENSMUSG00000045288	ENSMUSG00000045288	11	115131282	115138131	215	247		
10212	17	70443492	70457106	ENSMUSG00000018862	ENSMUSG00000018862	ENSMUSG00000018862	11	115150869	115163016	215	247		
10213	17	70458435	70480424	ENSMUSG00000034586	ENSMUSG00000034586	ENSMUSG00000034586	11	115163799	115183809	215	247		
10214	17	70495322	70513479	ENSMUSG00000050910	ENSMUSG00000050910	ENSMUSG00000050910	11	115198431	115210327	215	247		
10215	17	70520364	70528950	ENSMUSG00000018858	ENSMUSG00000018858	ENSMUSG00000018858	11	115219842	115227008	215	247		
10216	17	70554941	70573574	ENSMUSG00000016940	ENSMUSG00000016940	ENSMUSG00000016940	11	115236218	115247364	215	247		
10217	17	70595417	70613841	ENSMUSG00000045775	ENSMUSG00000045775	ENSMUSG00000045775	11	115278564	115286240	215	247		
10218	17	70617677	70637955	ENSMUSG00000057219	ENSMUSG00000057219	ENSMUSG00000057219	11	115291767	115306556	215	247		
10219	17	70637919	70639472	ENSMUSG00000020736	ENSMUSG00000020736	ENSMUSG00000020736	11	115306518	115307943	215	247		
10220	17	70642938	70662370	ENSMUSG00000020737	ENSMUSG00000020737	ENSMUSG00000020737	11	115313445	115330477	215	247		
10221	17	70713192	70743449	ENSMUSG00000020739	ENSMUSG00000020739	ENSMUSG00000020739	11	115380572	115400075	215	247		
10222	17	70744290	70769299	ENSMUSG00000020740	ENSMUSG00000020740	ENSMUSG00000020740	11	115400345	115419985	215	247		
10223	17	70769361	70774046	ENSMUSG00000046756	ENSMUSG00000046756	ENSMUSG00000046756	11	115420241	115423716	215	247		
10224	17	70774164	70778898	ENSMUSG00000020743	ENSMUSG00000020743	ENSMUSG00000020743	11	115424016	115429019	215	247		
10225	17	70780669	70797109	ENSMUSG00000020744	ENSMUSG00000020744	ENSMUSG00000020744	11	115431073	115444235	215	247		
10226	17	70825753	70913384	ENSMUSG00000059923	ENSMUSG00000059923	ENSMUSG00000059923	11	115460216	115524687	215	247		
10227	17	70964317	71007758	ENSMUSG00000020747	ENSMUSG00000020747	ENSMUSG00000020747	11	115581608	115615123	215	247		
10228	17	71007938	71023222	ENSMUSG00000034471	ENSMUSG00000034471	ENSMUSG00000034471	11	115615274	115629720	215	247		
10229	17	71024230	71032411	ENSMUSG00000020781	ENSMUSG00000020781	ENSMUSG00000020781	11	115630845	115639179	215	247		
10230	17	71033378	71082884	ENSMUSG00000020782	ENSMUSG00000020782	ENSMUSG00000020782	11	115640139	115671870	215	247		
10231	17	71103976	71128356	ENSMUSG00000034427	ENSMUSG00000034427	ENSMUSG00000034427	11	115696515	115703535	215	247		
10232	17	71134530	71174864	ENSMUSG00000020752	ENSMUSG00000020752	ENSMUSG00000020752	11	115708693	115749556	215	247		
10233	17	71174994	71215733	ENSMUSG00000020755	ENSMUSG00000020755	ENSMUSG00000020755	11	115749749	115781624	215	247		
10234	17	71229111	71265493	ENSMUSG00000020758	ENSMUSG00000020758	ENSMUSG00000020758	11	115791069	115824502	215	247		
10235	17	71265732	71272875	ENSMUSG00000020766	ENSMUSG00000020766	ENSMUSG00000020766	11	115824567	115828809	215	247		
10236	17	71292532	71333474	ENSMUSG00000020770	ENSMUSG00000020770	ENSMUSG00000020770	11	115846406	115877304	215	247		
10237	17	71334940	71352393	ENSMUSG00000057948	ENSMUSG00000057948	ENSMUSG00000057948	11	115878186	115894051	215	247		
10238	17	71381842	71386251	ENSMUSG00000020773	ENSMUSG00000020773	ENSMUSG00000020773	11	115921844	115926310	215	247		
10239	17	71396640	71404649	ENSMUSG00000054517	ENSMUSG00000054517	ENSMUSG00000054517	11	115940796	115947218	215	247		
10240	17	71406319	71413069	ENSMUSG00000020775	ENSMUSG00000020775	ENSMUSG00000020775	11	115947912	115954896	215	247		
10241	17	71418402	71445873	ENSMUSG00000020776	ENSMUSG00000020776	ENSMUSG00000020776	11	115958375	115984243	215	247		
10242	17	71453260	71487039	ENSMUSG00000020777	ENSMUSG00000020777	ENSMUSG00000020777	11	115987978	116015135	215	247		
10243	17	71486907	71513673	ENSMUSG00000034288	ENSMUSG00000034288	ENSMUSG00000034288	11	116032093	116035839	215	247		
10244	17	71514522	71534972	ENSMUSG00000034282	ENSMUSG00000034282	ENSMUSG00000034282	11	116036845	116054087	215	247		
10245	17	71546786	71580202	ENSMUSG00000020780	ENSMUSG00000020780	ENSMUSG00000020780	11	116061254	116090307	215	247		
10246	17	71582479	71585168	ENSMUSG00000020793	ENSMUSG00000020793	ENSMUSG00000020793	11	116097029	116100005	215	247		
10247	17	71590068	71611371	ENSMUSG00000020792	ENSMUSG00000020792	ENSMUSG00000020792	11	116105273	116122763	215	247		
10248	17	71644020	71648966	ENSMUSG00000034227	ENSMUSG00000034227	ENSMUSG00000034227	11	116146792	116151437	215	247		
10249	17	71651451	71747985	ENSMUSG00000052949	ENSMUSG00000052949	ENSMUSG00000052949	11	116160148	116228901	215	247		
10250	17	71781727	71815356	ENSMUSG00000070331	ENSMUSG00000070331	ENSMUSG00000070331	11	116257415	116270437	215	247		
10251	17	71818609	71861825	ENSMUSG00000015869	ENSMUSG00000015869	ENSMUSG00000015869	11	116286936	116306438	215	247		
10252	17	72073056	72093524	ENSMUSG00000057286	ENSMUSG00000057286	ENSMUSG00000057286	11	116315439	116510702	215	248	here	
10253	17	71892326	71895530	ENSMUSG00000061878	ENSMUSG00000061878	ENSMUSG00000061878	11	116348541	116352476	215	248	Single marker moved to different place with in a HSB	1 -> 2
10254	17	71897510	71960883	ENSMUSG00000020802	ENSMUSG00000020802	ENSMUSG00000020802	11	116353843	116397537	215	248		
10255	17	71961028	71977793	ENSMUSG00000020804	ENSMUSG00000020804	ENSMUSG00000020804	11	116409777	116413635	215	248		
10256	17	71978574	72009084	ENSMUSG00000020806	ENSMUSG00000020806	ENSMUSG00000020806	11	116414255	116440290	215	248		
10257	17	72035056	72045561	ENSMUSG00000020810	ENSMUSG00000020810	ENSMUSG00000020810	11	116461685	116470403	215	248		
10258	17	72132442	72151463	ENSMUSG00000009588	ENSMUSG00000009588	ENSMUSG00000009588	11	116581115	116591597	215	248		
10259	17	72187248	72197896	ENSMUSG00000020814	ENSMUSG00000020814	ENSMUSG00000020814	11	116619494	116644136	215	248		
10260	17	72220514	72234158	ENSMUSG00000056962	ENSMUSG00000056962	ENSMUSG00000056962	11	116653523	116659539	215	248		
10261	17	72234520	72241363	ENSMUSG00000020815	ENSMUSG00000020815	ENSMUSG00000020815	11	116659690	116665819	215	248		
10262	17	72245378	72290878	ENSMUSG00000020818	ENSMUSG00000020818	ENSMUSG00000020818	11	116670105	116691774	215	248		
10263	17	72376393	72458060	ENSMUSG00000043857	ENSMUSG00000043857	ENSMUSG00000043857	11	116734953	116803038	215	248		
10264	17	72648647	72722006	ENSMUSG00000020823	ENSMUSG00000020823	ENSMUSG00000020823	11	116931262	116975355	215	248		
10265	17	72909732	73008269	ENSMUSG00000059248	ENSMUSG00000059248	ENSMUSG00000059248	11	117015751	117178394	215	248		
10266	17	73556589	73612616	ENSMUSG00000025571	ENSMUSG00000025571	ENSMUSG00000025571	11	117516852	117578654	215	248		
10267	17	73620594	73640083	ENSMUSG00000025572	ENSMUSG00000025572	ENSMUSG00000025572	11	117582076	117596729	215	248		
10268	17	73638494	73650644	ENSMUSG00000050106	ENSMUSG00000050106	ENSMUSG00000050106	11	117599140	117609118	215	248		
10269	17	73654029	73673829	ENSMUSG00000025573	ENSMUSG00000025573	ENSMUSG00000025573	11	117613857	117622422	215	248		
10270	17	73676266	73680604	ENSMUSG00000048277	ENSMUSG00000048277	ENSMUSG00000048277	11	117625767	117630371	215	248		
10271	17	73695047	73715360	ENSMUSG00000017718	ENSMUSG00000017718	ENSMUSG00000017718	11	117642014	117655998	215	248		
10272	17	73721872	73733310	ENSMUSG00000017716	ENSMUSG00000017716	ENSMUSG00000017716	11	117665327	117671833	215	248		
10273	17	73739704	73748662	ENSMUSG00000070330	ENSMUSG00000070330	ENSMUSG00000070330	11	117676839	117680434	215	248		
10274	17	73864459	73867753	ENSMUSG00000053113	ENSMUSG00000053113	ENSMUSG00000053113	11	117782179	117785276	215	248		
10275	17	73886333	73932230	ENSMUSG00000017715	ENSMUSG00000017715	ENSMUSG00000017715	11	117802947	117840101	215	248		
10276	17	73931378	73980451	ENSMUSG00000033987	ENSMUSG00000033987	ENSMUSG00000033987	11	117839343	117910291	215	248		
10277	17	74181727	74289971	ENSMUSG00000017132	ENSMUSG00000017132	ENSMUSG00000017132	11	117982106	118009788	215	248		
10278	17	74295065	74348482	ENSMUSG00000033909	ENSMUSG00000033909	ENSMUSG00000033909	11	118078478	118106321	215	248		
10279	17	74360658	74433067	ENSMUSG00000017466	ENSMUSG00000017466	ENSMUSG00000017466	11	118117159	118171596	215	248		
10280	17	74398315	74409505	ENSMUSG00000076433	ENSMUSG00000076433	ENSMUSG00000076433	11	118148450	118158590	215	248		
10281	17	74478932	74487656	ENSMUSG00000033880	ENSMUSG00000033880	ENSMUSG00000033880	11	118208839	118218049	215	248		
10282	17	74499394	74517433	ENSMUSG00000025575	ENSMUSG00000025575	ENSMUSG00000025575	11	118222379	118235176	215	248		
10283	17	74531846	74557464	ENSMUSG00000017446	ENSMUSG00000017446	ENSMUSG00000017446	11	118244589	118266052	215	248		
10284	17	74582622	74594373	ENSMUSG00000033857	ENSMUSG00000033857	ENSMUSG00000033857	11	118293048	118305288	215	248		
10285	17	74600274	74623392	ENSMUSG00000025576	ENSMUSG00000025576	ENSMUSG00000025576	11	118305850	118725662	215	248		
10286	17	75319276	75330612	ENSMUSG00000046697	ENSMUSG00000046697	ENSMUSG00000046697	11	118804278	118808931	215	248		
10287	17	75366588	75375973	ENSMUSG00000025577	ENSMUSG00000025577	ENSMUSG00000025577	11	118839119	118847360	215	248		
10288	17	75382788	75385510	ENSMUSG00000025578	ENSMUSG00000025578	ENSMUSG00000025578	11	118854526	118857003	215	248		
10289	17	75422353	75427664	ENSMUSG00000039989	ENSMUSG00000039989	ENSMUSG00000039989	11	118896245	118902231	215	248		
10290	17	75528416	75624242	ENSMUSG00000039976	ENSMUSG00000039976	ENSMUSG00000039976	11	118963227	119044589	215	248		
10291	17	75625046	75678793	ENSMUSG00000039963	ENSMUSG00000039963	ENSMUSG00000039963	11	119044662	119081302	215	248		
10292	17	75689877	75708273	ENSMUSG00000025579	ENSMUSG00000025579	ENSMUSG00000025579	11	119084118	119101544	215	248		
10293	17	75766869	75797450	ENSMUSG00000013483	ENSMUSG00000013483	ENSMUSG00000013483	11	119130877	119161467	215	248		
10294	17	75798846	75808716	ENSMUSG00000005043	ENSMUSG00000005043	ENSMUSG00000005043	11	119159581	119171628	215	248		
10295	17	75808824	75841890	ENSMUSG00000039908	ENSMUSG00000039908	ENSMUSG00000039908	11	119171647	119197166	215	248		
10296	17	75928544	75981893	ENSMUSG00000070327	ENSMUSG00000070327	ENSMUSG00000070327	11	119246255	119272394	215	248		
10297	17	76003562	76026476	ENSMUSG00000039850	ENSMUSG00000039850	ENSMUSG00000039850	11	119307790	119327527	215	248		
10298	17	76055228	76064999	ENSMUSG00000025582	ENSMUSG00000025582	ENSMUSG00000025582	11	119355462	119363909	215	248		
10299	17	76133663	76554760	ENSMUSG00000025583	ENSMUSG00000025583	ENSMUSG00000025583	11	119419900	119713572	215	248		
10300	17	76623557	76705827	ENSMUSG00000025372	ENSMUSG00000025372	ENSMUSG00000025372	11	119759182	119822869	215	248		
10301	17	76705703	76720341	ENSMUSG00000025375	ENSMUSG00000025375	ENSMUSG00000025375	11	119823401	119862880	215	248		
10302	17	76777991	76811394	ENSMUSG00000039781	ENSMUSG00000039781	ENSMUSG00000039781	11	119880525	119902908	215	248		
10303	17	76816674	76827450	ENSMUSG00000025377	ENSMUSG00000025377	ENSMUSG00000025377	11	119906619	119914790	215	248		
10304	17	76839592	76883662	ENSMUSG00000061306	ENSMUSG00000061306	ENSMUSG00000061306	11	119920050	119967436	215	248		
10305	17	77025447	77043586	ENSMUSG00000039741	ENSMUSG00000039741	ENSMUSG00000039741	11	120075158	120108372	215	248		
10306	17	77091594	77094422	ENSMUSG00000062825	ENSMUSG00000062825	ENSMUSG00000062825	11	120161781	120164582	215	248		
10307	17	77110153	77114582	ENSMUSG00000025380	ENSMUSG00000025380	ENSMUSG00000025380	11	120177624	120184261	215	248		
10308	17	77142788	77207306	ENSMUSG00000039703	ENSMUSG00000039703	ENSMUSG00000039703	11	120198559	120249233	215	248		
10309	17	77219767	77226183	ENSMUSG00000039691	ENSMUSG00000039691	ENSMUSG00000039691	11	120259316	120263037	215	248		
10310	17	77227894	77234018	ENSMUSG00000025386	ENSMUSG00000025386	ENSMUSG00000025386	11	120263700	120269573	215	248		
10311	17	77242472	77244012	ENSMUSG00000039670	ENSMUSG00000039670	ENSMUSG00000039670	11	120272694	120274153	215	248		
10312	17	77244191	77251276	ENSMUSG00000049957	ENSMUSG00000049957	ENSMUSG00000049957	11	120274219	120280444	215	248		
10313	17	77261425	77279548	ENSMUSG00000025793	ENSMUSG00000025793	ENSMUSG00000025793	11	120283765	120300065	215	248		
10314	17	77289742	77298446	ENSMUSG00000025792	ENSMUSG00000025792	ENSMUSG00000025792	11	120307952	120317251	215	248		
10315	17	77384894	77386215	ENSMUSG00000025129	ENSMUSG00000025129	ENSMUSG00000025129	11	120366065	120367222	215	248		
10316	17	77394326	77411680	ENSMUSG00000025130	ENSMUSG00000025130	ENSMUSG00000025130	11	120376394	120389026	215	248		
10317	17	77420041	77422490	ENSMUSG00000025132	ENSMUSG00000025132	ENSMUSG00000025132	11	120394200	120397640	215	248		
10318	17	77442895	77451655	ENSMUSG00000025135	ENSMUSG00000025135	ENSMUSG00000025135	11	120414527	120424288	215	248		
10319	17	77453417	77453940	ENSMUSG00000044034	ENSMUSG00000044034	ENSMUSG00000044034	11	120424599	120425033	215	248		
10320	17	77455384	77462586	ENSMUSG00000025137	ENSMUSG00000025137	ENSMUSG00000025137	11	120426177	120433980	215	248		
10321	17	77463107	77469299	ENSMUSG00000025138	ENSMUSG00000025138	ENSMUSG00000025138	11	120434490	120441068	215	248		
10322	17	77469441	77478879	ENSMUSG00000051510	ENSMUSG00000051510	ENSMUSG00000051510	11	120444423	120449637	215	248		
10323	17	77483560	77488443	ENSMUSG00000025140	ENSMUSG00000025140	ENSMUSG00000025140	11	120455506	120459792	215	248		
10324	17	77491066	77493546	ENSMUSG00000025141	ENSMUSG00000025141	ENSMUSG00000025141	11	120462122	120464248	215	248		
10325	17	77503689	77512353	ENSMUSG00000042988	ENSMUSG00000042988	ENSMUSG00000042988	11	120469936	120474134	215	248		
10326	17	77528715	77568569	ENSMUSG00000025142	ENSMUSG00000025142	ENSMUSG00000025142	11	120489124	120525537	215	248		
10327	17	77569869	77574062	ENSMUSG00000025144	ENSMUSG00000025144	ENSMUSG00000025144	11	120527033	120529833	215	248		
10328	17	77574569	77582313	ENSMUSG00000025145	ENSMUSG00000025145	ENSMUSG00000025145	11	120530063	120537218	215	248		
10329	17	77582821	77585366	ENSMUSG00000018012	ENSMUSG00000018012	ENSMUSG00000018012	11	120537558	120540059	215	248		
10330	17	77587046	77588862	ENSMUSG00000039450	ENSMUSG00000039450	ENSMUSG00000039450	11	120541491	120543370	215	248		
10331	17	77599067	77602939	ENSMUSG00000025158	ENSMUSG00000025158	ENSMUSG00000025158	11	120596835	120600294	215	248		
10332	17	77609280	77616224	ENSMUSG00000025155	ENSMUSG00000025155	ENSMUSG00000025155	11	120605292	120612485	215	248		

10333	17	77629504	77649395	ENSMUSG00000025153	ENSMUSG00000025153	ENSMUSG00000025153	11	120623167	120640332	215	248		
10334	17	77652850	77753109	ENSMUSG00000048445	ENSMUSG00000048445	ENSMUSG00000048445	11	120642632	120748962	215	248		
10335	17	77784852	77790643	ENSMUSG00000025161	ENSMUSG00000025161	ENSMUSG00000025161	11	120764574	120775090	215	248		
10336	17	77795946	77824546	ENSMUSG00000025162	ENSMUSG00000025162	ENSMUSG00000025162	11	120775296	120807423	215	248		
10337	17	77866035	77868769	ENSMUSG00000025163	ENSMUSG00000025163	ENSMUSG00000025163	11	120852841	120855508	215	248		
10338	17	77925490	77926659	ENSMUSG00000039321	ENSMUSG00000039321	ENSMUSG00000039321	11	120976361	120978063	215	248		
10339	17	77943389	77969741	ENSMUSG00000025169	ENSMUSG00000025169	ENSMUSG00000025169	11	120993683	121020738	215	248		
10340	17	77969862	77993806	ENSMUSG00000039307	ENSMUSG00000039307	ENSMUSG00000039307	11	121023782	121038737	215	248		
10341	17	78009861	78039430	ENSMUSG00000000056	ENSMUSG00000000056	ENSMUSG00000000056	11	121053343	121071946	215	248		
10342	17	78165777	78199628	ENSMUSG00000025173	ENSMUSG00000025173	ENSMUSG00000025173	11	121143324	121170499	215	248		
10343	17	78267921	78279178	ENSMUSG00000039253	ENSMUSG00000039253	ENSMUSG00000039253	11	121237517	121246858	215	248		
10344	17	78286773	78302301	ENSMUSG00000025175	ENSMUSG00000025175	ENSMUSG00000025175	11	121251050	121266579	215	248		
10345	17	78303323	78493684	ENSMUSG00000039230	ENSMUSG00000039230	ENSMUSG00000039230	11	121268039	121433254	215	248		
10346	17	78380600	78391188	ENSMUSG00000039238	ENSMUSG00000039238	ENSMUSG00000039238	11	121327068	121335392	215	248		
10347	17	78494961	78602975	ENSMUSG00000046605	ENSMUSG00000046605	ENSMUSG00000046605	11	121432860	121489229	215	248		
10348	17	78630856	78646005	ENSMUSG00000039208	ENSMUSG00000039208	ENSMUSG00000039208	11	121518517	121533479	215	248		
10349	2	26110483	26213827	ENSMUSG00000020671	ENSMUSG00000020671	ENSMUSG00000020671	12	3247726	3309939	216	249		
10350	2	26002972	26058907	ENSMUSG00000020668	ENSMUSG00000020668	ENSMUSG00000020668	12	3365132	3406494	216	249		
10351	2	25814065	25954839	ENSMUSG00000037486	ENSMUSG00000037486	ENSMUSG00000037486	12	3426901	3502468	216	249		
10352	2	25453635	25750007	ENSMUSG00000071454	ENSMUSG00000071454	ENSMUSG00000071454	12	3572523	3781505	216	249		
10353	2	25309350	25418963	ENSMUSG00000020661	ENSMUSG00000020661	ENSMUSG00000020661	12	3807104	3914455	216	249		
10354	2	25237226	25245063	ENSMUSG00000020660	ENSMUSG00000020660	ENSMUSG00000020660	12	3954967	3960634	216	249		
10355	2	25118503	25224261	ENSMUSG00000020658	ENSMUSG00000020658	ENSMUSG00000020658	12	3966458	3985579	216	249		
10356	2	25020009	25048328	ENSMUSG00000020657	ENSMUSG00000020657	ENSMUSG00000020657	12	4082500	4110496	216	249		
10357	2	24895542	24996212	ENSMUSG00000020654	ENSMUSG00000020654	ENSMUSG00000020654	12	4133468	4214742	216	249		
10358	2	24869518	24898747	ENSMUSG00000020652	ENSMUSG00000020652	ENSMUSG00000020652	12	4212890	4241926	216	249		
10359	2	24866645	24869755	ENSMUSG00000073240	ENSMUSG00000073240	ENSMUSG00000073240	12	4241659	4244548	216	249		
10360	2	24660850	24847075	ENSMUSG00000020647	ENSMUSG00000020647	ENSMUSG00000020647	12	4257418	4491127	216	249		
10361	2	24279237	24437087	ENSMUSG00000020640	ENSMUSG00000020640	ENSMUSG00000020640	12	4606965	4727848	216	249		
10362	2	24191745	24199678	ENSMUSG00000020639	ENSMUSG00000020639	ENSMUSG00000020639	12	4786043	4795229	216	249		
10363	2	24143958	24152817	ENSMUSG00000037361	ENSMUSG00000037361	ENSMUSG00000037361	12	4832269	4849746	216	249		
10364	2	24126088	24140055	ENSMUSG00000020635	ENSMUSG00000020635	ENSMUSG00000020635	12	4855525	4863945	216	249		
10365	2	24105714	24123727	ENSMUSG00000051721	ENSMUSG00000051721	ENSMUSG00000051721	12	4865832	4879220	216	249		
10366	2	24098180	24100104	ENSMUSG00000037336	ENSMUSG00000037336	ENSMUSG00000037336	12	4889589	4895579	216	249		
10367	2	23462075	23784977	ENSMUSG00000020627	ENSMUSG00000020627	ENSMUSG00000020627	12	5105103	5401690	216	249		
10368	2	21213845	21217264	ENSMUSG00000071430	ENSMUSG00000071430	ENSMUSG00000071430	12	7906491	7909653	216	249		
10369	2	21077806	21120450	ENSMUSG00000020609	ENSMUSG00000020609	ENSMUSG00000020609	12	8003787	8042507	216	249		
10370	2	20747285	20886355	ENSMUSG00000037669	ENSMUSG00000037669	ENSMUSG00000037669	12	8234131	8311760	216	249		
10371	2	20729905	20734731	ENSMUSG00000037660	ENSMUSG00000037660	ENSMUSG00000037660	12	8323942	8327962	216	249		
10372	2	20681045	20714345	ENSMUSG00000020605	ENSMUSG00000020605	ENSMUSG00000020605	12	8339455	8369822	216	249		
10373	2	20510316	20512687	ENSMUSG00000054364	ENSMUSG00000054364	ENSMUSG00000054364	12	8525050	8525640	216	249		
10374	2	20314552	20413944	ENSMUSG00000020594	ENSMUSG00000020594	ENSMUSG00000020594	12	8700689	8776347	216	249		
10375	2	20095892	20115270	ENSMUSG00000020585	ENSMUSG00000020585	ENSMUSG00000020585	12	8947672	8964735	216	249		
10376	2	20056353	20075936	ENSMUSG00000020583	ENSMUSG00000020583	ENSMUSG00000020583	12	8973979	8998036	216	249		
10377	2	19973502	20053373	ENSMUSG00000066643	ENSMUSG00000066643	ENSMUSG00000066643	12	8999900	9054855	216	249		
10378	2	19960001	19965228	ENSMUSG00000066637	ENSMUSG00000066637	ENSMUSG00000066637	12	9056005	9062402	216	249		
10379	2	19414727	19421895	ENSMUSG00000048387	ENSMUSG00000048387	ENSMUSG00000048387	12	9600505	9606929	216	249		
10380	2	18607618	18634319	ENSMUSG00000020622	ENSMUSG00000020622	ENSMUSG00000020622	12	10396052	10416178	216	249		
10381	2	17861267	17861848	ENSMUSG00000047002	ENSMUSG00000047002	ENSMUSG00000047002	12	11234725	11235291	216	249		
10382	2	17798658	17829557	ENSMUSG00000051235	ENSMUSG00000051235	ENSMUSG00000051235	12	11266084	11292109	216	249		
10383	2	17708560	17798578	ENSMUSG00000020608	ENSMUSG00000020608	ENSMUSG00000020608	12	11292229	11346132	216	249		
10384	2	17583874	17700826	ENSMUSG00000054459	ENSMUSG00000054459	ENSMUSG00000054459	12	11351590	11462956	216	249		
10385	2	16594208	16710583	ENSMUSG00000020589	ENSMUSG00000020589	ENSMUSG00000020589	12	12288163	12399381	216	249		
10386	2	15648753	15688676	ENSMUSG00000037149	ENSMUSG00000037149	ENSMUSG00000037149	12	13245295	13275162	216	249		
10387	2	15224483	15618905	ENSMUSG00000020576	ENSMUSG00000020576	ENSMUSG00000020576	12	13348976	13610325	216	249		
10388	2	14691555	14692788	ENSMUSG00000020607	ENSMUSG00000020607	ENSMUSG00000020607	12	14173138	14177578	216	249		
10389	2	12774466	12800311	ENSMUSG00000020601	ENSMUSG00000020601	ENSMUSG00000020601	12	15817277	15842335	216	249		
10390	2	11804191	11884982	ENSMUSG00000020593	ENSMUSG00000020593	ENSMUSG00000020593	12	16562668	16636446	216	249		
10391	2	11715756	11727706	ENSMUSG00000020591	ENSMUSG00000020591	ENSMUSG00000020591	12	16678980	16685673	216	249		
10392	2	11591707	11700363	ENSMUSG00000036523	ENSMUSG00000036523	ENSMUSG00000036523	12	16697094	16765652	216	249		
10393	2	11502868	11523748	ENSMUSG00000057469	ENSMUSG00000057469	ENSMUSG00000057469	12	16836457	16852223	216	249		
10394	2	11239229	11402162	ENSMUSG00000020580	ENSMUSG00000020580	ENSMUSG00000020580	12	16920670	17003586	216	249		
10395	2	11190630	11203601	ENSMUSG00000071398	ENSMUSG00000071398	ENSMUSG00000071398	12	17030438	17037034	216	249		
10396	2	10969514	10971802	ENSMUSG00000051726	ENSMUSG00000051726	ENSMUSG00000051726	12	17200181	17201662	216	249		
10397	2	10841438	10878441	ENSMUSG00000020571	ENSMUSG00000020571	ENSMUSG00000020571	12	17292075	17310250	216	249		
10398	2	10779226	10842687	ENSMUSG00000020566	ENSMUSG00000020566	ENSMUSG00000020566	12	17310203	17350183	216	249		
10399	2	10629232	10747544	ENSMUSG00000061458	ENSMUSG00000061458	ENSMUSG00000061458	12	17373973	17455575	216	249		
10400	2	9264345	9463257	ENSMUSG00000052632	ENSMUSG00000052632	ENSMUSG00000052632	12	21358434	21516661	216	250	here	This inversion was ignored by our tool.
10401	2	9463264	9481127	ENSMUSG00000062352	ENSMUSG00000062352	ENSMUSG00000062352	12	21513910	21532709	216	250	Inversion ignored by ST	1 -> 3
10402	2	9532134	9547305	ENSMUSG00000062054	ENSMUSG00000062054	ENSMUSG00000062054	12	21562882	21570097	216	250		
10403	2	9546066	9613368	ENSMUSG00000052593	ENSMUSG00000052593	ENSMUSG00000052593	12	21569999	21620083	216	250		
10404	2	9900934	9991940	ENSMUSG00000059669	ENSMUSG00000059669	ENSMUSG00000059669	12	25089515	25147443	216	250		
10405	2	10009362	10058411	ENSMUSG00000020656	ENSMUSG00000020656	ENSMUSG00000020656	12	25161395	25206466	216	250		
10406	2	10101133	10112414	ENSMUSG00000020653	ENSMUSG00000020653	ENSMUSG00000020653	12	25240450	25251853	216	250		
10407	2	10116317	10137989	ENSMUSG00000062563	ENSMUSG00000062563	ENSMUSG00000062563	12	25254917	25270892	216	250		
10408	2	10179906	10188996	ENSMUSG00000020649	ENSMUSG00000020649	ENSMUSG00000020649	12	25297333	25303186	216	250		
10409	2	8910271	9061393	ENSMUSG00000020646	ENSMUSG00000020646	ENSMUSG00000020646	12	25418468	25548953	216	251		
10410	2	8786511	8895181	ENSMUSG00000036333	ENSMUSG00000036333	ENSMUSG00000036333	12	25577650	25648550	216	251		
10411	2	8736791	8741997	ENSMUSG00000020644	ENSMUSG00000020644	ENSMUSG00000020644	12	25682881	25685170	216	251		
10412	2	6974974	7101675	ENSMUSG00000020642	ENSMUSG00000020642	ENSMUSG00000020642	12	26895792	27001421	216	251		
10413	2	6935277	6955821	ENSMUSG00000020641	ENSMUSG00000020641	ENSMUSG00000020641	12	27029021	27042699	216	251		
10414	2	6905904	6923254	ENSMUSG00000020638	ENSMUSG00000020638	ENSMUSG00000020638	12	27055472	27066103	216	251		
10415	2	5750250	5758968	ENSMUSG00000063632	ENSMUSG00000063632	ENSMUSG00000063632	12	27927489	27928676	216	251		
10416	2	3683685	3728131	ENSMUSG00000020636	ENSMUSG00000020636	ENSMUSG00000020636	12	29140023	29168751	216	251		
10417	2	3620569	3670073	ENSMUSG00000036655	ENSMUSG00000036655	ENSMUSG00000036655	12	29180925	29209558	216	251		
10418	2	3600728	3606383	ENSMUSG00000061477	ENSMUSG00000061477	ENSMUSG00000061477	12	29217366	29222193	216	251		
10419	2	3570258	3583725	ENSMUSG00000020630	ENSMUSG00000020630	ENSMUSG00000020630	12	29235908	29245863	216	251		
10420	2	3480697	3502354	ENSMUSG00000020629	ENSMUSG00000020629	ENSMUSG00000020629	12	29261496	29267873	216	251		
10421	2	3362453	3462349	ENSMUSG00000020628	ENSMUSG00000020628	ENSMUSG00000020628	12	29277280	29336650	216	251		
10422	2	3171743	3360646	ENSMUSG00000036613	ENSMUSG00000036613	ENSMUSG00000036613	12	29338134	29453674	216	251		
10423	2	1771894	2314052	ENSMUSG00000061911	ENSMUSG00000061911	ENSMUSG00000061911	12	30369673	30506533	216	251		
10424	2	1614667	1727285	ENSMUSG00000020674	ENSMUSG00000020674	ENSMUSG00000020674	12	30524186	30603703	216	251		
10425	2	1396242	1525502	ENSMUSG00000020673	ENSMUSG00000020673	ENSMUSG00000020673	12	30640711	30718661	216	251		
10426	2	936552	1350389	ENSMUSG00000020672	ENSMUSG00000020672	ENSMUSG00000020672	12	30761046	30959380	216	251		
10427	2	657975	667439	ENSMUSG00000043061	ENSMUSG00000043061	ENSMUSG00000043061	12	31170523	31177275	216	251		
10428	2	273117	656568	ENSMUSG00000054204	ENSMUSG00000054204	ENSMUSG00000054204	12	31470393	31479876	216	251		
10429	2	254869	268283	ENSMUSG00000044573	ENSMUSG00000044573	ENSMUSG00000044573	12	31479708	31497634	216	251		
10430	2	208788	254745	ENSMUSG00000020669	ENSMUSG00000020669	ENSMUSG00000020669	12	31497781	31546069	216	251		
10431	2	35453	36445	ENSMUSG00000036136	ENSMUSG00000036136	ENSMUSG00000036136	12	31660023	31665995	216	251		
10432	7	107351499	107431040	ENSMUSG00000002900	ENSMUSG00000002900	ENSMUSG00000002900	12	31851443	31915700	217	252		
10433	7	107318847	107347645	ENSMUSG00000020664	ENSMUSG00000020664	ENSMUSG00000020664	12	31917620	31937471	217	252		
10434	7	107193156	107230888	ENSMUSG00000001225	ENSMUSG00000001225	ENSMUSG00000001225	12	32024267	32059683	217	252		
10435	7	107171822	107187260	ENSMUSG00000020659	ENSMUSG00000020659	ENSMUSG00000020659	12	32073243	32080720	217	252		
10436	7	107088316	107145490	ENSMUSG00000020651	ENSMUSG00000020651	ENSMUSG00000020651	12	32101856	32145728	217	252		
10437	7	107007946	107048159	ENSMUSG00000020650	ENSMUSG00000020650	ENSMUSG00000020650	12	32181110	32219846	217	252		
10438	7	106991668	107006204	ENSMUSG00000020648	ENSMUSG00000020648	ENSMUSG00000020648	12	32225302	32240073	217	252		
10439	7	106630227	106991721	ENSMUSG00000035933	ENSMUSG00000035933	ENSMUSG00000035933	12	32240116	32522877	217	252		
10440	7	106897741	106903334	ENSMUSG00000044067	ENSMUSG00000044067	ENSMUSG00000044067	12	32292121	32299173	217	252		
10441	7	106596696	106630209	ENSMUSG00000002996	ENSMUSG00000002996	ENSMUSG00000002996	12	32511707	32535470	217	252		
10442	7	106472375	106589491	ENSMUSG00000002997	ENSMUSG00000002997	ENSMUSG00000002997	12	32543731	32646482	217	252		
10443	7	106292977	106334801	ENSMUSG00000020573	ENSMUSG00000020573	ENSMUSG00000020573	12	32761825	32791233	217	252		
10444	7	105390921	105461133	ENSMUSG00000035860	ENSMUSG00000035860	ENSMUSG00000035860	12	33619043	33678122	217	252		
10445	7	105035538	105310239	ENSMUSG00000020564	ENSMUSG00000020564	ENSMUSG00000020564	12	33887798	33953520	217	252		
10446	7	19701610	19715185	ENSMUSG00000020561	ENSMUSG00000020561	ENSMUSG00000020561	12	34014875	34024653	218	253		
10447	7	19151010	19151510	ENSMUSG00000046518	ENSMUSG00000046518	ENSMUSG00000046518	12	34513672	34514557	218	253		
10448	7	19121633	19123820	ENSMUSG00000035799	ENSMUSG00000035799	ENSMUSG00000035799	12	34542918	34545078	218	253		
10449	7	18502451	19002210	ENSMUSG00000004698	ENSMUSG00000004698	ENSMUSG00000004698	12	34663701	35022635	218	253		
10450	7	17800194	17946616	ENSMUSG00000020590	ENSMUSG00000020590	ENSMUSG00000020590	12	35632289	35730455	218	253		
10451	7	17304832	17349400	ENSMUSG00000019256	ENSMUSG00000019256	ENSMUSG00000019256	12	36088776	36119695	218	253		
10452	7	16865554	16888138	ENSMUSG00000036231	ENSMUSG00000036231	ENSMUSG00000036231	12	36435886	36460002	218	253		
10453	7	16652284	16712662	ENSMUSG00000020547	ENSMUSG00000020547	ENSMUSG00000020547	12	36602112	36667086	218	253		
10454	7	16605932	16651937	ENSMUSG00000036188	ENSMUSG00000036188	ENSMUSG00000036188	12	36667419	36707557	218	253		
10455	7	16573567	16587639	ENSMUSG00000020545	ENSMUSG00000020545	ENSMUSG00000020545	12	36718611	36728468	218	253		
10456	7	16467633	16471999	ENSMUSG00000036169	ENSMUSG00000036169	ENSMUSG00000036169	12	36824492	36828711	218	253		
10457	7	15617362	15692819	ENSMUSG00000036144	ENSMUSG00000036144	ENSMUSG00000036144	12	37618812	37689794	218	253		
10458	7	14153770	14847413	ENSMUSG00000036095	ENSMUSG00000036095	ENSMUSG00000036095	12	38390603	39142506	218	253		
10459	7	12576870	12659250	ENSMUSG00000002565	ENSMUSG00000002565	ENSMUSG00000002565	12	40570196	40644613	218	253		
10460	7	111908282	111918171	ENSMUSG00000071342	ENSMUSG00000071342	ENSMUSG00000071342	12	40686809	40709738	219	254		
10461	7	111877751	111903480	ENSMUSG00000001627	ENSMUSG00000001627	ENSMUSG00000001627	12	40711990	40733608	219	254		
10462	7	111633879	111770320	ENSMUSG00000055917	ENSMUSG00000055917	ENSMUSG00000055917	12	40825983	40853824	219	254		
10463	7	111155566	111431423	ENSMUSG00000035954	ENSMUSG00000035954	ENSMUSG00000035954	12	40956476	41356918	219	254		
10464	7	110090894	110948739	ENSMUSG00000056899	ENSMUSG00000056899	ENSMUSG00000056899	12	41578735	42825386	219	254		
10465	7	110518419	110552696	ENSMUSG00000036295	ENSMUSG00000036295	ENSMUSG00000036295	12	41962144	41996391	219	254		
10466	7	107997592	108002530	ENSMUSG00000014905	ENSMUSG00000014905	ENSMUSG00000014905	12	45076183	45080354	219	254		
10467	7	107899341	107953788	ENSMUSG00000036257	ENSMUSG00000036257	ENSMUSG00000036257	12	45153700	45181882	219	254		
10468	7	107575318	107884062	ENSMUSG00000020598	ENSMUSG00000020598	ENSMUSG00000020598	12	45199270	45469135	219	254		
10469	14	24348708	24588944	ENSMUSG00000046314	ENSMUSG00000046314	ENSMUSG00000046314	12	45722772	45944769	220	255		
10470	14	25984929	26136800	ENSMUSG00000021047	ENSMUSG00000021047	ENSMUSG00000021047	12	47573930	47694666	220	255		
10471	14	28304801	28308621	ENSMUSG00000020950	ENSMUSG00000020950	ENSMUSG00000020950	12	50259134	50262983	220	255		
10472	14	29116195	29466469	ENSMUSG00000002688	ENSMUSG00000002688	ENSMUSG00000002688	12	51217854	51571105	220	255		
10473	14	30098128	30158797	ENSMUSG00000035293	ENSMUSG00000035293	ENSMUSG00000035293	12	52269947	52297188	220	255		
10474	14	30161272	30274769	ENSMUSG00000020952	ENSMUSG00000020952	ENSMUSG00000020952	12	52299205	52369263	220	255		
10475	14	30413896	30429573	ENSMUSG00000020953	ENSMUSG00000020953	ENSMUSG00000020953	12	52514977	52527381	220	255		
10476	14	30434172	30565142	ENSMUSG00000020954	ENSMUSG00000020954	ENSMUSG00000020954	12	52531245	52613507	220	255		
10477	14	30639075	30746274	ENSMUSG00000035247	ENSMUSG00000035247	ENSMUSG00000035247	12	52666144	52749111	220	255		
10478	14	30832265	30941932	ENSMUSG00000035181	ENSMUSG00000035181	ENSMUSG00000035181	12	52797493	52892941	220	255		
10479	14	30984996	30996418	ENSMUSG00000020956	ENSMUSG00000020956	ENSMUSG00000020956	12	52919127	52928121	220	255		
10480	14	31100397	31399259	ENSMUSG00000035142	ENSMUSG00000035142	ENSMUSG00000035142	12	53019385	53232571	220	255		
10481	14	31616246	31698685	ENSMUSG00000035133	ENSMUSG00000035133	ENSMUSG00000035133	12	53437868	53490452	220	255		
10482	14	31868274	32372018	ENSMUSG00000061603	ENSMUSG00000061603	ENSMUSG00000061603	12	53717491	54064354	220	255		
10483	14	32478274	33340066	ENSMUSG00000021010	ENSMUSG00000021010	ENSMUSG00000021010	12	54170303	54990700	220	255		
10484	14	33463174	33490037	ENSMUSG00000035105	ENSMUSG00000035105	ENSMUSG00000035105	12	55100608	55125474	220	255		
10485	14	33971905	34001313	ENSMUSG00000044408	ENSMUSG00000044408	ENSMUSG00000044408	12	55567027	55578155	220	255		
10486	14	34054887	34078668	ENSMUSG00000054302	ENSMUSG00000054302	ENSMUSG00000054302	12	55595087	55617498	220	255		
10487	14	34100369	34169093	ENSMUSG00000005656	ENSMUSG00000005656	ENSMUSG00000005656	12	55668632	55713638	220	255		
10488	14	34249401	34253649	ENSMUSG00000062929	ENSMUSG00000062929	ENSMUSG00000062929	12	55779260	55779968	220	255		
10489	14	34291694	34414604	ENSMUSG00000035021	ENSMUSG00000035021	ENSMUSG00000035021	12	55812438	55903908	220	255		
10490	14	34624455	34661388	ENSMUSG00000021022	ENSMUSG00000021022	ENSMUSG00000021022	12	56059108	56221093	220	255		
10491	14	34661520	34813019	ENSMUSG00000021023	ENSMUSG00000021023	ENSMUSG00000021023	12	56221391	56300621	220	255		
10492	14	34831314	34856431	ENSMUSG00000021024	ENSMUSG00000021024	ENSMUSG00000021024	12	56317018	56336579	220	255		
10493	14	34940475	34943703	ENSMUSG00000021025	ENSMUSG00000021025	ENSMUSG00000021025	12	56407731	56410725	220	255		
10494	14	35072999	35076011	ENSMUSG00000045440	ENSMUSG00000045440	ENSMUSG00000045440	12	56517603	56519084	220	255		
10495	14	35077310	35348183	ENSMUSG00000021027	ENSMUSG00000021027	ENSMUSG00000021027	12	56522250	56739297	220	255		
10496	14	35837522	35859596	ENSMUSG00000021028	ENSMUSG00000021028	ENSMUSG00000021028	12	57246328	57263886	220	255		
10497	14	36055357	36059167	ENSMUSG00000001496	ENSMUSG00000001496	ENSMUSG00000001496	12	57449968	57454920	220	255		
10498	14	36118968	36121537	ENSMUSG00000058669	ENSMUSG00000058669	ENSMUSG00000058669	12	57529732	57531071	220	255		
10499	14	36200857	36215837	ENSMUSG00000001497	ENSMUSG00000001497	ENSMUSG00000001497	12	57613651	57629242	220	255		
10500	14	36219614	36711306	ENSMUSG00000035472	ENSMUSG00000035472	ENSMUSG00000035472	12	57631825	58114951	220	255		
10501	14	37128947	37134240	ENSMUSG00000035451	ENSMUSG00000035451	ENSMUSG00000035451	12	58458640	58464133	220	255		
10502	14	37150207	37507549	ENSMUSG00000073070	ENSMUSG00000073070	ENSMUSG00000073070	12	58482127	58540121	220	255		
10503	14	37334223	37381641	ENSMUSG00000046782	ENSMUSG00000046782	ENSMUSG00000046782	12	58615367	58647494	220	255		
10504	14	37746955	37752019	ENSMUSG00000035431	ENSMUSG00000035431	ENSMUSG00000035431	12	59129816	59132456	220	255		
10505	14	37793506	37794978	ENSMUSG00000045930	ENSMUSG00000045930	ENSMUSG00000045930	12	59185467	59186846	220	255		
10506	14	38570874	38642190	ENSMUSG00000020986	ENSMUSG00000020986	ENSMUSG00000020986	12	59876396	59930029	220	255		
10507	14	38653239	38675926	ENSMUSG00000060121	ENSMUSG00000060121	ENSMUSG00000060121	12	59931403	59946480	220	255		
10508	14	38686770	38709487	ENSMUSG00000020993	ENSMUSG00000020993	ENSMUSG00000020993	12	59961102	59979473	220	255		
10509	14	38714151	38721178	ENSMUSG00000020994	ENSMUSG00000020994	ENSMUSG00000020994	12	59985004	59991917	220	255		
10510	14	38772871	38792592	ENSMUSG00000035349	ENSMUSG00000035349	ENSMUSG00000035349	12	60013812	60027142	220	255		
10511	14	38936718	38971371	ENSMUSG00000035329	ENSMUSG00000035329	ENSMUSG00000035329	12	60120423	60137491	220	255		
10512	14	41146523	41443498	ENSMUSG00000035653	ENSMUSG00000035653	ENSMUSG00000035653	12	62757441	62762180	220	255		
10513	14	44436257	44446210	ENSMUSG00000047227	ENSMUSG00000047227	ENSMUSG00000047227	12	65836516	65843196	220	255		
10514	14	44467783	44500900	ENSMUSG00000020948	ENSMUSG00000020948	ENSMUSG00000020948	12	65862055	65876342	220	255		
10515	14	44501161	44613383	ENSMUSG00000035614	ENSMUSG00000035614	ENSMUSG00000035614	12	65884573	65941177	220	255		
10516	14	44635625	44654552	ENSMUSG00000035597	ENSMUSG00000035597	ENSMUSG00000035597	12	65962396	65980082	220	255		
10517	14	44674900	44739835	ENSMUSG00000055884	ENSMUSG00000055884	ENSMUSG00000055884	12	65994211	66050663	220	255		
10518	14	44742144	44792146	ENSMUSG00000047534	ENSMUSG00000047534	ENSMUSG00000047534	12	66051337	66091185	220	255		
10519	14	46190045	46190689	ENSMUSG00000060879	ENSMUSG00000060879	ENSMUSG00000060879	12	67202318	67202962	220	255		
10520	14	46380884	47213542	ENSMUSG00000034912	ENSMUSG00000034912	ENSMUSG00000034912	12	67390120	68139854	220	255		
10521	14	49135165	49151139	ENSMUSG00000034883	ENSMUSG00000034883	ENSMUSG00000034883	12	70087426	70090704	220	255		
10522	14	49155159	49157099	ENSMUSG00000049751	ENSMUSG00000049751	ENSMUSG00000049751	12	70101346	70102645	220	255		
10523	14	49157239	49159948	ENSMUSG00000043998	ENSMUSG00000043998	ENSMUSG00000043998	12	70102818	70104856	220	255		
10524	14	49180028	49224685	ENSMUSG00000020974	ENSMUSG00000020974	ENSMUSG00000020974	12	70120384	70146795	220	255		
10525	14	49229610	49289610	ENSMUSG00000051890	ENSMUSG00000051890	ENSMUSG00000051890	12	70160437	70202566	220	255		
10526	14	49304076	49319605	ENSMUSG00000020978	ENSMUSG00000020978	ENSMUSG00000020978	12	70215335	70229069	220	255		
10527	14	49320283	49389289	ENSMUSG00000020982	ENSMUSG00000020982	ENSMUSG00000020982	12	70230146	70275771	220	255		

10528	14	49429589	49431484	ENSMUSG00000044147	ENSMUSG00000044147	ENSMUSG00000044147	12	70290755	70292386	220	255		
10529	14	49654812	49767751	ENSMUSG00000034801	ENSMUSG00000034801	ENSMUSG00000034801	12	70502371	70576665	220	255		
10530	14	49782933	49848697	ENSMUSG00000020988	ENSMUSG00000020988	ENSMUSG00000020988	12	70609049	70643423	220	255		
10531	14	49855702	49872026	ENSMUSG00000054894	ENSMUSG00000054894	ENSMUSG00000054894	12	70643567	70663263	220	255		
10532	14	49954999	50069126	ENSMUSG00000034761	ENSMUSG00000034761	ENSMUSG00000034761	12	70722355	70811768	220	255		
10533	14	50096593	50169532	ENSMUSG00000021066	ENSMUSG00000021066	ENSMUSG00000021066	12	70811874	70882636	220	255		
10534	14	50170834	50204773	ENSMUSG00000021067	ENSMUSG00000021067	ENSMUSG00000021067	12	70883617	70905607	220	255		
10535	14	50256232	50367589	ENSMUSG00000021068	ENSMUSG00000021068	ENSMUSG00000021068	12	70937359	71030088	220	255		
10536	14	50408628	50441438	ENSMUSG00000071290	ENSMUSG00000071290	ENSMUSG00000071290	12	71072776	71101811	220	255		
10537	14	50441718	50480942	ENSMUSG00000021069	ENSMUSG00000021069	ENSMUSG00000021069	12	71109416	71146222	220	255		
10538	14	50515246	50631797	ENSMUSG00000021071	ENSMUSG00000021071	ENSMUSG00000021071	12	71166338	71265932	220	255		
10539	14	50776686	50792509	ENSMUSG00000021072	ENSMUSG00000021072	ENSMUSG00000021072	12	71371741	71386228	220	255		
10540	14	51188415	51265405	ENSMUSG00000048285	ENSMUSG00000048285	ENSMUSG00000048285	12	71744119	71820839	220	255		
10541	14	57835116	57909391	ENSMUSG00000048118	ENSMUSG00000048118	ENSMUSG00000048118	12	71934435	72016206	221	255	here	Sub blocks comes here
10542	14	57932386	57945172	ENSMUSG00000021078	ENSMUSG00000021078	ENSMUSG00000021078	12	72030049	72041827	221	255	sub blocks	2 -> 1
10543	14	57945121	57963971	ENSMUSG00000021079	ENSMUSG00000021079	ENSMUSG00000021079	12	72041777	72055280	221	255		
10544	14	57963873	58084591	ENSMUSG00000034601	ENSMUSG00000034601	ENSMUSG00000034601	12	72089194	72161902	221	255		
10545	14	58174612	58184789	ENSMUSG00000044548	ENSMUSG00000044548	ENSMUSG00000044548	12	72228589	72237499	221	255		
10546	14	58725152	58906224	ENSMUSG00000034574	ENSMUSG00000034574	ENSMUSG00000034574	12	72749655	72910944	221	255		
10547	14	59000213	59001697	ENSMUSG00000043398	ENSMUSG00000043398	ENSMUSG00000043398	12	72988202	72989575	221	255		
10548	14	59009159	59020826	ENSMUSG00000019718	ENSMUSG00000019718	ENSMUSG00000019718	12	72992077	73004023	221	255		
10549	14	59020979	59041182	ENSMUSG00000005078	ENSMUSG00000005078	ENSMUSG00000005078	12	73004707	73019445	221	255		
10550	14	59047021	59113255	ENSMUSG00000021086	ENSMUSG00000021086	ENSMUSG00000021086	12	73078615	73103855	221	255		
10551	14	59132447	59407310	ENSMUSG00000021087	ENSMUSG00000021087	ENSMUSG00000021087	12	73130586	73327777	221	255		
10552	14	59456184	59600030	ENSMUSG00000021090	ENSMUSG00000021090	ENSMUSG00000021090	12	73360704	73425268	221	255		
10553	14	59652411	59671280	ENSMUSG00000034501	ENSMUSG00000034501	ENSMUSG00000034501	12	73455239	73498953	221	255		
10554	14	59681255	59701900	ENSMUSG00000021094	ENSMUSG00000021094	ENSMUSG00000021094	12	73569192	73583629	221	255		
10555	14	59972428	60022567	ENSMUSG00000021098	ENSMUSG00000021098	ENSMUSG00000021098	12	73799947	73836603	221	255		
10556	14	60045622	60048282	ENSMUSG00000021099	ENSMUSG00000021099	ENSMUSG00000021099	12	73858783	73863737	221	255		
10557	14	60182506	60185933	ENSMUSG00000051367	ENSMUSG00000051367	ENSMUSG00000051367	12	73960668	73965302	221	255		
10558	14	60246010	60260545	ENSMUSG00000034460	ENSMUSG00000034460	ENSMUSG00000034460	12	74019097	74032083	221	255		
10559	14	60271223	60505149	ENSMUSG00000021103	ENSMUSG00000021103	ENSMUSG00000021103	12	74042554	74191689	221	255		
10560	14	60507922	60517528	ENSMUSG00000034442	ENSMUSG00000034442	ENSMUSG00000034442	12	74199388	74204115	221	255		
10561	14	60517633	60620183	ENSMUSG00000044712	ENSMUSG00000044712	ENSMUSG00000044712	12	74205763	74271011	221	255		
10562	14	60814159	60817712	ENSMUSG00000034435	ENSMUSG00000034435	ENSMUSG00000034435	12	74464116	74465177	221	255		
10563	14	60858186	61087443	ENSMUSG00000021108	ENSMUSG00000021108	ENSMUSG00000021108	12	74503879	74697023	221	255		
10564	14	61231992	61284729	ENSMUSG00000021109	ENSMUSG00000021109	ENSMUSG00000021109	12	74826742	74866392	221	255		
10565	14	61298920	61332898	ENSMUSG00000021113	ENSMUSG00000021113	ENSMUSG00000021113	12	74883396	74903230	221	255		
10566	14	61532294	61638177	ENSMUSG00000044912	ENSMUSG00000044912	ENSMUSG00000044912	12	74916599	75186752	221	255		
10567	14	61653950	61666105	ENSMUSG00000071265	ENSMUSG00000071265	ENSMUSG00000071265	12	75203119	75214769	221	255		
10568	14	62243052	62581709	ENSMUSG00000034402	ENSMUSG00000034402	ENSMUSG00000034402	12	75816060	76096172	221	255		
10569	14	62740898	62828312	ENSMUSG00000046768	ENSMUSG00000046768	ENSMUSG00000046768	12	76227153	76320295	221	255		
10570	14	62849395	62854316	ENSMUSG00000048982	ENSMUSG00000048982	ENSMUSG00000048982	12	76330560	76335621	221	255		
10571	14	62911108	63079832	ENSMUSG00000021051	ENSMUSG00000021051	ENSMUSG00000021051	12	76369721	76515040	221	255		
10572	14	63135250	63136413	ENSMUSG00000045690	ENSMUSG00000045690	ENSMUSG00000045690	12	76550316	76588367	221	255		
10573	14	63220690	63264526	ENSMUSG00000021054	ENSMUSG00000021054	ENSMUSG00000021054	12	76633089	76654504	221	255		
10574	14	63389454	63762901	ENSMUSG00000063450	ENSMUSG00000063450	ENSMUSG00000063450	12	76845296	77028881	221	255		
10575	14	63769608	63830831	ENSMUSG00000021055	ENSMUSG00000021055	ENSMUSG00000021055	12	77039259	77096099	221	255		
10576	14	63924821	63996474	ENSMUSG00000021048	ENSMUSG00000021048	ENSMUSG00000021048	12	77174138	77238642	221	255		
10577	14	64003568	64006178	ENSMUSG00000021057	ENSMUSG00000021057	ENSMUSG00000021057	12	77246657	77248894	221	255		
10578	14	64023310	64040316	ENSMUSG00000056459	ENSMUSG00000056459	ENSMUSG00000056459	12	77267784	77287839	221	255		
10579	14	64041174	64070161	ENSMUSG00000033454	ENSMUSG00000033454	ENSMUSG00000033454	12	77289360	77307583	221	255		
10580	14	64077213	64079703	ENSMUSG00000059970	ENSMUSG00000059970	ENSMUSG00000059970	12	77323016	77325778	221	255		
10581	14	64086373	64125849	ENSMUSG00000052221	ENSMUSG00000052221	ENSMUSG00000052221	12	77336488	77358145	221	255		
10582	14	64240946	64280810	ENSMUSG00000052609	ENSMUSG00000052609	ENSMUSG00000052609	12	77452422	77497880	221	255		
10583	14	64285426	64416354	ENSMUSG00000021061	ENSMUSG00000021061	ENSMUSG00000021061	12	77499328	77629387	221	255		
10584	14	64450893	64471666	ENSMUSG00000033395	ENSMUSG00000033395	ENSMUSG00000033395	12	77684413	77702012	221	255		
10585	14	64475962	64479197	ENSMUSG00000042808	ENSMUSG00000042808	ENSMUSG00000042808	12	77711490	77714215	221	255		
10586	14	64523260	64599122	ENSMUSG00000033373	ENSMUSG00000033373	ENSMUSG00000033373	12	77756307	77840254	221	255		
10587	14	64947063	65279585	ENSMUSG00000021065	ENSMUSG00000021065	ENSMUSG00000021065	12	78156965	78394800	221	255		
10588	14	66022862	66035023	ENSMUSG00000071254	ENSMUSG00000071254	ENSMUSG00000071254	12	79116014	79121307	221	255		
10589	14	66043878	66718268	ENSMUSG00000047454	ENSMUSG00000047454	ENSMUSG00000047454	12	79145574	79603578	221	255		
10590	14	66725931	66765014	ENSMUSG00000056987	ENSMUSG00000056987	ENSMUSG00000056987	12	79629111	79638477	221	255		
10591	14	66777774	66872288	ENSMUSG00000021112	ENSMUSG00000021112	ENSMUSG00000021112	12	79667764	79759414	221	255		
10592	14	66830841	66896261	ENSMUSG00000021114	ENSMUSG00000021114	ENSMUSG00000021114	12	79761822	79780478	221	255		
10593	14	66896787	66922986	ENSMUSG00000021116	ENSMUSG00000021116	ENSMUSG00000021116	12	79780910	79804371	221	255		
10594	14	66923458	66948614	ENSMUSG00000021118	ENSMUSG00000021118	ENSMUSG00000021118	12	79807532	79825790	221	255		
10595	14	67069785	67126004	ENSMUSG00000060716	ENSMUSG00000060716	ENSMUSG00000060716	12	79948034	80000486	221	255		
10596	14	67125784	67136733	ENSMUSG00000021120	ENSMUSG00000021120	ENSMUSG00000021120	12	79999504	80008510	221	255		
10597	14	67156332	67188190	ENSMUSG00000021125	ENSMUSG00000021125	ENSMUSG00000021125	12	80049664	80075138	221	255		
10598	14	67213274	67232213	ENSMUSG00000066441	ENSMUSG00000066441	ENSMUSG00000066441	12	80093175	80111133	221	255		
10599	14	67258943	67270920	ENSMUSG00000021123	ENSMUSG00000021123	ENSMUSG00000021123	12	80127754	80141501	221	255		
10600	14	67282991	67353057	ENSMUSG00000066440	ENSMUSG00000066440	ENSMUSG00000066440	12	80151187	80215093	221	255		
10601	14	67360014	68131073	ENSMUSG00000059060	ENSMUSG00000059060	ENSMUSG00000059060	12	80219404	80722110	221	255		
10602	14	68325077	68331206	ENSMUSG00000021127	ENSMUSG00000021127	ENSMUSG00000021127	12	81026600	81031853	221	255		
10603	14	68410793	68515747	ENSMUSG00000015143	ENSMUSG00000015143	ENSMUSG00000015143	12	81086385	81179156	221	255		
10604	14	68590314	68689473	ENSMUSG00000049106	ENSMUSG00000049106	ENSMUSG00000049106	12	81254697	81355286	221	255		
10605	14	68727981	68778824	ENSMUSG00000032705	ENSMUSG00000032705	ENSMUSG00000032705	12	81394617	81416970	221	255		
10606	14	68796668	68890936	ENSMUSG00000021130	ENSMUSG00000021130	ENSMUSG00000021130	12	81437319	81522740	221	255		
10607	14	68935208	68998851	ENSMUSG00000048833	ENSMUSG00000048833	ENSMUSG00000048833	12	81563087	81602184	221	255		
10608	14	69058753	69064862	ENSMUSG00000066438	ENSMUSG00000066438	ENSMUSG00000066438	12	81636069	81641309	221	255		
10609	14	69107969	69110092	ENSMUSG00000062961	ENSMUSG00000062961	ENSMUSG00000062961	12	81674635	81679535	221	255		
10610	14	69148066	69251612	ENSMUSG00000021133	ENSMUSG00000021133	ENSMUSG00000021133	12	81709407	81799683	221	255		
10611	14	69312305	69333707	ENSMUSG00000021135	ENSMUSG00000021135	ENSMUSG00000021135	12	81872040	81887553	221	255		
10612	14	69415896	69568836	ENSMUSG00000021136	ENSMUSG00000021136	ENSMUSG00000021136	12	81945676	82105258	221	255		
10613	14	69582417	69704954	ENSMUSG00000053302	ENSMUSG00000053302	ENSMUSG00000053302	12	82118342	82234891	221	255		
10614	14	69993970	69996375	ENSMUSG00000008438	ENSMUSG00000008438	ENSMUSG00000008438	12	82477432	82487322	221	255		
10615	14	70120059	70137136	ENSMUSG00000002679	ENSMUSG00000002679	ENSMUSG00000002679	12	82492757	82513850	221	255		
10616	14	70178257	70211830	ENSMUSG00000042734	ENSMUSG00000042734	ENSMUSG00000042734	12	82550217	82583789	221	255		
10617	14	70264746	70345641	ENSMUSG00000042724	ENSMUSG00000042724	ENSMUSG00000042724	12	82640437	82699722	221	255		
10618	14	70443875	70646623	ENSMUSG00000021140	ENSMUSG00000021140	ENSMUSG00000021140	12	82779007	82919772	221	255		
10619	14	71065795	71275871	ENSMUSG00000042700	ENSMUSG00000042700	ENSMUSG00000042700	12	83088874	83369119	221	255		
10620	14	71501262	72099211	ENSMUSG00000021219	ENSMUSG00000021219	ENSMUSG00000021219	12	83570677	84052588	221	255		
10621	14	72145495	72430562	ENSMUSG00000021221	ENSMUSG00000021221	ENSMUSG00000021221	12	84188281	84260507	221	255		
10622	14	72505913	72563600	ENSMUSG00000042628	ENSMUSG00000042628	ENSMUSG00000042628	12	84436306	84486862	221	255		
10623	14	72595021	72657740	ENSMUSG00000010608	ENSMUSG00000010608	ENSMUSG00000010608	12	84549243	84572823	221	255		
10624	14	72672932	72756862	ENSMUSG00000019969	ENSMUSG00000019969	ENSMUSG00000019969	12	84577950	84624841	221	255		
10625	14	72774008	72811098	ENSMUSG00000021223	ENSMUSG00000021223	ENSMUSG00000021223	12	84653382	84682132	221	255		
10626	14	72811671	72892230	ENSMUSG00000021224	ENSMUSG00000021224	ENSMUSG00000021224	12	84685187	84732091	221	255		
10627	14	73014949	73095404	ENSMUSG00000071238	ENSMUSG00000071238	ENSMUSG00000071238	12	84859536	84870229	221	255		
10628	14	73181455	73232937	ENSMUSG00000042523	ENSMUSG00000042523	ENSMUSG00000042523	12	85004119	85028205	221	255		
10629	14	73249034	73250095	ENSMUSG00000054383	ENSMUSG00000054383	ENSMUSG00000054383	12	85036614	85037675	221	255		
10630	14	73251587	73296745	ENSMUSG00000042507	ENSMUSG00000042507	ENSMUSG00000042507	12	85042660	85083778	221	255		
10631	14	73388424	73421915	ENSMUSG00000072946	ENSMUSG00000072946	ENSMUSG00000072946	12	85175062	85205580	221	255		
10632	14	73423339	73468556	ENSMUSG00000042472	ENSMUSG00000042472	ENSMUSG00000042472	12	85206862	85233472	221	255		
10633	14	73470458	73486588	ENSMUSG00000021234	ENSMUSG00000021234	ENSMUSG00000021234	12	85235065	85251571	221	255		
10634	14	73486709	73499564	ENSMUSG00000021235	ENSMUSG00000021235	ENSMUSG00000021235	12	85251744	85263544	221	255		
10635	14	73502946	73552387	ENSMUSG00000021236	ENSMUSG00000021236	ENSMUSG00000021236	12	85263605	85295224	221	255		
10636	14	73582542	73602546	ENSMUSG00000057265	ENSMUSG00000057265	ENSMUSG00000057265	12	85298930	85320084	221	255		
10637	14	73596644	73620949	ENSMUSG00000021238	ENSMUSG00000021238	ENSMUSG00000021238	12	85320472	85340751	221	255		
10638	14	73775928	73799187	ENSMUSG00000021239	ENSMUSG00000021239	ENSMUSG00000021239	12	85459588	85485204	221	255		
10639	14	73821902	73839420	ENSMUSG00000021240	ENSMUSG00000021240	ENSMUSG00000021240	12	85492281	85507118	221	255		
10640	14	73884948	73896461	ENSMUSG00000071235	ENSMUSG00000071235	ENSMUSG00000071235	12	85538221	85541203	221	255		
10641	14	73943991	73946200	ENSMUSG00000071234	ENSMUSG00000071234	ENSMUSG00000071234	12	85567026	85588555	221	255		
10642	14	74016397	74029837	ENSMUSG00000021242	ENSMUSG00000021242	ENSMUSG00000021242	12	85644305	85662860	221	255		
10643	14	74030212	74031822	ENSMUSG00000021241	ENSMUSG00000021241	ENSMUSG00000021241	12	85663018	85664465	221	255		
10644	14	74034642	74148787	ENSMUSG00000002020	ENSMUSG00000002020	ENSMUSG00000002020	12	85672960	85766280	221	255		
10645	14	74200176	74221149	ENSMUSG00000042350	ENSMUSG00000042350	ENSMUSG00000042350	12	85807503	85859947	221	255		
10646	14	74299946	74372621	ENSMUSG00000021244	ENSMUSG00000021244	ENSMUSG00000021244	12	85885593	85958865	221	255		
10647	14	74391582	74400284	ENSMUSG00000042320	ENSMUSG00000042320	ENSMUSG00000042320	12	85975917	85984530	221	255		
10648	14	74418372	74440196	ENSMUSG00000004789	ENSMUSG00000004789	ENSMUSG00000004789	12	85999940	86023948	221	255		
10649	14	74442100	74458630	ENSMUSG00000019235	ENSMUSG00000019235	ENSMUSG00000019235	12	86024720	86040357	221	255		
10650	14	74479162	74491698	ENSMUSG00000004791	ENSMUSG00000004791	ENSMUSG00000004791	12	86058457	86066066	221	255		
10651	14	74539365	74546045	ENSMUSG00000004788	ENSMUSG00000004788	ENSMUSG00000004788	12	86108616	86115731	221	255		
10652	14	74553538	74586111	ENSMUSG00000021245	ENSMUSG00000021245	ENSMUSG00000021245	12	86123632	86159679	221	255		
10653	14	74589683	74600489	ENSMUSG00000008822	ENSMUSG00000008822	ENSMUSG00000008822	12	86167997	86169521	221	255		
10654	14	74607030	74614248	ENSMUSG00000045064	ENSMUSG00000045064	ENSMUSG00000045064	12	86177756	86188461	221	255		
10655	14	74618575	74663483	ENSMUSG00000034290	ENSMUSG00000034290	ENSMUSG00000034290	12	86188618	86228449	221	255		
10656	14	74815284	74818685	ENSMUSG00000021250	ENSMUSG00000021250	ENSMUSG00000021250	12	86363004	86366372	221	255		
10657	14	74964813	75006908	ENSMUSG00000034271	ENSMUSG00000034271	ENSMUSG00000034271	12	86488208	86528980	221	255		
10658	14	75058542	75083086	ENSMUSG00000034266	ENSMUSG00000034266	ENSMUSG00000034266	12	86575781	86598189	221	255		
10659	14	75114713	75184244	ENSMUSG00000034258	ENSMUSG00000034258	ENSMUSG00000034258	12	86635642	86702688	221	255		
10660	14	75187010	75197277	ENSMUSG00000021252	ENSMUSG00000021252	ENSMUSG00000021252	12	86704551	86713670	221	255		
10661	14	75197374	75491174	ENSMUSG00000012609	ENSMUSG00000012609	ENSMUSG00000012609	12	86715654	86942259	221	255		
10662	14	75494195	75517242	ENSMUSG00000021253	ENSMUSG00000021253	ENSMUSG00000021253	12	86945904	86968101	221	255		
10663	14	75521878	75619842	ENSMUSG00000007867	ENSMUSG00000007867	ENSMUSG00000007867	12	86971666	87051561	221	255		
10664	14	75688012	75738887	ENSMUSG00000021254	ENSMUSG00000021254	ENSMUSG00000021254	12	87131004	87180886	221	255		
10665	14	75943634	76036825	ENSMUSG00000021255	ENSMUSG00000021255	ENSMUSG00000021255	12	87250220	87410725	221	255		
10666	14	76298285	76319107	ENSMUSG00000021256	ENSMUSG00000021256	ENSMUSG00000021256	12	87567803	87581194	221	255		
10667	14	76323341	76348983	ENSMUSG00000021257	ENSMUSG00000021257	ENSMUSG00000021257	12	87589605	87615574	221	255		
10668	14	76367358	76404973	ENSMUSG00000059114	ENSMUSG00000059114	ENSMUSG00000059114	12	87626834	87638234	221	255		
10669	14	76561498	76563888	ENSMUSG00000034168	ENSMUSG00000034168	ENSMUSG00000034168	12	87770673	87773000	221	255		
10670	14	76634331	76653382	ENSMUSG00000034157	ENSMUSG00000034157	ENSMUSG00000034157	12	87836517	87854465	221	255		
10671	14	76669804	76675834	ENSMUSG00000071223	ENSMUSG00000071223	ENSMUSG00000071223	12	87877378	87877785	221	255		
10672	14	76717923	76795591	ENSMUSG00000034145	ENSMUSG00000034145	ENSMUSG00000034145	12	87915597	87979140	221	255		
10673	14	76801581	76807408	ENSMUSG00000021032	ENSMUSG00000021032	ENSMUSG00000021032	12	87986634	87991642	221	255		
10674	14	76811054	76856978	ENSMUSG00000034126	ENSMUSG00000034126	ENSMUSG00000034126	12	87995969	88037005	221	255		
10675	14	76857107	76867692	ENSMUSG00000021033	ENSMUSG00000021033	ENSMUSG00000021033	12	88036821	88053826	221	255		
10676	14	76871119	76913191	ENSMUSG00000034111	ENSMUSG00000034111	ENSMUSG00000034111	12	88055345	88089332	221	255		
10677	14	76930120	76959132	ENSMUSG00000072919	ENSMUSG00000072919	ENSMUSG00000072919	12	88110358	88122659	221	255		
10678	14	76962771	76993697	ENSMUSG00000021038	ENSMUSG00000021038	ENSMUSG00000021038	12	88128919	88153100	221	255		
10679	14	76994155	77005560	ENSMUSG00000021037	ENSMUSG00000021037	ENSMUSG00000021037	12	88155811	88163081	221	255		
10680	14	77010493	77034889	ENSMUSG00000050671	ENSMUSG00000050671	ENSMUSG00000050671	12	88169118	88176165	221	255		
10681	14	77043025	77152863	ENSMUSG00000021036	ENSMUSG00000021036	ENSMUSG00000021036	12	88196992	88277333	221	255		
10682	14	77244181	77253690	ENSMUSG00000021040	ENSMUSG00000021040	ENSMUSG00000021040	12	88333015	88339021	221	255		
10683	14	77336222	77470047	ENSMUSG00000021044	ENSMUSG00000021044	ENSMUSG00000021044	12	88873809	88975198	221	255		
10684	14	79738694	79747596	ENSMUSG00000007682	ENSMUSG00000007682	ENSMUSG00000007682	12	91134815	91143674	221	255		
10685	14	80032577	80475637	ENSMUSG00000061533	ENSMUSG00000061533	ENSMUSG00000061533	12	91424840	91772046	221	255		
10686	14	80491622	80682399	ENSMUSG00000020963	ENSMUSG00000020963	ENSMUSG00000020963	12	91806608	91945435	221	255		
10687	14	80716147	80757047	ENSMUSG00000020962	ENSMUSG00000020962	ENSMUSG00000020962	12	91965226	91995136	221	255		
10688	14	80806662	80934680	ENSMUSG00000020961	ENSMUSG00000020961	ENSMUSG00000020961	12	92044099	92191993	221	255		
10689	14	81007646	81069886	ENSMUSG00000020964	ENSMUSG00000020964	ENSMUSG00000020964	12	92211600	92254714	221	255		
10690	14	85066241	85164022	ENSMUSG00000047414	ENSMUSG00000047414	ENSMUSG00000047414	12	96088076	96181037	221	255		
10691	14	87469113	87529596	ENSMUSG00000021003	ENSMUSG00000021003	ENSMUSG00000021003	12	98603354	98660383	221	255		
10692	14	87541249	87548164	ENSMUSG00000021886	ENSMUSG00000021886	ENSMUSG00000021886	12	98669707	98677682	221	255		
10693	14	87721008	87859100	ENSMUSG00000033854	ENSMUSG00000033854	ENSMUSG00000033854	12	98835857	98919883	221	255		
10694	14	87921764	87974550	ENSMUSG00000021007	ENSMUSG00000021007	ENSMUSG00000021007	12	99029207	99070860	221	255		
10695	14	88003870	88086719	ENSMUSG00000021009	ENSMUSG00000021009	ENSMUSG00000021009	12	99077792	99135644	221	255		
10696	14	88360753	88414084	ENSMUSG00000021013	ENSMUSG00000021013	ENSMUSG00000021013	12	99321662	99384288	221	255		
10697	14	89331617	89490786	ENSMUSG00000021176	ENSMUSG00000021176	ENSMUSG00000021176	12	100118581	100284492	221	255		
10698	14	89491999	89580859	ENSMUSG00000021177	ENSMUSG00000021177	ENSMUSG00000021177	12	100292223	100356264	221	255		
10699	14	89597862	89721952	ENSMUSG00000045404	ENSMUSG00000045404	ENSMUSG00000045404	12	100366958	100463726	221	255		
10700	14	89814151	89868032	ENSMUSG00000021179	ENSMUSG00000021179	ENSMUSG00000021179	12	100526503	100560653	221	255		
10701	14	90076686	90352514	ENSMUSG00000033530	ENSMUSG00000033530	ENSMUSG00000033530	12	100701847	100921840	221	255		
10702	14	90406925	90596746	ENSMUSG00000021180	ENSMUSG00000021180	ENSMUSG00000021180	12	100950828	101126078	221	255		
10703	14	90650536	90761449	ENSMUSG00000021185	ENSMUSG00000021185	ENSMUSG00000021185	12	101180173	101273660	221	255		
10704	14	90768630	90789977	ENSMUSG00000047415	ENSMUSG00000047415	ENSMUSG00000047415	12	101277735	101288931	221	255		
10705	14	90808725	90953787	ENSMUSG00000021182	ENSMUSG00000021182	ENSMUSG00000021182	12	101313750	101430033	221	255		
10706	14	90993713	91046566	ENSMUSG00000041846	ENSMUSG00000041846	ENSMUSG00000041846	12	101440562	101484450	221	255		
10707	14	91116871	91268166	ENSMUSG00000047014	ENSMUSG00000047014	ENSMUSG00000047014	12	101810379	102026792	221	255		
10708	14	91318692	91403610	ENSMUSG00000021187	ENSMUSG00000021187	ENSMUSG00000021187	12	102050117	102110540	221	255		
10709	14	91405511	91483788	ENSMUSG00000021186	ENSMUSG00000021186	ENSMUSG00000021186	12	102147615	102215723	221	255		
10710	14	91505614	91576139	ENSMUSG00000021188	ENSMUSG00000021188	ENSMUSG00000021188	12	102238599	102313878	221	255		
10711	14	91598887	91642707	ENSMUSG00000021189	ENSMUSG00000021189	ENSMUSG00000021189	12	102320604	102359232	221	255		
10712	14	91658082	91700296	ENSMUSG00000041781	ENSMUSG00000041781	ENSMUSG00000041781	12	102377049	102406579	221	255		
10713	14	91859955	92032348	ENSMUSG00000041771	ENSMUSG00000041771	ENSMUSG00000041771	12	102530469	102667799	221	255		
10714	14	92049890	92225085	ENSMUSG00000044456	ENSMUSG00000044456	ENSMUSG00000044456	12	102684124	102791901	221	255		
10715	14	92239910	92284765	ENSMUSG00000021190	ENSMUSG00000021190	ENSMUSG00000021190	12	102795144	102840747	221	255		
10716	14	92330403	92376057	ENSMUSG00000021192	ENSMUSG00000021192	ENSMUSG00000021192	12	102873078	102898957	221	255		
10717	14	92459259	92471380	ENSMUSG00000021194	ENSMUSG00000021194	ENSMUSG00000021194	12	102956019	102966077	221	255		
10718	14	92475960	92651980	ENSMUSG00000057963	ENSMUSG00000057963	ENSMUSG00000057963	12	102969633	103105919	221	255		
10719	14	92718295	92721002	ENSMUSG00000041716	ENSMUSG00000041716	ENSMUSG00000041716	12	103143124	103159722	221	255		
10720	14	92721082	92723186	ENSMUSG00000046675	ENSMUSG00000046675	ENSMUSG00000046675	12	103144808	103146447	221	255		
10721	14	92743355	92765306	ENSMUSG00000041712	ENSMUSG00000041712	ENSMUSG00000041712	12	103159017	103178751	221	255		
10722	14	92773649	92869138	ENSMUSG00000041702	ENSMUSG00000041702	ENSMUSG00000041702	12	103185688	103279456	221	255		

10723	14	92869324	93243442	ENSMUSG00000021198	ENSMUSG00000021198	ENSMUSG00000021198	12	103475818	103584893	221	255		
10724	14	93257137	93323858	ENSMUSG00000041669	ENSMUSG00000041669	ENSMUSG00000041669	12	103597958	103643196	221	255		
10725	14	93455011	93465697	ENSMUSG00000048754	ENSMUSG00000048754	ENSMUSG00000048754	12	103716885	103717766	221	255		
10726	14	93470266	93493520	ENSMUSG00000021200	ENSMUSG00000021200	ENSMUSG00000021200	12	103722192	103757051	221	255		
10727	14	93562477	93585025	ENSMUSG00000021203	ENSMUSG00000021203	ENSMUSG00000021203	12	103801246	103805482	221	255		
10728	14	93587037	93615841	ENSMUSG00000041645	ENSMUSG00000041645	ENSMUSG00000041645	12	103809032	103826897	221	255		
10729	14	93646832	93652786	ENSMUSG00000064215	ENSMUSG00000064215	ENSMUSG00000064215	12	103835261	103841257	221	255		
10730	14	93710402	93815825	ENSMUSG00000021209	ENSMUSG00000021209	ENSMUSG00000021209	12	103933670	104011734	221	255		
10731	14	93819407	93829361	ENSMUSG00000061947	ENSMUSG00000061947	ENSMUSG00000061947	12	104015836	104032467	221	255		
10732	14	93840339	93859441	ENSMUSG00000060807	ENSMUSG00000060807	ENSMUSG00000060807	12	104047683	104058140	221	255		
10733	14	93900404	93902788	ENSMUSG00000021081	ENSMUSG00000021081	ENSMUSG00000021081	12	104090683	104096579	221	255		
10734	14	93978585	93988871	ENSMUSG00000063232	ENSMUSG00000063232	ENSMUSG00000063232	12	104381301	104390971	221	255		
10735	14	93998813	94012262	ENSMUSG00000058260	ENSMUSG00000058260	ENSMUSG00000058260	12	104397670	104414702	221	255		
10736	14	94023374	94053934	ENSMUSG00000041567	ENSMUSG00000041567	ENSMUSG00000041567	12	104429819	104445493	221	255		
10737	14	94123453	94129204	ENSMUSG00000041550	ENSMUSG00000041550	ENSMUSG00000041550	12	104502163	104507187	221	255		
10738	14	94304314	94306252	ENSMUSG00000021095	ENSMUSG00000021095	ENSMUSG00000021095	12	104872259	104874286	221	255		
10739	14	94622319	94693512	ENSMUSG00000041415	ENSMUSG00000041415	ENSMUSG00000041415	12	105092486	105152987	221	255		
10740	14	94727618	94855955	ENSMUSG00000021097	ENSMUSG00000021097	ENSMUSG00000021097	12	105172807	105266105	221	255		
10741	14	94953699	95011926	ENSMUSG00000054150	ENSMUSG00000054150	ENSMUSG00000054150	12	105333080	105399727	221	255		
10742	14	95071076	95080808	ENSMUSG00000021102	ENSMUSG00000021102	ENSMUSG00000021102	12	105433740	105443278	221	255		
10743	14	95246058	95250201	ENSMUSG00000041359	ENSMUSG00000041359	ENSMUSG00000041359	12	105617803	105623764	221	255		
10744	14	95740950	95780536	ENSMUSG00000021070	ENSMUSG00000021070	ENSMUSG00000021070	12	105964222	105994121	221	255		
10745	14	95799760	95800847	ENSMUSG00000041347	ENSMUSG00000041347	ENSMUSG00000041347	12	106005141	106006478	221	255		
10746	14	95820837	95853254	ENSMUSG00000041341	ENSMUSG00000041341	ENSMUSG00000041341	12	106017184	106047879	221	255		
10747	14	95861778	95899431	ENSMUSG00000046394	ENSMUSG00000046394	ENSMUSG00000046394	12	106060314	106086250	221	255		
10748	14	95899616	95923376	ENSMUSG00000044715	ENSMUSG00000044715	ENSMUSG00000044715	12	106086491	106104097	221	255		
10749	14	95928201	96024866	ENSMUSG00000041323	ENSMUSG00000041323	ENSMUSG00000041323	12	106107032	106183497	221	255		
10750	14	96038471	96103178	ENSMUSG00000021111	ENSMUSG00000021111	ENSMUSG00000021111	12	106181750	106239989	221	255		
10751	14	96333437	96417703	ENSMUSG00000021115	ENSMUSG00000021115	ENSMUSG00000021115	12	106411313	106478460	221	255		
10752	14	98705377	98807575	ENSMUSG00000048251	ENSMUSG00000048251	ENSMUSG00000048251	12	108361448	108451155	221	255		
10753	14	99028768	99046869	ENSMUSG00000021258	ENSMUSG00000021258	ENSMUSG00000021258	12	108634177	108650508	221	255		
10754	14	99051336	99140049	ENSMUSG00000071162	ENSMUSG00000071162	ENSMUSG00000071162	12	108722143	108722940	221	255		
10755	14	99181233	99205364	ENSMUSG00000021260	ENSMUSG00000021260	ENSMUSG00000021260	12	108754413	108777395	221	255		
10756	14	99220407	99263390	ENSMUSG00000021259	ENSMUSG00000021259	ENSMUSG00000021259	12	108782165	108810040	221	255		
10757	14	99329498	99478146	ENSMUSG00000058070	ENSMUSG00000058070	ENSMUSG00000058070	12	108858462	108987370	221	255		
10758	14	99601504	99680325	ENSMUSG00000021262	ENSMUSG00000021262	ENSMUSG00000021262	12	109096120	109135873	221	255		
10759	14	99682512	99695712	ENSMUSG00000021263	ENSMUSG00000021263	ENSMUSG00000021263	12	109138035	109150113	221	255		
10760	14	99774855	99814557	ENSMUSG00000021264	ENSMUSG00000021264	ENSMUSG00000021264	12	109241118	109264439	221	255		
10761	14	99827213	99842613	ENSMUSG00000021265	ENSMUSG00000021265	ENSMUSG00000021265	12	109273685	109283683	221	255		
10762	14	99859428	99866467	ENSMUSG00000048856	ENSMUSG00000048856	ENSMUSG00000048856	12	109298936	109304622	221	255		
10763	14	99869878	99912433	ENSMUSG00000021266	ENSMUSG00000021266	ENSMUSG00000021266	12	109308119	109341920	221	255		
10764	14	99912563	100066361	ENSMUSG00000072839	ENSMUSG00000072839	ENSMUSG00000072839	12	109345705	109346532	221	255		
10765	14	100073243	100105884	ENSMUSG00000040867	ENSMUSG00000040867	ENSMUSG00000040867	12	109479994	109486834	221	255		
10766	14	100263159	100270986	ENSMUSG00000040856	ENSMUSG00000040856	ENSMUSG00000040856	12	109901030	109908498	221	255		
10767	14	101097441	101099540	ENSMUSG00000075707	ENSMUSG00000075707	ENSMUSG00000075707	12	110727117	110727953	221	255		
10768	14	101297975	101463775	ENSMUSG00000017843	ENSMUSG00000017843	ENSMUSG00000017843	12	110933666	111028359	221	255		
10769	14	101500737	101586887	ENSMUSG00000018707	ENSMUSG00000018707	ENSMUSG00000018707	12	111049259	111114731	221	255		
10770	14	101764932	101841284	ENSMUSG00000056458	ENSMUSG00000056458	ENSMUSG00000056458	12	111255848	111282051	221	255		
10771	14	101867729	101878457	ENSMUSG00000021271	ENSMUSG00000021271	ENSMUSG00000021271	12	111298113	111316720	221	255		
10772	14	101878709	101895685	ENSMUSG00000021276	ENSMUSG00000021276	ENSMUSG00000021276	12	111320970	111331872	221	255		
10773	14	101899131	102038562	ENSMUSG00000021275	ENSMUSG00000021275	ENSMUSG00000021275	12	111337119	111420199	221	255		
10774	14	102042936	102044907	ENSMUSG00000037904	ENSMUSG00000037904	ENSMUSG00000037904	12	111423165	111426847	221	255		
10775	14	102128986	102262596	ENSMUSG00000037896	ENSMUSG00000037896	ENSMUSG00000037896	12	111529531	111563702	221	255		
10776	14	102406268	102441895	ENSMUSG00000021277	ENSMUSG00000021277	ENSMUSG00000021277	12	111614356	111710254	221	255		
10777	14	102458757	102467282	ENSMUSG00000021278	ENSMUSG00000021278	ENSMUSG00000021278	12	111718919	111724234	221	255		
10778	14	102468470	102593552	ENSMUSG00000021279	ENSMUSG00000021279	ENSMUSG00000021279	12	111740787	111825167	221	255		
10779	14	102636310	102646627	ENSMUSG00000021280	ENSMUSG00000021280	ENSMUSG00000021280	12	111865253	111878990	221	255		
10780	14	102662417	102673529	ENSMUSG00000021281	ENSMUSG00000021281	ENSMUSG00000021281	12	111892752	111902825	221	255		
10781	14	102921610	103039916	ENSMUSG00000007411	ENSMUSG00000007411	ENSMUSG00000007411	12	112022344	112104027	221	255		
10782	14	103055749	103058923	ENSMUSG00000001270	ENSMUSG00000001270	ENSMUSG00000001270	12	112117170	112120108	221	255		
10783	14	103065301	103071503	ENSMUSG00000060950	ENSMUSG00000060950	ENSMUSG00000060950	12	112125951	112131708	221	255		
10784	14	103092642	103098097	ENSMUSG00000049792	ENSMUSG00000049792	ENSMUSG00000049792	12	112157303	112160986	221	255		
10785	14	103099084	103128261	ENSMUSG00000037787	ENSMUSG00000037787	ENSMUSG00000037787	12	112161077	112182025	221	255		
10786	14	103165331	103247461	ENSMUSG00000021288	ENSMUSG00000021288	ENSMUSG00000021288	12	112206687	112254482	221	255		
10787	14	103233708	103251536	ENSMUSG00000021287	ENSMUSG00000021287	ENSMUSG00000021287	12	112251001	112261649	221	255		
10788	14	103251913	103269757	ENSMUSG00000021286	ENSMUSG00000021286	ENSMUSG00000021286	12	112262048	112276198	221	255		
10789	14	103269842	103383649	ENSMUSG00000021285	ENSMUSG00000021285	ENSMUSG00000021285	12	112276265	112320250	221	255		
10790	14	103501583	103588471	ENSMUSG00000054003	ENSMUSG00000054003	ENSMUSG00000054003	12	112441678	112516662	221	255		
10791	14	103621776	103643325	ENSMUSG00000037686	ENSMUSG00000037686	ENSMUSG00000037686	12	112554664	112575367	221	255		
10792	14	103675217	103716984	ENSMUSG00000021294	ENSMUSG00000021294	ENSMUSG00000021294	12	112621966	112629554	221	255		
10793	14	104117101	104127228	ENSMUSG00000043122	ENSMUSG00000043122	ENSMUSG00000043122	12	112937275	112946924	221	255		
10794	14	104131465	104142142	ENSMUSG00000054013	ENSMUSG00000054013	ENSMUSG00000054013	12	112947998	112958980	221	255		
10795	14	104247528	104256205	ENSMUSG00000037679	ENSMUSG00000037679	ENSMUSG00000037679	12	113052568	113063360	221	255		
10796	14	104267269	104284690	ENSMUSG00000011148	ENSMUSG00000011148	ENSMUSG00000011148	12	113067901	113089165	221	255		
10797	14	104306734	104333125	ENSMUSG00000001729	ENSMUSG00000001729	ENSMUSG00000001729	12	113101636	113122084	221	255		
10798	14	104415282	104434131	ENSMUSG00000072825	ENSMUSG00000072825	ENSMUSG00000072825	12	113170042	113194399	221	255		
10799	14	104462232	104470618	ENSMUSG00000052160	ENSMUSG00000052160	ENSMUSG00000052160	12	113208463	113216794	221	255		
10800	14	104474636	104491286	ENSMUSG00000072812	ENSMUSG00000072812	ENSMUSG00000072812	12	113220714	113223026	221	255		
10801	14	104523814	104532138	ENSMUSG00000037594	ENSMUSG00000037594	ENSMUSG00000037594	12	113256783	113264053	221	255		
10802	14	104547326	104558538	ENSMUSG00000047832	ENSMUSG00000047832	ENSMUSG00000047832	12	113268039	113305051	221	255		
10803	14	104586784	104602799	ENSMUSG00000021298	ENSMUSG00000021298	ENSMUSG00000021298	12	113299312	113308700	221	255		
10804	14	104679121	104706206	ENSMUSG00000002799	ENSMUSG00000002799	ENSMUSG00000002799	12	113356398	113377303	221	255		
10805	14	104710322	104718685	ENSMUSG00000002804	ENSMUSG00000002804	ENSMUSG00000002804	12	113382540	113389932	221	255		
10806	14	104746670	104838374	ENSMUSG00000011158	ENSMUSG00000011158	ENSMUSG00000011158	12	113407670	113448429	221	255		
10807	14	104786053	104788466	ENSMUSG00000002803	ENSMUSG00000002803	ENSMUSG00000002803	12	113424371	113426753	221	255		
10808	14	104852126	104932592	ENSMUSG00000021143	ENSMUSG00000021143	ENSMUSG00000021143	12	113462342	113519946	221	255		
10809	14	104957393	105008050	ENSMUSG00000021144	ENSMUSG00000021144	ENSMUSG00000021144	12	113546226	113584476	221	255		
10810	14	105028637	105036564	ENSMUSG00000037466	ENSMUSG00000037466	ENSMUSG00000037466	12	113604229	113612352	221	255		
10811	14	105066103	105067584	ENSMUSG00000049036	ENSMUSG00000049036	ENSMUSG00000049036	12	113633733	113637326	221	255		
10812	14	105137448	105139110	ENSMUSG00000021153	ENSMUSG00000021153	ENSMUSG00000021153	12	113718988	113721054	221	255		
10813	14	106353103	106354309	ENSMUSG00000072803	ENSMUSG00000072803	ENSMUSG00000072803	12	114588503	114588793	221	255		
10814	7	158513628	158630410	ENSMUSG00000011171	ENSMUSG00000011171	ENSMUSG00000011171	12	116519807	116587389	222	256		
10815	7	158342030	158431231	ENSMUSG00000042050	ENSMUSG00000042050	ENSMUSG00000042050	12	116649631	116699483	222	256		
10816	7	158216450	158315014	ENSMUSG00000021171	ENSMUSG00000021171	ENSMUSG00000021171	12	116723231	116833059	222	256		
10817	7	158126917	158190224	ENSMUSG00000042029	ENSMUSG00000042029	ENSMUSG00000042029	12	116847841	116903606	222	256		
10818	7	157026169	158027071	ENSMUSG00000056553	ENSMUSG00000056553	ENSMUSG00000056553	12	116927842	117718802	222	256		
10819	7	22128555	22324181	ENSMUSG00000041992	ENSMUSG00000041992	ENSMUSG00000041992	12	118017564	118198090	223	257		
10820	7	21907043	21952042	ENSMUSG00000021175	ENSMUSG00000021175	ENSMUSG00000021175	12	118309115	118322812	223	257		
10821	7	21549358	21907986	ENSMUSG00000018581	ENSMUSG00000018581	ENSMUSG00000018581	12	118322088	118643145	223	257		
10822	7	21434214	21520674	ENSMUSG00000025323	ENSMUSG00000025323	ENSMUSG00000025323	12	118682249	118744917	223	257		
10823	7	20788428	20793030	ENSMUSG00000048562	ENSMUSG00000048562	ENSMUSG00000048562	12	119290431	119295858	223	257		
10824	7	20337271	20416944	ENSMUSG00000025321	ENSMUSG00000025321	ENSMUSG00000025321	12	119612103	119652710	223	257		
10825	7	20146776	20223538	ENSMUSG00000041886	ENSMUSG00000041886	ENSMUSG00000041886	12	119890780	119909771	223	257		
10826	7	19725464	19779541	ENSMUSG00000048004	ENSMUSG00000048004	ENSMUSG00000048004	12	120458850	120462668	223	257		
10827	10	5847192	5895518	ENSMUSG00000021218	ENSMUSG00000021218	ENSMUSG00000021218	13	3537379	3566287	224	258		
10828	10	5766807	5845535	ENSMUSG00000033799	ENSMUSG00000033799	ENSMUSG00000033799	13	3566160	3574853	224	258		
10829	10	5720837	5748547	ENSMUSG00000033781	ENSMUSG00000033781	ENSMUSG00000033781	13	3633322	3651025	224	258		
10830	10	5555972	5558734	ENSMUSG00000063130	ENSMUSG00000063130	ENSMUSG00000063130	13	3803000	3803449	224	258		
10831	10	5444514	5490426	ENSMUSG00000021215	ENSMUSG00000021215	ENSMUSG00000021215	13	3882582	3917357	224	258		
10832	10	5425061	5436793	ENSMUSG00000021216	ENSMUSG00000021216	ENSMUSG00000021216	13	3927335	3932808	224	258		
10833	10	5396972	5406169	ENSMUSG00000044988	ENSMUSG00000044988	ENSMUSG00000044988	13	3940402	3940896	224	258		
10834	10	4858426	4880249	ENSMUSG00000045410	ENSMUSG00000045410	ENSMUSG00000045410	13	4591910	4608387	224	258		
10835	10	3808188	3817467	ENSMUSG00000000078	ENSMUSG00000000078	ENSMUSG00000000078	13	5860735	5869639	224	258		
10836	10	3169922	3204999	ENSMUSG00000021193	ENSMUSG00000021193	ENSMUSG00000021193	13	6547403	6579408	224	258		
10837	10	3099712	3168985	ENSMUSG00000021196	ENSMUSG00000021196	ENSMUSG00000021196	13	6579120	6647970	224	258		
10838	10	1218073	1769670	ENSMUSG00000052551	ENSMUSG00000052551	ENSMUSG00000052551	13	8202467	8756648	224	258		
10839	10	1085478	1168237	ENSMUSG00000021147	ENSMUSG00000021147	ENSMUSG00000021147	13	8802214	8870976	224	258		
10840	10	1024373	1053593	ENSMUSG00000021149	ENSMUSG00000021149	ENSMUSG00000021149	13	8971705	8995258	224	258		
10841	10	845484	921702	ENSMUSG00000033499	ENSMUSG00000033499	ENSMUSG00000033499	13	9093169	9173650	224	258		
10842	10	310130	725606	ENSMUSG00000048264	ENSMUSG00000048264	ENSMUSG00000048264	13	9562360	9665276	224	258		
10843	10	170643	290566	ENSMUSG00000021156	ENSMUSG00000021156	ENSMUSG00000021156	13	9685016	9764377	224	258		
10844	1	237859012	238145373	ENSMUSG00000046159	ENSMUSG00000046159	ENSMUSG00000046159	13	9876475	9878244	225	259		
10845	1	235272128	236063911	ENSMUSG00000021313	ENSMUSG00000021313	ENSMUSG00000021313	13	11608677	12000036	225	259		
10846	1	235025233	235130590	ENSMUSG00000021311	ENSMUSG00000021311	ENSMUSG00000021311	13	12266633	12308306	225	259		
10847	1	234916431	234994554	ENSMUSG00000052374	ENSMUSG00000052374	ENSMUSG00000052374	13	12323759	12395065	225	259		
10848	1	234748188	234779619	ENSMUSG00000057554	ENSMUSG00000057554	ENSMUSG00000057554	13	12495115	12519275	225	259		
10849	1	234445478	234511908	ENSMUSG00000057069	ENSMUSG00000057069	ENSMUSG00000057069	13	12629944	12662011	225	259		
10850	1	234205753	234295085	ENSMUSG00000005397	ENSMUSG00000005397	ENSMUSG00000005397	13	13229817	13304390	225	259		
10851	1	233890973	234096847	ENSMUSG00000019726	ENSMUSG00000019726	ENSMUSG00000019726	13	13382535	13569569	225	259		
10852	1	233780755	233880677	ENSMUSG00000021303	ENSMUSG00000021303	ENSMUSG00000021303	13	13576665	13620021	225	259		
10853	1	233679890	233734404	ENSMUSG00000039242	ENSMUSG00000039242	ENSMUSG00000039242	13	13746800	13791212	225	259		
10854	1	233597298	233678906	ENSMUSG00000039233	ENSMUSG00000039233	ENSMUSG00000039233	13	13790079	13831764	225	259		
10855	1	233557288	233573030	ENSMUSG00000021302	ENSMUSG00000021302	ENSMUSG00000021302	13	13844555	13855527	225	259		
10856	1	233396835	233558123	ENSMUSG00000039219	ENSMUSG00000039219	ENSMUSG00000039219	13	13912267	13989859	225	259		
10857	7	42915399	42918182	ENSMUSG00000039182	ENSMUSG00000039182	ENSMUSG00000039182	13	14422403	14431222	226	260		
10858	7	41970205	42229420	ENSMUSG00000021318	ENSMUSG00000021318	ENSMUSG00000021318	13	15254867	15517860	226	260		
10859	7	41695126	41709231	ENSMUSG00000041324	ENSMUSG00000041324	ENSMUSG00000041324	13	15805370	15818147	226	260		
10860	7	40138869	40140783	ENSMUSG00000012429	ENSMUSG00000012429	ENSMUSG00000012429	13	17480555	17484247	226	260		
10861	7	39956634	40101668	ENSMUSG00000041297	ENSMUSG00000041297	ENSMUSG00000041297	13	17502624	17590239	226	260		
10862	7	39629687	39714240	ENSMUSG00000008859	ENSMUSG00000008859	ENSMUSG00000008859	13	17665717	17729359	226	260		
10863	7	39012933	39470907	ENSMUSG00000009734	ENSMUSG00000009734	ENSMUSG00000009734	13	18132592	18389475	226	260		
10864	7	38730069	38915325	ENSMUSG00000041236	ENSMUSG00000041236	ENSMUSG00000041236	13	18724757	18874266	226	260		
10865	7	38389832	38637545	ENSMUSG00000021314	ENSMUSG00000021314	ENSMUSG00000021314	13	18955836	19158380	226	260		
10866	7	38184376	38236760	ENSMUSG00000003062	ENSMUSG00000003062	ENSMUSG00000003062	13	19365141	19403217	226	260		
10867	7	37926688	37958065	ENSMUSG00000002808	ENSMUSG00000002808	ENSMUSG00000002808	13	19599171	19627268	226	260		
10868	7	37912247	37922903	ENSMUSG00000021319	ENSMUSG00000021319	ENSMUSG00000021319	13	19630648	19640286	226	260		
10869	7	37854724	37906525	ENSMUSG00000041138	ENSMUSG00000041138	ENSMUSG00000041138	13	19652546	19705216	226	260		
10870	7	37746521	37747438	ENSMUSG00000053101	ENSMUSG00000053101	ENSMUSG00000053101	13	19757147	19831722	226	260		
10871	7	36860492	37454981	ENSMUSG00000041112	ENSMUSG00000041112	ENSMUSG00000041112	13	20098084	20613993	226	260		
10872	7	36519135	36730595	ENSMUSG00000021322	ENSMUSG00000021322	ENSMUSG00000021322	13	20801584	21031717	226	260		
10873	6	28978758	28999745	ENSMUSG00000021326	ENSMUSG00000021326	ENSMUSG00000021326	13	21187678	21202188	227	261		
10874	6	28601681	28610706	ENSMUSG00000004344	ENSMUSG00000004344	ENSMUSG00000004344	13	21293898	21300151	227	261		
10875	6	28579052	28591549	ENSMUSG00000004341	ENSMUSG00000004341	ENSMUSG00000004341	13	21319715	21327085	227	261		
10876	6	28454711	28475490	ENSMUSG00000036721	ENSMUSG00000036721	ENSMUSG00000036721	13	21370292	21377289	227	261		
10877	6	28357328	28378304	ENSMUSG00000055313	ENSMUSG00000055313	ENSMUSG00000055313	13	21428740	21448366	227	261		
10878	6	28347887	28353953	ENSMUSG00000022228	ENSMUSG00000022228	ENSMUSG00000022228	13	21449695	21456920	227	261		
10879	6	28335077	28336715	ENSMUSG00000059395	ENSMUSG00000059395	ENSMUSG00000059395	13	21474719	21475906	227	261		
10880	6	28217695	28233215	ENSMUSG00000063894	ENSMUSG00000063894	ENSMUSG00000063894	13	21520687	21538576	227	261		
10881	6	28032998	28033939	ENSMUSG00000036658	ENSMUSG00000036658	ENSMUSG00000036658	13	21646045	21646986	227	261		
10882	6	28012712	28014160	ENSMUSG00000049737	ENSMUSG00000049737	ENSMUSG00000049737	13	21665833	21666786	227	261		
10883	6	26266328	26279553	ENSMUSG00000058189	ENSMUSG00000058189	ENSMUSG00000058189	13	21729563	21729943	out-of-place			
10884	6	27906973	27907284	ENSMUSG00000069306	ENSMUSG00000069306	ENSMUSG00000069306	13	21819243	21819560	227			
10885	6	26348540	26348964	ENSMUSG00000069305	ENSMUSG00000069305	ENSMUSG00000069305	13	21839312	21839629	out-of-place			
10886	6	27526506	27548858	ENSMUSG00000006720	ENSMUSG00000006720	ENSMUSG00000006720	13	21952577	21968244	227	262	here	
10887	6	27384811	27387980	ENSMUSG00000016982	ENSMUSG00000016982	ENSMUSG00000016982	13	21989026	21991944	227	262	Single marker moved to different place with in a HSB	1 -> 2
10888	6	27323487	27332327	ENSMUSG00000006179	ENSMUSG00000006179	ENSMUSG00000006179	13	22009641	22017233	227	262		
10889	6	26705159	26708257	ENSMUSG00000036376	ENSMUSG00000036376	ENSMUSG00000036376	13	23425826	23431331	227	262		
10890	6	26609428	26618629	ENSMUSG00000000706	ENSMUSG00000000706	ENSMUSG00000000706	13	23464464	23473340	227	262		
10891	6	26153591	26155001	ENSMUSG00000062417	ENSMUSG00000062417	ENSMUSG00000062417	13	23542883	23543374	227	263	here	This inversion was ignored by our tool.
10892	6	26342475	26343140	ENSMUSG00000052565	ENSMUSG00000052565	ENSMUSG00000052565	13	23562499	23565473	227	263	Inversion ignored by ST	0 -> 2
10893	6	26264538	26265322	ENSMUSG00000051627	ENSMUSG00000051627	ENSMUSG00000051627	13	23629303	23629962	227	263		
10894	6	27222176	27222556	ENSMUSG00000018102	ENSMUSG00000018102	ENSMUSG00000018102	13	23691664	23699945	227	263		
10895	6	26215677	26216300	ENSMUSG00000036211	ENSMUSG00000036211	ENSMUSG00000036211	13	23703276	23704005	227	264		
10896	6	26135148	26135459	ENSMUSG00000060678	ENSMUSG00000060678	ENSMUSG00000060678	13	23705603	23705917	227	264		
10897	6	26195427	26205035	ENSMUSG00000006611	ENSMUSG00000006611	ENSMUSG00000006611	13	23711306	23718276	227	264		
10898	6	26163994	26164635	ENSMUSG00000036181	ENSMUSG00000036181	ENSMUSG00000036181	13	23746272	23747831	227	264		
10899	6	27890801	27891213	ENSMUSG00000075031	ENSMUSG00000075031	ENSMUSG00000075031	13	23754259	23754989	out-of-place			
10900	6	26128697	26129165	ENSMUSG00000069265	ENSMUSG00000069265	ENSMUSG00000069265	13	23769408	23769818	227	265	here	
10901	6	26125292	26125939	ENSMUSG00000049539	ENSMUSG00000049539	ENSMUSG00000049539	13	23771133	23771879	227	265	Single marker moved to different place with in a HSB	0 -> 1
10902	6	26071254	26093499	ENSMUSG00000064140	ENSMUSG00000064140	ENSMUSG00000064140	13	23790037	23798960	227	265		
10903	6	26020970	26038818	ENSMUSG00000036110	ENSMUSG00000036110	ENSMUSG00000036110	13	23814492	23832645	227	265		
10904	6	25953307	25990493	ENSMUSG00000036083	ENSMUSG00000036083	ENSMUSG00000036083	13	23847094	23867219	227	265		
10905	6	25891296	25938776	ENSMUSG00000021335	ENSMUSG00000021335	ENSMUSG00000021335	13	23877797	23903195	227	265		
10906	6	25862906	25889398	ENSMUSG00000021336	ENSMUSG00000021336	ENSMUSG00000021336	13	23905354	23922472	227	265		
10907	6	25834339	25834734	ENSMUSG00000060081	ENSMUSG00000060081	ENSMUSG00000060081	13	23941927	23942316	227	265		
10908	6	25760408	25809981	ENSMUSG00000021337	ENSMUSG00000021337	ENSMUSG00000021337	13	23960919	23998679	227	265		
10909	6	25387756	25728735	ENSMUSG00000021338	ENSMUSG00000021338	ENSMUSG00000021338	13	24020124	24288260	227	265		
10910	6	25189613	25246800	ENSMUSG00000016756	ENSMUSG00000016756	ENSMUSG00000016756	13	24334869	24476101	227	265		
10911	6	24912492	24983998	ENSMUSG00000036006	ENSMUSG00000036006	ENSMUSG00000036006	13	24677418	24741275	227	265		
10912	6	24883138	24894302	ENSMUSG00000006715	ENSMUSG00000006715	ENSMUSG00000006715	13	24759311	24769328	227	265		
10913	6	24813068	24829043	ENSMUSG00000019132	ENSMUSG00000019132	ENSMUSG00000019132	13	24809488	24821401	227	265		
10914	6	24775254	24809917	ENSMUSG00000006717	ENSMUSG00000006717	ENSMUSG00000006717	13	24825420	24838954	227	265		
10915	6	24758184	24775240	ENSMUSG00000035958	ENSMUSG00000035958	ENSMUSG00000035958	13	24839150	24849611	227	265		
10916	6	24652311	24754362	ENSMUSG00000006711	ENSMUSG00000006711	ENSMUSG00000006711	13	24852602	24908735	227	265		
10917	6	24603176	24645414	ENSMUSG00000035936	ENSMUSG00000035936	ENSMUSG00000035936	13	24918497	24945126	227	265		

10918	6	24536384	24597829	ENSMUSG00000021340	ENSMUSG00000021340	ENSMUSG00000021340	13	24950666	24998220	227	265		
10919	6	24511161	24533789	ENSMUSG00000021339	ENSMUSG00000021339	ENSMUSG00000021339	13	24999948	25027844	227	265		
10920	6	24254736	24465957	ENSMUSG00000035910	ENSMUSG00000035910	ENSMUSG00000035910	13	25063696	25213024	227	265		
10921	6	24234329	24255618	ENSMUSG00000048978	ENSMUSG00000048978	ENSMUSG00000048978	13	25259505	25277414	227	265		
10922	6	22677701	22679874	ENSMUSG00000045835	ENSMUSG00000045835	ENSMUSG00000045835	13	26776702	26777553	227	265		
10923	6	21700979	21706829	ENSMUSG00000076431	ENSMUSG00000076431	ENSMUSG00000076431	13	28956384	28961178	227	265		
10924	6	20642736	21340611	ENSMUSG00000006191	ENSMUSG00000006191	ENSMUSG00000006191	13	29333502	29857573	227	265		
10925	6	20510377	20601921	ENSMUSG00000016477	ENSMUSG00000016477	ENSMUSG00000016477	13	29914040	29993133	227	265		
10926	6	20208927	20320608	ENSMUSG00000038732	ENSMUSG00000038732	ENSMUSG00000038732	13	30143955	30254163	227	265		
10927	6	237053	296347	ENSMUSG00000069255	ENSMUSG00000069255	ENSMUSG00000069255	13	30667566	30717924	228	266		
10928	6	336739	356447	ENSMUSG00000021356	ENSMUSG00000021356	ENSMUSG00000021356	13	30756723	30774392	228	266		
10929	6	430139	638109	ENSMUSG00000021357	ENSMUSG00000021357	ENSMUSG00000021357	13	30821383	30981512	228	266		
10930	6	600939	601944	ENSMUSG00000076430	ENSMUSG00000076430	ENSMUSG00000076430	13	30954038	30955224	228	266		
10931	6	1257675	1259981	ENSMUSG00000038415	ENSMUSG00000038415	ENSMUSG00000038415	13	31565902	31567947	228	266		
10932	6	1555206	1559131	ENSMUSG00000050295	ENSMUSG00000050295	ENSMUSG00000050295	13	31814111	31818100	228	266		
10933	6	1569040	2190914	ENSMUSG00000038372	ENSMUSG00000038372	ENSMUSG00000038372	13	31827049	32346033	228	266		
10934	6	2608871	2696153	ENSMUSG00000044951	ENSMUSG00000044951	ENSMUSG00000044951	13	32716370	32732347	228	266		
10935	6	2710647	2730978	ENSMUSG00000021400	ENSMUSG00000021400	ENSMUSG00000021400	13	32809495	32830072	228	266		
10936	6	3009212	3060418	ENSMUSG00000021408	ENSMUSG00000021408	ENSMUSG00000021408	13	34010339	34042635	228	266		
10937	6	3063952	3098429	ENSMUSG00000038286	ENSMUSG00000038286	ENSMUSG00000038286	13	34045062	34081532	228	266		
10938	6	3098918	3102808	ENSMUSG00000058672	ENSMUSG00000058672	ENSMUSG00000058672	13	34081743	34085453	228	266		
10939	6	3169516	3172968	ENSMUSG00000045136	ENSMUSG00000045136	ENSMUSG00000045136	13	34134473	34137819	228	266		
10940	6	3204206	3212945	ENSMUSG00000071451	ENSMUSG00000071451	ENSMUSG00000071451	13	34170455	34185631	228	266		
10941	6	3218288	3355490	ENSMUSG00000038267	ENSMUSG00000038267	ENSMUSG00000038267	13	34186623	34352647	228	266		
10942	6	3667853	3697259	ENSMUSG00000021411	ENSMUSG00000021411	ENSMUSG00000021411	13	34635305	34660136	228	266		
10943	6	3794619	3796550	ENSMUSG00000038246	ENSMUSG00000038246	ENSMUSG00000038246	13	34742315	34755080	228	266		
10944	6	3966500	4010215	ENSMUSG00000021413	ENSMUSG00000021413	ENSMUSG00000021413	13	34882994	34910498	228	266		
10945	6	4013601	4024390	ENSMUSG00000021414	ENSMUSG00000021414	ENSMUSG00000021414	13	34917845	34927458	228	266		
10946	6	4032672	4075991	ENSMUSG00000021415	ENSMUSG00000021415	ENSMUSG00000021415	13	34931874	34960717	228	266		
10947	6	4651392	4900780	ENSMUSG00000059288	ENSMUSG00000059288	ENSMUSG00000059288	13	35691211	35880135	228	266		
10948	6	4940282	4949270	ENSMUSG00000021418	ENSMUSG00000021418	ENSMUSG00000021418	13	35900937	35913812	228	266		
10949	6	5206726	5716815	ENSMUSG00000021420	ENSMUSG00000021420	ENSMUSG00000021420	13	36124876	36545059	228	266		
10950	6	5943234	5952632	ENSMUSG00000039114	ENSMUSG00000039114	ENSMUSG00000039114	13	36733091	36741922	228	266		
10951	6	6089317	6265901	ENSMUSG00000039109	ENSMUSG00000039109	ENSMUSG00000039109	13	36874653	37056670	228	266		
10952	6	6533340	6600215	ENSMUSG00000021423	ENSMUSG00000021423	ENSMUSG00000021423	13	37352823	37426495	228	266		
10953	6	7053263	7196194	ENSMUSG00000039087	ENSMUSG00000039087	ENSMUSG00000039087	13	37901338	37959460	228	266		
10954	6	7232803	7258434	ENSMUSG00000021427	ENSMUSG00000021427	ENSMUSG00000021427	13	37985447	38001623	228	266		
10955	6	7271883	7334634	ENSMUSG00000044566	ENSMUSG00000044566	ENSMUSG00000044566	13	38013521	38039944	228	266		
10956	6	7335061	7363267	ENSMUSG00000021428	ENSMUSG00000021428	ENSMUSG00000021428	13	38044761	38068895	228	266		
10957	6	7486869	7531945	ENSMUSG00000054889	ENSMUSG00000054889	ENSMUSG00000054889	13	38203950	38206041	228	266		
10958	6	7535431	7557199	ENSMUSG00000021431	ENSMUSG00000021431	ENSMUSG00000021431	13	38212405	38235129	228	266		
10959	6	7672009	7826752	ENSMUSG00000039004	ENSMUSG00000039004	ENSMUSG00000039004	13	38353399	38507780	228	266		
10960	6	7826482	7856014	ENSMUSG00000038991	ENSMUSG00000038991	ENSMUSG00000038991	13	38507732	38535926	228	266		
10961	6	8024629	8047807	ENSMUSG00000001707	ENSMUSG00000001707	ENSMUSG00000001707	13	38653157	38666476	228	266		
10962	6	8358300	8380715	ENSMUSG00000021432	ENSMUSG00000021432	ENSMUSG00000021432	13	38939774	38967995	228	266		
10963	6	10504906	10523297	ENSMUSG00000021359	ENSMUSG00000021359	ENSMUSG00000021359	13	40727698	40745894	228	266		
10964	6	10802914	10818001	ENSMUSG00000038683	ENSMUSG00000038683	ENSMUSG00000038683	13	41011998	41023985	228	266		
10965	6	10870942	10946714	ENSMUSG00000021363	ENSMUSG00000021363	ENSMUSG00000021363	13	41036094	41090592	228	266		
10966	6	10981442	10990160	ENSMUSG00000021362	ENSMUSG00000021362	ENSMUSG00000021362	13	41112399	41121157	228	266		
10967	6	10995346	11082517	ENSMUSG00000038651	ENSMUSG00000038651	ENSMUSG00000038651	13	41148872	41185452	228	266		
10968	6	11088981	11152610	ENSMUSG00000021364	ENSMUSG00000021364	ENSMUSG00000021364	13	41196268	41208172	228	266		
10969	6	11291517	11340887	ENSMUSG00000021365	ENSMUSG00000021365	ENSMUSG00000021365	13	41322635	41350000	228	266		
10970	6	11646497	11691743	ENSMUSG00000069239	ENSMUSG00000069239	ENSMUSG00000069239	13	41638918	41645560	228	266		
10971	6	11825349	11915239	ENSMUSG00000058022	ENSMUSG00000058022	ENSMUSG00000058022	13	41774122	41858462	228	266		
10972	6	12120557	12273218	ENSMUSG00000021366	ENSMUSG00000021366	ENSMUSG00000021366	13	42066475	42195485	228	266		
10973	6	12398582	12405413	ENSMUSG00000021367	ENSMUSG00000021367	ENSMUSG00000021367	13	42312448	42318962	228	266		
10974	6	12826963	13394434	ENSMUSG00000054728	ENSMUSG00000054728	ENSMUSG00000054728	13	42693670	43146161	228	266		
10975	6	13466041	13595766	ENSMUSG00000051335	ENSMUSG00000051335	ENSMUSG00000051335	13	43206491	43315144	228	266		
10976	6	13682854	13720503	ENSMUSG00000054021	ENSMUSG00000054021	ENSMUSG00000054021	13	43376478	43406174	228	266		
10977	6	13723555	13729917	ENSMUSG00000063200	ENSMUSG00000063200	ENSMUSG00000063200	13	43409362	43413830	228	266		
10978	6	13729714	13819932	ENSMUSG00000038546	ENSMUSG00000038546	ENSMUSG00000038546	13	43414351	43491761	228	266		
10979	6	13898999	13922779	ENSMUSG00000021371	ENSMUSG00000021371	ENSMUSG00000021371	13	43552531	43571133	228	266		
10980	6	14033182	14088212	ENSMUSG00000044164	ENSMUSG00000044164	ENSMUSG00000044164	13	43626953	43679875	228	266		
10981	6	14225715	14245063	ENSMUSG00000015396	ENSMUSG00000015396	ENSMUSG00000015396	13	43796157	43814102	228	266		
10982	6	15354506	15630232	ENSMUSG00000038518	ENSMUSG00000038518	ENSMUSG00000038518	13	44834352	44930924	228	266		
10983	6	15631020	15771250	ENSMUSG00000057531	ENSMUSG00000057531	ENSMUSG00000057531	13	44933057	45013112	228	266		
10984	6	16237296	16256455	ENSMUSG00000038175	ENSMUSG00000038175	ENSMUSG00000038175	13	45400714	45422912	228	266		
10985	6	16346790	16403757	ENSMUSG00000000253	ENSMUSG00000000253	ENSMUSG00000000253	13	45518429	45557352	228	266		
10986	6	16407320	16869666	ENSMUSG00000046876	ENSMUSG00000046876	ENSMUSG00000046876	13	45565865	45975948	228	266		
10987	6	17389792	17402085	ENSMUSG00000038132	ENSMUSG00000038132	ENSMUSG00000038132	13	46430003	46440289	228	266		
10988	6	17501715	17666000	ENSMUSG00000021373	ENSMUSG00000021373	ENSMUSG00000021373	13	46513119	46661250	228	266		
10989	6	17708565	17719928	ENSMUSG00000069237	ENSMUSG00000069237	ENSMUSG00000069237	13	46680558	46689028	228	266		
10990	6	17723248	17814797	ENSMUSG00000021374	ENSMUSG00000021374	ENSMUSG00000021374	13	46691073	46738559	228	266		
10991	6	17871907	18095692	ENSMUSG00000021375	ENSMUSG00000021375	ENSMUSG00000021375	13	46763035	46940593	228	266		
10992	6	18229629	18230816	ENSMUSG00000044231	ENSMUSG00000044231	ENSMUSG00000044231	13	47024546	47025751	228	266		
10993	6	18268106	18330199	ENSMUSG00000038080	ENSMUSG00000038080	ENSMUSG00000038080	13	47054541	47095572	228	266		
10994	6	18332392	18372750	ENSMUSG00000021377	ENSMUSG00000021377	ENSMUSG00000021377	13	47095756	47117138	228	266		
10995	6	18495573	18576827	ENSMUSG00000038068	ENSMUSG00000038068	ENSMUSG00000038068	13	47133692	47257448	228	266		
10996	6	19945596	19948894	ENSMUSG00000021379	ENSMUSG00000021379	ENSMUSG00000021379	13	48273364	48275973	228	266		
10997	9	95886567	95911957	ENSMUSG00000038042	ENSMUSG00000038042	ENSMUSG00000038042	13	48589809	48637517	229	267		
10998	9	95753734	95757474	ENSMUSG00000021381	ENSMUSG00000021381	ENSMUSG00000021381	13	48674971	48678388	229	267		
10999	9	95378851	95481684	ENSMUSG00000038025	ENSMUSG00000038025	ENSMUSG00000038025	13	48813687	48882820	229	267		
11000	9	95253994	95368218	ENSMUSG00000038014	ENSMUSG00000038014	ENSMUSG00000038014	13	48891156	48979954	229	267		
11001	9	94987033	95120130	ENSMUSG00000037989	ENSMUSG00000037989	ENSMUSG00000037989	13	49048247	49159169	229	267		
11002	9	94923592	94936391	ENSMUSG00000037966	ENSMUSG00000037966	ENSMUSG00000037966	13	49199466	49208181	229	267		
11003	9	94898321	94915385	ENSMUSG00000037960	ENSMUSG00000037960	ENSMUSG00000037960	13	49215057	49220090	229	267		
11004	9	94860810	94887241	ENSMUSG00000021384	ENSMUSG00000021384	ENSMUSG00000021384	13	49242627	49260091	229	267		
11005	9	94749554	94838339	ENSMUSG00000037946	ENSMUSG00000037946	ENSMUSG00000037946	13	49275047	49321145	229	267		
11006	9	94513466	94566915	ENSMUSG00000037933	ENSMUSG00000037933	ENSMUSG00000037933	13	49353486	49398962	229	267		
11007	9	94415287	94472368	ENSMUSG00000021385	ENSMUSG00000021385	ENSMUSG00000021385	13	49433215	49474923	229	267		
11008	9	94297415	94338158	ENSMUSG00000043631	ENSMUSG00000043631	ENSMUSG00000043631	13	49516747	49544732	229	267		
11009	9	94258308	94284609	ENSMUSG00000021388	ENSMUSG00000021388	ENSMUSG00000021388	13	49556424	49579494	229	267		
11010	9	94216348	94226564	ENSMUSG00000048368	ENSMUSG00000048368	ENSMUSG00000048368	13	49594703	49604546	229	267		
11011	9	94186070	94206716	ENSMUSG00000021390	ENSMUSG00000021390	ENSMUSG00000021390	13	49619983	49636437	229	267		
11012	9	94099462	94127659	ENSMUSG00000021392	ENSMUSG00000021392	ENSMUSG00000021392	13	49665040	49690910	229	267		
11013	9	94012310	94095803	ENSMUSG00000037851	ENSMUSG00000037851	ENSMUSG00000037851	13	49694080	49746196	229	267		
11014	9	90193154	90283429	ENSMUSG00000021395	ENSMUSG00000021395	ENSMUSG00000021395	13	51146845	51163073	230	268		
11015	9	90339840	90380524	ENSMUSG00000021396	ENSMUSG00000021396	ENSMUSG00000021396	13	51183519	51187684	230	268		
11016	9	90796182	90809744	ENSMUSG00000067586	ENSMUSG00000067586	ENSMUSG00000067586	13	51421260	51435293	230	268		
11017	9	90817880	90983502	ENSMUSG00000021448	ENSMUSG00000021448	ENSMUSG00000021448	13	51443539	51579580	230	268		
11018	9	91123241	91164377	ENSMUSG00000035139	ENSMUSG00000035139	ENSMUSG00000035139	13	51664267	51696341	230	268		
11019	9	91166336	91169386	ENSMUSG00000051107	ENSMUSG00000051107	ENSMUSG00000051107	13	51698716	51704115	230	268		
11020	9	91181972	91223172	ENSMUSG00000021451	ENSMUSG00000021451	ENSMUSG00000021451	13	51713742	51806140	230	268		
11021	9	91409748	91411290	ENSMUSG00000021453	ENSMUSG00000021453	ENSMUSG00000021453	13	51859174	51860968	230	268		
11022	9	92411934	92445206	ENSMUSG00000047842	ENSMUSG00000047842	ENSMUSG00000047842	13	52516812	52543086	230	268		
11023	9	92603890	92700652	ENSMUSG00000021457	ENSMUSG00000021457	ENSMUSG00000021457	13	52595933	52661223	230	268		
11024	9	93015918	93164016	ENSMUSG00000021460	ENSMUSG00000021460	ENSMUSG00000021460	13	52847551	52942097	230	268		
11025	9	93211148	93225965	ENSMUSG00000056749	ENSMUSG00000056749	ENSMUSG00000056749	13	52979641	52993471	230	268		
11026	9	93524700	93752265	ENSMUSG00000021464	ENSMUSG00000021464	ENSMUSG00000021464	13	53121749	53293643	230	268		
11027	9	93833248	93917511	ENSMUSG00000021468	ENSMUSG00000021468	ENSMUSG00000021468	13	53340250	53384899	230	268		
11028	9	89687592	89693632	ENSMUSG00000043339	ENSMUSG00000043339	ENSMUSG00000043339	13	53424904	53426586	Singleton			
11029	5	174084208	174090507	ENSMUSG00000021469	ENSMUSG00000021469	ENSMUSG00000021469	13	53475420	53480608	231	269		
11030	5	174800643	174803769	ENSMUSG00000021478	ENSMUSG00000021478	ENSMUSG00000021478	13	54061276	54065279	231	269		
11031	5	174851713	174888290	ENSMUSG00000021474	ENSMUSG00000021474	ENSMUSG00000021474	13	54081490	54117966	231	269		
11032	5	175017637	175045847	ENSMUSG00000034987	ENSMUSG00000034987	ENSMUSG00000034987	13	54201750	54232053	231	269		
11033	5	177235129	177243843	ENSMUSG00000025872	ENSMUSG00000025872	ENSMUSG00000025872	13	54469140	54478441	Singleton			
11034	5	175623040	175705594	ENSMUSG00000043183	ENSMUSG00000043183	ENSMUSG00000043183	13	54534960	54560920	231	270	here	Presence of a singleton made AutoGRAPH to break the block
11035	5	175725077	175729567	ENSMUSG00000025870	ENSMUSG00000025870	ENSMUSG00000025870	13	54584687	54590750	231	270	Presence of singletons	1 -> 2
11036	5	175743590	175748959	ENSMUSG00000025869	ENSMUSG00000025869	ENSMUSG00000025869	13	54593813	54599696	231	270		
11037	5	175752062	175776146	ENSMUSG00000047547	ENSMUSG00000047547	ENSMUSG00000047547	13	54603208	54620896	231	270		
11038	5	175807984	175869671	ENSMUSG00000025873	ENSMUSG00000025873	ENSMUSG00000025873	13	54631441	54673690	231	270		
11039	5	175886306	175897027	ENSMUSG00000034928	ENSMUSG00000034928	ENSMUSG00000034928	13	54689033	54703469	231	270		
11040	5	175908971	175955279	ENSMUSG00000034918	ENSMUSG00000034918	ENSMUSG00000034918	13	54711085	54746277	231	270		
11041	5	175955411	175969737	ENSMUSG00000069227	ENSMUSG00000069227	ENSMUSG00000069227	13	54746295	54759291	231	270		
11042	5	175979817	175990163	ENSMUSG00000034891	ENSMUSG00000034891	ENSMUSG00000034891	13	54768482	54776062	231	270		
11043	5	176007104	176018660	ENSMUSG00000025875	ENSMUSG00000025875	ENSMUSG00000025875	13	54799082	54806389	231	270		
11044	5	176170166	176240503	ENSMUSG00000025876	ENSMUSG00000025876	ENSMUSG00000025876	13	54959054	55015640	231	270		
11045	5	176240680	176259284	ENSMUSG00000025877	ENSMUSG00000025877	ENSMUSG00000025877	13	55015608	55030956	231	270		
11046	5	176264673	176366322	ENSMUSG00000025878	ENSMUSG00000025878	ENSMUSG00000025878	13	55037502	55109917	231	270		
11047	5	176382342	176440796	ENSMUSG00000021481	ENSMUSG00000021481	ENSMUSG00000021481	13	55114943	55144447	231	270		
11048	5	176449676	176457726	ENSMUSG00000005320	ENSMUSG00000005320	ENSMUSG00000005320	13	55162262	55178355	231	270		
11049	5	176494711	176655367	ENSMUSG00000021488	ENSMUSG00000021488	ENSMUSG00000021488	13	55222843	55324503	231	270		
11050	5	176663406	176666555	ENSMUSG00000021486	ENSMUSG00000021486	ENSMUSG00000021486	13	55330122	55334893	231	270		
11051	5	176666830	176671916	ENSMUSG00000021485	ENSMUSG00000021485	ENSMUSG00000021485	13	55335103	55339367	231	270		
11052	5	176691170	176711258	ENSMUSG00000021484	ENSMUSG00000021484	ENSMUSG00000021484	13	55353455	55372405	231	270		
11053	5	176717450	176732204	ENSMUSG00000052087	ENSMUSG00000052087	ENSMUSG00000052087	13	55379393	55394309	231	270		
11054	5	176744051	176758454	ENSMUSG00000021490	ENSMUSG00000021490	ENSMUSG00000021490	13	55409315	55423714	231	270		
11055	5	176759770	176760183	ENSMUSG00000044444	ENSMUSG00000044444	ENSMUSG00000044444	13	55424375	55424788	231	270		
11056	5	176761899	176769134	ENSMUSG00000021492	ENSMUSG00000021492	ENSMUSG00000021492	13	55427588	55436415	231	270		
11057	5	176786294	176801434	ENSMUSG00000074886	ENSMUSG00000074886	ENSMUSG00000074886	13	55455086	55469496	231	270		
11058	5	176806400	176815889	ENSMUSG00000034686	ENSMUSG00000034686	ENSMUSG00000034686	13	55481647	55482774	231	270		
11059	5	176816220	176832805	ENSMUSG00000034675	ENSMUSG00000034675	ENSMUSG00000034675	13	55483638	55497688	231	270		
11060	5	176843001	176857208	ENSMUSG00000021493	ENSMUSG00000021493	ENSMUSG00000021493	13	55505417	55523060	231	270		
11061	5	176863358	176869459	ENSMUSG00000035711	ENSMUSG00000035711	ENSMUSG00000035711	13	55532857	55538160	231	270		
11062	5	176871185	176876557	ENSMUSG00000021494	ENSMUSG00000021494	ENSMUSG00000021494	13	55540039	55546143	231	270		
11063	5	176879397	176914114	ENSMUSG00000021495	ENSMUSG00000021495	ENSMUSG00000021495	13	55548939	55579591	231	270		
11064	5	176959753	176969918	ENSMUSG00000021504	ENSMUSG00000021504	ENSMUSG00000021504	13	55609733	55619576	231	270		
11065	5	134102105	134115740	ENSMUSG00000021501	ENSMUSG00000021501	ENSMUSG00000021501	13	55632627	55642038	232	271		
11066	5	134122360	134194708	ENSMUSG00000021500	ENSMUSG00000021500	ENSMUSG00000021500	13	55644673	55688735	232	271		
11067	5	134209269	134222684	ENSMUSG00000045767	ENSMUSG00000045767	ENSMUSG00000045767	13	55702746	55713122	232	271		
11068	5	134237905	134263469	ENSMUSG00000021497	ENSMUSG00000021497	ENSMUSG00000021497	13	55724272	55735855	232	271		
11069	5	134268706	134324339	ENSMUSG00000021496	ENSMUSG00000021496	ENSMUSG00000021496	13	55736990	55786452	232	271		
11070	5	134331513	134375285	ENSMUSG00000021499	ENSMUSG00000021499	ENSMUSG00000021499	13	55794201	55818620	232	271		
11071	5	134391368	134397887	ENSMUSG00000021506	ENSMUSG00000021506	ENSMUSG00000021506	13	55834676	55841047	232	271		
11072	5	134698517	134763473	ENSMUSG00000015937	ENSMUSG00000015937	ENSMUSG00000015937	13	56083781	56145137	232	271		
11073	5	134898566	134899279	ENSMUSG00000048904	ENSMUSG00000048904	ENSMUSG00000048904	13	56260820	56261554	232	271		
11074	5	134934274	134942868	ENSMUSG00000021508	ENSMUSG00000021508	ENSMUSG00000021508	13	56298265	56306173	232	271		
11075	5	135198315	135235503	ENSMUSG00000021509	ENSMUSG00000021509	ENSMUSG00000021509	13	56447977	56481943	232	271		
11076	5	135255834	135259415	ENSMUSG00000021538	ENSMUSG00000021538	ENSMUSG00000021538	13	56488899	56491868	232	271		
11077	5	135304062	135305266	ENSMUSG00000035509	ENSMUSG00000035509	ENSMUSG00000035509	13	56532111	56547520	232	271		
11078	5	135310499	135318622	ENSMUSG00000021539	ENSMUSG00000021539	ENSMUSG00000021539	13	56552087	56558124	232	271		
11079	5	135392644	135427253	ENSMUSG00000035493	ENSMUSG00000035493	ENSMUSG00000035493	13	56619208	56648972	232	271		
11080	5	135496435	135546321	ENSMUSG00000021540	ENSMUSG00000021540	ENSMUSG00000021540	13	56712729	56752001	232	271		
11081	5	135577022	135720974	ENSMUSG00000021541	ENSMUSG00000021541	ENSMUSG00000021541	13	56782730	56905210	232	271		
11082	5	136342242	136862146	ENSMUSG00000056222	ENSMUSG00000056222	ENSMUSG00000056222	13	57435716	57917210	232	271		
11083	5	136981090	137099448	ENSMUSG00000014164	ENSMUSG00000014164	ENSMUSG00000014164	13	57932795	58104873	232	271		
11084	5	137116737	137117654	ENSMUSG00000007836	ENSMUSG00000007836	ENSMUSG00000007836	13	58137019	58137936	232	271		
11085	9	85427893	85448858	ENSMUSG00000050002	ENSMUSG00000050002	ENSMUSG00000050002	13	58167303	58173569	233	272		
11086	9	85464698	85512938	ENSMUSG00000005312	ENSMUSG00000005312	ENSMUSG00000005312	13	58185786	58225259	233	272		
11087	9	85641433	85726162	ENSMUSG00000060176	ENSMUSG00000060176	ENSMUSG00000060176	13	58297125	58364485	233	272		
11088	9	85743046	85761452	ENSMUSG00000021550	ENSMUSG00000021550	ENSMUSG00000021550	13	58389669	58394848	233	272		
11089	9	86080192	86145418	ENSMUSG00000021553	ENSMUSG00000021553	ENSMUSG00000021553	13	58562928	58620491	233	272		
11090	9	86473286	86828325	ENSMUSG00000055254	ENSMUSG00000055254	ENSMUSG00000055254	13	58816219	59137976	233	272		
11091	9	87351277	87546621	ENSMUSG00000021557	ENSMUSG00000021557	ENSMUSG00000021557	13	59463473	59554062	233	272		
11092	9	87745881	87827033	ENSMUSG00000021555	ENSMUSG00000021555	ENSMUSG00000021555	13	59595034	59644421	233	272		
11093	9	87830876	87904903	ENSMUSG00000021556	ENSMUSG00000021556	ENSMUSG00000021556	13	59644619	59685407	233	272		
11094	9	88032046	88064339	ENSMUSG00000049902	ENSMUSG00000049902	ENSMUSG00000049902	13	59698952	59703724	233	272		
11095	9	88092470	88159198	ENSMUSG00000035248	ENSMUSG00000035248	ENSMUSG00000035248	13	59781499	59832724	233	272		
11096	9	88749098	88751924	ENSMUSG00000052957	ENSMUSG00000052957	ENSMUSG00000052957	13	60185410	60186564	233	272		
11097	9	89301963	89513365	ENSMUSG00000021559	ENSMUSG00000021559	ENSMUSG00000021559	13	60611581	60772425	233	272		
11098	9	96360823	96395896	ENSMUSG00000021456	ENSMUSG00000021456	ENSMUSG00000021456	13	62846507	62867988	234	273		
11099	9	96405244	96441686	ENSMUSG00000069805	ENSMUSG00000069805	ENSMUSG00000069805	13	62874376	62897859	234	273		
11100	9	96561887	96884818	ENSMUSG00000021458	ENSMUSG00000021458	ENSMUSG00000021458	13	63024790	63335318	234	273		
11101	9	96901158	97119328	ENSMUSG00000021461	ENSMUSG00000021461	ENSMUSG00000021461	13	63313931	63440720	234	273		
11102	9	97246221	97310652	ENSMUSG00000021466	ENSMUSG00000021466	ENSMUSG00000021466	13	63520755	63574742	234	273		
11103	9	97677804	97768823	ENSMUSG00000021470	ENSMUSG00000021470	ENSMUSG00000021470	13	63824591	63862999	234	273		
11104	9	97774970	97816670	ENSMUSG00000071321	ENSMUSG00000071321	ENSMUSG00000071321	13	63881677	63909524	234	273		
11105	9	98037409	98104255	ENSMUSG00000033122	ENSMUSG00000033122	ENSMUSG00000033122	13	64067488	64098408	234	273		
11106	9	98122809	98185800	ENSMUSG00000033114	ENSMUSG00000033114	ENSMUSG00000033114	13	64106787	64138533	234	273		
11107	9	98188044	98220432	ENSMUSG00000044934	ENSMUSG00000044934	ENSMUSG00000044934	13	64142279	64162421	234	273		
11108	9	98252308	98293439	ENSMUSG00000021476	ENSMUSG00000021476	ENSMUSG00000021476	13	64171088	64195758	234	273		
11109	9	98441680	98457384	ENSMUSG00000021482	ENSMUSG00000021482	ENSMUSG00000021482	13	64301606	64326737	234	273		
11110	9	98834761	98841236	ENSMUSG00000021477	ENSMUSG00000021477	ENSMUSG00000021477	13	64377945	64385037	234	273		
11111	9	89771818	89779444	ENSMUSG00000021483	ENSMUSG00000021483	ENSMUSG00000021483	13	64447367	64456036	Singleton			
11112	9	96176654	96263145	ENSMUSG00000038212	ENSMUSG00000038212	ENSMUSG00000038212	13	65079823	65127735	Singleton			

11113	8	97320839	97342993	ENSMUSG00000021519	ENSMUSG00000021519	ENSMUSG00000021519	13	67347875	67368816	235	274
11114	8	97343340	97415950	ENSMUSG00000021518	ENSMUSG00000021518	ENSMUSG00000021518	13	67368579	67433261	235	274
11115	5	7922217	7954237	ENSMUSG00000034617	ENSMUSG00000034617	ENSMUSG00000034617	13	69028151	69049492	236	275
11116	5	7912273	7922115	ENSMUSG00000021532	ENSMUSG00000021532	ENSMUSG00000021532	13	69049634	69059708	236	275
11117	5	7449345	7883194	ENSMUSG00000021536	ENSMUSG00000021536	ENSMUSG00000021536	13	69087426	69466728	236	275
11118	5	6767718	6810161	ENSMUSG00000034575	ENSMUSG00000034575	ENSMUSG00000034575	13	69965329	70001211	236	275
11119	5	6686456	6722675	ENSMUSG00000021594	ENSMUSG00000021594	ENSMUSG00000021594	13	70040821	70078835	236	275
11120	5	6652352	6686169	ENSMUSG00000021595	ENSMUSG00000021595	ENSMUSG00000021595	13	70079465	70103029	236	275
11121	5	6425040	6431639	ENSMUSG00000021598	ENSMUSG00000021598	ENSMUSG00000021598	13	70277260	70283473	236	275
11122	5	5475807	5543305	ENSMUSG00000034525	ENSMUSG00000034525	ENSMUSG00000034525	13	71056070	71073811	236	275
11123	5	5193581	5372251	ENSMUSG00000049538	ENSMUSG00000049538	ENSMUSG00000049538	13	71195180	71309182	236	275
11124	5	3649168	3654517	ENSMUSG00000060969	ENSMUSG00000060969	ENSMUSG00000060969	13	72424607	72429479	236	275
11125	5	2798959	2804774	ENSMUSG00000001504	ENSMUSG00000001504	ENSMUSG00000001504	13	73095295	73100584	236	275
11126	5	1930541	1935880	ENSMUSG00000021604	ENSMUSG00000021604	ENSMUSG00000021604	13	73726801	73735598	236	275
11127	5	1854509	1882610	ENSMUSG00000021606	ENSMUSG00000021606	ENSMUSG00000021606	13	73785878	73794457	236	275
11128	5	1851499	1852951	ENSMUSG00000021607	ENSMUSG00000021607	ENSMUSG00000021607	13	73797011	73798180	236	275
11129	5	1514544	1577076	ENSMUSG00000021608	ENSMUSG00000021608	ENSMUSG00000021608	13	73933228	73982413	236	275
11130	5	1445914	1498545	ENSMUSG00000021609	ENSMUSG00000021609	ENSMUSG00000021609	13	74002749	74044674	236	275
11131	5	1370983	1397999	ENSMUSG00000021610	ENSMUSG00000021610	ENSMUSG00000021610	13	74070042	74086641	236	275
11132	5	1306289	1348161	ENSMUSG00000021611	ENSMUSG00000021611	ENSMUSG00000021611	13	74092992	74115843	236	275
11133	5	1278470	1299304	ENSMUSG00000021612	ENSMUSG00000021612	ENSMUSG00000021612	13	74130039	74144003	236	275
11134	5	1254766	1275019	ENSMUSG00000021565	ENSMUSG00000021565	ENSMUSG00000021565	13	74145749	74166697	236	275
11135	5	1105475	1165106	ENSMUSG00000017756	ENSMUSG00000017756	ENSMUSG00000017756	13	74229705	74282754	236	275
11136	5	1061944	1091925	ENSMUSG00000021567	ENSMUSG00000021567	ENSMUSG00000021567	13	74286135	74313614	236	275
11137	5	945758	971235	ENSMUSG00000021569	ENSMUSG00000021569	ENSMUSG00000021569	13	74378462	74403727	236	275
11138	5	916856	945915	ENSMUSG00000057649	ENSMUSG00000057649	ENSMUSG00000057649	13	74403859	74426813	236	275
11139	5	712978	746466	ENSMUSG00000021573	ENSMUSG00000021573	ENSMUSG00000021573	13	74475480	74501755	236	275
11140	5	665461	706664	ENSMUSG00000021572	ENSMUSG00000021572	ENSMUSG00000021572	13	74502497	74528279	236	275
11141	5	526425	577447	ENSMUSG00000036123	ENSMUSG00000036123	ENSMUSG00000036123	13	74587517	74632063	236	275
11142	5	499264	520407	ENSMUSG00000034152	ENSMUSG00000034152	ENSMUSG00000034152	13	74635807	74674702	236	275
11143	5	357291	488225	ENSMUSG00000021575	ENSMUSG00000021575	ENSMUSG00000021575	13	74677120	74758311	236	275
11144	5	257875	271297	ENSMUSG00000021578	ENSMUSG00000021578	ENSMUSG00000021578	13	74816367	74825663	236	275
11145	5	244618	248468	ENSMUSG00000021579	ENSMUSG00000021579	ENSMUSG00000021579	13	74825583	74830007	236	275
11146	5	96122277	96169559	ENSMUSG00000021583	ENSMUSG00000021583	ENSMUSG00000021583	13	75105875	75158537	237	276
11147	5	96057245	96134274	ENSMUSG00000021585	ENSMUSG00000021585	ENSMUSG00000021585	13	75161895	75274762	237	276
11148	5	95751875	95794740	ENSMUSG00000021587	ENSMUSG00000021587	ENSMUSG00000021587	13	75555989	75598500	237	276
11149	5	95092635	95157827	ENSMUSG00000021589	ENSMUSG00000021589	ENSMUSG00000021589	13	76335549	76409869	237	276
11150	5	95014411	95044456	ENSMUSG00000021590	ENSMUSG00000021590	ENSMUSG00000021590	13	76433741	76464970	237	276
11151	5	95008239	95019542	ENSMUSG00000043190	ENSMUSG00000043190	ENSMUSG00000043190	13	76467537	76484564	237	276
11152	5	94981538	94983159	ENSMUSG00000045509	ENSMUSG00000045509	ENSMUSG00000045509	13	76521543	76522826	237	276
11153	5	94916608	94966524	ENSMUSG00000021592	ENSMUSG00000021592	ENSMUSG00000021592	13	76526714	76564615	237	276
11154	5	94825355	94916467	ENSMUSG00000033991	ENSMUSG00000033991	ENSMUSG00000033991	13	76574196	76651255	237	276
11155	5	94068957	94646035	ENSMUSG00000021596	ENSMUSG00000021596	ENSMUSG00000021596	13	77105255	77495357	237	276
11156	5	93979808	94059082	ENSMUSG00000021597	ENSMUSG00000021597	ENSMUSG00000021597	13	77506638	77554806	237	276
11157	5	93880683	93980058	ENSMUSG00000071252	ENSMUSG00000071252	ENSMUSG00000071252	13	77599098	77797914	237	276
11158	5	92979536	93436221	ENSMUSG00000064138	ENSMUSG00000064138	ENSMUSG00000064138	13	78172269	78629777	237	276
11159	5	92944799	92956077	ENSMUSG00000069171	ENSMUSG00000069171	ENSMUSG00000069171	13	78652996	78663332	237	276
11160	5	90700299	90714877	ENSMUSG00000074794	ENSMUSG00000074794	ENSMUSG00000074794	13	81347107	81359733	237	276
11161	5	89946407	90495788	ENSMUSG00000069170	ENSMUSG00000069170	ENSMUSG00000069170	13	81558819	82072696	237	276
11162	5	89847203	89861138	ENSMUSG00000035840	ENSMUSG00000035840	ENSMUSG00000035840	13	82135143	82149240	237	276
11163	5	89806482	89843881	ENSMUSG00000035834	ENSMUSG00000035834	ENSMUSG00000035834	13	82151180	82188304	237	276
11164	5	89789783	89805865	ENSMUSG00000051098	ENSMUSG00000051098	ENSMUSG00000051098	13	82188754	82230612	237	276
11165	5	89725287	89741359	ENSMUSG00000021537	ENSMUSG00000021537	ENSMUSG00000021537	13	82260629	82274494	237	276
11166	5	88051922	88214818	ENSMUSG00000005583	ENSMUSG00000005583	ENSMUSG00000005583	13	83981532	84141286	237	276
11167	5	87526781	87600421	ENSMUSG00000035762	ENSMUSG00000035762	ENSMUSG00000035762	13	84724340	84767990	237	276
11168	5	86725838	86744592	ENSMUSG00000021548	ENSMUSG00000021548	ENSMUSG00000021548	13	85662587	85686833	237	276
11169	5	86599461	86723487	ENSMUSG00000021549	ENSMUSG00000021549	ENSMUSG00000021549	13	85688185	85762139	237	276
11170	5	83272129	83716367	ENSMUSG00000034488	ENSMUSG00000034488	ENSMUSG00000034488	13	89295776	89796740	237	276
11171	5	82969380	83052906	ENSMUSG00000021613	ENSMUSG00000021613	ENSMUSG00000021613	13	90014993	90085856	237	276
11172	5	82803339	82912737	ENSMUSG00000021614	ENSMUSG00000021614	ENSMUSG00000021614	13	90131260	90205738	237	276
11173	5	82409073	82685333	ENSMUSG00000021615	ENSMUSG00000021615	ENSMUSG00000021615	13	90323160	90563768	237	276
11174	5	81303600	81587714	ENSMUSG00000021619	ENSMUSG00000021619	ENSMUSG00000021619	13	91409588	91698207	237	276
11175	5	80752008	81082665	ENSMUSG00000003992	ENSMUSG00000003992	ENSMUSG00000003992	13	91935401	92177668	237	276
11176	5	80661580	80725709	ENSMUSG00000021620	ENSMUSG00000021620	ENSMUSG00000021620	13	92215757	92260389	237	276
11177	5	80633178	80644872	ENSMUSG00000021621	ENSMUSG00000021621	ENSMUSG00000021621	13	92270773	92281944	237	276
11178	5	80564907	80597973	ENSMUSG00000021622	ENSMUSG00000021622	ENSMUSG00000021622	13	92327626	92351124	237	276
11179	5	80292314	80561731	ENSMUSG00000021708	ENSMUSG00000021708	ENSMUSG00000021708	13	92358674	92462287	237	276
11180	5	79986050	80208387	ENSMUSG00000014850	ENSMUSG00000014850	ENSMUSG00000014850	13	93312634	93455756	237	276
11181	5	79888331	79901854	ENSMUSG00000045034	ENSMUSG00000045034	ENSMUSG00000045034	13	93526846	93542410	237	276
11182	5	79819556	79873962	ENSMUSG00000034334	ENSMUSG00000034334	ENSMUSG00000034334	13	93550586	93584766	237	276
11183	5	79739601	79810925	ENSMUSG00000021706	ENSMUSG00000021706	ENSMUSG00000021706	13	93588502	93631563	237	276
11184	5	79651791	79653083	ENSMUSG00000046957	ENSMUSG00000046957	ENSMUSG00000046957	13	93675583	93676719	237	276
11185	5	79447709	79587594	ENSMUSG00000021703	ENSMUSG00000021703	ENSMUSG00000021703	13	93761771	93808890	237	276
11186	5	79366859	79414861	ENSMUSG00000021702	ENSMUSG00000021702	ENSMUSG00000021702	13	93852339	93895511	237	276
11187	5	79313964	79322841	ENSMUSG00000021704	ENSMUSG00000021704	ENSMUSG00000021704	13	93945540	93949833	237	276
11188	5	79021415	79131804	ENSMUSG00000047419	ENSMUSG00000047419	ENSMUSG00000047419	13	94141475	94199226	237	276
11189	5	78707505	78788599	ENSMUSG00000007617	ENSMUSG00000007617	ENSMUSG00000007617	13	94405552	94503051	237	276
11190	5	78609507	78653704	ENSMUSG00000021690	ENSMUSG00000021690	ENSMUSG00000021690	13	94534955	94600442	237	276
11191	5	78401303	78420708	ENSMUSG00000042118	ENSMUSG00000042118	ENSMUSG00000042118	13	94757013	94775052	237	276
11192	5	78329186	78401205	ENSMUSG00000042102	ENSMUSG00000042102	ENSMUSG00000042102	13	94775186	94853581	237	276
11193	5	78108788	78317366	ENSMUSG00000042093	ENSMUSG00000042093	ENSMUSG00000042093	13	94872512	95041668	237	276
11194	5	77816794	77841965	ENSMUSG00000045312	ENSMUSG00000045312	ENSMUSG00000045312	13	95158549	95296163	237	276
11195	5	77692110	77810634	ENSMUSG00000021687	ENSMUSG00000021687	ENSMUSG00000021687	13	95304753	95353775	237	276
11196	5	77332105	77626284	ENSMUSG00000021686	ENSMUSG00000021686	ENSMUSG00000021686	13	95459684	95667008	237	276
11197	5	77022827	77107941	ENSMUSG00000042043	ENSMUSG00000042043	ENSMUSG00000042043	13	95889634	95943621	237	276
11198	5	76960293	76970278	ENSMUSG00000021685	ENSMUSG00000021685	ENSMUSG00000021685	13	95976326	95984380	237	276
11199	5	76762514	76824088	ENSMUSG00000042015	ENSMUSG00000042015	ENSMUSG00000042015	13	96077075	96122780	237	276
11200	5	76542462	76759837	ENSMUSG00000021684	ENSMUSG00000021684	ENSMUSG00000021684	13	96125204	96323749	237	276
11201	5	76403653	76418904	ENSMUSG00000041995	ENSMUSG00000041995	ENSMUSG00000041995	13	96425936	96438540	237	276
11202	5	76361987	76396815	ENSMUSG00000021681	ENSMUSG00000021681	ENSMUSG00000021681	13	96451413	96476033	237	276
11203	5	76284436	76301055	ENSMUSG00000021680	ENSMUSG00000021680	ENSMUSG00000021680	13	96532075	96545471	237	276
11204	5	76206882	76252607	ENSMUSG00000021679	ENSMUSG00000021679	ENSMUSG00000021679	13	96578000	96579354	237	276
11205	5	76150610	76166895	ENSMUSG00000021678	ENSMUSG00000021678	ENSMUSG00000021678	13	96612414	96625917	237	276
11206	5	76047542	76067054	ENSMUSG00000048376	ENSMUSG00000048376	ENSMUSG00000048376	13	96702488	96719173	237	276
11207	5	75734905	76039711	ENSMUSG00000021676	ENSMUSG00000021676	ENSMUSG00000021676	13	96732731	96938623	237	276
11208	5	75947064	75954996	ENSMUSG00000021675	ENSMUSG00000021675	ENSMUSG00000021675	13	96797656	96803438	237	276
11209	5	75463332	75657172	ENSMUSG00000051111	ENSMUSG00000051111	ENSMUSG00000051111	13	97061862	97189573	237	276
11210	5	75005705	75048989	ENSMUSG00000021671	ENSMUSG00000021671	ENSMUSG00000021671	13	97489065	97516268	237	276
11211	5	74984708	75002621	ENSMUSG00000047117	ENSMUSG00000047117	ENSMUSG00000047117	13	97517456	97536685	237	276
11212	5	74843337	74930889	ENSMUSG00000021668	ENSMUSG00000021668	ENSMUSG00000021668	13	97581386	97643184	237	276
11213	5	74702684	74843719	ENSMUSG00000021669	ENSMUSG00000021669	ENSMUSG00000021669	13	97643434	97738249	237	276
11214	5	74668790	74693685	ENSMUSG00000021670	ENSMUSG00000021670	ENSMUSG00000021670	13	97750033	97771547	237	276
11215	5	74360257	74361618	ENSMUSG00000049586	ENSMUSG00000049586	ENSMUSG00000049586	13	98046888	98048213	237	276
11216	5	74113044	74197769	ENSMUSG00000041817	ENSMUSG00000041817	ENSMUSG00000041817	13	98172342	98230214	237	276
11217	5	74052785	74098952	ENSMUSG00000021666	ENSMUSG00000021666	ENSMUSG00000021666	13	98238737	98277162	237	276
11218	5	74016725	74052869	ENSMUSG00000021665	ENSMUSG00000021665	ENSMUSG00000021665	13	98277106	98298969	237	276
11219	5	73958990	73973005	ENSMUSG00000041773	ENSMUSG00000041773	ENSMUSG00000041773	13	98341869	98353733	237	276
11220	5	72897336	72913540	ENSMUSG00000041747	ENSMUSG00000041747	ENSMUSG00000041747	13	99347002	99363149	237	276
11221	5	72883919	72897267	ENSMUSG00000021661	ENSMUSG00000021661	ENSMUSG00000021661	13	99363399	99374070	237	276
11222	5	72830036	72837075	ENSMUSG00000021660	ENSMUSG00000021660	ENSMUSG00000021660	13	99410052	99417124	237	276
11223	5	72504827	72506724	ENSMUSG00000046082	ENSMUSG00000046082	ENSMUSG00000046082	13	99735135	99737495	237	276
11224	5	72452158	72463397	ENSMUSG00000052485	ENSMUSG00000052485	ENSMUSG00000052485	13	99786395	99794988	237	276
11225	5	72287655	72422104	ENSMUSG00000041685	ENSMUSG00000041685	ENSMUSG00000041685	13	99826210	99906515	237	276
11226	5	72148171	72248316	ENSMUSG00000009470	ENSMUSG00000009470	ENSMUSG00000009470	13	99942895	100026500	237	276
11227	5	71771486	71839002	ENSMUSG00000050919	ENSMUSG00000050919	ENSMUSG00000050919	13	100284980	100347194	237	276
11228	5	71651958	71690754	ENSMUSG00000021650	ENSMUSG00000021650	ENSMUSG00000021650	13	100419808	100444844	237	276
11229	5	71550992	71651844	ENSMUSG00000041632	ENSMUSG00000041632	ENSMUSG00000041632	13	100444943	100515718	237	276
11230	5	71438874	71538609	ENSMUSG00000052727	ENSMUSG00000052727	ENSMUSG00000052727	13	100524598	100616697	237	276
11231	5	71050750	71052628	ENSMUSG00000021647	ENSMUSG00000021647	ENSMUSG00000021647	13	100999170	101000885	237	276
11232	5	70918910	70988981	ENSMUSG00000021646	ENSMUSG00000021646	ENSMUSG00000021646	13	101048781	101115886	237	276
11233	5	70787451	70899402	ENSMUSG00000049658	ENSMUSG00000049658	ENSMUSG00000049658	13	101118254	101175878	237	276
11234	5	68823875	68885887	ENSMUSG00000021638	ENSMUSG00000021638	ENSMUSG00000021638	13	101597574	101652864	237	276
11235	5	68746710	68773644	ENSMUSG00000021636	ENSMUSG00000021636	ENSMUSG00000021636	13	101696216	101717205	237	276
11236	5	68701395	68746382	ENSMUSG00000021635	ENSMUSG00000021635	ENSMUSG00000021635	13	101717422	101751308	237	276
11237	5	68614312	68655430	ENSMUSG00000048924	ENSMUSG00000048924	ENSMUSG00000048924	13	101769767	101797497	237	276
11238	5	68566456	68609006	ENSMUSG00000069089	ENSMUSG00000069089	ENSMUSG00000069089	13	101803575	101831184	237	276
11239	5	68521131	68541940	ENSMUSG00000045273	ENSMUSG00000045273	ENSMUSG00000045273	13	101859943	101876156	237	276
11240	5	68425839	68462648	ENSMUSG00000021629	ENSMUSG00000021629	ENSMUSG00000021629	13	101902905	101933684	237	276
11241	5	67547360	67630810	ENSMUSG00000041417	ENSMUSG00000041417	ENSMUSG00000041417	13	102784340	102868441	237	276
11242	5	66513872	66528368	ENSMUSG00000021624	ENSMUSG00000021624	ENSMUSG00000021624	13	103813970	103827337	237	276
11243	5	66445670	66498848	ENSMUSG00000034751	ENSMUSG00000034751	ENSMUSG00000034751	13	103855390	103907831	237	276
11244	5	65475802	65515199	ENSMUSG00000032621	ENSMUSG00000032621	ENSMUSG00000032621	13	104861997	104884964	237	276
11245	5	65258059	65414133	ENSMUSG00000021709	ENSMUSG00000021709	ENSMUSG00000021709	13	104939168	105040896	237	276
11246	5	65053779	65160867	ENSMUSG00000021710	ENSMUSG00000021710	ENSMUSG00000021710	13	105145328	105193412	237	276
11247	5	64997511	65053697	ENSMUSG00000042743	ENSMUSG00000042743	ENSMUSG00000042743	13	105230172	105261837	237	276
11248	5	64956628	64997648	ENSMUSG00000021711	ENSMUSG00000021711	ENSMUSG00000021711	13	105262536	105298797	237	276
11249	5	64921263	64956199	ENSMUSG00000021712	ENSMUSG00000021712	ENSMUSG00000021712	13	105299209	105323857	237	276
11250	5	64894873	64919124	ENSMUSG00000021713	ENSMUSG00000021713	ENSMUSG00000021713	13	105325506	105349204	237	276
11251	5	64849350	64886531	ENSMUSG00000021714	ENSMUSG00000021714	ENSMUSG00000021714	13	105349770	105369999	237	276
11252	5	64480322	64813460	ENSMUSG00000046169	ENSMUSG00000046169	ENSMUSG00000046169	13	105415110	105614083	237	276
11253	5	64100511	64350346	ENSMUSG00000021715	ENSMUSG00000021715	ENSMUSG00000021715	13	105751545	105937277	237	276
11254	5	64052109	64100209	ENSMUSG00000021716	ENSMUSG00000021716	ENSMUSG00000021716	13	105937572	105957930	237	276
11255	5	64022207	64049599	ENSMUSG00000042655	ENSMUSG00000042655	ENSMUSG00000042655	13	105965665	105984275	237	276
11256	5	63838208	63943875	ENSMUSG00000021719	ENSMUSG00000021719	ENSMUSG00000021719	13	106069562	106175216	237	276
11257	5	63532391	63701394	ENSMUSG00000021720	ENSMUSG00000021720	ENSMUSG00000021720	13	106250869	106391421	237	276
11258	5	63292034	63293302	ENSMUSG00000021721	ENSMUSG00000021721	ENSMUSG00000021721	13	106564148	106568501	237	276
11259	5	61768864	61958901	ENSMUSG00000042590	ENSMUSG00000042590	ENSMUSG00000042590	13	107914824	108057300	237	276
11260	5	61720108	61735485	ENSMUSG00000021692	ENSMUSG00000021692	ENSMUSG00000021692	13	108067563	108080609	237	276
11261	5	61637835	61718998	ENSMUSG00000021693	ENSMUSG00000021693	ENSMUSG00000021693	13	108081645	108142499	237	276
11262	5	60852928	60877754	ENSMUSG00000032846	ENSMUSG00000032846	ENSMUSG00000032846	13	108846820	109010225	237	276
11263	5	60489297	60494052	ENSMUSG00000071180	ENSMUSG00000071180	ENSMUSG00000071180	13	109165193	109169916	237	276
11264	5	60276744	60484609	ENSMUSG00000068184	ENSMUSG00000068184	ENSMUSG00000068184	13	109173338	109279361	237	276
11265	5	60205415	60276648	ENSMUSG00000021694	ENSMUSG00000021694	ENSMUSG00000021694	13	109279483	109315258	237	276
11266	5	60083377	60119016	ENSMUSG00000021696	ENSMUSG00000021696	ENSMUSG00000021696	13	109335150	109404225	237	276
11267	5	59928496	60031745	ENSMUSG00000021697	ENSMUSG00000021697	ENSMUSG00000021697	13	109437084	109510306	237	276
11268	5	58306248	59225206	ENSMUSG00000021699	ENSMUSG00000021699	ENSMUSG00000021699	13	110153332	111072222	237	276
11269	5	57914671	58190970	ENSMUSG00000021700	ENSMUSG00000021700	ENSMUSG00000021700	13	111181960	111400980	237	276
11270	5	57825824	57827942	ENSMUSG00000046006	ENSMUSG00000046006	ENSMUSG00000046006	13	111473493	111478050	237	276
11271	5	57785566	57791844	ENSMUSG00000021701	ENSMUSG00000021701	ENSMUSG00000021701	13	111515940	111521724	237	276
11272	5	56813161	56814291	ENSMUSG00000055194	ENSMUSG00000055194	ENSMUSG00000055194	13	112375891	112378627	237	276
11273	5	56545715	56594666	ENSMUSG00000032745	ENSMUSG00000032745	ENSMUSG00000032745	13	112546560	112610736	237	276
11274	5	56251186	56283702	ENSMUSG00000032727	ENSMUSG00000032727	ENSMUSG00000032727	13	112807229	112838514	237	276
11275	5	56147216	56225472	ENSMUSG00000021754	ENSMUSG00000021754	ENSMUSG00000021754	13	112867468	112929861	237	276
11276	5	55431599	55564943	ENSMUSG00000049985	ENSMUSG00000049985	ENSMUSG00000049985	13	113409698	113504880	237	276
11277	5	55266680	55326529	ENSMUSG00000021756	ENSMUSG00000021756	ENSMUSG00000021756	13	113584987	113627719	237	276
11278	5	55183156	55248705	ENSMUSG00000050377	ENSMUSG00000050377	ENSMUSG00000050377	13	113643673	113701540	237	276
11279	5	55069609	55148362	ENSMUSG00000021758	ENSMUSG00000021758	ENSMUSG00000021758	13	113719214	113773179	237	276
11280	5	54957435	55029579	ENSMUSG00000047789	ENSMUSG00000047789	ENSMUSG00000047789	13	113781654	113853776	237	276
11281	5	54756442	54866630	ENSMUSG00000021759	ENSMUSG00000021759	ENSMUSG00000021759	13	113921772	113988759	237	276
11282	5	54639345	54757166	ENSMUSG00000016018	ENSMUSG00000016018	ENSMUSG00000016018	13	113988658	114048258	237	276
11283	5	54587831	54639278	ENSMUSG00000042426	ENSMUSG00000042426	ENSMUSG00000042426	13	114048598	114090305	237	276
11284	5	54562740	54565265	ENSMUSG00000042417	ENSMUSG00000042417	ENSMUSG00000042417	13	114108776	114111654	237	276
11285	5	54491778	54498628	ENSMUSG00000021760	ENSMUSG00000021760	ENSMUSG00000021760	13	114163641	114167266	237	276
11286	5	54434230	54441818	ENSMUSG00000023132	ENSMUSG00000023132	ENSMUSG00000023132	13	114214703	114221857	237	276
11287	5	54355838	54366155	ENSMUSG00000042385	ENSMUSG00000042385	ENSMUSG00000042385	13	114292752	114301775	237	276
11288	5	54309449	54317248	ENSMUSG00000042379	ENSMUSG00000042379	ENSMUSG00000042379	13	114330537	114338984	237	276
11289	5	53849540	53851426	ENSMUSG00000042364	ENSMUSG00000042364	ENSMUSG00000042364	13	114713063	114739442	237	276
11290	5	53787202	53787964	ENSMUSG00000051456	ENSMUSG00000051456	ENSMUSG00000051456	13	114783906	114784370	237	276
11291	5	53216371	53642160	ENSMUSG00000042348	ENSMUSG00000042348	ENSMUSG00000042348	13	115043125	115275734	237	276
11292	5	52892226	53014925	ENSMUSG00000021764	ENSMUSG00000021764	ENSMUSG00000021764	13	115409302	115508958	237	276
11293	5	52812174	52817659	ENSMUSG00000021765	ENSMUSG00000021765	ENSMUSG00000021765	13	115574301	115579608	237	276
11294	5	52427266	52441336	ENSMUSG00000015536	ENSMUSG00000015536	ENSMUSG00000015536	13	115939148	115949884	237	276
11295	5	52321014	52423805	ENSMUSG00000015533	ENSMUSG00000015533	ENSMUSG00000015533	13	115956703	116052930	237	276
11296	5	52119893	52285241	ENSMUSG00000042284	ENSMUSG00000042284	ENSMUSG00000042284	13	116080957	116222842	237	276
11297	5	50714715	50726024	ENSMUSG00000042258	ENSMUSG00000042258	ENSMUSG00000042258	13	117418963	117430370	237	276
11298	5	49998570	50178113	ENSMUSG00000021725	ENSMUSG00000021725	ENSMUSG00000021725	13	117974296	118144668	237	276
11299	5	49730236	49772958	ENSMUSG00000021728	ENSMUSG00000021728	ENSMUSG00000021728	13	118339913	118393379	237	276
11300	5	45297730	45731977	ENSMUSG00000021730	ENSMUSG00000021730	ENSMUSG00000021730	13	118721981	119096083	237	276
11301	5	44844784	44856287	ENSMUSG00000021731	ENSMUSG00000021731	ENSMUSG00000021731	13	119499391	119506533	237	276
11302	5	44340854	44424541	ENSMUSG00000021732	ENSMUSG00000021732	ENSMUSG00000021732	13	119833907	119910993	237	276
11303	5	43638582	43743264	ENSMUSG00000025453	ENSMUSG00000025453	ENSMUSG00000025453	13	120454321	120527870	237	276
11304	5	43522567	43550944	ENSMUSG00000062822	ENSMUSG00000062822	ENSMUSG00000062822	13	120581632	120604790	237	276
11305	5	43480512	43519695	ENSMUSG00000074634	ENSMUSG00000074634	ENSMUSG00000074634	13	120611577	120614343	237	276
11306	3	57969167	58133015	ENSMUSG00000025278	ENSMUSG00000025278	ENSMUSG00000025278	14	6608561	6743464	238	277
11307	3	58153454	58175893	ENSMUSG00000025279	ENSMUSG00000025279	ENSMUSG00000025279	14	6757066	6786061	238	277

11308	3	58198349	58255499	ENSMUSG00000025277	ENSMUSG00000025277	ENSMUSG00000025277	14	6794781	6848434	238	277		
11309	3	58267051	58279262	ENSMUSG00000023156	ENSMUSG00000023156	ENSMUSG00000023156	14	6872246	6883715	238	277		
11310	3	58293657	58385918	ENSMUSG00000033885	ENSMUSG00000033885	ENSMUSG00000033885	14	6890122	6956649	238	277		
11311	3	58388398	58394594	ENSMUSG00000021748	ENSMUSG00000021748	ENSMUSG00000021748	14	6956612	6964490	238	277		
11312	3	58452881	58463119	ENSMUSG00000021752	ENSMUSG00000021752	ENSMUSG00000021752	14	7007896	7017317	238	277		
11313	3	58465906	58497956	ENSMUSG00000021751	ENSMUSG00000021751	ENSMUSG00000021751	14	7019267	7052692	238	277		
11314	3	58526228	58588377	ENSMUSG00000021750	ENSMUSG00000021750	ENSMUSG00000021750	14	7090030	7103532	238	277		
11315	3	58594715	58627615	ENSMUSG00000021749	ENSMUSG00000021749	ENSMUSG00000021749	14	7143033	7158662	238	277		
11316	3	58702777	59010751	ENSMUSG00000021747	ENSMUSG00000021747	ENSMUSG00000021747	14	7224978	7460026	238	277		
11317	3	59712992	60497735	ENSMUSG00000060579	ENSMUSG00000060579	ENSMUSG00000060579	14	8367196	9215384	238	277		
11318	3	61523002	62254022	ENSMUSG00000021745	ENSMUSG00000021745	ENSMUSG00000021745	14	10347515	11031140	238	277		
11319	3	62279751	62294358	ENSMUSG00000033111	ENSMUSG00000033111	ENSMUSG00000033111	14	11077500	11096498	238	277		
11320	3	62330407	62334061	ENSMUSG00000021743	ENSMUSG00000021743	ENSMUSG00000021743	14	11135369	11139095	238	277		
11321	3	62359062	62836094	ENSMUSG00000054423	ENSMUSG00000054423	ENSMUSG00000054423	14	11165827	11616320	238	277		
11322	3	63404044	63577636	ENSMUSG00000056296	ENSMUSG00000056296	ENSMUSG00000056296	14	12078252	12407248	238	277		
11323	3	63613404	63624811	ENSMUSG00000044772	ENSMUSG00000044772	ENSMUSG00000044772	14	12464143	12476415	238	277		
11324	3	63794587	63824549	ENSMUSG00000053453	ENSMUSG00000053453	ENSMUSG00000053453	14	12742527	12747924	238	277		
11325	3	63971271	63984189	ENSMUSG00000021737	ENSMUSG00000021737	ENSMUSG00000021737	14	12905451	12914207	238	277		
11326	1	246617533	246618459	ENSMUSG00000052417	ENSMUSG00000052417	ENSMUSG00000052417	14	12968397	12969347	out-of-place	278	here	singletons were called as HSBs by AG
11327	1	246635919	246637028	ENSMUSG00000072707	ENSMUSG00000072707	ENSMUSG00000072707	14	13121380	13122333	out-of-place	278	singletons were called as HSBs by AG	0 -> 1
11328	3	26639301	26727266	ENSMUSG00000045201	ENSMUSG00000045201	ENSMUSG00000045201	14	14150787	14232140	239	279		
11329	3	25806575	25811028	ENSMUSG00000021786	ENSMUSG00000021786	ENSMUSG00000021786	14	15031926	15043075	239	279		
11330	3	25735808	25799997	ENSMUSG00000021785	ENSMUSG00000021785	ENSMUSG00000021785	14	15042581	15105193	239	279		
11331	3	25614479	25680792	ENSMUSG00000017485	ENSMUSG00000017485	ENSMUSG00000017485	14	15158453	15224054	239	279		
11332	3	25444758	25614424	ENSMUSG00000017491	ENSMUSG00000017491	ENSMUSG00000017491	14	15224109	15612134	239	279		
11333	3	24134709	24181849	ENSMUSG00000021779	ENSMUSG00000021779	ENSMUSG00000021779	14	16775144	16826933	239	279		
11334	3	23909590	23927399	ENSMUSG00000021772	ENSMUSG00000021772	ENSMUSG00000021772	14	17064260	17077121	239	279		
11335	3	23225195	23606326	ENSMUSG00000058317	ENSMUSG00000058317	ENSMUSG00000058317	14	17367420	17681567	239	279		
11336	14	51541271	51605696	ENSMUSG00000021806	ENSMUSG00000021806	ENSMUSG00000021806	14	18541980	18601770	240	280		
11337	14	51383702	51503506	ENSMUSG00000043004	ENSMUSG00000043004	ENSMUSG00000043004	14	18663255	18767945	240	280		
11338	6	39179818	39190943	ENSMUSG00000045107	ENSMUSG00000045107	ENSMUSG00000045107	14	18866356	18873794	241	281		
11339	6	39264727	39305229	ENSMUSG00000023243	ENSMUSG00000023243	ENSMUSG00000023243	14	18930754	18972478	241	281		
11340	6	39390878	39398294	ENSMUSG00000023387	ENSMUSG00000023387	ENSMUSG00000023387	14	19049928	19057684	241	281		
11341	10	74540223	74561592	ENSMUSG00000021809	ENSMUSG00000021809	ENSMUSG00000021809	14	19083274	19105311	242	282		
11342	10	74563420	74597823	ENSMUSG00000021810	ENSMUSG00000021810	ENSMUSG00000021810	14	19108691	19136636	242	282		
11343	10	74597930	74674268	ENSMUSG00000039599	ENSMUSG00000039599	ENSMUSG00000039599	14	19136774	19172043	242	282		
11344	10	74672588	74678626	ENSMUSG00000021811	ENSMUSG00000021811	ENSMUSG00000021811	14	19173190	19177432	242	282		
11345	10	74683526	74789458	ENSMUSG00000039543	ENSMUSG00000039543	ENSMUSG00000039543	14	19182876	19237169	242	282		
11346	10	74805209	74843831	ENSMUSG00000021814	ENSMUSG00000021814	ENSMUSG00000021814	14	19243838	19268492	242	282		
11347	10	74854193	74858051	ENSMUSG00000021815	ENSMUSG00000021815	ENSMUSG00000021815	14	19271421	19285233	242	282		
11348	10	74866192	74925765	ENSMUSG00000021816	ENSMUSG00000021816	ENSMUSG00000021816	14	19288592	19335096	242	282		
11349	10	74928059	74983104	ENSMUSG00000021817	ENSMUSG00000021817	ENSMUSG00000021817	14	19337470	19350548	242	282		
11350	10	75061418	75071521	ENSMUSG00000068697	ENSMUSG00000068697	ENSMUSG00000068697	14	19437657	19445055	242	282		
11351	10	75074645	75080771	ENSMUSG00000039376	ENSMUSG00000039376	ENSMUSG00000039376	14	19447970	19456858	242	282		
11352	10	75174138	75201923	ENSMUSG00000039367	ENSMUSG00000039367	ENSMUSG00000039367	14	19462895	19591970	242	282		
11353	10	75202055	75205983	ENSMUSG00000039357	ENSMUSG00000039357	ENSMUSG00000039357	14	19483520	19488749	242	282		
11354	10	75211827	75213416	ENSMUSG00000063787	ENSMUSG00000063787	ENSMUSG00000063787	14	19491600	19492969	242	282		
11355	10	75231675	75241348	ENSMUSG00000039308	ENSMUSG00000039308	ENSMUSG00000039308	14	19512289	19519664	242	282		
11356	10	75242265	75304344	ENSMUSG00000021820	ENSMUSG00000021820	ENSMUSG00000021820	14	19523427	19582640	242	282		
11357	10	75340896	75347260	ENSMUSG00000021822	ENSMUSG00000021822	ENSMUSG00000021822	14	19625259	19631940	242	282		
11358	10	75427878	75549916	ENSMUSG00000021823	ENSMUSG00000021823	ENSMUSG00000021823	14	19717985	19822228	242	282		
11359	10	75551530	75580827	ENSMUSG00000021824	ENSMUSG00000021824	ENSMUSG00000021824	14	19823362	19840737	242	282		
11360	10	75580971	76139066	ENSMUSG00000039197	ENSMUSG00000039197	ENSMUSG00000039197	14	19841183	20237107	242	282		
11361	10	76256389	76462386	ENSMUSG00000021767	ENSMUSG00000021767	ENSMUSG00000021767	14	20288420	20460685	242	282		
11362	10	76467600	76488278	ENSMUSG00000063821	ENSMUSG00000063821	ENSMUSG00000063821	14	20465124	20503128	242	282		
11363	10	76524199	76538976	ENSMUSG00000021768	ENSMUSG00000021768	ENSMUSG00000021768	14	20521947	20531431	242	282		
11364	10	76541235	76606595	ENSMUSG00000021770	ENSMUSG00000021770	ENSMUSG00000021770	14	20539083	20587277	242	282		
11365	10	76663733	76665775	ENSMUSG00000021773	ENSMUSG00000021773	ENSMUSG00000021773	14	20634413	20637462	242	282		
11366	10	76827594	76831670	ENSMUSG00000039081	ENSMUSG00000039081	ENSMUSG00000039081	14	20773440	20777568	242	282		
11367	10	76861217	77987095	ENSMUSG00000063458	ENSMUSG00000063458	ENSMUSG00000063458	14	20808431	21844586	242	282		
11368	10	78317030	79067406	ENSMUSG00000063142	ENSMUSG00000063142	ENSMUSG00000063142	14	22077983	22792677	242	282		
11369	10	79220557	79356384	ENSMUSG00000021782	ENSMUSG00000021782	ENSMUSG00000021782	14	22966420	23077915	242	282		
11370	10	79404913	79459309	ENSMUSG00000025280	ENSMUSG00000025280	ENSMUSG00000025280	14	23281170	23319474	242	282		
11371	10	79463580	79470477	ENSMUSG00000025290	ENSMUSG00000025290	ENSMUSG00000025290	14	23324215	23328251	242	282		
11372	10	80631396	80744273	ENSMUSG00000007817	ENSMUSG00000007817	ENSMUSG00000007817	14	24292476	24497903	242	282		
11373	10	80777226	80785087	ENSMUSG00000021868	ENSMUSG00000021868	ENSMUSG00000021868	14	24527338	24533648	242	282		
11374	10	80812091	80875380	ENSMUSG00000055538	ENSMUSG00000055538	ENSMUSG00000055538	14	24544833	24684033	242	282		
11375	10	81900625	81955413	ENSMUSG00000021866	ENSMUSG00000021866	ENSMUSG00000021866	14	24675354	24719254	out-of-place	282		
11376	3	57718214	57889934	ENSMUSG00000021870	ENSMUSG00000021870	ENSMUSG00000021870	14	25246359	25366924	243	283		
11377	3	57586222	57653843	ENSMUSG00000040818	ENSMUSG00000040818	ENSMUSG00000040818	14	25412762	25466586	243	283		
11378	3	57532145	57558175	ENSMUSG00000021877	ENSMUSG00000021877	ENSMUSG00000021877	14	25471381	25490442	243	283		
11379	3	57517044	57522721	ENSMUSG00000043702	ENSMUSG00000043702	ENSMUSG00000043702	14	25497299	25503030	243	283		
11380	3	57429635	57503637	ENSMUSG00000021879	ENSMUSG00000021879	ENSMUSG00000021879	14	25526596	25557686	243	283		
11381	3	57302817	57361295	ENSMUSG00000072668	ENSMUSG00000072668	ENSMUSG00000072668	14	25625669	25718515	243	283		
11382	3	57286815	57301165	ENSMUSG00000021898	ENSMUSG00000021898	ENSMUSG00000021898	14	25721486	25741300	243	283		
11383	3	57236894	57282536	ENSMUSG00000040760	ENSMUSG00000040760	ENSMUSG00000040760	14	25745872	25797435	243	283		
11384	3	57207265	57209320	ENSMUSG00000040726	ENSMUSG00000040726	ENSMUSG00000040726	14	25827246	25829210	243	283		
11385	3	57103318	57174443	ENSMUSG00000040717	ENSMUSG00000040717	ENSMUSG00000040717	14	25865966	25929393	243	283		
11386	3	56736486	57088333	ENSMUSG00000021895	ENSMUSG00000021895	ENSMUSG00000021895	14	26064923	26230788	243	283		
11387	3	56632105	56682707	ENSMUSG00000040651	ENSMUSG00000040651	ENSMUSG00000040651	14	26276669	26308272	243	283		
11388	3	56566231	56630876	ENSMUSG00000046753	ENSMUSG00000046753	ENSMUSG00000046753	14	26309285	26335344	243	283		
11389	3	55517376	56477431	ENSMUSG00000040640	ENSMUSG00000040640	ENSMUSG00000040640	14	26449326	27305403	243	283		
11390	3	55479112	55489996	ENSMUSG00000021994	ENSMUSG00000021994	ENSMUSG00000021994	14	27332339	27352300	243	283		
11391	3	54131733	55083622	ENSMUSG00000021991	ENSMUSG00000021991	ENSMUSG00000021991	14	27732276	28548875	243	283		
11392	3	54927425	54937112	ENSMUSG00000045776	ENSMUSG00000045776	ENSMUSG00000045776	14	27845265	27854891	243	283		
11393	3	53855612	53874866	ENSMUSG00000015966	ENSMUSG00000015966	ENSMUSG00000015966	14	28825177	28837905	243	283		
11394	3	53826683	53855216	ENSMUSG00000015970	ENSMUSG00000015970	ENSMUSG00000015970	14	28838032	28869475	243	283		
11395	3	53504116	53821001	ENSMUSG00000015968	ENSMUSG00000015968	ENSMUSG00000015968	14	28871082	29320165	243	283		
11396	3	53296430	53356677	ENSMUSG00000021962	ENSMUSG00000021962	ENSMUSG00000021962	14	29308621	29356072	243	283		
11397	3	53233770	53265056	ENSMUSG00000021957	ENSMUSG00000021957	ENSMUSG00000021957	14	29378158	29403731	243	283		
11398	3	53170263	53201771	ENSMUSG00000021948	ENSMUSG00000021948	ENSMUSG00000021948	14	29424378	29439321	243	283		
11399	3	52913668	53055110	ENSMUSG00000006527	ENSMUSG00000006527	ENSMUSG00000006527	14	29595009	29646972	243	283		
11400	3	52822341	52844785	ENSMUSG00000021922	ENSMUSG00000021922	ENSMUSG00000021922	14	29715511	29730990	243	283		
11401	3	52803852	52817852	ENSMUSG00000006522	ENSMUSG00000006522	ENSMUSG00000006522	14	29737582	29752553	243	283		
11402	3	52786648	52801117	ENSMUSG00000006529	ENSMUSG00000006529	ENSMUSG00000006529	14	29758373	29772286	243	283		
11403	3	52720833	52779995	ENSMUSG00000021918	ENSMUSG00000021918	ENSMUSG00000021918	14	29780495	29816457	243	283		
11404	3	52714897	52717249	ENSMUSG00000021917	ENSMUSG00000021917	ENSMUSG00000021917	14	29828836	29830549	243	283		
11405	3	52703548	52715088	ENSMUSG00000021916	ENSMUSG00000021916	ENSMUSG00000021916	14	29830409	29840999	243	283		
11406	3	52695149	52703548	ENSMUSG00000042354	ENSMUSG00000042354	ENSMUSG00000042354	14	29841445	29848070	243	283		
11407	3	52545661	52549584	ENSMUSG00000058351	ENSMUSG00000058351	ENSMUSG00000058351	14	29953515	29957939	243	283		
11408	3	52533443	52544114	ENSMUSG00000071547	ENSMUSG00000071547	ENSMUSG00000071547	14	29963862	29968132	243	283		
11409	3	52504396	52533549	ENSMUSG00000042286	ENSMUSG00000042286	ENSMUSG00000042286	14	29968022	29997581	243	283		
11410	3	52464174	52502124	ENSMUSG00000021910	ENSMUSG00000021910	ENSMUSG00000021910	14	29999937	30020559	243	283		
11411	3	52460158	52463098	ENSMUSG00000021909	ENSMUSG00000021909	ENSMUSG00000021909	14	30037321	30040720	243	283		
11412	3	52442308	52454083	ENSMUSG00000021904	ENSMUSG00000021904	ENSMUSG00000021904	14	30046869	30059359	243	283		
11413	3	52419567	52432696	ENSMUSG00000021902	ENSMUSG00000021902	ENSMUSG00000021902	14	30066705	30080227	243	283		
11414	3	52410069	52419058	ENSMUSG00000021901	ENSMUSG00000021901	ENSMUSG00000021901	14	30080483	30088938	243	283		
11415	3	52325560	52409547	ENSMUSG00000019027	ENSMUSG00000019027	ENSMUSG00000019027	14	30089438	30148946	243	283		
11416	3	15222737	15269426	ENSMUSG00000021893	ENSMUSG00000021893	ENSMUSG00000021893	14	30165733	30200993	244	284		
11417	3	15427841	15444029	ENSMUSG00000021891	ENSMUSG00000021891	ENSMUSG00000021891	14	30307329	30324039	244	284		
11418	3	15443866	15459121	ENSMUSG00000021890	ENSMUSG00000021890	ENSMUSG00000021890	14	30324152	30338867	244	284		
11419	3	15466645	15538262	ENSMUSG00000057606	ENSMUSG00000057606	ENSMUSG00000057606	14	30353422	30382865	244	284		
11420	3	15577246	15618071	ENSMUSG00000021884	ENSMUSG00000021884	ENSMUSG00000021884	14	30436235	30469970	244	284		
11421	3	15618259	15662328	ENSMUSG00000021900	ENSMUSG00000021900	ENSMUSG00000021900	14	30491068	30497192	244	284		
11422	3	15683747	15813032	ENSMUSG00000014496	ENSMUSG00000014496	ENSMUSG00000014496	14	30531029	30608014	244	284		
11423	3	16191160	16245151	ENSMUSG00000021903	ENSMUSG00000021903	ENSMUSG00000021903	14	30857998	30891206	244	284		
11424	3	16281712	16320487	ENSMUSG00000021906	ENSMUSG00000021906	ENSMUSG00000021906	14	30914959	30932211	244	284		
11425	10	51219559	51232596	ENSMUSG00000021907	ENSMUSG00000021907	ENSMUSG00000021907	14	30977084	30987336	245	285		
11426	10	50696446	50743434	ENSMUSG00000021911	ENSMUSG00000021911	ENSMUSG00000021911	14	31030980	31126556	245	285		
11427	10	50612695	50640375	ENSMUSG00000021913	ENSMUSG00000021913	ENSMUSG00000021913	14	31154804	31176632	245	285		
11428	10	50557738	50588313	ENSMUSG00000072473	ENSMUSG00000072473	ENSMUSG00000072473	14	31205096	31217400	245	285		
11429	10	50492062	50543156	ENSMUSG00000021919	ENSMUSG00000021919	ENSMUSG00000021919	14	31237212	31294918	245	285		
11430	10	50242243	50273992	ENSMUSG00000041730	ENSMUSG00000041730	ENSMUSG00000041730	14	31428936	31478241	245	285		
11431	10	50177244	50205538	ENSMUSG00000071540	ENSMUSG00000071540	ENSMUSG00000071540	14	31491914	31493098	245	285		
11432	10	49896258	49993560	ENSMUSG00000050666	ENSMUSG00000050666	ENSMUSG00000050666	14	31685848	31768581	245	285		
11433	10	49666640	49860813	ENSMUSG00000051506	ENSMUSG00000051506	ENSMUSG00000051506	14	31788662	31867678	245	285		
11434	10	49787534	49792283	ENSMUSG00000041673	ENSMUSG00000041673	ENSMUSG00000041673	14	31820490	31844384	245	285		
11435	10	49324086	49483182	ENSMUSG00000063506	ENSMUSG00000063506	ENSMUSG00000063506	14	32045915	32199025	245	285		
11436	10	49184739	49317409	ENSMUSG00000021936	ENSMUSG00000021936	ENSMUSG00000021936	14	32209888	32276220	245	285		
11437	10	48045795	48059172	ENSMUSG00000021943	ENSMUSG00000021943	ENSMUSG00000021943	14	32752689	32765606	245	285		
11438	10	48033098	48036859	ENSMUSG00000072625	ENSMUSG00000072625	ENSMUSG00000072625	14	32770131	32776290	245	285		
11439	10	48001493	48010997	ENSMUSG00000041534	ENSMUSG00000041534	ENSMUSG00000041534	14	32783114	32793308	245	285		
11440	10	47975081	47993872	ENSMUSG00000044519	ENSMUSG00000044519	ENSMUSG00000044519	14	32796162	32807856	245	285		
11441	10	46503540	46508326	ENSMUSG00000048337	ENSMUSG00000048337	ENSMUSG00000048337	14	32973084	32981581	245	285		
11442	10	46414093	46420579	ENSMUSG00000071531	ENSMUSG00000071531	ENSMUSG00000071531	14	33023536	33024903	245	285		
11443	10	46380452	46391406	ENSMUSG00000041479	ENSMUSG00000041479	ENSMUSG00000041479	14	33049139	33059335	245	285		
11444	10	88708365	88712997	ENSMUSG00000023064	ENSMUSG00000023064	ENSMUSG00000023064	14	33199367	33203752	246	286		
11445	10	88685277	88707352	ENSMUSG00000041445	ENSMUSG00000041445	ENSMUSG00000041445	14	33204621	33232834	246	286		
11446	10	88418408	88484731	ENSMUSG00000021798	ENSMUSG00000021798	ENSMUSG00000021798	14	33355797	33417766	246	286		
11447	10	88404354	88416192	ENSMUSG00000021799	ENSMUSG00000021799	ENSMUSG00000021799	14	33421454	33429234	246	286		
11448	10	88184993	88271552	ENSMUSG00000041408	ENSMUSG00000041408	ENSMUSG00000041408	14	33503020	33575309	246	286		
11449	10	87352010	88116129	ENSMUSG00000041078	ENSMUSG00000041078	ENSMUSG00000041078	14	33649320	34409901	246	286		
11450	10	86078390	86268256	ENSMUSG00000058690	ENSMUSG00000058690	ENSMUSG00000058690	14	35786177	35880005	246	286		
11451	10	85994789	86009696	ENSMUSG00000021804	ENSMUSG00000021804	ENSMUSG00000021804	14	35948731	35960205	246	286		
11452	10	85981329	85991197	ENSMUSG00000041044	ENSMUSG00000041044	ENSMUSG00000041044	14	35966076	35976187	246	286		
11453	10	85970221	85975264	ENSMUSG00000043418	ENSMUSG00000043418	ENSMUSG00000043418	14	35979290	35984976	246	286		
11454	10	85944394	85967102	ENSMUSG00000021803	ENSMUSG00000021803	ENSMUSG00000021803	14	35989090	36009703	246	286		
11455	10	85889176	85902981	ENSMUSG00000041028	ENSMUSG00000041028	ENSMUSG00000041028	14	36032557	36047038	246	286		
11456	10	83625077	84735341	ENSMUSG00000041014	ENSMUSG00000041014	ENSMUSG00000041014	14	37282310	38384679	246	286		
11457	10	82287638	82396296	ENSMUSG00000037833	ENSMUSG00000037833	ENSMUSG00000037833	14	39724581	39801678	246	286		
11458	10	82203902	82272367	ENSMUSG00000037824	ENSMUSG00000037824	ENSMUSG00000037824	14	39815141	39875521	246	286		
11459	10	82157565	82181844	ENSMUSG00000021792	ENSMUSG00000021792	ENSMUSG00000021792	14	39902453	39922488	246	286		
11460	10	82094481	82117809	ENSMUSG00000021791	ENSMUSG00000021791	ENSMUSG00000021791	14	39957832	39977921	246	286		
11461	10	82085844	82102352	ENSMUSG00000021790	ENSMUSG00000021790	ENSMUSG00000021790	14	39981624	39996229	246	286		
11462	10	82021556	82039420	ENSMUSG00000037798	ENSMUSG00000037798	ENSMUSG00000037798	14	40014094	40033130	246	286		
11463	10	81687476	81698839	ENSMUSG00000021795	ENSMUSG00000021795	ENSMUSG00000021795	14	40080927	40093876	246			
11464	14	51804181	51813191	ENSMUSG00000071489	ENSMUSG00000071489	ENSMUSG00000071489	14	43773112	43781252	247	287		
11465	14	51850863	51865062	ENSMUSG00000037759	ENSMUSG00000037759	ENSMUSG00000037759	14	43910072	43925090	247	287		
11466	14	51967059	52088963	ENSMUSG00000021830	ENSMUSG00000021830	ENSMUSG00000021830	14	44056784	44142205	247	287		
11467	14	52178357	52232173	ENSMUSG00000021831	ENSMUSG00000021831	ENSMUSG00000021831	14	44206198	44240404	247	287		
11468	14	52393743	52487460	ENSMUSG00000037712	ENSMUSG00000037712	ENSMUSG00000037712	14	44380669	44451942	247	287		
11469	14	52583090	52689566	ENSMUSG00000037697	ENSMUSG00000037697	ENSMUSG00000037697	14	44517294	44580020	247	287		
11470	14	53486207	53493362	ENSMUSG00000021835	ENSMUSG00000021835	ENSMUSG00000021835	14	45305599	45312453	247	287		
11471	14	53933423	53956682	ENSMUSG00000037628	ENSMUSG00000037628	ENSMUSG00000037628	14	45682434	45693398	247	287		
11472	14	53963397	53977898	ENSMUSG00000015759	ENSMUSG00000015759	ENSMUSG00000015759	14	45697459	45710186	247	287		
11473	14	54014519	54020236	ENSMUSG00000062014	ENSMUSG00000062014	ENSMUSG00000062014	14	45733629	45739359	247	287		
11474	14	54046337	54075084	ENSMUSG00000055128	ENSMUSG00000055128	ENSMUSG00000055128	14	45754121	45776067	247	287		
11475	14	54104387	54325595	ENSMUSG00000021838	ENSMUSG00000021838	ENSMUSG00000021838	14	45804844	46024079	247	287		
11476	14	54378476	54439292	ENSMUSG00000037580	ENSMUSG00000037580	ENSMUSG00000037580	14	46075772	46111279	247	287		
11477	14	54476692	54563557	ENSMUSG00000037572	ENSMUSG00000037572	ENSMUSG00000037572	14	46166968	46198690	247	287		
11478	14	54563594	54585957	ENSMUSG00000048379	ENSMUSG00000048379	ENSMUSG00000048379	14	46199020	46213468	247	287		
11479	14	54673452	54681879	ENSMUSG00000050335	ENSMUSG00000050335	ENSMUSG00000050335	14	46295795	46308037	247	287		
11480	14	54684601	54728149	ENSMUSG00000037544	ENSMUSG00000037544	ENSMUSG00000037544	14	46309656	46340284	247	287		
11481	14	55148488	55221047	ENSMUSG00000021843	ENSMUSG00000021843	ENSMUSG00000021843	14	46585654	46658434	247	287		
11482	14	55654956	55837773	ENSMUSG00000021846	ENSMUSG00000021846	ENSMUSG00000021846	14	47042774	47180324	247	287		
11483	14	56116264	56184038	ENSMUSG00000036339	ENSMUSG00000036339	ENSMUSG00000036339	14	47368527	47437086	247	287		
11484	14	56337182	56346940	ENSMUSG00000021848	ENSMUSG00000021848	ENSMUSG00000021848	14	47580137	47589457	247	287		
11485	14	56805380	56826544	ENSMUSG00000036291	ENSMUSG00000036291	ENSMUSG00000036291	14	47988500	48009674	247	287		
11486	14	56927024	56947636	ENSMUSG00000036282	ENSMUSG00000036282	ENSMUSG00000036282	14	48094801	48112817	247	287		
11487	14	57006348	57030323	ENSMUSG00000021850	ENSMUSG00000021850	ENSMUSG00000021850	14	48148309	48167351	247	287		
11488	14	57100606	57130593	ENSMUSG00000021852	ENSMUSG00000021852	ENSMUSG00000021852	14	48220670	48447564	247	287		
11489	14	57540572	57688600	ENSMUSG00000036242	ENSMUSG00000036242	ENSMUSG00000036242	14	48604299	48696196	247	287		
11490	14	19681735	19682661	ENSMUSG00000048933	ENSMUSG00000048933	ENSMUSG00000048933	14	48816209	48823892	248	288		
11491	14	19473666	19474601	ENSMUSG00000050030	ENSMUSG00000050030	ENSMUSG00000050030	14	49061653	49062588	248	288		
11492	14	19458606	19459577	ENSMUSG00000049011	ENSMUSG00000049011	ENSMUSG00000049011	14	49069852	49070823	248	288		
11493	14	19414267	19415211	ENSMUSG00000050431	ENSMUSG00000050431	ENSMUSG00000050431	14	49108213	49109169	248	288		
11494	14	19285427	19286368	ENSMUSG00000046210	ENSMUSG00000046210	ENSMUSG00000046210	14	49267327	49268391	248	288		
11495	14	19761709	19762701	ENSMUSG00000050028	ENSMUSG00000050028	ENSMUSG00000050028	14	49564234	49565220	248	288		
11496	14	19849370	19871297	ENSMUSG00000071470	ENSMUSG00000071470	ENSMUSG00000071470	14	49711764	49715805	248	288		
11497	14	19881634	19895902	ENSMUSG00000036023	ENSMUSG00000036023	ENSMUSG00000036023	14	49729897	49743251	248	288		
11498	14	19905755	19951426	ENSMUSG00000006281	ENSMUSG00000006281	ENSMUSG00000006281	14	49746013	49792511	248	288		
11499	14	19966848	19968719	ENSMUSG00000072578	ENSMUSG00000072578	ENSMUSG00000072578	14	49813460	49818873	248	288		
11500	14	19985056	19993038	ENSMUSG00000006289	ENSMUSG00000006289	ENSMUSG00000006289	14	49828429	49846785	248	288		
11501	14	19993130	19995765	ENSMUSG00000035960	ENSMUSG00000035960	ENSMUSG00000035960	14	49846929	49849089	248	288		
11502	14	19995858	19999476	ENSMUSG00000035953	ENSMUSG00000035953	ENSMUSG00000035953	14	49848021	49852800	248	288		

11503	14	20048471	20049121	ENSMUSG00000021872	ENSMUSG00000021872	ENSMUSG00000021872	14	49929880	49932712	248	288
11504	14	20094451	20095068	ENSMUSG00000052382	ENSMUSG00000052382	ENSMUSG00000052382	14	49960410	49963820	248	288
11505	14	20120894	20128257	ENSMUSG00000059648	ENSMUSG00000059648	ENSMUSG00000059648	14	49971468	49972046	248	288
11506	14	20128279	20128722	ENSMUSG00000068407	ENSMUSG00000068407	ENSMUSG00000068407	14	49978734	49979171	248	288
11507	14	20178695	20179690	ENSMUSG00000035932	ENSMUSG00000035932	ENSMUSG00000035932	14	49992347	49993342	248	288
11508	14	20222212	20238597	ENSMUSG00000021876	ENSMUSG00000021876	ENSMUSG00000021876	14	50013068	50028097	248	288
11509	14	20319050	20320464	ENSMUSG00000021880	ENSMUSG00000021880	ENSMUSG00000021880	14	50051041	50052975	248	288
11510	14	20339356	20340876	ENSMUSG00000035896	ENSMUSG00000035896	ENSMUSG00000035896	14	50066953	50068718	248	288
11511	14	20527805	20534882	ENSMUSG00000004561	ENSMUSG00000004561	ENSMUSG00000004561	14	50806886	50814990	248	288
11512	14	20537289	20539870	ENSMUSG00000072572	ENSMUSG00000072572	ENSMUSG00000072572	14	50815610	50818696	248	288
11513	14	20554763	20563775	ENSMUSG00000004558	ENSMUSG00000004558	ENSMUSG00000004558	14	50827232	50835302	248	288
11514	14	20568581	20570813	ENSMUSG00000008813	ENSMUSG00000008813	ENSMUSG00000008813	14	50840376	50842650	248	288
11515	14	20570822	20572784	ENSMUSG00000068392	ENSMUSG00000068392	ENSMUSG00000068392	14	50844170	50844631	248	288
11516	14	20608367	20627876	ENSMUSG00000004562	ENSMUSG00000004562	ENSMUSG00000004562	14	50909354	50926981	248	288
11517	14	20628076	20636639	ENSMUSG00000049295	ENSMUSG00000049295	ENSMUSG00000049295	14	50928037	50931616	248	288
11518	14	20692936	20694024	ENSMUSG00000044286	ENSMUSG00000044286	ENSMUSG00000044286	14	50957125	50958060	248	288
11519	14	20889478	20922265	ENSMUSG00000035726	ENSMUSG00000035726	ENSMUSG00000035726	14	51082365	51119152	248	288
11520	14	20997023	21014964	ENSMUSG00000022159	ENSMUSG00000022159	ENSMUSG00000022159	14	51183722	51201288	248	288
11521	14	21036123	21049297	ENSMUSG00000022160	ENSMUSG00000022160	ENSMUSG00000022160	14	51216792	51227075	248	288
11522	14	21059074	21075177	ENSMUSG00000049532	ENSMUSG00000049532	ENSMUSG00000049532	14	51233123	51250713	248	288
11523	14	22103647	22127970	ENSMUSG00000022174	ENSMUSG00000022174	ENSMUSG00000022174	14	53193491	53211919	248	288
11524	14	22136986	22151097	ENSMUSG00000040997	ENSMUSG00000040997	ENSMUSG00000040997	14	53217257	53227171	248	288
11525	14	22305571	22310607	ENSMUSG00000000959	ENSMUSG00000000959	ENSMUSG00000000959	14	53315893	53324721	248	288
11526	14	22312287	22358814	ENSMUSG00000000958	ENSMUSG00000000958	ENSMUSG00000000958	14	53324496	53363665	248	288
11527	14	22368950	22374079	ENSMUSG00000010406	ENSMUSG00000010406	ENSMUSG00000010406	14	53381114	53383955	248	288
11528	14	22375676	22385088	ENSMUSG00000000957	ENSMUSG00000000957	ENSMUSG00000000957	14	53385824	53395466	248	288
11529	14	22410800	22417133	ENSMUSG00000022175	ENSMUSG00000022175	ENSMUSG00000022175	14	53418352	53424497	248	288
11530	14	22422272	22426727	ENSMUSG00000022176	ENSMUSG00000022176	ENSMUSG00000022176	14	53430305	53434639	248	288
11531	14	22459579	22468501	ENSMUSG00000023110	ENSMUSG00000023110	ENSMUSG00000023110	14	53461387	53471564	248	288
11532	14	22485285	22496180	ENSMUSG00000022177	ENSMUSG00000022177	ENSMUSG00000022177	14	53495988	53508541	248	288
11533	14	22510249	22521691	ENSMUSG00000022178	ENSMUSG00000022178	ENSMUSG00000022178	14	53523136	53531469	248	288
11534	14	22526264	22549169	ENSMUSG00000022179	ENSMUSG00000022179	ENSMUSG00000022179	14	53537868	53560113	248	288
11535	14	22564900	22573949	ENSMUSG00000022193	ENSMUSG00000022193	ENSMUSG00000022193	14	53568325	53572219	248	288
11536	14	22581216	22582379	ENSMUSG00000072423	ENSMUSG00000072423	ENSMUSG00000072423	14	53579673	53580242	248	288
11537	14	22586709	22594875	ENSMUSG00000059674	ENSMUSG00000059674	ENSMUSG00000059674	14	53585436	53595523	248	288
11538	14	22597616	22634663	ENSMUSG00000022185	ENSMUSG00000022185	ENSMUSG00000022185	14	53596372	53641136	248	288
11539	14	22634628	22639500	ENSMUSG00000040822	ENSMUSG00000040822	ENSMUSG00000040822	14	53640421	53644949	248	288
11540	14	22656387	22658665	ENSMUSG00000052435	ENSMUSG00000052435	ENSMUSG00000052435	14	53664568	53666379	248	288
11541	14	22664346	22722689	ENSMUSG00000022180	ENSMUSG00000022180	ENSMUSG00000022180	14	53676418	53736091	248	288
11542	14	22815109	22838496	ENSMUSG00000057156	ENSMUSG00000057156	ENSMUSG00000057156	14	53806939	53814695	248	288
11543	14	22845866	22850798	ENSMUSG00000022194	ENSMUSG00000022194	ENSMUSG00000022194	14	53837600	53842435	248	288
11544	14	22859337	22865202	ENSMUSG00000022198	ENSMUSG00000022198	ENSMUSG00000022198	14	53848359	53852324	248	288
11545	14	22885368	22891319	ENSMUSG00000022199	ENSMUSG00000022199	ENSMUSG00000022199	14	53861283	53866717	248	288
11546	14	22895454	22904682	ENSMUSG00000022203	ENSMUSG00000022203	ENSMUSG00000022203	14	53871307	53875405	248	288
11547	14	22911858	22915445	ENSMUSG00000040770	ENSMUSG00000040770	ENSMUSG00000040770	14	53887188	53890043	248	288
11548	14	22916085	22918820	ENSMUSG00000040759	ENSMUSG00000040759	ENSMUSG00000040759	14	53890679	53893486	248	288
11549	14	22921038	22946665	ENSMUSG00000040752	ENSMUSG00000040752	ENSMUSG00000040752	14	53896562	53919669	248	288
11550	14	22951789	22974690	ENSMUSG00000053093	ENSMUSG00000053093	ENSMUSG00000053093	14	53924897	53948705	248	288
11551	14	23008750	23017853	ENSMUSG00000022204	ENSMUSG00000022204	ENSMUSG00000022204	14	53970093	53978213	248	288
11552	14	23059904	23065839	ENSMUSG00000040721	ENSMUSG00000040721	ENSMUSG00000040721	14	54015726	54046119	248	288
11553	14	23095075	23098608	ENSMUSG00000045691	ENSMUSG00000045691	ENSMUSG00000045691	14	54048906	54053053	248	288
11554	14	23098617	23107119	ENSMUSG00000040701	ENSMUSG00000040701	ENSMUSG00000040701	14	54052926	54060199	248	288
11555	14	23591046	23608781	ENSMUSG00000022211	ENSMUSG00000022211	ENSMUSG00000022211	14	54445164	54462335	248	288
11556	14	23609998	23617135	ENSMUSG00000022212	ENSMUSG00000022212	ENSMUSG00000022212	14	54464694	54471497	248	288
11557	14	23619216	23654063	ENSMUSG00000040632	ENSMUSG00000040632	ENSMUSG00000040632	14	54473589	54479052	248	288
11558	14	23633323	23643179	ENSMUSG00000040618	ENSMUSG00000040618	ENSMUSG00000040618	14	54494337	54504088	248	288
11559	14	23653861	23664288	ENSMUSG00000022214	ENSMUSG00000022214	ENSMUSG00000022214	14	54514103	54524136	248	288
11560	14	23670608	23671898	ENSMUSG00000022215	ENSMUSG00000022215	ENSMUSG00000022215	14	54529745	54531023	248	288
11561	14	23675218	23678015	ENSMUSG00000022216	ENSMUSG00000022216	ENSMUSG00000022216	14	54532646	54535608	248	288
11562	14	23678009	23680634	ENSMUSG00000022217	ENSMUSG00000022217	ENSMUSG00000022217	14	54535592	54539371	248	288
11563	14	23686579	23699706	ENSMUSG00000047098	ENSMUSG00000047098	ENSMUSG00000047098	14	54545861	54557742	248	288
11564	14	23711074	23719302	ENSMUSG00000002324	ENSMUSG00000002324	ENSMUSG00000002324	14	54572241	54579461	248	288
11565	14	23719488	23727833	ENSMUSG00000002319	ENSMUSG00000002319	ENSMUSG00000002319	14	54579824	54590028	248	288
11566	14	23728203	23734722	ENSMUSG00000002320	ENSMUSG00000002320	ENSMUSG00000002320	14	54590037	54597877	248	288
11567	14	23744766	23747292	ENSMUSG00000007591	ENSMUSG00000007591	ENSMUSG00000007591	14	54604285	54606602	248	288
11568	14	23755912	23771412	ENSMUSG00000010376	ENSMUSG00000010376	ENSMUSG00000010376	14	54616600	54617930	248	288
11569	14	23771488	23778285	ENSMUSG00000002326	ENSMUSG00000002326	ENSMUSG00000002326	14	54626307	54633271	248	288
11570	14	23778843	23781706	ENSMUSG00000007589	ENSMUSG00000007589	ENSMUSG00000007589	14	54631266	54635857	248	288
11571	14	23788162	23802217	ENSMUSG00000022218	ENSMUSG00000022218	ENSMUSG00000022218	14	54654090	54667159	248	288
11572	14	23804616	23810665	ENSMUSG00000040472	ENSMUSG00000040472	ENSMUSG00000040472	14	54670461	54675776	248	288
11573	14	23829649	23838815	ENSMUSG00000002332	ENSMUSG00000002332	ENSMUSG00000002332	14	54693089	54699663	248	288
11574	14	23838908	23848170	ENSMUSG00000019297	ENSMUSG00000019297	ENSMUSG00000019297	14	54699784	54708137	248	288
11575	14	23844241	23850495	ENSMUSG00000022219	ENSMUSG00000022219	ENSMUSG00000022219	14	54708131	54712529	248	288
11576	14	23857395	23874117	ENSMUSG00000022220	ENSMUSG00000022220	ENSMUSG00000022220	14	54723163	54738090	248	288
11577	14	23875072	23879036	ENSMUSG00000022221	ENSMUSG00000022221	ENSMUSG00000022221	14	54739067	54742879	248	288
11578	14	23907094	23918645	ENSMUSG00000023411	ENSMUSG00000023411	ENSMUSG00000023411	14	54779079	54788014	248	288
11579	14	23937832	23948312	ENSMUSG00000075592	ENSMUSG00000075592	ENSMUSG00000075592	14	54817501	54818986	248	288
11580	14	23965589	23968571	ENSMUSG00000040380	ENSMUSG00000040380	ENSMUSG00000040380	14	54832991	54838327	248	288
11581	14	23968332	23980380	ENSMUSG00000047153	ENSMUSG00000047153	ENSMUSG00000047153	14	54839040	54850852	248	288
11582	14	23978814	23981593	ENSMUSG00000022223	ENSMUSG00000022223	ENSMUSG00000022223	14	54851592	54854295	248	288
11583	14	24044552	24047311	ENSMUSG00000022225	ENSMUSG00000022225	ENSMUSG00000022225	14	54895522	54898732	248	288
11584	14	24112564	24115306	ENSMUSG00000040314	ENSMUSG00000040314	ENSMUSG00000040314	14	55053952	55056645	248	288
11585	13	24152695	24183909	ENSMUSG00000022229	ENSMUSG00000022229	ENSMUSG00000022229	14	55319139	55342661	249	289
11586	13	24236301	24352056	ENSMUSG00000000365	ENSMUSG00000000365	ENSMUSG00000000365	14	55356808	55479142	249	289
11587	13	24355171	24395018	ENSMUSG00000064128	ENSMUSG00000064128	ENSMUSG00000064128	14	55480871	55525942	249	289
11588	13	19105821	19145581	ENSMUSG00000054509	ENSMUSG00000054509	ENSMUSG00000054509	14	55588322	55652034	250	290
11589	13	19174640	19255057	ENSMUSG00000021938	ENSMUSG00000021938	ENSMUSG00000021938	14	55676559	55732426	250	290
11590	13	19295622	19323936	ENSMUSG00000040123	ENSMUSG00000040123	ENSMUSG00000040123	14	55747431	55765807	250	290
11591	13	19430810	19558939	ENSMUSG00000021945	ENSMUSG00000021945	ENSMUSG00000021945	14	55841904	55914459	250	290
11592	13	19614120	19615427	ENSMUSG00000048582	ENSMUSG00000048582	ENSMUSG00000048582	14	55989770	55991023	250	290
11593	13	19659609	19665037	ENSMUSG00000046352	ENSMUSG00000046352	ENSMUSG00000046352	14	56052721	56058775	250	290
11594	13	19694103	19704366	ENSMUSG00000040055	ENSMUSG00000040055	ENSMUSG00000040055	14	56077413	56087687	250	290
11595	13	19875813	19998012	ENSMUSG00000021947	ENSMUSG00000021947	ENSMUSG00000021947	14	56229103	56352613	250	290
11596	13	20038585	20163501	ENSMUSG00000040040	ENSMUSG00000040040	ENSMUSG00000040040	14	56378201	56471568	250	290
11597	13	20175479	20195236	ENSMUSG00000050222	ENSMUSG00000050222	ENSMUSG00000050222	14	56479017	56497275	250	290
11598	13	20200870	20246088	ENSMUSG00000021951	ENSMUSG00000021951	ENSMUSG00000021951	14	56503708	56525679	250	290
11599	13	20251014	20374949	ENSMUSG00000021952	ENSMUSG00000021952	ENSMUSG00000021952	14	56535109	56619066	250	290
11600	13	20445171	20533686	ENSMUSG00000021959	ENSMUSG00000021959	ENSMUSG00000021959	14	56643772	56700233	250	290
11601	13	20625735	20648710	ENSMUSG00000021965	ENSMUSG00000021965	ENSMUSG00000021965	14	56760671	56780273	250	290
11602	13	20648372	20651203	ENSMUSG00000021967	ENSMUSG00000021967	ENSMUSG00000021967	14	56780349	56782855	250	290
11603	13	20848263	20931509	ENSMUSG00000021969	ENSMUSG00000021969	ENSMUSG00000021969	14	56791740	56844290	250	290
11604	13	20964839	21076353	ENSMUSG00000021973	ENSMUSG00000021973	ENSMUSG00000021973	14	56870388	56953372	250	290
11605	13	21143170	21176637	ENSMUSG00000021974	ENSMUSG00000021974	ENSMUSG00000021974	14	57027028	57063760	250	290
11606	13	49004083	49039460	ENSMUSG00000035469	ENSMUSG00000035469	ENSMUSG00000035469	14	58155349	58191374	251	291
11607	13	48916511	48964307	ENSMUSG00000071350	ENSMUSG00000071350	ENSMUSG00000071350	14	58356412	58385388	251	291
11608	13	48780787	48873660	ENSMUSG00000021981	ENSMUSG00000021981	ENSMUSG00000021981	14	58395091	58503013	251	291
11609	13	48720063	48765622	ENSMUSG00000021982	ENSMUSG00000021982	ENSMUSG00000021982	14	58513498	58552049	251	291
11610	13	25516735	25523198	ENSMUSG00000044461	ENSMUSG00000044461	ENSMUSG00000044461	14	58579418	58585770	252	292
11611	13	24844209	25492996	ENSMUSG00000021983	ENSMUSG00000021983	ENSMUSG00000021983	14	58601851	59040944	252	292
11612	13	24773769	24814544	ENSMUSG00000063895	ENSMUSG00000063895	ENSMUSG00000063895	14	59173580	59205519	252	292
11613	13	24718339	24760147	ENSMUSG00000021987	ENSMUSG00000021987	ENSMUSG00000021987	14	59219338	59256480	252	292
11614	13	24640674	24643857	ENSMUSG00000021986	ENSMUSG00000021986	ENSMUSG00000021986	14	59332456	59335107	252	292
11615	13	23632887	23779210	ENSMUSG00000021990	ENSMUSG00000021990	ENSMUSG00000021990	14	59660982	59716182	252	292
11616	13	23202328	23361558	ENSMUSG00000021993	ENSMUSG00000021993	ENSMUSG00000021993	14	59738798	59858339	252	292
11617	13	23051639	23141412	ENSMUSG00000060548	ENSMUSG00000060548	ENSMUSG00000060548	14	59924933	59978922	252	292
11618	13	22800965	22883684	ENSMUSG00000048279	ENSMUSG00000048279	ENSMUSG00000048279	14	60127403	60168362	252	292
11619	13	22653091	22797304	ENSMUSG00000035296	ENSMUSG00000035296	ENSMUSG00000035296	14	60175169	60212543	252	292
11620	13	49100436	49106009	ENSMUSG00000043157	ENSMUSG00000043157	ENSMUSG00000043157	14	60263863	60266046	253	293
11621	13	49132868	49163624	ENSMUSG00000021928	ENSMUSG00000021928	ENSMUSG00000021928	14	60294860	60314549	253	293
11622	13	49171463	49265058	ENSMUSG00000021929	ENSMUSG00000021929	ENSMUSG00000021929	14	60319296	60393901	253	293
11623	13	49384843	49408477	ENSMUSG00000021930	ENSMUSG00000021930	ENSMUSG00000021930	14	60488415	60510996	253	293
11624	13	49487391	49493059	ENSMUSG00000046168	ENSMUSG00000046168	ENSMUSG00000046168	14	60561623	60566943	253	293
11625	13	50295045	50315833	ENSMUSG00000048281	ENSMUSG00000048281	ENSMUSG00000048281	14	61230339	61247073	253	293
11626	13	50381815	50428867	ENSMUSG00000021932	ENSMUSG00000021932	ENSMUSG00000021932	14	61286215	61327102	253	293
11627	13	50476394	50535031	ENSMUSG00000021933	ENSMUSG00000021933	ENSMUSG00000021933	14	61346786	61410399	253	293
11628	13	50694556	50755904	ENSMUSG00000035184	ENSMUSG00000035184	ENSMUSG00000035184	14	61509963	61560765	253	293
11629	13	50837365	50925150	ENSMUSG00000035161	ENSMUSG00000035161	ENSMUSG00000035161	14	61646115	61698530	253	293
11630	13	51056615	51232220	ENSMUSG00000014547	ENSMUSG00000014547	ENSMUSG00000014547	14	61791788	61910613	253	293
11631	8	11877239	11879472	ENSMUSG00000075571	ENSMUSG00000075571	ENSMUSG00000075571	14	61989908	61991925	254	294
11632	8	11868873	11869517	ENSMUSG00000054763	ENSMUSG00000054763	ENSMUSG00000054763	14	62001101	62002710	254	294
11633	8	11737442	11763147	ENSMUSG00000021939	ENSMUSG00000021939	ENSMUSG00000021939	14	62076572	62097243	254	294
11634	8	11664657	11682263	ENSMUSG00000035121	ENSMUSG00000035121	ENSMUSG00000035121	14	62136553	62147651	254	294
11635	8	11599122	11654920	ENSMUSG00000021944	ENSMUSG00000021944	ENSMUSG00000021944	14	62153032	62199381	254	294
11636	8	11388919	11459522	ENSMUSG00000014453	ENSMUSG00000014453	ENSMUSG00000014453	14	62326947	62371297	254	294
11637	8	11316391	11361663	ENSMUSG00000035095	ENSMUSG00000035095	ENSMUSG00000035095	14	62390594	62419638	254	294
11638	8	11179394	11223080	ENSMUSG00000035078	ENSMUSG00000035078	ENSMUSG00000035078	14	62477587	62498078	254	294
11639	8	10792872	11096258	ENSMUSG00000035067	ENSMUSG00000035067	ENSMUSG00000035067	14	62560934	62774518	254	294
11640	8	10659885	10734796	ENSMUSG00000021958	ENSMUSG00000021958	ENSMUSG00000021958	14	62814474	62873914	254	294
11641	8	10618688	10625432	ENSMUSG00000063060	ENSMUSG00000063060	ENSMUSG00000063060	14	62897816	62904842	254	294
11642	8	10567557	10595513	ENSMUSG00000021961	ENSMUSG00000021961	ENSMUSG00000021961	14	62925182	62941900	254	294
11643	8	10501815	10550027	ENSMUSG00000046049	ENSMUSG00000046049	ENSMUSG00000046049	14	62976000	62986656	254	294
11644	8	9949188	10323811	ENSMUSG00000054733	ENSMUSG00000054733	ENSMUSG00000054733	14	63076731	63410013	254	294
11645	8	28980715	29176560	ENSMUSG00000060012	ENSMUSG00000060012	ENSMUSG00000060012	14	63669497	63760403	255	295
11646	8	28877304	28965334	ENSMUSG00000021972	ENSMUSG00000021972	ENSMUSG00000021972	14	63776328	63851259	255	295
11647	8	28681099	28803398	ENSMUSG00000021975	ENSMUSG00000021975	ENSMUSG00000021975	14	63904155	63993936	255	295
11648	8	28615050	28667116	ENSMUSG00000021978	ENSMUSG00000021978	ENSMUSG00000021978	14	64006173	64034585	255	295
11649	8	28407692	28487707	ENSMUSG00000007989	ENSMUSG00000007989	ENSMUSG00000007989	14	64156448	64216534	255	295
11650	8	28259021	28299902	ENSMUSG00000034522	ENSMUSG00000034522	ENSMUSG00000034522	14	64329503	64351008	255	295
11651	8	28230568	28256786	ENSMUSG00000045731	ENSMUSG00000045731	ENSMUSG00000045731	14	64354783	64379319	255	295
11652	8	28006328	28104592	ENSMUSG00000022031	ENSMUSG00000022031	ENSMUSG00000022031	14	64484563	64547185	255	295
11653	8	27935607	27997307	ENSMUSG00000061356	ENSMUSG00000061356	ENSMUSG00000061356	14	64562257	64602426	255	295
11654	8	27783672	27906117	ENSMUSG00000022032	ENSMUSG00000022032	ENSMUSG00000022032	14	64620537	64718936	255	295
11655	8	27723056	27751435	ENSMUSG00000022033	ENSMUSG00000022033	ENSMUSG00000022033	14	64759949	64771905	255	295
11656	8	27687990	27718661	ENSMUSG00000022034	ENSMUSG00000022034	ENSMUSG00000022034	14	64773149	64788012	255	295
11657	8	27646756	27686030	ENSMUSG00000022035	ENSMUSG00000022035	ENSMUSG00000022035	14	64791412	64819471	255	295
11658	8	27547496	27586456	ENSMUSG00000034463	ENSMUSG00000034463	ENSMUSG00000034463	14	64874847	64892426	255	295
11659	8	27510351	27528288	ENSMUSG00000022037	ENSMUSG00000022037	ENSMUSG00000022037	14	64925869	64935143	255	295
11660	8	27404543	27458355	ENSMUSG00000022040	ENSMUSG00000022040	ENSMUSG00000022040	14	65038487	65078610	255	295
11661	8	27374182	27392675	ENSMUSG00000022041	ENSMUSG00000022041	ENSMUSG00000022041	14	65095070	65107060	255	295
11662	8	27238971	27372824	ENSMUSG00000059456	ENSMUSG00000059456	ENSMUSG00000059456	14	65107372	65235162	255	295
11663	8	27198321	27224751	ENSMUSG00000022043	ENSMUSG00000022043	ENSMUSG00000022043	14	65251135	65265534	255	295
11664	8	27148757	27171850	ENSMUSG00000022044	ENSMUSG00000022044	ENSMUSG00000022044	14	65298485	65315790	255	295
11665	8	26661953	26778839	ENSMUSG00000045875	ENSMUSG00000045875	ENSMUSG00000045875	14	65589368	65687439	255	295
11666	8	26491327	26571611	ENSMUSG00000022048	ENSMUSG00000022048	ENSMUSG00000022048	14	65756972	65822710	255	295
11667	8	26418113	26427342	ENSMUSG00000046204	ENSMUSG00000046204	ENSMUSG00000046204	14	65865318	65872925	255	295
11668	8	25757501	25803239	ENSMUSG00000022053	ENSMUSG00000022053	ENSMUSG00000022053	14	66187402	66383877	255	295
11669	8	25372428	25421342	ENSMUSG00000048922	ENSMUSG00000048922	ENSMUSG00000048922	14	66630013	66669171	255	295
11670	8	25332697	25338087	ENSMUSG00000015812	ENSMUSG00000015812	ENSMUSG00000015812	14	66699676	66702621	255	295
11671	8	25098204	25326535	ENSMUSG00000044447	ENSMUSG00000044447	ENSMUSG00000044447	14	66709751	66834616	255	295
11672	8	24866240	24869946	ENSMUSG00000022055	ENSMUSG00000022055	ENSMUSG00000022055	14	67037214	67041075	255	295
11673	8	24827188	24832508	ENSMUSG00000022054	ENSMUSG00000022054	ENSMUSG00000022054	14	67072614	67078173	255	295
11674	8	24354542	24422951	ENSMUSG00000022056	ENSMUSG00000022056	ENSMUSG00000022056	14	67450660	67487005	255	295
11675	8	24297817	24319471	ENSMUSG00000022057	ENSMUSG00000022057	ENSMUSG00000022057	14	67518844	67535376	255	295
11676	8	24207565	24268659	ENSMUSG00000014725	ENSMUSG00000014725	ENSMUSG00000014725	14	67560209	67609172	255	295
11677	8	23755373	23768265	ENSMUSG00000014813	ENSMUSG00000014813	ENSMUSG00000014813	14	67982603	67993272	255	295
11678	8	23592151	23596395	ENSMUSG00000022061	ENSMUSG00000022061	ENSMUSG00000022061	14	68144024	68147991	255	295
11679	8	23342610	23371153	ENSMUSG00000022066	ENSMUSG00000022066	ENSMUSG00000022066	14	68290504	68320600	255	295
11680	8	23211501	23281809	ENSMUSG00000034205	ENSMUSG00000034205	ENSMUSG00000034205	14	68344248	68428800	255	295
11681	8	23201535	23209733	ENSMUSG00000034194	ENSMUSG00000034194	ENSMUSG00000034194	14	68432387	68442633	255	295
11682	8	23159705	23175437	ENSMUSG00000034190	ENSMUSG00000034190	ENSMUSG00000034190	14	68452081	68467621	255	295
11683	8	22900875	22931891	ENSMUSG00000022075	ENSMUSG00000022075	ENSMUSG00000022075	14	68520072	68540550	255	295
11684	8	22626729	22841362	ENSMUSG00000022085	ENSMUSG00000022085	ENSMUSG00000022085	14	68575500	68794959	255	295
11685	8	22601117	22606760	ENSMUSG00000033730	ENSMUSG00000033730	ENSMUSG00000033730	14	68812525	68815237	255	295
11686	8	22534186	22558266	ENSMUSG00000022089	ENSMUSG00000022089	ENSMUSG00000022089	14	68835225	68873286	255	295
11687	8	22518202	22533920	ENSMUSG00000033712	ENSMUSG00000033712	ENSMUSG00000033712	14	68873257	68888891	255	295
11688	8	22513067	22517605	ENSMUSG00000044551	ENSMUSG00000044551	ENSMUSG00000044551	14	68889485	68894560	255	295
11689	8	22493929	22511483	ENSMUSG00000022090	ENSMUSG00000022090	ENSMUSG00000022090	14	68899298	68912752	255	295
11690	8	22465196	22488951	ENSMUSG00000022091	ENSMUSG00000022091	ENSMUSG00000022091	14	68915548	68942717	255	295
11691	8	22354541	22454580	ENSMUSG00000022092	ENSMUSG00000022092	ENSMUSG00000022092	14	68953164	69002587	255	295
11692	8	22188772	22271021	ENSMUSG00000033644	ENSMUSG00000033644	ENSMUSG00000033644	14	69107560	69164174	255	295
11693	8	22158562	22164624	ENSMUSG00000000776	ENSMUSG00000000776	ENSMUSG00000000776	14	69173876	69178527	255	295
11694	8	22133181	22145549	ENSMUSG00000003469	ENSMUSG00000003469	ENSMUSG00000003469	14	69192597	69203904	255	295
11695	8	22078376	22125784	ENSMUSG00000022098	ENSMUSG00000022098	ENSMUSG00000022098	14	69209640	69255268	255	295
11696	8	22075113	22077928	ENSMUSG00000022097	ENSMUSG00000022097	ENSMUSG00000022097	14	69256020	69259150	255	295
11697	8	22060290	22070290	ENSMUSG00000033595	ENSMUSG00000033595	ENSMUSG00000033595	14	69265950	69273403	255	295

11698	8	22051478	22055393	ENSMUSG00000033589	ENSMUSG00000033589	ENSMUSG00000033589	14	69280434	69284013	255	295
11699	8	22027873	22045326	ENSMUSG00000022096	ENSMUSG00000022096	ENSMUSG00000022096	14	69289136	69308630	255	295
11700	8	22020329	22023403	ENSMUSG00000045211	ENSMUSG00000045211	ENSMUSG00000045211	14	69312927	69317651	255	295
11701	8	22002660	22017835	ENSMUSG00000022095	ENSMUSG00000022095	ENSMUSG00000022095	14	69318376	69334916	255	295
11702	8	21971322	21995823	ENSMUSG00000022099	ENSMUSG00000022099	ENSMUSG00000022099	14	69338783	69353482	255	295
11703	8	21955883	21962266	ENSMUSG00000022101	ENSMUSG00000022101	ENSMUSG00000022101	14	69371285	69377348	255	295
11704	8	21938706	21950139	ENSMUSG00000047911	ENSMUSG00000047911	ENSMUSG00000047911	14	69382382	69388164	255	295
11705	8	21833128	21918924	ENSMUSG00000022100	ENSMUSG00000022100	ENSMUSG00000022100	14	69401081	69442531	255	295
11706	8	21822336	21827151	ENSMUSG00000022102	ENSMUSG00000022102	ENSMUSG00000022102	14	69509461	69513574	255	295
11707	8	21593812	21702292	ENSMUSG00000022103	ENSMUSG00000022103	ENSMUSG00000022103	14	69625210	69714918	255	295
11708	13	48448051	48681729	ENSMUSG00000033487	ENSMUSG00000033487	ENSMUSG00000033487	14	71273033	71445085	256	296
11709	13	48178692	48181499	ENSMUSG00000033470	ENSMUSG00000033470	ENSMUSG00000033470	14	71764210	71784144	256	296
11710	13	47961104	48005317	ENSMUSG00000022106	ENSMUSG00000022106	ENSMUSG00000022106	14	71885527	71917843	256	296
11711	13	47775912	47954123	ENSMUSG00000022105	ENSMUSG00000022105	ENSMUSG00000022105	14	71929657	72059946	256	296
11712	13	47883170	47885294	ENSMUSG00000033446	ENSMUSG00000033446	ENSMUSG00000033446	14	71972050	71974497	256	296
11713	13	46305514	46368179	ENSMUSG00000034997	ENSMUSG00000034997	ENSMUSG00000034997	14	73374995	73441014	256	296
11714	13	46243392	46269368	ENSMUSG00000021996	ENSMUSG00000021996	ENSMUSG00000021996	14	73466500	73484598	256	296
11715	13	46025304	46215731	ENSMUSG00000068015	ENSMUSG00000068015	ENSMUSG00000068015	14	73488830	73682021	256	296
11716	13	45815100	45859385	ENSMUSG00000034959	ENSMUSG00000034959	ENSMUSG00000034959	14	73750182	73786572	256	296
11717	13	45684084	45722643	ENSMUSG00000021997	ENSMUSG00000021997	ENSMUSG00000021997	14	73818459	73863634	256	296
11718	13	45598060	45654386	ENSMUSG00000021998	ENSMUSG00000021998	ENSMUSG00000021998	14	73870580	73964963	256	296
11719	13	45525323	45577169	ENSMUSG00000021999	ENSMUSG00000021999	ENSMUSG00000021999	14	73976442	74017703	256	296
11720	13	45426601	45524882	ENSMUSG00000022000	ENSMUSG00000022000	ENSMUSG00000022000	14	74018528	74078581	256	296
11721	13	45174447	45186694	ENSMUSG00000034913	ENSMUSG00000034913	ENSMUSG00000034913	14	74317045	74326140	256	296
11722	13	45013433	45087875	ENSMUSG00000022002	ENSMUSG00000022002	ENSMUSG00000022002	14	74376414	74401292	256	296
11723	13	44937072	45008766	ENSMUSG00000034893	ENSMUSG00000034893	ENSMUSG00000034893	14	74436519	74488579	256	296
11724	13	44936983	44937453	ENSMUSG00000053632	ENSMUSG00000053632	ENSMUSG00000053632	14	74488292	74488891	256	296
11725	13	44865456	44890509	ENSMUSG00000022003	ENSMUSG00000022003	ENSMUSG00000022003	14	74495231	74521160	256	296
11726	13	44592650	44755696	ENSMUSG00000067995	ENSMUSG00000067995	ENSMUSG00000067995	14	74631517	74745016	256	296
11727	13	44664988	44666852	ENSMUSG00000046523	ENSMUSG00000046523	ENSMUSG00000046523	14	74689158	74699372	256	296
11728	13	44461739	44505742	ENSMUSG00000022008	ENSMUSG00000022008	ENSMUSG00000022008	14	74820387	74844970	256	296
11729	13	44411384	44461595	ENSMUSG00000022009	ENSMUSG00000022009	ENSMUSG00000022009	14	74845046	74871534	256	296
11730	13	43905663	44048210	ENSMUSG00000022010	ENSMUSG00000022010	ENSMUSG00000022010	14	75149976	75241922	256	296
11731	13	43846010	43869846	ENSMUSG00000052584	ENSMUSG00000052584	ENSMUSG00000052584	14	75284135	75290669	256	296
11732	13	43351969	43364367	ENSMUSG00000044350	ENSMUSG00000044350	ENSMUSG00000044350	14	75758356	75770772	256	296
11733	13	43328576	43341512	ENSMUSG00000034795	ENSMUSG00000034795	ENSMUSG00000034795	14	75770927	75846359	256	296
11734	13	42685704	43259044	ENSMUSG00000022012	ENSMUSG00000022012	ENSMUSG00000022012	14	76114801	76455915	256	296
11735	13	42495362	42581304	ENSMUSG00000022013	ENSMUSG00000022013	ENSMUSG00000022013	14	76560372	76609072	256	296
11736	13	42360123	42464377	ENSMUSG00000022014	ENSMUSG00000022014	ENSMUSG00000022014	14	76638641	76728792	256	296
11737	13	42253751	42263683	ENSMUSG00000045655	ENSMUSG00000045655	ENSMUSG00000045655	14	76815179	76823162	256	296
11738	13	42046440	42079054	ENSMUSG00000022015	ENSMUSG00000022015	ENSMUSG00000022015	14	77011609	77042189	256	296
11739	13	41520889	41701888	ENSMUSG00000034731	ENSMUSG00000034731	ENSMUSG00000034731	14	77304260	77459876	256	296
11740	13	41038962	41433221	ENSMUSG00000058997	ENSMUSG00000058997	ENSMUSG00000058997	14	77583386	77799424	256	296
11741	13	40929695	40943018	ENSMUSG00000022018	ENSMUSG00000022018	ENSMUSG00000022018	14	78024392	78035652	256	296
11742	13	40783416	40849166	ENSMUSG00000022020	ENSMUSG00000022020	ENSMUSG00000022020	14	78068662	78124823	256	296
11743	13	40688505	40735684	ENSMUSG00000022022	ENSMUSG00000022022	ENSMUSG00000022022	14	78131927	78157742	256	296
11744	13	40661969	40666702	ENSMUSG00000043881	ENSMUSG00000043881	ENSMUSG00000043881	14	78160666	78165194	256	296
11745	13	40599705	40604882	ENSMUSG00000075502	ENSMUSG00000075502	ENSMUSG00000075502	14	78185990	78188971	256	296
11746	13	40533255	40556142	ENSMUSG00000022023	ENSMUSG00000022023	ENSMUSG00000022023	14	78195902	78215269	256	296
11747	13	40404168	40491450	ENSMUSG00000036461	ENSMUSG00000036461	ENSMUSG00000036461	14	78215349	78316631	256	296
11748	13	52125002	52160434	ENSMUSG00000022024	ENSMUSG00000022024	ENSMUSG00000022024	14	78321846	78363910	257	297
11749	13	52175400	52211948	ENSMUSG00000022025	ENSMUSG00000022025	ENSMUSG00000022025	14	78371848	78396325	257	297
11750	13	52316110	52320775	ENSMUSG00000036422	ENSMUSG00000036422	ENSMUSG00000036422	14	78500940	78505455	257	297
11751	13	52500960	52524187	ENSMUSG00000022026	ENSMUSG00000022026	ENSMUSG00000022026	14	78734457	78756085	257	297
11752	13	57104656	57201446	ENSMUSG00000035566	ENSMUSG00000035566	ENSMUSG00000035566	14	83253590	83342216	257	297
11753	13	59246299	59635901	ENSMUSG00000022021	ENSMUSG00000022021	ENSMUSG00000022021	14	85381007	85972925	257	297
11754	13	59868592	60046002	ENSMUSG00000022019	ENSMUSG00000022019	ENSMUSG00000022019	14	86248429	86377251	257	297
11755	13	60881819	60887397	ENSMUSG00000050505	ENSMUSG00000050505	ENSMUSG00000050505	14	87313336	87318675	257	297
11756	13	65776788	66700573	ENSMUSG00000055421	ENSMUSG00000055421	ENSMUSG00000055421	14	92769269	92770711	257	297
11757	13	69172726	69580592	ENSMUSG00000022076	ENSMUSG00000022076	ENSMUSG00000022076	14	94988399	95402168	257	297
11758	13	70910099	71339331	ENSMUSG00000055639	ENSMUSG00000055639	ENSMUSG00000055639	14	96710865	97052443	257	297
11759	13	72199894	72228337	ENSMUSG00000022070	ENSMUSG00000022070	ENSMUSG00000022070	14	97929511	97957241	257	297
11760	13	72227543	72254007	ENSMUSG00000033166	ENSMUSG00000033166	ENSMUSG00000033166	14	97960519	97982807	257	297
11761	13	72254198	72488592	ENSMUSG00000022064	ENSMUSG00000022064	ENSMUSG00000022064	14	97984184	98137627	257	297
11762	13	72530931	72549677	ENSMUSG00000005148	ENSMUSG00000005148	ENSMUSG00000005148	14	98181825	98196556	257	297
11763	13	73158150	73606043	ENSMUSG00000072294	ENSMUSG00000072294	ENSMUSG00000072294	14	98758267	98992997	257	297
11764	13	74757254	74953904	ENSMUSG00000033083	ENSMUSG00000033083	ENSMUSG00000033083	14	100327414	100392912	257	297
11765	13	74998575	75010062	ENSMUSG00000075486	ENSMUSG00000075486	ENSMUSG00000075486	14	100519523	100526201	257	297
11766	13	75092571	75343063	ENSMUSG00000033060	ENSMUSG00000033060	ENSMUSG00000033060	14	100727254	100821293	257	297
11767	13	76420633	76430778	ENSMUSG00000022126	ENSMUSG00000022126	ENSMUSG00000022126	14	101933477	101942596	257	297
11768	13	76462796	76474653	ENSMUSG00000022125	ENSMUSG00000022125	ENSMUSG00000022125	14	101956716	101964115	257	297
11769	13	76477388	76499295	ENSMUSG00000022124	ENSMUSG00000022124	ENSMUSG00000022124	14	101967535	101986017	257	297
11770	13	76516793	76799178	ENSMUSG00000033004	ENSMUSG00000033004	ENSMUSG00000033004	14	101999559	102232127	257	297
11771	13	77007860	77116642	ENSMUSG00000022123	ENSMUSG00000022123	ENSMUSG00000022123	14	102399020	102502446	257	297
11772	13	77170050	77235353	ENSMUSG00000055717	ENSMUSG00000055717	ENSMUSG00000055717	14	102535922	102590481	257	297
11773	13	77367617	77391904	ENSMUSG00000022122	ENSMUSG00000022122	ENSMUSG00000022122	14	102700304	102729852	257	297
11774	13	78071228	78075696	ENSMUSG00000048349	ENSMUSG00000048349	ENSMUSG00000048349	14	103349199	103353678	257	297
11775	13	78086427	78131315	ENSMUSG00000022120	ENSMUSG00000022120	ENSMUSG00000022120	14	103364446	103408324	257	297
11776	13	78792090	78878351	ENSMUSG00000022119	ENSMUSG00000022119	ENSMUSG00000022119	14	104001806	104062943	257	297
11777	13	78953288	79026222	ENSMUSG00000053253	ENSMUSG00000053253	ENSMUSG00000053253	14	104144433	104194975	257	297
11778	13	79808112	79813918	ENSMUSG00000022114	ENSMUSG00000022114	ENSMUSG00000022114	14	104778114	104782418	257	297
11779	13	83349341	83354529	ENSMUSG00000075478	ENSMUSG00000075478	ENSMUSG00000075478	14	107794000	107796090	257	297
11780	13	85266522	85268644	ENSMUSG00000045871	ENSMUSG00000045871	ENSMUSG00000045871	14	109630422	109636908	257	297
11781	13	87122871	87129872	ENSMUSG00000033214	ENSMUSG00000033214	ENSMUSG00000033214	14	110556952	110564972	257	297
11782	13	90848930	92317491	ENSMUSG00000022112	ENSMUSG00000022112	ENSMUSG00000022112	14	113974463	115405897	257	297
11783	13	92676866	93857656	ENSMUSG00000058571	ENSMUSG00000058571	ENSMUSG00000058571	14	115808893	116857881	257	297
11784	13	93887559	93929924	ENSMUSG00000022129	ENSMUSG00000022129	ENSMUSG00000022129	14	116895153	116934531	257	297
11785	13	94024277	94046484	ENSMUSG00000022130	ENSMUSG00000022130	ENSMUSG00000022130	14	116994816	117015077	257	297
11786	13	94052133	94079937	ENSMUSG00000022131	ENSMUSG00000022131	ENSMUSG00000022131	14	117019505	117047938	257	297
11787	13	94160749	94162511	ENSMUSG00000061517	ENSMUSG00000061517	ENSMUSG00000061517	14	117118272	117119794	257	297
11788	13	94470090	94751684	ENSMUSG00000032849	ENSMUSG00000032849	ENSMUSG00000032849	14	117366402	117589897	257	297
11789	13	95251835	95503638	ENSMUSG00000042104	ENSMUSG00000042104	ENSMUSG00000042104	14	118130122	118234546	257	297
11790	13	95541002	96283672	ENSMUSG00000053465	ENSMUSG00000053465	ENSMUSG00000053465	14	118273596	119004776	257	297
11791	13	96435974	96444605	ENSMUSG00000044819	ENSMUSG00000044819	ENSMUSG00000044819	14	119154767	119177617	257	297
11792	13	96672609	96842265	ENSMUSG00000022139	ENSMUSG00000022139	ENSMUSG00000022139	14	119410843	119566877	257	297
11793	13	96884475	96918243	ENSMUSG00000051615	ENSMUSG00000051615	ENSMUSG00000051615	14	119613624	119642370	257	297
11794	13	97403930	97474550	ENSMUSG00000030662	ENSMUSG00000030662	ENSMUSG00000030662	14	120046423	120083185	257	297
11795	13	97592817	97900017	ENSMUSG00000025555	ENSMUSG00000025555	ENSMUSG00000025555	14	120342434	120418923	257	297
11796	13	97900456	97972252	ENSMUSG00000063410	ENSMUSG00000063410	ENSMUSG00000063410	14	120422508	120472835	257	297
11797	13	98134056	98202908	ENSMUSG00000025557	ENSMUSG00000025557	ENSMUSG00000025557	14	120595762	120626569	257	297
11798	13	98243745	98536661	ENSMUSG00000025558	ENSMUSG00000025558	ENSMUSG00000025558	14	120681140	120873642	257	297
11799	13	98651109	98836679	ENSMUSG00000041765	ENSMUSG00000041765	ENSMUSG00000041765	14	121013838	121156214	257	297
11800	13	98704987	98711999	ENSMUSG00000050350	ENSMUSG00000050350	ENSMUSG00000050350	14	121046612	121050954	257	297
11801	13	98744794	98757708	ENSMUSG00000051212	ENSMUSG00000051212	ENSMUSG00000051212	14	121088870	121100285	257	297
11802	13	98951729	99013644	ENSMUSG00000025544	ENSMUSG00000025544	ENSMUSG00000025544	14	121242262	121294783	257	297
11803	13	99056924	99342825	ENSMUSG00000025545	ENSMUSG00000025545	ENSMUSG00000025545	14	121316884	121537412	257	297
11804	13	99415452	99422179	ENSMUSG00000041703	ENSMUSG00000041703	ENSMUSG00000041703	14	121594340	121600857	257	297
11805	13	99432294	99437019	ENSMUSG00000061524	ENSMUSG00000061524	ENSMUSG00000061524	14	121610615	121615032	257	297
11806	13	99539338	99980687	ENSMUSG00000041650	ENSMUSG00000041650	ENSMUSG00000041650	14	121669523	122025010	257	297
11807	13	99980520	100039047	ENSMUSG00000041625	ENSMUSG00000041625	ENSMUSG00000041625	14	122026040	122048332	257	297
11808	13	100054189	100125171	ENSMUSG00000041594	ENSMUSG00000041594	ENSMUSG00000041594	14	122054155	122118441	257	297
11809	13	100504129	100866681	ENSMUSG00000000197	ENSMUSG00000000197	ENSMUSG00000000197	14	122413351	122735130	257	297
11810	13	100902857	101166795	ENSMUSG00000032925	ENSMUSG00000032925	ENSMUSG00000032925	14	122795353	123109260	257	297
11811	13	101172841	101852156	ENSMUSG00000025551	ENSMUSG00000025551	ENSMUSG00000025551	14	123115526	123859945	257	297
11812	5	42835740	42847751	ENSMUSG00000064373	ENSMUSG00000064373	ENSMUSG00000064373	15	3224674	3229710	258	298
11813	5	42792705	42838216	ENSMUSG00000071814	ENSMUSG00000071814	ENSMUSG00000071814	15	3248111	3251191	258	298
11814	5	42459783	42757736	ENSMUSG00000055737	ENSMUSG00000055737	ENSMUSG00000055737	15	3267774	3533231	258	298
11815	5	41961113	41977602	ENSMUSG00000022184	ENSMUSG00000022184	ENSMUSG00000022184	15	3915461	3929573	258	298
11816	5	41940227	41957494	ENSMUSG00000041935	ENSMUSG00000041935	ENSMUSG00000041935	15	3932035	3945752	258	298
11817	5	41765924	41906360	ENSMUSG00000022186	ENSMUSG00000022186	ENSMUSG00000022186	15	3976428	4103962	258	298
11818	5	41342816	41546487	ENSMUSG00000049148	ENSMUSG00000049148	ENSMUSG00000049148	15	4325645	4525577	258	298
11819	5	41178093	41297297	ENSMUSG00000022181	ENSMUSG00000022181	ENSMUSG00000022181	15	4681660	4748442	258	298
11820	5	41033881	41107162	ENSMUSG00000022155	ENSMUSG00000022155	ENSMUSG00000022155	15	4847658	4909379	258	298
11821	5	40877043	40891211	ENSMUSG00000041849	ENSMUSG00000041849	ENSMUSG00000041849	15	5044667	5055532	258	298
11822	5	40795239	40834046	ENSMUSG00000050697	ENSMUSG00000050697	ENSMUSG00000050697	15	5091090	5129114	258	298
11823	5	40750335	40791799	ENSMUSG00000022151	ENSMUSG00000022151	ENSMUSG00000022151	15	5132775	5165551	258	298
11824	5	40715789	40729592	ENSMUSG00000039942	ENSMUSG00000039942	ENSMUSG00000039942	15	5180615	5190913	258	298
11825	5	39408503	39460703	ENSMUSG00000022150	ENSMUSG00000022150	ENSMUSG00000022150	15	6333980	6387920	258	298
11826	5	39320061	39400412	ENSMUSG00000022149	ENSMUSG00000022149	ENSMUSG00000022149	15	6392548	6445691	258	298
11827	5	39143302	39255432	ENSMUSG00000022148	ENSMUSG00000022148	ENSMUSG00000022148	15	6527086	6610536	258	298
11828	5	38973779	39110260	ENSMUSG00000050310	ENSMUSG00000050310	ENSMUSG00000050310	15	6682579	6747602	258	298
11829	5	38881893	38971500	ENSMUSG00000022146	ENSMUSG00000022146	ENSMUSG00000022146	15	6760805	6821498	258	298
11830	5	38510823	38631253	ENSMUSG00000054263	ENSMUSG00000054263	ENSMUSG00000054263	15	7101575	7138433	258	298
11831	5	38294418	38500405	ENSMUSG00000042961	ENSMUSG00000042961	ENSMUSG00000042961	15	7153866	7345335	258	298
11832	5	37848536	37875539	ENSMUSG00000022144	ENSMUSG00000022144	ENSMUSG00000022144	15	7758226	7784790	258	298
11833	5	37415169	37788529	ENSMUSG00000039828	ENSMUSG00000039828	ENSMUSG00000039828	15	7820381	8046358	258	298
11834	5	37323996	37406954	ENSMUSG00000022142	ENSMUSG00000022142	ENSMUSG00000022142	15	8056565	8106843	258	298
11835	5	37142087	37237668	ENSMUSG00000039801	ENSMUSG00000039801	ENSMUSG00000039801	15	8168081	8218372	258	298
11836	5	36912618	37102272	ENSMUSG00000022141	ENSMUSG00000022141	ENSMUSG00000022141	15	8238440	8316802	258	298
11837	5	36642446	36724191	ENSMUSG00000005360	ENSMUSG00000005360	ENSMUSG00000005360	15	8581335	8657945	258	298
11838	5	36284862	36337761	ENSMUSG00000048424	ENSMUSG00000048424	ENSMUSG00000048424	15	8915641	8991889	258	298
11839	5	36229574	36278001	ENSMUSG00000022253	ENSMUSG00000022253	ENSMUSG00000022253	15	8998142	9037020	258	298
11840	5	36188627	36219870	ENSMUSG00000054115	ENSMUSG00000054115	ENSMUSG00000054115	15	9040536	9066587	258	298
11841	5	36139171	36187772	ENSMUSG00000039704	ENSMUSG00000039704	ENSMUSG00000039704	15	9067547	9124726	258	298
11842	5	35940228	35974640	ENSMUSG00000039676	ENSMUSG00000039676	ENSMUSG00000039676	15	9380753	9410760	258	298
11843	5	35892748	35912678	ENSMUSG00000003882	ENSMUSG00000003882	ENSMUSG00000003882	15	9450884	9474583	258	298
11844	5	35653746	35850469	ENSMUSG00000072663	ENSMUSG00000072663	ENSMUSG00000072663	15	9649037	9693502	258	298
11845	5	35091564	35266334	ENSMUSG00000005268	ENSMUSG00000005268	ENSMUSG00000005268	15	10121963	10274098	258	298
11846	5	35033963	35083811	ENSMUSG00000005269	ENSMUSG00000005269	ENSMUSG00000005269	15	10303304	10354463	258	298
11847	5	34965455	34994825	ENSMUSG00000044224	ENSMUSG00000044224	ENSMUSG00000044224	15	10406036	10415298	258	298
11848	5	34951238	34961857	ENSMUSG00000022247	ENSMUSG00000022247	ENSMUSG00000022247	15	10420900	10430645	258	298
11849	5	34876534	34916175	ENSMUSG00000022249	ENSMUSG00000022249	ENSMUSG00000022249	15	10448672	10503364	258	298
11850	5	34692274	34868473	ENSMUSG00000022246	ENSMUSG00000022246	ENSMUSG00000022246	15	10515422	10577991	258	298
11851	5	34022040	34043970	ENSMUSG00000022244	ENSMUSG00000022244	ENSMUSG00000022244	15	10926481	10941349	258	298
11852	5	33980478	34020592	ENSMUSG00000022243	ENSMUSG00000022243	ENSMUSG00000022243	15	10945446	10973958	258	298
11853	5	33972246	33974099	ENSMUSG00000060735	ENSMUSG00000060735	ENSMUSG00000060735	15	10980591	10982009	258	298
11854	5	33563043	33928054	ENSMUSG00000047497	ENSMUSG00000047497	ENSMUSG00000047497	15	11009756	11290402	258	298
11855	5	33476639	33505401	ENSMUSG00000022241	ENSMUSG00000022241	ENSMUSG00000022241	15	11328388	11344383	258	298
11856	5	32747297	32822302	ENSMUSG00000022206	ENSMUSG00000022206	ENSMUSG00000022206	15	11784621	11850399	258	298
11857	5	32390214	32480601	ENSMUSG00000022201	ENSMUSG00000022201	ENSMUSG00000022201	15	12062578	12129188	258	298
11858	5	32160567	32210198	ENSMUSG00000022200	ENSMUSG00000022200	ENSMUSG00000022200	15	12266360	12296316	258	298
11859	5	31675274	32146795	ENSMUSG00000022197	ENSMUSG00000022197	ENSMUSG00000022197	15	12306804	12403026	258	298
11860	5	31568156	31590043	ENSMUSG00000022195	ENSMUSG00000022195	ENSMUSG00000022195	15	12753140	12769366	258	298
11861	5	31436926	31564970	ENSMUSG00000022191	ENSMUSG00000022191	ENSMUSG00000022191	15	12769602	12879970	258	298
11862	5	31229553	31360994	ENSMUSG00000039385	ENSMUSG00000039385	ENSMUSG00000039385	15	12978925	13036602	258	298
11863	5	24522966	24680794	ENSMUSG00000022321	ENSMUSG00000022321	ENSMUSG00000022321	15	18763672	18958960	258	298
11864	5	21786732	22541242	ENSMUSG00000040452	ENSMUSG00000040452	ENSMUSG00000040452	15	21056177	21534258	258	298
11865	5	17270750	17329935	ENSMUSG00000045763	ENSMUSG00000045763	ENSMUSG00000045763	15	25308099	25358462	258	298
11866	5	16718413	16989372	ENSMUSG00000022272	ENSMUSG00000022272	ENSMUSG00000022272	15	25567651	25756950	258	298
11867	5	16526148	16670118	ENSMUSG00000022270	ENSMUSG00000022270	ENSMUSG00000022270	15	25787989	25918412	258	298
11868	5	16504628	16518901	ENSMUSG00000052253	ENSMUSG00000052253	ENSMUSG00000052253	15	25929091	25943207	258	298
11869	5	16230823	16232684	ENSMUSG00000022269	ENSMUSG00000022269	ENSMUSG00000022269	15	26253773	26257096	258	298
11870	5	16120580	16144187	ENSMUSG00000056867	ENSMUSG00000056867	ENSMUSG00000056867	15	26332476	26354144	258	298
11871	5	15669091	15990295	ENSMUSG00000043556	ENSMUSG00000043556	ENSMUSG00000043556	15	26487809	26734328	258	298
11872	5	14757913	14924887	ENSMUSG00000022265	ENSMUSG00000022265	ENSMUSG00000022265	15	27411402	27539630	258	298
11873	5	14717857	14746507	ENSMUSG00000046034	ENSMUSG00000046034	ENSMUSG00000046034	15	27550642	27575334	258	298
11874	5	14634932	14664205	ENSMUSG00000056069	ENSMUSG00000056069	ENSMUSG00000056069	15	27599794	27626240	258	298
11875	5	14196829	14562448	ENSMUSG00000022263	ENSMUSG00000022263	ENSMUSG00000022263	15	27677210	27863884	258	298
11876	5	13743437	13997589	ENSMUSG00000022262	ENSMUSG00000022262	ENSMUSG00000022262	15	28148355	28415259	258	298
11877	5	11024952	11957110	ENSMUSG00000022240	ENSMUSG00000022240	ENSMUSG00000022240	15	30117182	30972978	258	298
11878	5	10617580	10703225	ENSMUSG00000022237	ENSMUSG00000022237	ENSMUSG00000022237	15	31241253	31312154	258	298
11879	5	10494988	10518140	ENSMUSG00000022236	ENSMUSG00000022236	ENSMUSG00000022236	15	31385802	31398236	258	298
11880	5	10406828	10488491	ENSMUSG00000039100	ENSMUSG00000039100	ENSMUSG00000039100	15	31403778	31454412	258	298
11881	5	10332389	10360959	ENSMUSG00000022235	ENSMUSG00000022235	ENSMUSG00000022235	15	31513494	31534712	258	298
11882	5	10303282	10319501	ENSMUSG00000022234	ENSMUSG00000022234	ENSMUSG00000022234	15	31535404	31546350	258	298
11883	5	10279455	10292469	ENSMUSG00000039065	ENSMUSG00000039065	ENSMUSG00000039065	15	31546705	31562124	258	298
11884	5	9682115	9683467	ENSMUSG00000045267	ENSMUSG00000045267	ENSMUSG00000045267	15	32121876	32122883	258	298
11885	5	9088138	9599158	ENSMUSG00000022231	ENSMUSG00000022231	ENSMUSG00000022231	15	32189692	32634004	258	298
11886	8	97575058	97693213	ENSMUSG00000022261	ENSMUSG00000022261	ENSMUSG00000022261	15	32865312	32979310	259	299
11887	8	97726675	98224894	ENSMUSG00000039007	ENSMUSG00000039007	ENSMUSG00000039007	15	33027740	33539086	259	299
11888	8	98357995	98359248	ENSMUSG00000038984	ENSMUSG00000038984	ENSMUSG00000038984	15	33631166	33632413	259	299
11889	8	98725583	98807711	ENSMUSG00000022255	ENSMUSG00000022255	ENSMUSG00000022255	15	34027370	34085968	259	299
11890	8	98950487	99117116	ENSMUSG00000022324	ENSMUSG00000022324	ENSMUSG00000022324	15	34251269	34380376	259	299
11891	8	99145926	99175014	ENSMUSG00000044726	ENSMUSG00000044726	ENSMUSG00000044726	15	34397901	34418481	259	299
11892	8	99183743	99198594	ENSMUSG00000022323	ENSMUSG00000022323	ENSMUSG00000022323	15	34428610	34439769	259	299

11893	8	99199244	99239813	ENSMUSG00000022325	ENSMUSG00000022325	ENSMUSG00000022325	15	34439921	34475237	259	299		
11894	8	99273563	99375797	ENSMUSG00000038879	ENSMUSG00000038879	ENSMUSG00000038879	15	34517388	34623295	259	299		
11895	8	99508426	99514252	ENSMUSG00000050963	ENSMUSG00000050963	ENSMUSG00000050963	15	34781944	34786929	259	299		
11896	8	99536041	99860304	ENSMUSG00000022329	ENSMUSG00000022329	ENSMUSG00000022329	15	34821251	35060236	259	299		
11897	8	100025846	100033508	ENSMUSG00000022330	ENSMUSG00000022330	ENSMUSG00000022330	15	35240687	35247893	259	299		
11898	8	100094670	100958983	ENSMUSG00000037646	ENSMUSG00000037646	ENSMUSG00000037646	15	35316550	35874683	259	299		
11899	8	101215209	101223489	ENSMUSG00000048230	ENSMUSG00000048230	ENSMUSG00000048230	15	36095792	36109297	259	299		
11900	8	101239439	101323306	ENSMUSG00000037617	ENSMUSG00000037617	ENSMUSG00000037617	15	36123957	36180199	259	299		
11901	8	101338464	101391503	ENSMUSG00000022280	ENSMUSG00000022280	ENSMUSG00000022280	15	36184523	36210618	259	299		
11902	8	101591163	101641146	ENSMUSG00000048307	ENSMUSG00000048307	ENSMUSG00000048307	15	36422257	36430727	259	299		
11903	8	101654299	101731069	ENSMUSG00000013611	ENSMUSG00000013611	ENSMUSG00000013611	15	36448651	36500162	259	299		
11904	8	102000090	102034745	ENSMUSG00000022285	ENSMUSG00000022285	ENSMUSG00000022285	15	36715359	36738833	259	299		
11905	8	102278464	102287020	ENSMUSG00000062397	ENSMUSG00000062397	ENSMUSG00000062397	15	36941614	36951991	259	299		
11906	8	102574162	102750995	ENSMUSG00000022286	ENSMUSG00000022286	ENSMUSG00000022286	15	37177836	37308160	259	299		
11907	8	102767953	102872355	ENSMUSG00000051359	ENSMUSG00000051359	ENSMUSG00000051359	15	37310764	37736996	259	299		
11908	8	103285908	103320522	ENSMUSG00000022292	ENSMUSG00000022292	ENSMUSG00000022292	15	37868541	37905644	259	299		
11909	8	103335706	103493638	ENSMUSG00000037487	ENSMUSG00000037487	ENSMUSG00000037487	15	37912458	38023050	259	299		
11910	8	103633042	103642422	ENSMUSG00000061923	ENSMUSG00000061923	ENSMUSG00000061923	15	38164019	38171228	259	299		
11911	8	103730189	103737127	ENSMUSG00000037465	ENSMUSG00000037465	ENSMUSG00000037465	15	38239002	38245296	259	299		
11912	8	103907712	103945573	ENSMUSG00000037458	ENSMUSG00000037458	ENSMUSG00000037458	15	38432019	38463855	259	299		
11913	8	104122241	104154460	ENSMUSG00000022295	ENSMUSG00000022295	ENSMUSG00000022295	15	38606522	38636785	259	299		
11914	8	104222097	104311706	ENSMUSG00000022296	ENSMUSG00000022296	ENSMUSG00000022296	15	38763974	38782988	259	299		
11915	8	104379843	104414214	ENSMUSG00000022297	ENSMUSG00000022297	ENSMUSG00000022297	15	38836426	38868268	259	299		
11916	8	104453061	104464397	ENSMUSG00000054196	ENSMUSG00000054196	ENSMUSG00000054196	15	38907006	38917193	259	299		
11917	8	104480045	104496447	ENSMUSG00000022299	ENSMUSG00000022299	ENSMUSG00000022299	15	38924351	38942712	259	299		
11918	8	104496118	104524376	ENSMUSG00000022300	ENSMUSG00000022300	ENSMUSG00000022300	15	38943006	38976932	259	299		
11919	8	104582291	105333224	ENSMUSG00000037386	ENSMUSG00000037386	ENSMUSG00000037386	15	39028406	39512014	259	299		
11920	8	105421228	105438092	ENSMUSG00000022303	ENSMUSG00000022303	ENSMUSG00000022303	15	39576006	39591008	259	299		
11921	8	105460829	105548453	ENSMUSG00000022304	ENSMUSG00000022304	ENSMUSG00000022304	15	39598559	39687541	259	299		
11922	8	105570643	105670327	ENSMUSG00000022305	ENSMUSG00000022305	ENSMUSG00000022305	15	39700677	39773831	259	299		
11923	8	106500550	106884942	ENSMUSG00000022306	ENSMUSG00000022306	ENSMUSG00000022306	15	40485116	40934666	259	299		
11924	8	107739270	107834093	ENSMUSG00000022307	ENSMUSG00000022307	ENSMUSG00000022307	15	41619589	41691122	259	299		
11925	8	107840888	107851648	ENSMUSG00000042895	ENSMUSG00000042895	ENSMUSG00000042895	15	41695367	41699794	259	299		
11926	8	108330899	108579459	ENSMUSG00000022309	ENSMUSG00000022309	ENSMUSG00000022309	15	42254801	42507051	259	299		
11927	8	108980721	109165052	ENSMUSG00000051920	ENSMUSG00000051920	ENSMUSG00000051920	15	42850869	43000892	259	299		
11928	8	109525029	109568311	ENSMUSG00000022337	ENSMUSG00000022337	ENSMUSG00000022337	15	43307303	43357851	259	299		
11929	8	109864522	109868946	ENSMUSG00000054409	ENSMUSG00000054409	ENSMUSG00000054409	15	43694721	43700098	259	299		
11930	8	110168900	110200989	ENSMUSG00000038760	ENSMUSG00000038760	ENSMUSG00000038760	15	44027160	44059621	259	299		
11931	8	110322325	110415491	ENSMUSG00000038736	ENSMUSG00000038736	ENSMUSG00000038736	15	44205305	44257947	259	299		
11932	8	110443882	110611740	ENSMUSG00000038725	ENSMUSG00000038725	ENSMUSG00000038725	15	44287627	44427217	259	299		
11933	8	110632230	110645964	ENSMUSG00000022339	ENSMUSG00000022339	ENSMUSG00000022339	15	44449387	44471098	259	299		
11934	8	110655593	110730184	ENSMUSG00000022340	ENSMUSG00000022340	ENSMUSG00000022340	15	44501930	44618137	259	299		
11935	8	111048411	111056135	ENSMUSG00000022342	ENSMUSG00000022342	ENSMUSG00000022342	15	44936358	44944901	259	299		
11936	8	113304337	114518418	ENSMUSG00000022311	ENSMUSG00000022311	ENSMUSG00000022311	15	47411797	47831695	259	299		
11937	8	116493971	116888939	ENSMUSG00000038679	ENSMUSG00000038679	ENSMUSG00000038679	15	50490508	50720115	259	299		
11938	8	117927355	117956182	ENSMUSG00000022314	ENSMUSG00000022314	ENSMUSG00000022314	15	51792679	51821774	259	299		
11939	8	118019664	118024403	ENSMUSG00000068522	ENSMUSG00000068522	ENSMUSG00000068522	15	51870257	51875795	259	299		
11940	8	118032399	118258131	ENSMUSG00000022315	ENSMUSG00000022315	ENSMUSG00000022315	15	52125636	52165816	259	299		
11941	8	118602211	118621680	ENSMUSG00000038622	ENSMUSG00000038622	ENSMUSG00000038622	15	52542528	52560514	259	299		
11942	8	118875910	119193119	ENSMUSG00000061731	ENSMUSG00000061731	ENSMUSG00000061731	15	52898747	53175446	259	299		
11943	8	119459408	119703301	ENSMUSG00000058656	ENSMUSG00000058656	ENSMUSG00000058656	15	53488434	53690375	259	299		
11944	8	120004978	120033492	ENSMUSG00000063727	ENSMUSG00000063727	ENSMUSG00000063727	15	54080702	54108567	259	299		
11945	8	120148627	120188376	ENSMUSG00000038591	ENSMUSG00000038591	ENSMUSG00000038591	15	54240857	54296441	259	299		
11946	8	120289893	120327090	ENSMUSG00000024479	ENSMUSG00000024479	ENSMUSG00000024479	15	54401568	54432921	259	299		
11947	8	120497882	120505776	ENSMUSG00000037362	ENSMUSG00000037362	ENSMUSG00000037362	15	54576005	54583842	259	299		
11948	8	120638507	120720251	ENSMUSG00000022425	ENSMUSG00000022425	ENSMUSG00000022425	15	54668984	54750085	259	299		
11949	8	120813345	120913985	ENSMUSG00000037343	ENSMUSG00000037343	ENSMUSG00000037343	15	54852286	54902012	259	299		
11950	8	120915398	120937330	ENSMUSG00000022422	ENSMUSG00000022422	ENSMUSG00000022422	15	54906185	54920574	259	299		
11951	8	120955146	121131937	ENSMUSG00000022419	ENSMUSG00000022419	ENSMUSG00000022419	15	54942407	55082862	259	299		
11952	8	121206533	121453447	ENSMUSG00000022371	ENSMUSG00000022371	ENSMUSG00000022371	15	55137834	55350884	259	299		
11953	8	121477264	121526828	ENSMUSG00000022370	ENSMUSG00000022370	ENSMUSG00000022370	15	55364178	55387401	259	299		
11954	8	121526847	121605056	ENSMUSG00000022369	ENSMUSG00000022369	ENSMUSG00000022369	15	55387491	55456506	259	299		
11955	8	121619297	121893264	ENSMUSG00000060429	ENSMUSG00000060429	ENSMUSG00000060429	15	55469237	55737032	259	299		
11956	8	122694719	122722811	ENSMUSG00000022367	ENSMUSG00000022367	ENSMUSG00000022367	15	56497742	56512287	259	299		
11957	8	123863082	124055936	ENSMUSG00000071757	ENSMUSG00000071757	ENSMUSG00000071757	15	57524854	57669854	259	299		
11958	8	124094769	124123722	ENSMUSG00000022365	ENSMUSG00000022365	ENSMUSG00000022365	15	57699585	57722501	259	299		
11959	8	124154101	124233571	ENSMUSG00000022364	ENSMUSG00000022364	ENSMUSG00000022364	15	57742336	57800144	259	299		
11960	8	124263933	124290384	ENSMUSG00000051225	ENSMUSG00000051225	ENSMUSG00000051225	15	57815993	57825685	259	299		
11961	8	124301389	124322793	ENSMUSG00000022362	ENSMUSG00000022362	ENSMUSG00000022362	15	57847890	57864299	259	299		
11962	8	124329878	124355728	ENSMUSG00000022361	ENSMUSG00000022361	ENSMUSG00000022361	15	57877389	57906552	259	299		
11963	8	124401922	124477868	ENSMUSG00000022360	ENSMUSG00000022360	ENSMUSG00000022360	15	57925610	57948546	259	299		
11964	8	124498146	124523441	ENSMUSG00000022359	ENSMUSG00000022359	ENSMUSG00000022359	15	57971519	57988737	259	299		
11965	8	124584540	124622627	ENSMUSG00000022358	ENSMUSG00000022358	ENSMUSG00000022358	15	58010162	58044957	259	299		
11966	8	124727160	124734347	ENSMUSG00000022357	ENSMUSG00000022357	ENSMUSG00000022357	15	58144656	58154252	259	299		
11967	8	124762216	124818828	ENSMUSG00000055114	ENSMUSG00000055114	ENSMUSG00000055114	15	58171638	58194231	259	299		
11968	8	124860078	124894260	ENSMUSG00000037119	ENSMUSG00000037119	ENSMUSG00000037119	15	58245565	58287820	259	299		
11969	8	125393412	125454110	ENSMUSG00000062373	ENSMUSG00000062373	ENSMUSG00000062373	15	58612352	58653510	259	299		
11970	8	125532243	125534274	ENSMUSG00000037085	ENSMUSG00000037085	ENSMUSG00000037085	15	58702757	58706861	259	299		
11971	8	125556189	125570027	ENSMUSG00000037075	ENSMUSG00000037075	ENSMUSG00000037075	15	58719312	58732473	259	299		
11972	8	125569932	125620510	ENSMUSG00000050891	ENSMUSG00000050891	ENSMUSG00000050891	15	58730015	58758854	259	299		
11973	8	125632212	125809840	ENSMUSG00000022353	ENSMUSG00000022353	ENSMUSG00000022353	15	58771324	58912106	259	299		
11974	8	126079901	126103707	ENSMUSG00000022351	ENSMUSG00000022351	ENSMUSG00000022351	15	59144773	59160874	259	299		
11975	8	126105691	126173191	ENSMUSG00000022350	ENSMUSG00000022350	ENSMUSG00000022350	15	59161679	59203848	259	299		
11976	8	126511745	126519824	ENSMUSG00000032501	ENSMUSG00000032501	ENSMUSG00000032501	15	59478335	59486780	259	299		
11977	8	127633871	127639648	ENSMUSG00000072568	ENSMUSG00000072568	ENSMUSG00000072568	15	60652746	60653576	259	299		
11978	8	128817498	128822853	ENSMUSG00000022346	ENSMUSG00000022346	ENSMUSG00000022346	15	61815052	61820027	259	299		
11979	8	130922903	131021187	ENSMUSG00000022378	ENSMUSG00000022378	ENSMUSG00000022378	15	63758763	63890121	259	299		
11980	8	131133535	131483399	ENSMUSG00000022377	ENSMUSG00000022377	ENSMUSG00000022377	15	63918209	64212592	259	299		
11981	8	131861736	132123854	ENSMUSG00000022376	ENSMUSG00000022376	ENSMUSG00000022376	15	64528707	64751833	259	299		
11982	8	132985549	133094951	ENSMUSG00000015002	ENSMUSG00000015002	ENSMUSG00000015002	15	65616714	65698924	259	299		
11983	8	133105910	133136455	ENSMUSG00000015001	ENSMUSG00000015001	ENSMUSG00000015001	15	65705729	65742070	259	299		
11984	8	133145215	133162535	ENSMUSG00000072511	ENSMUSG00000072511	ENSMUSG00000072511	15	65752758	65802512	259	299		
11985	8	133210691	133561961	ENSMUSG00000056258	ENSMUSG00000056258	ENSMUSG00000056258	15	65824844	66115893	259	299		
11986	8	133653631	133756995	ENSMUSG00000022375	ENSMUSG00000022375	ENSMUSG00000022375	15	66210063	66319313	259	299		
11987	8	133791376	133842010	ENSMUSG00000036944	ENSMUSG00000036944	ENSMUSG00000036944	15	66362254	66388988	259	299		
11988	8	133856786	133906756	ENSMUSG00000072501	ENSMUSG00000072501	ENSMUSG00000072501	15	66407235	66453755	259	299		
11989	8	133913661	133928255	ENSMUSG00000036933	ENSMUSG00000036933	ENSMUSG00000036933	15	66460527	66473607	259	299		
11990	8	133948387	134216325	ENSMUSG00000053469	ENSMUSG00000053469	ENSMUSG00000053469	15	66500422	66680286	259	299		
11991	8	134118156	134184074	ENSMUSG00000022372	ENSMUSG00000022372	ENSMUSG00000022372	15	66610488	66642363	259	299		
11992	8	134272494	134310751	ENSMUSG00000005124	ENSMUSG00000005124	ENSMUSG00000005124	15	66721062	66752868	259	299		
11993	8	134318601	134379011	ENSMUSG00000005125	ENSMUSG00000005125	ENSMUSG00000005125	15	66758991	66799308	259	299		
11994	8	134540327	134653349	ENSMUSG00000013846	ENSMUSG00000013846	ENSMUSG00000013846	15	66932544	66943661	259	299		
11995	8	135559217	135739173	ENSMUSG00000022335	ENSMUSG00000022335	ENSMUSG00000022335	15	67913991	68054613	259	299		
11996	8	136538884	136729028	ENSMUSG00000022332	ENSMUSG00000022332	ENSMUSG00000022332	15	68758089	68923188	259	299		
11997	8	139213708	139449429	ENSMUSG00000036800	ENSMUSG00000036800	ENSMUSG00000036800	15	71275572	71358915	259	299		
11998	8	140693986	140784481	ENSMUSG00000036760	ENSMUSG00000036760	ENSMUSG00000036760	15	72339375	72373535	259	299		
11999	8	140811770	141537860	ENSMUSG00000047921	ENSMUSG00000047921	ENSMUSG00000047921	15	72416874	72888460	259	299		
12000	8	141590586	141596434	ENSMUSG00000068391	ENSMUSG00000068391	ENSMUSG00000068391	15	72917668	72921331	259	299		
12001	8	141610447	141714827	ENSMUSG00000036698	ENSMUSG00000036698	ENSMUSG00000036698	15	72934164	72973825	259	299		
12002	8	141737683	142080514	ENSMUSG00000022607	ENSMUSG00000022607	ENSMUSG00000022607	15	73032591	73250447	259	299		
12003	8	142207902	142275077	ENSMUSG00000036661	ENSMUSG00000036661	ENSMUSG00000036661	15	73339916	73399481	259	299		
12004	8	142290052	142307855	ENSMUSG00000036649	ENSMUSG00000036649	ENSMUSG00000036649	15	73407549	73444580	259	299		
12005	8	142436129	142437205	ENSMUSG00000045281	ENSMUSG00000045281	ENSMUSG00000045281	15	73521862	73534760	259	299		
12006	8	142501189	142510802	ENSMUSG00000059895	ENSMUSG00000059895	ENSMUSG00000059895	15	73552053	73584482	259	299		
12007	8	143542379	143623348	ENSMUSG00000034730	ENSMUSG00000034730	ENSMUSG00000034730	15	74344086	74416719	259	299		
12008	8	143689412	143692835	ENSMUSG00000022602	ENSMUSG00000022602	ENSMUSG00000022602	15	74496338	74499825	259	299		
12009	8	143758926	143761144	ENSMUSG00000022598	ENSMUSG00000022598	ENSMUSG00000022598	15	74542094	74544324	259	299		
12010	8	143805623	143815345	ENSMUSG00000056665	ENSMUSG00000056665	ENSMUSG00000056665	15	74548489	74551628	259	299		
12011	8	143819364	143820831	ENSMUSG00000022596	ENSMUSG00000022596	ENSMUSG00000022596	15	74551573	74555289	259	299		
12012	8	143828631	143830954	ENSMUSG00000022595	ENSMUSG00000022595	ENSMUSG00000022595	15	74559508	74561584	259	299		
12013	8	143842762	143856641	ENSMUSG00000075605	ENSMUSG00000075605	ENSMUSG00000075605	15	74570252	74573930	259	299		
12014	8	143863300	143865010	ENSMUSG00000034634	ENSMUSG00000034634	ENSMUSG00000034634	15	74589311	74590822	259	299		
12015	8	143778533	143782602	ENSMUSG00000044678	ENSMUSG00000044678	ENSMUSG00000044678	15	74624129	74627223	259	299	here	
12016	8	144171274	144175190	ENSMUSG00000022587	ENSMUSG00000022587	ENSMUSG00000022587	15	74782872	74787158	259	300	Single marker moved to different place with in a HSB	1 -> 2
12017	8	144310955	144312497	ENSMUSG00000022577	ENSMUSG00000022577	ENSMUSG00000022577	15	75378523	75393903	259	300		
12018	8	144366443	144370418	ENSMUSG00000022579	ENSMUSG00000022579	ENSMUSG00000022579	15	75423897	75425467	259	300		
12019	8	144462910	144488425	ENSMUSG00000000934	ENSMUSG00000000934	ENSMUSG00000000934	15	75484288	75506045	259	300		
12020	8	144522432	144537532	ENSMUSG00000022580	ENSMUSG00000022580	ENSMUSG00000022580	15	75531626	75541501	259	300		
12021	8	144582658	144583719	ENSMUSG00000047591	ENSMUSG00000047591	ENSMUSG00000047591	15	75574098	75575177	259	300		
12022	8	144711631	144716375	ENSMUSG00000022575	ENSMUSG00000022575	ENSMUSG00000022575	15	75689582	75694659	259	300		
12023	8	144728151	144731652	ENSMUSG00000022574	ENSMUSG00000022574	ENSMUSG00000022574	15	75718219	75721736	259	300		
12024	8	144758220	144762907	ENSMUSG00000022571	ENSMUSG00000022571	ENSMUSG00000022571	15	75743773	75748815	259	300		
12025	8	144765937	144770852	ENSMUSG00000022570	ENSMUSG00000022570	ENSMUSG00000022570	15	75751939	75757008	259	300		
12026	8	144803097	144804902	ENSMUSG00000050846	ENSMUSG00000050846	ENSMUSG00000050846	15	75768207	75776655	259	300		
12027	8	144860852	144861670	ENSMUSG00000044095	ENSMUSG00000044095	ENSMUSG00000044095	15	75808022	75808885	259	300		
12028	8	144870503	144876619	ENSMUSG00000063704	ENSMUSG00000063704	ENSMUSG00000063704	15	75821024	75826408	259	300		
12029	8	144880082	144884740	ENSMUSG00000046761	ENSMUSG00000046761	ENSMUSG00000046761	15	75828358	75841591	259	300		
12030	8	144945084	144969532	ENSMUSG00000022568	ENSMUSG00000022568	ENSMUSG00000022568	15	75874441	75896985	259	300		
12031	8	144970537	144978578	ENSMUSG00000002524	ENSMUSG00000002524	ENSMUSG00000002524	15	75897583	75904957	259	300		
12032	8	144987902	144992234	ENSMUSG00000075590	ENSMUSG00000075590	ENSMUSG00000075590	15	75912850	75918760	259	300		
12033	8	145061309	145097032	ENSMUSG00000022565	ENSMUSG00000022565	ENSMUSG00000022565	15	75998231	76033576	259	300		
12034	8	145123309	145132568	ENSMUSG00000063268	ENSMUSG00000063268	ENSMUSG00000063268	15	76060258	76070689	259	300		
12035	8	145136214	145139571	ENSMUSG00000022564	ENSMUSG00000022564	ENSMUSG00000022564	15	76074030	76077159	259	300		
12036	8	145158595	145174002	ENSMUSG00000049653	ENSMUSG00000049653	ENSMUSG00000049653	15	76095344	76119828	259	300		
12037	8	145177412	145178083	ENSMUSG00000071724	ENSMUSG00000071724	ENSMUSG00000071724	15	76121755	76124150	259	300		
12038	8	145178165	145186923	ENSMUSG00000022562	ENSMUSG00000022562	ENSMUSG00000022562	15	76123858	76134500	259	300		
12039	8	145205620	145207536	ENSMUSG00000034259	ENSMUSG00000034259	ENSMUSG00000034259	15	76154720	76157928	259	300		
12040	8	145209512	145213102	ENSMUSG00000022561	ENSMUSG00000022561	ENSMUSG00000022561	15	76158549	76162154	259	300		
12041	8	145221968	145224415	ENSMUSG00000022551	ENSMUSG00000022551	ENSMUSG00000022551	15	76170778	76173189	259	300		
12042	8	145225529	145231081	ENSMUSG00000022552	ENSMUSG00000022552	ENSMUSG00000022552	15	76174295	76178352	259	300		
12043	8	145231306	145234503	ENSMUSG00000022553	ENSMUSG00000022553	ENSMUSG00000022553	15	76178561	76181630	259	300		
12044	8	145486088	145509187	ENSMUSG00000022556	ENSMUSG00000022556	ENSMUSG00000022556	15	76304700	76328241	259	300		
12045	8	145510763	145521360	ENSMUSG00000022555	ENSMUSG00000022555	ENSMUSG00000022555	15	76329270	76339073	259	300		
12046	8	145525262	145530751	ENSMUSG00000048385	ENSMUSG00000048385	ENSMUSG00000048385	15	76343464	76349157	259	300		
12047	8	145549900	145552915	ENSMUSG00000022559	ENSMUSG00000022559	ENSMUSG00000022559	15	76362985	76365971	259	300		
12048	8	145568539	145589259	ENSMUSG00000022550	ENSMUSG00000022550	ENSMUSG00000022550	15	76403614	76423063	259	300		
12049	8	145589254	145605541	ENSMUSG00000034022	ENSMUSG00000034022	ENSMUSG00000034022	15	76423064	76434846	259	300		
12050	8	145608607	145613081	ENSMUSG00000063354	ENSMUSG00000063354	ENSMUSG00000063354	15	76439640	76444147	259	300		
12051	8	145620078	145624735	ENSMUSG00000062381	ENSMUSG00000062381	ENSMUSG00000062381	15	76449364	76453329	259	300		
12052	8	145624999	145640466	ENSMUSG00000059323	ENSMUSG00000059323	ENSMUSG00000059323	15	76453492	76467166	259	300		
12053	8	145646123	145653832	ENSMUSG00000053929	ENSMUSG00000053929	ENSMUSG00000053929	15	76474184	76484175	259	300		
12054	8	145662546	145670306	ENSMUSG00000004187	ENSMUSG00000004187	ENSMUSG00000004187	15	76487911	76495451	259	300		
12055	8	145670429	145672029	ENSMUSG00000033837	ENSMUSG00000033837	ENSMUSG00000033837	15	76495480	76497126	259	300		
12056	8	145693386	145698311	ENSMUSG00000033819	ENSMUSG00000033819	ENSMUSG00000033819	15	76498935	76522170	259	300		
12057	8	145699164	145703365	ENSMUSG00000022546	ENSMUSG00000022546	ENSMUSG00000022546	15	76523988	76526929	259	300		
12058	8	145705271	145707387	ENSMUSG00000019080	ENSMUSG00000019080	ENSMUSG00000019080	15	76528797	76531494	259	300		
12059	8	145707480	145713976	ENSMUSG00000033762	ENSMUSG00000033762	ENSMUSG00000033762	15	76530815	76537803	259	300		
12060	8	145714199	145721365	ENSMUSG00000033728	ENSMUSG00000033728	ENSMUSG00000033728	15	76540147	76543903	259	300		
12061	8	145718585	145723210	ENSMUSG00000033707	ENSMUSG00000033707	ENSMUSG00000033707	15	76542531	76549428	259	300		
12062	8	145725371	145804869	ENSMUSG00000033697	ENSMUSG00000033697	ENSMUSG00000033697	15	76551240	76592819	259	300		
12063	8	145917715	145951659	ENSMUSG00000022526	ENSMUSG00000022526	ENSMUSG00000022526	15	76679386	76698690	259	300		
12064	8	146023749	146039409	ENSMUSG00000033669	ENSMUSG00000033669	ENSMUSG00000033669	15	76708367	76719072	259	300		
12065	8	146077704	146097616	ENSMUSG00000054967	ENSMUSG00000054967	ENSMUSG00000054967	15	76738106	76745265	259	300		
12066	8	146248770	146252219	ENSMUSG00000063236	ENSMUSG00000063236	ENSMUSG00000063236	15	76775878	76777989	259	300		
12067	22	34332757	34349347	ENSMUSG00000018893	ENSMUSG00000018893	ENSMUSG00000018893	15	76842742	76877925	260	301		
12068	22	34374370	34388903	ENSMUSG00000033576	ENSMUSG00000033576	ENSMUSG00000033576	15	76872531	76884362	260	301		
12069	22	34470098	34754495	ENSMUSG00000033565	ENSMUSG00000033565	ENSMUSG00000033565	15	76911057	77133860	260	301		
12070	22	35007273	35113958	ENSMUSG00000022443	ENSMUSG00000022443	ENSMUSG00000022443	15	77587843	77669354	260	301		
12071	22	35193039	35207633	ENSMUSG00000005354	ENSMUSG00000005354	ENSMUSG00000005354	15	77742999	77755209	260	301		
12072	22	35214250	35233015	ENSMUSG00000016552	ENSMUSG00000016552	ENSMUSG00000016552	15	77767783	77783907	260	301		
12073	22	35236844	35255429	ENSMUSG00000016554	ENSMUSG00000016554	ENSMUSG00000016554	15	77786756	77795834	260	301		
12074	22	35290050	35428849	ENSMUSG00000019146	ENSMUSG00000019146	ENSMUSG00000019146	15	77822086	77947283	260	301		
12075	22	35489793	35502115	ENSMUSG00000016637	ENSMUSG00000016637	ENSMUSG00000016637	15	77986715	78001363	260	301		
12076	22	35526694	35545463	ENSMUSG00000005716	ENSMUSG00000005716	ENSMUSG00000005716	15	78018360	78033606	260	301		
12077	22	35586991	35602060	ENSMUSG00000071715	ENSMUSG00000071715	ENSMUSG00000071715	15	78072089	78089829	260	301		
12078	22	35717143	35733789	ENSMUSG00000062154	ENSMUSG00000062154	ENSMUSG00000062154	15	78205659	78223140	260	301		
12079	22	35736853	35745437	ENSMUSG00000044986	ENSMUSG00000044986	ENSMUSG00000044986	15	78226879	78233102	260	301		
12080	22	35745648	35755808	ENSMUSG00000071711	ENSMUSG00000071711	ENSMUSG00000071711	15	78237294	78241257	260	301		
12081	22	35777739	35789373	ENSMUSG00000033287	ENSMUSG00000033287	ENSMUSG00000033287	15	78255934	78265811	260	301		
12082	22	35791425	35835395	ENSMUSG00000016942	ENSMUSG00000016942	ENSMUSG00000016942	15	78266923	78295866	260	301		
12083	22	35851824	35875908	ENSMUSG00000068227	ENSMUSG00000068227	ENSMUSG00000068227	15	78307808	78322321	260	301		
12084	22	35906152	35914276	ENSMUSG00000022440	ENSMUSG00000022440	ENSMUSG00000022440	15	78352106	78354729	260	301		
12085	22	35932191	35938299	ENSMUSG00000044933	ENSMUSG00000044933	ENSMUSG00000044933	15	78364270	78371600	260	301		
12086	22	35951238	35970241	ENSMUSG00000033220	ENSMUSG00000033220	ENSMUSG00000033220	15	78386424	78400038	260	301		
12087	22	36008370	36041326	ENSMUSG00000018008	ENSMUSG00000018008	ENSMUSG00000018008	15	78424373	78449268	260	301		

12088	22	36099058	36101520	ENSMUSG00000043460	ENSMUSG00000043460	ENSMUSG00000043460	15	78497246	78545368	260	301
12089	22	36195054	36212385	ENSMUSG00000018169	ENSMUSG00000018169	ENSMUSG00000018169	15	78583137	78600713	260	301
12090	22	36216351	36245193	ENSMUSG00000033170	ENSMUSG00000033170	ENSMUSG00000033170	15	78602391	78630297	260	301
12091	22	36286446	36295358	ENSMUSG00000049521	ENSMUSG00000049521	ENSMUSG00000049521	15	78669902	78678157	260	301
12092	22	36296211	36306010	ENSMUSG00000043501	ENSMUSG00000043501	ENSMUSG00000043501	15	78678262	78679613	260	301
12093	22	36334678	36358753	ENSMUSG00000033128	ENSMUSG00000033128	ENSMUSG00000033128	15	78704445	78721840	260	301
12094	22	36365630	36381939	ENSMUSG00000022436	ENSMUSG00000022436	ENSMUSG00000022436	15	78728714	78739295	260	301
12095	22	36401559	36405736	ENSMUSG00000068220	ENSMUSG00000068220	ENSMUSG00000068220	15	78753980	78757722	260	301
12096	22	36412307	36419429	ENSMUSG00000033099	ENSMUSG00000033099	ENSMUSG00000033099	15	78762186	78770889	260	301
12097	22	36441776	36500076	ENSMUSG00000033088	ENSMUSG00000033088	ENSMUSG00000033088	15	78778288	78815203	260	301
12098	22	36533891	36543129	ENSMUSG00000006378	ENSMUSG00000006378	ENSMUSG00000006378	15	78858175	78865609	260	301
12099	22	36549335	36551448	ENSMUSG00000047429	ENSMUSG00000047429	ENSMUSG00000047429	15	78869187	78870728	260	301
12100	22	36556809	36570266	ENSMUSG00000033055	ENSMUSG00000033055	ENSMUSG00000033055	15	78880357	78890140	260	301
12101	22	36574821	36614581	ENSMUSG00000033047	ENSMUSG00000033047	ENSMUSG00000033047	15	78902473	78921660	260	301
12102	22	36632292	36667971	ENSMUSG00000033039	ENSMUSG00000033039	ENSMUSG00000033039	15	78936500	78964153	260	301
12103	22	36669479	36679551	ENSMUSG00000033029	ENSMUSG00000033029	ENSMUSG00000033029	15	78961910	78968506	260	301
12104	22	36679665	36694653	ENSMUSG00000033020	ENSMUSG00000033020	ENSMUSG00000033020	15	78968610	78979021	260	301
12105	22	36698254	36713375	ENSMUSG00000033006	ENSMUSG00000033006	ENSMUSG00000033006	15	78982186	78992495	260	301
12106	22	36783208	36801646	ENSMUSG00000068206	ENSMUSG00000068206	ENSMUSG00000068206	15	79056646	79083929	260	301
12107	22	36804093	36809095	ENSMUSG00000032988	ENSMUSG00000032988	ENSMUSG00000032988	15	79078269	79082012	260	301
12108	22	36810856	36836622	ENSMUSG00000018126	ENSMUSG00000018126	ENSMUSG00000018126	15	79085450	79112702	260	301
12109	22	36837449	36907709	ENSMUSG00000042632	ENSMUSG00000042632	ENSMUSG00000042632	15	79113485	79155439	260	301
12110	22	36927944	36942456	ENSMUSG00000042622	ENSMUSG00000042622	ENSMUSG00000042622	15	79174933	79186336	260	301
12111	22	36945244	36998962	ENSMUSG00000009035	ENSMUSG00000009035	ENSMUSG00000009035	15	79187939	79230558	260	301
12112	22	37017870	37124473	ENSMUSG00000022433	ENSMUSG00000022433	ENSMUSG00000022433	15	79245107	79266120	260	301
12113	22	37152278	37181149	ENSMUSG00000044216	ENSMUSG00000044216	ENSMUSG00000044216	15	79310969	79332496	260	301
12114	22	37194029	37209391	ENSMUSG00000010830	ENSMUSG00000010830	ENSMUSG00000010830	15	79343723	79354994	260	301
12115	22	37209389	37232262	ENSMUSG00000055065	ENSMUSG00000055065	ENSMUSG00000055065	15	79354997	79373996	260	301
12116	22	37244900	37296135	ENSMUSG00000022429	ENSMUSG00000022429	ENSMUSG00000022429	15	79388755	79432339	260	301
12117	22	37308300	37376101	ENSMUSG00000042564	ENSMUSG00000042564	ENSMUSG00000042564	15	79462560	79486211	260	301
12118	22	37382604	37399795	ENSMUSG00000022428	ENSMUSG00000022428	ENSMUSG00000022428	15	79486482	79494915	260	301
12119	22	37431753	37459536	ENSMUSG00000042535	ENSMUSG00000042535	ENSMUSG00000042535	15	79518151	79548736	260	301
12120	22	37460689	37481928	ENSMUSG00000042524	ENSMUSG00000042524	ENSMUSG00000042524	15	79551326	79569711	260	301
12121	22	37548563	37598141	ENSMUSG00000022421	ENSMUSG00000022421	ENSMUSG00000022421	15	79616930	79661492	260	301
12122	22	37823236	37829946	ENSMUSG00000009585	ENSMUSG00000009585	ENSMUSG00000009585	15	79719704	79734624	260	301
12123	22	37856736	37878484	ENSMUSG00000053411	ENSMUSG00000053411	ENSMUSG00000053411	15	79743065	79759894	260	301
12124	22	37949310	37971006	ENSMUSG00000000489	ENSMUSG00000000489	ENSMUSG00000000489	15	79823129	79842063	260	301
12125	22	38075900	38111526	ENSMUSG00000022415	ENSMUSG00000022415	ENSMUSG00000022415	15	79918589	79946756	260	301
12126	22	38125692	38157831	ENSMUSG00000022414	ENSMUSG00000022414	ENSMUSG00000022414	15	79960409	79988964	260	301
12127	22	38183271	38215385	ENSMUSG00000042428	ENSMUSG00000042428	ENSMUSG00000042428	15	80001066	80042774	260	301
12128	22	38228230	38241324	ENSMUSG00000022412	ENSMUSG00000022412	ENSMUSG00000022412	15	80061375	80079979	260	301
12129	22	38246515	38248637	ENSMUSG00000042406	ENSMUSG00000042406	ENSMUSG00000042406	15	80082439	80084794	260	301
12130	22	38255048	38258806	ENSMUSG00000051518	ENSMUSG00000051518	ENSMUSG00000051518	15	80087869	80091561	260	301
12131	22	38296704	38415681	ENSMUSG00000022416	ENSMUSG00000022416	ENSMUSG00000022416	15	80125808	80216508	260	301
12132	22	38468996	38619740	ENSMUSG00000050439	ENSMUSG00000050439	ENSMUSG00000050439	15	80279630	80387607	260	301
12133	22	38627080	38698204	ENSMUSG00000042351	ENSMUSG00000042351	ENSMUSG00000042351	15	80399849	80476017	260	301
12134	22	38720899	38755777	ENSMUSG00000022408	ENSMUSG00000022408	ENSMUSG00000022408	15	80499120	80527677	260	301
12135	22	38971963	39061757	ENSMUSG00000047888	ENSMUSG00000047888	ENSMUSG00000047888	15	80611991	80768341	260	301
12136	22	39072466	39092929	ENSMUSG00000022407	ENSMUSG00000022407	ENSMUSG00000022407	15	80775774	80798202	260	301
12137	22	39096541	39136239	ENSMUSG00000042303	ENSMUSG00000042303	ENSMUSG00000042303	15	80805060	80839330	260	301
12138	22	39136252	39362651	ENSMUSG00000042292	ENSMUSG00000042292	ENSMUSG00000042292	15	80840540	80872613	260	301
12139	22	39405223	39407878	ENSMUSG00000050164	ENSMUSG00000050164	ENSMUSG00000050164	15	81062824	81065367	260	301
12140	22	39495587	39545280	ENSMUSG00000022404	ENSMUSG00000022404	ENSMUSG00000022404	15	81146176	81188029	260	301
12141	22	39583037	39658766	ENSMUSG00000022401	ENSMUSG00000022401	ENSMUSG00000022401	15	81227443	81282143	260	301
12142	22	39677331	39698628	ENSMUSG00000022400	ENSMUSG00000022400	ENSMUSG00000022400	15	81293628	81301187	260	301
12143	22	39817736	39905472	ENSMUSG00000055024	ENSMUSG00000055024	ENSMUSG00000055024	15	81413469	81479332	260	301
12144	22	39931259	39957220	ENSMUSG00000022394	ENSMUSG00000022394	ENSMUSG00000022394	15	81491191	81515560	260	301
12145	22	40027513	40086097	ENSMUSG00000022390	ENSMUSG00000022390	ENSMUSG00000022390	15	81595256	81613234	260	301
12146	22	40107909	40125272	ENSMUSG00000022389	ENSMUSG00000022389	ENSMUSG00000022389	15	81638728	81654118	260	301
12147	22	40159438	40172973	ENSMUSG00000048546	ENSMUSG00000048546	ENSMUSG00000048546	15	81676168	81685512	260	301
12148	22	40185668	40194654	ENSMUSG00000061360	ENSMUSG00000061360	ENSMUSG00000061360	15	81692576	81699163	260	301
12149	22	40195075	40254939	ENSMUSG00000022477	ENSMUSG00000022477	ENSMUSG00000022477	15	81699718	81742392	260	301
12150	22	40251754	40270412	ENSMUSG00000022476	ENSMUSG00000022476	ENSMUSG00000022476	15	81715483	81753151	260	301
12151	22	40287020	40302610	ENSMUSG00000042109	ENSMUSG00000042109	ENSMUSG00000042109	15	81764150	81778183	260	301
12152	22	40302853	40315817	ENSMUSG00000022474	ENSMUSG00000022474	ENSMUSG00000022474	15	81778367	81788119	260	301
12153	22	40326825	40346966	ENSMUSG00000022472	ENSMUSG00000022472	ENSMUSG00000022472	15	81819778	81843416	260	301
12154	22	40347241	40389998	ENSMUSG00000022471	ENSMUSG00000022471	ENSMUSG00000022471	15	81842774	81867339	260	301
12155	22	40425489	40525410	ENSMUSG00000068117	ENSMUSG00000068117	ENSMUSG00000068117	15	81909719	81954064	260	301
12156	22	40526624	40552252	ENSMUSG00000068114	ENSMUSG00000068114	ENSMUSG00000068114	15	81955232	81969455	260	301
12157	22	40559063	40633254	ENSMUSG00000022463	ENSMUSG00000022463	ENSMUSG00000022463	15	81974658	82031931	260	301
12158	22	40650991	40652728	ENSMUSG00000068105	ENSMUSG00000068105	ENSMUSG00000068105	15	82048999	82051615	260	301
12159	22	40665006	40673026	ENSMUSG00000068101	ENSMUSG00000068101	ENSMUSG00000068101	15	82061848	82072512	260	301
12160	22	40702760	40724137	ENSMUSG00000022456	ENSMUSG00000022456	ENSMUSG00000022456	15	82107208	82119070	260	301
12161	22	40724722	40784406	ENSMUSG00000022455	ENSMUSG00000022455	ENSMUSG00000022455	15	82126718	82142387	260	301
12162	22	40784331	40796719	ENSMUSG00000022453	ENSMUSG00000022453	ENSMUSG00000022453	15	82157304	82166570	260	301
12163	22	40800201	40805387	ENSMUSG00000049687	ENSMUSG00000049687	ENSMUSG00000049687	15	82168687	82173507	260	301
12164	22	40805645	40810233	ENSMUSG00000022452	ENSMUSG00000022452	ENSMUSG00000022452	15	82173810	82176826	260	301
12165	22	40811477	40816834	ENSMUSG00000022450	ENSMUSG00000022450	ENSMUSG00000022450	15	82177927	82182055	260	301
12166	22	40885963	40941389	ENSMUSG00000041852	ENSMUSG00000041852	ENSMUSG00000041852	15	82636395	82685014	260	301
12167	22	41309671	41340906	ENSMUSG00000041815	ENSMUSG00000041815	ENSMUSG00000041815	15	82953744	82977118	260	301
12168	22	41344765	41375349	ENSMUSG00000018042	ENSMUSG00000018042	ENSMUSG00000018042	15	82981267	82999974	260	301
12169	22	41418565	41419901	ENSMUSG00000047878	ENSMUSG00000047878	ENSMUSG00000047878	15	83054488	83079495	260	301
12170	22	41522476	41583244	ENSMUSG00000054277	ENSMUSG00000054277	ENSMUSG00000054277	15	83130869	83177915	260	301
12171	22	41595721	41741095	ENSMUSG00000016664	ENSMUSG00000016664	ENSMUSG00000016664	15	83203373	83229692	260	301
12172	22	41836701	41855661	ENSMUSG00000016758	ENSMUSG00000016758	ENSMUSG00000016758	15	83354629	83372401	260	301
12173	22	41858853	41869340	ENSMUSG00000048755	ENSMUSG00000048755	ENSMUSG00000048755	15	83374563	83383477	260	301
12174	22	41877479	41889191	ENSMUSG00000041736	ENSMUSG00000041736	ENSMUSG00000041736	15	83391377	83401962	260	301
12175	22	41892572	41913051	ENSMUSG00000016757	ENSMUSG00000016757	ENSMUSG00000016757	15	83402860	83422886	260	301
12176	22	41929942	42069231	ENSMUSG00000016763	ENSMUSG00000016763	ENSMUSG00000016763	15	83434272	83552763	260	301
12177	22	42150936	42200789	ENSMUSG00000041708	ENSMUSG00000041708	ENSMUSG00000041708	15	83607789	83686240	260	301
12178	22	42255987	42539451	ENSMUSG00000022441	ENSMUSG00000022441	ENSMUSG00000022441	15	83694476	83893115	260	301
12179	22	42551722	42589731	ENSMUSG00000018865	ENSMUSG00000018865	ENSMUSG00000018865	15	83903863	83933520	260	301
12180	22	42606892	42619223	ENSMUSG00000018868	ENSMUSG00000018868	ENSMUSG00000018868	15	83940122	83950941	260	301
12181	22	42650952	42674781	ENSMUSG00000041653	ENSMUSG00000041653	ENSMUSG00000041653	15	83995640	84013851	260	301
12182	22	42682634	42723744	ENSMUSG00000022437	ENSMUSG00000022437	ENSMUSG00000022437	15	84023510	84041937	260	301
12183	22	42726506	42896434	ENSMUSG00000022438	ENSMUSG00000022438	ENSMUSG00000022438	15	84059809	84140797	260	301
12184	22	42909088	42933741	ENSMUSG00000022439	ENSMUSG00000022439	ENSMUSG00000022439	15	84152486	84170705	260	301
12185	22	43012643	43028158	ENSMUSG00000062760	ENSMUSG00000062760	ENSMUSG00000062760	15	84234214	84263043	260	301
12186	22	43267114	43272669	ENSMUSG00000055745	ENSMUSG00000055745	ENSMUSG00000055745	15	84384228	84384959	260	301
12187	22	43477019	43637250	ENSMUSG00000036106	ENSMUSG00000036106	ENSMUSG00000036106	15	84547836	84599970	260	301
12188	22	43655709	43784245	ENSMUSG00000016624	ENSMUSG00000016624	ENSMUSG00000016624	15	84613150	84682865	260	301
12189	22	43966787	43987011	ENSMUSG00000036046	ENSMUSG00000036046	ENSMUSG00000036046	15	84773486	84815737	260	301
12190	22	44059527	44070418	ENSMUSG00000022435	ENSMUSG00000022435	ENSMUSG00000022435	15	84844986	84850300	260	301
12191	22	44188244	44206966	ENSMUSG00000022431	ENSMUSG00000022431	ENSMUSG00000022431	15	84959844	84972335	260	301
12192	22	44277383	44375678	ENSMUSG00000006369	ENSMUSG00000006369	ENSMUSG00000006369	15	85033833	85114060	260	301
12193	22	44446351	44619493	ENSMUSG00000016541	ENSMUSG00000016541	ENSMUSG00000016541	15	85164231	85290233	260	301
12194	22	44696323	44751395	ENSMUSG00000022382	ENSMUSG00000022382	ENSMUSG00000022382	15	85363211	85409587	260	301
12195	22	44925163	45018317	ENSMUSG00000022383	ENSMUSG00000022383	ENSMUSG00000022383	15	85605326	85629024	260	301
12196	22	45018575	45024857	ENSMUSG00000064284	ENSMUSG00000064284	ENSMUSG00000064284	15	85635170	85637090	260	301
12197	22	45031122	45037883	ENSMUSG00000052496	ENSMUSG00000052496	ENSMUSG00000052496	15	85643119	85649499	260	301
12198	22	45042569	45068567	ENSMUSG00000035944	ENSMUSG00000035944	ENSMUSG00000035944	15	85660156	85684327	260	301
12199	22	45071476	45108257	ENSMUSG00000022385	ENSMUSG00000022385	ENSMUSG00000022385	15	85687511	85704296	260	301
12200	22	45109986	45131898	ENSMUSG00000022386	ENSMUSG00000022386	ENSMUSG00000022386	15	85707086	85725163	260	301
12201	22	45135395	45311731	ENSMUSG00000016028	ENSMUSG00000016028	ENSMUSG00000016028	15	85728699	85861536	260	301
12202	22	45394963	45454344	ENSMUSG00000035900	ENSMUSG00000035900	ENSMUSG00000035900	15	85886535	85965384	260	301
12203	22	45458974	45512816	ENSMUSG00000035891	ENSMUSG00000035891	ENSMUSG00000035891	15	85966893	86013907	260	301
12204	22	45537193	45948393	ENSMUSG00000051864	ENSMUSG00000051864	ENSMUSG00000051864	15	86042225	86326269	260	301
12205	22	48552936	48606828	ENSMUSG00000022387	ENSMUSG00000022387	ENSMUSG00000022387	15	88515066	88557682	260	301
12206	22	48682862	48698110	ENSMUSG00000035845	ENSMUSG00000035845	ENSMUSG00000035845	15	88633550	88647044	260	301
12207	22	48698348	48707178	ENSMUSG00000023272	ENSMUSG00000023272	ENSMUSG00000023272	15	88647412	88654447	260	301
12208	22	48740165	48743721	ENSMUSG00000035828	ENSMUSG00000035828	ENSMUSG00000035828	15	88689960	88693495	260	301
12209	22	48810981	48835873	ENSMUSG00000022388	ENSMUSG00000022388	ENSMUSG00000022388	15	88741664	88782176	260	301
12210	22	48839954	48866161	ENSMUSG00000035805	ENSMUSG00000035805	ENSMUSG00000035805	15	88783649	88806773	260	301
12211	22	48870621	48942242	ENSMUSG00000015365	ENSMUSG00000015365	ENSMUSG00000015365	15	88813324	88882914	260	301
12212	22	48951317	48959816	ENSMUSG00000058441	ENSMUSG00000058441	ENSMUSG00000058441	15	88887870	88897643	260	301
12213	22	48966474	48980146	ENSMUSG00000015363	ENSMUSG00000015363	ENSMUSG00000015363	15	88903837	88914836	260	301
12214	22	48981535	48998171	ENSMUSG00000035757	ENSMUSG00000035757	ENSMUSG00000035757	15	88916875	88927752	260	301
12215	22	49025742	49031941	ENSMUSG00000062906	ENSMUSG00000062906	ENSMUSG00000062906	15	88951077	88956447	260	301
12216	22	49033484	49042312	ENSMUSG00000022610	ENSMUSG00000022610	ENSMUSG00000022610	15	88958350	88968387	260	301
12217	22	49044295	49050949	ENSMUSG00000053137	ENSMUSG00000053137	ENSMUSG00000053137	15	88970253	88977372	260	301
12218	22	49055535	49071150	ENSMUSG00000036606	ENSMUSG00000036606	ENSMUSG00000036606	15	88983315	88995780	260	301
12219	22	49092965	49100030	ENSMUSG00000015377	ENSMUSG00000015377	ENSMUSG00000015377	15	89012578	89024258	260	301
12220	22	49157367	49229685	ENSMUSG00000036561	ENSMUSG00000036561	ENSMUSG00000036561	15	89039326	89114028	260	301
12221	22	49232437	49260135	ENSMUSG00000036529	ENSMUSG00000036529	ENSMUSG00000036529	15	89116053	89142934	260	301
12222	22	49267074	49271730	ENSMUSG00000054136	ENSMUSG00000054136	ENSMUSG00000054136	15	89150739	89152495	260	301
12223	22	49272079	49275616	ENSMUSG00000022613	ENSMUSG00000022613	ENSMUSG00000022613	15	89162230	89164776	260	301
12224	22	49288246	49292979	ENSMUSG00000022614	ENSMUSG00000022614	ENSMUSG00000022614	15	89178771	89183384	260	301
12225	22	49311047	49315321	ENSMUSG00000022615	ENSMUSG00000022615	ENSMUSG00000022615	15	89199698	89204806	260	301
12226	22	49333328	49335407	ENSMUSG00000061775	ENSMUSG00000061775	ENSMUSG00000061775	15	89214979	89216475	260	301
12227	22	49354156	49363862	ENSMUSG00000022617	ENSMUSG00000022617	ENSMUSG00000022617	15	89244388	89253629	260	301
12228	22	49364260	49368076	ENSMUSG00000022618	ENSMUSG00000022618	ENSMUSG00000022618	15	89254134	89257666	260	301
12229	22	49385997	49396842	ENSMUSG00000022619	ENSMUSG00000022619	ENSMUSG00000022619	15	89281975	89290671	260	301
12230	22	49410316	49413442	ENSMUSG00000022620	ENSMUSG00000022620	ENSMUSG00000022620	15	89300257	89305192	260	301
12231	12	33419627	33484021	ENSMUSG00000063260	ENSMUSG00000063260	ENSMUSG00000063260	15	89610160	89669536	261	302
12232	12	37332259	37585700	ENSMUSG00000052560	ENSMUSG00000052560	ENSMUSG00000052560	15	90315248	90507155	261	302
12233	12	37974490	38123055	ENSMUSG00000022629	ENSMUSG00000022629	ENSMUSG00000022629	15	90762217	90825824	261	302
12234	12	38232814	38300237	ENSMUSG00000055782	ENSMUSG00000055782	ENSMUSG00000055782	15	90973638	91019574	261	302
12235	12	38435090	38785928	ENSMUSG00000036298	ENSMUSG00000036298	ENSMUSG00000036298	15	91095464	91401028	261	302
12236	12	38905201	39047834	ENSMUSG00000036273	ENSMUSG00000036273	ENSMUSG00000036273	15	91501048	91643302	261	302
12237	12	39588423	39750361	ENSMUSG00000055022	ENSMUSG00000055022	ENSMUSG00000055022	15	91956368	92168291	261	302
12238	12	39868525	40253959	ENSMUSG00000036218	ENSMUSG00000036218	ENSMUSG00000036218	15	92425548	92599070	261	302
12239	12	40837173	40918317	ENSMUSG00000022634	ENSMUSG00000022634	ENSMUSG00000022634	15	93112073	93165090	261	302
12240	12	40992155	41006199	ENSMUSG00000022635	ENSMUSG00000022635	ENSMUSG00000022635	15	93214293	93226502	261	302
12241	12	41139341	41269745	ENSMUSG00000036158	ENSMUSG00000036158	ENSMUSG00000036158	15	93327294	93424071	261	302
12242	12	42408685	42438856	ENSMUSG00000033356	ENSMUSG00000033356	ENSMUSG00000033356	15	94350820	94371670	261	302
12243	12	42439067	42468164	ENSMUSG00000059883	ENSMUSG00000059883	ENSMUSG00000059883	15	94371840	94396486	261	302
12244	12	42516168	43069807	ENSMUSG00000063296	ENSMUSG00000063296	ENSMUSG00000063296	15	94457365	94924277	261	302
12245	12	43188332	43556405	ENSMUSG00000022454	ENSMUSG00000022454	ENSMUSG00000022454	15	94903410	95356739	261	302
12246	12	43696336	43730977	ENSMUSG00000045608	ENSMUSG00000045608	ENSMUSG00000045608	15	95451597	95482401	261	302
12247	12	43896372	44109361	ENSMUSG00000064210	ENSMUSG00000064210	ENSMUSG00000064210	15	95618877	95802808	261	302
12248	12	44409887	44588086	ENSMUSG00000033237	ENSMUSG00000033237	ENSMUSG00000033237	15	96115729	96230125	261	302
12249	12	44601459	44670615	ENSMUSG00000033228	ENSMUSG00000033228	ENSMUSG00000033228	15	96241690	96288769	261	302
12250	12	44867851	44949045	ENSMUSG00000023169	ENSMUSG00000023169	ENSMUSG00000023169	15	96404032	96470284	261	302
12251	12	45038239	45052824	ENSMUSG00000022462	ENSMUSG00000022462	ENSMUSG00000022462	15	96515426	96527732	261	302
12252	12	45444813	45506006	ENSMUSG00000022464	ENSMUSG00000022464	ENSMUSG00000022464	15	96822857	96883990	261	302
12253	12	45755757	45759915	ENSMUSG00000048218	ENSMUSG00000048218	ENSMUSG00000048218	15	97072178	97075321	261	302
12254	12	45759653	45916706	ENSMUSG00000044250	ENSMUSG00000044250	ENSMUSG00000044250	15	97075141	97213710	261	302
12255	12	46389795	46405617	ENSMUSG00000022468	ENSMUSG00000022468	ENSMUSG00000022468	15	97539053	97549184	261	302
12256	12	46417307	46439128	ENSMUSG00000022469	ENSMUSG00000022469	ENSMUSG00000022469	15	97572987	97595706	261	302
12257	12	46463891	46499835	ENSMUSG00000022475	ENSMUSG00000022475	ENSMUSG00000022475	15	97621677	97639667	261	302
12258	12	46521589	46585081	ENSMUSG00000022479	ENSMUSG00000022479	ENSMUSG00000022479	15	97682461	97736330	261	302
12259	12	46643670	46648901	ENSMUSG00000052369	ENSMUSG00000052369	ENSMUSG00000052369	15	97792313	97798309	261	302
12260	12	46653018	46684528	ENSMUSG00000022483	ENSMUSG00000022483	ENSMUSG00000022483	15	97803642	97832691	261	302
12261	12	46723275	46786042	ENSMUSG00000033075	ENSMUSG00000033075	ENSMUSG00000033075	15	97870924	97913138	261	302
12262	12	46785973	46826154	ENSMUSG00000033065	ENSMUSG00000033065	ENSMUSG00000033065	15	97936598	97960480	261	302
12263	12	46827841	46837644	ENSMUSG00000048175	ENSMUSG00000048175	ENSMUSG00000048175	15	97962674	97973678	261	302
12264	12	46863666	46865976	ENSMUSG00000029875	ENSMUSG00000029875	ENSMUSG00000029875	15	97995694	97998168	261	302
12265	12	47020178	47030821	ENSMUSG00000022987	ENSMUSG00000022987	ENSMUSG00000022987	15	98113622	98124164	261	302
12266	12	47239947	47243006	ENSMUSG00000052728	ENSMUSG00000052728	ENSMUSG00000052728	15	98299390	98303495	261	302
12267	12	47247735	47250096	ENSMUSG00000022991	ENSMUSG00000022991	ENSMUSG00000022991	15	98308434	98310717	261	302
12268	12	47373019	47397048	ENSMUSG00000011960	ENSMUSG00000011960	ENSMUSG00000011960	15	98371245	98395670	261	302
12269	12	47407485	47445836	ENSMUSG00000022993	ENSMUSG00000022993	ENSMUSG00000022993	15	98399038	98417614	261	302
12270	12	47446248	47464157	ENSMUSG00000022994	ENSMUSG00000022994	ENSMUSG00000022994	15	98418007	98435667	261	302
12271	12	47498779	47508991	ENSMUSG00000003352	ENSMUSG00000003352	ENSMUSG00000003352	15	98463140	98472563	261	302
12272	12	47509807	47532224	ENSMUSG00000003360	ENSMUSG00000003360	ENSMUSG00000003360	15	98473831	98486469	261	302
12273	12	47537198	47545948	ENSMUSG00000054855	ENSMUSG00000054855	ENSMUSG00000054855	15	98497239	98505495	261	302
12274	12	47584187	47601624	ENSMUSG00000003354	ENSMUSG00000003354	ENSMUSG00000003354	15	98536228	98551360	261	302
12275	12	47602034	47605560	ENSMUSG00000003355	ENSMUSG00000003355	ENSMUSG00000003355	15	98552400	98556232	261	302
12276	12	47645455	47651548	ENSMUSG00000022996	ENSMUSG00000022996	ENSMUSG00000022996	15	98599796	98606184	261	302
12277	12	47658503	47662746	ENSMUSG00000022997	ENSMUSG00000022997	ENSMUSG00000022997	15	98617891	98621868	261	302
12278	12	47676790	47679460	ENSMUSG00000059213	ENSMUSG00000059213	ENSMUSG00000059213	15	98631816	98635834	261	302
12279	12	47701830	47735374	ENSMUSG00000048154	ENSMUSG00000048154	ENSMUSG00000048154	15	98662537	98692840	261	302
12280	12	47744735	47750075	ENSMUSG00000023755	ENSMUSG00000023755	ENSMUSG00000023755	15	98705812	98709440	261	302
12281	12	47769475	47774869	ENSMUSG00000023000	ENSMUSG00000023000	ENSMUSG00000023000	15	98719189	98726555	261	302
12282	12	47777208	47790914	ENSMUSG00000022999	ENSMUSG00000022999	ENSMUSG00000022999	15	98731955	98746132	261	302

12283	12	47864847	47869153	ENSMUSG00000072235	ENSMUSG00000072235	ENSMUSG00000072235	15	98778068	98779725	261	302		
12284	12	47945087	47953376	ENSMUSG00000043091	ENSMUSG00000043091	ENSMUSG00000043091	15	98862128	98866043	261	302		
12285	12	47973752	47978732	ENSMUSG00000023484	ENSMUSG00000023484	ENSMUSG00000023484	15	98883215	98887000	261	302		
12286	12	48003329	48011781	ENSMUSG00000032783	ENSMUSG00000032783	ENSMUSG00000032783	15	98903012	98911441	261	302		
12287	12	48012467	48017238	ENSMUSG00000001076	ENSMUSG00000001076	ENSMUSG00000001076	15	98912788	98915762	261	302		
12288	12	48026967	48031777	ENSMUSG00000038009	ENSMUSG00000038009	ENSMUSG00000038009	15	98927518	98932753	261	302		
12289	12	48047584	48207443	ENSMUSG00000051934	ENSMUSG00000051934	ENSMUSG00000051934	15	98954612	99041240	261	302		
12290	12	48219207	48238326	ENSMUSG00000037579	ENSMUSG00000037579	ENSMUSG00000037579	15	99053010	99070851	261	302		
12291	12	48310594	48324716	ENSMUSG00000023007	ENSMUSG00000023007	ENSMUSG00000023007	15	99123273	99145050	261	302		
12292	12	48317991	48387464	ENSMUSG00000023008	ENSMUSG00000023008	ENSMUSG00000023008	15	99145259	99198419	261	302		
12293	12	48421607	48444984	ENSMUSG00000023010	ENSMUSG00000023010	ENSMUSG00000023010	15	99229642	99235377	261	302		
12294	12	48471196	48477052	ENSMUSG00000023009	ENSMUSG00000023009	ENSMUSG00000023009	15	99250068	99285782	261	302		
12295	12	48517827	48523142	ENSMUSG00000037525	ENSMUSG00000037525	ENSMUSG00000037525	15	99298118	99302759	261	302		
12296	12	48546947	48583987	ENSMUSG00000023011	ENSMUSG00000023011	ENSMUSG00000023011	15	99325039	99353082	261	302		
12297	12	48630796	48638926	ENSMUSG00000023013	ENSMUSG00000023013	ENSMUSG00000023013	15	99407090	99412579	261	302		
12298	12	48641920	48645701	ENSMUSG00000044217	ENSMUSG00000044217	ENSMUSG00000044217	15	99418814	99422858	261	302		
12299	12	48647244	48655721	ENSMUSG00000043144	ENSMUSG00000043144	ENSMUSG00000043144	15	99429434	99433511	261	302		
12300	12	48669214	48705493	ENSMUSG00000023015	ENSMUSG00000023015	ENSMUSG00000023015	15	99448531	99479665	261	302		
12301	12	48738817	48762777	ENSMUSG00000023017	ENSMUSG00000023017	ENSMUSG00000023017	15	99498752	99529162	261	302		
12302	12	48765402	48780690	ENSMUSG00000023018	ENSMUSG00000023018	ENSMUSG00000023018	15	99530351	99542024	261	302		
12303	12	48784068	48791358	ENSMUSG00000023019	ENSMUSG00000023019	ENSMUSG00000023019	15	99545621	99553037	261	302		
12304	12	48792167	48800501	ENSMUSG00000023020	ENSMUSG00000023020	ENSMUSG00000023020	15	99553681	99556148	261	302		
12305	12	48809849	48847389	ENSMUSG00000023021	ENSMUSG00000023021	ENSMUSG00000023021	15	99562917	99600496	261	302		
12306	12	48855840	48963539	ENSMUSG00000023022	ENSMUSG00000023022	ENSMUSG00000023022	15	99607455	99703439	261	302		
12307	12	49010521	49011835	ENSMUSG00000045350	ENSMUSG00000045350	ENSMUSG00000045350	15	99755730	99757976	261	302		
12308	12	49033694	49076672	ENSMUSG00000075413	ENSMUSG00000075413	ENSMUSG00000075413	15	99773756	99794981	261	302		
12309	12	49090176	49160046	ENSMUSG00000023025	ENSMUSG00000023025	ENSMUSG00000023025	15	99809573	99840201	261	302		
12310	12	49185035	49428717	ENSMUSG00000023026	ENSMUSG00000023026	ENSMUSG00000023026	15	99866844	100043700	261	302		
12311	12	49523015	49567928	ENSMUSG00000045631	ENSMUSG00000045631	ENSMUSG00000045631	15	100108917	100120795	261	302		
12312	12	49659585	49706409	ENSMUSG00000023030	ENSMUSG00000023030	ENSMUSG00000023030	15	100215936	100251059	261	302		
12313	12	49728351	49740474	ENSMUSG00000037353	ENSMUSG00000037353	ENSMUSG00000037353	15	100297128	100307198	261	302		
12314	12	49741257	49763600	ENSMUSG00000044636	ENSMUSG00000044636	ENSMUSG00000044636	15	100307604	100323273	261	302		
12315	12	49870300	49886887	ENSMUSG00000009739	ENSMUSG00000009739	ENSMUSG00000009739	15	100406281	100423068	261	302		
12316	12	50008494	50026730	ENSMUSG00000023031	ENSMUSG00000023031	ENSMUSG00000023031	15	100503340	100515927	261	302		
12317	12	50033954	50071464	ENSMUSG00000037280	ENSMUSG00000037280	ENSMUSG00000037280	15	100519848	100557410	261	302		
12318	12	50071399	50177915	ENSMUSG00000023032	ENSMUSG00000023032	ENSMUSG00000023032	15	100589781	100644023	261	302		
12319	12	50364210	50488365	ENSMUSG00000023033	ENSMUSG00000023033	ENSMUSG00000023033	15	100783432	100868722	261	302		
12320	12	50500506	50502482	ENSMUSG00000047202	ENSMUSG00000047202	ENSMUSG00000047202	15	100880499	100882460	261	302		
12321	12	50568011	50571705	ENSMUSG00000047034	ENSMUSG00000047034	ENSMUSG00000047034	15	100943789	100948062	261	302		
12322	12	50587469	50601120	ENSMUSG00000000530	ENSMUSG00000000530	ENSMUSG00000000530	15	100956556	100971674	261	302		
12323	12	50494095	50677124	ENSMUSG00000000532	ENSMUSG00000000532	ENSMUSG00000000532	15	101002159	101040635	261	302		
12324	12	50686991	50695915	ENSMUSG00000000531	ENSMUSG00000000531	ENSMUSG00000000531	15	101052243	101060790	261	302		
12325	12	50717766	50739552	ENSMUSG00000023034	ENSMUSG00000023034	ENSMUSG00000023034	15	101094887	101102826	261	302		
12326	12	50750027	50757534	ENSMUSG00000037204	ENSMUSG00000037204	ENSMUSG00000037204	15	101112335	101118968	261	302		
12327	12	50759831	50760100	ENSMUSG00000075408	ENSMUSG00000075408	ENSMUSG00000075408	15	101121246	101124624	261	302		
12328	12	50913348	50928969	ENSMUSG00000023039	ENSMUSG00000023039	ENSMUSG00000023039	15	101240436	101255827	261	302		
12329	12	51057299	51065684	ENSMUSG00000044294	ENSMUSG00000044294	ENSMUSG00000044294	15	101353057	101360810	261	302		
12330	12	51074002	51086443	ENSMUSG00000049548	ENSMUSG00000049548	ENSMUSG00000049548	15	101369248	101378701	261	302		
12331	12	51104121	51114373	ENSMUSG00000022986	ENSMUSG00000022986	ENSMUSG00000022986	15	101391377	101401938	261	302		
12332	12	51194628	51200347	ENSMUSG00000061527	ENSMUSG00000061527	ENSMUSG00000061527	15	101535104	101540925	261	302		
12333	12	51223961	51233128	ENSMUSG00000051879	ENSMUSG00000051879	ENSMUSG00000051879	15	101561984	101571143	261	302		
12334	12	51245870	51253876	ENSMUSG00000067596	ENSMUSG00000067596	ENSMUSG00000067596	15	101582386	101591471	261	302		
12335	12	51265640	51281559	ENSMUSG00000056605	ENSMUSG00000056605	ENSMUSG00000056605	15	101604594	101614492	261	302		
12336	12	51287622	51298610	ENSMUSG00000063661	ENSMUSG00000063661	ENSMUSG00000063661	15	101621342	101630366	261	302		
12337	12	51324610	51332226	ENSMUSG00000064201	ENSMUSG00000064201	ENSMUSG00000064201	15	101638733	101646169	261	302		
12338	12	51354719	51360446	ENSMUSG00000046834	ENSMUSG00000046834	ENSMUSG00000046834	15	101673462	101678820	261	302		
12339	12	51369910	51383514	ENSMUSG00000067594	ENSMUSG00000067594	ENSMUSG00000067594	15	101687890	101697652	261	302		
12340	12	51448207	51457372	ENSMUSG00000075402	ENSMUSG00000075402	ENSMUSG00000075402	15	101712385	101720954	261	302		
12341	12	51486601	51494109	ENSMUSG00000059668	ENSMUSG00000059668	ENSMUSG00000059668	15	101746570	101752704	261	302		
12342	12	51501499	51514346	ENSMUSG00000061397	ENSMUSG00000061397	ENSMUSG00000061397	15	101757366	101768358	261	302		
12343	12	51696514	51720363	ENSMUSG00000058655	ENSMUSG00000058655	ENSMUSG00000058655	15	101901964	101925205	261	302		
12344	12	51729000	51744430	ENSMUSG00000037003	ENSMUSG00000037003	ENSMUSG00000037003	15	101931022	101944427	261	302		
12345	12	51744656	51759437	ENSMUSG00000036966	ENSMUSG00000036966	ENSMUSG00000036966	15	101944846	101964251	261	302		
12346	12	51777703	51782395	ENSMUSG00000023046	ENSMUSG00000023046	ENSMUSG00000023046	15	101972396	101977546	261	302		
12347	12	51783578	51804589	ENSMUSG00000023045	ENSMUSG00000023045	ENSMUSG00000023045	15	101978609	101991489	261	302		
12348	12	51837714	51860647	ENSMUSG00000023044	ENSMUSG00000023044	ENSMUSG00000023044	15	102005032	102017077	261	302		
12349	12	51860799	51868724	ENSMUSG00000046897	ENSMUSG00000046897	ENSMUSG00000046897	15	102035805	102043639	261	302		
12350	12	51871375	51887267	ENSMUSG00000001281	ENSMUSG00000001281	ENSMUSG00000001281	15	102044030	102059969	261	302		
12351	12	51890621	51912253	ENSMUSG00000001288	ENSMUSG00000001288	ENSMUSG00000001288	15	102062974	102085313	261	302		
12352	12	51932147	51934455	ENSMUSG00000045665	ENSMUSG00000045665	ENSMUSG00000045665	15	102107488	102109778	261	302		
12353	12	51948818	51973686	ENSMUSG00000058290	ENSMUSG00000058290	ENSMUSG00000058290	15	102124359	102152390	261	302		
12354	12	51975584	51979497	ENSMUSG00000001289	ENSMUSG00000001289	ENSMUSG00000001289	15	102154190	102159521	261	302		
12355	12	51979749	51987227	ENSMUSG00000001285	ENSMUSG00000001285	ENSMUSG00000001285	15	102159752	102165883	261	302		
12356	12	51987508	52001641	ENSMUSG00000036678	ENSMUSG00000036678	ENSMUSG00000036678	15	102166291	102178767	261	302		
12357	12	52008197	52009489	ENSMUSG00000060284	ENSMUSG00000060284	ENSMUSG00000060284	15	102186171	102187442	261	302		
12358	12	52060246	52096488	ENSMUSG00000001280	ENSMUSG00000001280	ENSMUSG00000001280	15	102234414	102260419	261	302		
12359	12	52103908	52111579	ENSMUSG00000023047	ENSMUSG00000023047	ENSMUSG00000023047	15	102273454	102282664	261	302		
12360	12	52132180	52159883	ENSMUSG00000056851	ENSMUSG00000056851	ENSMUSG00000056851	15	102298819	102326922	261	302		
12361	12	52160547	52179538	ENSMUSG00000023050	ENSMUSG00000023050	ENSMUSG00000023050	15	102325683	102344846	261	302		
12362	12	52180972	52186480	ENSMUSG00000023051	ENSMUSG00000023051	ENSMUSG00000023051	15	102346493	102351708	261	302		
12363	12	52186741	52188271	ENSMUSG00000023052	ENSMUSG00000023052	ENSMUSG00000023052	15	102351873	102352655	261	302		
12364	12	52196111	52306405	ENSMUSG00000052414	ENSMUSG00000052414	ENSMUSG00000052414	15	102362255	102391447	261	302		
12365	12	52391170	52407486	ENSMUSG00000023055	ENSMUSG00000023055	ENSMUSG00000023055	15	102534813	102550123	261	302		
12366	12	52618843	52626594	ENSMUSG00000001655	ENSMUSG00000001655	ENSMUSG00000001655	15	102749165	102756848	261	302		
12367	12	52634981	52636617	ENSMUSG00000050328	ENSMUSG00000050328	ENSMUSG00000050328	15	102764887	102766551	261	302		
12368	12	52653196	52656468	ENSMUSG00000001656	ENSMUSG00000001656	ENSMUSG00000001656	15	102782560	102784735	261	302		
12369	12	52665221	52670327	ENSMUSG00000022484	ENSMUSG00000022484	ENSMUSG00000022484	15	102794826	102799749	261	302		
12370	12	52675560	52683349	ENSMUSG00000036139	ENSMUSG00000036139	ENSMUSG00000036139	15	102805066	102812478	261	302		
12371	12	52689157	52692814	ENSMUSG00000001657	ENSMUSG00000001657	ENSMUSG00000001657	15	102818573	102821404	261	302		
12372	12	52708461	52710865	ENSMUSG00000001661	ENSMUSG00000001661	ENSMUSG00000001661	15	102837640	102839915	261	302		
12373	12	52713099	52715412	ENSMUSG00000022485	ENSMUSG00000022485	ENSMUSG00000022485	15	102842042	102845463	261	302		
12374	12	52733928	52736080	ENSMUSG00000075394	ENSMUSG00000075394	ENSMUSG00000075394	15	102862757	102864026	261	302		
12375	12	52861519	52869024	ENSMUSG00000036061	ENSMUSG00000036061	ENSMUSG00000036061	15	102981341	102985963	261	302		
12376	12	52921003	52960153	ENSMUSG00000009575	ENSMUSG00000009575	ENSMUSG00000009575	15	103024721	103067850	261	302		
12377	12	52972167	52981058	ENSMUSG00000058794	ENSMUSG00000058794	ENSMUSG00000058794	15	103076249	103083450	261	302		
12378	12	53005178	53031900	ENSMUSG00000060992	ENSMUSG00000060992	ENSMUSG00000060992	15	103100933	103127903	261	302		
12379	12	53042498	53044535	ENSMUSG00000063234	ENSMUSG00000063234	ENSMUSG00000063234	15	103136273	103138481	261	302		
12380	12	53049191	53071337	ENSMUSG00000000552	ENSMUSG00000000552	ENSMUSG00000000552	15	103143029	103161242	261	302		
12381	12	53075316	53099317	ENSMUSG00000000555	ENSMUSG00000000555	ENSMUSG00000000555	15	103172320	103194797	261	302		
12382	12	53136012	53153653	ENSMUSG00000022487	ENSMUSG00000022487	ENSMUSG00000022487	15	103230495	103272838	261	302		
12383	12	53177796	53223165	ENSMUSG00000022488	ENSMUSG00000022488	ENSMUSG00000022488	15	103281828	103326835	261	302		
12384	12	53229671	53259290	ENSMUSG00000022489	ENSMUSG00000022489	ENSMUSG00000022489	15	103331332	103358086	261	302		
12385	12	53260992	53268539	ENSMUSG00000022490	ENSMUSG00000022490	ENSMUSG00000022490	15	103359385	103365912	261	302		
12386	16	3194248	3195189	ENSMUSG00000051003	ENSMUSG00000051003	ENSMUSG00000051003	16	3507626	3508567	262	303		
12387	16	3273488	3281456	ENSMUSG00000022529	ENSMUSG00000022529	ENSMUSG00000022529	16	3659331	3666009	262	303		
12388	16	3345942	3346880	ENSMUSG00000059043	ENSMUSG00000059043	ENSMUSG00000059043	16	3754203	3755141	262	303		
12389	16	3391421	3399361	ENSMUSG00000054939	ENSMUSG00000054939	ENSMUSG00000054939	16	3762541	3770034	262	303		
12390	16	3426107	3433498	ENSMUSG00000039789	ENSMUSG00000039789	ENSMUSG00000039789	16	3776772	3787602	262	303		
12391	16	3433736	3474856	ENSMUSG00000005982	ENSMUSG00000005982	ENSMUSG00000005982	16	3799911	3817167	262	303		
12392	16	3491019	3526272	ENSMUSG00000014232	ENSMUSG00000014232	ENSMUSG00000014232	16	3824237	3856375	262	303		
12393	16	3531826	3567290	ENSMUSG00000049871	ENSMUSG00000049871	ENSMUSG00000049871	16	3860838	3880721	262	303		
12394	16	3571187	3601586	ENSMUSG00000039738	ENSMUSG00000039738	ENSMUSG00000039738	16	3895049	3914580	262	303		
12395	16	3630847	3648096	ENSMUSG00000005980	ENSMUSG00000005980	ENSMUSG00000005980	16	3952371	3955247	262	303		
12396	16	3648040	3707521	ENSMUSG00000005981	ENSMUSG00000005981	ENSMUSG00000005981	16	3955205	3993038	262	303		
12397	16	3717720	3870723	ENSMUSG00000022521	ENSMUSG00000022521	ENSMUSG00000022521	16	3999276	4128632	262	303		
12398	16	3952658	4106187	ENSMUSG00000005580	ENSMUSG00000005580	ENSMUSG00000005580	16	4202773	4335034	262	303		
12399	16	4247189	4263000	ENSMUSG00000005718	ENSMUSG00000005718	ENSMUSG00000005718	16	4459889	4474948	262	303		
12400	16	4304763	4329596	ENSMUSG00000014303	ENSMUSG00000014303	ENSMUSG00000014303	16	4509941	4531181	262	303		
12401	16	4344546	4406572	ENSMUSG00000039637	ENSMUSG00000039637	ENSMUSG00000039637	16	4542835	4594839	262	303		
12402	16	4361850	4373527	ENSMUSG00000039646	ENSMUSG00000039646	ENSMUSG00000039646	16	4555188	4566024	262	303		
12403	16	4415883	4446774	ENSMUSG00000004069	ENSMUSG00000004069	ENSMUSG00000004069	16	4599351	4622919	262	303		
12404	16	4451730	4466308	ENSMUSG00000063445	ENSMUSG00000063445	ENSMUSG00000063445	16	4626652	4634340	262	303		
12405	16	4466426	4500348	ENSMUSG00000004070	ENSMUSG00000004070	ENSMUSG00000004070	16	4641617	4681474	262	303		
12406	16	4502414	4528472	ENSMUSG00000004071	ENSMUSG00000004071	ENSMUSG00000004071	16	4680689	4705163	262	303		
12407	16	4598887	4604928	ENSMUSG00000039568	ENSMUSG00000039568	ENSMUSG00000039568	16	4790011	4795046	262	303		
12408	16	4614870	4680975	ENSMUSG00000022517	ENSMUSG00000022517	ENSMUSG00000022517	16	4801480	4853524	262	303		
12409	16	4683720	4685857	ENSMUSG00000022516	ENSMUSG00000022516	ENSMUSG00000022516	16	4854339	4856253	262	303		
12410	16	4686527	4724201	ENSMUSG00000022515	ENSMUSG00000022515	ENSMUSG00000022515	16	4856671	4879505	262	303		
12411	16	4724290	4739396	ENSMUSG00000022543	ENSMUSG00000022543	ENSMUSG00000022543	16	4884187	4893002	262	303		
12412	16	4767675	4778349	ENSMUSG00000022542	ENSMUSG00000022542	ENSMUSG00000022542	16	4902179	4913173	262	303		
12413	16	4787001	4792952	ENSMUSG00000022540	ENSMUSG00000022540	ENSMUSG00000022540	16	4924066	4928840	262	303		
12414	16	4793208	4837294	ENSMUSG00000022536	ENSMUSG00000022536	ENSMUSG00000022536	16	4929258	4965181	262	303		
12415	16	4838138	4872364	ENSMUSG00000039473	ENSMUSG00000039473	ENSMUSG00000039473	16	4970410	5001610	262	303		
12416	16	4872509	4927137	ENSMUSG00000039457	ENSMUSG00000039457	ENSMUSG00000039457	16	5002046	5047742	262	303		
12417	16	4948319	5009154	ENSMUSG00000039447	ENSMUSG00000039447	ENSMUSG00000039447	16	5063098	5099071	262	303		
12418	16	5014848	5023937	ENSMUSG00000023143	ENSMUSG00000023143	ENSMUSG00000023143	16	5110609	5119333	262	303		
12419	16	5034125	5056112	ENSMUSG00000051669	ENSMUSG00000051669	ENSMUSG00000051669	16	5127147	5137601	262	303		
12420	16	5061821	5075588	ENSMUSG00000039427	ENSMUSG00000039427	ENSMUSG00000039427	16	5148957	5160230	262	303		
12421	16	7322752	7702500	ENSMUSG00000008658	ENSMUSG00000008658	ENSMUSG00000008658	16	6985156	7325300	262	303		
12422	16	8627048	8647492	ENSMUSG00000039345	ENSMUSG00000039345	ENSMUSG00000039345	16	8386134	8405518	262	303		
12423	16	8675946	8785932	ENSMUSG00000057880	ENSMUSG00000057880	ENSMUSG00000057880	16	8428810	8536889	262	303		
12424	16	8796825	8798991	ENSMUSG00000043140	ENSMUSG00000043140	ENSMUSG00000043140	16	8549205	8553032	262	303		
12425	16	8799193	8850684	ENSMUSG00000022711	ENSMUSG00000022711	ENSMUSG00000022711	16	8553047	8572870	262	303		
12426	16	8895146	8938044	ENSMUSG00000022710	ENSMUSG00000022710	ENSMUSG00000022710	16	8606101	8672887	262	303		
12427	16	9093038	9121037	ENSMUSG00000022507	ENSMUSG00000022507	ENSMUSG00000022507	16	8745971	8772066	262	303		
12428	16	9764507	10181769	ENSMUSG00000059003	ENSMUSG00000059003	ENSMUSG00000059003	16	9491382	9906111	262	303		
12429	16	10431953	10484995	ENSMUSG00000039200	ENSMUSG00000039200	ENSMUSG00000039200	16	10106500	10155534	262	303		
12430	16	10628862	10696303	ENSMUSG00000039179	ENSMUSG00000039179	ENSMUSG00000039179	16	10271531	10308997	262	303		
12431	16	10745203	10770709	ENSMUSG00000022503	ENSMUSG00000022503	ENSMUSG00000022503	16	10325526	10337996	262	303		
12432	16	10772956	10820009	ENSMUSG00000050908	ENSMUSG00000050908	ENSMUSG00000050908	16	10337777	10360623	262	303		
12433	16	10867648	10924658	ENSMUSG00000022504	ENSMUSG00000022504	ENSMUSG00000022504	16	10393637	10440002	262	303		
12434	16	10945943	11183527	ENSMUSG00000068663	ENSMUSG00000068663	ENSMUSG00000068663	16	10458965	10658449	262	303		
12435	16	11255775	11257540	ENSMUSG00000038037	ENSMUSG00000038037	ENSMUSG00000038037	16	10695821	10699114	262	303		
12436	16	11270219	11270620	ENSMUSG00000043050	ENSMUSG00000043050	ENSMUSG00000043050	16	10701514	10702238	262	303		
12437	16	11274645	11274953	ENSMUSG00000050058	ENSMUSG00000050058	ENSMUSG00000050058	16	10704162	10704467	262	303		
12438	16	11276998	11277838	ENSMUSG00000038015	ENSMUSG00000038015	ENSMUSG00000038015	16	10704961	10705683	262	303		
12439	16	11282199	11282708	ENSMUSG00000022501	ENSMUSG00000022501	ENSMUSG00000022501	16	10709905	10710404	262	303		
12440	16	11549357	11588823	ENSMUSG00000022500	ENSMUSG00000022500	ENSMUSG00000022500	16	10872437	10906698	262	303		
12441	16	11669818	11680507	ENSMUSG00000037972	ENSMUSG00000037972	ENSMUSG00000037972	16	10974523	10986561	262	303		
12442	16	11680446	11744087	ENSMUSG00000022498	ENSMUSG00000022498	ENSMUSG00000022498	16	10988489	11048110	262	303		
12443	16	11751957	11798508	ENSMUSG00000037965	ENSMUSG00000037965	ENSMUSG00000037965	16	11050181	11089867	262	303		
12444	16	11838712	11852896	ENSMUSG00000005846	ENSMUSG00000005846	ENSMUSG00000005846	16	11106615	11116872	262	303		
12445	16	11874040	11917309	ENSMUSG00000062203	ENSMUSG00000062203	ENSMUSG00000062203	16	11133733	11167787	262	303		
12446	16	11966465	11969426	ENSMUSG00000022496	ENSMUSG00000022496	ENSMUSG00000022496	16	11227390	11233650	262	303		
12447	16	12080192	12574366	ENSMUSG00000071669	ENSMUSG00000071669	ENSMUSG00000071669	16	11319226	11669050	262	303		
12448	16	12664438	12805229	ENSMUSG00000065979	ENSMUSG00000065979	ENSMUSG00000065979	16	11717299	11823001	262	303		
12449	16	12903300	12904004	ENSMUSG00000022494	ENSMUSG00000022494	ENSMUSG00000022494	16	11898036	11898722	262	303		
12450	16	13921524	13949705	ENSMUSG00000022545	ENSMUSG00000022545	ENSMUSG00000022545	16	13023314	13062312	262	303		
12451	16	14188273	14268130	ENSMUSG00000009569	ENSMUSG00000009569	ENSMUSG00000009569	16	13272217	13326281	262	303		
12452	16	14438075	14631567	ENSMUSG00000022685	ENSMUSG00000022685	ENSMUSG00000022685	16	13452428	13581193	262	303		
12453	16	14634297	14670193	ENSMUSG00000022684	ENSMUSG00000022684	ENSMUSG00000022684	16	13585572	13616279	262	303		
12454	16	14673908	14696027	ENSMUSG00000022683	ENSMUSG00000022683	ENSMUSG00000022683	16	13628640	13644163	262	303		
12455	16	15039212	15057344	ENSMUSG00000022681	ENSMUSG00000022681	ENSMUSG00000022681	16	13732855	13749028	262	303		
12456	16	15397163	15409749	ENSMUSG00000022679	ENSMUSG00000022679	ENSMUSG00000022679	16	13854273	13862956	262	303		
12457	16	15435826	15589418	ENSMUSG00000044117	ENSMUSG00000044117	ENSMUSG00000044117	16	13900184	14014886	262	303		
12458	16	15595746	15644510	ENSMUSG00000060657	ENSMUSG00000060657	ENSMUSG00000060657	16	14025111	14067553	262	303		
12459	16	15666085	15726490	ENSMUSG00000022678	ENSMUSG00000022678	ENSMUSG00000022678	16	14076884	14106406	262	303		
12460	16	15704495	15858369	ENSMUSG00000018830	ENSMUSG00000018830	ENSMUSG00000018830	16	14108110	14204950	262	303		
12461	16	15867077	15889969	ENSMUSG00000022677	ENSMUSG00000022677	ENSMUSG00000022677	16	14212822	14230910	262	303		
12462	16	16009174	16142883	ENSMUSG00000023088	ENSMUSG00000023088	ENSMUSG00000023088	16	14303385	14387146	262	303		
12463	8	49992802	49996541	ENSMUSG00000022676	ENSMUSG00000022676	ENSMUSG00000022676	16	14619437	14622963	263	304		
12464	8	49798533	49810344	ENSMUSG00000068617	ENSMUSG00000068617	ENSMUSG00000068617	16	14820215	14839457	263	304		
12465	8	49116860	49136683	ENSMUSG00000022674	ENSMUSG00000022674	ENSMUSG00000022674	16	15469975	15494795	263	304		
12466	8	49036047	49052621	ENSMUSG00000022673	ENSMUSG00000022673	ENSMUSG00000022673	16	15537479	15550936	263	304		
12467	8	48848222	49035296	ENSMUSG00000022672	ENSMUSG00000022672	ENSMUSG00000022672	16	15551467	15755813	263	304		
12468	8	48336095	48811026	ENSMUSG00000041974	ENSMUSG00000041974	ENSMUSG00000041974	16	15802805	16060411	263	304		
12469	12	32834954	32940993	ENSMUSG00000041957	ENSMUSG00000041957	ENSMUSG00000041957	16	16126923	16186290	264	305		
12470	12	32791227	32800075	ENSMUSG00000022792	ENSMUSG00000022792	ENSMUSG00000022792	16	16216560	16223206	264	305		
12471	12	32723491	32788621	ENSMUSG00000022789	ENSMUSG00000022789	ENSMUSG00000022789	16	16227565	16272443	264	305		
12472	12	32546361	32684940	ENSMUSG00000022788	ENSMUSG00000022788	ENSMUSG00000022788	16	16335539	16473712	264	305		
12473	22	20641486	20667187	ENSMUSG00000022779	ENSMUSG00000022779	ENSMUSG00000022779	16	16784454	16806555	265	306	here	Blocks from 306 to 310 defined by AG do not have orientation, and so ST made them single block
12474	22	20603793	20637217	ENSMUSG00000026181	ENSMUSG00000026181	ENSMUSG00000026181	16	16816884	16840925	265	306	Blocks without orientation merged with the neighboring block	1 -> 5
12475	22	20446873	20551730	ENSMUSG00000063358	ENSMUSG00000063358	ENSMUSG00000063358	16	16896945	16961016	265	306		
12476	22	20385155	20395033	ENSMUSG00000022773	ENSMUSG00000022773	ENSMUSG00000022773	16	16983867	16996257	265	306		
12477	22	20350329	20382548	ENSMUSG00000022771	ENSMUSG00000022771	ENSMUSG00000022771	16	17001553	17024795	265	306		

12478	22	20326542	20328588	ENSMUSG00000022769	ENSMUSG00000022769	ENSMUSG00000022769	16	17043701	17045928	265	306
12479	22	20317086	20321615	ENSMUSG00000022768	ENSMUSG00000022768	ENSMUSG00000022768	16	17052627	17060792	265	306
12480	22	20312378	20314353	ENSMUSG00000041774	ENSMUSG00000041774	ENSMUSG00000041774	16	17060542	17062406	265	306
12481	22	20251957	20308323	ENSMUSG00000038965	ENSMUSG00000038965	ENSMUSG00000038965	16	17065576	17115055	265	306
12482	22	20101693	20135748	ENSMUSG00000050240	ENSMUSG00000050240	ENSMUSG00000050240	16	17147150	17176993	265	307
12483	22	19458383	19472008	ENSMUSG00000022766	ENSMUSG00000022766	ENSMUSG00000022766	16	17244978	17257137	265	307
12484	22	19543292	19574109	ENSMUSG00000022765	ENSMUSG00000022765	ENSMUSG00000022765	16	17319595	17344389	265	307
12485	22	19601722	19638034	ENSMUSG00000006134	ENSMUSG00000006134	ENSMUSG00000006134	16	17365550	17399818	265	307
12486	22	19652090	19665649	ENSMUSG00000022763	ENSMUSG00000022763	ENSMUSG00000022763	16	17403254	17421046	265	307
12487	22	19666656	19683325	ENSMUSG00000022761	ENSMUSG00000022761	ENSMUSG00000022761	16	17422531	17439894	265	307
12488	22	19684063	19686404	ENSMUSG00000022760	ENSMUSG00000022760	ENSMUSG00000022760	16	17441751	17444420	265	307
12489	22	19699449	19713119	ENSMUSG00000022758	ENSMUSG00000022758	ENSMUSG00000022758	16	17475572	17485574	265	307
12490	22	19713008	19716847	ENSMUSG00000022756	ENSMUSG00000022756	ENSMUSG00000022756	16	17485581	17490234	265	307
12491	22	19191886	19271918	ENSMUSG00000012114	ENSMUSG00000012114	ENSMUSG00000012114	16	17564785	17636386	265	308
12492	22	19125806	19180122	ENSMUSG00000022750	ENSMUSG00000022750	ENSMUSG00000022750	16	17673234	17706765	265	308
12493	22	19108875	19122146	ENSMUSG00000012017	ENSMUSG00000012017	ENSMUSG00000012017	16	17710845	17721850	265	308
12494	22	17403801	17489962	ENSMUSG00000003166	ENSMUSG00000003166	ENSMUSG00000003166	16	17748840	17805234	265	309
12495	22	17497793	17500813	ENSMUSG00000045521	ENSMUSG00000045521	ENSMUSG00000045521	16	17812217	17813587	265	309
12496	22	17500125	17512167	ENSMUSG00000003527	ENSMUSG00000003527	ENSMUSG00000003527	16	17815328	17824904	265	309
12497	22	17516504	17517796	ENSMUSG00000022738	ENSMUSG00000022738	ENSMUSG00000022738	16	17826677	17828566	265	309
12498	22	17543092	17546260	ENSMUSG00000003528	ENSMUSG00000003528	ENSMUSG00000003528	16	17838786	17841736	265	309
12499	22	18608940	18635816	ENSMUSG00000043811	ENSMUSG00000043811	ENSMUSG00000043811	16	18064294	18065694	265	309
12500	22	18499471	18515529	ENSMUSG00000060166	ENSMUSG00000060166	ENSMUSG00000060166	16	18134316	18148699	265	310
12501	22	18485024	18494704	ENSMUSG00000005732	ENSMUSG00000005732	ENSMUSG00000005732	16	18153650	18161000	265	310
12502	22	18479398	18484768	ENSMUSG00000022721	ENSMUSG00000022721	ENSMUSG00000022721	16	18162841	18167061	265	310
12503	22	18447814	18479391	ENSMUSG00000022718	ENSMUSG00000022718	ENSMUSG00000022718	16	18167513	18202751	265	310
12504	22	18388631	18433447	ENSMUSG00000013539	ENSMUSG00000013539	ENSMUSG00000013539	16	18214390	18257459	265	310
12505	22	18337421	18384309	ENSMUSG00000000325	ENSMUSG00000000325	ENSMUSG00000000325	16	18261837	18320639	265	310
12506	22	18309334	18337450	ENSMUSG00000000326	ENSMUSG00000000326	ENSMUSG00000000326	16	18321400	18325520	265	310
12507	22	18243047	18309515	ENSMUSG00000075704	ENSMUSG00000075704	ENSMUSG00000075704	16	18340017	18391343	265	310
12508	22	18124226	18134855	ENSMUSG00000009097	ENSMUSG00000009097	ENSMUSG00000009097	16	18495276	18500532	265	310
12509	22	18085897	18090007	ENSMUSG00000072214	ENSMUSG00000072214	ENSMUSG00000072214	16	18535374	18543501	265	310
12510	22	17890550	17895068	ENSMUSG00000041378	ENSMUSG00000041378	ENSMUSG00000041378	16	18690410	18691823	265	310
12511	22	17847416	17888134	ENSMUSG00000000028	ENSMUSG00000000028	ENSMUSG00000000028	16	18694015	18725535	265	310
12512	22	17817464	17846693	ENSMUSG00000005262	ENSMUSG00000005262	ENSMUSG00000005262	16	18725889	18748818	265	310
12513	22	17809056	17815215	ENSMUSG00000071632	ENSMUSG00000071632	ENSMUSG00000071632	16	18750154	18753506	265	310
12514	22	17799425	17803594	ENSMUSG00000022706	ENSMUSG00000022706	ENSMUSG00000022706	16	18785780	18790200	265	310
12515	22	17698224	17799219	ENSMUSG00000022702	ENSMUSG00000022702	ENSMUSG00000022702	16	18790313	18883872	265	310
12516	3	184322698	184363317	ENSMUSG00000041247	ENSMUSG00000041247	ENSMUSG00000041247	16	19566949	19619879	266	311
12517	3	184453739	184473872	ENSMUSG00000022686	ENSMUSG00000022686	ENSMUSG00000022686	16	19673797	19685863	266	311
12518	3	184691931	184756147	ENSMUSG00000043008	ENSMUSG00000043008	ENSMUSG00000043008	16	19860063	19896579	266	311
12519	3	184836105	184884996	ENSMUSG00000062901	ENSMUSG00000062901	ENSMUSG00000062901	16	20011097	20041287	266	311
12520	3	184898300	185013102	ENSMUSG00000041215	ENSMUSG00000041215	ENSMUSG00000041215	16	20054651	20146116	266	311
12521	3	185016364	185026059	ENSMUSG00000041205	ENSMUSG00000041205	ENSMUSG00000041205	16	20148791	20154863	266	311
12522	3	185029870	185085362	ENSMUSG00000033918	ENSMUSG00000033918	ENSMUSG00000033918	16	20193361	20215905	266	311
12523	3	185120420	185218421	ENSMUSG00000022822	ENSMUSG00000022822	ENSMUSG00000022822	16	20244846	20339937	266	311
12524	3	185335520	185345792	ENSMUSG00000003235	ENSMUSG00000003235	ENSMUSG00000003235	16	20412360	20422866	266	311
12525	3	185355978	185374092	ENSMUSG00000003233	ENSMUSG00000003233	ENSMUSG00000003233	16	20430525	20445059	266	311
12526	3	185375328	185384572	ENSMUSG00000022841	ENSMUSG00000022841	ENSMUSG00000022841	16	20449047	20457598	266	311
12527	3	185386580	185394487	ENSMUSG00000003234	ENSMUSG00000003234	ENSMUSG00000003234	16	20462183	20474922	266	311
12528	3	185476517	185492832	ENSMUSG00000022842	ENSMUSG00000022842	ENSMUSG00000022842	16	20525337	20559458	266	311
12529	3	185459697	185461945	ENSMUSG00000051146	ENSMUSG00000051146	ENSMUSG00000051146	16	20532764	20534768	266	311
12530	3	185515890	185535833	ENSMUSG00000045983	ENSMUSG00000045983	ENSMUSG00000045983	16	20586292	20606426	266	311
12531	3	185540952	185546759	ENSMUSG00000050821	ENSMUSG00000050821	ENSMUSG00000050821	16	20608569	20616576	266	311
12532	3	185547088	185561961	ENSMUSG00000022843	ENSMUSG00000022843	ENSMUSG00000022843	16	20616507	20629657	266	311
12533	3	185562254	185568989	ENSMUSG00000021018	ENSMUSG00000021018	ENSMUSG00000021018	16	20631460	20635808	266	311
12534	3	185572995	185578626	ENSMUSG00000022847	ENSMUSG00000022847	ENSMUSG00000022847	16	20638951	20642402	266	311
12535	3	185580555	185590311	ENSMUSG00000006958	ENSMUSG00000006958	ENSMUSG00000006958	16	20646670	20655925	266	311
12536	3	185762281	185782889	ENSMUSG00000005958	ENSMUSG00000005958	ENSMUSG00000005958	16	21118777	21137286	266	311
12537	3	186012653	186253086	ENSMUSG00000033653	ENSMUSG00000033653	ENSMUSG00000033653	16	21337152	21558658	266	311
12538	3	186278532	186353496	ENSMUSG00000043391	ENSMUSG00000043391	ENSMUSG00000043391	16	21563023	21608597	266	311
12539	3	186391108	186454531	ENSMUSG00000022853	ENSMUSG00000022853	ENSMUSG00000022853	16	21675270	21701786	266	311
12540	3	186691495	186699516	ENSMUSG00000022856	ENSMUSG00000022856	ENSMUSG00000022856	16	21848310	21860209	266	311
12541	3	186708282	186753063	ENSMUSG00000044626	ENSMUSG00000044626	ENSMUSG00000044626	16	21868757	21895160	266	311
12542	3	186786725	186831577	ENSMUSG00000022855	ENSMUSG00000022855	ENSMUSG00000022855	16	21924664	21963134	266	311
12543	3	186844228	187025521	ENSMUSG00000033581	ENSMUSG00000033581	ENSMUSG00000033581	16	21974311	22078275	266	311
12544	3	187249122	187306351	ENSMUSG00000013089	ENSMUSG00000013089	ENSMUSG00000013089	16	22296570	22354874	266	311
12545	3	187349706	187562697	ENSMUSG00000022861	ENSMUSG00000022861	ENSMUSG00000022861	16	22383717	22572429	266	311
12546	3	187746824	187767823	ENSMUSG00000004462	ENSMUSG00000004462	ENSMUSG00000004462	16	22728549	22772872	266	311
12547	3	187767886	187786282	ENSMUSG00000004460	ENSMUSG00000004460	ENSMUSG00000004460	16	22773198	22787702	266	311
12548	3	187813549	187821786	ENSMUSG00000022868	ENSMUSG00000022868	ENSMUSG00000022868	16	22807376	22814772	266	311
12549	3	187840890	187853468	ENSMUSG00000022871	ENSMUSG00000022871	ENSMUSG00000022871	16	22835571	22855085	266	311
12550	3	187866487	187878718	ENSMUSG00000022877	ENSMUSG00000022877	ENSMUSG00000022877	16	22866448	22876990	266	311
12551	3	187990378	188006984	ENSMUSG00000022881	ENSMUSG00000022881	ENSMUSG00000022881	16	23029267	23043064	266	311
12552	3	188043157	188058944	ENSMUSG00000022878	ENSMUSG00000022878	ENSMUSG00000022878	16	23061870	23073302	266	311
12553	3	188397968	188401943	ENSMUSG00000033383	ENSMUSG00000033383	ENSMUSG00000033383	16	23344467	23349294	266	311
12554	3	188434568	188492446	ENSMUSG00000022887	ENSMUSG00000022887	ENSMUSG00000022887	16	23385181	23429417	266	311
12555	3	188568862	188572061	ENSMUSG00000033355	ENSMUSG00000033355	ENSMUSG00000033355	16	23525276	23529566	266	311
12556	3	188869399	188870895	ENSMUSG00000004366	ENSMUSG00000004366	ENSMUSG00000004366	16	23804930	23806193	266	311
12557	3	188898741	188903039	ENSMUSG00000047531	ENSMUSG00000047531	ENSMUSG00000047531	16	23840897	23846143	266	311
12558	3	188921859	188946169	ENSMUSG00000022508	ENSMUSG00000022508	ENSMUSG00000022508	16	23880401	23903982	266	311
12559	3	189606603	190074961	ENSMUSG00000033306	ENSMUSG00000033306	ENSMUSG00000033306	16	24308839	24896379	266	311
12560	3	190372457	190523964	ENSMUSG00000048399	ENSMUSG00000048399	ENSMUSG00000048399	16	25202166	25337693	266	311
12561	3	190831910	191097731	ENSMUSG00000022510	ENSMUSG00000022510	ENSMUSG00000022510	16	25599639	25804653	266	311
12562	3	191157213	191321407	ENSMUSG00000038168	ENSMUSG00000038168	ENSMUSG00000038168	16	25875672	26021133	266	311
12563	3	191506197	191522909	ENSMUSG00000022512	ENSMUSG00000022512	ENSMUSG00000022512	16	26272000	26287188	266	311
12564	3	191588535	191611027	ENSMUSG00000038148	ENSMUSG00000038148	ENSMUSG00000038148	16	26378509	26398125	266	311
12565	3	191714585	191851995	ENSMUSG00000022514	ENSMUSG00000022514	ENSMUSG00000022514	16	26497062	26640497	266	311
12566	3	192053362	192061350	ENSMUSG00000068428	ENSMUSG00000068428	ENSMUSG00000068428	16	26875487	26880986	266	311
12567	3	192413016	192450604	ENSMUSG00000052276	ENSMUSG00000052276	ENSMUSG00000052276	16	27236572	27262188	266	311
12568	3	192468042	192482734	ENSMUSG00000056423	ENSMUSG00000056423	ENSMUSG00000056423	16	27268544	27285472	266	311
12569	3	193342992	193609532	ENSMUSG00000022523	ENSMUSG00000022523	ENSMUSG00000022523	16	28075353	28360460	266	311
12570	3	193997301	194118644	ENSMUSG00000051065	ENSMUSG00000051065	ENSMUSG00000051065	16	28745923	28849445	266	311
12571	3	194441608	194471337	ENSMUSG00000022525	ENSMUSG00000022525	ENSMUSG00000022525	16	29137359	29147981	266	311
12572	3	194475525	194579208	ENSMUSG00000048939	ENSMUSG00000048939	ENSMUSG00000048939	16	29151661	29298453	266	311
12573	3	194602563	194755390	ENSMUSG00000038094	ENSMUSG00000038094	ENSMUSG00000038094	16	29315788	29461230	266	311
12574	3	194793806	194898009	ENSMUSG00000038084	ENSMUSG00000038084	ENSMUSG00000038084	16	29499089	29572237	266	311
12575	3	195336628	195339066	ENSMUSG00000022528	ENSMUSG00000022528	ENSMUSG00000022528	16	29985104	29987543	266	311
12576	3	195542190	195553306	ENSMUSG00000023176	ENSMUSG00000023176	ENSMUSG00000023176	16	30176128	30187246	266	311
12577	3	195557274	195571761	ENSMUSG00000052316	ENSMUSG00000052316	ENSMUSG00000052316	16	30189051	30202972	266	311
12578	3	195598618	195600300	ENSMUSG00000047953	ENSMUSG00000047953	ENSMUSG00000047953	16	30227440	30230526	266	311
12579	3	195607790	195664208	ENSMUSG00000022533	ENSMUSG00000022533	ENSMUSG00000022533	16	30235451	30286632	266	311
12580	3	195789692	195835402	ENSMUSG00000022537	ENSMUSG00000022537	ENSMUSG00000022537	16	30431604	30470156	266	311
12581	3	195842814	195874209	ENSMUSG00000022538	ENSMUSG00000022538	ENSMUSG00000022538	16	30481116	30507336	266	311
12582	3	195887911	195891051	ENSMUSG00000046546	ENSMUSG00000046546	ENSMUSG00000046546	16	30519470	30522502	266	311
12583	3	196270619	196473152	ENSMUSG00000047434	ENSMUSG00000047434	ENSMUSG00000047434	16	30875372	31001127	266	311
12584	3	196476768	196645041	ENSMUSG00000049076	ENSMUSG00000049076	ENSMUSG00000049076	16	31016093	31080137	266	311
12585	3	196776867	196792278	ENSMUSG00000022548	ENSMUSG00000022548	ENSMUSG00000022548	16	31215939	31234343	266	311
12586	3	198721051	198784591	ENSMUSG00000046598	ENSMUSG00000046598	ENSMUSG00000046598	16	31342041	31377244	267	312
12587	3	198255820	198509844	ENSMUSG00000022770	ENSMUSG00000022770	ENSMUSG00000022770	16	31583786	31793093	267	312
12588	3	198214553	198241039	ENSMUSG00000022780	ENSMUSG00000022780	ENSMUSG00000022780	16	31798557	31818767	267	312
12589	3	198157612	198180101	ENSMUSG00000045625	ENSMUSG00000045625	ENSMUSG00000045625	16	31853701	31865793	267	312
12590	3	198079174	198142480	ENSMUSG00000022772	ENSMUSG00000022772	ENSMUSG00000022772	16	31882255	31922910	267	312
12591	3	197951312	198043749	ENSMUSG00000022781	ENSMUSG00000022781	ENSMUSG00000022781	16	31937610	31999020	267	312
12592	3	197923682	197946161	ENSMUSG00000023791	ENSMUSG00000023791	ENSMUSG00000023791	16	32004233	32016796	267	312
12593	3	197918318	197923491	ENSMUSG00000035790	ENSMUSG00000035790	ENSMUSG00000035790	16	32023625	32027802	267	312
12594	3	197851046	197873269	ENSMUSG00000052384	ENSMUSG00000052384	ENSMUSG00000052384	16	32062572	32085223	267	312
12595	3	197780253	197798553	ENSMUSG00000035764	ENSMUSG00000035764	ENSMUSG00000035764	16	32149861	32166849	267	312
12596	3	197683026	197714979	ENSMUSG00000014074	ENSMUSG00000014074	ENSMUSG00000014074	16	32197236	32220653	267	312
12597	3	197564768	197643670	ENSMUSG00000053774	ENSMUSG00000053774	ENSMUSG00000053774	16	32252015	32306502	267	312
12598	3	197449652	197498981	ENSMUSG00000005615	ENSMUSG00000005615	ENSMUSG00000005615	16	32350743	32391762	267	312
12599	3	197427780	197444696	ENSMUSG00000035699	ENSMUSG00000035699	ENSMUSG00000035699	16	32395325	32407626	267	312
12600	3	197408722	197422697	ENSMUSG00000052363	ENSMUSG00000052363	ENSMUSG00000052363	16	32416028	32426964	267	312
12601	3	197260553	197293343	ENSMUSG00000022797	ENSMUSG00000022797	ENSMUSG00000022797	16	32528764	32552537	267	312
12602	3	197074633	197106829	ENSMUSG00000022791	ENSMUSG00000022791	ENSMUSG00000022791	16	32583504	32601030	267	312
12603	3	196933520	196946094	ENSMUSG00000035638	ENSMUSG00000035638	ENSMUSG00000035638	16	32705787	32717181	267	312
12604	3	198886289	198947987	ENSMUSG00000035629	ENSMUSG00000035629	ENSMUSG00000035629	16	32741454	32788086	268	313
12605	3	198961018	198995573	ENSMUSG00000022800	ENSMUSG00000022800	ENSMUSG00000022800	16	32797587	32828622	268	313
12606	3	199002542	199082851	ENSMUSG00000022801	ENSMUSG00000022801	ENSMUSG00000022801	16	32870071	32932237	268	313
12607	3	199100346	199171271	ENSMUSG00000035578	ENSMUSG00000035578	ENSMUSG00000035578	16	32936685	32969862	268	313
12608	3	199171468	199249965	ENSMUSG00000022802	ENSMUSG00000022802	ENSMUSG00000022802	16	32982268	33045406	268	313
12609	3	126730392	126796624	ENSMUSG00000022807	ENSMUSG00000022807	ENSMUSG00000022807	16	33104818	33163013	269	314
12610	3	126649166	126721706	ENSMUSG00000022808	ENSMUSG00000022808	ENSMUSG00000022808	16	33171191	33220016	269	314
12611	3	126433609	126576785	ENSMUSG00000022811	ENSMUSG00000022811	ENSMUSG00000022811	16	33300583	33420121	269	314
12612	3	126106979	126136270	ENSMUSG00000022824	ENSMUSG00000022824	ENSMUSG00000022824	16	33714274	33735616	269	314
12613	3	125964488	126088842	ENSMUSG00000022817	ENSMUSG00000022817	ENSMUSG00000022817	16	33749412	33868892	269	314
12614	3	125931903	125946318	ENSMUSG00000022814	ENSMUSG00000022814	ENSMUSG00000022814	16	33874759	33886750	269	314
12615	3	125296275	125922726	ENSMUSG00000061751	ENSMUSG00000061751	ENSMUSG00000061751	16	33895284	34305208	269	314
12616	3	125115258	125158620	ENSMUSG00000022833	ENSMUSG00000022833	ENSMUSG00000022833	16	34610479	34644941	269	314
12617	3	124813835	125085839	ENSMUSG00000022836	ENSMUSG00000022836	ENSMUSG00000022836	16	34735182	34920279	269	314
12618	3	124693087	124786722	ENSMUSG00000035376	ENSMUSG00000035376	ENSMUSG00000035376	16	34942168	35028922	269	314
12619	3	124486089	124650082	ENSMUSG00000022840	ENSMUSG00000022840	ENSMUSG00000022840	16	35074624	35224118	269	314
12620	3	124403499	124474043	ENSMUSG00000034473	ENSMUSG00000034473	ENSMUSG00000034473	16	35230878	35283665	269	314
12621	3	124110730	124229115	ENSMUSG00000052133	ENSMUSG00000052133	ENSMUSG00000052133	16	35461109	35584005	269	314
12622	3	123996332	124081446	ENSMUSG00000022848	ENSMUSG00000022848	ENSMUSG00000022848	16	35614650	35689103	269	314
12623	3	123941536	123995340	ENSMUSG00000022849	ENSMUSG00000022849	ENSMUSG00000022849	16	35690154	35747773	269	314
12624	3	123893960	123930263	ENSMUSG00000034422	ENSMUSG00000034422	ENSMUSG00000034422	16	35752621	35791247	269	314
12625	3	123765875	123776734	ENSMUSG00000049502	ENSMUSG00000049502	ENSMUSG00000049502	16	35846258	35858670	269	314
12626	3	123729463	123766114	ENSMUSG00000022906	ENSMUSG00000022906	ENSMUSG00000022906	16	35863116	35892352	269	314
12627	3	123627536	123716447	ENSMUSG00000022905	ENSMUSG00000022905	ENSMUSG00000022905	16	35903110	35955909	269	314
12628	3	123616073	123617065	ENSMUSG00000034379	ENSMUSG00000034379	ENSMUSG00000034379	16	35960937	35962732	269	314
12629	3	123561126	123584764	ENSMUSG00000075229	ENSMUSG00000075229	ENSMUSG00000075229	16	35991432	36011413	269	314
12630	3	123385871	123488032	ENSMUSG00000051980	ENSMUSG00000051980	ENSMUSG00000051980	16	36412933	36481373	269	314
12631	3	123256911	123322672	ENSMUSG00000022901	ENSMUSG00000022901	ENSMUSG00000022901	16	36523108	36580691	269	314
12632	3	123188861	123223720	ENSMUSG00000022900	ENSMUSG00000022900	ENSMUSG00000022900	16	36613289	36644936	269	314
12633	3	123095977	123143147	ENSMUSG00000022899	ENSMUSG00000022899	ENSMUSG00000022899	16	36669420	36704201	269	314
12634	3	123036724	123088062	ENSMUSG00000022838	ENSMUSG00000022838	ENSMUSG00000022838	16	36713119	36747487	269	314
12635	3	122971300	123036563	ENSMUSG00000022837	ENSMUSG00000022837	ENSMUSG00000022837	16	36747647	36791952	269	314
12636	3	122865627	122951292	ENSMUSG00000034243	ENSMUSG00000034243	ENSMUSG00000034243	16	36805375	36851193	269	314
12637	3	122832937	122862405	ENSMUSG00000022831	ENSMUSG00000022831	ENSMUSG00000022831	16	36856526	36881988	269	314
12638	3	122794656	122831829	ENSMUSG00000047746	ENSMUSG00000047746	ENSMUSG00000047746	16	36882699	36889982	269	314
12639	3	122632968	122747543	ENSMUSG00000034206	ENSMUSG00000034206	ENSMUSG00000034206	16	36931025	37014636	269	314
12640	3	122109773	122621336	ENSMUSG00000022829	ENSMUSG00000022829	ENSMUSG00000022829	16	37034895	37302944	269	314
12641	3	121888765	121944074	ENSMUSG00000022827	ENSMUSG00000022827	ENSMUSG00000022827	16	37459139	37491621	269	314
12642	3	121829709	121883815	ENSMUSG00000022821	ENSMUSG00000022821	ENSMUSG00000022821	16	37507930	37551064	269	314
12643	3	121595817	121652533	ENSMUSG00000022816	ENSMUSG00000022816	ENSMUSG00000022816	16	37734945	37753165	269	314
12644	3	121531624	121550876	ENSMUSG00000034158	ENSMUSG00000034158	ENSMUSG00000034158	16	37787628	37801693	269	314
12645	3	121368569	121445635	ENSMUSG00000046961	ENSMUSG00000046961	ENSMUSG00000046961	16	37835735	37926772	269	314
12646	3	121028238	121295203	ENSMUSG00000022812	ENSMUSG00000022812	ENSMUSG00000022812	16	38008240	38165318	269	314
12647	3	120984236	121020021	ENSMUSG00000022809	ENSMUSG00000022809	ENSMUSG00000022809	16	38167590	38214062	269	314
12648	3	120904563	120968377	ENSMUSG00000022805	ENSMUSG00000022805	ENSMUSG00000022805	16	38217431	38261016	269	314
12649	3	120843596	120862127	ENSMUSG00000022803	ENSMUSG00000022803	ENSMUSG00000022803	16	38281453	38297346	269	314
12650	3	120799424	120831341	ENSMUSG00000002847	ENSMUSG00000002847	ENSMUSG00000002847	16	38315356	38352378	269	314
12651	3	120780805	120791481	ENSMUSG00000002844	ENSMUSG00000002844	ENSMUSG00000002844	16	38364632	38371922	269	314
12652	3	120725835	120761139	ENSMUSG00000075122	ENSMUSG00000075122	ENSMUSG00000075122	16	38378357	38405776	269	314
12653	3	120700058	120725810	ENSMUSG00000002846	ENSMUSG00000002846	ENSMUSG00000002846	16	38417660	38441908	269	314
12654	3	120670489	120696240	ENSMUSG00000034064	ENSMUSG00000034064	ENSMUSG00000034064	16	38444448	38469525	269	314
12655	3	120631041	120665134	ENSMUSG00000002845	ENSMUSG00000002845	ENSMUSG00000002845	16	38482173	38510716	269	314
12656	3	120495910	120619254	ENSMUSG00000022799	ENSMUSG00000022799	ENSMUSG00000022799	16	38517675	38632367	269	314
12657	3	120413286	120442442	ENSMUSG00000022793	ENSMUSG00000022793	ENSMUSG00000022793	16	38661620	38688381	269	314
12658	3	120375115	120406690	ENSMUSG00000049436	ENSMUSG00000049436	ENSMUSG00000049436	16	38692522	38719533	269	314
12659	3	120347687	120352992	ENSMUSG00000022798	ENSMUSG00000022798	ENSMUSG00000022798	16	38731537	38748081	269	314
12660	3	120103691	120306747	ENSMUSG00000022790	ENSMUSG00000022790	ENSMUSG00000022790	16	38821677	38946493	269	314
12661	3	117011854	117646568	ENSMUSG00000061080	ENSMUSG00000061080	ENSMUSG00000061080	16	41452709	42064139	269	314
12662	3	116824861	116922935	ENSMUSG00000047261	ENSMUSG00000047261	ENSMUSG00000047261	16	42167776	42259983	269	314
12663	3	115540230	116348817	ENSMUSG00000022708	ENSMUSG00000022708	ENSMUSG00000022708	16	43152087	43538403	269	314
12664	3	115492596	115509826	ENSMUSG00000071552	ENSMUSG00000071552	ENSMUSG00000071552	16	43568333	43583419	269	314
12665	3	115330247	115373529	ENSMUSG00000022705	ENSMUSG00000022705	ENSMUSG00000022705	16	43681574	43742171	269	314
12666	3	115258301	115289954	ENSMUSG00000022704	ENSMUSG00000022704	ENSMUSG00000022704	16	43780739	43809008	269	314
12667	3	115165676	115258150	ENSMUSG00000022701	ENSMUSG00000022701	ENSMUSG00000022701	16	43862839	43882729	269	314
12668	3	115149438	115164515	ENSMUSG00000036304	ENSMUSG00000036304	ENSMUSG00000036304	16	43888478	43898382	269	314
12669	3	115040370	115148710	ENSMUSG00000036292	ENSMUSG00000036292	ENSMUSG00000036292	16	43899708	43935770	269	314
12670	3	114948598	115013591	ENSMUSG00000052459	ENSMUSG00000052459	ENSMUSG00000052459	16	44006114	44058329	269	314
12671	3	114920531	114947786	ENSMUSG00000022698	ENSMUSG00000022698	ENSMUSG00000022698	16	44064225	44082697	269	314
12672	3	114855129	114871770	ENSMUSG00000068284	ENSMUSG00000068284	ENSMUSG00000068284	16	44092729	44143369	269	314

12673	3	114734183	114830999	ENSMUSG00000022696	ENSMUSG00000022696	ENSMUSG00000022696	16	44159857	44252528	269	314
12674	3	114644262	114716693	ENSMUSG00000043065	ENSMUSG00000043065	ENSMUSG00000043065	16	44274772	44306171	269	314
12675	3	114564402	114643030	ENSMUSG00000071550	ENSMUSG00000071550	ENSMUSG00000071550	16	44323951	44371033	269	314
12676	3	114414065	114488988	ENSMUSG00000022687	ENSMUSG00000022687	ENSMUSG00000022687	16	44404377	44478202	269	314
12677	3	114203981	114221245	ENSMUSG00000036208	ENSMUSG00000036208	ENSMUSG00000036208	16	44643631	44656464	269	314
12678	3	114192522	114202481	ENSMUSG00000022668	ENSMUSG00000022668	ENSMUSG00000022668	16	44658213	44665680	269	314
12679	3	113806103	113842667	ENSMUSG00000022665	ENSMUSG00000022665	ENSMUSG00000022665	16	45013416	45047159	269	314
12680	3	113763585	113785692	ENSMUSG00000022664	ENSMUSG00000022664	ENSMUSG00000022664	16	45058905	45077971	269	314
12681	3	113734236	113763453	ENSMUSG00000022663	ENSMUSG00000022663	ENSMUSG00000022663	16	45078161	45107870	269	314
12682	3	113667464	113701066	ENSMUSG00000052013	ENSMUSG00000052013	ENSMUSG00000052013	16	45143669	45172227	269	314
12683	3	113534664	113564346	ENSMUSG00000022661	ENSMUSG00000022661	ENSMUSG00000022661	16	45311578	45319635	269	314
12684	3	113342424	113495795	ENSMUSG00000033210	ENSMUSG00000033210	ENSMUSG00000033210	16	45454641	45526337	269	314
12685	3	113322382	113334807	ENSMUSG00000022659	ENSMUSG00000022659	ENSMUSG00000022659	16	45529864	45539473	269	314
12686	3	113287926	113319747	ENSMUSG00000033187	ENSMUSG00000033187	ENSMUSG00000033187	16	45548732	45573373	269	314
12687	3	113249359	113282621	ENSMUSG00000033177	ENSMUSG00000033177	ENSMUSG00000033177	16	45575691	45612990	269	314
12688	3	113200276	113215424	ENSMUSG00000022658	ENSMUSG00000022658	ENSMUSG00000022658	16	45630562	45643940	269	314
12689	3	113180589	113194896	ENSMUSG00000033157	ENSMUSG00000033157	ENSMUSG00000033157	16	45649729	45662236	269	314
12690	3	113061377	113177784	ENSMUSG00000033149	ENSMUSG00000033149	ENSMUSG00000033149	16	45665728	45763571	269	314
12691	3	112743546	112853906	ENSMUSG00000022657	ENSMUSG00000022657	ENSMUSG00000022657	16	45955071	46039588	269	314
12692	3	112273555	112335752	ENSMUSG00000022656	ENSMUSG00000022656	ENSMUSG00000022656	16	46367399	46416301	269	314
12693	3	110159779	110319658	ENSMUSG00000022652	ENSMUSG00000022652	ENSMUSG00000022652	16	48350863	48550531	269	314
12694	3	110024321	110056542	ENSMUSG00000030775	ENSMUSG00000030775	ENSMUSG00000030775	16	48651536	48691576	269	314
12695	3	109791274	109896382	ENSMUSG00000064061	ENSMUSG00000064061	ENSMUSG00000064061	16	48845618	48913716	269	314
12696	3	109752104	109790980	ENSMUSG00000033031	ENSMUSG00000033031	ENSMUSG00000033031	16	48913814	48939328	269	314
12697	3	109581906	109730859	ENSMUSG00000022647	ENSMUSG00000022647	ENSMUSG00000022647	16	48977103	49117784	269	314
12698	3	109362350	109423938	ENSMUSG00000032965	ENSMUSG00000032965	ENSMUSG00000032965	16	49618920	49684752	269	314
12699	3	109244631	109292625	ENSMUSG00000055447	ENSMUSG00000055447	ENSMUSG00000055447	16	49775244	49834636	269	314
12700	3	108911979	109007396	ENSMUSG00000022641	ENSMUSG00000022641	ENSMUSG00000022641	16	50116507	50351729	269	314
12701	3	108578878	108580171	ENSMUSG00000050685	ENSMUSG00000050685	ENSMUSG00000050685	16	50509260	50510249	269	314
12702	3	106859799	107070577	ENSMUSG00000022637	ENSMUSG00000022637	ENSMUSG00000022637	16	51952371	52127389	269	314
12703	3	106568403	106778433	ENSMUSG00000022636	ENSMUSG00000022636	ENSMUSG00000022636	16	52170261	52373422	269	314
12704	3	103636549	103681375	ENSMUSG00000064310	ENSMUSG00000064310	ENSMUSG00000064310	16	55150126	55208188	269	314
12705	3	103051048	103061057	ENSMUSG00000035356	ENSMUSG00000035356	ENSMUSG00000035356	16	55736329	55763805	269	314
12706	3	102987071	103024148	ENSMUSG00000075033	ENSMUSG00000075033	ENSMUSG00000075033	16	55769136	55791336	269	314
12707	3	102926177	102972095	ENSMUSG00000022604	ENSMUSG00000022604	ENSMUSG00000022604	16	55827214	55859799	269	314
12708	3	102877964	102879121	ENSMUSG00000059144	ENSMUSG00000059144	ENSMUSG00000059144	16	55899016	55899249	269	314
12709	3	102850981	102878607	ENSMUSG00000050077	ENSMUSG00000050077	ENSMUSG00000050077	16	55915206	55931303	269	314
12710	3	102775730	102795969	ENSMUSG00000071533	ENSMUSG00000071533	ENSMUSG00000071533	16	55943389	55954632	269	314
12711	3	102763402	102767777	ENSMUSG00000044763	ENSMUSG00000044763	ENSMUSG00000044763	16	55958671	55962727	269	314
12712	3	102527388	102714757	ENSMUSG00000052917	ENSMUSG00000052917	ENSMUSG00000052917	16	56000360	56114962	269	314
12713	3	102427981	102522109	ENSMUSG00000035270	ENSMUSG00000035270	ENSMUSG00000035270	16	56129483	56194040	269	314
12714	3	101950871	102194939	ENSMUSG00000035258	ENSMUSG00000035258	ENSMUSG00000035258	16	56402865	56614268	269	314
12715	3	101910850	101950500	ENSMUSG00000022757	ENSMUSG00000022757	ENSMUSG00000022757	16	56615283	56642305	269	314
12716	3	101811143	101896955	ENSMUSG00000022755	ENSMUSG00000022755	ENSMUSG00000022755	16	56649560	56720809	269	314
12717	3	101720754	101778965	ENSMUSG00000022754	ENSMUSG00000022754	ENSMUSG00000022754	16	56730112	56811114	269	314
12718	3	101566831	101602483	ENSMUSG00000022752	ENSMUSG00000022752	ENSMUSG00000022752	16	57043140	57078064	269	314
12719	3	101536258	101556881	ENSMUSG00000022751	ENSMUSG00000022751	ENSMUSG00000022751	16	57078434	57087579	269	314
12720	3	101462546	101525205	ENSMUSG00000022749	ENSMUSG00000022749	ENSMUSG00000022749	16	57090221	57152825	269	314
12721	3	101019514	101379922	ENSMUSG00000022748	ENSMUSG00000022748	ENSMUSG00000022748	16	57223376	57244904	269	314
12722	3	101034679	101316047	ENSMUSG00000043336	ENSMUSG00000043336	ENSMUSG00000043336	16	57470600	57494148	269	314
12723	3	100840144	100997848	ENSMUSG00000068196	ENSMUSG00000068196	ENSMUSG00000068196	16	57547898	57675734	269	314
12724	3	99997504	100103223	ENSMUSG00000035107	ENSMUSG00000035107	ENSMUSG00000035107	16	58351078	58412288	269	314
12725	3	99934029	99996784	ENSMUSG00000022747	ENSMUSG00000022747	ENSMUSG00000022747	16	58410668	58465899	269	314
12726	3	99780982	99795131	ENSMUSG00000022742	ENSMUSG00000022742	ENSMUSG00000022742	16	58612835	58622904	269	314
12727	3	99733568	99734650	ENSMUSG00000047293	ENSMUSG00000047293	ENSMUSG00000047293	16	58660185	58661267	269	314
12728	3	99717045	99724459	ENSMUSG00000022744	ENSMUSG00000022744	ENSMUSG00000022744	16	58670561	58676782	269	314
12729	3	99145885	99173524	ENSMUSG00000022724	ENSMUSG00000022724	ENSMUSG00000022724	16	59414098	59434705	269	314
12730	3	99078327	99144845	ENSMUSG00000022723	ENSMUSG00000022723	ENSMUSG00000022723	16	59434352	59498008	269	314
12731	3	98966285	99000059	ENSMUSG00000022722	ENSMUSG00000022722	ENSMUSG00000022722	16	59555205	59581423	269	314
12732	3	98016440	98950232	ENSMUSG00000055540	ENSMUSG00000055540	ENSMUSG00000055540	16	59596276	60547219	269	314
12733	3	95264537	95328320	ENSMUSG00000050312	ENSMUSG00000050312	ENSMUSG00000050312	16	62674701	62745920	269	314
12734	3	95181672	95256811	ENSMUSG00000022911	ENSMUSG00000022911	ENSMUSG00000022911	16	62735941	62789253	269	314
12735	3	95215907	95230144	ENSMUSG00000047854	ENSMUSG00000047854	ENSMUSG00000047854	16	62757001	62766602	269	314
12736	3	95074647	95175412	ENSMUSG00000022912	ENSMUSG00000022912	ENSMUSG00000022912	16	62796685	62871594	269	314
12737	3	89239364	89613972	ENSMUSG00000052504	ENSMUSG00000052504	ENSMUSG00000052504	16	63485738	63806360	269	314
12738	3	88270952	88273991	ENSMUSG00000047141	ENSMUSG00000047141	ENSMUSG00000047141	16	64726704	64728416	269	314
12739	3	88122536	88124663	ENSMUSG00000050783	ENSMUSG00000050783	ENSMUSG00000050783	16	64866931	65048056	269	314
12740	3	87391473	87408427	ENSMUSG00000004842	ENSMUSG00000004842	ENSMUSG00000004842	16	65438862	65453881	269	314
12741	3	87359140	87387338	ENSMUSG00000004843	ENSMUSG00000004843	ENSMUSG00000004843	16	65457393	65480986	269	314
12742	3	87093746	87122959	ENSMUSG00000071487	ENSMUSG00000071487	ENSMUSG00000071487	16	65733893	65758016	269	314
12743	3	85858322	86200638	ENSMUSG00000064115	ENSMUSG00000064115	ENSMUSG00000064115	16	66581353	67538491	269	314
12744	3	81621542	81894002	ENSMUSG00000022707	ENSMUSG00000022707	ENSMUSG00000022707	16	70196743	70452387	269	314
12745	3	78731953	79721751	ENSMUSG00000022883	ENSMUSG00000022883	ENSMUSG00000022883	16	72624802	72925867	269	314
12746	3	77172627	77776744	ENSMUSG00000052516	ENSMUSG00000052516	ENSMUSG00000052516	16	73779694	74293592	269	314
12747	21	14510350	14522558	ENSMUSG00000032940	ENSMUSG00000032940	ENSMUSG00000032940	16	75475524	75484382	270	315
12748	21	14665310	14677380	ENSMUSG00000032932	ENSMUSG00000032932	ENSMUSG00000032932	16	75637868	75649458	270	315
12749	21	14780107	15180402	ENSMUSG00000022876	ENSMUSG00000022876	ENSMUSG00000022876	16	75741474	75835706	270	315
12750	21	15258908	15262384	ENSMUSG00000048490	ENSMUSG00000048490	ENSMUSG00000048490	16	76172581	76256507	270	315
12751	21	16024215	16174248	ENSMUSG00000022867	ENSMUSG00000022867	ENSMUSG00000022867	16	76896661	76999372	270	315
12752	21	17807201	17861135	ENSMUSG00000022865	ENSMUSG00000022865	ENSMUSG00000022865	16	78184283	78222167	270	315
12753	21	18083163	18113539	ENSMUSG00000022864	ENSMUSG00000022864	ENSMUSG00000022864	16	78426122	78459239	270	315
12754	21	18539021	18561553	ENSMUSG00000022860	ENSMUSG00000022860	ENSMUSG00000022860	16	78813540	78832496	270	315
12755	21	18563561	18697844	ENSMUSG00000022857	ENSMUSG00000022857	ENSMUSG00000022857	16	78835600	78973684	270	315
12756	21	21574767	21834285	ENSMUSG00000022762	ENSMUSG00000022762	ENSMUSG00000022762	16	81315299	81506877	270	315
12757	21	25879843	25901672	ENSMUSG00000022889	ENSMUSG00000022889	ENSMUSG00000022889	16	84600170	84617815	270	315
12758	21	25933515	26009078	ENSMUSG00000053062	ENSMUSG00000053062	ENSMUSG00000053062	16	84657025	84707359	270	315
12759	21	26174733	26465003	ENSMUSG00000022892	ENSMUSG00000022892	ENSMUSG00000022892	16	84838761	85057004	270	315
12760	21	26760402	26867452	ENSMUSG00000041134	ENSMUSG00000041134	ENSMUSG00000041134	16	85345428	85439569	270	315
12761	21	27130479	27139599	ENSMUSG00000022893	ENSMUSG00000022893	ENSMUSG00000022893	16	85683023	85692295	270	315
12762	21	27212112	27260703	ENSMUSG00000022894	ENSMUSG00000022894	ENSMUSG00000022894	16	85751616	85789480	270	315
12763	21	29166400	29179546	ENSMUSG00000044442	ENSMUSG00000044442	ENSMUSG00000044442	16	87243381	87257845	270	315
12764	21	29222337	29287095	ENSMUSG00000052299	ENSMUSG00000052299	ENSMUSG00000052299	16	87267869	87321673	270	315
12765	21	29299953	29313563	ENSMUSG00000041079	ENSMUSG00000041079	ENSMUSG00000041079	16	87322527	87329762	270	315
12766	21	29318809	29348676	ENSMUSG00000025616	ENSMUSG00000025616	ENSMUSG00000025616	16	87344207	87372707	270	315
12767	21	29350518	29367881	ENSMUSG00000025613	ENSMUSG00000025613	ENSMUSG00000025613	16	87372523	87385053	270	315
12768	21	29380053	29470078	ENSMUSG00000025610	ENSMUSG00000025610	ENSMUSG00000025610	16	87442526	87484532	270	315
12769	21	29593091	29640340	ENSMUSG00000025612	ENSMUSG00000025612	ENSMUSG00000025612	16	87588150	87622542	270	315
12770	21	29847747	30233689	ENSMUSG00000022935	ENSMUSG00000022935	ENSMUSG00000022935	16	87785053	88178561	270	315
12771	21	30460132	30460806	ENSMUSG00000055811	ENSMUSG00000055811	ENSMUSG00000055811	16	88395024	88395698	270	315
12772	21	30508196	30510223	ENSMUSG00000050520	ENSMUSG00000050520	ENSMUSG00000050520	16	88451217	88451894	270	315
12773	21	30576357	30577121	ENSMUSG00000050239	ENSMUSG00000050239	ENSMUSG00000050239	16	88500349	88501137	270	315
12774	21	30613592	30614224	ENSMUSG00000071471	ENSMUSG00000071471	ENSMUSG00000071471	16	88535943	88536590	270	315
12775	21	30734517	30734969	ENSMUSG00000022931	ENSMUSG00000022931	ENSMUSG00000022931	16	88709074	88718343	270	315
12776	21	30929454	30929651	ENSMUSG00000048643	ENSMUSG00000048643	ENSMUSG00000048643	16	88812909	88813076	270	315
12777	21	31107218	31107409	ENSMUSG00000059632	ENSMUSG00000059632	ENSMUSG00000059632	16	89376581	89376766	270	315
12778	21	31414352	31853161	ENSMUSG00000002489	ENSMUSG00000002489	ENSMUSG00000002489	16	89675971	89849686	270	315
12779	21	31965223	32026259	ENSMUSG00000022983	ENSMUSG00000022983	ENSMUSG00000022983	16	90118157	90152035	270	315
12780	21	32167499	32298248	ENSMUSG00000053414	ENSMUSG00000053414	ENSMUSG00000053414	16	90275244	90388410	270	315
12781	21	32562403	32573247	ENSMUSG00000022978	ENSMUSG00000022978	ENSMUSG00000022978	16	90608171	90616230	270	315
12782	21	32593030	32606462	ENSMUSG00000039956	ENSMUSG00000039956	ENSMUSG00000039956	16	90627183	90638635	270	315
12783	21	32605200	32687206	ENSMUSG00000039929	ENSMUSG00000039929	ENSMUSG00000039929	16	90640868	90699199	270	315
12784	21	32706560	32809570	ENSMUSG00000039903	ENSMUSG00000039903	ENSMUSG00000039903	16	90720006	90793552	270	315
12785	21	32895848	32907047	ENSMUSG00000022972	ENSMUSG00000022972	ENSMUSG00000022972	16	90814670	90823685	270	315
12786	21	32922944	33022148	ENSMUSG00000022973	ENSMUSG00000022973	ENSMUSG00000022973	16	90827099	90899913	270	315
12787	21	33028081	33066040	ENSMUSG00000022974	ENSMUSG00000022974	ENSMUSG00000022974	16	90903775	90933023	270	315
12788	21	33087746	33107868	ENSMUSG00000039851	ENSMUSG00000039851	ENSMUSG00000039851	16	90942794	90958004	270	315
12789	21	33320023	33323374	ENSMUSG00000039830	ENSMUSG00000039830	ENSMUSG00000039830	16	91114409	91117536	270	315
12790	21	33364320	33366596	ENSMUSG00000046160	ENSMUSG00000046160	ENSMUSG00000046160	16	91158628	91160798	270	315
12791	21	33524101	33558688	ENSMUSG00000022971	ENSMUSG00000022971	ENSMUSG00000022971	16	91261758	91294225	270	315
12792	21	33560533	33591409	ENSMUSG00000022969	ENSMUSG00000022969	ENSMUSG00000022969	16	91295167	91314688	270	315
12793	21	33619079	33654038	ENSMUSG00000022967	ENSMUSG00000022967	ENSMUSG00000022967	16	91374108	91396296	270	315
12794	21	33697608	33731698	ENSMUSG00000022965	ENSMUSG00000022965	ENSMUSG00000022965	16	91435953	91452866	270	315
12795	21	33779707	33785650	ENSMUSG00000039763	ENSMUSG00000039763	ENSMUSG00000039763	16	91504573	91507852	270	315
12796	21	33798108	33837037	ENSMUSG00000022962	ENSMUSG00000022962	ENSMUSG00000022962	16	91510268	91535812	270	315
12797	21	33836794	33871657	ENSMUSG00000022961	ENSMUSG00000022961	ENSMUSG00000022961	16	91536739	91568048	270	315
12798	21	33872081	33882884	ENSMUSG00000022960	ENSMUSG00000022960	ENSMUSG00000022960	16	91568126	91577612	270	315
12799	21	33883517	33935936	ENSMUSG00000058240	ENSMUSG00000058240	ENSMUSG00000058240	16	91578183	91617437	270	315
12800	21	33936634	34194035	ENSMUSG00000022957	ENSMUSG00000022957	ENSMUSG00000022957	16	91671052	91800868	270	315
12801	21	34658193	34665307	ENSMUSG00000039672	ENSMUSG00000039672	ENSMUSG00000039672	16	92181248	92186988	270	315
12802	21	34740858	34806443	ENSMUSG00000039639	ENSMUSG00000039639	ENSMUSG00000039639	16	92234860	92248327	270	315
12803	21	34810658	34909303	ENSMUSG00000022951	ENSMUSG00000022951	ENSMUSG00000022951	16	92280814	92354984	270	315
12804	21	34963558	35012389	ENSMUSG00000022949	ENSMUSG00000022949	ENSMUSG00000022949	16	92386993	92430100	270	315
12805	21	35081975	35343511	ENSMUSG00000022952	ENSMUSG00000022952	ENSMUSG00000022952	16	92492515	92585724	270	315
12806	21	36328710	36354807	ENSMUSG00000022948	ENSMUSG00000022948	ENSMUSG00000022948	16	93471997	93492343	270	315
12807	21	36364155	36367332	ENSMUSG00000051483	ENSMUSG00000051483	ENSMUSG00000051483	16	93496379	93498862	270	315
12808	21	36429080	36440722	ENSMUSG00000022947	ENSMUSG00000022947	ENSMUSG00000022947	16	93571755	93579526	270	315
12809	21	36458876	36588441	ENSMUSG00000022946	ENSMUSG00000022946	ENSMUSG00000022946	16	93600447	93699128	270	315
12810	21	36614376	36670813	ENSMUSG00000039456	ENSMUSG00000039456	ENSMUSG00000039456	16	93720824	93764612	270	315
12811	21	36679559	36710994	ENSMUSG00000022945	ENSMUSG00000022945	ENSMUSG00000022945	16	93772441	93794646	270	315
12812	21	36754793	36870737	ENSMUSG00000047109	ENSMUSG00000047109	ENSMUSG00000047109	16	93807573	93897377	270	315
12813	21	36993851	37044088	ENSMUSG00000062713	ENSMUSG00000062713	ENSMUSG00000062713	16	93974356	94236097	270	315
12814	21	37300733	37313826	ENSMUSG00000022941	ENSMUSG00000022941	ENSMUSG00000022941	16	94437218	94445733	270	315
12815	21	37367441	37497274	ENSMUSG00000040785	ENSMUSG00000040785	ENSMUSG00000040785	16	94492710	94578019	270	315
12816	21	37517598	37562090	ENSMUSG00000022898	ENSMUSG00000022898	ENSMUSG00000022898	16	94606586	94635241	270	315
12817	21	37713077	37809347	ENSMUSG00000022897	ENSMUSG00000022897	ENSMUSG00000022897	16	94720258	94801197	270	315
12818	21	37918656	38023562	ENSMUSG00000043301	ENSMUSG00000043301	ENSMUSG00000043301	16	94869867	94942425	270	315
12819	21	38550534	38595618	ENSMUSG00000062609	ENSMUSG00000062609	ENSMUSG00000062609	16	95366449	95409058	270	315
12820	21	38675671	38792267	ENSMUSG00000040732	ENSMUSG00000040732	ENSMUSG00000040732	16	95469604	95518756	270	315
12821	21	39099719	39118749	ENSMUSG00000022895	ENSMUSG00000022895	ENSMUSG00000022895	16	95815219	95827936	270	315
12822	21	39468565	39477647	ENSMUSG00000022913	ENSMUSG00000022913	ENSMUSG00000022913	16	96084845	96095827	270	315
12823	21	39477972	39607366	ENSMUSG00000022914	ENSMUSG00000022914	ENSMUSG00000022914	16	96097357	96187327	270	315
12824	21	39674040	39691685	ENSMUSG00000023147	ENSMUSG00000023147	ENSMUSG00000023147	16	96250329	96262764	270	315
12825	21	39699640	39739573	ENSMUSG00000045275	ENSMUSG00000045275	ENSMUSG00000045275	16	96263961	96283850	270	315
12826	21	39745626	39809303	ENSMUSG00000040666	ENSMUSG00000040666	ENSMUSG00000040666	16	96305581	96329470	270	315
12827	21	39850239	39956686	ENSMUSG00000074892	ENSMUSG00000074892	ENSMUSG00000074892	16	96420079	96421005	270	315
12828	21	40039204	40095893	ENSMUSG00000000159	ENSMUSG00000000159	ENSMUSG00000000159	16	96466733	96508271	270	315
12829	21	40161113	40223192	ENSMUSG00000048360	ENSMUSG00000048360	ENSMUSG00000048360	16	96572516	96630703	270	315
12830	21	40306831	41002568	ENSMUSG00000050272	ENSMUSG00000050272	ENSMUSG00000050272	16	96697969	97161676	270	315
12831	21	41462061	41569619	ENSMUSG00000040605	ENSMUSG00000040605	ENSMUSG00000040605	16	97461672	97543922	270	315
12832	21	41616384	41651212	ENSMUSG00000022938	ENSMUSG00000022938	ENSMUSG00000022938	16	97576044	97655920	270	315
12833	21	41758351	41801948	ENSMUSG00000000385	ENSMUSG00000000385	ENSMUSG00000000385	16	97721930	97754185	270	315
12834	21	42032614	42060335	ENSMUSG00000005251	ENSMUSG00000005251	ENSMUSG00000005251	16	97896851	97918633	270	315
12835	21	42094394	42172660	ENSMUSG00000014039	ENSMUSG00000014039	ENSMUSG00000014039	16	97946794	98006699	270	315
12836	21	42178290	42247068	ENSMUSG00000045975	ENSMUSG00000045975	ENSMUSG00000045975	16	98010132	98077537	270	315
12837	21	42280009	42303535	ENSMUSG00000046962	ENSMUSG00000046962	ENSMUSG00000046962	16	98102306	98117072	270	315
12838	6	155195523	155620546	ENSMUSG00000023800	ENSMUSG00000023800	ENSMUSG00000023800	17	3283055	3475456	271	316
12839	6	155620335	155677310	ENSMUSG00000036983	ENSMUSG00000036983	ENSMUSG00000036983	17	3475713	3514195	271	316
12840	6	155758194	155818729	ENSMUSG00000023802	ENSMUSG00000023802	ENSMUSG00000023802	17	3591722	3652743	271	316
12841	6	157140756	157572093	ENSMUSG00000069729	ENSMUSG00000069729	ENSMUSG00000069729	17	5054053	5299566	271	316
12842	6	157632650	157664621	ENSMUSG00000004945	ENSMUSG00000004945	ENSMUSG00000004945	17	5367352	5396725	271	316
12843	6	157722545	158014965	ENSMUSG00000034265	ENSMUSG00000034265	ENSMUSG00000034265	17	5449082	5710373	271	316
12844	6	158164282	158286097	ENSMUSG00000002365	ENSMUSG00000002365	ENSMUSG00000002365	17	5797862	5887223	271	316
12845	6	158322907	158440196	ENSMUSG00000023805	ENSMUSG00000023805	ENSMUSG00000023805	17	5897885	5995037	271	316
12846	6	158450535	158509290	ENSMUSG00000015659	ENSMUSG00000015659	ENSMUSG00000015659	17	6000548	6036209	271	316
12847	6	158509372	158535008	ENSMUSG00000034345	ENSMUSG00000034345	ENSMUSG00000034345	17	6036347	6041961	271	316
12848	6	158653680	158852848	ENSMUSG00000034377	ENSMUSG00000034377	ENSMUSG00000034377	17	6095226	6197103	271	316
12849	6	158877454	158976448	ENSMUSG00000038141	ENSMUSG00000038141	ENSMUSG00000038141	17	6380408	6408178	271	316
12850	6	159002339	159105889	ENSMUSG00000041831	ENSMUSG00000041831	ENSMUSG00000041831	17	6523545	6587494	271	316
12851	6	159106769	159160383	ENSMUSG00000052397	ENSMUSG00000052397	ENSMUSG00000052397	17	6587789	6622259	271	316
12852	6	166746240	167195791	ENSMUSG00000023809	ENSMUSG00000023809	ENSMUSG00000023809	17	7019773	7152975	out-of-place	
12853	6	159510417	159613127	ENSMUSG00000071984	ENSMUSG00000071984	ENSMUSG00000071984	17	7577392	7611179	271	
12854	6	167332660	167375896	ENSMUSG00000069135	ENSMUSG00000069135	ENSMUSG00000069135	17	8003706	8034977	272	317
12855	6	166698397	166716476	ENSMUSG00000023861	ENSMUSG00000023861	ENSMUSG00000023861	17	8122753	8135130	272	317
12856	6	166640733	166641620	ENSMUSG00000055945	ENSMUSG00000055945	ENSMUSG00000055945	17	8178580	8182289	272	317
12857	6	166490785	166501353	ENSMUSG00000062327	ENSMUSG00000062327	ENSMUSG00000062327	17	8272597	8280670	272	317
12858	6	165664543	165995547	ENSMUSG00000023868	ENSMUSG00000023868	ENSMUSG00000023868	17	8731554	8820670	272	317
12859	6	165613156	165643088	ENSMUSG00000023873	ENSMUSG00000023873	ENSMUSG00000023873	17	8826535	8846490	272	317
12860	6	163755665	163914886	ENSMUSG00000062078	ENSMUSG00000062078	ENSMUSG00000062078	17	10049913	10162244	272	317
12861	6	163069258	163656009	ENSMUSG00000037196	ENSMUSG00000037196	ENSMUSG00000037196	17	10246499	10683544	272	317
12862	6	161471050	161615097	ENSMUSG00000023827	ENSMUSG00000023827	ENSMUSG00000023827	17	11962642	12062998	272	317
12863	6	161332812	161458406	ENSMUSG00000014426	ENSMUSG00000014426	ENSMUSG00000014426	17	12072857	12161986	272	317
12864	6	161043264	161094339	ENSMUSG00000059481	ENSMUSG00000059481	ENSMUSG00000059481	17	12221966	12262743	272	317
12865	6	160689415	160796004	ENSMUSG00000023828	ENSMUSG00000023828	ENSMUSG00000023828	17	12263330	12351062	272	317
12866	6	160557784	160600038	ENSMUSG00000040966	ENSMUSG00000040966	ENSMUSG00000040966	17	12427547	12471862	272	317
12867	6	160462795	160499740	ENSMUSG00000023829	ENSMUSG00000023829	ENSMUSG00000023829	17	12492232	12519196	272	317

12868	6	160310121	160447573	ENSMUSG00000023830	ENSMUSG00000023830	ENSMUSG00000023830	17	12525771	12613022	272	317
12869	6	160247712	160249098	ENSMUSG00000068037	ENSMUSG00000068037	ENSMUSG00000068037	17	12684492	12685466	272	317
12870	6	160141291	160161725	ENSMUSG00000073460	ENSMUSG00000073460	ENSMUSG00000073460	17	12742465	12749485	272	317
12871	6	160131488	160139458	ENSMUSG00000057388	ENSMUSG00000057388	ENSMUSG00000057388	17	12754287	12759023	272	317
12872	6	160119520	160130731	ENSMUSG00000068039	ENSMUSG00000068039	ENSMUSG00000068039	17	12759413	12767488	272	317
12873	6	160020138	160034342	ENSMUSG00000006818	ENSMUSG00000006818	ENSMUSG00000006818	17	12850771	12861051	272	317
12874	6	167970520	168115552	ENSMUSG00000068036	ENSMUSG00000068036	ENSMUSG00000068036	17	13497214	13598396	273	318
12875	6	168450433	168463251	ENSMUSG00000048826	ENSMUSG00000048826	ENSMUSG00000048826	17	13931649	13940329	273	318
12876	6	168584680	168810599	ENSMUSG00000023886	ENSMUSG00000023886	ENSMUSG00000023886	17	14016132	14138248	273	318
12877	6	169357800	169396064	ENSMUSG00000023885	ENSMUSG00000023885	ENSMUSG00000023885	17	14405317	14428011	273	318
12878	6	169714433	169831026	ENSMUSG00000046991	ENSMUSG00000046991	ENSMUSG00000046991	17	14565909	14671229	273	318
12879	6	169844182	169848326	ENSMUSG00000050088	ENSMUSG00000050088	ENSMUSG00000050088	17	14679760	14682512	273	318
12880	6	169845929	169866026	ENSMUSG00000023883	ENSMUSG00000023883	ENSMUSG00000023883	17	14681585	14697808	273	318
12881	6	169882135	169893580	ENSMUSG00000036648	ENSMUSG00000036648	ENSMUSG00000036648	17	14700750	14746500	273	318
12882	6	170433219	170441486	ENSMUSG00000014773	ENSMUSG00000014773	ENSMUSG00000014773	17	15073094	15080203	273	318
12883	6	170457769	170558078	ENSMUSG00000014763	ENSMUSG00000014763	ENSMUSG00000014763	17	15106725	15138546	273	318
12884	6	170686133	170704325	ENSMUSG00000014769	ENSMUSG00000014769	ENSMUSG00000014769	17	15180923	15203261	273	318
12885	6	170705390	170723871	ENSMUSG00000014767	ENSMUSG00000014767	ENSMUSG00000014767	17	15204851	15222372	273	318
12886	6	170728532	170735623	ENSMUSG00000014771	ENSMUSG00000014771	ENSMUSG00000014771	17	15226524	15232244	273	318
12887	5	98218819	98290138	ENSMUSG00000023852	ENSMUSG00000023852	ENSMUSG00000023852	17	15409664	15475013	274	319
12888	5	98132900	98160096	ENSMUSG00000048027	ENSMUSG00000048027	ENSMUSG00000048027	17	15509352	15531232	274	319
12889	5	96522327	96544720	ENSMUSG00000023846	ENSMUSG00000023846	ENSMUSG00000023846	17	17079192	17098589	274	319
12890	5	96453330	96504332	ENSMUSG00000047786	ENSMUSG00000047786	ENSMUSG00000047786	17	17107511	17164003	274	319
12891	5	96296924	96398975	ENSMUSG00000023845	ENSMUSG00000023845	ENSMUSG00000023845	17	17234367	17283991	274	319
12892	19	56908177	56919033	ENSMUSG00000003665	ENSMUSG00000003665	ENSMUSG00000003665	17	17548242	17555272	275	320
12893	19	56940237	56946912	ENSMUSG00000045551	ENSMUSG00000045551	ENSMUSG00000045551	17	17581087	17588557	275	320
12894	19	57385046	57421482	ENSMUSG00000007564	ENSMUSG00000007564	ENSMUSG00000007564	17	20650151	20670602	275	320
12895	16	3134245	3139965	ENSMUSG00000067882	ENSMUSG00000067882	ENSMUSG00000067882	17	23278695	23281110	276	321
12896	16	3127163	3132805	ENSMUSG00000071256	ENSMUSG00000071256	ENSMUSG00000071256	17	23284392	23291751	276	321
12897	16	3102607	3110517	ENSMUSG00000062012	ENSMUSG00000062012	ENSMUSG00000062012	17	23303474	23327109	276	321
12898	16	3078896	3082862	ENSMUSG00000023902	ENSMUSG00000023902	ENSMUSG00000023902	17	23328478	23338634	276	321
12899	16	3036683	3050728	ENSMUSG00000023903	ENSMUSG00000023903	ENSMUSG00000023903	17	23356801	23372865	276	321
12900	16	3018111	3025498	ENSMUSG00000043782	ENSMUSG00000043782	ENSMUSG00000043782	17	23388142	23396251	276	321
12901	16	3014041	3017756	ENSMUSG00000041319	ENSMUSG00000041319	ENSMUSG00000041319	17	23396240	23401486	276	321
12902	16	3012627	3014288	ENSMUSG00000023904	ENSMUSG00000023904	ENSMUSG00000023904	17	23401230	23403146	276	321
12903	16	3010377	3012382	ENSMUSG00000023905	ENSMUSG00000023905	ENSMUSG00000023905	17	23403063	23405066	276	321
12904	16	3004715	3008187	ENSMUSG00000023906	ENSMUSG00000023906	ENSMUSG00000023906	17	23406987	23410062	276	321
12905	16	3002458	3004507	ENSMUSG00000066720	ENSMUSG00000066720	ENSMUSG00000066720	17	23410618	23411271	276	321
12906	16	2962795	2970475	ENSMUSG00000023908	ENSMUSG00000023908	ENSMUSG00000023908	17	23454063	23464364	276	321
12907	16	2959386	2963456	ENSMUSG00000023909	ENSMUSG00000023909	ENSMUSG00000023909	17	23463810	23467974	276	321
12908	16	2954218	2958022	ENSMUSG00000040680	ENSMUSG00000040680	ENSMUSG00000040680	17	23468819	23473455	276	321
12909	16	2901981	2941201	ENSMUSG00000040097	ENSMUSG00000040097	ENSMUSG00000040097	17	23483036	23491408	276	321
12910	16	2873215	2889359	ENSMUSG00000023911	ENSMUSG00000023911	ENSMUSG00000023911	17	23504540	23513701	276	321
12911	16	2742655	2761412	ENSMUSG00000039218	ENSMUSG00000039218	ENSMUSG00000039218	17	23535734	23551749	276	321
12912	16	2774633	2777950	ENSMUSG00000049620	ENSMUSG00000049620	ENSMUSG00000049620	17	23560988	23563365	276	321
12913	16	2788487	2795134	ENSMUSG00000024114	ENSMUSG00000024114	ENSMUSG00000024114	17	23564407	23571778	276	321
12914	16	2807165	2811721	ENSMUSG00000024116	ENSMUSG00000024116	ENSMUSG00000024116	17	23595680	23600735	276	321
12915	16	2842729	2848172	ENSMUSG00000045027	ENSMUSG00000045027	ENSMUSG00000045027	17	23721157	23725720	276	321
12916	16	2702424	2710553	ENSMUSG00000050762	ENSMUSG00000050762	ENSMUSG00000050762	17	23765865	23773571	276	321
12917	16	2672499	2699030	ENSMUSG00000016946	ENSMUSG00000016946	ENSMUSG00000016946	17	23775356	23801107	276	321
12918	16	2527971	2593189	ENSMUSG00000024122	ENSMUSG00000024122	ENSMUSG00000024122	17	23803292	23869207	276	321
12919	16	2510367	2519693	ENSMUSG00000036820	ENSMUSG00000036820	ENSMUSG00000036820	17	23883455	23891369	276	321
12920	16	2461501	2464147	ENSMUSG00000036473	ENSMUSG00000036473	ENSMUSG00000036473	17	23931431	23936987	276	321
12921	16	2450120	2454670	ENSMUSG00000024118	ENSMUSG00000024118	ENSMUSG00000024118	17	23942654	23948369	276	321
12922	16	2419441	2448856	ENSMUSG00000072082	ENSMUSG00000072082	ENSMUSG00000072082	17	23951498	23977006	276	321
12923	16	2265886	2330748	ENSMUSG00000024130	ENSMUSG00000024130	ENSMUSG00000024130	17	24079728	24137208	276	321
12924	16	2229397	2241583	ENSMUSG00000024132	ENSMUSG00000024132	ENSMUSG00000024132	17	24154283	24166914	276	321
12925	16	2226727	2228710	ENSMUSG00000024136	ENSMUSG00000024136	ENSMUSG00000024136	17	24168165	24170684	276	321
12926	16	2213568	2225744	ENSMUSG00000024137	ENSMUSG00000024137	ENSMUSG00000024137	17	24171379	24182911	276	321
12927	16	2202030	2204788	ENSMUSG00000043445	ENSMUSG00000043445	ENSMUSG00000043445	17	24198073	24199208	276	321
12928	16	2195496	2199412	ENSMUSG00000024142	ENSMUSG00000024142	ENSMUSG00000024142	17	24201153	24206678	276	321
12929	16	2199255	2201012	ENSMUSG00000045744	ENSMUSG00000045744	ENSMUSG00000045744	17	24201484	24203069	276	321
12930	16	2168552	2186434	ENSMUSG00000033597	ENSMUSG00000033597	ENSMUSG00000033597	17	24216383	24236520	276	321
12931	16	2145800	2168130	ENSMUSG00000052752	ENSMUSG00000052752	ENSMUSG00000052752	17	24236450	24255446	276	321
12932	16	2038600	2078713	ENSMUSG00000002496	ENSMUSG00000002496	ENSMUSG00000002496	17	24324121	24360198	276	321
12933	16	2029817	2037868	ENSMUSG00000041429	ENSMUSG00000041429	ENSMUSG00000041429	17	24360310	24366437	276	321
12934	16	2016897	2028481	ENSMUSG00000002504	ENSMUSG00000002504	ENSMUSG00000002504	17	24366888	24377905	276	321
12935	16	2009742	2010680	ENSMUSG00000071230	ENSMUSG00000071230	ENSMUSG00000071230	17	24385005	24385806	276	321
12936	16	1987769	1999745	ENSMUSG00000041130	ENSMUSG00000041130	ENSMUSG00000041130	17	24397333	24409619	276	321
12937	16	1979969	1984276	ENSMUSG00000007021	ENSMUSG00000007021	ENSMUSG00000007021	17	24412692	24417549	276	321
12938	16	1974151	1977747	ENSMUSG00000040888	ENSMUSG00000040888	ENSMUSG00000040888	17	24420790	24423748	276	321
12939	16	1968920	1971361	ENSMUSG00000019320	ENSMUSG00000019320	ENSMUSG00000019320	17	24423874	24428127	276	321
12940	16	1962065	1968752	ENSMUSG00000040688	ENSMUSG00000040688	ENSMUSG00000040688	17	24428058	24435231	276	321
12941	16	1957276	1958949	ENSMUSG00000008482	ENSMUSG00000008482	ENSMUSG00000008482	17	24443836	24445208	276	321
12942	16	1949520	1951977	ENSMUSG00000040048	ENSMUSG00000040048	ENSMUSG00000040048	17	24449660	24451988	276	321
12943	16	1934582	1944680	ENSMUSG00000002500	ENSMUSG00000002500	ENSMUSG00000002500	17	24460903	24463653	276	321
12944	16	1928238	1933295	ENSMUSG00000075705	ENSMUSG00000075705	ENSMUSG00000075705	17	24464308	24469537	276	321
12945	16	1901592	1908325	ENSMUSG00000039628	ENSMUSG00000039628	ENSMUSG00000039628	17	24480603	24486284	276	321
12946	16	1823990	1867442	ENSMUSG00000024155	ENSMUSG00000024155	ENSMUSG00000024155	17	24556727	24567387	276	321
12947	16	1817222	1828128	ENSMUSG00000045316	ENSMUSG00000045316	ENSMUSG00000045316	17	24577018	24577701	276	321
12948	16	1799109	1817163	ENSMUSG00000024158	ENSMUSG00000024158	ENSMUSG00000024158	17	24578147	24592050	276	321
12949	16	1780417	1783735	ENSMUSG00000046070	ENSMUSG00000046070	ENSMUSG00000046070	17	24607540	24609348	276	321
12950	16	1772934	1779185	ENSMUSG00000039183	ENSMUSG00000039183	ENSMUSG00000039183	17	24610219	24613948	276	321
12951	16	1766715	1772582	ENSMUSG00000024160	ENSMUSG00000024160	ENSMUSG00000024160	17	24614311	24619747	276	321
12952	16	1763230	1766240	ENSMUSG00000073436	ENSMUSG00000073436	ENSMUSG00000073436	17	24619753	24622645	276	321
12953	16	1762223	1763152	ENSMUSG00000038880	ENSMUSG00000038880	ENSMUSG00000038880	17	24622720	24623874	276	321
12954	16	1760323	1761711	ENSMUSG00000073435	ENSMUSG00000073435	ENSMUSG00000073435	17	24624100	24625122	276	321
12955	16	1696222	1760318	ENSMUSG00000024163	ENSMUSG00000024163	ENSMUSG00000024163	17	24625134	24664555	276	321
12956	16	1603974	1667910	ENSMUSG00000038002	ENSMUSG00000038002	ENSMUSG00000038002	17	24698975	24743382	276	321
12957	16	1500946	1597268	ENSMUSG00000024169	ENSMUSG00000024169	ENSMUSG00000024169	17	24744323	24827086	276	321
12958	16	1518743	1545568	ENSMUSG00000024168	ENSMUSG00000024168	ENSMUSG00000024168	17	24785829	24808143	276	321
12959	16	1483365	1500454	ENSMUSG00000024170	ENSMUSG00000024170	ENSMUSG00000024170	17	24827177	24843375	276	321
12960	16	1475941	1478469	ENSMUSG00000044172	ENSMUSG00000044172	ENSMUSG00000044172	17	24848451	24852868	276	321
12961	16	1435346	1465582	ENSMUSG00000036636	ENSMUSG00000036636	ENSMUSG00000036636	17	24860994	24889699	276	321
12962	16	1424462	1434003	ENSMUSG00000059562	ENSMUSG00000059562	ENSMUSG00000059562	17	24890709	24899451	276	321
12963	16	1341933	1353352	ENSMUSG00000035521	ENSMUSG00000035521	ENSMUSG00000035521	17	24960931	24967724	276	321
12964	16	1339244	1341876	ENSMUSG00000015126	ENSMUSG00000015126	ENSMUSG00000015126	17	24967799	24970395	276	321
12965	16	1324654	1339440	ENSMUSG00000047507	ENSMUSG00000047507	ENSMUSG00000047507	17	24970260	24979735	276	321
12966	16	1251423	1252964	ENSMUSG00000034039	ENSMUSG00000034039	ENSMUSG00000034039	17	25047883	25050284	276	321
12967	16	1211659	1215257	ENSMUSG00000033200	ENSMUSG00000033200	ENSMUSG00000033200	17	25098184	25102042	276	321
12968	16	1143739	1211772	ENSMUSG00000024112	ENSMUSG00000024112	ENSMUSG00000024112	17	25101887	25160768	276	321
12969	16	1068870	1069964	ENSMUSG00000050824	ENSMUSG00000050824	ENSMUSG00000050824	17	25217475	25224853	276	321
12970	16	971809	976979	ENSMUSG00000024176	ENSMUSG00000024176	ENSMUSG00000024176	17	25293492	25298286	276	321
12971	16	843639	960983	ENSMUSG00000002279	ENSMUSG00000002279	ENSMUSG00000002279	17	25306787	25390323	276	321
12972	16	788043	790700	ENSMUSG00000025739	ENSMUSG00000025739	ENSMUSG00000025739	17	25444824	25446685	276	321
12973	16	778047	787976	ENSMUSG00000019214	ENSMUSG00000019214	ENSMUSG00000019214	17	25446628	25455002	276	321
12974	16	774976	778384	ENSMUSG00000041199	ENSMUSG00000041199	ENSMUSG00000041199	17	25455351	25459056	276	321
12975	16	759429	772927	ENSMUSG00000041062	ENSMUSG00000041062	ENSMUSG00000041062	17	25463640	25475930	276	321
12976	16	752638	758866	ENSMUSG00000063011	ENSMUSG00000063011	ENSMUSG00000063011	17	25476211	25481927	276	321
12977	16	719767	730986	ENSMUSG00000002280	ENSMUSG00000002280	ENSMUSG00000002280	17	25501400	25510932	276	321
12978	16	716937	719457	ENSMUSG00000061046	ENSMUSG00000061046	ENSMUSG00000061046	17	25510390	25513182	276	321
12979	16	712584	716460	ENSMUSG00000071202	ENSMUSG00000071202	ENSMUSG00000071202	17	25514246	25517729	276	321
12980	16	711159	712590	ENSMUSG00000057411	ENSMUSG00000057411	ENSMUSG00000057411	17	25518106	25519884	276	321
12981	16	705174	707479	ENSMUSG00000002274	ENSMUSG00000002274	ENSMUSG00000002274	17	25522171	25524645	276	321
12982	16	682519	695809	ENSMUSG00000025738	ENSMUSG00000025738	ENSMUSG00000025738	17	25544015	25548855	276	321
12983	16	674735	680445	ENSMUSG00000025737	ENSMUSG00000025737	ENSMUSG00000025737	17	25551248	25556325	276	321
12984	16	671668	674530	ENSMUSG00000025736	ENSMUSG00000025736	ENSMUSG00000025736	17	25556647	25559366	276	321
12985	16	670116	672768	ENSMUSG00000039615	ENSMUSG00000039615	ENSMUSG00000039615	17	25558234	25560961	276	321
12986	16	666076	668268	ENSMUSG00000025735	ENSMUSG00000025735	ENSMUSG00000025735	17	25562065	25564727	276	321
12987	16	658087	664133	ENSMUSG00000025733	ENSMUSG00000025733	ENSMUSG00000025733	17	25566438	25572451	276	321
12988	16	645059	653434	ENSMUSG00000073434	ENSMUSG00000073434	ENSMUSG00000073434	17	25572668	25578557	276	321
12989	16	631850	638475	ENSMUSG00000025732	ENSMUSG00000025732	ENSMUSG00000025732	17	25591298	25596338	276	321
12990	16	624435	626291	ENSMUSG00000025731	ENSMUSG00000025731	ENSMUSG00000025731	17	25603115	25604762	276	321
12991	16	620933	624117	ENSMUSG00000071192	ENSMUSG00000071192	ENSMUSG00000071192	17	25605293	25606456	276	321
12992	16	580177	619272	ENSMUSG00000025730	ENSMUSG00000025730	ENSMUSG00000025730	17	25609714	25647302	276	321
12993	16	560005	574109	ENSMUSG00000025728	ENSMUSG00000025728	ENSMUSG00000025728	17	25654024	25669562	276	321
12994	16	550423	558420	ENSMUSG00000025727	ENSMUSG00000025727	ENSMUSG00000025727	17	25670964	25676161	276	321
12995	16	517857	544635	ENSMUSG00000037326	ENSMUSG00000037326	ENSMUSG00000037326	17	25685969	25713306	276	321
12996	16	415669	512479	ENSMUSG00000037098	ENSMUSG00000037098	ENSMUSG00000037098	17	25716641	25796218	276	321
12997	16	391827	402463	ENSMUSG00000036775	ENSMUSG00000036775	ENSMUSG00000036775	17	25810476	25817643	276	321
12998	16	387193	390754	ENSMUSG00000024177	ENSMUSG00000024177	ENSMUSG00000024177	17	25819339	25823108	276	321
12999	16	361861	371908	ENSMUSG00000024180	ENSMUSG00000024180	ENSMUSG00000024180	17	25840917	25850854	276	321
13000	16	357437	360585	ENSMUSG00000024181	ENSMUSG00000024181	ENSMUSG00000024181	17	25851122	25854211	276	321
13001	16	277441	342465	ENSMUSG00000024182	ENSMUSG00000024182	ENSMUSG00000024182	17	25866334	25923411	276	321
13002	16	273171	277209	ENSMUSG00000024184	ENSMUSG00000024184	ENSMUSG00000024184	17	25923602	25926621	276	321
13003	16	270856	272809	ENSMUSG00000073433	ENSMUSG00000073433	ENSMUSG00000073433	17	25926783	25928950	276	321
13004	16	259269	265912	ENSMUSG00000024186	ENSMUSG00000024186	ENSMUSG00000024186	17	25930576	25938917	276	321
13005	16	237927	255600	ENSMUSG00000024187	ENSMUSG00000024187	ENSMUSG00000024187	17	25940313	25971741	276	321
13006	16	179198	219450	ENSMUSG00000024188	ENSMUSG00000024188	ENSMUSG00000024188	17	25980551	26009568	276	321
13007	5	172127707	172130809	ENSMUSG00000024190	ENSMUSG00000024190	ENSMUSG00000024190	17	26233191	26236061	277	322
13008	5	172193947	172312287	ENSMUSG00000001576	ENSMUSG00000001576	ENSMUSG00000001576	17	26289112	26384536	277	322
13009	5	172343369	172394506	ENSMUSG00000015575	ENSMUSG00000015575	ENSMUSG00000015575	17	26403996	26427237	277	322
13010	5	172415976	172496567	ENSMUSG00000048249	ENSMUSG00000048249	ENSMUSG00000048249	17	26467133	26499130	277	322
13011	5	172504146	172523950	ENSMUSG00000024191	ENSMUSG00000024191	ENSMUSG00000024191	17	26508814	26520252	277	322
13012	5	172591744	172594868	ENSMUSG00000015579	ENSMUSG00000015579	ENSMUSG00000015579	17	26566254	26569154	277	322
13013	6	33467291	33485679	ENSMUSG00000024301	ENSMUSG00000024301	ENSMUSG00000024301	17	26644762	26659870	278	323
13014	6	33486718	33492194	ENSMUSG00000024193	ENSMUSG00000024193	ENSMUSG00000024193	17	26660921	26665478	278	323
13015	6	33492297	33493940	ENSMUSG00000024194	ENSMUSG00000024194	ENSMUSG00000024194	17	26665562	26667068	278	323
13016	6	33495919	33533296	ENSMUSG00000067629	ENSMUSG00000067629	ENSMUSG00000067629	17	26685007	26698234	278	323
13017	6	33659493	33664781	ENSMUSG00000048731	ENSMUSG00000048731	ENSMUSG00000048731	17	26700806	26763965	278	323
13018	6	33696500	33772329	ENSMUSG00000042644	ENSMUSG00000042644	ENSMUSG00000042644	17	26785125	26849812	278	323
13019	6	33773323	33787482	ENSMUSG00000024208	ENSMUSG00000024208	ENSMUSG00000024208	17	26850256	26861483	278	323
13020	6	33797422	33822740	ENSMUSG00000024210	ENSMUSG00000024210	ENSMUSG00000024210	17	26871560	26895353	278	323
13021	6	33846977	33864890	ENSMUSG00000044857	ENSMUSG00000044857	ENSMUSG00000044857	17	26918229	26932007	278	323
13022	6	34097606	34209421	ENSMUSG00000063239	ENSMUSG00000063239	ENSMUSG00000063239	17	27149976	27230893	278	323
13023	6	34363975	34468429	ENSMUSG00000024213	ENSMUSG00000024213	ENSMUSG00000024213	17	27306980	27351048	278	323
13024	6	34541883	34610976	ENSMUSG00000040276	ENSMUSG00000040276	ENSMUSG00000040276	17	27383271	27438695	278	323
13025	6	34613557	34632069	ENSMUSG00000024215	ENSMUSG00000024215	ENSMUSG00000024215	17	27442045	27456540	278	323
13026	6	34663043	34772602	ENSMUSG00000056692	ENSMUSG00000056692	ENSMUSG00000056692	17	27481414	27548131	278	323
13027	6	34867835	34953269	ENSMUSG00000039512	ENSMUSG00000039512	ENSMUSG00000039512	17	27584096	27627629	278	323
13028	6	34953533	34963844	ENSMUSG00000024218	ENSMUSG00000024218	ENSMUSG00000024218	17	27628717	27635313	278	323
13029	6	34965019	35167166	ENSMUSG00000024219	ENSMUSG00000024219	ENSMUSG00000024219	17	27636961	27790188	278	323
13030	6	35290174	35328834	ENSMUSG00000038677	ENSMUSG00000038677	ENSMUSG00000038677	17	27870115	27898934	278	323
13031	6	35334737	35371003	ENSMUSG00000024220	ENSMUSG00000024220	ENSMUSG00000024220	17	27904796	27933474	278	323
13032	6	35373607	35397526	ENSMUSG00000002257	ENSMUSG00000002257	ENSMUSG00000002257	17	27935419	27956188	278	323
13033	6	35418320	35503931	ENSMUSG00000002250	ENSMUSG00000002250	ENSMUSG00000002250	17	27960392	28029058	278	323
13034	6	35528116	35542859	ENSMUSG00000007570	ENSMUSG00000007570	ENSMUSG00000007570	17	28041151	28054155	278	323
13035	6	35549354	35572763	ENSMUSG00000002249	ENSMUSG00000002249	ENSMUSG00000002249	17	28059264	28078189	278	323
13036	6	35573632	35588624	ENSMUSG00000037446	ENSMUSG00000037446	ENSMUSG00000037446	17	28079105	28092737	278	323
13037	6	35649345	35804336	ENSMUSG00000024222	ENSMUSG00000024222	ENSMUSG00000024222	17	28126686	28168647	278	323
13038	6	35812837	35824657	ENSMUSG00000024223	ENSMUSG00000024223	ENSMUSG00000024223	17	28258450	28266696	278	323
13039	6	35852374	35855285	ENSMUSG00000024224	ENSMUSG00000024224	ENSMUSG00000024224	17	28277074	28280207	278	323
13040	6	35870738	35873080	ENSMUSG00000024225	ENSMUSG00000024225	ENSMUSG00000024225	17	28285803	28288303	278	323
13041	6	35881048	35895202	ENSMUSG00000062252	ENSMUSG00000062252	ENSMUSG00000062252	17	28303590	28307764	278	323
13042	6	35908790	35996811	ENSMUSG00000004865	ENSMUSG00000004865	ENSMUSG00000004865	17	28317192	28349639	278	323
13043	6	36019273	36100355	ENSMUSG00000036196	ENSMUSG00000036196	ENSMUSG00000036196	17	28365916	28412477	278	323
13044	6	36103551	36186513	ENSMUSG00000053436	ENSMUSG00000053436	ENSMUSG00000053436	17	28418955	28475993	278	323
13045	6	36129769	36215283	ENSMUSG00000004864	ENSMUSG00000004864	ENSMUSG00000004864	17	28496937	28506286	278	323
13046	6	36272528	36308541	ENSMUSG00000063952	ENSMUSG00000063952	ENSMUSG00000063952	17	28538100	28564279	278	323
13047	6	36367155	36384350	ENSMUSG00000043286	ENSMUSG00000043286	ENSMUSG00000043286	17	28586456	28614537	278	323
13048	6	36391517	36412640	ENSMUSG00000048905	ENSMUSG00000048905	ENSMUSG00000048905	17	28623984	28642913	278	323
13049	6	36466309	36518644	ENSMUSG00000045378	ENSMUSG00000045378	ENSMUSG00000045378	17	28661575	28669851	278	323
13050	6	36518522	36566291	ENSMUSG00000005936	ENSMUSG00000005936	ENSMUSG00000005936	17	28680805	28695526	278	323
13051	6	36569647	36623225	ENSMUSG00000024006	ENSMUSG00000024006	ENSMUSG00000024006	17	28698474	28735604	278	323
13052	6	36754413	36763094	ENSMUSG00000023067	ENSMUSG00000023067	ENSMUSG00000023067	17	28821439	28828386	278	323
13053	6	36816533	36915756	ENSMUSG00000024008	ENSMUSG00000024008	ENSMUSG00000024008	17	28883831	28964809	278	323
13054	6	36930581	36950778	ENSMUSG00000024007	ENSMUSG00000024007	ENSMUSG00000024007	17	28977847	28990983	278	323
13055	6	36961727	37002294	ENSMUSG00000052712	ENSMUSG00000052712	ENSMUSG00000052712	17	28995991	29029893	278	323
13056	6	37030187	37040591	ENSMUSG00000024011	ENSMUSG00000024011	ENSMUSG00000024011	17	29046245	29056425	278	323
13057	6	37081400	37104052	ENSMUSG00000024013	ENSMUSG00000024013	ENSMUSG00000024013	17	29087926	29106565	278	323
13058	6	37245964	37251180	ENSMUSG00000024014	ENSMUSG00000024014	ENSMUSG00000024014	17	29217824	29222496	278	323
13059	6	37333526	37408724	ENSMUSG00000042203	ENSMUSG00000042203	ENSMUSG00000042203	17	29276814	29333820	278	323
13060	6	37508974	37557260	ENSMUSG00000024019	ENSMUSG00000024019	ENSMUSG00000024019	17	29387613	29430371	278	323
13061	6	37558686	37575676	ENSMUSG00000024018	ENSMUSG00000024018	ENSMUSG00000024018	17	29425333	29443997	278	323
13062	6	37895285	38230375	ENSMUSG00000044477	ENSMUSG00000044477	ENSMUSG00000044477	17	29732248	29935414	278	323

13063	6	38798600	39106279	ENSMUSG00000033826	ENSMUSG00000033826	ENSMUSG00000033826	17	30351421	30602276	278	323		
13064	6	39124552	39163497	ENSMUSG00000024027	ENSMUSG00000024027	ENSMUSG00000024027	17	30628889	30663545	278	323		
13065	21	42364495	42436174	ENSMUSG00000054134	ENSMUSG00000054134	ENSMUSG00000054134	17	30681691	30737722	279	324		
13066	21	42549263	42590421	ENSMUSG00000024030	ENSMUSG00000024030	ENSMUSG00000024030	17	30784710	30842784	279	324		
13067	21	42605233	42608775	ENSMUSG00000024029	ENSMUSG00000024029	ENSMUSG00000024029	17	30852327	30856623	279	324		
13068	21	42639538	42644176	ENSMUSG00000024028	ENSMUSG00000024028	ENSMUSG00000024028	17	30868938	30871294	279	324		
13069	21	42655462	42659713	ENSMUSG00000024032	ENSMUSG00000024032	ENSMUSG00000024032	17	30872609	30892065	279	324		
13070	21	42665069	42690024	ENSMUSG00000024034	ENSMUSG00000024034	ENSMUSG00000024034	17	30908773	30924769	279	324		
13071	21	42765668	42789533	ENSMUSG00000024033	ENSMUSG00000024033	ENSMUSG00000024033	17	30983729	31006066	279	324		
13072	21	42807093	42874599	ENSMUSG00000024036	ENSMUSG00000024036	ENSMUSG00000024036	17	31029162	31078133	279	324		
13073	21	42946931	43068685	ENSMUSG00000041119	ENSMUSG00000041119	ENSMUSG00000041119	17	31114961	31205019	279	324		
13074	21	43136275	43172747	ENSMUSG00000024037	ENSMUSG00000024037	ENSMUSG00000024037	17	31224334	31240967	279	324		
13075	21	43186447	43202841	ENSMUSG00000024038	ENSMUSG00000024038	ENSMUSG00000024038	17	31248868	31260036	279	324		
13076	21	43267712	43326757	ENSMUSG00000006705	ENSMUSG00000006705	ENSMUSG00000006705	17	31293511	31334756	279	324		
13077	21	43346374	43369541	ENSMUSG00000024039	ENSMUSG00000024039	ENSMUSG00000024039	17	31341357	31365875	279	324		
13078	21	43462210	43465982	ENSMUSG00000024041	ENSMUSG00000024041	ENSMUSG00000024041	17	31406661	31410057	279	324		
13079	21	43658829	43671436	ENSMUSG00000024042	ENSMUSG00000024042	ENSMUSG00000024042	17	31572967	31584494	279	324		
13080	21	43773501	43903802	ENSMUSG00000002076	ENSMUSG00000002076	ENSMUSG00000002076	17	31673479	31763595	279	324		
13081	21	43903860	43940388	ENSMUSG00000058392	ENSMUSG00000058392	ENSMUSG00000058392	17	31765265	31789709	279	324		
13082	19	15131445	15172792	ENSMUSG00000038146	ENSMUSG00000038146	ENSMUSG00000038146	17	31849599	31895558	280	325		
13083	19	15198731	15204231	ENSMUSG00000037577	ENSMUSG00000037577	ENSMUSG00000037577	17	31912476	31918169	280	325		
13084	19	15209301	15252262	ENSMUSG00000024002	ENSMUSG00000024002	ENSMUSG00000024002	17	31924980	32013849	280	325		
13085	19	15325341	15351603	ENSMUSG00000024045	ENSMUSG00000024045	ENSMUSG00000024045	17	32036865	32050418	280	325		
13086	19	15351862	15390799	ENSMUSG00000002625	ENSMUSG00000002625	ENSMUSG00000002625	17	32053100	32077025	280	325		
13087	19	15394890	15412420	ENSMUSG00000024050	ENSMUSG00000024050	ENSMUSG00000024050	17	32085677	32109848	280	325		
13088	19	15497144	15524127	ENSMUSG00000061126	ENSMUSG00000061126	ENSMUSG00000061126	17	32186858	32228975	280	325		
13089	19	8551126	8579147	ENSMUSG00000024299	ENSMUSG00000024299	ENSMUSG00000024299	17	33135370	33160455	281	326		
13090	19	8491689	8548330	ENSMUSG00000024300	ENSMUSG00000024300	ENSMUSG00000024300	17	33182479	33214063	281	326		
13091	19	8482224	8485032	ENSMUSG00000073423	ENSMUSG00000073423	ENSMUSG00000073423	17	33236485	33238294	281	326		
13092	19	8460941	8473495	ENSMUSG00000032739	ENSMUSG00000032739	ENSMUSG00000032739	17	33247152	33252263	281	326		
13093	19	8361229	8375309	ENSMUSG00000002290	ENSMUSG00000002290	ENSMUSG00000002290	17	33353479	33367182	281	326		
13094	19	8335011	8345257	ENSMUSG00000002289	ENSMUSG00000002289	ENSMUSG00000002289	17	33380630	33388455	281	326		
13095	19	8294282	8314146	ENSMUSG00000042099	ENSMUSG00000042099	ENSMUSG00000042099	17	33416632	33428874	281	326		
13096	19	8282234	8292280	ENSMUSG00000041881	ENSMUSG00000041881	ENSMUSG00000041881	17	33430562	33444388	281	326		
13097	19	8273011	8279239	ENSMUSG00000002308	ENSMUSG00000002308	ENSMUSG00000002308	17	33449214	33455642	281	326		
13098	6	33394313	33398769	ENSMUSG00000002307	ENSMUSG00000002307	ENSMUSG00000002307	17	33519969	33525972	282	327		
13099	6	33390174	33393488	ENSMUSG00000051390	ENSMUSG00000051390	ENSMUSG00000051390	17	33526652	33529778	282	327		
13100	6	33375449	33390142	ENSMUSG00000024308	ENSMUSG00000024308	ENSMUSG00000024308	17	33529932	33537169	282	327		
13101	6	33367409	33375059	ENSMUSG00000041354	ENSMUSG00000041354	ENSMUSG00000041354	17	33540348	33548141	282	327		
13102	6	33365352	33366629	ENSMUSG00000024309	ENSMUSG00000024309	ENSMUSG00000024309	17	33549375	33550785	282	327		
13103	6	33354860	33365048	ENSMUSG00000024312	ENSMUSG00000024312	ENSMUSG00000024312	17	33551177	33560151	282	327		
13104	6	33352951	33354579	ENSMUSG00000067370	ENSMUSG00000067370	ENSMUSG00000067370	17	33560601	33561716	282	327		
13105	6	33326034	33347630	ENSMUSG00000024319	ENSMUSG00000024319	ENSMUSG00000024319	17	33566266	33577438	282	327		
13106	6	33284255	33288477	ENSMUSG00000024325	ENSMUSG00000024325	ENSMUSG00000024325	17	33631538	33635097	282	327		
13107	6	33280397	33282586	ENSMUSG00000073422	ENSMUSG00000073422	ENSMUSG00000073422	17	33636488	33638509	282	327		
13108	6	33276631	33280192	ENSMUSG00000024327	ENSMUSG00000024327	ENSMUSG00000024327	17	33638727	33642012	282	327		
13109	6	33269343	33276417	ENSMUSG00000039656	ENSMUSG00000039656	ENSMUSG00000039656	17	33642306	33648853	282	327		
13110	6	33238447	33268223	ENSMUSG00000024330	ENSMUSG00000024330	ENSMUSG00000024330	17	33649891	33676696	282	327		
13111	6	33167508	33173050	ENSMUSG00000024333	ENSMUSG00000024333	ENSMUSG00000024333	17	33695069	33696277	282	327		
13112	6	33079937	33085367	ENSMUSG00000024334	ENSMUSG00000024334	ENSMUSG00000024334	17	33702901	33705273	282	327		
13113	6	33044415	33057059	ENSMUSG00000024335	ENSMUSG00000024335	ENSMUSG00000024335	17	33722473	33730354	282	327		
13114	6	33024367	33028877	ENSMUSG00000037649	ENSMUSG00000037649	ENSMUSG00000037649	17	33746125	33748991	282	327		
13115	6	33010384	33016795	ENSMUSG00000037548	ENSMUSG00000037548	ENSMUSG00000037548	17	33756075	33761497	282	327		
13116	6	32929916	32935606	ENSMUSG00000024337	ENSMUSG00000024337	ENSMUSG00000024337	17	33792336	33797608	282	327		
13117	6	32920804	32929729	ENSMUSG00000037321	ENSMUSG00000037321	ENSMUSG00000037321	17	33798022	33807437	282	327		
13118	6	32916471	32919802	ENSMUSG00000024338	ENSMUSG00000024338	ENSMUSG00000024338	17	33808583	33811657	282	327		
13119	6	32901165	32914525	ENSMUSG00000024339	ENSMUSG00000024339	ENSMUSG00000024339	17	33814426	33826594	282	327		
13120	6	32888518	32892803	ENSMUSG00000041538	ENSMUSG00000041538	ENSMUSG00000041538	17	33850627	33862896	282	327		
13121	6	32515597	32520943	ENSMUSG00000036322	ENSMUSG00000036322	ENSMUSG00000036322	17	33950514	33952226	282	327		
13122	6	32470491	32482878	ENSMUSG00000024340	ENSMUSG00000024340	ENSMUSG00000024340	17	33962403	33977115	282	327		
13123	6	32270599	32299822	ENSMUSG00000015468	ENSMUSG00000015468	ENSMUSG00000015468	17	34172386	34196554	282	327		
13124	6	32256724	32260001	ENSMUSG00000015452	ENSMUSG00000015452	ENSMUSG00000015452	17	34205918	34208988	282	327		
13125	6	32254047	32256548	ENSMUSG00000015478	ENSMUSG00000015478	ENSMUSG00000015478	17	34209153	34211627	282	327		
13126	6	32243963	32253820	ENSMUSG00000034254	ENSMUSG00000034254	ENSMUSG00000034254	17	34213976	34221477	282	327		
13127	6	32229202	32239436	ENSMUSG00000015474	ENSMUSG00000015474	ENSMUSG00000015474	17	34224720	34235402	282	327		
13128	6	32204462	32206045	ENSMUSG00000033739	ENSMUSG00000033739	ENSMUSG00000033739	17	34252939	34254383	282	327		
13129	6	32084175	32185131	ENSMUSG00000033327	ENSMUSG00000033327	ENSMUSG00000033327	17	34278591	34327683	282	327		
13130	6	32114072	32117426	ENSMUSG00000024365	ENSMUSG00000024365	ENSMUSG00000024365	17	34409836	34412455	282	327		
13131	6	32047625	32057202	ENSMUSG00000061207	ENSMUSG00000061207	ENSMUSG00000061207	17	34432049	34444959	282	327		
13132	6	32045566	32048040	ENSMUSG00000040482	ENSMUSG00000040482	ENSMUSG00000040482	17	34445139	34447289	282	327		
13133	6	32034560	32045509	ENSMUSG00000040356	ENSMUSG00000040356	ENSMUSG00000040356	17	34447282	34458260	282	327		
13134	6	32027844	32034843	ENSMUSG00000024369	ENSMUSG00000024369	ENSMUSG00000024369	17	34458557	34464310	282	327		
13135	6	32021465	32027840	ENSMUSG00000024371	ENSMUSG00000024371	ENSMUSG00000024371	17	34464437	34470273	282	327		
13136	6	32003239	32021430	ENSMUSG00000024372	ENSMUSG00000024372	ENSMUSG00000024372	17	34470664	34490119	282	327		
13137	6	31955515	31973443	ENSMUSG00000013787	ENSMUSG00000013787	ENSMUSG00000013787	17	34507030	34522101	282	327		
13138	6	31939175	31954736	ENSMUSG00000007034	ENSMUSG00000007034	ENSMUSG00000007034	17	34522536	34538415	282	327		
13139	6	31934931	31938662	ENSMUSG00000007038	ENSMUSG00000007038	ENSMUSG00000007038	17	34539370	34544009	282	327		
13140	6	31885493	31890821	ENSMUSG00000007033	ENSMUSG00000007033	ENSMUSG00000007033	17	34580788	34587281	282	327		
13141	6	31873152	31882731	ENSMUSG00000007050	ENSMUSG00000007050	ENSMUSG00000007050	17	34590193	34593945	282	327		
13142	6	31853274	31871565	ENSMUSG00000007029	ENSMUSG00000007029	ENSMUSG00000007029	17	34609189	34624382	282	327		
13143	6	31841495	31852535	ENSMUSG00000007030	ENSMUSG00000007030	ENSMUSG00000007030	17	34624635	34634797	282	327		
13144	6	31815776	31840601	ENSMUSG00000007035	ENSMUSG00000007035	ENSMUSG00000007035	17	34636661	34654678	282	327		
13145	6	31802766	31806373	ENSMUSG00000007039	ENSMUSG00000007039	ENSMUSG00000007039	17	34667141	34670147	282	327		
13146	6	31799129	31802468	ENSMUSG00000073414	ENSMUSG00000073414	ENSMUSG00000073414	17	34670749	34674240	282	327		
13147	6	31794404	31797489	ENSMUSG00000007044	ENSMUSG00000007044	ENSMUSG00000007044	17	34675381	34678104	282	327		
13148	6	31788092	31789568	ENSMUSG00000013766	ENSMUSG00000013766	ENSMUSG00000013766	17	34685142	34686860	282	327		
13149	6	31782660	31793674	ENSMUSG00000034923	ENSMUSG00000034923	ENSMUSG00000034923	17	34688738	34693630	282	327		
13150	6	31762715	31779172	ENSMUSG00000007036	ENSMUSG00000007036	ENSMUSG00000007036	17	34697381	34710809	282	327		
13151	6	31752713	31759796	ENSMUSG00000034482	ENSMUSG00000034482	ENSMUSG00000034482	17	34716356	34720009	282	327		
13152	6	31740992	31745769	ENSMUSG00000024387	ENSMUSG00000024387	ENSMUSG00000024387	17	34724251	34729503	282	327		
13153	6	31734054	31736528	ENSMUSG00000043311	ENSMUSG00000043311	ENSMUSG00000043311	17	34735171	34736052	282	327		
13154	6	31731650	31733966	ENSMUSG00000024391	ENSMUSG00000024391	ENSMUSG00000024391	17	34737057	34739852	282	327		
13155	6	31714797	31728426	ENSMUSG00000024392	ENSMUSG00000024392	ENSMUSG00000024392	17	34743298	34755292	282	327		
13156	6	31696471	31713528	ENSMUSG00000024393	ENSMUSG00000024393	ENSMUSG00000024393	17	34757144	34772909	282	327		
13157	6	31691644	31692781	ENSMUSG00000024397	ENSMUSG00000024397	ENSMUSG00000024397	17	34779048	34781334	282	327		
13158	6	31656314	31658183	ENSMUSG00000024399	ENSMUSG00000024399	ENSMUSG00000024399	17	34802574	34804354	282	327		
13159	6	31651314	31654092	ENSMUSG00000024401	ENSMUSG00000024401	ENSMUSG00000024401	17	34807442	34810048	282	327		
13160	6	31648042	31650080	ENSMUSG00000024402	ENSMUSG00000024402	ENSMUSG00000024402	17	34811218	34813403	282	327		
13161	6	31623337	31634585	ENSMUSG00000042419	ENSMUSG00000042419	ENSMUSG00000042419	17	34828227	34843867	282	327		
13162	6	31605975	31622338	ENSMUSG00000019432	ENSMUSG00000019432	ENSMUSG00000019432	17	34849866	34861733	282	327		
13163	6	31234294	31239534	ENSMUSG00000050410	ENSMUSG00000050410	ENSMUSG00000050410	17	35122523	35124168	282	327		
13164	6	31218195	31233961	ENSMUSG00000040312	ENSMUSG00000040312	ENSMUSG00000040312	17	35125229	35139061	282	327		
13165	6	31213292	31215106	ENSMUSG00000024409	ENSMUSG00000024409	ENSMUSG00000024409	17	35141293	35142698	282	327		
13166	6	31190849	31196202	ENSMUSG00000039518	ENSMUSG00000039518	ENSMUSG00000039518	17	35160194	35165236	282	327		
13167	6	31186982	31188315	ENSMUSG00000039269	ENSMUSG00000039269	ENSMUSG00000039269	17	35175538	35176998	282	327		
13168	6	31026879	31029977	ENSMUSG00000073408	ENSMUSG00000073408	ENSMUSG00000073408	17	35243808	35251748	282	327		
13169	6	30983998	31002212	ENSMUSG00000038838	ENSMUSG00000038838	ENSMUSG00000038838	17	35264443	35275645	282	327		
13170	6	30631412	30975908	ENSMUSG00000003534	ENSMUSG00000003534	ENSMUSG00000003534	17	35289621	35308541	282			
13171	6	30818955	30820303	ENSMUSG00000003541	ENSMUSG00000003541	ENSMUSG00000003541	17	35429737	35430969	282	328	here	
13172	6	30803465	30818443	ENSMUSG00000059714	ENSMUSG00000059714	ENSMUSG00000059714	17	35431400	35440840	282	328	Single marker moved to different place with in a HSB	1 -> 2
13173	6	30796101	30801178	ENSMUSG00000001525	ENSMUSG00000001525	ENSMUSG00000001525	17	35442860	35446285	282	328		
13174	6	30775563	30793645	ENSMUSG00000061607	ENSMUSG00000061607	ENSMUSG00000061607	17	35452488	35467722	282	328		
13175	6	30763805	30766730	ENSMUSG00000059791	ENSMUSG00000059791	ENSMUSG00000059791	17	35469371	35473455	282	328		
13176	6	30752147	30763651	ENSMUSG00000034595	ENSMUSG00000034595	ENSMUSG00000034595	17	35473646	35482869	282	328		
13177	6	30728866	30748760	ENSMUSG00000024422	ENSMUSG00000024422	ENSMUSG00000024422	17	35487872	35500723	282	328		
13178	6	30722829	30728858	ENSMUSG00000050705	ENSMUSG00000050705	ENSMUSG00000050705	17	35500740	35505173	282	328		
13179	6	30702612	30722582	ENSMUSG00000024426	ENSMUSG00000024426	ENSMUSG00000024426	17	35505653	35518110	282	328		
13180	6	30693585	30702148	ENSMUSG00000024436	ENSMUSG00000024436	ENSMUSG00000024436	17	35518434	35524429	282	328		
13181	6	30676156	30692756	ENSMUSG00000039220	ENSMUSG00000039220	ENSMUSG00000039220	17	35525249	35540325	282	328		
13182	6	30647149	30667286	ENSMUSG00000038762	ENSMUSG00000038762	ENSMUSG00000038762	17	35564872	35577803	282	328		
13183	6	30632287	30639477	ENSMUSG00000038500	ENSMUSG00000038500	ENSMUSG00000038500	17	35580592	35588289	282	328		
13184	6	30621633	30632012	ENSMUSG00000024429	ENSMUSG00000024429	ENSMUSG00000024429	17	35588049	35597515	282	328		
13185	6	30420928	30422649	ENSMUSG00000024446	ENSMUSG00000024446	ENSMUSG00000024446	17	35863698	35865909	282	328		
13186	6	30403019	30419484	ENSMUSG00000045409	ENSMUSG00000045409	ENSMUSG00000045409	17	35866925	35880057	282	328		
13187	6	30260211	30281426	ENSMUSG00000024457	ENSMUSG00000024457	ENSMUSG00000024457	17	36445239	36467433	282	328		
13188	6	30238972	30248452	ENSMUSG00000050747	ENSMUSG00000050747	ENSMUSG00000050747	17	36470375	36475154	282	328		
13189	6	30227701	30236690	ENSMUSG00000073400	ENSMUSG00000073400	ENSMUSG00000073400	17	36477627	36485878	282	328		
13190	6	30212487	30224491	ENSMUSG00000073399	ENSMUSG00000073399	ENSMUSG00000073399	17	36489651	36498098	282	328		
13191	6	30178653	30188646	ENSMUSG00000058063	ENSMUSG00000058063	ENSMUSG00000058063	17	36506171	36518267	282	328		
13192	6	30146026	30151608	ENSMUSG00000036492	ENSMUSG00000036492	ENSMUSG00000036492	17	36551031	36555689	282	328		
13193	6	30142968	30146089	ENSMUSG00000036398	ENSMUSG00000036398	ENSMUSG00000036398	17	36556801	36559727	282	328		
13194	6	30137015	30140665	ENSMUSG00000036315	ENSMUSG00000036315	ENSMUSG00000036315	17	36562411	36566535	282	328		
13195	6	29748239	29756866	ENSMUSG00000036036	ENSMUSG00000036036	ENSMUSG00000036036	17	36610595	36618660	282	328		
13196	6	29732848	29748128	ENSMUSG00000076439	ENSMUSG00000076439	ENSMUSG00000076439	17	36618799	36631451	282	328		
13197	6	29677986	29708916	ENSMUSG00000024462	ENSMUSG00000024462	ENSMUSG00000024462	17	36654748	36681079	282	328		
13198	6	29515062	29516710	ENSMUSG00000049561	ENSMUSG00000049561	ENSMUSG00000049561	17	36818966	36819904	282	328		
13199	6	29449179	29525854	ENSMUSG00000029184	ENSMUSG00000029184	ENSMUSG00000029184	17	37074261	37075205	282	328		
13200	6	29382446	29383411	ENSMUSG00000050613	ENSMUSG00000050613	ENSMUSG00000050613	17	37443054	37444016	282	328		
13201	6	49861606	49862859	ENSMUSG00000031233	ENSMUSG00000031233	ENSMUSG00000031233	17	39670753	39672006	283	329		
13202	6	49768043	49789258	ENSMUSG00000023930	ENSMUSG00000023930	ENSMUSG00000023930	17	40228219	40257582	283	329		
13203	6	49680830	49712511	ENSMUSG00000023926	ENSMUSG00000023926	ENSMUSG00000023926	17	40274633	40304238	283	329		
13204	6	49626347	49637576	ENSMUSG00000054951	ENSMUSG00000054951	ENSMUSG00000054951	17	40338966	40344036	283	329		
13205	6	49587663	49602565	ENSMUSG00000071081	ENSMUSG00000071081	ENSMUSG00000071081	17	40368012	40376209	283	329		
13206	6	49539055	49568779	ENSMUSG00000023919	ENSMUSG00000023919	ENSMUSG00000023919	17	40386539	40398035	283	329		
13207	6	49506958	49538811	ENSMUSG00000023921	ENSMUSG00000023921	ENSMUSG00000023921	17	40398191	40424383	283	329		
13208	6	47953998	47955530	ENSMUSG00000042256	ENSMUSG00000042256	ENSMUSG00000042256	17	41965867	41967399	283	329		
13209	6	47857757	47902073	ENSMUSG00000043972	ENSMUSG00000043972	ENSMUSG00000043972	17	42020267	42074797	283	329		
13210	6	47761559	47797716	ENSMUSG00000023918	ENSMUSG00000023918	ENSMUSG00000023918	17	42120375	42155768	283	329		
13211	6	47732182	47758381	ENSMUSG00000057899	ENSMUSG00000057899	ENSMUSG00000057899	17	42172421	42205663	283	329		
13212	6	47553899	47702620	ENSMUSG00000061665	ENSMUSG00000061665	ENSMUSG00000061665	17	42259812	42316016	283	329		
13213	6	47307228	47385639	ENSMUSG00000023915	ENSMUSG00000023915	ENSMUSG00000023915	17	42480039	42552546	283	329		
13214	6	47075773	47104756	ENSMUSG00000041293	ENSMUSG00000041293	ENSMUSG00000041293	17	42735599	42789390	283	329		
13215	6	46928298	47030639	ENSMUSG00000056492	ENSMUSG00000056492	ENSMUSG00000056492	17	42891663	42921412	283	329		
13216	6	46869085	46915478	ENSMUSG00000023914	ENSMUSG00000023914	ENSMUSG00000023914	17	42938373	42967156	283	329		
13217	6	46779897	46811038	ENSMUSG00000023913	ENSMUSG00000023913	ENSMUSG00000023913	17	43031500	43075242	283	329		
13218	6	46763825	46778109	ENSMUSG00000040140	ENSMUSG00000040140	ENSMUSG00000040140	17	43078390	43093354	283	329		
13219	6	46728637	46753889	ENSMUSG00000023912	ENSMUSG00000023912	ENSMUSG00000023912	17	43104955	43130215	283	329		
13220	6	46625405	46728482	ENSMUSG00000023963	ENSMUSG00000023963	ENSMUSG00000023963	17	43130480	43214486	283	329		
13221	6	46296434	46567668	ENSMUSG00000039601	ENSMUSG00000039601	ENSMUSG00000039601	17	43264906	43502571	283	329		
13222	6	46234885	46246676	ENSMUSG00000023960	ENSMUSG00000023960	ENSMUSG00000023960	17	43541924	43549614	283	329		
13223	6	46205689	46222237	ENSMUSG00000023961	ENSMUSG00000023961	ENSMUSG00000023961	17	43559364	43568863	283	329		
13224	6	45977383	46156044	ENSMUSG00000023959	ENSMUSG00000023959	ENSMUSG00000023959	17	43651627	43743223	283	329		
13225	6	45404038	45626794	ENSMUSG00000039153	ENSMUSG00000039153	ENSMUSG00000039153	17	44070960	44198674	283	329		
13226	6	44904447	45453648	ENSMUSG00000038954	ENSMUSG00000038954	ENSMUSG00000038954	17	44249665	44511708	283	329		
13227	6	44418464	44452881	ENSMUSG00000023935	ENSMUSG00000023935	ENSMUSG00000023935	17	44909462	44928162	283	329		
13228	6	44375166	44389041	ENSMUSG00000023938	ENSMUSG00000023938	ENSMUSG00000023938	17	44970422	44984424	283	329		
13229	6	44354458	44373403	ENSMUSG00000023949	ENSMUSG00000023949	ENSMUSG00000023949	17	44996634	45006261	283	329		
13230	6	44346458	44352239	ENSMUSG00000050405	ENSMUSG00000050405	ENSMUSG00000050405	17	45005533	45013258	283	329		
13231	6	44333881	44341503	ENSMUSG00000023947	ENSMUSG00000023947	ENSMUSG00000023947	17	45019297	45026752	283	329		
13232	6	44330421	44333243	ENSMUSG00000037089	ENSMUSG00000037089	ENSMUSG00000037089	17	45027545	45031250	283	329		
13233	6	44324345	44329353	ENSMUSG00000023944	ENSMUSG00000023944	ENSMUSG00000023944	17	45031596	45035492	283	329		
13234	6	44295301	44309859	ENSMUSG00000023942	ENSMUSG00000023942	ENSMUSG00000023942	17	45048784	45057184	283	329		
13235	6	44234526	44260117	ENSMUSG00000058626	ENSMUSG00000058626	ENSMUSG00000058626	17	45094166	45117341	283	329		
13236	6	44202629	44231233	ENSMUSG00000036026	ENSMUSG00000036026	ENSMUSG00000036026	17	45124312	45143271	283	329		
13237	6	44189172	44203172	ENSMUSG00000023939	ENSMUSG00000023939	ENSMUSG00000023939	17	45149953	45162075	283	329		
13238	6	43845931	43862199	ENSMUSG00000023951	ENSMUSG00000023951	ENSMUSG00000023951	17	45480574	45495331	283	329		
13239	6	43747020	43763506	ENSMUSG00000023967	ENSMUSG00000023967	ENSMUSG00000023967	17	45574585	45592489	283	329		
13240	6	43720761	43748314	ENSMUSG00000023966	ENSMUSG00000023966	ENSMUSG00000023966	17	45592858	45607779	283	329		
13241	6	43708910	43716664	ENSMUSG00000034509	ENSMUSG00000034509	ENSMUSG00000034509	17	45610970	45617117	283	329		
13242	6	43696197	43704914	ENSMUSG00000023952	ENSMUSG00000023952	ENSMUSG00000023952	17	45624652	45632951	283	329		
13243	6	43651865	43691375	ENSMUSG00000023953	ENSMUSG00000023953	ENSMUSG00000023953	17	45634936	45666176	283	329		
13244	6	43598053	43651729	ENSMUSG00000067150	ENSMUSG00000067150	ENSMUSG00000067150	17	45666363	45707178	283	329		
13245	6	43592769	43597218	ENSMUSG00000067148	ENSMUSG00000067148	ENSMUSG00000067148	17	45707509	45711547	283	329		
13246	6	43587544	43592680	ENSMUSG00000071074	ENSMUSG00000071074	ENSMUSG00000071074	17	45711674	45715597	283	329		
13247	6	43582685	43586402	ENSMUSG00000071073	ENSMUSG00000071073	ENSMUSG00000071073	17	45717926	45720644	283	329		
13248	6	43553289	43582269	ENSMUSG00000012296	ENSMUSG00000012296	ENSMUSG00000012296	17	45721457	45746594	283	329		
13249	6	43526068	43531306	ENSMUSG00000047428	ENSMUSG00000047428	ENSMUSG00000047428	17	45760792	45766439	283	329		
13250	6	43503270	43526142	ENSMUSG00000032842	ENSMUSG00000032842	ENSMUSG00000032842	17	45766762	45791226	283	329		
13251	6	43411787	43445159	ENSMUSG00000015597	ENSMUSG00000015597	ENSMUSG00000015597	17	45849929	45877074	283	329		
13252	6	43381682	43384508	ENSMUSG00000023968	ENSMUSG00000023968	ENSMUSG00000023968	17	45892131	45894604	283	329		
13253	6	43373980	43381254	ENSMUSG00000067144	ENSMUSG00000067144	ENSMUSG00000067144	17	45895372	45901620	283	329		
13254	6	43319200	43363975	ENSMUSG00000015599	ENSMUSG00000015599	ENSMUSG00000015599	17	45908368	45947446	283	329		
13255	6	43301345	43305188	ENSMUSG00000040658	ENSMUSG00000040658	ENSMUSG00000040658	17	45959957	45962807	283	329		
13256	6	43257900	43300303	ENSMUSG00000040327	ENSMUSG00000040327	ENSMUSG00000040327	17	45963799	46001002	283	329		
13257	6	43246898	43257219	ENSMUSG00000015605	ENSMUSG00000015605	ENSMUSG00000015605	17	46010028	46019351	283	329		

13258	6	43152007	43237435	ENSMUSG00000023972	ENSMUSG00000023972	ENSMUSG00000023972	17	46028524	46054995	283	329
13259	6	43135607	43150811	ENSMUSG00000003546	ENSMUSG00000003546	ENSMUSG00000003546	17	46093820	46108333	283	329
13260	6	43129844	43135220	ENSMUSG00000002767	ENSMUSG00000002767	ENSMUSG00000002767	17	46109437	46113323	283	329
13261	6	43113336	43129632	ENSMUSG00000038545	ENSMUSG00000038545	ENSMUSG00000038545	17	46113529	46127555	283	329
13262	6	43097351	43105312	ENSMUSG00000023971	ENSMUSG00000023971	ENSMUSG00000023971	17	46130633	46137478	283	329
13263	6	43089955	43097010	ENSMUSG00000063576	ENSMUSG00000063576	ENSMUSG00000063576	17	46137745	46144095	283	329
13264	6	43087943	43089596	ENSMUSG00000002768	ENSMUSG00000002768	ENSMUSG00000002768	17	46144300	46146299	283	329
13265	6	43060296	43088054	ENSMUSG00000059409	ENSMUSG00000059409	ENSMUSG00000059409	17	46146183	46168138	283	329
13266	6	43039586	43054936	ENSMUSG00000002763	ENSMUSG00000002763	ENSMUSG00000002763	17	46174654	46188732	283	329
13267	6	43036474	43039596	ENSMUSG00000002769	ENSMUSG00000002769	ENSMUSG00000002769	17	46188855	46192356	283	329
13268	6	43004916	43015003	ENSMUSG00000023973	ENSMUSG00000023973	ENSMUSG00000023973	17	46198896	46215391	283	329
13269	6	42991705	43001551	ENSMUSG00000036858	ENSMUSG00000036858	ENSMUSG00000036858	17	46219418	46226805	283	329
13270	6	42966199	42966504	ENSMUSG00000062619	ENSMUSG00000062619	ENSMUSG00000062619	17	46235826	46236587	283	329
13271	6	42822674	42944106	ENSMUSG00000036568	ENSMUSG00000036568	ENSMUSG00000036568	17	46263843	46295505	283	329
13272	6	42820197	42821812	ENSMUSG00000036430	ENSMUSG00000036430	ENSMUSG00000036430	17	46353812	46355654	283	329
13273	6	42772318	42798290	ENSMUSG00000023978	ENSMUSG00000023978	ENSMUSG00000023978	17	46373676	46388117	283	329
13274	6	42639778	42769220	ENSMUSG00000023977	ENSMUSG00000023977	ENSMUSG00000023977	17	46391482	46473684	283	329
13275	6	42300941	42527761	ENSMUSG00000064043	ENSMUSG00000064043	ENSMUSG00000064043	17	46604133	46822642	283	329
13276	6	42259001	42270672	ENSMUSG00000023979	ENSMUSG00000023979	ENSMUSG00000023979	17	46750145	46856152	283	329
13277	6	42282517	42293581	ENSMUSG00000034729	ENSMUSG00000034729	ENSMUSG00000034729	17	46832083	46841872	283	329
13278	6	42231152	42255768	ENSMUSG00000023982	ENSMUSG00000023982	ENSMUSG00000023982	17	46857749	46863775	283	329
13279	6	42177963	42180645	ENSMUSG00000034382	ENSMUSG00000034382	ENSMUSG00000034382	17	46931567	46933828	283	329
13280	6	42126238	42163177	ENSMUSG00000023980	ENSMUSG00000023980	ENSMUSG00000023980	17	46951473	46965478	283	329
13281	6	42010649	42017530	ENSMUSG00000034165	ENSMUSG00000034165	ENSMUSG00000034165	17	46968322	47062874	283	329
13282	6	41996943	42008760	ENSMUSG00000023988	ENSMUSG00000023988	ENSMUSG00000023988	17	47063271	47074675	283	329
13283	6	41981070	41996855	ENSMUSG00000073387	ENSMUSG00000073387	ENSMUSG00000073387	17	47074787	47087609	283	329
13284	6	41872214	41882699	ENSMUSG00000023984	ENSMUSG00000023984	ENSMUSG00000023984	17	47093881	47147258	283	329
13285	6	41845892	41855616	ENSMUSG00000023266	ENSMUSG00000023266	ENSMUSG00000023266	17	47160378	47167477	283	329
13286	6	41812427	41823113	ENSMUSG00000023987	ENSMUSG00000023987	ENSMUSG00000023987	17	47190033	47197669	283	329
13287	6	41759694	41811975	ENSMUSG00000023990	ENSMUSG00000023990	ENSMUSG00000023990	17	47248935	47255607	283	329
13288	6	41712908	41729907	ENSMUSG00000032717	ENSMUSG00000032717	ENSMUSG00000032717	17	47283005	47296222	283	329
13289	6	41622142	41678099	ENSMUSG00000023991	ENSMUSG00000023991	ENSMUSG00000023991	17	47330538	47387634	283	329
13290	6	41351690	41362435	ENSMUSG00000042265	ENSMUSG00000042265	ENSMUSG00000042265	17	47698178	47712333	283	329
13291	6	41265993	41276901	ENSMUSG00000071068	ENSMUSG00000071068	ENSMUSG00000071068	17	47765507	47776658	283	329
13292	6	41234231	41238892	ENSMUSG00000023992	ENSMUSG00000023992	ENSMUSG00000023992	17	47811968	47817840	283	329
13293	6	41224979	41230014	ENSMUSG00000023993	ENSMUSG00000023993	ENSMUSG00000023993	17	47825483	47832713	283	329
13294	6	41148662	41175693	ENSMUSG00000023994	ENSMUSG00000023994	ENSMUSG00000023994	17	47852463	47875332	283	329
13295	6	41142510	41148294	ENSMUSG00000040771	ENSMUSG00000040771	ENSMUSG00000040771	17	47875648	47882353	283	329
13296	6	41128991	41140233	ENSMUSG00000040694	ENSMUSG00000040694	ENSMUSG00000040694	17	47884798	47898295	283	329
13297	6	41118271	41120054	ENSMUSG00000023995	ENSMUSG00000023995	ENSMUSG00000023995	17	47914002	47917068	283	329
13298	6	41102750	41114906	ENSMUSG00000043592	ENSMUSG00000043592	ENSMUSG00000043592	17	47920490	47934255	283	329
13299	6	40467303	40663182	ENSMUSG00000040490	ENSMUSG00000040490	ENSMUSG00000040490	17	48397946	48563155	283	329
13300	6	39981077	40010151	ENSMUSG00000064120	ENSMUSG00000064120	ENSMUSG00000064120	17	48894009	48920354	283	329
13301	6	39868137	39980622	ENSMUSG00000040260	ENSMUSG00000040260	ENSMUSG00000040260	17	48923992	49029920	283	329
13302	3	16332357	16530226	ENSMUSG00000039316	ENSMUSG00000039316	ENSMUSG00000039316	17	49458874	49656064	284	330
13303	3	16603307	16621249	ENSMUSG00000010592	ENSMUSG00000010592	ENSMUSG00000010592	17	49746816	49754805	284	330
13304	3	17026221	17106585	ENSMUSG00000038910	ENSMUSG00000038910	ENSMUSG00000038910	17	49974970	50154051	284	330
13305	3	17174903	17757403	ENSMUSG00000023923	ENSMUSG00000023923	ENSMUSG00000023923	17	50077281	50644264	284	330
13306	3	18365244	18441069	ENSMUSG00000023927	ENSMUSG00000023927	ENSMUSG00000023927	17	51203830	51297576	284	330
13307	3	19165021	19552137	ENSMUSG00000035580	ENSMUSG00000035580	ENSMUSG00000035580	17	52067734	52370165	284	330
13308	3	19895972	19952799	ENSMUSG00000023931	ENSMUSG00000023931	ENSMUSG00000023931	17	52863818	52928250	284	330
13309	3	19963749	20001647	ENSMUSG00000017831	ENSMUSG00000017831	ENSMUSG00000017831	17	52944163	52972607	284	330
13310	3	20001879	20018615	ENSMUSG00000044957	ENSMUSG00000044957	ENSMUSG00000044957	17	52972389	52980962	284	330
13311	2	108271527	108292802	ENSMUSG00000023122	ENSMUSG00000023122	ENSMUSG00000023122	17	53294566	53310887	285	331
13312	2	108230083	108248239	ENSMUSG00000023943	ENSMUSG00000023943	ENSMUSG00000023943	17	53426544	53455560	285	331
13313	2	107969427	107996873	ENSMUSG00000023945	ENSMUSG00000023945	ENSMUSG00000023945	17	53750416	53774985	285	331
13314	2	106784492	106869042	ENSMUSG00000024172	ENSMUSG00000024172	ENSMUSG00000024172	17	54921736	54986431	285	331
13315	19	4180495	4188515	ENSMUSG00000003206	ENSMUSG00000003206	ENSMUSG00000003206	17	55545916	55550315	286	332
13316	19	4229598	4241721	ENSMUSG00000039154	ENSMUSG00000039154	ENSMUSG00000039154	17	55563776	55569910	286	332
13317	19	4255668	4274833	ENSMUSG00000011589	ENSMUSG00000011589	ENSMUSG00000011589	17	55579804	55590174	286	332
13318	19	4275041	4289847	ENSMUSG00000038781	ENSMUSG00000038781	ENSMUSG00000038781	17	55590375	55598878	286	332
13319	19	4294556	4311060	ENSMUSG00000003199	ENSMUSG00000003199	ENSMUSG00000003199	17	55602619	55609961	286	332
13320	19	4311370	4351496	ENSMUSG00000003200	ENSMUSG00000003200	ENSMUSG00000003200	17	55610043	55629869	286	332
13321	19	4353661	4394022	ENSMUSG00000002835	ENSMUSG00000002835	ENSMUSG00000002835	17	55633805	55660843	286	332
13322	19	4396261	4408790	ENSMUSG00000019578	ENSMUSG00000019578	ENSMUSG00000019578	17	55660339	55668321	286	332
13323	19	4423255	4453213	ENSMUSG00000002833	ENSMUSG00000002833	ENSMUSG00000002833	17	55672950	55693889	286	332
13324	19	4455471	4464704	ENSMUSG00000002831	ENSMUSG00000002831	ENSMUSG00000002831	17	55695065	55702881	286	332
13325	19	4487439	4491048	ENSMUSG00000037095	ENSMUSG00000037095	ENSMUSG00000037095	17	55712969	55715239	286	332
13326	19	4590530	4604952	ENSMUSG00000044469	ENSMUSG00000044469	ENSMUSG00000044469	17	55796274	55807662	286	332
13327	19	4608566	4621415	ENSMUSG00000019579	ENSMUSG00000019579	ENSMUSG00000019579	17	55810250	55817629	286	332
13328	19	4626238	4674875	ENSMUSG00000001229	ENSMUSG00000001229	ENSMUSG00000001229	17	55820391	55852545	286	332
13329	19	4742770	4746567	ENSMUSG00000043683	ENSMUSG00000043683	ENSMUSG00000043683	17	55890531	55894784	286	332
13330	19	4766944	4782716	ENSMUSG00000047123	ENSMUSG00000047123	ENSMUSG00000047123	17	55903179	55910466	286	332
13331	19	4789346	4818676	ENSMUSG00000024197	ENSMUSG00000024197	ENSMUSG00000024197	17	55912671	55924220	286	332
13332	19	4842058	4853837	ENSMUSG00000073380	ENSMUSG00000073380	ENSMUSG00000073380	17	55927822	55933995	286	332
13333	19	4860513	4913158	ENSMUSG00000001228	ENSMUSG00000001228	ENSMUSG00000001228	17	55937126	55957195	286	332
13334	19	4920132	5104606	ENSMUSG00000024201	ENSMUSG00000024201	ENSMUSG00000024201	17	55959801	56036572	286	332
13335	19	5157379	5237399	ENSMUSG00000013236	ENSMUSG00000013236	ENSMUSG00000013236	17	56046141	56110147	286	332
13336	19	5406442	5407866	ENSMUSG00000044526	ENSMUSG00000044526	ENSMUSG00000044526	17	56145030	56146013	286	332
13337	19	5538010	5573762	ENSMUSG00000042625	ENSMUSG00000042625	ENSMUSG00000042625	17	56247917	56269640	286	332
13338	19	5574164	5619488	ENSMUSG00000071054	ENSMUSG00000071054	ENSMUSG00000071054	17	56270255	56291221	286	332
13339	19	5629433	5631911	ENSMUSG00000049760	ENSMUSG00000049760	ENSMUSG00000049760	17	56292571	56294890	286	332
13340	19	5641272	5642674	ENSMUSG00000057863	ENSMUSG00000057863	ENSMUSG00000057863	17	56298535	56299364	286	332
13341	19	5642846	5671143	ENSMUSG00000041168	ENSMUSG00000041168	ENSMUSG00000041168	17	56299420	56312007	286	332
13342	19	5671707	5729739	ENSMUSG00000040828	ENSMUSG00000040828	ENSMUSG00000040828	17	56321462	56349578	286	332
13343	19	5868391	5908986	ENSMUSG00000002372	ENSMUSG00000002372	ENSMUSG00000002372	17	56358482	56396885	286	332
13344	19	5862386	5866887	ENSMUSG00000040175	ENSMUSG00000040175	ENSMUSG00000040175	17	56397176	56397750	286	332
13345	19	5855885	5861864	ENSMUSG00000054723	ENSMUSG00000054723	ENSMUSG00000054723	17	56399062	56402101	286	332
13346	19	5774818	5779334	ENSMUSG00000039481	ENSMUSG00000039481	ENSMUSG00000039481	17	56436450	56442679	286	332
13347	19	5736155	5742194	ENSMUSG00000007603	ENSMUSG00000007603	ENSMUSG00000007603	17	56449908	56455216	286	332
13348	19	5944175	6061554	ENSMUSG00000024206	ENSMUSG00000024206	ENSMUSG00000024206	17	56461024	56516132	286	332
13349	19	6086669	6144104	ENSMUSG00000024207	ENSMUSG00000024207	ENSMUSG00000024207	17	56528228	56559571	286	332
13350	19	6163966	6230959	ENSMUSG00000024212	ENSMUSG00000024212	ENSMUSG00000024212	17	56577735	56620512	286	332
13351	19	6257725	6284562	ENSMUSG00000045019	ENSMUSG00000045019	ENSMUSG00000045019	17	56638614	56667250	286	332
13352	19	6312463	6319915	ENSMUSG00000002660	ENSMUSG00000002660	ENSMUSG00000002660	17	56675429	56681311	286	332
13353	19	6323444	6326040	ENSMUSG00000002661	ENSMUSG00000002661	ENSMUSG00000002661	17	56682477	56684446	286	332
13354	19	6326305	6326860	ENSMUSG00000002664	ENSMUSG00000002664	ENSMUSG00000002664	17	56684581	56685139	286	332
13355	19	6235811	6344184	ENSMUSG00000002658	ENSMUSG00000002658	ENSMUSG00000002658	17	56688529	56696547	286	332
13356	19	6377050	6384790	ENSMUSG00000011486	ENSMUSG00000011486	ENSMUSG00000011486	17	56717902	56724351	286	332
13357	19	6389747	6410781	ENSMUSG00000046329	ENSMUSG00000046329	ENSMUSG00000046329	17	56728619	56745010	286	332
13358	19	6414788	6418033	ENSMUSG00000044279	ENSMUSG00000044279	ENSMUSG00000044279	17	56747348	56751059	286	332
13359	19	6418219	6432798	ENSMUSG00000002668	ENSMUSG00000002668	ENSMUSG00000002668	17	56751258	56762269	286	332
13360	19	6445331	6453330	ENSMUSG00000062591	ENSMUSG00000062591	ENSMUSG00000062591	17	56765319	56772683	286	332
13361	19	6482037	6486933	ENSMUSG00000035678	ENSMUSG00000035678	ENSMUSG00000035678	17	56790776	56792848	286	332
13362	19	6536867	6542163	ENSMUSG00000019489	ENSMUSG00000019489	ENSMUSG00000019489	17	56831330	56835110	286	332
13363	19	6615568	6621599	ENSMUSG00000005824	ENSMUSG00000005824	ENSMUSG00000005824	17	56874827	56879512	286	332
13364	19	6628878	6671660	ENSMUSG00000024164	ENSMUSG00000024164	ENSMUSG00000024164	17	56889309	56913426	286	332
13365	19	6681068	6688566	ENSMUSG00000005823	ENSMUSG00000005823	ENSMUSG00000005823	17	56919970	56932975	286	332
13366	19	6690734	6702484	ENSMUSG00000019487	ENSMUSG00000019487	ENSMUSG00000019487	17	56934883	56948483	286	332
13367	19	6723722	6808371	ENSMUSG00000034116	ENSMUSG00000034116	ENSMUSG00000034116	17	56964450	57013288	286	332
13368	19	6838582	6891463	ENSMUSG00000004730	ENSMUSG00000004730	ENSMUSG00000004730	17	57044061	57166362	286	332
13369	5	102912455	102926393	ENSMUSG00000024228	ENSMUSG00000024228	ENSMUSG00000024228	17	58684949	58698610	287	333
13370	5	106740489	107034495	ENSMUSG00000048915	ENSMUSG00000048915	ENSMUSG00000048915	17	62292804	62566592	287	333
13371	5	107241854	107731554	ENSMUSG00000023965	ENSMUSG00000023965	ENSMUSG00000023965	17	62745733	63184762	287	333
13372	5	108111422	108560441	ENSMUSG00000000127	ENSMUSG00000000127	ENSMUSG00000000127	17	63581484	63824640	287	333
13373	5	109053055	109231328	ENSMUSG00000024085	ENSMUSG00000024085	ENSMUSG00000024085	17	64497940	64651401	287	333
13374	5	109932047	109989052	ENSMUSG00000045036	ENSMUSG00000045036	ENSMUSG00000045036	17	65151334	65440365	287	333
13375	18	9875973	9878150	ENSMUSG00000050612	ENSMUSG00000050612	ENSMUSG00000050612	17	65537050	65541711	288	334
13376	18	9698265	9852547	ENSMUSG00000056515	ENSMUSG00000056515	ENSMUSG00000056515	17	65553949	65621630	288	334
13377	18	9536843	9604556	ENSMUSG00000061950	ENSMUSG00000061950	ENSMUSG00000061950	17	65682963	65741405	288	334
13378	18	9324851	9392384	ENSMUSG00000024098	ENSMUSG00000024098	ENSMUSG00000024098	17	65821516	65850658	288	334
13379	18	9126801	9273757	ENSMUSG00000034647	ENSMUSG00000034647	ENSMUSG00000034647	17	65867044	65976589	288	334
13380	18	8707369	8822074	ENSMUSG00000052105	ENSMUSG00000052105	ENSMUSG00000052105	17	66241927	66354290	288	334
13381	18	7557817	8396160	ENSMUSG00000033278	ENSMUSG00000033278	ENSMUSG00000033278	17	66571895	67259018	288	334
13382	18	7221137	7222042	ENSMUSG00000073375	ENSMUSG00000073375	ENSMUSG00000073375	17	67536626	67537528	288	334
13383	18	6932084	7107725	ENSMUSG00000032796	ENSMUSG00000032796	ENSMUSG00000032796	17	67602232	67727594	288	334
13384	18	6827192	6905699	ENSMUSG00000024043	ENSMUSG00000024043	ENSMUSG00000024043	17	67747658	67909003	288	334
13385	18	5881421	5882062	ENSMUSG00000071046	ENSMUSG00000071046	ENSMUSG00000071046	17	68745334	68745999	288	334
13386	18	5382388	5533986	ENSMUSG00000024044	ENSMUSG00000024044	ENSMUSG00000024044	17	69061712	69194891	288	334
13387	18	5279719	5286039	ENSMUSG00000049672	ENSMUSG00000049672	ENSMUSG00000049672	17	69292214	69293560	288	334
13388	18	3488837	3870135	ENSMUSG00000003279	ENSMUSG00000003279	ENSMUSG00000003279	17	70420924	70723091	288	334
13389	18	3441591	3448404	ENSMUSG00000047407	ENSMUSG00000047407	ENSMUSG00000047407	17	70749554	70751256	288	334
13390	18	3252123	3268247	ENSMUSG00000034868	ENSMUSG00000034868	ENSMUSG00000034868	17	70878820	70895373	288	334
13391	18	3056806	3210106	ENSMUSG00000024049	ENSMUSG00000024049	ENSMUSG00000024049	17	70924378	71031712	288	334
13392	18	2906994	3001945	ENSMUSG00000024052	ENSMUSG00000024052	ENSMUSG00000024052	17	71088835	71151936	288	334
13393	18	2837028	2904090	ENSMUSG00000024053	ENSMUSG00000024053	ENSMUSG00000024053	17	71157029	71216323	288	334
13394	18	2693699	2774877	ENSMUSG00000024054	ENSMUSG00000024054	ENSMUSG00000024054	17	71249353	71353384	288	334
13395	18	2645886	2678689	ENSMUSG00000041913	ENSMUSG00000041913	ENSMUSG00000041913	17	71353426	71380200	288	334
13396	2	28946737	29023149	ENSMUSG00000041057	ENSMUSG00000041057	ENSMUSG00000041057	17	71521086	71562752	289	335
13397	2	29090837	29128600	ENSMUSG00000045761	ENSMUSG00000045761	ENSMUSG00000045761	17	71590022	71634400	289	335
13398	2	29138062	29150631	ENSMUSG00000044375	ENSMUSG00000044375	ENSMUSG00000044375	17	71648412	71657742	289	335
13399	2	29191822	29260176	ENSMUSG00000024059	ENSMUSG00000024059	ENSMUSG00000024059	17	71686804	71761633	289	335
13400	2	29269594	29997029	ENSMUSG00000055471	ENSMUSG00000055471	ENSMUSG00000055471	17	71774299	72508566	289	335
13401	2	30223311	30236903	ENSMUSG00000039770	ENSMUSG00000039770	ENSMUSG00000039770	17	72741704	72756195	289	335
13402	2	30307899	30336393	ENSMUSG00000024063	ENSMUSG00000024063	ENSMUSG00000024063	17	72823326	72846942	289	335
13403	2	30523627	30720595	ENSMUSG00000054469	ENSMUSG00000054469	ENSMUSG00000054469	17	73066706	73145099	289	335
13404	2	30799146	30883815	ENSMUSG00000043705	ENSMUSG00000043705	ENSMUSG00000043705	17	73211346	73304178	289	335
13405	2	30986837	31214517	ENSMUSG00000024064	ENSMUSG00000024064	ENSMUSG00000024064	17	73398110	73614946	289	335
13406	2	31310707	31344764	ENSMUSG00000024065	ENSMUSG00000024065	ENSMUSG00000024065	17	73709746	73736975	289	335
13407	2	31410691	31491085	ENSMUSG00000024066	ENSMUSG00000024066	ENSMUSG00000024066	17	73788790	73855081	289	335
13408	2	31603160	31659473	ENSMUSG00000038541	ENSMUSG00000038541	ENSMUSG00000038541	17	73922606	73952814	289	335
13409	2	32102465	32118385	ENSMUSG00000024067	ENSMUSG00000024067	ENSMUSG00000024067	17	74204462	74228926	289	335
13410	2	32142184	32236209	ENSMUSG00000024068	ENSMUSG00000024068	ENSMUSG00000024068	17	74243995	74296095	289	335
13411	2	32244437	32300313	ENSMUSG00000024069	ENSMUSG00000024069	ENSMUSG00000024069	17	74306894	74328496	289	335
13412	2	32303029	32344427	ENSMUSG00000039193	ENSMUSG00000039193	ENSMUSG00000039193	17	74331824	74353201	289	335
13413	2	32356483	32385159	ENSMUSG00000024072	ENSMUSG00000024072	ENSMUSG00000024072	17	74394532	74404942	289	335
13414	2	32435234	32697467	ENSMUSG00000024073	ENSMUSG00000024073	ENSMUSG00000024073	17	74433277	74608126	289	335
13415	2	32706633	32899620	ENSMUSG00000024078	ENSMUSG00000024078	ENSMUSG00000024078	17	74622813	74768630	289	335
13416	2	33025896	33478077	ENSMUSG00000001870	ENSMUSG00000001870	ENSMUSG00000001870	17	74910589	75296829	289	335
13417	2	33514920	33643162	ENSMUSG00000071042	ENSMUSG00000071042	ENSMUSG00000071042	17	75370673	75434118	289	335
13418	2	33662233	33677870	ENSMUSG00000002017	ENSMUSG00000002017	ENSMUSG00000002017	17	75442151	75456903	289	335
13419	2	36436573	36631782	ENSMUSG00000024074	ENSMUSG00000024074	ENSMUSG00000024074	17	78105544	78279372	289	335
13420	2	36632910	36678782	ENSMUSG00000056121	ENSMUSG00000056121	ENSMUSG00000056121	17	78282943	78323189	289	335
13421	2	36777418	36895433	ENSMUSG00000024076	ENSMUSG00000024076	ENSMUSG00000024076	17	78413198	78532456	289	335
13422	2	36924287	37047119	ENSMUSG00000024077	ENSMUSG00000024077	ENSMUSG00000024077	17	78559022	78641618	289	335
13423	2	37061657	37164989	ENSMUSG00000039414	ENSMUSG00000039414	ENSMUSG00000039414	17	78658093	78739279	289	335
13424	2	37179857	37237694	ENSMUSG00000024079	ENSMUSG00000024079	ENSMUSG00000024079	17	78757622	78787630	289	335
13425	2	37248467	37269194	ENSMUSG00000038045	ENSMUSG00000038045	ENSMUSG00000038045	17	78789955	78811936	289	335
13426	2	37282259	37312243	ENSMUSG00000024081	ENSMUSG00000024081	ENSMUSG00000024081	17	78824297	78842125	289	335
13427	2	37312294	37329805	ENSMUSG00000024082	ENSMUSG00000024082	ENSMUSG00000024082	17	78842194	78852557	289	335
13428	2	37331149	37398541	ENSMUSG00000024070	ENSMUSG00000024070	ENSMUSG00000024070	17	78855620	78925773	289	335
13429	2	37425221	37453969	ENSMUSG00000024084	ENSMUSG00000024084	ENSMUSG00000024084	17	78956964	78995301	289	335
13430	2	37724247	37752830	ENSMUSG00000036533	ENSMUSG00000036533	ENSMUSG00000036533	17	79238785	79259937	289	335
13431	2	38031110	38147784	ENSMUSG00000036368	ENSMUSG00000036368	ENSMUSG00000036368	17	79523226	79590478	289	335
13432	2	38148154	38156780	ENSMUSG00000024087	ENSMUSG00000024087	ENSMUSG00000024087	17	79615279	79623367	289	335
13433	2	38375526	38457908	ENSMUSG00000059811	ENSMUSG00000059811	ENSMUSG00000059811	17	79757412	79804354	289	335
13434	2	38642624	38683612	ENSMUSG00000024095	ENSMUSG00000024095	ENSMUSG00000024095	17	79937814	79970531	289	335
13435	2	38746559	38815414	ENSMUSG00000035473	ENSMUSG00000035473	ENSMUSG00000035473	17	80035797	80093358	289	335
13436	2	38824245	38832045	ENSMUSG00000024097	ENSMUSG00000024097	ENSMUSG00000024097	17	80108451	80115183	289	335
13437	2	38878375	38949057	ENSMUSG00000035051	ENSMUSG00000035051	ENSMUSG00000035051	17	80146638	80198469	289	335
13438	2	38956607	38963354	ENSMUSG00000045257	ENSMUSG00000045257	ENSMUSG00000045257	17	80203839	80205682	289	335
13439	2	39040268	39040987	ENSMUSG00000066938	ENSMUSG00000066938	ENSMUSG00000066938	17	80281544	80281867	289	335
13440	2	39066469	39201067	ENSMUSG00000024241	ENSMUSG00000024241	ENSMUSG00000024241	17	80306507	80388261	289	335
13441	2	39259192	39310177	ENSMUSG00000033966	ENSMUSG00000033966	ENSMUSG00000033966	17	80432482	80472160	289	335
13442	2	39329911	39517946	ENSMUSG00000024242	ENSMUSG00000024242	ENSMUSG00000024242	17	80488847	80636215	289	335
13443	2	39746563	39798607	ENSMUSG00000024245	ENSMUSG00000024245	ENSMUSG00000024245	17	80852958	80910136	289	335
13444	2	39816949	39859911	ENSMUSG00000024246	ENSMUSG00000024246	ENSMUSG00000024246	17	80934658	80973408	289	335
13445	2	40192790	40534188	ENSMUSG00000054640	ENSMUSG00000054640	ENSMUSG00000054640	17	81297017	81557933	289	335
13446	2	42129257	42139137	ENSMUSG00000024247	ENSMUSG00000024247	ENSMUSG00000024247	17	83123628	83133392	289	335
13447	2	42522661	42574741	ENSMUSG00000045053	ENSMUSG00000045053	ENSMUSG00000045053	17	83494283	83540221	289	335
13448	2	42649390	42837590	ENSMUSG00000055817	ENSMUSG00000055817	ENSMUSG00000055817	17	83614525	83722987	289	335
13449	2	42848081	42873180	ENSMUSG00000000673	ENSMUSG00000000673	ENSMUSG00000000673	17	83739482	83755075	289	335
13450	2	43303045	43307238	ENSMUSG00000045817	ENSMUSG00000045817	ENSMUSG00000045817	17	84092250	84096273	289	335
13451	2	43311495	43676684	ENSMUSG00000024251	ENSMUSG00000024251	ENSMUSG00000024251	17	84100280	84372758	289	335
13452	2	43717916	43848630	ENSMUSG00000040852	ENSMUSG00000040852	ENSMUSG00000040852	17	84420221	84530455	289	335

13453	2	43854682	43890653	ENSMUSG00000024253	ENSMUSG00000024253	ENSMUSG00000024253	17	84534822	84563904	289	335		
13454	2	43893115	43919462	ENSMUSG00000040505	ENSMUSG00000040505	ENSMUSG00000040505	17	84566560	84591337	289	335		
13455	2	43919607	43959109	ENSMUSG00000024254	ENSMUSG00000024254	ENSMUSG00000024254	17	84591457	84608659	289	335		
13456	2	43968391	44076648	ENSMUSG00000024120	ENSMUSG00000024120	ENSMUSG00000024120	17	84613575	84698884	289	335		
13457	2	44249504	44311361	ENSMUSG00000061130	ENSMUSG00000061130	ENSMUSG00000061130	17	84866325	84922931	289	335		
13458	2	44356103	44402137	ENSMUSG00000024131	ENSMUSG00000024131	ENSMUSG00000024131	17	84936702	84972562	289	335		
13459	2	44396924	44441317	ENSMUSG00000024127	ENSMUSG00000024127	ENSMUSG00000024127	17	84971805	84998547	289	335		
13460	2	44442607	44853235	ENSMUSG00000071037	ENSMUSG00000071037	ENSMUSG00000071037	17	84999052	85366465	289	335		
13461	2	45022365	45025945	ENSMUSG00000038805	ENSMUSG00000038805	ENSMUSG00000038805	17	85529357	85539117	289	335		
13462	2	45085825	45090046	ENSMUSG00000024134	ENSMUSG00000024134	ENSMUSG00000024134	17	85592594	85596573	289	335		
13463	2	45469323	45691924	ENSMUSG00000024135	ENSMUSG00000024135	ENSMUSG00000024135	17	85893523	86047589	289	335		
13464	2	45732547	46268632	ENSMUSG00000045038	ENSMUSG00000045038	ENSMUSG00000045038	17	86076111	86563725	289	335		
13465	2	46378045	46467340	ENSMUSG00000024140	ENSMUSG00000024140	ENSMUSG00000024140	17	86662228	86739907	289	335		
13466	2	46560199	46565066	ENSMUSG00000037689	ENSMUSG00000037689	ENSMUSG00000037689	17	86825741	86830691	289	335		
13467	2	46592490	46600600	ENSMUSG00000053375	ENSMUSG00000053375	ENSMUSG00000053375	17	86852435	86856213	289	335		
13468	2	46661580	46697708	ENSMUSG00000024145	ENSMUSG00000024145	ENSMUSG00000024145	17	86905585	86933727	289	335		
13469	2	46779595	46843424	ENSMUSG00000037104	ENSMUSG00000037104	ENSMUSG00000037104	17	87016005	87045911	289	335		
13470	2	47021817	47154785	ENSMUSG00000036918	ENSMUSG00000036918	ENSMUSG00000036918	17	87191212	87290084	289	335		
13471	2	47209091	47235930	ENSMUSG00000036557	ENSMUSG00000036557	ENSMUSG00000036557	17	87297902	87336011	289	335		
13472	2	47483767	47563864	ENSMUSG00000024151	ENSMUSG00000024151	ENSMUSG00000024151	17	87580866	87632038	289	335		
13473	2	47601440	47651002	ENSMUSG00000050138	ENSMUSG00000050138	ENSMUSG00000050138	17	87654147	87706320	289	335		
13474	2	47863790	47887588	ENSMUSG00000005370	ENSMUSG00000005370	ENSMUSG00000005370	17	87883410	87899389	289	335		
13475	2	47887565	47986326	ENSMUSG00000005371	ENSMUSG00000005371	ENSMUSG00000005371	17	87899187	87924042	289	335		
13476	2	48395374	48459938	ENSMUSG00000034998	ENSMUSG00000034998	ENSMUSG00000034998	17	88349084	88386397	289	335		
13477	2	48661228	48760246	ENSMUSG00000033855	ENSMUSG00000033855	ENSMUSG00000033855	17	88534935	88570912	289	335		
13478	2	48768340	48836314	ENSMUSG00000024107	ENSMUSG00000024107	ENSMUSG00000024107	17	88649875	88700302	289	335		
13479	2	49043156	49235134	ENSMUSG00000032937	ENSMUSG00000032937	ENSMUSG00000032937	17	88893278	89109001	289	335		
13480	2	50000992	50428370	ENSMUSG00000024109	ENSMUSG00000024109	ENSMUSG00000024109	17	89945405	90612558	289	335		
13481	18	895387	901054	ENSMUSG00000024256	ENSMUSG00000024256	ENSMUSG00000024256	17	93503312	93510828	290	336		
13482	18	2527530	2561470	ENSMUSG00000055660	ENSMUSG00000055660	ENSMUSG00000055660	17	95031189	95054001	290	336		
13483	10	35477306	35540764	ENSMUSG00000063889	ENSMUSG00000063889	ENSMUSG00000063889	18	3267586	3327505	291	337		
13484	10	35337485	35419576	ENSMUSG00000024231	ENSMUSG00000024231	ENSMUSG00000024231	18	3383248	3435387	291	337		
13485	10	29006277	29011874	ENSMUSG00000024232	ENSMUSG00000024232	ENSMUSG00000024232	18	3507955	3516402	292	338		
13486	10	30762872	30790768	ENSMUSG00000024235	ENSMUSG00000024235	ENSMUSG00000024235	18	4331325	4352951	292	338		
13487	10	30642003	30703376	ENSMUSG00000024234	ENSMUSG00000024234	ENSMUSG00000024234	18	4375590	4396782	292	338		
13488	10	30341735	30388459	ENSMUSG00000033960	ENSMUSG00000033960	ENSMUSG00000033960	18	4673922	4680300	292	338		
13489	10	29786283	29963907	ENSMUSG00000024236	ENSMUSG00000024236	ENSMUSG00000024236	18	5046574	5119288	292	338		
13490	10	31173717	31360860	ENSMUSG00000050945	ENSMUSG00000050945	ENSMUSG00000050945	18	5210638	5214918	293	338	here	This block and upper block have different orientation, and so were not merged by ST
13491	10	31648151	31858124	ENSMUSG00000024238	ENSMUSG00000024238	ENSMUSG00000024238	18	5591872	5774908	293	338	Blocks with different orientation were merged by AG	2 -> 1
13492	10	32135231	32257776	ENSMUSG00000041225	ENSMUSG00000041225	ENSMUSG00000041225	18	6025210	6135937	293	338		
13493	10	32337944	32385365	ENSMUSG00000006740	ENSMUSG00000006740	ENSMUSG00000006740	18	6202209	6241498	293	338		
13494	10	32597865	32676119	ENSMUSG00000024240	ENSMUSG00000024240	ENSMUSG00000024240	18	6436758	6490854	293	338		
13495	10	27833555	27869096	ENSMUSG00000073639	ENSMUSG00000073639	ENSMUSG00000073639	18	6765203	6790229	294	339		
13496	10	28001809	28074754	ENSMUSG00000061013	ENSMUSG00000061013	ENSMUSG00000061013	18	6934998	7004754	294	339		
13497	10	28141103	28327983	ENSMUSG00000061802	ENSMUSG00000061802	ENSMUSG00000061802	18	7088458	7265163	294	339		
13498	10	28379928	28611073	ENSMUSG00000057440	ENSMUSG00000057440	ENSMUSG00000057440	18	7379907	7513064	294	339		
13499	10	28862431	28949703	ENSMUSG00000024283	ENSMUSG00000024283	ENSMUSG00000024283	18	7869237	7927065	294	339		
13500	10	35967183	35970368	ENSMUSG00000036904	ENSMUSG00000036904	ENSMUSG00000036904	18	9212918	9214975	295	340		
13501	10	35934344	35937869	ENSMUSG00000036855	ENSMUSG00000036855	ENSMUSG00000036855	18	9278605	9282807	295	340		
13502	10	35665808	35900858	ENSMUSG00000024286	ENSMUSG00000024286	ENSMUSG00000024286	18	9314042	9450148	295	340		
13503	18	570357	571624	ENSMUSG00000050996	ENSMUSG00000050996	ENSMUSG00000050996	18	9615522	9619493	296	341		
13504	18	309356	490685	ENSMUSG00000036103	ENSMUSG00000036103	ENSMUSG00000036103	18	9707657	9875049	296	341		
13505	18	204527	258049	ENSMUSG00000024287	ENSMUSG00000024287	ENSMUSG00000024287	18	9958178	9995482	296	341		
13506	18	148483	203739	ENSMUSG00000047879	ENSMUSG00000047879	ENSMUSG00000047879	18	9996095	10024693	296	341		
13507	18	16787533	16944869	ENSMUSG00000024290	ENSMUSG00000024290	ENSMUSG00000024290	18	10067465	10181315	297	342		
13508	18	17363262	17434843	ENSMUSG00000024293	ENSMUSG00000024293	ENSMUSG00000024293	18	10566617	10610080	297	342		
13509	18	17446235	17464342	ENSMUSG00000002477	ENSMUSG00000002477	ENSMUSG00000002477	18	10617794	10628228	297	342		
13510	18	17484856	17538724	ENSMUSG00000002475	ENSMUSG00000002475	ENSMUSG00000002475	18	10644409	10706694	297	342		
13511	18	17575543	17704912	ENSMUSG00000024294	ENSMUSG00000024294	ENSMUSG00000024294	18	10725623	10812215	297	342		
13512	18	18768709	18860328	ENSMUSG00000041238	ENSMUSG00000041238	ENSMUSG00000041238	18	11776812	11886351	297	342		
13513	18	18969725	19091329	ENSMUSG00000040957	ENSMUSG00000040957	ENSMUSG00000040957	18	11982810	12089100	297	342		
13514	18	19129979	19271923	ENSMUSG00000049411	ENSMUSG00000049411	ENSMUSG00000049411	18	12124839	12265016	297	342		
13515	18	19287254	19317031	ENSMUSG00000024404	ENSMUSG00000024404	ENSMUSG00000024404	18	12272295	12300233	297	342		
13516	18	19337471	19365740	ENSMUSG00000024410	ENSMUSG00000024410	ENSMUSG00000024410	18	12312283	12333518	297	342		
13517	18	19365463	19420426	ENSMUSG00000024413	ENSMUSG00000024413	ENSMUSG00000024413	18	12333387	12379894	297	342		
13518	18	19433978	19496849	ENSMUSG00000057766	ENSMUSG00000057766	ENSMUSG00000057766	18	12397492	12415231	297	342		
13519	18	19523560	19789025	ENSMUSG00000024421	ENSMUSG00000024421	ENSMUSG00000024421	18	12477560	12725832	297	342		
13520	18	19826735	19966989	ENSMUSG00000024424	ENSMUSG00000024424	ENSMUSG00000024424	18	12786969	12880220	297	342		
13521	18	19972953	19995562	ENSMUSG00000024430	ENSMUSG00000024430	ENSMUSG00000024430	18	12884524	12898311	297	342		
13522	18	20260680	20287490	ENSMUSG00000024423	ENSMUSG00000024423	ENSMUSG00000024423	18	13115421	13136117	297	342		
13523	18	20294591	20311567	ENSMUSG00000037346	ENSMUSG00000037346	ENSMUSG00000037346	18	13150159	13166051	297	342		
13524	18	20895889	21186114	ENSMUSG00000024420	ENSMUSG00000024420	ENSMUSG00000024420	18	13858310	14082317	297	342		
13525	18	21850217	21924609	ENSMUSG00000037013	ENSMUSG00000037013	ENSMUSG00000037013	18	14768647	14825932	297	342		
13526	18	22690173	22699651	ENSMUSG00000024411	ENSMUSG00000024411	ENSMUSG00000024411	18	15535837	15546671	297	342		
13527	18	22749670	23019279	ENSMUSG00000047161	ENSMUSG00000047161	ENSMUSG00000047161	18	15594942	15903175	297	342		
13528	18	23784934	24011189	ENSMUSG00000024304	ENSMUSG00000024304	ENSMUSG00000024304	18	16732027	16952264	297	342		
13529	18	26825029	26876687	ENSMUSG00000059898	ENSMUSG00000059898	ENSMUSG00000059898	18	20106382	20145107	297	342		
13530	18	26900005	26936375	ENSMUSG00000024331	ENSMUSG00000024331	ENSMUSG00000024331	18	20173808	20202515	297	342		
13531	18	26963214	26996817	ENSMUSG00000044322	ENSMUSG00000044322	ENSMUSG00000044322	18	20227713	20257783	297	342		
13532	18	27210844	27247878	ENSMUSG00000001804	ENSMUSG00000001804	ENSMUSG00000001804	18	20579185	20614831	297	342		
13533	18	27281756	27310474	ENSMUSG00000056632	ENSMUSG00000056632	ENSMUSG00000056632	18	20653284	20683759	297	342		
13534	18	27332233	27380855	ENSMUSG00000044393	ENSMUSG00000044393	ENSMUSG00000044393	18	20701117	20745840	297	342		
13535	18	27425838	27432793	ENSMUSG00000061808	ENSMUSG00000061808	ENSMUSG00000061808	18	20808423	20817331	297	342		
13536	18	27457131	27518684	ENSMUSG00000056124	ENSMUSG00000056124	ENSMUSG00000056124	18	20829013	20889414	297	342		
13537	18	27663902	27777067	ENSMUSG00000033382	ENSMUSG00000033382	ENSMUSG00000033382	18	20960322	21008062	297	342		
13538	18	27852443	27903161	ENSMUSG00000033107	ENSMUSG00000033107	ENSMUSG00000033107	18	21104482	21126734	297	342		
13539	18	27925835	27965521	ENSMUSG00000024317	ENSMUSG00000024317	ENSMUSG00000024317	18	21144351	21171233	297	342		
13540	18	28023985	28054362	ENSMUSG00000024313	ENSMUSG00000024313	ENSMUSG00000024313	18	21215384	21240502	297	342		
13541	18	28101489	28304445	ENSMUSG00000042680	ENSMUSG00000042680	ENSMUSG00000042680	18	21270211	21443148	297	342		
13542	18	28506632	28606972	ENSMUSG00000042514	ENSMUSG00000042514	ENSMUSG00000042514	18	21693391	21795093	297	342		
13543	18	28791970	29274655	ENSMUSG00000024306	ENSMUSG00000024306	ENSMUSG00000024306	18	21953907	22314406	297	342		
13544	18	29565930	29581375	ENSMUSG00000045215	ENSMUSG00000045215	ENSMUSG00000045215	18	22659574	22668556	297	342		
13545	18	29686412	30057519	ENSMUSG00000041923	ENSMUSG00000041923	ENSMUSG00000041923	18	22836391	23181666	297	342		
13546	18	30544246	30725341	ENSMUSG00000024302	ENSMUSG00000024302	ENSMUSG00000024302	18	23678173	23794673	297	342		
13547	18	30875379	30975820	ENSMUSG00000024277	ENSMUSG00000024277	ENSMUSG00000024277	18	23946994	24036863	297	342		
13548	18	31074999	31081151	ENSMUSG00000024276	ENSMUSG00000024276	ENSMUSG00000024276	18	24097696	24105876	297	342		
13549	18	31086817	31124170	ENSMUSG00000024274	ENSMUSG00000024274	ENSMUSG00000024274	18	24107834	24114754	297	342		
13550	18	31169948	31178405	ENSMUSG00000051469	ENSMUSG00000051469	ENSMUSG00000051469	18	24155276	24163820	297	342		
13551	18	31288784	31331953	ENSMUSG00000047989	ENSMUSG00000047989	ENSMUSG00000047989	18	24247771	24264829	297	342		
13552	18	31488599	31545792	ENSMUSG00000000420	ENSMUSG00000000420	ENSMUSG00000000420	18	24348451	24428655	297	342		
13553	18	31806586	31813238	ENSMUSG00000024273	ENSMUSG00000024273	ENSMUSG00000024273	18	24613914	24620920	297	342		
13554	18	31823785	31901491	ENSMUSG00000040446	ENSMUSG00000040446	ENSMUSG00000040446	18	24627972	24673214	297	342		
13555	18	31943197	31963203	ENSMUSG00000024270	ENSMUSG00000024270	ENSMUSG00000024270	18	24722898	24746785	297	342		
13556	18	31963882	32008616	ENSMUSG00000024271	ENSMUSG00000024271	ENSMUSG00000024271	18	24746973	24781840	297	342		
13557	18	32021480	32102682	ENSMUSG00000039616	ENSMUSG00000039616	ENSMUSG00000039616	18	24787406	24844581	297	342		
13558	18	32131700	32614016	ENSMUSG00000034295	ENSMUSG00000034295	ENSMUSG00000034295	18	24852455	25276521	297	342		
13559	18	32630033	32663156	ENSMUSG00000024269	ENSMUSG00000024269	ENSMUSG00000024269	18	25281227	25311640	297	342		
13560	18	32663078	33059285	ENSMUSG00000033632	ENSMUSG00000033632	ENSMUSG00000033632	18	25432957	25607102	297	342		
13561	18	33077828	33399941	ENSMUSG00000024268	ENSMUSG00000024268	ENSMUSG00000024268	18	25622125	25897167	297	342		
13562	18	37789197	37915442	ENSMUSG00000033628	ENSMUSG00000033628	ENSMUSG00000033628	18	30415961	30487098	297	342		
13563	18	38577191	38949655	ENSMUSG00000057455	ENSMUSG00000057455	ENSMUSG00000057455	18	31117324	31460138	297	342		
13564	18	39101857	39111430	ENSMUSG00000024261	ENSMUSG00000024261	ENSMUSG00000024261	18	31580818	31590425	297	342		
13565	2	128415267	128501337	ENSMUSG00000024260	ENSMUSG00000024260	ENSMUSG00000024260	18	31777381	31866071	298	343		
13566	2	128335681	128359966	ENSMUSG00000041915	ENSMUSG00000041915	ENSMUSG00000041915	18	31904086	31925737	298	343		
13567	2	128320710	128332174	ENSMUSG00000024258	ENSMUSG00000024258	ENSMUSG00000024258	18	31932169	31939652	298	343		
13568	2	128178280	128285215	ENSMUSG00000024400	ENSMUSG00000024400	ENSMUSG00000024400	18	31970295	32050005	298	343		
13569	2	128113093	128155507	ENSMUSG00000024395	ENSMUSG00000024395	ENSMUSG00000024395	18	32074517	32101629	298	343		
13570	2	128120217	128126682	ENSMUSG00000052229	ENSMUSG00000052229	ENSMUSG00000052229	18	32086022	32092646	298	343		
13571	2	128038198	128111456	ENSMUSG00000024388	ENSMUSG00000024388	ENSMUSG00000024388	18	32102411	32158555	298	343		
13572	2	127954860	128000514	ENSMUSG00000024384	ENSMUSG00000024384	ENSMUSG00000024384	18	32210749	32241454	298	343		
13573	2	127892486	127903288	ENSMUSG00000024386	ENSMUSG00000024386	ENSMUSG00000024386	18	32266137	32281209	298	343		
13574	2	127731336	127768222	ENSMUSG00000024382	ENSMUSG00000024382	ENSMUSG00000024382	18	32383341	32413157	298	343		
13575	2	127522107	127581334	ENSMUSG00000024381	ENSMUSG00000024381	ENSMUSG00000024381	18	32520267	32578741	298	343		
13576	5	110433677	110440390	ENSMUSG00000024379	ENSMUSG00000024379	ENSMUSG00000024379	18	32958393	32962802	299	344		
13577	5	110455769	110494099	ENSMUSG00000038299	ENSMUSG00000038299	ENSMUSG00000038299	18	32980263	33009040	299	344		
13578	5	110587968	110858483	ENSMUSG00000038128	ENSMUSG00000038128	ENSMUSG00000038128	18	33082366	33311737	299	344		
13579	5	110859630	110876089	ENSMUSG00000024378	ENSMUSG00000024378	ENSMUSG00000024378	18	33326232	33338627	299	344		
13580	5	111092408	111121320	ENSMUSG00000042834	ENSMUSG00000042834	ENSMUSG00000042834	18	33563525	33567704	299	344		
13581	5	111532650	111782909	ENSMUSG00000024376	ENSMUSG00000024376	ENSMUSG00000024376	18	33921128	34132007	299	344		
13582	5	112101483	112209834	ENSMUSG00000005871	ENSMUSG00000005871	ENSMUSG00000005871	18	34345794	34443382	299	344		
13583	5	112224818	112231988	ENSMUSG00000014504	ENSMUSG00000014504	ENSMUSG00000014504	18	34455984	34461399	299	344		
13584	5	112239983	112285965	ENSMUSG00000005873	ENSMUSG00000005873	ENSMUSG00000005873	18	34469675	34498138	299	344		
13585	5	137253054	137306335	ENSMUSG00000014503	ENSMUSG00000014503	ENSMUSG00000014503	18	34534224	34567599	300	345		
13586	5	137301550	137396619	ENSMUSG00000036501	ENSMUSG00000036501	ENSMUSG00000036501	18	34567152	34631624	300	345		
13587	5	137447578	137454975	ENSMUSG00000012282	ENSMUSG00000012282	ENSMUSG00000012282	18	34667114	34673074	300	345		
13588	5	137478761	137503031	ENSMUSG00000035984	ENSMUSG00000035984	ENSMUSG00000035984	18	34687524	34703487	300	345		
13589	5	137503364	137542257	ENSMUSG00000003778	ENSMUSG00000003778	ENSMUSG00000003778	18	34723416	34749611	300	345		
13590	5	137542757	137551304	ENSMUSG00000003779	ENSMUSG00000003779	ENSMUSG00000003779	18	34749796	34758078	300	345		
13591	5	137551259	137576918	ENSMUSG00000024370	ENSMUSG00000024370	ENSMUSG00000024370	18	34756484	34776537	300	345		
13592	5	137615970	137638152	ENSMUSG00000024366	ENSMUSG00000024366	ENSMUSG00000024366	18	34814721	34845204	300	345		
13593	5	137649168	137701943	ENSMUSG00000044201	ENSMUSG00000044201	ENSMUSG00000044201	18	34858971	34877482	300	345		
13594	5	137701867	137713315	ENSMUSG00000034300	ENSMUSG00000034300	ENSMUSG00000034300	18	34884880	34899734	300	345		
13595	5	137716184	137800615	ENSMUSG00000038773	ENSMUSG00000038773	ENSMUSG00000038773	18	34903105	34963641	300	345		
13596	5	137802636	137810552	ENSMUSG00000038555	ENSMUSG00000038555	ENSMUSG00000038555	18	34966632	34973429	300	345		
13597	5	137829338	137831669	ENSMUSG00000038418	ENSMUSG00000038418	ENSMUSG00000038418	18	34987181	34990930	300	345		
13598	5	137869690	137906506	ENSMUSG00000024360	ENSMUSG00000024360	ENSMUSG00000024360	18	35029893	35057927	300	345		
13599	5	137918924	137939014	ENSMUSG00000024359	ENSMUSG00000024359	ENSMUSG00000024359	18	35063391	35080287	300	345		
13600	5	138236598	138238145	ENSMUSG00000071862	ENSMUSG00000071862	ENSMUSG00000071862	18	35338674	35340218	300	345		
13601	5	138310342	138561904	ENSMUSG00000024357	ENSMUSG00000024357	ENSMUSG00000024357	18	35392372	35624876	300	345		
13602	5	138637691	138694027	ENSMUSG00000037236	ENSMUSG00000037236	ENSMUSG00000037236	18	35688179	35717400	300	345		
13603	5	138705418	138733307	ENSMUSG00000037058	ENSMUSG00000037058	ENSMUSG00000037058	18	35724641	35743159	300	345		
13604	5	138730793	138746900	ENSMUSG00000024354	ENSMUSG00000024354	ENSMUSG00000024354	18	35740578	35753192	300	345		
13605	5	138755622	138758657	ENSMUSG00000073600	ENSMUSG00000073600	ENSMUSG00000073600	18	35778141	35781161	300	345		
13606	5	138760153	138767676	ENSMUSG00000024352	ENSMUSG00000024352	ENSMUSG00000024352	18	35782670	35788129	300	345		
13607	5	138775283	138803065	ENSMUSG00000024350	ENSMUSG00000024350	ENSMUSG00000024350	18	35799801	35829095	300	345		
13608	5	138832292	138832690	ENSMUSG00000073598	ENSMUSG00000073598	ENSMUSG00000073598	18	35854442	35854822	300	345		
13609	5	138835303	138841473	ENSMUSG00000024349	ENSMUSG00000024349	ENSMUSG00000024349	18	35859658	35866504	300	345		
13610	5	139008130	139043649	ENSMUSG00000046668	ENSMUSG00000046668	ENSMUSG00000046668	18	35955792	35987667	300	345		
13611	5	139155590	139204232	ENSMUSG00000024347	ENSMUSG00000024347	ENSMUSG00000024347	18	36090804	36140686	300	345		
13612	5	139207444	139403063	ENSMUSG00000060275	ENSMUSG00000060275	ENSMUSG00000060275	18	36143888	36322825	300	345		
13613	5	139473892	139476505	ENSMUSG00000043991	ENSMUSG00000043991	ENSMUSG00000043991	18	36407136	36414223	300	345		
13614	5	139534920	139603555	ENSMUSG00000046727	ENSMUSG00000046727	ENSMUSG00000046727	18	36474671	36528954	300	345		
13615	5	139604831	139662867	ENSMUSG00000024346	ENSMUSG00000024346	ENSMUSG00000024346	18	36529653	36580469	300	345		
13616	5	139692615	139706359	ENSMUSG00000024486	ENSMUSG00000024486	ENSMUSG00000024486	18	36630906	36641748	300	345		
13617	5	139719984	139734777	ENSMUSG00000024485	ENSMUSG00000024485	ENSMUSG00000024485	18	36654125	36682247	300	345		
13618	5	139761613	139909347	ENSMUSG00000024483	ENSMUSG00000024483	ENSMUSG00000024483	18	36720056	36784112	300	345		
13619	5	139918038	139924014	ENSMUSG00000001379	ENSMUSG00000001379	ENSMUSG00000001379	18	36797133	36805340	300	345		
13620	5	139924604	139928867	ENSMUSG00000033272	ENSMUSG00000033272	ENSMUSG00000033272	18	36805566	36809831	300	345		
13621	5	139991522	139993157	ENSMUSG00000051439	ENSMUSG00000051439	ENSMUSG00000051439	18	36851038	36852628	300	345		
13622	5	139999232	140005171	ENSMUSG00000006850	ENSMUSG00000006850	ENSMUSG00000006850	18	36861042	36868371	300	345		
13623	5	140005143	140007424	ENSMUSG00000014294	ENSMUSG00000014294	ENSMUSG00000014294	18	36868340	36870522	300	345		
13624	5	139991756	140022248	ENSMUSG00000024474	ENSMUSG00000024474	ENSMUSG00000024474	18	36870685	36883609	300	345		
13625	5	140024637	140032113	ENSMUSG00000042660	ENSMUSG00000042660	ENSMUSG00000042660	18	36886213	36889682	300	345		
13626	5	140030568	140033355	ENSMUSG00000044595	ENSMUSG00000044595	ENSMUSG00000044595	18	36889643	36892188	300	345		
13627	5	140033675	140051155	ENSMUSG00000001380	ENSMUSG00000001380	ENSMUSG00000001380	18	36892504	36909094	300	345		
13628	5	140051202	140059073	ENSMUSG00000019143	ENSMUSG00000019143	ENSMUSG00000019143	18	36909254	36918543	300	345		
13629	5	140060216	140066423	ENSMUSG00000001383	ENSMUSG00000001383	ENSMUSG00000001383	18	36920271	36923996	300	345		
13630	5	140222581	140223159	ENSMUSG00000073594	ENSMUSG00000073594	ENSMUSG00000073594	18	37138634	37139047	300	345		
13631	5	140326296	140372113	ENSMUSG00000007440	ENSMUSG00000007440	ENSMUSG00000007440	18	37269943	37313631	300	345		
13632	5	140411163	140413696	ENSMUSG00000051663	ENSMUSG00000051663	ENSMUSG00000051663	18	37390972	37393428	300	345		
13633	5	140454403	140457146	ENSMUSG00000051599	ENSMUSG00000051599	ENSMUSG00000051599	18	37420822	37423587	300	345		
13634	5	140460418	140463590	ENSMUSG00000045498	ENSMUSG00000045498	ENSMUSG00000045498	18	37426773	37430559	300	345		
13635	5	140510023	140513052	ENSMUSG00000047307	ENSMUSG00000047307	ENSMUSG00000047307	18	37568491	37572183	300	345		
13636	5	140532427	140536141	ENSMUSG00000047033	ENSMUSG00000047033	ENSMUSG00000047033	18	37599520	37602314	300	345		
13637	5	140541164	140546158	ENSMUSG00000046387	ENSMUSG00000046387	ENSMUSG00000046387	18	37610769	37615428	300	345		
13638	5	140552126	140555399	ENSMUSG00000048347	ENSMUSG00000048347	ENSMUSG00000048347	18	37615439	37620477	300	345		
13639	5	140594122	140597285	ENSMUSG00000044022	ENSMUSG00000044022	ENSMUSG00000044022	18	37639626	37642337	300	345		
13640	5	140605165	140607926	ENSMUSG00000073591	ENSMUSG00000073591	ENSMUSG00000073591	18	37644327	37647393	300	345		
13641	5	140662711	140663616	ENSMUSG00000050304	ENSMUSG00000050304	ENSMUSG00000050304	18	37763651	37764448	300	345		
13642	5	140678746	140679795	ENSMUSG00000051316	ENSMUSG00000051316	ENSMUSG00000051316	18	37766876	37770140	300	345		
13643	5	140690436	140692907	ENSMUSG00000069395	ENSMUSG00000069395	ENSMUSG00000069395	18	37787919	37790339	300	345		
13644	5	140698538	140701194	ENSMUSG00000069394	ENSMUSG00000069394	ENSMUSG00000069394	18	37795079	37797499	300	345		
13645	5	140848992	140872730	ENSMUSG00000023036	ENSMUSG00000023036	ENSMUSG00000023036	18	37945520	37967847	300	345		
13646	5	140876605	140978665	ENSMUSG00000024456	ENSMUSG00000024456	ENSMUSG00000024456	18	38015670	38061292	300	345		
13647	5	140980627	140996596	ENSMUSG00000024454	ENSMUSG00000024454	ENSMUSG00000024454	18	38062953	38080909	300	345		

13648	5	140996777	141000814	ENSMUSG00000044024	ENSMUSG00000044024	ENSMUSG00000044024	18	38081572	38085139	300	345
13649	5	140999054	141011170	ENSMUSG00000038524	ENSMUSG00000038524	ENSMUSG00000038524	18	38083408	38095705	300	345
13650	5	141013154	141041984	ENSMUSG00000024451	ENSMUSG00000024451	ENSMUSG00000024451	18	38098611	38123536	300	345
13651	5	141212867	141238128	ENSMUSG00000051375	ENSMUSG00000051375	ENSMUSG00000051375	18	38322668	38335736	300	345
13652	5	141283593	141299900	ENSMUSG00000024442	ENSMUSG00000024442	ENSMUSG00000024442	18	38376233	38388600	300	345
13653	5	141304715	141318811	ENSMUSG00000024440	ENSMUSG00000024440	ENSMUSG00000024440	18	38394750	38410331	300	345
13654	5	141326621	141348934	ENSMUSG00000060450	ENSMUSG00000060450	ENSMUSG00000060450	18	38422658	38443821	300	345
13655	5	141468508	141513128	ENSMUSG00000024425	ENSMUSG00000024425	ENSMUSG00000024425	18	38544911	38591161	300	345
13656	5	141670189	141684750	ENSMUSG00000024427	ENSMUSG00000024427	ENSMUSG00000024427	18	38712235	38727242	300	345
13657	5	141951927	141981091	ENSMUSG00000036585	ENSMUSG00000036585	ENSMUSG00000036585	18	38965154	39044673	300	345
13658	5	142130133	142586243	ENSMUSG00000036452	ENSMUSG00000036452	ENSMUSG00000036452	18	39119119	39502259	300	345
13659	5	142637689	142763447	ENSMUSG00000024431	ENSMUSG00000024431	ENSMUSG00000024431	18	39537961	39613206	300	345
13660	5	143518164	143530387	ENSMUSG00000024487	ENSMUSG00000024487	ENSMUSG00000024487	18	40330839	40345373	300	345
13661	5	143566471	143833870	ENSMUSG00000051401	ENSMUSG00000051401	ENSMUSG00000051401	18	40384362	40657077	300	345
13662	5	145118775	145195060	ENSMUSG00000056671	ENSMUSG00000056671	ENSMUSG00000056671	18	42038343	42077148	300	345
13663	5	145219489	145232724	ENSMUSG00000073574	ENSMUSG00000073574	ENSMUSG00000073574	18	42112174	42125022	300	345
13664	5	145297633	145441527	ENSMUSG00000057719	ENSMUSG00000057719	ENSMUSG00000057719	18	42179640	42283829	300	345
13665	5	145444069	145464139	ENSMUSG00000059455	ENSMUSG00000059455	ENSMUSG00000059455	18	42304656	42322659	300	345
13666	5	145473221	145542321	ENSMUSG00000024493	ENSMUSG00000024493	ENSMUSG00000024493	18	42328486	42383167	300	345
13667	5	145563356	145646165	ENSMUSG00000024491	ENSMUSG00000024491	ENSMUSG00000024491	18	42401351	42464786	300	345
13668	5	145698869	145700200	ENSMUSG00000024497	ENSMUSG00000024497	ENSMUSG00000024497	18	42520616	42521983	300	345
13669	5	145807087	145871263	ENSMUSG00000024498	ENSMUSG00000024498	ENSMUSG00000024498	18	42637460	42701758	300	345
13670	5	145874610	145875869	ENSMUSG00000042816	ENSMUSG00000042816	ENSMUSG00000042816	18	42704316	42705584	300	345
13671	5	145949703	146415499	ENSMUSG00000024500	ENSMUSG00000024500	ENSMUSG00000024500	18	42763405	43025476	300	345
13672	5	146594719	146735672	ENSMUSG00000039954	ENSMUSG00000039954	ENSMUSG00000039954	18	43333028	43442812	300	345
13673	5	146750569	146869812	ENSMUSG00000024501	ENSMUSG00000024501	ENSMUSG00000024501	18	43449663	43488922	300	345
13674	5	146950899	147142445	ENSMUSG00000024502	ENSMUSG00000024502	ENSMUSG00000024502	18	43663010	43716007	300	345
13675	5	147184339	147191453	ENSMUSG00000024503	ENSMUSG00000024503	ENSMUSG00000024503	18	43853416	43862528	300	345
13676	5	147238467	147241946	ENSMUSG00000038791	ENSMUSG00000038791	ENSMUSG00000038791	18	43889658	43892730	300	345
13677	5	147252465	147266258	ENSMUSG00000071858	ENSMUSG00000071858	ENSMUSG00000071858	18	43902527	43918208	300	345
13678	5	147423759	147497120	ENSMUSG00000055561	ENSMUSG00000055561	ENSMUSG00000055561	18	44088637	44144137	300	345
13679	5	147562597	147574892	ENSMUSG00000055095	ENSMUSG00000055095	ENSMUSG00000055095	18	44196724	44208941	300	345
13680	5	147695315	147699605	ENSMUSG00000061144	ENSMUSG00000061144	ENSMUSG00000061144	18	44229954	44233578	out-of-place	345
13681	5	137171361	137173313	ENSMUSG00000038071	ENSMUSG00000038071	ENSMUSG00000038071	18	44395458	44403031	301	346
13682	5	137231459	137251438	ENSMUSG00000024471	ENSMUSG00000024471	ENSMUSG00000024471	18	44459405	44481053	301	346
13683	5	112340530	112377080	ENSMUSG00000024472	ENSMUSG00000024472	ENSMUSG00000024472	18	44505840	44543282	302	347
13684	5	112391051	112852426	ENSMUSG00000071856	ENSMUSG00000071856	ENSMUSG00000071856	18	44555218	44937494	302	347
13685	5	112877309	112958878	ENSMUSG00000034653	ENSMUSG00000034653	ENSMUSG00000034653	18	44959789	45013016	302	347
13686	5	113725565	113859775	ENSMUSG00000054477	ENSMUSG00000054477	ENSMUSG00000054477	18	45394336	45811355	302	347
13687	5	114488377	114544142	ENSMUSG00000033949	ENSMUSG00000033949	ENSMUSG00000033949	18	46291176	46337856	302	347
13688	5	114575476	114626449	ENSMUSG00000024477	ENSMUSG00000024477	ENSMUSG00000024477	18	46365280	46406181	302	347
13689	5	114631399	114660254	ENSMUSG00000071855	ENSMUSG00000071855	ENSMUSG00000071855	18	46408587	46421711	302	347
13690	5	114884507	114908490	ENSMUSG00000033319	ENSMUSG00000033319	ENSMUSG00000033319	18	46629153	46651235	302	347
13691	5	115168333	115180304	ENSMUSG00000033022	ENSMUSG00000033022	ENSMUSG00000033022	18	46838536	46853673	302	347
13692	5	115193714	115205398	ENSMUSG00000032905	ENSMUSG00000032905	ENSMUSG00000032905	18	46857758	46866888	302	347
13693	5	115326214	115391197	ENSMUSG00000024481	ENSMUSG00000024481	ENSMUSG00000024481	18	46975370	47007067	302	347
13694	5	115448622	115656882	ENSMUSG00000042705	ENSMUSG00000042705	ENSMUSG00000042705	18	47084207	47213323	302	347
13695	5	115809345	115937990	ENSMUSG00000019647	ENSMUSG00000019647	ENSMUSG00000019647	18	47370585	47494199	302	347
13696	5	118203135	118352139	ENSMUSG00000024505	ENSMUSG00000024505	ENSMUSG00000024505	18	49822804	49880905	302	347
13697	5	118435084	118612721	ENSMUSG00000037416	ENSMUSG00000037416	ENSMUSG00000037416	18	49958327	50089584	302	347
13698	5	118756403	118756975	ENSMUSG00000062210	ENSMUSG00000062210	ENSMUSG00000062210	18	50215529	50216682	302	347
13699	5	118816103	118905926	ENSMUSG00000024507	ENSMUSG00000024507	ENSMUSG00000024507	18	50253531	50321514	302	347
13700	5	118993192	118999415	ENSMUSG00000035420	ENSMUSG00000035420	ENSMUSG00000035420	18	50403699	50408349	302	347
13701	5	119828081	120050303	ENSMUSG00000073565	ENSMUSG00000073565	ENSMUSG00000073565	18	51242923	51545229	302	347
13702	5	121215549	121216418	ENSMUSG00000024510	ENSMUSG00000024510	ENSMUSG00000024510	18	52456905	52458154	302	347
13703	5	121325555	121391528	ENSMUSG00000024528	ENSMUSG00000024528	ENSMUSG00000024528	18	52591085	52616636	302	347
13704	5	121429918	121441853	ENSMUSG00000024529	ENSMUSG00000024529	ENSMUSG00000024529	18	52646159	52654698	302	347
13705	5	121493114	121517156	ENSMUSG00000046886	ENSMUSG00000046886	ENSMUSG00000046886	18	52741284	52765199	302	347
13706	5	121543150	121546236	ENSMUSG00000024532	ENSMUSG00000024532	ENSMUSG00000024532	18	52771585	52789102	302	347
13707	5	121675801	121827648	ENSMUSG00000024534	ENSMUSG00000024534	ENSMUSG00000024534	18	52893463	53041300	302	347
13708	5	122138649	122193699	ENSMUSG00000034484	ENSMUSG00000034484	ENSMUSG00000034484	18	53301734	53346229	302	347
13709	5	122209170	122372796	ENSMUSG00000024535	ENSMUSG00000024535	ENSMUSG00000024535	18	53371031	53516213	302	347
13710	5	122386979	122400324	ENSMUSG00000024538	ENSMUSG00000024538	ENSMUSG00000024538	18	53531710	53543376	302	347
13711	5	122452737	122551594	ENSMUSG00000069378	ENSMUSG00000069378	ENSMUSG00000069378	18	53589915	53701226	302	347
13712	5	122709997	122786937	ENSMUSG00000048799	ENSMUSG00000048799	ENSMUSG00000048799	18	53807095	53869922	302	347
13713	5	124000509	124108704	ENSMUSG00000052713	ENSMUSG00000052713	ENSMUSG00000052713	18	55015179	55113882	302	347
13714	5	125787000	125857945	ENSMUSG00000001700	ENSMUSG00000001700	ENSMUSG00000001700	18	56525715	56628208	302	347
13715	5	125908348	125958839	ENSMUSG00000053644	ENSMUSG00000053644	ENSMUSG00000053644	18	56651105	56698241	302	347
13716	5	125964506	125990326	ENSMUSG00000008301	ENSMUSG00000008301	ENSMUSG00000008301	18	56698371	56713081	302	347
13717	5	125995313	125999873	ENSMUSG00000032900	ENSMUSG00000032900	ENSMUSG00000032900	18	56713719	56720151	302	347
13718	5	126140214	126200582	ENSMUSG00000024590	ENSMUSG00000024590	ENSMUSG00000024590	18	56833233	56878791	302	347
13719	5	126233454	126394149	ENSMUSG00000032656	ENSMUSG00000032656	ENSMUSG00000032656	18	56887085	57050917	302	347
13720	5	126415368	126422586	ENSMUSG00000024592	ENSMUSG00000024592	ENSMUSG00000024592	18	57082457	57100725	302	347
13721	5	126654422	126824804	ENSMUSG00000024593	ENSMUSG00000024593	ENSMUSG00000024593	18	57258844	57422838	302	347
13722	5	126887057	126917499	ENSMUSG00000024594	ENSMUSG00000024594	ENSMUSG00000024594	18	57480186	57514982	302	347
13723	5	127012635	127022221	ENSMUSG00000069372	ENSMUSG00000069372	ENSMUSG00000069372	18	57593896	57603478	302	347
13724	5	127447382	127553278	ENSMUSG00000024597	ENSMUSG00000024597	ENSMUSG00000024597	18	58004047	58072174	302	347
13725	5	127621500	127901634	ENSMUSG00000024598	ENSMUSG00000024598	ENSMUSG00000024598	18	58135506	58335174	302	347
13726	5	128328720	128396887	ENSMUSG00000024600	ENSMUSG00000024600	ENSMUSG00000024600	18	58681609	58738238	302	347
13727	5	128458359	128476590	ENSMUSG00000024601	ENSMUSG00000024601	ENSMUSG00000024601	18	58784844	58803609	302	347
13728	5	128824002	129102275	ENSMUSG00000053441	ENSMUSG00000053441	ENSMUSG00000053441	18	58962133	59179047	302	347
13729	5	129123970	129128652	ENSMUSG00000050875	ENSMUSG00000050875	ENSMUSG00000050875	18	59187878	59202326	302	347
13730	5	129268422	129549383	ENSMUSG00000058152	ENSMUSG00000058152	ENSMUSG00000058152	18	59301772	59535181	302	347
13731	5	150068503	150118774	ENSMUSG00000024603	ENSMUSG00000024603	ENSMUSG00000024603	18	60651590	60684131	303	348
13732	5	150050549	150060817	ENSMUSG00000024604	ENSMUSG00000024604	ENSMUSG00000024604	18	60686137	60698179	303	348
13733	5	149960835	150012089	ENSMUSG00000043079	ENSMUSG00000043079	ENSMUSG00000043079	18	60725581	60755202	303	348
13734	5	149867947	149913642	ENSMUSG00000054008	ENSMUSG00000054008	ENSMUSG00000054008	18	60811332	60838758	303	348
13735	5	149762254	149772516	ENSMUSG00000024610	ENSMUSG00000024610	ENSMUSG00000024610	18	60929225	60938017	303	348
13736	5	149717428	149760063	ENSMUSG00000024613	ENSMUSG00000024613	ENSMUSG00000024613	18	60941362	60974227	303	348
13737	5	149656970	149662129	ENSMUSG00000036412	ENSMUSG00000036412	ENSMUSG00000036412	18	61037609	61043137	303	348
13738	5	149582736	149649385	ENSMUSG00000024617	ENSMUSG00000024617	ENSMUSG00000024617	18	61050987	61113521	303	348
13739	5	149549713	149570827	ENSMUSG00000052026	ENSMUSG00000052026	ENSMUSG00000052026	18	61120749	61139568	303	348
13740	5	149526633	149544181	ENSMUSG00000024619	ENSMUSG00000024619	ENSMUSG00000024619	18	61144231	61161568	303	348
13741	5	149473598	149515615	ENSMUSG00000024620	ENSMUSG00000024620	ENSMUSG00000024620	18	61170519	61210428	303	348
13742	5	149413051	149473128	ENSMUSG00000024621	ENSMUSG00000024621	ENSMUSG00000024621	18	61230941	61256506	303	348
13743	5	149360489	149412883	ENSMUSG00000024622	ENSMUSG00000024622	ENSMUSG00000024622	18	61256640	61302419	303	348
13744	5	149320639	149347149	ENSMUSG00000034320	ENSMUSG00000034320	ENSMUSG00000034320	18	61322590	61336831	303	348
13745	5	149220380	149304549	ENSMUSG00000024575	ENSMUSG00000024575	ENSMUSG00000024575	18	61345868	61415119	303	348
13746	5	149090057	149207460	ENSMUSG00000033871	ENSMUSG00000033871	ENSMUSG00000033871	18	61423879	61525769	303	348
13747	5	148976294	148991944	ENSMUSG00000045094	ENSMUSG00000045094	ENSMUSG00000045094	18	61619163	61634648	303	348
13748	5	148865196	148910720	ENSMUSG00000024576	ENSMUSG00000024576	ENSMUSG00000024576	18	61680702	61713672	303	348
13749	5	148734025	148739031	ENSMUSG00000024578	ENSMUSG00000024578	ENSMUSG00000024578	18	61813304	61817907	303	348
13750	5	148717802	148729408	ENSMUSG00000024579	ENSMUSG00000024579	ENSMUSG00000024579	18	61822206	61833004	303	348
13751	5	148705252	148714338	ENSMUSG00000024580	ENSMUSG00000024580	ENSMUSG00000024580	18	61837793	61851700	303	348
13752	5	148631651	148701558	ENSMUSG00000033032	ENSMUSG00000033032	ENSMUSG00000033032	18	61848126	61884107	303	348
13753	5	148501326	148620192	ENSMUSG00000032735	ENSMUSG00000032735	ENSMUSG00000032735	18	61926664	61997085	303	348
13754	5	148364377	148422880	ENSMUSG00000045629	ENSMUSG00000045629	ENSMUSG00000045629	18	62078453	62141084	303	348
13755	5	148185001	148188447	ENSMUSG00000045730	ENSMUSG00000045730	ENSMUSG00000045730	18	62303865	62305121	303	348
13756	5	147842713	147996776	ENSMUSG00000026322	ENSMUSG00000026322	ENSMUSG00000026322	18	62449573	62593171	303	348
13757	5	147743739	147802583	ENSMUSG00000042211	ENSMUSG00000042211	ENSMUSG00000042211	18	62629529	62674083	303	348
13758	5	147672183	147675670	ENSMUSG00000060201	ENSMUSG00000060201	ENSMUSG00000060201	18	62717782	62721633	303	348
13759	18	10444543	10478697	ENSMUSG00000071847	ENSMUSG00000071847	ENSMUSG00000071847	18	63048033	63077647	304	349
13760	18	10516031	10540371	ENSMUSG00000024581	ENSMUSG00000024581	ENSMUSG00000024581	18	63103285	63124817	304	349
13761	18	10660864	10687814	ENSMUSG00000041482	ENSMUSG00000041482	ENSMUSG00000041482	18	63135583	63167055	304	349
13762	18	10721474	10760217	ENSMUSG00000073549	ENSMUSG00000073549	ENSMUSG00000073549	18	63199980	63212003	304	349
13763	18	10784970	10861413	ENSMUSG00000048320	ENSMUSG00000048320	ENSMUSG00000048320	18	63234892	63282730	304	349
13764	18	52421053	52469503	ENSMUSG00000024583	ENSMUSG00000024583	ENSMUSG00000024583	18	63788170	63817728	305	350
13765	18	52469614	52848034	ENSMUSG00000040560	ENSMUSG00000040560	ENSMUSG00000040560	18	63845600	64112728	305	350
13766	18	53169042	53178833	ENSMUSG00000056812	ENSMUSG00000056812	ENSMUSG00000056812	18	64379745	64397165	305	350
13767	18	53253915	53295062	ENSMUSG00000045991	ENSMUSG00000045991	ENSMUSG00000045991	18	64465733	64512126	305	350
13768	18	53366535	53404988	ENSMUSG00000024588	ENSMUSG00000024588	ENSMUSG00000024588	18	64581919	64614435	305	350
13769	18	53418894	53440016	ENSMUSG00000024587	ENSMUSG00000024587	ENSMUSG00000024587	18	64625034	64641873	305	350
13770	18	53466591	53550037	ENSMUSG00000039529	ENSMUSG00000039529	ENSMUSG00000039529	18	64654348	64786369	305	350
13771	18	54013720	54216369	ENSMUSG00000024589	ENSMUSG00000024589	ENSMUSG00000024589	18	65046505	65338560	305	350
13772	18	54299464	54447169	ENSMUSG00000032845	ENSMUSG00000032845	ENSMUSG00000032845	18	65390903	65433080	305	350
13773	18	54489598	54568350	ENSMUSG00000032688	ENSMUSG00000032688	ENSMUSG00000032688	18	65556332	65604192	305	350
13774	18	54681041	54804663	ENSMUSG00000042439	ENSMUSG00000042439	ENSMUSG00000042439	18	65705599	65814302	305	350
13775	18	55038380	55048980	ENSMUSG00000024517	ENSMUSG00000024517	ENSMUSG00000024517	18	65998863	66011948	305	350
13776	18	55086733	55091605	ENSMUSG00000024518	ENSMUSG00000024518	ENSMUSG00000024518	18	66060008	66064458	305	350
13777	18	55113634	55136861	ENSMUSG00000024519	ENSMUSG00000024519	ENSMUSG00000024519	18	66081093	66095552	305	350
13778	18	55148088	55177463	ENSMUSG00000041891	ENSMUSG00000041891	ENSMUSG00000041891	18	66106123	66127981	305	350
13779	18	55254120	55515554	ENSMUSG00000046318	ENSMUSG00000046318	ENSMUSG00000046318	18	66183640	66417136	305	350
13780	18	56189564	56190562	ENSMUSG00000047259	ENSMUSG00000047259	ENSMUSG00000047259	18	66984411	66985409	305	350
13781	18	11679263	11871921	ENSMUSG00000024524	ENSMUSG00000024524	ENSMUSG00000024524	18	67213705	67349624	306	351
13782	18	11873470	11898641	ENSMUSG00000062526	ENSMUSG00000062526	ENSMUSG00000062526	18	67350899	67371199	306	351
13783	18	11971427	12020877	ENSMUSG00000024525	ENSMUSG00000024525	ENSMUSG00000024525	18	67414592	67444210	306	351
13784	18	12244360	12267592	ENSMUSG00000024526	ENSMUSG00000024526	ENSMUSG00000024526	18	67469172	67493147	306	351
13785	18	12298215	12316551	ENSMUSG00000001473	ENSMUSG00000001473	ENSMUSG00000001473	18	67516088	67528117	306	351
13786	18	12319108	12367194	ENSMUSG00000024527	ENSMUSG00000024527	ENSMUSG00000024527	18	67530134	67574541	306	351
13787	18	12397928	12422234	ENSMUSG00000024530	ENSMUSG00000024530	ENSMUSG00000024530	18	67590218	67605950	306	351
13788	18	12436512	12647964	ENSMUSG00000024533	ENSMUSG00000024533	ENSMUSG00000024533	18	67613578	67678090	306	351
13789	18	12662632	12692703	ENSMUSG00000073542	ENSMUSG00000073542	ENSMUSG00000073542	18	67743170	67765532	306	351
13790	18	12692425	12715737	ENSMUSG00000024537	ENSMUSG00000024537	ENSMUSG00000024537	18	67766968	67779531	306	351
13791	18	12783248	12874334	ENSMUSG00000024539	ENSMUSG00000024539	ENSMUSG00000024539	18	67797358	67849964	306	351
13792	18	12937983	12977056	ENSMUSG00000024542	ENSMUSG00000024542	ENSMUSG00000024542	18	67900183	67975922	306	351
13793	18	13208795	13642753	ENSMUSG00000024544	ENSMUSG00000024544	ENSMUSG00000024544	18	68058626	68380918	306	351
13794	18	13655741	13672108	ENSMUSG00000038121	ENSMUSG00000038121	ENSMUSG00000038121	18	68392047	68425589	306	351
13795	18	13721473	13752159	ENSMUSG00000009535	ENSMUSG00000009535	ENSMUSG00000009535	18	68425758	68449176	306	351
13796	18	13815543	13816861	ENSMUSG00000007480	ENSMUSG00000007480	ENSMUSG00000007480	18	68462965	68465092	306	351
13797	18	13872043	13905535	ENSMUSG00000045569	ENSMUSG00000045569	ENSMUSG00000045569	18	68532276	68554628	306	351
13798	18	51046093	51406858	ENSMUSG00000053477	ENSMUSG00000053477	ENSMUSG00000053477	18	69469890	69809713	307	352
13799	18	50719792	50777635	ENSMUSG00000038903	ENSMUSG00000038903	ENSMUSG00000038903	18	70050928	70094853	307	352
13800	18	50646706	50708124	ENSMUSG00000024511	ENSMUSG00000024511	ENSMUSG00000024511	18	70107054	70266948	307	352
13801	18	50409388	50417722	ENSMUSG00000071843	ENSMUSG00000071843	ENSMUSG00000071843	18	70393089	70401622	307	352
13802	18	50140941	50160736	ENSMUSG00000044906	ENSMUSG00000044906	ENSMUSG00000044906	18	70578509	70597849	307	352
13803	18	50049923	50075090	ENSMUSG00000038425	ENSMUSG00000038425	ENSMUSG00000038425	18	70634052	70655690	307	352
13804	18	49934573	50005156	ENSMUSG00000024513	ENSMUSG00000024513	ENSMUSG00000024513	18	70693557	70751499	307	352
13805	18	48121156	49311021	ENSMUSG00000060534	ENSMUSG00000060534	ENSMUSG00000060534	18	71384359	72475815	307	352
13806	18	46954920	46977380	ENSMUSG00000037253	ENSMUSG00000037253	ENSMUSG00000037253	18	73698977	73716162	307	352
13807	18	46810611	46860142	ENSMUSG00000024515	ENSMUSG00000024515	ENSMUSG00000024515	18	73764378	73829110	307	352
13808	18	46748385	46768488	ENSMUSG00000036941	ENSMUSG00000036941	ENSMUSG00000036941	18	73863540	73879818	307	352
13809	18	46659433	46728256	ENSMUSG00000024556	ENSMUSG00000024556	ENSMUSG00000024556	18	73895272	73940797	307	352
13810	18	46579499	46605731	ENSMUSG00000064036	ENSMUSG00000064036	ENSMUSG00000064036	18	73984763	74003267	307	352
13811	18	46340482	46512189	ENSMUSG00000024558	ENSMUSG00000024558	ENSMUSG00000024558	18	74053858	74190298	307	352
13812	18	46156199	46172615	ENSMUSG00000036223	ENSMUSG00000036223	ENSMUSG00000036223	18	74320663	74333023	307	352
13813	18	46062717	46068690	ENSMUSG00000024560	ENSMUSG00000024560	ENSMUSG00000024560	18	74342536	74346574	307	352
13814	18	46049215	46061844	ENSMUSG00000024561	ENSMUSG00000024561	ENSMUSG00000024561	18	74393660	74408054	307	352
13815	18	46007564	46046863	ENSMUSG00000035394	ENSMUSG00000035394	ENSMUSG00000035394	18	74408491	74485349	307	352
13816	18	45603184	45975176	ENSMUSG00000025885	ENSMUSG00000025885	ENSMUSG00000025885	18	74567984	74896170	307	352
13817	18	45563873	45594249	ENSMUSG00000036880	ENSMUSG00000036880	ENSMUSG00000036880	18	74904577	74931572	307	352
13818	18	45342425	45373276	ENSMUSG00000053846	ENSMUSG00000053846	ENSMUSG00000053846	18	75064688	75086625	307	352
13819	18	44824172	45241077	ENSMUSG00000035765	ENSMUSG00000035765	ENSMUSG00000035765	18	75144141	75412333	307	352
13820	18	44700222	44731079	ENSMUSG00000025880	ENSMUSG00000025880	ENSMUSG00000025880	18	75492894	75521292	307	352
13821	18	44319574	44642274	ENSMUSG00000052928	ENSMUSG00000052928	ENSMUSG00000052928	18	75556570	75822919	307	352
13822	18	43809629	43821476	ENSMUSG00000044646	ENSMUSG00000044646	ENSMUSG00000044646	18	75945543	76273929	307	352
13823	18	43618435	43711221	ENSMUSG00000024563	ENSMUSG00000024563	ENSMUSG00000024563	18	76367290	76431096	307	352
13824	18	42887777	42916808	ENSMUSG00000025421	ENSMUSG00000025421	ENSMUSG00000025421	18	77134576	77175161	307	352
13825	18	42780785	42881656	ENSMUSG00000025420	ENSMUSG00000025420	ENSMUSG00000025420	18	77198021	77223705	307	352
13826	18	42646058	42751464	ENSMUSG00000025423	ENSMUSG00000025423	ENSMUSG00000025423	18	77301699	77357244	307	352
13827	18	42513079	42591037	ENSMUSG00000025425	ENSMUSG00000025425	ENSMUSG00000025425	18	77390303	77459772	307	352
13828	18	42343528	42435624	ENSMUSG00000032818	ENSMUSG00000032818	ENSMUSG00000032818	18	77536981	77615087	307	352
13829	18	42168185	42294781	ENSMUSG00000025427	ENSMUSG00000025427	ENSMUSG00000025427	18	77666785	77672819	307	352
13830	18	42008021	42100944	ENSMUSG00000047466	ENSMUSG00000047466	ENSMUSG00000047466	18	77837357	77918086	307	352
13831	18	41938296	41962279	ENSMUSG00000041840	ENSMUSG00000041840	ENSMUSG00000041840	18	77961643	77971856	307	352
13832	18	41918108	41938197	ENSMUSG00000025428	ENSMUSG00000025428	ENSMUSG00000025428	18	77978072	77986938	307	352
13833	18	41817501	41906221	ENSMUSG00000071842	ENSMUSG00000071842	ENSMUSG00000071842	18	78039201	78083481	307	352
13834	18	41659692	41678045	ENSMUSG00000071841	ENSMUSG00000071841	ENSMUSG00000071841	18	78205732	78219344	307	352
13835	18	41558157	41585046	ENSMUSG00000059336	ENSMUSG00000059336	ENSMUSG00000059336	18	78263725	78282370	307	352
13836	18	41458628	41516482	ENSMUSG00000024552	ENSMUSG00000024552	ENSMUSG00000024552	18	78308224	78488490	307	352
13837	18	40535467	40898771	ENSMUSG00000024548	ENSMUSG00000024548	ENSMUSG00000024548	18	78916076	79249018	307	352
13838	18	76016114	76106388	ENSMUSG00000056214	ENSMUSG00000056214	ENSMUSG00000056214	18	80209011	80281439	308	353
13839	18	75967903	75999217	ENSMUSG00000053950	ENSMUSG00000053950	ENSMUSG00000053950	18	80291080	80304815	308	353
13840	18	75895346	75907373	ENSMUSG00000024570	ENSMUSG00000024570	ENSMUSG00000024570	18	80354324	80362696	308	353
13841	18	75763476	75812018	ENSMUSG00000034006	ENSMUSG00000034006	ENSMUSG00000034006	18	80415351	80454682	308	353
13842	18	75724656	75760804	ENSMUSG00000059852	ENSMUSG00000059852	ENSMUSG00000059852	18	80457718	80458467	308	353

13843	18	75540789	75615495	ENSMUSG00000033323	ENSMUSG00000033323	ENSMUSG00000033323	18	80570018	80631726	308	353
13844	18	75261314	75390310	ENSMUSG00000033016	ENSMUSG00000033016	ENSMUSG00000033016	18	80797750	80874825	308	353
13845	18	74930385	75239266	ENSMUSG00000024566	ENSMUSG00000024566	ENSMUSG00000024566	18	80896202	81096042	308	353
13846	18	74841263	74859182	ENSMUSG00000024565	ENSMUSG00000024565	ENSMUSG00000024565	18	81131280	81136683	308	353
13847	18	73090721	73111081	ENSMUSG00000024553	ENSMUSG00000024553	ENSMUSG00000024553	18	82527585	82540899	308	353
13848	18	72819778	72973762	ENSMUSG00000041607	ENSMUSG00000041607	ENSMUSG00000041607	18	82609551	82720065	308	353
13849	18	72201218	72336134	ENSMUSG00000058881	ENSMUSG00000058881	ENSMUSG00000058881	18	83090047	83137158	308	353
13850	18	71052130	71130665	ENSMUSG00000046982	ENSMUSG00000046982	ENSMUSG00000046982	18	84146749	84220583	308	353
13851	18	71039477	71050105	ENSMUSG00000049090	ENSMUSG00000049090	ENSMUSG00000049090	18	84222351	84231707	308	353
13852	18	70474282	70761999	ENSMUSG00000048410	ENSMUSG00000048410	ENSMUSG00000048410	18	84341897	84693116	308	353
13853	18	70352817	70403234	ENSMUSG00000056162	ENSMUSG00000056162	ENSMUSG00000056162	18	84744708	84784277	308	353
13854	18	70314577	70339336	ENSMUSG00000024644	ENSMUSG00000024644	ENSMUSG00000024644	18	84801658	84819826	308	353
13855	18	70253972	70275483	ENSMUSG00000047992	ENSMUSG00000047992	ENSMUSG00000047992	18	84854435	84874629	308	353
13856	18	70071508	70110201	ENSMUSG00000024646	ENSMUSG00000024646	ENSMUSG00000024646	18	84985602	85014051	308	353
13857	18	69966733	69977177	ENSMUSG00000024645	ENSMUSG00000024645	ENSMUSG00000024645	18	85081483	85085717	308	353
13858	18	69891581	69966020	ENSMUSG00000034391	ENSMUSG00000034391	ENSMUSG00000034391	18	85091205	85115669	308	353
13859	18	68560531	68685790	ENSMUSG00000050321	ENSMUSG00000050321	ENSMUSG00000050321	18	86529809	86637024	308	353
13860	18	68354895	68362542	ENSMUSG00000024647	ENSMUSG00000024647	ENSMUSG00000024647	18	86845303	86851785	308	353
13861	18	66107243	66145328	ENSMUSG00000056153	ENSMUSG00000056153	ENSMUSG00000056153	18	89002071	89028400	308	353
13862	18	65822025	66023944	ENSMUSG00000023066	ENSMUSG00000023066	ENSMUSG00000023066	18	89105983	89265209	308	353
13863	18	65681175	65775140	ENSMUSG00000034028	ENSMUSG00000034028	ENSMUSG00000034028	18	89331620	89404520	308	353
13864	18	65219271	65660357	ENSMUSG00000073514	ENSMUSG00000073514	ENSMUSG00000073514	18	89435328	89903334	308	353
13865	18	64491907	64533342	ENSMUSG00000024614	ENSMUSG00000024614	ENSMUSG00000024614	18	90644458	90677447	308	353
13866	11	68427927	68464643	ENSMUSG00000024831	ENSMUSG00000024831	ENSMUSG00000024831	19	3259364	3282906	309	354
13867	11	68415323	68427879	ENSMUSG00000024829	ENSMUSG00000024829	ENSMUSG00000024829	19	3283047	3292837	309	354
13868	11	68278934	68365960	ENSMUSG00000024900	ENSMUSG00000024900	ENSMUSG00000024900	19	3323320	3385732	309	354
13869	11	68231486	68275531	ENSMUSG00000024905	ENSMUSG00000024905	ENSMUSG00000024905	19	3388869	3407784	309	354
13870	11	68208602	68215218	ENSMUSG00000024907	ENSMUSG00000024907	ENSMUSG00000024907	19	3410095	3414112	309	354
13871	11	67984775	68139373	ENSMUSG00000024908	ENSMUSG00000024908	ENSMUSG00000024908	19	3454928	3575720	309	354
13872	11	67836674	67973301	ENSMUSG00000024913	ENSMUSG00000024913	ENSMUSG00000024913	19	3584836	3686546	309	354
13873	11	67785689	67792270	ENSMUSG00000035372	ENSMUSG00000035372	ENSMUSG00000035372	19	3708333	3717881	309	354
13874	11	67680083	67737360	ENSMUSG00000045098	ENSMUSG00000045098	ENSMUSG00000045098	19	3768022	3816391	309	354
13875	11	67576926	67645434	ENSMUSG00000024843	ENSMUSG00000024843	ENSMUSG00000024843	19	3851773	3894365	309	354
13876	11	67563059	67574941	ENSMUSG00000001750	ENSMUSG00000001750	ENSMUSG00000001750	19	3896052	3907112	309	354
13877	11	67554670	67560686	ENSMUSG00000059734	ENSMUSG00000059734	ENSMUSG00000059734	19	3908943	3911746	309	354
13878	11	67166605	67174706	ENSMUSG00000024866	ENSMUSG00000024866	ENSMUSG00000024866	19	3986571	3990003	309	354
13879	11	67155351	67163607	ENSMUSG00000037477	ENSMUSG00000037477	ENSMUSG00000037477	19	3992752	3999512	309	354
13880	11	67151993	67153977	ENSMUSG00000024869	ENSMUSG00000024869	ENSMUSG00000024869	19	4000580	4002102	309	354
13881	11	67130974	67136581	ENSMUSG00000037916	ENSMUSG00000037916	ENSMUSG00000037916	19	4007505	4012725	309	354
13882	11	67042995	67047475	ENSMUSG00000024857	ENSMUSG00000024857	ENSMUSG00000024857	19	4083490	4087342	309	354
13883	11	67030545	67032678	ENSMUSG00000024856	ENSMUSG00000024856	ENSMUSG00000024856	19	4097351	4099017	309	354
13884	11	67015815	67029366	ENSMUSG00000024851	ENSMUSG00000024851	ENSMUSG00000024851	19	4100117	4113964	309	354
13885	11	67007097	67015150	ENSMUSG00000024847	ENSMUSG00000024847	ENSMUSG00000024847	19	4114446	4125827	309	354
13886	11	66988406	66993319	ENSMUSG00000024845	ENSMUSG00000024845	ENSMUSG00000024845	19	4125959	4132271	309	354
13887	11	66979394	66983227	ENSMUSG00000024842	ENSMUSG00000024842	ENSMUSG00000024842	19	4135423	4139609	309	354
13888	11	66975359	66976771	ENSMUSG00000044724	ENSMUSG00000044724	ENSMUSG00000044724	19	4139799	4145750	309	354
13889	11	66959557	66967531	ENSMUSG00000024835	ENSMUSG00000024835	ENSMUSG00000024835	19	4148663	4154035	309	354
13890	11	66952511	66959454	ENSMUSG00000024830	ENSMUSG00000024830	ENSMUSG00000024830	19	4153991	4163297	309	354
13891	11	66940243	66949652	ENSMUSG00000075289	ENSMUSG00000075289	ENSMUSG00000075289	19	4165338	4166330	309	354
13892	11	66928176	66934133	ENSMUSG00000040247	ENSMUSG00000040247	ENSMUSG00000040247	19	4184372	4191047	309	354
13893	11	66922228	66925952	ENSMUSG00000040385	ENSMUSG00000040385	ENSMUSG00000040385	19	4192157	4195419	309	354
13894	11	66915999	66922437	ENSMUSG00000024824	ENSMUSG00000024824	ENSMUSG00000024824	19	4195198	4201603	309	354
13895	11	66888219	66897782	ENSMUSG00000040663	ENSMUSG00000040663	ENSMUSG00000040663	19	4214392	4222615	309	354
13896	11	66875597	66877593	ENSMUSG00000024854	ENSMUSG00000024854	ENSMUSG00000024854	19	4231937	4233631	309	354
13897	11	66827577	66836646	ENSMUSG00000034616	ENSMUSG00000034616	ENSMUSG00000034616	19	4261668	4269172	309	354
13898	11	66813395	66826530	ENSMUSG00000005986	ENSMUSG00000005986	ENSMUSG00000005986	19	4270182	4283137	309	354
13899	11	66790507	66810602	ENSMUSG00000024858	ENSMUSG00000024858	ENSMUSG00000024858	19	4287100	4306014	309	354
13900	11	66644074	66781717	ENSMUSG00000054611	ENSMUSG00000054611	ENSMUSG00000054611	19	4319103	4394833	309	354
13901	11	66580897	66596059	ENSMUSG00000041845	ENSMUSG00000041845	ENSMUSG00000041845	19	4425457	4439424	309	354
13902	11	66530825	66574890	ENSMUSG00000049303	ENSMUSG00000049303	ENSMUSG00000049303	19	4445908	4477143	309	354
13903	11	66499324	66500298	ENSMUSG00000042041	ENSMUSG00000042041	ENSMUSG00000042041	19	4496783	4498583	309	354
13904	11	66372605	66396206	ENSMUSG00000024892	ENSMUSG00000024892	ENSMUSG00000024892	19	4601600	4621288	309	354
13905	11	66381549	66384521	ENSMUSG00000045045	ENSMUSG00000045045	ENSMUSG00000045045	19	4611792	4615502	309	354
13906	11	66367459	66370593	ENSMUSG00000024889	ENSMUSG00000024889	ENSMUSG00000024889	19	4622551	4625617	309	354
13907	11	66312206	66367339	ENSMUSG00000071691	ENSMUSG00000071691	ENSMUSG00000071691	19	4660031	4698315	309	354
13908	11	66209299	66245446	ENSMUSG00000067889	ENSMUSG00000067889	ENSMUSG00000067889	19	4711223	4752352	309	354
13909	11	66189048	66201910	ENSMUSG00000033760	ENSMUSG00000033760	ENSMUSG00000033760	19	4756525	4765940	309	354
13910	11	66162729	66170515	ENSMUSG00000056951	ENSMUSG00000056951	ENSMUSG00000056951	19	4784293	4793877	309	354
13911	11	66140673	66151390	ENSMUSG00000006456	ENSMUSG00000006456	ENSMUSG00000006456	19	4800569	4811599	309	354
13912	11	66117245	66130065	ENSMUSG00000034108	ENSMUSG00000034108	ENSMUSG00000034108	19	4825367	4839287	309	354
13913	11	66114513	66117062	ENSMUSG00000067872	ENSMUSG00000067872	ENSMUSG00000067872	19	4839415	4842163	309	354
13914	11	66087516	66092623	ENSMUSG00000006458	ENSMUSG00000006458	ENSMUSG00000006458	19	4855129	4860912	309	354
13915	11	66070985	66087237	ENSMUSG00000006457	ENSMUSG00000006457	ENSMUSG00000006457	19	4861223	4877884	309	354
13916	11	66063311	66070247	ENSMUSG00000006463	ENSMUSG00000006463	ENSMUSG00000006463	19	4878727	4885392	309	354
13917	11	66004456	66033702	ENSMUSG00000063904	ENSMUSG00000063904	ENSMUSG00000063904	19	4907232	4928252	309	354
13918	11	65990991	66001374	ENSMUSG00000024901	ENSMUSG00000024901	ENSMUSG00000024901	19	4931854	4943050	309	354
13919	11	65959159	65962859	ENSMUSG00000024902	ENSMUSG00000024902	ENSMUSG00000024902	19	4962315	4964943	309	354
13920	11	65945051	65950753	ENSMUSG00000045903	ENSMUSG00000045903	ENSMUSG00000045903	19	4984353	4989971	309	354
13921	11	65886569	65896221	ENSMUSG00000024891	ENSMUSG00000024891	ENSMUSG00000024891	19	5023860	5031964	309	354
13922	11	65869420	65871676	ENSMUSG00000047379	ENSMUSG00000047379	ENSMUSG00000047379	19	5038853	5041130	309	354
13923	11	65861380	65869158	ENSMUSG00000024888	ENSMUSG00000024888	ENSMUSG00000024888	19	5045900	5049037	309	354
13924	11	65856118	65860576	ENSMUSG00000024883	ENSMUSG00000024883	ENSMUSG00000024883	19	5050808	5057095	309	354
13925	11	65838801	65841074	ENSMUSG00000056481	ENSMUSG00000056481	ENSMUSG00000056481	19	5068078	5070640	309	354
13926	11	65815917	65820707	ENSMUSG00000061451	ENSMUSG00000061451	ENSMUSG00000061451	19	5079333	5085477	309	354
13927	11	65808667	65813196	ENSMUSG00000024875	ENSMUSG00000024875	ENSMUSG00000024875	19	5088553	5092856	309	354
13928	11	65802237	65807644	ENSMUSG00000024873	ENSMUSG00000024873	ENSMUSG00000024873	19	5092879	5098507	309	354
13929	11	65792681	65801537	ENSMUSG00000024870	ENSMUSG00000024870	ENSMUSG00000024870	19	5099207	5106996	309	354
13930	11	65781805	65791906	ENSMUSG00000024862	ENSMUSG00000024862	ENSMUSG00000024862	19	5107748	5118252	309	354
13931	11	65594400	65768789	ENSMUSG00000024855	ENSMUSG00000024855	ENSMUSG00000024855	19	5133685	5273119	309	354
13932	11	65576392	65592958	ENSMUSG00000024853	ENSMUSG00000024853	ENSMUSG00000024853	19	5273932	5295448	309	354
13933	11	65566029	65573227	ENSMUSG00000047658	ENSMUSG00000047658	ENSMUSG00000047658	19	5298341	5308739	309	354
13934	11	65540800	65550564	ENSMUSG00000038498	ENSMUSG00000038498	ENSMUSG00000038498	19	5335741	5344153	309	354
13935	11	65536038	65537551	ENSMUSG00000024846	ENSMUSG00000024846	ENSMUSG00000024846	19	5344705	5349574	309	354
13936	11	65526126	65528192	ENSMUSG00000024844	ENSMUSG00000024844	ENSMUSG00000024844	19	5364641	5366651	309	354
13937	11	65520593	65526154	ENSMUSG00000024841	ENSMUSG00000024841	ENSMUSG00000024841	19	5366813	5371511	309	354
13938	11	65485776	65504183	ENSMUSG00000039148	ENSMUSG00000039148	ENSMUSG00000039148	19	5380060	5388703	309	354
13939	11	65469691	65484010	ENSMUSG00000039330	ENSMUSG00000039330	ENSMUSG00000039330	19	5390049	5394401	309	354
13940	11	65443304	65445607	ENSMUSG00000024914	ENSMUSG00000024914	ENSMUSG00000024914	19	5422811	5424916	309	354
13941	11	65440861	65443107	ENSMUSG00000047423	ENSMUSG00000047423	ENSMUSG00000047423	19	5425157	5427319	309	354
13942	11	65416268	65424573	ENSMUSG00000024912	ENSMUSG00000024912	ENSMUSG00000024912	19	5447698	5455938	309	354
13943	11	65407787	65412586	ENSMUSG00000024911	ENSMUSG00000024911	ENSMUSG00000024911	19	5460629	5465049	309	354
13944	11	65403860	65407786	ENSMUSG00000024910	ENSMUSG00000024910	ENSMUSG00000024910	19	5465044	5468499	309	354
13945	11	65390489	65397655	ENSMUSG00000024909	ENSMUSG00000024909	ENSMUSG00000024909	19	5474701	5481852	309	354
13946	11	65384525	65390485	ENSMUSG00000024906	ENSMUSG00000024906	ENSMUSG00000024906	19	5482851	5488339	309	354
13947	11	65357986	65377746	ENSMUSG00000056185	ENSMUSG00000056185	ENSMUSG00000056185	19	5495274	5510489	309	354
13948	11	65311105	65321259	ENSMUSG00000024922	ENSMUSG00000024922	ENSMUSG00000024922	19	5549137	5560575	309	354
13949	11	65241746	65244829	ENSMUSG00000024925	ENSMUSG00000024925	ENSMUSG00000024925	19	5601873	5602959	309	354
13950	11	65236315	65243650	ENSMUSG00000024926	ENSMUSG00000024926	ENSMUSG00000024926	19	5603418	5610029	309	354
13951	11	65178425	65186959	ENSMUSG00000024927	ENSMUSG00000024927	ENSMUSG00000024927	19	5637490	5648130	309	354
13952	11	65150900	65161352	ENSMUSG00000054874	ENSMUSG00000054874	ENSMUSG00000054874	19	5664641	5688908	309	354
13953	11	65121802	65138296	ENSMUSG00000004054	ENSMUSG00000004054	ENSMUSG00000004054	19	5689131	5702865	309	354
13954	11	65117052	65120043	ENSMUSG00000024936	ENSMUSG00000024936	ENSMUSG00000024936	19	5704367	5707100	309	354
13955	11	65100388	65116518	ENSMUSG00000024937	ENSMUSG00000024937	ENSMUSG00000024937	19	5708049	5726270	309	354
13956	11	65094519	65095793	ENSMUSG00000024939	ENSMUSG00000024939	ENSMUSG00000024939	19	5728088	5731713	309	354
13957	11	65062866	65082275	ENSMUSG00000024940	ENSMUSG00000024940	ENSMUSG00000024940	19	5742127	5758525	309	354
13958	11	65049160	65062748	ENSMUSG00000024941	ENSMUSG00000024941	ENSMUSG00000024941	19	5758427	5771401	309	354
13959	11	64910699	64937566	ENSMUSG00000024816	ENSMUSG00000024816	ENSMUSG00000024816	19	5852621	5874794	309	354
13960	11	64900241	64906420	ENSMUSG00000024818	ENSMUSG00000024818	ENSMUSG00000024818	19	5878466	5885768	309	354
13961	11	64878858	64881617	ENSMUSG00000044390	ENSMUSG00000044390	ENSMUSG00000044390	19	5891147	5894128	309	354
13962	11	64857920	64877027	ENSMUSG00000024826	ENSMUSG00000024826	ENSMUSG00000024826	19	5898555	5912823	309	354
13963	11	64838907	64846475	ENSMUSG00000045664	ENSMUSG00000045664	ENSMUSG00000045664	19	5916409	5924816	309	354
13964	11	64786006	64821663	ENSMUSG00000024833	ENSMUSG00000024833	ENSMUSG00000024833	19	5940546	5964170	309	354
13965	11	64737926	64766130	ENSMUSG00000037451	ENSMUSG00000037451	ENSMUSG00000037451	19	5970232	5986143	309	354
13966	11	64705792	64736048	ENSMUSG00000024942	ENSMUSG00000024942	ENSMUSG00000024942	19	5988546	6015163	309	354
13967	11	64651329	64658580	ENSMUSG00000024807	ENSMUSG00000024807	ENSMUSG00000024807	19	6046662	6053707	309	354
13968	11	64646304	64651417	ENSMUSG00000007338	ENSMUSG00000007338	ENSMUSG00000007338	19	6053630	6057751	309	354
13969	11	64636205	64640404	ENSMUSG00000024799	ENSMUSG00000024799	ENSMUSG00000024799	19	6062822	6067842	309	354
13970	11	64620259	64635756	ENSMUSG00000024797	ENSMUSG00000024797	ENSMUSG00000024797	19	6068115	6077203	309	354
13971	11	64612277	64612810	ENSMUSG00000047733	ENSMUSG00000047733	ENSMUSG00000047733	19	6080033	6080787	309	354
13972	11	64608270	64612450	ENSMUSG00000024792	ENSMUSG00000024792	ENSMUSG00000024792	19	6080765	6084904	309	354
13973	11	64601503	64608100	ENSMUSG00000024791	ENSMUSG00000024791	ENSMUSG00000024791	19	6085097	6091773	309	354
13974	11	64568873	64582585	ENSMUSG00000054999	ENSMUSG00000054999	ENSMUSG00000054999	19	6105798	6115555	309	354
13975	11	64564981	64568875	ENSMUSG00000024790	ENSMUSG00000024790	ENSMUSG00000024790	19	6116094	6118586	309	354
13976	11	64538228	64564618	ENSMUSG00000024787	ENSMUSG00000024787	ENSMUSG00000024787	19	6119399	6128215	309	354
13977	11	64511992	64521093	ENSMUSG00000039699	ENSMUSG00000039699	ENSMUSG00000039699	19	6164458	6172478	309	354
13978	11	64458521	64459936	ENSMUSG00000024784	ENSMUSG00000024784	ENSMUSG00000024784	19	6226401	6227768	309	354
13979	11	64448756	64458523	ENSMUSG00000024777	ENSMUSG00000024777	ENSMUSG00000024777	19	6227765	6235631	309	354
13980	11	64418595	64441239	ENSMUSG00000024773	ENSMUSG00000024773	ENSMUSG00000024773	19	6241727	6262304	309	354
13981	11	64376779	64403725	ENSMUSG00000024772	ENSMUSG00000024772	ENSMUSG00000024772	19	6276896	6300096	309	354
13982	11	64347435	64368617	ENSMUSG00000024769	ENSMUSG00000024769	ENSMUSG00000024769	19	6306716	6325646	309	354
13983	11	64327572	64335342	ENSMUSG00000024947	ENSMUSG00000024947	ENSMUSG00000024947	19	6335039	6340895	309	354
13984	11	64313185	64327289	ENSMUSG00000024948	ENSMUSG00000024948	ENSMUSG00000024948	19	6341250	6353527	309	354
13985	11	64288654	64302440	ENSMUSG00000024949	ENSMUSG00000024949	ENSMUSG00000024949	19	6363945	6376425	309	354
13986	11	64270437	64284022	ENSMUSG00000032648	ENSMUSG00000032648	ENSMUSG00000032648	19	6384429	6398459	309	354
13987	11	64250959	64269504	ENSMUSG00000032946	ENSMUSG00000032946	ENSMUSG00000032946	19	6400562	6415213	309	354
13988	11	64114858	64126396	ENSMUSG00000061742	ENSMUSG00000061742	ENSMUSG00000061742	19	6465196	6543006	309	354
13989	11	63883201	63896262	ENSMUSG00000024952	ENSMUSG00000024952	ENSMUSG00000024952	19	6896129	6907671	309	354
13990	11	63864271	63881582	ENSMUSG00000047810	ENSMUSG00000047810	ENSMUSG00000047810	19	6911667	6922282	309	354
13991	11	63842145	63845858	ENSMUSG00000024953	ENSMUSG00000024953	ENSMUSG00000024953	19	6973751	6977120	309	354
13992	11	63829616	63840786	ENSMUSG00000024955	ENSMUSG00000024955	ENSMUSG00000024955	19	6978022	6988862	309	354
13993	11	63815770	63828817	ENSMUSG00000024957	ENSMUSG00000024957	ENSMUSG00000024957	19	6992734	7001559	309	354
13994	11	63809907	63813539	ENSMUSG00000024958	ENSMUSG00000024958	ENSMUSG00000024958	19	7005114	7008523	309	354
13995	11	63793878	63808740	ENSMUSG00000024959	ENSMUSG00000024959	ENSMUSG00000024959	19	7008905	7018937	309	354
13996	11	63775698	63791604	ENSMUSG00000024960	ENSMUSG00000024960	ENSMUSG00000024960	19	7020758	7036804	309	354
13997	11	63764989	63768262	ENSMUSG00000056629	ENSMUSG00000056629	ENSMUSG00000056629	19	7044781	7047474	309	354
13998	11	63758842	63762834	ENSMUSG00000024962	ENSMUSG00000024962	ENSMUSG00000024962	19	7049516	7054647	309	354
13999	11	63754326	63758326	ENSMUSG00000024963	ENSMUSG00000024963	ENSMUSG00000024963	19	7054955	7059562	309	354
14000	11	63750338	63754063	ENSMUSG00000037349	ENSMUSG00000037349	ENSMUSG00000037349	19	7060063	7063081	309	354
14001	11	63747913	63750277	ENSMUSG00000047656	ENSMUSG00000047656	ENSMUSG00000047656	19	7063203	7066054	309	354
14002	11	63730782	63747930	ENSMUSG00000024965	ENSMUSG00000024965	ENSMUSG00000024965	19	7066020	7086475	309	354
14003	11	63709320	63728588	ENSMUSG00000024966	ENSMUSG00000024966	ENSMUSG00000024966	19	7088143	7102755	309	354
14004	11	63522607	63690109	ENSMUSG00000036278	ENSMUSG00000036278	ENSMUSG00000036278	19	7123935	7264659	309	354
14005	11	63627817	63643221	ENSMUSG00000047787	ENSMUSG00000047787	ENSMUSG00000047787	19	7162042	7172777	309	354
14006	11	63510461	63522463	ENSMUSG00000024767	ENSMUSG00000024767	ENSMUSG00000024767	19	7265250	7273328	309	354
14007	11	63498655	63500590	ENSMUSG00000035885	ENSMUSG00000035885	ENSMUSG00000035885	19	7282202	7284660	309	354
14008	11	63463044	63481020	ENSMUSG00000024764	ENSMUSG00000024764	ENSMUSG00000024764	19	7292718	7308117	309	354
14009	11	63363584	63433621	ENSMUSG00000024969	ENSMUSG00000024969	ENSMUSG00000024969	19	7344134	7357829	309	354
14010	11	63337436	63351727	ENSMUSG00000024970	ENSMUSG00000024970	ENSMUSG00000024970	19	7423508	7450038	309	354
14011	11	63287466	63290010	ENSMUSG00000053080	ENSMUSG00000053080	ENSMUSG00000053080	19	7487578	7492943	309	354
14012	11	63205531	63283913	ENSMUSG00000024758	ENSMUSG00000024758	ENSMUSG00000024758	19	7492945	7550333	309	354
14013	11	63153172	63196020	ENSMUSG00000024759	ENSMUSG00000024759	ENSMUSG00000024759	19	7561621	7602228	309	354
14014	11	63098825	63138471	ENSMUSG00000060675	ENSMUSG00000060675	ENSMUSG00000060675	19	7624503	7655590	309	354
14015	11	63030132	63040815	ENSMUSG00000024972	ENSMUSG00000024972	ENSMUSG00000024972	19	7663705	7674217	309	354
14016	11	62987386	63015242	ENSMUSG00000024973	ENSMUSG00000024973	ENSMUSG00000024973	19	7679614	7706283	309	354
14017	11	62517285	62539887	ENSMUSG00000063796	ENSMUSG00000063796	ENSMUSG00000063796	19	8658334	8678879	309	354
14018	11	62500646	62508738	ENSMUSG00000024650	ENSMUSG00000024650	ENSMUSG00000024650	19	8685224	8695344	309	354
14019	11	62432728	62445588	ENSMUSG00000032773	ENSMUSG00000032773	ENSMUSG00000032773	19	8731396	8748612	309	354
14020	11	62380094	62412928	ENSMUSG00000010095	ENSMUSG00000010095	ENSMUSG00000010095	19	8773927	8780921	309	354
14021	11	62356960	62363709	ENSMUSG00000042729	ENSMUSG00000042729	ENSMUSG00000042729	19	8802884	8807669	309	354
14022	11	62330946	62356136	ENSMUSG00000010110	ENSMUSG00000010110	ENSMUSG00000010110	19	8808536	8822680	309	354
14023	11	62316220	62329529	ENSMUSG00000010097	ENSMUSG00000010097	ENSMUSG00000010097	19	8824148	8837955	309	354
14024	11	62314503	62316056	ENSMUSG00000075043	ENSMUSG00000075043	ENSMUSG00000075043	19	8838041	8839520	309	354
14025	11	62295451	62311390	ENSMUSG00000003680	ENSMUSG00000003680	ENSMUSG00000003680	19	8841399	8853334	309	354
14026	11	62285591	62290757	ENSMUSG00000071662	ENSMUSG00000071662	ENSMUSG00000071662	19	8860173	8865621	309	354
14027	11	62276138	62278110	ENSMUSG00000071661	ENSMUSG00000071661	ENSMUSG00000071661	19	8869557	8871899	309	354
14028	11	62252160	62260267	ENSMUSG00000071660	ENSMUSG00000071660	ENSMUSG00000071660	19	8878341	8886333	309	354
14029	11	62231706	62233251	ENSMUSG00000071658	ENSMUSG00000071658	ENSMUSG00000071658	19	8903974	8906291	309	354
14030	11	62210452	62213776	ENSMUSG00000071656	ENSMUSG00000071656	ENSMUSG00000071656	19	8917830	8921631	309	354
14031	11	62200549	62203103	ENSMUSG00000071655	ENSMUSG00000071655	ENSMUSG00000071655	19	8938604	8942701	309	354
14032	11	62195734	62197619	ENSMUSG00000071654	ENSMUSG00000071654	ENSMUSG00000071654	19	8947320	8947903	309	354
14033	11	62149863	62170663	ENSMUSG00000071650	ENSMUSG00000071650	ENSMUSG00000071650	19	8965156	8983717	309	354
14034	11	62139381	62146023	ENSMUSG00000071649	ENSMUSG00000071649	ENSMUSG00000071649	19	8987438	8994281	309	354
14035	11	62137199	62139162	ENSMUSG00000071648	ENSMUSG00000071648	ENSMUSG00000071648	19	8994443	8996401	309	354
14036	11	62126267	62135645	ENSMUSG00000071647	ENSMUSG00000071647	ENSMUSG00000071647	19	8996718	9008625	309	354
14037	11	62117251	62125879	ENSMUSG00000071646	ENSMUSG00000071646	ENSMUSG00000071646	19	9008965	9019345	309	354

14038	11	62099099	62115592	ENSMUSG00000071645	ENSMUSG00000071645	ENSMUSG00000071645	19	9020883	9033255	309	354
14039	11	62039950	62070908	ENSMUSG00000069833	ENSMUSG00000069833	ENSMUSG00000069833	19	9067145	9085369	309	354
14040	11	61943099	61947242	ENSMUSG00000024653	ENSMUSG00000024653	ENSMUSG00000024653	19	9150685	9154981	309	354
14041	11	61861496	61917458	ENSMUSG00000024654	ENSMUSG00000024654	ENSMUSG00000024654	19	9178764	9202611	309	354
14042	11	61648021	61676372	ENSMUSG00000024660	ENSMUSG00000024660	ENSMUSG00000024660	19	9939391	9966514	309	354
14043	11	61474418	61487642	ENSMUSG00000037418	ENSMUSG00000037418	ENSMUSG00000037418	19	10052219	10064259	309	354
14044	11	61421350	61441573	ENSMUSG00000024663	ENSMUSG00000024663	ENSMUSG00000024663	19	10085273	10102631	309	354
14045	11	61352289	61391401	ENSMUSG00000024665	ENSMUSG00000024665	ENSMUSG00000024665	19	10129825	10168474	309	354
14046	11	61325947	61341039	ENSMUSG00000010663	ENSMUSG00000010663	ENSMUSG00000010663	19	10249966	10263915	309	354
14047	11	61316726	61321284	ENSMUSG00000024742	ENSMUSG00000024742	ENSMUSG00000024742	19	10266184	10270988	309	354
14048	11	61313179	61316658	ENSMUSG00000036372	ENSMUSG00000036372	ENSMUSG00000036372	19	10273469	10274335	309	354
14049	11	61276697	61312565	ENSMUSG00000036098	ENSMUSG00000036098	ENSMUSG00000036098	19	10283102	10307793	309	354
14050	11	61204486	61271048	ENSMUSG00000035735	ENSMUSG00000035735	ENSMUSG00000035735	19	10314709	10338633	309	354
14051	11	61039369	61104874	ENSMUSG00000024743	ENSMUSG00000024743	ENSMUSG00000024743	19	10456528	10515489	309	354
14052	11	61033113	61033838	ENSMUSG00000075030	ENSMUSG00000075030	ENSMUSG00000075030	19	10523659	10524375	309	354
14053	11	61005168	61014975	ENSMUSG00000035179	ENSMUSG00000035179	ENSMUSG00000035179	19	10541284	10549973	309	354
14054	11	60954173	60970801	ENSMUSG00000024668	ENSMUSG00000024668	ENSMUSG00000024668	19	10568685	10592254	309	354
14055	11	60926779	60953341	ENSMUSG00000034820	ENSMUSG00000034820	ENSMUSG00000034820	19	10592311	10614698	309	354
14056	11	60886083	60893239	ENSMUSG00000024666	ENSMUSG00000024666	ENSMUSG00000024666	19	10637523	10644407	309	354
14057	11	60872856	60886322	ENSMUSG00000034445	ENSMUSG00000034445	ENSMUSG00000034445	19	10649358	10662313	309	354
14058	11	60858777	60870964	ENSMUSG00000034371	ENSMUSG00000034371	ENSMUSG00000034371	19	10659673	10667805	309	354
14059	11	60823510	60857153	ENSMUSG00000024740	ENSMUSG00000024740	ENSMUSG00000024740	19	10672670	10696867	309	354
14060	11	60782336	60819122	ENSMUSG00000043789	ENSMUSG00000043789	ENSMUSG00000043789	19	10701278	10732186	309	354
14061	11	60654306	60685500	ENSMUSG00000048832	ENSMUSG00000048832	ENSMUSG00000048832	19	10755860	10781464	309	354
14062	11	60626543	60651895	ENSMUSG00000024669	ENSMUSG00000024669	ENSMUSG00000024669	19	10785188	10806019	309	354
14063	11	60495728	60544422	ENSMUSG00000024670	ENSMUSG00000024670	ENSMUSG00000024670	19	10857218	10867464	309	354
14064	11	60461134	60475833	ENSMUSG00000024737	ENSMUSG00000024737	ENSMUSG00000024737	19	10909589	10924948	309	354
14065	11	60448522	60461206	ENSMUSG00000024736	ENSMUSG00000024736	ENSMUSG00000024736	19	10924874	10936824	309	354
14066	11	60438253	60447489	ENSMUSG00000034659	ENSMUSG00000034659	ENSMUSG00000034659	19	10937705	10948788	309	354
14067	11	60414768	60430623	ENSMUSG00000024735	ENSMUSG00000024735	ENSMUSG00000024735	19	10955201	10972575	309	354
14068	11	60391591	60399740	ENSMUSG00000024734	ENSMUSG00000024734	ENSMUSG00000024734	19	10981334	10987677	309	354
14069	11	60374983	60380020	ENSMUSG00000034117	ENSMUSG00000034117	ENSMUSG00000034117	19	11004205	11009556	309	354
14070	11	60366174	60375129	ENSMUSG00000024732	ENSMUSG00000024732	ENSMUSG00000024732	19	11008526	11016298	309	354
14071	11	60309397	60325354	ENSMUSG00000024731	ENSMUSG00000024731	ENSMUSG00000024731	19	11029334	11041721	309	354
14072	11	60281053	60300781	ENSMUSG00000067571	ENSMUSG00000067571	ENSMUSG00000067571	19	11045352	11060295	309	354
14073	11	60223623	60239858	ENSMUSG00000024730	ENSMUSG00000024730	ENSMUSG00000024730	19	11134517	11148134	309	354
14074	11	60039462	60066765	ENSMUSG00000057240	ENSMUSG00000057240	ENSMUSG00000057240	19	11236463	11263768	309	354
14075	11	59979858	59994801	ENSMUSG00000024673	ENSMUSG00000024673	ENSMUSG00000024673	19	11316865	11333194	309	354
14076	11	59953638	59971835	ENSMUSG00000054523	ENSMUSG00000054523	ENSMUSG00000054523	19	11341210	11350918	309	354
14077	11	59920553	59941734	ENSMUSG00000024671	ENSMUSG00000024671	ENSMUSG00000024671	19	11368539	11381378	309	354
14078	11	59902534	59920000	ENSMUSG00000024672	ENSMUSG00000024672	ENSMUSG00000024672	19	11388084	11401740	309	354
14079	11	59804650	59832967	ENSMUSG00000071635	ENSMUSG00000071635	ENSMUSG00000071635	19	11445741	11459784	309	354
14080	11	59612713	59622593	ENSMUSG00000024680	ENSMUSG00000024680	ENSMUSG00000024680	19	11684238	11690688	309	354
14081	11	59580677	59595163	ENSMUSG00000024681	ENSMUSG00000024681	ENSMUSG00000024681	19	11696542	11707879	309	354
14082	11	59564324	59572090	ENSMUSG00000055895	ENSMUSG00000055895	ENSMUSG00000055895	19	11714329	11727604	309	354
14083	11	59353324	59369550	ENSMUSG00000024682	ENSMUSG00000024682	ENSMUSG00000024682	19	11814604	11830492	309	354
14084	11	59330190	59334921	ENSMUSG00000024683	ENSMUSG00000024683	ENSMUSG00000024683	19	11837436	11841985	309	354
14085	11	59279108	59326752	ENSMUSG00000041488	ENSMUSG00000041488	ENSMUSG00000041488	19	11844532	11886448	309	354
14086	11	59236965	59237894	ENSMUSG00000060878	ENSMUSG00000060878	ENSMUSG00000060878	19	11963068	11963997	309	354
14087	11	59160770	59193028	ENSMUSG00000046139	ENSMUSG00000046139	ENSMUSG00000046139	19	11979608	12010023	309	354
14088	11	59098447	59140193	ENSMUSG00000024687	ENSMUSG00000024687	ENSMUSG00000024687	19	12033160	12061139	309	354
14089	11	59027625	59028560	ENSMUSG00000067529	ENSMUSG00000067529	ENSMUSG00000067529	19	12102853	12103785	309	354
14090	11	58981010	58981954	ENSMUSG00000067524	ENSMUSG00000067524	ENSMUSG00000067524	19	12175645	12176589	309	354
14091	11	58967218	58968165	ENSMUSG00000067522	ENSMUSG00000067522	ENSMUSG00000067522	19	12186766	12187719	309	354
14092	11	58915528	58916358	ENSMUSG00000067520	ENSMUSG00000067520	ENSMUSG00000067520	19	12295660	12296589	309	354
14093	11	58696541	58732636	ENSMUSG00000039982	ENSMUSG00000039982	ENSMUSG00000039982	19	12533374	12568499	309	354
14094	11	58668828	58679083	ENSMUSG00000024691	ENSMUSG00000024691	ENSMUSG00000024691	19	12640570	12656741	309	354
14095	11	58232809	58256023	ENSMUSG00000063683	ENSMUSG00000063683	ENSMUSG00000063683	19	12700353	12718782	309	354
14096	11	58050925	58099910	ENSMUSG00000024696	ENSMUSG00000024696	ENSMUSG00000024696	19	12865611	12900826	309	354
14097	11	58031225	58032154	ENSMUSG00000046272	ENSMUSG00000046272	ENSMUSG00000046272	19	12928796	12929755	309	354
14098	11	57714539	57715483	ENSMUSG00000044040	ENSMUSG00000044040	ENSMUSG00000044040	19	13861684	13862628	309	354
14099	11	57703493	57704425	ENSMUSG00000054526	ENSMUSG00000054526	ENSMUSG00000054526	19	13894478	13895413	309	354
14100	9	81377350	81530075	ENSMUSG00000024642	ENSMUSG00000024642	ENSMUSG00000024642	19	14515169	14665118	310	
14101	9	80101879	80134829	ENSMUSG00000024640	ENSMUSG00000024640	ENSMUSG00000024640	19	15972152	15992078	310	355
14102	9	80040831	80076416	ENSMUSG00000041491	ENSMUSG00000041491	ENSMUSG00000041491	19	16022770	16052008	310	355
14103	9	79520823	79836194	ENSMUSG00000024639	ENSMUSG00000024639	ENSMUSG00000024639	19	16199850	16454472	310	355
14104	9	79227815	79453043	ENSMUSG00000024697	ENSMUSG00000024697	ENSMUSG00000024697	19	16502781	16677839	310	355
14105	9	78982181	79222219	ENSMUSG00000046230	ENSMUSG00000046230	ENSMUSG00000046230	19	16683690	16847946	310	355
14106	9	78824391	78825689	ENSMUSG00000056829	ENSMUSG00000056829	ENSMUSG00000056829	19	16939373	16940659	310	355
14107	9	78593080	78710821	ENSMUSG00000054366	ENSMUSG00000054366	ENSMUSG00000054366	19	17066958	17186552	310	355
14108	9	78419138	78515932	ENSMUSG00000039126	ENSMUSG00000039126	ENSMUSG00000039126	19	17189770	17288682	310	355
14109	9	78263966	78312152	ENSMUSG00000038843	ENSMUSG00000038843	ENSMUSG00000038843	19	17393162	17423636	310	355
14110	9	78190266	78199253	ENSMUSG00000024712	ENSMUSG00000024712	ENSMUSG00000024712	19	17461102	17468354	310	355
14111	9	76893279	76952001	ENSMUSG00000024725	ENSMUSG00000024725	ENSMUSG00000024725	19	18648289	18671232	310	355
14112	9	76865309	76892926	ENSMUSG00000037847	ENSMUSG00000037847	ENSMUSG00000037847	19	18703133	18712162	310	355
14113	9	76751317	76757622	ENSMUSG00000047044	ENSMUSG00000047044	ENSMUSG00000047044	19	18780255	18785447	310	355
14114	9	76527231	76692830	ENSMUSG00000024727	ENSMUSG00000024727	ENSMUSG00000024727	19	18817002	18959534	310	355
14115	9	76302072	76491937	ENSMUSG00000036192	ENSMUSG00000036192	ENSMUSG00000036192	19	19004903	19055146	310	355
14116	9	74956493	74975129	ENSMUSG00000024659	ENSMUSG00000024659	ENSMUSG00000024659	19	20440453	20457695	310	355
14117	9	74326537	74641087	ENSMUSG00000024749	ENSMUSG00000024749	ENSMUSG00000024749	19	20856683	20980648	310	355
14118	9	74159194	74169344	ENSMUSG00000024750	ENSMUSG00000024750	ENSMUSG00000024750	19	21339375	21349386	310	355
14119	9	73954113	74056959	ENSMUSG00000058624	ENSMUSG00000058624	ENSMUSG00000058624	19	21370247	21539684	310	355
14120	9	73716281	73787986	ENSMUSG00000035171	ENSMUSG00000035171	ENSMUSG00000035171	19	21664410	21719888	310	355
14121	9	73667188	73715968	ENSMUSG00000047368	ENSMUSG00000047368	ENSMUSG00000047368	19	21720406	21752734	310	355
14122	9	73488102	73573228	ENSMUSG00000024754	ENSMUSG00000024754	ENSMUSG00000024754	19	21845439	21923448	310	355
14123	9	72340689	72926090	ENSMUSG00000052387	ENSMUSG00000052387	ENSMUSG00000052387	19	22206216	23055405	310	355
14124	9	72189333	72219393	ENSMUSG00000033863	ENSMUSG00000033863	ENSMUSG00000033863	19	23208323	23234008	310	355
14125	9	72063757	72159609	ENSMUSG00000024943	ENSMUSG00000024943	ENSMUSG00000024943	19	23275925	23340915	310	355
14126	9	71848317	72031706	ENSMUSG00000033207	ENSMUSG00000033207	ENSMUSG00000033207	19	23369706	23515419	310	355
14127	9	71625529	71710968	ENSMUSG00000033053	ENSMUSG00000033053	ENSMUSG00000033053	19	23625857	23719911	310	355
14128	9	71514261	71564677	ENSMUSG00000074925	ENSMUSG00000074925	ENSMUSG00000074925	19	23754554	23787497	310	355
14129	9	71235022	71477042	ENSMUSG00000024897	ENSMUSG00000024897	ENSMUSG00000024897	19	23825973	24016694	310	355
14130	9	71134061	71197188	ENSMUSG00000071604	ENSMUSG00000071604	ENSMUSG00000071604	19	24040525	24046832	310	355
14131	9	70978901	71059940	ENSMUSG00000024812	ENSMUSG00000024812	ENSMUSG00000024812	19	24161608	24292051	310	355
14132	9	70839995	70878949	ENSMUSG00000059363	ENSMUSG00000059363	ENSMUSG00000059363	19	24328550	24347683	310	355
14133	9	70546991	70813912	ENSMUSG00000024867	ENSMUSG00000024867	ENSMUSG00000024867	19	24371129	24506218	310	355
14134	9	70584784	70588429	ENSMUSG00000074922	ENSMUSG00000074922	ENSMUSG00000074922	19	24543599	24544453	310	355
14135	9	70341316	70345603	ENSMUSG00000048572	ENSMUSG00000048572	ENSMUSG00000048572	19	24741102	24745357	310	355
14136	9	261625	455252	ENSMUSG00000052085	ENSMUSG00000052085	ENSMUSG00000052085	19	25066626	25269529	311	356
14137	9	460291	736105	ENSMUSG00000032702	ENSMUSG00000032702	ENSMUSG00000032702	19	25471374	25501593	311	356
14138	9	831690	959090	ENSMUSG00000024837	ENSMUSG00000024837	ENSMUSG00000024837	19	25572803	25671425	311	356
14139	9	966964	981732	ENSMUSG00000042372	ENSMUSG00000042372	ENSMUSG00000042372	19	25677634	25691016	311	356
14140	9	1040354	1047542	ENSMUSG00000048138	ENSMUSG00000048138	ENSMUSG00000048138	19	25739508	25746088	311	356
14141	9	2005342	2183624	ENSMUSG00000024921	ENSMUSG00000024921	ENSMUSG00000024921	19	26672257	26845418	311	356
14142	9	2611793	2644485	ENSMUSG00000024924	ENSMUSG00000024924	ENSMUSG00000024924	19	27284529	27316717	311	356
14143	9	2707502	2720037	ENSMUSG00000047298	ENSMUSG00000047298	ENSMUSG00000047298	19	27389685	27404275	311	356
14144	9	2794152	2834094	ENSMUSG00000041360	ENSMUSG00000041360	ENSMUSG00000041360	19	27456980	27496919	311	356
14145	9	3208297	3515983	ENSMUSG00000040929	ENSMUSG00000040929	ENSMUSG00000040929	19	27834136	28077137	311	356
14146	9	3814127	4142183	ENSMUSG00000052942	ENSMUSG00000052942	ENSMUSG00000052942	19	28328970	28606417	311	356
14147	9	4480444	4577469	ENSMUSG00000024935	ENSMUSG00000024935	ENSMUSG00000024935	19	28901132	28979956	311	356
14148	9	4578830	4656428	ENSMUSG00000064202	ENSMUSG00000064202	ENSMUSG00000064202	19	28967265	29033727	311	356
14149	9	4652315	4655255	ENSMUSG00000040105	ENSMUSG00000040105	ENSMUSG00000040105	19	29029917	29032791	311	356
14150	9	4669566	4698398	ENSMUSG00000024780	ENSMUSG00000024780	ENSMUSG00000024780	19	29056491	29083566	311	356
14151	9	4701155	4731318	ENSMUSG00000024782	ENSMUSG00000024782	ENSMUSG00000024782	19	29086831	29113952	311	356
14152	9	4782869	4851064	ENSMUSG00000024785	ENSMUSG00000024785	ENSMUSG00000024785	19	29167372	29209840	311	356
14153	9	4975245	5118183	ENSMUSG00000024789	ENSMUSG00000024789	ENSMUSG00000024789	19	29318438	29378334	311	356
14154	9	5153863	5175668	ENSMUSG00000050957	ENSMUSG00000050957	ENSMUSG00000050957	19	29387344	29391353	311	356
14155	9	5347973	5427878	ENSMUSG00000016495	ENSMUSG00000016495	ENSMUSG00000016495	19	29414603	29427890	311	356
14156	9	5440525	5460547	ENSMUSG00000016496	ENSMUSG00000016496	ENSMUSG00000016496	19	29433452	29454094	311	356
14157	9	5500570	5561252	ENSMUSG00000016498	ENSMUSG00000016498	ENSMUSG00000016498	19	29476955	29537158	311	356
14158	9	5646654	5764309	ENSMUSG00000038658	ENSMUSG00000038658	ENSMUSG00000038658	19	29599195	29671182	311	356
14159	9	5755076	5823070	ENSMUSG00000046324	ENSMUSG00000046324	ENSMUSG00000046324	19	29676191	29714358	311	356
14160	9	5880802	5900606	ENSMUSG00000024806	ENSMUSG00000024806	ENSMUSG00000024806	19	29763918	29774445	311	356
14161	9	5909022	5958348	ENSMUSG00000046138	ENSMUSG00000046138	ENSMUSG00000046138	19	29782008	29784032	311	356
14162	9	6205809	6247976	ENSMUSG00000024810	ENSMUSG00000024810	ENSMUSG00000024810	19	29991134	30026715	311	356
14163	9	6318428	6319073	ENSMUSG00000024815	ENSMUSG00000024815	ENSMUSG00000024815	19	30069787	30072015	311	356
14164	9	6403151	6497051	ENSMUSG00000024817	ENSMUSG00000024817	ENSMUSG00000024817	19	30096510	30159719	311	356
14165	9	6522464	6635650	ENSMUSG00000024827	ENSMUSG00000024827	ENSMUSG00000024827	19	30164449	30241426	311	356
14166	10	54195146	54201466	ENSMUSG00000024863	ENSMUSG00000024863	ENSMUSG00000024863	19	30298939	30305678	312	357
14167	10	53744064	53747595	ENSMUSG00000024868	ENSMUSG00000024868	ENSMUSG00000024868	19	30611873	30615493	312	357
14168	10	52503940	53728116	ENSMUSG00000052920	ENSMUSG00000052920	ENSMUSG00000052920	19	30635235	31730278	312	357
14169	10	53125253	53129357	ENSMUSG00000053536	ENSMUSG00000053536	ENSMUSG00000053536	19	31148966	31153066	312	357
14170	10	52236328	52315441	ENSMUSG00000052595	ENSMUSG00000052595	ENSMUSG00000052595	19	31934761	32014567	312	357
14171	10	51735352	52053743	ENSMUSG00000040451	ENSMUSG00000040451	ENSMUSG00000040451	19	32188730	32415313	312	357
14172	10	89409456	89497440	ENSMUSG00000024899	ENSMUSG00000024899	ENSMUSG00000024899	19	32686002	32733184	313	358
14173	10	89501249	89567897	ENSMUSG00000013662	ENSMUSG00000013662	ENSMUSG00000013662	19	32738557	32778319	313	358
14174	10	89612850	89721667	ENSMUSG00000013663	ENSMUSG00000013663	ENSMUSG00000013663	19	32823574	32892157	313	358
14175	10	90034696	90332950	ENSMUSG00000071573	ENSMUSG00000071573	ENSMUSG00000071573	19	33204122	33458069	313	358
14176	10	90414181	90428545	ENSMUSG00000024768	ENSMUSG00000024768	ENSMUSG00000024768	19	34027184	34042801	313	358
14177	10	90476541	90502493	ENSMUSG00000024771	ENSMUSG00000024771	ENSMUSG00000024771	19	34074298	34113828	313	358
14178	10	90511137	90527880	ENSMUSG00000024770	ENSMUSG00000024770	ENSMUSG00000024770	19	34134624	34150846	313	358
14179	10	90558035	90570232	ENSMUSG00000056078	ENSMUSG00000056078	ENSMUSG00000056078	19	34166965	34188684	313	358
14180	10	90571869	90601555	ENSMUSG00000024774	ENSMUSG00000024774	ENSMUSG00000024774	19	34189458	34215419	313	358
14181	10	90629471	90724890	ENSMUSG00000024776	ENSMUSG00000024776	ENSMUSG00000024776	19	34258267	34306328	313	358
14182	10	90684811	90702510	ENSMUSG00000035783	ENSMUSG00000035783	ENSMUSG00000035783	19	34307088	34321333	313	358
14183	10	90739206	90764692	ENSMUSG00000024778	ENSMUSG00000024778	ENSMUSG00000024778	19	34356663	34393767	313	358
14184	10	90955366	90957051	ENSMUSG00000050370	ENSMUSG00000050370	ENSMUSG00000050370	19	34540227	34541123	313	358
14185	10	90963308	91162588	ENSMUSG00000024781	ENSMUSG00000024781	ENSMUSG00000024781	19	34558311	34593419	313	358
14186	10	91051692	91059013	ENSMUSG00000045932	ENSMUSG00000045932	ENSMUSG00000045932	19	34616707	34642416	313	358
14187	10	91180753	91285293	ENSMUSG00000009378	ENSMUSG00000009378	ENSMUSG00000009378	19	34734403	34813108	313	358
14188	10	91333009	91395039	ENSMUSG00000033610	ENSMUSG00000033610	ENSMUSG00000033610	19	34876892	34945452	313	358
14189	10	91451354	91524676	ENSMUSG00000024795	ENSMUSG00000024795	ENSMUSG00000024795	19	35001683	35041740	313	358
14190	10	92490558	92607651	ENSMUSG00000024798	ENSMUSG00000024798	ENSMUSG00000024798	19	36025416	36122492	313	358
14191	10	92621255	92651928	ENSMUSG00000024800	ENSMUSG00000024800	ENSMUSG00000024800	19	36148875	36169921	313	358
14192	10	92661833	92671013	ENSMUSG00000024803	ENSMUSG00000024803	ENSMUSG00000024803	19	36177107	36184986	313	358
14193	10	92912774	93030715	ENSMUSG00000024805	ENSMUSG00000024805	ENSMUSG00000024805	19	36413598	36521361	313	358
14194	10	93160082	93264498	ENSMUSG00000041180	ENSMUSG00000041180	ENSMUSG00000041180	19	36619924	36686272	313	358
14195	10	93378183	93382838	ENSMUSG00000067279	ENSMUSG00000067279	ENSMUSG00000067279	19	36798559	36799512	313	358
14196	10	93548049	93615013	ENSMUSG00000024811	ENSMUSG00000024811	ENSMUSG00000024811	19	36899682	36955944	313	358
14197	10	93656326	93659220	ENSMUSG00000047632	ENSMUSG00000047632	ENSMUSG00000047632	19	36982690	36984743	313	358
14198	10	93673716	93780058	ENSMUSG00000040565	ENSMUSG00000040565	ENSMUSG00000040565	19	37051590	37076545	313	358
14199	10	93798379	94040824	ENSMUSG00000039652	ENSMUSG00000039652	ENSMUSG00000039652	19	37086435	37272437	313	358
14200	10	94201421	94323813	ENSMUSG00000056999	ENSMUSG00000056999	ENSMUSG00000056999	19	37335574	37395757	313	358
14201	10	94342971	94405130	ENSMUSG00000012443	ENSMUSG00000012443	ENSMUSG00000012443	19	37441547	37487003	313	358
14202	10	94439690	94445383	ENSMUSG00000024986	ENSMUSG00000024986	ENSMUSG00000024986	19	37499985	37505875	313	358
14203	10	94598222	94809241	ENSMUSG00000053799	ENSMUSG00000053799	ENSMUSG00000053799	19	37615562	37748397	313	358
14204	10	94811011	94818444	ENSMUSG00000062432	ENSMUSG00000062432	ENSMUSG00000062432	19	37750824	37758607	313	358
14205	10	94823637	94827637	ENSMUSG00000024987	ENSMUSG00000024987	ENSMUSG00000024987	19	37762998	37766680	313	358
14206	10	95056177	95232032	ENSMUSG00000048612	ENSMUSG00000048612	ENSMUSG00000048612	19	37964377	38108502	313	358
14207	10	95246379	95278839	ENSMUSG00000024989	ENSMUSG00000024989	ENSMUSG00000024989	19	38120184	38139565	313	358
14208	10	95316412	95339819	ENSMUSG00000054200	ENSMUSG00000054200	ENSMUSG00000054200	19	38162223	38179413	313	358
14209	10	95341434	95351410	ENSMUSG00000024990	ENSMUSG00000024990	ENSMUSG00000024990	19	38181773	38190392	313	358
14210	10	95362335	95415417	ENSMUSG00000024992	ENSMUSG00000024992	ENSMUSG00000024992	19	38198090	38248883	313	358
14211	10	95417630	95452319	ENSMUSG00000054237	ENSMUSG00000054237	ENSMUSG00000054237	19	38253902	38287057	313	358
14212	10	95507632	95547906	ENSMUSG00000067242	ENSMUSG00000067242	ENSMUSG00000067242	19	38329926	38374083	313	358
14213	10	95643720	95653571	ENSMUSG00000044026	ENSMUSG00000044026	ENSMUSG00000044026	19	38461124	38470751	313	358
14214	10	95743736	96078136	ENSMUSG00000024998	ENSMUSG00000024998	ENSMUSG00000024998	19	38589341	38848017	313	358
14215	10	96082973	96112686	ENSMUSG00000024999	ENSMUSG00000024999	ENSMUSG00000024999	19	38854689	38883289	313	358
14216	10	96152342	96283858	ENSMUSG00000048720	ENSMUSG00000048720	ENSMUSG00000048720	19	38901723	38984814	313	358
14217	10	96295669	96351369	ENSMUSG00000025001	ENSMUSG00000025001	ENSMUSG00000025001	19	38996098	39033417	313	358
14218	10	96433368	96485937	ENSMUSG00000025002	ENSMUSG00000025002	ENSMUSG00000025002	19	39072063	39107731	313	358
14219	10	96987319	97040771	ENSMUSG00000055044	ENSMUSG00000055044	ENSMUSG00000055044	19	40274484	40324825	313	358
14220	10	97061521	97190927	ENSMUSG00000025006	ENSMUSG00000025006	ENSMUSG00000025006	19	40348360	40451928	313	358
14221	10	97355677	97406557	ENSMUSG00000025007	ENSMUSG00000025007	ENSMUSG00000025007	19	40603568	40641680	313	358
14222	10	97413166	97443890	ENSMUSG00000025008	ENSMUSG00000025008	ENSMUSG00000025008	19	40649757	40665526	313	358
14223	10	97461526	97619442	ENSMUSG00000048120	ENSMUSG00000048120	ENSMUSG00000048120	19	40764473	40794913	313	358
14224	10	97671763	97688675	ENSMUSG00000058993	ENSMUSG00000058993	ENSMUSG00000058993	19	40818757	40849165	313	358
14225	10	97793141	97810612	ENSMUSG00000025010	ENSMUSG00000025010	ENSMUSG00000025010	19	40884590	40901765	313	358
14226	10	97905090	97913384	ENSMUSG00000049164	ENSMUSG00000049164	ENSMUSG00000049164	19	40964940	40969373	313	358
14227	10	97941453	98021316	ENSMUSG00000061132	ENSMUSG00000061132	ENSMUSG00000061132	19	40982484	41025964	313	358
14228	10	98054075	98088290	ENSMUSG00000025014	ENSMUSG00000025014	ENSMUSG00000025014	19	41082586	41112834	313	358
14229	10	98092963	98109049	ENSMUSG00000050121	ENSMUSG00000050121	ENSMUSG00000050121	19	41117114	41125542	313	358
14230	10	98114353	98263658	ENSMUSG00000025013	ENSMUSG00000025013	ENSMUSG00000025013	19	41136064	41260146	313	358
14231	10	98267856	98336799	ENSMUSG00000025016	ENSMUSG00000025016	ENSMUSG00000025016	19	41264153	41317315	313	358
14232	10	98343063	98470269	ENSMUSG00000025017	ENSMUSG00000025017	ENSMUSG00000025017	19	41327607	41429589	313	358

14233	10	98582029	98708680	ENSMUSG00000025019	ENSMUSG00000025019	ENSMUSG00000025019	19	41581365	41615557	313	358
14234	10	98731031	98735959	ENSMUSG00000043269	ENSMUSG00000043269	ENSMUSG00000043269	19	41635681	41639847	313	358
14235	10	98747785	98935673	ENSMUSG00000025020	ENSMUSG00000025020	ENSMUSG00000025020	19	41653570	41797167	313	358
14236	10	99106448	99151090	ENSMUSG00000035049	ENSMUSG00000035049	ENSMUSG00000035049	19	41916167	41949484	313	358
14237	10	99185889	99195764	ENSMUSG00000034321	ENSMUSG00000034321	ENSMUSG00000034321	19	41976877	41986607	313	358
14238	10	99195917	99207113	ENSMUSG00000025157	ENSMUSG00000025157	ENSMUSG00000025157	19	41986826	41997415	313	358
14239	10	99208071	99248503	ENSMUSG00000025159	ENSMUSG00000025159	ENSMUSG00000025159	19	41997018	42034447	313	358
14240	10	99248615	99320956	ENSMUSG00000025171	ENSMUSG00000025171	ENSMUSG00000025171	19	42035074	42087952	313	358
14241	10	99322188	99333606	ENSMUSG00000025172	ENSMUSG00000025172	ENSMUSG00000025172	19	42089337	42098421	313	358
14242	10	99334090	99362549	ENSMUSG00000025176	ENSMUSG00000025176	ENSMUSG00000025176	19	42099162	42124250	313	358
14243	10	99364767	99383334	ENSMUSG00000049670	ENSMUSG00000049670	ENSMUSG00000049670	19	42129421	42139755	313	358
14244	10	99390433	99426181	ENSMUSG00000025178	ENSMUSG00000025178	ENSMUSG00000025178	19	42143746	42175529	313	358
14245	10	99427452	99429652	ENSMUSG00000018821	ENSMUSG00000018821	ENSMUSG00000018821	19	42176586	42182304	313	358
14246	10	99463469	99467897	ENSMUSG00000044345	ENSMUSG00000044345	ENSMUSG00000044345	19	42200746	42205009	313	358
14247	10	99486875	99510642	ENSMUSG00000018820	ENSMUSG00000018820	ENSMUSG00000018820	19	42223932	42247904	313	358
14248	10	99516369	99521727	ENSMUSG00000018822	ENSMUSG00000018822	ENSMUSG00000018822	19	42251282	42255442	313	358
14249	10	99599986	99617772	ENSMUSG00000042532	ENSMUSG00000042532	ENSMUSG00000042532	19	42309051	42323659	313	358
14250	10	99614747	99780575	ENSMUSG00000042401	ENSMUSG00000042401	ENSMUSG00000042401	19	42336346	42484958	313	358
14251	10	99913034	99994639	ENSMUSG00000025184	ENSMUSG00000025184	ENSMUSG00000025184	19	42572116	42645567	313	358
14252	10	99997433	100017997	ENSMUSG00000025185	ENSMUSG00000025185	ENSMUSG00000025185	19	42647283	42666059	313	358
14253	10	100133312	100164931	ENSMUSG00000060224	ENSMUSG00000060224	ENSMUSG00000060224	19	42780233	42805774	313	358
14254	10	100165945	100196673	ENSMUSG00000025188	ENSMUSG00000025188	ENSMUSG00000025188	19	42808508	42833270	313	358
14255	10	100208865	100985609	ENSMUSG00000074852	ENSMUSG00000074852	ENSMUSG00000074852	19	42961927	43066533	313	358
14256	10	101080023	101144075	ENSMUSG00000025189	ENSMUSG00000025189	ENSMUSG00000025189	19	43494522	43549076	313	358
14257	10	101146648	101180403	ENSMUSG00000025190	ENSMUSG00000025190	ENSMUSG00000025190	19	43553074	43577820	313	358
14258	10	101282700	101286271	ENSMUSG00000044220	ENSMUSG00000044220	ENSMUSG00000044220	19	43665636	43669203	313	358
14259	10	101360305	101370213	ENSMUSG00000040414	ENSMUSG00000040414	ENSMUSG00000040414	19	43717112	43728192	313	358
14260	10	101409253	101455987	ENSMUSG00000025192	ENSMUSG00000025192	ENSMUSG00000025192	19	43743136	43787008	313	358
14261	10	101461596	101482413	ENSMUSG00000040018	ENSMUSG00000040018	ENSMUSG00000040018	19	43788436	43806292	313	358
14262	10	101481967	101505881	ENSMUSG00000025193	ENSMUSG00000025193	ENSMUSG00000025193	19	43806309	43821942	313	358
14263	10	101532479	101601939	ENSMUSG00000025194	ENSMUSG00000025194	ENSMUSG00000025194	19	43835715	43891262	313	358
14264	10	101625324	101759666	ENSMUSG00000025195	ENSMUSG00000025195	ENSMUSG00000025195	19	43901278	43965703	313	358
14265	10	101791940	101831624	ENSMUSG00000025196	ENSMUSG00000025196	ENSMUSG00000025196	19	44009618	44039831	313	358
14266	10	101938045	101979366	ENSMUSG00000025199	ENSMUSG00000025199	ENSMUSG00000025199	19	44126647	44160756	313	358
14267	10	101982040	102017420	ENSMUSG00000025200	ENSMUSG00000025200	ENSMUSG00000025200	19	44163331	44189154	313	358
14268	10	102037893	102080233	ENSMUSG00000037578	ENSMUSG00000037578	ENSMUSG00000037578	19	44200949	44245753	313	358
14269	10	102212788	102233491	ENSMUSG00000036961	ENSMUSG00000036961	ENSMUSG00000036961	19	44546783	44566123	313	358
14270	10	102236389	102269581	ENSMUSG00000051984	ENSMUSG00000051984	ENSMUSG00000051984	19	44570269	44589039	313	358
14271	10	102273487	102279626	ENSMUSG00000025204	ENSMUSG00000025204	ENSMUSG00000025204	19	44603563	44608726	313	358
14272	10	102285686	102299745	ENSMUSG00000036450	ENSMUSG00000036450	ENSMUSG00000036450	19	44616165	44629585	313	358
14273	10	102495322	102577430	ENSMUSG00000004231	ENSMUSG00000004231	ENSMUSG00000004231	19	44810705	44889338	313	358
14274	10	102722276	102735362	ENSMUSG00000025207	ENSMUSG00000025207	ENSMUSG00000025207	19	45042617	45056707	313	358
14275	10	102727569	102737262	ENSMUSG00000025208	ENSMUSG00000025208	ENSMUSG00000025208	19	45058325	45059753	313	358
14276	10	102737302	102744148	ENSMUSG00000025209	ENSMUSG00000025209	ENSMUSG00000025209	19	45059869	45066073	313	358
14277	10	102746819	102757583	ENSMUSG00000035342	ENSMUSG00000035342	ENSMUSG00000035342	19	45068507	45080375	313	358
14278	10	102767079	102780880	ENSMUSG00000035243	ENSMUSG00000035243	ENSMUSG00000035243	19	45089333	45098954	313	358
14279	10	102782197	102790956	ENSMUSG00000025212	ENSMUSG00000025212	ENSMUSG00000025212	19	45100985	45109693	313	358
14280	10	102810989	102817848	ENSMUSG00000025213	ENSMUSG00000025213	ENSMUSG00000025213	19	45129450	45132593	313	358
14281	10	102880252	102887536	ENSMUSG00000025215	ENSMUSG00000025215	ENSMUSG00000025215	19	45204026	45210254	313	358
14282	10	102976723	102978707	ENSMUSG00000025216	ENSMUSG00000025216	ENSMUSG00000025216	19	45287039	45288547	313	358
14283	10	103103810	103307068	ENSMUSG00000025217	ENSMUSG00000025217	ENSMUSG00000025217	19	45417062	45583324	313	358
14284	10	103328629	103337956	ENSMUSG00000025218	ENSMUSG00000025218	ENSMUSG00000025218	19	45605586	45613854	313	358
14285	10	103338035	103359415	ENSMUSG00000041035	ENSMUSG00000041035	ENSMUSG00000041035	19	45613926	45631598	313	358
14286	10	103360413	103445006	ENSMUSG00000040913	ENSMUSG00000040913	ENSMUSG00000040913	19	45631568	45713504	313	358
14287	10	103519877	103525817	ENSMUSG00000025219	ENSMUSG00000025219	ENSMUSG00000025219	19	45790129	45796184	313	358
14288	10	103534199	103568165	ENSMUSG00000025220	ENSMUSG00000025220	ENSMUSG00000025220	19	45803572	45836831	313	358
14289	10	103575722	103593667	ENSMUSG00000025221	ENSMUSG00000025221	ENSMUSG00000025221	19	45845657	45869333	313	358
14290	10	103595346	103805940	ENSMUSG00000039901	ENSMUSG00000039901	ENSMUSG00000039901	19	45972068	46051830	313	358
[truncated: 17,013 more chars]
